# Social status impacts T-cell responses through synapse strength in the prefrontal cortex

**DOI:** 10.1038/s41422-026-01235-7

**Published:** 2026-03-23

**Authors:** Hui Xiong, Daniel Amado-Ruiz, Tessa R. Lodder, Mireille Toebes, Ton N. Schumacher, Hailan Hu, Helmut W. Kessels

**Affiliations:** 1 Department of Cellular and Circuit Neuroscience, Swammerdam Institute for Life Sciences, Amsterdam Neuroscience, University of Amsterdam, Amsterdam, The Netherlands; 2 The Netherlands Institute for Neuroscience, Royal Netherlands Academy of Arts and Sciences, Amsterdam, The Netherlands; 3 Department of Molecular Oncology and Immunology, The Netherlands Cancer Institute, Amsterdam, The Netherlands; 4 Department of Neurobiology, Zhejiang University School of Medicine, Hangzhou, Zhejiang China

**Keywords:** Cell signalling, Immunology

## Abstract

Social status affects health by influencing the capacity of the immune system to respond to infection and disease. However, the neuronal mechanisms that explain how social status causes individual differences in immunity are unknown. In this study, we observed that among social groups of four male mice, those ranked second in the hierarchy displayed, on average, superior T-cell responses upon vaccination. The greater T-cell responses in second-ranked mice were dependent on synaptic communication ability in the brain. The brain circuits that control position in the social hierarchy are beginning to emerge, with the dorsomedial prefrontal cortex (dmPFC) as a central player. We found that selectively increasing the strength of dmPFC synapses or increasing the activity of dmPFC neurons was sufficient to boost antigen-specific T-cell percentages in response to vaccination. These findings reveal a causal link between the dmPFC and the peripheral immune system, enriching our understanding of the origin of health problems caused by social inequality.

## Introduction

Social hierarchies form spontaneously within social groups and serve to define social roles and allocate limited resources among group members.^1,2^ As well as providing better access to resources and greater reproductive success, a high social status also has a positive impact on health.^2,3^ In human societies, socioeconomic status, defined by relative income, education, and occupational position, has been identified as one of the strongest predictors of health, showing a steep inverse association with mortality and morbidity rates.^4–6^ Although these health differences can be partly explained by lifestyle-dependent risk factors and access to health care, a strong correlation between hierarchical status and health is conserved in groups of social animals, suggesting that this gradient also has an evolutionary origin.^2^

A direct relationship between social dominance and the regulation and function of the immune system has been observed within social groups of nonhuman primates.^7–9^ A genomic analysis of immune cells showed that they respond differently to immune challenges depending on social status. Interestingly, when groups of macaques were rearranged, immune characteristics adapted to the newly acquired social status, indicating a causal link between social rank and the immune system.^9^ A possible explanation for the correlation between social status and the immune system is that individuals with high or low social positions experience different levels of stress. The level of chronic psychosocial stress experienced by subordinates can vary among species, social contexts, and group size.^10–13^ In primate societies, subordinates suffer from increased chronic levels of the stress hormone cortisol when these low-ranked individuals are frequently intimidated and lack social support.^10^ The influence of chronic stress on health as a consequence of social defeat has been studied extensively in laboratory rodents. Social defeat induced by repeated violent acts by a dominant intruder for consecutive days results in profound physiological changes, including chronically high levels of corticosterone, the primary adrenal corticosteroid in rodents.^14,15^ Social defeat thereby negatively impacts the immune system, causing decreased levels of lymphocytes circulating in the blood and reduced clonal expansion of CD8^+^ T-cells upon antigenic challenge.^16,17^ However, violent acts of social subordination are not an intrinsic part of social hierarchies, and chronic stress due to social subordination may not be the only factor that shapes the effects of social status on the immune system.^9^ For instance, social integration and support may also affect health.^18,19^ How the brain is able to translate social dominance into altered immune function remains unknown.

The brains of social animals have evolved to be able to adapt to social norms and compete for a high rank in a social hierarchy.^20^ Multiple brain regions concertedly regulate social hierarchical behavior, but both human and animal studies point to the prefrontal cortex (PFC) as a key node that plays a central role.^21–23^ A direct causal relationship between social status and PFC function was demonstrated in mice: socially dominant mice had increased synapse strength in the dorsomedial region of the PFC (dmPFC).^24,25^ Synaptic transmission depends on the number of AMPA-type glutamate receptors (AMPARs) at synapses, and the addition of AMPARs at synapses through induction of long-term potentiation (LTP) of synaptic strength mediates several forms of adaptive behavior.^26^ Manipulation of AMPAR plasticity to strengthen or weaken dmPFC synapses is sufficient to increase or decrease, respectively, the position of a mouse in a social hierarchy.^24^ Triggering LTP at dmPFC synapses also promotes winning in social contests and an increase in social status.^27^ We set out to investigate the neuronal mechanism that explains the link between social status and the adaptive immune system in groups of laboratory mice in a non-violent setting.

## Results

### Social status influences T-cell responses to vaccination

**Fig. 1:**
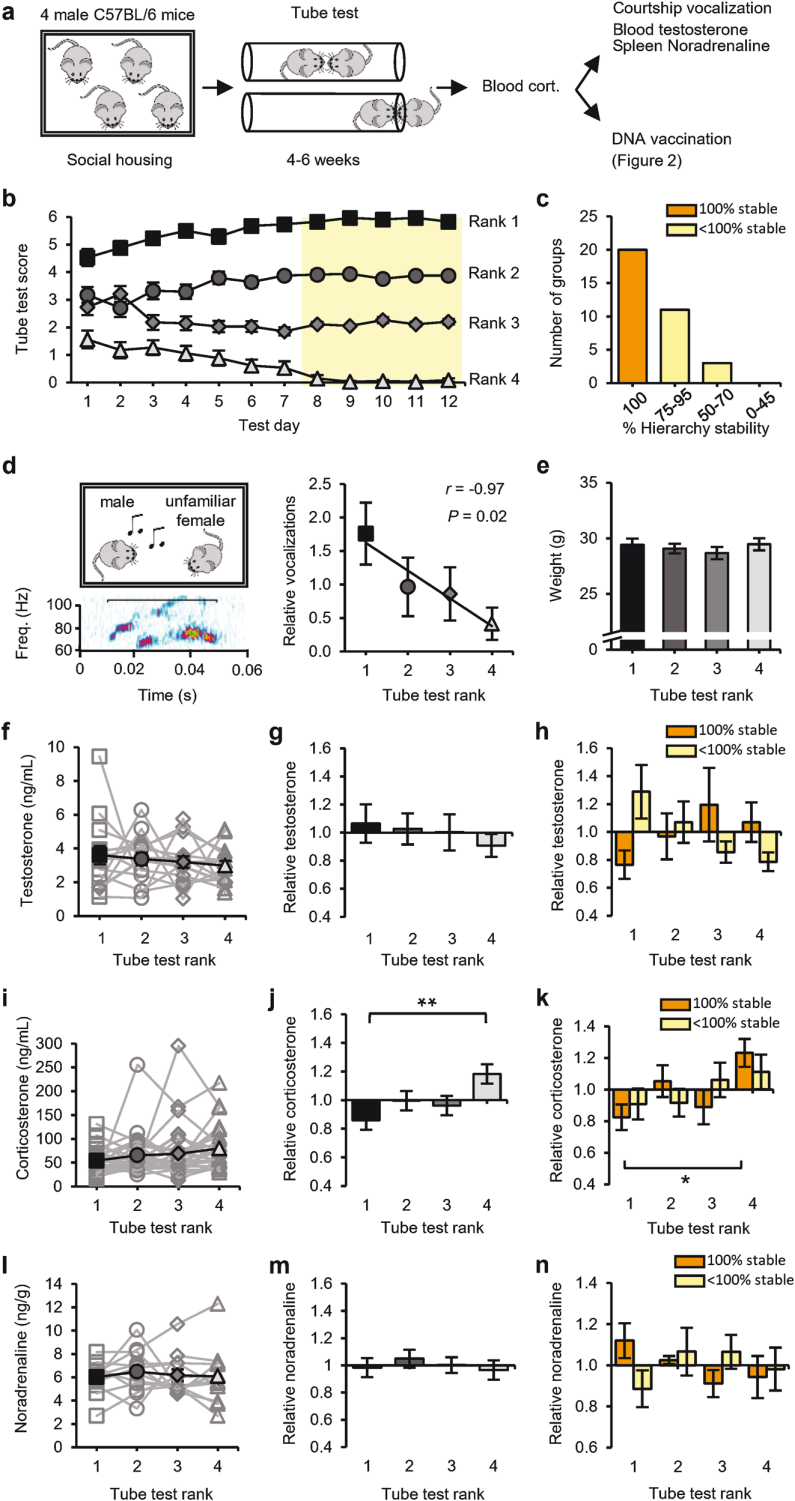
Social hierarchy and hormonal profile of group-housed mice. **a** Schematic of the experimental design: 34 groups of four male mice were subjected to the tube test. At the end of this testing period, blood samples were taken for measurement of corticosterone levels. Mice were then exposed to a courtship vocalization test, followed by measurement of blood testosterone and spleen noradrenaline levels (14 groups), or received DNA vaccination (20 groups; see Fig. 2). **b** Tube-test scores on each test day (*n* = 34 groups). Social ranks are based on performance on the last 5 test days (yellow). **c** Percentage of hierarchy stability during the last 5 test days. **d** Example vocalization (left) and the amount of vocalization relative to the group average per rank of social groups (*n* = 9). **e** Weight of mice per rank (*n* = 14 groups). **f–h** Blood testosterone per rank of social groups (*n* = 14) in absolute levels (**f**), relative to the group average (**g**), and relative to the group average split between 100% (orange; *n* = 6) and < 100% (yellow; *n* = 8) hierarchy stability (**h**). **i**–**k** Blood corticosterone per rank of social groups (*n* = 29) in absolute levels (**i**), relative to the group average (**j**), and relative to the group average split between 100% (orange; *n* = 17) and < 100% (yellow; n = 12) hierarchy stability (**k**). **l–n** Noradrenaline levels in spleen per rank of social groups (*n* = 12) in absolute levels (**l**), relative to the group average (**m**), and relative to the group average split between 100% (orange; *n* = 5) and < 100% (yellow; *n* = 7) hierarchy stability (**n**). Black square: rank 1; dark-gray circle: rank 2; gray diamond: rank 3; light-gray triangle: rank 4. Data are mean ± SEM. *P < 0.05, **P < 0.01. Statistics: one-way ANOVA with Tukey’s multiple-comparison test (**e**–**n**) and Pearson’s correlation (**d**).

We used the tube test to determine the social hierarchy among groups of four 2-month-old male mice that were housed together since weaning^24^ (Fig. 1a). Pairs of mice were allowed to enter a tube from opposite ends to meet in the middle, and the mouse that forced the opponent to retreat was scored as the more dominant of the pair. On each testing day, the mice were confronted with each of their fellow group members twice (in both directions of the tube) and were therefore able to score a maximum of 6 wins. Groups were tested for at least 4 weeks, during which a transitive social hierarchy became evident (Fig. 1b). The social status of each mouse was determined on the basis of the final 5 testing days, when the tube-test results remained stable in the majority of groups (Fig. 1c). Typically, encounters in the tube between two high-ranked mice took longer on average before a winner was decided than encounters that included a subordinate mouse (Supplementary information, Fig. S1a). As a complementary test for social status, mice were exposed to a female mouse to record ultrasonic courtship vocalizations.^24^ A linear correlation between tube-test rank and relative amount of vocalization confirmed that tube-test ranks were a reflection of social status (Fig. 1d). As reported for groups of age-matched mice,^28^ social ranks were not determined by physical size, since different ranks had, on average, comparable body mass (Fig. 1e). Although testosterone levels can be a predictor of social status,^13,29^ blood testosterone levels were similar among social ranks, both in absolute levels (Fig. 1f) and in levels relative to the group average (Fig. 1g). Studies in nonhuman primates indicate that high testosterone primarily correlates with high social status when the social hierarchy is unstable, suggesting that testosterone production increases when high social status is under threat.^30^ Consistent with these studies, we observed a positive correlation between social status and testosterone in unstable hierarchies but not in fully stable hierarchies (Fig. 1h and Supplementary information, Fig. S2a).

To assess whether a subordinate role within these groups of 4 male mice was accompanied by chronic psychosocial stress, blood samples were taken and analyzed for levels of corticosterone. The serum corticosterone concentration was low in the majority of groups (on average 67 ng/mL), and although it did not differ significantly among ranks (Fig. 1i), we did observe a negative correlation between social status and corticosterone levels (Supplementary information, Fig. S2b). When we calculated corticosterone levels relative to the group average, they were significantly higher in 4th-ranked mice than in 1st-ranked mice (Fig. 1j), particularly in groups whose hierarchy was fully stable (Fig. 1k). As an index for basal stress levels mediated by the sympathetic nervous system (SNS), spleens were isolated, and the concentration of noradrenaline was measured. Noradrenaline levels were similar among social ranks (Fig. 1l, m) in both stable and unstable hierarchies (Fig. 1n and Supplementary information, Fig. S2c), indicating that social status did not influence basal SNS activity. We used this experimental setup of social groups with four male mice as a model to study the relationship between social status and adaptive immune responses.

**Fig. 2:**
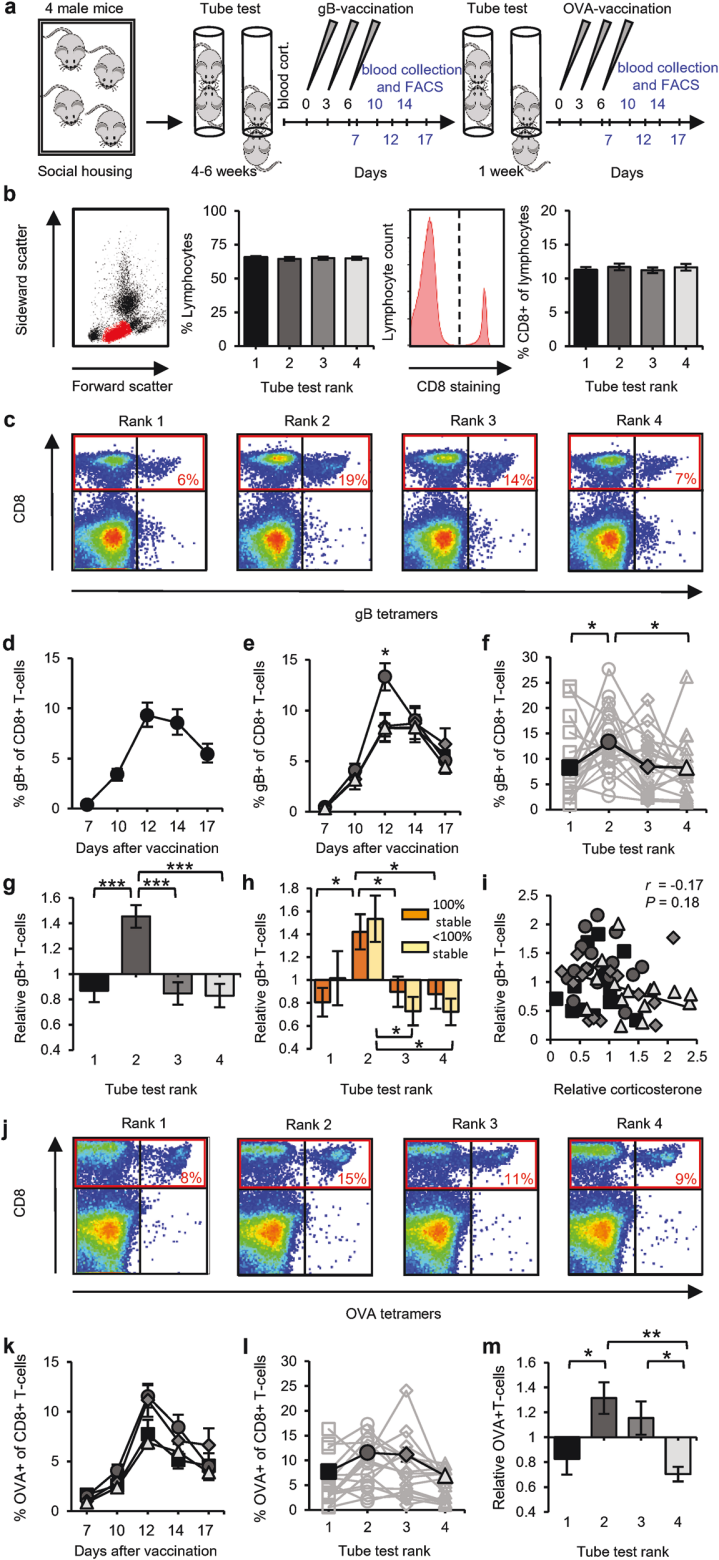
Second-ranked mice show greater antigen-specific T-cell responses. **a** Schematic of the experimental design: 20 groups of four male mice were subjected to the tube test and then received gB vaccination. Afterwards, for 15 of the 20 groups, 1 more week of the tube test was performed, followed by OVA vaccination. Sampling time points for FACS analysis are indicated in blue. **b** Basal T-cell levels in blood did not differ among social ranks. Example of a forward–side scatter FACS dot plot from a blood sample, with the lymphocyte population in red, and the percentage of lymphocytes in blood leukocytes (left). Representative FACS histogram of CD8 immunostaining of the lymphocyte population and the percentage of CD8^+^ cells in lymphocytes (right). Data are presented by rank (*n* = 20) within social groups of C57BL/6 mice. **c** Representative FACS plots of CD8^+^ T-cells (in red box) that recognize MHC tetramers loaded with gB peptide at day 12 after vaccination. **d**, **e** Time course of the percentage of gB-specific T-cells for all mice (*n* = 80; **d**) and split by rank (*n* = 20 groups; **e**). **f**–**h** Percentage of gB-specific T-cells (day 12) per rank of social groups (*n* = 20) in absolute levels (**f**), relative to the group average (**g**), and relative to the group average split between 100% (orange; *n* = 14) and < 100% (yellow; *n* = 6) hierarchy stability (**h**). **i** Relative blood corticosterone levels vs relative gB-specific T-cell expansion (*n* = 60 mice). **j**–**m** Representative FACS plots of CD8^+^ T-cells (in red box) that recognize MHC tetramers loaded with OVA peptide at day 12 after vaccination (**j**) and percentages of OVA-specific CD8^+^ T-cells per rank (*n* = 15 groups) shown as a time course (**k**), on day 12 (**l**), and relative to the group average (**m**). Black square: rank 1; dark-gray circle: rank 2; gray diamond: rank 3; light-gray triangle: rank 4. Data are mean ± SEM. *P < 0.05, **P < 0.01. Statistics: one-way ANOVA with Tukey’s multiple-comparison test (**b,** **f,** **g,** **h,** **l,** **m**), two-way ANOVA with Tukey’s multiple-comparison test (**e,** **k**), and Pearson’s correlation (**i**).

To challenge the adaptive immune system, we chose a vaccination strategy that triggers robust immune responses of CD8^+^ T-cells. We applied a DNA vaccination by intradermally tattooing a plasmid encoding the immunodominant MHC class I epitope of herpes simplex virus glycoprotein B (gB). This vaccination method was designed to selectively activate T-cells and was previously shown to induce the clonal expansion of antigen-specific CD8^+^ T-cells, leading to functional T-cell responses.^31^ Mice were vaccinated three times at the hind leg at two-day intervals, and blood samples were drawn at multiple time points after vaccination and analyzed by flow cytometry (Fig. 2a). The percentage of gB-specific T-cells among the CD8^+^ population was determined by MHC-tetramer staining (Fig. 2b, c). To test whether chronic elevations in corticosterone or testosterone affected T-cell populations in our model system, mice were implanted with pellets that released either of these hormones. Corticosterone, and to a lesser extent testosterone, suppressed the basal levels of lymphocytes circulating in the blood (Supplementary information, Fig. S3). Chronically high levels of corticosterone also severely suppressed the increase in gB-specific T-cells upon DNA vaccination, whereas high testosterone levels had no significant effect (Supplementary information, Fig. S3).

We compared the basal T-cell levels among mice of different social status. The percentages of total lymphocytes among leukocytes and of CD8^+^ T-cells among lymphocytes did not differ among ranks (Fig. 2b), consistent with the above finding that hormone levels were not sufficiently different among ranks to affect the basal percentages of T-cells in blood. Upon vaccination, the percentages of antigen-specific CD8^+^ T-cells gradually increased, peaking at day 12 after the 1st vaccination (Fig. 2d). Whereas gB-specific CD8^+^ T-cell levels progressed similarly for 1st-, 3rd-, and 4th-ranked mice, these percentages were 60% higher, on average, for 2nd-ranked mice than for the other ranks at day 12 post vaccination (Fig. 2e). The average antigen-specific CD8^+^ T-cell percentages at peak day 12 were significantly higher in 2nd-ranked mice than in 1st- and 4th-ranked mice in terms of absolute value (Fig. 2f) and significantly higher in 2nd-ranked mice than in the three other ranks when calculated relative to the group average (Fig. 2g). The greater potential for T-cell responses in 2nd-ranked mice was seen in both stable and unstable hierarchies (Fig. 2h). Although the increased gB-specific T-cell levels in blood induced by DNA vaccination were highly sensitive to elevated corticosterone levels (Supplementary information, Fig. S3), we observed no significant correlation between relative corticosterone level and the relative magnitude of T-cell responses (Fig. 2i). To examine whether our observation of greater T-cell responses in 2nd-ranked mice was independent of the specific antigen used, we re-established the tube-test ranks and then challenged the mice with a DNA vaccine encoding the immunodominant ovalbumin epitope. As observed after vaccination with the gB epitope, 2nd-ranked mice showed the largest increase in ovalbumin-specific CD8^+^ T-cell levels (Fig. 2j–m).

### GluA1 expression is required for the distinct T-cell responses of 2nd-ranked mice

Social hierarchies in mice have a strong genetic basis^32^ and are influenced by the capacity for social learning and synaptic plasticity.^33,34^ To explore potential brain mechanisms that may link social status to peripheral T-cell responses, we used a mouse model in which the gene encoding the AMPAR subunit GluA1 was knocked out (GRIA1^−/−^). Owing to the absence of GluA1 in the brain, GRIA1^−/−^ mice display impairments in LTP, memory encoding, and other forms of adaptive behavior.^35–37^ GluA1 expression is enriched in the nervous system and is undetectable in lymphoid tissues,^38^ making it unlikely that GluA1 expressed outside the brain directly influences immune responses.

**Fig. 3:**
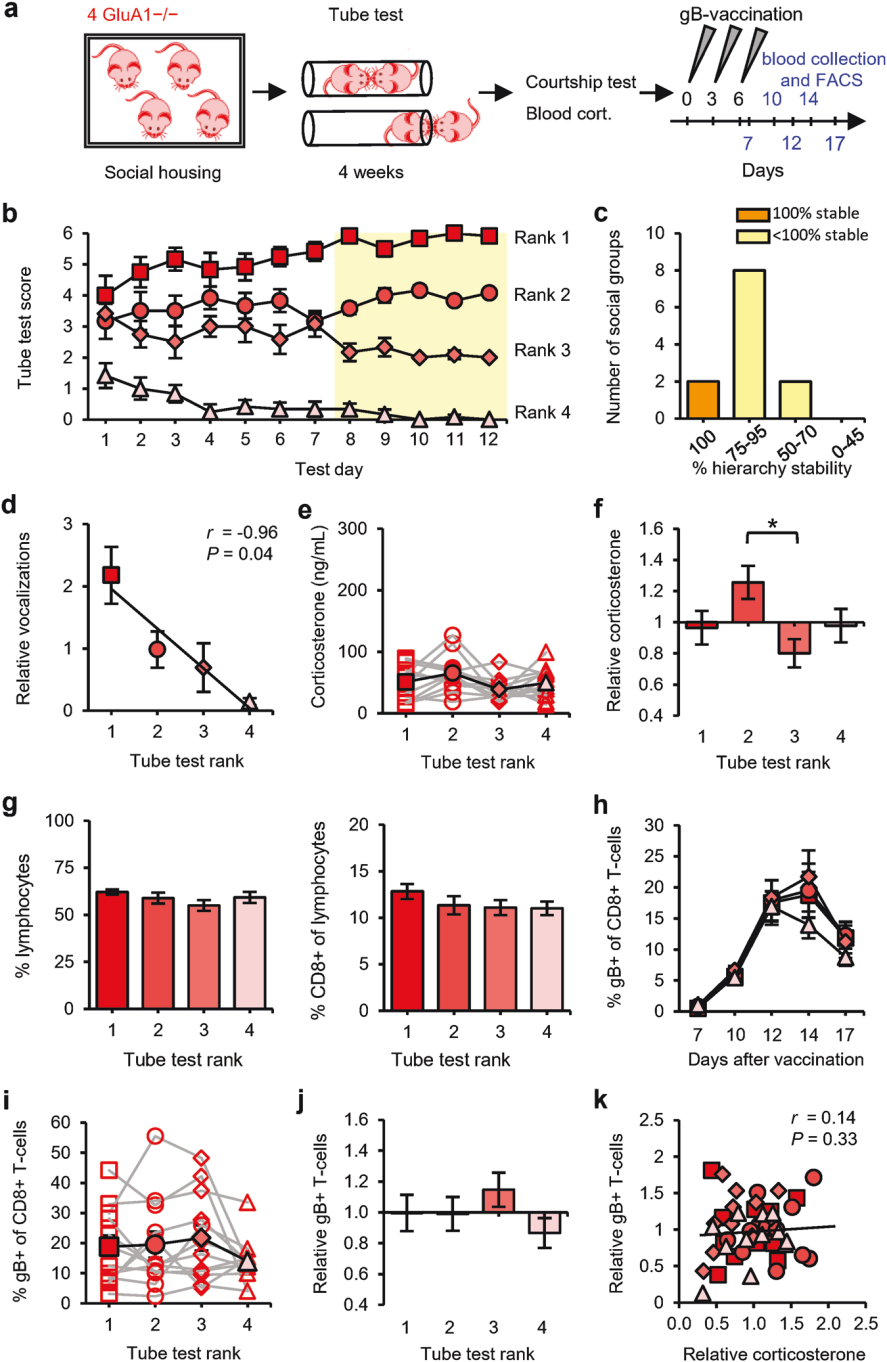
Equivalent T-cell responses among ranks in social groups of GluA1-deficient mice. **a** Schematic of the experimental design: social groups (*n* = 12) consisting of 4 male GRIA1^−/−^ mice (red) were subjected to the tube test. At the end of the testing period, courtship vocalizations and blood corticosterone were measured, and the mice then received gB vaccination. Sampling time points for FACS analysis are indicated in blue. **b** Tube-test scores on each test day. Social ranks are based on the last 5 test days (yellow). **c** Percentage of hierarchy stability during the last 5 test days. **d** Amount of vocalization relative to the group average per rank. **e**, **f** Blood corticosterone levels among ranks of social groups calculated as absolute levels, with individual groups shown as red open symbols and the average shown as red solid symbols (**e**), and as levels relative to the group average (**f**). **g** Percentage of lymphocytes in blood leukocytes (left) and percentage of CD8^+^ cells in lymphocytes (right) among ranks of GRIA1^−/−^ groups. **h** Time course of the percentage of gB-specific T-cells per rank. **i**, **j** Percentage of gB-specific T-cells (day 12) per rank of social groups calculated as absolute levels (**i**) and relative to the group average (**j**). **k** Relative blood corticosterone levels vs relative gB-specific T-cell expansion. Red square: rank 1; dark-pink circle: rank 2; pink diamond: rank 3; light-pink triangle: rank 4. Data are mean ± SEM. *P < 0.05. Statistics: one-way ANOVA with Tukey’s multiple comparison test (**e**–**g,** **i,** **j**), two-way ANOVA with Tukey’s multiple comparison test (**h**), and Pearson’s correlation (**d,** **k**).

We exposed social groups consisting of four GRIA1^−/−^ littermate mice to the tube test (Fig. 3a). GRIA1^−/−^ mice were able to establish social hierarchies (Fig. 3b), although with reduced stability compared with the social groups of wild-type (WT) mice (Fig. 3c and Supplementary information, Fig. S1b). Compared with WT mice, pairs of GRIA1^−/−^ mice spent less time in the tube on average before a winner was decided (Supplementary information, Fig. S1a). Specifically, whereas encounters between WT mice were shorter when they involved a subordinate, encounters between GRIA1^−/−^ mice were shorter irrespective of social rank. The tube-test results correlated significantly with the amount of courtship vocalizations (Fig. 3d), indicating that the behavior of GRIA1^−/−^ mice in the tube test reflected hierarchical behavior. Serum corticosterone levels were low on average in these groups of GRIA1^−/−^ mice (64 ng/mL), with slight differences between 2nd- and 3rd-ranked mice (Fig. 3e, f). The percentages of circulating lymphocytes were similar among ranks (Fig. 3g), indicating that basal T-cell levels were independent of social rank. Vaccination induced an increase in gB-specific CD8^+^ T-cell percentages in blood (Fig. 3h), which, in contrast to results from WT mice, was of a similar magnitude for the four different ranks (Fig. 3i, j). Antigen-specific T-cell levels were not correlated with relative corticosterone levels (Fig. 3k). These data indicate that social status does not influence T-cell immunity in the absence of GluA1 expression. In other words, 2nd-ranked mice must express GluA1 in order to show improved T-cell responses.

**Fig. 4:**
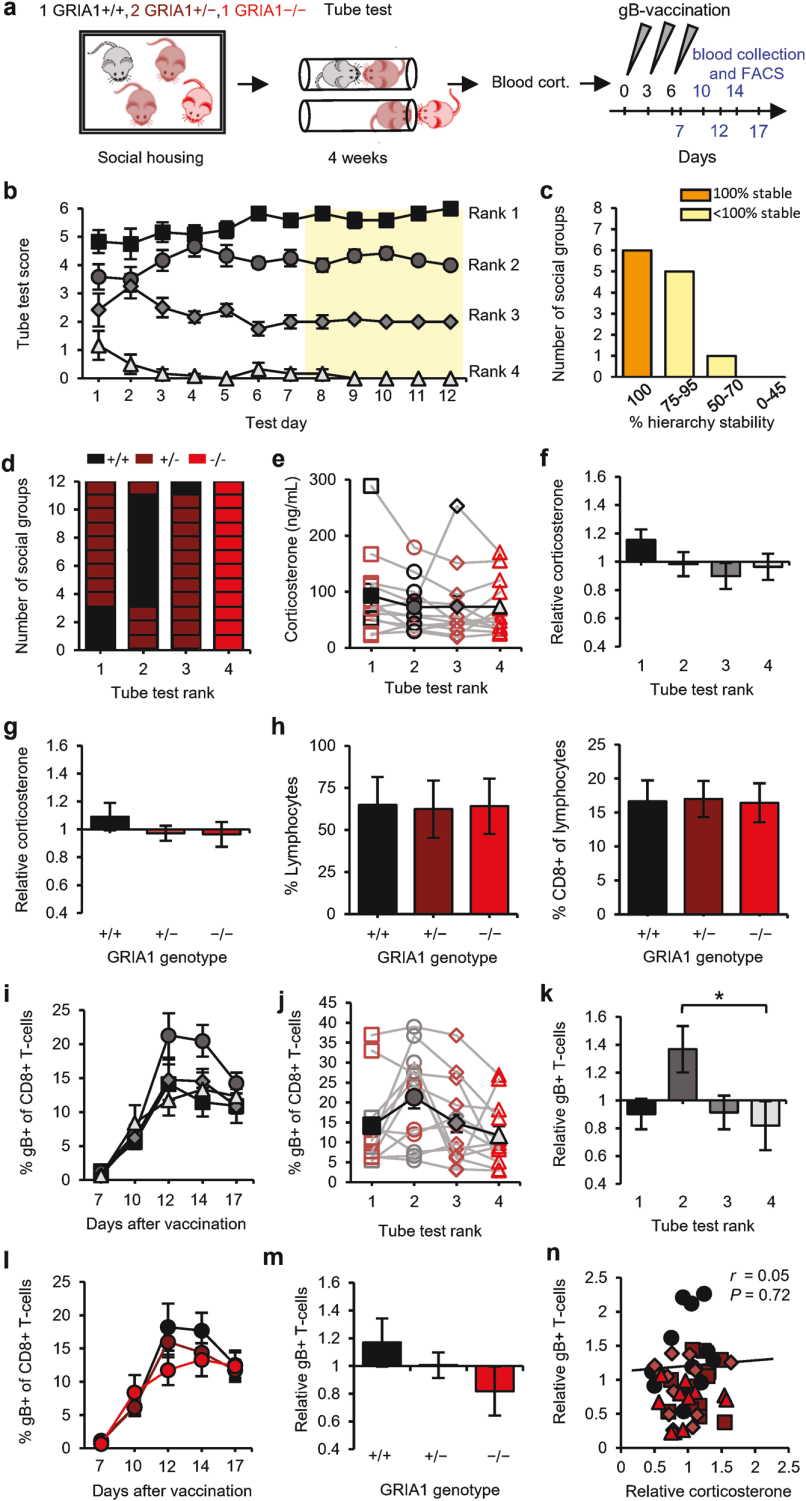
GluA1 expression promotes T-cell responses in 2nd-ranked mice. **a** Schematic of the experimental design: social groups (*n* = 12) consisting of one GRIA1^+/+^ (black), two GRIA1^+/−^ (brown), and one GRIA1^−/−^(red) littermate were subjected to the tube test. At the end of the testing period, blood corticosterone was measured, and the mice then received gB vaccination. Sampling time points for FACS analysis are indicated in blue. **b** Tube-test scores for each test day. Social rank was based on the last 5 test days (yellow). **c** Percentage of hierarchy stability during the last 5 test days. **d** Distribution of GRIA1 genotypes among ranks within social groups. **e**–**g** Blood corticosterone levels of social groups presented as absolute levels per rank, with individual groups shown as open symbols and the average shown as black solid symbols (**e**), levels relative to the group average per rank (**f**), and levels relative to the group average per genotype (**g**). **h** Percentage of lymphocytes in blood leukocytes (left) and percentage of CD8^+^ cells in lymphocytes (right) among GRIA1 genotypes. **i–k** Percentage of gB-specific CD8^+^ T-cells shown as a time course (**i**), as absolute levels (day 12) (**j**), and relative to the group average (**k**) per rank. **l**, **m** Percentage of gB-specific CD8^+^ T-cells shown as a time course (**l**) and relative to the group average (**m**) per genotype. **n** Relative blood corticosterone levels vs relative gB-specific T-cell responses. Black square: rank 1; dark-gray circle: rank 2; gray diamond: rank 3; light-gray triangle: rank 4. Data are mean ± SEM. *P < 0.05, **P < 0.01. Statistics: one-way ANOVA with Tukey’s multiple comparison test (**e**–**h,** **j,** **k,** **m**), two-way ANOVA with Tukey’s multiple comparison test (**i,** **l**), and Pearson’s correlation (**n**).

To examine whether mice benefit from expressing GluA1 in terms of becoming socially dominant and having improved T-cell responses, we established the social hierarchy in social groups composed of one GRIA1^+/+^ (WT), one GRIA1^−/−^ (knockout), and two GRIA1^+/−^ (heterozygote) littermates (Fig. 4a). These groups of mice developed stable hierarchies (Fig. 4b, c) with GRIA1^−/−^ mice consistently ending up at the lowest rank (Fig. 4d), indicating that the lack of GluA1 provides a competitive disadvantage in tube-test encounters. Interestingly, the presence of GluA1 throughout the brain is not necessarily a prerequisite for becoming 1st in rank, since WT mice were ranked 2^nd^ in the majority of groups (8 out of 12) (Fig. 4d). We did not observe differences in basal corticosterone levels among social ranks (Fig. 4e, f and Supplementary information, Fig. S1c) or GRIA1 genotypes (Fig. 4g), and the basal levels of circulating lymphocytes did not differ among GRIA1^−/−^, GRIA1^+/−^, and GRIA1^+/+^ mice (Fig. 4h). Upon DNA vaccination, gB-specific T-cells reached higher levels in 2nd-ranked mice compared with other ranks (Fig. 4i–k and Supplementary information, Fig. S1d). Correspondingly, T-cell responses tended to be greater in WT GRIA1^+/+^ mice than in GRIA1^−/−^ littermates, with GRIA1^+/−^ heterozygotes showing intermediate responses (Fig. 4l, m). Antigen-specific CD8^+^ T-cell responses were not correlated with relative glucocorticoid levels in these groups of mice (Fig. 4n). These results suggest that mice with increased synaptic plasticity throughout the brain have greater T-cell responses. However, our results do not reveal whether there is a direct or indirect causal relationship between brain synapses and the immune system.

### GluA1-expression in the dmPFC promotes T-cell responses

**Fig. 5:**
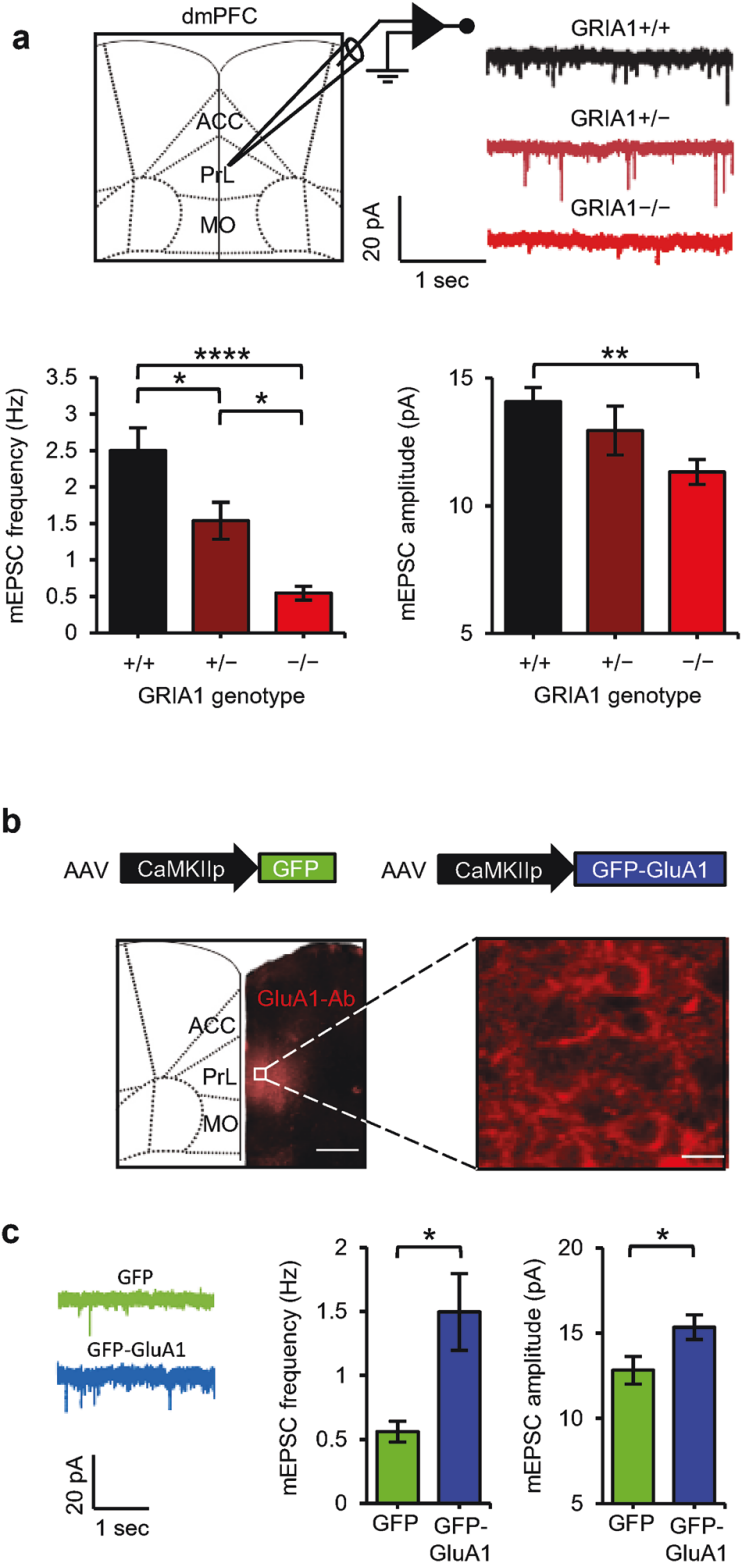
GluA1 expression in PL-dmPFC neurons increases synapse strength. **a** Representative traces, average mEPSC frequency, and mEPSC amplitude recorded from PL–dmPFC layer 5 neurons of WT (black, *n* = 24 cells), GRIA1-heterozygous (brown, *n* = 16 cells), and GRIA1-knockout (red, *n* = 21 cells) littermate mice. **b** AAV-mediated expression of GFP-GluA1 selectively in the PL-dmPFC of GRIA1^−/−^ mice assessed by GluA1 immunostaining. Scale bars: left, 500 µm; right, 15 µm. **c** Representative traces, average mEPSC frequency, and mEPSC amplitude recorded from GFP-expressing PL–dmPFC layer 5 neurons of GRIA1^−/−^ mice injected with AAV-GFP (green, *n* = 25 cells) or AAV-GFP-GluA1 (blue, *n* = 30 cells). Data are mean ± SEM. *P < 0.05, **P < 0.01, ****P < 0.0001. Statistics: one-way ANOVA with Tukey’s multiple-comparison test (**a**) and unpaired Student’s *t*-test (**c**).

AMPAR plasticity in dmPFC neurons helps to promote a high social status.^24,27^ To assess whether GluA1 expression affects synaptic currents onto dmPFC neurons, we performed whole-cell electrophysiology on layer 5 excitatory neurons in the prelimbic (PL) area of the dmPFC of brain slices from mice with different GRIA1 backgrounds. PL synapses were significantly weaker in GRIA1^−/−^ mice than in WT littermates, with those of GRIA1^+/−^ heterozygotes being intermediate (Fig. 5a). To assess whether GluA1 expression in the dmPFC helps to promote a high social status and possibly improved T-cell immunity, we applied adeno-associated virus (AAV)-mediated gene transfer to GRIA1^−/−^ mice. AAV encoding GFP-tagged GluA1 (or GFP as a control) was targeted bilaterally to the PL of the dmPFC in 3–4-week-old GRIA1^−/−^ mice. The transgenes were placed under the control of the CaMKIIα promoter to selectively express GFP-GluA1 in excitatory neurons.^39^ Immunohistochemistry with a GluA1-specific antibody showed that GluA1 expression was largely confined to the PL area of the dmPFC (Fig. 5b). Whole-cell electrophysiology recordings demonstrated that GluA1 expression significantly increased synaptic strength at GFP-expressing layer 5 pyramidal neurons in the PL area (Fig. 5c).

**Fig. 6:**
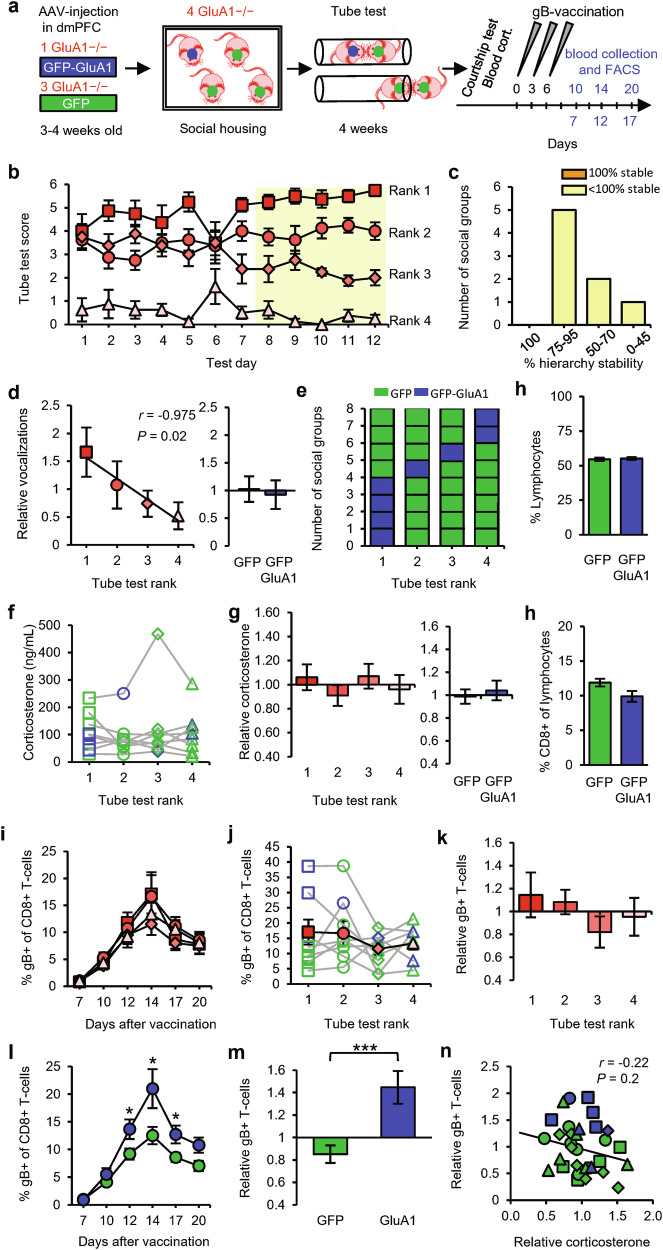
GluA1 expression in the dmPFC promotes T-cell responses in mice within social groups. **a** Schematic of the experimental design: social groups (*n* = 8) consisting of four male GRIA1^−/−^ littermate mice, one of which received GFP-GluA1 (blue) and three of which received GFP (green), were subjected to the tube test. At the end of the testing period, courtship vocalizations and blood corticosterone were measured, and the mice then received gB vaccination. Sampling time points for FACS analysis are indicated in blue. **b** Tube-test scores for each test day. Social rank was based on the last 5 test days (yellow). **c** Percentage of hierarchy stability during the last 5 test days. **d** Number of courtship vocalizations relative to the group average per rank (left) and comparison between GFP-GluA1-expressing and GFP-expressing mice (right). **e** Distribution of GFP-GluA1-expressing (blue) or GFP-expressing (green) mice among ranks of social groups. **f**, **g** Blood corticosterone levels shown as absolute levels per rank (**f**), shown relative to the group average per rank (**g**, left), and compared between GFP-GluA1-expressing and GFP-expressing mice (**g**, right). **h** Percentage of lymphocytes in blood leukocytes (top) and percentage of CD8^+^ cells in lymphocytes (bottom) for mice receiving GFP-GluA1 vs GFP. **i**–**k** Percentage of gB-specific CD8^+^ T-cells shown as a time course (**i**), in absolute levels (day 12) (**j**), and relative to the group average (**k**) per rank. **l**, **m** Percentage of gB-specific CD8^+^ T-cells shown as a time course (**l**) and relative to the group average (**m**), comparing GFP-GluA1-expressing vs GFP-expressing mice. **n** Relative blood corticosterone levels vs relative gB-specific T-cell expansion. Square: rank 1; circle: rank 2; diamond: rank 3; triangle: rank 4. Data are mean ± SEM. *P < 0.05, ****P < 0.0001. Statistics: one-way ANOVA with Tukey’s multiple-comparison test (**f,** **g** left, **j,** **k**), Pearson’s correlation (**e** left, **n**), unpaired Student’s *t*-test (**e** right, **g** right, **h,** **m**), and two-way ANOVA with Tukey’s multiple-comparison test (**i,** **l**).

After viral injection, male GRIA1^−/−^ littermate mice were placed in social groups of four, in which one mouse received AAV encoding GFP-GluA1 and the other three received AAV encoding GFP (Fig. 6a). When exposed to the tube test, a clear social hierarchy became evident in all groups (Fig. 6b). These hierarchies were as stable, on average, as those in groups of GRIA1^−/−^ mice (Fig. 6c and Supplementary information, Fig. S1b). Social ranks as established in the tube test were significantly correlated with performance in the courtship vocalization test, and the amount of vocalization was independent of GluA1 expression in the dmPFC (Fig. 6e). In the 8 social groups tested, mice expressing GluA1 in the dmPFC were ranked 1st in 4 groups, 2nd in 1 group, 3rd in 1 group, and 4th in 2 groups (Fig. 6d). It was recently shown that different cortical layers within the dmPFC have opposite effects on social status: whereas increased activity of layer 5 dmPFC neurons leads to an increase in social status, increased activity of layer 2/3 neurons lowers social status.^40^ For each mouse that was injected with AAV expressing GFP-GluA1, we calculated the extent to which GluA1 was expressed in layer 5 vs layer 2/3 in the dmPFC (Supplementary information, Fig. S4a). Mice in which GluA1 expression was predominantly targeted to layer 5 ended up at a high social status, whereas those in which GluA1 expression tended to be relatively higher in layer 2/3 had a low status within their social group (Supplementary information, Fig. S4b). Neither social rank nor GluA1-expression in the dmPFC influenced blood corticosterone levels (Fig. 6f, g) or basal levels of CD8^+^ lymphocytes circulating in blood (Fig. 6h) in these groups of mice. DNA vaccination triggered an increase in the percentage of gB-specific CD8^+^ T-cells, but this increase did not differ significantly among the social ranks (Fig. 6i–k). Direct comparison between mice that received GFP-GluA1 or GFP revealed that expression of GluA1 in the dmPFC enhanced T-cell responses (Fig. 6j, l, m), suggesting that dmPFC neurons can regulate T-cell immunity. The magnitude of T-cell responses was not correlated with relative blood corticosterone levels (Fig. 6n).

**Fig. 7:**
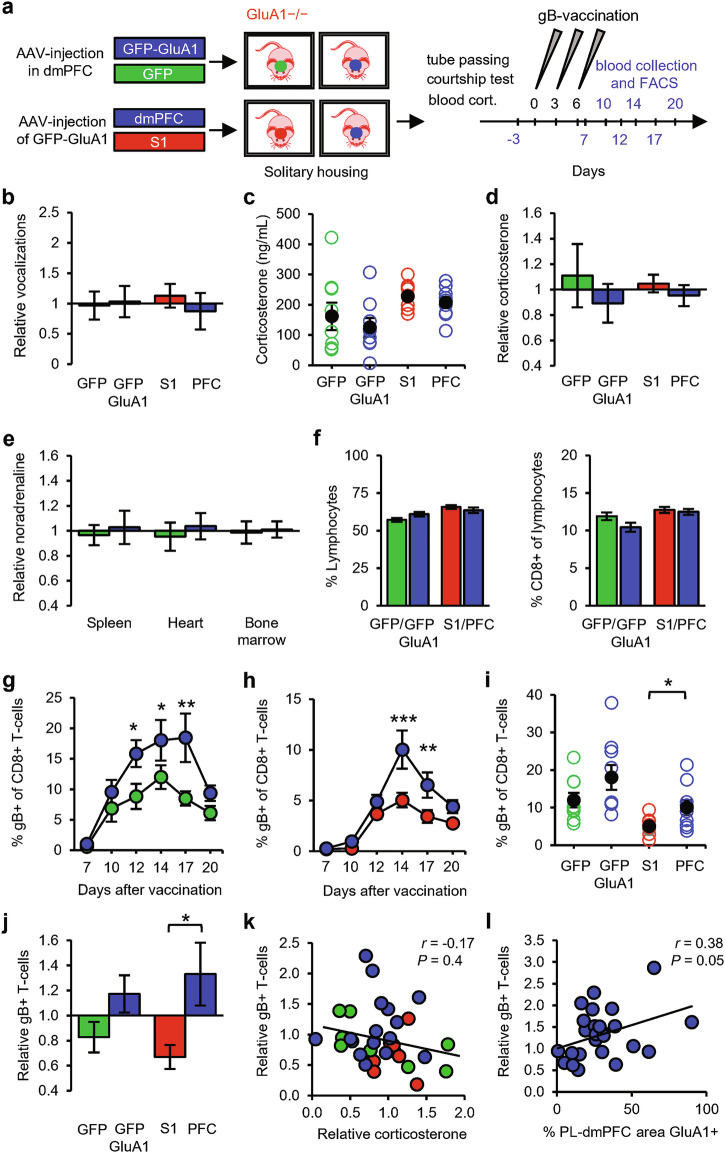
GluA1 expression in the dmPFC promotes T-cell responses in solitary mice. **a** Schematic of the experimental design: solitary GRIA1^−/−^ mice that received AAV expressing GFP-GluA1 (blue, *n* = 9) or GFP (green, *n* = 9) in the dmPFC or that received AAV expressing GFP-GluA1 in either the dmPFC (blue, *n* = 9) or the somatosensory cortex (S1, red, *n* = 9) were subjected to tube passing without an opponent. Courtship vocalizations and blood corticosterone were measured, and the mice then received gB vaccination. Sampling time points for FACS analysis are indicated in blue. **b** Number of courtship vocalizations relative to the average of littermates. **c**, **d** Blood corticosterone levels shown as absolute levels (**c**) and relative to the littermate average (**d**). **e** Relative noradrenaline levels in spleen, heart, and bone marrow of GRIA1^−/−^ littermate mice with AAV-injection in the dmPFC expressing either GFP-GluA1 (*n* = 5) or GFP (*n* = 4). **f** Percentage of lymphocytes in blood leukocytes (left) and percentage of CD8^+^ cells in lymphocytes (right). **g**, **h** Time course of the percentage of gB-specific CD8^+^ T-cells upon expression of GFP-GluA1 or GFP in the dmPFC (**g**) and upon GFP-GluA1 expression in either the dmPFC or S1 (**h**). **i**, **j** Percentage of gB-specific T-cells (day 14) in absolute levels (**i**) and relative to the littermate average (**j**). **k** No correlation was observed between relative blood corticosterone levels and relative gB-specific T-cell expansion. **l** Significant correlation between the percentage of the PL area in the dmPFC positive for GluA1-immunostaining and the relative T-cell response of social (Fig. 7) and solitary GRIA1^−/−^ mice that received GFP-GluA1 in the PL-dmPFC (*n* = 26). Data are mean ± SEM. *P < 0.05. Statistics: unpaired Student’s *t*-test (**b–f**, **i**, **j**), Pearson’s correlation (**k**, **l**), and two-way ANOVA with Tukey’s multiple comparison test (**g,** **h**).

On the basis of these findings, we considered two possibilities: either GluA1 expression in the dmPFC enhances social status, which in turn promotes T-cell responses, or GluA1 expression regulates social status and T-cell responses in parallel. To differentiate between these possibilities, we tested whether dmPFC synapses could stimulate T-cell responses in the absence of social interactions. Littermate GRIA1^−/−^ mice were injected in the PL region with AAV expressing either GFP-GluA1 or GFP at 3–4 weeks of age and were then solitarily housed (Fig. 7a). To enable comparison with socially housed mice, these mice were trained in crossing the tube (without an opponent) and were exposed to a female to record courtship vocalization, which was not influenced by GluA1 expression in dmPFC neurons (Fig. 7b). These experiments with solitary mice (Fig. 7) were performed largely in parallel with those of socially housed mice (Fig. 6). The solitary mice were socially isolated after weaning, which has previously been reported to cause neurochemical and behavioral changes^41^ but without affecting basal stress levels.^42–45^ Correspondingly, we found that plasma corticosterone levels of solitary mice were comparable to those of socially housed mice (Supplementary information, Fig. S5a). As seen in social groups, GluA1 in the dmPFC did not affect corticosterone levels (Fig. 7c, d). In addition, noradrenaline levels in immune organs were not altered in solitary mice with GluA1 in the dmPFC (Fig. 7e). These data indicate that the increased strength of dmPFC synapses in GRIA1^−/−^ mice did not change basal hypothalamic-pituitary-adrenal (HPA) axis or SNS activity. In line with this observation, basal levels of CD8^+^ T-cells in blood serum were not affected by GluA1 expression in the dmPFC (Fig. 7f). However, solitary mice expressing GluA1 in the dmPFC showed, on average, a greater increase in antigen-specific T-cells in response to vaccination (Fig. 7g). Although this increase in T-cell levels was not significantly greater in GluA1-transfected mice at peak day 14 (Fig. 7i, j), statistical significance was observed when taking the average of days 12, 14, and 17 combined (Supplementary information, Fig. S5b). These data suggest that dmPFC synapses do not require social behavior to boost T-cell responses.

Because GluA1 can be considered a foreign antigen to GRIA1^−/−^ mice, the injected AAV expressing GluA1 may theoretically have primed their immune systems, possibly further influencing immune responses. To control for this possibility, we included an experiment in which littermate GRIA1^−/−^ mice were injected with AAV expressing GFP-GluA1 either in the dmPFC or in a brain region not expected to be involved in linking social status to the immune system: the primary somatosensory cortex (S1). The mice were subsequently solitarily housed (Fig. 7a). These solitary mice showed similar levels of courtship vocalization irrespective of whether GluA1 was expressed in the dmPFC or S1 (Fig. 7b). Blood corticosterone levels were higher in these groups of solitary mice than in solitary mice from the previous experiment (Fig. 7c), and although this had no effect on the basal percentages of circulating CD8^+^ T-cells (Fig. 7f), the overall magnitude of T-cell increase was lower than that in the previous experiment (Fig. 7i; Supplementary information, Fig. S5b). Nevertheless, GRIA1^−/−^ mice that received GFP-GluA1 in the dmPFC showed a significantly greater expansion of antigen-specific CD8^+^ T-cells than those that received GFP-GluA1 in S1 in this experiment (Fig. 7h–j; Supplementary information, Fig. S5b), indicating that GluA1 expression specifically in the dmPFC increases T-cell responses without a contribution of antigenic effects. The relative magnitude of gB-specific T-cell expansion was not correlated with relative basal corticosterone levels (Fig. 7k). When we combined data from all GRIA1^−/−^ mice that received GFP-GluA1 in the dmPFC (i.e., both socially and solitarily housed mice), the relative gB-specific T-cell level was positively correlated with the percentage of GluA1-expression in the PL region (Fig. 7l). When percentages of GluA1-expression in layers 2/3 and 5 were calculated separately, this correlation was statistically significant for layer 5 neurons but not for layer 2/3 neurons (Supplementary information, Fig. S4c, d). These results indicate that strengthening dmPFC excitatory synapses in GRIA1^−/−^ mice was sufficient to promote T-cell expansion upon vaccination, demonstrating a mechanistic link between synaptic strength in the dmPFC and the peripheral adaptive immune system.

### Activation of dmPFC neurons promotes T-cell responses

**Fig. 8:**
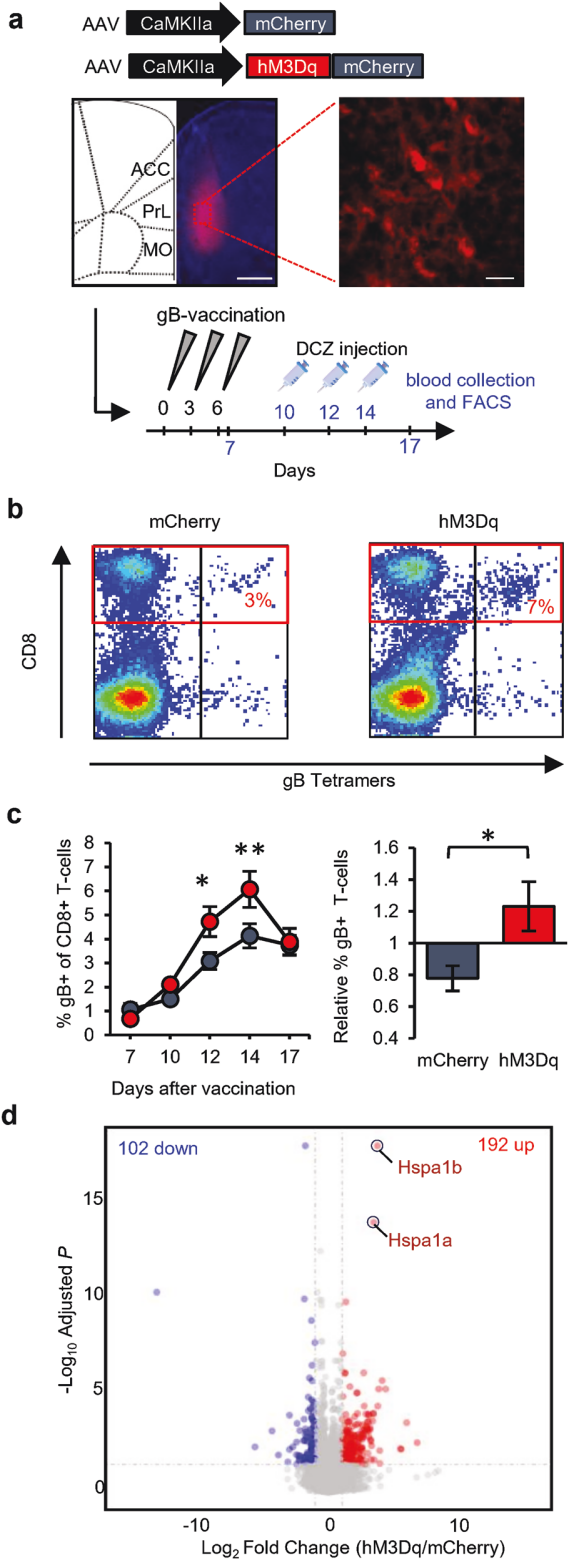
Chemogenetic manipulation of dmPFC neurons influences the activation and gene expression of CD8^+^ T-cells. **a** Representative image of AAV-mediated hM3Dq-mCherry expression (scale bars: left, 500 µm; right, 25 µm) selectively in the PL-dmPFC of WT mice and schematic of the experimental design with sampling time points indicated in blue. **b** Representative FACS plots of CD8^+^ T-cells (in red box) that recognize MHC tetramers loaded with gB peptide at day 12 after vaccination. **c** Time course of the absolute percentage of gB-specific CD8^+^ T-cells, showing a comparison between hM3Dq-mCherry (red; *n* = 22) and mCherry (gray; *n* = 21) in the dmPFC and relative to the littermate average (day 14). **d** Volcano plot of differentially expressed genes in splenic CD8^+^ T-cells at day 14 after vaccination. Data are mean ± SEM. *P < 0.05. Statistics: unpaired Student’s *t-*test (**c**, right) and two-way ANOVA with Tukey’s multiple-comparison test (**c**, left).

We observed that increasing synaptic strength onto dmPFC neurons selectively boosted the T-cell responses induced by vaccination. Since increased synaptic strength may translate to increased output activity, we next tested whether T-cell responses were enhanced when dmPFC neurons increased their activity. We applied the excitatory DREADD (designer receptors exclusively activated by designer drugs) system by bilaterally injecting AAV encoding the Gq-coupled hM3D receptor into the PL area of the dmPFC of WT mice (Fig. 8a). Expression of hM3Dq, which was under the control of a CaMKIIα promoter, only affects neuronal function in the presence of clozapine-based drugs such as deschloroclozapine (DCZ),^46,47^ showing a significant increase in intrinsic excitability in dmPFC neurons (Supplementary information, Fig. S6a). Mice received injections of AAV expressing either hM3Dq-mCherry or mCherry as a control and were vaccinated 3–4 weeks later with the gB epitope (Fig. 8a). As expected, basal lymphocyte levels circulating in the blood did not differ between the two groups of mice (Supplementary information, Fig. S6b). On days 10, 12, and 14 after the first vaccination, which were the time points when expansion of gB-specific T-cells was observed, the mice received an injection of DCZ. Two hours after DCZ injection, blood samples were taken and analyzed by fluorescence-activated cell sorting (FACS). On the days when mice were injected with DCZ, gB-specific T-cell levels were significantly higher in mCherry-hM3Dq-expressing mice than in mCherry-expressing mice (Fig. 8b, c). Injection of DCZ together with propranolol, a drug that selectively blocks β-adrenergic receptors (the type of adrenergic receptor that CD8^+^ T-cells primarily express), still boosted gB-specific CD8^+^ T-cell responses (Supplementary information, Fig. S6c), arguing against involvement of the SNS in communication between the dmPFC and the immune system. Inhibition of dmPFC neurons by expression of the inhibitory DREADD hM4Di in the PL area tended to reduce the expansion of gB-specific T-cells upon vaccination (Supplementary information, Fig. S6d). These data indicate that the activity of dmPFC neurons influences antigen-specific T-cell responses to vaccination.

We next asked whether activation of dmPFC neurons altered the gene expression profiles of peripheral CD8^+^ T-cells. Mice that expressed hM3Dq-mCherry or mCherry in PL-dmPFC neurons received injections of DCZ on days 10, 12, and 14 after vaccination, and their spleens were isolated 2 h after the third DCZ injection (day 14) (Supplementary information, Fig. S7a). DNA vaccination led to an accumulation of gB-specific CD8^+^ T-cells in the spleen (Supplementary information, Fig. S7b) similar to that observed in the blood. FACS was used to sort splenocytes for CD8^+^ cells, on which we then performed gene expression (RNA sequencing) analysis. This analysis revealed that 192 genes were significantly upregulated and 102 genes were significantly downregulated in CD8^+^ cells of mice with hM3Dq-mCherry in the dmPFC (Fig. 8d and Supplementary information, Fig. S7c, d), indicating that activation of dmPFC neurons alters the gene expression patterns of CD8^+^ splenocytes. To understand which functional pathways were linked to these differences in gene expression, we performed a Kyoto Encyclopedia of Genes and Genomes (KEGG) analysis, which revealed significant activation of the p38 MAPK pathway in CD8^+^ splenocytes, specifically on the basis of the genes that were upregulated (Supplementary information, Fig. S8). The MAPK pathway is activated upon T-cell receptor activation and promotes both proliferation and survival of CD8^+^ T-cells.^48^ The two genes that were most significantly upregulated, *Hspa1a* (10-fold) and *Hspa1b* (12-fold), encode Hsp70 heat-shock proteins, which interact with MAPK and are known to maintain protein homeostasis and avert apoptosis.^49^ Other notable genes whose expression changed significantly encoded the interleukin-7 receptor (IL7R; 1.4-fold down), chemokine receptor CXCR4 (1.6-fold down), invariant chain CD74 (1.6-fold up), and CD8 alpha chain (CD8a; 1.2-fold up) (Supplementary information, Fig. S7d and Table S1). IL7R and CXCR4 are expressed on naïve T-cells and are downregulated upon T-cell receptor activation.^50,51^ CD74 has been identified as an activation marker of T-cells,^52^ and CD8a gene expression increases in CD8^+^ effector T-cells upon exposure to pro-inflammatory cytokines.^53^ Combined, these gene expression patterns point toward increased proliferation and/or survival of CD8^+^ T-cells in response to vaccination as a consequence of dmPFC neuronal stimulation, which leads to higher levels of activated effector T-cells in the spleen.

## Discussion

In this study, we examined the neuronal mechanism underlying the link between social status and the immune system under laboratory conditions. We examined the social hierarchies among groups of four male isogenic, age-matched mice. The tube test enables assessment of social hierarchies in a non-violent setting, and tube-test results correlate well with the results of several other hierarchy tests,^24^ including the amount of courtship vocalization, as confirmed in this study. The notion that high social status is necessarily correlated with aggressive behavior or high testosterone levels^54^ is a misconception. Whereas such correlations may be present in unstable hierarchies where status needs to be reestablished, this is not the case for stable hierarchies, which in fact function to reduce aggression among group members.^30,34^ As a readout for the effectiveness of the immune system, we measured the level of CD8^+^ T-cell expansion upon DNA vaccination, which can be used to generate antiviral or antitumor immunity.^55^ We found that in groups of four WT mice, 2nd-ranked mice showed higher CD8^+^ T-cell responses to the vaccine on average compared with the other ranks.

The use of GluA1-knockout mice provided an important clue as to why being ranked 2nd can boost immune responses. First, GluA1 expression appeared to be necessary for the greater T-cell expansion of 2nd-ranked mice. Second, when we compared social groups in which individual mice had different levels of GluA1 expression throughout the brain, we observed that although a lack of GluA1 was predictive of becoming subordinate, WT mice (i.e., those with the highest levels of GluA1 expression) most often became second in rank. This observation suggests that although having GluA1 in some neuronal types or brain regions promotes dominance behavior, its presence in other regions may suppress social dominance.^23^ A brain region for which AMPAR plasticity is known to promote dominance is the dmPFC.^24,27^ However, anatomically segregated neural pathways downstream of the dmPFC were recently shown to differentially mediate social status.^40^ Neurons in layer 5 of the dmPFC that project to the dorsal raphe nucleus (DRN) and periaqueductal gray (PAG) promote winning in social competition. Expression of GluA1 in these pathways and in these downstream brain regions is therefore likely beneficial for achieving high social status. By contrast, layer 2/3 dmPFC neurons projecting to the basolateral amygdala (BLA) suppress social competition. Increased activity in BLA neurons also lowers the chances of achieving a high social status.^40^ The BLA is therefore an example of a brain region in which enhanced GluA1 plasticity likely prevents social dominance. Consistent with this model, when viral expression of GluA1 in the dmPFC of GRIA1^−/−^ mice was skewed more toward layer 5 neurons than toward layer 2/3 neurons, these mice became dominant in their social group. Conversely, when GluA1 expression was relatively higher in layer 2/3, the mice were ranked 3rd or 4th. Mice with a superior capacity for GluA1 plasticity throughout the brain (i.e., including both layer 5 and layer 2/3 neural pathways) most likely ended up 2nd rather than 1st in social rank. GluA1 expression selectively in dmPFC neurons of GluA1-knockout mice did not increase dominance in courtship behavior, suggesting that the stimulation of complex, socially competitive behaviors such as vocalization likely requires GluA1 expression in more brain regions than the dmPFC alone.^56,57^ Further studies are required to assess whether 2nd-ranked mice inherently have stronger synaptic connectivity and/or capacity for synaptic potentiation than the other three ranks within a social group. It is tempting to speculate that the second position in a hierarchy may be the most advantageous or cleverest position, since it still allows relatively good access to resources without having the primary responsibility of fighting off danger. Furthermore, our finding that mice with superior synaptic plasticity, i.e., with a better ability to learn, within a social group displayed greater T-cell responses may support the notion that the relationship between intelligence (i.e., IQ or education level) and health, besides being attributed to lifestyle factors, may also have a biological origin.^58^

We reasoned that a brain region that promotes social status — the dmPFC — may also be beneficial for boosting T-cell expansion. Our data demonstrate that synapse strength in the dmPFC affects the response of CD8^+^ T-cells to vaccination. The notion that dmPFC synapses link social status to peripheral T-cell responses in mice is consistent with a neuroimaging study in humans in which dmPFC activity correlated with the severity of inflammation dependent on subjective social status.^59^ Notably, we found that dmPFC neurons were capable of boosting T-cell responses in the absence of social interactions, indicating that dmPFC neurons did not change immune responses indirectly by altering social behavior such as grooming or acts of subordination, but instead controlled social behavior and peripheral immune responses in parallel. It is possible that other brain regions, particularly those downstream of the dmPFC, may be involved in modulating adaptive immunity, dependently or independently of social behavior.^60^ In future studies, it will be interesting to assess the extent to which downstream projecting regions of the dmPFC, such as the DRN, PAG, and BLA, are involved in mediating peripheral immune responses. Our observation that immune responses correlate well with GluA1 expression levels in layer 5 rather than layer 2/3 dmPFC pyramidal neurons suggests that a neural pathway downstream of these layer 5 neurons could be responsible for boosting immune responses. A limitation of this study is that we used only male mice as subjects. The question therefore remains whether the same findings hold true for social groups consisting of female mice.^61^

We did not find evidence that differences in hormone levels could explain the enhanced T-cell immunity of 2nd-ranked mice or mice with enhanced synapse strength in the dmPFC. In the majority of social groups, all ranks showed low absolute levels of glucocorticoids, indicating low basal activity of the HPA axis. The lowest-ranked mice had higher corticosterone levels than the top-ranked mice only in groups whose hierarchy was fully stable. This hormonal profile is consistent with previous literature on nonhuman primates, supporting the notion that chronic stress in subordinates is dependent on social context.^62^ What we did not observe in these groups of male mice, but has been reported for groups of nonhuman primates in the wild, is that the top-ranked individuals can display increased cortisol levels when the hierarchy is unstable.^13^ Increased cortisol in alpha males has been attributed to metabolic rather than psychosocial stress^63^ and does not necessarily negatively impact health.^64^ A likely explanation for the absence of increased metabolic stress in dominant male mice is that mice do not compete for food or females in the standard laboratory setting. Our data suggest that, as previously suggested for nonhuman primates,^9^ social status in groups of laboratory mice can influence T-cell immunity independently of increased chronic stress mediated by the HPA axis.

Sympathetic activation of the autonomic nervous system can affect adaptive immune responses by releasing noradrenaline in immune organs.^65,66^ For instance, the central amygdala and paraventricular nucleus control the adaptive immune system via their connection with the splenic nerve.^67^ Although splenic noradrenaline levels are not altered upon social defeat after frequent social subordination,^68^ we did consider the possibility that rewarding experiences, such as winning in the tube test or being groomed, may influence sympathetic activity. Elegant previous studies have shown that activation of the ventral tegmental area, a brain region that mediates positive emotions and motivation, boosts T-cell responses by inhibiting SNS-mediated noradrenaline release in immune organs.^69,70^ However, we did not detect differences in splenic noradrenaline levels among mice of different social status, and GluA1 expression in the dmPFC did not produce changes in noradrenaline levels in immune organs. In addition, activation of dmPFC neurons still promoted T-cell responses when β-adrenergic receptors were blocked. The increased T-cell expansion observed in our experiments is therefore unlikely to have been a consequence of altered basal SNS activity. In this study, we focused on the neuronal mechanism that explains the link between social status and the immune system. We found that increased dmPFC activity after vaccination was sufficient to increase the levels of antigen-specific CD8^+^ T-cells in blood and spleen and to change the gene expression profile of CD8^+^ splenocytes. These gene expression changes were reflective of enhanced proliferation and/or survival of CD8^+^ T-cells, pointing toward increased clonal expansion in response to DNA vaccination.

How dmPFC neurons play a part in conveying social status information to the immune system remains to be determined. The brain and immune system can potentially interact through a variety of neuronal and endocrine pathways to direct peripheral T-cell responses.^71^ dmPFC neurons, in particular those of the PL area, are critical for the development of value-based behavioral strategies in response to fearful or rewarding cues.^72^ As such, the dmPFC is part of the mentalizing network that plays a central role in processing social hierarchy information in rodents, non-human primates, and humans, mediating appropriate behavioral responses during social encounters.^34^ Our experiments suggest that the dmPFC can also direct appropriate downstream signaling in response to an immunological challenge. This study, combined with the observation that winning at social encounters leads to strengthening of dmPFC synapses,^27^ may inspire future incentives to design social therapies that offer the potential to voluntarily improve immune responses for individuals suffering from social status inequality.

## Materials and methods

### Animals

C57BL/6 mice (Harlan Sprague Dawley Inc. SLAC Laboratory Animal Co.) and GRIA1-deficient mice (a kind gift from Dr. R. Huganir,^73^ backcrossed at least 6 times to the C57BL/6 background) were maintained under a 12-h light cycle (lights on at 07:00) with ad libitum access to food and water. Male mice were socially housed in groups of 4 or solitarily housed from weaning (3 weeks of age). Mice were kept group-housed or solitarily housed for the entire program of experimentation, which lasted up to 18 weeks. All protocols were approved by the Animal Welfare Authority (IvD) at the Central Committee of Animal Experimentation (NVWA). The experimental protocols were approved by the Animal Experiment Committee of the Royal Netherlands Academy of Arts and Sciences (KNAW), the University of Amsterdam, and Zhejiang University.

### Dominance tube test

This test uses a transparent Plexiglas tube, 30 cm in length and 3 cm in inside diameter, which is just sufficient to permit the passage of one adult mouse but not for two mice to pass each other. During the initial 5-day training stage, each mouse was released individually at alternating ends of the tube and allowed to run through the tube; its back was gently pushed with a plastic stick if necessary. Each mouse was given eight trials per training day. On test days, during each test trial, two mice were released simultaneously into opposite ends to meet in the middle of the tube. The mouse that first retreated from the tube within 2 min was designated the “loser” of that trial. In rare cases when mice failed to retreat within 2 min, the test was repeated. The tube was cleaned with 30% ethanol between trials. Within each cage of four males, paired encounters were staged using a round-robin design, such that each male met each of the other three males twice in alternating directions and could gain a maximum of six wins per testing day. All social groups were exposed to at least 12 testing days spanning at least 4 weeks. The stability of the hierarchy for each social group was determined by calculating the percentage of times that the tube-test scores of each mouse in the group remained unaltered between consecutive testing days during the last 5 test days and averaging these 4 individual percentages per social group. In all experiments, social groups consisted of 4 male littermates (GRIA1 mice) or mice that had been cage mates at least since weaning (C57BL/6 mice). Solitarily housed GRIA1 mice (male littermates) were allowed to pass through the tube without an opponent on at least 12 testing days to exclude nonsocial effects of tube testing experiences on potential differences in brain and immune physiology between solitary and socially housed mice.

### Ultrasonic courtship vocalization test

Male mice were individually habituated in a home-like cage for 10 min. The remaining cage mates were placed in close proximity during habituation and testing. To trigger courtship vocalizations, a 5–6-week-old female, injected 2 days prior with Folligonan (MSD, 141281 R1) to induce the estrous cycle, was placed with the male for 5 min. Ultrasonic vocalizations were recorded using an Avisoft-UltraSoundGate 116H high-quality condenser microphone (Avisoft Bioacoustics, 51162), and the number and duration of 55–125 kHz vocalizations were analyzed using Avisoft SASLab Pro software (Avisoft Bioacoustics, 51164). For each mouse, the amount of vocalization was quantified as the number of vocalizations multiplied by the average duration of vocalizations, divided by the average amount of vocalization of the group (socially housed) or littermates (solitarily housed).

### Hormone assays

To measure corticosterone levels in blood, mice were fixed in a short Plexiglas tube, and approximately 20 μL of blood was immediately drawn (within 1 min) and collected in a heparin-coated tube. Blood samples were taken between 10 a.m. and 11 a.m. to avoid variation due to circadian rhythms on two consecutive days. For testosterone, mice were sacrificed, and 0.3–0.5 mL of blood was drawn by cardiac puncture. Samples were centrifuged at 2500× *g* for 15 min at 4 °C, and plasma was stored at −20 °C. Corticosterone levels were measured using a corticosterone enzyme immunoassay (Arbor Bioassays, K019-H5) and testosterone levels using an enzyme-linked immunosorbent assay (Enzo Life Sciences, ADI-900-065) according to the manufacturers’ instructions. To measure noradrenaline levels in immune organs, the spleen, heart, and/or bone marrow were extracted and frozen in liquid nitrogen. Spleen and heart samples were homogenized using a T10 basic Ultra-Turrax instrument (IKA, 0003737000) at 2% mass/volume in homogenate solution (0.08 M acetic acid + 80 mg/mL reduced glutathione (Sigma, 27221 and G4251)), then centrifuged at 2500 × *g* for 15 min at 4 °C. Bone marrow samples were collected from the tibias and femurs, homogenized in 2 mL of homogenate solution, and centrifuged at 300 × *g* for 10 min at 4 °C. Noradrenaline levels were quantified by liquid chromatography–tandem mass spectrometry (LC–MS/MS) at the Department of Laboratory Medicine, University Medical Center Groningen, Groningen.^74^

### Pellet implantation

Pellets were constructed by gently heating 100 mg cholesterol (≥99%, Sigma-Aldrich, C8667), a mixture of 70 mg cholesterol and 30 mg corticosterone (≥ 98.5% HPLC, Sigma-Aldrich, C2505), or a mixture of 70 mg cholesterol and 30 mg testosterone (≥ 99% HPLC, Sigma-Aldrich, T1500) in a small stainless steel spoon over a low gas flame until a liquid was formed, which was then poured into a pellet mold. Pellets were implanted subcutaneously in the interscapular region of the mouse back.

### Vaccination and analysis of antigen-specific CD8^+^ T-cells

DNA vaccines were generated by introducing gene fragments encoding herpes simplex virus (HSV) glycoprotein B epitope (gB_498–505_: SSIEFARL) or the ovalbumin epitope (OVA_257–264_: SIINFEKL) fused to tetanus toxin fragment C (TTFC) and separated by a GVQI peptide linker, into pVAX as described previously.^75^ This non-toxic fragment of TTFC displays functional characteristics of helper antigens and provides enhanced immunogenicity when fused to a class I epitope. Intradermal DNA tattoo vaccination was performed as described previously.^31^ Briefly, mice were anesthetized, and 15 μL of a 1.8 μg/μL DNA solution in water was applied to the shaved skin of the hind leg. The DNA vaccine was applied to the skin by a 45-s tattoo using a sterile disposable 9-needle bar oscillating at a frequency of 100 Hz and set to a depth of 0.5 mm (Sapphire Pro MC-8800-Pro Mei·cha). Mice were vaccinated three times at 2-day intervals. At days 7, 10, 12, 14, 17, and (20) after the first vaccination, ∼50 μL of peripheral blood was drawn and collected in 1 mL PBS containing heparin (1:500). Erythrocytes were removed by incubation in erythrolysis buffer (155 mM NH_4_Cl, 10 mM KHCO_3_, 0.1 mM EDTA, pH 7.4) on ice for 15 min, and cells were washed twice in PBA (1× PBS, 0.5% BSA, and 0.02% sodium azide). Samples were stained with FITC-conjugated anti-CD8a (BD Biosciences, 553030) and APC- or PE-conjugated gB_498–505_ or OVA_257–264_ loaded H-2Kb (MHC class I) tetramers for 15 min at room temperature. MHC tetramers were produced as described previously^76^ or purchased from Helixgen (Guangzhou) Co., Ltd. Cells were washed twice in PBA and analyzed by flow cytometry (BD FACScalibur). Live cells were selected on the basis of 7-aminoactinomycin D (BD Pharmingen, 559925) exclusion. Data were analyzed using FlowJo software. In all experiments, antigen-specific T-cell responses were calculated as the percentage of MHC tetramer-positive CD8^+^ T-cells relative to total CD8^+^ T-cells.

### AAV-mediated gene transfer

The gene encoding GFP or GluA1 (rat) fused with GFP (pH-sensitive version super-ecliptic pHluorin, SEP) was inserted into the transfer plasmid pTRCGW with a short version of the calcium/calmodulin-dependent protein kinase II (CaMKII, 0.4 kb) promoter and packaged in the coat of AAV1. For the GFP-GluA1 construct, the woodchuck hepatitis virus post-transcriptional regulatory element (WPRE) was removed to reduce the plasmid size for packaging into the AAV capsid. AAV1-GFP and AAV1-GFP-GluA1 virions were produced as described previously.^77^ Titers (genomic copies/mL) were determined by quantitative PCR on viral DNA using primers directed against the enhancer portion of GFP or SEP (5′-TCAGTGGAGAGGGTGAAGGT-3′ and 5′-AACTACCTGTTCCTTGGCCA-3′ for SEP and 5′-GTCTATATCATGGCCGACAA-3′ and 5′-GCATCAAGGTGAACTTCAAG-3′ for GFP). Titers of the vectors were 3–5 × 10^12^ gc/mL. AAV2/9-mCaMKII-hM3Dq-mCherry-WPRE-pA (S-0140-9) and AAV2/9-mCaMKII-mCherry-WPRE-pA (S-0242-9) were purchased from Taitool Bioscience Co. (Shanghai) (titer: 1E + 13 V.G./mL). For stereotaxic AAV injection, 3–4-week-old mice under isoflurane (IsoFlo, Zoetis, B506) anesthesia were fixed in a stereotactic frame (KOPF, Model 940) aimed at the PL area of the dmPFC (anterior–posterior +2.43 mm; medial–lateral ±0.28 mm; dorsal–ventral −1.71 mm, angled 14° toward the midline in the coronal plane). Virus (0.5 μL) was injected into each hemisphere using a Nanoject II injector (Drummond, 3-000-204). AAV-hM3Dq (0.2 μL, 1:5 dilution) or AAV-mCherry (0.2 μL, 1:5 dilution) was injected into each hemisphere. To activate hM3Dq, mice were injected i.p. with 0.1 mg/kg deschloroclozapine (DCZ, MedChemExpress, Cat# HY-42110)^47^ 2 h before collection of blood samples. To identify the region infected with the stereotactic viral injection, brains were fixed in 4% paraformaldehyde (Sigma-Aldrich, 158127) and sectioned on a vibratome at 100-µm thickness. For identification of GFP-GluA1 expression, sections were stained with an antibody against GluA1 (anti-glutamate receptor 1 rabbit polyclonal, Millipore Corp., AB1504) as the primary antibody and Alexa Fluor 633 goat anti-rabbit as the secondary antibody (Thermo Fisher, A-21070), then imaged by confocal microscopy (Leica TCS SP5). To calculate the infection rate in the PL area, standard landmarks were used to identify sub-regions according to Paxinos’ mouse brain atlas from Bregma +2.22 mm to +1.98 mm using the Fiji package for ImageJ. Infection rates were determined by calculating the PL-dmPFC area that contained GluA1-labeled cells as a percentage of the total PL-dmPFC area.

### Electrophysiology

Coronal acute slices were prepared from 8–9-week-old mice. Dissection was performed in ice-cold choline chloride cutting solution that contained 139 mM choline Cl, 3.5 mM KCl, 0.5 mM CaCl_2_, 6 mM MgSO_4_, 1.25 mM NaH_2_PO_4_, 10 mM glucose, and 25 mM NaHCO_3_ (Sigma-Aldrich, C7017, P9333, C1016, M7506, S8282, D9434, and S5761), bubbled with 95% O_2_/5% CO_2_. Brain slices (300 μm) were cut in cutting solution using a vibratome (Thermo Scientific) and then placed in a holding chamber that contained normal ACSF (118.1 mM NaCl, 2.5 mM KCl, 26.2 mM NaHCO_3_, 1 mM NaH_2_PO_4_, 20 mM glucose, 2 mM CaCl_2_, and 1 mM MgCl_2_, bubbled with 95% O_2_/5% CO_2_). Slices were allowed to recover at 34 °C for 40 min and then at room temperature for at least 40 min. Whole-cell recordings (3–5 MΩ pipettes, R_access_ < 26 MΩ, and R_input_ > 10 × R_access_) were made in normal ACSF containing TTX (1 μM) (TOCRIS, 1078) and picrotoxin (50 μM) (TOCRIS, 1128) at 29 °C and bubbled with 95% O_2_/5% CO_2_. For voltage-clamp recordings, the internal solution contained 115 mM CsMeSO3, 20 mM CsCl, 10 mM HEPES, 2.5 mM MgCl_2_, 4 mM Na_2_-ATP, 0.4 mM Na-GTP, 10 mM Na-phosphocreatine, and 0.6 mM EGTA. For current-clamp recordings, the internal solution contained 127 mM K-gluconate, 13 mM KCl, 4 mM Mg_3_-ATP, 0.3 mM Na_3_-GTP, 0.3 mM EGTA, 10 mM HEPES, and 10 mM Na-phosphocreatine (pH = 7.25). CNO (Sigma, Cat# C0832, 5 μM) was dissolved in DMSO and diluted in ACSF to a final concentration of 0.0014% DMSO. Data were acquired using a MultiClamp 700B amplifier (Molecular Devices), digitized with a 1500 A Digitizer (Molecular Devices), and analyzed with Clampfit 10.2 software. mEPSC recordings were analyzed with MiniAnalysis (Synaptosoft) using an amplitude threshold of 5 pA.

### RNA sequencing

Mice were sacrificed, and spleens were dissected for preparation of single-cell suspensions. Twenty-five percent of each spleen single-cell suspension was used for anti-CD8a (BD Biosciences, 553030) staining. A BD Aria II flow cytometer was used to sort 200,000 CD8^+^ T-cells per sample for RNA extraction. cDNA libraries were constructed using pooled RNA from mouse spleen samples and sequenced on the Illumina NovaSeq 6000 platform. The transcriptome was sequenced using the Illumina paired-end RNA-seq approach, generating a total of one million 2 × 150-bp paired-end reads. Raw reads were filtered using Cutadapt (https://cutadapt.readthedocs.io/en/stable/, version1.9) to remove adapters and low-quality bases. The resulting clean reads from all samples were aligned to the mouse reference genome using the HISAT2 package (https://daehwankimlab.github.io/hisat2/, version2.2.1), which initially removed a portion of the reads on the basis of the quality information accompanying each read and then mapped the reads to the reference genome. HISAT2 allows multiple alignments per read (up to 20 by default) and a maximum of two mismatches when mapping reads to the reference genome. It builds a database of potential splice junctions and confirms these by comparing previously unmapped reads against the database of putative junctions. Mapped reads from each sample were assembled using the default parameters of StringTie (http://ccb.jhu.edu/software/stringtie/, version2.1.6). All transcriptomes from all samples were then merged to construct a comprehensive transcriptome using GffCompare software (http://ccb.jhu.edu/software/stringtie/gffcompare.shtml, version0.9.8). After construction of the final transcriptome, StringTie and Ballgown (http://www.bioconductor.org/packages/release/bioc/html/ballgown.html) were used to estimate expression levels of all transcripts and analyze mRNA expression abundance by calculating FPKM (fragments per kilobase of transcript per million mapped reads) values. DESeq2 software was used to analyze differential gene expression between two groups, and edgeR was used to analyze differential gene expression between two samples. Genes with a false discovery rate (FDR) below 0.05 and an absolute fold change ≥ 2 were considered differentially expressed. Differentially expressed genes were subjected to KEGG pathway enrichment analysis.

### Statistical analyses

Sample sizes were based on power analyses of preliminary data. Data sets were tested for normality using the Kolmogorov–Smirnov test. Experimental conditions were compared using two-tailed Student’s *t*-tests for two conditions (unpaired, unless otherwise indicated in the figure captions) or one-way ANOVA with post hoc Tukey’s multiple comparison test for more than two conditions. Where indicated in the figure captions, a two-way repeated-measures ANOVA was used. *P < 0.05 was considered statistically significant for all analyses.

## Supplementary information

Supplementary information, Fig. S1

Supplementary information, Fig. S2

Supplementary information, Fig. S3

Supplementary information, Fig. S4

Supplementary information, Fig. S5

Supplementary information, Fig. S6

Supplementary information, Fig. S7

Supplementary information, Fig. S8

Supplementary information, Table S1
