# Supplementary material for: Social status impacts T-cell responses through synapse strength in the prefrontal cortex
Source: Cell Res. 2026 Mar 23;36(6):395–410. doi: 10.1038/s41422-026-01235-7 (PMC13201679; doi:10.1038/s41422-026-01235-7)
Supplement: Supplementary file 1 — Supplementary information, Fig. S1 [file 41422_2026_1235_MOESM1_ESM.pdf]

Figure S1

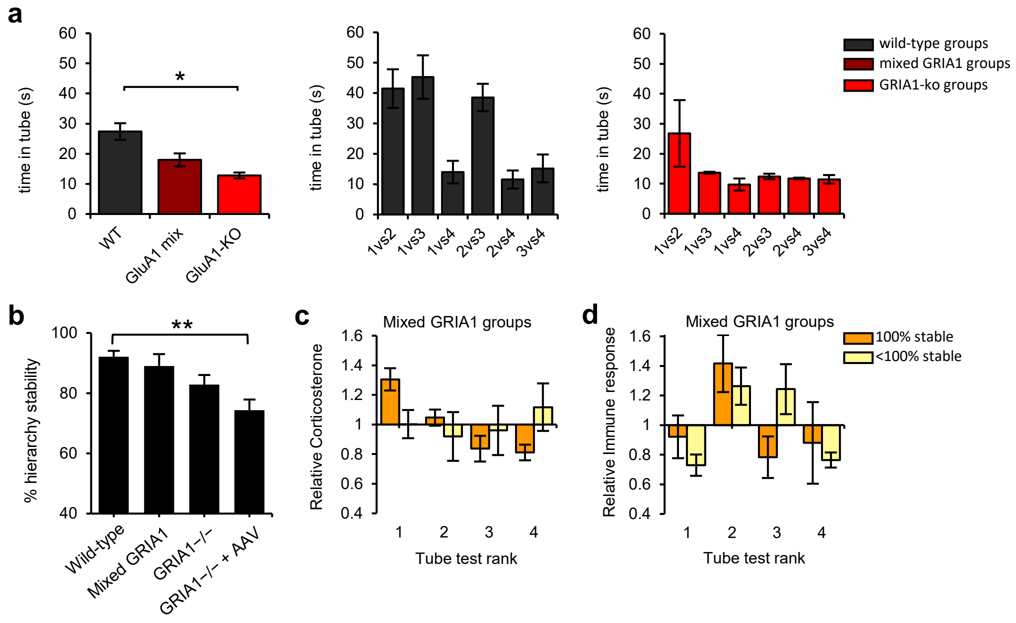

Fig. S1: Tube test behavior in groups of mice with varying GluA1-expression

(**a**) Time spent in tube depends on social rank and on GRIA-genotype. Average time spent in tube for wild-type (black, *n* = 5 groups), GRIA-mixed genotype (brown, *n* = 5 groups) and GRIA-knockout mice (red, *n* = 3 groups).

(**b**) Average group stability quantified as percentage of times social status remained unchanged during the last 5 test days (see methods) in social groups (*n* = 29) of wild-type mice, groups (*n* = 12) of mixed GRIA1-genotypes (1 GRIA1$^{+/+}$, 2 GRIA1$^{+/-}$ and 1 GRIA1$^{-/-}$), groups (*n* = 12) of 4 GRIA1$^{-/-}$ mice, and groups (*n* = 8) of 4 GRIA1−/− mice receiving AAV-injection in PrL-dmPFC expressing either GFP-GluA1 (1 per group) or GFP (3 per group).

(**c**) Blood corticosterone relative to group average of social groups with mixed GRIA1-genotype (as in Figure 5) per rank split between 100% (orange, *n* = 6) and <100% (yellow, *n* = 6) stability of hierarchy.

(**d**) Percentage gB-specific CD8+ T-cells relative to group average of social groups with mixed GRIA1-genotype (as in Figure 5) per rank split between 100% (orange, *n* = 6) and <100% (yellow, *n* = 6) stability of hierarchy.

Data are mean±SEM. **P* < 0.05, ***P* < 0.01. Statistics: One-way ANOVA with Tukey multiple comparisons.
