# Supplementary material for: Social status impacts T-cell responses through synapse strength in the prefrontal cortex
Source: Cell Res. 2026 Mar 23;36(6):395–410. doi: 10.1038/s41422-026-01235-7 (PMC13201679; doi:10.1038/s41422-026-01235-7)
Supplement: Supplementary file 2 — Supplementary information, Fig. S2 [file 41422_2026_1235_MOESM2_ESM.pdf]

Figure S2

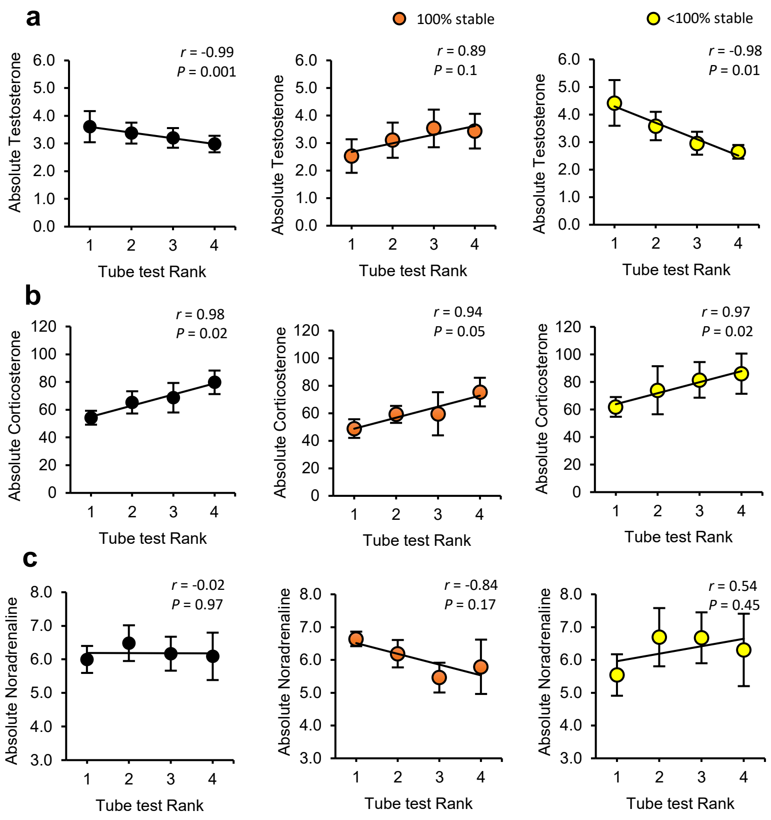

Fig. S2: Correlations between social rank and hormone levels
(**a**) Significant positive correlation between social rank in tube test and blood testosterone concentration in absolute levels (black; *n* = 14). However, only fully stable hierarchies show positive correlation (orange; *n* = 6), but not unstable hierarchies (yellow; *n* = 8).
(**b**) Significant negative correlation between social rank in tube test and blood corticosterone concentration in absolute levels (black; *n* = 29). This negative correlation is present in both stable hierarchies (orange; *n* = 17) and in unstable hierarchies (yellow; *n* = 12).
(**c**) No correlation between social rank in tube test and spleen noradrenaline concentration in absolute levels (black; *n* = 12), neither in stable hierarchies (orange; *n* = 5) nor in unstable hierarchies (yellow; *n* = 7).
Data are mean±SEM. Statistics: Pearson’s correlation.
