# Supplementary material for: Social status impacts T-cell responses through synapse strength in the prefrontal cortex
Source: Cell Res. 2026 Mar 23;36(6):395–410. doi: 10.1038/s41422-026-01235-7 (PMC13201679; doi:10.1038/s41422-026-01235-7)
Supplement: Supplementary file 3 — Supplementary information, Fig. S3 [file 41422_2026_1235_MOESM3_ESM.pdf]

Figure S3

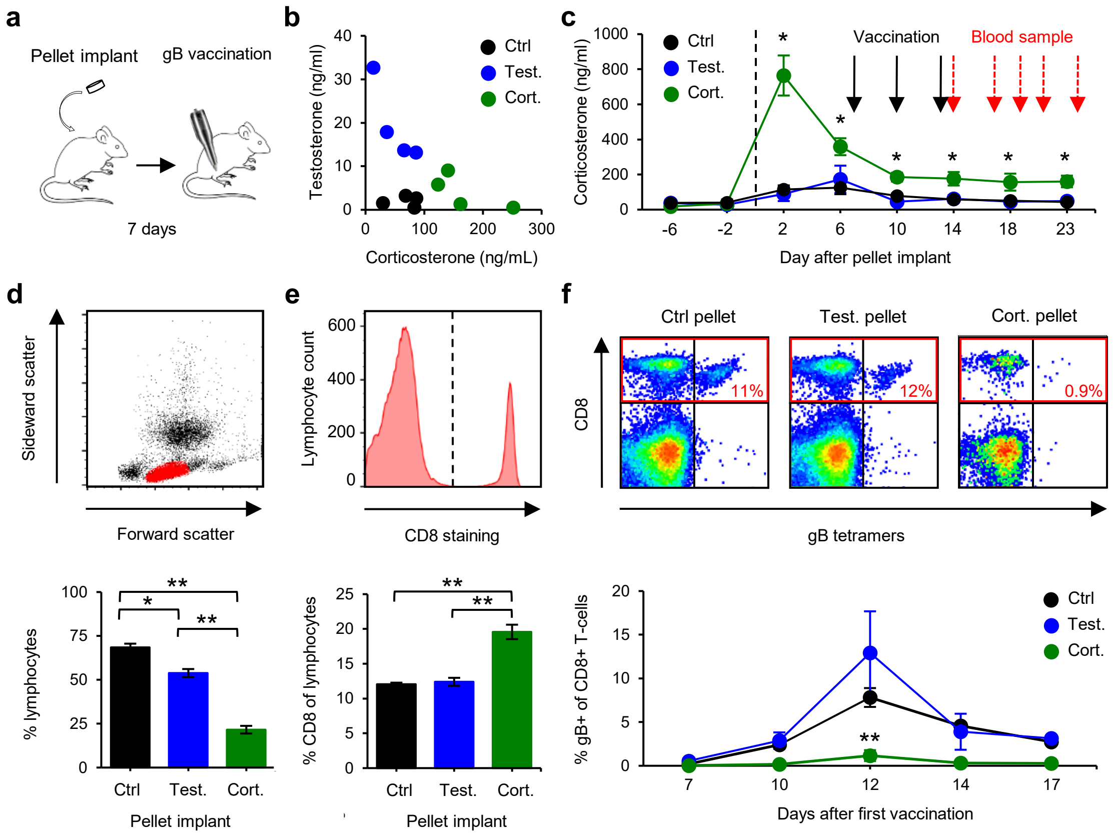

Fig. S3: Effects of chronic testosterone or corticosterone on T-cell response
(**a**) Schematic of experimental design, mice were implanted with pellets and 7 days later vaccinated as illustrated.
(**b**) Blood corticosterone and testosterone levels of mice with control pellet (black, n = 4), pellet containing corticosterone (green, n = 4) or testosterone (blue, n = 4) 24 days after pellet implantation.
(**c**) Corticosterone levels before and after pellet implantation (day 0) and time-point indication of gB-vaccinations and drawing blood samples.
(**d**) Example of forward-sideward scatter FACS dot plot with lymphocyte population in red (top) and percentage lymphocytes among blood leukocytes depending on pellet treatment (bottom).
(**e**) Example of FACS histogram of CD8 immunostaining of lymphocyte population (top) and percentage CD8+ cells depending on pellet treatment (bottom).
(**f**) Example FACS dot-plots of lymphocyte population, immunostained for CD8+ cells (y-axis) and gB-specific cells (x-axis) 12 days after vaccination (top) and time course of percentage gB-specific T-cells among CD8+ lymphocytes depending on pellet treatment (bottom).
Data are mean±SEM. *P < 0.05; **P < 0.01. Statistics: One-way ANOVA with Tukey multiple comparisons (**d**,**e**); Two-way ANOVA with Tukey multiple comparisons (**c**,**f**).
