# Supplementary material for: Social status impacts T-cell responses through synapse strength in the prefrontal cortex
Source: Cell Res. 2026 Mar 23;36(6):395–410. doi: 10.1038/s41422-026-01235-7 (PMC13201679; doi:10.1038/s41422-026-01235-7)
Supplement: Supplementary file 4 — Supplementary information, Fig. S4 [file 41422_2026_1235_MOESM4_ESM.pdf]

Figure S4

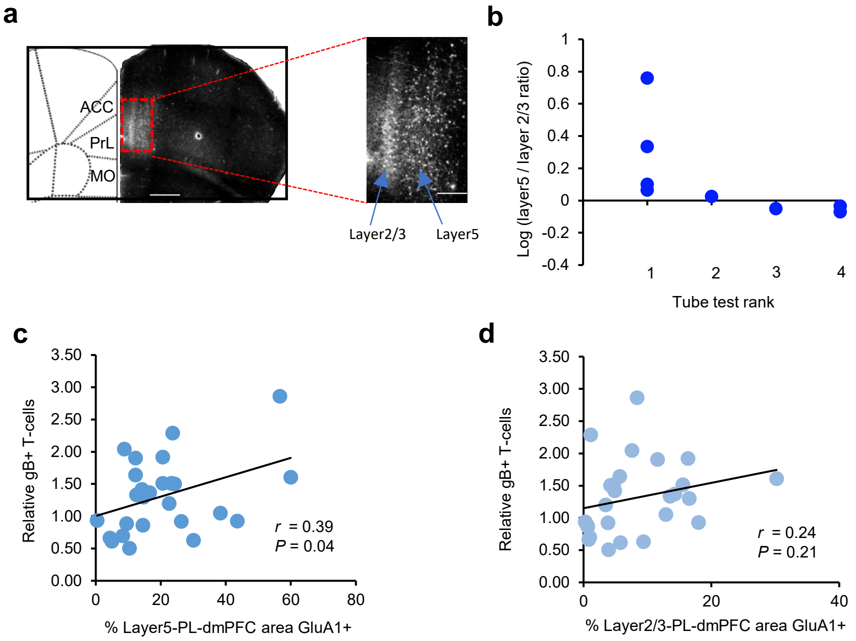

Fig. S4: GluA1 expression in layer 5 dmPFC neurons promotes social status and T-cell response

(**a**) Example picture of AAV-mediated GluA1 expression in layers 2/3 and 5 in PL-dmPFC of a GRIA1$^{-/-}$ mouse (scale bar left: 500 µm; right: 200 µm).

(**b**) Log ratio GluA1-immunostaining of layer 5 over layer 2/3 in dmPFC versus tube test ranks (*n* = 8). When AAV expression of GluA1 in the dmPFC of GRIA1$^{-/-}$ mice was higher in layer 5 than in layer 2/3, mice ended up as dominant in their social group. When GluA1-expression was relatively more in layer 2/3, they became subordinate (3rd or 4th ranked).

(**c**) Significant correlation between GluA1-immunostaining in layer 5 of PL-dmPFC and relative specific CD8 T-cell response (*n* = 26, Pearson's correlation).

(**d**) Nonsignificant correlation between GluA1-immunostaining in layer 2/3 PL-dmPFC and relative specific CD8 T-cell response (*n* = 26, Pearson's correlation).
