# Supplementary material for: Social status impacts T-cell responses through synapse strength in the prefrontal cortex
Source: Cell Res. 2026 Mar 23;36(6):395–410. doi: 10.1038/s41422-026-01235-7 (PMC13201679; doi:10.1038/s41422-026-01235-7)
Supplement: Supplementary file 5 — Supplementary information, Fig. S5 [file 41422_2026_1235_MOESM5_ESM.pdf]

Figure S5

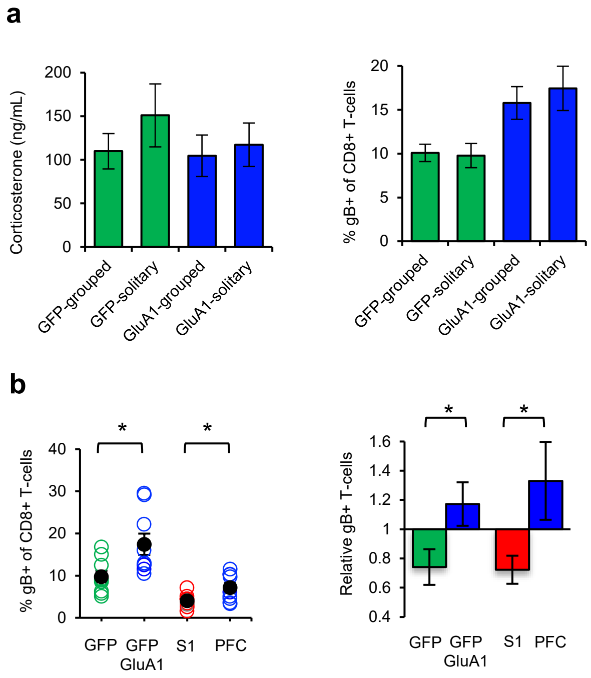

Fig. S5: GluA1-expression in dmPFC promotes T-cell responses in solitary GRIA$^{-/-}$mice
(**a**) Corticosterone levels (left, *n* = 9 each) and percentages gB-specific T-cells of averages day 10+12+14 (right, *n* = 9 each) in absolute levels between social and solitary housed GRIA$^{-/-}$ mice with or without GluA1 expression in dmPFC neurons.
(**b**) Percentage gB-specific CD8+ T-cell taking averages day 10+12+14 (*n* = 9 each) in absolute levels (left) and relative to littermate average in absolute levels, for both GRIA$^{-/-}$ mice GFP (green) or GluA1 (blue) expression in dmPFC neurons, and for GRIA$^{-/-}$ mice with GluA1 expression in somatosensory cortex (red) or dmPFC (blue).
Data are mean±SEM. *P < 0.05. Statistics: unpaired t-test.
