# Supplementary material for: Social status impacts T-cell responses through synapse strength in the prefrontal cortex
Source: Cell Res. 2026 Mar 23;36(6):395–410. doi: 10.1038/s41422-026-01235-7 (PMC13201679; doi:10.1038/s41422-026-01235-7)
Supplement: Supplementary file 6 — Supplementary information, Fig. S6 [file 41422_2026_1235_MOESM6_ESM.pdf]

Figure S6

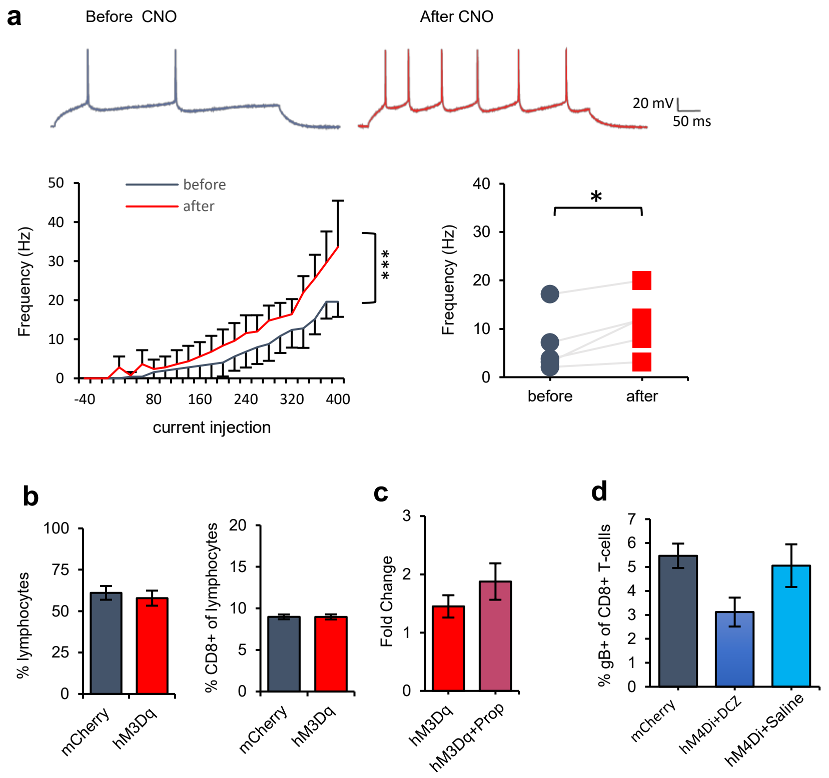

Fig. S6: Effects of hM3Dq expression on dmPFC neurons and on T-cells
(**a**) hM3Dq activation increases dmPFC neuron intrinsic excitability. Top: sample traces before and after CNO application (current injection: 260 pA); We note that CNO was used in this electrophysiology experiment, while DCZ was used for in vivo experiments. Bottom: firing frequency at different current injection step (left, *n* = 5), firing frequency summary of before and after CNO application (5 μM, *n* = 5).
(**b**) hM3Dq expression does not affect blood T-cell levels. Percentage lymphocytes of blood leukocytes (left) and percentage $CD8^+$ cells of lymphocytes (right)
(**c**) Injection of β-adrenergic blocker propranolol did not prevent a boost in T-cell expansion upon activation of hM3Dq in dmPFC neurons by DCZ, as measured by the fold change in gB-specific $CD8^+$ cells percentage relative to control (mCherry expressed in dmPFC and injected with DCZ).
(**d**) Chemogenetic inhibition of dmPFC neurons show a trend to suppress T-cell expansion.
Data are mean±SEM. *$P < 0.05$. Statistics: Two-way ANOVA (**a**,left); paired Student's t-test (**a**,right); unpaired Student's t-test (**b**,**c**); One-way ANOVA (**d**).
