# Supplementary material for: Social status impacts T-cell responses through synapse strength in the prefrontal cortex
Source: Cell Res. 2026 Mar 23;36(6):395–410. doi: 10.1038/s41422-026-01235-7 (PMC13201679; doi:10.1038/s41422-026-01235-7)
Supplement: Supplementary file 7 — Supplementary information, Fig. S7 [file 41422_2026_1235_MOESM7_ESM.pdf]

Figure S7

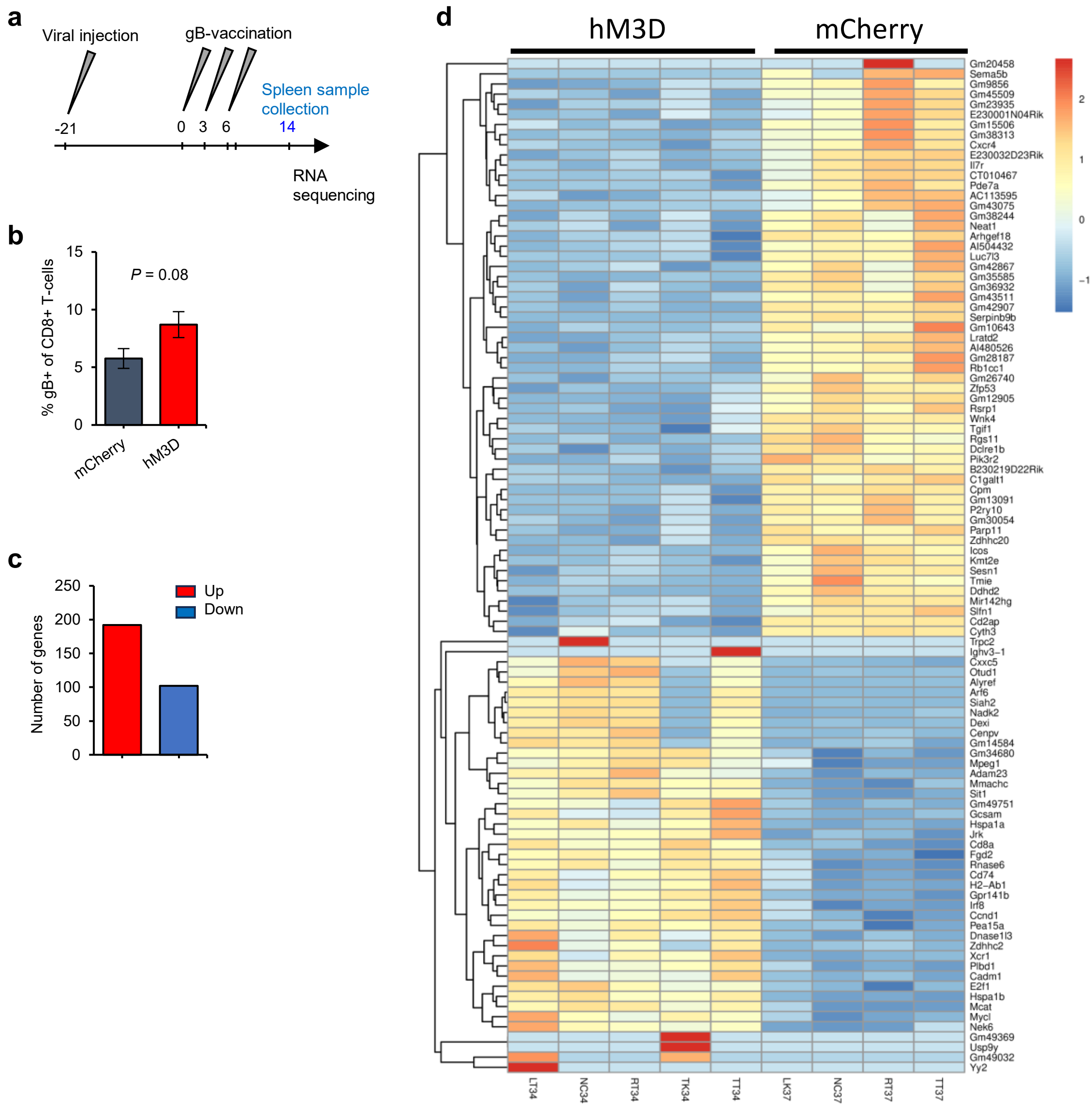

Fig. S7: Effects of chemogenetic activation of dmPFC neurons on splenic CD8^{+} T-cells.
(**a**) Schematic of experimental design (hM3Dq *n* = 5, mCherry *n* =4). Mice were injected with AAV-mCherry or hM3Dq-mCherry 21 days before gB vaccination (day 0, day 3, day 6), spleen samples were collected at day 14 for RNA sequencing.
(**b**) hM3Dq activation tends to increase splenic T-cell expansion upon vaccination. Data are mean ± s.e.m $^{*}P < 0.05$. Statistics: unpaired Student's t-test.
(**c**) Bar plot of differentially expressed genes in splenic CD8+T-cells at day 14 after vaccination.
(**d**) Heatmap of differentially expressed genes in splenic CD8+T-cells at day 14 after vaccination upon hM3Dq activation on dmPFC neurons.
