# Supplementary material for: Social status impacts T-cell responses through synapse strength in the prefrontal cortex
Source: Cell Res. 2026 Mar 23;36(6):395–410. doi: 10.1038/s41422-026-01235-7 (PMC13201679; doi:10.1038/s41422-026-01235-7)
Supplement: Supplementary file 8 — Supplementary information, Fig. S8 [file 41422_2026_1235_MOESM8_ESM.pdf]

Figure S8

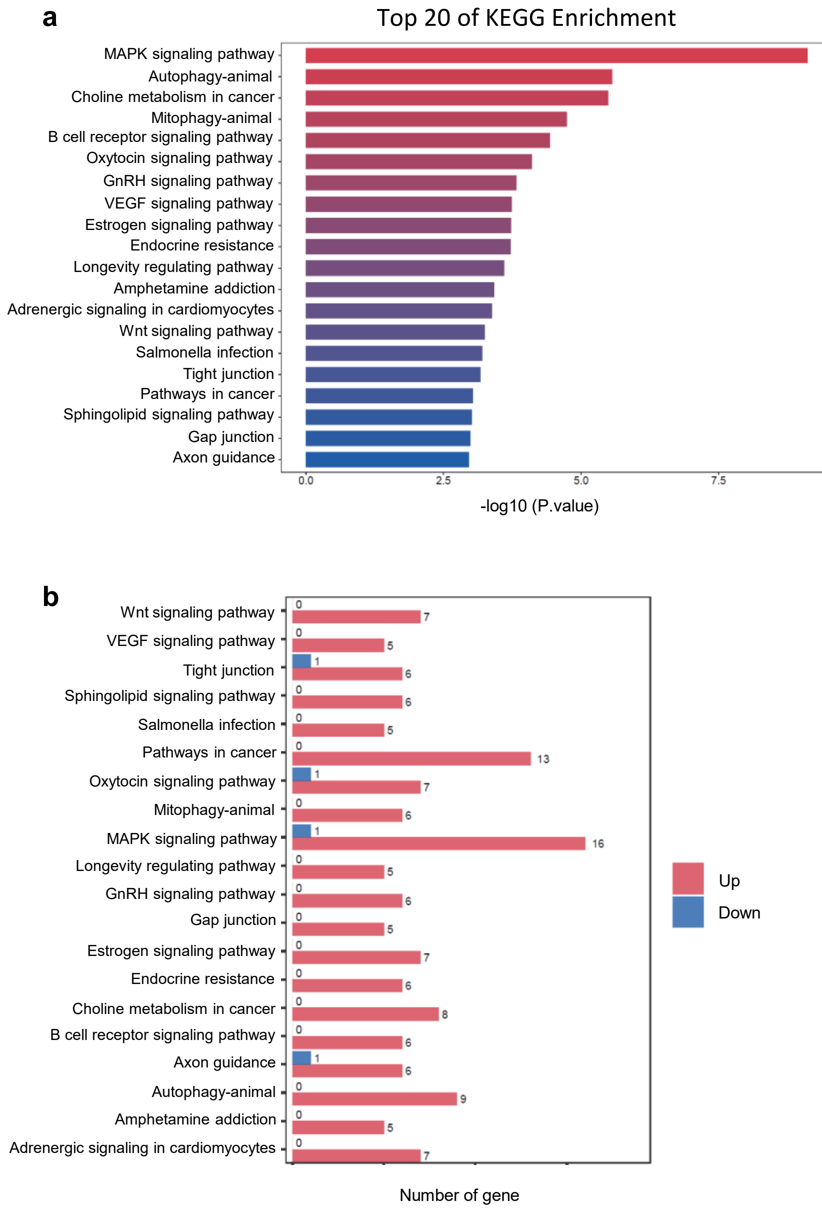

Fig. S8: KEGG pathway analysis of differentially expressed genes in CD8⁺ splenocytes upon hM3Dq activation of dmPFC neurons.

(**a**) Top 20 KEGG Enrichment upon activation of dmPFC neurons on splenic CD8 T-cells.

(**b**) Number of genes that involved in top 20 KEGG enrichment on splenic CD8 T-cells RNA-sequencing.
