# Supplementary material for: Social status impacts T-cell responses through synapse strength in the prefrontal cortex
Source: Cell Res. 2026 Mar 23;36(6):395–410. doi: 10.1038/s41422-026-01235-7 (PMC13201679; doi:10.1038/s41422-026-01235-7)
Supplement: Supplementary file 9 — Supplementary information, Table S1 [file 41422_2026_1235_MOESM9_ESM.pdf]

DEG_DEseq2_treatment_hm3d vs mCherry

	baseMean	log2FoldChange	lfcSE	stat	pvalue	padj
Hspa1b	4840.81149160992	3.62465553387172	0.386664327852287	9.37416584044549	6.97249079585663e-21	5.49327687351565e-17
Gm35585	296.984260875575	-1.73448223295098	0.184460299958613	-9.40301101830659	5.30235311723706e-21	5.49327687351565e-17
Hspa1a	5629.75581896186	3.3491230025993	0.398197672663461	8.41070461360991	4.07554921406361e-17	2.14061429886667e-13
Cpm	5177.67275107007	-0.617807453065774	0.0772103779258764	-8.00161156650318	1.22801250239028e-15	4.83744825004093e-12
Sema5b	5044.54220206048	-12.7839331982656	1.73169635782683	-7.38231800308727	1.55557095925703e-13	4.08518860083551e-10
Lratd2	1343.81093576171	-0.842655434763367	0.113824596553069	-7.40310495517978	1.33036517398258e-13	4.08518860083551e-10
Gm42907	87.9541180136504	-1.80728947175568	0.248823171838687	-7.26333874039525	3.77622672134424e-13	8.50028634974589e-10
P2ry10	3485.08903664389	-0.67674814144152	0.0935423276701387	-7.23467288335991	4.66652541181416e-13	9.19130511424447e-10
B230219D22Rik	2877.89814139188	-0.508761219112423	0.0705432525290748	-7.21204652284404	5.51171349240071e-13	9.64978549997311e-10
Gpr141b	293.965006078583	1.27849467051641	0.17827335119222	7.17154113032798	7.41581496394303e-13	1.1685099638685e-09
Mmachc	950.969023795653	0.736715718080788	0.103813239958301	7.09654874827825	1.27910842365017e-12	1.68116068247838e-09
Zfp53	10769.4446847892	-0.533316224022567	0.0751528725887729	-7.0964183490471	1.2803153004849e-12	1.68116068247838e-09
Gm49032	54.446702985712	22.7537675953449	3.22657781187806	7.05198167283647	1.76387439971566e-12	2.13795145510151e-09
CT010467.1	14062.5535454627	-1.2814991653309	0.187264768138272	-6.84324754768967	7.74176985180962e-12	8.71336196821173e-09
Dclre1b	2842.15225733713	-0.534388759530622	0.0821540465177336	-6.50471622740177	7.78401781705333e-11	8.17685124955395e-08
Gm26740	8738.83441235981	-0.498771524949755	0.0768385368633398	-6.49116374817025	8.51758382366463e-11	8.38822301934272e-08
Gm12905	480.04016427449	-1.02359860997096	0.158358667924898	-6.46379906691566	1.02106436276611e-10	9.46406539065031e-08
Plbd1	2604.70321800916	0.914964339469027	0.14175927904079	6.45435237580287	1.0868266102688e-10	9.51395938778082e-08
Fgd2	761.533946414151	1.05520519938429	0.168472658183617	6.26336172742184	3.7676524811626e-10	3.12457369187785e-07
Irf8	4647.23238326227	0.65099427606698	0.106109323224964	6.13512796313664	8.50906741035048e-10	6.70386875924463e-07
Jrk	519.380813095709	0.87961350824958	0.143710700230418	6.12072383503284	9.31512280410413e-10	6.79276610995287e-07
Mcat	3193.91130020177	0.459603631242099	0.0751249141275391	6.11785898965332	9.48409306460387e-10	6.79276610995287e-07
Icos	3140.25179728585	-0.46229500651922	0.0757802446269096	-6.1004686484617	1.05757925091700e-09	6.94344844029129e-07
E230032D23Rik	757.240001603974	-0.932325198486415	0.152827392013951	-6.10051108116342	1.05729851750037e-09	6.94344844029129e-07
Sesn1	10343.5518461218	-0.573836940931059	0.0943510014989411	-6.08193799551241	1.18738489575168e-09	7.48384952094369e-07
Pde7a	3111.13502099213	-0.504270411857771	0.0838371647754246	-6.014879119643	1.80020982115467e-09	1.09099639045901e-06
Wnk4	170.200131485314	-1.2380505320818	0.206118621099377	-6.00649531555371	1.8957645067391e-09	1.10635412343289e-06
Il7r	54137.8572855738	-0.500777338739302	0.0840004652051194	-5.96160196870888	2.4977697171143e-09	1.39373517976048e-06
Dnase1l3	3511.833174813	0.91442229191793	0.153640266174075	5.95170989148044	2.65355431825947e-09	1.39373517976048e-06
Gm43511	528.170060363923	-0.790615888049736	0.132731559873776	-5.95650264941956	2.57692936887886e-09	1.39373517976048e-06
Xcr1	569.117324087905	0.984096473430753	0.167065164766602	5.89049473482747	3.85041139619682e-09	1.95712685064107e-06
Cadm1	550.166330049162	1.21765672310358	0.208467484730776	5.84099110072786	5.18911768660776e-09	2.47772507235995e-06
Otud1	405.236100723606	2.60393898849577	0.445515336269816	5.84477968883827	5.07239127262067e-09	2.47772507235995e-06
Gm34680	274.196361405399	1.21129670448808	0.207966569522635	5.82447797868897	5.72913913670531e-09	2.6551189816784e-06
Zdhhc20	14661.6378405296	-0.239520490319298	0.0416173772995875	-5.75529996989196	8.64880781412472e-09	3.89369327791895e-06
Gm28187	222.866328839277	-1.43593060052262	0.250523035880406	-5.73173079863242	9.94109613843578e-09	4.35116255148146e-06
Cd74	40586.7756971334	0.642016906157611	0.113104795647682	5.67630136707443	1.37638207991304e-08	5.70727695610259e-06
Arf6	3757.58289689011	3.97045271019285	0.699110738269706	5.67929012221968	1.3525489715899e-08	5.70727695610259e-06
AC113595	3095.48092932884	-1.2179040517486	0.215043966261918	-5.66351185257262	1.48305921479577e-08	5.99193949936845e-06
Rnase6	693.594517535601	0.807425491317026	0.142957552454698	5.64800863929798	1.62317022334784e-08	6.39407330232299e-06
Tmie	6756.24307602421	-0.520828969955957	0.0926511054166879	-5.62140049612562	1.89415586266661e-08	7.27956437269215e-06
Parp11	3037.7643756387	-0.354302247316593	0.0636576897243586	-5.56574152864708	2.61039935015116e-08	9.79334822865043e-06
Arhgef18	22213.2201097009	-0.41555816710904	0.0750905728647373	-5.5340923801127	3.12844065002124e-08	1.10476085609974e-05
E2f1	323.79941572251	1.57883129020605	0.285227228633514	5.5353456181937	3.10615228493261e-08	1.10476085609974e-05
H2-Ab1	20122.5097675443	0.765663341828181	0.138391057350036	5.53260706644915	3.15505734114923e-08	1.10476085609974e-05
Siah2	482.889710650259	3.71904835684066	0.678347487809043	5.48251216917247	4.19328145957608e-08	1.43638121649001e-05
Sit1	13090.5102400497	0.319232937109396	0.0582728649472599	5.47824338821025	4.2956899307213e-08	1.44015289868884e-05
Alyref	1283.80489624893	4.24284788794786	0.775506503694929	5.47106680309276	4.47334627153754e-08	1.44042941384193e-05
Ddhd2	3419.33722554353	-0.307005408707116	0.0561167951739985	-5.47082932578741	4.47934513411529e-08	1.44042941384193e-05
Kmt2e	23579.9751031123	-0.375400060281293	0.0690244480655128	-5.43865356119895	5.36847276121145e-08	1.69182050596817e-05
Cd8a	81420.6247088876	0.238646906178239	0.0441491705586202	5.40546749029791	6.4639574784395e-08	1.99710937230924e-05
Pea15a	12775.9236363891	0.237131538451098	0.043901157340036	5.40148717753378	6.60906641632523e-08	2.00267422157763e-05
Adam23	55.6106035505513	2.12637137994897	0.394839522982842	5.38540661756744	7.22810548173668e-08	2.14892939765519e-05

**Nadk2**	537.351370436426	2.09197647189073	0.389019946353309	5.37755580787313	7.55038132881678e-08	2.20317330737344e-05
**Rgs11**	4792.49244383222	-0.334850733387149	0.0625793106909579	-5.35082169633952	8.75557576732435e-08	2.50839286119509e-05
**Gm30054**	3304.73094697694	-0.386563690608706	0.0726255443740074	-5.32269594590545	1.02240558238189e-07	2.87679370742706e-05
**Mycl**	248.206461206158	1.0648557113487	0.201091469808502	5.29537982074902	1.18769283979103e-07	3.22663380630814e-05
**C1galt1**	895.122748867211	-0.443845015318341	0.0837910424997919	-5.29704610513043	1.17690987257511e-07	3.22663380630814e-05
**Nek6**	688.459540618975	0.805339167376329	0.152680497298833	5.27466953294031	1.32995523450493e-07	3.43542698854004e-05
**Cxxc5**	2131.47587916406	0.902475390985855	0.170955628812391	5.2790036646073	1.29888207689114e-07	3.43542698854004e-05
**Gm43075**	123.107640293605	-1.51478523391232	0.287118664213226	-5.27581596989939	1.32166671338009e-07	3.43542698854004e-05
**Pik3r2**	1810.78198890802	-0.397488790707616	0.0761644964666614	-5.21881991147416	1.80066740859425e-07	4.57630908987413e-05
**Gm15506**	331.673025381068	-1.25182153424986	0.240056486339176	-5.21469572990902	1.84119201634349e-07	4.60502580976577e-05
**Gm9856**	1005.64511264602	-0.75363109987511	0.145169238934731	-5.19139664439478	2.08722441209985e-07	5.13881172835271e-05
**L3mbtl2**	9038.29806415378	0.308832142492906	0.0595700104133859	5.1843560266276	2.16762514584469e-07	5.25465683431919e-05
**Gm23935**	2309.48237824897	-1.15025484841849	0.222930211348543	-5.15970823990341	2.47334981317473e-07	5.90493530396883e-05
**Rb1cc1**	8432.27581030057	-0.329695926258486	0.0640476091933836	-5.14766952913123	2.63742609335726e-07	6.20267506761647e-05
**Luc7l3**	5870.54027253689	-0.321537280173178	0.0626279544617324	-5.13408561618673	2.83519019365104e-07	6.47450606976223e-05
**Msto1**	3201.39353439492	0.26728882695933	0.0520496113488635	5.13527037056669	2.81738692250337e-07	6.47450606976223e-05
**Tgif1**	2795.06121526352	-0.589032036609187	0.11539150277021	-5.10463961789439	3.31425506255932e-07	7.46038814582102e-05
**AI480526**	190.488281634773	-0.890084818603783	0.174745079746826	-5.0936187725191	3.51292802314855e-07	7.79622631841573e-05
**Rsrp1**	127072.776760146	-0.364470603613512	0.0718232310118639	-5.07455037149899	3.88413333601214e-07	8.5003179132699e-05
**Nup153**	6494.27362470757	-0.267174969387252	0.0527711625807215	-5.06289716430953	4.12932620608737e-07	8.91312233278339e-05
**Dexi**	1004.11150391377	3.65492631267641	0.723047455559782	5.05489132777153	4.306359057993e-07	9.02105718359697e-05
**Gm13091**	199.84345315536	-1.1307966153584	0.223769071798917	-5.05340888384501	4.33993406420285e-07	9.02105718359697e-05
**Gm49751**	61.0741054802611	2.64067571044645	0.522604004389806	5.05291901375633	4.35108425432106e-07	9.02105718359697e-05
**Ccnd1**	426.366030721949	0.967250756083378	0.191708500510436	5.04542445174842	4.52515672678848e-07	9.26011617454625e-05
**Cyth3**	5910.94186764279	-0.468199318530705	0.0930938881291886	-5.02932392168403	4.92212263606687e-07	9.94331876621868e-05
**Cd2ap**	3237.60351389916	-0.380348101904527	0.0759576518568204	-5.00737045717896	5.5178663660849e-07	0.00011005698776
**Slfn1**	10818.0165558219	-0.416146518768431	0.0831519183774471	-5.00465325260975	5.59626746736957e-07	0.000110225483104178
**Gm38244**	1177.3225380511	-0.709241656042015	0.141863696845777	-4.99945843659389	5.74915631624076e-07	0.000111838834660501
**Gm45509**	199.519516434698	-1.37119940127554	0.274601136179809	-4.99342216988382	5.93186967057563e-07	0.000113985939511293
**Slc2a3**	6595.08819908376	0.408085471888546	0.0819260690082675	4.9811430821533	6.32097804633674e-07	0.000119239607292542
**Tmem221**	1626.76733926712	0.418469044661335	0.0840290006219221	4.98005499963261	6.35662055757667e-07	0.000119239607292542
**H2-Aa**	8845.83051040912	0.666366008836711	0.133876039464665	4.97748522813591	6.44156962783184e-07	0.000119411544265584
**Ptpn6**	65442.6412907243	0.330387165700159	0.0667316443255645	4.95098193726945	7.38399576644391e-07	0.00013429352481683
**Gpr108**	7139.46856541016	0.25471944920089	0.0514566949976345	4.95017119176813	7.41482303678633e-07	0.00013429352481683
**Cst3**	10223.3634269205	0.585024362133245	0.118432329628416	4.93973532369727	7.82286829021284e-07	0.000138499927695375
**H2-Eb1**	6363.6676527632	0.693214310185738	0.140273316007647	4.94188296046234	7.73716601559334e-07	0.000138499927695375
**Gm10643**	89.9591262154961	-1.50707093744625	0.306373711419267	-4.91906087655104	8.69604232450993e-07	0.000150855840057087
**Neat1**	5330.66148333841	-0.538153125515393	0.109409703513868	-4.91869649794982	8.712243095256e-07	0.000150855840057087
**Mir142hg**	290.581724310119	-0.895166692362394	0.182838072169929	-4.89595346165337	9.78302184501495e-07	0.000167555516534674
**AI504432**	429.244276181044	-0.890762412508042	0.182325671806446	-4.88555672759928	1.03137004355005e-06	0.000172886146555513
**Gm42867**	239.383917778648	-1.10631419188192	0.226432771756902	-4.8858395509536	1.02989045600735e-06	0.000172886146555513
**Map3k2**	2123.92752003124	-0.495242826582941	0.10185546502769	-4.86221162947226	1.16081417785446e-06	0.000192536305267924
**Mtch1**	1757.96156016978	3.3342305461684	0.6874643028609	4.85004171459219	1.23435512401175e-06	0.000200584362777206
**Zdhhc2**	158.000862045241	2.14451738806201	0.442171190758922	4.84997085491088	1.2347961661096e-06	0.000200584362777206
**Mpeg1**	1466.54864571881	0.875511050875403	0.180743391567762	4.84394501664072	1.27286169891183e-06	0.000204657977446466
**Cblb**	5303.54122705658	-0.570647517157043	0.117997442689835	-4.83610071666583	1.32410849890191e-06	0.000207371461549683
**Dusp2**	3218.85428029658	3.32198255576231	0.687003808226954	4.83546454325459	1.32835051506786e-06	0.000207371461549683
**E230001N04Rik**	1079.38177134668	-0.940029096085434	0.194408290522932	-4.83533440655686	1.32921987792841e-06	0.000207371461549683
**Cenpv**	203.23901005684	2.7696633018903	0.573655915778477	4.82809158889566	1.37847695779244e-06	0.000212947661018975
**Lrrc47**	3095.12715020202	2.96624507213446	0.615082439116599	4.82251627341966	1.41758460214055e-06	0.000214777697845468
**Cxcr4**	658.387886105472	-0.647741446033002	0.134288298150906	-4.82351370113505	1.41051073191219e-06	0.000214777697845468
**Gm36932**	328.369925567532	-0.712921820117951	0.147961972715924	-4.81827733864233	1.44803001005627e-06	0.000217301036842445
**Gcsam**	204.623765101439	1.27746637868982	0.265443601865653	4.81257174673352	1.49000354372432e-06	0.00022149043243834
**Otulin**	3233.79131640714	2.97227472790601	0.620363041827253	4.79118601126092	1.65798326277832e-06	0.000242326838383579
**Gm38313**	23.1932571379795	-2.91236233093459	0.607903554516381	-4.79082958028027	1.66093155711281e-06	0.000242326838383579
**Gm43813**	578.243887278452	-1.26556815360174	0.265772428711998	-4.76184892366381	1.91827271942643e-06	0.000277304800366993

**Gm14584**	159.442384100324	1.93941154438947	0.407811712066332	4.75565435470871	1.97804656288863e-06	0.000283346179013056
**Wdr38**	304.387575040023	-1.23407241189528	0.261152629564988	-4.72548338475826	2.29568605722501e-06	0.000325884010844095
**Aplp2**	7733.60993925994	0.302467178494531	0.0641909813216427	4.71198869789752	2.45310988246751e-06	0.000345121896589648
**Dcaf17**	4646.29846866551	-0.26006959822843	0.0553200564870537	-4.70118099552723	2.5866111203623e-06	0.00036068346392521
**Gm26981**	29.4084064891948	-1.82758253640647	0.389497834387994	-4.69215070034496	2.70347861501508e-06	0.000373672916989409
**Man1a2**	5908.05095970213	-0.428517839508895	0.0913950413629706	-4.6886333560161	2.75035657338793e-06	0.000376844682842379
**Kif13a**	121.511833673408	-1.23358332202657	0.263242602386317	-4.68610821669452	2.78449073094036e-06	0.000378234659029546
**Junb**	27420.0489906261	1.01258624848523	0.216314980135033	4.68107316401819	2.85377060394301e-06	0.000384332165866069
**Slc38a2**	9345.89458199266	-0.386762868598488	0.0826861293838868	-4.67748183982424	2.90419284937707e-06	0.000387808192607071
**Tbrg4**	5434.56177177011	0.351095937794377	0.0751509877790875	4.67187389241579	2.98464195254458e-06	0.000395201707951638
**H2-DMb1**	945.324362791866	0.863959245856742	0.185100934103387	4.66750343558062	3.04881653352259e-06	0.000400335017655961
**Tagap**	16885.3682641176	-0.522864080165274	0.112410107956972	-4.65139736691128	3.29693347144002e-06	0.000425817874667872
**Zfp36l2**	4402.26132325137	-0.566007833252072	0.121675266972316	-4.65179035424557	3.29065530028748e-06	0.000425817874667872
**St3gal5**	103.545376930359	1.55092947120279	0.333908513057426	4.64477367468634	3.40449470710753e-06	0.000436135147153605
**Irgm2**	4848.99454248333	-0.679017370166403	0.146361748683779	-4.63930894699441	3.49576134425228e-06	0.000440689896546612
**Gm38248**	798.000584456172	-1.0885606249903	0.234639197625452	-4.63929571873126	3.49598509032979e-06	0.000440689896546612
**Pdxp**	959.611007831351	3.27623128043863	0.707633851210731	4.6298396760318	3.65948960598147e-06	0.000457639505725794
**E430021H15Rik**	797.501473997555	-0.667542060337573	0.144524852258951	-4.61887384698038	3.85828330464793e-06	0.000478700551427853
**Ccng2**	8059.74161624905	-0.439807729649704	0.0954278603741419	-4.60879797498718	4.05003624409096e-06	0.000488006049158469
**Rps6ka3**	1316.32764881406	-0.711603532122144	0.154413369449342	-4.60843212384921	4.05716776288376e-06	0.000488006049158469
**Dapk3**	1689.46450771112	2.94027847155145	0.637828414009029	4.60982672921471	4.03004711658823e-06	0.000488006049158469
**Ppp2r2d**	1317.56343745524	1.42707341839695	0.309386032526255	4.61259807608103	3.97666802029977e-06	0.000488006049158469
**Cep97**	3597.67326443937	-0.336682322685694	0.0732041215410775	-4.59922632220604	4.2406288630869e-06	0.000502402924779401
**1300014J16Rik**	767.868766185782	-1.13472169080819	0.246679367332839	-4.5999862212924	4.22518886953288e-06	0.000502402924779401
**Pabpc1**	13562.5522654888	-0.346309771594798	0.0753324286138566	-4.59708757525837	4.28437570830593e-06	0.000503797821162512
**Btbd2**	1949.89381090534	2.87131136814202	0.625287134409582	4.59198856034869	4.39042439834218e-06	0.000512443831442057
**Mpp6**	1007.67617394821	1.46082905433499	0.318365274762114	4.58853138247107	4.46375210386328e-06	0.000517171631621865
**Cables1**	30.6420161578793	5.79408068276526	1.2661399168572	4.57617724994191	4.7354936619624e-06	0.000544650902420011
**Gm20045**	64.2770333959878	-2.35144982907135	0.51578780754632	-4.55894806869815	5.14104808137227e-06	0.000587010830566543
**Klhl22**	2275.29260616114	0.425424082501221	0.0935885779643033	4.54568379769043	5.47571897539427e-06	0.000617869359621966
**Trnp1**	215.880147185203	2.19797851628653	0.48358815406233	4.54514548758617	5.48973220454879e-06	0.000617869359621966
**Zfr**	10306.6641242763	-0.243793145001439	0.053693855309645	-4.54042913468437	5.61398459935482e-06	0.000627372732851304
**Ddx56**	7964.74919165355	0.301502142249755	0.0665049377691152	4.53353017630779	5.80059747482696e-06	0.000643662073315834
**Smarca5**	5782.46445156177	2.0252444454047	0.448070628046772	4.51992234847693	6.18623175534834e-06	0.000681653522860307
**Gm14121**	159.565998754867	2.51224791274998	0.556393757004151	4.51523382698781	6.32469409570341e-06	0.000692070867124991
**Zfp949**	1358.66290990433	-0.309237659832227	0.0686428741205464	-4.50502202587209	6.63660585005246e-06	0.000721193092270873
**Ube2q2**	1907.08955648212	1.16251062528543	0.258555844717749	4.49616842564312	6.91889378130289e-06	0.000746719241862942
**Amn1**	1657.87287915257	-0.641710639879437	0.142848492933053	-4.49224648229352	7.04757889908915e-06	0.000755433338183318
**Cst7**	12000.426414975	0.502148910863782	0.111962438954723	4.48497652919877	7.29219611046622e-06	0.000776372527787947
**Zfp945**	4352.39131070905	-0.391825311432964	0.0874178986806226	-4.48220921969859	7.38742642464332e-06	0.000781232739416811
**Gm10101**	311.927451610776	-1.1037859082812	0.246484580439758	-4.4781134232085	7.53055895753411e-06	0.000791060116625767
**Cd81**	1028.51215045817	0.62083607002537	0.138783106787025	4.47342680531066	7.69759151652277e-06	0.000797966773196376
**A130010J15Rik**	908.679953296583	-0.589203783362021	0.13169703367931	-4.47393359516941	7.67935997215737e-06	0.000797966773196376
**Gm13140**	58.1938020862722	1.77853259805254	0.397986260861779	4.4688291354616	7.86489223270698e-06	0.000809981090920025
**Btbd1**	3338.68255893757	2.8099926449408	0.629340083260484	4.4649827965554	8.00751857171873e-06	0.000819314741133585
**Ube2j1**	7024.65622731778	0.350171363719264	0.0785448573992575	4.45823412651041	8.26376139047357e-06	0.000834692873267257
**Fcho2**	1951.09435250836	-0.408957369389875	0.091723022356977	-4.45861201344034	8.24920848115322e-06	0.000834692873267257
**Tasor**	5172.38175853536	-0.558080594363708	0.125580133595976	-4.44401975362758	8.8293492698319e-06	0.00088614048690918
**Dnaaf5**	5055.7415539826	0.913895006717786	0.205816547840505	4.4403378460414	8.98177607663872e-06	0.00089573320025061
**Prkaca**	1325.17766080141	3.07695954173602	0.69513921550614	4.42639326497447	9.58217616498014e-06	0.000949599684475422
**Smarcb1**	6324.00273301755	0.217760094017172	0.0493540363242396	4.41220435521343	1.02323464533258e-05	0.000988685874431151
**Gtf2i**	35387.1511507355	-0.240981740246699	0.0545946946565656	-4.41401388473043	1.01471446542324e-05	0.000988685874431151
**Ctdsp2**	2157.848173109	-0.632651035576638	0.14342636565501	-4.4109814306972	1.02903143622967e-05	0.000988685874431151
**Gm16741**	181.567391597215	-1.15075803738938	0.260817931166505	-4.41211243507159	1.02366927026878e-05	0.000988685874431151
**A130071D04Rik**	4593.53399986065	-0.501048587652927	0.113560253842692	-4.41218270211865	1.02333701153831e-05	0.000988685874431151
**Krit1**	6125.48232717077	-0.337451471800273	0.0767244101791244	-4.39822829543353	1.09138145348975e-05	0.00104223621591745

**Itga4**	12465.8258121139	-0.609481151912446	0.138668884992579	-4.39522645577674	1.10657332486571e-05	0.00104408837604246
**Coq9**	5383.80230834622	0.28437156622595	0.064688256658481	4.39603076223368	1.10248316418733e-05	0.00104408837604246
**Bach2**	6070.54557063352	-0.416625538491331	0.0948580918130977	-4.3920927622308	1.12264778326428e-05	0.00105295006671996
**Ankrd13a**	8714.32411005555	2.81865836822135	0.642086138938288	4.38984459761442	1.13431693954262e-05	0.00105759952759604
**Ttc14**	7012.76654414157	-0.286171464153794	0.0652594779803984	-4.38513259698078	1.15915148090278e-05	0.00106811402833831
**4930439D14Rik**	68.5899356710101	-1.72536876317524	0.393352568687757	-4.38631624786677	1.15286469814101e-05	0.00106811402833831
**Ctnna1**	2107.1325928125	0.3577864074084	0.0816824997122464	4.38020884116942	1.18565620021335e-05	0.00108618515969545
**2510039O18Rik**	3617.49956528345	2.87808146780327	0.657434118496503	4.3777488676511	1.19911403403075e-05	0.0010921641522672
**Nudt4**	1013.92438211106	0.97864318952925	0.223622030102123	4.3763272745638	1.2069575142284e-05	0.00109299020412051
**Gm47004**	118.301697494084	-1.31903891554368	0.301501866722654	-4.37489468931559	1.21491116157607e-05	0.0010939060098831
**5730405O15Rik**	137.679930771279	-1.01147692407825	0.23152118277721	-4.36883101556882	1.24913372947674e-05	0.00111832955541847
**2810402E24Rik**	212.263379128884	-1.02060142286918	0.233918283022911	-4.36306820347692	1.2825093822176e-05	0.00114172318280242
**Bcdin3d**	1996.60204131582	-0.3299986466863	0.075675544028157	-4.36070398864282	1.29644654878901e-05	0.00114764653198137
**BC043934**	2479.10978818043	-0.425056630254415	0.0975564022206071	-4.35703470586402	1.31836369870434e-05	0.00115760463922028
**Gm11346**	14685.5899272576	-0.322566008088075	0.0740447253284981	-4.35636713698399	1.32238900209209e-05	0.00115760463922028
**Yaf2**	1271.19131243064	1.33210847056496	0.30590167735196	4.35469488790112	1.33252385719015e-05	0.0011600319567815
**Actr5**	2046.40338089932	3.06813403543394	0.705422699566442	4.34935541104594	1.36538272489111e-05	0.00118210635143457
**Dnajc25**	249.519468664584	1.42486673583908	0.328411597701025	4.33866144135454	1.43353160072332e-05	0.00123432554276488
**Gm12216**	3005.60601325562	-0.351113794739958	0.0810210258902501	-4.33361329212952	1.46681740703812e-05	0.00125612184145107
**Stk39**	472.928812328355	2.6427892611844	0.610024834769534	4.33226503341105	1.47583131294964e-05	0.00125700940530527
**Art2a**	321.848114344809	-0.830719538499261	0.191826807404012	-4.33057063160968	1.48723430493823e-05	0.00125991134101676
**Rac1**	12515.3282852452	2.92726629045709	0.676528544952884	4.32689250482543	1.51227717635245e-05	0.00127427547956072
**Ighg2c**	226.495483548689	2.45582047004565	0.567933382868634	4.32413473855207	1.53131688466439e-05	0.0012789652865662
**Zfx**	3582.64778294158	-0.399352361030555	0.092362762308533	-4.32373773855462	1.5340765320874e-05	0.0012789652865662
**Ptk2**	878.931763957259	0.522691389503172	0.120969060760903	4.32086837919887	1.55416361757923e-05	0.001288892427484
**H3f3aos**	39.6013237027059	-2.14873412541223	0.497454886729408	-4.31945525661514	1.56414816230246e-05	0.00129038128761256
**Atrip**	5188.06617277771	0.319182276616221	0.0740482442164624	4.3104638063148	1.62912492716061e-05	0.00133059298072082
**4930578M07Rik**	31.7891895497814	-4.21487983217743	0.977845298634418	-4.31037490088013	1.6297800677738e-05	0.00133059298072082
**mt-Nd6**	7569.13046022681	-0.831461765641244	0.193084099028349	-4.30621563259421	1.66071177816899e-05	0.00134885749941282
**Rnf130**	708.713238379914	1.56467883399008	0.363808911640508	4.30082602137079	1.70162620832923e-05	0.0013612043447794
**Scaper**	2661.34231679807	-0.491985941365516	0.114423916210809	-4.29967752946949	1.71046811110186e-05	0.0013612043447794
**Jaml**	5024.96142372171	0.341241097903777	0.0793617488347907	4.29981827409256	1.70938221026968e-05	0.0013612043447794
**Tmem39b**	1835.837977144	0.302879200843505	0.07042240505148	4.30089260118417	1.70111496782321e-05	0.0013612043447794
**Atr**	2028.56653722085	-0.454721998059103	0.105785565689316	-4.29852593873333	1.71937781896726e-05	0.00136141890921945
**Tlk2**	1649.53863201083	0.886606175990191	0.206513048790415	4.29322108788382	1.7609949386778e-05	0.0013873998624373
**C130036L24Rik**	922.122436262416	-0.530670569366203	0.123723421944048	-4.2891682191444	1.7934350355258e-05	0.00140592815197911
**Mrps27**	3635.56231912881	0.322742926434243	0.0753497236993567	4.28326622300538	1.84169589718538e-05	0.00143661397286881
**Vps37b**	16745.7854238206	-0.404168546557556	0.0944585644312469	-4.27879196546266	1.87910387297894e-05	0.00145142351600633
**Suds3**	1562.59245555717	2.68288431161504	0.62701576237862	4.27881478040898	1.87891130084176e-05	0.00145142351600633
**Ccdc171**	1045.60957228233	-0.515470705542297	0.120541052230152	-4.2763083282042	1.90018008581799e-05	0.00146054329815776
**Car7**	468.611755595644	-0.612714384769839	0.143345012851884	-4.27440322184733	1.91649926513278e-05	0.00146593586993676
**Trp53inp2**	173.07193623935	1.37153960278586	0.321306161932434	4.26863772091078	1.96670385591734e-05	0.00149707017669997
**Gm23849**	76.0326581288641	1.23098284938423	0.288644116907719	4.26470791288562	2.00163878967517e-05	0.00151633761581306
**Ctsh**	709.215516090613	0.832593019728532	0.195313387758822	4.2628568849394	2.01829790483558e-05	0.00152164210940164
**B230317F23Rik**	93.8534240577325	-1.64986145062058	0.387267761980066	-4.26026024522408	2.04189011131623e-05	0.00152483708455023
**Gm34220**	333.097247469252	-0.8302082603907	0.194861354038773	-4.26050750024813	2.03963237248451e-05	0.00152483708455023
**Gm38394**	3378.10430443978	-0.975146532468302	0.229053167402318	-4.25729337658761	2.06916748349294e-05	0.00153791849233011
**Cd28**	12423.831065058	-0.401597830115863	0.0943629694422822	-4.25588376976101	2.08224864586017e-05	0.00154037520717458
**Usp34**	5178.64518866262	-0.517002994660474	0.121521515612954	-4.25441529471314	2.09595982311787e-05	0.00154327284733029
**Gm13136**	126.428939193073	1.43366484600691	0.337157886887334	4.2522061673912	2.11674851253425e-05	0.00155133052613964
**Mepce**	1549.23569092034	-0.477065741969028	0.112232534454521	-4.25069026809106	2.13112707864857e-05	0.001554637471216
**Ago3**	1424.39642099176	-0.397272400028924	0.0940421917811515	-4.22440600867139	2.39571809817255e-05	0.00173960046418916
**Osbpl8**	4282.56799778901	-0.39610566381378	0.0939181124689311	-4.21756414605133	2.46955579572826e-05	0.00177683975677124
**Smad4**	1730.63303104271	-0.41822037760924	0.0991483796450066	-4.21812619738867	2.46340941566238e-05	0.00177683975677124
**Gm12107**	47.6547913104297	-1.28989624739346	0.305968367134157	-4.21578302186998	2.48913000803052e-05	0.00178278279711532
**Pi4k2a**	2458.45500337605	1.12263642921672	0.266729103656064	4.20890114287758	2.56615623454194e-05	0.00182963456052839

**Gm48720**	1407.08781299271	-0.958964528587249	0.227920827930908	-4.20744579287835	2.58273329832339e-05	0.0018331589451208
**Pik3c2a**	2444.09029314632	-0.47187070596262	0.112271340199539	-4.20294890150922	2.63460078622156e-05	0.00186158764970821
**Arl8a**	1949.17802685807	2.53680310262019	0.603772995060435	4.20158424337323	2.65053577397782e-05	0.00186448625850752
**Fndc7**	98.0712242736547	1.68327848858462	0.40109954902916	4.19666013751176	2.70879951942584e-05	0.00189700240122636
**Actn2**	2422.78195695606	-0.748829496516132	0.178580586356887	-4.1932301365593	2.75010191225326e-05	0.00191740512528206
**Ube2g2**	13750.8369466678	0.33749065737448	0.0807198515326349	4.18101186965183	2.90214712282782e-05	0.0019796161131774
**Slc12a4**	2941.65127844618	0.459346969535863	0.109785616834804	4.1840359673622	2.86378770773238e-05	0.0019796161131774
**Sympk**	1981.28808846915	-0.470241285460012	0.112447721173431	-4.18186585333061	2.8912654939835e-05	0.0019796161131774
**Klf3**	35465.8007843349	-0.342774908853761	0.081946952321351	-4.18288782125277	2.8782943534669e-05	0.0019796161131774
**Tmem243**	1186.51247331041	2.16690533169559	0.518081849467152	4.18255403837107	2.88252473611029e-05	0.0019796161131774
**Nbeal1**	1357.62171147927	-0.550645547278743	0.131845019344128	-4.17646074169479	2.96079813706924e-05	0.00201091794162931
**Fbxo7**	5386.18706534092	0.240241461244185	0.0576183241673727	4.16953225759082	3.05225414735125e-05	0.00205088806116173
**Kdm1a**	3051.46415866116	1.4314378381722	0.343313286444373	4.1694798736085	3.05295573410606e-05	0.00205088806116173
**Cables2**	1953.88819314574	2.44275390828985	0.586399312741981	4.16568344336495	3.10421194084925e-05	0.00205088806116173
**Gm15697**	40.8066914141152	-1.72588310658408	0.414070647444117	-4.16808850672519	3.07164671784571e-05	0.00205088806116173
**Gm37266**	300.18331019952	-1.01439351097631	0.243540002870582	-4.16520283739736	3.11075868894875e-05	0.00205088806116173
**Zc3h11a**	3024.3496636988	0.76796907642384	0.184316217425957	4.16658440124698	3.09197447804975e-05	0.00205088806116173
**Gm47794**	113.715787268619	-1.17462025665512	0.281996626002641	-4.16536989575229	3.10848155690267e-05	0.00205088806116173
**Man1a**	7301.30045191076	-0.314986264468103	0.075787499899496	-4.15617700657516	3.23617136688938e-05	0.0021246813428365
**Gnb1**	16456.3205630162	0.653246660931924	0.157214383695688	4.15513291834914	3.25098495331469e-05	0.00212555061864646
**Glipr2**	30403.0349990258	0.208583261308047	0.0502568501795778	4.15034488955709	3.31974674442143e-05	0.00215264401036414
**mt-Nd5**	41986.8812430924	-0.763094287195938	0.183854256738057	-4.150539132108	3.31693050612567e-05	0.00215264401036414
**Trim41**	201.352530660501	-0.824483599398754	0.198917756356033	-4.14484666679556	3.40041168642659e-05	0.00218233756203488
**Grsf1**	905.540845705148	1.99001864487135	0.480107521952859	4.14494369256466	3.39897221293127e-05	0.00218233756203488
**Tle5**	15510.8499765885	1.88667452115835	0.455234978768344	4.14439708974653	3.40708916837329e-05	0.00218233756203488
**Mier1**	4959.67369694931	-0.485864978134761	0.117281080655667	-4.14273960828554	3.43181518242283e-05	0.00218927578256828
**Ssc4d**	101.553939239157	0.950440136111001	0.229762353892984	4.13662255807879	3.52455143997513e-05	0.00223936923547129
**2310058D17Rik**	202.645876317701	-0.955624442204229	0.231259054741085	-4.13226821874775	3.59200911650155e-05	0.00227306376099257
**Nadk**	13362.405187425	0.211702962512598	0.0512748853719028	4.12878470575002	3.64685675172221e-05	0.00229854087347548
**Gm50394**	60.5201053028191	1.51249679373173	0.367454858288308	4.11614313871724	3.85265093797767e-05	0.00241857453504837
**Rapgef6**	56002.0967329958	-0.322140980009343	0.0784133072218118	-4.10824375890799	3.98679204043425e-05	0.00249285246750486
**Lrig1**	1002.3823415289	1.40317008076232	0.341705523963706	4.10637224849592	4.01921584045185e-05	0.00249333795267715
**Mras**	28.2372313918674	2.98310701611584	0.726452976305858	4.10640070784136	4.01872091569132e-05	0.00249333795267715
**Gm49747**	488.058401610696	-0.707480790055274	0.172483309798201	-4.10173477586323	4.10064148121022e-05	0.00253387481644821
**Mapkapk2**	2345.83100135588	1.89385648505259	0.462248104867467	4.09705624557528	4.18437260601271e-05	0.00257551402941181
**Lars2**	13623.5662182777	-0.785605305044241	0.191845887018174	-4.09498122297414	4.22202604356164e-05	0.00258857838009341
**Gfi1**	968.304135844507	2.57061524694693	0.627910107628189	4.09392238748464	4.24136336081523e-05	0.00259035513474285
**Gm2058**	77.2399867180167	2.46602828723461	0.602730563457302	4.09142730889456	4.28726334254317e-05	0.00259824648032511
**Gm17137**	83.8882594013084	-1.61776043993261	0.395355974561142	-4.09190841678408	4.27837627102022e-05	0.00259824648032511
**Fth1**	46028.0713790193	0.218033850126617	0.0533156629948902	4.08948961485322	4.32323423206397e-05	0.00261000773159509
**Gm7285**	280.763672221119	-0.68698302356919	0.168265814837411	-4.08272484956613	4.45107115988031e-05	0.00267692855977992
**Hipk1**	6962.13433561073	-0.481317665305909	0.117974741946808	-4.07983655961649	4.50673763449751e-05	0.00269246643112433
**Gm20559**	6102.91596885255	-0.327207975304834	0.0802056489180526	-4.07961259236425	4.5110816641291e-05	0.00269246643112433
**Pdgfb**	141.231109871585	1.36975783845574	0.335897150756594	4.07790847695616	4.54426468775698e-05	0.00270203693150894
**Gm7741**	71.2514095121887	-1.33374609391521	0.327492715365186	-4.07259774443517	4.6491679142402e-05	0.00275402025656703
**Eps8**	61.9861327441021	2.28106654302396	0.560235436147897	4.07162131461777	4.66870362903424e-05	0.00275523457238549
**Eml5**	2854.76396554478	-0.436842875375102	0.107316130357782	-4.07061710032506	4.68887641372494e-05	0.00275681438996507
**Ubl3**	3334.69453155259	0.978429453546834	0.240520617210601	4.06796500397352	4.74255017374967e-05	0.00277640317196825
**F2rl3**	745.585945638436	0.606828737935452	0.149199344224275	4.0672346188283	4.75743387974505e-05	0.00277640317196825
**BE692007**	8235.35109439086	-0.709215461502079	0.174615977861388	-4.06157254443841	4.87432663945111e-05	0.00283412416449562
**Fos**	4999.67984254529	1.78469187874305	0.439584943741052	4.0599476941921	4.90837102390479e-05	0.00284342655234073
**Tpr**	20646.8584730062	-0.20110948831734	0.0496108084956366	-4.0537434163168	5.04044999496501e-05	0.00290924434324776
**Pglyrp2**	11656.2441059407	0.265621082450858	0.0655761376406092	4.05057528558022	5.10918678680103e-05	0.00293815533575269
**4632404H12Rik**	311.551504697081	-0.763251936230521	0.188757656897711	-4.04355483520402	5.26468360421736e-05	0.00299478770944595
**Ccdc189**	113.114993183454	-1.11546535194097	0.275813163792866	-4.0442788756039	5.24844169981903e-05	0.00299478770944595
**mt-Nd1**	92286.9702333	-0.828028243635352	0.204694706166085	-4.04518641026069	5.22815064704658e-05	0.00299478770944595

**Fam117a**	2035.47007533542	-0.357518900285512	0.0884538011780632	-4.04187152529267	5.30262842559491e-05	0.00300552216194601
**E130215H24Rik**	582.175190908194	-0.486318347214466	0.120427775319356	-4.03825733660549	5.38497604511627e-05	0.00304125690117911
**Pfkl**	14365.8627924807	0.189532882719695	0.0470729339534217	4.0263664658599	5.66453838729059e-05	0.00316511104143751
**Rgs1**	1193.02215484049	0.777181851648685	0.193017237277685	4.02648935716861	5.66158007694761e-05	0.00316511104143751
**mt-Nd4l**	7172.98000032012	-0.85856435602437	0.213218430245225	-4.02668922680335	5.65677182535767e-05	0.00316511104143751
**Gm10222**	6973.49536890447	-0.825814776841438	0.205306258061031	-4.02235560007113	5.7618979114605e-05	0.00319683892221419
**Zfp950**	3483.72934659725	-0.271069108015411	0.0673844969353847	-4.0227221444621	5.75293505422343e-05	0.00319683892221419
**Dpagt1**	2086.2344474579	0.343673392889841	0.0856005279324305	4.01485132382738	5.94832790335743e-05	0.00328869483414748
**Gclc**	3708.18190168238	2.49655727343126	0.622008282382893	4.0137042289325	5.97732380437738e-05	0.00329317102047463
**Ptcd2**	5359.17107084555	0.228152211695018	0.0569088583406132	4.0090808065323	6.09555568639743e-05	0.0033466087439221
**Tox4**	15549.2827746491	0.212823915485941	0.0531271353902677	4.00593621174102	6.17723192575536e-05	0.00337967511993497
**Ube2v1**	17783.9652021777	0.210573000039622	0.052581656753683	4.0046855318014	6.21000361002484e-05	0.00338584868107825
**Ppp2ca**	16529.2209691538	3.12594584807583	0.780939543614672	4.00280133543631	6.25968632040902e-05	0.00340116818450638
**Gsr**	3431.99200242899	0.611121577245067	0.152984739755928	3.99465710253225	6.4787950115634e-05	0.00350208979288808
**Dusp22**	390.563488425384	1.12954088095775	0.282963220788983	3.99182931904812	6.55655574603023e-05	0.00350208979288808
**Gm8221**	442.733675647705	1.03887066293114	0.260201495989397	3.99256222175398	6.53631741075123e-05	0.00350208979288808
**Gm45555**	257.864789383572	-0.912088562417897	0.228353511559769	-3.99419547432344	6.49142934650896e-05	0.00350208979288808
**A730091E23Rik**	63.7266208740116	-1.27394295531313	0.319011239649589	-3.99341088016983	6.51295647413493e-05	0.00350208979288808
**Sec23b**	10853.2031376237	0.206938025937776	0.0518800504415708	3.98877842593536	6.6414419662332e-05	0.00353544598182218
**4732440D04Rik**	679.339741141426	-0.862813117713097	0.216482182226907	-3.98560800171875	6.7307553089043e-05	0.00357092630984529
**Mon2**	3246.18985749629	0.85666132915088	0.215041389198701	3.98370440380347	6.7849259761192e-05	0.00357558791323446
**Clec9a**	160.207640901206	1.06496385043845	0.267299501238682	3.9841595120954	6.77193756595757e-05	0.00357558791323446
**Rab18**	8978.46030159881	0.371674388670451	0.0934504297930437	3.97723573335687	6.97210389484433e-05	0.00366198136903541
**Usp7**	4186.53634435794	-0.38478841595163	0.0968498046218758	-3.97304276920262	7.09603017089525e-05	0.00371468928248493
**Dck**	4251.52610299293	-0.315843895244762	0.0795460033791957	-3.97058157327069	7.16974022894518e-05	0.00374084757574467
**Pkn1**	7790.99119299058	1.66771632337884	0.420350812767186	3.96743927387744	7.2649015086754e-05	0.00376556095632231
**D430013B06Rik**	20.7472742878036	-1.7728943973935	0.446788159399368	-3.96808724693344	7.24518107064945e-05	0.00376556095632231
**6530413G14Rik**	29.1680245283416	-1.87933814263596	0.473839621045498	-3.96619037152131	7.30305396729007e-05	0.00377292529057671
**Prkcb**	8822.83550479681	1.9326417468023	0.487639220124515	3.96326149957506	7.39327213737315e-05	0.00380705193034604
**Pms2**	3286.83039377812	-0.204543600874085	0.0517656010830671	-3.9513421383026	7.77141275319935e-05	0.00398873455218769
**Pex16**	3454.53108181399	0.290797739025909	0.0736099090419443	3.95052436296596	7.7980159160865e-05	0.00398939405161607
**Ano8**	325.048286798964	3.13571029934938	0.794286589146553	3.94783235949967	7.88619937057921e-05	0.00402145124537918
**Gm37469**	171.907912574713	-0.989908781846308	0.25110407946846	-3.94222500861698	8.07291857861744e-05	0.00410338638849274
**Tmed7**	1924.86122467156	2.19031866393644	0.555926753438132	3.9399410990574	8.15016172144058e-05	0.00412932791783727
**Trim12a**	9757.89556563529	-0.239183185076288	0.0607415636147592	-3.93771860390782	8.22599803935593e-05	0.00413220619923239
**Gm28439**	14364.5756499473	-0.791212151034079	0.200904093809532	-3.93825798186169	8.2075322526981e-05	0.00413220619923239
**1700019C18Rik**	103.864473513375	-1.59658572790636	0.40548516130205	-3.93747017222424	8.23451638356903e-05	0.00413220619923239
**mt-Nd4**	69732.8946419027	-0.765796542214284	0.194717636136884	-3.93285660922845	8.39423153832052e-05	0.00419898115394655
**Lncpint**	1988.71222696429	-0.526701095113451	0.134021954651299	-3.92996123987682	8.49595347097485e-05	0.00423641578614401
**Myl6b**	519.691780338783	-1.04793293948359	0.266806004800899	-3.92769623107096	8.57634385765676e-05	0.00426301104621759
**Ube3a**	8153.70715006525	-0.21596669909791	0.0550473070359943	-3.92329272268839	8.73469165983506e-05	0.0043144995763016
**Mad2l1**	1363.45727702951	0.438820068959512	0.111831895464674	3.92392588121809	8.71175480658443e-05	0.0043144995763016
**Vps13c**	1298.71031786732	-0.5924120744303	0.151057619317398	-3.92176228585691	8.79036924865565e-05	0.00432843275784585
**Jun**	14357.7192919599	2.20036774249344	0.561914535133065	3.9158405859218	9.00897640255969e-05	0.00442225673442782
**Taok1**	3841.72739736306	-0.419466633765462	0.107165794025441	-3.91418397614709	9.07104550872742e-05	0.00442515368671882
**Gm8517**	31.2986086391104	-2.39074847287108	0.610770815757461	-3.91431353822323	9.06617661234702e-05	0.00442515368671882
**Lck**	166063.260429485	0.178620385620113	0.0456515051368879	3.91269433690111	9.12720335414032e-05	0.0044388068904688
**Tasor2**	3940.44284625805	-0.535165870330088	0.13686709260925	-3.91011352785848	9.22527523144639e-05	0.00444573150250097
**Mcm3**	8880.21754060493	0.213467514983448	0.0545939832234779	3.91009232848296	9.22608492300448e-05	0.00444573150250097
**Tor3a**	3735.47828421312	0.281565201046397	0.0720032886852922	3.9104491779125	9.2124643120848e-05	0.00444573150250097
**Uck1**	8177.96309789451	0.300194987599256	0.0767957792190731	3.90900373239131	9.2677532290628e-05	0.00445219474482752
**Crlf3**	37363.5430108603	-0.236677407836109	0.0605830368462421	-3.9066613388297	9.35801672341257e-05	0.00448189269029823
**Sertad2**	2809.72752840994	-0.351075197364407	0.0899638136469324	-3.90240456837701	9.52417932520381e-05	0.00454765132203747
**Adgre5**	33640.9780793269	-0.279518158084381	0.0716418505652096	-3.90160438178462	9.55572399695537e-05	0.00454892879214579
**Dyrk2**	2834.68908285063	-0.342300391952532	0.087809949731981	-3.89819596751077	9.69119768875893e-05	0.00459952415607755
**Prkar2a**	1354.72386869185	1.08327137726663	0.278226022127344	3.89349410592089	9.88106043257221e-05	0.00467555162870992

**Mpo**	55.1935187967594	6.58276151447089	1.69209846327979	3.8902946000622	0.000100122594907205	0.00472344828728391
**Sidt1**	35423.5332105588	-0.282540636430214	0.0726859056005256	-3.88714475104745	0.000101430274059754	0.00474285464087227
**Usp25**	5710.94773472077	1.67032851755103	0.429555893667934	3.88850098944811	0.000100865259252873	0.00474285464087227
**Atxn7l3**	1283.26498730574	-0.488964966234063	0.125790781614628	-3.8871287701515	0.000101436949544581	0.00474285464087227
**Smu1**	14995.8081844051	0.214728246540458	0.0553061325729539	3.88253954038842	0.000103371200769988	0.00481899411400208
**Ddx60**	2862.23656642073	-0.503332825684571	0.129736492979328	-3.87965493845106	0.000104604752785193	0.00486211530866162
**Snora31**	107.302583973982	1.50166506944619	0.387280257125594	3.87746352109863	0.000105551147467002	0.00489167479599281
**Cd163l1**	1170.9315702963	0.673970231103306	0.174070474064058	3.87182395364354	0.000108023964056177	0.00495743114091786
**Trp53inp1**	9132.83940237835	-0.429435696397242	0.110926229069376	-3.87136297699854	0.000108228489717316	0.00495743114091786
**Ints6**	4541.62049052642	-0.307669333502182	0.0794529646066877	-3.87234554462786	0.000107792984857257	0.00495743114091786
**Gm10382**	232.198724314731	1.59727617816879	0.41256453743234	3.87157894885898	0.00010813262208897	0.00495743114091786
**Pim2**	32154.1938807053	0.527337722944641	0.136533035661929	3.86234525869906	0.000112303699226811	0.00512918663396193
**Exo1**	159.663473766765	0.738007676744987	0.191152248683672	3.86083701252335	0.000112999264583248	0.0051460387631163
**Tmem19**	2384.68688905142	-0.290087578201759	0.0751530738140843	-3.85995626631833	0.000113407319912967	0.00514973815524097
**Prdm11**	369.636356356125	1.69011634928937	0.43807249160561	3.85807459193524	0.000114283773038196	0.005174624746445
**Gm43654**	94.1308303451869	-1.27117486220915	0.329613014792205	-3.85656756609119	0.000114990324213327	0.00519169781842231
**Asb2**	460.94377610626	0.641322650606601	0.166335291175153	3.85560181531939	0.000115445268832339	0.00519734600283191
**Tbc1d8**	420.383039051112	0.702656981335831	0.182389650865464	3.85250466789988	0.000116915747202029	0.00524855107881017
**Psap**	83742.0021898406	0.171007000636389	0.0445033454207444	3.84256507055034	0.000121755119269467	0.00536290139240495
**Rassf1**	7192.49572359733	0.22174313335275	0.0577200749524703	3.84169863839131	0.000122185796780693	0.00536290139240495
**Cab39**	9842.58562683836	1.60193170362828	0.416883395310651	3.84263734571285	0.000121719258194541	0.00536290139240495
**Rpf2**	3271.51632054103	0.343014383867893	0.0892754217868334	3.84220401318206	0.000121934415862318	0.00536290139240495
**Emp3**	15189.7649514254	0.24778377103324	0.0644216934207854	3.84627844870178	0.000119925469573271	0.00536290139240495
**Znrf2**	4813.46586194834	-0.405334375200364	0.105447729404062	-3.84393649338028	0.000121076350339139	0.00536290139240495
**Mir6973a**	226.604798354582	-0.643735744089384	0.167487255238415	-3.84349091620756	0.000121296491058909	0.00536290139240495
**Gm47963**	157.249523945094	-1.22342884343169	0.318412504119387	-3.84227637923721	0.000121898459846644	0.00536290139240495
**Abhd10**	2034.94778864595	0.428830570491969	0.111698994305961	3.83916232331811	0.00012345479367515	0.00540354773316484
**Atrx**	15014.7627203405	-0.256660941930228	0.0669154611689588	-3.83559998611037	0.00012525813451907	0.00546729203772018
**Rexo4**	7984.28365481229	0.308311716114132	0.0804127065062154	3.8341168891051	0.000126016210269883	0.00548518625752086
**Atf7ip**	14223.6238059325	-0.446919204939595	0.116627671146897	-3.83201688368348	0.00012709701457507	0.00551699079520491
**Snrnp40**	10086.5842708475	0.247912894278505	0.0647370545681386	3.82953620507349	0.000128384994603888	0.00555758890102599
**Nlk**	2433.08776661314	-0.454174985378913	0.118727414985051	-3.82535899931871	0.000130581656349253	0.00563719221669911
**Akap9**	8683.64931881672	-0.29593850415408	0.077409278687954	-3.82303658127397	0.00013181821411266	0.00565956294216126
**Sacs**	1574.91946733912	-0.548737704222209	0.143531827983391	-3.82310817002697	0.000131779932843712	0.00565956294216126
**Nr2f6**	225.469523437522	1.66300400886507	0.435073463362996	3.82235219774269	0.000132184709696956	0.00565987627906234
**Sox4**	186.385136046663	1.68609149360369	0.441344622595091	3.82035127943677	0.000133261740360118	0.0056905291134265
**Klf7**	5202.94685199631	-0.350173839903734	0.0917157315166703	-3.8180346393474	0.000134519041451364	0.00569789391437941
**Atp2a2**	2910.7406673119	1.66464402058015	0.435950571112384	3.81842376380561	0.0001343070753992	0.00569789391437941
**Ppp6r1**	12174.3189769173	1.52767289241852	0.400009587718069	3.81909069013425	0.000133944515250929	0.00569789391437941
**Rbl1**	4940.19330397159	-0.319733635538458	0.0837839543252393	-3.8161678821853	0.000135540301969949	0.00571045063673929
**9430034N14Rik**	109.861040356283	-1.24493998961112	0.326191585880356	-3.81659136378751	0.000135307986232145	0.00571045063673929
**Cct7**	39622.1612630874	0.167139601675092	0.0438570906015927	3.81100523045234	0.000138402838426671	0.00581550273357081
**Setd5**	3395.31263509544	-0.451584485629774	0.118519765575924	-3.81020400635613	0.000138852165552406	0.00581886588459911
**Csnk1g3**	1951.91585130896	1.63911928444413	0.431048098081161	3.80263662394239	0.000143164267718641	0.00598365879693003
**Herc4**	6887.60285242509	-0.245570644095408	0.0646299036685789	-3.79964428470589	0.000144903928258179	0.00603332677502584
**Gm2682**	23127.6384941806	-0.356352422425461	0.0937947816929172	-3.79927769960758	0.000145118413894447	0.00603332677502584
**Bcl2l1**	3305.65587971478	0.432169083959359	0.11391561902425	3.79376496095202	0.000148380136097652	0.00615269948550183
**Kmo**	480.22932475909	0.857767156351615	0.226210840365948	3.79189235566244	0.000149503718248802	0.00616683269226801
**Lrrc8d**	901.033599047368	1.77621424642283	0.468349345342164	3.79249862114181	0.000149139078894384	0.00616683269226801
**Ubash3b**	3610.83107843003	-0.270575800753018	0.0714246234348873	-3.78827059549964	0.000151699575627282	0.00624107105263466
**Map3k3**	9377.84462689387	2.09033630339719	0.552079490350983	3.78629588660913	0.000152909587184108	0.00627446970119787
**Gm37520**	374.727771981415	-0.886938112080662	0.234917690225188	-3.7755271270991	0.000159669645793491	0.00653484313965725
**Pgpep1l**	77.0295843024169	-1.30251100974884	0.345225653511009	-3.77292648012121	0.00016134384326334	0.00658045481902659
**Mocs3**	299.467894812148	3.19019874776039	0.845645576233165	3.7725009595282	0.000161619344733343	0.00658045481902659
**Ssbp3**	2242.03324267349	0.970071458653547	0.25723906755464	3.77108915793864	0.000162536583607056	0.00660074471107316
**Rbbp6**	5217.16703780612	-0.359340337188208	0.0953159749654073	-3.76999067909259	0.00016325364437266	0.00661282178503856

**Foxo1**	15266.1436167321	1.61414769421366	0.428911138742937	3.76336156469249	0.00016764454688997	0.00677326955216732
**Ptms**	144.400455940213	1.21990039057517	0.324557785720383	3.75865391078971	0.000170829941370895	0.00686675353617651
**Gm44321**	248.978087016845	-0.975467961509035	0.259525485542645	-3.75865961475582	0.000170826047610639	0.00686675353617651
**Tgoln1**	16656.2522539617	0.24901150408151	0.0662661957034018	3.75774558111122	0.000171451070567909	0.00687418452656118
**Smim19**	396.57180351463	1.40384437045686	0.373996799564071	3.75362669438127	0.000174294375042595	0.006970447887173
**Pik3ca**	1474.04172299638	-0.423194872866176	0.112920048926037	-3.74773901438326	0.000178435775044416	0.00711800634778447
**Sh3bp4**	122.327375394145	1.33582186313047	0.356684350167525	3.74510926118028	0.000180315284425183	0.0071567454324625
**Gm48562**	146.359252722222	-1.16546372433301	0.311143836005592	-3.74573939594953	0.000179863232904416	0.0071567454324625
**Agfg1**	6587.94831447152	1.88638683292439	0.504591871689586	3.73844078504074	0.000185165092937354	0.00733076977239671
**Ncor1**	17478.4349875372	0.686642764896619	0.183770839242701	3.73640762444247	0.000186667979815851	0.00735331839489592
**Ubtf**	12968.0561237264	0.276876310710892	0.0740997443133841	3.73653530489824	0.000186573263607972	0.00735331839489592
**Pcdhgb5**	24.9645227710339	-2.65928685572373	0.712181243294705	-3.73400293922554	0.000188460292532249	0.00740540855219613
**Naaa**	1926.3178178398	0.691334174304907	0.185381617839919	3.72924879154894	0.000192051461369409	0.00749048236831134
**St7**	957.045632206696	0.797192980037183	0.213710894972165	3.7302402394647	0.000191297279311114	0.00749048236831134
**Gm42878**	29.6514179830683	-5.47852763654064	1.46903706501098	-3.72933247705339	0.000191987695034158	0.00749048236831134
**Zdhhc14**	167.025181296353	2.08773478278958	0.56010002883208	3.72743202163893	0.000193440712374121	0.00750750075093354
**Gm44175**	1070.35449472857	-0.443012728162001	0.118841049699697	-3.72777528708693	0.000193177502020437	0.00750750075093354
**Gcsh**	1291.54763564758	0.378977595865993	0.101783067162269	3.72338549458134	0.000196569033571778	0.00759846232389649
**Mbd5**	770.232622115868	-0.488197420492943	0.13112466842839	-3.72315466147056	0.00019674891338134	0.00759846232389649
**Galk1**	3613.6832998002	0.308527800260076	0.0829723488970806	3.71844119590703	0.000200455941943677	0.00772269994427023
**N4bp2l2**	4024.38635456918	-0.225464098807084	0.0606749140948774	-3.71593602018992	0.00020245281120107	0.00776167626787169
**Ctdp1**	5760.24895957389	2.49734662684449	0.672059061465341	3.71596303068861	0.00020243118189582	0.00776167626787169
**Nup93**	7069.64529584492	0.274169589159628	0.0738888292792242	3.71056886181736	0.000206794033879584	0.00790886794136071
**Mapre2**	4471.05827858337	0.407173726399218	0.109785469876595	3.70881253099251	0.000208233525355378	0.00794463839957554
**Abhd17b**	734.28631948536	1.91036379930313	0.515221478168696	3.70784969231742	0.000209026657999633	0.0079556353867155
**Tlr1**	6139.90092947297	-0.289377966081054	0.0781200730664295	-3.70427157479719	0.000211999043531477	0.00802997338684011
**Klf13**	51403.1964783067	0.469147017498098	0.126632827285638	3.70478198705832	0.000211572624082659	0.00802997338684011
**Arhgap22**	146.349914063615	0.938606951897464	0.253475333936382	3.70295183093847	0.000213105356346589	0.00805252062338898
**Mesd**	6714.72251235652	0.224047485716369	0.0605520641886051	3.70008006694067	0.000215531455316054	0.00812471086462933
**Lrif1**	2416.31819143267	-0.261742203054269	0.0707642050209378	-3.69879380368682	0.000216626493565915	0.00814650037975685
**Smap1**	1664.50945171914	0.460491456497408	0.12455319048231	3.69714701577885	0.000218036084368132	0.00817998709854443
**Ankrd24**	284.252219591973	-0.879088640004181	0.237847745784763	-3.69601417538637	0.000219010749476333	0.00819703653087548
**Sgf29**	6941.90152393966	0.227707176505138	0.0616404975342422	3.69411645937224	0.000220652664142872	0.00820005667193217
**Rnf149**	1271.87117742739	1.5596512920863	0.422144136175019	3.69459423555674	0.000220238204453126	0.00820005667193217
**AU041133**	1114.35486739808	-0.382409779957907	0.103507047916276	-3.69452890074915	0.000220294837703417	0.00820005667193217
**Zmiz2**	2075.36640008694	-0.408582952955719	0.110637746873305	-3.69297969727818	0.000221641724279631	0.00821743211640977
**St3gal4**	2212.63508291208	0.538102582509678	0.145746555420966	3.69204322500421	0.000222459643728197	0.00822839578926104
**4930563E22Rik**	214.011881135898	-0.882868180922067	0.239263211514595	-3.68994537577797	0.000224302210197991	0.00827711926484717
**Zbtb24**	3832.82244664263	0.231051244693792	0.0626458211793731	3.68821479779513	0.000225832971207069	0.00831413581147146
**Crebrf**	7732.42859869949	-0.401373401850539	0.109045992303647	-3.68077169432218	0.00023252914046009	0.0085221752731194
**A830005F24Rik**	81.5503137238507	-1.26645595609302	0.344077219707188	-3.68073177634596	0.000232565549751941	0.0085221752731194
**Bambi-ps1**	1407.61381775518	-0.817958952552451	0.222284172600962	-3.6797894469114	0.000233426606349868	0.00853388175465166
**Pgm2l1**	1053.06758822516	-0.430071757226435	0.116934707411641	-3.67787944867787	0.000235181058602767	0.00856651448670755
**Gan**	822.347725120027	-0.467037475448471	0.126993973440554	-3.67763495223725	0.000235406535047558	0.00856651448670755
**Fam91a1**	1765.9025576742	1.7124575012138	0.465758425931416	3.67670750730758	0.000236263677105092	0.00857789576070262
**Rnf103**	1030.80312884159	2.12042318874542	0.576975049637954	3.67506912140476	0.000237785029126932	0.00861328437690361
**Map7d1**	4829.45133635035	2.40013181221646	0.653522009828062	3.67261052592233	0.000240085256080905	0.00863703968051784
**Arcn1**	20958.6332265225	0.17088019785896	0.0465280946475736	3.67262401680726	0.000240072577432093	0.00863703968051784
**Ctbp1**	18390.7809189258	2.44028637274311	0.664303105278843	3.67345320735599	0.000239294514085121	0.00863703968051784
**Mark3**	3543.91032111147	1.76468343797109	0.481068515391936	3.66825801628978	0.000244208674396569	0.00876536693044816
**Gm42748**	72.7565191832901	-1.11035534852369	0.302784692252445	-3.66714492817866	0.000245273792575387	0.00878358897638721
**Mrpl10**	5257.13509963778	0.233957102532239	0.063887583107935	3.66201210236706	0.000250242034653922	0.00883998133695039
**Plpp1**	80.9528869342098	1.20560235715701	0.329242133650755	3.66174992182456	0.000250498325021091	0.00883998133695039
**Wdr53**	845.302401500265	0.389033014962127	0.10619286026334	3.66345735482963	0.000248833660183152	0.00883998133695039
**Cpt2**	2082.2721223896	0.353550777029671	0.0964855105615482	3.66428881364669	0.000248026789780041	0.00883998133695039
**Stx2**	7703.76056415393	0.263178968077256	0.0718780211157434	3.66146652331268	0.00025077563353537	0.00883998133695039

**Fbrs**	1218.22467532521	-0.470180960591779	0.128410397065169	-3.66154899710466	0.000250694902348042	0.00883998133695039
**Armc7**	16431.1019885241	0.267471823630668	0.0730049101840804	3.66375114983695	0.00024854827260427	0.00883998133695039
**Cnot8**	11467.4549663169	0.218447576546094	0.0597119068725979	3.65835874262359	0.000253835580367226	0.00888819386632528
**Zbtb25**	3276.59046662926	0.551409023006849	0.150714753120472	3.65862672094274	0.000253570353514533	0.00888819386632528
**2900076A07Rik**	580.289635194981	-0.423700363153274	0.115807800647285	-3.65865132387528	0.00025354601622256	0.00888819386632528
**Jpt2**	3457.64076476129	0.214020191846711	0.0585267584861144	3.65679216451886	0.000255391286865743	0.00892283926195902
**Gm13034**	70.5375835822428	1.66640616888297	0.456105439200606	3.65355469516784	0.000258634663257033	0.00901616457730326
**Golgb1**	3238.38352017302	-0.344596262218146	0.0944002200676206	-3.65037562382064	0.000261857086006494	0.00910834901590359
**Tmem158**	23.5983433992735	5.34711739531764	1.46545119906491	3.6487857110012	0.00026348276388388	0.00914470905400505
**Gm24270**	60.421127340323	-1.28133994813694	0.35145600185624	-3.64580471344763	0.000266556350913432	0.00923105147547901
**Csnk1d**	22037.9295355995	0.224611213743834	0.061624125906876	3.64485841281152	0.000267539050631025	0.00924476495787951
**Shprh**	4411.86959771909	-0.333271955747272	0.0915079287396499	-3.64200086634534	0.000270527162514822	0.00932756345677473
**Eif4e2**	8149.29841510642	0.316646219416254	0.0869796503634595	3.64046323586142	0.000272147966468202	0.00934256101882234
**Kif22**	1122.95325124741	0.517686361961762	0.142188121473101	3.6408552036444	0.000271733934214721	0.00934256101882234
**Zfpm1**	297.755133245396	1.71563414439079	0.471521671562795	3.63850539192515	0.000274224887828221	0.00937301856292684
**Aqp10-ps**	82.1183321949954	-1.04239023254391	0.286481460230455	-3.63859578105115	0.00027412867499812	0.00937301856292684
**AA474408**	522.802338392053	-1.27060412150985	0.349443005633084	-3.63608399947198	0.000276814099177411	0.00943287421297184
**1810034E14Rik**	2051.10424562726	-0.324502417201891	0.0892532337047917	-3.63574969479755	0.000277173368065365	0.00943287421297184
**Sirt1**	382.34259417096	-0.459670057708806	0.126454897639018	-3.63505143961285	0.000277925175456683	0.00943807540877361
**Heca**	1479.21354837866	-0.358382840684017	0.0986160267124925	-3.63412370819659	0.000278927015084532	0.00945172683158487
**Rac3**	22.951351792418	5.38122728749179	1.48221731814392	3.63052517442607	0.00028284511397273	0.00954344852434328
**Tent5c**	4704.44857856533	-0.61430411664416	0.169199808489499	-3.63064309663381	0.000282715906619736	0.00954344852434328
**Neurl4**	963.650140107228	-0.46354545210925	0.127714394850312	-3.62954741830432	0.000283918574589185	0.00954690618754016
**Hmgb1-ps8**	720.927212415335	-0.711361014490598	0.196003475367977	-3.62932857774633	0.00028415935786992	0.00954690618754016
**Snrpn**	273.64439644524	0.881430947086803	0.242928660287539	3.6283530565867	0.000285235021866971	0.00956265582884653
**Cdc25a**	2258.81565955096	0.31242110141473	0.0861304347303164	3.62730203780991	0.000286398202394644	0.00958126640155501
**Cstf1**	5184.47529981458	0.215380864258912	0.0594245101662138	3.62444492443402	0.000289582719344159	0.00966727734895319
**2700038G22Rik**	285.460592258452	-0.700995407624832	0.193527877990382	-3.62219342713855	0.000292115558888255	0.00973121535180175
**Gm26581**	619.482493119436	-0.570073953713044	0.157499028673353	-3.61953948868699	0.000295127772634506	0.0098108192265863
**Satb1**	39394.631232736	0.855066206326173	0.236506034498808	3.61540967924216	0.000299873015427867	0.00987318118931709
**Rock1**	14605.7779469446	-0.266631315821295	0.0737532206158977	-3.61518200282933	0.000300136687801161	0.00987318118931709
**Gnas**	17875.5982305969	1.55309018209076	0.429450171635125	3.61646189632988	0.000298657256975272	0.00987318118931709
**Lef1**	50399.9558666345	-0.304637904517297	0.0842434317530487	-3.61616209332869	0.000299003185904068	0.00987318118931709
**Gm29776**	20.6530776016345	-2.91437249189313	0.806071952484467	-3.6155240024299	0.000299740699413204	0.00987318118931709
**Gas8**	305.290202373018	0.605957133382236	0.167698433680727	3.61337384066382	0.000302238445666056	0.00990097960158012
**Zfp141**	2684.42145308383	-0.284940043165403	0.0788478070434061	-3.61379794632135	0.000301744243276688	0.00990097960158012
**Imp4**	11278.0972663709	0.174032655940524	0.0481954109865305	3.61097980862041	0.00030504241235463	0.00997210226446454
**Rnf138**	6678.25112088191	-0.221645677422118	0.0614953589869682	-3.60426674587082	0.000313035434457974	0.0102023906996996
**Dbn1**	87.1799270856636	1.53663616904204	0.426372063471952	3.60397948338641	0.000313381804826718	0.0102023906996996
**Cd36**	221.40103692248	0.797179867728229	0.221269945069979	3.60274807080606	0.000314870665797124	0.0102203129747885
**Gm45137**	113.341521163073	-1.27647447312155	0.354334895739488	-3.60245205445425	0.000315229555483099	0.0102203129747885
**Lamp1**	9513.92245391405	2.50559381047028	0.696536965435886	3.59721584754989	0.000321641564966481	0.0104001277042494
**Gins3**	1070.49157331705	0.366313244288598	0.1018428286332	3.59684868541823	0.000322095723784586	0.0104001277042494
**Zbtb11os1**	629.330236344487	-0.541117747562741	0.150471091224618	-3.59615752872409	0.000322952274412862	0.010406460098003
**Dnah8**	2275.65487991531	-0.58371120885647	0.162366575904433	-3.59502074614197	0.000324365727801609	0.0104306750468775
**Gem**	382.160757791156	1.25544498020847	0.349325162541599	3.59391511070712	0.000325746005780831	0.0104537267069013
**Ash1l**	9288.04745864941	-0.451988722101842	0.125815552017736	-3.59247100102637	0.000327557115544979	0.0104904826618745
**Itga6**	3653.75813325869	0.906394058572659	0.252396827295718	3.59114679960178	0.000329226122458521	0.0105225476908295
**Heatr3**	3720.59307028168	0.260574830415495	0.0725850304540382	3.58992520614143	0.000330772857668467	0.0105292685217819
**Gm43379**	91.8088101096891	-1.23503936035651	0.343998407666789	-3.59024731751905	0.000330364353682014	0.0105292685217819
**Puf60**	31954.4141016485	0.180676310425592	0.0503558293099894	3.58799195448362	0.000333234570333272	0.010578249141394
**Apol7e**	16059.8620860338	-0.270962111848661	0.075526061972921	-3.5876637119755	0.000333654237689459	0.010578249141394
**Kcna2**	909.587416218604	-0.751201327966366	0.209541965956099	-3.58496840734877	0.000337119011350111	0.0106666350639432
**Slc35c2**	4018.26566922625	0.181489290516811	0.0507056671374987	3.57927034121584	0.000344554893111808	0.0107912576926098
**Pkib**	200.499665770599	0.80758023033239	0.225628081577533	3.57925407460809	0.00034457633870872	0.0107912576926098
**Endod1**	904.350593105321	0.572119443558885	0.159863193436654	3.57880654864804	0.000345166838679657	0.0107912576926098

**BC028528**	196.204702958981	1.21041880847957	0.338195020184762	3.57905568159549	0.000344837996916186	0.0107912576926098
**Rab7b**	348.101300547775	1.05328081738125	0.294243332305677	3.57962509847816	0.000344087498082989	0.0107912576926098
**Mthfs**	1453.76063064654	0.314679166800549	0.0879201340637705	3.57914794092904	0.000344716294056532	0.0107912576926098
**Nefh**	1162.09823891981	0.751844262150703	0.210240537129804	3.57611463714302	0.000348738771586171	0.0108775567898705
**Tent4b**	1488.76653520763	0.880978012910903	0.246379990666022	3.57568814955068	0.000349307846396807	0.0108775567898705
**Sertad3**	2456.22376808967	-0.414814090365253	0.116105603373841	-3.57273101651795	0.000353277589463465	0.0109794772725361
**Myo9a**	1685.62755597665	-0.649467647295233	0.181885986657932	-3.57074043596701	0.000355973521944854	0.0110414857978052
**Sipa1l2**	813.219071392395	1.32211890492944	0.370426850933932	3.5691767527005	0.000358104768502445	0.0110857698178645
**Iigp1**	2098.69553024062	-0.789724718295992	0.221393071103349	-3.56707061499381	0.000360994225465371	0.0111251182985428
**Mex3b**	129.180391461058	-1.01525120543853	0.284585264546351	-3.5674763662023	0.00036043587652216	0.0111251182985428
**Heg1**	873.029835140654	0.610199805610737	0.17109795194423	3.56637702951367	0.000361950532703637	0.0111251182985428
**Gm10652**	29.6042921053786	3.79341258262355	1.06371388266709	3.56619636580486	0.000362200018223801	0.0111251182985428
**4930431P19Rik**	807.451994480437	-0.792590529236485	0.222315637709917	-3.56515869689148	0.000363636092352653	0.0111474978739314
**Setd7**	1047.35511058055	-0.420134807944464	0.117893586898929	-3.5636782202975	0.000365694205428063	0.0111888225144272
**Unc5a**	545.534781285092	1.28395023558547	0.36038710780256	3.56269746555109	0.000367063612345032	0.01120895608473
**Mxd1**	2284.27751281127	0.539827032182547	0.151731406865158	3.55778044463982	0.000374001711363365	0.0113987330095794
**Gm16023**	245.478964108145	-0.643792238431685	0.181059889466367	-3.55568668648323	0.000376993136291432	0.0114456278391986
**Gm44567**	109.250591319206	-0.98670662705937	0.277482390886568	-3.55592520270133	0.000376651234579219	0.0114456278391986
**Zfp738**	1073.68875896286	-0.357726233429388	0.100648864620359	-3.55420038545601	0.000379130223377083	0.011488374864909
**Pld4**	1616.42685755852	0.649010130708972	0.182731077576803	3.5517227792639	0.000382717853118752	0.011552653662054
**Zfp746**	2671.00662238491	1.74737511761419	0.491940880487921	3.55200225661485	0.000382311581663125	0.011552653662054
**Gm15760**	98.5163328294957	-1.25118300017016	0.352328138396314	-3.55118670301255	0.000383498267539438	0.0115540768673402
**Ankrd13d**	963.516506021744	0.375422972689965	0.105774322684864	3.54928269130564	0.000386282147326639	0.0116157400676066
**Akirin2**	809.451380639256	1.81604203386266	0.512195112637646	3.54560594010865	0.000391711497211804	0.0117565677363169
**Gm42611**	233.233667802827	-0.896252017278786	0.252912631077691	-3.54372185153327	0.00039452122823998	0.0118183859189684
**Phf21b**	296.275646008715	1.600988228346	0.452022387253041	3.54183393011854	0.000397355555166375	0.0118357873019973
**Nfkb1**	3926.62742821037	-0.275359164438039	0.077737649493662	-3.54215963862516	0.000396865216554533	0.0118357873019973
**Usp53**	1969.91580004455	-0.4557892846481	0.128666543688351	-3.54240715249247	0.000396492974346769	0.0118357873019973
**Fxr2**	1066.05932400041	0.985555821223279	0.278469568226024	3.53918680415138	0.000401361722371866	0.0119325597347424
**Srsf9**	1684.57357980588	2.16812211032312	0.613254725038907	3.53543482308361	0.000407104659465547	0.0120805049325774
**F2rl2**	476.384569790044	0.980404072055932	0.277789234933163	3.52930909036781	0.000416646194979489	0.0123172497078645
**Hmgcl**	4921.07219198044	0.296522537815476	0.0840078131959229	3.52970189955935	0.000416028136267128	0.0123172497078645
**Clic4**	5680.44012709141	0.476550744437445	0.135147744858202	3.52614647723088	0.000421653689356676	0.0124419422906239
**4732416N19Rik**	228.258236412579	-0.538746002503219	0.152829139895291	-3.52515235558044	0.000423239295562164	0.0124653861311645
**Zfp281**	5262.01052632492	-0.402756390706547	0.114306754810721	-3.52346973171854	0.000425935743976539	0.012521398354176
**Gm39469**	273.005869188783	-0.524562467156926	0.148915529431212	-3.52255046307467	0.000427415663007651	0.0125415057765578
**2810403D21Rik**	141.444238799503	-0.786282366097744	0.223248384561957	-3.52200696833948	0.000428292885283775	0.0125438866048633
**Galnt1**	2972.11383492549	1.41215379786812	0.401471033010034	3.51744878648026	0.000435716420952099	0.01271404378693
**Ehd4**	1289.96422239182	0.359979796413955	0.102340851718793	3.51745945405154	0.000435698908177345	0.01271404378693
**Map3k7**	2064.72187248356	1.75858272585983	0.500041037975403	3.51687680071238	0.000436656403334301	0.0127179204202192
**Dok2**	13318.0355322127	0.308547331421534	0.0877636779444845	3.51566090492137	0.000438660858699215	0.0127527290600065
**Gm13135**	131.844451961022	0.907238625863278	0.258140034197192	3.514521212042	0.000440547486094793	0.0127839903101209
**Idnk**	8137.14171269898	-0.212991214155814	0.0606524234522994	-3.51166865283335	0.000445302817210628	0.01289822884336
**Tipin**	1942.90028841206	0.296864601929846	0.0845592277198885	3.51072981547611	0.000446878346524583	0.012920113956308
**Uba6**	1752.30722051454	-0.38672588372304	0.110218377018009	-3.50872417273801	0.000450261602708565	0.0129940880473972
**Ensa**	13526.9374115899	0.209053792803885	0.0595954473590177	3.50788192837102	0.000451689473001546	0.0130114643986935
**Tmem65**	641.788663645976	2.02444466076201	0.577448135029857	3.50584673835225	0.000455157224288386	0.013087431356044
**Rpap3**	1282.91216004632	0.520252212983534	0.148418854339805	3.50529732423629	0.000456097618255831	0.0130905831891751
**Cct8**	32792.230290808	0.158790161218414	0.0453451001244345	3.5018152078762	0.000462100001671202	0.0132046972958579
**Tbx6**	181.406553041861	-1.02844353644451	0.293670904270416	-3.50202734247553	0.000461732231350643	0.0132046972958579
**Gimap3**	126453.221084389	-0.189900107502678	0.054233400837208	-3.50153419426342	0.00046258760597281	0.0132046972958579
**Esco1**	6740.82602380925	-0.291566966138334	0.083285704853955	-3.50080444957041	0.000463856074544963	0.0132169623265913
**Asb8**	3493.61961571515	0.368243107870378	0.105234185095426	3.49927267015426	0.000466529231469409	0.0132691355600424
**Myc**	14606.2845204559	0.37336411402745	0.106729457213441	3.49822929653605	0.000468358277509457	0.013297155637327
**Zfp942**	2869.52198365884	-0.197653610302059	0.0565132247701208	-3.49747534503748	0.000469684124541795	0.0133108142992897
**Tob2**	4149.84381920078	-0.317243534351172	0.0907320299584895	-3.49648888596797	0.000471424131267467	0.0133257995552651

**Zhx2**	1192.86728116507	-0.423100154843834	0.121016551166992	-3.49621725924078	0.00047190430613936	0.0133257995552651
**Rflnb**	10077.1187782436	-0.344300856804023	0.0986258950010558	-3.49097827502947	0.000481255384677424	0.0135655475784654
**Cldn10**	299.385100309562	-0.736729578401365	0.211129052285266	-3.48947513583273	0.000483970079303408	0.0136177081063996
**Gm43560**	28.4930899359967	-1.57781737190265	0.452296416970707	-3.48845870252569	0.000485813861617593	0.0136452210650774
**9130002K18Rik**	208.32753544621	-0.808833022494009	0.232028329552277	-3.48592356827607	0.000490441110518548	0.0137506771858377
**Bptf**	6468.76444469221	-0.40456101614996	0.116129284744357	-3.48371228704756	0.000494510773910208	0.0138401532229185
**Clptm1l**	6749.78054750911	2.26291556812856	0.649775260448493	3.48261269068114	0.000496546183758202	0.0138724791090035
**Gm37176**	309.359166862807	-0.585797977471036	0.168239815531655	-3.48192237146632	0.000497827988975114	0.0138836736677537
**Rela**	15809.4440665523	0.36800783147781	0.105778568556197	3.47903962495295	0.000503214173615328	0.014007609404536
**Cdca8**	421.088422892088	0.68215204095945	0.196114130843353	3.47834211653174	0.000504525551457444	0.014007609404536
**Gm12183**	3519.28608009469	0.450267993918685	0.129457193956771	3.47812261456124	0.000504938893303067	0.014007609404536
**Phf2**	459.658025498878	-0.716872850041411	0.206174116386472	-3.4770264211907	0.000507007852803306	0.0140142285237938
**1110002L01Rik**	988.437283786073	-0.470124508979388	0.1351914761659	-3.47747152640359	0.000506166808369826	0.0140142285237938
**Gm29666**	111.606748128018	-0.821148922676635	0.236194204942346	-3.47658369889758	0.000507845686811337	0.0140142285237938
**H3f3b**	53556.0557018544	0.333742796868508	0.0961356696306759	3.47158134073073	0.000517402556404477	0.0142033311520302
**Ciita**	841.148385552322	0.781682603783759	0.225161703060309	3.47164989942533	0.000517270451458035	0.0142033311520302
**Gzmm**	1998.95329820819	0.391300239221987	0.112708413660328	3.47179262411817	0.00051699553775926	0.0142033311520302
**Nab1**	3034.68904215639	0.787355708086935	0.226861215978244	3.47064924558299	0.000519201722464298	0.0142268134912516
**Tollip**	8889.94678753419	0.297316808871793	0.0856885519639396	3.46973781278172	0.000520966642409862	0.0142268134912516
**Tgfbi**	178.993614943992	1.03019770558896	0.296908386411586	3.46974943362111	0.00052094410439335	0.0142268134912516
**Gemin4**	3879.67397078981	0.311400804886146	0.0898881348369771	3.46431490041382	0.00053158383425508	0.0144916375023483
**Klrd1**	6182.38291017822	-0.309772647679023	0.0894312613418195	-3.4638072082538	0.000532588067742177	0.0144939381406105
**Xrn1**	5482.94322431288	-0.379249370084944	0.109550191122489	-3.461877758487	0.000536420736891447	0.0145730716399975
**Tex10**	7565.92699322524	0.180718627651427	0.0522260147209752	3.46031816934418	0.000539537477869955	0.0146157885238923
**Mgat4b**	289.308298440495	0.796023127247255	0.230053641821867	3.46016312084129	0.000539848252897461	0.0146157885238923
**Hck**	366.92694232809	0.798867109583642	0.231201618120738	3.45528338459317	0.000549714710869422	0.0148373997438727
**Rab11fip3**	565.679183963485	1.06627792394371	0.308602337101881	3.4551842152501	0.000549916954396247	0.0148373997438727
**Lfng**	3872.59070715092	-0.377231977105362	0.109196116252321	-3.45462815026944	0.000551052264214758	0.0148426162858666
**Qpct**	630.757391406665	-0.428223915194639	0.12401303855142	-3.45305558348271	0.000554274780186552	0.0149039380740606
**Yrdc**	2211.36500910659	2.91909168931728	0.845657388908617	3.45186091625662	0.000556734625007059	0.0149445783411179
**Hectd1**	17109.495770656	-0.294326216193313	0.0853514821901438	-3.44840193328595	0.00056391420255922	0.0150974406398071
**Larp1**	7061.56119268898	-0.200273813332232	0.0580807605208163	-3.44819543574078	0.000564345531309663	0.0150974406398071
**A530088E08Rik**	865.249568423006	-0.495511358889715	0.143754763906871	-3.44692130836599	0.000567013711946355	0.0151431102697267
**Rel**	1216.0096100784	-0.526021937316145	0.15266643338302	-3.44556380639629	0.0005698694122496	0.0151936249218561
**Ddr1**	485.102528085339	-0.512632196307483	0.14888721437665	-3.44309078824353	0.000575106212947165	0.0153073456037305
**Xlr3a**	260.598373171442	1.80459255776628	0.524827118060015	3.43845143604016	0.000585051467110615	0.0155457942112343
**Zfp553**	3089.10814457559	-0.361662602925187	0.105205689480042	-3.43767152435037	0.000586738980782925	0.0155643874077383
**3830406C13Rik**	1960.32620490009	-0.291098220585528	0.0847665573849571	-3.43411634925245	0.000594488961539798	0.0157434664991304
**Slc28a2**	16342.7198134866	-0.282171872631163	0.0822335438802156	-3.43134759025083	0.000600590507582589	0.0158783634697632
**Trim33**	2073.18706438276	-0.412505624525017	0.120324762098921	-3.42826877302188	0.000607443748059892	0.0160326484726628
**Vgll4**	9063.99690100973	-0.346597210443867	0.101179400303444	-3.4255709107229	0.000613508763581383	0.0161656481400533
**Insyn2b**	567.139669929274	-0.609312210312586	0.177997524638823	-3.42314990924143	0.000618999298726101	0.0162559532500453
**Entpd4**	2774.95109355779	-0.33516043144851	0.0979079784005161	-3.42321879099021	0.00061884245347936	0.0162559532500453
**Ctla2a**	3200.55051405288	0.357154357414787	0.104382746308118	3.42158421814786	0.000622574402420154	0.0163129351019689
**Sag**	148.863131053605	-0.719349867797708	0.210256677223079	-3.42129380763726	0.000623239635170736	0.0163129351019689
**Gm15675**	636.178185297222	-0.768068086167537	0.224625395399694	-3.41932881097817	0.000627758186618754	0.0164039564619431
**Phtf1os**	154.726186125438	-0.965976447156848	0.283083921225882	-3.41233243828802	0.000644095114889804	0.0168029912670838
**Nudt16**	6396.95313984468	-0.341571635551921	0.100124071659057	-3.41148367112999	0.000646103710093713	0.016827530842887
**Scml4**	16032.140805372	-0.267566484829657	0.078504192401742	-3.40830822716316	0.00065367013476847	0.0169965021675689
**Whamm**	715.696998042205	1.76651705072467	0.518480611600675	3.40710339248945	0.00065656250837553	0.017043583928292
**Sdc3**	393.114647337644	1.58467910941945	0.465209438066511	3.4063778155612	0.000658310094031056	0.0170585637359537
**Npat**	3690.85861444694	-0.283601237323703	0.0833013860553862	-3.40452002965641	0.00066280439288821	0.0170585637359537
**Fam210a**	1550.22287769448	-0.336438923075232	0.0988044104796605	-3.4051002525286	0.000661397680925787	0.0170585637359537
**Pik3r1**	9986.98441400949	-0.340610636340643	0.100056650089135	-3.4041778935954	0.000663635182467452	0.0170585637359537
**Mfhas1**	2628.73906231753	1.40881899376118	0.413706944600914	3.40535495511251	0.000660781048584601	0.0170585637359537
**Gm10766**	50.0239850396632	-1.59804909014365	0.469323693055455	-3.40500408095703	0.000661630650439237	0.0170585637359537

**Ythdf1**	2221.58882357629	1.52350136447936	0.447979992985108	3.40082456434611	0.000671829289436043	0.0172130310790955
**Rpgrip1**	1403.32513306236	-0.274416003279926	0.0806900286620437	-3.40086635028065	0.000671726606206972	0.0172130310790955
**Cdk19**	1861.84644140344	-0.461782422346982	0.135850413398275	-3.39919777051517	0.000675838274966385	0.0172876358744242
**Aph1a**	2269.03464815155	0.261500470756335	0.0769430240232863	3.39862481460559	0.000677255525245936	0.0172958108773099
**Gm16069**	164.064187512406	-0.590657440515246	0.173818419301836	-3.39812916771247	0.000678483774067332	0.0172991404983478
**Pax5**	34.1243680905191	-1.50198612039208	0.442201666042437	-3.39660891338193	0.000682264002297966	0.0173674214607577
**Ranbp9**	1853.76952575694	0.428144621414489	0.126073726597546	3.39598608662704	0.00068381835448901	0.0173699251330125
**Pramef8**	5445.43471439415	0.330852038976031	0.0974582976664196	3.39480625968322	0.000686771806680638	0.0173699251330125
**4632427E13Rik**	4930.94635011994	-0.445912821831001	0.131327631027573	-3.39542271753442	0.000685227156335291	0.0173699251330125
**2310044K18Rik**	44.68372640809	-1.03727163237153	0.305509666176412	-3.39521706580825	0.000685742095851678	0.0173699251330125
**Man2a2**	4398.60244545665	0.682983837018743	0.201248527599833	3.39373333640882	0.000689467937338349	0.0174101703343596
**Pias1**	14894.6492564957	-0.21686610230929	0.0639252535808492	-3.39249498689918	0.000692591993450557	0.0174610752652807
**Gm26533**	26.3504945367786	-2.39406513545733	0.705838686579079	-3.39180209441398	0.000694345728521477	0.017477325310404
**Mss51**	137.349765486567	-0.901323564042822	0.265917541939896	-3.38948516697159	0.000700239971384344	0.0175975777178678
**Zmynd8**	4475.93327690179	-0.292036218316945	0.0861800725449897	-3.38867454729154	0.000702313137136156	0.0176215734105962
**Fdft1**	2396.18551299398	0.255616891946396	0.0754745979513455	3.38679368800584	0.000707145439258313	0.0176769146449461
**Ino80d**	3043.34011455568	-0.453161150664819	0.133787090676525	-3.38718144159728	0.000706146702065143	0.0176769146449461
**Grk6**	8879.56992930193	1.49697886547779	0.442042139125388	3.38650715164773	0.00070788431433401	0.0176769146449461
**Camkk1**	1684.65079642296	0.364385172302734	0.107617896654017	3.38591613134944	0.000709410611305421	0.0176869984214233
**Fnbp4**	7587.63750511168	-0.259436278611316	0.0766374139603201	-3.38524312349112	0.000711152362680026	0.0177024135525263
**Lsr**	170.400292696623	1.13142613331538	0.334279069994245	3.38467536521165	0.000712624816108737	0.0177110871095037
**Gm26316**	22.9106009327421	1.58710520229624	0.469188865078162	3.38265743376463	0.00071788117992663	0.017807852433475
**5830408C22Rik**	207.206878175459	-0.523434856812177	0.154756421736228	-3.38231429067505	0.000718778583974749	0.017807852433475
**Sgta**	10378.4388829604	0.199997604725302	0.0591671696997367	3.38021246816868	0.000724298152297007	0.0178603536553113
**Trim28**	6322.66995671557	1.31137311835304	0.387928599834898	3.38044969850421	0.000723673199336735	0.0178603536553113
**Clcf1**	8850.40562837487	-0.424534715151258	0.125576535334293	-3.38068504614431	0.000723053701126751	0.0178603536553113
**Gm48374**	129.729057327369	-0.957672460450853	0.28338837589107	-3.37936394687892	0.000726537579167158	0.0178875822420889
**Rassf4**	436.313168188377	0.890622542536872	0.263604914388469	3.37862647440776	0.000728489147508509	0.0179076497617653
**Dock3**	24.7333817726668	-3.73213582641362	1.10491784524668	-3.37774961502264	0.000730815911346661	0.0179368634191423
**AA386476**	189.773895540515	-0.862856079525592	0.255518843651276	-3.37687846107816	0.000733134370081228	0.0179657826895333
**Cers4**	3211.79589531975	0.449865155090717	0.133246815398765	3.37617941370243	0.000734999729109433	0.0179835259807102
**Ino80**	1818.15170501024	-0.394248704923469	0.116803831123327	-3.37530628175376	0.000737335812538807	0.0180127137956186
**Pgm1**	4399.71312981254	0.26575663968534	0.0787778037580867	3.3734964292916	0.000742200091129998	0.0181034780742034
**mt-Nd2**	20774.3250078614	-0.645516514134446	0.191403385964682	-3.37254490499744	0.000744769407982029	0.0181380704197416
**mt-Atp8**	26180.1903437049	-0.545882612052211	0.161915309448788	-3.37140826219937	0.000747849410603345	0.0181849740167853
**Tpcn1**	3443.68471746773	-0.361386316595811	0.107269882011136	-3.36894485031965	0.0007545652502808	0.0182918225364224
**Syde2**	87.1290710185772	1.73178899112784	0.514043348628895	3.36895515863989	0.000754537031063236	0.0182918225364224
**Gpr107**	3505.70190664687	0.248500088013521	0.0739048553531643	3.36243250630868	0.000772590102627686	0.0186544527901829
**Rc3h1**	4315.03794759537	-0.457463330499799	0.13605832856581	-3.36225893204715	0.000773075945420414	0.0186544527901829
**Zfp870**	56.3419492766708	1.77815503555884	0.528832451735885	3.36241664013256	0.000772634501051834	0.0186544527901829
**Zfp251**	1663.35527080493	-0.420320279566879	0.125237559624978	-3.35618388625202	0.000790260053893755	0.0189530101509953
**Zfp60**	2983.47225450534	-0.336204800433733	0.100169541683696	-3.35635757918671	0.00078976385525861	0.0189530101509953
**Gm37039**	18.8944361371638	-2.21772778073593	0.660685853799658	-3.35670541147748	0.000788771051937553	0.0189530101509953
**Gm37010**	524.371430060984	-0.804594228187643	0.239672269599474	-3.3570601619129	0.000787759695716885	0.0189530101509953
**Jmjd1c**	5316.62177489105	-0.334899970837636	0.0997985104018527	-3.35576121816963	0.000791468723231966	0.0189531499573953
**Tut4**	6283.64996241313	-0.312710833546121	0.0932908837411561	-3.35199776232975	0.000802306672525551	0.0191544639984623
**Trav5-4**	482.901390813519	0.399162924483728	0.119069650192532	3.35234817468847	0.000801291775126133	0.0191544639984623
**Bola2**	1258.81005374383	-0.3788042588518	0.113110073834152	-3.34898781347471	0.000811073630794044	0.0193344738281721
**Usp28**	11019.1261401255	-0.206409916376572	0.0616849038890766	-3.34619823267851	0.000819278018061946	0.0194125770384994
**Cnot6l**	8787.78790674139	-0.396316421276716	0.118430347960426	-3.34640932921303	0.000818654483006926	0.0194125770384994
**Ulbp1**	598.812079251862	-0.625404861987064	0.186842073145442	-3.3472378648905	0.00081621141472255	0.0194125770384994
**Gm44093**	210.032250562938	-0.955131748059833	0.28541469680629	-3.34647009683625	0.000818475069778178	0.0194125770384994
**Frmd4a**	1333.58586529941	-0.404857995385787	0.121078760959133	-3.34375733760966	0.000826519986605289	0.0195547679113206
**Dhx9**	1437.71361037786	-0.590864901590778	0.176752153569712	-3.34290072091108	0.000829075555909792	0.0195858223905106
**Trav9d-3**	475.174671969399	0.571769022588543	0.171108151330143	3.34156507532682	0.000833074854800246	0.0196508390525262
**Cited2**	1759.39701172683	2.48418935650825	0.744150732628379	3.33828785968397	0.000842963708551445	0.0197951999338973

**mt-Cytb**	208499.351114836	-0.599339139508251	0.1795341592796	-3.33830142360185	0.000842922556592155	0.0197951999338973
**A630072L19Rik**	118.247530988504	-0.848807693013854	0.254233446577812	-3.33869404061304	0.000841732191846234	0.0197951999338973
**Maml2**	3357.72388931096	-0.600301996709809	0.179959149260449	-3.33576814058513	0.000850640764221941	0.0199457537527457
**Gm27206**	101.271135571292	-1.08190703528951	0.324541198641575	-3.33365082713083	0.000857141856414327	0.0200683272385149
**Sos1**	880.525544125697	-0.424589578223415	0.127387916669222	-3.33304436813982	0.00085901242568854	0.0200822830735524
**Rnpc3**	2421.55282216638	-0.336980171184889	0.101141152711108	-3.33178100260941	0.000862921328256506	0.0201437798064263
**Gpr137b-ps**	604.840475489112	0.338434661460469	0.101608681070029	3.33076522494391	0.000866076139205749	0.0201875173453624
**Rictor**	11058.2907487902	-0.284603090609925	0.0854672224018964	-3.32996770705409	0.000868560568027299	0.0201938841437055
**Gm43681**	97.5848156313258	-1.20275551528389	0.361203578949786	-3.32985492220468	0.000868912448399589	0.0201938841437055
**Rpn1**	16501.8837247107	0.269162310613164	0.0808475868863538	3.32925596148616	0.000870783377393117	0.0202075606444526
**Gm10388**	26.8279805920715	-2.09276444511141	0.628796368214607	-3.32820695363361	0.00087406908700827	0.0202539802999843
**Scamp3**	20253.5273898938	0.19416596196629	0.0583511188108681	3.3275447998801	0.000876149003388067	0.0202723639447662
**Bbc3**	4969.31889858403	-0.485760993635019	0.146098820777144	-3.32487963319012	0.000884567149721504	0.0204371328125539
**Gm36999**	125.757838379282	-0.771247171357657	0.232215518680238	-3.32125594250084	0.000896133183654966	0.020674041837264
**Tmx2**	13344.547704855	0.26660295233105	0.0802816592176673	3.32084507132831	0.00089745340604499	0.0206742299986124
**Slamf1**	1258.97287689168	0.404764067462329	0.121983176294737	3.31819583451683	0.000906009385932055	0.0207629367457984
**Strn3**	3344.95530495858	1.27114066273541	0.383101990829093	3.31802155343661	0.000906574886152778	0.0207629367457984
**Rbm26**	3114.17069430942	0.548450404582249	0.165238072879149	3.31915275351445	0.000902910234816134	0.0207629367457984
**Ppfia1**	2407.58267544642	1.18198040460811	0.356191013316763	3.31838918001272	0.000905382408790761	0.0207629367457984
**Arl5a**	1748.15993339062	1.53898887806451	0.464057420701739	3.31637596859733	0.000911930563000964	0.0208468392669425
**Snx22**	643.744395540051	0.552716045511593	0.166677343552828	3.31608384037157	0.000912884374829618	0.0208468392669425
**Cacnb3**	160.99694145955	1.16393704704287	0.351228984203927	3.31389805337672	0.00092005045904114	0.0209195311444607
**Gabpb2**	3292.87766512154	-0.498371455056918	0.150369217478844	-3.31431833863894	0.0009186685224672	0.0209195311444607
**mt-Nd3**	681.670446875877	-0.981386387336315	0.296114181888599	-3.31421609420086	0.000919004534421579	0.0209195311444607
**Gm17259**	297.55114008018	-0.965745690283607	0.292145917914194	-3.30569633551154	0.000947407029101083	0.021510508007991
**Drc1**	523.258411070913	0.92127433077729	0.278912629412591	3.30309291736827	0.00095624696880688	0.0216799762409928
**Foxp1**	23088.7811525857	-0.432891133493635	0.131095540954811	-3.30210417792055	0.000959624217271144	0.0217146954999072
**Tspan3**	4270.56684202887	0.384348576421175	0.116404424133673	3.30183821862138	0.000960534541057012	0.0217146954999072
**Uchl3**	3109.10269880644	0.309061055514311	0.0936353083393068	3.30068924848695	0.000964476427326788	0.0217725717269172
**Prnp**	130.328134303233	0.880037962302545	0.266775699591106	3.29879356947204	0.000971012887868841	0.0218887697770377
**Spi1**	650.298974769216	0.763756716053186	0.231598167262867	3.29776666663477	0.000974570842400532	0.021937589662436
**B4galt7**	8066.32795186079	0.227976950846888	0.0691447651076141	3.29709632380809	0.000976899916630677	0.0219586476267469
**Nmt1**	6513.97315953707	0.203953739610281	0.0618681101414276	3.2965891336272	0.000978665548820889	0.0219669986506706
**Yae1d1**	1811.42423233507	-0.332745697049373	0.100984758650295	-3.2950090835158	0.000984184984554116	0.022059463444693
**Ppil2**	19768.875646711	0.16717555743794	0.0507751819758143	3.29246594364882	0.000993129250601689	0.022228320457004
**Aldh3a2**	6299.25164826795	0.26746147656887	0.0812444612686193	3.29205797407604	0.000994571071615837	0.0222290161353911
**Lcor**	3939.75632721172	-0.329925529137419	0.10023622727656	-3.29147991800537	0.000996617320407679	0.0222334620980911
**Car12**	861.444561561378	0.501325697986817	0.15232284386799	3.29120495164394	0.000997592035498533	0.0222334620980911
**Wdr26**	3307.18319780729	-0.293230777493359	0.0891683624166232	-3.28850692719118	0.00100720305856388	0.0224159584658066
**Tmem143**	423.27869423682	-0.364110721192439	0.110747803678164	-3.28774665591161	0.0010099267695985	0.0224448746242082
**Rfx1**	3555.40508278958	-0.543276265804095	0.165339351508272	-3.28582555119623	0.00101683963607352	0.0225666790783246
**Fbxl20**	3270.07981325864	-0.284523229969671	0.086633053737873	-3.284234108041	0.00102259939556129	0.0226321820832169
**Gm30934**	28.1375568697953	-1.82619394910042	0.556051657521383	-3.2842163572369	0.001022663809307	0.0226321820832169
**Poc1a**	539.539107679009	0.517392421473628	0.157785098643497	3.27909559218033	0.00104140353210984	0.023006815168995
**Yif1a**	4490.40028840403	0.285604992604231	0.087110892243464	3.27863697924251	0.00104309726220663	0.023006815168995
**Amfr**	8232.09271447039	1.48236164372563	0.45221446675793	3.27800579745525	0.0010454324846735	0.023006815168995
**Supt7l**	2339.37611101882	0.280896907953006	0.0856898007435183	3.27806699882253	0.00104520584256821	0.023006815168995
**Usp3**	10994.3577359947	-0.202956834064863	0.0619403875283632	-3.27664779255582	0.00105047318915888	0.023085503544737
**Pecam1**	12955.2141593248	-0.311914854207454	0.0952130607199002	-3.27596709788642	0.00105300827486818	0.0231089852187992
**Gm44901**	202.526902092163	-0.901915856435169	0.275346032471221	-3.27557237102895	0.00105448093305707	0.0231091183062312
**Tlr7**	384.586503204746	0.586249092934419	0.179036876072751	3.27446002071774	0.00105864117844665	0.0231680681233109
**Mecp2**	2888.9166486893	-0.209664610349238	0.0640759920981792	-3.27212429310472	0.00106742638133737	0.023327929945538
**Sesn3**	2811.13252745048	-0.45066431858859	0.137772046704925	-3.27108676518252	0.00107135035126559	0.0233812569042825
**Copb2**	11114.0070386756	0.162777932177652	0.0497847830341342	3.26963225020075	0.00107687385075778	0.0234459982546346
**Camk2d**	7545.45999542476	-0.145229774138621	0.0444192652358639	-3.26952220770557	0.00107729280550584	0.0234459982546346
**Cstf3**	2996.65044728995	-0.260334089236836	0.0796800753315573	-3.26724200690772	0.00108600800641995	0.0236030733202195

**Gm47884**	32.6630445076161	-1.76585641736772	0.540723944408075	-3.26572632048094	0.00109183717208476	0.0236970775765007
**Setd2**	6328.23087322559	-0.384382184918767	0.11776154288441	-3.26407225571135	0.00109823153688151	0.0238030733516396
**Tifab**	364.023892241298	0.938121407450693	0.287534907405312	3.26263484290034	0.00110381646410551	0.0238663281860447
**AC161757**	649.657105303955	-0.640470265338732	0.196310216385693	-3.26254169105694	0.00110417930111231	0.0238663281860447
**Stk38**	28397.1750067933	-0.187737845182357	0.0575801655042092	-3.2604603258501	0.00111231527362041	0.0240092489951189
**Ficd**	915.732158166121	0.590406743853229	0.181115814171762	3.2598298859387	0.00111479055381323	0.0240297602687211
**Pum2**	8655.83478063562	-0.247258574713643	0.0759066862553786	-3.25740177725282	0.00112437163415305	0.0241701553060704
**Mpig6b**	102.680361748718	1.10887360302906	0.3404003104966	3.25755755454912	0.00112375467399472	0.0241701553060704
**Olfm1**	82.5949438957662	1.78581130449067	0.548621358520927	3.25508891834832	0.0011335686913642	0.0243015535643886
**Ung**	2947.96559131655	0.217016687573589	0.0666631955465003	3.25541981290427	0.00113224864354684	0.0243015535643886
**Lmf1**	1972.9928505028	0.275478880857635	0.0846410983586591	3.25467044024308	0.00113524017631821	0.0243043199160951
**Gm30671**	65.5903743629027	-1.03050307406399	0.316890586708706	-3.25192074894688	0.00114627978375703	0.0245073684567971
**Rubcnl**	142.366084091652	1.14212509689479	0.351287786335755	3.251251940206	0.0011489799141859	0.0245318109862158
**Brpf3**	603.925727737587	1.10149074107367	0.338903497954343	3.25016043718162	0.00115339918499208	0.0245928429741816
**Trap1**	14715.4463501315	0.237979083966556	0.0732592620332545	3.24845046703488	0.00116035409608831	0.0247077020163021
**Ddx41**	11349.0829360716	0.223881840665901	0.0689383957360201	3.24756383254395	0.00116397551734683	0.0247290984651013
**2810454H06Rik**	221.868848788572	-1.08590259266017	0.334387661855391	-3.24743618420281	0.00116449775091103	0.0247290984651013
**Psmd2**	30498.961188393	0.180417790266736	0.0555720989233386	3.24655346409753	0.00116811505648439	0.0247474701901236
**Mndal**	8963.54656082864	-0.236118582976287	0.0727311218132939	-3.24645869731558	0.0011685040186236	0.0247474701901236
**C130051F05Rik**	151.881902653264	-0.92353004962603	0.284509171350238	-3.24604667485092	0.0011701965213516	0.0247500491099827
**Spon1**	625.767319668254	-0.530257253813873	0.163482448457506	-3.24351181926237	0.00118065912104832	0.0249378629629469
**Alg3**	4054.01326916951	0.314248990814953	0.0970094141683087	3.23936592658666	0.0011979577263358	0.0252628177628754
**Gm14966**	108.947383940305	-0.783406820225385	0.241890979068276	-3.23867728859893	0.0012008536208919	0.0252628177628754
**Gm47615**	27.7889046403347	-1.32355648437484	0.408625143884303	-3.23904807176915	0.0011992935825235	0.0252628177628754
**Ube2h**	11228.9986557293	0.529705177790378	0.163705616670263	3.23571779981938	0.00121337277699454	0.0254921531294707
**Zdhhc21**	1202.30938016792	-0.414823068811854	0.128220110138015	-3.23524186935529	0.00121539726253954	0.0254987273433907
**Far1**	5428.46208392463	0.263946355026522	0.081593765438919	3.23488386161209	0.00121692219091387	0.0254987273433907
**Srsf2**	12897.6622516266	0.630735711598804	0.195021045870111	3.23419305226621	0.00121986967866	0.0255265425320659
**Tefm**	949.026924238101	0.443606646233561	0.137183388461332	3.23367611202139	0.00122207962801524	0.0255388709530984
**Slain1**	1700.10975489933	2.28404241655026	0.706785268244947	3.23159312901622	0.00123102202205721	0.0256653477903027
**Rps15a-ps4**	101.279607370253	-1.45196189646096	0.449314055007279	-3.23150785131225	0.00123138940975242	0.0256653477903027
**Gm12966**	600.282044380001	0.741364304423082	0.229855019482374	3.2253561662156	0.00125816038293211	0.0261886831623001
**BC051226**	612.173624400965	-0.424757084344264	0.131722386970878	-3.224638530413	0.0012613181561534	0.026219775971648
**Eif2b5**	8458.61608990671	0.250268314735336	0.0776385959152452	3.22350387439442	0.00126632585455613	0.0262849821042804
**Ocstamp**	62.001645159561	2.3807434065433	0.738633539055248	3.22317262981099	0.00126779122924751	0.0262849821042804
**G6pdx**	8751.85457173442	0.208619880770253	0.0647454974275211	3.22215272195246	0.00127231298003091	0.0263440678401407
**Fyb**	27068.5512470682	-0.259490374205415	0.080567075966619	-3.22079920478842	0.00127833677504746	0.0264160167994364
**Plp2**	2711.86173058209	0.270647208177371	0.0840455704770918	3.22024357311182	0.00128081721360471	0.0264160167994364
**Tnfrsf14**	690.350777284575	-0.565345609753708	0.175543627764877	-3.22054190717154	0.00127948484555684	0.0264160167994364
**BC026513**	24.3931438688454	-2.1516709788096	0.668399968754676	-3.21913686324443	0.00128577100580387	0.0264835212267342
**Gm10840**	88.3484818135209	-1.48809558508629	0.462493512390123	-3.21754910116674	0.00129290894895086	0.0265957784707817
**Ppp1r13b**	3957.64303440839	1.53215266415973	0.476371767884063	3.21629611042068	0.0012985676948921	0.0266773548479984
**Gm43668**	153.314042223055	-0.934443795574901	0.290599063108511	-3.2155774543085	0.00130182358960521	0.0267094196632934
**Hras**	406.907475582105	2.26495751060467	0.7047066011817	3.21404327248621	0.00130879947480259	0.0267999879888483
**Kcnq1ot1**	4742.94132036509	-0.493328738297948	0.153500428895836	-3.21385902206642	0.00130963957297793	0.0267999879888483
**Cd101**	1232.41322488301	-0.332731778778305	0.103550256588619	-3.21323953932988	0.00131246778336814	0.0268230283560724
**Apc**	2866.35631270505	-0.384057937800927	0.11954447163365	-3.21267836607193	0.00131503464975281	0.026840674839579
**Pcf11**	5906.99352298018	-0.284764397033614	0.0886557504276798	-3.21202398783944	0.00131803369591748	0.0268670853125119
**Gorasp2**	12801.5401157361	0.17097343979399	0.0532356863439484	3.2116321125148	0.00131983269911946	0.0268689972093351
**Btnl7-ps**	196.23311065986	-0.60005188055126	0.186954410084184	-3.20961607849241	0.00132912368051832	0.0270232281728092
**Uhrf1**	1665.02703026295	0.481262254492001	0.150015061501642	3.20809290530293	0.0013361832709225	0.0270796477961491
**Lsm6**	4029.31450768159	0.224962184190449	0.0701178082209492	3.2083459237854	0.00133500819200929	0.0270796477961491
**Gm5884**	821.272236714878	0.403636185810942	0.125825461431436	3.20790546856757	0.00133705438759942	0.0270796477961491
**Smurf1**	2836.06532447472	1.58877325330278	0.495414763688457	3.20695580703744	0.00134147602015889	0.0271343230419046
**Rere**	3251.73594826802	-0.573123569949564	0.178795415575383	-3.20547128182896	0.0013484150163875	0.0272048340758232
**Gimap6**	88158.6086854678	0.116837900433191	0.0364459894221358	3.20578209799481	0.00134695945875762	0.0272048340758232

**Lypd6b**	3065.16110645186	-0.580620298412517	0.181237587448912	-3.20364173119544	0.00135701228370194	0.0273432769236464
**Gm1972**	89.1190845490454	-1.03226443490991	0.32247829452896	-3.20103539501077	0.00136934710033119	0.0275565801531528
**Hs3st1**	71.5198913189856	0.922295649112032	0.288203739432533	3.20015156960841	0.00137355334044146	0.0276059693690512
**Psmc1**	9411.01900406345	0.258564578541971	0.0808264371126854	3.19900997468798	0.00137900397593851	0.0276802110176599
**Rnf167**	46105.8009026766	-0.215655695932065	0.0674662241227882	-3.19649867375966	0.00139106465996826	0.027886775886921
**Gm4759**	18547.3149470706	-0.473305775945319	0.148106531531579	-3.19571170191371	0.00139486411321212	0.0279274127469929
**Tmem199**	2479.42034499816	0.357627308309915	0.111937267345736	3.19489046668725	0.00139883918964791	0.0279714582630484
**Mcm2**	9412.97903329147	0.213677190979554	0.0669219733597253	3.19293021786695	0.00140836976583858	0.0281237868788744
**Zbtb5**	1433.5116735665	-0.249841542455142	0.0782566790895427	-3.19259065631023	0.00141002675853975	0.0281237868788744
**Gm38120**	101.560211252772	-1.08201697680089	0.338979339110709	-3.19198503259665	0.00141298654405723	0.028147192129848
**Srm**	9731.8914308724	0.26402100434284	0.0827948231153001	3.18885884900272	0.00142835603666877	0.0284174319063003
**Sgtb**	33.7435901355521	-1.74890622203452	0.548806460753004	-3.18674495856862	0.00143883589541843	0.028589832539859
**Gm11400**	260.836195260775	-0.680137078420572	0.213451405823267	-3.18637900648783	0.00144065732642996	0.0285899716530943
**Ranbp6**	1190.99673021343	-0.376692004215444	0.118235131723977	-3.18595665030291	0.00144276213600136	0.0285957270150609
**Atl1**	36.1585335265824	1.66134935869479	0.522469404336038	3.1798021949363	0.00147375614403264	0.0290936354155196
**Ptprj**	145.807478302545	1.29109710797926	0.405934817257783	3.18055277125778	0.00146994364156433	0.0290936354155196
**Trim44**	1300.47732812486	0.684970528179981	0.215433082537472	3.1795048379389	0.00147526906752555	0.0290936354155196
**Gm42646**	170.497042645199	-1.12174356940834	0.352779309488061	-3.17973174514165	0.00147411445644277	0.0290936354155196
**Azi2**	7430.34203026747	-0.21167817793226	0.0666026729324918	-3.17822346479722	0.00148180495520624	0.0291860008489808
**Ifitm10**	6197.69297086334	0.443617831474026	0.139626480124379	3.1771754976481	0.00148717013337669	0.0292186281690979
**Cerox1**	183.341391161491	1.19572942572823	0.376347416051055	3.1771957896637	0.00148706607655548	0.0292186281690979
**Tmem39a**	2341.79694994451	0.274340373903768	0.0863738394788352	3.17619751025415	0.00149219318286877	0.0292808069520089
**Brsk2**	52.1901368573131	-1.52563846001279	0.480549025565496	-3.17478213220277	0.00149949040358405	0.0293874008573058
**Etv3**	5711.62956093295	-0.393162582929333	0.123917055158358	-3.17278830123017	0.00150982572226933	0.0294159958189131
**Gadd45g**	1354.64138509502	3.1508602680425	0.992986521346755	3.17311484124588	0.00150812857230649	0.0294159958189131
**Fkbp1a**	10709.9438157234	0.151688015273922	0.0477955640099131	3.17368396871435	0.00150517480529448	0.0294159958189131
**Mlycd**	1739.45960436952	2.34818331965338	0.740199012545555	3.17236753880276	0.0015120151752977	0.0294159958189131
**Gm11342**	445.419714760293	-0.530326493832467	0.167171941523835	-3.17234153649436	0.00151215057519322	0.0294159958189131
**Gm16083**	123.653120054358	-1.01782199101046	0.320710053711568	-3.17365164961695	0.00150534239831634	0.0294159958189131
**D1Ertd622e**	2166.47705335279	0.581589242594844	0.18338042818913	3.17149026391748	0.00151658951719919	0.0294659692016123
**Clec11a**	141.872260433911	-1.03579185925132	0.326727007430144	-3.17020581615915	0.00152330996485765	0.0295600925077119
**Ccnt1**	4045.48004182811	-0.351967676527709	0.111055244195721	-3.16930262120184	0.00152805204781252	0.0296156409807896
**Pank1**	176.902142645351	1.57290332838053	0.496511601393665	3.16790851203783	0.00153539829546344	0.02972146307324
**Hnrnpd**	2765.56327225193	1.81032440574291	0.571536232799996	3.16747093508666	0.0015377108024365	0.0297297044343459
**Kansl2**	8180.27506257956	0.293064272547967	0.0925390816718642	3.16692436593598	0.0015406038171573	0.0297491352291024
**Kifbp**	4050.12737700572	0.28126133434168	0.088840915807191	3.16589863787643	0.00154604657606671	0.0298176938789268
**Fubp1**	13163.6162145655	-0.157374195722445	0.0497268688517132	-3.16477186994698	0.00155204588367308	0.0298869777428019
**Slc25a4**	15138.205972158	0.169196852961463	0.0534669647732513	3.16451202493001	0.00155343242821316	0.0298869777428019
**Gm44141**	130.826366640477	-1.03290805809721	0.326462397369078	-3.1639419008783	0.00155647863336906	0.029909065641459
**Prrc2c**	17021.7678060345	-0.401898546634805	0.127100677407268	-3.16204881699412	0.00156663298793409	0.0300675225223843
**Gm48375**	224.334788198949	-0.653996465807914	0.207008977174253	-3.15926620543321	0.00158166946077052	0.0303191796756218
**Cebpb**	16.8743896325937	3.37713997882754	1.06946379608865	3.15778803469435	0.00158971102238927	0.0304173407187168
**Gm1966**	13175.3188893923	-0.589348961796443	0.186643663971316	-3.15761569000818	0.00159065105998747	0.0304173407187168
**Eif2ak2**	927.953474324382	-0.347273248548019	0.110019600160108	-3.15646710261301	0.00159692900101924	0.0305003760837093
**Cdk12**	3178.19390871954	-0.383878429841716	0.121707643777793	-3.15410288069152	0.00160992320347971	0.0305072350076227
**Dbnl**	26905.5128274659	0.185193230679218	0.0586942684274503	3.15521831417199	0.00160378049510056	0.0305072350076227
**Tanc1**	6492.68025043448	-0.385015487970102	0.122045224945104	-3.15469522173671	0.00160665848136124	0.0305072350076227
**Heatr5a**	2742.37851772908	-0.293860901534704	0.093172138170998	-3.15395682983451	0.00161072910912245	0.0305072350076227
**F13a1**	112.856206017187	1.3587663453125	0.430658035469086	3.15509344631754	0.00160446707021207	0.0305072350076227
**Btaf1**	3303.34209581842	-0.357031484816364	0.113128828710886	-3.15597261003029	0.00159963881231348	0.0305072350076227
**Msantd2**	544.780538976274	0.803588812931457	0.25478914748639	3.15393658191184	0.00161084086604951	0.0305072350076227
**Clns1a**	10562.1933084362	0.188977305451371	0.0599835018674165	3.15048804368031	0.00162997928086853	0.0307667478391723
**Egln1**	1100.30024722335	-0.393325094964737	0.12486262195235	-3.15006275548849	0.00163235395021565	0.0307667478391723
**Pom121**	1862.78838100833	1.29213827130423	0.410104078646452	3.15075693850432	0.00162847950159264	0.0307667478391723
**Lcmt2**	2678.32357801356	-0.257501837056726	0.0817377586192376	-3.15034130378173	0.00163079826871825	0.0307667478391723
**Akt3**	443.408320404419	-0.530241350017408	0.168436215522264	-3.14802460013311	0.0016437785920672	0.0308712983017913

**Ppbp**	622.595970091099	0.676307556384923	0.214827558968379	3.14814151234883	0.0016431212701841	0.0308712983017913
**Gm44430**	29.7398290613952	-1.51904469344976	0.48252340149846	-3.14812647165385	0.00164320582073889	0.0308712983017913
**Ap3s2**	2505.90264849263	0.212039682362753	0.0673761060589518	3.1471050312291	0.00164895717681564	0.0309316883751001
**Ppp2r5e**	1389.28966987841	-0.31491317842756	0.100095360417573	-3.14613162002533	0.00165445532951217	0.0309979222676853
**Bcl7a**	1306.75907867644	-0.307209090447192	0.0977195403647703	-3.14378362096704	0.00166778710409327	0.031210595485983
**Slc25a17**	4428.82038367499	0.209603952227886	0.0666845408586551	3.14321654657808	0.00167102169632345	0.0312340318730351
**Ahsa2**	2584.66181371039	0.336455439268858	0.107058959181937	3.14271165944255	0.00167390642824894	0.0312508810307091
**Ank**	1552.80293842125	1.51658743068958	0.483062696510835	3.13952503814494	0.00169221952802353	0.031555388287653
**Mcur1**	1931.45310093783	1.20301412730659	0.383413504879929	3.13764150713295	0.00170313041360524	0.0317213072425268
**Rfc5**	3641.46387847593	0.213272221896671	0.0680095576147766	3.13591544154278	0.00171318591631825	0.0318709214680362
**Prrc2a**	2323.07590626254	-0.460677442257535	0.146985025084933	-3.13417943080486	0.00172335440530091	0.031951637561626
**Fam169a**	32.4847694118784	-1.25369447582512	0.400012755072882	-3.13413624922209	0.0017236080426085	0.031951637561626
**Zfp458**	169.111518163768	-0.998334984064095	0.318471774703928	-3.13476754727232	0.00171990337870783	0.031951637561626
**Fuz**	2855.63344345101	-0.223299254589217	0.0712741460294726	-3.13296289087603	0.00173051319596879	0.0320043385315497
**Hdac6**	1412.21729058174	0.526430993472464	0.168025324022945	3.1330455485423	0.00173002592821021	0.0320043385315497
**Adck1**	2754.25836498654	0.214799236309019	0.068619924396795	3.13027503596392	0.00174642707075053	0.0322094410370834
**Ifi205**	383.844799692569	0.890971386697013	0.284650392850011	3.13005500458412	0.00174773574200078	0.0322094410370834
**Suclg2**	4053.07214928091	0.283346413024499	0.0905073126567761	3.13064662630093	0.00174421902423895	0.0322094410370834
**Ago2**	4736.95283649381	0.70827485150278	0.226444411462967	3.1278089263802	0.00176114632037807	0.032418671226866
**Dhx15**	11724.5666207616	-0.174642896202519	0.0558591102987617	-3.12648904124043	0.00176907097626492	0.0324508164994253
**Slc19a2**	647.514892593386	0.456861334462355	0.146096448913593	3.12712141780092	0.00176527007443516	0.0324508164994253
**Gm28424**	34.7918183763261	-1.63254095201222	0.522146193949605	-3.12659743751725	0.00176841892619164	0.0324508164994253
**Lrrcc1**	1246.75547085907	-0.359114748509329	0.114904775183647	-3.12532484342249	0.00177608807324687	0.0325416508955243
**Zfp598**	1023.89273983329	1.07087930443848	0.342753921461643	3.12433859216493	0.00178205262267981	0.0326129542380989
**Gm47257**	31.677322064	-2.2088028657945	0.707043717794809	-3.12399758346417	0.00178411922023489	0.0326129542380989
**mt-Co1**	493370.862905664	-0.575592571481733	0.18435719967912	-3.12215944093082	0.00179529681191179	0.0327792489748482
**Slc25a27**	889.207857267287	-0.468084799096786	0.150045213332786	-3.11962500302238	0.0018108140883945	0.0329915315976461
**Gm37233**	100.356026582054	-1.09000215738277	0.349407069206006	-3.11957671566203	0.0018111109241584	0.0329915315976461
**Csnk1g2**	3193.17966437136	1.88686425661476	0.605096593114148	3.11828603579464	0.00181906168142198	0.0330599249298342
**Mrps6**	962.290863928227	0.37557198828483	0.120429830066526	3.11859601626409	0.00181714923980419	0.0330599249298342
**Decr1**	811.075802932244	0.384537837775456	0.123366661169646	3.1170320581722	0.00182681705624188	0.0331626225290361
**Gm527**	37.5574189939841	2.70710294594895	0.869120975705632	3.11475964983019	0.00184094850223375	0.0333806968350945
**Ksr1**	540.561203202962	0.549382300837949	0.176408811157581	3.11425657954921	0.00184409049810199	0.033399234458153
**Manbal**	2141.31475231619	0.191193895385163	0.0614043620945766	3.11368588261988	0.00184766083031511	0.0334254784193746
**Ccdc181**	534.314215016091	0.398974281379741	0.128226991643558	3.11146878099425	0.00186159156672447	0.0336388742166027
**Myl9**	512.651066445693	0.666731364852315	0.214426493907233	3.10937026811977	0.00187486599697927	0.033839935297139
**Inpp4b**	34026.8608654884	-0.449953800664347	0.144758096018297	-3.10831527244924	0.00188157230987585	0.0339221222960112
**Zc3h14**	9469.1562528655	0.604165218215913	0.194412788760072	3.10764133403551	0.00188586787436846	0.0339607086816272
**Dkk3**	69.9280462449488	1.32591432990226	0.426804645272026	3.10660707325989	0.00189247759143234	0.0340020175692125
**Cbx6**	1412.35843847829	-0.294200327604561	0.0946989591342572	-3.10669019273449	0.0018919456092004	0.0340020175692125
**Sap30l**	288.10574020436	0.983210392531683	0.316646992677323	3.10506783664172	0.00190235389414771	0.0340563309202363
**Smc6**	9708.38371959301	-0.206460381872505	0.0665042973432583	-3.10446678064833	0.00190622332277351	0.0340563309202363
**Retreg1**	3379.79983389793	-0.349470385074508	0.112570652714715	-3.10445375101593	0.00190630728385152	0.0340563309202363
**Uqcrc1**	37985.5658087997	0.208481725831083	0.0671271426035744	3.10577387543949	0.00189781783017049	0.0340563309202363
**Ankrd11**	15184.2137191866	-0.298830089931803	0.0962539019322955	-3.10460234788195	0.00190534994847917	0.0340563309202363
**Gm45546**	92.5429146570361	-1.1191786783588	0.360562241453366	-3.10398191959198	0.00190934998699452	0.0340720586014412
**Dmac1**	3648.21894839113	0.217336426072418	0.0700388738120612	3.10308282020079	0.00191516036679265	0.034137083596778
**Ankrd27**	5805.08655583955	0.192261809080922	0.0619868534452345	3.10165459924152	0.00192442355639801	0.0342634372634615
**Cmpk1**	3954.17237510499	0.274087968061947	0.088399081667899	3.10057483506052	0.00193145400676141	0.034349797725214
**Bmt2**	1278.18079977873	0.847993717020392	0.27354775398323	3.09998420631294	0.00193530961919858	0.0343719048370404
**1700120C14Rik**	181.913097556749	-1.07747902363527	0.347605672619327	-3.09971645605235	0.00193705981438674	0.0343719048370404
**Srsf1**	7646.51948269002	-0.163034759848413	0.0526290259090325	-3.0978107048801	0.00194955912649309	0.0345159586024176
**Gm37090**	54.163098763951	-1.08077773296627	0.348884116360587	-3.09781294786503	0.00194954437192986	0.0345159586024176
**Ranbp2**	5138.0383024413	-0.409972726596183	0.132517832098904	-3.0937174273286	0.00197665654184348	0.0349172389347845
**Dnajb2**	2259.34003682182	0.344517730849569	0.111349082269641	3.09403296216928	0.00197455547354589	0.0349172389347845
**Tesk1**	258.275860640512	1.5971290823704	0.516315880918026	3.09331775642976	0.00197932079723117	0.0349251487144138

**Trim34a**	7248.97006342113	-0.279482315300188	0.090428681722918	-3.09063794777578	0.00199727004780046	0.03520244311319
**Coq10a**	814.494675014878	0.798308258760424	0.258424141124063	3.08913964186178	0.00200737063360761	0.0353409375125755
**Pdlim4**	242.682207968782	-0.896662021723847	0.290328408943638	-3.08844051805456	0.00201209968480231	0.0353846593007031
**Wsb2**	1386.64709539748	1.50448083186866	0.487245201512752	3.08772837002334	0.00201692734471342	0.0354300157978253
**Ciart**	391.059830910975	0.949023915589917	0.307431601272523	3.08694327994166	0.00202226179629577	0.0354841638354481
**Gm2981**	514.653205323204	0.32872579536717	0.106641250001757	3.08253884272506	0.00205242942923941	0.0359734488504176
**Gm11529**	37.232974007889	-1.25463993883174	0.407134702373386	-3.0816335024203	0.00205868137195798	0.0360429359754909
**Arf2**	1831.32392480106	0.193054145412052	0.0626692385768482	3.08052482838641	0.00206636125783101	0.0361372412204697
**Irf6**	2028.04329941244	-0.561385162167231	0.182290476752014	-3.07961870619787	0.00207265755717288	0.0361670710170244
**Unc93b1**	2979.58835445444	0.364309640932257	0.118284702458376	3.07993876943181	0.00207043155166102	0.0361670710170244
**Anpep**	51.699116356281	1.42493840668651	0.462752114690553	3.07926935707118	0.00207508974867505	0.0361694570463194
**Rfx7**	2633.08132485135	-0.354440593776301	0.115142805100914	-3.078269575469	0.00208206476832273	0.0362109211417895
**Gm2822**	67.5092569914423	-1.5906326938138	0.516690786144166	-3.07850021031724	0.00208045382872792	0.0362109211417895
**Smc4**	30790.3595294094	-0.403733808576606	0.13121164856357	-3.07696620686084	0.00209119006794295	0.0363295280050463
**Boll**	79.6416346485878	1.84059579450653	0.598435726873593	3.07567832576165	0.0021002429528149	0.0364466169686172
**B230217O12Rik**	236.328291554722	-1.67378712802607	0.544404688618778	-3.07452739298163	0.00210836358756139	0.0365472882829535
**Mto1**	3995.17993468517	0.249148959755739	0.0810603418237842	3.07362335452964	0.0021147623917554	0.0366179241833954
**Lgmn**	422.732805346841	0.391939161400661	0.127577772982154	3.07215867026843	0.00212516727035058	0.03674836264524
**Apex1**	2855.5046170197	0.310270669974609	0.101002625022509	3.0719069915803	0.00212695987386297	0.03674836264524
**Tnfaip3**	8106.81769238821	-0.413036283428947	0.134671551036658	-3.06698987462109	0.00216226181469184	0.0372023395625198
**Kif2a**	1338.80951877514	-0.306947699815138	0.100102818499049	-3.066324249582	0.00216708166341268	0.0372023395625198
**Med27**	3332.97272492821	0.182168516148861	0.0593895647834385	3.06734889897123	0.00215966617010167	0.0372023395625198
**Tlr12**	3325.33412246102	-0.308638630450516	0.10061329267183	-3.06757310345864	0.0021580466842982	0.0372023395625198
**4833420G17Rik**	6008.55459606805	-0.273167292872751	0.0890875207929367	-3.06628010793638	0.00216740164488121	0.0372023395625198
**Zfp799**	1023.96995131058	-0.316598424306087	0.103240640666151	-3.06660654431495	0.00216503634120195	0.0372023395625198
**Bysl**	5861.2947395799	0.292294237365189	0.0953488900812528	3.06552322859874	0.00217289498336494	0.03725604597702
**Zfp654**	4115.90858988123	-0.282207191046992	0.0921247402207645	-3.06331600361337	0.00218898771794139	0.0374911733386983
**Ppp2r5a**	9328.44123974637	0.477217286118079	0.155806867942575	3.06287708892246	0.00219220080943894	0.0375054377354282
**Gm9574**	84.2821734584226	-0.745105760148679	0.243379092917439	-3.06150274132807	0.00220228975362425	0.0376371796614504
**Gramd4**	17071.1230566062	0.387938803211073	0.126763238073785	3.0603415399129	0.00221084716207266	0.0377424905013856
**Gm28370**	199.886653876074	-0.780068976494625	0.254933000007118	-3.05989799858333	0.00221412385022223	0.0377575211125019
**mt-Atp6**	177549.313382607	-0.509985338900149	0.166713283388505	-3.05905641430917	0.0022203533414331	0.0378228190280663
**Gsk3a**	353.745226030677	1.25847147591028	0.411540425260608	3.0579534807871	0.00222854168446222	0.0379213081231871
**Thumpd2**	1682.61460395033	0.305808068700136	0.100046140499517	3.05667032404526	0.00223810285070035	0.0379240974224152
**Plcg2**	1206.39384166782	0.676180838126079	0.221276105816172	3.05582401512355	0.00224442949496805	0.0379240974224152
**Hgsnat**	4863.80599522315	0.254608075341332	0.0833029488101421	3.05641131530188	0.0022400373520616	0.0379240974224152
**Samd3**	2486.34655035378	0.474909832148975	0.155339032291327	3.05724726840267	0.0022337992327628	0.0379240974224152
**Lair1**	2039.43161613101	0.345307602109764	0.113015483131811	3.05540084014002	0.0022475991121626	0.0379240974224152
**Cd200r2**	23.1948306898567	-3.03523137528931	0.993414407833062	-3.05535268197899	0.00224796008076003	0.0379240974224152
**Gm44751**	60.5103549391069	-1.08494690101045	0.354913475379254	-3.05693352401201	0.0022361386139587	0.0379240974224152
**B930059L03Rik**	189.606835368579	-0.770220388766226	0.252066303650831	-3.05562614919428	0.00224591101772479	0.0379240974224152
**Gm6630**	43.0723153771279	-1.5342734858618	0.502671826491223	-3.05223687703251	0.00227142774643863	0.0382790235300892
**Ggnbp1**	43.1657367588327	-1.28054692767032	0.420248622446108	-3.04711749015795	0.00231047381558983	0.0388539337377256
**Zfp668**	2706.74019773981	-0.255915122741752	0.0839819904840605	-3.04726193397767	0.00230936375264034	0.0388539337377256
**Atp10a**	2252.02504120195	0.363650447927974	0.119378508200236	3.04619695295585	0.00231755971096902	0.0389315440999348
**Klhl28**	3014.24012775257	-0.250489669170527	0.0822467643431451	-3.04558691361339	0.00232226648800182	0.0389690660824758
**Mycbp2**	20546.4398365388	-0.360926711105127	0.11860775728475	-3.04302787075407	0.00234210640143818	0.0392184596891194
**Olfr56**	80.4729325650855	-0.905196318398824	0.297463867663558	-3.04304628830629	0.00234196305981881	0.0392184596891194
**Tut1**	6072.77537167578	0.205685103595519	0.0676184208948521	3.04185014783713	0.00235128917861506	0.0393304284367702
**Crcp**	3935.91977416707	0.265216825679256	0.0872241876524874	3.04063394360192	0.00236080658687797	0.0394477512083099
**Trim25**	6024.95908567755	-0.276345941455858	0.0909069132213682	-3.03987817497363	0.00236673861207923	0.0395049791425132
**Ccdc186**	1255.23671318011	-0.47406929237652	0.155973799301242	-3.03941620003063	0.00237037136907167	0.0395107267375966
**Rhob**	153.148287779628	0.828360072844411	0.272558873746456	3.03919685849223	0.00237209795606819	0.0395107267375966
**Gstt3**	47.5508612740775	1.1932803072835	0.392708836345018	3.03858787184289	0.00237689774390506	0.0395330008594771
**Ly6k**	514.237072246973	0.984926725367515	0.324161842021526	3.03837959219799	0.00237854135973742	0.0395330008594771
**Gm37420**	129.387269220339	-0.928627243968394	0.305663233303474	-3.03807309087259	0.0023809619734495	0.0395330008594771

**Adgra3**	140.458286127494	1.31271151900605	0.432154833702304	3.03759536312472	0.00238473935604729	0.0395506498952942
**Tsc22d3**	104020.981776475	-0.375544109585335	0.123643894814564	-3.0373041074819	0.002387044999075	0.0395506498952942
**Map2k1**	7728.2935680026	0.244588597192225	0.0805729421058977	3.03561705455363	0.00240044026892525	0.0397308165099319
**Sh2d5**	56.4556049279119	1.26050590469908	0.415398458008781	3.03445012950057	0.00240974592196501	0.0398429868755536
**Stat6**	39938.9416378348	0.218382899813433	0.0720085881300227	3.03273408748286	0.00242349050628741	0.0399444978112665
**Gprin3**	5242.17958549352	-0.43930712553669	0.144845539890598	-3.03293512432967	0.0024218766056637	0.0399444978112665
**Trav7-5**	523.585505022309	0.378377625144557	0.124750660191899	3.03307112413283	0.0024207853728092	0.0399444978112665
**Usp9x**	3665.0429118813	-0.459823783895784	0.151685969062188	-3.03141936422126	0.00243406924364301	0.040076937379397
**Gm44777**	151.979419971281	-0.839143629200638	0.276877799483689	-3.03073641427894	0.00243958116787231	0.0401257624866013
**Gm49783**	183.392647348564	-1.0521403320083	0.347370653907328	-3.02886936525441	0.00245470802015884	0.0403324653531208
**P2ry13**	121.370085832181	0.997858838791843	0.329486386883691	3.02852827465703	0.00245748079492495	0.0403359634225338
**Xiap**	1475.68240851358	-0.345245658590857	0.114015257181575	-3.02806542848066	0.00246124792167678	0.0403557580664526
**Mbtps2**	1663.73676702815	-0.446731922740086	0.147548287832699	-3.02769980798844	0.00246422745893774	0.0403626112998773
**Dgkz**	8341.79752937045	1.15568340424146	0.381801437736483	3.0269226095453	0.00247057202044132	0.0404245102036282
**Gk5**	245.520361894395	-0.632700485633034	0.20905223508393	-3.02651863721532	0.00247387569787651	0.0404365761114525
**Atp6v1e1**	5638.63581837839	0.186070215231579	0.061494210098636	3.02581681971563	0.00247962475954071	0.0404885464622622
**Thoc2**	11189.0809874365	-0.188419014581171	0.062282243673017	-3.02524449135735	0.00248432213417618	0.0405232545219607
**Phgdh**	14415.0160920493	0.208533374018169	0.0689439815109546	3.02467843382435	0.00248897604802056	0.0405498920697544
**Gm43652**	25.8102189061378	-1.8114470851194	0.598940288068084	-3.02442016542638	0.00249110208310733	0.0405498920697544
**4930453N24Rik**	5289.37921691167	-0.19273429789118	0.063737244715798	-3.02388813245026	0.0024954869534533	0.0405793477044
**Zfp148**	8191.25708540882	-0.226537581483049	0.0749328393346536	-3.02320829551542	0.00250110025956522	0.0406286977216176
**Vwa3b**	299.27884013783	-0.588504820750048	0.194735068727016	-3.02207930290679	0.00251044769787612	0.0407385420962245
**Treml2**	10660.9670920957	0.297977808708765	0.098637174481625	3.02094834198921	0.00251984346339329	0.0408069614107791
**Gm48621**	43.1036615897033	-1.16844069542057	0.386758905563785	-3.02110870263558	0.0025185092694679	0.0408069614107791
**Ulk1**	1930.28098804266	0.830342539469959	0.275034085788843	3.01905320967181	0.00253565992796722	0.0410209378695887
**Gm47121**	49.8036000824021	-1.43064070085054	0.473931785211711	-3.01866375181284	0.00253892150588485	0.0410315755571565
**Msh2**	10079.8954247433	0.184521285009639	0.06113890942387	3.01806634675753	0.00254393202913364	0.0410704272367405
**Gm44771**	21.7398803435409	-1.77199860228278	0.587240360476326	-3.01750138707337	0.0025486787469567	0.04110494474493
**Tagap1**	5846.80484282257	0.670930067370882	0.222418985560734	3.01651437569244	0.00255699092994307	0.0411968364858005
**Slfn8**	10119.3344843888	-0.329520577677227	0.109324465349456	-3.0141521993628	0.00257698492053452	0.0413566856441246
**Runx2**	1092.14022147312	0.688489403986813	0.228422725719938	3.01410204180362	0.00257741101113984	0.0413566856441246
**Gm16867**	137.085237459973	-0.81554312737255	0.270535442552145	-3.01455188155377	0.00257359190420672	0.0413566856441246
**Gm43275**	42.2917866524467	-1.17322295628823	0.389173224137329	-3.01465487223301	0.00257271824911905	0.0413566856441246
**Gm15859**	19.3208794710594	-1.83851699780483	0.610062731236805	-3.01365237321993	0.00258123384362159	0.0413758918351429
**Kctd2**	1098.74196030329	1.56601128483693	0.519862830975171	3.01235478193232	0.0025922943292531	0.0414866409183395
**Tbl1xr1**	3196.18880741232	-0.290779561653649	0.09653316963057	-3.01222432420332	0.00259340872657006	0.0414866409183395
**Ranbp3**	15554.4948078546	0.201716848622239	0.0669756152890169	3.01179537883719	0.00259707595652188	0.0415031702301371
**Rev3l**	4544.27081837709	-0.386485658687843	0.128418387168793	-3.00958193922687	0.00261607502902069	0.0417221601541286
**1700055D18Rik**	94.4464888354672	-1.30954207857885	0.435083773081268	-3.00986191533796	0.00261366485267847	0.0417221601541286
**Gm47652**	52.548224377541	-0.965321977575259	0.320842244210948	-3.00871220979422	0.00262357506643824	0.0417994664528487
**Igip**	242.472758129131	-0.600457781533027	0.199744152148098	-3.00613447290223	0.00264591957115583	0.0421128835178813
**Csad**	633.330296998293	0.415969510907554	0.138441132897316	3.00466705380175	0.002658717110537	0.0422034998307686
**Stip1**	25433.4498295384	0.258716609764715	0.0861079699303799	3.0045605531508	0.00265964811397812	0.0422034998307686
**Gm8369**	14738.0876062752	-0.405651809781552	0.135009050617815	-3.00462678557658	0.00265906909078897	0.0422034998307686
**Ptpra**	8445.26498478084	1.00443565716627	0.334417499790117	3.00353796615508	0.00266860250461058	0.0422966821796139
**Ppt1**	10959.1122719404	0.328592930532576	0.109411450436173	3.00327734640777	0.0026708890504992	0.0422966821796139
**Map4k4**	1805.80776065516	-0.310982027376994	0.103580853753955	-3.00231187624402	0.00267937520895167	0.042388469043626
**Rnf168**	1412.0707698789	-0.294459876571413	0.0981012955045589	-3.00159009172034	0.00268573554120349	0.0424464743457808
**Trim8**	8278.03995987515	0.702959073095828	0.234260764273318	3.00075463032156	0.00269311481517239	0.0425204510447609
**Mettl7a1**	521.392021867033	0.503316508462067	0.167770645673627	3.00002724815875	0.00269955455371323	0.0425794605634229
**Cox15**	4366.31449555293	0.249343496109478	0.0831371219121452	2.99918364233213	0.00270704087724181	0.0425933558586699
**Vmn2r97**	4674.40751174093	-0.438535541168341	0.146223062909981	-2.99908600217406	0.00270790857890787	0.0425933558586699
**Gm28661**	56429.7419136051	-0.583531798675804	0.194574522953105	-2.9990144126754	0.00270854493687804	0.0425933558586699
**Ciapin1**	4934.19036557318	0.235386928222501	0.0785006012662451	2.99853662807187	0.0027127954572814	0.0426176650253071
**Natd1**	1939.95975396494	0.288820583413058	0.0963480122076449	2.99768076990115	0.00272042467779776	0.0426938896257804
**Treml4**	258.253854521229	0.717292844479115	0.239306216818309	2.99738491551063	0.00272306651481306	0.0426938896257804

**Sp110**	17731.1277699306	-0.195809131955835	0.0653722440170647	-2.99529463765571	0.0027417985721546	0.0429022046687587
**Selenot**	1932.73004694904	-0.376261695129697	0.125613652752945	-2.99538853367893	0.0027409546026551	0.0429022046687587
**Gm26648**	314.845173898386	-0.540447883330885	0.180530649769398	-2.99366276043003	0.00275650440923365	0.0430895237860066
**Fam168b**	8361.4888681126	1.08484206835579	0.362478020272126	2.99284924239367	0.00276386239498049	0.0431600839260826
**Malat1**	132019.391014447	-0.274549255814349	0.0917439897606875	-2.99255849380985	0.00276649646286371	0.0431600839260826
**2510017J16Rik**	75.5018863550272	-0.886459993378147	0.296288911391436	-2.99187704735605	0.00277267908753195	0.0432137530981611
**Dstyk**	984.033056919264	-0.488408459509679	0.163307554189716	-2.99072790559516	0.00278313360814725	0.0433338302999765
**Cdc16**	5629.97280403842	0.300032912627299	0.100339546873274	2.9901760768985	0.00278816675224585	0.0433693420682506
**Lysmd1**	1871.45301119923	-0.238351669311926	0.0797601074482332	-2.98835692349868	0.00280481789992794	0.0435853211530222
**Prpf39**	7095.60157192473	-0.208516703717557	0.069791018946914	-2.98772975182041	0.00281057957126684	0.0436318249304942
**Ralgapa1**	3582.40079220908	0.552941312050886	0.185132697964436	2.98672961681305	0.00281978992872425	0.0437317223493188
**Gm10925**	178221.176584667	-0.505834582847053	0.169403246025474	-2.98597928147721	0.00282671793866548	0.0437960615138171
**Ctla4**	459.089350519429	-0.662680857441122	0.222043358863093	-2.98446601075656	0.00284073760253562	0.0439700416533928
**Spen**	2323.16614127582	1.17724146118475	0.394560883872027	2.98367504054604	0.00284809076662478	0.0440405949064835
**C030034I22Rik**	343.742362947471	-0.555546490351918	0.186223809164706	-2.98321945428881	0.00285233395534668	0.0440629667984291
**Tspan33**	39.553796856015	3.79777065235	1.27402466877917	2.98092395337149	0.00287380147158895	0.0443406869899739
**Ppp1r9b**	1562.93875158315	-0.316803246134591	0.106284984433713	-2.98069617098332	0.00287593971592012	0.0443406869899739
**Tuba1b**	42619.2703482905	0.193546044667084	0.064953716675774	2.9797531930805	0.00288480712106966	0.0443675074929911
**4930581F22Rik**	970.987259237581	-0.428882822794587	0.143924097049558	-2.97992366522827	0.00288320222010221	0.0443675074929911
**D730003K21Rik**	21.0680458569964	1.78934426482557	0.600529060700448	2.97961311437369	0.00288612649491121	0.0443675074929911
**Tacc3**	1086.15224433464	0.381382852175865	0.128013744261282	2.97923363133138	0.00288970353901067	0.0443791994777691
**Card6**	4007.68331468889	-0.555936679337872	0.186626019627064	-2.97888086799903	0.0028930323494851	0.0443870601079228
**Ubxn11**	5999.0522803865	-0.231217154596729	0.0776778213805639	-2.97661739847125	0.00291447473255842	0.0446725470436994
**Arl11**	18.6048153645046	2.2356965331367	0.75138455583801	2.97543583477472	0.00292572549500867	0.0447579190532539
**Sort1**	25.7463475056313	2.15278690465882	0.723497276920506	2.97552869006217	0.0029248399008243	0.0447579190532539
**Bbx**	6449.19468656673	-0.268052214187652	0.0901022023620897	-2.97497960272316	0.00293008029984536	0.0447810623517588
**Armc10**	4128.89530716637	0.162719611209552	0.0547180651475013	2.97378225584029	0.00294153730809513	0.0449126001585804
**Rbbp7**	25866.7795685075	0.163013346529881	0.0548231098373773	2.97344216724352	0.00294479894783051	0.0449188741732481
**Egr3**	134.908287647196	-0.757078333814533	0.25476307145424	-2.97169573868368	0.00296160022174525	0.0451314648878528
**Zfp871**	7020.30858075413	-0.391790257858351	0.131866861911057	-2.97110473533988	0.00296730566543458	0.0451747201644953
**Tdrp**	12979.2749189269	-0.511643794497018	0.172263567327822	-2.97012190350933	0.00297681596373647	0.0452757617187216
**Cd200r1**	433.166407677571	0.554632813189362	0.186829120093413	2.96866362648418	0.00299097812264122	0.0452774198788374
**Gm8399**	464.163996676543	0.377527631565247	0.127206212664312	2.96783957055238	0.00299900815452317	0.0452774198788374
**Rnasel**	4032.03137877586	-0.280840235857145	0.0946105624002064	-2.96838142309291	0.00299372584885913	0.0452774198788374
**Gm20634**	220.011841272985	1.23359706404014	0.415642404287505	2.96792880446058	0.00299813766409979	0.0452774198788374
**Gm16531**	55.7486850362892	-1.02609232350014	0.345529253726658	-2.96962503878721	0.00298163441378977	0.0452774198788374
**Gm38102**	82.3216335371491	-1.01580220333014	0.342227734624518	-2.96820538067919	0.00299544108497951	0.0452774198788374
**Gm37174**	88.8344977000095	-0.856001689332109	0.288434873412998	-2.96774685807993	0.00299991282309489	0.0452774198788374
**Gm17949**	71.9168630976218	-1.34995377490621	0.454670272856584	-2.96908299375892	0.0029868991244149	0.0452774198788374
**Gm37593**	653.774143442603	-0.616105682548175	0.207621908082222	-2.96744061471676	0.00300290285027014	0.0452791772360828
**Hsd3b7**	210.560061266721	0.687694301333041	0.231879720747254	2.96573714646922	0.00301958445158932	0.0454871818390946
**Ess2**	5225.07316975904	0.210855929053528	0.0711678713292711	2.96279662599383	0.00304857918605918	0.0458363189262733
**4930481B07Rik**	150.495298338156	-0.566585877877457	0.191229130645857	-2.96286384801244	0.00304791352323464	0.0458363189262733
**Samd9l**	9156.1965691772	-0.298546148325686	0.100792862747862	-2.96197707046493	0.00305670546481423	0.045914688283201
**Acadvl**	11420.5084112923	0.288001159337044	0.0972618243281653	2.96109147989366	0.00306550871510374	0.0460030674513234
**Nudt9**	3865.28537234738	0.210462786816766	0.0711086358414324	2.95973596352044	0.00307902806909686	0.0461619840958699
**Spo11**	537.393309458757	-0.773775486514055	0.261486595011397	-2.95914016732035	0.00308498747379367	0.0461712903847151
**Map3k10**	339.824698238352	1.36457359836334	0.461146668448472	2.95908805533486	0.00308550921971854	0.0461712903847151
**Fdxr**	2063.44526815333	0.292576890732317	0.098928524093782	2.95745735026796	0.00310187657092977	0.0463721718483305
**Ppp4r3b**	17286.7714438323	-0.197178051528534	0.0666964962178431	-2.9563479749298	0.00311305655165704	0.0464500180625308
**Grap2**	19639.278283707	-0.272066957255806	0.0920313449467852	-2.95624232605882	0.00311412316571597	0.0464500180625308
**Timm10b**	2979.38571113313	0.28346082206062	0.0958913110050327	2.95606368386957	0.00311592746665577	0.0464500180625308
**Ubp1**	3711.48541510441	0.703115872326389	0.237953707193792	2.95484311052891	0.0031282808837757	0.0465900963002398
**Vps13a**	5056.32193283817	-0.428594691745647	0.145106382919558	-2.95365843405555	0.00314031367210539	0.0467251393119591
**Cd55os**	182.997299749095	-1.03245442174794	0.349717660609489	-2.95225131023859	0.00315466067552841	0.0468943285512276
**Trpm7**	6951.36457127215	-0.348658254783646	0.118189487652153	-2.94999379140886	0.00317780315242429	0.0471938211807252

**Gm8995**	37357.7043704005	-0.448528904669801	0.152076476948235	-2.9493641204121	0.00318428563310826	0.0472455637673134
**Ryr1**	78.5525540724468	1.89244239054318	0.641799668133518	2.94864968697597	0.00319165533888448	0.0473103604654777
**2700062C07Rik**	905.347812395788	0.312648841327873	0.106042155450695	2.94834483511834	0.00319480475342008	0.0473120978895854
**Swsap1**	1474.03502618165	0.38217843437305	0.129637382941423	2.94805730956277	0.00319777776559043	0.0473120978895854
**Commd9**	1764.67660511213	0.297930782180077	0.101077798927922	2.94753927509373	0.00320314060261018	0.0473455377447805
**Ninj2**	139.483302871065	-0.574235099869054	0.194837011395368	-2.94725881780131	0.00320604739313834	0.0473455377447805
**Ric8a**	12364.877794098	0.145229044643591	0.0493088888515683	2.94529136685205	0.00322650665076587	0.0476030573933688
**Gm48855**	117.225795772099	-0.694494282440631	0.23588149558113	-2.94425080157152	0.00323737538238232	0.0477187314314295
**Gm13231**	45.5806272001889	1.44744698420243	0.492027855127164	2.94179886183139	0.00326311799526171	0.0480532245339614
**Apbb1ip**	5241.68655193737	-0.556376237436036	0.189181382274162	-2.94096718581818	0.00327189192486376	0.0480925383023118
**Gm37186**	21.3494280809463	-1.5974287127199	0.543144797318031	-2.94107339443877	0.00327077026034877	0.0480925383023118
**Zfp97**	1029.66658264659	-0.373098192238509	0.126877996808464	-2.94060594920757	0.0032757095519416	0.0481037795060054
**Cog8**	10458.9371772876	0.193169579630592	0.0657549886097664	2.93771748295766	0.00330638174366466	0.0485019489287419
**Jund**	12132.6324369831	2.65110026665481	0.902510225449102	2.9374739386865	0.00330897982473805	0.0485019489287419
**Rnf126**	2014.80699334697	1.13459387064646	0.386511636754053	2.93547143929443	0.00333041268712621	0.0486802529787085
**Ago1**	1183.62336337619	-0.388791512270213	0.132431929993199	-2.93578378182799	0.00332706136680484	0.0486802529787085
**Gm49435**	20.5918010319556	-2.38349080517935	0.811912250126162	-2.93565074896823	0.00332848838470938	0.0486802529787085
**Tfrc**	1600.41630410081	0.315396636866631	0.107547408561977	2.93262888510118	0.00336105385078618	0.0490826001175513
**Nfatc3**	11036.1016763987	-0.313346092845594	0.10688249355602	-2.93168771068537	0.00337125562104256	0.0491859952044145
**Usp32**	826.359749468844	-0.424647994773063	0.144903591761223	-2.93055534104919	0.00338356719869783	0.0493199522200571
**mt-Co2**	57095.9548861064	-0.572292227397667	0.195331583740099	-2.92984993230351	0.00339125736550372	0.0493741301904743
**2410022M11Rik**	319.1964849054	-0.71172464628744	0.24294495547965	-2.92957161790939	0.00339429584567803	0.0493741301904743
**Tigd5**	81.8437707196886	2.74431492229267	0.936833127940746	2.92935298768197	0.00339668446572788	0.0493741301904743
**Mllt3**	3787.37780079938	-0.356721831878945	0.121813625795262	-2.92842306884867	0.00340686129692807	0.0494308595356313
**Ppp1r15a**	9724.28971144724	1.07273218605159	0.366297203182215	2.92858415716038	0.00340509639577587	0.0494308595356313
**Zfp85os**	103.753277252646	-0.850369923622971	0.290435289036212	-2.92791529033837	0.00341243002517365	0.0494661084697895
**Tmem79**	46.415781281886	-1.12593830615142	0.384658021993286	-2.92711510426025	0.00342122236204815	0.0495024800356223
**Gadd45b**	690.901373552572	0.732534501668576	0.250256499003385	2.92713477806092	0.00342100594192106	0.0495024800356223
**Prmt6**	2020.7170883817	-0.252282069722439	0.0861977336841956	-2.92678309439932	0.00342487649132596	0.0495098888750672
**Trappc13**	2225.18163965678	-0.259118024315579	0.0885456535572414	-2.92637768095607	0.00342934332456686	0.0495285381763038
**F2r**	6885.42561745656	0.289576921746094	0.0989635848148692	2.9260957178118	0.00343245311217388	0.0495285381763038
**Icam1**	15859.3897010569	0.475110987609358	0.162528971086744	2.92323875818905	0.00346410776856629	0.0499395664311976
**Inpp5a**	509.620934824982	0.743964553682945	0.254568046184221	2.92245851289823	0.00347279883030519	0.0499734165928026
**Mst1**	440.38200731765	-0.540015522198704	0.184779141742373	-2.92249177643447	0.003472427907032	0.0499734165928026
**Ptgis**	49.2656832053959	1.18207275617571	0.404656654584802	2.92117463727015	0.00348714297369096	0.0501340436463946
**Rgs2**	1008.20607386794	0.409160228681892	0.140146586245671	2.91951619830861	0.00350575174201325	0.0503556337273499
**Scpep1**	994.555552747985	0.394751782140172	0.135271559144104	2.91821713771811	0.0035203911127161	0.0504653164236945
**Cflar**	8780.52969286142	-0.30119459095932	0.103200731974021	-2.91853153750052	0.00351684298642229	0.0504653164236945
**Mars1**	9680.37139759744	0.355751557568686	0.121916793446315	2.91798650138661	0.00352299600596966	0.0504653164236945
**Bccip**	6496.92143739595	0.240989232152716	0.0826059078030577	2.91733652667147	0.00353034650265167	0.050485134470688
**Phf21a**	2427.54302539899	-0.386134591628356	0.132363666345984	-2.91722496277069	0.00353160956942061	0.050485134470688
**Gm21885**	22.6211224844156	-1.66726297909059	0.571564823917569	-2.91701467501619	0.00353399145276187	0.050485134470688
**Cdc73**	1051.75184825528	0.672818338944886	0.230811748675988	2.91500906173275	0.00355678215067977	0.050764688721251
**Fuca1**	6932.37062251021	0.163620206962203	0.0561435797895211	2.91431731955115	0.00356467369112178	0.0507895467717448
**Usp39**	6780.97697951214	0.218079182234033	0.074830947551283	2.91429133761241	0.00356497040867866	0.0507895467717448
**Rcan3**	1584.1123115168	-0.394530126125757	0.135425191261292	-2.9132698462618	0.00357665381124882	0.0509099675734848
**Mcm5**	6098.95470580173	0.224771174113481	0.0771770317835732	2.91241019405624	0.00358651314190567	0.0510042306651694
**Trim30d**	2228.58143596377	-0.298670707662615	0.102562323689945	-2.91208990706494	0.00359019282245996	0.0510105214639328
**Gm25821**	80.2988788405497	-0.741754744111294	0.254765183403283	-2.91152320816587	0.00359671186915875	0.0510571071372382
**Gm47405**	77.5371504370128	-0.971458749989052	0.333757813724233	-2.91066968335225	0.00360655075257416	0.0511506932568057
**Hdgf**	5061.82803868461	0.553275364678002	0.190344189044618	2.90671003646089	0.00365251598537361	0.0516167662614636
**Dnajb12**	1557.14496904153	0.284223673271451	0.0977625852359638	2.90728475096518	0.00364581157731287	0.0516167662614636
**Slk**	1691.74362714664	-0.403357781873019	0.138741940779152	-2.90725197880199	0.00364619358423366	0.0516167662614636
**Pptc7**	3664.40637242426	1.26619341968096	0.435603659682716	2.90675569760645	0.00365198290954771	0.0516167662614636
**Dstn**	1408.19514367093	0.237317162738124	0.0816928404490571	2.90499340497425	0.00367260838592435	0.0518542028109409
**Ppm1a**	1907.60980135814	0.743378492914457	0.255952271636785	2.9043637243797	0.00368000367130331	0.0519121019236582

**Gps1**	9656.6413964518	0.167625595279493	0.0577234522993071	2.90394265419751	0.00368495648259049	0.0519354734312866
**Trim56**	6287.58661166016	-0.418530408936327	0.144256386937658	-2.90129551849376	0.00371623230972063	0.0523294660449222
**Dag1**	3798.02058916079	-0.317324340631906	0.109397776369254	-2.90064708043819	0.00372393029018228	0.0523595592649183
**Tnrc6b**	7514.75009587022	-0.437126403789527	0.150704360268229	-2.90055578359853	0.00372501529072624	0.0523595592649183
**Scarna2**	136.993586203052	0.774339216592876	0.266997332941529	2.90017584843236	0.00372953364749669	0.0523763473115912
**Grina**	6160.52474360986	-0.18210261594086	0.0628043286137822	-2.89952332841112	0.00373730533081103	0.0524149329974477
**Trac**	46164.1576638874	0.179893962459161	0.0620455217447588	2.89938673091031	0.00373893410478716	0.0524149329974477
**Cd68**	598.328502324957	0.562122397243861	0.193914463338921	2.89881624900455	0.0037457434466693	0.0524171221040569
**Pfkp**	25464.120795128	0.242831607249472	0.0837632976094225	2.89902157842167	0.00374329131227061	0.0524171221040569
**Ptov1**	777.415023715885	1.51725520770472	0.523582382860438	2.89783471975441	0.003757485485357	0.0525347815375069
**Tsc22d2**	1160.28488391449	1.2183812203689	0.420543101605907	2.89716135092057	0.00376556033766206	0.0525812903619212
**Mbtd1**	1331.02055864316	-0.436373067882396	0.15062924504461	-2.89700096254986	0.00376748599470769	0.0525812903619212
**mt-Co3**	108953.132305912	-0.597477285093303	0.206261426568839	-2.8966990824816	0.00377111285906833	0.0525853321418936
**Pard6b**	149.284890675818	2.94817974255169	1.01808010982122	2.89582294567114	0.00378165696693557	0.052685737248456
**Sigmar1**	3633.11187352164	0.21840252629634	0.0754356841464327	2.8952150267821	0.00378898887014023	0.052715151280116
**Gm47015**	2602.57954817654	-0.473979396502734	0.163718180103122	-2.89509324012878	0.00379045924988078	0.052715151280116
**Dcaf15**	1573.09576062909	-0.220903388878198	0.0763751961416698	-2.89234463592656	0.00382378248221018	0.0531316936262661
**Gm28437**	108291.150515483	-0.597664418797721	0.206763903435161	-2.89056459501957	0.00384550493349068	0.0533864504290861
**Slc44a1**	499.001075380347	1.11783832433344	0.386959215395052	2.88877556047405	0.00386745004581877	0.0535495697468948
**Creb3**	7069.25901627438	0.186592227444288	0.0645823368713097	2.88921455128051	0.00386205467699301	0.0535495697468948
**Kcna3**	5876.46237708596	-0.35918879891036	0.124327992096504	-2.88904206408767	0.00386417379603612	0.0535495697468948
**Klhdc4**	4034.53331997045	0.214689173054818	0.0743422133666796	2.88785016388875	0.00387884596928676	0.0536602071449092
**Med13**	1527.08766691613	-0.555035388184884	0.192217449832287	-2.88753902764375	0.00388268434597894	0.053666190561044
**Mthfd2**	2927.86085003781	0.385176681682136	0.1334804559276	2.8856410401464	0.00390617395438614	0.0538491539801071
**9930111J21Rik2**	20080.4262342307	-0.375982256308156	0.130271134147677	-2.88615170788286	0.00389984124009173	0.0538491539801071
**Gm12490**	166.233769892513	-0.980189576950935	0.339662621046647	-2.8857740481733	0.00390452364278949	0.0538491539801071
**Arfgap2**	13803.2297664058	0.178981667864486	0.0620360285119195	2.88512453420671	0.00391258856696281	0.0538904353580708
**Nek7**	7143.00346306388	-0.187455406246614	0.0649821384818068	-2.88472202710129	0.00391759403178026	0.0539122525404032
**A630081D01Rik**	259.643580829939	-0.777514407555549	0.26955520913806	-2.8844347324682	0.00392117030315873	0.0539143808611449
**Itga7**	74.7364492403315	-0.821696296699275	0.284917844271552	-2.88397625217228	0.00392688365215624	0.0539458637375988
**Mrtfa**	11125.4846874009	-0.317732704184187	0.110258990050524	-2.8816943093583	0.00395543268546812	0.0542907254572484
**Nin**	4519.61264552	-0.359970125410766	0.124968824829781	-2.88047939877069	0.00397070900870063	0.0544354361562709
**Gm26528**	82.0758396080989	-0.796642745811738	0.276582612640746	-2.88030667656792	0.00397288516720896	0.0544354361562709
**Twsg1**	2370.0386206284	-0.23181366137516	0.0805131211682108	-2.87920351380798	0.0039868096811893	0.0545313890160589
**Gm50335**	450.322662552345	-0.337934243605552	0.117362282558441	-2.87941096780627	0.00398418774463652	0.0545313890160589
**Ptprk**	243.16130278339	-0.418786793618299	0.14553931142237	-2.87748230718872	0.00400862388782479	0.0546646942525487
**Ift80**	4957.38239133148	-0.452694084026765	0.157310035770049	-2.87771903305966	0.00400561726138829	0.0546646942525487
**Amigo2**	1051.74183146362	0.677757323501592	0.235549962518466	2.8773399760081	0.00401043260493408	0.0546646942525487
**Gpn1**	5568.01846111308	0.207328164206436	0.0720527050918426	2.87745149806886	0.00400901534134433	0.0546646942525487
**Dcaf12**	6845.5240258384	-0.208553876793304	0.0725055497137088	-2.87638501627513	0.00402258722303463	0.05478297914724
**Gm43149**	33.099827642835	-1.72299285971166	0.599102926880194	-2.87595466889818	0.00402807555818203	0.0548103510969553
**Tpst2**	34932.7863539932	0.233667347376767	0.0812626926734693	2.87545661716739	0.00403443583053056	0.0548495300963503
**Zbtb1**	4909.54243569757	-0.235538615202938	0.0819326693607197	-2.87478263604409	0.00404305729062456	0.0548720531682784
**Rcsd1**	24233.9119861345	-0.160172499385001	0.055712446205196	-2.87498593752401	0.00404045493035617	0.0548720531682784
**Gbp9**	14733.5464224163	-0.248723098407533	0.0865612337989262	-2.8733774634647	0.00406108584012954	0.0550693025670579
**Syt11**	5203.03438130456	0.478338237577087	0.166510545025732	2.8727203883884	0.0040695412122715	0.0551055014661646
**Gm45153**	124.976968151823	1.85729662248143	0.646549969150177	2.87262657350777	0.00407074974339123	0.0551055014661646
**Fam20b**	1842.22870821142	0.376638783206105	0.131149786013669	2.87182156108781	0.00408113337832311	0.0551798390493166
**Zc3h12b**	261.021586917919	-0.549534040724702	0.191382776350918	-2.87138712899158	0.00408674698042473	0.0551798390493166
**Usf3**	830.729594435013	-0.371475221694592	0.129367225296194	-2.87147862098827	0.00408556416591923	0.0551798390493166
**Sdf2l1**	1618.47366813337	0.365017145801774	0.127145236154676	2.87086765372568	0.00409346868431934	0.0552232757352909
**Mxi1**	1336.30027942628	1.34636625146801	0.4691598853495	2.8697386403039	0.0041081120650517	0.0552414556775508
**mt-Ts1**	3435.08323965489	-0.502769614150413	0.175196213466669	-2.86975160137274	0.00410794368972804	0.0552414556775508
**Hnrnpr**	9436.21232386184	-0.179107849632665	0.062413817870902	-2.86968263987849	0.00410883963026525	0.0552414556775508
**Rc3h2**	2164.62033682023	-0.521991395945184	0.181876921867876	-2.87002545778943	0.0041043875254066	0.0552414556775508
**Srp14**	5448.10672686692	0.192224715847793	0.0670097923436839	2.86860635027638	0.00412284567188863	0.0553825057561373

**C430039J01Rik**	22.0688342667107	-1.52983904017973	0.53335539213706	-2.86832956548904	0.0041264545419498	0.0553837684987249
**Ifi203**	15574.038550996	-0.283343482681129	0.0988043722783715	-2.8677221073055	0.0041343849619563	0.0554429819962088
**Taf2**	4453.83338424087	-0.22634187463736	0.0790050602474804	-2.86490351286807	0.0041713632187806	0.0558913012230663
**Kctd5**	3943.40351489475	0.356811194900633	0.124620531181924	2.86318146389338	0.00419410286600751	0.0561162790211561
**Atmin**	1875.8138893315	0.811493622397619	0.283432526013748	2.86309279252677	0.00419527680947655	0.0561162790211561
**Ccdc15**	718.45453151508	-0.318253621651079	0.111197023433748	-2.86206961142892	0.00420884455290402	0.056250011552255
**Gm47834**	129.273619853348	-1.03303419622939	0.361057930443765	-2.86113143937793	0.00422132001250738	0.056368931726338
**Maf1**	22999.6119067195	-0.144817895497642	0.050627362494579	-2.86046691674188	0.00423017686766552	0.0563916217460284
**Adam22**	180.3625151254	-0.576587350797083	0.20156459118199	-2.8605587291693	0.00422895217551328	0.0563916217460284
**Matk**	3621.71314222678	-0.204867768074705	0.0716505136032947	-2.85926447378997	0.00424624605715093	0.0565579874239453
**Eral1**	3402.43571127657	0.174828541617539	0.0611511902726066	2.85895566117631	0.0042503818876376	0.0565652596313393
**Taf5l**	9042.43372317524	0.284813460933805	0.0996625198714396	2.85777904573558	0.00426617342873115	0.0567275060898875
**Gm26532**	98.8483688360852	-0.837857621944435	0.293304020703284	-2.85661826229119	0.00428180460524677	0.0568873483683587
**Psmd10**	3466.59910963577	0.242733409288539	0.0849907409031328	2.85599827356714	0.00429017466975997	0.0569304868811764
**Ufc1**	1935.03478245938	0.272168909705277	0.0953114035534929	2.85557550888991	0.00429589064553651	0.0569304868811764
**Ipcef1**	9980.51406789691	-0.392013378070582	0.137271032006249	-2.85576186279955	0.0042933702031361	0.0569304868811764
**Gm42722**	692.961689929125	-0.829870367067769	0.290693889681435	-2.85479123065574	0.00430651274452392	0.0570232952230785
**Ccdc130**	2503.06020613186	0.321456660324374	0.112680526771589	2.85281467467744	0.00433338852073353	0.0573309848204854
**Lpar6**	4494.7968584327	-0.380617293851916	0.133444808542275	-2.85224504429737	0.00434116211887667	0.0573856472375333
**Lrrk1**	1201.188677544	0.566007094214088	0.198625727363671	2.84961621903977	0.00437720106973232	0.0575242345752896
**Vps13d**	2438.82073315459	-0.406809918572376	0.142715619758811	-2.85049330451624	0.00436514694075044	0.0575242345752896
**Hdgfl3**	3557.7984357052	0.424604059512406	0.148984577826854	2.84998666107488	0.00437210626395214	0.0575242345752896
**Mrc1**	141.294807554062	0.851145695507414	0.298656955335265	2.84991084353598	0.00437314856868802	0.0575242345752896
**Klf2**	12263.7342331166	1.89848838832138	0.666045737878604	2.85038741388568	0.00436660063824106	0.0575242345752896
**6720427I07Rik**	145.084397649283	-0.911164816866407	0.319729950163433	-2.84979501107312	0.00437474141517526	0.0575242345752896
**Gm37893**	111.161597000082	-0.890529691532568	0.312478213873669	-2.84989369496524	0.00437338435060229	0.0575242345752896
**Rnf139**	3887.78934421701	0.528394531513536	0.185447704968752	2.84929129536853	0.0043816742768102	0.0575350346497486
**Angptl4**	603.219865780833	0.497217184893807	0.174534332303566	2.84882165205755	0.00438814715467735	0.0575720522200258
**Mgat1**	10274.3069651018	0.246559386948892	0.0865688678535621	2.84812996937844	0.00439769608233746	0.05764933208768
**Fuom**	66.9388411941116	0.826438133620698	0.290358053203759	2.84627247118489	0.00442343278465062	0.0579385123755111
**Thra**	440.791374632168	-0.521727883779445	0.183333165404852	-2.8457910636481	0.00443012519758275	0.0579779756962719
**Vps11**	9760.912874052	0.18772538379475	0.0659794762199425	2.84520876111486	0.00443823249037368	0.058035874979932
**Tradd**	5183.19773435361	0.195089959302062	0.0685988045668459	2.84392651641556	0.0044561323720001	0.0582216233711489
**Gm28438**	394.703849271978	-0.923511113188634	0.32487490480218	-2.84266682202176	0.00447378113272599	0.0583554381691751
**Gm4544**	66.3582139470493	-0.950970681336153	0.33453302768887	-2.84268099896132	0.00447358215694458	0.0583554381691751
**9530046B11Rik**	21.8914858329032	2.12520541451202	0.747691049463779	2.84235770380848	0.00447812165406077	0.0583637410281518
**Gpatch2l**	3569.34110452864	0.200189990167121	0.0704766334296721	2.84051579119324	0.00450406429615648	0.0584600832903934
**Emilin1**	1918.94656735755	-0.44529644171633	0.156753474712053	-2.84074367432246	0.00450084728431318	0.0584600832903934
**Pid1**	314.475144118543	0.656028251268584	0.230910876941316	2.84104525502844	0.00449659308888186	0.0584600832903934
**Gm20342**	337.702640578416	-0.606406747780062	0.213448089042861	-2.8410034050869	0.00449718321997555	0.0584600832903934
**Gm47087**	652.184693880646	-0.609122199632392	0.21436162990746	-2.84156357597836	0.00448928999571004	0.0584600832903934
**Pde3b**	7108.85028698651	0.700105899938098	0.246516933820637	2.83999110765951	0.00451147914201717	0.0584655343376586
**Gm37558**	256.724876510494	-0.780092647296403	0.274684283117703	-2.83996098517996	0.00451190516942266	0.0584655343376586
**Tmem222**	4692.35256783108	0.226109240249368	0.0796431332745192	2.83902994461556	0.00452509102228461	0.0585882163008534
**Otub1**	7169.77348402212	0.176653769335203	0.0622418145852729	2.83818475589562	0.00453709121791989	0.0585991363284949
**5830411N06Rik**	966.805202824427	0.688870874873034	0.242684579996565	2.83854406770625	0.00453198610289566	0.0585991363284949
**Gm19325**	54.9532705297772	-1.3877156702099	0.488928237055586	-2.83828088671453	0.00453572487764	0.0585991363284949
**Prorp**	1137.84098544547	-0.31013205782557	0.109316883245366	-2.8370005493979	0.00455395336404358	0.0587687495145248
**Zfp260**	5873.10407548455	-0.200570668677209	0.0707049842793755	-2.83672602039902	0.00455787053194592	0.0587711669164254
**Gm10825**	225.189033444642	-0.566262133703174	0.199638632074509	-2.83643565285418	0.00456201701603324	0.0587765348500701
**Khsrp**	493.961507585803	0.755949107295261	0.266548087843971	2.83607026938332	0.00456723959149652	0.0587957469307277
**Ticrr**	136.616477146696	0.843457288838409	0.297564787559279	2.83453326503016	0.00458926797674822	0.0590310983752014
**Srf**	1050.73097250922	1.42184743474304	0.501740979484629	2.83382759806367	0.00459941380733368	0.0590776430087863
**Dcps**	5030.36528070566	0.151940320856552	0.0536179191640741	2.833760116494	0.00460038509689539	0.0590776430087863
**Wasl**	1144.76815016209	-0.383389888736483	0.135407601703281	-2.83137640659649	0.00463481419787654	0.0594029120800278
**Tmem238**	276.744464003965	1.53775644652259	0.543141926064712	2.83122398166585	0.00463702366303447	0.0594029120800278

**Traj43**	23.5582462919751	-2.03689118437041	0.719360635536348	-2.83152994999751	0.00463258948386404	0.0594029120800278
**Nkiras2**	3016.95079308321	0.187059020644338	0.0660829234299244	2.8306710861952	0.00464504612800901	0.0594259985958274
**Larp4b**	6307.62178940019	-0.202667204095849	0.0715991783552844	-2.83058002551632	0.0046463686152224	0.0594259985958274
**Acvrl1**	824.454649337191	0.428085940272908	0.15129746320427	2.82943237253714	0.00466306539272653	0.0595428860560713
**Hdac1-ps**	1988.56847659034	0.195239805134513	0.0689999161151249	2.82956583322158	0.00466112093525763	0.0595428860560713
**Galnt12**	4979.58905601945	0.215726243588535	0.0762543070216102	2.82903683758346	0.00466883245971003	0.0595682534960736
**Polr3c**	8901.71335432995	0.16347382479964	0.0578055062425742	2.82799745950915	0.0046840178247915	0.0596029005698815
**Tspan5**	1241.04688083132	0.836307020749109	0.295726411930992	2.82797540905566	0.00468434046682668	0.0596029005698815
**Cmtm6**	28938.9388283087	0.120673557876215	0.0426719517316477	2.82793621991088	0.00468491393169253	0.0596029005698815
**Ighv1-64**	18.2930956174979	2.62948284285662	0.929863598210032	2.82781565803664	0.0046866785432559	0.0596029005698815
**Slamf6**	23707.3969962132	-0.196765045866659	0.0696303529489979	-2.82585162265058	0.00471551014481684	0.0599212043160314
**Optn**	120.742170182146	1.33002730288064	0.470771487081127	2.82520785429691	0.00472499540067998	0.0599933541728562
**Maml3**	474.038110472375	-0.716576401396686	0.253761916835386	-2.82381379496563	0.0047455946300416	0.0602063885552057
**AA986860**	32.1147374140813	1.30577428135147	0.462694146595455	2.82211108776601	0.00477086482787921	0.0604682359289085
**Trmt61a**	2451.28022242125	0.256517688577491	0.0909022630869694	2.82190651658553	0.00477390908774273	0.0604682359289085
**Batf3**	44.4501959424944	1.68079015973569	0.595717643042413	2.82145439096223	0.00478064348615936	0.0604962368448502
**Skap2**	1766.23946887797	0.642864034985123	0.227865549042939	2.8212427797235	0.00478379838222272	0.0604962368448502
**A130012E19Rik**	150.733119325815	-0.632572873001645	0.224342637274402	-2.81967298185908	0.00480726127574334	0.0607441988146654
**Slc38a1**	13277.6156571811	-0.179967998550587	0.0638610059288456	-2.81812032136025	0.00483057041131187	0.0609898220921804
**Pex6**	2182.0512509859	1.73348789400262	0.615446617072136	2.81663404415048	0.00485297872297571	0.0610984303017692
**Rab32**	1296.52360066136	0.393845338702474	0.139859427343385	2.81600851786343	0.00486243774820198	0.0610984303017692
**Pdia4**	9551.43793725964	0.178558727070866	0.0633828955762659	2.81714373329653	0.00484528366548038	0.0610984303017692
**Baz2b**	2273.18616563248	-0.363491552785839	0.129070038943539	-2.81623493539695	0.00485901200424863	0.0610984303017692
**Bdp1**	3583.63993765704	-0.285023852404833	0.101208806738273	-2.81619615516175	0.00485959860218753	0.0610984303017692
**Gm11725**	248.296764789179	-0.554249889764984	0.196813209177912	-2.81612139794928	0.00486072957619738	0.0610984303017692
**Zfp384**	4337.84963907833	0.503877763432499	0.178956064677232	2.81565067013124	0.00486785651412441	0.0611177809506441
**Slco4a1**	496.960989468406	1.01780364845204	0.361645640563531	2.8143672542715	0.00488733580386867	0.0613134954311773
**Vezf1**	6462.71236068943	-0.193012185806417	0.0686036413676072	-2.81343937375242	0.00490146277672781	0.0614418050699285
**Dync2h1**	696.481300214986	-0.364683665134605	0.129662680390746	-2.81255689019856	0.00491493284123294	0.0615616826544574
**Ube2m**	539.20399814344	0.980182925174082	0.348539146137387	2.81226064858641	0.00491946211906657	0.0615694714933535
**Gm47772**	123.579886316147	-0.745704488536879	0.265293845983893	-2.81086236950311	0.00494089160205449	0.0617885944234704
**Fahd2a**	704.901213222107	-0.384502874736299	0.136809594064902	-2.81049642288898	0.0049465138794452	0.0618098486902601
**Pnn**	7611.77951638897	-0.191222464320532	0.0680658309397887	-2.80937530153254	0.00496377443561426	0.0618129834751394
**Lpar2**	87.7356431631315	0.734785403254558	0.26156333609747	2.80920642096699	0.00496637920159462	0.0618129834751394
**Hexim2**	118.859252768681	-1.15285177346679	0.410325222358578	-2.80960494419552	0.00496023447447727	0.0618129834751394
**Etohd2**	487.678289676185	-0.454192852377644	0.161676823174379	-2.80926383547114	0.00496549351790141	0.0618129834751394
**Trav12d-1**	88.5308901284262	0.678959794523329	0.241676145378328	2.80937861475927	0.00496372334570346	0.0618129834751394
**Por**	9364.15706250996	0.158928494807677	0.0565823412994489	2.80880025742633	0.00497264881505012	0.0618421684125847
**Lman1l**	175.501048282304	-1.08299156411591	0.385702109547036	-2.80784454455737	0.00498742961558075	0.0619770729122286
**Mogs**	1734.43519376347	1.73024781759374	0.616434887701459	2.80686225279271	0.00500266286877419	0.0621173828394601
**Atp6ap1**	22428.7498050516	0.21249313303241	0.0758276671665881	2.80231663418548	0.00507370497094602	0.0629498970292885
**Ndor1**	10490.9248155858	-0.192618788988037	0.0687425402719445	-2.80203187467382	0.00507818559977297	0.0629559169910485
**Gm47508**	133.878610656737	-0.684665851178414	0.244551401378736	-2.79968075144282	0.00511531691330968	0.0633663904111797
**Mpv17l2**	3082.40487955353	0.328887346278102	0.117523875945485	2.79847259658676	0.00513449260677961	0.0635539670110183
**Pja1**	10993.1105529035	0.306187491420211	0.109449590356381	2.79752067068709	0.00514964719773706	0.0636915156159677
**P4hb**	16135.4018817768	0.148396372367343	0.0530545621884355	2.79705205822411	0.00515712231143311	0.0637243514294044
**Gm44148**	156.938333678688	-0.638855906530471	0.228420010654442	-2.79684737208486	0.00516039045655391	0.0637243514294044
**Brd8**	10551.8677650335	0.154896813278024	0.0554487339707946	2.79351397562314	0.00521387760042515	0.063742308438842
**Acpp**	1707.90215170002	-0.496999138294027	0.177921157734716	-2.79336726796182	0.00521624311436228	0.063742308438842
**Galnt9**	668.713146778272	-0.566579299479785	0.202769094761705	-2.79420934509586	0.00520267864379891	0.063742308438842
**Egr1**	5218.66167816305	-0.293548254758142	0.105015341508812	-2.79528924574805	0.00518532987583176	0.063742308438842
**Metap1d**	2510.51483212371	-0.301990437117282	0.108102192497167	-2.79356440550634	0.00521306469299384	0.063742308438842
**Shld1**	2173.8336776222	-0.231551977376499	0.0828786976417069	-2.79386602305845	0.00520820514146825	0.063742308438842
**Gm9967**	66.0163366595962	-1.2753687776403	0.456558338441231	-2.79344099156009	0.00521505427408575	0.063742308438842
**Zfp763**	1046.00112416085	0.349320993559388	0.125071158947114	2.79297798549302	0.0052225246045913	0.063742308438842
**Snora57**	27.7885405703919	-1.30774263131626	0.468168437249876	-2.79331652299807	0.0052170615516957	0.063742308438842

**Vmn2r84**	193.334916563229	-0.881542046059772	0.315616492881399	-2.79307978493707	0.0052208813017957	0.063742308438842
**Tmsb10**	73001.6029733441	-0.164508317002544	0.0588530353886145	-2.79523929252372	0.00518613122744145	0.063742308438842
**Gm9903**	59.3160140412601	-1.28589703550148	0.459836782967255	-2.79642056297409	0.00516721117486731	0.063742308438842
**Gm26670**	43.8213151082394	-1.32732626510556	0.474713892298501	-2.79605523798519	0.00517305580214524	0.063742308438842
**Pcdhgb6**	180.85127731382	-0.811137338077436	0.290380454457782	-2.79336066055839	0.00521634967493272	0.063742308438842
**Gm45342**	47.7344179184667	-1.27651080691257	0.456901070098454	-2.79384508037484	0.00520854243003963	0.063742308438842
**Rps6ka4**	3794.0527039679	0.182427850975531	0.0653446279101054	2.79178039281969	0.00524189192248562	0.0639291726181161
**Uso1**	2321.46725443392	-0.367887582453079	0.131814907230973	-2.79094064686057	0.00525551086271108	0.0639359875857946
**Zfp606**	1319.41531482478	-0.239212246701398	0.0857034329897615	-2.79116294827974	0.00525190248675331	0.0639359875857946
**Skap1**	41463.2876435131	-0.131209088824787	0.047015785363794	-2.7907454445253	0.00525868121540838	0.0639359875857946
**Gm24950**	39.5131506819062	1.24594181354174	0.446376135048845	2.79123751408754	0.00525069264285056	0.0639359875857946
**Gm37320**	30.8801340718889	-1.36867669806969	0.490643237787466	-2.78955581705697	0.00527803976693989	0.0641218755648973
**Arpc3**	11146.3553454813	0.158766406919493	0.0569324448235494	2.78868064442968	0.00529232233485031	0.06424585749633
**Snx3**	10915.2890712957	0.184203203176158	0.0660935261212746	2.78700825914727	0.00531971231591705	0.0645013504668224
**Fam199x**	853.557485215556	-0.391139157940732	0.140349392801045	-2.78689597535486	0.00532155585497678	0.0645013504668224
**Gm42728**	20.1805460461566	-2.23498014980175	0.802124864479625	-2.78632448484399	0.00533094785561802	0.0645655229523237
**Klhl5**	421.669801166034	0.995445972598883	0.357529543406752	2.78423417296844	0.00536542814933477	0.0649332191621105
**Gm16279**	392.367509371248	0.600910988453137	0.215876534370576	2.78358641528684	0.00537615389706448	0.0649632338619978
**9430060I03Rik**	167.108546781488	-0.794082028586048	0.285268491171815	-2.78363034530785	0.00537542588031481	0.0649632338619978
**Nxpe3**	749.757726174381	-0.309117035906939	0.111115523226328	-2.78194285489082	0.00540345535322783	0.0652431003837631
**Irf2bpl**	7739.34656704508	-0.314394529680965	0.113037933441077	-2.78131880254914	0.00541385433576904	0.0653186085518474
**Vcpip1**	8123.08145630646	-0.372581226968469	0.133981462149135	-2.78084162534178	0.00542181803053581	0.0653646417040189
**Pdlim2**	7116.07143951915	0.158796304122884	0.0571536093791284	2.77841252456181	0.00546252190898954	0.065805013547361
**Fndc3a**	2903.47584643109	-0.247402034214317	0.0890557780153099	-2.77805707532851	0.00546850116900041	0.0658267172803204
**Alox12**	395.152518698247	0.708774236173948	0.255237357285737	2.77692201373359	0.00548763440868095	0.0660066071584623
**Akap11**	3377.0506102139	-0.353563618372448	0.127356243991292	-2.77617812281448	0.00550020660923466	0.0661073650203742
**Gm42372**	211.243284961767	-0.538051113417574	0.193842433082002	-2.77571378393687	0.00550806738551453	0.0661513855133784
**Dido1**	5978.26882081402	-0.370189479163006	0.133387316565955	-2.77529744726486	0.00551512415400139	0.06618569024722
**Lcn4**	1607.488698705	-0.434774084486976	0.156790375591153	-2.77296411114349	0.00555482465115967	0.0666113942376887
**Zfp12**	3826.26774217046	-0.222002794691535	0.0800698509353318	-2.77261406257438	0.00556080274006084	0.0666323716921206
**Gm48382**	73.0611949689158	-1.22186088045054	0.440789265040479	-2.77198420505619	0.00557157399651089	0.066710707798649
**Crebzf**	1334.21374839435	1.57840471337421	0.569543488703063	2.7713506425444	0.00558242760144488	0.066789910186763
**Tial1**	632.636909928637	-0.372589212975382	0.134497135910564	-2.77023901254774	0.0056015171251146	0.066967454734773
**Gimap8**	6535.56125493536	-0.311513414428117	0.112496516921023	-2.76909386133983	0.00562124386149886	0.0671523423242134
**Znhit1**	1422.15494424196	0.274078382794304	0.0990230186827962	2.767824960702	0.00564317552696705	0.0673632702866816
**Cfap74**	470.11723927285	1.01957405190409	0.368691562936964	2.76538482134566	0.00568556794035069	0.0678179364391415
**Vac14**	8010.42809476816	0.206281360962508	0.0746269942905535	2.76416547287661	0.00570685906292351	0.0680202391741076
**Rab3ip**	7982.79666530576	-0.223095212359784	0.0807169702712095	-2.76391955260687	0.00571116179649326	0.0680202391741076
**Dnajb5**	751.073102112722	0.339346144864907	0.122794706167036	2.76352422231702	0.00571808480857812	0.0680512555353213
**Vamp1**	2951.62778567692	-0.357261455405642	0.129415919984474	-2.76056806186212	0.00577009336113157	0.0685666373237935
**Serpinb1a**	902.52337040393	2.11074839591497	0.764590359895405	2.76062648266154	0.005769061429951	0.0685666373237935
**Spag9**	1813.13287646704	-0.290812855439899	0.105358279526523	-2.76022783161231	0.0057761064156623	0.068586366836165
**Sult1a1**	84.4300301264928	1.21409155916785	0.440086040031048	2.75875953502683	0.00580212119388934	0.0687398674076047
**Lpp**	5217.08342994189	-0.300741018384983	0.108999805228019	-2.75909684201596	0.00579613557484273	0.0687398674076047
**Arhgap15**	34164.004997593	-0.183061898557408	0.0663521053240043	-2.75894634636682	0.00579880547946411	0.0687398674076047
**Pdia6**	11511.6481176449	0.203937160420655	0.0739323607990309	2.75842889658311	0.00580799388580268	0.0687577457990931
**Cep350**	5672.34377420133	-0.378733026543471	0.137324326893186	-2.75794562487139	0.00581658724118497	0.0688077816511649
**Ireb2**	1135.82389849755	-0.374544227830011	0.135843607241598	-2.75717227652739	0.0058303624803176	0.0689173893023294
**Syngr2**	6920.35796067957	0.251888888296639	0.0913655622361842	2.75693469324356	0.00583460032552563	0.0689173893023294
**Zfp41**	370.122324880053	0.520332158005948	0.188767024047813	2.7564780481688	0.00584275344119593	0.0689619969834638
**Sgpp2**	108.851436072442	-1.10121465722996	0.399586510797117	-2.75588546528561	0.00585334895363085	0.0690353439089531
**Nipa1**	123.189502342757	1.05595410543518	0.383206119251645	2.75557735742146	0.0058588648313849	0.0690487158923949
**Gemin6**	1634.75359940571	0.277399778243583	0.100681336141822	2.75522543575338	0.00586517081441581	0.0690713725880044
**Gga2**	2015.60965957599	0.261228894379831	0.094868621133127	2.75358586706193	0.00589463049686947	0.0693306846901285
**A930004J17Rik**	273.876436509265	-0.484207784407746	0.175856998359178	-2.75341777083434	0.00589765836601655	0.0693306846901285
**Gm47992**	359.203093019401	-0.583979096695435	0.212104118955059	-2.75326617687783	0.00590039018654962	0.0693306846901285

**Nkapd1**	1522.59104582806	-0.283159105329248	0.10287480527369	-2.752463098967	0.00591488120007893	0.0694491677121041
**Xylt1**	223.202598194337	0.880585438565109	0.319984760001545	2.75196055762423	0.00592396553645759	0.0695040394325855
**Tnfrsf13b**	2530.31087131274	0.289132818698506	0.105125201744219	2.75036636221634	0.00595286675021735	0.069687460165806
**Exoc7**	7640.43516623267	0.1227563104795	0.0446301125459818	2.75052657223361	0.00594995656615075	0.069687460165806
**E330034L11Rik**	78.3679904382974	-0.79039064492059	0.287366660266068	-2.75046048901003	0.00595115679977959	0.069687460165806
**Ctsz**	8163.90840662515	0.206134106256933	0.0749820829746002	2.74911149543231	0.00597570556947595	0.0698510331292527
**Get4**	7247.41275872659	0.337423971838139	0.122738132306421	2.74913725260008	0.00597523599145532	0.0698510331292527
**Cyp2s1**	1998.36409565854	-0.33944648077893	0.123498220689816	-2.7485941002462	0.00598514522826335	0.0699095132407306
**Mccc1**	2435.29201486561	0.201882156199197	0.0734667848982404	2.74793781269762	0.0059971382494203	0.0699545691037948
**Tbc1d4**	651.890206702649	-0.415283034011344	0.151127574676585	-2.74789716502799	0.00599788175789978	0.0699545691037948
**Nr1i3**	73.7786240825008	-1.49512830553549	0.544283123830178	-2.74696796588899	0.00601490090663532	0.0699976318950168
**Napa**	21214.0744513702	0.196877375649491	0.0716585098341991	2.74743887508991	0.00600627033546531	0.0699976318950168
**Plgrkt**	7490.41527656003	-0.221873253998742	0.0807669636806901	-2.74707929935204	0.00601285943874934	0.0699976318950168
**H13**	20897.7160980973	0.134983019027517	0.0491753639536395	2.74493177426757	0.00605234788126932	0.0701742792973957
**Mgat4a**	1265.90936635543	-0.468023640742799	0.170491257920058	-2.74514744305689	0.00604837166261225	0.0701742792973957
**Abcb6**	1743.19596788621	0.434874724541884	0.158408890302795	2.74526716089375	0.00604616547778693	0.0701742792973957
**Selplg**	143957.724405929	0.157983595931711	0.0575359807441207	2.74582259463535	0.006035939311902	0.0701742792973957
**Gm42782**	29.3285108252097	-1.32021382689731	0.480851140345246	-2.74557699072817	0.00604045923551021	0.0701742792973957
**Ccdc71l**	225.812142603838	1.27879828244479	0.466001708901256	2.74419225942316	0.00606599997394682	0.0702808541099118
**Gm29216**	57353.3196348292	-0.501108001934371	0.182680206641853	-2.7430886528216	0.00608642511050482	0.0704656873374169
**Paf1**	9507.50411081903	0.173478608954859	0.0632813687578976	2.74138521906116	0.00611807324107596	0.070780088149511
**Api5**	16310.6770180548	0.170257451491574	0.0621275758339127	2.74044897465061	0.00613553079045157	0.0708933265272025
**Ttc39a**	120.363470921209	0.936375538868608	0.341695782421562	2.740377806927	0.00613685964226085	0.0708933265272025
**Ly86**	378.578910829103	0.33025576871504	0.120581704048989	2.73885471531292	0.00616536118314298	0.0711182988014524
**Ythdc1**	5382.30040612246	0.382601908059263	0.139687590323635	2.73898280565105	0.0061629596521562	0.0711182988014524
**Ppp4r1**	2290.10946686883	0.26178944018443	0.0956404222490309	2.73722589286334	0.00619597310760348	0.0714191282051997
**Hsd17b1**	35.2133183955409	-1.84512684790415	0.67425807088033	-2.73652912377467	0.00620910985691385	0.071489329083967
**Ptp4a1**	146.45460723554	0.97088467038091	0.35481755458672	2.73629266035544	0.00621357378849319	0.071489329083967
**Gm37352**	92.1840780282335	-1.06147930889351	0.387941856715189	-2.73618144193396	0.00621567435711333	0.071489329083967
**9530018F02Rik**	67.5652784394766	-0.963418245237698	0.352280991258319	-2.73480054031995	0.00624180857770913	0.0717375475995352
**Abl2**	1018.42022439487	-0.294489631352987	0.107736881268235	-2.73341522314711	0.00626812572220381	0.0719875051055142
**Tmed3**	3229.88478567646	0.165475969845836	0.0605540705603181	2.73269770825737	0.00628179572237745	0.0720919557155874
**Tab2**	2169.94812768839	-0.401022148199369	0.146763726720931	-2.73243366844933	0.00628683292516423	0.0720972535675493
**Lnpep**	8587.22246125863	-0.363307187555733	0.13297770183527	-2.73209103888553	0.00629337483907113	0.0721197871558137
**Dedd2**	12397.6602394054	-0.155588243742264	0.056998415225369	-2.72969420512966	0.00633930981426116	0.0725933900750822
**Kifc2**	1053.13339366401	-0.299372741964246	0.109682297922157	-2.72945359128703	0.0063439377696307	0.0725936292200951
**Hsd11b1**	16922.2173066534	0.15003338175415	0.0550006723742753	2.72784632037195	0.00637493001383781	0.0726261524655057
**Agap2**	1195.55968811293	-0.352772821642751	0.129325854798038	-2.72778264016626	0.00637616073031377	0.0726261524655057
**Pqlc2**	807.058853033228	0.421973298671372	0.154680199445243	2.7280369445137	0.00637124719102373	0.0726261524655057
**Sltm**	7589.47785628635	-0.170548958128069	0.0624954868842711	-2.7289803893182	0.00635304818504656	0.0726261524655057
**Ddx11**	2771.43645038355	0.322178452030883	0.11811647421681	2.72763350046755	0.00637904391776733	0.0726261524655057
**Slc16a5**	7380.96944721484	0.278370280520832	0.102021746929821	2.72853865864812	0.00636156331676112	0.0726261524655057
**Ict1os**	132.444656840419	-0.845566031168627	0.309992915381632	-2.72769469627282	0.00637786073084748	0.0726261524655057
**Cog5**	4750.72389624415	-0.213202935237934	0.0781825856724517	-2.72698751779781	0.00639154568645211	0.0727159461237732
**Ptpn12**	1295.87131591027	0.526120022184303	0.192964780862711	2.72650801784717	0.00640083974941966	0.0727200418373252
**Rbm17**	5500.83664254262	0.205681854523448	0.0754392577477001	2.72645649843656	0.00640183906419228	0.0727200418373252
**1700028E10Rik**	624.482596837477	-0.414194795551402	0.151928125640704	-2.72625489062463	0.00640575097227945	0.0727200418373252
**Plpbp**	4435.80653078475	0.206803536460548	0.0758704529908023	2.72574537660423	0.00641564694346079	0.0727799488035361
**Ppme1**	8112.41464532707	0.229224860077598	0.0841248418421675	2.72481772396866	0.00643369948465468	0.0729055856445153
**Gm44116**	103.764446204962	-0.813087234809002	0.29841338847009	-2.72470092235991	0.00643597573342139	0.0729055856445153
**Chtop**	13272.0924875049	-0.14569551548239	0.0534789474375338	-2.72435271192594	0.00644276601549683	0.0729300747889249
**Brd4**	10534.3283363953	0.869893542200862	0.319535212997712	2.72237145333679	0.00648152438629751	0.0733161376560587
**Arpc1a**	2735.25247622181	-0.201891331924539	0.074192671761961	-2.72117619071981	0.00650500802288192	0.0735289895384149
**Nipbl**	13366.3579381582	-0.297165553139083	0.109277922256595	-2.71935581316517	0.00654092051398276	0.0738819244005924
**Snx27**	2393.91428305301	-0.251423773433538	0.0924652826036701	-2.71911539503107	0.0065456768002146	0.073882685774342
**Sulf2**	162.445123898121	0.778138129731481	0.286378643031826	2.71716536363713	0.00658437011534743	0.0739486243104273

**Smurf2**	1873.03695412436	-0.303948955451425	0.11182199811053	-2.71814992208408	0.0065648084727818	0.0739486243104273
**Rrm1**	5438.3723598894	0.234302616785999	0.0861965594649477	2.71823629899381	0.00656309479442208	0.0739486243104273
**Ubtd2**	59.796830530843	-0.741124063119732	0.272707979385622	-2.71764715058721	0.00657479121949307	0.0739486243104273
**Zfp976**	35.2543862319969	-1.87176162527931	0.688844503058196	-2.71724840217122	0.0065827182473072	0.0739486243104273
**Gm43653**	129.878675297386	-1.23940443138352	0.45599387134519	-2.71802870448074	0.00656721405316731	0.0739486243104273
**Lef1os1**	2773.8917121884	-0.425767741876781	0.156679567321639	-2.71744267076477	0.00657885515885988	0.0739486243104273
**Rnf11**	946.484420027009	1.4403851040987	0.530170958226645	2.71683139513452	0.00659101744531365	0.0739705568987231
**Zfp397**	3743.63628675835	-0.337304890114749	0.124175811639073	-2.71634938932514	0.00660062195931257	0.073985075557756
**Ece1**	3433.76533956977	0.289495688155467	0.106577401669903	2.71629523350652	0.0066017018616618	0.073985075557756
**5830444F18Rik**	1461.39025305057	-0.531409425590488	0.195753294233638	-2.71468956714584	0.00663379216703884	0.0742918714826091
**Efhd2**	19028.5016869051	0.424657640017538	0.156448673763192	2.71435755767619	0.00664044506858819	0.0743135603307841
**Pp2d1**	49.3125972458343	-1.15546587073614	0.425786120493169	-2.71372366341536	0.00665316388743434	0.0744030542046152
**Nlrc5**	18463.4331273789	-0.286105264008238	0.105447060100951	-2.71325975076338	0.00666248597290534	0.0744544620390563
**Erf**	369.062339256513	-0.473499740861638	0.174690517900356	-2.71050625158559	0.00671805821083764	0.075022284357313
**Ppcs**	2763.75503273491	0.355269719165798	0.131116099557257	2.7095812060109	0.00673682114569793	0.0751785345557806
**Nemp2**	1959.57626431773	0.316126308206057	0.11673352122926	2.7081022218562	0.00676691762681621	0.075460949076959
**Cdk20**	864.281593338982	0.384911874724851	0.142178129855678	2.7072509331468	0.0067842956078043	0.0755478062842207
**Seh1l**	6409.46587923542	0.189276417191065	0.0699135779909992	2.70729123912715	0.00678347190860525	0.0755478062842207
**Asf1b**	2279.54241908287	0.43351033288281	0.160148965245367	2.70691934986043	0.00679107530685843	0.0755698966173505
**Stxbp5**	1228.17578916988	1.19459445358231	0.441403183144525	2.70635668069293	0.00680259382356674	0.0756446512900079
**Tada1**	4330.63111258909	0.255066852439729	0.0942688687379603	2.7057379159682	0.00681528095071402	0.0756942002393795
**R74862**	46.8736659781393	-0.840311552095098	0.310574197314057	-2.70567084890624	0.00681665736749886	0.0756942002393795
**Timm13**	4426.77209578415	0.248561755243634	0.0918938793527712	2.7048782464546	0.00683294287167012	0.0757149654211716
**Nagpa**	5471.47550925188	0.190904045408454	0.0705683502113669	2.70523605605993	0.00682558667824317	0.0757149654211716
**Zfp329**	1570.25426676393	-0.274720058508278	0.101563925734942	-2.70489798932381	0.00683253679319274	0.0757149654211716
**Nars**	13071.1645769554	0.368063373380234	0.136102251093892	2.70431510443072	0.00684453491567766	0.0757901171232135
**Ahcyl1**	2232.56884144237	1.10759609757522	0.409884812514622	2.70221307000905	0.00688796065655157	0.0761105161748128
**Mtf1**	1664.56084510109	-0.252938733764951	0.0935914757021768	-2.70258302764499	0.00688029983233712	0.0761105161748128
**Mcl1**	5766.15062267268	0.961171252577921	0.355668287678027	2.70243731554733	0.00688331622242174	0.0761105161748128
**Bach1**	1403.13182568511	-0.412723326966863	0.152818174632264	-2.70074765622626	0.00691838077138886	0.0761795428475012
**Midn**	882.254113492455	-0.414662360620301	0.153521361495332	-2.70100757693522	0.00691297636113375	0.0761795428475012
**Acat3**	347.304218445161	0.646958044835248	0.239541874232989	2.70081398881428	0.00691700118819114	0.0761795428475012
**Gm37829**	235.377563225215	-0.720108297869088	0.266559693284373	-2.70148982014643	0.00690295934743635	0.0761795428475012
**Gm45069**	26.6097611760826	-1.34312182995048	0.497234906894928	-2.70118169767654	0.00690935807123819	0.0761795428475012
**Asah1**	2706.12882209497	0.239263707212203	0.0886206416572054	2.69986430630577	0.00693677623449218	0.0763287591668249
**Ifitm2**	207.355886420722	1.07634394574056	0.399024632238214	2.69743734792292	0.00698754321249682	0.0767266330308797
**Fam169b**	23089.7365587297	-0.263444718207361	0.0976599997188593	-2.69757033550848	0.00698475276787403	0.0767266330308797
**Gm44667**	60.4878605021811	-1.10669151806052	0.410267241475878	-2.69748935859308	0.00698645176666335	0.0767266330308797
**Dph7**	2293.43208425244	0.287929052492491	0.106769818517214	2.69672700104918	0.00700246520388017	0.0767834684881975
**Ints11**	12652.5685293037	0.189214034895113	0.0701583997774012	2.69695482644205	0.00699767624651458	0.0767834684881975
**Pex11b**	2882.90734332171	0.184990330930448	0.0686465168527056	2.69482472544646	0.00704256663305768	0.0771335250004775
**Apobec1**	1498.76652849128	0.25658175957625	0.0952154788911268	2.69474840188154	0.00704417988676062	0.0771335250004775
**Mob3b**	643.897836180286	-0.433816255624258	0.16105363100253	-2.69361362996804	0.00706820477402279	0.0773428490446369
**Gm38062**	619.104382624766	-0.667364708578401	0.247820486679702	-2.69293599379031	0.00708258644711044	0.0774464362575428
**Wac**	7907.8811562142	0.511501856522474	0.189981101616584	2.69238283266079	0.00709434582101638	0.0775212254519799
**Gm9958**	174.537684998098	-0.895338111942213	0.332666438239293	-2.69139897815054	0.00711530441144516	0.0776963628628839
**Snx11**	2324.16261623084	0.280860830790089	0.104377905900322	2.69080729650107	0.00712793547860086	0.0777451059830798
**Carf**	361.45075715236	-0.52041869267375	0.193445054729205	-2.69026620195725	0.00713950424303589	0.0777451059830798
**Zap70**	32626.3209639417	0.18392259582599	0.0683614795083859	2.69044200255245	0.00713574372610894	0.0777451059830798
**Abce1**	10828.1060902172	0.195382656201534	0.0726222768632785	2.69039562845666	0.00713673553282761	0.0777451059830798
**Sun2**	4524.21567570379	-0.372751273295172	0.13860208141693	-2.68936273888916	0.00715885812755109	0.0779020217650708
**Gm45693**	17.4263225183376	-2.28565692910932	0.850083328631447	-2.68874456435819	0.00717212771562656	0.0779925579124415
**Hat1**	3767.82615128387	0.205710498779265	0.0765262824788348	2.68810259842636	0.00718593136927702	0.0780887728177227
**Slpi**	871.28906168294	0.634688862883687	0.236131537325552	2.68786147785354	0.0071911221393076	0.0780913242929496
**Noc4l**	7355.46066874128	0.201584468390291	0.0750082774270018	2.6874963044775	0.00719898988843286	0.0781229226391437
**Zmynd19**	430.722342865467	1.90696106479451	0.709749439414442	2.68680883547832	0.00721382254150445	0.0781762048050107

**Serpinb6a**	1613.32660459539	-0.313227402868706	0.11657370464905	-2.68694731639259	0.00721083250897174	0.0781762048050107
**Tgm2**	218.572657436822	0.676073273605581	0.251672318082158	2.68632354466922	0.007224309559168	0.0782360451710036
**Cd160**	5043.60637187495	0.902876838447536	0.336245568239873	2.68517096946074	0.00724927128927818	0.0783986051511024
**Aida**	1514.15445142645	-0.224463755174368	0.0835880388673062	-2.68535735753651	0.00724522937758487	0.0783986051511024
**Tmem163**	113.47235291506	1.34296769761463	0.50046882443652	2.68341929015596	0.00728735625022048	0.0787564282817038
**Ndufa9**	10310.4261343616	0.141145763833195	0.0526160406091873	2.68256148123296	0.00730607211422576	0.0788273497984947
**Abraxas2**	2653.21189020368	-0.205329154849725	0.076552459181144	-2.68220194421005	0.00731392939045499	0.0788273497984947
**Mga**	6590.77257648422	-0.333188811329909	0.124207727688689	-2.6825127351577	0.00730713696073524	0.0788273497984947
**A630023P12Rik**	1255.82446940715	-0.355947354054527	0.132702269020413	-2.68230043602173	0.00731177620933148	0.0788273497984947
**Paxip1**	3533.5933989308	1.13687459795057	0.423937183666487	2.68170531331584	0.0073247951678351	0.0788904972382622
**E230029C05Rik**	64.8833400424706	-0.780866436375279	0.291255230570304	-2.68103832795131	0.00733941091390218	0.0789939192420469
**Nsun4**	3055.3297991095	0.280537567323107	0.104670705348406	2.68019181096862	0.00735799843636649	0.0791399190183118
**Gm49628**	226.342441526004	-0.570181156738934	0.212858773803967	-2.67868289640738	0.00739123540618416	0.0794431761904801
**A130014A01Rik**	364.542454948035	-0.624939333263334	0.23342186033424	-2.67729565846349	0.00742191095297435	0.0796642036008289
**Gm49768**	17.4982423817752	-1.66213947743911	0.620787912578453	-2.67746752757376	0.00741810427929443	0.0796642036008289
**Hars2**	5445.15825782148	0.18991480387208	0.0709612819338245	2.67631585417506	0.00744364580837279	0.0797433667404395
**Trim46**	318.922168508959	0.853672851178794	0.31893872925439	2.67660454148825	0.0074372359753918	0.0797433667404395
**9330160F10Rik**	145.538424072146	-0.871822406937591	0.325759185966401	-2.67627881114459	0.00744446864727972	0.0797433667404395
**Gm44694**	501.506929185063	-0.518664895975988	0.193835005278112	-2.67580613332363	0.00745497543045125	0.0798016629467529
**0610040J01Rik**	22.4988097032272	2.23952935423745	0.837155883472321	2.6751640864643	0.00746926828538084	0.0799003804295627
**Mpp5**	1189.4901998735	-0.371456995790868	0.138868819052185	-2.67487689696044	0.0074756694736683	0.0799146023721787
**Tnfaip8l2**	11340.1503298239	-0.154743253039181	0.0578751465870928	-2.67374274044071	0.00750099687770627	0.0799683408674004
**Cdkn2aipnl**	15121.7243828416	0.192439801380238	0.0719712861247283	2.67384135732596	0.00749879156528518	0.0799683408674004
**Acsbg1**	2404.65273734541	-0.396919943579405	0.14841771643459	-2.67434342148993	0.00748757320680231	0.0799683408674004
**Tom1**	1690.22191078189	0.264858988678874	0.0990472608833002	2.6740667668835	0.00749375304471884	0.0799683408674004
**Gm47445**	74.5826188216342	-1.23590121699688	0.462433160153705	-2.67260508867073	0.00752647961250494	0.0801857601448549
**Slc35e1**	1720.64910965101	1.14872206843911	0.429901532001779	2.67205855975957	0.00753874912152248	0.0802135775906159
**Gm19265**	101.688202567746	-0.70412849648782	0.263517663731266	-2.67203528794899	0.00753927196875688	0.0802135775906159
**Asxl2**	6293.4787316727	-0.22452469983892	0.084060211298906	-2.67099851843748	0.00756259806067903	0.0804074612969767
**Gm42908**	216.777157123244	-0.854415344827053	0.31994777650644	-2.67048377130965	0.00757420328477416	0.0804765483197481
**Trdv2-2**	188.262549837244	-0.537730787533131	0.201382324926824	-2.67019852774331	0.00758064111250743	0.0804906752087463
**Gm43197**	212.355988845813	-0.640978040403104	0.240095656527357	-2.66967778457112	0.0075924067217636	0.0805613149594809
**Pmpca**	12600.6502090479	0.252573398109212	0.0946328717397511	2.66898164946121	0.00760816068498779	0.0806741506819331
**Gtf2h1**	4775.11515864503	0.169588002459053	0.0635490858488028	2.66861435052804	0.00761648468864331	0.0807081030524227
**Mttp**	286.066418540828	-0.527644612742774	0.197860884742758	-2.66674544303576	0.00765896585495309	0.0811037130218386
**Gm23245**	35.2054133086667	-0.964544437160463	0.36173436706603	-2.6664440124496	0.00766583737067363	0.081121960678109
**Wipf1**	2587.30242591838	-0.39826349935586	0.149384441173803	-2.6660306537044	0.00767526942366114	0.0811672619520997
**Rcor1**	2597.79946038896	-0.234637375143387	0.088023139440759	-2.66563288510406	0.00768435555848869	0.0811965866755906
**Gm14636**	24.4535651637325	-1.52767611451467	0.573138271170094	-2.66545821725677	0.00768834850034786	0.0811965866755906
**Hif1a**	8268.00651910997	0.167726128463212	0.0629346993648383	2.66508190483104	0.00769695739690644	0.0812330594126288
**Star**	211.935608263317	-0.554525164229061	0.208136443281303	-2.66423868634866	0.00771627907493528	0.0813628429076437
**Zmym5**	4439.96708898685	-0.198795953643446	0.0746204519314875	-2.66409474209524	0.00771958178250475	0.0813628429076437
**Rnh1**	7038.89154167947	0.177799805316123	0.0667541883676827	2.66350036849823	0.00773323271866064	0.0814522379331121
**Wfs1**	168.952232991601	0.694045229046455	0.260600924026423	2.66324930212483	0.00773900543793025	0.0814585896362504
**Cldnd1**	4955.9395658724	0.378927075269455	0.142297836393206	2.66291522678096	0.00774669275356245	0.0814850719077994
**Gm17764**	77.3624679565089	0.89322559053369	0.335482817643891	2.66250771591478	0.0077560791372708	0.0815293788965817
**Wipi2**	8304.10536058374	-0.170036616654715	0.0638848188232696	-2.66161225447163	0.00777674051883015	0.0816920669034711
**Ifrd1**	2799.59658135381	-0.255665258836497	0.0961229754137576	-2.659772627054	0.00781934185899749	0.0817423099248933
**Phf7**	1249.73541550883	-0.309426086387042	0.116316697303288	-2.6602035095635	0.00780934495059179	0.0817423099248933
**Sla**	21851.4596182512	0.196969395298954	0.0740620855111139	2.65951726770368	0.00782527186254297	0.0817423099248933
**Maged1**	229.951721919613	0.822546897258014	0.309402773188963	2.65849878713161	0.00784896331258257	0.0817423099248933
**Snx16**	392.183665294769	0.390649409635518	0.146872426723974	2.65978726129233	0.00781900214192711	0.0817423099248933
**Tprgl**	29543.1563114352	0.450045786124868	0.169125747485525	2.66101284290487	0.00779059855023566	0.0817423099248933
**Tmem43**	8592.54979431092	0.184027686739156	0.0692119129743035	2.65890189753144	0.00783957866232988	0.0817423099248933
**Rrp8**	3320.07432770685	0.286111750094579	0.107606987751451	2.65885846331315	0.00784058935338634	0.0817423099248933
**Cipc**	4334.83807108828	0.294523798808964	0.110784797435525	2.65852179745486	0.00784842734783383	0.0817423099248933

**Gatad2a**	11675.0129740968	0.468562608644621	0.176192886583806	2.65937301856821	0.0078286234239538	0.0817423099248933
**Pars2**	2011.68300170918	0.234301410763129	0.0881124076427819	2.65911937979285	0.00783451972138707	0.0817423099248933
**Tgfbrap1**	2021.17413356543	0.294935341691352	0.110861633564597	2.66039144659995	0.00780498820953168	0.0817423099248933
**Snhg1**	1124.02103259679	0.300673161314875	0.1130439354691	2.65979028478766	0.00781893195659651	0.0817423099248933
**Hspa9**	17652.1452929684	0.131821636599005	0.0496099786191017	2.65715971399851	0.00788021001661749	0.0819769901300947
**Cercam**	666.588607076043	0.547206445196761	0.205959457377457	2.65686486148537	0.00788710522543781	0.0819769901300947
**Gm43237**	71.3105273026114	-0.848440170667046	0.31931936884217	-2.65702695625208	0.00788331392525461	0.0819769901300947
**Aqp3**	1315.30386848973	-0.284089191678531	0.106948698190606	-2.65631276008821	0.00790003080667537	0.0820572085832457
**Hif1an**	4926.14258673083	-0.220806630769906	0.0831831509136391	-2.6544634141011	0.00794346529981306	0.0824540070679542
**Art2b**	9483.85393361889	-0.340638012114811	0.128384337376696	-2.65326767326247	0.00797166267982695	0.0826922243884353
**Ldhb**	1625.4195939505	0.328549144089996	0.123854561494592	2.65270120151643	0.00798505220530037	0.0827766234203407
**Trappc2l**	2586.16540088497	0.251958415831087	0.0950109197080615	2.6518890313374	0.00800428436801999	0.0829214390446358
**Trim68**	848.125782362471	-0.376409099204541	0.141964520134168	-2.65143078600769	0.00801515389582076	0.08297948747467
**Xrcc6**	6937.86419941481	0.137057862974223	0.0517154427110656	2.65023087474985	0.00804367829534051	0.0831622785972868
**Tmem64**	679.941917475912	0.904096272170236	0.341113225586974	2.65042866811893	0.00803897008623299	0.0831622785972868
**Sap18b**	1430.29538270172	0.24979334653022	0.0942687219955719	2.64980092274884	0.0080539212502037	0.0831622785972868
**Gm26631**	152.009156784958	-0.871557529140241	0.328901170201119	-2.64990704839175	0.00805139188268098	0.0831622785972868
**Gm43544**	67.3213458422953	-1.09280862234294	0.412723008364637	-2.64780155260319	0.0081017067926194	0.0836009128561257
**Rnf213**	6109.79117612716	-0.364298518711672	0.137667614886135	-2.64621798680092	0.00813973443531344	0.0838834502925009
**Gm26685**	77.8146587595454	-0.896556207863338	0.338800776581286	-2.64626373324807	0.00813863364605082	0.0838834502925009
**Ppm1b**	14434.638989524	0.13973026276648	0.0528103338199633	2.64588864828685	0.0081476631880157	0.0839102802964466
**Chsy1**	901.734046728192	0.42816449483908	0.161856477394897	2.64533432168083	0.00816102409045947	0.0839929827520378
**Sfi1**	5122.99914007467	-0.246041910887607	0.09302579340645	-2.64487839208848	0.00817202802918342	0.0840513352844929
**Maged2**	295.431678965171	0.554340855224879	0.209637226215956	2.64428634756801	0.008186336941972	0.084143581992598
**Gm12592**	369.252965866738	-0.507253302703739	0.191871488351687	-2.64371380584686	0.00820019582130825	0.0842310857603351
**Smc3**	10516.0577545903	-0.17601118830262	0.0666097652486128	-2.64242318893753	0.00823151338652484	0.0843328715419193
**Xcl1**	492.748573020396	0.420683335842435	0.159182356816283	2.64277614841423	0.00822293800549163	0.0843328715419193
**Zfp275**	813.635794664641	-0.370468929705457	0.140192618510405	-2.64257086886469	0.00822792443302256	0.0843328715419193
**Nmnat2**	423.308613655061	-0.422137078333007	0.159734770133656	-2.64273757040994	0.00822387489400703	0.0843328715419193
**Rpl17-ps3**	1650.85731441586	-0.182655922659696	0.069152964931754	-2.64133176126225	0.00825808099001047	0.0845500858736809
**Pcif1**	14282.4192082358	-0.142993076904588	0.0541433837832104	-2.64100739394367	0.00826599153657781	0.0845761224947121
**Dcaf13**	6822.48903575813	0.184237203435923	0.0697991310214684	2.63953434290258	0.00830200112056378	0.0848343914764744
**Vav3**	781.424486489612	0.422627828297668	0.16010491625618	2.639693009935	0.0082981156801347	0.0848343914764744
**Tet3**	9712.58341115817	-0.470436230454353	0.178248083171509	-2.63922181985936	0.00830965894975481	0.0848576124894923
**Scai**	744.169858813008	-0.389461233089184	0.147630692405957	-2.63807767031423	0.0083377482749711	0.085089313192176
**Nrde2**	3502.57261414663	0.230267658283535	0.0872954598536687	2.63779649788806	0.00834466415855307	0.0851047722629907
**Gm43329**	145.630282860974	-1.13832368889588	0.43161252997589	-2.63737405621534	0.00835506443127775	0.0851557246077901
**Gpn2**	1599.85153129652	0.353895930428442	0.134198058140665	2.63711662695962	0.00836140787703233	0.0851652901864243
**Gm26944**	39.0539061585156	-1.02221058054231	0.387792199786921	-2.63597509466146	0.00838958889677217	0.0853971267741854
**Kpnb1**	4161.15045490598	-0.278942749584837	0.105835615289508	-2.63562269489154	0.00839830573873818	0.0854306672209797
**Rnu2-10**	42.8414378892816	1.09145185203937	0.414222048547497	2.63494388062304	0.0084151194939068	0.08550473834162
**D630030B08Rik**	64.9552512048462	-1.11644552515496	0.423716081872702	-2.63489060934529	0.0084164402594309	0.08550473834162
**Igkv6-29**	18.3932962189859	-3.06719704453692	1.16482831656596	-2.63317520781032	0.00845906978736084	0.0858824501542814
**Slc11a1**	182.717357472357	0.550550793888652	0.209120793523545	2.63269273519979	0.00847109447944273	0.0859481244788197
**Zfp36**	12787.7838006997	0.420896964159476	0.159886214058993	2.63247814476474	0.00847644763851532	0.0859481244788197
**Yy1**	3337.34464245557	-0.194178556398472	0.0738244978348903	-2.63027263433279	0.00853164172823132	0.0863965801489338
**4931414P19Rik**	440.125201472681	0.4374635824497	0.166305113943675	2.63048785497878	0.00852624160213024	0.0863965801489338
**Aqp11**	84.7223703075556	-1.07641467717705	0.409421620915753	-2.62911048705595	0.00856085417278102	0.0866367239566542
**Cbll1**	3547.31926944774	-0.181200269199937	0.0689580241599838	-2.62768940101226	0.00859669697204037	0.0867765241437797
**Creld2**	4276.07595699338	0.281304305265233	0.107048983801625	2.62780920729267	0.00859367003401307	0.0867765241437797
**1700030K09Rik**	707.079778306624	0.244990818232572	0.0932210075324496	2.62806447513767	0.00858722380049774	0.0867765241437797
**Gm42548**	224.245796086516	-0.542328911933176	0.206372509105684	-2.6279125755817	0.00859105917197345	0.0867765241437797
**Sbf1**	16451.5858167639	-0.30540322614744	0.116280147074179	-2.62644341129542	0.00862823378485654	0.0870391035518467
**Syvn1**	2703.22605968496	-0.274257274098579	0.104443680233092	-2.62588673136092	0.00864235710982157	0.0871257971717585
**Ifit1bl1**	15188.0690732815	-0.304214820296195	0.115995887532298	-2.62263453272419	0.00872528122042006	0.0879055346484392
**Pcmtd1**	6315.45449223033	-0.249840504538897	0.0952841231863341	-2.6220580741486	0.0087400536435891	0.0879419062975949

**Zfp467**	223.312334472885	0.781959885234887	0.298217221082914	2.62211512264571	0.00873859071352776	0.0879419062975949
**Utrn**	32672.9062557874	-0.394104656644926	0.150317720583653	-2.62181102211168	0.008746391478828	0.087947132296663
**Zbtb6**	391.05596257489	-0.439779919228163	0.167752306543818	-2.62160281601427	0.00875173595488783	0.087947132296663
**Plk3**	967.176537411832	1.70839121317658	0.651760393723494	2.62119519631528	0.00876220766077567	0.0879675426873467
**Gm49668**	54.824053294074	-0.82494741640058	0.31473458306363	-2.62108919957423	0.008764932539134	0.0879675426873467
**Bmp7**	54.881542660115	1.0725739935596	0.409475669349149	2.61938394353058	0.00880887407689902	0.0879800157166838
**Ccr9**	9143.32133943748	-0.328522620665907	0.125362198239011	-2.62058758765189	0.00877783784486568	0.0879800157166838
**Trim3**	457.869168907006	0.772181419937207	0.294715787254547	2.62008841511524	0.00879069724263962	0.0879800157166838
**Trub2**	2253.44971161074	0.284953502692727	0.108765174286942	2.61989652994043	0.00879564495600552	0.0879800157166838
**2610024D14Rik**	70.4999633724062	-0.990542323826299	0.377988724690327	-2.6205605065014	0.00877853506255863	0.0879800157166838
**4930523C07Rik**	30924.4344561386	-0.223360773669141	0.0852539644356135	-2.61994588929445	0.00879437199922514	0.0879800157166838
**Gm38342**	32.3394898034549	-1.36575039978457	0.521350101908601	-2.61964157057747	0.00880222287147	0.0879800157166838
**Gm4356**	119.457244016247	-0.835664579884452	0.319040250569031	-2.61930768419968	0.00881084373934931	0.0879800157166838
**Rab21**	616.391066087014	-0.424840483356325	0.162225075956879	-2.61883362267158	0.00882309685542007	0.088014664966392
**Pmel**	269.528023992839	-0.71526653857621	0.273133715799851	-2.61874128751043	0.00882548522224405	0.088014664966392
**Tctn3**	943.780145534511	0.372687006042397	0.142351809456603	2.61807002991426	0.00884286552644691	0.0880765057523539
**Klhdc8b**	441.766959272021	-0.51029827873731	0.194906594230985	-2.61816836290594	0.00884031756354049	0.0880765057523539
**Tm9sf1**	8059.22769094107	0.143345949625285	0.0547744499469938	2.61702216569957	0.00887005811396217	0.0881479966989897
**Tsacc**	377.39089509716	-0.348520277738436	0.133178994747052	-2.61693128409915	0.00887242005233215	0.0881479966989897
**Asap1**	5961.72017277713	-0.242368583997304	0.0926102270411513	-2.61708227850049	0.00886849614013683	0.0881479966989897
**Matr3**	16099.6998977921	-0.130136947933968	0.0497216282707823	-2.61731066459141	0.00886256398514072	0.0881479966989897
**Gm42671**	63.8360462540396	-1.24526389534467	0.475956668560803	-2.61633879216379	0.00888783220809346	0.0882454770654875
**Zfp266**	1617.54809090512	-0.226853209475356	0.0867544842337114	-2.61488742027705	0.00892568700536847	0.0885655227604478
**Agbl5**	777.589065006895	-0.43874119839023	0.167916491108211	-2.61285354103481	0.00897897709685352	0.089038226630032
**Tspan13**	14073.9641540407	0.191566047498269	0.0733507261239945	2.61164486871526	0.00901078019869665	0.0890446326948592
**Hsp90aa1**	24932.2554421131	0.215313388806614	0.0824420585858927	2.61169350328981	0.00900949856528422	0.0890446326948592
**Fkbp5**	10409.274316134	1.96952079632165	0.753870266300249	2.61254606311431	0.00898705806611825	0.0890446326948592
**Ldb1**	7981.0064584572	1.19377650580812	0.45711582235713	2.61154054929094	0.00901352980569273	0.0890446326948592
**Cdc14b**	2523.30173152691	-0.390275637656343	0.149439246498663	-2.61160067920869	0.00901194483545632	0.0890446326948592
**Gm26910**	281.669344671336	1.29228945405801	0.494678952458834	2.61238010559092	0.00899142237062681	0.0890446326948592
**Mcm6**	16397.8594977708	0.12965652921126	0.0496871467392181	2.60945813394678	0.00906857419917273	0.089308452285228
**Zfp395**	1888.86445299633	-0.354826173707243	0.135951971807523	-2.6099376786502	0.00905587188441586	0.089308452285228
**Pcbp1**	3535.5131885036	1.04839001886268	0.401762425921198	2.60947752010118	0.00906806038489305	0.089308452285228
**Gm44199**	48.9930310108634	-0.950235180607802	0.364124595335101	-2.6096429430517	0.00906367704098016	0.089308452285228
**Gm45412**	34.5147503096219	-1.41314593863747	0.54152441709416	-2.60957012099373	0.00906560643201282	0.089308452285228
**Bloc1s6**	4581.45947539479	0.185225630310754	0.0710006587184285	2.60878749090644	0.00908636505921825	0.0894277665447233
**Dym**	4702.4086094154	0.2522416311216	0.0967233811184579	2.60786614575309	0.00911085736901431	0.0895677323962199
**Vmn2r96**	233.053881359616	-0.654846674065839	0.251108374771304	-2.60782490692411	0.00911195500610144	0.0895677323962199
**Gramd2**	34.2782390995369	2.3484197925874	0.900761662393962	2.60714891700207	0.00912996438959827	0.0896888085329801
**Lcorl**	1771.29880202737	-0.230250822096639	0.0883508475732183	-2.60609635811161	0.00915806939375748	0.0898491687881999
**Trak2**	2758.80704338955	-0.331795490014279	0.127325285916521	-2.60588843469643	0.00916363040677127	0.0898491687881999
**Ankrd61**	18.3387499538012	-2.19919722133103	0.843934522324476	-2.60588607665167	0.00916369349110857	0.0898491687881999
**Gm43859**	37.6122087325441	-1.1969601658076	0.459365026015743	-2.60568414663459	0.00916909712581237	0.0898491687881999
**Taf15**	638.992299092069	-0.498036080393527	0.191254574156777	-2.60404794284958	0.0092129868320066	0.089965756971158
**Per1**	564.508291917733	-0.402948658734778	0.154718980710194	-2.60439059826504	0.00920377991552021	0.089965756971158
**Tmx1**	7149.42804205212	0.220272923350733	0.0846051879037528	2.60353920141772	0.0092266715279172	0.089965756971158
**Smim5**	163.061642480601	0.693925328580418	0.266471740130386	2.6041235301007	0.00921095514797728	0.089965756971158
**Grwd1**	6140.67017948087	0.200448015870113	0.0769892665306552	2.60358391374335	0.00922546807908626	0.089965756971158
**Gm37305**	135.229944588382	-0.8927018774981	0.342855600726967	-2.60372552061357	0.00922165760197384	0.089965756971158
**Gm37645**	291.134104565963	-0.571124793774328	0.219293106483656	-2.60439009202004	0.00920379351191759	0.089965756971158
**Gm37728**	303.772811764477	-0.656562816018955	0.252068450132892	-2.6047004917625	0.00919546036188018	0.089965756971158
**Zfp959**	2135.08655776785	-0.284411134532803	0.10931060865643	-2.60186214337828	0.00927191146675383	0.0900169494649661
**Lamp2**	2463.98804555542	0.325342959327683	0.12502223719765	2.60228073517308	0.00926060113061445	0.0900169494649661
**Agpat5**	2118.85079379622	0.553368805111054	0.212615895080556	2.60266902858506	0.00925012046490774	0.0900169494649661
**Snx1**	10672.9183662111	0.162800696657583	0.0625558237585696	2.60248665713208	0.00925504164569402	0.0900169494649661
**Pth1r**	46.7451559237046	4.47846505225704	1.72117829784133	2.60197624956917	0.00926882709987112	0.0900169494649661

**Ccdc84**	1956.31570774927	-0.272479004149606	0.104724393979426	-2.60186756681673	0.00927176484684555	0.0900169494649661
**Phf13**	1889.900561455	-0.396347989123082	0.152259712770323	-2.60310479976378	0.00923837090441302	0.0900169494649661
**Ftl1-ps1**	2475.64732366612	0.325049459716413	0.124944627226985	2.60154811719836	0.00928040453420333	0.0900439250279814
**Fez2**	3026.0392627519	0.167943564626507	0.0645961029721952	2.59990242288759	0.00932502715754453	0.0904212017978025
**Oxsr1**	1526.49532837322	0.651119764664229	0.250499608764224	2.59928455727481	0.00934182980668426	0.0905284208265215
**Rab12**	325.580887006187	0.740755067162933	0.285128342583247	2.59797065578165	0.00937765074428188	0.0908196943931466
**Gm26789**	202.795688966041	-0.812126635708695	0.312931481807496	-2.59522190294771	0.00945298665393792	0.0914930655442874
**Map3k5**	1031.37765113787	0.567789292119103	0.218823221129363	2.5947396678867	0.00946625893511216	0.0915652805651089
**Usf1**	3794.7212055205	-0.180866445307291	0.0697179345062372	-2.59425995030174	0.00947947841695477	0.0915911611889796
**Abcf1**	10217.8389444309	0.134912693300885	0.0520050941960733	2.59422072753542	0.00948055999868159	0.0915911611889796
**9430081H08Rik**	130.298424825319	-0.717313061867726	0.276708393208102	-2.59230684530867	0.00953346987533873	0.0920458853098728
**Slc28a2b**	13226.0433089474	-0.461001260192405	0.178041684099829	-2.58928835976365	0.00961745210431858	0.0927998731217072
**Bbip1**	1321.97265868207	-0.289353977682178	0.111828538414594	-2.5874788473889	0.009668113149066	0.09317459259317
**Gm30238**	231.145855348959	-0.675429208279355	0.261035152523188	-2.58750287748834	0.00966743882107054	0.09317459259317
**Imp3**	3144.35507048107	-0.160509618580126	0.0620526596608132	-2.58666783112101	0.00969089633226892	0.0933370742711255
**Add3**	40705.8206100513	-0.183305022205513	0.0708850067449614	-2.58594913964007	0.0097111258973047	0.0934747774977581
**Atpaf1**	2476.57662144653	-0.217953932874956	0.0843123774302796	-2.5850763496165	0.00973574359876064	0.0935973837008368
**Slc26a10**	1043.81055232811	-0.646187774895614	0.249951401592783	-2.58525365642228	0.00973073802919125	0.0935973837008368
**Psmd1**	10240.0624953778	0.142249927694447	0.0550370748729775	2.58462005880131	0.00974863575310645	0.0936071015001209
**N4bp2**	2765.68682931	-0.500921408144703	0.193806939938165	-2.58464123268509	0.00974803716443861	0.0936071015001209
**Mnt**	552.690365312995	-0.405498171588451	0.156919423719263	-2.58411713462514	0.00976286312348603	0.0936296008744792
**Ccdc51**	1110.69385622253	0.290689837927869	0.11249023126162	2.58413405917718	0.00976238403911249	0.0936296008744792
**Gm43466**	37.4341816065835	-1.24147773534706	0.480468999874611	-2.58388727612197	0.00976937181784728	0.0936350314682601
**Myo1g**	28954.6728708326	0.215378740852191	0.0833799149229957	2.58310099082136	0.00979166559368581	0.0937916563888798
**S1pr4**	47047.3776486461	-0.191704625273615	0.0742221729025083	-2.58284846396805	0.00979883517338876	0.0938033085219239
**Dglucy**	3298.33645710863	0.316316293441383	0.122483810595463	2.58251512508951	0.00980830627923028	0.0938369654170197
**Clcn3**	4256.51716766619	-0.181676478900308	0.0704047247322708	-2.5804586210822	0.00986691802661322	0.0943404292144081
**Tyw1**	5877.20145410241	0.179808301252642	0.0697036964537621	2.57960926608702	0.00989121613430592	0.0945153988042804
**Srprb**	6485.11793991904	0.169421185836081	0.0657286829255191	2.57758376245059	0.00994937645105132	0.0950135301449791
**Cybb**	791.163353488768	0.772750143631602	0.299916045252538	2.57655485881365	0.0099790369116077	0.0950291828133155
**Rabep2**	6466.06876688909	0.307075188561785	0.119168099685808	2.5768237420199	0.00997127815650271	0.0950291828133155
**Fnip1**	712.477537006078	-0.352529709888669	0.136826048702817	-2.57648096419386	0.00998117011842592	0.0950291828133155
**Tm7sf3**	2114.26832180938	0.500650506229917	0.194269982857995	2.57708627377538	0.00996370786060311	0.0950291828133155
**Tgtp2**	47964.8730962758	-0.347593720868804	0.134898715124944	-2.57670149450171	0.00997480500142839	0.0950291828133155
**Atp23**	653.636373418292	0.276051593278365	0.107262205795647	2.57361473438549	0.0100642271626289	0.0957043013889825
**H2bc6**	437.864077026457	-0.469023570555153	0.182240479735165	-2.5736519747794	0.0100631440831472	0.0957043013889825
**Bod1**	599.653319289981	1.52952494111849	0.594381204138285	2.57330637387153	0.0100731993424249	0.0957318468266522
**Golga4**	6611.95686363205	-0.163731822256609	0.0636366652969362	-2.57291643885828	0.0100845552445608	0.0957819993903218
**Klhdc1**	3554.45476274618	0.195579561801614	0.0760334824239705	2.57228204688879	0.010103054718966	0.0958998995221372
**Gtf3c6**	2817.64236858227	0.248469471251502	0.0966099366134043	2.57188318263556	0.0101147014476105	0.0959526494340753
**Oat**	8189.78697446348	0.187573016669299	0.0729605341647732	2.57088327020313	0.0101439511892318	0.0961722255648164
**Pip4k2a**	8727.66279977054	0.341518328559291	0.132897998516972	2.56977781735122	0.010176375865023	0.0964216202676892
**Gdpgp1**	1463.73417192965	-0.380979911399242	0.148285415734392	-2.56923386236211	0.0101923647707349	0.0965150791421089
**Aco2**	20551.7712871463	0.196195644060722	0.0763795317851904	2.56869398744814	0.0102082558573781	0.0965495123317563
**Ppp1r10**	8532.05994423468	-0.447222216486358	0.174104793467446	-2.56869559751656	0.0102082084326333	0.0965495123317563
**Hspe1**	2509.52576717735	0.263946823273781	0.102773082775329	2.56824857390717	0.0102213830977637	0.0966156769474876
**Wasf2**	7525.67057187317	-0.318139571607236	0.123892561303595	-2.56786661168176	0.0102326522657385	0.0966642096829986
**Nr1h2**	12385.9887733299	-0.205791184880543	0.0801630195326877	-2.56715859856837	0.0102535702836932	0.0968037788856528
**Gm38366**	105.265832050013	-0.902658327966099	0.351656020206543	-2.56687864304421	0.0102618519741921	0.0968239530283499
**Mrpl2**	3607.37261612227	0.207904706730089	0.0810471047514648	2.56523298849034	0.0103106544742315	0.0971680517646328
**Mllt11**	1384.04185358162	-0.293123035453115	0.114267090215922	-2.56524459404036	0.010310309585333	0.0971680517646328
**Marcksl1**	255.681675842359	0.377124418942189	0.147037445319448	2.56481890121842	0.0103229668681619	0.0972259348126881
**Pcyox1**	2187.83327777802	0.266518366475911	0.103929504751167	2.56441486095814	0.0103349931371439	0.0972810554731039
**Myo1f**	18481.4269283503	0.227899457215995	0.0888818966292876	2.56407059096103	0.0103452501794189	0.0973194669117034
**Mrpl27**	1857.22032311038	0.222366393475237	0.0867595857806022	2.56301815499163	0.0103766622350153	0.0975567224565254
**Tbc1d20**	9891.18481102664	-0.139122480384296	0.054322226267449	-2.56105999226436	0.0104353334573896	0.097991388133545

**Gm20507**	28.6621993551744	8.1491411101916	3.18173391339108	2.56122646708262	0.0104303340199772	0.097991388133545
**Hid1**	19085.6097786915	0.105478699986638	0.041190420825689	2.56075800810597	0.0104444078438954	0.0980181860609055
**Zdhhc8**	1878.51356010072	1.02583627260291	0.400816821621819	2.55936432121808	0.0104863780700323	0.0983534876485116
**Rps13-ps4**	347.483057245189	-0.527310179768233	0.206048936027708	-2.55915021903983	0.0104928389345826	0.0983555402095285
**Pxmp4**	2710.99990493846	0.244354290088543	0.095500379673064	2.55867349349883	0.0105072375880839	0.0984001053700366
**Net1**	1964.22668802279	0.310738752350571	0.121449722404895	2.55857935446419	0.0105100829686978	0.0984001053700366
**Nfx1**	10809.0016139235	-0.163590747385205	0.063946598272821	-2.55824002845724	0.0105203448882896	0.0984376926394177
**Enpp2**	184.03938203298	0.569150901460904	0.222506437109349	2.55790757721405	0.0105304075437774	0.0984733719093774
**Aagab**	5354.37109526096	0.190843642493821	0.0746525593638243	2.55642464397947	0.0105753974076407	0.0988354311697472
**Rab11b-ps2**	1568.40495914944	0.2273490079131	0.0889562142291592	2.55574059533861	0.0105962079271281	0.0989712200994411
**Cdc23**	10347.6087401212	0.189777485767721	0.0742914451452633	2.55449985387477	0.0106340474356388	0.0992147851086518
**Diaph1**	4621.6882765063	-0.38398463337753	0.150329341719952	-2.55428932891127	0.0106404798312125	0.0992147851086518
**Pycr2**	9168.71286320823	0.2154250668603	0.0843419573016786	2.5541862407788	0.0106436308555814	0.0992147851086518
**Pomk**	1392.41663219106	0.333703715398392	0.130667870841149	2.55383142964097	0.010654482468803	0.0992147851086518
**Map4k1**	14203.6983426764	0.195067495087663	0.0763827283972407	2.55381680100746	0.0106549300848951	0.0992147851086518
**Vma21**	1540.03901718041	-0.251616054343448	0.0985319631765215	-2.55364905185817	0.0106600641739511	0.0992147851086518
**Taf9b**	2960.38722469142	0.323900093937671	0.126889963051401	2.55260610176445	0.0106920338327604	0.0994535874278667
**Strada**	3782.59253930275	-0.175141545088955	0.0686548899279683	-2.55104254442343	0.0107401214339253	0.0998419430291215
**R3hdm1**	4564.62623360216	-0.224728566697775	0.0881395944240541	-2.54968913989517	0.0107819008873016	0.100171233656375
**Ech1**	8908.6136405252	0.202099238894963	0.0792957642314432	2.54867634928253	0.0108132600762433	0.100403381862914
**Khdrbs1**	11152.2296124455	-0.235814698513867	0.0925923576288758	-2.54680520674339	0.0108714098750578	0.100859013050365
**Kctd13**	2006.34504628376	0.189148444921952	0.074286842016821	2.54619041255143	0.0108905765051526	0.100859013050365
**Gm12474**	107.317570682768	-0.595175634024143	0.233754085235329	-2.54616142184192	0.010891481051408	0.100859013050365
**Ighv1-76**	46.2650464864563	2.97898592029517	1.16982943107539	2.54651305665704	0.0108805141125614	0.100859013050365
**Gm17690**	222.666423271341	-0.625359231492296	0.24561745806332	-2.54606995945328	0.0108943352295311	0.100859013050365
**Anxa4**	364.826915569823	0.626655195742022	0.246243613590907	2.54485867309885	0.0109321974148949	0.101150108435995
**Gm20234**	154.52924473519	-0.57651105793505	0.226602683469172	-2.54414929739118	0.0109544252157225	0.101296289978955
**Aqp9**	689.781058034875	0.64009015901273	0.251662977787423	2.54344188660681	0.0109766314323117	0.101442100574156
**Tspan4**	574.281178632184	0.408298566168629	0.160594570787689	2.54241824095295	0.0110088353443149	0.101568119709683
**Pogz**	2227.40259638378	-0.366400905180948	0.144128099823771	-2.54218924435245	0.0110160510619946	0.101568119709683
**Ccser2**	7476.46937665443	-0.16403270457808	0.0645227250599608	-2.54224700561926	0.0110142305995174	0.101568119709683
**Noc2l**	20170.1755629679	0.251589217594013	0.0989638429278061	2.54223371032133	0.0110146496038839	0.101568119709683
**Ranbp10**	5416.64647602049	-0.364629850934738	0.143555948683243	-2.53998426592057	0.0110857455902647	0.101988853464369
**1810009A15Rik**	1528.1541518797	0.462776290156287	0.182200656416923	2.53992658016189	0.0110875741565313	0.101988853464369
**Pak6**	199.989907331005	0.561138457511822	0.22087525091548	2.54052210551442	0.011068709637961	0.101988853464369
**Gm45501**	48.9944975043409	-0.975829071719967	0.384142588061118	-2.54027827699414	0.0110764299693373	0.101988853464369
**Gm42869**	47.3511776642317	-1.56442683121833	0.616303559408375	-2.53840304398065	0.0111359654325378	0.10237421663973
**Cdkn1b**	2578.33714932032	-0.517666912823234	0.204060806322707	-2.53682675351474	0.0111862296822217	0.102746987046948
**Sh3pxd2b**	24.1696369793646	1.972234393998	0.777473397925346	2.53672267020431	0.0111895557385647	0.102746987046948
**Gm42809**	461.427638883147	-0.479740841836879	0.189216067282587	-2.53541281523627	0.0112314882282442	0.103011967411201
**Gm46339**	42.7275507744098	-1.04510729453701	0.412197169687314	-2.53545480511136	0.0112301418410336	0.103011967411201
**Ssx2ip**	514.455129242759	0.679856780174697	0.268244389399168	2.53446784738904	0.0112618261511322	0.103230130694235
**Snx17**	11684.1473705322	0.140368304298901	0.0553947639450098	2.53396339838624	0.0112780511151232	0.10331875082616
**Adam8**	461.662691270194	0.880939485823345	0.34776153965056	2.53317111118307	0.0113035759329807	0.103492414861114
**Polr3g**	934.589857097624	-0.333490551627652	0.131725262239463	-2.5317129452466	0.0113506872161901	0.103863402128634
**Cdc45**	450.134556625573	0.46298349561713	0.183084836206885	2.52879214471897	0.0114455787611944	0.104670913836413
**Nhp2**	5397.21489456097	0.259519385361507	0.102637025191336	2.52851624331193	0.0114545785960614	0.104692456460638
**Trnau1ap**	3463.39795179124	0.223570244661214	0.0884498518762454	2.52764973506142	0.0114828847333559	0.104833295251286
**Rsbn1**	2370.84244793923	-0.240633555905382	0.0952009864003435	-2.52763721263831	0.0114832942567569	0.104833295251286
**Gk**	826.574243318053	-0.355339863140822	0.140612812226643	-2.52708026753692	0.0115015212618429	0.104902013962041
**Snx20**	3365.65957724818	0.660880519882875	0.261527665441768	2.52700041797309	0.0115041365822432	0.104902013962041
**Pcyox1l**	135.173171036568	0.621393435063553	0.245983090197958	2.52616321944521	0.0115315892182698	0.105091527653139
**Ly6d**	930.179710015235	0.656091225310809	0.259820234613534	2.52517370822447	0.0115641113026183	0.105326995257431
**Gm16534**	961.899012702194	-0.410793880303829	0.162697502993311	-2.52489357701278	0.0115733330941072	0.105350092180154
**Iglc3**	306.249002832664	0.538978276067044	0.213488467319708	2.52462478574967	0.0115821877136611	0.105369822057828
**Aven**	2363.69931790666	0.588603428342556	0.233179160318164	2.52425400082679	0.011594412110854	0.105420168280858

**Gm43597**	186.797205907098	-0.589704115888537	0.233639645876022	-2.52398994048063	0.0116031248869265	0.105438546045733
**Mtdh**	6204.43506329593	-0.18252873769885	0.0723588827844954	-2.52254776020341	0.0116508127821036	0.105738099147489
**1190007I07Rik**	316.284966337493	-0.345041124580498	0.136791643293139	-2.52238452784055	0.0116562212489172	0.105738099147489
**Gm43460**	322.707469115251	-0.499926484632505	0.198176221368018	-2.52263607198428	0.011647887627957	0.105738099147489
**Lap3**	2705.79234482132	0.205923750061181	0.0816517661673486	2.52197545413937	0.0116697850976157	0.105764062349942
**Gm45760**	25.1901762851919	-1.38461611338706	0.549038320762724	-2.52189339254781	0.0116725077379291	0.105764062349942
**Phf10**	1109.64773283116	0.546471471322351	0.216784706955701	2.52080268482232	0.0117087488077354	0.105916015274196
**Ubr1**	7804.07876400676	-0.148791450244814	0.0590306433406315	-2.52057985182755	0.01171616517249	0.105916015274196
**Fan1**	422.275841482779	-0.578390658727098	0.229459376394583	-2.52066691636295	0.011713266980309	0.105916015274196
**Timm29**	4738.16666148046	0.464083048782623	0.18408695438791	2.52099911330329	0.011702214696187	0.105916015274196
**Prdm15**	2128.72960000349	0.340346815758675	0.135130745840951	2.51864824426606	0.0117806280729659	0.106437704441356
**Mbnl1**	45053.4768570377	-0.215782988015169	0.0857228103620423	-2.51721784556327	0.0118285668247102	0.10680958593522
**Il10rb**	11317.9094129489	0.184557624307628	0.0733600138512802	2.51577957280344	0.0118769438216366	0.106940002170016
**Slc23a1**	55.6654694833857	-0.72113993219822	0.286614780716964	-2.51605981517875	0.0118675039954616	0.106940002170016
**Ifng**	139.027547704945	1.35383565476274	0.53809638318379	2.51597241139665	0.0118704474345717	0.106940002170016
**Galnt4**	489.305407964502	-0.357840621058673	0.142231826180141	-2.51589697375788	0.0118729884182939	0.106940002170016
**Gm43860**	63.7835736494024	-1.03966361877509	0.413237650299713	-2.51589761489799	0.0118729668205891	0.106940002170016
**Rars**	6715.06208660219	0.151089296211019	0.0600996292259304	2.51398050465559	0.0119377032746349	0.107425694173857
**Paxbp1**	2711.32610205483	0.494937254406756	0.196951332917098	2.51299266207601	0.011971182490148	0.107594967719912
**2810001G20Rik**	823.701520870244	-0.289153672930408	0.115071324099857	-2.51282128881637	0.0119769990087406	0.107594967719912
**Gm7890**	190.146174412189	-0.519717067879023	0.206796995806508	-2.51317513512286	0.0119649919905888	0.107594967719912
**Anxa5**	11296.8381848438	0.202754692132298	0.0807121990297852	2.51206997913011	0.0120025285221731	0.107762872891101
**CT010467**	293784.739052695	-0.981660897969241	0.390839217237901	-2.51167450622467	0.0120159860666705	0.107822262216701
**Txnip**	34483.8786601098	-0.404619711172414	0.161122676420276	-2.51125242059034	0.0120303639700588	0.107889837835069
**Adamts6**	946.708110521489	-0.60536521599249	0.24129411330272	-2.50882712266178	0.0121132751382397	0.108571602021185
**Muc1**	70.4620495582742	3.36845180979477	1.34276530185184	2.50859312878376	0.0121213011710891	0.108581775186384
**Wrap73**	3183.53897197502	0.170352203110612	0.067913253050881	2.50837937306557	0.0121286371501535	0.108585758849414
**Zer1**	1938.81629880045	-0.298123049098048	0.118875244693238	-2.50786486174951	0.0121463110325158	0.108682239034271
**Spout1**	5455.97948545085	0.206107957851019	0.0821995273511274	2.50741049848861	0.0121619377535808	0.108760302601119
**Gm44091**	43.6135738200331	-1.24052489353402	0.49508176761835	-2.50569698719004	0.0122210302171276	0.109226757306455
**Tarbp1**	605.036432784658	0.831442701024036	0.332367404037655	2.50157714301562	0.0123641502567569	0.110443262809364
**Rundc1**	5417.43516630218	0.160836589946998	0.0643079221758405	2.50103851135502	0.0123829711999518	0.11054871229328
**Cdk9**	9096.68090357524	0.291333701894129	0.116503262545546	2.50064844132786	0.0123966169198775	0.110607866821353
**Timm10**	1437.5426704656	0.236534045971037	0.094608432869391	2.50013702581436	0.0124145278149561	0.110704988557025
**Chd3**	6505.00961214596	-0.411699156947246	0.164738343082961	-2.49910949231726	0.0124505835957593	0.110900986839106
**Wdfy4**	351.762995489308	0.559431772922371	0.223839716597251	2.49925161372922	0.012445591086507	0.110900986839106
**Flot2**	4777.45805703296	0.189840893360644	0.0759919256816855	2.49817189994432	0.0124835642600562	0.111069182408642
**Marcks**	98.8460571228329	0.769048228610711	0.307820694851156	2.49836427983693	0.012476790820913	0.111069182408642
**Fbxo25**	1433.68170970316	-0.239950544506256	0.0960811866060217	-2.49737282585993	0.0125117334566386	0.111256988756351
**Nipsnap3b**	3887.94144561643	0.171462069331669	0.0686870716202934	2.49627863420241	0.0125503975250036	0.111474979594973
**Gm38372**	47.3666291900741	-0.990842933197507	0.396903767602761	-2.49643116058597	0.0125450015551386	0.111474979594973
**Brwd3**	343.970755151368	-0.494340126924781	0.198060127469766	-2.49590936469655	0.0125634697898128	0.111498040682445
**Snhg6**	2001.20340207512	0.298896202260294	0.119759409508847	2.49580557791757	0.01256714604633	0.111498040682445
**Psmd9**	2227.58656997189	0.170399487278249	0.0682836476065374	2.49546550676556	0.0125791984625832	0.111542166671313
**Adgrg5**	34037.7167777037	0.253291617693754	0.101516201689726	2.4950856462096	0.0125926731484585	0.111598847469213
**Tgfbr2**	21039.5387553558	-0.23788342079881	0.095396001341154	-2.4936414257878	0.012644020293191	0.111927993123489
**St8sia4**	3015.4428824206	-0.22603156240211	0.0906376948565077	-2.49379204490968	0.0126386566075799	0.111927993123489
**Fam13b**	3306.34753720093	-0.257402361097885	0.103244491594032	-2.49313408515796	0.012662101987918	0.112025121293444
**Man2a1**	1696.76702333899	-0.344731010209952	0.138289042841331	-2.49282953390232	0.0126729672302393	0.112058330329339
**Gm49167**	218.157610826101	-0.845109690002485	0.339213832652191	-2.49137743999081	0.0127248860365377	0.112454306942078
**Prkch**	56418.9752964533	-0.174739531080587	0.0701532024742529	-2.4908275733345	0.0127445952983761	0.112565352083247
**Cmah**	24575.3143707143	-0.311716196877529	0.125201974260231	-2.48970672163388	0.0127848544685767	0.112671078617891
**Timp2**	4338.60447626201	1.78076726157985	0.715236101831343	2.48976143265174	0.0127828867281891	0.112671078617891
**Phka2**	1139.52887610511	-0.284647860566486	0.114330275912415	-2.48969801126471	0.0127851677710725	0.112671078617891
**Virma**	5317.496084425	-0.283803264366146	0.113965140593439	-2.49026380249545	0.0127648309839763	0.112671078617891
**Trav17**	359.878581582731	0.478236865407092	0.192103955005659	2.48946912828008	0.0127934028781978	0.112680631163646

**9530068E07Rik**	8432.95856043721	0.125420949263491	0.0503928394001263	2.48886450449101	0.0128151795530258	0.112809376657557
**Vav1**	29640.0505448555	0.158565014254066	0.0637374464782556	2.48778423070591	0.0128541693350854	0.113089417204322
**Gm44673**	79.9911429384173	-0.836326496716979	0.33621941729906	-2.48744258566448	0.0128665219867019	0.113134925750258
**Pcca**	2900.15462661121	-0.228472056599701	0.0918827117256473	-2.48656196915363	0.0128984103416235	0.113288880575787
**Gm48865**	35.0095059366691	1.11415623583752	0.448069493963221	2.48657016567384	0.0128981132120785	0.113288880575787
**Ndufs8**	6328.04774666111	0.230006898495994	0.0925288368625619	2.48578612133249	0.012926562794307	0.113472896908019
**Ubr5**	2830.06562413526	-0.295476498308344	0.11891840107833	-2.48469955556936	0.012966081346773	0.113756427495045
**Clptm1**	19717.3729237974	0.14609316014101	0.0588293333798745	2.48333869768084	0.0130157266662571	0.114120829471222
**Rab31**	238.33116260401	0.51432384624422	0.207124372897513	2.48316428940361	0.0130221013764839	0.114120829471222
**Gm43213**	18.8784486743955	-1.74699645437928	0.70395616695823	-2.48168357119165	0.0130763337138829	0.114532401517317
**C1qtnf12**	78.9029388370785	0.731675076007256	0.294880206213015	2.48126208742106	0.0130918073279798	0.114584831158367
**Kcnj8**	732.369537128097	1.65217221831904	0.665949659005956	2.48092659253732	0.0131041356696946	0.114584831158367
**Plekhf1**	183.215581419699	1.66868613419757	0.672586450688946	2.48099873627888	0.013101483756561	0.114584831158367
**Pheta1**	609.438711091008	-0.35231570750117	0.142050219074601	-2.48021938858217	0.0131301567418686	0.114748685403008
**1700062C10Rik**	253.964700039715	-0.800067209391596	0.322703165106056	-2.47926669429677	0.0131652826902217	0.114991884340257
**Gm43808**	128.038377024189	-0.854741314117668	0.34478991899419	-2.47902060655106	0.0131743694665218	0.115007501209964
**Capns1**	1090.17617170245	-0.303490837117637	0.122453450552239	-2.47841800903902	0.0131966437659038	0.115138159368409
**Fbxo32**	4073.94620766648	-0.180185527978538	0.0727242651401896	-2.47765347138533	0.0132249519084156	0.115321287892033
**Cxcr3**	15743.3797967374	0.308951153797967	0.124754987660134	2.47646334300984	0.0132691250184181	0.115642479488503
**Gpr4**	97.9380496453481	1.0380470946319	0.419241483099921	2.47601236155462	0.0132858978089082	0.115724649958522
**Gm43065**	1210.84956488038	-1.06086090896585	0.428504753389158	-2.47572728324533	0.0132965100365558	0.115753098699453
**Glrx2**	1340.71916805754	0.213236349222039	0.0861384663704307	2.47550668368108	0.01330472714244	0.115760676744024
**Prmt5**	11428.1082981038	0.144730801113892	0.058494493856618	2.47426367118684	0.0133511120342978	0.116100150289421
**1700102P08Rik**	72.2835614591431	-0.889140157120385	0.359444585519058	-2.47365016178062	0.013374058706214	0.116171468045102
**Kcnc1**	590.301606539868	0.675415814007529	0.273026780689992	2.4738079257303	0.013368154642445	0.116171468045102
**Slc25a25**	2339.09943794103	0.349114992916094	0.141150161909736	2.47335878466331	0.0133849690885076	0.116202180676372
**Cdc25b**	6146.01917570036	-0.281847924313695	0.11396278962439	-2.47315746870218	0.0133925117974612	0.116203638982707
**Gm47260**	244.964854098896	-0.814525571399725	0.329396575053985	-2.47278093667559	0.0134066294144093	0.116262113199145
**Rpn2**	23344.5003943009	0.12874035881371	0.052072997935418	2.4723054926351	0.013424474406448	0.116313996198595
**Gm10390**	66.9532526199296	-0.975781122223871	0.394697020315108	-2.47222824597169	0.0134273757114453	0.116313996198595
**Rnf220**	12908.6701733297	1.6575466233717	0.670654167257685	2.47153705187494	0.0134533609209494	0.116347205286169
**Metrnl**	234.790249781489	1.96883247540422	0.796547442829457	2.47170773458342	0.0134469400334162	0.116347205286169
**B230354K17Rik**	572.696646752085	-0.408579112719638	0.165296094828097	-2.47180136436101	0.0134434189390446	0.116347205286169
**Zeb1**	4671.94632781089	-0.291133340639857	0.117817003097329	-2.4710638786097	0.0134711753247606	0.116437361268378
**Zfp426**	1887.89911188597	-0.226273972819727	0.0916020967546802	-2.47018333462073	0.013504382241682	0.116660389792864
**Ddx5**	227877.094843529	0.21356713502436	0.0864947951283316	2.46913279241245	0.0135440947061636	0.116939342621929
**Ctdspl2**	3692.27618401541	-0.164085970910538	0.0664751176356209	-2.46838180580536	0.0135725466158977	0.117056714300328
**Tuba3a**	49.003763273924	-1.07487783992028	0.435441651632021	-2.46847731697616	0.0135689251473785	0.117056714300328
**Ppp1r3e**	79.231120955224	0.992152864908152	0.402009097531163	2.46798610031765	0.0135875595617268	0.117122087535082
**Snx18**	1225.2613403681	1.3800998708521	0.55924733076763	2.46778088141745	0.0135953512799964	0.117125177757738
**Upb1**	52.5378211514309	1.16906049667032	0.474038628828608	2.46617137417509	0.0136565977943477	0.117460159085992
**Gm10642**	42.2316207911265	2.17640553944027	0.882398835092931	2.46646465621304	0.01364541941919	0.117460159085992
**Trav14n-3**	494.12552100744	0.367028215873825	0.148820289062785	2.46625119589024	0.0136535546077644	0.117460159085992
**Dnajc11**	6246.58064360852	0.197977971100057	0.0802873462908296	2.46586766466175	0.0136681821348357	0.11749566061026
**Gm43011**	60.2323098398752	-1.19564962858261	0.484981612935327	-2.46535043121739	0.0136879308616547	0.117601268586201
**1700008J07Rik**	323.504638359616	-0.635012516306238	0.257694242631121	-2.464209172167	0.0137315949745488	0.117912120988537
**Itch**	6901.23254467437	-0.21425731345776	0.0869611802542573	-2.46382711034181	0.013746239966337	0.117973585593449
**St3gal1**	2759.3613669616	0.228066870636637	0.0926414741881198	2.4618225544805	0.0138233038100571	0.11850587493747
**Apopt1**	1950.8590438417	0.183990752661821	0.0747320114340861	2.46200723265827	0.0138161880580897	0.11850587493747
**Phb2**	12420.3912403127	0.138145548678443	0.0561203796059579	2.46159326876287	0.0138321428179272	0.118512874442279
**Ahcyl2**	8211.84849743339	0.233511888370169	0.0948691070743775	2.46141125990672	0.0138391628465948	0.118512874442279
**Tnk2**	376.879573102906	-0.414462589577351	0.1684728400805	-2.4601151697764	0.0138892436926609	0.118877138981672
**Hdhd2**	2376.29548173549	0.156385050306287	0.0635750908473948	2.45984784641004	0.0138995929337041	0.11890113238674
**Ten1**	2133.7647526893	0.215603815743356	0.0876811370668787	2.45895323618927	0.0139342766886562	0.119004009638567
**Slc35d2**	1603.77190524595	0.389204617029463	0.158276962915924	2.45900988911574	0.0139320780087662	0.119004009638567
**9430015G10Rik**	3408.99158154791	0.476217713552982	0.193662211528283	2.45901205916692	0.013931993795944	0.119004009638567

**Ubox5**	2040.79519152001	0.306596848434868	0.124699451395325	2.45868642567549	0.0139446356285221	0.119027965113014
**Chid1**	1666.51317532972	0.225447014252882	0.0917023901748612	2.45846388325312	0.0139532810555035	0.11903727644373
**Dera**	3149.60398192213	0.227419397242579	0.0925208659699614	2.45803359986292	0.0139700103155881	0.119115504622685
**Pou2f2**	903.018042455658	-0.466105716601603	0.189757805329337	-2.45631907363518	0.0140368464292968	0.119620653967782
**Abi3**	4452.10198494485	0.414828202419985	0.168922063301919	2.45573724539791	0.0140595914633314	0.119749720371737
**Smchd1**	11186.4664498812	-0.27363585962204	0.111450879014705	-2.45521490760011	0.0140800385788932	0.119859085838801
**Ginm1**	2800.490578128	0.218492065665448	0.0890251859779928	2.4542724990146	0.0141169959247142	0.119979236669753
**Ksr2**	148.012362249348	-0.734042879955437	0.299057559809233	-2.45452039541711	0.0141072661705777	0.119979236669753
**Tmem217**	84.6121544602611	-0.672731397484946	0.274103923260049	-2.45429320924644	0.0141161828364803	0.119979236669753
**Susd3**	2931.39894580716	0.400660232593107	0.163286881293986	2.45371967066814	0.0141387153681057	0.120099050164551
**Kcnk1**	24.3925116300925	7.91647207264984	3.22738976558394	2.45290239098763	0.0141708785584788	0.120307399485965
**Kmt2a**	8539.90637254234	-0.44638954322105	0.182008288265074	-2.45257810771197	0.0141836582683298	0.120351051876183
**Gm27008**	182.775646420832	-0.488116671970031	0.199089952007948	-2.45173936226849	0.0142167596432291	0.120566997684802
**Ulk2**	2623.03229205441	1.21216312684359	0.494626537682787	2.4506633479924	0.0142593247086225	0.12079568783668
**Nt5c3b**	9102.99406050272	0.203594326921995	0.0830901215789305	2.45028317510154	0.0142743904773686	0.12079568783668
**Mob1a**	12756.4346732813	0.149355405264844	0.0609455677469477	2.45063604764473	0.0142604061190211	0.12079568783668
**Ifi209**	21483.1008623118	-0.179654612414659	0.0733184262980628	-2.45033372217109	0.0142723865521359	0.12079568783668
**mt-Te**	89.71184040116	-0.780968309484703	0.318752178686841	-2.45007991067559	0.0142824513480988	0.120799026243689
**Sirt2**	5843.30045497871	0.195532349272959	0.0798252454640184	2.44950514259422	0.0143052666997429	0.12087281936528
**Pgap1**	852.092420378762	-0.483490469717909	0.197385455586321	-2.4494736366554	0.0143065182532364	0.12087281936528
**Polr3d**	2199.13583749944	0.32625041575637	0.133248828472556	2.44842989988136	0.0143480346767826	0.12106410001309
**Lig1**	985.31992509237	0.244904040543637	0.10002642269556	2.44839347388265	0.0143494855005685	0.12106410001309
**Fbxw10**	228.951944970467	-0.48482102605154	0.198021504402717	-2.44832513273689	0.0143522078329918	0.12106410001309
**Crtam**	5127.57591414189	0.127286910749751	0.0519953786595897	2.44804276901396	0.014363460468547	0.121094192938948
**Auts2**	236.028717230001	-0.618634110457068	0.25274150168439	-2.44769500194545	0.014377330223192	0.121146306057132
**Morf4l2**	4714.16409483607	0.180864729568746	0.073931105806087	2.44639556782951	0.0144292592459067	0.121518887192812
**Mid1-ps1**	92.9331976410146	1.22757318722037	0.501876513324446	2.44596659662132	0.0144464384149026	0.121598573773301
**Tbc1d12**	122.954913882888	0.988476829366823	0.404237388069988	2.44528798804647	0.0144736517415824	0.121762589691465
**Pfn1**	106361.03432386	0.116833541812791	0.047800415578083	2.4441951058342	0.0145175731557853	0.122055910643116
**Tnks2**	6155.10619904209	0.940960566178945	0.385002873996596	2.44403517410434	0.0145240104370022	0.122055910643116
**Fam161a**	62.2302329213529	-0.846021028619646	0.346236229178774	-2.44347921252	0.0145464075776353	0.122113875439957
**Gm49125**	110.024621797129	-0.62142532362198	0.254306024446089	-2.44361227767028	0.0145410442232001	0.122113875439957
**Zbtb26**	282.957353597911	-0.673595899457869	0.275755139077719	-2.44273198936837	0.0145765577350805	0.122234233298667
**Ep300**	4978.72763550662	-0.332444588690954	0.136090637373306	-2.44281748625393	0.0145731051805936	0.122234233298667
**Gm17530**	17.3053212419104	-1.25608446531643	0.514251842803173	-2.44254732947489	0.0145840171734146	0.122234233298667
**Ngfr**	41.7441585940306	1.39007584493653	0.569187416425722	2.44221113261019	0.01459760667309	0.122283087904242
**Fbxo9**	5069.49904958238	0.145737060751286	0.0596858794182148	2.44173432932297	0.014616898804138	0.122314643896337
**Rhd**	37.6501977769375	-1.68853486860764	0.691516231118096	-2.44178631335593	0.0146147943659708	0.122314643896337
**Ehbp1l1**	3976.04284076244	0.221244414939558	0.090632037467644	2.44112811673845	0.0146414594706006	0.122455136347268
**Ptpn4**	3465.65103378142	0.294154406161382	0.12052632827637	2.44058215634744	0.0146636101515646	0.12251034207752
**Gmfg-ps**	52.5359827293739	0.714636239271603	0.292811832223996	2.44059891242687	0.014662929885501	0.12251034207752
**Sh3bgrl3**	37581.2571779428	0.112870485683893	0.0462617921358167	2.43982086453815	0.0146945465613553	0.122582814832668
**Slc5a11**	31.1306806619828	1.30381004812309	0.53443529520594	2.43960318455516	0.0147034029341717	0.122582814832668
**Gm37248**	642.497400708015	-0.562275265257783	0.230432221230784	-2.44008959447841	0.014683619691971	0.122582814832668
**Gm45269**	29.3275063377745	-1.51598136505354	0.621383934374386	-2.43968548459471	0.0147000539803754	0.122582814832668
**Ralgds**	950.937511431847	0.776085336518693	0.318249210912876	2.43860883202992	0.0147439183114666	0.12283933226497
**1810026B05Rik**	2973.21944296823	0.577576123456301	0.236860477719655	2.43846558538108	0.0147497630669114	0.12283933226497
**Ppm1k**	1707.28866136758	-0.303369024229976	0.124483583442096	-2.43702033506363	0.014808846556599	0.123201159024462
**Gm43081**	43.8654559189646	-1.189712183847	0.488145806262743	-2.43720660627093	0.0148012198864418	0.123201159024462
**mt-Rnr2**	154931.820385656	-0.940374553489769	0.385916438338611	-2.43673101238736	0.0148206994230402	0.123234702273796
**Itprid2**	757.956888600345	0.329240198942891	0.135159161578421	2.43594437179059	0.0148529685053818	0.123355300341899
**Ncoa6**	1589.54582273781	0.476641174619959	0.195681164422826	2.43580508132115	0.0148586888398124	0.123355300341899
**Irgm1**	13563.3969020816	-0.294179547528856	0.120772063601865	-2.43582446764048	0.0148578925726609	0.123355300341899
**Hagh**	2376.3764989222	0.171147268786379	0.0702696424444139	2.43557904712214	0.0148679756678194	0.123367399998857
**Rnf44**	4848.9456188382	-0.239315589376019	0.0982722182626069	-2.43523137675096	0.0148822700159092	0.123421015074043
**Lims1**	9362.59652849596	-0.171466572700539	0.0704268495977313	-2.43467617364588	0.014905122110243	0.123545507149447

**Phf11a**	855.405381847923	0.379439209801459	0.155871073428576	2.43431447192366	0.01492002633683	0.123604024705274
**Wipf2**	1009.7320616701	-0.322386866380363	0.13249460899664	-2.43320742497937	0.0149657247721952	0.123852376699307
**Gm17509**	154.061860825692	-0.754256395618385	0.309975981606444	-2.43327367401006	0.0149629865751367	0.123852376699307
**Huwe1**	8501.74472693524	-0.348803944624069	0.143400271366263	-2.43237994810469	0.0149999631573592	0.12389950003486
**Pgam5**	6527.28168908943	0.140885524877498	0.0579140028099477	2.43266771491917	0.0149880484559625	0.12389950003486
**Sbno2**	9494.90288428196	0.417038469552366	0.171456680840362	2.43232557348207	0.015002215422008	0.12389950003486
**Pdcd5-ps**	632.031745187386	0.317054290031875	0.130351116615894	2.43230973591227	0.01500287148991	0.12389950003486
**Thy1**	4131.62753746107	-0.218670693606011	0.0899248414386814	-2.43170507845843	0.0150279382034843	0.12404149935689
**Klf6**	19880.2943603278	0.261298077814581	0.107469139084917	2.43137778937742	0.0150415217007148	0.124088616459771
**Adam15**	284.493797965918	0.48644396532915	0.200084989611449	2.43118669858139	0.0150494575522883	0.124089117033703
**Lsm14b**	53.9115199743969	1.69386522724404	0.696851181988639	2.43074170070312	0.0150679522620456	0.124176633782978
**Plod3**	815.973083528012	0.314507100745363	0.129421971024771	2.43009048815342	0.0150950536142689	0.124334950235251
**Nudc**	8192.71587964001	0.134878288388245	0.0555195431966747	2.42938397224283	0.0151245050590481	0.124512448388412
**Gm26782**	639.112310049774	-0.362937660675566	0.149413503268823	-2.42908206243296	0.015137105735128	0.124551109696299
**mt-Rnr1**	41756.8089608313	-0.856346716094061	0.352594943566701	-2.42869823211762	0.0151531388389139	0.124552951843905
**Gm7967**	131.63208111419	-0.690930552135412	0.284470075974657	-2.42883385807148	0.0151474718543263	0.124552951843905
**Adss**	6344.57983329746	0.141188633747636	0.0581445230860735	2.42823616488571	0.0151724598396997	0.124581787229885
**Srrm1**	8176.55669215654	-0.179705224851078	0.0740041603956419	-2.42831246095268	0.0151692680813578	0.124581787229885
**Nif3l1**	2666.83661968133	0.280016882707018	0.115343796203141	2.42767181178827	0.015196087274832	0.124645886095538
**Stk24**	7852.20057649008	0.555183931003616	0.228673112001127	2.42784963280195	0.0151886390547053	0.124645886095538
**Zdhhc5**	4822.90997598141	0.328529892323731	0.135351258240136	2.42723929275089	0.0152142172163462	0.124729667366268
**Psmd12**	6415.48022217924	0.186350530022591	0.076794249631697	2.42662088523975	0.015240172145511	0.124872728473079
**Gm47664**	311.764816953357	-0.621992717023447	0.256338985052719	-2.42644604719617	0.0152475172673862	0.124872728473079
**Cd244a**	429.707664011595	0.935678372427072	0.385733774123376	2.42571025716768	0.0152784627164874	0.124996228984264
**Kdm8**	1081.21441929744	0.23012892357854	0.0948648080500793	2.42586190083318	0.0152720804513966	0.124996228984264
**Ctsb**	30577.5093173526	0.179177110153208	0.0738958135547132	2.42472613175229	0.0153199389485828	0.125205538388392
**Tatdn2**	160.885412628768	1.28569477435276	0.530239234917447	2.42474469953723	0.0153191554877305	0.125205538388392
**Slamf8**	155.911701058475	0.612376123680858	0.252714133551768	2.42319697388597	0.0153845823973535	0.125668670210005
**Wdr75**	3483.51144707839	0.17165479589359	0.0708972431338146	2.4211773026153	0.0154703296672898	0.126303618946884
**Pgrmc1**	3937.98364845326	0.200419697806747	0.0828186341352192	2.41998313422458	0.0155212269712076	0.126388652939016
**Gclm**	1027.54200241367	1.30841219126077	0.540692533002666	2.41988211672661	0.0155255392454981	0.126388652939016
**Tes**	17293.4119698787	-0.133647750817288	0.0552247202209116	-2.42007112544286	0.0155174716267593	0.126388652939016
**Thsd1**	34.057675598854	1.2012442733852	0.496460653007383	2.41961626990676	0.0155368928566906	0.126388652939016
**Pomt1**	961.499561169152	0.278387265012368	0.11504001496509	2.41991680109609	0.0155240585067902	0.126388652939016
**Man2c1os**	289.908587563292	-0.484051115374822	0.199996175859693	-2.4203018547435	0.0155076282137705	0.126388652939016
**Gm43457**	90.3663093169689	-0.687453924866855	0.284114490948775	-2.41963696596806	0.0155360087207336	0.126388652939016
**Dyrk1b**	618.005928361662	-0.445210319244579	0.184043671250684	-2.41904715451021	0.0155612228277052	0.126456002112507
**Pkm**	71336.6486281088	0.111600665112039	0.0461310529163212	2.41920914561554	0.0155542942151977	0.126456002112507
**Sdr39u1**	2839.5358391936	-0.228294783460588	0.0943867906747566	-2.41871539257288	0.0155754212807382	0.126476732807123
**Phf12**	2115.76478667362	-0.222962322515808	0.0921931887778087	-2.41842510788038	0.0155878539767363	0.126476732807123
**Cplane1**	1272.47034312168	-0.464129127141176	0.191907919736586	-2.41849907902834	0.0155846850139916	0.126476732807123
**Fam3c**	5607.2247527636	-0.156947678494077	0.0649201207939515	-2.41755062336081	0.0156253603448491	0.126652021192233
**Rbm5**	27510.7196771593	0.177875214484198	0.0735767471674071	2.4175465936199	0.0156255333628039	0.126652021192233
**Ift20**	1446.30967930672	-0.231658093025059	0.0958563131752145	-2.41672233524791	0.0156609585586438	0.126808696818371
**Btf3l4**	1990.06508658947	-0.199493811548533	0.0825421460742512	-2.41687211971782	0.0156545158333942	0.126808696818371
**Wdr83**	3487.24269482717	0.169631508152576	0.0702011734439934	2.41636285877625	0.0156764303482373	0.126868779145955
**Slc35f5**	38.9909713518042	1.3358939615775	0.553038597930948	2.41555285033523	0.0157113422316688	0.127086046994048
**Gbp10**	2821.48588975264	-0.391595168566487	0.162137408475634	-2.41520554847979	0.0157263321016051	0.127142029207282
**Prkcq**	38115.790130789	-0.125770829494509	0.0520861270325061	-2.41467040573045	0.015749453985432	0.127256806708175
**Gars**	11728.3101172427	0.165623713642275	0.0685953568972336	2.41450327156114	0.0157566814677699	0.127256806708175
**Syngr1**	32.6093881102235	-1.12109496194321	0.464478498986093	-2.41366384965169	0.0157930252276725	0.127484988992027
**Uqcc1**	5414.98249233217	0.184425190386398	0.0764373107919511	2.41276398234849	0.0158320679081701	0.127604037866515
**Carm1**	957.138821942414	0.632684328921416	0.262218867431387	2.41281008921666	0.0158300654010998	0.127604037866515
**Purb**	2122.127357769	-0.354928637528382	0.147087701978611	-2.4130408780198	0.0158200451617405	0.127604037866515
**Gm36371**	123.15689228118	-0.985717210055036	0.408617162313548	-2.41232454474992	0.0158511646836665	0.127692639018677
**Ndor1.1**	355.604198184261	2.67018730490906	1.10704879095835	2.41198701151874	0.0158658467223648	0.127745603885694

**Sars**	12960.4529811345	0.240692360029179	0.0998096179963571	2.41151469027728	0.015886411853106	0.127845858819914
**Kdm5a**	14746.1938522677	-0.171818138239038	0.0712778993101784	-2.41053874906359	0.0159289791495614	0.128057614520224
**Sys1**	6381.34719149732	0.162560644503071	0.0674370528631749	2.41055380686483	0.0159283216175435	0.128057614520224
**Clca3a1**	17.6607758735713	2.61266633975971	1.08394220591752	2.41033730903409	0.0159377777672324	0.128063010850729
**Mtx3**	208.853947103427	-0.555366669225337	0.230509296732426	-2.40930269233351	0.0159830357443009	0.12810004792622
**Phf14**	2604.59915185693	-0.155747360157589	0.0646434959961785	-2.40932761691595	0.0159819441236219	0.12810004792622
**Jak3**	18766.8224002637	0.398904301162247	0.165533862204271	2.40980483298331	0.0159610561544298	0.12810004792622
**Slamf7**	2791.96344576482	0.585617761687461	0.243038010507279	2.4095727267728	0.0159712125511025	0.12810004792622
**9930111J21Rik1**	17306.749378955	-0.330461076673603	0.13712416971418	-2.40994040191756	0.0159551266188928	0.12810004792622
**Tbrg1**	12128.0606405618	0.138275965001969	0.0574405528959207	2.40728819676437	0.0160714815580081	0.128678015706064
**Gm5499**	81.2581967331566	0.544537934007851	0.226199210435073	2.40733790785779	0.0160692938406507	0.128678015706064
**Apmap**	5381.23815244624	0.225311566797226	0.0936139687793153	2.40681566795201	0.0160922899821813	0.128713813832097
**Tet2**	3731.45553731417	-0.356751282812716	0.148214687439582	-2.40699008293723	0.0160846066344111	0.128713813832097
**Gm17552**	326.017216494539	-0.677606268652629	0.281685149071097	-2.40554488189082	0.0161483682382891	0.128994161373398
**Rnu3b1.1**	115.373200389673	1.07839404863461	0.448310016179631	2.40546499010746	0.0161518994979827	0.128994161373398
**Gm43737**	571.431356776578	-0.381827692026753	0.158723516627135	-2.40561512333249	0.0161452640894624	0.128994161373398
**Arfrp1**	7607.89082630844	0.156440641489247	0.0650438026988326	2.40515829330586	0.0161654619658476	0.12903707406072
**Nkg7**	87392.3838799869	0.33210259230184	0.138130286388435	2.40427064176162	0.0162047713090064	0.129285357729627
**Atp1a3**	164.085775790819	0.932412344608555	0.387896074643153	2.40376844613943	0.0162270480569898	0.129332117467875
**Utp18**	4430.11795685428	0.171832954210386	0.0714826037427648	2.40384296616753	0.0162237407454528	0.129332117467875
**Btg3**	530.3493045963	1.34446689271675	0.559799650006678	2.4016929855185	0.016319398495193	0.129965541437124
**Gm37531**	529.526949191252	-0.609445767400207	0.253765308149783	-2.40161183513898	0.016323018753828	0.129965541437124
**Dclk2**	126.737999681857	0.61419573932883	0.255841709992325	2.40068650005214	0.0163643494752198	0.129966257399717
**Kras**	7083.60361101543	0.735905562372271	0.306536603078698	2.40071024139111	0.0163632879039901	0.129966257399717
**Fdxacb1**	814.929535743187	0.2286556535445	0.0952457479819502	2.40069145751085	0.0163641278022042	0.129966257399717
**Epm2aip1**	1620.07185730003	-0.344754080266449	0.143573852637242	-2.40123165836828	0.0163399884946051	0.129966257399717
**Ccr4**	36.4323603075163	-1.07260655408542	0.446775246718606	-2.40077435346589	0.0163604214962732	0.129966257399717
**Gm47766**	29.8665937140027	-0.942671438194209	0.392774908246122	-2.40002968214942	0.016393742460955	0.130134105771923
**Cd46**	165.837385585527	-0.694663174726297	0.289511208248425	-2.39943447761172	0.0164204182612204	0.130214660564696
**Zfp961**	2322.12974724258	0.238173033289264	0.0992588457376721	2.39951443641329	0.0164168324621736	0.130214660564696
**Gm28875**	2455.51964067062	0.370710491659398	0.154553591036246	2.39858866541936	0.0164583913557057	0.130450137118639
**Cep135**	1090.60175450926	-0.33333679799778	0.138993312007544	-2.39822185098868	0.0164748836105016	0.130515204148151
**Zfp827**	652.375151197648	-0.560091424125809	0.23376580734832	-2.39595101815404	0.0165773054442818	0.131260603962587
**Got1**	2602.54566941937	0.200561327168532	0.0837277064829978	2.39539974989353	0.0166022536021471	0.131314081757111
**Bet1l**	2332.5670012499	-0.281160104231323	0.117382388704304	-2.39524946914813	0.0166090604139064	0.131314081757111
**Kat6a**	7296.83143261578	-0.381876959971742	0.159420405912544	-2.39540827779121	0.0166018674132574	0.131314081757111
**Zkscan6**	3379.15107497723	0.17841950951248	0.0745210720140729	2.39421555125759	0.0166559571276193	0.13139766632741
**Rab3il1**	176.828739244788	0.763207902590452	0.318843091832887	2.3936786530425	0.0166803557305368	0.13139766632741
**Chek2**	553.626199934867	0.427542889248854	0.178617122576194	2.39362768295898	0.0166826736262794	0.13139766632741
**Sytl4**	31.3385870671562	-1.12224171107451	0.468900678853715	-2.393346313377	0.0166954741706795	0.13139766632741
**Tmem97**	973.684321045568	0.265199205445913	0.110747185717221	2.39463606888456	0.0166368691708631	0.13139766632741
**Ubn2**	3468.10275959028	-0.310163771047855	0.12958705083826	-2.39347812178376	0.0166894766447842	0.13139766632741
**Zfp2**	303.14108711673	-0.418395536744756	0.174828242144539	-2.39318048166868	0.0167030225076983	0.13139766632741
**Zfyve26**	2052.35993441747	-0.347006743316361	0.144936433927277	-2.39419953916125	0.0166566843217704	0.13139766632741
**Zfp948**	373.666582678812	-0.427719461342855	0.178646137989395	-2.39422730408114	0.0166554233873585	0.13139766632741
**4632418H02Rik**	87.7463585541799	-0.678127581157932	0.283310717469657	-2.39358252033145	0.0166847276607154	0.13139766632741
**Atp5o**	8355.71969517955	0.19140050984016	0.0799974652604895	2.3925821801593	0.0167302809425514	0.131546425554781
**Lefty1**	51.0366513410533	0.739675635984164	0.309304451792389	2.39141606820663	0.0167835209102294	0.131833469083991
**Ss18l1**	556.254802070761	-0.288611616755135	0.120684203053629	-2.39146142952018	0.0167814471186343	0.131833469083991
**2810405F17Rik**	149.738447988185	-0.725633925371822	0.303576201527441	-2.39028593717427	0.0168352599957782	0.13217398692251
**Rab4b**	7184.10397052486	0.265452613876954	0.111071174606238	2.38993253486352	0.0168514679916093	0.132235349175193
**Gm14150**	1610.38285325606	0.242843589542112	0.101627256312296	2.38955176351377	0.0168689465338183	0.132306615496951
**Cep192**	2921.42692707898	-0.274475759695269	0.114881982286011	-2.38919762902359	0.0168852166442578	0.132368337643567
**Ttc19**	860.768037750546	0.652151397417504	0.273012604706614	2.38872266765237	0.0169070595649534	0.132473663632506
**Pip5k1b**	19.6753937024401	-1.23325486362575	0.516658134347357	-2.38698431639637	0.016987215945115	0.133035567419074
**Gm26490**	19.4237379120487	-1.26077257129565	0.528265769086034	-2.38662553032151	0.0170038012344959	0.133097002424142

Gm26640	83.6552942637546	-0.608453460371182	0.254961824594115	-2.38644927074791	0.0170119542350842	0.133097002424142
Ctr9	6858.54437508033	-0.154722595052389	0.0648496619854709	-2.38586586753611	0.0170389644008306	0.133242164795974
Gm44036	20.3715422456257	-1.27512512019306	0.534526679423673	-2.38552193796557	0.0170549051372424	0.133300664805322
Zfc3h1	4044.46257903659	-0.379102634195264	0.158966069539012	-2.38480221153248	0.0170883059713486	0.133495506787575
Ghitm	31685.9792746262	0.220073935374651	0.0923001403284812	2.38432936928853	0.017110280708828	0.133593308782111
Gm50256	27.8027089338184	-1.06432287406381	0.446412684488189	-2.38416808268801	0.0171177819655443	0.133593308782111
Asxl1	2560.07326058145	-0.241717886277523	0.101403622428952	-2.38372042820147	0.0171386169866812	0.133653451286205
Rlf	5877.0007764509	-0.243445154988651	0.102131761538895	-2.38363807027787	0.0171424525639031	0.133653451286205
Cpne1	14059.4547791425	0.348726326722942	0.146312901438069	2.38342841468803	0.0171522200530121	0.133663467544664
Eif3m	11695.9205737397	0.113378444701617	0.0475820339789157	2.3827994564473	0.0171815514224939	0.13382585554337
Samsn1	1791.28935011027	0.315699769292159	0.13254117713265	2.38189954338643	0.0172235952419539	0.133967864774397
Mrpl55	1728.45386550018	0.240758474419535	0.101066700273303	2.38217408670194	0.0172107590585127	0.133967864774397
Zfhx2	674.411830567747	-0.515357444461412	0.216367346684616	-2.38186330958993	0.0172252899684539	0.133967864774397
Pls3	86.6815540348873	0.772855310577355	0.324534285109583	2.38142885370768	0.0172456217256534	0.133993773502081
Cd200r3	37.7208938613875	-1.26589101157009	0.531567881891015	-2.38142870307885	0.0172456287784616	0.133993773502081
Gm20274	139.507517751715	-0.622316771785062	0.261364445231874	-2.38103071453718	0.0172642724065493	0.134072518634794
Appbp2	6100.53246528327	-0.226289590837801	0.0950884901406251	-2.37977898800522	0.0173230243614197	0.134462509784675
Rps6ka1	32419.8562344917	0.171376547868315	0.0720385322225922	2.37895668582999	0.0173617159050101	0.134666562282557
Sash3	76124.4954041029	0.123180455433243	0.051781359287432	2.37885712403731	0.0173664056963987	0.134666562282557
Prpsap1	12597.7784638758	0.187952196937478	0.0790432039223782	2.37784132740938	0.0174143176375434	0.134839018680477
Vps29	4805.88862335299	0.309207111476129	0.130018025828372	2.37818648226741	0.0173980247806421	0.134839018680477
Gpbp1l1	1186.60852319551	-0.295172622269707	0.124129539396314	-2.37794020428366	0.0174096488389216	0.134839018680477
Cdkn2d	14515.5190763051	0.435437238088782	0.183149210258509	2.37749994921724	0.0174304453775056	0.134897606981019
Synj2bp	4000.24454828196	-0.168512189467179	0.0709202019140881	-2.37608163709562	0.0174975910581327	0.135311084602494
Gm49785	20.9787674060737	-1.20171858534401	0.505771947189876	-2.37600877632872	0.0175010465456548	0.135311084602494
Itgb1	14178.4728313951	0.427518475144175	0.179947719126547	2.37579268700553	0.017511298295099	0.135323946658104
Erc1	893.035943809848	-0.318557150137361	0.134138871156698	-2.37483100454328	0.0175569864905479	0.135460329544428
Rpap1	6033.35659688851	-0.239621910618791	0.100906285292078	-2.37469757136727	0.0175633339632714	0.135460329544428
B630019A10Rik	271.963330983639	-0.644385948760732	0.271349439562307	-2.37474582516235	0.0175610382776263	0.135460329544428
Gm12543	124.528092726062	0.510363320399798	0.214903114170378	2.37485306981255	0.0175559370301315	0.135460329544428
Aif1	180.018114571076	0.699821238531286	0.29490959222399	2.37300263193799	0.0176441381947443	0.136016969439621
Sec11a	13618.7319109392	0.130602396658251	0.0550543336364128	2.37224552604285	0.0176803374801826	0.136229377836302
Snord87	60.8765102391586	0.776812562667187	0.327565387715051	2.37147327465177	0.0177173279529948	0.136447671825679
Matn2	58.2985113646115	-0.957776533929546	0.404045965824734	-2.37046429104803	0.0177657599712392	0.13652563432131
Dnal4	814.342564799503	0.311946534960239	0.131601428080954	2.37038867669694	0.0177693941889776	0.13652563432131
Jak2	2705.56007006391	0.94063986251467	0.396870396833033	2.37014367919814	0.0177811738662694	0.13652563432131
Atf2	4020.44569186796	-0.229139294050556	0.0966539776622693	-2.37071768376901	0.0177535860216772	0.13652563432131
Clec2d	367.67232914203	-0.716328674725574	0.302137727593804	-2.37086801582294	0.0177463669539294	0.13652563432131
Gmip	15748.1927434198	0.203432322487138	0.0858364357850331	2.36999964673055	0.0177881022568795	0.13652563432131
Gm11110	444.038173078258	0.431084534567557	0.181888994804865	2.37004187653043	0.0177860706326776	0.13652563432131
Sntb1	3871.59014992835	-0.355589170411311	0.150102860740208	-2.36896997604029	0.0178377014232935	0.136773071205272
Gm3235	92.1795358325535	-0.551662965682369	0.232862622943595	-2.36904900713068	0.0178338902122904	0.136773071205272
Thap4	1640.32381395987	0.340024219754106	0.143557510586795	2.36855750956009	0.0178576038722598	0.13685907792568
Enthd1	169.667268397106	1.10805119709466	0.467925470937712	2.36800786859102	0.017884155539428	0.136995935262405
Frs2	1966.84566669712	-0.415026546585169	0.175328999650424	-2.36713006640464	0.0179266315158427	0.137054795145625
Fam241a	2258.07808547862	0.981141134714209	0.414453991102955	2.36731013761786	0.0179179108503802	0.137054795145625
B3gat3	3639.76270099179	0.164021015857863	0.0692880399053909	2.36723417319677	0.0179215892766134	0.137054795145625
H60b	161.076008549432	-0.909280903347466	0.384063274807478	-2.36752890211455	0.0179073213089276	0.137054795145625
Celsr1	633.836464282056	0.95562842572672	0.403805544404989	2.36655597964819	0.0179544587807259	0.137130200519296
Pot1b	965.724547851801	-0.365425699400435	0.154423400672415	-2.36638811092905	0.0179626028985103	0.137130200519296
Icmt	2442.82451106364	0.210447239779611	0.0889241453297615	2.36659277409077	0.0179526741378533	0.137130200519296
Dennd4a	2354.81551811761	-0.297466493511726	0.125742465756731	-2.36568045426454	0.0179969703063597	0.137326034439375
Cdc34	1907.38827759895	0.26255133670847	0.111025590525622	2.36478216837657	0.0180406785882986	0.13741847677838
Tuba4a	14821.6223023597	0.257702420996705	0.108960496183756	2.3650995546322	0.018025224778968	0.13741847677838
Ncapd3	5271.95838323781	-0.189515055313564	0.0801443626663982	-2.36467106367073	0.0180460911133301	0.13741847677838
4921524J17Rik	1233.93963925187	0.258713926993701	0.109430820203044	2.36417790265729	0.0180701328860065	0.13741847677838

**Tob1**	633.377118091088	-0.436964035516835	0.18481466327848	-2.36433639931705	0.0180624030609299	0.13741847677838
**1700067K01Rik**	47.6861252997025	-1.1427588023347	0.483246968952772	-2.36475110192856	0.018042191863672	0.13741847677838
**B130055M24Rik**	617.278745546082	-0.287379210093632	0.121554392289964	-2.36420259835694	0.0180689282951122	0.13741847677838
**Gm11425**	168.556643961038	-0.488192897801039	0.206527136303698	-2.36381962457056	0.0180876166566381	0.13748508232448
**Gm15510**	30.4817010514614	-1.08019481657209	0.457223531261809	-2.36250923829546	0.0181516890217577	0.137905575658551
**Syt6**	1219.32182971439	0.67536301885704	0.285964053838031	2.36170598994083	0.0181910626606892	0.138005091162484
**Gp2**	125.629482872583	1.05301686189252	0.445850462083585	2.3618162398475	0.0181856540052172	0.138005091162484
**Gm43254**	103.509263383642	1.55215529902341	0.657151100883112	2.36194582484538	0.0181792986063615	0.138005091162484
**Gpr160**	525.02027733117	0.560801094102297	0.237631981462624	2.35995630996538	0.018277087454125	0.138590985088858
**Prag1**	3397.88886801284	0.204202632619643	0.0865401404036084	2.35962908850479	0.018293215102141	0.13864655621185
**Irf5**	760.211120027443	0.394964535343935	0.167422925558241	2.35908274823772	0.018320170148015	0.138784096645323
**Nr2c2ap**	2862.74228713252	0.249116419873159	0.105611160267973	2.35880771730053	0.0183337526309945	0.138820249979136
**Fam102b**	478.066847254435	-0.332088837572937	0.140827738425724	-2.35812093047344	0.0183677082959642	0.138958500745732
**Gm21992**	1447.65536616385	-0.245154091772898	0.103963357286838	-2.35808171427661	0.0183696488578638	0.138958500745732
**4930518I15Rik**	309.839837257498	-0.455512397377671	0.193198788417425	-2.35773940980154	0.0183865949656669	0.139019950515361
**Chpt1**	334.361645011378	0.636644654138283	0.270135950345694	2.35675648992134	0.0184353314294868	0.139321591047685
**Tmem87b**	2081.15882831473	-0.181063220386999	0.0768512419318757	-2.35602204773088	0.0184718213051528	0.139338125488841
**Prpf40b**	680.071336452489	-0.356735255836894	0.15141461013764	-2.35601607739578	0.0184721181928878	0.139338125488841
**Rps4l**	161.079653453497	0.440900544947065	0.187104721031642	2.35643730695872	0.0184511818920659	0.139338125488841
**3110056K07Rik**	1200.33999643663	-0.305064154699657	0.129483907171685	-2.3560005359984	0.0184728910418347	0.139338125488841
**Rpusd4**	2614.3181274313	0.17064999693488	0.0724383661219059	2.35579577606284	0.0184830760702682	0.139348243846515
**Hmces**	2929.20561840336	0.179987838976023	0.0764428336951772	2.35454169181824	0.0185455631594482	0.139752481445923
**Zcchc24**	76.4623063172979	0.883203024986116	0.375151835435693	2.35425484180394	0.0185598819477828	0.139793527653544
**Antxr2**	3223.02676635215	0.16663062529186	0.0707879198452132	2.35394154336247	0.0185755320232501	0.139812216379396
**Cltb**	2800.14328695117	0.256299487186587	0.108885227167432	2.35384995608704	0.0185801092275468	0.139812216379396
**Ckap4**	18.0234971493977	2.0796888407735	0.883695464068767	2.35339992716277	0.0186026144042761	0.139847993877947
**Gm21781**	411.042639749454	-0.556371672841298	0.236411372333503	-2.35340486098287	0.018602367543117	0.139847993877947
**Saraf**	91257.4013079848	-0.155366141735751	0.0660248433000802	-2.35314669403482	0.0186152886436926	0.139863692617453
**Fam126b**	1102.09264130783	-0.247514659191834	0.10519343583722	-2.35294775973329	0.0186252505420942	0.139863692617453
**Proser1**	1345.37851545005	-0.245288257493737	0.104252596150792	-2.35282637123923	0.0186313315227539	0.139863692617453
**Zfp369**	576.404854233955	-0.378234225941097	0.160789188245257	-2.3523610640049	0.0186546572749465	0.139972111753016
**Mroh2a**	193.530330903624	-0.704835109658724	0.299682940362835	-2.35193604549314	0.0186759856851153	0.140065448091558
**Dtx1**	22191.9778998502	0.347463625169422	0.147799449379799	2.35091285270317	0.0187274193840387	0.140384370710893
**Flt1**	19.5719382034525	2.53192374264435	1.07718569288891	2.35049885953644	0.018748265114159	0.140473805707942
**Osm**	2129.63458161805	0.481659711458349	0.20499184485091	2.34965304014244	0.0187909176253701	0.140726468166805
**Ap3s1-ps2**	30.6427592667768	0.91182555141712	0.388177355690418	2.34899212447705	0.0188243049617253	0.140909536000905
**Nfil3**	311.395956476554	0.873850654031001	0.372041524929894	2.34879871056239	0.0188340854199988	0.140915804350865
**Reep3**	1584.86403651686	0.442844312839514	0.188669640512797	2.34719434263976	0.0189153857785885	0.14125579796835
**Dusp1**	4008.48913047127	1.04960404823679	0.447167469806955	2.34722809485653	0.0189136722504359	0.14125579796835
**Flywch1**	5110.79542468116	0.190445272176395	0.0811290872399072	2.34743516358354	0.0189031627829173	0.14125579796835
**Gm9888**	167.874431145818	-0.379502684757353	0.161660150768608	-2.34753390339561	0.0188981531879784	0.14125579796835
**Tbx21**	6958.54092744392	0.904011275660336	0.385265667410148	2.34646207054297	0.0189525951107456	0.141265866206252
**Vrk3**	10746.5611181297	0.192345506204075	0.0819684452701876	2.34657990120538	0.0189466034009633	0.141265866206252
**Rsbn1l**	2893.0071482814	-0.221540025466381	0.0943972170951874	-2.34689148985172	0.0189307670446383	0.141265866206252
**Irgc1**	181.770319271412	-0.585399705192107	0.249449175187982	-2.34676945614656	0.0189369679747205	0.141265866206252
**Procr**	18.1032495077032	1.98440311605757	0.846227189910087	2.34500042035806	0.0190270581474233	0.14175383225955
**Setx**	12855.7718782606	-0.408621008299231	0.174314692517515	-2.34415700935922	0.0190701415782601	0.141940586135402
**Gm15441**	120.013972696968	-0.791623073656626	0.337679583223001	-2.34430244819937	0.0190627061379731	0.141940586135402
**Ets2**	3585.76237166673	-0.248704155643916	0.10610471323731	-2.34395012300419	0.019080722840544	0.141952289800969
**Unk**	6748.39165146695	-0.254104645097309	0.108426149121398	-2.34357345673877	0.0191000007252329	0.142028650980413
**Abcf3**	8644.16808831726	0.199624675596339	0.0851910544993031	2.34325865279636	0.0191161255388346	0.142081504771423
**Ap2a2**	7145.79722451957	0.190104106361835	0.0811524862896705	2.34255430798837	0.0191522464289872	0.142215809133625
**Polr3e**	8819.38445856125	0.200832248796668	0.0857321327874216	2.34255514550938	0.0191522034430216	0.142215809133625
**Tmem88**	41.1416646168844	-1.18799490876735	0.507177198311619	-2.34236655891108	0.0191618848238703	0.142220357592899
**Chaf1a**	907.00070382754	0.41431720921865	0.176928593545124	2.34171990471953	0.0191951142943569	0.142399913340952
**Ccr3**	81.5248896416284	0.873681639772869	0.373260599733763	2.34067469321981	0.0192489308416823	0.142664818096137

**Gm48706**	32.7131794677688	-1.60257639600457	0.684634066148464	-2.34077805245679	0.0192436031432511	0.142664818096137
**Zfp955b**	1942.13357769312	-0.277977087929607	0.118773973569765	-2.34038720415739	0.0192637563749885	0.142707573672164
**Ptprs**	697.63082063299	0.462661353319388	0.197763188801994	2.3394715473698	0.0193110424412708	0.1429906464977
**Atp1a1**	66645.7997088448	0.151023674389996	0.0646002831262862	2.33781753084212	0.0193967157977822	0.14349016470688
**Trim65**	1731.96791010871	-0.186314251790493	0.0796901321383393	-2.33798397356222	0.0193880795352173	0.14349016470688
**Lman2l**	2761.69474800567	0.267029311632098	0.114311661200796	2.33597612725655	0.0194924857764575	0.143596431811871
**Smpd2**	4099.45021664912	0.186137554289753	0.0796833881379845	2.33596435391811	0.0194930994253724	0.143596431811871
**Zc3h13**	6982.02632779859	-0.172548278014206	0.0738585219187455	-2.33619998791788	0.0194808209414521	0.143596431811871
**Crim1**	1273.14654850656	1.01544598163369	0.434467121748994	2.33722169250897	0.0194276597978997	0.143596431811871
**Alas2**	27.9006673753994	1.36336599354791	0.583456707527012	2.33670463628151	0.0194545473089873	0.143596431811871
**Sigirr**	14984.9480952488	0.228386044690517	0.0977621183664703	2.33614050622749	0.0194839197926985	0.143596431811871
**Lancl1**	5145.78573664536	-0.176219827563869	0.0754190767543865	-2.33654182930076	0.0194630201842738	0.143596431811871
**Pdha1**	13351.1562703645	0.16396168852097	0.0701643362981165	2.33682376505814	0.0194483495966271	0.143596431811871
**Nxnl1**	140.153971092909	0.579300538735095	0.247931885529079	2.33653100930881	0.0194635833974706	0.143596431811871
**Gstm4**	506.811327258141	-0.417053257590616	0.178585526690527	-2.3353138707222	0.0195270300897503	0.143779165011306
**Agl**	761.782781814067	-0.355610340027206	0.152364280960162	-2.33394820483015	0.019598434195672	0.14417018096228
**Wnk3**	51.7329924385521	-1.08572112122861	0.46517623475019	-2.33399954709997	0.0195957456349387	0.14417018096228
**Gm13341**	152.901658316848	-0.700429513370657	0.300132645251209	-2.33373318248803	0.0196096974365533	0.144185722122151
**Tha1**	3003.40291744928	0.474898159696324	0.203522601150904	2.33339273874652	0.01962754202889	0.144235902171654
**Rpe**	3139.44373368105	0.198749961426212	0.0851843597345155	2.33317432972008	0.0196389975545445	0.144235902171654
**Rnu3b2**	37.3102746402324	1.26008195328113	0.540093922317952	2.33307930567551	0.0196439833763006	0.144235902171654
**Tinf2**	5979.73464945769	0.201973320471548	0.0865926657517612	2.33245297067714	0.0196768742479362	0.144410110630988
**Ski**	1677.17182718545	-0.363342005803037	0.155839802181564	-2.3315096702941	0.0197265007568103	0.144639586982345
**Fryl**	9373.22569866177	-0.391976491391591	0.168112865894902	-2.3316269656401	0.0197203239692872	0.144639586982345
**Ckb**	514.609918665899	0.38909034742431	0.166911272159573	2.33112085475166	0.0197469879157328	0.144722459808466
**Tubb6**	244.291845976668	0.636377791508478	0.273218821866694	2.32918723227265	0.0198491490034622	0.14500423757922
**Aamp**	23078.0737349739	0.146842381189567	0.0630448328986564	2.32917392969562	0.01984985342758	0.14500423757922
**Rcan1**	1218.21009407706	0.197535164176592	0.0847903403279937	2.32968948364246	0.0198225687684973	0.14500423757922
**2900041M22Rik**	23.3451500215728	1.44371826036549	0.619738410686504	2.32956072347725	0.0198293800724527	0.14500423757922
**Gm19569**	66.43819640132	-0.945059599050544	0.405746647274201	-2.32918646500085	0.0198491896329485	0.14500423757922
**Gm42979**	156.876093276308	-0.511594935283383	0.219552925206542	-2.33016679145634	0.0197973374070864	0.14500423757922
**Gm48194**	171.101542958124	-0.601642352015342	0.258249563403644	-2.32969358819389	0.0198223516747961	0.14500423757922
**Mn1**	25.138792128452	2.08570261029485	0.895634501165379	2.32874303924311	0.0198726825896693	0.145103734738378
**2210016F16Rik**	6433.85350705604	0.192105667235348	0.0825156776409122	2.32811112660729	0.0199062036373594	0.145207246213071
**Srxn1**	90.793901516797	0.952676503101299	0.409233252043329	2.32795477480024	0.0199145052399852	0.145207246213071
**Shb**	20.2127508822475	1.41745344790141	0.608877394337943	2.32797844210108	0.0199132484149011	0.145207246213071
**Trgv2**	115.381751026824	1.13083117332853	0.485810733356884	2.32771961523914	0.0199269968837436	0.145231123911724
**Slc6a19**	14853.7322346509	-0.461895117017278	0.198457669780182	-2.32742386589991	0.0199427167586343	0.145278496516782
**Vps35l**	8920.54988186609	0.162106989424015	0.0696778106531817	2.32652243094857	0.0199906972579492	0.14554435761948
**Gnpda1**	4841.53321466797	0.244329746958035	0.105025215567345	2.32639129220701	0.0199976857426017	0.14554435761948
**Jade2**	2552.09068393205	-0.320952673836691	0.13803315874705	-2.32518531597792	0.0200620531857031	0.145878067395996
**Msl1**	3308.58224070555	-0.265666185127945	0.114254663837584	-2.32521085971244	0.0200606879492549	0.145878067395996
**Stt3b**	13038.1233489919	-0.144207719627743	0.0620252782474898	-2.32498303437404	0.02007286739829	0.145889378041908
**Selenop**	10630.2113059207	-0.293731766180035	0.126347537305112	-2.32479217597026	0.0200830755774494	0.145896275644938
**Dcun1d3**	813.193572965194	-0.405319011059495	0.174378545374463	-2.32436284056108	0.0201060554066125	0.145995905549306
**Tmem189**	9518.68594398676	0.447980665271368	0.192777859268409	2.3238180306154	0.0201352489490588	0.146140542464449
**Ddx39**	23672.5667906408	0.214379051814641	0.0922649197078694	2.3235163753831	0.0201514289954567	0.146179672913194
**Lilrb4a**	722.418451389401	0.843542589453918	0.363068294190251	2.32337167125889	0.0201591945954415	0.146179672913194
**Ifi30**	1482.99133215123	0.253719563850981	0.109237320346999	2.32264543880265	0.0201982075797547	0.14632788819963
**Eef2**	81982.0357267363	-0.200770024943242	0.08643444557413	-2.32280109636444	0.0201898401610315	0.14632788819963
**Ruvbl1**	7291.68855289789	0.184253343827286	0.0793396447233302	2.32233638642833	0.0202148297507102	0.146344542956492
**Zfp969**	86.2378191835943	0.716705792749315	0.308624618000036	2.32225736687418	0.0202190816790178	0.146344542956492
**Klhl42**	2060.98923805392	-0.228267379406975	0.0983358484524789	-2.32130380730163	0.0202704529028092	0.146581700958956
**Gm37488**	111.387694458708	-1.05113160138385	0.45278887774682	-2.3214607359936	0.0202619908453951	0.146581700958956
**Smim7**	3924.83090685559	0.207356286350343	0.0893366124023896	2.3210672620579	0.0202832139457459	0.146606698230788
**Il17rc**	19.7083172335409	-2.34068593089677	1.00860481646065	-2.32071659057765	0.0203021447194169	0.146640102048446

**Syne1**	3287.62789344379	-0.451391425973627	0.194511870011874	-2.32063691509455	0.0203064480973351	0.146640102048446
**Itgal**	46940.1225544046	0.169116227172414	0.0728803513369401	2.3204639394582	0.0203157934788374	0.146640383804874
**Fgr**	1056.64460866444	0.466050867966873	0.200999715213969	2.31866431985115	0.0204132446023298	0.147164919063081
**Kctd7**	496.07165675366	0.612079573081161	0.26398617432618	2.31860465663963	0.0204164823933423	0.147164919063081
**Gm48552**	61.2736569238237	-1.05477836203655	0.454906895125108	-2.31866866240061	0.0204130089592264	0.147164919063081
**Stk11ip**	4540.29517603275	-0.183073139489073	0.0789741393912078	-2.31814035455579	0.0204416943545357	0.147211964325603
**Wdr35**	170.749897864327	0.805823023777155	0.347591961570194	2.31830166652005	0.0204329319138644	0.147211964325603
**Mknk2**	5037.67345829788	0.732914733591119	0.316336472549369	2.31688343643883	0.0205100822817969	0.147636987900537
**Wdr44**	1654.58307520484	-0.263115680475295	0.113576867541097	-2.31663089651674	0.0205238468272795	0.147668609341298
**Gm37733**	22.3298307683235	-1.65878066601572	0.716118933323513	-2.31634800984427	0.0205392749706839	0.147712166003225
**Zbtb37**	943.426420884446	-0.376873019659105	0.162749535939028	-2.31566263758992	0.0205766959140476	0.147846328097423
**Atp8b2**	6718.855235472	0.311435341441941	0.134486474497251	2.31573727102429	0.0205726180876618	0.147846328097423
**Zc3h8**	557.852846318551	0.329389992484563	0.142277853218139	2.31511781372991	0.0206064853937942	0.147925462574039
**Kif7**	70.6716855201763	2.00356474055193	0.865409735141652	2.31516316398264	0.02060400433189	0.147925462574039
**Prelid2**	378.15884709448	0.876053001519037	0.378474018733986	2.31469786076592	0.0206294729318728	0.147955669088539
**3110001I22Rik**	493.601616750989	-0.406530986540038	0.175620468920964	-2.31482690507445	0.020622406879025	0.147955669088539
**Zfhx3**	37.5135303022025	-1.2832098384822	0.554510823384481	-2.31412947117978	0.0206606212568834	0.148111651112244
**Cdkn1c**	66.908984401093	0.785231597736988	0.339480808801717	2.3130367825759	0.020720616901425	0.148474197596977
**Itgb3bp**	499.744835687561	-0.327960947469782	0.14184435440258	-2.31211844032204	0.020771157216815	0.148768692847888
**Mcfd2**	4579.35816647399	0.233665989417725	0.101084438655968	2.31159209592078	0.0208001726266032	0.148908823297313
**Kifc3**	249.804513694441	0.698615745734707	0.302283874938935	2.31112475277034	0.0208259651224077	0.149025764048037
**Copb1**	14739.1529846526	0.114058158646698	0.0493678929551102	2.3103712113136	0.0208676115114513	0.149171258620761
**AA914427**	102.948530824925	-0.820858339992562	0.355312536757959	-2.31024311014315	0.0208746985631008	0.149171258620761
**Gm42141**	284.995772873354	-0.412410182743158	0.17850671297904	-2.31033430541956	0.0208696530730039	0.149171258620761
**Hyal1**	337.836452496527	-0.470518116073943	0.203717252689324	-2.30966258312691	0.0209068418611796	0.149200245405245
**Klrb1f**	239.966974093029	1.86907744237016	0.809473286973688	2.3090044754385	0.0209433328967257	0.149200245405245
**Tdg**	3159.68430469944	0.280675807622765	0.121558681586616	2.30897377266115	0.020945036671727	0.149200245405245
**Ccdc115**	5378.57636728463	0.157428084955177	0.0681739592465499	2.30921141584048	0.0209318523990506	0.149200245405245
**Upf1**	2100.31554315879	1.01958773334857	0.441498070029569	2.30938208468337	0.0209223882772184	0.149200245405245
**Qk**	991.625174916748	-0.29509829248304	0.127753016877206	-2.30991251476027	0.0208929980704142	0.149200245405245
**Gm17491**	333.68307283061	-0.522762080353388	0.226397271455625	-2.30904761789875	0.020940939016262	0.149200245405245
**Ddx1**	7376.6778853484	0.155540199319599	0.0673817100824024	2.30834449184186	0.0209799836789563	0.149314183753078
**Peak1**	5190.7034541729	-0.462883212880843	0.200511482962316	-2.30851224100635	0.0209706627929791	0.149314183753078
**Tbcb**	3273.50425829692	0.222974497711055	0.0966374562729461	2.30732995580179	0.0210364327701218	0.149531113615197
**Brca2**	566.406810159392	-0.372978259360564	0.161661273443108	-2.30715898382318	0.0210459587215755	0.149531113615197
**Tmem29**	353.783806267968	0.247671002208054	0.10733600477048	2.30743637922482	0.0210305051322258	0.149531113615197
**Fzd1**	70.1895239410276	1.19119869008768	0.516315318540279	2.30711475587327	0.0210484235576891	0.149531113615197
**Nrip3**	161.036686537288	-0.989973876540516	0.429240167815374	-2.30634025137723	0.0210916276980472	0.149703052990149
**Enc1**	488.070006800373	-0.434485705036694	0.188384621529707	-2.30637565587156	0.0210896510472163	0.149703052990149
**Desi2**	3111.72534447399	-0.185258562147853	0.0803349254021208	-2.30607747776613	0.0211063035271754	0.149739767977354
**Gm43868**	16.919386743403	-1.32935492157724	0.576553834119298	-2.30569088766509	0.0211279106431444	0.149825602162028
**Xpo1**	4357.07202254424	-0.246552520714272	0.106975725293499	-2.30475203638797	0.0211804647315001	0.150130716497637
**Atxn3**	1331.88049852819	-0.335041119996919	0.145394707877414	-2.30435567351875	0.0212026861043888	0.150166059231549
**Oas1b**	973.843095059131	-0.282987909045996	0.122807389527736	-2.30432313669597	0.021204511124592	0.150166059231549
**Zzz3**	7121.406335116	-0.180897590798926	0.0785199349397389	-2.30384285134416	0.0212314666924969	0.150289407310725
**Tnpo1**	6605.19005018419	-0.202507382722112	0.087933721125375	-2.3029547724175	0.0212813879226365	0.150391145570092
**Endog**	1323.38263289827	0.31229275787008	0.135675126877816	2.30176868123602	0.0213482206303407	0.150391145570092
**P4ha1**	4745.86807933268	0.215604364164147	0.093670877448951	2.30172247806313	0.0213508277362629	0.150391145570092
**Lamb3**	1817.15225787847	0.284355170937626	0.123463571350962	2.30315037728261	0.0212703836930913	0.150391145570092
**Rprd2**	2232.09797087589	-0.282060138515642	0.122474718213828	-2.3030070420182	0.0212784468831905	0.150391145570092
**Dctn5**	11479.2111492393	0.143571325297369	0.0623689528470822	2.30196786611731	0.0213369844009479	0.150391145570092
**Chd6**	4104.95062245684	-0.356552767475073	0.154896168464092	-2.30188242233847	0.0213418037437278	0.150391145570092
**Ndufaf3**	1902.19793782581	0.286297838610541	0.124336645245184	2.30260224607138	0.0213012326748198	0.150391145570092
**Gm14681**	355.325960368306	0.320513857605455	0.139227879004462	2.30208101924173	0.0213306036080579	0.150391145570092
**Gm42783**	107.278769829911	-0.800591726893004	0.347712553405436	-2.30245275602549	0.021309652777414	0.150391145570092
**Tmem179b**	3722.35234656604	0.152649364219782	0.0662941051177597	2.30260841365348	0.0213008853448827	0.150391145570092

**Atp13a3**	1157.08953781904	-0.290782849101508	0.126353754970066	-2.30133919779745	0.0213724657802348	0.150409085886181
**Zfp764**	1949.6959318675	-0.209029310963125	0.0908288351468344	-2.30135408678541	0.0213716248677291	0.150409085886181
**Chmp6**	4028.65069165672	0.194728510399988	0.084622957007287	2.30113100849477	0.0213842270855202	0.150424672404706
**Coil**	1595.69326949304	0.26055762144035	0.11324958682617	2.30073794300272	0.0214064480277655	0.150513789189425
**Ttll4**	1934.28305016765	-0.236981155635444	0.103045609850181	-2.29976954845522	0.0214612795231571	0.150832016702224
**Stat3**	38658.2507557395	0.227212969140244	0.0988688772706278	2.29812429768275	0.0215547157137688	0.151274717971765
**Havcr2**	536.101612476057	0.78653484089792	0.342271604788825	2.29798449504217	0.021562671610369	0.151274717971765
**Dnpep**	5783.73496375164	0.174899610995753	0.0760945634216705	2.2984508108229	0.0215361444390178	0.151274717971765
**Gm15472**	260.727392777602	-0.794178470642091	0.345568718038273	-2.29817813125704	0.0215516528312397	0.151274717971765
**Chchd6**	448.729853244032	0.524594444550084	0.228435380171187	2.29646757939579	0.0216491608229966	0.151746364362971
**Ado**	2115.97220546825	0.483151700997668	0.210387572118442	2.29648403721141	0.0216482208380738	0.151746364362971
**B930095G15Rik**	31.9485674531896	1.27468512512741	0.555700194339616	2.29383602545293	0.0217999192574802	0.15273513905741
**Timm50**	5476.24178414529	0.173498723033744	0.0756451736319764	2.29358615630705	0.0218142813279821	0.152767835948895
**Tbxa2r**	11088.1910372829	-0.281170829030902	0.122612477581038	-2.29316652414162	0.021838419625567	0.152868937378969
**Actr8**	6284.46274682396	0.135086134812041	0.0589219074935028	2.29263003454084	0.0218693137067214	0.152949301410035
**Trim2**	588.978400175346	0.330463754153972	0.144137881056138	2.2926919123035	0.0218657484988986	0.152949301410035
**Ttll11**	19.1284819265452	2.30958219777688	1.00757275159701	2.29222375666291	0.0218927347638418	0.153045173768347
**Glo1**	5682.11392746826	0.128226132445984	0.0559501666921928	2.29179178591932	0.0219176608958495	0.153151477931663
**Bcas3os1**	48.6410430621757	-0.922185405043925	0.402850770344572	-2.28914891798556	0.0220707013988129	0.154152500860414
**Macf1**	33374.6161150254	-0.443708425367752	0.193867860477953	-2.28871574831358	0.0220958734064991	0.154259936759507
**Msra**	2954.91790428927	0.190331507378792	0.0831697898461518	2.28846925946151	0.0221102083133497	0.154291652964327
**Il12rb1**	1396.5255187907	0.348453831286403	0.15229196129309	2.28806450667343	0.0221337648298924	0.154344002157472
**Zup1**	4632.78177022434	-0.142375097547341	0.0622267752914692	-2.28800378744453	0.0221373005569516	0.154344002157472
**Ighv2-4**	16.8582468000056	7.38374444885962	3.22804669456211	2.28737225558047	0.0221741042750029	0.154532225148704
**Ppp4r2**	1618.22177248311	-0.297703605410068	0.130183750057126	-2.28679543552427	0.0222077660460981	0.154698395043487
**Nfix**	190.509014256234	-0.461926515213043	0.202128792157905	-2.28530784892922	0.0222947830313548	0.15503040433586
**Banp**	4742.52930029479	0.243560999314377	0.106563737984798	2.28558986312139	0.0222782637573431	0.15503040433586
**Rnf125**	12164.9399166118	-0.217150071365681	0.0950178771417643	-2.28536016482139	0.0222917177692629	0.15503040433586
**Dzip1**	2356.29266086715	-0.348429806286265	0.152449791985184	-2.28553808928862	0.0222812956654689	0.15503040433586
**Rab6b**	1508.70720657033	0.522038877851593	0.228500882433426	2.28462521585092	0.0223348130723376	0.155240251248709
**Patl2**	37.7420726847397	-1.584490011011	0.693599755264718	-2.28444430521212	0.0223454322578398	0.155245580285177
**Hivep2**	3954.62481815181	-0.317694294603207	0.139080210525126	-2.28425232751437	0.0223567058638957	0.155255449227591
**Rad21**	2664.48528190481	-0.314337313825286	0.137643978741006	-2.28369825327957	0.0223892707896904	0.155344667473867
**Aacs**	5739.35008518116	0.214498887170863	0.0939257637042254	2.28370660734072	0.0223887794855743	0.155344667473867
**Ctnnbip1**	62.9030450932058	1.24127609513949	0.543604649172319	2.28341699621118	0.0224058170486865	0.155391047199011
**A130048G24Rik**	64.7990684814121	-0.807805261958276	0.353826130542895	-2.2830571069435	0.0224270047173918	0.155469561518673
**Gmcl1**	4868.43110652611	0.124136966637025	0.0544053823013296	2.2817037834507	0.0225068345608595	0.15554947065329
**Celf2**	19737.3316077883	-0.18402014633318	0.0806324394252075	-2.28220983570604	0.0224769546855493	0.15554947065329
**Tmcc3**	138.480459169367	0.666941324942584	0.292203054057501	2.28245843320769	0.0224622888711139	0.15554947065329
**Add1**	43510.7582949008	-0.177742005299601	0.0778796221153437	-2.28226589282054	0.022473646913433	0.15554947065329
**Tmem176b**	525.663602320072	0.559659175452043	0.245264214159153	2.2818623473903	0.0224974684346182	0.15554947065329
**Igkv14-111**	96.254489835864	1.78254648986391	0.781190805539904	2.28183239897702	0.0224992371814207	0.15554947065329
**Gm37274**	277.163783609505	-0.519089786171618	0.227502303054757	-2.28169024753424	0.0225076342634703	0.15554947065329
**Tube1**	258.591660540324	-0.515500562533162	0.225980901312963	-2.28116871619713	0.0225384651753033	0.155694255049213
**Trps1**	330.292569503554	-0.491128576134783	0.215335965045254	-2.28075498689487	0.0225629493528358	0.155795088936298
**Trappc10**	2361.90576357286	0.440845323292916	0.193325937314939	2.28032166514085	0.0225886177790579	0.155864644067318
**Kif9**	175.784692249691	-0.563439285504586	0.247095291576733	-2.28025099915599	0.0225928061845374	0.155864644067318
**Bpnt1**	2159.52065514861	0.211541363989376	0.0927816807180519	2.27999064418994	0.0226082433683504	0.155902884356717
**Gm10614**	17.2060405063536	2.29772451865692	1.00801282192016	2.2794596146902	0.0226397580316321	0.156051910456879
**Gm8373**	50.1745629728258	-0.86417251958061	0.379196631808771	-2.27895621186955	0.0226696683950084	0.156189752907804
**Alpl**	33.2331881948654	1.70187228891194	0.747272282457372	2.27744602451386	0.0227596043563851	0.156672383505269
**Klra7**	305.283871302688	1.03182645476315	0.45304936903175	2.27751438428963	0.0227555266478968	0.156672383505269
**Uqcrc2**	10962.3155550267	0.13855880893162	0.0608554179922552	2.27685247267966	0.0227950368360913	0.156847770928511
**Acaca**	960.582474176598	-0.400066797342905	0.17577153107644	-2.27606140137064	0.0228423348723315	0.157068134225245
**Gm9514**	16.9829612961962	1.24572885694416	0.54733651351631	2.27598346936714	0.0228469990254656	0.157068134225245
**Gcdh**	3318.9852043069	0.166341894931442	0.0731596153526504	2.273684656892	0.0229849533312266	0.157947627405206

**Map3k11**	6344.23750332601	-0.169884549086114	0.0747476241120539	-2.2727752367278	0.0230397280531993	0.158048321695368
**Zfp770**	189.592330134122	-0.450061006178805	0.197982161409444	-2.2732401897969	0.0230117096058695	0.158048321695368
**Erdr1**	494.106092611909	0.496997122899088	0.218644863605196	2.27307934293169	0.0230213990179837	0.158048321695368
**Gm35558**	37.9310835952466	-0.95189753155748	0.41880184063277	-2.27290675255689	0.023031799799677	0.158048321695368
**Ralbp1**	5404.268370459	-0.156602162255972	0.0689453826995136	-2.27139448827915	0.0231231077763418	0.158381274507767
**Mllt1**	2065.17777802254	0.470782393778526	0.207247470104665	2.27159537118001	0.0231109607280418	0.158381274507767
**Sorbs1**	1447.4763051956	-0.23521010199321	0.103557213262512	-2.27130582779361	0.0231284706887333	0.158381274507767
**Lrrc8c**	11142.7830873686	0.19248462629953	0.0847343511786024	2.27162447841033	0.0231092011229046	0.158381274507767
**Rangap1**	14580.7094686247	0.119330352368119	0.0525488420658756	2.27084646734033	0.023156273870134	0.158502783393441
**Msi2**	1501.50586769376	-0.38173546393816	0.168163350107438	-2.27002770635975	0.0232059020432574	0.158773512156147
**Pik3ap1**	1638.22470947809	0.386621848828516	0.170405800555696	2.26883033070316	0.0232786457464513	0.158857349946658
**Pycr1**	115.580779260702	1.3189148687156	0.581266327696009	2.26903711065364	0.0232660691994929	0.158857349946658
**Pla2g12a**	904.064197362782	1.34168390579705	0.591253075346818	2.26922101844468	0.0232548887150973	0.158857349946658
**4932438A13Rik**	21582.7633795856	-0.284409383447176	0.125349217470851	-2.26893625014702	0.0232722028912371	0.158857349946658
**Ms4a4b**	155893.945474166	-0.179346125520036	0.0790451396231918	-2.26890769470431	0.0232739397058355	0.158857349946658
**Ipo7**	3257.16007609061	-0.242211172307105	0.106717078024916	-2.26965708572489	0.0232283970961051	0.158857349946658
**Trip13**	169.072589000021	0.633769661447857	0.279427439126996	2.2681010262554	0.0233230498434829	0.159091470296
**Elp5**	3926.7503724476	0.218879090091974	0.096544665178766	2.26712775570446	0.023382422476691	0.159358490901912
**Pes1**	7676.74580402578	0.143509660218093	0.0632990236347788	2.2671702022785	0.0233798303656823	0.159358490901912
**Psmd8**	8734.38841578191	0.146534224286169	0.0646483405131721	2.26663551025433	0.0234125009575556	0.159445775746764
**Zfp229**	31.8670655878964	1.93698283889151	0.854627925009097	2.26646331369396	0.0234230308925332	0.159445775746764
**F830016B08Rik**	20.3239084417669	-2.21115591597507	0.975615475466564	-2.26642152731089	0.0234255867775438	0.159445775746764
**Chst10**	4187.96403566016	0.149172500636545	0.0658236463418388	2.26624486680448	0.023436394980952	0.159450464471011
**Rgs10**	3469.13692103518	-0.192479084057834	0.084951000765631	-2.26576593946031	0.0234657178389093	0.159581059986057
**Chd2**	15777.0196322651	-0.266887038822711	0.117828422732666	-2.26504804726305	0.0235097312210826	0.15981140416333
**Gm48314**	35.399112547944	-1.47402100968903	0.651008523804945	-2.26421153608532	0.0235611073991808	0.16009157795985
**Aars**	7495.22026639717	0.358178778624742	0.158240724210161	2.26350568358776	0.0236045346489533	0.160248450005841
**Apol7b**	2865.27127084857	-0.214932953783904	0.0949506459061179	-2.26362813788985	0.0235969957285705	0.160248450005841
**Cfap97**	2035.79169384915	-0.270522456082356	0.119527182812532	-2.26327141422422	0.0236189633070527	0.160277349194328
**H2bc8**	149.372417003449	0.802929664509288	0.35482115078334	2.26291376017652	0.0236410059923033	0.160357869746329
**Meak7**	1363.27241366366	0.295350606598361	0.130536561240794	2.2625891458374	0.0236610278472693	0.160424619530732
**Umps**	5576.36972196569	0.226826224574197	0.100282244117714	2.26187822749501	0.0237049279070481	0.160653139368326
**Foxd2**	22.4772430566151	2.09401812212224	0.925920685733816	2.26155237093842	0.0237250735471381	0.160720543371563
**Gm16091**	194.627679900611	-0.626668112185852	0.277191022846285	-2.26078069105928	0.0237728408568668	0.160974926249098
**Rnu3b4**	50.3590778059098	1.14289001266072	0.505574546468556	2.26057664620147	0.0237854852576831	0.160991362201595
**Abhd4**	823.282125875048	0.263657910424188	0.116663783003271	2.25998080669813	0.023822442073415	0.1611722712541
**Gm49204**	42.5951941559346	-1.21096748106334	0.535917392747587	-2.25961593605844	0.0238450976767558	0.161256310769374
**Cdca5**	202.92023268866	0.613134661006082	0.271391573803442	2.25922512041641	0.0238693849883364	0.16135130813437
**Dtwd2**	494.182583078614	0.303467168822473	0.134341038904397	2.25893123424804	0.023887662730903	0.161405618203619
**Pld3**	33618.1258004505	0.15571887663325	0.0689451358678923	2.25859119244652	0.023908826190981	0.161479371749373
**Bid**	5475.34259642146	0.177849719233984	0.0787539182382802	2.25829677065561	0.0239271634949896	0.161533982515232
**Nelfe**	1962.3521101173	0.195429074829773	0.0865499372244035	2.25799210371547	0.0239461517322144	0.161592939119701
**Rsf1**	4318.425141827	-0.223698893982533	0.0990864176831561	-2.2576141030534	0.023969728626861	0.161682797077675
**Pmf1**	2481.83699887954	0.14898184524495	0.0660006698107288	2.25727777721328	0.0239907230750885	0.161755166236273
**Trip11**	6091.8557946792	-0.164076662383917	0.0727036361644006	-2.25678756992153	0.0240213518152647	0.161823189633658
**Tmco1**	4572.61491030527	0.21241758567586	0.0941213865408108	2.25684717876268	0.0240176255732543	0.161823189633658
**Fam76b**	2405.14405076541	-0.21422749437887	0.0949521081880752	-2.25616364361854	0.0240603845204404	0.162016871319906
**Gm33023**	38.3909609657429	-1.01006923515017	0.447748526257927	-2.25588511388715	0.0240778270328963	0.162065066449102
**Tmpo**	8411.67973563929	0.116841703520769	0.0518199339150416	2.25476365354557	0.0241481678198611	0.162469120554036
**Klhl7**	1677.47295425065	0.285524689539611	0.126659239213305	2.25427447151142	0.0241789062990826	0.162537127369729
**Gm14295**	202.581322193933	-0.605128182447632	0.26842820672639	-2.25433902728577	0.0241748478992883	0.162537127369729
**3110070M22Rik**	36.4130402845054	1.43959786301459	0.638865439416401	2.2533663181556	0.0242360614125016	0.162851863401615
**Synj1**	2173.31508811716	-0.2397566355928	0.106417717618004	-2.2529766749314	0.0242606196978837	0.162947393256416
**Tstd3**	1124.69953781914	0.269897050385056	0.119880926189814	2.2513760859481	0.0243617274512136	0.163487112201351
**Sdhc**	10371.5994392805	0.163217670133467	0.0724928412175307	2.25150052601327	0.0243538536131771	0.163487112201351
**Gm19552**	30.9848119776464	-1.02085346817032	0.453527692397858	-2.25091760719823	0.024390756341885	0.163542616033652

**Gm48735**	22.9580233642935	1.26803297816264	0.5633331451299	2.25094686709806	0.0243889028364954	0.163542616033652
**Kdm4d**	104.678825185506	-0.70685362692199	0.314075415632363	-2.25058566108654	0.0244117924384943	0.163614042302575
**Bcl2l12**	1383.85843080942	0.181443669821268	0.0806308295900149	2.25030141378748	0.0244298182595675	0.163665240780615
**Cwf19l2**	1381.07544037785	-0.318487575553521	0.141549532120755	-2.25000797093289	0.0244484393253573	0.163706262406452
**2810428J06Rik**	87.6553939098009	-0.785962429710489	0.349335649187165	-2.24987753622989	0.0244567202960454	0.163706262406452
**Nktr**	7588.87318414346	-0.214288419393091	0.095270103284572	-2.249272458045	0.0244951669841196	0.163824425368749
**Lgals1**	4137.51221582379	0.494485571416798	0.219827678870679	2.24942361197243	0.0244855577544126	0.163824425368749
**Clic3**	167.333225421727	-0.756346964072992	0.336435512374731	-2.24811869215088	0.024568622466396	0.164245983963938
**1110065P20Rik**	137.342177646464	0.767028745401422	0.341251622016958	2.24769259957776	0.0245957982267392	0.164357927336187
**Styk1**	25.3918213230678	2.18132844776797	0.970814152757606	2.24690631216272	0.0246460152321777	0.16462368037873
**Traj27**	90.6996915233505	0.83435292127958	0.371375338431808	2.24665677802616	0.0246619705268401	0.164660453216703
**Hmgb1-ps5**	89.3737635041298	-0.682878292167506	0.303986899987507	-2.24640697410175	0.024677952033669	0.164697369840966
**Dtwd1**	1256.41784555716	0.253924796497181	0.113047955925141	2.24616884417909	0.0246931950337988	0.164729328597616
**Tuft1**	585.948918900429	0.311791315535879	0.13884746144077	2.24556727433497	0.0247317386189872	0.164916633694194
**Shmt1**	2132.16062334524	0.282124686711956	0.125680096357233	2.24478413757771	0.0247819935722311	0.165181841251119
**Nckap1**	539.821325470893	0.525466529005845	0.23413279118636	2.24430984802806	0.0248124723637952	0.165315064285971
**Rnu1a1**	29.3581288425688	-0.853701056611685	0.38042173677786	-2.24409116009632	0.0248265366184628	0.165338857775621
**Ahctf1**	3898.13434790741	-0.29215035996634	0.130208259706251	-2.24371603326417	0.0248506778488523	0.165346903416239
**Trav12n-3**	1079.15937493268	0.297049293705066	0.132399494590964	2.24358329027443	0.0248592253724104	0.165346903416239
**Trav14-3**	347.977566367049	0.323194862944973	0.144048448455839	2.24365389845942	0.0248546784867782	0.165346903416239
**Gm11100**	57.2663888335288	-0.874118388834013	0.389640291316064	-2.24339835565146	0.024871137839306	0.16535633710293
**Eif4a1**	45968.2656573634	0.176199546054034	0.078567414665713	2.24265424544926	0.0249191192852322	0.165605467135134
**Trgc1**	116.160389398483	0.686047583088638	0.305960277141992	2.24227664289326	0.0249434983616474	0.165697598517908
**Sec22c**	2542.42783597979	-0.27244676021217	0.121527429113191	-2.24185405879369	0.0249708060595752	0.165809098643374
**Clec7a**	85.3740176985511	0.845654836182538	0.377366754123148	2.24093624290647	0.0250302051842047	0.166133505934083
**Gm43167**	54.0039418090557	-1.61287258175234	0.719971662654288	-2.24018897605807	0.0250786570421605	0.166385010110873
**Bcl2l11**	3363.54021516105	-0.172569408160613	0.0770461869397273	-2.23981763426674	0.0251027645706173	0.166474857466001
**Gpr35**	163.008460173035	0.76333502316383	0.340902396355937	2.23916003912988	0.0251455049037081	0.166478033936021
**Atcay**	48.7151101742135	1.31628264418707	0.587817945773276	2.23926923914426	0.0251384031003614	0.166478033936021
**Vsig10l**	251.126949129303	0.909570480997983	0.406204105051552	2.23919568927682	0.0251431862118734	0.166478033936021
**Gm45718**	113.12576780877	-0.766894174349376	0.342466897807956	-2.23932350617847	0.0251348744994028	0.166478033936021
**Slc39a4**	1072.16143859937	-0.426835009869453	0.19063914458104	-2.23896834413254	0.0251579759539772	0.166490645571953
**Prkag2**	1338.78263583296	-0.220215097581528	0.0983650820608901	-2.23875274607315	0.0251720084561535	0.166513575669022
**Slc19a1**	5700.60891882863	0.181035647614625	0.0808737935710133	2.2384958046473	0.025188740697525	0.166554337881201
**Vti1b**	5450.74766003233	0.19668672411845	0.0879046593139742	2.23750055632355	0.025253643004033	0.166564107498764
**Rfk**	4737.88600556269	0.135759543813073	0.0606732312445626	2.23755255865394	0.0252502482387153	0.166564107498764
**Trim59**	4724.49130188824	-0.308881922948576	0.138008282260216	-2.23814047888934	0.0252118956714384	0.166564107498764
**S1pr2**	1040.74633785324	0.315789130713309	0.141108056216147	2.23792417797597	0.0252260000439887	0.166564107498764
**Runx3**	11528.4507179605	0.154354993880148	0.0689689832537825	2.23803493393796	0.0252187771070867	0.166564107498764
**Gm43461**	141.930606563867	-0.714353753456219	0.319236123597732	-2.23769711712317	0.0252408133889994	0.166564107498764
**Plek**	4162.2346288637	0.446710518693904	0.199668160155009	2.23726466126151	0.0252690474357427	0.166595975081589
**Gm47483**	186.431090532977	-0.770570889527245	0.344452491349205	-2.23708902934321	0.0252805218414644	0.166601916627334
**S100a6**	1713.39392502784	0.607645688338135	0.271720339928526	2.23629077049577	0.0253327306284885	0.166806450694983
**Mapk11**	1505.30116995465	0.348917442715422	0.156024147760715	2.23630410883921	0.0253318574904528	0.166806450694983
**Tyro3**	296.242727169634	0.40384045179384	0.180614227478305	2.2359282401623	0.0253564721139347	0.16689303721774
**Rad9a**	10693.3494443329	0.162376686626124	0.0726484182882832	2.2351028481003	0.0254105974495431	0.167138287749921
**Abcb9**	5330.01253781236	-0.259718963195473	0.116203451390685	-2.23503656808159	0.0254149481150479	0.167138287749921
**Cdip1**	7824.54368821982	-0.134900076630067	0.060366697330328	-2.23467710833824	0.0254385545257847	0.167223739533913
**Napsa**	641.524229317304	0.471342241117421	0.211081393448717	2.23298810670365	0.0255497286820369	0.167266494706174
**Xk**	187.390910082955	0.587315149879177	0.26308659520939	2.23240241264187	0.0255883785058852	0.167266494706174
**Calm3**	4023.35935997382	0.222570886658695	0.0996721487029561	2.23302988402511	0.0255469737364155	0.167266494706174
**Pisd**	11579.9994051328	0.195456036625229	0.0875727001708827	2.23192885732461	0.0256196652878114	0.167266494706174
**Hspd1**	14089.5341458598	0.135879291056345	0.0608782757865024	2.23198323705598	0.0256160708548503	0.167266494706174
**Toe1**	2499.22328039859	0.225078803426765	0.100846383687133	2.23189761692448	0.0256217304369208	0.167266494706174
**Bcl2l14**	177.818850015576	0.52996544534692	0.237355926398222	2.23278792060904	0.0255629332340581	0.167266494706174
**Ppm1n**	241.135057432998	-0.429773492514567	0.192472115923283	-2.23291301419406	0.0255546811962045	0.167266494706174

**Cul4b**	1334.79633543419	-0.185025402045369	0.0829026173261542	-2.23184029663919	0.0256255199733898	0.167266494706174
**4930590J08Rik**	33.1821087095125	-1.52736360234554	0.684118453830526	-2.23260108508038	0.0255752624901925	0.167266494706174
**Fam222b**	3103.72723106963	-0.205481850278029	0.0920405434335787	-2.2325145268869	0.0255809761994196	0.167266494706174
**AI837181**	2134.53327977505	0.635154607979852	0.284544475634806	2.23218042298256	0.0256030407677043	0.167266494706174
**Mblac2**	1433.20538337471	-0.190363237811455	0.0852915795745726	-2.23191127144052	0.0256208277863492	0.167266494706174
**Zfp874b**	721.437919677976	-0.347514189680737	0.15556757114797	-2.23384724153207	0.0254931259424926	0.167266494706174
**Zfp94**	974.958827652337	0.323433529566956	0.144865544464352	2.23264635329863	0.0255722747733004	0.167266494706174
**Esrp2**	96.9558768038637	-0.980226614195064	0.438840798070024	-2.23367248101361	0.0255046309648213	0.167266494706174
**Gm45756**	22.5144921662258	1.17333901997608	0.525188283543422	2.23413022860985	0.0254745055553849	0.167266494706174
**Stxbp3-ps**	339.681903072131	-0.423059616229377	0.189592716680817	-2.23141280760065	0.0256537972572196	0.167381732249279
**Eif3a**	20738.9588697341	-0.158336377367975	0.0710004619148839	-2.23007531356331	0.0257424433921635	0.167709587386976
**Lrrk2**	133.07932512037	0.654812768859337	0.293635653060214	2.23001792198939	0.0257462530987927	0.167709587386976
**Chd9**	3800.80155724957	-0.177175907370262	0.0794464887794635	-2.23012885896175	0.0257388894379263	0.167709587386976
**Gm29395**	99.1870483581933	-0.606848775920381	0.272127983403865	-2.23001239464505	0.0257466200348477	0.167709587386976
**Gm26724**	80.000844528634	-0.728898512191568	0.326928500529954	-2.22953493198059	0.0257783337536651	0.167846778907645
**Yipf1**	4201.88206594453	0.169287573435716	0.0759976899455075	2.22753577848352	0.0259114873753034	0.168644075412084
**Rpia**	156.169666526054	-0.59956396409337	0.269185615145793	-2.22732542290063	0.0259255326281763	0.168665820653251
**Asb5**	215.007113255058	-0.37972199317757	0.17052806766539	-2.22674189871582	0.0259645284746214	0.168849804034094
**Prdm9**	271.617842510327	-0.383056480877961	0.17205357927676	-2.22637902964976	0.0259888039109484	0.168937946874923
**Gm10392**	78.9035823892893	-0.717196659710753	0.322162461601173	-2.22619561616903	0.0260010814818339	0.16894805810691
**Zfp119b**	670.392476824023	-0.361221679557389	0.162289903467684	-2.2257803587228	0.0260288970562855	0.169059081168958
**Gm47283**	1065.94852175906	0.358819577010457	0.161223483589574	2.22560367150919	0.0260407400588703	0.16906631277611
**Trav6-7-dv9**	326.026933519458	0.451233800951019	0.20280617764993	2.2249509663848	0.0260845300203459	0.169280864716059
**Epn1**	340.101335260755	-0.506480654896453	0.227663707862249	-2.22468771879489	0.0261022092962555	0.169325859152366
**Bcs1l**	1220.5545258355	0.207092143138573	0.0930956481320876	2.22450938678404	0.0261141916654363	0.169333875749909
**Prkar1a**	43632.0556867722	0.136131796532192	0.0612017311193103	2.22431284283134	0.026127403227155	0.169349853002995
**Lad1**	411.619004543592	0.441068430771718	0.198346738061814	2.22372414631926	0.02616700961298	0.169536829963703
**Gm38020**	82.7913329335228	-0.877975349385385	0.394993959324875	-2.22275639578393	0.0262322308079559	0.169889544118767
**Rrp12**	6746.01077251725	0.198277294438	0.089225075826665	2.22221491661366	0.0262687848601964	0.170056385802019
**Fbxl12os**	77.1408689885246	-0.863147007078431	0.388510807500828	-2.22168081405713	0.0263048840495667	0.170184715624176
**Gm16565**	212.51164161481	-0.573111365301619	0.257972105227946	-2.22160207901243	0.0263102092568694	0.170184715624176
**Sp4**	2072.45873907635	-0.318455777139315	0.143371207130631	-2.22119757176319	0.0263375826021485	0.170276894949946
**D830024N08Rik**	56.5446233562043	-1.41749606671201	0.638203511981204	-2.22107218168012	0.0263460728493983	0.170276894949946
**Lmnb2**	2112.59543082479	0.189027410331269	0.0851155287622724	2.22083341406739	0.0263622465052846	0.170311569571041
**Gtf2f1**	7280.63138831052	0.147078963091417	0.0662345259741156	2.22057848121228	0.0263795246329059	0.170353348213401
**Trgc4**	77.4705569051356	1.13314816975967	0.510411063443925	2.22006976516912	0.0264140322150651	0.170506311189177
**Dapk2**	304.335652707648	0.852652152789951	0.384359526908649	2.21837132449329	0.0265295246541581	0.171181703511699
**Tnrc6a**	4135.74891279246	-0.261815508032369	0.118071911635409	-2.21742414775855	0.0265941210948475	0.171528271015765
**Mrgpre**	96.0598001932422	1.90326270911934	0.858762659543428	2.21628489311731	0.0266719969829095	0.171960170400861
**Zeb2**	645.655082013044	1.39645839667394	0.630221128346654	2.21582288162483	0.0267036347330578	0.172093731079261
**Nabp1**	3932.62366564994	0.232577619259343	0.105008127778544	2.21485349924374	0.0267701217237301	0.17245167947703
**Mtss1**	1055.04042447233	-0.501449960979187	0.226449409939133	-2.21440171168461	0.026801157264758	0.172510553521565
**Luc7l2**	2128.08485676866	-0.304653109800303	0.137570950652196	-2.21451627946165	0.0267932840936693	0.172510553521565
**Eif2b1**	4355.43469390707	0.222191285289168	0.100368598224534	2.21375299864312	0.0268457749322419	0.172627188103328
**Etfa**	6393.1781375551	0.144862826095935	0.0654357077276493	2.21381919943268	0.0268412187885158	0.172627188103328
**Mapkbp1**	378.234960334361	-0.682434652669285	0.308286041833805	-2.21364109970694	0.0268534776825273	0.172627188103328
**Pnpla8**	3240.95097837939	-0.211002771399267	0.0953253409583568	-2.21350135523191	0.0268630999066676	0.172627188103328
**Cdk2ap1**	242.601818362117	0.837437832699742	0.378380191052977	2.21321795511883	0.0268826228021691	0.172682220747565
**Gm18959**	58.0417238948651	-0.677075873851725	0.305946576860006	-2.21305262114941	0.0268940180024112	0.172685021052972
**Trip6**	134.130673903225	-0.706923530932946	0.319488644318181	-2.21267185392954	0.0269202772245434	0.17278322127378
**Tspan31**	7110.15857116811	0.210593159958161	0.0952043467529845	2.21201202613744	0.0269658339912432	0.172969943128614
**Ercc6l2**	1864.72943754576	-0.171249009996896	0.077425170408061	-2.21180023362359	0.0269804709738046	0.172969943128614
**Lrrfip2**	902.455015953155	-0.207835340076342	0.0939677213187981	-2.21177375762079	0.0269823012128028	0.172969943128614
**Cyp27a1**	137.022201541557	1.13749853068143	0.514553766911892	2.2106504778075	0.0270600503478857	0.173397809406928
**Fam192a**	7753.66447592393	0.123962455785329	0.0560901259325941	2.21005843228627	0.0271011072442753	0.173449287915534
**Ccdc17**	809.37541559231	-0.237963829757062	0.107668212253182	-2.21015864178639	0.0270941541848208	0.173449287915534

**Rn7s2**	640.143451404498	-0.62863373154719	0.284428291747343	-2.21016597078045	0.0270936457212937	0.173449287915534
**Gm18101**	79.0085950844618	1.17585024891753	0.532098584461584	2.20983532611225	0.0271165930497083	0.173477936128402
**Gpx8**	18.5732588541663	3.85277107947242	1.74380400108747	2.20940603248401	0.0271464118189887	0.173598218762908
**Mkln1**	9116.67902508797	0.155659249125447	0.0704609083397206	2.20915757110242	0.0271636828923993	0.173638195267966
**Arl10**	340.542828544464	-0.372343259795654	0.168577352296005	-2.20873833124308	0.0271928466384146	0.173754129960057
**Zfp207**	16048.8438287166	-0.146467788430831	0.0663562794270968	-2.20729356280063	0.0272935567405205	0.174326945099466
**Pih1d1**	2395.2343440486	0.209580409202994	0.0949583766767081	2.20707657963155	0.0273087096941854	0.174353054558865
**Sptlc1**	3861.76336912439	0.19039891375455	0.0863016627068729	2.20620214932877	0.0273698488629013	0.174636794698941
**Runx1**	3574.24817455422	-0.267368656777793	0.121193847318928	-2.20612401283208	0.0273753178210563	0.174636794698941
**Ltk**	41.5408211241884	1.07243958725347	0.486194132842264	2.20578471604347	0.0273990769431269	0.174656816311916
**Fbxo36**	89.9432888499783	-0.748798086354099	0.339473559374903	-2.20576261589537	0.0274006251141116	0.174656816311916
**Eno1b**	6380.1963318681	0.148338495378451	0.0672639496289616	2.20531943480436	0.0274316870002516	0.174784105160924
**Rpl9**	13961.9248727398	-0.0958612133104989	0.0434858837124106	-2.20442141510719	0.0274947209826575	0.175114922604581
**Cpsf7**	2184.88048987982	-0.14329221318686	0.0650115126456112	-2.20410520161207	0.0275169464297996	0.175172487616533
**Fut11**	2831.4821869288	-0.21538457722976	0.097725439723655	-2.20397654734344	0.0275259934847074	0.175172487616533
**Pip5k1a**	3207.27853602406	-0.239025171778222	0.108461733735659	-2.20377421184112	0.0275402270443549	0.175192312288211
**Ppp1r12c**	2054.11061833871	0.99031657295022	0.449468234478331	2.20330714605364	0.0275731076516746	0.175259966626638
**Abtb2**	13712.8037685811	0.183542382330732	0.0832972899587562	2.20346163028367	0.0275622284888706	0.175259966626638
**Rnf2**	5286.17641270073	0.109393206970028	0.0496601414537164	2.20283720037292	0.027606225166714	0.175399713690287
**Slc39a7**	9828.53813727428	0.230124662230181	0.104498787061156	2.20217543860585	0.0276529183084797	0.175594720984594
**Gm49705**	26.6387925888514	-1.78044627493507	0.808526980145945	-2.20208640979883	0.0276592052728161	0.175594720984594
**Edem2**	7279.32674495668	0.14071917302162	0.063913650420036	2.20170764925526	0.0276859660586351	0.175693824883574
**Slco3a1**	11161.1008141005	-0.257118283240604	0.116803261254997	-2.20129370086062	0.027715238525568	0.175808781581069
**Dnttip1**	3584.55217818459	0.17833669255382	0.0810226378479012	2.20107240754862	0.0277308982839481	0.175837329682161
**Smim3**	231.111880138559	0.567231686402821	0.257740389248829	2.20078695487342	0.0277511095299819	0.175894703485086
**Hivep1**	1634.66012165789	-0.358913258697157	0.163122564614263	-2.20026738511549	0.0277879298496666	0.175986499453857
**Rrp1**	26341.7753794744	0.128801040420344	0.0585376152245102	2.20031239616358	0.0277847383886242	0.175986499453857
**Gm2a**	12828.1127119901	0.210057427142544	0.0955032894946474	2.19947844994718	0.0278439198141102	0.176016877623714
**Relt**	9849.79873142154	0.210654965587577	0.0958434988947555	2.1979056275783	0.0279558317945653	0.176016877623714
**Dennd6b**	3756.34976562641	-0.294927173118392	0.13418685482802	-2.19788423759081	0.0279573564379217	0.176016877623714
**Btg2**	18255.4455272022	-0.201777669958732	0.0917406438956996	-2.19943594671228	0.0278469389868071	0.176016877623714
**Nudt16l1**	10240.1930266679	0.132763676478413	0.0604069447054886	2.19782141152307	0.0279618349918374	0.176016877623714
**Tbl1x**	9934.31270745784	-0.247113829800493	0.112406159750607	-2.19840114054922	0.027920532496903	0.176016877623714
**Eif5b**	6129.37526895987	-0.137451878314107	0.0625138730626211	-2.19874200045835	0.0278962726702084	0.176016877623714
**Tcf12**	6866.24254646772	-0.242842254766739	0.110477647752066	-2.19811210419437	0.0279411181637048	0.176016877623714
**Lxn**	512.80227720686	0.396173218114289	0.180251018071105	2.19789725658031	0.027956428457087	0.176016877623714
**Arid4a**	11189.27516746	-0.206622797102728	0.0939706703609983	-2.19880092702292	0.0278920805665547	0.176016877623714
**Cd177**	124.384257500336	1.06790052962041	0.485752155546675	2.19844733044689	0.027917243984856	0.176016877623714
**Zfp867**	501.044071073514	-0.355720353580158	0.161812539271109	-2.19834850366056	0.027924280412699	0.176016877623714
**Mpp7**	1992.67391630564	0.514055008150468	0.233863373713452	2.19809968524755	0.0279420029555731	0.176016877623714
**Ifi213**	13098.307052573	-0.282485488858128	0.12854080493829	-2.19763279834559	0.0279752839908026	0.176016877623714
**Ccdc61**	2111.44185370113	0.222642069531926	0.101314697368621	2.19752982848944	0.027982628574437	0.176016877623714
**Tgtp1**	44637.2117209451	-0.312940556896162	0.14225939299676	-2.19978836057096	0.0278219141682714	0.176016877623714
**Gm36989**	42.7996640046819	-1.0361120818428	0.471063395068202	-2.19951728937203	0.0278411611431062	0.176016877623714
**Snx2**	7893.47184775856	0.16857838680836	0.0767339751812142	2.19691976611725	0.0280261769108552	0.17622045873278
**Fra10ac1**	1514.26055955893	-0.2469286743118	0.112413561547661	-2.1966092961756	0.0280483617370503	0.176289603466574
**Slc2a4rg-ps**	3175.07618016711	-0.267668020641587	0.121864097674095	-2.19644690889535	0.0280599712506518	0.176292251593509
**Capza1-ps1**	10950.0427015528	0.151027692224293	0.0687898151912709	2.1954949552395	0.0281281123793991	0.176649926967792
**Zfp346**	2310.98949648225	0.31243867128031	0.142355868784209	2.19477197497156	0.0281799587635529	0.176693836146957
**Zfp91**	1817.52309983749	-0.2934167128099	0.133670829584123	-2.19506913903937	0.0281586385644342	0.176693836146957
**Cd300c**	65.5913859436055	0.841426360580642	0.383377208037043	2.19477408395999	0.0281798074040198	0.176693836146957
**Alox5ap**	212.34980256862	0.659697901845882	0.300571270237344	2.19481356726129	0.0281769738648805	0.176693836146957
**Asrgl1**	2622.64801096121	0.286171188359335	0.130445247999079	2.1938030917106	0.0282495684713871	0.177059845029295
**Rtcb**	16223.3712440889	0.138700663340508	0.0632415391002283	2.19318924418787	0.0282937471615922	0.177266232216783
**Rce1**	4574.2518908532	0.185536280557013	0.0846662018228131	2.19138542372903	0.028423913018258	0.177518815566804
**Nudt19**	3638.04838925313	0.15595873130344	0.0711691347687974	2.19138158430749	0.0284241906248047	0.177518815566804

**Rccd1**	1696.7460648328	-0.29813242356405	0.136007983283462	-2.19202150025778	0.0283779542081398	0.177518815566804
**Mcmbp**	12343.7559973052	0.919047093559303	0.419209503724182	2.1923336312623	0.0283554250495199	0.177518815566804
**H2ax**	3127.62866240247	0.180525696788701	0.0823568666103888	2.19199326320568	0.0283799930776376	0.177518815566804
**Usp46**	272.867303232057	-0.473315097725885	0.215965531867194	-2.19162332819361	0.0284067160668367	0.177518815566804
**0610030E20Rik**	5503.48795906376	-0.14099738596498	0.0643285394732727	-2.19183253839552	0.0283916006911251	0.177518815566804
**Gm48069**	66.7462200104566	-1.00942174898096	0.460532490409595	-2.19185783848428	0.0283897732368601	0.177518815566804
**Ppp4c**	23729.754040824	0.130077781462998	0.0593823316993421	2.19051320048517	0.0284870385132144	0.177788404122016
**Mturn**	4574.10482834377	-0.202641985925106	0.092517167211804	-2.19031766786793	0.0285012063725463	0.177788404122016
**Gm22107**	263.823605094511	-0.572965485410863	0.261576255888294	-2.19043385059976	0.0284927872981572	0.177788404122016
**Entpd4b**	3075.27684033052	-0.229546747817757	0.104816584590515	-2.18998499821877	0.0285253248244585	0.177868438171347
**Rabgap1l**	5370.2581325779	-0.160907349731841	0.0734926671348004	-2.18943407560246	0.0285653052160762	0.177998548372566
**Ptges2**	2345.52565820222	0.211153749622	0.096444269384874	2.1893861705703	0.0285687839585085	0.177998548372566
**Trav9n-3**	606.8871745363	-0.26934250042075	0.123063629842439	-2.18864420597373	0.0286227101623073	0.178264049022718
**Ndufs7**	9120.69891809537	0.129330986612763	0.0591029264386742	2.18823321290119	0.02865261897096	0.178309366953166
**Tmco4**	6539.5446857175	0.183462898760328	0.083840462715246	2.18823814681745	0.0286522597602047	0.178309366953166
**P2ry14**	179.567337413673	0.640218723025243	0.292621553237672	2.18787275216616	0.028678872588048	0.178402287947048
**Eif3c**	18174.2689531701	0.113610649917762	0.0519364497960672	2.18749356884932	0.0287065121911265	0.178503753984049
**Rassf3**	482.767652192769	0.82336571892458	0.376485083219053	2.18698098709402	0.0287439119947808	0.178569103765692
**Arpc4**	16983.322532689	0.133195373879506	0.0608997649776439	2.18712459610315	0.0287334295379033	0.178569103765692
**Jmjd7**	724.957981251019	0.365059760560276	0.166931497456455	2.18688363863448	0.0287510196264238	0.178569103765692
**Zfand3**	1765.79097916949	-0.223223623460818	0.102091347529043	-2.18650873814076	0.0287784060992776	0.178668772618722
**Actn1**	5267.9669250283	-0.25578897811427	0.117005512504505	-2.18612758184724	0.0288062725820441	0.178771341896522
**Dennd4c**	3169.07319776094	-0.309822983094695	0.141764807692589	-2.18547175520833	0.0288542746985422	0.17899874268698
**Tbc1d30**	358.43504868232	0.415140873682738	0.189998236819074	2.18497224307431	0.0288908818268505	0.179084824919624
**Gm48226**	842.138078713325	-0.301552122711783	0.138010228631883	-2.18499835628936	0.0288889671099532	0.179084824919624
**Gm45250**	137.125735576256	-0.845870576559871	0.387163479100902	-2.18478917103496	0.0289043084096608	0.179097596386561
**Tcf19**	1091.5663308496	0.34148419505938	0.156327735892222	2.18441208215803	0.0289319811998928	0.179151450048265
**Zfp128**	228.183281942055	0.467662857736266	0.214095966458826	2.18436089885984	0.0289357390602801	0.179151450048265
**Mocos**	259.983852718309	0.582381882588008	0.266671940818497	2.18388886659954	0.0289704153188602	0.179225298068033
**Ap5s1**	2197.89700437872	0.161950778212041	0.0741550503392154	2.18394805844257	0.0289660650290402	0.179225298068033
**Mtres1**	949.490395710914	-0.276664785696577	0.126708740151949	-2.1834704169957	0.0290011852129583	0.17927488246394
**Ankrd10**	22879.2139735899	-0.117499917134369	0.0538116871241227	-2.18353899336666	0.0289961406537493	0.17927488246394
**Tuba1c**	12276.1741373741	0.153422949250199	0.0702900149601868	2.18271328206574	0.0290569311877184	0.179478661201442
**Gm12000**	176.367445896622	-0.47412501652876	0.217213725625761	-2.18275808843513	0.0290536296398321	0.179478661201442
**Akt2**	13237.6220972527	0.215663519949424	0.098860327900545	2.18149711344661	0.0291466677284476	0.179700990478869
**Fam71b**	1018.49848283966	-0.309885701858229	0.142024880266488	-2.1819113755053	0.0291160741315714	0.179700990478869
**Copg1**	26417.1637252322	0.136314329867312	0.0624814755328114	2.18167590801738	0.0291334602093245	0.179700990478869
**2410131K14Rik**	2437.3781651787	0.21223896377111	0.0972797653712554	2.18173803114274	0.0291288723923511	0.179700990478869
**Gm5165**	1213.68509225848	-0.323445393626079	0.148270641425659	-2.1814527172478	0.0291499480652401	0.179700990478869
**Mmp8**	46.06196583294	1.56003174518267	0.715321693856174	2.18088135531416	0.0291921930966243	0.179750444167061
**Msl3l2**	716.832973083904	0.295048931816764	0.135279179538243	2.18103726548219	0.0291806602765542	0.179750444167061
**Exoc8**	1109.52733351043	0.607669876846952	0.278632054716549	2.18090440981434	0.0291904874867389	0.179750444167061
**Gm37716**	18.0139895558958	-1.25238260958168	0.574392341211047	-2.18036091313677	0.029230719091907	0.179917359660617
**Gmps**	6863.87869106121	0.115864633414831	0.0531663789167764	2.17928389661817	0.0293105847528463	0.180291254300285
**Gm14288**	335.348404105868	-0.382046551616722	0.175337165751099	-2.17892510113377	0.0293372327236105	0.180291254300285
**Gm3448**	89.6635951820239	-0.819743627008975	0.376189553943198	-2.17907067970513	0.0293264179978941	0.180291254300285
**Gm47689**	39.3313400084271	-1.14391838247655	0.524984141453144	-2.17895797634993	0.0293347901930946	0.180291254300285
**Gm49331**	234.46437606942	-0.586091863462082	0.269046601111609	-2.17840277870283	0.0293760632029941	0.180459504050518
**Mad1l1**	3600.52561782517	0.221004581728253	0.101485596451895	2.17769407142432	0.0294288205915914	0.180713143437921
**Ube4b**	3734.99645412721	-0.315753202352414	0.145021187395993	-2.17729014650957	0.0294589259066622	0.180827540129052
**Ahi1**	894.542696927377	-0.21063631501999	0.0967633354874175	-2.17681949427405	0.0294940379541279	0.180972568552645
**Letm1**	15519.577330931	0.172520314425609	0.0792670190526847	2.1764450901193	0.0295219953127515	0.181073600678484
**Ly9**	31437.0884635177	0.130118714669977	0.059811188118719	2.17549122100208	0.0295933252809384	0.181316712620168
**Slc4a2**	2728.85190411185	-0.277701160319666	0.127652081376247	-2.17545344600499	0.0295961531293439	0.181316712620168
**9330104G04Rik**	26.8837356471077	-1.20311025453767	0.552964366252158	-2.17574644581898	0.0295742251575536	0.181316712620168
**Tmem184a**	18.899925433663	-1.71027449894367	0.786279413986481	-2.17514851402821	0.0296189889460094	0.181386089709394

**Trgc2**	373.983751161708	0.860659122310874	0.395708123271966	2.17498472155284	0.0296312613294351	0.18139074777308
**S1pr5**	1222.53143605337	1.78644086096181	0.821455281347745	2.17472685552747	0.0296505911686593	0.181438588366821
**Kat2b**	1934.10824375851	-0.20205222121444	0.092951518179725	-2.17373772017112	0.029724838138203	0.181484586615004
**Eef1e1**	2648.74684324973	0.225233716852709	0.103608597186266	2.17389022696434	0.0297133801838072	0.181484586615004
**Prr13**	15661.2551996116	0.33115762828953	0.152311178524211	2.1742174901292	0.0296888054654167	0.181484586615004
**Prkab1**	8363.41814162322	0.123528959600445	0.0568282808026596	2.17372332676064	0.0297259197225452	0.181484586615004
**Nlrx1**	503.94114943647	0.35536395120754	0.163482980340388	2.17370609752548	0.0297272144477582	0.181484586615004
**Rfx3**	1504.25219880132	-0.517071793567803	0.237835707489085	-2.17407133279822	0.0296997784942827	0.181484586615004
**Arhgef11**	1166.48319535156	-0.469815269671672	0.216206543612664	-2.17299283278562	0.0297808567207838	0.181741657377765
**Gm13213**	51.515387272415	-1.03903795543334	0.478281556779324	-2.17243993774309	0.0298224953630341	0.181882412449209
**Gm37963**	52.38267648528	-0.777523181270705	0.357913052726434	-2.17238006646546	0.029827007283668	0.181882412449209
**Nt5c3**	5026.05055586705	-0.162693563947511	0.0749107188932588	-2.17183290123453	0.029868269046408	0.182063564937815
**Yars**	5404.14092460721	0.216485620869306	0.0997052546724137	2.17125588396097	0.0299118351051205	0.182245863932949
**Gm37490**	23.0171105163568	-1.36875317611796	0.630433392628184	-2.17113051453672	0.029921307989753	0.182245863932949
**Gm47917**	70.8953112529409	-0.697763673659311	0.321446361069448	-2.17070017945719	0.0299538436300842	0.182373537124899
**Nudt1**	580.727941557451	0.263561743814747	0.121439792286677	2.17030792668494	0.029983526532627	0.182483749546004
**2700049A03Rik**	1788.29266420965	-0.317810447753272	0.146450464828956	-2.17008835120089	0.0300001534803835	0.182514447254982
**Phxr4**	208.023517988629	-0.57683084816382	0.265938004171221	-2.16904255546881	0.0300794532345719	0.182926261913219
**Psat1**	2002.40578077878	0.299892466410302	0.138299046440255	2.16843480941755	0.0301256196264046	0.183136338137831
**Senp6**	5389.16183610303	-0.158917476018285	0.0733070643130484	-2.16783303911405	0.0301713920664042	0.183334177109543
**Uggt1**	16827.5702157228	-0.149821778626233	0.0691155146099133	-2.16770112284954	0.030181433992648	0.183334177109543
**Tor1a**	6897.68117059552	0.189859173480489	0.0876409701592484	2.1663289798767	0.0302860567225761	0.183898803767873
**Siglech**	933.324520292758	0.704149761202314	0.325079768923731	2.16608300028513	0.030304844998088	0.183942004096638
**Fen1**	4088.92189181692	0.212821878513233	0.0982816201767305	2.16542908155701	0.030354840956656	0.184103629312559
**H2-Ob**	9383.95457919572	-0.25059394294157	0.115721111386395	-2.16549893048323	0.0303494972145299	0.184103629312559
**Gm17816**	25.1646412456501	-1.22535268584673	0.56605066286553	-2.16474030724318	0.0304075784427899	0.184352525403247
**Cks1b**	672.453279332653	0.395184160193159	0.182572933902297	2.16452763148693	0.0304238783151705	0.184380404081593
**Siae**	3047.66327612845	-0.172940965251863	0.0799205995770353	-2.16390975752335	0.030471275930663	0.184531238356702
**Tmem237**	481.782276205172	0.319443100756429	0.147623927289502	2.16389786277651	0.0304721890083226	0.184531238356702
**Pitpnm2**	861.807999506973	-0.402337009845348	0.186008543265768	-2.16300285342535	0.0305409601504184	0.184805648652129
**Prkaa1**	4735.87826415857	-0.181851700640227	0.0840722337306513	-2.16304114415277	0.030538015222541	0.184805648652129
**Ppic**	2678.13450002931	0.350464563525637	0.162053649496755	2.16264530057779	0.0305684712033709	0.184901113532251
**Zranb1**	1147.21409453265	0.520789854417493	0.24084092177266	2.16238108783311	0.0305888140967291	0.184953163362303
**Lrmp**	9126.37007615262	-0.124545359512679	0.057612737738354	-2.16176776875795	0.0306360810364757	0.185167905213559
**Clec10a**	68.123914754864	1.1549537925209	0.534354034548318	2.16140183819734	0.0306643122386637	0.185196461458269
**Coq7**	717.556003339784	0.247676736296268	0.114584894345875	2.16151297874093	0.0306557354878917	0.185196461458269
**Npm3**	1794.25974619303	0.16172098066944	0.0748418660827287	2.16083576123926	0.0307080285592163	0.185389427589108
**H2bc4**	475.744819331235	0.475677793376348	0.220232284863352	2.15989128783499	0.03078108636827	0.18562794584782
**Nop2**	9169.25909157359	0.175962531813648	0.0814796532304184	2.15958861921073	0.030804530234336	0.18562794584782
**Fcnaos**	24.3807390666077	-0.867905028332108	0.401816021352958	-2.15995625413287	0.0307760562578257	0.18562794584782
**Atad2b**	3017.13154246384	-0.268985810574611	0.124555901280684	-2.15955894348561	0.0308068296581234	0.18562794584782
**Trbj1-1**	565.357624325412	-0.203126890160927	0.0940650768938278	-2.15942937451907	0.0308168710369492	0.18562794584782
**4933406I18Rik**	101.304335426398	-0.654642783526731	0.303157894724984	-2.15941195963444	0.0308182208756677	0.18562794584782
**Gm13423**	24.38241743568	-1.83585519200854	0.850419446366297	-2.15876436016704	0.0308684527749934	0.185859461358644
**Sass6**	524.714752807136	-0.367532379944259	0.170307062411423	-2.15805718647407	0.030923385898117	0.186119095338667
**Lman2**	21684.2458322333	0.165201303058697	0.0765803796739662	2.15722752697265	0.0309879406583713	0.186436418844581
**Cyld**	13715.7644263675	-0.173380619585826	0.0803879488509455	-2.1567986503463	0.0310213563378849	0.186505740295441
**Zfp760**	371.486118366478	-0.426427342321423	0.197715189928827	-2.15677582726409	0.0310231354518215	0.186505740295441
**Carhsp1**	1127.87361057011	0.475482671343514	0.220572062913524	2.15567948661717	0.0311087010407904	0.186595901432879
**Pdcd4**	19966.6380142178	-0.166767627528593	0.0773593465968028	-2.15575279348966	0.0311029733813484	0.186595901432879
**Ndufa10**	12309.2013725876	0.144395551441345	0.0669658660519071	2.15625601451073	0.0310636798200773	0.186595901432879
**AW011738**	1213.84763034652	0.537954851841442	0.249551970791027	2.15568264252228	0.0311084544429493	0.186595901432879
**6030443J06Rik**	49.6604135671338	-1.06019141890812	0.491814517681532	-2.15567328899923	0.0311091853185361	0.186595901432879
**Gm29585**	46.1674025954944	-0.798217200179476	0.370242283011511	-2.15593203911466	0.0310889722594473	0.186595901432879
**Mrpl12**	4067.45356664257	0.22978907143145	0.106607257257582	2.15547306386693	0.0311248342586273	0.186618726565141
**Arc**	362.144834315982	0.414624873356773	0.192397961639944	2.15503776559082	0.0311588790555209	0.18675179052029

**Pttg1ip**	12987.886514988	0.129722075053817	0.0602053583939003	2.15465995908696	0.0311884533048994	0.186786947444051
**Gm49484**	231.658654383856	-0.463488327921359	0.215097789073697	-2.15477960009416	0.0311790853422657	0.186786947444051
**Faf1**	3340.615074005	1.49736475492392	0.695342747132884	2.15341967842194	0.0312857104144763	0.187298229103687
**Ccdc102a**	2722.42174797761	0.320505391532201	0.148880608727101	2.15276787401971	0.0313369261504183	0.187533591094623
**Gm29394**	44.414789787634	-0.66989441159684	0.311283650717006	-2.15203853480199	0.0313943194688952	0.187651960600065
**BC106179**	572.800042682433	-0.475838812416394	0.22111325933307	-2.15201392196758	0.0313962578792541	0.187651960600065
**Gm44639**	55.7613384720035	-0.770055780268417	0.357846115612245	-2.15191879043012	0.0314037510316412	0.187651960600065
**Gm48045**	29.5324091493036	-1.15316285200103	0.535878434942199	-2.15191128585985	0.0314043422036156	0.187651960600065
**Calm1**	45810.1621286007	0.184825062308511	0.085919269832666	2.15114796329708	0.0314645227752936	0.18794029013279
**Slc41a3**	535.144659255593	0.888085968333487	0.412898770777221	2.15085641127436	0.0314875349154784	0.188006475052366
**Oas2**	404.779566008208	-0.37476637901881	0.17428264847921	-2.15033672192281	0.0315285896893911	0.188180298384748
**Crip1**	11453.051883152	-0.170713942689179	0.0793978486785221	-2.15010791262609	0.0315466798746371	0.188199008558878
**Bmi1**	1615.52846986748	0.453624412595058	0.210988591863912	2.1499949764471	0.0315556121477791	0.188199008558878
**Stard3**	5150.19534198124	0.168571747362068	0.0784195255781738	2.14961447572167	0.031585722425707	0.188268668145425
**Rnaseh2c**	2382.82593908017	0.198158564957152	0.092186264988314	2.14954543371804	0.0315911885877073	0.188268668145425
**Nfya**	497.91622452673	-0.495024937034081	0.230334712366225	-2.14915473203625	0.0316221363311779	0.1883333018805
**Kif21b**	8907.96636216655	-0.347547644376784	0.161717254964168	-2.14910675087697	0.0316259387431493	0.1883333018805
**4931406C07Rik**	145.969134905177	-0.786933319476013	0.366235908802197	-2.1487060677631	0.0316577073999562	0.188451263884061
**Kansl3**	7716.07963405033	-0.201760777070962	0.0939086216017566	-2.14847980547068	0.0316756589733193	0.188486917840858
**D330041H03Rik**	273.613909726567	-0.5105703728306	0.237661725235644	-2.14830710466469	0.0316893668702509	0.188497302293146
**4833422C13Rik**	23.9702262845282	-1.30998821728478	0.610006847889306	-2.14749755976919	0.0317536912531097	0.18880864644349
**Xxylt1**	1952.14050099618	1.00703480219503	0.468988885344292	2.14724662708317	0.0317736524438012	0.188856069995087
**Pold2**	7207.17278339981	0.110295606103536	0.0513777884880176	2.14675659169835	0.0318126647964483	0.18901665128116
**Stc2**	41.2717196788567	1.34090900855057	0.624968906562542	2.14556115427541	0.0319080074564652	0.189226373162033
**Son**	23473.5031432197	-0.266144190875169	0.124021167852842	-2.14595778674625	0.0318763467330377	0.189226373162033
**Coq10b**	1510.37754916254	0.394427260636223	0.1837857959187	2.14612483333969	0.0318630204963476	0.189226373162033
**Ppa2**	2785.60653248273	-0.211491090519372	0.0985651630109649	-2.14569817630033	0.031897066786899	0.189226373162033
**Phlda3**	133.449045786281	0.666887760841743	0.310818718482283	2.14558429459504	0.0319061595672486	0.189226373162033
**Mob4**	4848.23675631133	-0.168915628133233	0.0787372746159299	-2.14530702208301	0.0319283074286913	0.189275523007483
**Mindy1**	2718.04842811573	-0.138746128784087	0.0646861226723988	-2.14491336088829	0.0319597747765177	0.189390812769308
**Engase**	1493.45478788901	0.288085075735435	0.134325294576881	2.14468225543737	0.0319782605963063	0.189429117374436
**Impdh1**	14149.6867087469	0.179307189175967	0.0836317271011858	2.1440091624441	0.0320321526452929	0.189645866956596
**Psip1**	9959.28962870373	0.429736801587807	0.200443982652119	2.14392468110972	0.0320389222465228	0.189645866956596
**Wbp4**	2197.57008120626	-0.198900358385921	0.0927820281640659	-2.14373798807466	0.0320538865560545	0.189663195818156
**D630008O14Rik**	156.616634638033	-0.589411859752401	0.275042997889861	-2.14298078582036	0.0321146412532233	0.189951352187327
**Sirt7**	29545.3282203821	0.147804828261014	0.0689838779932324	2.14259958356523	0.0321452646362679	0.190061138789371
**Pkp4**	2339.90666807285	-0.287524011423012	0.134228806471027	-2.14204401411462	0.0321899403918379	0.190253897507198
**Mbd1**	3832.62831601355	0.148995013691502	0.0695845062270038	2.14120961360909	0.0322571381278064	0.190579574608116
**Ankrd12**	8563.52634913687	-0.150074808877894	0.0701037893611388	-2.14075173746723	0.0322940638455859	0.190726223393889
**Gchfr**	20.9501389429165	1.16281287864859	0.543269640275541	2.14039731367802	0.0323226714439078	0.190823654530407
**Gm5431**	727.679359421365	-0.385764092479176	0.18030185746227	-2.13954585886561	0.0323914860479817	0.191144491691193
**Zfp213**	1595.51531944691	0.217216590145217	0.101530368048906	2.13942482746233	0.0324012779911897	0.191144491691193
**Tet1**	1329.25837511063	-0.477234640521463	0.223145988929333	-2.13866555617362	0.0324627640651551	0.191435543927638
**Gm45203**	58.0780324391149	-1.09149470297861	0.510405325410081	-2.1384861180703	0.0324773096233267	0.19144966993444
**Zcchc4**	1321.08200089112	0.290949136989169	0.136073039344341	2.1381835695821	0.0325018473614748	0.191522665996544
**Aldh18a1**	9124.81991275755	0.191372702151775	0.0895143510213187	2.13789967718358	0.0325248864656918	0.191586779828002
**Slc35b3**	6883.47222840197	-0.171902373200306	0.0804235975294823	-2.13746187040798	0.0325604438123511	0.191724556484012
**Isoc1**	6743.9927472683	0.288618190938232	0.135132348552451	2.135818655044	0.0326941979647785	0.192368363454449
**Rufy3**	451.026944322707	0.54028222729586	0.252948238994608	2.13593986439011	0.0326843157393993	0.192368363454449
**Iqgap2**	6302.50651736783	-0.193954017107325	0.0908242280847268	-2.13548764682472	0.0327211981757518	0.19245536381311
**1110059E24Rik**	2750.95591504929	0.206987867129082	0.0969379322716584	2.1352618348514	0.0327396285244112	0.192468271222875
**Lgals4**	1346.31948826939	-0.324286427944865	0.151889751034284	-2.13501191315844	0.0327600370260678	0.192468271222875
**Got2-ps1**	298.820652213365	0.491419382395834	0.230162597590544	2.13509661230911	0.0327531193085961	0.192468271222875
**Vps35**	11812.0303876001	0.132181560354498	0.0619258392632148	2.13451383020684	0.0328007427637535	0.19248399505675
**Tmem87a**	1238.86225603315	-0.258110145789551	0.120924551451431	-2.13447263348518	0.0328041114965572	0.19248399505675
**Siah1b**	265.817404688865	0.57563108109501	0.269694578944967	2.13438135592807	0.0328115764880643	0.19248399505675

**9330162G02Rik**	527.148750187189	-0.548856265117429	0.25711810128125	-2.13464653940121	0.0327898928992355	0.19248399505675
**Polr2d**	1745.09323491666	0.354030172006992	0.1658959389507	2.13404965936027	0.0328387160183111	0.192558841971564
**Vim**	9758.61939890098	0.152467152045571	0.0714540998270659	2.13377752171777	0.0328609967672486	0.192558841971564
**Stoml2**	5357.55173004365	0.178865078968913	0.0838217757290354	2.13387365530309	0.0328531245357619	0.192558841971564
**Acap3**	3112.44321886506	0.245386307701808	0.115033387514434	2.13317466349514	0.0329104007296405	0.192695079566753
**Hoxb4**	30.8612884466026	1.06709146349712	0.500274441869641	2.13301215130869	0.0329237294069329	0.192695079566753
**Cdc42se1**	32753.6831421176	-0.120531757657335	0.0565024835199261	-2.13321167758632	0.0329073656101975	0.192695079566753
**Mmgt2**	1288.06814335059	0.207075030871457	0.0970862704004046	2.13289716473231	0.0329331629925282	0.192695079566753
**Sh3bp1**	13152.4198893826	0.174857520514667	0.0820087692251295	2.13218076757926	0.0329919888904238	0.192967620247368
**Glmp**	11647.0009191744	0.151866972803558	0.071241754426837	2.13171298244095	0.0330304489013816	0.193013793538811
**Mplkip**	3785.25995304535	-0.169947143537754	0.0797186569869584	-2.13183651056184	0.0330202890308981	0.193013793538811
**Zfand5**	2351.90002337845	1.24162504093311	0.582500643021642	2.13154278163944	0.0330444518631659	0.193013793538811
**Txndc5**	12619.6676544742	0.239916940207166	0.112572520181052	2.13122118809549	0.0330709242594969	0.193013793538811
**Fgl2**	698.436094020806	1.1857203630564	0.55635247611492	2.13123948209314	0.0330694178781957	0.193013793538811
**4930549G23Rik**	91.966968063615	-0.586442533193921	0.275171221681346	-2.13119137099676	0.0330733796125397	0.193013793538811
**Gm1826**	37.6423239056012	0.865257958976992	0.406146675698457	2.13040758609927	0.0331379780450754	0.193319185507683
**Dennd3**	986.45215860041	0.282893148955135	0.132813777688975	2.12999851278696	0.0331717361503163	0.193444502783321
**Kansl2-ps**	1585.38797181429	0.232966368855101	0.10938409546146	2.12980111845588	0.0331880363188864	0.193467957187086
**Mdn1**	5465.77599118292	-0.415764820141368	0.195311118021294	-2.12873094145126	0.0332765273533581	0.193840385030264
**Gm10698**	2157.69859506311	0.297849986941095	0.139913430951241	2.12881626099852	0.0332694650326269	0.193840385030264
**Rmnd5a**	2255.75219487783	-0.273456634327033	0.128493100755364	-2.12818145658779	0.0333220416743567	0.194001131878927
**Sf3a2**	837.48030222675	-0.306094413214318	0.143845750784413	-2.12793503836671	0.0333424700088328	0.194001131878927
**Pbxip1**	15145.4479742942	-0.171157690747837	0.0804401904129257	-2.12776337138474	0.0333567077174466	0.194001131878927
**Trim5**	3990.88186846672	-0.209813934399998	0.0986127525417548	-2.12765518649485	0.0333656830229036	0.194001131878927
**Copz1**	14837.3160875091	0.121510745179116	0.0570993967977166	2.12805654689464	0.0333323954829109	0.194001131878927
**Nsd1**	15350.2211066531	-0.234193894154218	0.11013659503531	-2.12639490152328	0.0334703919370306	0.194394753318021
**BC055324**	356.735355699855	0.290693855830602	0.136702715666544	2.12646730837218	0.0334643685187492	0.194394753318021
**Cntrl**	15373.0328863787	-0.149589205282141	0.0703429827737808	-2.12656898219986	0.0334559119892949	0.194394753318021
**Cct3**	29303.0274790067	0.129054962947973	0.0606965327654909	2.12623286813752	0.0334838746141042	0.194401404677391
**Ggcx**	595.28521781238	0.402485633858896	0.189357986501519	2.1255276383902	0.0335426103850566	0.194670685759608
**Pwp1**	6491.07308474756	0.138620945015416	0.0652289268435863	2.12514526488866	0.0335744935790237	0.194736762673293
**BC005537**	3455.94590974144	-0.268704707550645	0.126455195987245	-2.12490048710809	0.0335949173266934	0.194736762673293
**Mrps5**	2709.14478634471	0.174752227469471	0.0822352561611523	2.12502806736587	0.0335842709704234	0.194736762673293
**Ap3m2**	4862.28747332394	-0.168956052221033	0.0795218106926761	-2.12465046695163	0.0336157894568355	0.194736762673293
**9930012K11Rik**	848.923189054403	0.312895047625022	0.14726530278265	2.12470311548421	0.0336113933414626	0.194736762673293
**Atm**	4509.01779856648	-0.362336704120961	0.170554298187939	-2.12446539296062	0.0336312469077978	0.194754706918842
**Gm13270**	36.3769996513278	-1.16765255372035	0.549778844571762	-2.12385864834408	0.0336819650929698	0.194976753846409
**Snhg20**	3025.20908540402	-0.236125351678064	0.111206170771831	-2.12331159358538	0.0337277498267902	0.195170089614665
**Dnajc13**	3241.78768269972	-0.27008483738734	0.127216168594014	-2.12303860721717	0.033750616814035	0.195223338927813
**B3galt4**	2768.59665669015	-0.297333521301935	0.140059674812234	-2.12290598061537	0.0337617312038008	0.195223338927813
**Mdh2**	31030.477408787	0.166167520277991	0.0783039581620922	2.12208327877907	0.0338307453061698	0.195431758232126
**Npc1**	1965.88683752169	-0.299877447355466	0.141299096125065	-2.12228850416738	0.0338135182530343	0.195431758232126
**Gm7334**	49.3218875531728	0.890337952433448	0.419568421202644	2.1220328019001	0.0338349835918792	0.195431758232126
**Nr3c2**	135.377714671231	-0.732736640446509	0.345393226104542	-2.12145631433064	0.0338834205194088	0.195496542337724
**Trpm4**	503.887111653779	-0.316534652512194	0.149203224241216	-2.1215000823337	0.0338797410196737	0.195496542337724
**Iqcc**	2737.26998611597	0.159227377438039	0.0750540870992752	2.12150175416066	0.033879600478869	0.195496542337724
**Cd83**	1300.29115262221	0.419033076612879	0.197556482440366	2.12107986251156	0.0339150822775551	0.195607595698183
**Pitpnm1**	19352.2288069031	-0.201452219911078	0.0950033505918312	-2.12047489542332	0.0339660164677982	0.195780140628493
**Zc3h7a**	10660.0846986701	-0.177696339901924	0.083802053957189	-2.12042941086744	0.0339698486055912	0.195780140628493
**H4c14**	312.800554748245	0.86982392363192	0.410274786540834	2.12010084988576	0.033997541310256	0.195868101800989
**6430511E19Rik**	193.36617287534	-0.90231215891028	0.425680998728664	-2.11969094604909	0.0340321170388503	0.195995638955104
**Parp14**	3409.67529962236	-0.304876327986939	0.143877377710645	-2.11900114415542	0.0340903702566939	0.196187715169732
**Ube2d2a**	7005.02054656935	-0.155431341477966	0.073350700760601	-2.11901644928051	0.0340890768271494	0.196187715169732
**Ccdc91**	2957.77532154286	0.169869967676511	0.0801723054186227	2.11881106311623	0.0341064374195954	0.196208519321126
**Ylpm1**	2332.61507513417	-0.316285081565535	0.149311456436207	-2.11829078032379	0.0341504489730556	0.196356188677416
**Tppp**	179.162355051636	-0.69691320420289	0.329009968112534	-2.1182130383494	0.0341570294576885	0.196356188677416

**Zmat3**	554.330837454378	-0.274188972070339	0.129493025565601	-2.11740339584108	0.0342256262464675	0.196364814665135
**Sik3**	813.276379615869	1.02960532409057	0.486209503265962	2.11761661829831	0.0342075496089482	0.196364814665135
**Bcl3**	5799.52673262483	1.2013671720046	0.567441187604944	2.11716596934976	0.0342457644665731	0.196364814665135
**Kdm5d**	3366.53645262322	-0.240713748752033	0.113689609088905	-2.11728891216256	0.0342353353460645	0.196364814665135
**Gm15712**	65.2685801720687	-0.906530907558693	0.428069183301508	-2.11772055294198	0.034198741164964	0.196364814665135
**2610021A01Rik**	1905.16205792521	-0.215424443111958	0.101750615803044	-2.11718073066948	0.0342445121346184	0.196364814665135
**Rn7s1**	622.643092976764	-0.60426561248032	0.285338931598833	-2.11771176507339	0.0341994858603543	0.196364814665135
**Sytl1**	2557.21509503535	-0.221537004960099	0.104680282010004	-2.11632029171383	0.0343175760830785	0.196705000487838
**Sec13**	11464.7359002392	0.141878479617507	0.06704562379188	2.11614825238885	0.03433220072617	0.19671726794264
**Lekr1**	25.255564132026	-1.35077731053632	0.638596027235507	-2.11522974294697	0.0344103711204742	0.197093499725668
**B3gnt3**	633.857041262077	0.273732114959765	0.129441257207265	2.11472076883074	0.0344537531670167	0.197199994300125
**AC163703**	48.3387182244372	1.31860365768232	0.623536420688083	2.11471794418555	0.0344539940539597	0.197199994300125
**Slc25a22**	3584.44138170548	-0.164110269212583	0.0776110954039354	-2.11452071844177	0.0344708171074108	0.197224642397049
**Nol8**	4185.1951894864	-0.207806601057015	0.0982983831618098	-2.11403885163546	0.0345119491283579	0.197330314108819
**Cyfip1**	1071.09292014945	0.364251673480555	0.172303589722363	2.11401093887528	0.0345143330382626	0.197330314108819
**Anxa7**	5431.03383143645	0.175877081296379	0.0832052672047275	2.11377340888325	0.0345346251553916	0.197374714752813
**Dhps**	2899.53545847458	0.169555221085697	0.080230640458497	2.11334747070114	0.0345710384531355	0.197511186695452
**Sipa1**	33596.6714678141	0.164670506136887	0.0779375637448851	2.11285159843984	0.0346134717016157	0.197650367306706
**Gm15492**	21.9444487114761	-1.14014843144355	0.539646346066105	-2.11276966805199	0.0346204870068229	0.197650367306706
**Zfp597**	806.598407617298	-0.256230442716435	0.121305237838783	-2.11227847438021	0.0346625710311911	0.197818953907453
**Gm37902**	43.3020643385015	-1.37155347160261	0.649535274338461	-2.11159197319886	0.0347214616300851	0.198083298662292
**Vps26a**	5027.23842222296	0.174656441549357	0.0827266205480986	2.11124835502991	0.0347509705378695	0.198108192027934
**Pyroxd1**	1338.55916546445	-0.181281972998573	0.0858632912313098	-2.11128609675829	0.0347477283436333	0.198108192027934
**Brat1**	8918.90342952486	-0.151415992129976	0.0717519805804685	-2.11026916476774	0.0348351779949461	0.198516419409174
**Gm37760**	87.6697102025389	-0.691176695794417	0.327658862002892	-2.10943995706216	0.0349066235924085	0.19885165146261
**Plaur**	1120.6686012805	0.287286274962354	0.136217564492494	2.10902519093404	0.0349424072916455	0.198983560424452
**Fastkd3**	1946.78102801623	0.319849505743737	0.151727554095136	2.10805155102669	0.0350265305666685	0.199309236613187
**Crmp1**	628.081879518064	0.635359494687304	0.301414769438622	2.10792422637632	0.0350375442926019	0.199309236613187
**Tbc1d8b**	442.012182959878	-0.404975111855129	0.192110104012577	-2.10803650300776	0.0350278320832528	0.199309236613187
**Adsl**	9391.09728409352	0.14702400005566	0.069769855651632	2.10727109412073	0.0350940874618514	0.199558836570333
**Zfp267**	802.001972958295	-0.445711967570885	0.211661018089494	-2.10578202634568	0.0352232908014441	0.200149077951084
**Esm1**	70.1676430128094	1.52266195163857	0.723044363931141	2.10590390797042	0.0352127001504215	0.200149077951084
**Commd1b**	100.301632930937	6.53439868811174	3.10396447300207	2.10517831146174	0.0352757894469142	0.200375131332021
**Gmnn**	720.207954124363	0.310600765609032	0.147566579906518	2.1048178104134	0.0353071702115003	0.200481110278418
**Gm15753**	413.28108956644	-0.324011203180987	0.153980926626735	-2.10422946711071	0.035358435272571	0.200699879175036
**Igkv5-39**	36.5871953204592	2.45877867999878	1.168595934595	2.10404521118834	0.0353745033953046	0.200718779258126
**Atpaf2**	4666.67583870086	0.206145165890154	0.0979891840802125	2.10375428497707	0.0353998864356977	0.200790500564179
**Gm42576**	161.952387982421	-0.768629936645842	0.365591492164538	-2.10242840197144	0.0355157653999083	0.201375284421143
**Elmo3**	848.690199810734	-0.338893800148561	0.161299255451335	-2.10102519816533	0.0356387543470512	0.201999946851254
**Hrh1**	46.3285217915009	1.91329776242655	0.910761387306379	2.10076732401361	0.0356613961478049	0.202055598382223
**4931422A03Rik**	70.1460049411318	-0.603321141693801	0.28730245842337	-2.09995119778873	0.0357331343391088	0.202316564060847
**Gm50287**	272.657614625354	-0.384965997117323	0.18331864595135	-2.09998276563469	0.0357303572117898	0.202316564060847
**Trio**	406.64949213235	-0.350843806884539	0.167110537949482	-2.09947147073753	0.0357753601697909	0.202482884409265
**Eftud2**	17366.9073420103	0.106586672735404	0.050772552119606	2.09929712582332	0.0357907166421687	0.202483148519169
**Gm26510**	1515.38968828373	-0.279235240968727	0.133021155141219	-2.09917919200358	0.0358011075569211	0.202483148519169
**Rab7**	33017.3063386094	0.116021024260055	0.0552877480353007	2.09849430267944	0.0358615026657808	0.202751954612382
**Il21r**	41424.414298754	0.231833909548319	0.110508571923428	2.09788168929517	0.0359155979118936	0.202768195439601
**Znrf1**	7061.54035652452	0.837307425876048	0.399120963084169	2.09787884706891	0.0359158490494337	0.202768195439601
**Cxcr1**	21.8952709762459	2.9154466041204	1.38953526767375	2.09814509350397	0.0358923301607713	0.202768195439601
**Ethe1**	1564.5779092819	0.218148221347308	0.103967027984825	2.09824427585974	0.0358835722418161	0.202768195439601
**Sft2d3**	577.429795338713	1.22121065415411	0.58229794801173	2.09722644279268	0.0359735347652794	0.203021127255196
**Xpr1**	1599.23933630047	-0.232626649675721	0.110943833580352	-2.09679656965556	0.0360115873944644	0.203049041048847
**Tpcn2**	1402.3277268269	0.263149905748628	0.125504675806503	2.09673387909738	0.0360171396669117	0.203049041048847
**4732496C06Rik**	78.9129495647794	0.832502963805263	0.39702455340865	2.09685511049082	0.0360064033066659	0.203049041048847
**Pabpc4**	643.326273940716	-0.275268615088636	0.131346928600081	-2.0957369770462	0.0361055297538405	0.203432927543205
**Tbcd**	5495.81337118586	0.165271051556872	0.0788684037611694	2.09552930800207	0.0361239659367828	0.203432927543205

**Tmed9**	12860.8107010722	0.196875840954978	0.0939467700649545	2.09561053369753	0.0361167540280867	0.203432927543205
**Atxn2**	903.822318968243	-0.422280317887662	0.201541009198545	-2.09525753377397	0.0361481052928772	0.203496139728427
**Tsen54**	3602.86386961229	0.197674366297449	0.0943754924159073	2.09455189305196	0.0362108454717739	0.203776532892407
**Tcf7**	55797.9064103724	0.213851549515603	0.102120181982004	2.0941164162172	0.0362496110425301	0.203875361568831
**Oma1**	2335.35609115865	0.164129177284075	0.0783783025993765	2.09406394168813	0.0362542846427533	0.203875361568831
**Ciao3**	4292.03070617283	0.188416405569294	0.0900047928042629	2.09340413658916	0.036313093476783	0.20396159028578
**Vdac2**	9333.70935969843	0.158030055351617	0.0754789638018894	2.09369667244507	0.0362870095509864	0.20396159028578
**Dnajb13**	489.447096291247	0.383104166881071	0.183026109527599	2.09316675019693	0.0363342717507238	0.20396159028578
**Ogt**	30991.1378798944	-0.232594525911732	0.111120917610524	-2.0931659935258	0.0363343392734774	0.20396159028578
**Igha**	1442.20195791382	1.49963062382703	0.71629982611262	2.09357948886512	0.0362974562934869	0.20396159028578
**Map1s**	9796.6693554629	-0.126321041074814	0.0603595205550669	-2.09281054443713	0.0363660701664039	0.203994363692426
**P2ry6**	131.548154724876	0.877490209338469	0.419266966837467	2.09291520378384	0.0363567247882796	0.203994363692426
**Tor2a**	4968.02901929077	0.281266636364355	0.134413646817684	2.09254523646591	0.0363897695629927	0.204054661567287
**Eri3**	4337.00876855142	0.0937451693626326	0.0448195926283203	2.091611366039	0.0364732949532158	0.204450269860484
**Ywhah**	24896.2900266061	0.185966475265089	0.0889605362837993	2.09043788441005	0.0365784827411671	0.204894117508912
**Gm28229**	34.5497035813133	-0.960233589221939	0.459334894279225	-2.09048692181052	0.0365740819893222	0.204894117508912
**Bod1l**	8794.54611008856	0.277450981088995	0.13276987395007	2.08971337272891	0.0366435550138166	0.205185677452988
**Golga1**	2210.9396377706	-0.248750284696351	0.119059873634034	-2.08928732329214	0.0366818668240155	0.205294841402843
**Was**	1683.66850981776	-0.200722490049025	0.0960825882272859	-2.08906206371347	0.0367021367158601	0.205294841402843
**Lats1**	970.04981492653	-0.245459084230235	0.117492004456735	-2.08915564395382	0.0366937147775835	0.205294841402843
**Tpm2**	21.7161371014031	1.31374396873086	0.628922983335854	2.0888789303941	0.0367186229245103	0.205314173676902
**Nrbp1**	18532.3079688556	0.133740682302277	0.0640344510829989	2.08857388546874	0.0367460979854642	0.205394915202894
**Scyl1**	6831.71474769869	0.0957325256578413	0.0458455751653944	2.08815191678745	0.036784133106293	0.205470906044796
**BC002059**	1262.6147723523	-0.183411920327076	0.0878353323723615	-2.08813373130457	0.0367857730502233	0.205470906044796
**Washc5**	3549.93771238118	-0.174737529729373	0.0837049670362886	-2.08754075076116	0.0368392814213896	0.205696866533251
**Pitpnc1**	11285.7297141929	-0.196395538532064	0.0941160176555095	-2.08673872337996	0.0369117588616571	0.205955589370797
**Stn1**	3429.85338452173	0.226283915302817	0.108438965463686	2.08673989405216	0.0369116529821664	0.205955589370797
**Khdc4**	9082.77046755594	-0.173160689354066	0.0830087343272266	-2.08605384430335	0.0369737459603913	0.206228430123145
**Gm45762**	145.341717329057	-0.733793927560809	0.35183093316744	-2.08564358157669	0.0370109204958206	0.20636272974262
**1700109H08Rik**	151.699114613291	-0.669204636871475	0.320890667439048	-2.08545995498167	0.0370275694913116	0.206382530058223
**Lins1**	1618.87079880807	0.197612226206521	0.0947778782384567	2.08500369368196	0.0370689652515907	0.206540199953789
**Pdcd7**	1226.17194037883	0.422081166923445	0.202456293189746	2.08480141700442	0.0370873300512694	0.206569480246678
**Rbm48**	2935.53700892874	0.240655009158441	0.115471270440453	2.08411155641128	0.0371500211097464	0.206772477084519
**Gm36948**	22.5355618393408	1.33134586508162	0.638766140722608	2.08424614300239	0.037137783478863	0.206772477084519
**Ddx43**	249.953075668223	0.490362019363412	0.235302570563794	2.08396371611448	0.0371634678246515	0.206774280548388
**Ncf1**	5684.30307175618	-0.395862032329121	0.190000199350058	-2.0834821946675	0.0372072930150138	0.206801173895488
**Rfc2**	7287.51147628629	0.179064316892758	0.0859449039019749	2.08347800466437	0.0372076745569402	0.206801173895488
**Cd151**	970.487526186562	0.356476145211526	0.171092206312976	2.08353234138223	0.0372027269107472	0.206801173895488
**Anxa2**	5272.88291536675	0.714391225696505	0.342950124266204	2.08307615349333	0.0372442826293778	0.206875631268155
**Gtf3c3**	2087.94147789969	-0.151625931917408	0.0727906009818197	-2.08304272628932	0.0372473291811739	0.206875631268155
**Pde4a**	114.99666529769	1.19111024523106	0.571862491394572	2.08286128773084	0.0372638691730065	0.206894568907352
**Dhrs4**	2656.23041436841	0.218843309756418	0.105088755200448	2.08246171856345	0.0373003160613249	0.206951084569823
**Cep164**	1794.28520303137	-0.288883766451567	0.138714234376177	-2.08258199132	0.0372893421347705	0.206951084569823
**Camk1d**	4192.11378928153	-0.245085511133778	0.117700279326168	-2.08228487253291	0.0373164568390962	0.206967761497233
**Kcnk5**	547.986379738769	0.63938578553046	0.307104715335736	2.08197970790343	0.0373443232658788	0.207049437614515
**Mtx1**	2893.54414023118	0.160865558373859	0.077297086395854	2.08113353134728	0.0374216854279919	0.207405380685497
**Gcfc2**	885.048261342368	-0.29083773931442	0.139780548591595	-2.08067390094581	0.0374637645300881	0.20756558990879
**Vps72**	5208.28225095851	0.129853111486302	0.0624184812049071	2.08036320300747	0.0374922316894287	0.207584942029767
**Mypopos**	1307.19011907315	-0.280825064188888	0.134989451572982	-2.08034821177904	0.0374936057001153	0.207584942029767
**Pdcd11**	2785.14922686454	-0.209736958345232	0.100848169027628	-2.07972995808951	0.0375503086523494	0.207825856492824
**Ino80b**	1977.79683025676	0.172929596000878	0.0831617516907452	2.07943666992433	0.0375772329975648	0.207901847030417
**Ilrun**	3988.91376355699	0.493840774229735	0.237513340133363	2.07921278843725	0.0375977967477494	0.20794260559996
**Wdr73**	4368.12257435173	0.150679954833256	0.072488840498223	2.07866416123665	0.0376482292199373	0.20814847291879
**Btbd19**	2460.77530943249	0.237798795194526	0.114416292637201	2.07836480026978	0.0376757721953936	0.208227689401198
**Fam49b**	15293.1104355193	0.368566022169435	0.177363518744521	2.07802610580999	0.0377069547532402	0.208289311112754
**Sf3a3**	7725.65089352346	0.108623761137806	0.0522743173343943	2.07795656981897	0.0377133594341998	0.208289311112754

**Gm38162**	142.411479836352	-0.537786001976215	0.258830109469548	-2.07775673038336	0.0377317709945244	0.208317980224499
**Slc29a1**	5191.61657934879	0.196085359628859	0.0944232256735861	2.07666448831891	0.0378325363556351	0.208801147234936
**Gm44597**	850.943840653293	-0.405580114576912	0.195323365861355	-2.07645466679498	0.0378519197375609	0.208834978748161
**Tars**	7283.52742381583	0.244476735415188	0.11776378740473	2.07599246596045	0.0378946478054296	0.208997537791443
**C530005A16Rik**	34.5978857709808	-0.979840065481268	0.472132812022955	-2.07534837767986	0.0379542588371492	0.209253063854779
**Ccdc86**	2980.53304596847	0.222341808032719	0.107162409582017	2.07481157711884	0.0380040011960325	0.20937057612538
**Gp1bb**	90.6496764381686	1.22135102834414	0.588670095221222	2.07476316235354	0.0380084902508361	0.20937057612538
**Gm10517**	52.4996030323843	-1.08042266911059	0.520763859672068	-2.07468826617681	0.0380154355711565	0.20937057612538
**Slc10a1**	58.8768179983465	-0.701870053444154	0.338350219206003	-2.07438923814269	0.0380431759888205	0.20945014816766
**Dync1h1**	7272.96925643047	-0.293515930804584	0.141573279469779	-2.07324384872527	0.0381495914906936	0.209834762215554
**Vapa**	9759.55607202447	0.156422222361445	0.0754490567286865	2.07321640778011	0.038152144067374	0.209834762215554
**Mirt1**	2446.96685671782	-0.294852446777998	0.142220432086772	-2.07320736164056	0.0381529855776836	0.209834762215554
**Gcm2**	18.7518342781418	1.59571283848064	0.769948710085399	2.07249238498451	0.0382195456785679	0.210080605727837
**Exosc3**	3977.45161164171	0.206884755808219	0.099826617416939	2.07244080949009	0.0382243508676594	0.210080605727837
**Cmklr1**	37.2569940518651	2.16507275395698	1.04503645338007	2.07176768518864	0.0382871116701072	0.210205581388808
**Mterf2**	685.308696941324	0.26858922322106	0.129630402718081	2.07196165088823	0.0382690177063535	0.210205581388808
**A430035B10Rik**	129.021246898702	-0.593375434650139	0.286397004999456	-2.07186326774355	0.0382781944050853	0.210205581388808
**Dot1l**	1694.58290354064	-0.207850758854866	0.100347402428216	-2.07131180105588	0.0383296671615212	0.210365923184984
**Spidr**	2186.96740343249	0.252740432854602	0.122046739250725	2.07084953195994	0.0383728597230374	0.210529648557068
**Txn2**	17959.4842130673	0.157986600716314	0.0763311249030027	2.06975334003101	0.0384754488922326	0.210551599257875
**Cd7**	18502.3069413345	0.412500101287196	0.199290227826113	2.06984610227409	0.0384667585449726	0.210551599257875
**Dnajc6**	88.4474866840709	-0.722666393451376	0.349170752088288	-2.06966473889729	0.0384837509677665	0.210551599257875
**Tnpo2**	2040.25779423855	-0.231608867931295	0.111859628895549	-2.07053134556314	0.0384026138123719	0.210551599257875
**Angptl6**	747.521482933028	0.217245279605229	0.10493497315406	2.07028479710269	0.038425682413469	0.210551599257875
**1110051M20Rik**	1166.9514713535	-0.225483628203339	0.108928979161653	-2.07000588767765	0.0384517931052276	0.210551599257875
**Gm45871**	1259.95196554636	0.332868659921366	0.160778849871264	2.07035104547578	0.038419482648347	0.210551599257875
**Gm4977**	25.8538921585845	-0.866265049120363	0.418553340090431	-2.06966464282235	0.0384837599709766	0.210551599257875
**Fads3**	37.6350869715193	1.66829828224095	0.806176676332257	2.06939537103823	0.0385090005417666	0.210616564226524
**Zfp687**	4558.18161310549	-0.303849580930329	0.146872488922866	-2.06879847382381	0.0385650017119849	0.210823759249709
**Gm26583**	101.733857374558	-0.925582133568327	0.447420726066646	-2.06870643142816	0.0385736433278487	0.210823759249709
**Sdcbp**	36580.0330627218	0.148411313882052	0.0717632172549586	2.06806940322617	0.0386334973325603	0.211077675960177
**Fbxo17**	547.875103094296	0.444519610226239	0.214967824033382	2.06784253515638	0.0386548324915206	0.211099476865773
**Trim12c**	3986.38812368926	-0.190233291469062	0.0920004929728318	-2.06774208835206	0.0386642819213442	0.211099476865773
**Tlk1**	4187.59475083051	-0.248344208203597	0.120130099356672	-2.06729378843057	0.0387064792086877	0.211256665358951
**Pacsin2**	4095.59409832123	0.209858921709161	0.101521163719926	2.06714456394645	0.038720533993112	0.211260198798291
**Nkd2**	87.5912867361777	-0.798917738409054	0.386577398514844	-2.06664368242516	0.0387677414852625	0.211371384976914
**Fastk**	8116.91567208239	-0.131809126145627	0.0637783862949092	-2.0666738969554	0.0387648924161502	0.211371384976914
**Ipmk**	831.32561851832	1.05330795147874	0.509751759509783	2.06631547969874	0.0387987007165237	0.211467010442845
**Hba-a2**	395.390634747407	0.641256965524911	0.310413374132728	2.06581616309714	0.0388458413369531	0.211650733729727
**Cacna2d4**	1566.41828751542	-0.2807713694445	0.135940122360735	-2.06540471325629	0.0388847229920671	0.211789346763222
**Gm20531**	215.276602896836	-0.475870899918372	0.230458115171711	-2.06489105217149	0.0389333099176803	0.211980706417722
**Wdr33**	12776.2305979132	-0.175988670249916	0.0852594878401621	-2.0641535001928	0.0390031647435369	0.212141134575047
**Dus2**	1827.8894947203	0.230419203568783	0.111623882755789	2.06424644870036	0.0389943555707658	0.212141134575047
**Gm28053**	312.557563348358	-0.269475930985133	0.130533438553285	-2.06442068769321	0.0389778466648133	0.212141134575047
**Ctnnd2**	39.2635541830898	2.27800119496034	1.10367927828578	2.06400649154027	0.0390171009046564	0.212143705643434
**Plpp6**	833.871640503313	1.55197599133375	0.75204119543788	2.06368480975315	0.0390476105246874	0.212236357032597
**Gm7901**	267.784236061927	0.435752881067601	0.211215554368606	2.06307192843923	0.0391057947836356	0.212479313243361
**Ptger1**	110.431806651691	0.87077771655041	0.422236688145643	2.0622976188418	0.0391794095975323	0.212805914177289
**Chmp2a**	7214.76184325037	0.191788830189511	0.0930171733894415	2.06186474175618	0.0392206151533188	0.212956317357286
**Ifi214**	8240.28828121088	-0.257114665241947	0.124738994922889	-2.06122123559589	0.0392819384954997	0.213215812908574
**Insr**	1767.85717909904	-0.457574375552501	0.222078060786526	-2.06042133982946	0.0393582785660285	0.213448552750191
**Slc41a2**	50.4021136615756	1.77245039512043	0.860268238283681	2.06034619929319	0.0393654562602053	0.213448552750191
**Lin9**	1167.87769640865	0.244441766727715	0.118625999424705	2.06060870225054	0.0393403858733724	0.213448552750191
**Gm10069**	179.421852890386	0.582971567248362	0.283005336165549	2.05993136082545	0.03940510312941	0.213585906698482
**3000002C10Rik**	312.609073578371	-0.397844690075701	0.193147471207729	-2.05979756083797	0.0394178978662934	0.213585906698482
**Trappc3**	4442.90816646358	0.138606032469563	0.0673085594233119	2.05926309606261	0.0394690416996579	0.213750330835618

**Ercc2**	2910.38805644498	0.194592949626872	0.0944994348708217	2.05919696655198	0.0394753736581613	0.213750330835618
**Klra3**	216.709722326818	0.568860981462529	0.276313642724256	2.05875097535527	0.0395181002284265	0.213908177705021
**Tbc1d15**	8899.38370336284	0.16501714643056	0.080175648504487	2.05819534370619	0.0395713854161026	0.214049543426546
**Tkfc**	494.075876705051	-0.356714802924855	0.173307717821059	-2.05827419234246	0.0395638201048817	0.214049543426546
**Uevld**	406.494299623231	-0.387905298582946	0.188498479933627	-2.05786963756702	0.0396026490434635	0.21414514103564
**Fam193a**	2241.22267268176	-0.296017739321938	0.143874833158242	-2.05746712488875	0.0396413140775945	0.214245311408877
**Gm20683**	178.676041904777	0.774389177385068	0.376419159966159	2.05725228613412	0.0396619644208728	0.214245311408877
**A530064N14Rik**	32.3270521813036	-0.944474892205009	0.459091785724668	-2.05726811407478	0.0396604427246392	0.214245311408877
**Sp1**	2599.31274312135	-0.307910522654073	0.149684361044147	-2.05706541756396	0.0396799336880136	0.214268922248811
**B3gnt7**	40.2209442891437	0.957800135016468	0.465696119157907	2.05670628466565	0.0397144872787232	0.21438204044222
**Rusc1**	5036.73366738365	-0.169605171944838	0.0824894589031609	-2.05608297350996	0.0397745191197833	0.214412281139386
**Nipal1**	1186.77116955816	-0.275804035981255	0.134131695609028	-2.05621821694686	0.0397614871258525	0.214412281139386
**Ighg3**	164.495477933307	1.40363902051689	0.682616288140008	2.05626359186582	0.0397571156318588	0.214412281139386
**Gm16185**	51.1990239382295	-0.90811829605479	0.441595574899156	-2.0564479077087	0.0397393625346641	0.214412281139386
**Tsr1**	8552.2133056772	0.146417788683959	0.071239721862899	2.05528299178006	0.0398516791330966	0.214754756532217
**Bckdha**	6058.08008981865	0.173444095707393	0.0844034385721896	2.05494110952657	0.0398846931518656	0.21478575187763
**Gm5511**	620.843294889984	-0.371563103337102	0.180804341467257	-2.05505631292814	0.0398735658905368	0.21478575187763
**Gm15872**	17.3707627984162	-1.61119726961355	0.784117050244926	-2.05479178027092	0.039899120480569	0.214790038063658
**Tespa1**	10477.6099470011	-0.125819482107631	0.061238398110944	-2.05458480281746	0.0399191247662015	0.214824333654726
**Plscr1**	313.105541739384	0.772404110519477	0.37602029280416	2.05415538815551	0.0399606546582205	0.214974406094087
**Phip**	10555.4533639268	-0.156001415136407	0.0759502246083857	-2.0539954416301	0.0399761329001132	0.214984275121872
**Idh3g**	21623.7926589792	0.0872833280070303	0.0425051130938868	2.0534783148144	0.0400262107722519	0.215180144366555
**Tlr2**	551.441712240465	0.417499727673647	0.203349772491657	2.05311135861131	0.0400617785932828	0.215224495497565
**Gm42670**	28.8015945672903	0.86736844529364	0.422439069392369	2.05323917255393	0.0400493869793624	0.215224495497565
**Il12rb2**	242.287089183463	0.858313044530743	0.418150099279611	2.05264340725841	0.0401071744230613	0.215394937758752
**Col4a3bp**	2183.54103058854	-0.215262878485884	0.104900318849559	-2.05207077391825	0.0401627847819301	0.21562010214953
**Pakap**	2745.46003032348	-0.345107715723507	0.168249545825507	-2.05116580868171	0.040250802354299	0.216019037022033
**Thoc7**	1898.8193671709	-0.188695843029632	0.0920077024587163	-2.05087006834346	0.0402796017153363	0.216099994630083
**Tmem241**	2425.92061276606	0.227981495199831	0.111186529964162	2.05044167916127	0.0403213494838953	0.216250341667032
**Polr2e**	10460.4862363623	0.105209969674463	0.0513207079720496	2.05004907047974	0.0403596425567324	0.216382064568367
**Prx**	57.4364001232767	1.11355526677006	0.543299135544363	2.04961722542467	0.0404017981578134	0.216534399174376
**Pepd**	9245.86509736634	0.120084228430206	0.0586044525327418	2.04906322370499	0.0404559330427417	0.216677136966173
**Gm14036**	413.943503176995	0.581489444304727	0.283765486985791	2.04919016220547	0.0404435236797387	0.216677136966173
**Hdac5**	2782.87752704172	-0.263924722313822	0.12886755477292	-2.0480308079011	0.0405569807876473	0.217079885290145
**Traf3ip2**	7461.96802908613	-0.176271297621171	0.0860930342788252	-2.04745133096703	0.0406137908126767	0.217079885290145
**Clu**	586.280954731992	0.367991771272114	0.179724479700608	2.04753282293614	0.0406057975324061	0.217079885290145
**Akap7**	66.7980968545336	0.790207730227893	0.385924916996676	2.04756856949638	0.0406022916902367	0.217079885290145
**Gm7488**	88.1522537472287	0.714298669876572	0.3488104662317	2.04781317944196	0.0405783084663874	0.217079885290145
**Armcx3**	576.068806882698	0.3473971391172	0.16965932040265	2.04761600065783	0.0405976402774803	0.217079885290145
**Trpm2**	187.92580678449	1.03133138668389	0.503790883682875	2.0471418203214	0.0406441618523954	0.217094935019727
**Bivm**	637.524701011041	0.191314809073231	0.0934536658572187	2.04716216660273	0.0406421647623076	0.217094935019727
**Naa16**	2507.89715549139	-0.148326971130954	0.0724748351840151	-2.04659963357417	0.0406974108667251	0.21712395989316
**Stat1**	63314.8649175735	-0.249347350789034	0.1218384720205	-2.04654036326949	0.0407032354804563	0.21712395989316
**Trim62**	755.833230421288	1.3965142257423	0.682252658033967	2.04691650416812	0.0406662833305808	0.21712395989316
**Stat4**	16293.8171350481	-0.142660941125504	0.0697088571249204	-2.04652531987222	0.0407047139382112	0.21712395989316
**Fbxl22**	256.283156440806	-0.425193935691897	0.207789975311482	-2.04626780023685	0.040730029902257	0.217185475861206
**Mmp15**	250.247822380974	-0.409741044174644	0.200317689365904	-2.04545612258038	0.0408099107753695	0.217442925099666
**Setd1a**	854.57460147995	0.545448214145319	0.266675954977441	2.04535956078777	0.0408194226898674	0.217442925099666
**Pigm**	1097.21830347274	-0.177150117600565	0.0866108696471657	-2.0453566431354	0.0408197101253292	0.217442925099666
**Cd86**	2402.65766802078	-0.263088343150753	0.128664757778736	-2.04475839144069	0.0408786837397033	0.217602594594099
**Aldh9a1**	7858.38967383171	0.121958830633082	0.0596448633756453	2.04474993705629	0.0408795176612258	0.217602594594099
**Rsrc1**	1892.34441826958	-0.20163424321152	0.0986211983950594	-2.04453247874568	0.0409009722161998	0.217602594594099
**Izumo4**	3961.947953694	-0.217092093005476	0.106189788164207	-2.0443782472734	0.0409161945598501	0.217602594594099
**Trav16**	669.501496038261	0.250547215112404	0.122555778436805	2.04435252509613	0.0409187337553033	0.217602594594099
**E2f4**	13371.7594427671	0.116849991359663	0.0571796712889958	2.04355829135644	0.0409972032287508	0.21781685837804
**Prf1**	18407.9973786985	0.224015140737527	0.109621923252401	2.0435250002113	0.0410004951417951	0.21781685837804

**Gm47242**	82.1653211795881	-0.612150474971551	0.299521688651841	-2.04376009539364	0.0409772531255342	0.21781685837804
**Ube2n**	8917.91443960598	0.165348930642533	0.080966762786079	2.04218280381789	0.0411334015926498	0.218375676851544
**Gm12250**	2698.41845191521	-0.441030489717929	0.215954556006535	-2.04223748678209	0.0411279796740974	0.218375676851544
**Ighv1-53**	40.490889141379	-1.49623366177886	0.732782432997101	-2.041852525939	0.0411661621393636	0.218475990848755
**Fam174c**	685.312666823691	0.264250853472373	0.129449558256387	2.04134225741427	0.0412168195653199	0.218619703451566
**Ldha**	73389.3323065967	0.112782364303317	0.0552502568094139	2.04130027290842	0.0412209899698295	0.218619703451566
**Rpap2**	1770.98453546443	-0.208235092808369	0.102064371189219	-2.04023294693423	0.0413271296887783	0.218961527406214
**Strip2**	41.0129112123639	1.63295551779678	0.800341951308236	2.04032228365333	0.0413182367740101	0.218961527406214
**Gm6344**	315.62246502426	0.654841047502993	0.320957665342678	2.04027234184869	0.0413232079702632	0.218961527406214
**D030028A08Rik**	1730.66036399508	-0.24361792656304	0.119416040634746	-2.04007707229372	0.0413426499216091	0.218964977060879
**Gm43775**	77.4267953926341	-0.659083944015938	0.323088708886287	-2.03994731443217	0.041355573505945	0.218964977060879
**Kif5b**	15495.6469461415	-0.126746518029826	0.0621549648992819	-2.03920182780589	0.0414298885716492	0.219284767962203
**Mvp**	4749.46629107198	0.151043887393057	0.074078308536305	2.03897592125814	0.041452430754406	0.219330406782127
**Znrd1as**	393.415885904072	0.358377698817037	0.175783340501425	2.03874666276541	0.0414753180304844	0.219377840284103
**Spred2**	545.512052450899	-0.466239537585893	0.228740175663691	-2.03829316923927	0.0415206226020317	0.219543775281951
**Gm10252**	28.3741582114922	1.17349174370604	0.576044075026223	2.03715617360115	0.0416343941595287	0.220071502439347
**Slc25a24**	605.341181770777	0.430847815989642	0.211574234317832	2.03639076080697	0.0417111324597237	0.220403190532483
**Celsr2**	125.317687435823	-0.557613849632194	0.273858364982432	-2.03613955581735	0.041736343702973	0.220462476610039
**Nasp**	2873.14208629056	-0.175301155378773	0.0861015192248513	-2.03598214011741	0.0417521487107664	0.220472053363119
**Fmr1**	1840.00840255205	0.394922346994106	0.194008207335766	2.0355960833689	0.0417909314153333	0.220487968875323
**Ndufv2**	7059.10602878799	0.190688574111136	0.0936717545328772	2.03571049845349	0.0417794342625894	0.220487968875323
**Lrrc23**	190.214591406461	-0.45663394988195	0.224331248958535	-2.03553429137442	0.0417971417801987	0.220487968875323
**Zfp933**	468.915956885819	-0.295965219163776	0.145432746992485	-2.03506586573016	0.0418442460122791	0.220662578452303
**Zyg11b**	4269.69364730375	-0.206496084337931	0.10149376715307	-2.03456911818534	0.0418942473373146	0.220852343691558
**S100a1**	86.1440421847766	0.633393597062055	0.311392194808316	2.03407024203659	0.0419445138078716	0.221043379287837
**Eif3g**	13840.8379047914	0.141404939797215	0.0695243463427017	2.03389096389625	0.0419625902321174	0.221064705545795
**Nnt**	2176.89381585964	-0.329632374758375	0.162103244322986	-2.03347179222146	0.0420048805960733	0.221139626980396
**Gm46218**	62.4353076189298	-1.20194410395255	0.591067767903508	-2.0335131929386	0.0420007020598814	0.221139626980396
**Noct**	796.681529038518	0.733767347243578	0.36086986656291	2.03332950526547	0.0420192441696095	0.221141359512537
**Neb**	1413.69521876824	1.28123393303528	0.630338110540493	2.0326137855391	0.042091557645869	0.221242151381411
**Gtf2h5**	1748.75716489518	0.207791947563561	0.102220329713743	2.03278494743129	0.0420742545551383	0.221242151381411
**E130308A19Rik**	744.430655480581	0.597447149781163	0.293922139473204	2.0326714784125	0.0420857246877499	0.221242151381411
**Zfp934**	379.805609259923	-0.267780288948369	0.131743768415679	-2.03258409994367	0.0420945592334499	0.221242151381411
**5430400D12Rik**	39.0108627649778	-1.65513077600177	0.814371935241023	-2.0324015408413	0.042113022240078	0.221265385607505
**Itgb2**	84362.0260470265	0.154375913824364	0.0759729762679594	2.03198454776702	0.0421552202895359	0.221296600486234
**Fam184b**	21.4960330316578	-1.18872871421685	0.585015262930253	-2.03196188123825	0.0421575150774063	0.221296600486234
**Aak1**	5841.94804076801	-0.317334802200325	0.15617435012416	-2.03192651000655	0.042161096316537	0.221296600486234
**D10Wsu102e**	3389.0483059189	0.166790845823418	0.0821188665796079	2.03109045181143	0.0422458198668181	0.221667460420064
**Pros1**	509.024821704623	0.646565544057446	0.318360643906831	2.0309217123165	0.0422629368625766	0.221683454109061
**Il1b**	93.3328318055855	1.00251548601862	0.493797716394621	2.03021490933234	0.0423346989884741	0.221910609684107
**Arid4b**	11315.3495736561	-0.142068597322229	0.0699792105023851	-2.03015433158376	0.0423408542761165	0.221910609684107
**Krtcap2**	4017.21643052413	0.196299507064979	0.0966954936594332	2.03007916538858	0.0423484929440954	0.221910609684107
**Srp68**	9602.78475310229	0.117322223997556	0.0578227591783043	2.02899733019963	0.0424585622451357	0.222303401173074
**Tnrc6c**	2798.52787640942	-0.313066800193246	0.154301695463598	-2.02892650824504	0.0424657763235992	0.222303401173074
**Cdc42se2**	41045.5833765672	-0.114421501213826	0.056394177179112	-2.02895949435373	0.0424624161577914	0.222303401173074
**Akap13**	58899.4515851344	-0.287280489140335	0.141603038757165	-2.0287734759209	0.0424813680696225	0.2223048459793
**Gm38118**	1198.10280569862	-0.397668663929709	0.19602655635786	-2.02864689008635	0.042494268965517	0.2223048459793
**Stimate**	1531.10921578658	0.187757122131141	0.0925701861051565	2.02826773965708	0.0425329296107749	0.22237084981382
**Kpna3**	3060.52617478613	-0.130528162969302	0.0643595710424471	-2.02810803203171	0.0425492233412876	0.22237084981382
**Gm18180**	213.377421333486	-0.319910362008099	0.157728987530454	-2.02822808297258	0.0425369749822191	0.22237084981382
**Dnajb11**	7089.60321809858	0.172386636830486	0.0850146950151916	2.02772752169118	0.0425880651326666	0.222399117023805
**Cenpk**	95.7892876666177	0.70507106388976	0.347753554426347	2.02750210577385	0.0426110893123607	0.222399117023805
**Spr**	4084.88524107358	0.186737593920712	0.0920842524663099	2.02789933044233	0.0425705235019845	0.222399117023805
**1600023N17Rik**	30.4459838524646	-1.00402559618499	0.495184348681116	-2.02757942341904	0.0426031908344569	0.222399117023805
**Itgb7**	138655.717614324	0.111473154898296	0.0550237104242368	2.025911266958	0.042773878443941	0.222987963411757
**Aim2**	969.662753493457	-0.281807064605645	0.139091798206185	-2.02605091198766	0.0427595676701897	0.222987963411757

**Bri3**	203.063218405984	0.632095800609391	0.312032872213217	2.02573464816704	0.0427919840752207	0.222987963411757
**Rnu3b3.1**	68.0064373585045	0.971528481527989	0.479521851384747	2.02603589121631	0.0427611067992558	0.222987963411757
**E230034D01Rik**	42.1160974874551	-1.00768867243343	0.497449999225182	-2.02570846115789	0.0427946691221141	0.222987963411757
**Pnpo**	4137.35779370538	0.148630377668959	0.0733973181535418	2.02501101413589	0.0428662332709128	0.223176216072634
**Opn3**	60.4304804763788	0.828607009032857	0.40915481484752	2.02516744020636	0.0428501737967384	0.223176216072634
**Npepl1**	8061.16477718445	-0.128948209023258	0.0636799421022453	-2.02494230940438	0.0428732884465229	0.223176216072634
**Tmem219**	1908.57987689043	0.190713701116772	0.0942023259802027	2.02451159387351	0.0429175402845525	0.223332788065949
**Gm4951**	58.3940108439096	-0.929677483271464	0.459302854285378	-2.02410560830921	0.0429592867010595	0.22347622335708
**Gm44624**	23.3623500422048	-1.08267876175003	0.535233743127199	-2.02281484613486	0.0430922404575639	0.22409387224087
**Slc29a2**	326.672897680014	0.298565230207816	0.147644688998797	2.02218740296339	0.0431569951651624	0.224356573017969
**Zfas1**	645.676120308112	0.358473392070474	0.177304926001688	2.02179037071459	0.0431980129868278	0.224495742293353
**Nt5c2**	2590.13125278374	-0.135345424163691	0.0669766868798776	-2.02078410367524	0.0433021190785625	0.224814329595028
**Car9**	57.5063676068134	0.793522970169195	0.392650357692008	2.02094039805161	0.0432859353263504	0.224814329595028
**Fbxl2**	203.910873754809	0.866994592212677	0.429033113190221	2.0208104352735	0.0432993921728762	0.224814329595028
**Gm48296**	44.3970452707127	-0.789600871751114	0.390797881483163	-2.0204840127444	0.0433332068080736	0.224827573156015
**Gm10366**	168.431404276624	0.354769713570483	0.175576674385092	2.02059706856257	0.0433214926491608	0.224827573156015
**Gpatch3**	1505.45762606992	0.297532280144689	0.147307914055739	2.01979833909065	0.04340430950827	0.225048274077595
**6430590A07Rik**	839.561140850887	-0.2713450191588	0.13434021671238	-2.01983460946579	0.0434005458905712	0.225048274077595
**Ubtd1**	627.730364302558	-0.384434501868439	0.190462031787183	-2.01843117109028	0.0435463757466303	0.225710606131465
**Top3b**	8621.14085783922	-0.127785275154352	0.0633266586663046	-2.01787490206466	0.0436042915264803	0.225936475364272
**Kdm7a**	3804.2139545455	-0.282574763252148	0.140055616180695	-2.0175896615783	0.0436340144776331	0.225941888308927
**Gm12924**	17.1565247713128	-1.27829042520089	0.633572995524245	-2.01758981874406	0.0436339980957587	0.225941888308927
**Msh3**	3710.49564306558	-0.189014655267817	0.0936997925632118	-2.01723664585814	0.0436708235127994	0.2260375425939
**Snrnp48**	3564.78808018965	-0.165247303343863	0.0819216897161096	-2.01713738957911	0.0436811777113934	0.2260375425939
**Tmem116**	55.5813546351813	0.836702972603078	0.414854502357925	2.01685884532403	0.0437102459146819	0.226113704818661
**1700048O20Rik**	27.3823895463729	0.988508525185052	0.49023794447102	2.01638517853138	0.0437597141430294	0.226295312028787
**Park7**	13602.0401450435	0.134868327645568	0.0669003479104726	2.01595853919999	0.0438043114371606	0.226451619197946
**Yars2**	1954.11373365634	0.290033194311918	0.143882472403204	2.01576459917337	0.0438245969847124	0.226482182580555
**Gm2810**	109.267443248525	-0.521620183137556	0.258808347260126	-2.01546893158465	0.0438555381961338	0.226567775526715
**Prkci**	883.153223577077	0.24550623256947	0.121859727759383	2.01466257215207	0.0439400163804557	0.226929806000276
**Rnaseh2a**	4557.17585343991	0.11285349808622	0.0560283714760001	2.01422056563898	0.0439863814272019	0.22709482704732
**Mafk**	5561.17551330341	-0.166996481011146	0.0829254432008561	-2.01381475413595	0.0440289860973308	0.227240332111248
**Ndfip2**	4880.54036623511	0.154837187303754	0.0769147022675136	2.01310260248062	0.0441038365138111	0.227552112622175
**Ptp4a1.1**	2732.45265386152	0.205923452888652	0.102299070436437	2.01295526938928	0.0441193353022021	0.227557566728903
**Crebbp**	8190.83126308735	-0.299680164556516	0.148886064320532	-2.01281540971722	0.0441340521733425	0.227558985633297
**Slc39a13**	1591.22163395252	-0.211528784044438	0.105119745538664	-2.01226499322752	0.0441920105378954	0.227783287551723
**Gm6085**	117.630461093678	0.387970516874698	0.192866371939485	2.01160271214325	0.0442618332855063	0.228068576546672
**Ube2f**	3594.40621874172	0.158601630812432	0.0788716245071184	2.01088327777651	0.0443377870521972	0.228385260078938
**Slc35g1**	3002.92755573221	0.466661292446455	0.232095266090589	2.01064545738866	0.0443629189457596	0.228400157581258
**Adam6b**	163.278316744009	-1.06501456134019	0.5297047197417	-2.01058159696882	0.0443696695028388	0.228400157581258
**Cpne7**	126.632651502933	1.12728067045165	0.560854670277703	2.00993364269123	0.0444382124823143	0.228678286768069
**Adora2a**	4690.20947078717	0.267778883257026	0.133259480699148	2.00945465082198	0.0444889394165852	0.228864583214865
**Amer1**	2150.29806260208	-0.219552824446548	0.109324490122612	-2.00826753639839	0.0446148696645578	0.22943750042573
**Sdc4**	83.2608586140931	0.739596377754305	0.368304243719797	2.00811256010665	0.0446313318698335	0.229447274477314
**Terf1**	2780.22593324793	0.144648013145988	0.0720522385653206	2.007543638146	0.0446918089905399	0.229683246661428
**Prkrip1**	1020.79648830338	0.221763945005326	0.110498937810053	2.00693282125967	0.0447568165302214	0.229924858981985
**Sgsm3**	1194.81782087065	0.242487814897884	0.120831403455238	2.00682776135853	0.0447680057978505	0.229924858981985
**Naa10**	5810.94726575182	0.110150096029647	0.0549037427769333	2.00624020255181	0.0448306264868102	0.230171450489628
**Surf6**	4758.11381794486	0.185474390787754	0.0924634888923997	2.00592031524564	0.0448647504006189	0.230196636946451
**Kmt2c**	10897.5005802077	-0.361876839090638	0.18039362126647	-2.0060401057978	0.0448519692028617	0.230196636946451
**Fanca**	207.695040058131	-0.504562097451296	0.251562285338393	-2.00571439702329	0.0448867282726565	0.230234432744872
**Rbm14**	6806.68905120131	-0.16495979864111	0.0822940003896888	-2.00451792184086	0.0450146090734762	0.230594791579551
**Sqle**	2196.29572607057	0.246411262256472	0.122913352095253	2.00475585488477	0.0449891540417582	0.230594791579551
**Grhpr**	1274.20962720894	0.175620659624999	0.0876112959652839	2.00454356587294	0.0450118649882375	0.230594791579551
**Rasal2**	22.859732517888	2.02210807290601	1.00884836391954	2.004372654231	0.0450301563552883	0.230594791579551
**Trav8-2**	89.0835881668273	-0.494697677506268	0.246803410607707	-2.00441994009794	0.0450250950832417	0.230594791579551

**Stk38l**	1428.68913733685	-0.214861716154427	0.107221863701523	-2.00389835372143	0.0450809499482445	0.230730103008066
**Sema4a**	38239.3154036218	0.202743851408033	0.101190832711605	2.00357923712175	0.0451151518288921	0.230730103008066
**Lsm10**	2271.31199402963	0.213159409169528	0.10638666935054	2.00362893650872	0.0451098237721784	0.230730103008066
**Gm3435**	934.784986465634	0.388901991926087	0.194078849257318	2.00383500527904	0.0450877376888468	0.230730103008066
**Qtrt1**	5469.45743824725	0.191092026941036	0.0954007524594375	2.00304528019614	0.0451724285256334	0.230948071472552
**Ascc3**	3620.49065403544	-0.247908310529454	0.123801218648538	-2.00247068030281	0.045234133397352	0.231188530633174
**Elovl1**	9397.21506302478	0.219228868551299	0.109533102763966	2.00148505811725	0.0453401424165143	0.23123154332264
**Mrpl58**	5728.36884507805	0.171416673077194	0.0856349881850359	2.00171304638712	0.0453156024407767	0.23123154332264
**Nop16**	4141.24449800647	0.235233547798508	0.117493757463574	2.00209400802792	0.0452746218528598	0.23123154332264
**Prr5**	110.869195263264	0.652750412010474	0.326163015662835	2.00130113061391	0.0453599479856749	0.23123154332264
**Gramd1b**	2628.16835154665	-0.166160231375663	0.0829995224129905	-2.00194201779716	0.045290967911977	0.23123154332264
**Klhl15**	377.270113331783	-0.406890792522921	0.203275030672412	-2.00167620773181	0.0453195668849721	0.23123154332264
**Gm43679**	47.7854093920279	-0.722855608707008	0.361065891893032	-2.00200468927472	0.045284227194064	0.23123154332264
**Mrpl33**	4100.4433512533	0.213289181804948	0.106568927065801	2.00141999809431	0.045347147336872	0.23123154332264
**Gm44888**	44.0887142600997	-1.06559807829683	0.532540803374583	-2.00096982530615	0.0453956417994697	0.231338657126211
**Rnf146**	7080.08618533443	0.124318048382792	0.0621530965026308	2.00019074476088	0.0454796708367572	0.231691940955313
**Cacna1e**	60.6528153134495	0.775148300998442	0.387642774288994	1.99964594315012	0.0455385091780172	0.231916706243703
**Gm43162**	344.225879950758	-0.441160913378442	0.22074704732397	-1.99849066488754	0.045663490997906	0.232402980508399
**Gm5547**	1351.19932052124	-0.203203460225819	0.101678115515923	-1.99849750553251	0.0456627501045884	0.232402980508399
**H2-M2**	40.6019668228852	1.19858665434281	0.599823277227537	1.99823297935824	0.0456914076576023	0.232469974317352
**Apbb3**	4088.40744698908	0.142624860368779	0.0713974323560257	1.99761890116126	0.0457579924986452	0.232733598386428
**Rrp36**	3764.1294021262	0.184372574534547	0.0923204109902275	1.99709438635475	0.0458149306467469	0.232872858774449
**Gm7061**	29.6071176548635	-1.43709934506903	0.719560604282213	-1.99719014147889	0.0458045315996169	0.232872858774449
**Gm44249**	36.8762499181645	-0.852392300239916	0.427022573144607	-1.9961293707798	0.0459198428150207	0.233330849157137
**Gm19582**	53.2363918970941	3.66947238908244	1.83849397819088	1.99591210665444	0.0459434906848325	0.23337575200545
**Tmed1**	1115.90734065939	0.32630127076749	0.163525212139157	1.99541872778502	0.0459972300519073	0.233573430205576
**Dtx3**	1417.10502094541	-0.283033041924473	0.141876939353909	-1.99491928155042	0.0460516841959388	0.233774609495943
**Numb**	5297.25483854526	-0.145435670140818	0.0729277546521628	-1.99424308117657	0.0461254961798997	0.23407389478476
**Dtymk**	2800.30560054191	0.12600979218545	0.0632197473955279	1.99320303191157	0.0462392192552444	0.234499996826528
**Slc25a53**	1732.43793797854	0.303749361626626	0.152392589560239	1.99320296677915	0.0462392263844654	0.234499996826528
**Wdr37**	2941.57818441496	-0.160511172224728	0.0805502615365857	-1.9926834396661	0.0462961218914688	0.234712996346163
**Prpf4b**	6346.82536737078	-0.139022986179593	0.0698063523019046	-1.99155208079537	0.0464202255224216	0.23481308116146
**Il2ra**	144.723861647629	0.655986984863658	0.329422295968028	1.99132539871353	0.0464451249058671	0.23481308116146
**Clcc1**	4318.5801371788	0.149318617084583	0.0749703200129905	1.99170307741397	0.0464036458749635	0.23481308116146
**Sgcb**	1307.86191789099	0.357086916463896	0.17926375206145	1.99196386529659	0.0463750227277507	0.23481308116146
**Cox6a2**	51.7118633720372	0.647822907161438	0.325251399981146	1.99176054952874	0.0463973366634594	0.23481308116146
**Ets1**	108099.379258622	-0.221651865620581	0.111298727892299	-1.99150403439533	0.0464255021324798	0.23481308116146
**Tex2**	1830.83817582488	0.188215991245832	0.0945200468999014	1.99128118763162	0.0464499824827234	0.23481308116146
**Pop5**	4590.77199814244	0.18590247274676	0.0933226854648097	1.9920394684405	0.0463667275761786	0.23481308116146
**9230104M06Rik**	42.5913033874658	-1.54898086921992	0.77777377249633	-1.99155708767131	0.0464196756802645	0.23481308116146
**6230400D17Rik**	221.790197352146	-0.564396904336326	0.283497535811124	-1.99083530910246	0.0464989961290843	0.234985465685048
**Vipr1**	6085.98078387515	-0.345105556774638	0.173360821457151	-1.99067790446492	0.0465163093862059	0.234997591214635
**Myo6**	348.817197863684	0.429304816851775	0.21570949199598	1.99019900737505	0.0465690176253015	0.23518846497496
**Amacr**	733.311854550137	0.241802021241044	0.121538630336264	1.98950753823739	0.0466452105953436	0.2354705089423
**Gm12188**	59.1064155567188	-0.81352311151286	0.40893699985242	-1.9893604927078	0.0466614270600003	0.2354705089423
**Gm13316**	345.938005013867	0.288336731700447	0.144944869614226	1.98928552951106	0.0466696959717461	0.2354705089423
**Rnmt**	4600.24855888766	-0.123685234342386	0.0621901704517315	-1.98882288702495	0.0467207556412754	0.235652671779634
**Dad1**	12476.8244923003	0.159065892323596	0.0799867526249738	1.98865295943934	0.0467395215482844	0.235671885131622
**Zfp273**	635.331413021149	-0.292204841291645	0.14699628119324	-1.9878383243418	0.0468295736708812	0.236034508042344
**Rec114**	47.2176844222611	-0.793633459174278	0.399265935189008	-1.98773145722682	0.0468413978960723	0.236034508042344
**Hsdl2**	4968.52710336142	0.122230237834342	0.0615046334760471	1.98733381415799	0.0468854168568897	0.236109329654937
**H1f2**	1354.63349872914	0.458309959364485	0.230616325517407	1.98732660550474	0.0468862151735926	0.236109329654937
**Cbfa2t3**	104.315261983405	0.420486672743974	0.211639741986832	1.98680393765617	0.0469441281025486	0.23624996055952
**Foxk1**	1051.44494069333	0.627442122846727	0.315791939911088	1.98688453867247	0.0469351933818096	0.23624996055952
**Themis**	18332.2534086304	-0.237518242679394	0.11957621493832	-1.98633350956886	0.0469963041814847	0.236437025858127
**Tm9sf3**	16521.9712788993	0.329890661624262	0.166101734987452	1.98607595308492	0.0470248909247816	0.236505332365714

**Aldh3b1**	167.368413532408	0.520414573789527	0.262103806203494	1.9855284870815	0.047085703939323	0.236612874676731
**Ndrg4**	54.3142181597325	0.881434739571394	0.443967329428324	1.98535946486508	0.0471044924382682	0.236612874676731
**Znfx1**	5043.87835893953	-0.332244397893694	0.167324753646906	-1.98562609926088	0.0470748562474226	0.236612874676731
**Gm4482**	200.925767898727	0.435085169288405	0.219148631414656	1.98534285375102	0.0471063392689537	0.236612874676731
**Ntrk3**	2744.52774794674	-0.28952466820351	0.145856280766923	-1.98499966323814	0.0471445089657463	0.236653720220855
**Gm37642**	92.7019999368293	-0.622715441568822	0.313703950459646	-1.9850417588188	0.0471398256900574	0.236653720220855
**Piga**	3392.93117127822	-0.127008701353778	0.0640035321079402	-1.98440144115143	0.0472111055053113	0.236837118575992
**Rpsa**	56844.392501929	-0.164930771951993	0.0831131589176263	-1.98441226515594	0.0472098998301911	0.236837118575992
**Ipo8**	2596.31161271083	-0.225057175744854	0.113423123257181	-1.98422657816034	0.0472305869092402	0.236859439188064
**Aip**	13942.8491579366	0.120727125544263	0.060857322554989	1.98377320059024	0.0472811289842739	0.236968099977395
**Gm6430**	10230.8935574026	0.116059577012505	0.0585047766901789	1.98376241357379	0.0472823320637767	0.236968099977395
**Plekho1**	529.033216384508	0.448538393706197	0.226145086673774	1.98340985560848	0.0473216671435253	0.237089828038324
**Kntc1**	123.555980797241	0.680633530294782	0.343190610444407	1.98325219158359	0.0473392667056075	0.23710261458368
**Mydgf**	5194.42374302803	0.184439274236812	0.0930129095235139	1.98294274613768	0.0473738251760494	0.237124956575289
**Zdhhc13**	3964.69767921441	0.177558356424623	0.0895394288849562	1.98301863922716	0.047365347570319	0.237124956575289
**4632415L05Rik**	675.865868692467	-0.276007780039271	0.139226394890088	-1.98243860481459	0.0474301724663742	0.23733160608214
**Tspan6**	161.535235964115	-0.566519747040521	0.285818555820537	-1.98209575796833	0.0474685242450789	0.237448106834828
**Fosb**	199.288097055512	0.991703931788848	0.500425718273487	1.9817205542719	0.0475105254272102	0.237582782975738
**Cd69**	15910.581661851	-0.276651299548242	0.139693723044573	-1.98041324634157	0.0476571127206435	0.23823666067012
**Map4k5**	612.882386362016	-0.25532472440667	0.128933328154513	-1.98028491206472	0.047671523201935	0.23823666067012
**Swap70**	664.223889825857	0.425595294397871	0.214948419281454	1.97998801675575	0.0477048752116153	0.238327748481111
**Eapp**	2969.34592294396	0.264309821326512	0.133506277645659	1.97975575371835	0.0477309803671345	0.238382585624386
**Srcap**	1473.24534343795	-0.41793783870211	0.211145659959905	-1.97938162111158	0.0477730561719669	0.23851712487379
**0610038B21Rik**	41.3197827763445	0.817341323146249	0.413009739910816	1.97898800963566	0.0478173562624923	0.23866268059173
**Pskh1**	6010.21945184288	0.207705365280604	0.104965542494002	1.97879571091126	0.0478390116046237	0.238695157015217
**Hccs**	2724.26883173409	0.174260253395511	0.0880798395846395	1.97843518127729	0.0478796341524306	0.238822220747024
**Gm32856**	701.288210685768	-0.307576368289148	0.155478354214696	-1.97825845174836	0.0478995576748862	0.238845990595943
**Psmb1**	12143.8020377267	0.125755056308952	0.0636031400296984	1.97718314300572	0.0480209323334113	0.239224100783295
**Ogfod2**	2493.82031355498	0.165918717449054	0.0839055485295874	1.97744631143847	0.0479912035371728	0.239224100783295
**Gm45495**	23.0216185270254	-1.62002022797427	0.819326486187896	-1.97725846202261	0.0480124223478286	0.239224100783295
**Sphk2**	5530.63341446114	-0.157441026919975	0.0796492587137637	-1.97667912372885	0.0480779120206563	0.239432256545348
**Tle3**	2566.95233308411	-0.222003126137196	0.112366793667099	-1.97570046178329	0.0481887125987591	0.239908228884249
**Tmem104**	4480.99663900004	0.203129191424615	0.102833446097713	1.97532222377923	0.0482315927811289	0.240045864640634
**Uap1l1**	1836.79342719691	0.216294069309041	0.109507438621634	1.97515412680203	0.0482506599277969	0.240064934790747
**4930524O07Rik**	75.2334313844017	-0.75135827196897	0.380517648192197	-1.97456878948611	0.0483171038348527	0.240319635456368
**Lrrc57**	1328.86567586778	0.263540681007599	0.133498179246572	1.97411442234608	0.0483687337798511	0.240424649264705
**Ms4a4a**	156.803990258947	0.624415701549595	0.316282198539248	1.9742359969466	0.0483549146639983	0.240424649264705
**Atad5**	589.042945292817	-0.317613255782621	0.160950096267239	-1.9733648077803	0.0484540141109804	0.240744862699017
**Exosc5**	8643.31815509476	0.0943960933015148	0.0478371597992248	1.97327963653571	0.0484637116507762	0.240744862699017
**Carns1**	1453.90360739268	-0.226662067441739	0.114886670157206	-1.9729187653501	0.0485048182840813	0.240873123763715
**Hilpda**	1370.22149719597	0.282202812191334	0.143072289496415	1.97244912473708	0.0485583586273472	0.24106302989638
**Supv3l1**	2140.80443439808	0.15206715660489	0.0771391119893614	1.9713366239666	0.0486853848761957	0.241617514801328
**Birc6**	19092.840688446	-0.325712366425617	0.16525888369456	-1.97092198097874	0.0487328004712548	0.241776680423666
**Pcgf3**	710.489453132644	-0.313345012051376	0.159025554029773	-1.97040666805482	0.0487917819853682	0.241859704117592
**Stub1**	17492.0213153566	0.129190402909317	0.0655624159612402	1.97049484853781	0.0487816848050331	0.241859704117592
**Gm9316**	48.8697593878414	0.870947765895654	0.442021665207156	1.97037347815854	0.0487955828768056	0.241859704117592
**Gatc**	2092.1149890121	0.205475304225241	0.104305903216169	1.96992977280876	0.0488464196855001	0.242035545592587
**Fosl2**	247.253141311046	0.469545688911979	0.238396703327686	1.96959807899093	0.0488844519934091	0.242071750490304
**4930526A20Rik**	52.9963457492927	-0.73106334845162	0.371170958770869	-1.9696135464707	0.0488826779260394	0.242071750490304
**Gm39556**	153.633142037079	-0.621012235862558	0.315451646877224	-1.968644773328	0.0489938972791362	0.242537492751288
**Rbm12b1**	932.327260779692	-0.251197253636068	0.127667064947532	-1.96759637060118	0.0491144976739993	0.243058146937565
**Zfp212**	1337.74994380532	-0.256799144287247	0.130529058296995	-1.9673714622452	0.0491404018757471	0.243109988180894
**Nup133**	2596.82283845553	0.134287790674601	0.0682696092306913	1.96702152228272	0.0491807296027126	0.243233131308833
**1810013L24Rik**	1643.51574744962	-0.259258003235753	0.131814554150427	-1.96683898001041	0.0492017771307465	0.243260872999427
**E2f3**	124.862826694485	-0.485775164533272	0.247077519735855	-1.96608402517803	0.049288905384802	0.243530884428252
**D2hgdh**	3500.02262636379	0.231473500362911	0.117728576308313	1.9661624018685	0.0492798540252745	0.243530884428252

**Igkv8-30**	121.850825560811	-3.05024291920043	1.55152522486198	-1.96596411732318	0.0493027556848464	0.243530884428252
**Bsg**	13175.8249119096	0.182452642396508	0.0928306936741257	1.96543443957225	0.0493639767087709	0.24375687276719
**Zfp607b**	199.605185246328	-0.428298289602054	0.218002348506414	-1.96464988811555	0.0494547736437798	0.24405226066553
**Exosc6**	237.751399733677	3.10668643358461	1.58121428644624	1.96474725798668	0.0494434973205456	0.24405226066553
**Pip4k2b**	188.869998620579	-0.353019892459024	0.179810795453441	-1.96328530536105	0.0496130319416813	0.244606622387081
**Mrps24**	3619.68692360355	0.218048982747425	0.111055295189341	1.96342715919729	0.0495965605999254	0.244606622387081
**Rab14**	12508.6302762155	-0.12967369444092	0.0660551865057529	-1.96311147239932	0.0496332227918934	0.244606622387081
**Ttyh1**	37.1159325021529	-1.10926226586768	0.565081626719447	-1.96301244531246	0.0496447279554411	0.244606622387081
**Arl1**	4569.96918709492	0.189833587284432	0.0966991244194233	1.96313656844551	0.0496303074386343	0.244606622387081
**Hook3**	2360.05266903065	-0.204413038073809	0.104169754771541	-1.96230699133463	0.0497267537411163	0.244934185276264
**Mrpl45**	2716.52133244508	0.219706201856651	0.111983939144194	1.96194386030437	0.0497690206423794	0.245065768206867
**Calcrl**	1421.9773420892	-0.398689759083087	0.203254445971432	-1.96153032312574	0.049817191290799	0.245226330262143
**Commd2**	2220.24542562497	0.18234517932871	0.0929734730973373	1.96126027407631	0.0498486689009542	0.245304645806476
**Cdk5rap3**	11290.9874389257	0.18435290956952	0.0940315739846011	1.96054263219831	0.0499324001389274	0.245639971585725
**Gm6545**	157.828129612537	-0.700710806345608	0.357483752665151	-1.96011930925978	0.0499818468352318	0.24580647958263
**Plekho2**	5437.99397167268	0.185875035647108	0.0948456192134624	1.95976405856734	0.0500233739539225	0.245917287212922
**BC046401**	25.6522827497357	-0.959410873535652	0.489580374633603	-1.9596595845037	0.0500355919784622	0.245917287212922
**Cep85**	4033.00581752556	0.150496116518604	0.0768037155855198	1.95949005033524	0.0500554239708035	0.245938046619255
**Unc119b**	10559.8386095964	0.135992523862281	0.0694309392327216	1.95867325669405	0.050151064315384	0.246331147262315
**Capn2**	8085.9393642667	0.237886649392683	0.121515633808447	1.95766291082907	0.0502695800386507	0.246836326789971
**Mknk1**	3875.04130460857	0.164015711893886	0.0837932326092841	1.95738613712002	0.0503020871138112	0.24691899895711
**2310015A10Rik**	429.702793514423	-0.312701714826136	0.159786060645889	-1.95700246668658	0.0503471783466972	0.247063372534696
**Orm3**	41.8339877495036	-0.825688413418807	0.421963452205327	-1.95677708366323	0.0503736824917587	0.247116474166451
**Gm6204**	48.9714764499782	0.702747730734435	0.359169982362342	1.95658814835334	0.0503959095392426	0.247148567261079
**Gm43422**	52.6245696243208	-0.794629082496037	0.406354567789515	-1.95550670641813	0.0505232925742168	0.247545619520679
**Gm45731**	312.82946831656	-0.438715357667993	0.224349375820273	-1.95550068309283	0.050524002816431	0.247545619520679
**I730030J21Rik**	2642.76035727685	-0.335573505000415	0.171597998442427	-1.95557936599712	0.0505147255572177	0.247545619520679
**Gm47230**	60.6652234744128	1.33944968350248	0.685142674373948	1.95499380435821	0.0505838015557012	0.24776156702306
**Egfl8**	81.3323119171986	-0.636515454394271	0.325617404952796	-1.9547955505835	0.0506072065622015	0.247799177688194
**Mtrex**	7704.13354297786	0.14764563435556	0.0755513514777707	1.95424213422577	0.0506725885860639	0.247811290611611
**L1cam**	781.874961946601	0.321747364949588	0.164634025632099	1.9543187607438	0.0506635315146932	0.247811290611611
**Wdr19**	584.266424871218	-0.47433509046994	0.242689687914015	-1.95449215229119	0.0506430420543671	0.247811290611611
**Krba1**	3227.87196687882	-0.24755659612202	0.126675088991446	-1.9542642368993	0.0506699759636683	0.247811290611611
**Mthfsl**	1516.00532867233	0.229792904051131	0.117609572870035	1.95386224474316	0.050717510649287	0.247954022743039
**Scarf1**	82.0799117693947	-0.888180509334237	0.454651666599101	-1.95354064349534	0.0507555661793491	0.248063106789083
**Zfp74**	920.481070462776	-0.181104728825028	0.0927190546184445	-1.95326332403093	0.0507884009864246	0.248146615300184
**Gm26707**	20.8182298055305	-1.64781313212356	0.843841563507135	-1.95275180008318	0.05084901243601	0.248365743631187
**Capza2**	22425.3031743568	0.131072116057975	0.0671410091723619	1.95219162883733	0.0509154576872784	0.248536204082542
**Gm15983**	44.8206202793297	-0.852759979643627	0.436796026534051	-1.95230709036029	0.0509017561613571	0.248536204082542
**Setd3**	4395.47113613412	-0.135373080354925	0.0693553704980932	-1.95187595975206	0.0509529331037923	0.248642108057125
**Hspa5**	29607.0136918674	0.274428031617106	0.140625901820419	1.95147571012597	0.0510004829141854	0.248720089532287
**Nebl**	198.675221160286	1.20037631807097	0.615072474844714	1.95160142448909	0.050985543997986	0.248720089532287
**Gm12961**	28.1254266009971	-1.01109988486571	0.518229515240911	-1.95106580217778	0.0510492186474179	0.248803754477997
**Gm37298**	23.088632609098	-1.48015360536436	0.758597156593206	-1.95117209773314	0.0510365769642425	0.248803754477997
**Oit3**	30.6104249678297	0.702573868436872	0.360199727625735	1.95051193699647	0.0511151320223209	0.249047970091438
**Mlh3**	1313.34585795302	-0.291277862613305	0.149352800210818	-1.95026716741939	0.0511442838094209	0.249112976811451
**Sdha**	44502.3320852875	0.0965803382312239	0.0495289672273057	1.9499768244305	0.0511788813881997	0.249204460455458
**Ogfrl1**	101.483019512772	0.628687972237349	0.322460916678361	1.94965634506471	0.0512170927994655	0.249313478912937
**Gm2011**	226.178031728555	-0.41231275199907	0.211526924598266	-1.94922113476636	0.051269022055315	0.249489184844224
**Tapbpl**	20730.1018543067	0.116971880463913	0.0600273385606561	1.94864345594326	0.0513380187213551	0.249747811359182
**Fry**	1014.3048566668	-0.358769476842919	0.184146434188717	-1.94828359519166	0.0513810389639406	0.249879947825559
**Zc3h12d**	3338.19580416498	-0.190813182306279	0.0979662181565249	-1.94774470115207	0.0514455185117303	0.250039184203989
**Gm44164**	294.934598181125	-0.538599302513639	0.276513932628086	-1.94781976226153	0.0514365332701087	0.250039184203989
**Arid2**	3384.98293936453	-0.233562069333893	0.119935632973328	-1.94739514474263	0.0514873797085764	0.250165260963101
**Fam135b**	33.6520848600555	-1.75552937594918	0.901536860944147	-1.94726300387838	0.0515032116877768	0.250165260963101
**Hnrnpc**	20301.0531278375	0.153810943249567	0.0790180656476784	1.94652883475244	0.051591247727116	0.250419416952855

**Hmgn2-ps1**	81.7564118274693	-0.551927657284348	0.283559083174339	-1.94642912195131	0.0516032142442038	0.250419416952855
**Hmgb1-ps6**	24.7660322553945	-0.784092289459509	0.402800771228514	-1.94660076510798	0.051582616818766	0.250419416952855
**Gm44226**	302.505554784745	-0.418883274708353	0.215223114155603	-1.94627457348985	0.051621766170111	0.250432318208879
**Rnf26**	3365.82796170762	0.287183825805904	0.147581088322777	1.94593920582697	0.0516620427468885	0.250550571733679
**Ppp1r7**	4154.22781130058	0.134024702174004	0.0688856413349501	1.94561159011819	0.0517014137274927	0.250586967555858
**Epx**	41.655498778506	1.78132435164189	0.915568346090706	1.94559407743593	0.0517035190081212	0.250586967555858
**C130013H08Rik**	68.1448255618381	-0.580857163553709	0.298567561379241	-1.94547981324704	0.0517172569963603	0.250586967555858
**Map3k9**	112.522576234099	1.01537740137885	0.521987488942971	1.94521405759168	0.0517492206234762	0.250587728753569
**Kdm3a**	10081.8568542214	-0.169064500946389	0.0869112851550684	-1.94525372216901	0.0517444489373169	0.250587728753569
**Slc36a1**	629.919357768688	0.246919060476304	0.126997842593704	1.94427759900029	0.0518619844820759	0.250805587308968
**B3gat2**	549.858955374943	-0.384698525588935	0.197865671752876	-1.94424086897399	0.0518664115222952	0.250805587308968
**Ccdc137**	3441.71581547419	0.139941097137117	0.0719795109421521	1.9441796047987	0.0518737963470158	0.250805587308968
**Psme3**	16258.1307752687	0.0774106441612159	0.03981481623249	1.94426727249457	0.0518632290954704	0.250805587308968
**Gm47857**	30.1379013368535	-0.991205580745558	0.509829231819571	-1.94419134659651	0.0518723809147058	0.250805587308968
**Fam118a**	2962.85086587322	-0.21036694534808	0.108211405560818	-1.94403671459426	0.051891023846523	0.250811921088854
**Klra17**	75.6187073582154	1.07180222679404	0.551407173215221	1.94375822233946	0.0519246139075675	0.250897314118841
**Ftsj3**	11342.9114937311	0.115965840696543	0.0596732019151989	1.94334872228478	0.0519740383779644	0.250905307206368
**Frg1**	4822.49255329613	0.160450761612677	0.082563705150905	1.94335708795305	0.0519730282928388	0.250905307206368
**Crebl2**	907.72865613933	-0.336032661273802	0.17290249785344	-1.94348066364338	0.0519581094649608	0.250905307206368
**Cdc42**	53734.6129801075	0.0943354083649862	0.0485468967028147	1.94318102231067	0.0519942902246363	0.250926196345971
**Eno1**	95569.3059357026	0.114076728205617	0.0587115147223983	1.94300434497387	0.0520156333389993	0.250952337575815
**Csnk1e**	774.369960231434	0.326562437777141	0.168104833535928	1.94261182684761	0.0520630768496352	0.250975324693498
**Mrps12**	2448.93814324186	0.235773133182127	0.121371780842593	1.94256961169501	0.0520681815334801	0.250975324693498
**Usp31**	457.960534457917	-0.286072794359359	0.147250228876252	-1.94276638170643	0.0520443915466403	0.250975324693498
**Mmut**	2828.1282288708	-0.16601202738609	0.0855035204862463	-1.9415811938737	0.0521878213654802	0.251039454569349
**Selp**	94.7002015831881	1.19433228102251	0.615147399382924	1.94153837311283	0.0521930096572435	0.251039454569349
**Pole3**	2487.91307402806	0.157752565183078	0.0812302065336285	1.94204313782915	0.0521318782710358	0.251039454569349
**Vps13b**	10054.128722967	-0.343910520788424	0.177071701599136	-1.94221051518998	0.052111620646268	0.251039454569349
**Alg1**	4731.2307679121	0.191242345131949	0.0984919237915413	1.94170585536246	0.0521727194613875	0.251039454569349
**Prss2**	76.9903976245406	0.662090800064464	0.340908995736111	1.94213355571577	0.0521209342073117	0.251039454569349
**Rapgef2**	1554.30130463908	0.514731946813558	0.265100781168495	1.94164628464976	0.0521799355980617	0.251039454569349
**Ergic2**	5543.8223601712	0.199651954008646	0.102866891336564	1.94087671372722	0.0522732331368191	0.251348591558394
**Rnf4**	16145.3782787755	0.112922164032205	0.0582052744172019	1.9400675482226	0.0523714812604487	0.251744182495696
**Spopl**	2486.16533594961	-0.168898542097103	0.0870690906537143	-1.93982205199357	0.0524013196954407	0.251810794279066
**Gm6568**	54.1751102687402	-0.665777690002661	0.343390226986357	-1.93883703635256	0.0525211847708685	0.252309850132492
**Dnajb4**	1561.37973388659	-0.306345240296349	0.15804709333428	-1.93831619318938	0.0525846579854272	0.252383934168863
**Tnks**	1405.45569256309	0.346502965242213	0.178760647647055	1.93836266428363	0.0525789921221695	0.252383934168863
**Cd24a**	195.20384539124	0.597985555297087	0.308467364640148	1.9385699229307	0.0525537288819706	0.252383934168863
**Cabcoco1**	68.6382086890973	0.8036874690296	0.414832974363852	1.93737604938966	0.0526993921449369	0.252649886242303
**F2rl1**	3050.24693094584	-0.153949703703351	0.079463294360955	-1.93736875549167	0.052700283101896	0.252649886242303
**Mrpl16**	1609.7163785854	0.285226640955026	0.147197188094696	1.9377180002347	0.0526576366347591	0.252649886242303
**Trp53rka**	4936.50597701688	0.229106793638454	0.11825863861462	1.93733664045521	0.0527042061355873	0.252649886242303
**Sptlc2**	8051.6999018361	0.146541835439677	0.0756562960341654	1.9369417103568	0.0527524690455495	0.252727471800159
**Zfp444**	2975.48113118013	-0.152681771076226	0.0788238386560641	-1.93699994417209	0.0527453501908214	0.252727471800159
**Gm8325**	520.590563929513	0.253262915844161	0.130763767928452	1.93679732433785	0.052770123132048	0.252735206745192
**Unc119**	843.968723674319	0.319472714484403	0.164983593175979	1.93639081519844	0.0528198535502755	0.252852103684391
**Rab3d**	2653.98335340244	0.207093577112678	0.106951289220837	1.93633549087065	0.0528266246956283	0.252852103684391
**Myo1c**	2396.80598618375	0.173913189010247	0.0898265385175932	1.93610030933325	0.0528554166645354	0.252913088485601
**Gpsm1**	1531.18995156656	0.239243653940367	0.12361156875327	1.93544711351329	0.0529354525999909	0.253142314603356
**Tmem191c**	1673.44591959948	-0.155131964180203	0.0801486224878091	-1.93555371714341	0.0529223835703119	0.253142314603356
**Gin1**	1127.16942786935	-0.213859404522326	0.110507772521928	-1.93524310228849	0.0529604707941027	0.253185114776298
**Atxn2l**	7107.06791573381	-0.247856058429674	0.128111354625861	-1.93469235536162	0.0530280590360355	0.253286014071377
**Faah**	15926.9493767072	-0.202864233391306	0.104856836618527	-1.93467817581922	0.0530298001155976	0.253286014071377
**Gm36839**	54.5939622601851	-0.827435542433026	0.427668920362208	-1.93475724570362	0.0530200918813975	0.253286014071377
**Grhl1**	80.2234741389467	0.772188209121591	0.399309018333544	1.93381109283282	0.0531363584173268	0.253688048721385
**Taf1d**	2738.38406632771	-0.141446165641631	0.0731467528376396	-1.93373130254452	0.0531461730551052	0.253688048721385

**Gm14539**	20.3629792329357	-0.874831925423167	0.452590026759117	-1.93294565434333	0.0532428929377255	0.254072763179813
**Tdgf1**	211.367432485675	-0.383658191119976	0.198497510760827	-1.93281109495752	0.0532594730658212	0.254074937056659
**Abt1**	1975.6094525454	0.209016446118136	0.108167100535756	1.93234768319451	0.053316606707824	0.254116688413546
**Zfp292**	14535.8796770096	-0.253688963231446	0.131270537019633	-1.93256589781075	0.0532896968391148	0.254116688413546
**Naa15**	6817.49803193518	-0.113611808900296	0.0587911185865793	-1.93246550893541	0.0533020752212422	0.254116688413546
**Celsr3**	18.5511401822182	-1.26902745608929	0.65689349889813	-1.93186179832492	0.0533765660085971	0.254325536920915
**A430103D13Rik**	91.9708479069217	-0.555128933057749	0.287513354856866	-1.93079355682143	0.0535085874836175	0.254877512992552
**Pdk1**	25770.4050981054	-0.249995386557766	0.129522598459285	-1.93012948729832	0.0535907956546041	0.255039780922059
**Mrpl9**	4635.03565114593	0.124985183115821	0.0647646590358743	1.92983619425202	0.0536271372891606	0.255039780922059
**Sox5**	30.4784870287815	1.51837340971707	0.786687683561106	1.93008412543569	0.0535964150508432	0.255039780922059
**Camkmt**	388.586204430868	0.36947495406066	0.191405294470716	1.93032776382885	0.0535662390694281	0.255039780922059
**Snhg14**	132.703168372606	-0.874244150200699	0.45302811850565	-1.92977900154292	0.0536342263749761	0.255039780922059
**Gm42549**	79.4826020144402	-0.986223775759931	0.511067134707443	-1.92973429278423	0.0536397686092342	0.255039780922059
**Clic1**	31333.3227407421	0.134497636582243	0.0697127084642534	1.92931302692405	0.0536920134958247	0.255134214913664
**Inppl1**	187.404748650001	0.519647084192729	0.26933231895697	1.92939000490227	0.0536824636081187	0.255134214913664
**Slc22a17**	597.768681751107	-0.280620749851036	0.145490367951296	-1.92879263282209	0.0537566108001201	0.255318505711375
**Klhdc3**	6570.95140003505	-0.130346938468376	0.067581410104664	-1.92873954933031	0.0537632037792945	0.255318505711375
**Lca5**	643.477535043279	-0.32514932025937	0.168611233775066	-1.9283965426238	0.0538058215494533	0.255443907850176
**Ppp1r18**	14396.3205897153	-0.16033455833124	0.0831499677629596	-1.92825761265855	0.0538230912913933	0.255448930565809
**1700012D14Rik**	297.379048266792	-0.404617601749948	0.209850309866853	-1.92812487151757	0.0538395960516482	0.255450320682271
**Cpsf3**	14445.2412941281	0.128347312115181	0.0665738672875502	1.92789329123416	0.0538684003825543	0.25551004961707
**Cd82**	32009.804012036	0.206981884284843	0.107380063375678	1.92756343941332	0.0539094500962063	0.255627807753813
**Ccdc191**	714.21738647474	-0.222346263309015	0.115393683683668	-1.92684951386543	0.0539983868055188	0.25584799217432
**Nme4**	1546.78660819541	-0.20627994057084	0.107044308481375	-1.92705192361283	0.053973159342306	0.25584799217432
**Pcna-ps2**	80.9514636208351	0.5416265879532	0.281101655939311	1.92679970576244	0.0540045961776854	0.25584799217432
**Gm44008**	28.0771820810591	-0.776137676359834	0.402840326922742	-1.92666330674656	0.0540216035349603	0.255851640186465
**Traip**	409.032128887169	-0.275805191902102	0.143214675435152	-1.92581654822789	0.0541272844323006	0.256216732969995
**Gm12751**	312.205494093787	0.429132208067028	0.222834939600484	1.92578510729246	0.0541312117825166	0.256216732969995
**Taf7**	1279.33742896586	0.21609364871092	0.112286140067807	1.9244908461581	0.0542930870330602	0.256828631756208
**Klhl24**	5311.44420581036	-0.14445158292632	0.0750548365620534	-1.92461391621166	0.0542776771131889	0.256828631756208
**A730061H03Rik**	100.844947696622	-0.950430795970997	0.494047367155983	-1.92376451966988	0.0543841066140157	0.257181983168381
**Zbtb20**	9179.59940516164	-0.278486659114884	0.14477839541502	-1.92353740567837	0.0544125935101485	0.257239494731296
**Zkscan3**	6258.12440044533	-0.207082782710801	0.10771337327295	-1.92253548856969	0.05453841255517	0.257756978593826
**Siva1**	1356.81185951433	0.173200955043459	0.0900985117838654	1.92235089808082	0.0545616195685358	0.257789337193829
**Zfp773**	1588.80776243439	-0.222613534495688	0.11582414290308	-1.92199595797542	0.0546062663569106	0.257922943341079
**Uchl5**	4651.07420799081	0.143034131247844	0.0744508811051362	1.92118789092446	0.0547080242095841	0.258326142484392
**Spag5**	167.572477026696	0.762796900372579	0.397196798286789	1.92045077821049	0.0548009848392788	0.258551207846261
**Cerkl**	265.494502980826	-0.354827603399979	0.184765660253652	-1.920419643525	0.0548049142734347	0.258551207846261
**Gm26771**	565.066225096416	0.435598988522583	0.226808310283843	1.92056008872623	0.0547871908810178	0.258551207846261
**Aste1**	1637.71643773507	0.196391342572132	0.102278403534385	1.92016433367682	0.0548371451822204	0.258557538022102
**Vps25**	7071.99919801826	0.143575397916896	0.0747730481455134	1.92014905741824	0.0548390741936832	0.258557538022102
**Snrpb2**	1966.8976101527	0.170298482836201	0.0887016398472154	1.9199023054087	0.05487024067398	0.258627096111248
**Fancc**	1577.12685777698	0.238446538527852	0.124220794554955	1.9195380240654	0.054916278915891	0.258766688659598
**Paqr4**	685.639115298517	0.253277040901659	0.131984351910734	1.91899295056553	0.054985226000654	0.258936702358728
**Eif4a-ps4**	6844.49118711542	0.188520695574753	0.0982360857717522	1.91905748375168	0.0549770593459733	0.258936702358728
**Smg1**	14150.9110306922	-0.312062086848782	0.16264380045275	-1.91868417966192	0.0550243148611332	0.259043360999963
**Gm20692**	68.6670346084411	-0.842761662359515	0.439432349744044	-1.91784164923315	0.0551310928919021	0.259468527687486
**Bhlhe40**	870.213623576136	1.35905680933068	0.70872782608799	1.91760046565739	0.0551616910555431	0.259535015217137
**Zfp68**	5130.18210428441	0.140520185111438	0.0732928511204802	1.9172427182625	0.0552071033566321	0.259594016555929
**Jazf1**	101.729788089547	-0.428105161582195	0.2232921757187	-1.91724210758516	0.0552071809023875	0.259594016555929
**Scand1**	1630.18405364254	-0.162802745021524	0.0849335788355945	-1.91682426730964	0.0552602608800922	0.25967993179902
**Lifr**	179.174394774372	0.736429420178129	0.384215530608896	1.9167091424208	0.0552748931429785	0.25967993179902
**Gm48965**	46.3622870592163	-1.76184007522022	0.919121642312612	-1.91687366950389	0.0552539828986337	0.25967993179902
**Zfp729b**	1811.7754222345	-0.274511038588327	0.143231169248823	-1.91655936363574	0.0552939347163154	0.259691961050665
**Ndufv1**	8955.25220560873	0.164223528464093	0.085714469647027	1.91593705403962	0.0553731083078822	0.259754113607413
**Rasgrp3**	117.847854812288	0.473853953508045	0.247305375266088	1.91606815257534	0.0553564213928233	0.259754113607413

**Ftx**	897.453114435719	0.240796832804429	0.12567833861152	1.91597721186265	0.0553679963624304	0.259754113607413
**Gm14230**	87.5664313047947	-0.596455217109166	0.311263076876762	-1.91624147359219	0.0553343666101295	0.259754113607413
**Trappc8**	3468.77581497004	0.709233685761619	0.370282829287446	1.91538367341103	0.0554435917225511	0.260007343682214
**Cars**	4828.06218238435	0.434874103959365	0.227112587678279	1.91479524937382	0.0555186205723072	0.260126941527756
**Slc52a3**	900.096456301327	0.295198113568807	0.15415984092636	1.91488335609933	0.0555073808657166	0.260126941527756
**Pirb**	547.710239177528	0.530821841324148	0.277196426176954	1.91496639637514	0.0554967892179067	0.260126941527756
**Pim1**	1350.82590114048	0.37076495921214	0.193675839539849	1.91435834274959	0.0555743844335886	0.260242437223673
**Tmem167b**	2675.23146735348	0.139692966526305	0.0729717418352222	1.91434331993536	0.0555763026754877	0.260242437223673
**Sh3glb2**	4924.54548054927	-0.113079774905077	0.0590742784004161	-1.91419646531443	0.0555950572399909	0.260252916497486
**B330016D10Rik**	70.9277730488002	1.01704866684782	0.531498660070076	1.91354888216223	0.055677821936016	0.260485582020726
**B130021K23Rik**	162.944963586786	-0.396625653407106	0.207261843994036	-1.91364529893172	0.0556654928414666	0.260485582020726
**Lmbrd2**	290.089815260857	-0.344083196194174	0.179856977621842	-1.91309339645207	0.0557360969907259	0.260603465959308
**Ighv5-17**	78.8625691005399	2.94910854487429	1.54145686296628	1.91319563701524	0.0557230118645788	0.260603465959308
**Crocc**	162.634527621803	0.746618382631622	0.390306713552131	1.91290171731033	0.0557606356956174	0.260640859286812
**Dhx36**	3651.7116433912	0.362925941865834	0.189740881711315	1.91274510054199	0.0557806924065689	0.260657286551099
**Zfp579**	590.77686861592	-0.345530418999917	0.180672188776483	-1.91247153942097	0.0558157397016346	0.260666452424024
**Gm16008**	132.869876655868	-0.606433023975693	0.317090040199757	-1.91249470842306	0.0558127706931118	0.260666452424024
**Kdm5b**	8871.78433303886	-0.205469022025502	0.107447734057488	-1.91226947527409	0.0558416389352723	0.26071013472684
**Zcchc10**	1026.32373656239	0.317341178957281	0.165987593027747	1.91183674134146	0.0558971375570948	0.260737476757591
**Dmac2**	5065.14929085196	0.138594510169412	0.0724876883160943	1.91197310038427	0.055879644398556	0.260737476757591
**Dph1**	3824.02330648764	0.18997449770757	0.0993658975375412	1.91186818028586	0.0558931039294808	0.260737476757591
**Grb7**	111.815626387903	-0.567900379403047	0.297123789448419	-1.91132584993379	0.0559627190941813	0.260821609668395
**Xdh**	4881.16865872536	-0.301853654058622	0.157930293583555	-1.91130939612242	0.0559648322833561	0.260821609668395
**Zfp623**	1795.20989589585	0.203747394799991	0.10658706546145	1.91155834826584	0.0559328660667777	0.260821609668395
**Ubn1**	5771.47328641985	0.324770410375761	0.169987355436173	1.91055628545093	0.0560616266445176	0.261195461572343
**Snrpd1**	1599.66741358749	0.233525279977785	0.122277413843225	1.90979897789787	0.0561591009556884	0.261245452416044
**Rab23**	581.19757646185	-0.282106273529094	0.14768906228998	-1.91013653384292	0.0561156361517648	0.261245452416044
**Mrpl49**	3779.54040301303	0.217438497868372	0.113832901093515	1.91015511139213	0.0561132448594544	0.261245452416044
**Rad50**	5296.77325830349	-0.161630858390362	0.0846354112345546	-1.90973088016818	0.0561678728385816	0.261245452416044
**Trappc5**	2969.37188242077	0.113696862383358	0.0595332823851128	1.90980335416194	0.0561585372743504	0.261245452416044
**Rrp1b**	4368.26788950114	0.18849177983418	0.0987022920021331	1.90970012965967	0.0561718342822591	0.261245452416044
**Otud7b**	1341.26899948784	-0.291873667312357	0.152873373210124	-1.90925117424588	0.0562296975988316	0.261437398956857
**Ebna1bp2**	6455.22772309886	0.175686225554899	0.0920389031450952	1.90882571990169	0.0562845777871098	0.261615366428168
**Dnajc15**	12490.6810770105	-0.179636748788327	0.094125723575247	-1.90847668379112	0.0563296339304168	0.261747579428362
**Flt3**	674.046738729634	0.478648671139088	0.250913830114498	1.90762171587221	0.0564401263369871	0.262183688293604
**Psmd5**	9453.06509684356	0.170243413323319	0.0892715365376988	1.90702904784691	0.056516826056362	0.262462607771911
**Lpcat1**	1922.98471281454	0.21186882597968	0.111118804356504	1.90668741628949	0.0565610774710118	0.262545984394695
**Spint1**	129.398963056773	0.733799855748823	0.384866821750247	1.90663318914253	0.0565681041454586	0.262545984394695
**Cnga1**	651.953092097116	-0.268428991636194	0.140809660400036	-1.90632511202424	0.0566080381185549	0.262653962495309
**H2-Q2**	100.961292405091	1.31339892678854	0.689370714999493	1.90521427471648	0.056752223387402	0.263167976431811
**Gm37653**	93.4392512832879	-0.717813526110781	0.376756445318026	-1.90524551080968	0.0567481648103408	0.263167976431811
**St6gal1**	8731.49186794856	-0.288941937934741	0.151678863011583	-1.90495849057542	0.056785467094257	0.263186340303971
**Tcaim**	1100.62633162816	-0.275975754278422	0.1448770184926	-1.90489669893723	0.0567935004482856	0.263186340303971
**Eef2-ps2**	134.86193130567	-0.437305941394575	0.229581229780535	-1.90479832263557	0.0568062920209308	0.263186340303971
**Sdad1**	4421.40877329894	-0.131349233661155	0.0689660386030687	-1.90454949017922	0.0568386576527413	0.263258885547985
**Prr7**	88.6216712397392	1.05767316316863	0.555387401397204	1.90438810910693	0.0568596566864476	0.263278757099134
**Furin**	584.577084119019	-0.307390108704976	0.161425766346665	-1.90421960298983	0.0568815897251105	0.263302940451988
**Apobr**	225.369090183638	0.329504577985779	0.17309925170992	1.90355865049009	0.0569676883781996	0.263394561583508
**Gm13285**	32.8842958862974	-0.639346359191308	0.335835562219813	-1.90374823608716	0.0569429810211058	0.263394561583508
**Ptrhd1**	396.408913526131	0.297972213114242	0.156534648265316	1.90355436586281	0.0569682468665733	0.263394561583508
**Tmem185b**	2431.92220765185	0.166154530390248	0.0872727739427281	1.9038529759497	0.0569293348353425	0.263394561583508
**Gm8873**	23.3001759706667	-0.911124143787131	0.478678116467604	-1.90341716582064	0.0569861328908575	0.263399969481151
**Zfp174**	69.1943753698995	-0.610037156619368	0.3205431561199	-1.90313580237908	0.0570228273287871	0.263492284521906
**Ppif**	3026.043318123	0.144419138936427	0.0759041413857643	1.90265163797126	0.0570860163740967	0.263495381286739
**Parp1**	10298.1370253402	0.133833052739171	0.0703394885032005	1.90267310137009	0.0570832139194144	0.263495381286739
**Raly**	15241.3111239767	0.226865490962549	0.119242425760447	1.90255682502058	0.0570983973764308	0.263495381286739

**Nsdhl**	2118.23715819058	0.292044392753147	0.153506391510413	1.90249011705377	0.0571071096715245	0.263495381286739
**Gm18666**	318.445074081853	0.360995823510803	0.189716446398682	1.90281775968006	0.0570643290122274	0.263495381286739
**Hps6**	1586.11095118334	-0.283954527765642	0.149296704045447	-1.90194773274569	0.057177988024272	0.263745186562779
**Myg1**	3647.3177164345	0.0958549362711635	0.0504107440772885	1.90147830637455	0.0572393913457465	0.263938504968326
**Trav10**	473.221691410167	0.329961381825497	0.173538638129802	1.90137127605378	0.0572533991230398	0.263938504968326
**Hectd2**	261.262661066207	0.657785918159765	0.345988178657762	1.90118032561575	0.0572783971705819	0.263976514833828
**Nsun2**	20901.5891185299	0.124274481265465	0.0653772218205625	1.90088348517707	0.0573172757038865	0.264001260820269
**Gm38057**	73.6664018884165	-0.922487886937841	0.485268394812547	-1.90098489165812	0.0573039915716374	0.264001260820269
**Cdyl**	533.92880168231	0.545057064225712	0.286765585562754	1.90070598309794	0.0573405344366056	0.264031210145411
**Rpl22**	2256.6037292013	-0.171302827912169	0.0901422659933051	-1.90036079107321	0.0573857886571831	0.264162393184702
**Snx10**	1287.09564978792	0.381991516462352	0.201048648967641	1.89999544102301	0.0574337179165746	0.264305809933255
**Ahsa1**	11807.2639305054	0.142905666611059	0.0752368666094116	1.89941013031478	0.0575105725822088	0.264537892855761
**Gm7160**	212.396442160781	-0.284131011180693	0.149593367065656	-1.89935567835697	0.0575177267832605	0.264537892855761
**Ralgapa2**	2158.36486075328	-0.206464688715661	0.10871449474331	-1.89914591612786	0.0575452934276068	0.264587449238051
**Kansl1l**	911.06238372108	-0.322992042342264	0.170232226551342	-1.89736132156416	0.057780266782744	0.265590333633517
**Gm10076**	1707.34966785172	-0.439106614207154	0.231532668986379	-1.8965211956028	0.0578911599731104	0.266022457770866
**Pdia5**	124.074540471978	0.595648444336819	0.314281604477551	1.89526983396626	0.058056662499816	0.266705198545073
**5830444B04Rik**	408.726804686221	1.44741353434651	0.763922614583712	1.89471224796147	0.0581305342082144	0.266888935757236
**Scarna13**	41.4498568590671	-0.85943141031137	0.453583521166573	-1.89475889269742	0.0581243514945074	0.266888935757236
**Epsti1**	19086.822546358	-0.188674646774684	0.0995885055477534	-1.89454240463739	0.0581530513907668	0.266914544353135
**Vps33b**	4274.60264695124	0.162798964045076	0.085941359008681	1.89430288190616	0.0581848187207099	0.26698258258073
**Tmem38b**	273.312703401646	0.437606261064185	0.231066155064591	1.89385702523963	0.0582439900284821	0.267020817829151
**Micu3**	769.777040125275	-0.246593247275663	0.130202749596478	-1.89391735612266	0.0582359803675364	0.267020817829151
**Gm9774**	265.116536317834	0.296182845172796	0.156389885618372	1.89387468378583	0.058241645546252	0.267020817829151
**Lzic**	1655.02001305162	0.144831041584956	0.0764987711580915	1.89324664164408	0.0583250773825798	0.267277510528314
**Ube3c**	2190.27285274492	0.732161888723559	0.3867364969512	1.89318022605957	0.0583339061183521	0.267277510528314
**Sh3bgrl2**	155.983638414438	0.439681308966699	0.232278302412601	1.89290736327874	0.0583701898793437	0.26736601218861
**Fyco1**	6541.16677658317	-0.288545809894757	0.152501893820832	-1.89208017464856	0.0584802993597098	0.267560786170748
**Tlr13**	91.8888554945008	0.697555772677017	0.36867185001264	1.8920776637899	0.058480633849848	0.267560786170748
**Spty2d1**	3661.01872073194	0.242199046463863	0.128002309451412	1.89214591128763	0.058471542658967	0.267560786170748
**AI506816**	13431.5245463341	0.138482817109854	0.0731866163679747	1.89218772478258	0.0584659732989617	0.267560786170748
**Akt2-ps**	358.010544710387	0.334720258026112	0.176931508621632	1.89180695193139	0.0585167067108431	0.267648112523296
**Gsn**	4593.29665445306	-0.158696867394031	0.0839006321724242	-1.89148595528926	0.0585595040601288	0.267766136237797
**Lpcat4**	6872.19222168584	0.167081789083068	0.0883402192658262	1.8913445141029	0.0585783701635303	0.267774696451044
**Hnrnpm**	32495.2617881735	0.0912411869186859	0.0482447784158582	1.89121372124894	0.0585958204332094	0.267776781486682
**Sf3b2**	27725.4790817801	0.0864519732464569	0.0457188227547389	1.89094924228984	0.0586311201791923	0.267860411905925
**Tmem37**	450.785875572071	0.512046480485207	0.270834291643195	1.89062646896942	0.0586742243422672	0.26797963853945
**Cab39l**	6088.36408973291	-0.157244868668613	0.0831771233562665	-1.89048217014067	0.0586935029713495	0.267990010524356
**Edaradd**	328.108785433443	0.509767111427182	0.269725013240239	1.88995119623237	0.0587644875041909	0.268236393280283
**Lrba**	1182.79336249152	-0.426343128796788	0.225620497650048	-1.88964714304493	0.058805167678305	0.268343551197278
**Slc35d1**	846.680939106187	-0.191456834969362	0.101325581341112	-1.88952120910931	0.0588220235981086	0.268343551197278
**Lrrc59**	9072.39310868291	0.207168981181577	0.109648653373386	1.8893891972946	0.058839697332252	0.268346486501967
**Tmem9b**	13446.6340780424	-0.15611803655658	0.0826501569553588	-1.88890187638607	0.0589049780104016	0.268566475263281
**Eif2b2**	7223.09657387024	0.132208974195998	0.0700078344074018	1.88848827156436	0.0589604309710482	0.268660202526188
**Cd274**	2281.41982809491	0.245660286248278	0.130074189421528	1.88861669898379	0.0589432077709179	0.268660202526188
**Fam104a**	3718.6762889643	-0.147288244756609	0.0779993724326143	-1.88832602318506	0.0589821958201012	0.268660202526188
**Inpp5f**	1716.91762425719	-0.278165384087076	0.147314632638054	-1.88824001462582	0.0589937361642832	0.268660202526188
**Prelid3b**	3039.94291773746	0.168188919375084	0.0890915467448186	1.88782129753365	0.0590499450178419	0.268838481261524
**Fhod1**	263.144635919051	-0.399955605185455	0.211879028384976	-1.88766018153883	0.0590715851740095	0.268859320504583
**Gm5637**	710.765041208206	0.215548453271644	0.114196814741102	1.88751721105635	0.0590907936434623	0.268869083291954
**Tspan32**	13036.6984574234	-0.189508219180836	0.10041906726682	-1.8871736647115	0.05913697121802	0.268923883256087
**Ptpn7**	15272.0316550508	0.135148244068372	0.0716106845263647	1.88726368086336	0.0591248688564085	0.268923883256087
**Ubc**	114930.994079572	0.212399677053303	0.112567536119641	1.88686440491651	0.0591785658002049	0.26899398467595
**Fam149b**	4634.80285685059	-0.219111305954916	0.116128206681573	-1.8868052148237	0.0591865294708077	0.26899398467595
**Trrap**	5589.75249933845	-0.21895976200828	0.116067246245512	-1.88649053967494	0.0592288820526843	0.269108850779743
**Tmem123**	26429.1422097212	0.116470391273771	0.0617438728375076	1.88634735596013	0.0592481616798449	0.269118847964634

**Tjap1**	2339.32496546804	-0.200114918918318	0.106109825638546	-1.88592260626261	0.0593053846922075	0.269233544047035
**Gm20069**	881.389964830809	0.212798932452048	0.112836430627417	1.88590627396488	0.0593075859229078	0.269233544047035
**Zfp326**	793.749055228614	-0.280393671173834	0.148689584531036	-1.88576538200836	0.0593265778423317	0.269242190973969
**Ube2d1**	2683.03279637136	-0.116452576225027	0.0617731651608581	-1.88516447104148	0.0594076359659718	0.269532427272047
**Rad23a**	12027.0546843958	0.118056031970071	0.0626519122831217	1.88431649837885	0.0595221770732718	0.269550372530609
**Pak4**	716.232879388217	0.263102671799522	0.139628462272962	1.88430544544119	0.0595236712733097	0.269550372530609
**Dtnbp1**	1959.81714242164	0.298852998476915	0.158588974971064	1.88445002895973	0.0595041280958808	0.269550372530609
**Fam133b**	1332.68476552752	-0.201163139049061	0.106736374895718	-1.88467276732603	0.0594740312415711	0.269550372530609
**Patj**	6060.11811456773	-0.280090918616597	0.148648590513569	-1.88424873487804	0.0595313382246949	0.269550372530609
**Cenpb**	313.395728516479	-0.444265199315418	0.235730897529156	-1.88462863363285	0.0594799936710251	0.269550372530609
**Gm34794**	425.083947121713	-0.489688871297934	0.259867708590462	-1.88437753176043	0.0595139267881106	0.269550372530609
**Daxx**	11927.6577248085	-0.121392806855968	0.0644486260230847	-1.88355926800498	0.0596246158445119	0.269861386635796
**Arih1**	2743.95746149373	0.292665682536913	0.155440687216747	1.88281258772884	0.0597257705588608	0.269861386635796
**Ect2**	402.453728634606	0.252572673718031	0.134161397523226	1.88260318080174	0.0597541650042707	0.269861386635796
**Cenpu**	226.659465037961	-0.397287003477381	0.211017667886025	-1.88271914601939	0.0597384393655642	0.269861386635796
**Cd300a**	266.452833925098	0.565152951743301	0.300163689520439	1.88281584840001	0.0597253285180147	0.269861386635796
**Qsox2**	1967.63468323002	0.184703384674794	0.098092383011364	1.88295338541624	0.0597066854479476	0.269861386635796
**Ccdc126**	934.031327353751	-0.236029827337928	0.125358999641241	-1.88283113309304	0.059723256447469	0.269861386635796
**1700122E12Rik**	43.5466506913815	-0.919192878265945	0.488167127616722	-1.88294710205812	0.0597075370485876	0.269861386635796
**Gm5837**	68.3960569433525	0.651919967999746	0.346141478154659	1.8833916451598	0.0596473117660196	0.269861386635796
**Aadacl2fm2**	138.964630951627	-0.355513548896083	0.188916392978851	-1.88185653605974	0.0598554971384373	0.270164155946822
**Gm5297**	43.1952137054375	-0.874229361672874	0.464536036332616	-1.88194089004305	0.0598440417606393	0.270164155946822
**Bzw2**	7612.17149483924	0.0911083961119933	0.0484240990469774	1.88146806869048	0.0599082749743349	0.270170202853633
**Nrip1**	7823.84697085274	-0.218211337011915	0.115976133977759	-1.88151932236129	0.059901309354295	0.270170202853633
**Gm12195**	25.6087563138931	-1.0511268111388	0.558632529008801	-1.88160688208373	0.0598894111213512	0.270170202853633
**Fam129a**	1273.47796504246	0.299583395026459	0.159250806692383	1.88120488208985	0.059944053879429	0.270254208005197
**Katnb1**	4119.33366869993	0.191988365699088	0.102138743997598	1.87968206955436	0.0601514207022452	0.271111537758947
**Hdhd5**	7331.26565074252	0.174487263431014	0.0928415783581981	1.87940862829597	0.060188719090573	0.271202072264844
**Reep2**	83.9599786648993	-0.712800316387993	0.379303011028536	-1.87923716833971	0.0602121166359981	0.271212853864834
**D930030I03Rik**	32.1223041371067	-0.731849831410078	0.389467239690153	-1.87910498452273	0.0602301596791349	0.271212853864834
**Gm37017**	36.7105366805281	-0.843895222510288	0.44911627664818	-1.87901277773409	0.0602427485261736	0.271212853864834
**Afg3l2**	7536.90861902919	0.132672344955971	0.070642484462124	1.87808152510697	0.0603700132847212	0.271555343392765
**Mrm3**	1710.9117658889	0.247598495217254	0.131836123971532	1.87807778140322	0.0603705253477728	0.271555343392765
**Gm43361**	352.197290501226	0.470883389279518	0.250712146936452	1.87818338693766	0.0603560820254848	0.271555343392765
**Naga**	2776.93306554826	0.231515024828413	0.123305822606495	1.8775676601035	0.0604403333137837	0.271791761422743
**Rab30**	34.175890253925	0.92854603320716	0.494676239299405	1.87707829776144	0.0605073633680399	0.271813619847883
**Asprv1**	40.3142609749171	0.882530376441594	0.470118218320492	1.87725202310698	0.0604835604136035	0.271813619847883
**Rp2**	1063.55233037861	0.157826162931762	0.0840829894873679	1.87702844408824	0.0605141954957399	0.271813619847883
**Gm38142**	138.158168631773	-0.747422352357957	0.39819446351854	-1.87702848943091	0.0605141892815259	0.271813619847883
**N4bp2l1**	7423.91007894973	0.17062451251019	0.0909106514698342	1.87683741950531	0.060540380114344	0.271853738803567
**Pdlim7**	401.331634204276	0.268048072363785	0.142953770809601	1.87506821852776	0.0607833391337862	0.272720874394485
**Tm2d2**	2586.3008976221	0.197816780246573	0.105490340013834	1.87521227271266	0.0607635264427867	0.272720874394485
**Sde2**	4212.61973971327	0.204160398157561	0.108904374286771	1.87467582908983	0.060837334105262	0.272720874394485
**Epg5**	2270.1640530667	-0.25094772003369	0.133856092306605	-1.8747575527521	0.0608260851990728	0.272720874394485
**Zfp84**	1913.308641392	-0.173438599471656	0.0925099169477	-1.87481088724475	0.0608187448677912	0.272720874394485
**Tlr11**	173.362191237756	0.713853597430836	0.380764140787241	1.87479208508166	0.0608213324922633	0.272720874394485
**Ly75**	6692.97388659708	-0.250038141038218	0.133386190936056	-1.87454292894595	0.0608556309117467	0.272725306108189
**Trib1**	268.818290644981	0.528116372431144	0.281770885050487	1.87427587607754	0.0608924107565125	0.272746124551295
**F830112A20Rik**	18.9278586779338	-1.26977590762728	0.677481977206208	-1.87425784057543	0.0608948953589805	0.272746124551295
**Chst14**	386.702111476208	-0.284972807093585	0.152058680427372	-1.87409759372268	0.060916974935641	0.272767483393264
**Mettl17**	4120.10207984136	-0.119818203796344	0.0639756817367267	-1.87287107450329	0.0610861903531899	0.27296458119169
**Dip2b**	4823.94249723296	-0.241605290762558	0.129048683304105	-1.87220268023356	0.0611785684348497	0.27296458119169
**Ndrg3**	14800.8019003516	0.189274501142916	0.101091133377131	1.8723155515213	0.061162960496191	0.27296458119169
**Amt**	73.021300784094	0.622188928305917	0.33225497690297	1.8726248560834	0.061120206496347	0.27296458119169
**Nhsl1**	73.1257247179794	-0.801366456767909	0.427949570978868	-1.87257216997532	0.0611274873482066	0.27296458119169
**Plcb4**	165.580124624582	0.557775063613074	0.297834853448001	1.87276625672172	0.0611006694672357	0.27296458119169

**Elavl1**	7569.31005633629	0.691797567262351	0.369511570704656	1.87219459986895	0.0611796859211293	0.27296458119169
**Mdfic**	243.05657292136	0.483497571785238	0.258080314446539	1.87343840161584	0.0610078713398692	0.27296458119169
**Micos10**	3093.59516526436	0.124048191695591	0.0662359818200841	1.8728218150754	0.061092994501851	0.27296458119169
**H4c4**	455.274601532021	0.591010503165836	0.31568151141531	1.87217331960979	0.0611826289877349	0.27296458119169
**Ccdc47**	9374.63775704702	0.135359441692456	0.072286879357319	1.87253126564456	0.06113314053615	0.27296458119169
**Gm43647**	38.2064791288344	-0.830327680691131	0.443252293915939	-1.87326200470516	0.0610322138640947	0.27296458119169
**Gm47826**	45.9110066543722	-0.856519505743302	0.457506416923167	-1.87214752418903	0.0611861966598369	0.27296458119169
**Wwp1**	2349.19115376605	-0.196150110554791	0.10479829367946	-1.87169183455165	0.0612492498705267	0.273168533883354
**Pvr**	1248.50475712596	0.237781309075517	0.127059266598407	1.87142044371361	0.0612868274451464	0.27325878326349
**Dcbld2**	102.484691975729	-0.884630863018326	0.472861572546232	-1.87080303069422	0.0613723873968561	0.273472423267195
**Trgv4**	17.2773125570726	1.52074930746229	0.812872995336493	1.87083260999803	0.0613682860972115	0.273472423267195
**D130040H23Rik**	807.013680394735	-0.331583419240623	0.177251078032555	-1.87069902717163	0.0613868097414526	0.273472423267195
**Plcxd2**	5712.38169976706	-0.264854345518519	0.141602399496193	-1.87040859802408	0.0614270989043268	0.273574561174527
**Alyref2**	418.537900896698	-0.321337398574907	0.17185838396926	-1.86978017105283	0.0615143510375974	0.273885738711337
**Gtdc1**	252.066478901372	-0.391272744885165	0.209293569073277	-1.86949243886311	0.0615543346168152	0.273973716787708
**Gm11739**	20.5895030165723	-1.26531929818953	0.676862940031044	-1.86938776428135	0.0615688856473487	0.273973716787708
**Tent5a**	4572.52686784446	-0.337692757372039	0.180657328161904	-1.86924472318887	0.0615887746905843	0.273975026486521
**2610035D17Rik**	4461.91538869714	0.388422726767647	0.207808749887317	1.86913557286817	0.0616039549940815	0.273975026486521
**Rragd**	259.452664967146	0.61469538983329	0.328901255655256	1.86893597778658	0.0616317220980343	0.273993441751158
**Gm49588**	63.0662252723443	0.763090107797954	0.408319405943364	1.86885584346632	0.061642873072784	0.273993441751158
**Rapgef4**	4483.69832896917	-0.254786888660863	0.136355524566934	-1.86854833693074	0.0616856791998107	0.274106386675527
**Nop58**	7113.84121433331	0.148998391025097	0.0797724962405093	1.86779150768801	0.0617911376390498	0.27411119250521
**Sord**	228.120283350301	0.447239992168447	0.239410984361825	1.86808468024394	0.0617502685642941	0.27411119250521
**Zmpste24**	3360.5755182989	-0.14530308387005	0.077791841506595	-1.86784476438614	0.0617837118426641	0.27411119250521
**Amigo1**	147.828596562836	0.659336061817972	0.352986290229322	1.86788008505834	0.0617787873461646	0.27411119250521
**Ceacam1**	139.163671073175	0.587537353812892	0.314496348427615	1.86818497814171	0.0617362918942564	0.27411119250521
**Gm16794**	192.632421988964	-0.510736172010899	0.273425132383837	-1.86791962961884	0.0617732743300699	0.27411119250521
**Capza1**	56026.3063094087	0.128166382790588	0.0686472262103368	1.86702930134253	0.0618974961123012	0.274505726496349
**Kmt2b**	929.069027337265	-0.304804207569821	0.163277044938441	-1.86679154858994	0.0619307031776443	0.274575714679274
**Gm32051**	47.8092264300592	-0.760748341596325	0.407544453804416	-1.86666346332225	0.0619485990314357	0.274577798857477
**Gm19951**	128.26760080016	0.666031691372765	0.356994138192242	1.86566562337812	0.0620881620975225	0.275119001735282
**A630035G10Rik**	172.14250117109	-0.52124121499984	0.279441778480807	-1.86529450905151	0.0621401343981268	0.275271885777701
**Gm34006**	1973.4734985402	-0.46366640680051	0.248608650561395	-1.8650453463847	0.0621750483036075	0.275349138875757
**Hnrnpul2**	1026.80018232012	-0.308933391895673	0.165677068639758	-1.86467200580067	0.0622273930102675	0.275448557652813
**Gm10640**	441.445620200044	-0.316886841650127	0.169945695296871	-1.86463588322476	0.0622324595572771	0.275448557652813
**Pcsk4**	902.581406515257	0.291733133783786	0.156501830061315	1.864087682997	0.062309391936841	0.275634219188322
**Gsap**	2453.30239676829	0.242031824407023	0.129837468881998	1.86411385319744	0.0623057175209228	0.275634219188322
**Zfp551**	381.901908903073	-0.415397685671167	0.222905813997847	-1.86355698050648	0.0623839436795208	0.275886556429472
**Inpp5b**	21192.8185785623	0.145653186984995	0.0781661879719415	1.8633784090543	0.0624090455292896	0.275920126376267
**Stk40**	5487.68249674601	0.206990147900548	0.111105459635132	1.86300608971241	0.0624614094550471	0.276074173571719
**Trmt11**	2448.07587113649	0.238811305902772	0.128240569808148	1.86221338738624	0.0625730181509583	0.276423387792367
**Erbin**	4358.4140468351	-0.209259993419806	0.112380228766776	-1.86207125324586	0.0625930473848554	0.276423387792367
**Pik3ip1**	40975.6171791868	-0.179178363495165	0.0962194401241429	-1.86218464027631	0.0625770687035004	0.276423387792367
**Canx**	17161.7311268874	0.131510541686174	0.0706586884338145	1.86120836094147	0.0627147581228403	0.276883284881366
**Gorasp1**	926.320640827663	-0.271847998453161	0.146124940975238	-1.86038055268901	0.0628317041194525	0.277166618647876
**Zfp617**	1881.8127229067	-0.225102252675779	0.120985990431793	-1.86056461473267	0.0628056857598329	0.277166618647876
**Gm43961**	43.0777665209323	-0.941550282812529	0.506092413366924	-1.86043152978445	0.062824497285389	0.277166618647876
**Naa35**	3395.46536968613	0.220528130530947	0.118564080233677	1.85999107062029	0.0628867893076652	0.277331972885777
**Cntrob**	532.854361069339	-0.424197495061189	0.22808641233164	-1.85981045834682	0.0629123471911905	0.27736705503402
**Cyth2**	5492.48826398068	0.157494113956369	0.084690837045623	1.8596358171726	0.0629370682882689	0.277398429375735
**Ccdc88b**	5154.45402024626	-0.185623900845555	0.0998252819120832	-1.85948787010724	0.0629580170191844	0.277413163918146
**Sec11c**	13312.4813796406	-0.156086246435814	0.0839821334047893	-1.85856491265216	0.0630888344001079	0.277907529491776
**Slc18a1**	24.6004290157854	-1.36656514913828	0.73532618814578	-1.85844754500619	0.0631054858489289	0.277907529491776
**Stambpl1**	4501.02378215121	-0.146784086089821	0.0789987405537193	-1.85805602799462	0.0631610583102613	0.277996870333739
**Ndufaf5**	970.098490770601	0.204444119068156	0.11002646178472	1.85813590432615	0.0631497172712125	0.277996870333739
**Esyt2**	15830.4304753257	-0.182845018123295	0.0984236450723908	-1.85773467329738	0.0632067020553991	0.278086184864631

**Fech**	4781.60830683633	0.186297983625717	0.100287462433228	1.85763981963105	0.0632201798440458	0.278086184864631
**Vegfb**	217.91991894951	0.651507567190016	0.350736670350823	1.85754049195469	0.0632342958919828	0.278086184864631
**Ltv1**	4427.44328854632	0.137550595475614	0.0740657199607937	1.85714248843359	0.0632908846763861	0.278179768436769
**Csnk1g1**	2771.25427003617	-0.24427719301489	0.131531258033599	-1.85717978119309	0.0632855805552053	0.278179768436769
**Gm48086**	111.292235888625	-0.747119566217984	0.402397057246695	-1.85667253963031	0.0633577566313965	0.27839603213634
**Prr5l**	245.289770227659	0.843431206163062	0.454471951655392	1.85584875610234	0.0634751188111305	0.278833969084746
**Hacd4**	79.1673363676368	-0.546786001890286	0.294719279362185	-1.85527734416836	0.063556631764052	0.278881048929593
**Zfp626**	459.265560791116	-0.474927776661708	0.25595755224065	-1.85549428998752	0.0635256738698984	0.278881048929593
**Gpr22**	41.208885790859	-0.939949579359023	0.506619807372047	-1.85533523498569	0.0635483695986338	0.278881048929593
**Tsnax**	12505.6432919835	0.126175017885338	0.0680035240550154	1.85541881304947	0.0635364429200557	0.278881048929593
**A930005H10Rik**	4309.04773358423	-0.176874251922513	0.0953605817380885	-1.85479417909074	0.0636256236017591	0.278948378910273
**Borcs7**	395.339274949219	0.377603521286852	0.203573114185706	1.85487913174227	0.0636134886095235	0.278948378910273
**3830408C21Rik**	77.1614112841614	0.639400680270912	0.344731914906574	1.85477657455706	0.0636281385462059	0.278948378910273
**Trav6d-6**	456.51804941295	0.251020835288865	0.135344988144392	1.85467403507447	0.0636427887403967	0.278948378910273
**Bnip1**	1389.07817377286	0.163357144749577	0.0880910093970744	1.85441336031509	0.0636800448515259	0.279034056375276
**Whrn**	123.193500402604	0.872795613819965	0.470795965898346	1.85387232907688	0.0637574275213635	0.279295464402036
**Tubb4b**	11816.0697986762	0.254829589364885	0.137476514846602	1.8536227052979	0.0637931569122871	0.279374311691747
**9130019O22Rik**	1438.8973689244	-0.363292364291147	0.196030766086564	-1.85324156786044	0.0638477421436315	0.279535669062851
**Ndufa12**	2341.43026599592	0.269892267506011	0.145700925629241	1.85237167396447	0.063972469946061	0.279585987242222
**E2f5**	850.307295323505	0.398133337073088	0.214938971559781	1.85230874691495	0.0639815003999568	0.279585987242222
**Cd22**	879.596263049033	0.538239202952571	0.290555653056174	1.8524478780267	0.0639615355608528	0.279585987242222
**Ddx24**	15264.7948318931	0.105672660690528	0.0570323754146865	1.85285392590041	0.0639032984933908	0.279585987242222
**Map2k3os**	55.7019931896285	0.894649780362839	0.482885054771103	1.85271789119032	0.0639228042749569	0.279585987242222
**Gm16299**	571.411384320429	-0.736689371365117	0.397717037558546	-1.85229522951144	0.0639834403754175	0.279585987242222
**Gm16754**	185.520091697073	-0.39525734537886	0.213346798513702	-1.85265187072153	0.0639322726077545	0.279585987242222
**Slc38a9**	1933.2571352988	-0.245261833771674	0.132431662167726	-1.85198788384191	0.0640275627739833	0.279701221688288
**1700019D03Rik**	50.1816231279192	-0.68878568687893	0.371964736536611	-1.85174996235466	0.0640617359200416	0.27977294148894
**Htr7**	30.8361628667037	1.54831644115888	0.836267254920282	1.85146127873496	0.0641032203558314	0.279799014722115
**Faap100**	2999.33641727037	0.180489437206271	0.097482560634814	1.8515048848831	0.0640969526399236	0.279799014722115
**Lat2**	127.54792045111	0.549610647048163	0.296886978164379	1.85124538114251	0.0641342597836725	0.279856973528476
**Hnrnpa2b1**	49172.5685061216	-0.150788692194379	0.0814679273844609	-1.85089638383436	0.0641844610957213	0.27999849210556
**Becn1**	18765.5945816589	0.157419547390002	0.0850911389701791	1.85001105044758	0.0643119569092756	0.280058351829422
**Slc39a11**	3834.06205502311	-0.114950140545517	0.0621225789384668	-1.85037618382483	0.0642593491746878	0.280058351829422
**Gm6377**	100.607370557843	0.850662532618738	0.459816380324763	1.85000484762619	0.0643128509072162	0.280058351829422
**Kbtbd2**	6710.18462236799	0.600926680090386	0.324836255024752	1.84993722466292	0.0643225979101781	0.280058351829422
**Spaca6**	56.3060693916771	-0.642802222122939	0.347393151837862	-1.85035950974345	0.0642617507708006	0.280058351829422
**Gm16077**	43.2444295894359	-0.745434253370175	0.402906381034847	-1.85014258512253	0.064293001543649	0.280058351829422
**Gm44892**	32.6177243306543	-0.864694735916423	0.467365603607516	-1.85014628642329	0.0642924682186337	0.280058351829422
**Sfswap**	4291.39161159074	-0.166874529317849	0.0902363996724291	-1.84930393858385	0.0644139373615718	0.280223746826694
**Marchf1**	279.48463209249	0.632640878107034	0.342058329901583	1.84951168500723	0.0643839621055472	0.280223746826694
**Bri3bp**	1578.72624685735	-0.160592261562485	0.0868343097399817	-1.84941023937848	0.0643985980243681	0.280223746826694
**Gm42829**	16.9364292221967	-1.30027275995216	0.703173883862445	-1.84914825449707	0.0644364082121855	0.280244130333813
**Slc2a1**	6200.13697229326	0.150047781643712	0.0811546778126776	1.8489110632668	0.0644706559326896	0.280315707928088
**Arrdc3**	1241.03746347028	-0.347521005999497	0.188079255275837	-1.84773703771754	0.0646403931823277	0.280910212192751
**Gm37879**	208.117554808249	-0.507514634697762	0.274670936980148	-1.84771872946442	0.0646430430545735	0.280910212192751
**Ttc9c**	3192.14037481047	-0.181559120424931	0.0982757341299278	-1.84744608658834	0.0646825150352488	0.280956560692683
**Gm12184**	85.6788376900154	0.694066044863705	0.375699098046688	1.84739875201258	0.0646893699430763	0.280956560692683
**Cela1**	98.0804403610226	0.859757865292397	0.465472153334563	1.84706616525444	0.0647375514625041	0.281086406197697
**Gm42715**	3205.67068702355	-0.352361545710077	0.190780626850615	-1.84694615761999	0.0647549441199239	0.281086406197697
**Sgpp1**	7540.59821027885	1.15668226627922	0.626380180670641	1.84661376903848	0.0648031371907877	0.281218130739532
**Sdhd**	10595.8087466905	0.157385486623698	0.0852866106048064	1.84537157131238	0.0649835056551034	0.281576740240079
**Zfp324**	670.647462555277	-0.205480499952456	0.111395708622857	-1.84459978299644	0.0650957786031688	0.281576740240079
**Pelp1**	1100.10330357872	-0.204995558561665	0.111099499567398	-1.84515285271204	0.0650153066733689	0.281576740240079
**Tubd1**	1543.17243209603	0.237349537092636	0.128676721063055	1.84454138349025	0.0651042805549932	0.281576740240079
**Ngly1**	11789.5696776403	0.110984407497639	0.0601458431895895	1.8452548274666	0.0650004782576505	0.281576740240079
**Kdm4b**	12263.703995162	0.175430482784399	0.0951146878170482	1.8444100150109	0.0651234088669816	0.281576740240079

**Prdx3**	4817.88043303918	0.141740959079651	0.0768525315240687	1.84432387936707	0.0651359534286405	0.281576740240079
**Pxn**	3937.5779780964	-0.142369337092904	0.0771917630872986	-1.8443591828819	0.0651308116794294	0.281576740240079
**Ublcp1**	1523.97606833886	-0.197867002733971	0.10725908900713	-1.84475744261465	0.0650728306797027	0.281576740240079
**Stard5**	5569.09010965222	-0.228759475668702	0.123960752905422	-1.84541857246735	0.0649766735056127	0.281576740240079
**Zfp706**	14038.3217359932	0.212120524944801	0.114922543262606	1.84576949763538	0.0649256812896525	0.281576740240079
**Ftl1-ps2**	35.581979042284	0.839738660966899	0.45495669478188	1.84575514680467	0.0649277659317859	0.281576740240079
**Gm44064**	18.6873655359626	1.30586361466831	0.707977231957349	1.84449944959102	0.0651103859659167	0.281576740240079
**Gm48876**	447.281598516325	-0.44555080008664	0.241408328731192	-1.84563143462528	0.0649457390016856	0.281576740240079
**Ext2**	11543.9296323586	0.109919127052739	0.0596068867334717	1.84406757467866	0.0651732927379284	0.281660881423899
**Eif4ebp1**	1209.68928897731	0.226493293511496	0.122836042499824	1.8438667422212	0.0652025630050314	0.281710113866268
**P2ry10b**	1093.96680165073	-0.278726504472617	0.151179904226612	-1.84367430247091	0.065230620250407	0.281754079848043
**5330438D12Rik**	396.235864105065	-0.398220295550649	0.216037279173455	-1.84329434750435	0.06528604599392	0.281916203542394
**Cog1**	6432.25011209214	0.138658979229779	0.0752592291543374	1.8424182759755	0.0654139905190642	0.282391300988738
**4933439C10Rik**	417.966353312327	-0.268136320176577	0.145558307104566	-1.84212310180245	0.0654571453172733	0.282422847416286
**Gm43267**	124.092328892621	-0.512486754205889	0.278187653871478	-1.84223399950976	0.0654409291947807	0.282422847416286
**Ogdh**	34288.9017151267	0.122639500335509	0.0666138607356973	1.84105078103946	0.065614117411632	0.282943555428337
**Immt**	12353.9885512164	0.130017188877239	0.0706257881825855	1.84093080194888	0.0656316998851668	0.282943555428337
**9330151L19Rik**	946.630200781897	-0.198450723172964	0.107786856331132	-1.8411402830351	0.0656010037679343	0.282943555428337
**Ccm2**	27937.160226235	-0.118993378068055	0.0646515104035648	-1.84053516035866	0.0656897071602528	0.282961376633161
**Ints10**	4474.43580187177	0.166082285097063	0.0902342369700689	1.84056839924466	0.0656848321915917	0.282961376633161
**Mgst2**	3641.08002018012	-0.236881256496964	0.128696549723539	-1.84061854809491	0.0656774776938201	0.282961376633161
**Fam120c**	510.939728849675	-0.368130556399432	0.200121907464666	-1.83953151887896	0.0658370463801373	0.283518540533431
**Tnfsf14**	647.886932104561	0.438736956313217	0.238574008120193	1.83899729802998	0.0659155834877808	0.283779193720481
**Pdhb**	15601.6653889362	0.0991341385200761	0.0539229887213103	1.83843924216497	0.0659977070782745	0.283977572482898
**Soat1**	2744.96685332934	0.186491278795342	0.101439347170688	1.83845109414535	0.0659959620630096	0.283977572482898
**Cyp4f41-ps**	50.9639363519002	-0.532397014819859	0.289627229914876	-1.83821464858311	0.0660307820924747	0.284042324169021
**Cby1**	542.51151560988	0.245866619201791	0.133810015211492	1.83743061992175	0.0661463499962064	0.284461800461306
**Bcl7b**	9537.91293501894	0.114975113969255	0.062585631539455	1.83708482508117	0.0661973740080627	0.284603553136438
**Mdp1**	6747.45459126629	0.12342304443967	0.0672224038780435	1.83604032762034	0.0663516925402095	0.284909967373976
**Crybg1**	7964.03549084728	0.216982830411333	0.118193488821065	1.8358272742065	0.0663832063260126	0.284909967373976
**Uqcrb**	2321.58810465496	0.198845707730497	0.108316320391148	1.83578713727011	0.0663891445590262	0.284909967373976
**Kdm1b**	5369.32202413766	0.11769339155586	0.0640931754360273	1.83628585657036	0.0663153904229636	0.284909967373976
**Rgmb**	1241.76683165224	1.03434616001006	0.563447283846727	1.83574611088449	0.0663952148376747	0.284909967373976
**Otulinl**	32706.6614633558	-0.11625314582618	0.0633264505234935	-1.8357754913652	0.0663908676448007	0.284909967373976
**4930466K18Rik**	17.6447116128924	-1.1448085811171	0.623520954009435	-1.83603866679319	0.0663519381537115	0.284909967373976
**Arl13b**	864.394467907598	-0.241306517899862	0.131501084140156	-1.83501542574868	0.0665034038762141	0.28498616667868
**Cmas**	6860.59267930647	0.335244115098	0.182660584803994	1.8353391097359	0.0664554596420436	0.28498616667868
**Ciao2a**	4625.97130803535	0.222356310314187	0.121163447386434	1.83517649184257	0.0664795431259	0.28498616667868
**Gm42571**	72.7541299043827	-1.00180424275879	0.545931193372555	-1.83503755586858	0.0665001250447159	0.28498616667868
**Usf2-ps1**	77.0115788225318	-0.452230187688669	0.246397469270666	-1.83536863843312	0.066451087254084	0.28498616667868
**Rnf8**	2396.57520657232	0.248986509665405	0.135700867361246	1.83481885198704	0.0665325343820656	0.285033481310007
**Grn**	4118.36788733533	0.195361949973485	0.1064942636687	1.83448331622113	0.0665822821160129	0.285169072927974
**Siah1a**	2459.09034254209	0.393469390405836	0.214557649389176	1.83386326018207	0.0666742944579172	0.285408002655094
**Rbm33**	3007.33188311092	-0.230763659314905	0.125834349385012	-1.8338685775602	0.0666735049481772	0.285408002655094
**Sbk1**	5889.38805516735	0.704096228633671	0.384060059535197	1.83329719181368	0.0667583867843485	0.285690358653172
**Snf8**	2128.33409888933	-0.168462594430957	0.0918984567672897	-1.83313844820645	0.066781984625461	0.285713747418786
**Cept1**	7658.04729503862	0.140611685105616	0.0767328128305572	1.83248443421614	0.0668792787442269	0.285974706966834
**1700096K18Rik**	243.816798677872	-0.475702040314548	0.259587829407514	-1.83252828686266	0.0668727513753228	0.285974706966834
**Nedd4**	3521.10738660235	-0.252438627999633	0.137771328578481	-1.83230161604947	0.0669064964738528	0.286013473938822
**Pnkd**	2033.29192009733	0.144422267216743	0.0788497700789119	1.83161304176546	0.0670090923841528	0.286374360915947
**4933413J09Rik**	24.6143511437553	-1.39638269687441	0.762434266509523	-1.83147945758937	0.0670290110968888	0.286381813409348
**Marchf6**	2598.09842908	-0.246526191210594	0.13462200498806	-1.83124735983882	0.0670636307422788	0.286452054650606
**Ddx51**	4304.54741058715	0.145761250272713	0.0796387786813168	1.83027982957891	0.0672081059283387	0.286991361819196
**Hspa1l**	45.6978203669694	-1.67734278636167	0.916666287910709	-1.8298292502768	0.0672754754658837	0.28720121021835
**Ccni**	6677.64328027865	-0.190454187234887	0.104094333286498	-1.82963069383135	0.0673051807868106	0.28725019871554
**Eif1b**	2096.45109792735	0.248402712689537	0.13578263970313	1.8294143730939	0.0673375560452807	0.287310552560381

**Dnhd1**	409.340910629499	-0.499812649546202	0.273315869870671	-1.82869970112861	0.0674446072771494	0.287652111157269
**Ccdc18**	58.1240135395126	-0.760667154247166	0.4159751046208	-1.82863624721144	0.0674541188504226	0.287652111157269
**Fcer1g**	440.329653715518	0.347512962253367	0.190066875543171	1.82837204673486	0.0674937336645473	0.287743171361545
**Xrn2**	22330.1776247767	-0.0876851012016728	0.0479761046777723	-1.82768279731343	0.0675971713932255	0.28810620222966
**Ptpn18**	5107.74265976071	-0.153824212497836	0.0842046185294634	-1.8267906818438	0.0677312474528602	0.288599585212201
**Aff3**	2925.30569790894	0.468672878435272	0.256627653396675	1.826275821144	0.0678087253883143	0.28879575247958
**Ahdc1**	309.488747068673	-0.37385613222693	0.204713450665192	-1.82624117278141	0.0678139420051055	0.28879575247958
**Phf20l1**	15575.4462075574	-0.117914098506405	0.0645777753661859	-1.8259238234482	0.067861737107641	0.288921208215374
**Tnip2**	1145.55041917199	0.348754967106455	0.191044475805495	1.82551715057978	0.0679230254783748	0.289104028217923
**Ube2r2**	4108.36594471204	0.883868154800948	0.484280663386875	1.82511552003648	0.0679835986098989	0.289283705994107
**Extl3**	4568.4291466897	-0.339154217575113	0.185901275672985	-1.8243781079357	0.0680949293184664	0.289666461217292
**Nanos1**	371.498967785481	0.75860672283307	0.415839822791245	1.82427627479511	0.0681103153398531	0.289666461217292
**Gm6807**	240.622338745581	-0.270283295306297	0.148240228897132	-1.82327899327418	0.0682611462505527	0.290229595647588
**Usp40**	1566.19732917554	-0.182771232357177	0.100264627894821	-1.82288845223568	0.0683202872602921	0.290288500901892
**Ubr3**	1330.97486157712	0.362664615539834	0.198948053323015	1.82291110409111	0.0683168558593602	0.290288500901892
**Gm10288**	75.1636962186935	0.475251414754756	0.260722806542098	1.822822564155	0.0683302690769256	0.290288500901892
**Cox5b-ps**	344.745781176901	0.302377078369846	0.165981537203616	1.82175128308945	0.0684927327475152	0.290821878173699
**Gm7435**	161.160767344424	-0.444632937692869	0.244062398874251	-1.82180024347773	0.0684853008088356	0.290821878173699
**Mrpl4**	7444.76896312696	0.130778198689463	0.0717954552314872	1.8215386791239	0.0685250126412512	0.290880556085182
**Gm9726**	159.449077966722	-0.398428080632101	0.218790723664782	-1.82104649574883	0.0685997893672513	0.291119547821109
**Gm43061**	57.4935105384538	-0.926856980663892	0.509136553411828	-1.82044870762634	0.0686907006529203	0.291426863270885
**Blvra**	2331.14281983189	0.154150481641529	0.0846832772946842	1.82031785455245	0.0687106139185546	0.291432878469627
**Thrap3**	16945.4861573667	-0.217132724156579	0.119294020788756	-1.82014758762365	0.0687365323005294	0.291464353998773
**Ddx39b**	18717.4311144635	0.0926655686625514	0.0509618893404316	1.81833071461566	0.0690136010297831	0.292024664230283
**Jmy**	1293.94492980156	0.554192096407914	0.304804687835013	1.81818757560544	0.0690354683161645	0.292024664230283
**Mrpl39**	2800.25439087295	0.152203892538414	0.0837054036988052	1.81832815819257	0.0690139915235507	0.292024664230283
**Rae1**	11383.4442317686	0.119422125209746	0.0656793562968353	1.81825967766832	0.0690244526029788	0.292024664230283
**Vps8**	2245.64497706876	-0.166408422887896	0.0914909737456003	-1.81885071363009	0.0689342088797855	0.292024664230283
**Plekha6**	681.44289985977	-0.476783458135029	0.262152251957862	-1.81872730283341	0.068953044137457	0.292024664230283
**Plagl2**	1899.87517903011	-0.210653743092518	0.115821045037613	-1.81878641333368	0.0689440220207802	0.292024664230283
**Rad51b**	56.122853883497	0.598435015207031	0.328972585516471	1.8191029938483	0.0688957184088287	0.292024664230283
**Ptar1**	1170.73422095043	-0.230607310622636	0.126810921301847	-1.81851301335256	0.0689857595673008	0.292024664230283
**Washc3**	4054.52949590052	-0.188525870534295	0.103738977799748	-1.81730989193102	0.0691696761692719	0.29235691722082
**Tcerg1**	10088.3329144725	-0.132553109873394	0.0729351132394914	-1.81741144951869	0.0691541358988199	0.29235691722082
**Ccl5**	26477.4721413485	0.701298443298483	0.385894832503344	1.81733048548248	0.0691665247267107	0.29235691722082
**Armc6**	1290.90272441561	0.248697361589926	0.136884306412481	1.81684349439235	0.0692410808320006	0.292580238849513
**B3gnt8**	762.06555278617	0.30152639831927	0.165981341630659	1.81662827494325	0.0692740509691055	0.29264107804831
**Accs**	1157.31036787317	-0.248091386445941	0.13659551473687	-1.81624840994121	0.0693322751336571	0.292808539072912
**Taf6**	6668.51632470689	0.135744885739354	0.0747552260094804	1.81585814110359	0.0693921358064039	0.2929828199093
**Mospd1**	1264.57550892417	0.320036530881823	0.176298840288506	1.81530706814688	0.069476733397106	0.293182883807766
**Rcc1l**	2705.31568181307	0.179241523677031	0.098732909561883	1.81541822754334	0.0694596620226516	0.293182883807766
**Gle1**	9778.50365693822	0.116731800662516	0.0643320527499691	1.81452006694396	0.0695976961722269	0.293614698416541
**Gm12435**	11341.4582827571	0.112718700534422	0.0621270215525174	1.81432648335054	0.0696274766230848	0.293661710157909
**Kdm3b**	3241.77954616331	-0.269115425117632	0.148347828560894	-1.81408401948509	0.0696647914591248	0.293740465352269
**P2rx4**	2703.9387875967	0.182656517527419	0.100747139014031	1.8130194000048	0.0698288292071611	0.29419595235755
**Gm21988**	5080.95514886852	0.124652733325829	0.0687526600908017	1.81306051520334	0.0698224882511291	0.29419595235755
**mt-Tp**	106.314605735997	-0.624177643505628	0.34425602785098	-1.8131204481794	0.0698132459862494	0.29419595235755
**Acot7**	3438.50035303296	0.341484307990073	0.188381642716544	1.81272603352282	0.0698740871203131	0.294307936582404
**Slc25a11**	10514.4052107904	0.100460666598	0.0554313700654358	1.81234319987775	0.0699331834217303	0.29432082563467
**Cdkn1a**	67.4366640363759	0.827661556351653	0.456678417501411	1.81235093368321	0.0699319891832337	0.29432082563467
**Fam110a**	767.781528161836	0.31820957513776	0.175555937525439	1.81258224371733	0.0698962784949887	0.29432082563467
**Dtx2**	2770.84833009766	-0.189439719894578	0.104541761562927	-1.81209611415003	0.0699713467080649	0.294348575348277
**Apoo**	631.539167458579	0.244545291901381	0.13495440381856	1.81205862855843	0.0699771379865865	0.294348575348277
**Dynlt3**	1095.40147345031	0.191082191031217	0.105465743851327	1.81179389679915	0.0700180485108753	0.294442057749096
**Avil**	23.2098683769166	-1.06650731501462	0.588753319511826	-1.81146717932551	0.0700685651038849	0.294531760630931
**Aurka**	404.742590717849	0.36014592399915	0.198831252391864	1.81131446725166	0.0700921874839461	0.294531760630931

**Smad3**	4124.28298964953	0.198484593293527	0.109581694444049	1.81129333964507	0.07009545613797	0.294531760630931
**Diaph2**	1312.55087103601	-0.224711652409734	0.124070773975906	-1.81115701312038	0.0701165502353088	0.294541850721877
**Gon4l**	3627.52443021472	-0.184684396434753	0.101986649033973	-1.81086836545862	0.0701612305025641	0.294650988547149
**Ktn1**	2038.74162245527	-0.148306300308137	0.0819151498896096	-1.81048683311936	0.0702203243959782	0.294742048883172
**Kif1b**	6121.36455873085	-0.28106570931527	0.155241737338341	-1.81050350333752	0.0702177415650562	0.294742048883172
**Atp5j2**	3515.06838698639	-0.194173522635393	0.107278388190096	-1.80999664435039	0.0702963075496518	0.294825370790488
**Pwwp2b**	79.763752217346	0.584659167151578	0.323016683094301	1.80999681363484	0.0702962812975781	0.294825370790488
**4930478L05Rik**	74.5114956046245	-0.785212327378445	0.433763143751888	-1.81023293170244	0.070259672715612	0.294825370790488
**Ric1**	932.926608922844	0.381344713463709	0.210703143411072	1.80986722499779	0.0703163798299206	0.294831079558291
**Acads**	1167.74368389025	0.183476307853725	0.101394127990772	1.80953583298653	0.0703677984746405	0.294968183177683
**Mavs**	5314.61910321765	0.165572413023277	0.0915091288643557	1.80935405109916	0.0703960167745014	0.295007988381866
**Prss41**	53.1919855463492	-0.792869602066147	0.438332611386611	-1.8088309686975	0.070477267506143	0.295269955887874
**Mapk8**	1203.5199423471	-0.177275446866392	0.0980340865420399	-1.80830416357653	0.070559174226546	0.295299046025946
**Strbp**	2583.60004545012	0.285347565994882	0.15778059251219	1.80850864768324	0.0705273721355553	0.295299046025946
**Gm14853**	32.9803854626201	-0.948229613128967	0.524355696409041	-1.80837095815446	0.0705487848043775	0.295299046025946
**Gm47583**	116.041928049964	-0.612254452173476	0.338571788633332	-1.80834456008542	0.0705528906865433	0.295299046025946
**Ncbp3**	3866.84544485153	0.116848239437844	0.064624866257101	1.80810029026566	0.0705908930333754	0.295353346130349
**Ggt5**	2076.84831997073	0.275819346396913	0.152578548359515	1.80772034707665	0.0706500361693767	0.295443901252884
**Wdr43**	16537.7559396439	0.166242026942986	0.0919598595157422	1.80776729997643	0.0706427251351209	0.295443901252884
**Crnkl1**	3918.15671646208	0.10999460906767	0.0608657243736525	1.80716832338045	0.0707360383057005	0.295725061178807
**Cul4a**	4095.58153916059	0.135030975141686	0.074756813832465	1.80626979962387	0.0708762067658556	0.296153908780055
**Tmem229b**	14246.3916909105	-0.148592146772615	0.0822597417585623	-1.80637750126596	0.0708593934545163	0.296153908780055
**Kmt2d**	7214.01094201232	-0.400088195788275	0.221591313654633	-1.8055229205051	0.0709928921417818	0.296562831781033
**Cse1l**	8473.4053845968	0.135645597795729	0.0751605007602063	1.80474579631255	0.0711144698767043	0.296810064385739
**Lars**	6986.78782007908	0.179987943897983	0.0997359088723565	1.80464534722729	0.0711301971534905	0.296810064385739
**Arhgef1**	93855.5033501074	-0.170497339811689	0.0944674171851436	-1.80482694342682	0.0711017667842975	0.296810064385739
**Bbof1**	51.5969939281069	0.810574581082506	0.449103840563009	1.80487118539522	0.0710948417540163	0.296810064385739
**Gm17705**	59.2832671732596	-0.817216002231543	0.452865963123859	-1.80454277595606	0.071146259642377	0.296810064385739
**Desi1**	2333.99980620691	-0.14913323563661	0.0826866427688117	-1.80359524395712	0.0712947822139342	0.297350948476697
**Ttc39c**	299.895202594457	0.705175535166354	0.391204958332505	1.80257310176266	0.0714552845299957	0.297941497311231
**Ppil3**	2314.06792039255	0.169692739602855	0.0941494068763766	1.80237714960511	0.0714860878013421	0.297991080816336
**Pex11g**	1065.00704207392	-0.326272534020636	0.181072789714281	-1.80188605110392	0.0715633352770667	0.298210637499015
**Gm16153**	358.616442322486	-0.410488248026812	0.227820989725632	-1.80180170633605	0.0715766091909168	0.298210637499015
**Casc4**	553.521475918234	-0.376105374065589	0.208758139234827	-1.80163214447183	0.0716033004076859	0.298242982956359
**Ankrd49**	2075.88811906765	-0.169676081412467	0.0942072121666399	-1.80109439086609	0.071688003688608	0.298359184923771
**Angptl1**	28.6527417441793	-1.08687413460584	0.603441766001726	-1.80112513889656	0.0716831582566477	0.298359184923771
**Rfwd3**	4858.74998883553	0.121791821461536	0.0676200553717296	1.80111981263556	0.0716839975768917	0.298359184923771
**Rrp7a**	8079.67141615043	0.151441690985296	0.0841112099339937	1.80049355019551	0.0717827411035347	0.29846910285973
**Rcbtb2**	5804.96494533795	-0.156503720709232	0.0869340606927324	-1.80025779840646	0.0718199412194947	0.29846910285973
**Capn10**	1447.07715368553	-0.197574476247198	0.109736692143941	-1.80044133267696	0.07179097933083	0.29846910285973
**Rbck1**	27152.3893778241	-0.120572367524398	0.0669769715401737	-1.80020632094536	0.0718280661321379	0.29846910285973
**Egr2**	1306.34108393772	-0.257616541227283	0.143085745532056	-1.80043469927315	0.0717920259218027	0.29846910285973
**Acsf2**	441.316841455741	-0.403843234539726	0.224284330486724	-1.80058604033255	0.0717681510667579	0.29846910285973
**Gm5129**	56.6377600913528	-0.655432468321442	0.364142246646893	-1.79993525705083	0.071870861753716	0.298568196323043
**Tubb2b**	179.680756008742	0.41503307122677	0.230601318370864	1.79978620312697	0.0718944033161019	0.298587272812814
**Ska2**	909.508145205524	0.256688505602	0.142640776886715	1.79954506140879	0.0719325025914297	0.298625035609608
**Gm7107**	17.9831915305808	1.46198688255931	0.812445685590706	1.79948876397361	0.0719413997064207	0.298625035609608
**Kif20a**	429.726768055753	0.328429157973321	0.182532790551297	1.799288538686	0.0719730501467983	0.298677732726653
**Slx1b**	769.033421480866	-0.171077857827745	0.0950931273805246	-1.79905596272126	0.0720098287102394	0.298751677458463
**Ascc2**	5419.58678042831	0.154174768852764	0.085730544644246	1.79836450931857	0.0721192631009728	0.2988992001313
**Ebag9**	1534.38858020612	0.221050161757169	0.122913187435351	1.79842510286729	0.0721096676882867	0.2988992001313
**Prkd3**	4717.07205763068	-0.157073071819994	0.0873427909712071	-1.7983518739603	0.0721212641301771	0.2988992001313
**Gm4070**	11997.7032561685	-0.238474272138855	0.132603247354231	-1.79840446517727	0.072112935693579	0.2988992001313
**4930426L09Rik**	20.0932205229433	-1.27846574686356	0.711154901141399	-1.79773175269078	0.0722195270318461	0.2991490766143
**G630030J09Rik**	91.3980020643936	-0.593934993355915	0.330358882795449	-1.79784780820822	0.0722011288236996	0.2991490766143
**Atad1**	5965.65085255397	0.130191116031118	0.0724259377520869	1.797575841914	0.0722442495121147	0.299172835627435

**1810012K16Rik**	28.3107503105152	-0.892053622571901	0.496371334043392	-1.79714975743124	0.0723118482822995	0.299374091798264
**Tec**	4197.07777120374	-0.151491176947478	0.0843138218608028	-1.79675376591968	0.0723747191950652	0.299477009022228
**Rpl19-ps11**	1150.24806947962	0.185055092387507	0.102992676527156	1.79677913641475	0.0723706898224111	0.299477009022228
**Rab37**	9576.6734381687	0.238134151893857	0.132580018988426	1.79615415438012	0.0724700037068402	0.299571406427092
**Elk4**	5439.23063147814	-0.235942287229971	0.131346878397137	-1.7963296129245	0.0724421108954711	0.299571406427092
**Cox17**	2023.1248733915	-0.172716748653287	0.0961604053692372	-1.79613166136403	0.0724735800786999	0.299571406427092
**Nlrp1b**	91.8654470795736	0.639359810569074	0.355951716914216	1.79619813639826	0.0724630110180361	0.299571406427092
**Tagln2**	47465.0481588821	-0.129150434533649	0.0719256926036528	-1.79560918857373	0.0725566935075529	0.299836302019017
**Hfe**	230.915488185027	0.45650307923103	0.254341646827031	1.79484203600161	0.0726788712518079	0.300191254603178
**Smtn**	93.3260766594621	0.92190121810699	0.513663406405652	1.7947574357262	0.0726923551078907	0.300191254603178
**Zfp62**	5900.72677698197	-0.164302211083452	0.091547999750865	-1.79471109724491	0.0726997415476123	0.300191254603178
**Gm12229**	48.0769249887215	-0.56428767073779	0.314486313819955	-1.79431551053394	0.0727628238127241	0.300373019339034
**Gm48138**	37.1437261236612	-1.48178688809834	0.825923383732716	-1.7940972701384	0.072797644704786	0.300438053329836
**Cdc37l1**	5741.55741451513	-0.138363251051366	0.0771369428248946	-1.79373521926398	0.0728554410563522	0.300586019024523
**Stt3a**	19825.2333060646	0.164771008567055	0.0918745396536064	1.79343492972361	0.0729034065605372	0.300586019024523
**Oaz1-ps**	1280.54305121856	0.173096796538282	0.0965190638155097	1.79339489729352	0.072909802926428	0.300586019024523
**Gm45778**	213.870908296545	0.94320545960713	0.525916033385934	1.79345256605816	0.0729005887796786	0.300586019024523
**Rab40c**	2233.11127420532	0.274953058463302	0.15337174585941	1.79272301376383	0.0730172247635539	0.300792787084789
**Mrpl46**	2678.66985407535	0.203305065682158	0.113393418015403	1.79291769522761	0.0729860854555848	0.300792787084789
**Gm16152**	34.117733892004	-0.762335512437356	0.425221604433013	-1.79279581397057	0.0730055790953268	0.300792787084789
**Arpc1b**	86447.2397718467	0.129040109354527	0.0719990677974948	1.79224694571695	0.0730934176623434	0.301027961867628
**Cpq**	700.282108389508	0.24869415658118	0.138803701693017	1.79169686073071	0.0731815376878149	0.301233408920297
**Ccdc62**	258.076899667848	-0.318789818462074	0.177923439610959	-1.79172468315095	0.0731770786318276	0.301233408920297
**Gm30373**	33.7375386914168	-1.34025707439796	0.748204913533211	-1.79129680941139	0.0732456778706117	0.301418685350543
**Ccdc92**	4714.98508786907	0.189960141054624	0.106077236326358	1.79077196610017	0.0733298956745305	0.301686466356025
**Zfp473**	95.2372880489514	-0.649989468976117	0.363180022306975	-1.78971702476167	0.0734994140810458	0.302304951102855
**Pde6d**	1817.60048941358	0.190632827915835	0.106538830173252	1.78932721155122	0.0735621341598469	0.302307472159871
**Rnpepl1**	2126.45566973501	0.699498707824796	0.390948200339881	1.78923629068165	0.0735767694188682	0.302307472159871
**Cish**	939.955407323651	0.627995179102457	0.35092825390591	1.78952584214218	0.0735301694723985	0.302307472159871
**C87436**	4486.96733266043	-0.139972946250767	0.0782296980946896	-1.78925586650409	0.0735736181564568	0.302307472159871
**Bcl2a1d**	354.769540254581	0.431648583862029	0.241265592440213	1.78910129495151	0.0735985036679226	0.302317941161485
**Zfp729a**	1295.75973407787	-0.263528797684841	0.147386001564868	-1.78801782317743	0.0737731323454103	0.30290430840604
**Tpd52**	4158.26116422137	0.166093755221811	0.0929009781699469	1.78785798054753	0.0737989236511257	0.30290430840604
**Lilr4b**	634.633365864623	1.01344192401626	0.56684293002107	1.78787080219664	0.073796854550184	0.30290430840604
**Ncoa2**	6745.28090984322	-0.299332299223478	0.16745987913179	-1.78748665516416	0.0738588670775383	0.302935655385409
**Ttc5**	7660.73156209646	0.093271636105511	0.0521840112543804	1.78736041679206	0.0738792549280865	0.302935655385409
**Srpr**	11808.2955503373	0.15774265569512	0.0882558175567143	1.78733436573462	0.0738834628194534	0.302935655385409
**Klri1**	37.4111080110876	0.728466058969855	0.407555929692466	1.78740144823691	0.0738726277302294	0.302935655385409
**Yju2**	3023.58637664991	-0.111826026549302	0.0625760528306158	-1.78704187130497	0.073930721369561	0.303050566238338
**C2cd3**	4029.86031424326	-0.237003489633642	0.132664954272633	-1.78648152357244	0.0740213262007755	0.303343052521618
**Sucla2**	7645.29670581831	0.175972661671541	0.0985559510439832	1.78551025896963	0.0741785887989964	0.303908482502804
**Zfp809**	3154.48172284124	-0.254854465484037	0.14274506921913	-1.7853819181159	0.0741993895569422	0.303914681894655
**Nisch**	18405.22762148	-0.189342105650653	0.106060763134581	-1.78522292367815	0.0742251650846026	0.303941249022371
**Thap2**	619.994190750779	-0.263098844396789	0.147402308739903	-1.78490314463824	0.0742770285134721	0.304074600750008
**Trdc**	322.301609803978	-0.28165405657395	0.157871011094551	-1.78407710586755	0.0744111368448157	0.304544489159418
**Mrpl22**	1955.53148596484	0.187087961228753	0.104917163075509	1.78319691215921	0.074554254954924	0.304592276291826
**Mctp1**	120.246020146558	0.616881411652375	0.345945890409896	1.78317311681737	0.0745581271598382	0.304592276291826
**Traf3ip3**	35890.5311592132	-0.0944072233599376	0.0529335283010123	-1.7835052071927	0.0745041010999476	0.304592276291826
**Ankrd55**	144.232156287571	-0.509346732166826	0.285566879163707	-1.78363378014449	0.0744831928243771	0.304592276291826
**Gm12247**	26.5305655519939	-0.860641677188646	0.482580933829577	-1.78341417336762	0.0745189077373942	0.304592276291826
**Gm10209**	44.4253217042497	-0.889700058805304	0.498908496267625	-1.78329305967171	0.074538610588036	0.304592276291826
**Tmem223**	3087.45363408496	0.191370525409477	0.107282230731091	1.78380449497883	0.0744554389291694	0.304592276291826
**Itpr2**	20043.357981476	-0.221109773444364	0.124032494800087	-1.78267617530989	0.0746390317734475	0.304843759371232
**Pgd**	5643.21742519198	0.118440426995445	0.0664463169883771	1.78249799783731	0.0746680574367518	0.304883280909795
**Pqlc3**	2517.05819755461	0.238762904480299	0.133991012038399	1.78193224193183	0.0747602819561596	0.305032017210411
**Rdh16f1**	31.0634805218216	1.39330478332186	0.781912841397213	1.78191827727518	0.0747625595269788	0.305032017210411

**Gm47258**	37.936150492637	-0.847129333349036	0.475345941948294	-1.78213225062345	0.0747276676832693	0.305032017210411
**Emc10**	17624.3551527451	0.109798115898832	0.0616329854300888	1.78148300188018	0.0748335793476669	0.305108794533664
**Reck**	2603.63131397795	-0.204342335185387	0.114705822513564	-1.78144692839131	0.0748394675936163	0.305108794533664
**Zfp787**	677.103235707897	-0.204877665737386	0.115006261281726	-1.78144792686987	0.0748393046076885	0.305108794533664
**Pofut2**	2974.1236801154	0.157946051886887	0.088698244019967	1.78071227488259	0.0749594668317485	0.305518965046007
**Srgn**	14381.7380857523	-0.182721922087131	0.10267798877171	-1.77956273075612	0.0751475500910289	0.305968978497246
**Elmsan1**	5055.45444160542	-0.373822903785354	0.210061588082712	-1.77958715440236	0.0751435500007509	0.305968978497246
**Olfr1444**	20.3458074476133	-2.1683882479616	1.21843812272903	-1.77964576740663	0.0751339511069616	0.305968978497246
**Atp8b4**	10185.5557321966	-0.124503875485634	0.069957915509826	-1.77969675880561	0.0751256011973303	0.305968978497246
**Dpy30**	1961.31538128776	0.188625438637431	0.106003888816366	1.7794199886779	0.0751709317813804	0.305985112911189
**Med13l**	3008.67037298332	-0.316064019146798	0.177697462798352	-1.77866365771053	0.0752949207826868	0.306344308466736
**Calm2**	22505.9459976439	0.208699209960218	0.117340412626354	1.77857913815914	0.0753087868477718	0.306344308466736
**Acacb**	174.129940082739	0.699344239665698	0.393215632895702	1.77852603294436	0.0753175002221321	0.306344308466736
**Vcl**	179.73968898067	0.325746932985273	0.183219739101429	1.77790305009081	0.0754197792044598	0.306522945814979
**Galm**	1851.00520063252	0.197064445122794	0.110839334416543	1.77792880262354	0.0754155490051607	0.306522945814979
**Tbkbp1**	72.0387862224178	1.36333309160953	0.766722233595717	1.77813167777315	0.0753822308100723	0.306522945814979
**Slc45a4**	302.904066618143	-0.414080212752119	0.233041866998825	-1.7768490189542	0.0755930842365697	0.307068891032645
**Gm48885**	37.9743962174498	-0.959883072039552	0.540195528884317	-1.77691783940165	0.0755817587890197	0.307068891032645
**Pex2**	3785.88033381132	0.176599586692252	0.0994041649393816	1.77658136155508	0.0756371444868315	0.307168681876032
**Vegfa**	21.9609841522993	1.74286088304921	0.981173307110094	1.77630279015901	0.0756830235932558	0.307246377583425
**Lrrc25**	64.1292114448882	0.833071639904641	0.469011545166325	1.7762284286823	0.0756952743402206	0.307246377583425
**Mgme1**	1108.1878794469	0.178566985231612	0.100550578574039	1.77589217052717	0.0757506916916495	0.307392132110564
**Ighg2b**	222.992565877658	-1.82307303232115	1.02670210908299	-1.77565918701526	0.075789108178338	0.307468840773963
**Pdxdc1**	4609.92329811209	0.127747330079986	0.0719667421244709	1.77508841318729	0.0758832898547351	0.307771685518935
**Dtd2**	890.414840712389	0.23041470872728	0.129861613440767	1.77430961022502	0.0760119518131797	0.308214185465845
**Gm5776**	388.625347491155	0.293566730974967	0.165474501881183	1.77409043470491	0.0760481927257548	0.308281804162521
**Hk2**	123.0524103345	0.614165910314762	0.346252809275463	1.773749970722	0.0761045167722673	0.308430779521763
**Set**	28037.570436404	0.2213215005541	0.124795988833627	1.77346646012122	0.0761514448007239	0.308541608569043
**Rbm25**	17459.6101986723	-0.116767366506368	0.0658730920675923	-1.77261098335195	0.0762931904850879	0.308624319617843
**Tmbim6**	91907.0498802382	0.14621392499541	0.0824949833325906	1.77239777606752	0.0763285507108418	0.308624319617843
**Eif3i**	8018.56288576822	0.108691896584741	0.0613203224206707	1.77252650172142	0.0763072000848921	0.308624319617843
**Zmym3**	967.27480395229	0.288786034996645	0.162890819994552	1.7728809702493	0.0762484325864856	0.308624319617843
**Rbm4b**	4452.46087027319	-0.180670266466362	0.10191204119627	-1.77280588579728	0.0762608777894506	0.308624319617843
**Mynn**	3487.76549015811	0.175226435032614	0.0988276775604118	1.77305021587197	0.0762203863009226	0.308624319617843
**Nudcd1**	2342.4855673559	0.234749771436149	0.132447021261258	1.77240506581944	0.0763273414917597	0.308624319617843
**1700086O06Rik**	39.8012823364318	-0.807346588064932	0.455393468492063	-1.77285500105719	0.0762527367771305	0.308624319617843
**Gm11672**	25.358256857854	-0.844358388511102	0.476428441732673	-1.77226696508786	0.0763502522027742	0.308632869153184
**R3hcc1l**	5650.33356314723	0.140874441328058	0.0795200947976307	1.77155776394089	0.0764679960136452	0.309029549419596
**Zfp661**	685.292909982184	0.217102324753882	0.122560725315109	1.77138576975374	0.0764965733286142	0.309065770753583
**Unc5cl**	2319.99630387847	-0.190506734501219	0.107580927948181	-1.77082256246183	0.0765902127481809	0.309285490075112
**Gm4735**	3226.66352935879	0.178380245151732	0.100726326937231	1.77093963987085	0.0765707396478523	0.309285490075112
**Gm13830**	84.7424493881869	-0.507524408909694	0.286662894352136	-1.77045728243521	0.0766509945266244	0.309451632271591
**Ube2v2**	2025.51355181857	0.262074909761078	0.148037034293553	1.77033342373903	0.0766716132623658	0.30945558662272
**Git2**	15927.3450889191	-0.154514486653615	0.0872960107544363	-1.77000627311899	0.0767260957154103	0.309553433561808
**Gm11998**	165.714882082139	-0.434647785255493	0.24557038818233	-1.76995194116311	0.0767351470135447	0.309553433561808
**Tmem176a**	326.702485847645	0.512692144876403	0.289730202998232	1.76955022145044	0.0768020975329391	0.309664956710983
**1500004A13Rik**	765.170485536165	-0.170018652066313	0.0960772433670883	-1.76960376992408	0.0767931704063844	0.309664956710983
**Etfb**	4047.34462337198	0.194887069772685	0.110146565736144	1.76934313358018	0.0768366293480462	0.309724934417284
**Litaf**	891.595225931306	0.232172476696219	0.131230042149865	1.76920218032909	0.0768601404813041	0.309740468942176
**Lyst**	5398.55581262407	-0.363772920496259	0.205741232693497	-1.76810897715476	0.0770426869626634	0.310158819231141
**Sptbn1**	25212.149006122	-0.290980811025107	0.164547617529944	-1.76836842363977	0.0769993318315761	0.310158819231141
**Prr3**	1929.00303560113	0.201543164069791	0.11397794819274	1.7682645394614	0.0770166891402668	0.310158819231141
**Cd79b**	2789.13159363315	0.135333600547383	0.0765392035109289	1.76816055484637	0.0770340664236091	0.310158819231141
**Spred1**	147.312512466215	-0.609271573846134	0.34463791631124	-1.76785996261626	0.0770843175592714	0.310247149880317
**Ybey**	290.644058226301	-0.346637876879653	0.196102493986909	-1.76763624894435	0.0771217339533534	0.310318478524767
**Gm26596**	239.768116241538	-0.571781186408187	0.323570506699325	-1.76709920888899	0.0772116149927011	0.310600821404134

**Cbfa2t2**	2510.7034446055	0.210722095399543	0.119256209638168	1.76696958622858	0.0772333219101303	0.310608844649802
**Dpp3**	12384.268444837	0.0987421274414556	0.0558894038192944	1.76674146964809	0.0772715349280144	0.310683229359715
**Cx3cr1**	2097.12970002416	1.62235429890991	0.918415381837015	1.76647117523759	0.0773168333174581	0.310786056781425
**Senp5**	1361.04579294278	-0.18080699243612	0.102375752406696	-1.76611148817592	0.0773771464884818	0.310949170420558
**Nolc1**	6690.17412529647	0.104704206748123	0.0592942370040335	1.76584120208849	0.0774224939141522	0.311052074606144
**Fam117b**	1412.20277990664	0.485589101789926	0.275049776942801	1.76545899141343	0.0774866565143911	0.311230498775748
**Mlxip**	11990.9160007971	-0.283553684435797	0.160642564099664	-1.76512175353401	0.0775433053913351	0.311299328165928
**Ttc1**	3853.69955683727	0.168388852467249	0.0953969279030137	1.76513915247295	0.0775403819100898	0.311299328165928
**Eif2ak4**	857.997550989727	0.216810848873399	0.122891375711947	1.76424787839951	0.0776902550343418	0.311809818791677
**Grk5**	211.203514367116	-0.444960025384531	0.25230965133044	-1.76354738329762	0.0778082130354055	0.312137154007627
**Slc12a6**	3282.71500697247	-0.315519553819831	0.178913804753124	-1.76352827695551	0.0778114324390403	0.312137154007627
**Gm15267**	26.4515912946019	-0.763804734939786	0.433196631180035	-1.76318253643656	0.0778697081894087	0.312291420702599
**Insl6**	878.478077300915	0.301163432947886	0.170831231305341	1.7629295922453	0.0779123653088223	0.312382987320894
**Acrbp**	477.004766074619	-0.378675751762154	0.214818414850105	-1.76277137146918	0.0779390577150433	0.312410514478743
**Kctd11**	136.969833732029	0.539437998506433	0.306097189975075	1.76230954145759	0.0780170126668429	0.312643455898129
**Reep5**	6659.54067618432	0.128822360671973	0.0731204720617332	1.76178239882276	0.0781060696867358	0.312644345365931
**Alg9**	2469.3840853278	0.151545765252145	0.0860097446946216	1.76196041262777	0.0780759862625513	0.312644345365931
**Wdr5b**	481.431620500877	-0.346287982807966	0.196542627292315	-1.76189759737432	0.0780866006424048	0.312644345365931
**Togaram1**	4291.54404567667	-0.141455672118968	0.0802927288719089	-1.76174946481932	0.0781116363994981	0.312644345365931
**Hlcs**	3184.0976366779	-0.140431770283208	0.0797128306944748	-1.76172103110298	0.0781164427051895	0.312644345365931
**Stx12**	2091.45597077673	-0.1284522797076	0.0729596419404511	-1.76059361437712	0.0783072102057966	0.313248720795313
**Rpgrip1l**	143.181322496367	0.551767667095263	0.313377896564484	1.76071022603767	0.0782874610582129	0.313248720795313
**Itpkb**	4133.90545268629	-0.331529060515909	0.188434899474208	-1.75938247872861	0.0785125658990118	0.3139904824545
**Chmp1a**	16806.7866669976	0.0962185011364448	0.0546963546056251	1.75913919364837	0.0785538692334235	0.314057770644896
**Pex1**	822.288435948747	-0.216044211167803	0.122829000657687	-1.75890229515014	0.0785941052864251	0.314057770644896
**Acin1**	8203.32966175648	-0.262460888974095	0.149234422911793	-1.75871547497608	0.0786258476069054	0.314057770644896
**Aftph**	5155.40001196679	-0.115231369252964	0.0655208899442764	-1.75869664394005	0.0786290477371399	0.314057770644896
**Ftl2-ps**	1779.37349979975	0.211956977473044	0.120497738678131	1.7590120760624	0.0785754574499816	0.314057770644896
**Rtf1**	3532.4812322496	-0.106686966488628	0.0606725697037288	-1.75840527291976	0.0786785765885696	0.314175958263074
**2310043P16Rik**	214.570290119373	-0.461971202411476	0.26274079277522	-1.7582774168102	0.078700318286793	0.314183155623258
**Arl4c**	34219.172193941	0.759532466909526	0.432031792937654	1.75804762363666	0.0787394064850699	0.314259581556547
**Errfi1**	1289.75928175335	0.365511369087703	0.207938877357174	1.75778273756797	0.0787844836395863	0.314359865461879
**Fut7**	446.040613106311	0.560356157856451	0.318822852282393	1.75757839767434	0.0788192716540227	0.314419054038591
**Selenos**	5587.16562023357	0.17991911814926	0.102391251435116	1.7571727625897	0.0788883663622152	0.314615031326101
**Tiam1**	81.5033135609472	-0.745437284715387	0.42430771896915	-1.75683177889485	0.0789464866522072	0.314767153385331
**Rin3**	3730.93776447638	-0.245223054427581	0.139607879887698	-1.75651298927282	0.0790008554879227	0.314904244857879
**Mthfd1**	8494.20859033399	0.12352447391868	0.0703365943716521	1.75619071440946	0.0790558496775766	0.315043759071718
**Socs3**	7590.09536144964	1.04486670626272	0.595175514726551	1.75556063784441	0.0791634582517985	0.315392822167785
**2300009A05Rik**	938.987577702263	-0.219887395155639	0.125275957883019	-1.75522421757067	0.0792209630598494	0.315542142298799
**Emg1**	15854.500028121	0.111420335244069	0.0635086611305881	1.75441165441929	0.0793599957861297	0.315617227057558
**Slc18a2**	356.989552231827	-0.560871617371644	0.319691554081194	-1.75441487337259	0.0793594446194754	0.315617227057558
**Sec61a1**	19486.8606259798	0.0875832687288824	0.0499085562778715	1.75487482028638	0.0792807220042198	0.315617227057558
**Ripor3**	41.5476142064013	-0.935529533103066	0.533169547703482	-1.75465672623777	0.0793180421610151	0.315617227057558
**BC037032**	102.990121710914	-0.620274342868982	0.353456470237036	-1.7548818457136	0.079279520053673	0.315617227057558
**Gm48768**	38.1861804970956	0.778178795271009	0.443531243454624	1.75450727937416	0.079343623689547	0.315617227057558
**Tcf20**	1281.99706513301	-0.33858263417664	0.193038430904779	-1.75396491045689	0.079436519910239	0.315841848151813
**Ubqln1**	14472.8269352246	0.210928648421897	0.120313660471312	1.75315627166203	0.0795751866729886	0.316176938821995
**Rgcc**	8076.77961900681	-0.208079337667208	0.118685203307593	-1.75320370078428	0.0795670480170437	0.316176938821995
**Snrpa1**	5463.02099901431	0.177249268154166	0.101104900401379	1.75312242483301	0.0795809950731758	0.316176938821995
**Xbp1**	5159.19592949562	0.188418630079269	0.107489405069385	1.75290420444363	0.0796184518003028	0.316246015885397
**Ankrd44**	19823.061846841	-0.250843810816667	0.143146171128634	-1.75236130200963	0.0797117011899513	0.316456859574216
**Gm43852**	169.674118833568	-0.487546063976086	0.278208424565272	-1.75244895886214	0.0796966391665995	0.316456859574216
**Mta2**	28430.0390340971	0.096061882722966	0.0548256154879848	1.75213505344082	0.0797505880369682	0.316515768176629
**Gm13919**	165.194436978304	-0.706957426838969	0.403505011372823	-1.75204125577951	0.0797667141860374	0.316515768176629
**Bspry**	217.78972308642	0.386506573802301	0.220639855881863	1.75175320096859	0.0798162545357319	0.316552912841563
**Rbm12b2**	930.51694043023	-0.220935968742929	0.12611541954567	-1.75185532061701	0.079798688895862	0.316552912841563

**Ctse**	1610.6428035583	-0.204641659267807	0.116844993706946	-1.75139432829326	0.0798780092917285	0.316638438342079
**Ggact**	1118.06320477493	-0.282428569873188	0.161250333654485	-1.75149138282315	0.0798613043331814	0.316638438342079
**Chchd5**	1473.7966052176	0.199936039226425	0.11422044443718	1.75044003909814	0.0800424121713985	0.317210334151088
**Zfp975**	240.386361332538	-0.482038419739457	0.27542101887289	-1.75018748282942	0.0800859680026519	0.317303142523959
**Sh3bp2**	1171.05881950767	-0.168153782583529	0.0960848269516733	-1.75005552820638	0.0801087325436377	0.317313549193087
**Plekhm2**	378.169901781212	-0.446982430342563	0.25549250381425	-1.74949332629943	0.0802057813928418	0.317322477647688
**Usp4**	18107.3796282992	0.121913320457275	0.0696882458485832	1.74941009022046	0.080220157949981	0.317322477647688
**Sp140**	3676.57118822258	-0.179830558096677	0.10277053570594	-1.74982602612126	0.0801483382974124	0.317322477647688
**Zfp951**	1072.2885999611	-0.343747651050418	0.196486206871007	-1.749474716442	0.0802089955105393	0.317322477647688
**Gm26692**	72.8955539108772	-0.594757404198904	0.339989094565282	-1.74934259276573	0.0802318176650625	0.317322477647688
**Gm43768**	17.6168002614647	-1.05707524764602	0.604153411698164	-1.74968017589238	0.0801735163064819	0.317322477647688
**Mef2a**	4659.17510214102	-0.191558219734687	0.109596388183552	-1.74785157530798	0.0804897318673261	0.318046665725525
**Traj37**	25.9498076665537	-1.15376294973897	0.660083330053417	-1.74790499503389	0.0804804797773013	0.318046665725525
**Hm629797**	18.5235215514649	-1.13715419663167	0.650613860649942	-1.74781735436084	0.0804956592570537	0.318046665725525
**Gm49164**	18.0009476254703	1.62255624809176	0.928223881539201	1.74802251952535	0.0804601280304212	0.318046665725525
**Tbc1d24**	51.3054579983956	0.874729274339671	0.500606819991219	1.74733790952951	0.0805787408946855	0.31829511664015
**Synj2**	1724.24186858417	-0.22868258121906	0.130937640354451	-1.74649994149896	0.0807241172292977	0.318384879926603
**Nans**	4552.51416302601	0.129119499008449	0.0739350153044075	1.74639172625898	0.0807429066565149	0.318384879926603
**Zbtb33**	436.253132625135	-0.2608144284879	0.149342068862972	-1.74642303052068	0.0807374709281436	0.318384879926603
**AF357399**	19.2198462602798	-0.814059243871571	0.466096009500445	-1.74654840908007	0.0807157029477721	0.318384879926603
**Gm10814**	42.3824785929897	-0.631280914806698	0.361436056364241	-1.74659086632607	0.0807083326840331	0.318384879926603
**Gm39323**	734.37256607951	-0.213403347948979	0.122153454201976	-1.74701034320426	0.0806355439716992	0.318384879926603
**Gm19121**	39.9602405625655	-0.694047151882385	0.397390826498999	-1.74651025036717	0.0807223274843207	0.318384879926603
**Ino80c**	2911.94634284194	0.173893084643956	0.0995902899774897	1.74608473058227	0.0807962296878434	0.318435765680678
**Gm1043**	3773.82130849287	-0.4365987382498	0.250032589508242	-1.74616732606134	0.0807818806119742	0.318435765680678
**mt-Tm**	49.5184016472908	-0.772190266453026	0.442343939940008	-1.74567841159473	0.0808668484573386	0.318634391383417
**St13**	7673.06915983559	0.155183587990071	0.088913591129669	1.7453303372232	0.0809273840841945	0.318793197753663
**Rap2b**	5059.91193858066	-0.167241536336822	0.0958287522929678	-1.74521249974675	0.0809478862154274	0.31879426220857
**Hmgxb4**	1589.88781814476	-0.203252058967915	0.116471734891854	-1.74507625525316	0.0809715961755571	0.318807956256435
**Adipor2**	3140.19329062923	0.336985239177839	0.193166008743647	1.74453694710366	0.0810655046608993	0.319018270964483
**Nt5dc2**	219.632275634561	0.593323338302393	0.340088609050287	1.74461396975122	0.0810520874753945	0.319018270964483
**Trbv16**	5466.6852691432	-0.159310790494723	0.0913464656916391	-1.74402796307975	0.0811542139542509	0.319207925431136
**Gm43753**	119.065925365176	-0.60286289223055	0.345663023910262	-1.7440768914498	0.0811456829540964	0.319207925431136
**Gm43039**	24.5023269408543	-1.37164855713854	0.786575802050759	-1.74382246893736	0.0811900512309685	0.319269188232186
**Mtmr1**	6174.3463464145	0.183859960910173	0.105451638593357	1.74354769032252	0.0812379914866245	0.319354145229671
**Gm43720**	31.279230179406	1.69327066904649	0.97120927406038	1.74346633034851	0.0812521906597544	0.319354145229671
**Gm5529**	138.390617398727	0.330709921055112	0.189706332335365	1.74327296819212	0.0812859448536129	0.319407140413561
**Dapp1**	3746.1390284625	0.12800615240651	0.0734588715056527	1.7425553889248	0.0814113083129378	0.319719043951353
**Fus**	2433.10973940206	-0.318828903358095	0.182954183905959	-1.7426707416649	0.0813911452323228	0.319719043951353
**Acat1**	7684.56315315601	0.140927880026769	0.0808782130663474	1.74247024858426	0.0814261930175019	0.319719043951353
**Gm45251**	95.5246882234707	-0.535213581540874	0.307199728715222	-1.74223324929113	0.0814676381731338	0.319802086371218
**Col11a2**	236.351483175624	-0.424367727585907	0.24362085234721	-1.74191873765015	0.081522664699246	0.319808237657628
**Cc2d1a**	2109.01636719533	0.140767994092526	0.0808193821841658	1.7417603338238	0.0815503902334423	0.319808237657628
**Vill**	43.5156728037629	0.984263532162315	0.565072884470911	1.74183465392062	0.081537380981525	0.319808237657628
**Gm15710**	2222.00297395283	0.242798969979892	0.139375814484741	1.74204520976251	0.0815005336873404	0.319808237657628
**Hexb**	4015.0988888425	-0.0990569130309996	0.0568917937297887	-1.74114589357961	0.0816580085527496	0.320076373097852
**Zmym2**	5424.51336759198	-0.18036605526673	0.103598849968363	-1.74100441580007	0.0816828044937783	0.320076373097852
**Emc3**	6975.43369668019	0.144553760232562	0.0830321704816327	1.74093678864553	0.0816946592472698	0.320076373097852
**Flad1**	7426.4285465618	0.135792961842634	0.0780013075120877	1.74090622547053	0.0817000173002195	0.320076373097852
**Gm30948**	705.15702314202	-0.539632165395585	0.310066832789777	-1.7403737140807	0.0817934180344032	0.320362636830249
**Rnf180**	20.0125019958344	2.53543382681311	1.45756020097354	1.7395053906656	0.0819459048491145	0.32088012492731
**2810002D19Rik**	737.144003754991	-0.219951927270054	0.126457237476154	-1.7393383855276	0.0819752591471336	0.320915318852518
**Gm4419**	127.000037578034	-0.589552193414317	0.338974928571474	-1.73922064354092	0.0819959596441531	0.320916625959493
**Coro1b**	24426.6040207563	0.0955952405401821	0.054976210601134	1.73884739408013	0.0820616095385128	0.321093812142624
**Nudt3**	4486.10427237667	-0.11011889928741	0.0633506929066664	-1.73824301258467	0.0821680030486049	0.321336296393295
**Sele**	75.2259432696608	-0.509932754545547	0.293368377556196	-1.73819945691954	0.0821756747766955	0.321336296393295

**Trav15-2-dv6-2**	75.8214017601288	-0.71960000453212	0.414031475891669	-1.73803212179067	0.0822051539481736	0.321336296393295
**Gm47643**	21.5554640021568	-1.4258702702943	0.820360876297609	-1.73810125700952	0.0821929734639183	0.321336296393295
**Immp2l**	289.534888030174	0.266195695172313	0.153185792007986	1.73773097154099	0.0822582287263662	0.32146401538724
**6330408M09Rik**	42.5754557033029	-0.8510348922269	0.489947852062064	-1.73699076063935	0.0823888017256346	0.321894457919867
**Fth-ps2**	20.1298107616188	-1.5263092223067	0.878790923495209	-1.73682861474732	0.0824174266413094	0.321926472877321
**Gm45902**	301.149435592101	-0.305661617911972	0.176047428715372	-1.73624585228198	0.0825203729484508	0.322248702986057
**Zmiz1**	2603.44099153916	-0.267341279239066	0.154011550583724	-1.73585213723131	0.0825899825861361	0.322337343600501
**Zswim8**	1644.3889907443	-0.244995041610598	0.141144844594435	-1.73577038760831	0.0826044420548851	0.322337343600501
**Kctd21**	318.904527631737	0.428644876173776	0.246926315173661	1.73592221579265	0.0825775890702855	0.322337343600501
**Anxa3**	108.40426752797	0.557388676333725	0.321142181686984	1.73564454661708	0.0826267041971296	0.322344386737849
**Rnf41**	2901.14148754683	-0.129945323654113	0.0748750246021331	-1.7354962398291	0.0826529469369981	0.322366951704524
**Rnf114**	31610.4372707965	0.107294094493426	0.0618444005211694	1.73490394585844	0.0827578201451774	0.322616272149322
**Slc31a2**	1707.43336425426	0.204204546533297	0.117702043014745	1.73492779991691	0.0827535943963858	0.322616272149322
**Plekha3**	452.481510939648	0.723355277315765	0.417168336682591	1.73396495781065	0.0829243009535505	0.323105393206008
**Rheb**	5326.3349379555	0.146654640546463	0.084572962431332	1.73406058307982	0.0829073343721202	0.323105393206008
**Pou2f1**	3209.67032417446	-0.235097809347924	0.135603149360854	-1.73371938967512	0.0829678844587299	0.323195291821065
**Cobll1**	318.497578165684	0.427284532512344	0.24651286413623	1.73331535459429	0.0830396331151896	0.323394834156214
**Taf5**	942.628307425105	0.895265661150643	0.516724700800914	1.73257763711121	0.0831707668725835	0.323658551042008
**Mtf2**	6866.91485250805	-0.181689312009301	0.104865426359164	-1.73259498690269	0.0831676809166592	0.323658551042008
**Mrpl35**	3138.00333678837	0.181580376395753	0.104812840607773	1.73242491418829	0.0831979352481129	0.323658551042008
**Stk19**	1263.82863034945	0.153989088185827	0.0888899370532795	1.73235681439991	0.0832100520575729	0.323658551042008
**Nme3**	167.624472188443	0.348577553251684	0.201200553823092	1.73248804055567	0.0831867046225312	0.323658551042008
**Cope**	19292.2754068177	0.111824148325754	0.0645577399980825	1.7321571097296	0.0832455932077862	0.323716883557524
**Pgs1**	6930.85974815207	0.138461187330286	0.0799413911255567	1.73203374848478	0.083267553773153	0.323722389539495
**Chmp4c**	82.3677518337653	-0.600855060691603	0.346931913581154	-1.73191060600152	0.0832894800751944	0.323727759631189
**Tufm**	10177.4002153933	0.11350836557507	0.0655462487611392	1.73172939291633	0.0833217547222111	0.323773338879872
**Taldo1**	10736.4943429785	0.119276538664385	0.0689040057207499	1.73105376700133	0.0834421752273053	0.32392861287514
**Saysd1**	2905.34128970135	-0.125917056860994	0.0727331414491808	-1.73121983118209	0.0834125636418345	0.32392861287514
**Zfp1**	1476.79127935774	-0.235838427829501	0.136224307342068	-1.73125070283746	0.0834070597286166	0.32392861287514
**Gm47251**	24.0687211864712	-0.902288642469245	0.521239624027754	-1.73104384409041	0.0834439448918063	0.32392861287514
**Gm11831**	35.137795665391	-0.750885787993242	0.434174264034579	-1.72945715624784	0.0837273080910795	0.324948569850034
**Spry4**	19.7283431593268	-1.57046644390141	0.90815154425558	-1.72929997623771	0.0837554208983321	0.32497763287245
**Dnajc5**	14351.1437047923	-0.139127420833501	0.0804776011003844	-1.72877196799094	0.0838499150434822	0.325024135631033
**Ube2q1**	1476.61528395558	0.345471974968523	0.199816956713701	1.7289422311817	0.0838194347415961	0.325024135631033
**F730043M19Rik**	124.441168234951	0.895858529555865	0.51820159137923	1.72878382556	0.0838477920257347	0.325024135631033
**Gm37868**	48.1511754970732	-0.997256302267714	0.576811380256899	-1.72891232108416	0.0838247885599537	0.325024135631033
**Atn1**	1184.10026753898	-0.268904993158343	0.15563709819138	-1.72776925478064	0.0840296015842551	0.325640539144886
**Gm5124**	217.708113385831	-0.417510270475455	0.241686821704923	-1.72748463292382	0.0840806626715776	0.325758298922067
**Sec24a**	2930.36070774281	-0.159414029173773	0.0922952247799133	-1.72721860262989	0.0841284111421616	0.325783085369142
**Igkv6-20**	44.8803676875337	-1.42355517813865	0.824157103464116	-1.72728618385394	0.0841162792411979	0.325783085369142
**4933417C20Rik**	74.804663300649	-0.887522373886791	0.51393107507707	-1.72692879829012	0.0841804516741209	0.325904515240571
**Arsb**	3479.20446868093	0.195778500581319	0.113379516392558	1.72675371010993	0.084211905103676	0.325946202092513
**Gtpbp8**	1802.05469706425	-0.176202256173449	0.10206464918811	-1.72637889391751	0.0842792703204822	0.32612683262275
**Ppm1f**	4506.52024396704	0.183970736117737	0.106582297981538	1.72609091379887	0.0843310582254828	0.326230891988674
**Pold1**	8422.4534555466	0.12339542762057	0.0715040190101854	1.72571317429014	0.0843990267384966	0.326230891988674
**Fam110b**	95.9243265045015	-0.715421647056314	0.414607641334683	-1.72553898127219	0.0844303850688463	0.326230891988674
**Rpusd3**	902.610708934689	0.199100597696539	0.115368561114812	1.72577863304023	0.0843872452519781	0.326230891988674
**Tuba1a**	15104.5763125536	0.158822868161783	0.0920250796842847	1.72586504360186	0.0843716948257463	0.326230891988674
**Gm18911**	27.6706695739906	-0.975344205675657	0.565227034203608	-1.72557953999828	0.0844230828210547	0.326230891988674
**Kif5c**	493.68128355896	-0.235130589030551	0.136291599900787	-1.7252023543763	0.0844910115553829	0.326262682541321
**Polr3f**	1593.54804846237	-0.207430839658489	0.120227114079924	-1.7253249505814	0.0844689279083176	0.326262682541321
**Tshz1**	1271.91521866719	-0.223597148230792	0.129610384246182	-1.72514840945222	0.0845007303072367	0.326262682541321
**Rbm3**	10878.7109621854	-0.147678372249849	0.085645792608036	-1.72429220108581	0.0846551064958995	0.326778665618787
**Gm43387**	18.4845050757033	-1.44437533959941	0.83792303573006	-1.7237565719159	0.0847517975322547	0.327071779014386
**Cyb5a**	8573.79465025301	0.226781740124734	0.131592051928888	1.72336958654073	0.0848217111688667	0.327101248871227
**Slc37a2**	400.128330724472	0.242671868134609	0.140799840999629	1.72352373704206	0.0847938564091331	0.327101248871227

**8030462N17Rik**	1537.67055997743	0.236414742338388	0.137176955163715	1.72342899764933	0.0848109747960019	0.327101248871227
**2500002B13Rik**	60.0716431661957	0.680886344742541	0.39515286731482	1.72309604981324	0.0848711570559081	0.327211847744053
**Rnf135**	1088.89374284303	0.309650269728734	0.179777655261959	1.72240687685871	0.0849958388252836	0.327214274791828
**Oplah**	332.193186269106	-0.260317272388242	0.151119912496409	-1.72258750079959	0.0849631469180374	0.327214274791828
**Cdc7**	2543.62177837524	0.231697591833542	0.134536090313805	1.72219655925119	0.0850339179358205	0.327214274791828
**Stx11**	823.696341535897	0.413702576100095	0.240137773511527	1.72277176576819	0.0849298064852207	0.327214274791828
**Flot1**	7902.12142433809	0.385623764079867	0.223916780323767	1.722174477153	0.0850379168161793	0.327214274791828
**Timm17a**	4049.43989549883	0.188603044504567	0.109497061853769	1.72244845031955	0.0849883133638987	0.327214274791828
**Gm19261**	871.808819925657	-0.311719857602081	0.1809226674562	-1.72294528919405	0.0848984192745445	0.327214274791828
**Gm31812**	108.379263776681	-0.537185182276085	0.311918103204273	-1.72219943875552	0.0850333964932619	0.327214274791828
**Ptbp3**	8852.23336317862	-0.262483759856013	0.152463699272364	-1.72161479164367	0.0851393218915139	0.327364640079206
**Gm15489**	68.7573700295647	-0.545184121923546	0.31666999457488	-1.72161597645347	0.0851391071217867	0.327364640079206
**Gm12248**	18.40536131665	-0.986246070535119	0.572860738108298	-1.72161575218421	0.0851391477749007	0.327364640079206
**Hnrnpl**	6109.98287775803	-0.238471291103566	0.138531209031307	-1.72142647690076	0.085173463155167	0.327416018281524
**Utp25**	982.3520660324	-0.231329104498727	0.134486925773695	-1.72008619550119	0.0854167746466501	0.327474502620482
**2310011J03Rik**	4185.79668367639	0.173339991804694	0.100761708151434	1.72029628104539	0.0853785991254492	0.327474502620482
**Gtf2h2**	2551.5584140532	0.178238674069883	0.103599564229389	1.72045775863718	0.0853492657345941	0.327474502620482
**St3gal6**	5336.97962941657	0.140522443653536	0.0816505405481225	1.72102282128453	0.0852466827880445	0.327474502620482
**Pcgf6**	2630.82367314199	0.193794865705435	0.112636736936796	1.72052982868439	0.0853361764002711	0.327474502620482
**Spg21**	8903.35962318488	0.156787473114254	0.0911367017394616	1.72035491872936	0.0853679462897042	0.327474502620482
**Tmem109**	6672.9008102557	0.0955839898381145	0.055555655641874	1.72050871749716	0.0853400104379161	0.327474502620482
**Dipk1b**	179.211839897236	-0.438212963649297	0.254778346010232	-1.71997726852226	0.0854365736541167	0.327474502620482
**Zfp236**	2262.68091705948	-0.175532387992492	0.102048967030633	-1.7200800076673	0.0854178992729443	0.327474502620482
**Scd4**	39.1588804742251	0.824023977409384	0.478811988959933	1.72097607497113	0.0852551654515424	0.327474502620482
**Zfp459**	189.059209540381	-0.510441410648216	0.296760428805174	-1.72004540060604	0.0854241892570157	0.327474502620482
**Eme2**	6554.09856933937	-0.141399993895509	0.0822107789022504	-1.71996903305874	0.08543807071605	0.327474502620482
**Hnrnph1**	7587.50934429626	-0.152869409350447	0.0889113677043138	-1.71934605548791	0.0855513785331632	0.327829054364556
**Arsg**	115.162083787299	-0.592759451216493	0.344870588665334	-1.71878806340228	0.0856529698100563	0.328103861181831
**Trpm1**	903.729744330373	-0.337266201166611	0.196230638255532	-1.71872345809436	0.0856647385226916	0.328103861181831
**Snora43.1**	20.3785322024841	-0.850125063175617	0.495128268437058	-1.71697945233294	0.0859829263917528	0.329242520329247
**Phf8**	4947.73931988632	-0.211991065510422	0.123495624737	-1.7165876601854	0.0860545387194426	0.329305596060679
**Eif3b**	31034.6558017083	0.0981006875995223	0.0571500377641605	1.71654633028156	0.086062095867099	0.329305596060679
**Gm50323**	20.1366724491171	-1.87213890104779	1.09057011131738	-1.71666074617275	0.0860411763036162	0.329305596060679
**Rrp15**	3036.17421836382	0.129892137768906	0.0756832180167404	1.71626076655693	0.0861143256711742	0.329340370041426
**Plk2**	81.5231465411137	0.731781086979241	0.426473910537987	1.71588711266327	0.0861827058997204	0.329340370041426
**Usp36**	7197.0605830285	-0.189264073396318	0.110320558210991	-1.71558299255834	0.0862383935261105	0.329340370041426
**9530077C05Rik**	39.3183216485098	-1.02788130784449	0.599103784577963	-1.71569823844224	0.0862172873594957	0.329340370041426
**Tmprss13**	85.9062247195563	1.28556033521629	0.749056943691424	1.71623845963021	0.0861184067016559	0.329340370041426
**Nlrc4**	166.689949405928	-0.492251300674949	0.286880112465855	-1.71587809431557	0.0861843568370875	0.329340370041426
**Mettl8**	2717.49043937032	-0.147684040516747	0.0860827911532379	-1.71560469332193	0.0862344189226725	0.329340370041426
**Basp1**	36.396095639753	0.757358732059608	0.441408322908716	1.71577809650008	0.0862026645778406	0.329340370041426
**Filip1l**	3355.36559973816	-0.231922015149984	0.135212862261856	-1.71523634120576	0.086301904519916	0.329503055372017
**Pdzd4**	71.9570892097858	-0.783253480391556	0.456692519635218	-1.71505651333457	0.0863348661822509	0.329549051946155
**Polr2g**	4798.81224569277	0.172380932383835	0.100558516300839	1.71423504169578	0.0864855676799585	0.330044342439599
**Hbb-bt**	205.822509466072	0.68497090515159	0.399629405655044	1.71401527379807	0.0865259207752184	0.33011838587291
**Enox2**	824.572769389757	-0.168770871678523	0.0984818032503381	-1.71372645614045	0.0865789756913049	0.330226734452617
**Zmat1**	1106.4292622784	-0.166532126403665	0.0971810298060907	-1.71362792446173	0.0865970816664025	0.330226734452617
**Gm9816**	1594.13079923247	0.171035781571528	0.0998155439088573	1.71351850497056	0.0866171919459709	0.330226734452617
**M6pr**	19047.0831269833	0.121823003166875	0.0711097017109528	1.71316993653077	0.0866812807088575	0.33031123098657
**Smim11**	908.943170881642	0.187065937875395	0.109189527739119	1.71322233687412	0.086671643791417	0.33031123098657
**Tspoap1**	168.35476418111	-0.513367708890528	0.299687158712997	-1.71301203259819	0.0867103259633992	0.330342022776906
**Sike1**	8155.03918134794	-0.115017837942598	0.0671706900642402	-1.71232181525303	0.0868373785705928	0.33053395968703
**Mrpl18**	4598.01595382519	0.13313673352098	0.0777527164901374	1.71230973695778	0.0868396032349403	0.33053395968703
**Arhgap35**	714.456717316246	-0.336552283038632	0.196564912889655	-1.7121686576259	0.0868655916141392	0.33053395968703
**Snu13**	9109.03526092968	0.143315526113229	0.0836949472290739	1.71235577365229	0.0868311241231697	0.33053395968703
**Gm44185**	40.2310659070032	-1.12700941030716	0.658223609127097	-1.71219839987468	0.0868601122394734	0.33053395968703

**Bud23**	5820.72224631435	0.198681467942579	0.116067744242507	1.71177159717573	0.0869387682609934	0.330732537780896
**Zfp931**	591.824323524344	0.279931635839589	0.163545920435243	1.71163936767491	0.0869631486693835	0.330745434125869
**Camk1**	138.249625304388	-0.494105137046488	0.288695803773279	-1.71150785909768	0.086987401627819	0.330757839635508
**Chn2**	275.375652416443	0.573499877379907	0.33516193568412	1.71111279748788	0.0870602921680496	0.33087530720983
**Trmt1l**	6736.52453695765	-0.112113216043629	0.0655195712792144	-1.71114086760205	0.0870551114866775	0.33087530720983
**Psen2**	4620.69519490663	0.166103097271591	0.0970901599403717	1.7108128915804	0.0871156589887837	0.330888708685291
**Gm10676**	165.367506337686	-0.29213194120548	0.170755775682415	-1.71081733568304	0.0871148383379556	0.330888708685291
**Traj8**	81.313130977793	-0.403200601809353	0.235686113861771	-1.71075247159376	0.0871268168011214	0.330888708685291
**Gm44027**	42.4106840722217	-1.04372159098714	0.610183082930594	-1.71050561738674	0.087172415568989	0.330982108944713
**Ift46**	1417.98412789891	-0.165625679148735	0.0968435271675543	-1.71024005416673	0.0872214917577614	0.331088664328366
**Gm27201**	71.40136960763	-0.563064102414451	0.329258217906156	-1.71009885795754	0.0872475939509851	0.331107981186337
**Zfp599**	717.969520741747	0.286965862404235	0.16783402422528	1.70981935116474	0.087299283477531	0.331224370275814
**Ergic1**	4150.94155999746	0.169400987730716	0.0990939041008622	1.70949958292381	0.0873584489061456	0.331271021700609
**Irf9**	12208.1948963135	-0.208920193613418	0.122225694138997	-1.70929848331097	0.0873956741263839	0.331271021700609
**Camk2a**	75.8534897820724	0.670795048051062	0.39240071758487	1.70946437656802	0.0873649649730387	0.331271021700609
**Gm43920**	43.4955449518933	-0.941030133626979	0.550499967578916	-1.70940997102253	0.0873750352379136	0.331271021700609
**Ctsd**	70715.8053936725	0.118405984895967	0.0693327069344726	1.70779405754162	0.0876745621089476	0.331874926875194
**Rab1b**	24028.1209700725	0.1473779989571	0.0863253875365829	1.70723819681255	0.0877777881116345	0.331874926875194
**Nhlrc2**	4239.75875807551	-0.160950641394558	0.0942885318015058	-1.70700124733502	0.0878218205554888	0.331874926875194
**Tbx19**	40.7693672271029	0.803729242937781	0.470829386043402	1.70704987148719	0.0878127832516406	0.331874926875194
**Smim27**	320.088308522607	-0.300392944712821	0.176033027431931	-1.70645786813487	0.0879228642657693	0.331874926875194
**Ppig**	4265.51657631624	-0.142995030006248	0.0838085991070166	-1.70620952419996	0.0879690761006101	0.331874926875194
**Commd10**	2316.07967845067	0.160010052663537	0.0936672672856905	1.70828142317312	0.0875841365616938	0.331874926875194
**Spcs3**	8464.90626620446	0.105045999833049	0.0615152766376936	1.70764085889979	0.0877030020514335	0.331874926875194
**AI987944**	2868.77677372203	0.145814128574245	0.0854587936419804	1.70625072459034	0.0879614081777874	0.331874926875194
**Nmral1**	163.337178999339	0.39315368664457	0.230322362096634	1.70697140766392	0.0878273669397963	0.331874926875194
**Dzip3**	988.777994842507	-0.254679952778739	0.149257260858429	-1.70631533309661	0.0879493847887564	0.331874926875194
**Hs3st3b1**	1213.54620146673	-0.286716324832447	0.168009451159109	-1.70654878552588	0.0879059512557598	0.331874926875194
**Tmem185a**	2700.04624126041	0.141033286912912	0.0826024742891049	1.70737363652452	0.0877526272792867	0.331874926875194
**Gm16618**	41.820479636293	-0.809157851923516	0.47409164068055	-1.70675410087802	0.087867766889306	0.331874926875194
**Tmem170b**	623.230144857555	-0.28794743310817	0.168611157534555	-1.70776025334598	0.0876808369124326	0.331874926875194
**Gm13561**	106.609986643387	-0.600468731712919	0.351810091689407	-1.70679791710762	0.0878596197182066	0.331874926875194
**E130317F20Rik**	252.572159115445	-0.453594964995717	0.265853760102122	-1.70618224403325	0.0879741535873036	0.331874926875194
**Trav14d-1**	671.827070839696	0.350305733451431	0.205072689467211	1.70820275660081	0.0875987272194762	0.331874926875194
**Gm42721**	79.3783991491413	-0.804030810496654	0.47124865392061	-1.70617104963043	0.0879762371998276	0.331874926875194
**Trav7-1**	135.546063302327	0.385485581855166	0.225860874040715	1.70673908658335	0.0878705587810345	0.331874926875194
**Dnajc21**	392.992326731526	0.288325898418626	0.169047569820112	1.70559031830769	0.0880843832284569	0.33210740417441
**Pou5f2**	150.749045900749	-0.470014037202798	0.275587137693442	-1.70550063089531	0.0881010947165725	0.33210740417441
**D7Bwg0826e**	189.671708058407	-0.52702706853213	0.308979213180043	-1.7057039634088	0.0880632113670461	0.33210740417441
**Tm6sf2**	20.6948254459255	0.902064297238296	0.529008700974285	1.70519746759732	0.0881576021707834	0.332240932170542
**Map2k3**	6305.95536154954	0.0779726479770317	0.0457538243316317	1.7041777188257	0.0883478904931955	0.332560370401644
**Ighmbp2**	3607.36901242506	0.130406820724576	0.0765204631756998	1.70420846022779	0.0883421492145128	0.332560370401644
**Rap1gds1**	8485.39661858203	0.230722006815266	0.135351904983353	1.70460849327273	0.0882674662743315	0.332560370401644
**Nacad**	76.5499251567728	-0.552078520147128	0.323938480064017	-1.70426965032998	0.0883307222174795	0.332560370401644
**Mrps9**	3795.60787929218	0.100279844398244	0.0588374001122086	1.70435546450049	0.0883146987854694	0.332560370401644
**Ndufb9**	5791.48301256535	0.156159641557341	0.0916763324152143	1.70338011396522	0.0884969567244613	0.333041926703448
**Srrm2**	33536.2062969556	-0.194713327195326	0.114380437417041	-1.7023306746536	0.0886933976591227	0.333701496397994
**Mcm4**	10952.4402953703	0.187756691550098	0.110321799465047	1.70190019071965	0.0887740800375635	0.333925323263759
**Hdac2**	8739.94849838927	0.0771383366938245	0.0453357693722206	1.70148952498181	0.0888511031516479	0.333975866498215
**Aldh7a1**	483.540429481542	0.313671575595966	0.184338333232927	1.70160796235266	0.0888288839047697	0.333975866498215
**Tmem254c**	2668.72244337168	0.258769311595116	0.152074231837358	1.70159867630873	0.0888306258358939	0.333975866498215
**Mob1b**	567.184102664065	-0.274416100891621	0.16135669848544	-1.70067994367388	0.089003103265919	0.334387672427536
**Abcf2**	11921.5885781275	0.131686500121787	0.0774283203645706	1.70075367128903	0.0889892521300147	0.334387672427536
**Lta4h**	15056.1104519118	0.10661672729857	0.0627175619774941	1.69995012460512	0.0891403073845572	0.334823319060421
**Inka1**	40.241885843531	0.901101309792959	0.530121093438885	1.69980278269543	0.0891680279472287	0.334847620677903
**Pdia3**	41166.099946095	0.15366463925131	0.0904396512990654	1.69908482666714	0.0893032018868164	0.335043803510106

**Fam78a**	29526.2904428238	-0.256278035470853	0.150833984095154	-1.69907356759323	0.0893053230146884	0.335043803510106
**Chchd3**	6138.31318404701	0.106189851660882	0.0624893046862839	1.69932842418376	0.0892573198165228	0.335043803510106
**Gm14029**	1662.08650559967	-0.227689134793156	0.133993729430381	-1.69925216471757	0.0892716813945764	0.335043803510106
**Btbd16**	41.3430881732429	-0.665558400625903	0.391768350689486	-1.69885698896954	0.0893461327548371	0.335117118261835
**Eif2b4**	4994.57097548197	0.140353064934551	0.0826386882454594	1.69839415308317	0.0894333949028024	0.33536458912029
**Trit1**	1971.09206807282	0.195696092189392	0.115252358986878	1.69797906012208	0.0895117140636082	0.335378430934837
**Qpctl**	1327.54511127689	0.13045858693643	0.0768244574679395	1.69813873389048	0.0894815805058404	0.335378430934837
**Zfp689**	833.741010820371	-0.251903863732705	0.148339767000119	-1.69815464070739	0.0894785790263949	0.335378430934837
**Gm14027**	29.7329282269445	-0.824772985084833	0.485753947751311	-1.69792338055704	0.0895222238060497	0.335378430934837
**Psmc3**	11902.2445969342	0.145426391510567	0.0856625949494308	1.69766502633286	0.0895710022103047	0.335481407612971
**Rhebl1**	964.012312356574	-0.242440823720482	0.142832864670711	-1.69737423021941	0.0896259313832289	0.335607367111582
**Cstb**	1821.82449830484	0.230465369025901	0.135849079601696	1.69648090146518	0.0897948440449782	0.335819774951189
**Pdcd6**	11723.627078299	0.156743781750367	0.0923766899076747	1.69678932972185	0.0897364967757374	0.335819774951189
**Tnfrsf25**	2546.08472604427	-0.178828027431029	0.10542340887207	-1.69628386469683	0.0898321346868977	0.335819774951189
**Eif3l**	13142.2843748753	0.10462248897741	0.0616763856532036	1.69631355452127	0.0898265148740234	0.335819774951189
**Szt2**	1805.58462673783	-0.305471730182912	0.180087614797746	-1.69623952500001	0.0898405280152408	0.335819774951189
**Arhgap44**	18.3860845401713	-1.29005038015517	0.760580420286963	-1.69613935061389	0.0898594929525988	0.335819774951189
**Zdhhc18**	2882.27852500517	-0.139106168243168	0.0819764699598525	-1.6969036152849	0.0897148844253596	0.335819774951189
**AA414768**	41.8366508004713	0.780796386263586	0.460230891897951	1.69653189303233	0.0897851955500373	0.335819774951189
**Gm43328**	111.560196568928	-0.608666957570149	0.358871067629442	-1.69606026362269	0.0898744679170632	0.335819774951189
**Tmem9**	4097.83424990757	0.149101411578716	0.08791777205869	1.69591890339512	0.0899012391992589	0.335840167392774
**Ager**	94.3127326797999	-0.657448095144451	0.387726104888954	-1.69565083922514	0.089952023734312	0.335950234174343
**Pnrc2**	9878.44957927546	-0.198031849667846	0.116805278122715	-1.69540155077406	0.0899992719446764	0.336047044557409
**Thnsl1**	756.136861106477	0.316175999543352	0.186521545156255	1.69511784431381	0.0900530677795543	0.336168251362814
**Snapc3**	4364.81424967881	-0.0932939384908374	0.0550508221400034	-1.69468746994505	0.0901347238862836	0.336313721116782
**Dock11**	11561.0145317361	-0.240239798278822	0.141754065706054	-1.69476478210503	0.0901200508494746	0.336313721116782
**Usp5**	15082.1697557642	0.142621007862793	0.0841643077441068	1.69455451705749	0.0901599614454892	0.336328246329681
**Fkbp2**	1378.71755113052	0.202333336760599	0.119413346097148	1.69439466670662	0.0901903122992794	0.336361834532484
**Pcm1**	2973.56586849423	-0.168196310953732	0.0992741812583376	-1.69426036882682	0.0902158178498097	0.336377340714494
**Alg5**	4893.46044874976	0.178191455206953	0.105202649478518	1.69379246711215	0.0903047260318302	0.336629185730671
**Gm45220**	189.176382418242	-0.429377559188572	0.253604807469336	-1.6930970807424	0.0904369898701183	0.337042490393438
**Gm43462**	445.493681202231	-0.418431478801432	0.247202315984635	-1.6926681173466	0.090518657237776	0.337267080183409
**Fam193b**	2128.54032137417	-0.264243349095817	0.156121153799501	-1.69255314007718	0.090540557042713	0.337268926080858
**Cyp2r1**	140.02589699003	0.388875467049844	0.229790349035052	1.69230548055143	0.0905877434131881	0.337285225179964
**Gm16580**	196.389735727796	-0.496164394245347	0.2931861906615	-1.69231843125312	0.0905852754363016	0.337285225179964
**Gtf2h4**	1551.6893824684	-0.158282233827957	0.093559499556568	-1.69178153558054	0.0906876354242882	0.337417962545575
**Brcc3**	5587.46224907512	0.141120150259677	0.0834109806024968	1.69186537839902	0.0906716445343611	0.337417962545575
**Gm50012**	64.5202021676056	0.677723898635879	0.400593017808911	1.69180157543126	0.0906838131250325	0.337417962545575
**Lztr1**	6745.55188963548	0.128098101834872	0.0757340462106179	1.6914202824794	0.090756561231177	0.337594696723243
**Bloc1s2**	2245.08056386021	0.149350058457075	0.0883063419109375	1.69127216941795	0.0907848328527961	0.337620158428489
**Mkln1os**	26.4640857178607	0.780932463832287	0.46181687006941	1.69100029566896	0.0908367461816398	0.337733508632397
**Hbp1**	20529.3377917359	-0.128311570017791	0.0759227979742359	-1.69002688838381	0.0910228106466774	0.33821794302431
**Ptpn23**	589.622492159912	-0.36848738831541	0.218042220741237	-1.68998181665336	0.0910314334179157	0.33821794302431
**Gm16044**	19.241583381782	-1.4337651593911	0.848352273265525	-1.69005872274281	0.0910167207416706	0.33821794302431
**Mrps2**	2693.67914396008	0.168977054272291	0.100025760831606	1.68933535588662	0.0911551815270151	0.338497046579019
**Gm48673**	62.6725624222257	-0.538921869065438	0.319029739243293	-1.68925276478521	0.0911710011855908	0.338497046579019
**Gm6566**	88.933900749826	-0.514649647507598	0.304651191475391	-1.68930784421098	0.0911604509211794	0.338497046579019
**Mx1**	612.091989417718	0.266277725711765	0.157647730307518	1.68906793134507	0.0912064125361225	0.338548749665885
**Gm26891**	22.4741404172545	-1.32290778745447	0.783309645050789	-1.68886952409311	0.0912444367329305	0.338610124729342
**Usp24**	4669.63690761463	0.325139008657734	0.192541677962025	1.68866819952541	0.091283033051057	0.338673593544974
**Pnck**	67.4827502441042	-0.632826016025495	0.374776492793731	-1.688542446481	0.0913071480666191	0.338683317345978
**Rwdd2a**	171.681497725963	-0.364461322282488	0.215879928638322	-1.68825941615487	0.0913614420736161	0.338804952401499
**Gm38043**	28.8024040078423	-1.27154852666819	0.753227364501216	-1.68813373835695	0.0913855592977426	0.338814648906948
**Gm11703**	478.584467090624	-0.19541284436726	0.115766051742718	-1.68799783205487	0.0914116451026785	0.338831637704753
**Ecd**	9361.23905295084	0.142861265559717	0.0846528506406752	1.68761316929678	0.0914855095744963	0.33891855840535
**Ttc7b**	518.481715197904	0.33128829534474	0.196314370952554	1.68753970347289	0.0914996222286195	0.33891855840535

**Cd80**	45.7279579486878	0.995191835828861	0.589686966097337	1.68766123900488	0.0914762764159845	0.33891855840535
**Fbxo5**	1052.13089193213	0.324065699134374	0.192049862158273	1.68740396630592	0.0915257017001145	0.338935483358097
**Pcgf2**	17.3746275130207	1.41192076785072	0.836980338219313	1.6869222649299	0.0916183002374553	0.339063306183403
**Ncln**	13532.6215019506	-0.20103594245368	0.119165017818794	-1.68703824438966	0.0915959983667529	0.339063306183403
**Gm23971**	19.7992759345605	1.00938437912938	0.598370500718695	1.68688860483098	0.0916247736072176	0.339063306183403
**Dync1i2**	4851.30861474974	0.121885496982084	0.0722695581935374	1.68653994889073	0.091691847287591	0.339152215425017
**Esf1**	1955.83600634697	-0.175440914659809	0.104018812054463	-1.6866267860082	0.0916751380607695	0.339152215425017
**Tfeb**	2063.97608613209	-0.186681883743111	0.110746426256693	-1.68566959723298	0.0918594558687457	0.339214775280953
**Gsto1**	1629.64634819549	0.240122001922804	0.142415764482363	1.686063356789	0.0917835968782126	0.339214775280953
**Fah**	260.574537998436	-0.341167538243465	0.202387670294811	-1.6857130562672	0.0918510808796521	0.339214775280953
**Gpt2**	59.4878244139164	1.48924637051918	0.883269100911349	1.68606189097138	0.0917838791791129	0.339214775280953
**P3h2**	25.6373349425593	2.2653943403643	1.34346389376186	1.68623388457499	0.091750759799837	0.339214775280953
**6430550D23Rik**	102.292428069191	-0.546066190681382	0.32388902917813	-1.68596692536029	0.0918021700338415	0.339214775280953
**Gm15638**	118.134675208743	-0.713302631030975	0.423154836954306	-1.68567760247061	0.0918579131335594	0.339214775280953
**Rpgr**	359.276872081122	-0.27796486631008	0.164924452310117	-1.68540724202258	0.0919100273659394	0.339322001219566
**Dpy19l4**	530.226966356889	-0.275470087233934	0.163465536322852	-1.68518755347835	0.0919523916574848	0.33939888389482
**Gfra2**	26.611592301767	1.25479117147677	0.744676481620429	1.68501517430271	0.0919856438899395	0.339442105567629
**Cd55**	8830.81251434434	-0.196108866000521	0.116392234065725	-1.68489648450069	0.0920085449673718	0.339447118485338
**Mt1**	65.6279165314369	0.87016835859062	0.516493351660504	1.68476197378546	0.0920345042107589	0.339463408906584
**Kat6b**	1348.77684406766	-0.236395072481091	0.140347690867034	-1.68435313057664	0.0921134430461386	0.339675057823076
**Bend3**	106.526430481368	0.656767479672353	0.389958951939794	1.68419644274188	0.0921437105114371	0.339707170455946
**Zrsr1**	370.997526677935	-0.29129576332895	0.172974608051996	-1.68403771287279	0.0921743805827803	0.339740752010028
**Nipa2**	2735.85965766672	-0.167100427973434	0.0992479390571996	-1.68366647772029	0.0922461433238321	0.339925743768387
**Pcgf1**	1706.83361687757	0.181931987522405	0.108069044723783	1.68347918673113	0.0922823652057302	0.339979712075448
**Mcm9**	1548.55111040223	0.657811895542542	0.390795902972636	1.68326200591873	0.0923243820508478	0.340054999526697
**Impad1**	2367.02906844573	-0.128893762095135	0.0766179270739098	-1.68229247406808	0.0925121397742034	0.340666928352915
**Hars**	7121.97802633722	0.125559900355869	0.0746930344841966	1.6810121750032	0.0927605497416752	0.340820252110848
**Tbl2**	2187.18163671219	0.182365887713279	0.108496905118934	1.68083953651368	0.0927940868499511	0.340820252110848
**Anxa6**	79988.570215641	0.122333861971648	0.0727846242022107	1.68076517963162	0.092808534569654	0.340820252110848
**Tmc4**	323.684210528568	-0.260264466803718	0.154806568569695	-1.68122366646571	0.0927194782375683	0.340820252110848
**Psmc5**	11450.2746459767	0.133241323904879	0.0792595551804462	1.68107584759383	0.092748183029693	0.340820252110848
**Rph3al**	545.826039030848	-0.232415520357332	0.138281622244919	-1.68074048152029	0.0928133338711462	0.340820252110848
**Polh**	488.801593167244	-0.28691069113942	0.170694393712811	-1.68084425562412	0.0927931699764617	0.340820252110848
**Atic**	3755.08807469786	-0.279970153000274	0.166534800790732	-1.68115103672586	0.0927335812393893	0.340820252110848
**Fastkd1**	897.931916027131	-0.202393990769342	0.120347242849306	-1.68175012553277	0.0926173036328339	0.340820252110848
**Ap2m1-ps**	3402.65313190534	0.132329265734601	0.0787157648426794	1.68110245767252	0.0927430151198559	0.340820252110848
**Gm15790**	59.3804236548542	0.508374321699475	0.30230844740664	1.68164113858073	0.0926384482785912	0.340820252110848
**Sirpb1a**	43.9108409403312	1.25421570416488	0.745791284117844	1.68172480809885	0.0926222151428812	0.340820252110848
**Luc7l**	7664.79134103296	-0.166014982726792	0.0987946528525799	-1.68040453540049	0.0928786342257869	0.340946203151448
**Slc35a4**	29723.4647916417	0.101254564675317	0.0602583285576642	1.68034140838177	0.0928909088106344	0.340946203151448
**Dhx29**	2606.42955186446	0.162208780622674	0.0965455605908626	1.68012676740339	0.0929326539123451	0.341019987819474
**Shc4**	35.6105403019288	0.939936377437604	0.559531263628033	1.67986391205918	0.0929837966418017	0.341128215060505
**Lrp8**	132.539576523876	0.560352613209879	0.333653591808153	1.67944427084746	0.0930654913979559	0.341348451572996
**Spib**	585.192840524559	0.412004423955005	0.245353879852894	1.6792252244066	0.0931081577166561	0.341425469197429
**Gvin1**	12623.7884234323	-0.229077275458737	0.136428123529658	-1.67910596094168	0.0931313947005183	0.341431220636591
**Gm49602**	836.706328142616	-0.260839079220473	0.155360660050032	-1.67892617820027	0.0931664318985688	0.341480220382821
**Snx15**	3235.76872983156	0.0892363388562268	0.0531546605818988	1.67880554365943	0.0931899478508326	0.341486978671063
**Pank3**	914.285172865385	-0.280479436306312	0.167101188718488	-1.67850054483353	0.0932494241892627	0.341586392039552
**Cep57l1**	1791.21233560062	-0.172127410554517	0.102557988869166	-1.67834229641634	0.0932802954437553	0.341586392039552
**Gna13**	8329.6597555258	-0.129713910782989	0.077303417896074	-1.67798416051118	0.093350191224359	0.341586392039552
**Brf2**	408.01960348939	0.322249720531387	0.192056670752281	1.6778887151857	0.093368825951282	0.341586392039552
**Atg9a**	2580.65775112534	-0.1674827709309	0.0998158233495419	-1.67791804255722	0.0933630997633072	0.341586392039552
**Slc25a23**	461.486120739438	-0.366250831894408	0.218227026844018	-1.6783018913427	0.0932881790187814	0.341586392039552
**AC169509**	17.3954272291184	-0.841197408757012	0.501281639684127	-1.67809339533576	0.0933288679003614	0.341586392039552
**Rhbdf1**	18.2620271214304	2.41411508926392	1.43923029286712	1.67736539539806	0.0934710518569308	0.341663265076313
**Ndufs4**	3092.51352009971	0.128927894065272	0.0768646266934018	1.67733715249201	0.0934765714123238	0.341663265076313

**Asns**	108.761905394657	0.690036324035324	0.411310663194481	1.67765240676256	0.093414975604813	0.341663265076313
**Tstd1**	173.773496217226	-0.407508933556701	0.242922853013327	-1.6775240719503	0.0934400463106847	0.341663265076313
**Bckdk**	7454.12671248926	0.13000902678575	0.0775298341136832	1.67689029999878	0.0935639352975222	0.341903276549874
**Ccne1**	2396.4912732416	0.278994668083938	0.166452613107652	1.6761206860928	0.0937145556315291	0.342224546032855
**Dennd1c**	21013.2476922875	-0.120870431714739	0.0721824542984116	-1.67451263453921	0.0940298934159437	0.342224546032855
**Rabl6**	4485.99964001413	-0.11623258069945	0.0693484809905624	-1.67606527265202	0.0937254080394941	0.342224546032855
**Slc25a5**	41301.3469401456	0.169254467764334	0.101055114249809	1.674872855479	0.0939591804940579	0.342224546032855
**Lpin1**	9277.52459152957	0.180426827492777	0.107721752065747	1.67493402244939	0.0939471773907293	0.342224546032855
**Ndufs6**	3004.34308316258	0.182683611268548	0.10911999329939	1.67415343187681	0.0941004489313446	0.342224546032855
**Arhgap31**	1245.96460822245	-0.293455828614676	0.175297020876943	-1.67404914896232	0.0941209403996703	0.342224546032855
**Hgs**	7832.49384392377	-0.130900021796087	0.0781638495244923	-1.67468750058261	0.0939955611307412	0.342224546032855
**Asb1**	705.220891659833	0.539111371178774	0.321754580991914	1.67553596134292	0.09382912146371	0.342224546032855
**Mcm10**	111.284601083181	0.546529465234179	0.326216864647648	1.67535625671743	0.0938643537663771	0.342224546032855
**Tnik**	7521.41657570916	-0.211904519465908	0.126578409435821	-1.6740968733167	0.094111562176985	0.342224546032855
**D5Ertd579e**	2855.865312118	-0.164117002970916	0.097948204578534	-1.6755488645974	0.0938265921018966	0.342224546032855
**Myl12b**	47578.5345804934	0.167450070268114	0.100019741687531	1.67417019323285	0.094097155678571	0.342224546032855
**Gm7008**	16.860340394581	1.36994904693978	0.818207048470467	1.67433053711859	0.0940656561587686	0.342224546032855
**Themis2**	1727.07813546682	-0.227724748945918	0.136034434178542	-1.67402283341756	0.0941261119381227	0.342224546032855
**Gm15429**	76.3406749249864	0.416175494489185	0.248506248555388	1.67470837014557	0.0939914643812994	0.342224546032855
**Gm12715**	7108.52470777239	0.128971691572331	0.0769424753393989	1.67620928496812	0.0936972061385618	0.342224546032855
**AI413582**	5724.16405081306	0.154687832577765	0.0924058511261747	1.67400473771459	0.0941296682430915	0.342224546032855
**Gm7964**	7808.15806033494	0.15985197655971	0.0954470701479959	1.67477091032601	0.0939791884387908	0.342224546032855
**Snrpe**	2306.31314932215	0.189327605150765	0.112966769579515	1.67595838896235	0.0937463434508927	0.342224546032855
**5830428M24Rik**	2187.37952796063	-0.208822334363693	0.124616875248734	-1.67571473724475	0.0937940817683992	0.342224546032855
**Gm46440**	17.8006521735867	-1.59200884581154	0.950183827601334	-1.67547457614644	0.0938411552522083	0.342224546032855
**Gps2**	8344.8657503056	-0.105094167858069	0.0627845076080861	-1.67388694857797	0.0941528196917039	0.342229753144678
**Appl2**	4177.87149301643	-0.273761473149529	0.163593271890632	-1.67342745814478	0.0942431761565232	0.34246526121153
**Gm10762**	159.171389872755	-0.482337040117341	0.288248689695633	-1.67333645341684	0.0942610800199533	0.34246526121153
**F8a**	2334.84520740785	-0.151501597550721	0.0905465977428909	-1.67318928957346	0.0942900381465854	0.342491500939545
**Fam102a**	17703.0134621543	-0.152333878195981	0.0910654025377563	-1.67279640731641	0.0943673823852328	0.342693441863128
**4833403J16Rik**	333.790146966303	-0.428381694591993	0.256117565017113	-1.67259787341555	0.094406485833706	0.342756450986568
**Fktn**	607.514237928485	-0.255995942840197	0.153170754664756	-1.67131084129274	0.0946602961626081	0.34351964224648
**Prdm2**	2456.56382503582	-0.317135041845866	0.189741573315086	-1.67140514492957	0.0946416803821469	0.34351964224648
**Slbp**	8450.43284489324	0.197674212607294	0.118283840071025	1.67118528184914	0.0946850864740091	0.343530487582538
**Gm10605**	91.3308161335769	-0.492365899738995	0.294687041556486	-1.67080947006832	0.0947593174090655	0.343720663999688
**Sf3b3**	31050.6442226448	0.093965151388978	0.0562544197241054	1.67036033523804	0.0948480923875748	0.343963496375378
**Gm49405**	886.008363959217	-0.192342076972727	0.115180590856858	-1.66991743610486	0.094935700076485	0.343964549575805
**Itgb1bp1**	5051.75316708412	0.158142058619627	0.0946820666282356	1.67024299586281	0.0948712963997061	0.343964549575805
**Gm3788**	134.84863897676	-0.367988205268096	0.220354974164612	-1.66997911739082	0.0949234953218257	0.343964549575805
**Gm6201**	36.356726290315	0.595259706337476	0.356458536369844	1.66992692165426	0.0949338231076948	0.343964549575805
**Ing4**	3918.89433950277	-0.126034471555436	0.0754868247345485	-1.66962210953553	0.094994153184796	0.344097211892605
**Eif6**	13558.7462733935	0.143268898295715	0.0858149066508531	1.66951062335381	0.0950162268079826	0.344098066148789
**Fam221b**	1521.34251182265	0.157788836538606	0.0945328797017403	1.66914238766917	0.0950891645586661	0.344283080411512
**Pam16**	771.94226231047	0.219199962475837	0.131368420541268	1.66858946444422	0.0951987682538019	0.344501354700315
**Morn3**	37.908948023227	-0.677331943941483	0.405950814050857	-1.66850741640988	0.09521504091641	0.344501354700315
**Mrpl17**	2757.15427057584	0.197962361635997	0.118636854574366	1.66864135387126	0.0951884781256151	0.344501354700315
**Rrs1**	2232.91492208447	0.233659056555794	0.140116064525742	1.66761075788613	0.0953930212841345	0.345066078139143
**Clpx**	4642.05611563266	0.138629563147015	0.0831735015060368	1.66675155712848	0.0955638164198736	0.3453228111267
**Rbfa**	4125.29048698857	0.106622641602865	0.0639618914537632	1.66697136653535	0.0955200985944698	0.3453228111267
**Dnah10**	17.0150672404878	1.52417096999231	0.914482911715462	1.66670251621558	0.0955735723375984	0.3453228111267
**Kif13b**	1069.89633473876	-0.249010606841845	0.149391961873463	-1.66682734277739	0.0955487416261558	0.3453228111267
**Nutf2-ps1**	1069.62931666041	0.260889665035083	0.156502993306271	1.66699472977191	0.0955154528292192	0.3453228111267
**Senp2**	4804.30570941976	0.222058607129455	0.133333453469999	1.66543805286961	0.0958253928919273	0.346006770373928
**Tctn1**	973.236837306138	-0.218183785341033	0.131006808740398	-1.66543851757648	0.0958253002470992	0.346006770373928
**Gm15829**	32.4307349247791	1.00841105325386	0.60549909644257	1.66542123543781	0.0958287456947275	0.346006770373928
**Gja5**	46.1769462882379	-0.749427572080014	0.450044645975077	-1.66522939175576	0.0958669992052515	0.346065591403699

**Rftn2**	860.903051970409	-0.180783907912073	0.108583034388112	-1.66493696672623	0.0959253320891887	0.346196852434573
**Nelfa**	5875.73018887805	0.0987169397941731	0.0593003889973833	1.66469295502478	0.0959740292342632	0.34628658823748
**Nupl2**	733.038459606995	0.330946954730001	0.198815638968055	1.66459216411631	0.0959941497379775	0.34628658823748
**Slc9a3r2**	22.8850743820937	1.35527466122266	0.814403057199799	1.66413258059537	0.0960859374377999	0.346423454328792
**Cox7a2**	2449.4728678952	0.154041820327492	0.0925611617979278	1.66421658215336	0.0960691554614282	0.346423454328792
**Tnfrsf18**	9281.36824291498	0.159146556789124	0.0956368228349999	1.66407197637354	0.0960980465108301	0.346423454328792
**Bag5**	3778.54620479731	0.228985903814976	0.137626794608743	1.66381775050386	0.0961488556132684	0.346527337122203
**Sec63**	4391.65464120351	0.242365230341989	0.145807712975165	1.66222503183539	0.0964676627872355	0.347199854394351
**Smap2**	36111.2162944145	-0.0873545785132282	0.0525390287049254	-1.66266070512718	0.0963803721553617	0.347199854394351
**Rpl27a**	6889.01393740193	-0.134654270496626	0.0810043032666301	-1.6623100880629	0.0964506161242478	0.347199854394351
**Sco1**	657.718001526544	0.266294602900306	0.160153441759411	1.66274667578075	0.0963631547146104	0.347199854394351
**Igtp**	28331.7290972138	-0.233586511039108	0.140522473067689	-1.66227156368498	0.0964583367428155	0.347199854394351
**Gm7390**	44.0068238304291	0.68035579738876	0.409268691140453	1.66236951938079	0.0964387065437432	0.347199854394351
**Fam50a**	3248.75518033854	0.114570092165035	0.0689387299840546	1.6619118482678	0.0965304506885962	0.347336867274685
**Setbp1**	59.848588150576	0.711823537203956	0.428340951152782	1.66181527889931	0.096549817773886	0.347336867274685
**Ncs1**	34.8930714031337	1.19877378622075	0.721495810882137	1.6615117761461	0.0966107057973752	0.347476578691906
**Bcap31**	16020.0440020109	0.17503435888964	0.105357491383675	1.66133757164193	0.0966456681763342	0.347523001701163
**1600020E01Rik**	2782.97629506831	-0.195371885898351	0.117613466453341	-1.66113534265958	0.0966862676863695	0.347589669161334
**Gm49673**	367.270195314833	1.18541715510831	0.71371841333181	1.66090314186305	0.0967329011640953	0.347677993531626
**Lag3**	1090.57966810423	0.267050223406705	0.160837273207577	1.66037522323603	0.0968389913536056	0.347880727602076
**Cpeb2**	166.291138135127	-0.4479901502876	0.269825972558948	-1.66029291412905	0.0968555405210576	0.347880727602076
**Ankrd16**	3484.16019032531	-0.229355691042001	0.13814122170613	-1.66029870164253	0.0968543768027554	0.347880727602076
**Cd72**	10703.6812658832	0.0984870472648865	0.0593627112291431	1.65907259331066	0.0971011651332532	0.348604023468824
**Cyb561d2**	2842.4773060369	0.203555881105696	0.122689236926985	1.65911767164088	0.0970920829740115	0.348604023468824
**Stim1**	15963.8077501758	-0.136233122218971	0.0821270732543049	-1.65880892646847	0.0971543010328366	0.348628293700921
**Atp6v1g1**	6891.29374562143	0.140626842627875	0.0847920823217321	1.65849025967172	0.0972185519148218	0.348628293700921
**Gm9795**	22.9931481842007	-0.842429046215834	0.507924767956814	-1.65857051941885	0.0972023664220098	0.348628293700921
**Zbtb44**	2922.85001304151	-0.216731974848184	0.130667500388891	-1.65865248974036	0.0971858381918927	0.348628293700921
**Zfp947**	23.3593818058285	1.11025099305086	0.669363816617679	1.65866598326303	0.0971831176170858	0.348628293700921
**Tmed10**	11947.4227501192	0.122700205598942	0.0740092114499497	1.65790451208794	0.0973367413050148	0.348691977421936
**Pias3**	1771.36803566193	0.160435456304575	0.0967869271859894	1.65761493797893	0.0973952126178119	0.348691977421936
**Abcd3**	1869.79410310543	0.18039792355458	0.10883570722407	1.65752516481726	0.0974133454725748	0.348691977421936
**Lsm4**	11065.3504055611	0.156277999798732	0.0942703806119408	1.6577635391337	0.0973652033054258	0.348691977421936
**Atg101**	3903.22324189463	0.284949773589248	0.17185930568501	1.65804099145795	0.097309192881987	0.348691977421936
**Sbf2**	365.052805607259	-0.310482133353864	0.187238795405553	-1.65821475555517	0.0972741275397022	0.348691977421936
**Slc25a51**	3837.37350071218	-0.142016605678664	0.0856528542169268	-1.65804872443584	0.0973076321634773	0.348691977421936
**Gm13205**	261.693720142563	0.253447267368379	0.152906774196734	1.65752805066888	0.0974127625311306	0.348691977421936
**Tmed4**	4539.97058683373	0.128890165410951	0.077773490363871	1.65725062367556	0.0974688154798514	0.34881129355349
**Gm45809**	43.6493816047852	-0.754108073624031	0.455071512675446	-1.65711993086646	0.0974952303397776	0.348826599560371
**Akap1**	430.46926631537	0.284607054753296	0.17176733812492	1.6569334884046	0.0975329228937521	0.348882239735948
**Dnajc3**	18429.79924771	0.0948667594949112	0.057258724353029	1.65680881938636	0.0975581333795136	0.348893215538129
**A630052C17Rik**	643.825855114436	-0.260123714663957	0.157015890061826	-1.65667127423557	0.0975859537106718	0.34891351772613
**Zc3hc1**	3737.65859798997	0.114968820672478	0.0694074854371509	1.6564325871823	0.0976342463809593	0.349006991884023
**Tnrc18**	1892.3114034146	-0.362396440143755	0.218867669228264	-1.6557787699827	0.0977666286176007	0.349400945141196
**Polk**	1042.08355709408	0.132126995643102	0.0798457338588874	1.65477839901392	0.0979694572207052	0.350029897588231
**Ptbp2**	793.747000357511	-0.222356874925667	0.13437963761574	-1.65469172912566	0.0979870456471211	0.350029897588231
**Iqsec1**	2528.68550703341	-0.368719024257046	0.22287496757044	-1.65437612073017	0.0980511152213678	0.350164260665542
**Gstp1**	12336.7297352601	0.139050196224078	0.084054430308388	1.65428753385058	0.098069104672021	0.350164260665542
**5730521K06Rik**	26.9044508526809	-1.17591538398479	0.710990428814348	-1.65391169322175	0.0981454564322297	0.350357489126108
**Hbs1l**	5525.77651572349	0.141312176472837	0.085472129487709	1.65331292574333	0.0982671936998021	0.350530685939326
**Coro2a**	2450.49913015983	0.358190654942231	0.216660403044828	1.65323543161747	0.0982829580808493	0.350530685939326
**Dhx32**	3515.40615762918	0.170349836847849	0.103027563903519	1.6534394330373	0.0982414630670089	0.350530685939326
**Mterf1b**	440.987783818061	0.31210690242854	0.188783436076117	1.65325363769043	0.0982792542964054	0.350530685939326
**Cysltr2**	710.843910499854	0.577271381648003	0.349233673518637	1.65296598071948	0.0983377873334031	0.35064686920399
**Cdc37**	20628.3698616273	0.0828098304126084	0.0501061552190953	1.65268777958541	0.0983944227577352	0.35076943877684
**Psmc3ip**	525.996281019906	0.327079763156748	0.1979670262942	1.65219314185521	0.0984951840788924	0.35084388184722

**Evl**	15302.7394943142	-0.192969425444179	0.116795966470077	-1.65219254805018	0.0984953050908176	0.35084388184722
**Ttll1**	1309.33961166703	0.175433626298065	0.106185171203058	1.65214807595482	0.0985043684262298	0.35084388184722
**Trav14d-2**	315.247805057104	0.328387656963693	0.198759470619855	1.6521862125089	0.0984965962223937	0.35084388184722
**Ttl**	435.558112249621	-0.352776146647136	0.213551007802592	-1.6519526190822	0.0985442100914456	0.350906467437493
**Cd84**	11190.7075813834	-0.104368448801569	0.063200957442467	-1.65137448901114	0.0986621306662928	0.351246993427197
**Prkd2**	25436.3061672475	-0.184956829017449	0.112044096317952	-1.65075033041089	0.0987895660750364	0.351541823090413
**Trbv5**	1222.4316940709	0.22830323326491	0.138302220012422	1.65075609953625	0.098788387582578	0.351541823090413
**Cask**	167.093192829353	0.371116985714555	0.22495853128416	1.64971287639575	0.0990016753268272	0.35185418784713
**Nrg4**	76.2040798628992	-0.623124812422796	0.377725670598604	-1.64967557390339	0.0990093086498623	0.35185418784713
**Resf1**	20589.6070767347	-0.213938709751689	0.129714969737724	-1.64929853650862	0.0990864893368979	0.35185418784713
**Chst12**	4834.50616992034	0.168905930835863	0.102381442211403	1.64977096617858	0.0989897891722985	0.35185418784713
**Lyn**	554.037701292275	0.395615634080414	0.239782471328482	1.64989388877577	0.0989646408799591	0.35185418784713
**Fgfbp3**	177.699788993447	-0.49151307496577	0.297999659952261	-1.6493746168855	0.0990709115894838	0.35185418784713
**Zfp644**	3389.05763795555	-0.185515785614955	0.112465230604443	-1.64953901412821	0.0990372573018395	0.35185418784713
**Mdc1**	892.642095087231	-0.400287884853356	0.242713135072253	-1.64922217635315	0.0991021263356505	0.35185418784713
**Lpar5**	2370.44500630538	0.199849063956758	0.121185249488939	1.64912037396926	0.0991229764456059	0.35185418784713
**Gm15925**	47.5955498718829	0.831921307348796	0.504209781144388	1.64995075157132	0.0989530092502463	0.35185418784713
**B020010K11Rik**	830.335628655404	0.166047467993487	0.100655741823102	1.64965718781655	0.0990130712238412	0.35185418784713
**Cnot6**	4298.61307121239	0.334338149575845	0.202770323289605	1.64885148946736	0.0991780634193738	0.351891183359395
**Abcb10**	461.82293889705	-0.399523735055412	0.242301428982955	-1.64887073399603	0.0991741199378851	0.351891183359395
**Gm38253**	50.0618777082966	-0.798847151757802	0.484534293785419	-1.64869063346748	0.0992110300282422	0.351928905933141
**Hyls1**	614.176401159415	-0.270638715252894	0.164202783738643	-1.64819809439809	0.0993120277581478	0.352128627674423
**Sh2d1b1**	21.8803441009841	-0.820844181710721	0.498019596244645	-1.64821663223769	0.0993082249913063	0.352128627674423
**Usp12**	1567.42300662114	0.363455234946384	0.220580709921231	1.64771994376196	0.0994101535278923	0.352159572999989
**Gas7**	900.035306231097	0.259428090346291	0.157454524633083	1.64763820506803	0.0994269356302293	0.352159572999989
**Rbm8a**	4407.85203918745	0.144815857851025	0.0878859147698895	1.64777095658838	0.0993996810232963	0.352159572999989
**Ptafr**	84.1804280150616	0.570636108163776	0.346341506327755	1.64761109407361	0.099432502397471	0.352159572999989
**Gm37529**	64.3619728840051	-0.63629581461814	0.386162782705205	-1.64773987322307	0.0994060620722232	0.352159572999989
**Mrpl52**	614.80816887409	0.212364857488164	0.128920579792782	1.64725335419299	0.0995059812997961	0.352340617379975
**Gm46620**	492.977052339108	-0.192438907109698	0.11684405460708	-1.64697217806097	0.0995637645935281	0.352466016333458
**Gpr174**	3885.47804284828	-0.175289546797564	0.106471175184529	-1.64635683314064	0.0996903148652344	0.35283474648057
**Ftl1**	104006.142562496	0.162554369493165	0.0987620559066943	1.64591925513113	0.0997803840800415	0.352994951043829
**Rap1a**	5474.31438127599	-0.118142579657615	0.0717783817284111	-1.6459354030108	0.099777059115492	0.352994951043829
**Llgl1**	7102.19100390877	0.18532617918245	0.112624569843337	1.64552174929718	0.0998622612570889	0.353136651877835
**Slc12a2**	256.055700038746	0.335300960697612	0.203799160375184	1.64525192390558	0.0999178696857023	0.353136651877835
**Clnk**	20.8970267471088	1.75980777674541	1.06960514732614	1.64528731106491	0.0999105753244827	0.353136651877835
**Gm13288**	40.5106112948167	-0.582790187027347	0.354240776843137	-1.64518097611731	0.0999324954447717	0.353136651877835
**Gm49502**	119.455061046671	-0.366415527151781	0.222694122347878	-1.64537583340161	0.0998923300599467	0.353136651877835
**Klhl8**	958.382132950479	0.324234697465924	0.197115206376437	1.64489946476642	0.0999905453316354	0.353233867225957
**H1f1**	17.1268208517316	-1.60484330093859	0.975689370387317	-1.64483015767766	0.100004841130608	0.353233867225957
**Cdkn2c**	280.087691236694	0.391716187151827	0.238191041209709	1.64454626489058	0.100063415994077	0.353282376387783
**Emid1**	472.222468327329	-0.379772243843021	0.230926967832837	-1.644555624694	0.100061484374076	0.353282376387783
**Smad5**	1533.18715015565	-0.246663955305334	0.150030148591672	-1.64409592085832	0.10015639042698	0.353376232852272
**Rras**	1093.40771605169	0.196991192077424	0.119825562449013	1.64398303710234	0.100179706298858	0.353376232852272
**Ube2s**	6285.67460134414	0.178908245187012	0.10881524354724	1.64414689849352	0.100145862535637	0.353376232852272
**Tia1**	4755.79007030522	-0.129065282300281	0.0785034499801563	-1.64407146861578	0.100161440612508	0.353376232852272
**Gm43421**	20.5937903913083	-1.58873324841239	0.96648368221104	-1.64382832080291	0.100211669613315	0.353409865285811
**Gm45606**	2299.40987900747	-0.201835803391486	0.122815974230355	-1.64340025518928	0.100300147369971	0.353642743814865
**Adprhl2**	7392.20245573699	0.100400397358081	0.0611275183668775	1.642474617659	0.100491682209178	0.354238800127519
**Ifi211**	181.174588029025	0.42422268120744	0.258311299020095	1.6422923922288	0.10052942299679	0.354292578429977
**Naa60**	9973.75945881814	0.106176247762691	0.0646696734744445	1.64182439864412	0.100626401114623	0.354308801283694
**Atp6v0a1**	98.4836913924326	-0.717390388627384	0.436891851322274	-1.64203197302986	0.100583378137373	0.354308801283694
**Ccnk**	237.516099026239	-0.32230287938286	0.196319332261729	-1.64172766721299	0.100646455197424	0.354308801283694
**Mpv17**	3931.24089842755	0.170789062337284	0.10402980081921	1.64173209015456	0.100645538176383	0.354308801283694
**Gm45797**	52.0498860195148	-0.959419972926432	0.584378982577682	-1.64177700008041	0.100636227250932	0.354308801283694
**Zfp790**	2057.07170933963	-0.17528247747261	0.106800410284011	-1.64121539427131	0.100752711257798	0.354603634417943

**BC055308**	37.9409956388677	-0.707599892681067	0.431281489479267	-1.64069154355646	0.100861461214469	0.35490711128995
**Srebf2**	23881.9578581461	-0.241737989327617	0.147371318183262	-1.6403326801149	0.10093601423762	0.355090148770302
**H2-T10**	4245.93149718012	-0.115936471111669	0.0706971512409733	-1.63990300990341	0.101025334945487	0.355325045253578
**Nucb1**	17418.5642096754	0.0859958358363383	0.0524649785521887	1.63910932987031	0.10119049232758	0.355822177591651
**Rbm15b**	1057.5840695246	0.380586101276542	0.232205320921462	1.63900680555579	0.101211842353607	0.355822177591651
**Ganab**	18120.359924521	0.131058188776038	0.079972659581229	1.63878742388105	0.1012575392231	0.35590342305117
**Lsg1**	6068.15339191675	0.117313209587937	0.0715913932399493	1.63864962363205	0.101286251207606	0.355908454447035
**Fbxo21**	4986.06353038578	0.186939443100178	0.114087378141808	1.63856375827847	0.101304145344606	0.355908454447035
**Abca2**	6447.21998270961	0.369236509676942	0.225369356300215	1.63836164658109	0.101346274883812	0.355977096153415
**Kifap3**	341.34229048227	-0.305974946133789	0.186774648382167	-1.63820383967594	0.10137917893025	0.356013310096714
**Cystm1**	68.879490673113	0.706930742966701	0.431643740041556	1.63776438156764	0.101470854422498	0.356255849629077
**Neurl3**	12705.987111581	-0.249517914240458	0.152389305241054	-1.63737155862587	0.101552857207515	0.356384937866105
**Hspd1-ps3**	282.56270954669	0.285548656523441	0.174393756387328	1.63737889726533	0.101551324764362	0.356384937866105
**Ddb1**	22685.428748695	0.113753365460301	0.0694836672325967	1.63712380176353	0.101604604211682	0.356434519940546
**Hsd17b12**	7696.83017830434	0.140881657634422	0.086061979819995	1.63697904613729	0.101634847883028	0.356434519940546
**Catsperz**	17.2057764357898	-0.983084671616231	0.600530943070444	-1.63702584015044	0.101625070465246	0.356434519940546
**Ccsap**	1603.12309017562	0.177308303472092	0.108358642539228	1.63630975173857	0.101774776198007	0.356607771525907
**Gpc1**	1104.24198464126	0.308657656720748	0.188623903257163	1.63636554747749	0.101763105214739	0.356607771525907
**2600006K01Rik**	28.8239745914012	0.941853813047521	0.575582813267899	1.63634804816374	0.101766765490643	0.356607771525907
**Serpina3g**	2691.74250317956	0.371084268688526	0.226739345442014	1.63661171361821	0.101711626531236	0.356607771525907
**Prr14l**	8643.1818127671	-0.183161713605785	0.111950181460006	-1.63610019400655	0.101818619614286	0.35668207853764
**Ift52**	3429.46171309557	0.107533303883987	0.0657318473538214	1.63593917123852	0.101852318818375	0.356691216793831
**Clasp1**	3668.25239785445	-0.236170015100308	0.144369547222716	-1.63587141224439	0.10186650222582	0.356691216793831
**Vps50**	2225.92302123364	0.124292280360479	0.0759919616503542	1.6355977350915	0.101923804710898	0.356733316488144
**Zfp866**	2965.74524161122	-0.163152776787485	0.0997488295447532	-1.63563600226793	0.10191579079199	0.356733316488144
**Sephs1**	2928.72989660601	0.138844453775692	0.0849291346759447	1.63482713329727	0.102085291057326	0.357219171927668
**Hrh2**	1962.43095284612	0.248209504083238	0.151896844813189	1.63406622690878	0.102244945389099	0.357558694604753
**Gm37052**	131.183044155294	-0.494425062513147	0.302578260555983	-1.63404026979548	0.10225039524586	0.357558694604753
**Gm4430.1**	45.252793646026	-0.554404225303032	0.339279149615055	-1.63406512287023	0.102245177184127	0.357558694604753
**Pds5b**	3841.75338989957	-0.2490537163038	0.152466618095429	-1.63349669202945	0.102364575882171	0.357799161973242
**Gcnt7**	158.887015237837	-0.339813531020311	0.208024176938634	-1.63352902542936	0.102357781288372	0.357799161973242
**Mtcp1**	553.460463755235	-0.270887924451388	0.165855103727134	-1.6332806067702	0.102409993607708	0.357822324153207
**Gm15922**	64.6660663894754	-0.598652400473618	0.366540783928676	-1.633249086383	0.102416620037505	0.357822324153207
**C230037L18Rik**	163.082577806993	-0.449681599298952	0.275359458430064	-1.63307119306077	0.102454024375463	0.357836060378022
**Gm9299**	97.8226964798553	0.380653574896412	0.233098727136133	1.63301438653547	0.102465970960566	0.357836060378022
**Traj30**	23.5745822632185	1.33026395292926	0.814702108968531	1.63282252284024	0.102506328672776	0.357897678018377
**Tomm22**	5407.42746157318	0.119526071768338	0.0732360392035225	1.63206630326055	0.102665519410169	0.358294704174092
**Gm13387**	29.3535930055579	0.924255365508896	0.566273265442922	1.63217199523974	0.102643258523073	0.358294704174092
**Stk4**	28752.1069934815	-0.156242225096282	0.0957525747384951	-1.6317287083192	0.102736649517595	0.358463548815044
**Arid3b**	4968.32738978856	-0.247859046804704	0.151916980751373	-1.63154273853263	0.102775849461095	0.3585209342392
**Samd10**	2550.81300715661	-0.177030207601656	0.108548369467028	-1.63088776432915	0.102914004003305	0.35885307885984
**Csnk2a2**	1176.8953858317	-0.214211121483157	0.131347324818375	-1.63087540442384	0.102916612513018	0.35885307885984
**Supt6**	4096.41123674276	-0.240041383858447	0.147270129103738	-1.62993938634603	0.103114308288301	0.358986335770828
**Cytip**	22657.6117992122	-0.109584854262869	0.0672296298288163	-1.63000829458529	0.103099743934235	0.358986335770828
**Rragc**	7769.37706024457	0.134600457209519	0.0825589633358862	1.63035546681837	0.103026390950682	0.358986335770828
**Rpl29**	7813.43226607852	-0.157886635163386	0.096851556924099	-1.63019201939231	0.103060920097267	0.358986335770828
**Rpl23**	6603.49726094459	-0.109728934904745	0.0673063351044953	-1.63029133490016	0.10303993806604	0.358986335770828
**5330406M23Rik**	469.07150511167	-0.353450770880543	0.216818810878086	-1.63016654066645	0.103066303443885	0.358986335770828
**Gm49521**	33.3249150725589	-0.665180398457323	0.408054753313899	-1.63012535218681	0.103075006544381	0.358986335770828
**Zkscan14**	3554.36775779988	-0.187278836821828	0.114921684495384	-1.62962140386438	0.103181537837984	0.359061725201661
**Cox8a**	6305.83013585577	0.136801296383893	0.0839424336731656	1.62970371953399	0.103164130878422	0.359061725201661
**Vwa8**	1208.2998979761	-0.176316366177007	0.108231335028049	-1.62906949388837	0.103298308320296	0.359388704836147
**Gm11263**	470.797096038524	0.23702622734087	0.145566333565973	1.62830389097796	0.103460464859209	0.359873409445155
**Cldn4**	246.663554846511	-0.324038950554636	0.199041347630782	-1.62799817430759	0.103525272889533	0.360019361050624
**9330175E14Rik**	1703.79029088419	-0.217929109920089	0.133891509326641	-1.62765444213816	0.103598178251416	0.360193401303524
**Pigu**	3457.50684120105	0.15299702274525	0.0940521281027306	1.62672579378677	0.103795347996897	0.360799315770373

**Slc35b4**	5699.72510531806	0.100462605578302	0.0617656302707278	1.62651308078553	0.103840552969706	0.360876840128729
**Nelfcd**	6269.82025323522	0.0965446939374445	0.0593699980230496	1.62615289122904	0.103917134781473	0.361023850817029
**Rnf185**	3717.52741756315	0.154901153403466	0.0952593845923099	1.62609861554754	0.103928678511521	0.361023850817029
**Adamtsl4**	483.251953536342	0.301583118709068	0.185564378240855	1.62522096949893	0.104115483867608	0.361188753120869
**Slc46a3**	2199.41045306738	0.18287921551791	0.1125235815553	1.62525235146406	0.104108799678419	0.361188753120869
**Plekha2**	19159.8520400064	0.085758085186171	0.0527581781285661	1.62549368132439	0.104057409110975	0.361188753120869
**Tbc1d10c**	90431.100201834	0.109671913753544	0.0674661514022261	1.62558425927828	0.104038125973177	0.361188753120869
**Lgals8**	5197.33675067686	0.16838771443976	0.103622302054829	1.62501422088329	0.104159528728897	0.361188753120869
**Trbv14**	2261.47563145258	-0.189544361704754	0.116609017245821	-1.62546916337679	0.104062629224369	0.361188753120869
**Gm8818**	23.1940232976663	1.02780575039187	0.632450082553042	1.62511758436789	0.104137506754152	0.361188753120869
**Gm48904**	39.1095932130548	-0.706286219404018	0.43461276095772	-1.6250931469376	0.104142712905657	0.361188753120869
**Picalm**	2063.70358232763	-0.161224196245561	0.0992277547542542	-1.6247893207384	0.104207457322577	0.36127544665167
**Preb**	13677.0961217805	0.112646557697007	0.0693499319483186	1.62432109927597	0.10430729645765	0.361542030418653
**Nacc1**	1571.47991101119	0.465946527016045	0.286912740058026	1.62400082659909	0.104375632134537	0.361699326048802
**Acvr2a**	286.285715978265	-0.359314840290271	0.221330805306066	-1.62342896549531	0.104497736944523	0.362042841036687
**Plcg1**	13525.2736460287	0.301223854683212	0.185623376705023	1.62276896385681	0.104638802662968	0.362262611119594
**Ppfia4**	264.388995716524	0.400890788832493	0.247019294424415	1.6229128569354	0.104608034734852	0.362262611119594
**Kcnab1**	25.138558241773	-1.22255194295656	0.753585237110245	-1.62231408306862	0.104736114782942	0.362262611119594
**Ep400**	14084.2394707164	-0.195876211612829	0.120695794786653	-1.62289176651985	0.104612543942221	0.362262611119594
**1700021J08Rik**	61.6783455065734	0.599580008260475	0.369592745485886	1.62227212407061	0.104745094641167	0.362262611119594
**Gm8210**	46.9400960466608	0.619916605059049	0.382037563604236	1.62265877525394	0.104662368591581	0.362262611119594
**Nupr1l**	22.9723410969802	0.994306100780933	0.612899207177553	1.62229953822226	0.104739227530149	0.362262611119594
**Gm26520**	85.3694580240778	-0.628673915851274	0.387496812402626	-1.62239764490773	0.104718233110203	0.362262611119594
**A430088P11Rik**	872.379609232569	-0.346620500655498	0.213705800408589	-1.62195176730246	0.104813675971656	0.362420252860519
**Ciao1**	6715.69949658615	0.088579787381738	0.0546550541418581	1.62070624158249	0.105080654401255	0.362627216688693
**Rnaseh1**	2062.50817967878	0.155470258182509	0.095925246156782	1.62074390644154	0.105072573032133	0.362627216688693
**Asic1**	48.7936462017998	-0.71945297302324	0.443811782228562	-1.62107677585883	0.105001174039487	0.362627216688693
**Mrps15**	3435.0024377143	0.113632968691122	0.0701099774795665	1.62078170292153	0.105064463918336	0.362627216688693
**Dhx57**	2386.87496552491	-0.199438200578696	0.123044581262428	-1.62086130516659	0.105047387136209	0.362627216688693
**Gm5805**	474.647604010022	-0.186320277044734	0.114933651174781	-1.62111161648728	0.104993703108355	0.362627216688693
**Zfp27**	626.168826377691	0.261788282571519	0.161516666926198	1.62081280869384	0.105057790647087	0.362627216688693
**Cfl2**	1444.00410969619	-0.183309943024628	0.113088876520526	-1.62093698924807	0.105031152946746	0.362627216688693
**Gm44937**	17.1680307181554	-1.14497461240813	0.70630926830179	-1.62106695153675	0.105003280760698	0.362627216688693
**Dcaf11**	14417.1637372198	0.0804575965115667	0.0496601234849259	1.62016505126068	0.105196826632073	0.362894364905207
**Ran**	19260.3188200665	0.107285714966678	0.0662203931001641	1.62013104942459	0.105204128887922	0.362894364905207
**Dhodh**	4826.44373955125	0.0935169812347969	0.0577298214753567	1.61990768107116	0.105252109619108	0.362916003529182
**Nek9**	3256.37948593949	-0.174023086217879	0.107450451696292	-1.61956588800347	0.10532556223513	0.362916003529182
**Ndufb10**	4894.26937668775	0.154962668075356	0.0956801678839999	1.61959026099566	0.105320323040869	0.362916003529182
**Cep295**	1821.70647746642	-0.187172249754793	0.115555529165647	-1.61976022355871	0.105283793805847	0.362916003529182
**Gm12992**	333.680927248907	-0.542036719074398	0.334667582941627	-1.61962719636619	0.105312383844371	0.362916003529182
**Pwwp2a**	1013.22519445012	0.838035636595178	0.517544717030871	1.61925261531592	0.105392921436202	0.362989347119177
**Zscan22**	1789.98631727617	0.198057288925758	0.122311235728777	1.61928941152183	0.105385007826064	0.362989347119177
**Fam185a**	839.425905656688	0.166273820648683	0.102701306761836	1.61900394348702	0.105446414635503	0.363094221025267
**Ankrd33b**	25.3040393238132	1.8365123916515	1.13476188032372	1.61841212988895	0.105573809640377	0.36337407568882
**H2-Q4**	1303.64456862562	-0.267432730531261	0.165235045547313	-1.61849884596476	0.105555135329115	0.36337407568882
**Msrb2**	908.634065384308	0.171721966144114	0.106118437345823	1.61821046784263	0.105617247707457	0.363444195703517
**B3glct**	583.46743293858	0.323019817582747	0.199659333741307	1.61785483067511	0.105693886612584	0.363628509029364
**Adgrl1**	1813.91842937767	-0.21627035477172	0.133702345890252	-1.61755093623595	0.10575941003976	0.363669475164793
**Rufy2**	2915.24403901133	-0.153068471806273	0.0946340072518195	-1.61747849691032	0.105775033615551	0.363669475164793
**Wrn**	2731.10540949615	-0.183028125048951	0.113144973229133	-1.61764256798484	0.105739649700979	0.363669475164793
**Gcc2**	2536.22803854759	-0.119841348891556	0.0741154760753742	-1.61695445050746	0.105888113480655	0.363899455641151
**Clec12a**	229.774646152981	0.519524330528852	0.321296379278066	1.61696291659493	0.105886285888335	0.363899455641151
**Rab5c**	2091.00156672868	0.132460983027169	0.0819323935762194	1.6167107690311	0.105940728244144	0.363921529309567
**Pikfyve**	1428.52947733129	-0.165472688911345	0.102346809988293	-1.6167840397788	0.105924905727721	0.363921529309567
**Unc45a**	8212.27645522226	0.107818810161483	0.066695849670663	1.61657450491868	0.105970158902916	0.363943285491116
**Wdyhv1**	3308.19910434433	-0.118861094398696	0.0735381658098855	-1.6163184530055	0.106025479165414	0.363974613335386

**Gm12036**	44.6096040977771	-0.769921508752311	0.476318450661816	-1.61640076650936	0.106007692756367	0.363974613335386
**Derl3**	41.9445349344605	0.672106105110946	0.415927309155052	1.6159220380992	0.106111170160035	0.364030853083315
**Cdk5rap1**	1805.50818695903	0.387273409768215	0.239647811514404	1.61601062542955	0.106092015923804	0.364030853083315
**Gm17828**	91.7169937336841	0.430109093898122	0.266151323123447	1.61603214611346	0.106087363163753	0.364030853083315
**Dnd1**	101.098335098384	-0.66330197446211	0.410526026885584	-1.61573671587691	0.106151249156332	0.364089079877302
**Nfat5**	825.442112310935	-0.343808390771075	0.212833788536874	-1.61538444217239	0.106227467266945	0.364269280427637
**Anapc10**	1027.92756405846	0.192650916752343	0.119267798368013	1.6152802297725	0.10625002302757	0.364269280427637
**Tmem106b**	3009.9848143434	-0.146526355828052	0.0907376076656311	-1.6148360045815	0.106346213846945	0.364519750181926
**Sharpin**	8126.00158535138	-0.10094610012333	0.0625178661082246	-1.61467603434485	0.106380870091412	0.364559236631226
**Pa2g4**	10961.0438249062	0.121512067378161	0.0752740464538313	1.6142624596738	0.106470509160615	0.364707785400828
**Zfp608**	40.3913418842463	-0.550102918157919	0.340759043728198	-1.61434576215298	0.106452449187173	0.364707785400828
**Arsa**	2312.06191645596	-0.153230929046712	0.0949858893434621	-1.61319676118039	0.106701766894807	0.365420504447181
**Radx**	25.3669491460993	1.81240026249286	1.12395796447065	1.61251605467866	0.106849689176764	0.36584757765282
**Abhd15**	3238.64507225322	-0.186253180234064	0.115606193069098	-1.61110036832318	0.107157848112444	0.366663672683555
**Mtmr9**	1996.77779479752	-0.17519519327346	0.108729275627884	-1.61129734620002	0.107114928916093	0.366663672683555
**Colq**	128.525272335885	-0.578176616657916	0.358847703476904	-1.61120333516396	0.107135411132104	0.366663672683555
**Ddx46**	7081.11363700366	-0.144462648453061	0.0896753694690361	-1.61095124902655	0.1071903485387	0.36666965049586
**Slc49a4**	5618.24387520359	1.06293391035287	0.65990537212958	1.61073686507912	0.107237087023768	0.36666965049586
**Wfikkn2**	292.123694372683	-0.318523120951779	0.197751351336702	-1.61072538214641	0.107239590907681	0.36666965049586
**2700097O09Rik**	1026.78269103334	-0.215182694560454	0.133598634328976	-1.61066537574466	0.107252676215994	0.36666965049586
**Orai3**	2711.1796021132	0.132819059675073	0.0824869827018499	1.61018205933352	0.10735811683301	0.366950509097123
**Gm10355**	122.59269968976	-0.477424544080387	0.296532246015994	-1.6100257239971	0.107392240618767	0.366987537503774
**Foxred2**	764.289068341766	-0.180172186906058	0.111983352725351	-1.60891938418692	0.10763397031753	0.367717030199869
**Pnisr**	8759.70311618508	-0.168637975094373	0.104819920370083	-1.60883517654822	0.107652386895475	0.367717030199869
**Dhx16**	7188.3870869289	0.189584880424089	0.117860432576477	1.60855408621609	0.107713880644536	0.367847337953176
**Crkl**	9429.20164858284	-0.172457920803863	0.10728984608379	-1.60740207110725	0.107966196056788	0.368034195558389
**Mlst8**	3682.4092240233	0.128439649439351	0.0799102501057738	1.60729880421274	0.107988836498854	0.368034195558389
**Stom**	136.778168975269	0.537650221207383	0.334340505865532	1.60809178599383	0.107815077920573	0.368034195558389
**Mcm8**	215.418201703647	0.369793667333867	0.230072227215946	1.60729381294152	0.107989930890426	0.368034195558389
**Lypla2**	17444.9078020074	-0.0860594046948197	0.05352291652626	-1.60789826639205	0.1078574616037	0.368034195558389
**Adcy7**	36923.7230660135	-0.170494306402331	0.106092786045292	-1.60703015499608	0.10804775330133	0.368034195558389
**Rab8b**	8764.7691061385	0.0977167703004092	0.0607770889524499	1.60778957966973	0.107881271403318	0.368034195558389
**Dpy19l3**	1102.68387075619	0.145550262847422	0.0905712486171757	1.6070250225061	0.108048879142801	0.368034195558389
**Nhsl2**	244.420118433804	0.725124084406012	0.450946334121655	1.60800527587921	0.107834023297649	0.368034195558389
**Jpx**	378.133251625432	-0.328568556204045	0.204454172708463	-1.6070523377019	0.108042887502838	0.368034195558389
**Dancr**	317.190773590727	-0.21377820958473	0.133022502860408	-1.6070830497684	0.108036151066957	0.368034195558389
**Gm19040**	74.0676568269004	-0.877216510899883	0.54570830885742	-1.60748241626843	0.107948583620974	0.368034195558389
**Csrp1**	2719.45917819306	0.15202679131591	0.0946383771927231	1.60639685321653	0.108186741855428	0.368424139056837
**Angptl7**	25.6580389509526	1.29955142997171	0.809120428425527	1.60612856172785	0.108245665517514	0.368545149429445
**Serpind1**	31.2619065900481	-0.929044440819304	0.578481748720481	-1.60600475792749	0.108272864556678	0.368558117697034
**Prpf19**	2032.58164511789	0.217926803517167	0.135709787904892	1.60582966697948	0.108311340344071	0.368609457840503
**Atp5a1**	50445.1547152547	0.0821753283059103	0.0511836754681666	1.6054987758161	0.10838408237564	0.368697751725597
**Gm14305**	348.042709253616	-0.493536027502513	0.307396923080733	-1.60553340142866	0.10837646858358	0.368697751725597
**Ddx47**	12841.0975457248	0.0974148797129673	0.0606812351494391	1.6053542659945	0.108415863068625	0.368726258228431
**Gm43389**	21.0941178918634	-1.41607140041103	0.882188477659208	-1.60518011317539	0.108454172666378	0.368776952676764
**Smyd2**	2090.6498022817	0.202371991531894	0.126110663562335	1.60471752201878	0.108555984051985	0.369032582731904
**Mex3c**	267.29891354001	-0.320900119753718	0.199984401576814	-1.60462574692586	0.108576191758907	0.369032582731904
**Ggh**	1018.95065259625	0.183236234324165	0.114204210819028	1.604461280456	0.108612412627177	0.369076080605226
**Herc1**	14218.2362563013	-0.257445999147079	0.160494829857864	-1.60407658847999	0.108697171649762	0.369284461769145
**Gm20605**	16995.3924779872	-0.260412476223712	0.162366944990012	-1.60385154896973	0.108746778764798	0.369373354817186
**Gm6658**	43.3143650906065	-0.758888689007095	0.47326719897283	-1.60351000587865	0.108822101836869	0.369549538500765
**Ino80dos**	114.089943354126	-0.465695235769348	0.290444651600209	-1.60338719685005	0.108849195922877	0.369561900486267
**Phf20**	4752.01479554485	-0.116577180490413	0.0727150100594312	-1.60320655109766	0.108889059623891	0.369617602863776
**Qars**	5835.70273234956	0.101449341304929	0.0633045553064179	1.60255989184153	0.10903185474274	0.369878871110656
**Fbxo31**	3741.72337096509	0.322428338481939	0.20117891793901	1.60269446612536	0.109002125889797	0.369878871110656
**Gm10863**	118.46233582192	0.498069643049243	0.310813844482557	1.6024692975901	0.109051871562253	0.369878871110656

**Lrrc32**	54.7541618956043	1.70817980913276	1.0659915052672	1.60243285306911	0.109059924806759	0.369878871110656
**Pip4p2**	1485.31858792271	0.144508342245264	0.0902116497813801	1.60188116053157	0.109181891258831	0.370158942240024
**Exo5**	1602.15757025574	-0.174882227411402	0.109189831524146	-1.60163473988629	0.109236403996966	0.370158942240024
**Zfp747**	1616.71480988747	-0.294876479843861	0.184099508255131	-1.60172334319988	0.10921680085166	0.370158942240024
**Ndufb6**	2416.57918392072	0.187831974870434	0.117275185141152	1.60163443480698	0.109236471499404	0.370158942240024
**Klf11**	846.738437749926	-0.44430079888421	0.277476889541305	-1.60121731081345	0.109328796020156	0.370392139086133
**Tab1**	4780.38064146369	0.188631501106839	0.11784504333524	1.60067403573546	0.109449134799115	0.370448845262111
**Pdcl3**	2786.72961073204	0.128053081745073	0.0800023404559472	1.60061669465263	0.109461842309655	0.370448845262111
**Uspl1**	6128.17511459734	0.170905871455456	0.106775388842245	1.60061108939589	0.109463084568153	0.370448845262111
**Gm15519**	476.915439759063	0.20554039383728	0.128394416012613	1.60085150289626	0.109409813217848	0.370448845262111
**6820402A03Rik**	79.9851823847327	-0.58855136446218	0.367630007279321	-1.60093396297491	0.109391546254302	0.370448845262111
**Utp20**	2526.17277635847	-0.196349912737481	0.12268463685759	-1.60044417758191	0.109500081312174	0.370479851668369
**Ifrd2**	2251.31202588576	0.160804332427876	0.100481529743189	1.60033722455123	0.10952379312185	0.370479851668369
**Nde1**	7218.0506135696	0.123232285716481	0.077008069824997	1.60025158397724	0.109542782821789	0.370479851668369
**Stk11**	6870.68860153745	0.382183696082629	0.238869753936905	1.59996688481364	0.109605929921962	0.370613870768316
**Gns**	3264.86637592047	0.137747369185233	0.0861438315483105	1.59903926618336	0.10981187805352	0.371230586245293
**Vti1a**	3778.41871897455	-0.119939211304302	0.0750283697420238	-1.59858479821298	0.109912889732388	0.371492364545953
**Dctn3**	5046.27256978818	0.153253413344559	0.0958800918161233	1.59838617633435	0.109957059193856	0.371561951901692
**Akr1e1**	372.511007582007	0.231089774558855	0.144624016879507	1.59786582854621	0.110072840453288	0.371873444901899
**Tmem17**	25.1120793959043	0.967544945699255	0.605700269220996	1.59739890316317	0.1101768168105	0.372144930864533
**Il2rg**	194648.803583559	0.135119264068907	0.0845938946181366	1.59726969279339	0.110205603470007	0.372162386171646
**Zfp280c**	832.247513986655	-0.173580215884871	0.108680972106255	-1.59715369232404	0.110231452169503	0.372169914684991
**Smarcd3**	41.6442989532643	-0.650183779360257	0.407157390292866	-1.59688561441212	0.110291207023129	0.372250841814205
**Cyp4f16**	583.928886082963	0.328036147602117	0.20542905950378	1.5968341986012	0.110302670586439	0.372250841814205
**Gpx2-ps1**	43.5623532020201	-0.732260947248329	0.458601859949559	-1.59672476541824	0.110327072717152	0.372253465696824
**Rnf38**	7795.18736464736	-0.202492295870485	0.126934617656602	-1.59524879507881	0.110656611087093	0.373205526733588
**Gpr171**	9142.9279852124	-0.233829405407001	0.146574801741529	-1.59529061358949	0.110647263623685	0.373205526733588
**Kif20b**	1445.94755175583	-0.1758727399937	0.11030568143511	-1.59441234309551	0.110843709647158	0.373756544598815
**Ckap2**	144.926786191256	0.467419861420969	0.293231134850752	1.59403216735111	0.110928830324937	0.373963538602915
**Rpl32l**	37.5537222529348	0.653079943670236	0.409810262257595	1.59361539672652	0.111022203821655	0.374198260025201
**Mfsd14b**	6348.98757538076	-0.139120972362708	0.0873056116926754	-1.59349404540487	0.111049403091992	0.374209889760589
**Stard3nl**	1852.7892850773	0.141957425136346	0.0891020696659638	1.59320008691754	0.111115311747378	0.374351927988761
**Alkbh5**	7722.27501355775	0.507828697149149	0.318798475817403	1.59294581270212	0.11117234766555	0.374464019274492
**Capn12**	34.895784314219	-1.43004269967055	0.897919146413718	-1.59261856190742	0.111245786871888	0.374631302359551
**Zfp146**	1008.14576195955	-0.313728298572866	0.197052574320624	-1.59210454191986	0.111361216741872	0.374939891496085
**Naf1**	98.7990666135339	-0.557009574508428	0.349974825750443	-1.59157040313984	0.111481264672986	0.374949721658555
**Eif4a3**	11614.7446042213	0.110366215577294	0.0693441597594389	1.59157189242994	0.111480929812452	0.374949721658555
**Ebp**	4913.7154025024	0.148185907650086	0.0931106446605385	1.59150340103798	0.111496330631296	0.374949721658555
**Ankrd50**	737.422384127095	0.374143008624481	0.235028437688242	1.59190527029232	0.11140599119329	0.374949721658555
**Ap1ar**	5401.74587223491	0.565751951046758	0.355493225014139	1.59145635201418	0.11150691094066	0.374949721658555
**Gm47572**	85.8965743284028	-0.551584263649686	0.3465889251657	-1.59146534583002	0.111504888364465	0.374949721658555
**Gm49584**	81.5103178927431	-0.400393267463513	0.251608963683603	-1.5913314915402	0.111534993205902	0.374964132269128
**Dolpp1**	2330.90394055159	0.127889910036891	0.0803739929803478	1.59118522415779	0.1115678971786	0.374994743140616
**Herpud1**	11768.9919116887	0.209262495166926	0.131527534995953	1.59101662760855	0.111605833771161	0.375042252662016
**Slc4a10**	66.8334031886581	-0.653356746885222	0.410689005797708	-1.59087956497926	0.111636682288704	0.375065928107271
**Gtf3c5**	2548.27044161299	0.170064009207715	0.106920255634751	1.59056867380367	0.111706679120064	0.375141121674094
**Hoxa7**	184.603411571048	-0.444514366720869	0.279454718522598	-1.59064899340722	0.111688591926731	0.375141121674094
**Ppp1r15b**	3428.33029025842	-0.122642263817776	0.0771184631775601	-1.59031000832318	0.111764943775053	0.375256812074047
**Gm7099**	75.1761688661492	0.635039907178953	0.399407635027677	1.58995435111036	0.11184509505614	0.375445923050619
**Enpp4**	4505.77940594986	-0.190759933973615	0.119995720407446	-1.58972281116267	0.111897299504693	0.375541160446314
**Atp2b1**	11503.2936283074	-0.169263650665533	0.106520803770502	-1.58901965319572	0.112055955735723	0.375993546534877
**Map2k7**	2390.32617592111	-0.21978754787448	0.13834095152345	-1.58873815348323	0.112119521307142	0.375999455999552
**Klra9**	202.831875615063	1.37209932310722	0.863683523500545	1.58865983403972	0.112137211710232	0.375999455999552
**Galt**	3224.31487612503	0.136977731432663	0.0862260242008884	1.58858920728543	0.112153166414793	0.375999455999552
**Usp38**	6234.90181763797	-0.158921976272355	0.10002933852368	-1.58875364585893	0.112116022227784	0.375999455999552
**Tent4a**	260.582682372211	-0.327503511294289	0.206189971141719	-1.58835810238893	0.112205385919555	0.376094504559547

**Tulp2**	41.718531261693	1.01933042767842	0.641840786406655	1.58813595094998	0.112255600410453	0.376182793634095
**Cnnm3**	492.374054097681	-0.323473869477042	0.20377778347351	-1.58738535655479	0.112425393768338	0.376271650299003
**Psmb8**	56385.3418714287	0.099547080178866	0.062699763212013	1.58767872603055	0.112359006022702	0.376271650299003
**Zrsr2**	1223.28911364308	-0.161374628734975	0.101654237582536	-1.58748550550045	0.112402727189117	0.376271650299003
**Hipk2**	1468.20071025083	-0.180241700168899	0.113514332318857	-1.58783209562127	0.112324311713949	0.376271650299003
**H3c7**	143.209199781027	0.909555768292802	0.57297321100127	1.58743157765326	0.112414932160106	0.376271650299003
**Gm36931**	194.930318316127	-0.472685765757548	0.297729358947256	-1.58763572201587	0.112368735638344	0.376271650299003
**Gm47657**	24.1297352675734	-1.45944763093515	0.919470164819181	-1.58727024190301	0.11245145196789	0.376278940042057
**Tnip3**	85.1058880513418	0.670904149493898	0.422818849848114	1.58674134262203	0.112571238707441	0.376599789450776
**Pcna**	7708.437521146	0.134620115691298	0.0848540929681437	1.58648936052899	0.112628343756641	0.376630902498597
**Gas2l1**	195.312936931523	0.511848294582731	0.322625131258646	1.58651092240048	0.112623456437872	0.376630902498597
**Pgp**	3977.44424507462	0.283329393019519	0.178625559937987	1.58616377811709	0.112702162175384	0.376784046188967
**Grb2**	5535.44202018984	0.698718881795607	0.440532910157581	1.58607646712631	0.112721964443409	0.376784046188967
**St3gal2**	212.502735295775	0.431670882149617	0.27221749897124	1.58575728518916	0.112794378741028	0.376869929728289
**Stx17**	1549.64176394476	-0.240559405989861	0.151700486150753	-1.5857523736002	0.112795493342553	0.376869929728289
**Degs2**	197.698415877678	-0.41951937642504	0.264661690367272	-1.58511560869603	0.112940069834423	0.377272987148823
**Tchp**	880.032757995063	-0.187469181113923	0.118295778548669	-1.58474954401517	0.113023250344079	0.377310880438911
**Anapc16**	4281.63464988524	0.153542743317792	0.0968860057366005	1.58477730762502	0.113016939955512	0.377310880438911
**Cbwd1**	581.537874732328	0.224843162916445	0.141877286272633	1.58477208595873	0.113018126766409	0.377310880438911
**Psmd14**	9957.10121333339	0.120312943028836	0.0759274929508001	1.58457678968535	0.113062521900415	0.377362032955907
**Brip1**	301.500619645789	0.442774959409455	0.279458084676908	1.58440561818551	0.113101444242736	0.377411998503344
**Gimap4**	52004.3792980885	-0.104221305161697	0.0658024902466136	-1.58385047087273	0.113227750723813	0.377753476213236
**Fam149a**	99.2908252833916	0.662050894215811	0.418091239792088	1.58350817047743	0.113305685888549	0.377933465822581
**Vcp**	33889.6850359422	0.103828759708698	0.0655792441573862	1.5832564257605	0.113363030245746	0.378044712715813
**Gm7879**	271.223295125276	0.344103808486411	0.217378566945933	1.58297026850857	0.113428241118117	0.378169525144321
**mt-Tt**	47.7630163958447	-0.551334409401697	0.348310584852736	-1.58288158149084	0.113448457533617	0.378169525144321
**Chmp4b**	669.471929840981	-0.269177567328825	0.170088745590359	-1.58257130061451	0.113519209129982	0.378325333811576
**Prpf31**	8934.32409606859	0.100702943584818	0.0636499760124334	1.58213639491627	0.11361843671311	0.378537027491394
**Tsfm**	1949.81552691487	0.109157616414881	0.0689961667168251	1.58208233310826	0.113630776165151	0.378537027491394
**Rnf141**	1019.15577008241	0.175253055895217	0.11081833432787	1.58144459541061	0.113776417634327	0.378862006057499
**Hip1**	267.860246284447	0.493753918181211	0.312202863382507	1.58151630267487	0.113760034367484	0.378862006057499
**Zgpat**	28684.1084782206	0.120036811436588	0.0759141210553958	1.58121848435808	0.113828090365169	0.378873937449086
**Purg**	27.7536455757619	-1.17776956060655	0.744804184227496	-1.58131437168028	0.11380617516068	0.378873937449086
**Tm9sf2**	16643.0211579155	-0.139613382410588	0.0883015767890262	-1.58109727467447	0.113855797792072	0.378886125830976
**Cct5**	25773.3175350943	0.0870897125844984	0.0550919932726447	1.58080525700895	0.113922572173738	0.379028287529898
**Wdr66**	39.4260630438362	-0.732274435487688	0.463273837729354	-1.58065138984923	0.11395776869548	0.379047908240316
**Pigs**	17726.4396674772	0.105657276853246	0.0668499109983849	1.58051484699506	0.113989009518404	0.379047908240316
**Pdzd8**	2253.45836304112	-0.258913214046466	0.163821009742206	-1.58046403482618	0.11400063699631	0.379047908240316
**Rbm27**	3175.09062325032	-0.155381568049455	0.0983264038994966	-1.58026289874566	0.114046672642005	0.379056018473253
**Fubp3**	852.618103208317	-0.198127962970431	0.125386486045822	-1.58013809317558	0.114075245262434	0.379056018473253
**1600014C10Rik**	11948.6944950051	0.18040139547037	0.114161978965058	1.58022309271273	0.114055785106186	0.379056018473253
**Mri1**	7904.54461495201	-0.112002612564397	0.0708955256099666	-1.579826252796	0.114146661715143	0.379213356239829
**Septin3**	38.7463628763541	1.45666198391401	0.922673107396865	1.57874112969835	0.114395446684264	0.379879674057736
**Nckipsd**	1966.03865547627	0.141663237089308	0.0897282396366574	1.5788032581822	0.114381191047537	0.379879674057736
**B4gat1**	335.119520230101	0.58482284802038	0.37049923319695	1.57847249230203	0.114457102738072	0.380004333721829
**Gpr146**	6865.32797293444	-0.13455489993792	0.0852507229114886	-1.57834321331939	0.114486783399364	0.380022803038505
**Tmem131**	1722.83239854512	0.341187491916317	0.216204729550392	1.57807598670867	0.114548154123063	0.38014643313334
**Slco5a1**	20.2743796709098	1.79310669190201	1.13644521480473	1.57782061866494	0.114606825620272	0.380181000273393
**Gm43133**	52.3439066692657	-0.661105451825884	0.418990867988824	-1.57785169638475	0.114599684166715	0.380181000273393
**Ifnar1**	16560.4854664264	-0.100671186789738	0.0638139260848627	-1.57757393983032	0.114663523200014	0.380208993068733
**Proser3**	229.732738223109	0.29490696627477	0.186935658575351	1.57758540303266	0.114660887966111	0.380208993068733
**Gm14049**	27.5688028429981	-0.642092922430811	0.407074788851562	-1.57733404282363	0.114718683185841	0.380231865999011
**Gm19427**	47.652772646599	-0.567724312958639	0.359905710138129	-1.57742513376837	0.114697736009315	0.380231865999011
**Lrpap1**	7021.90479207063	0.128327894470579	0.0813791222643158	1.57691421214615	0.114815265909651	0.380471954771476
**Rps6-ps3**	175.645821533989	-0.262290757051221	0.166420083680434	-1.57607634397589	0.115008210062452	0.381031195532813
**Gm38384**	35.2071591849881	-1.02413421190139	0.64987757759609	-1.57588790136395	0.115051639710327	0.381094952052896

**Gm15706**	32.3417553453925	0.934979508795337	0.593372250811668	1.57570480843414	0.115093848792299	0.38115463964276
**Eci1**	3026.68558069928	0.139903451996464	0.0888088168683001	1.57533291096463	0.115179621169786	0.38119833874655
**Tfdp1**	3220.84393457306	0.180219305791105	0.114389923914159	1.57548234690974	0.115145150049158	0.38119833874655
**Parp12**	973.346905578822	0.83074714285478	0.527328509639776	1.5753882592509	0.115166852763537	0.38119833874655
**Farp1**	203.309591441255	-0.405666493291154	0.257592656448398	-1.57483718241175	0.115294031424595	0.381345592609728
**0610010F05Rik**	534.153975928014	0.241191508689822	0.153154430334431	1.57482554153446	0.1152967191212	0.381345592609728
**Gm10125**	47.494315899865	0.790701284798954	0.502023092308156	1.57502970862065	0.115249587278125	0.381345592609728
**Prg4**	91.0512770756766	-0.487142465256539	0.309386003396368	-1.57454590675985	0.115361297228859	0.381399068492473
**Lrrc24**	56.5096167752411	0.760827784851721	0.483176637460245	1.57463694612992	0.115340269718051	0.381399068492473
**Gm6794**	34.3676290294607	-0.55129798898289	0.350207540927286	-1.57420364942215	0.115440375931076	0.381580449663512
**Reps1**	709.070654368442	-0.223421575898777	0.141937683664831	-1.57408216148123	0.115468456019018	0.381582789270485
**Tbc1d1**	7344.29732802549	-0.159844917274829	0.10156064995751	-1.57388631661676	0.115513733884636	0.381582789270485
**Gm12663**	180.812135257154	-0.32867799695803	0.20882659280611	-1.57392788217925	0.115504123071158	0.381582789270485
**Cmtr1**	15833.1319630994	0.112521220088419	0.0715455677105529	1.57272104602816	0.115783424409639	0.382393506271783
**C1qbp**	5863.44284301765	0.0916331250431236	0.0582700108453156	1.57256063134044	0.115820589552635	0.382436091697585
**Gm38368**	20.1763601204032	-1.98493508558318	1.26248439850061	-1.57224523957729	0.115893687385223	0.382458288979604
**Particl**	317.744867617092	-0.359542294759215	0.22868483855674	-1.57221745450347	0.115900128823863	0.382458288979604
**Gm45799**	570.923209726012	0.468010740111373	0.297638637277133	1.57241258861028	0.115854896659461	0.382458288979604
**Adat3**	157.892347132807	-0.627163238099319	0.39896872566519	-1.57196090258369	0.115959618781504	0.382574479300704
**Gm12191**	279.472529640743	-0.594924167564116	0.378517610144892	-1.5717212399613	0.116015214081684	0.382677774395039
**Rtf2**	9269.62525886639	0.106218653584175	0.0676093931752122	1.5710635548659	0.116167887005281	0.383101171105528
**Atp6v0c**	15430.7635149517	0.100483659075997	0.0639777594103919	1.57060297206462	0.116274899193481	0.383169947790638
**Rgp1**	1520.14266391297	-0.281764146479514	0.179394641602911	-1.57063858742893	0.116266621528206	0.383169947790638
**Rnf181**	8713.73828319141	0.109950096265884	0.06999531932442	1.57082069668514	0.116224303226328	0.383169947790638
**Gm43545**	88.1353610343527	-0.458104234793974	0.291682995920727	-1.57055516159906	0.116286011952455	0.383169947790638
**Cep83**	1123.62238375996	-0.179207036885602	0.114116140009343	-1.57039168053643	0.116324016750821	0.383215039084819
**Fxr1**	3688.59242988525	0.1143830443536	0.0728531726084758	1.57004891150468	0.116403732694267	0.383317370128228
**Gm37183**	122.046120137617	-0.611101664205483	0.389203746510198	-1.57013304646971	0.11638416190634	0.383317370128228
**Vars**	31843.4581284602	0.089678523801339	0.0571459982446584	1.56928790389486	0.116580869542057	0.383555703839713
**Ccdc88c**	11039.6356310645	-0.22837029783849	0.145540770880052	-1.56911562620967	0.116620999341363	0.383555703839713
**Kctd9**	681.966779951624	-0.201911263283174	0.128661898398174	-1.56931668036106	0.116574167503697	0.383555703839713
**Amdhd2**	2369.40955146954	0.179199119171987	0.114193601082206	1.569257099117	0.116588044309568	0.383555703839713
**Sp3os**	107.988647309447	-0.462141983595713	0.294458750314309	-1.56946255834617	0.116540197179904	0.383555703839713
**Trbv15**	1806.64280552653	-0.184153560438539	0.117361743217117	-1.56911064364354	0.116622160125409	0.383555703839713
**Echdc1**	2396.48854777132	-0.18711605864911	0.119273611827907	-1.5687967839784	0.11669529802795	0.383556072387654
**Mad2l2**	1915.54528356935	0.134191991607073	0.0855321257528602	1.56890747687965	0.116669499437516	0.383556072387654
**Gm7729**	38.7387032692439	0.628383170020465	0.400541224639154	1.56883519439621	0.116686345413891	0.383556072387654
**Gm20705**	62.6785574268164	-0.567180920953703	0.361566303039698	-1.56867749064389	0.116723106090575	0.383567462496181
**Kat8**	2360.18191119518	0.1216553990664	0.0775694500879941	1.56834164646514	0.116801421534156	0.38374478713797
**Emc7**	4592.88515167083	0.163145934763705	0.104033325330385	1.56820840096759	0.116832504459571	0.383766890300074
**Patz1**	3838.09581777079	0.588090163282369	0.375135190617831	1.56767527544886	0.116956934639134	0.383855534078075
**Mrps26**	5348.65659330538	0.124359205896974	0.0793251786411801	1.5677141612186	0.11694785527964	0.383855534078075
**Rbpj**	2464.98775914167	-0.158235266844457	0.100920045827064	-1.56792702131356	0.116898164814539	0.383855534078075
**Gm49172**	147.653379392912	-0.313813369563258	0.200160931780107	-1.56780529932788	0.116926577796111	0.383855534078075
**Shc1**	10112.8535612902	0.108725013434065	0.0693775644449799	1.56714947121399	0.117079757953798	0.384178622673468
**Kctd10**	25088.3881910533	-0.108143574739548	0.0690552997698493	-1.56604308575843	0.117338530740739	0.384491952213651
**Ppie**	5303.35056871091	0.122465821570897	0.078190897412725	1.56624141202101	0.117292111161189	0.384491952213651
**Brap**	5055.45082110276	0.337146245838446	0.215354877229289	1.56553800952224	0.117456812317291	0.384491952213651
**Emp1**	89.4316381614887	1.02737464118837	0.656291575016664	1.56542408938022	0.117483503703096	0.384491952213651
**Arhgap5**	1887.12656730695	-0.224115493589641	0.143169489648076	-1.56538585239451	0.117492463661149	0.384491952213651
**Gnptab**	2809.88003790606	0.167622304946807	0.107077824263139	1.56542501774114	0.117483286169739	0.384491952213651
**Tmem51**	120.410837526116	0.476891741630652	0.304487033735082	1.56621362749249	0.117298613446252	0.384491952213651
**Arhgap12**	3043.57593401442	-0.178049275137026	0.113673178869144	-1.5663261721746	0.117272276881572	0.384491952213651
**Olig3**	34.5672065268156	0.973651877801935	0.62195620120903	1.56546695074225	0.117473460766336	0.384491952213651
**Gm26558**	163.243146927233	-0.354440128179219	0.226250634491078	-1.56658180860569	0.11721247268452	0.384491952213651
**A330009N23Rik**	112.932606543743	0.446093376227008	0.284936503410581	1.56558872200452	0.117444931959856	0.384491952213651

**Gm43201**	170.90672564969	-0.459044194439549	0.293097049980514	-1.56618497002978	0.117305320316515	0.384491952213651
**Gm45767**	55.9081872574907	-0.830304961589261	0.530322217888085	-1.56566127833717	0.117427935907729	0.384491952213651
**Capsl**	20.7932115333241	-1.26882593886782	0.810691670227172	-1.56511530272943	0.11755587607553	0.384619588729677
**Tpgs2**	2847.17163565118	0.193160539501854	0.123511134785713	1.5639119488051	0.117838248023483	0.385463415840985
**Igkv19-93**	43.7874343419052	-1.0153894876238	0.649376940248393	-1.56363650245327	0.117902957432549	0.385595039490384
**Gm19684**	125.887828180794	-0.327726301110426	0.209641861392449	-1.56326746449233	0.117989697604186	0.385798643940478
**Poll**	1818.39191692405	0.126770575611615	0.0811204953204919	1.56274410197778	0.118112796675994	0.385881959363459
**Eprs**	15072.2993383021	0.122220932449013	0.0782248758736568	1.56243050671522	0.118186605057311	0.385881959363459
**Pop7**	1324.28944686391	0.147278204531005	0.0942573682555493	1.56251131616264	0.118167582131841	0.385881959363459
**Apeh**	13303.9980115745	0.0942686456146735	0.06032775126377	1.5626083127565	0.118144751847298	0.385881959363459
**Gm12840**	89.4513795659011	-0.488565683628429	0.312591545242737	-1.5629523288899	0.118063807969763	0.385881959363459
**Gm37570**	64.8357746992547	-0.83519774655845	0.534503452708162	-1.56256754250466	0.118154347602416	0.385881959363459
**Gm38228**	150.304190182678	-0.428890048823729	0.274414759504504	-1.56292631488974	0.118069927307307	0.385881959363459
**Gpr15**	1354.23707762613	0.276591258589327	0.177053374680237	1.56219139617563	0.118242906876669	0.385888030880885
**Gm5464**	54.3513057662075	-1.23329916560995	0.789508220345248	-1.56211060737359	0.118261934449692	0.385888030880885
**Gm27003**	61.318505122707	-0.462187322859645	0.295838474047577	-1.56229619675944	0.11821822756889	0.385888030880885
**Zfp944**	1050.44094469682	-0.211964873193518	0.135718711890949	-1.5617954977633	0.118336172775085	0.386050325966254
**Rad17**	1789.63347303853	-0.213522076245945	0.136733182476744	-1.56159662474222	0.118383045122392	0.386123295796632
**Dnmt1**	11476.7641038525	0.154854771988307	0.0991718030726433	1.56147984800554	0.118410574995078	0.38613316022298
**H2-M3**	9324.86072567642	0.113468089705395	0.0727165839216052	1.56041556940743	0.118661707903962	0.386386819763888
**Lats2**	5975.15903904701	0.125900676882451	0.0806775479634818	1.56054168799775	0.118631926489288	0.386386819763888
**Ccdc77**	930.806263792112	0.208995403856505	0.133925170492632	1.56053864324184	0.118632645403373	0.386386819763888
**Cd99l2**	657.77835700285	0.219437618316388	0.140645746104968	1.56021511061284	0.11870905594193	0.386386819763888
**Ttn**	119.136536764043	-0.683231186280478	0.437896980564731	-1.5602555317905	0.118699507334111	0.386386819763888
**Hba-a1**	805.554890018927	0.397069955560555	0.25439934977479	1.56081356305378	0.118567746341763	0.386386819763888
**Adam1a**	261.249578737794	1.35174634648917	0.865940509400037	1.56101525660893	0.118520151173729	0.386386819763888
**Gm3336**	19.6704424756402	1.34827075563637	0.864093172462107	1.5603302960891	0.118681847511709	0.386386819763888
**Gm48796**	51.8307661585814	-0.883386688596363	0.565932701689662	-1.56093946499098	0.118538034543047	0.386386819763888
**Slc25a45**	4736.65838265144	-0.135692302451581	0.0869939574095981	-1.5597899726839	0.118809521840092	0.386395090771259
**Sh3tc1**	414.239951692604	-0.359226189758876	0.230298149525771	-1.55983098647815	0.118799826816358	0.386395090771259
**Cggbp1**	6117.45312882247	-0.154518486624965	0.0990540513530878	-1.55994110805391	0.118773798854988	0.386395090771259
**Zfp420**	721.35966152743	-0.212593360540824	0.136296205667623	-1.5597892802626	0.118809685523053	0.386395090771259
**Taf12**	5626.45188450565	0.134005394047323	0.0859263624769987	1.55953761086064	0.118869189904564	0.386508837252622
**Gm45570**	17.5902774451131	-1.06173723153562	0.680991632568326	-1.55910466554682	0.118971609584036	0.386762049147031
**9630010A21Rik**	180.000979507981	-0.333832183806195	0.214172758184723	-1.55870516229831	0.119066179393681	0.386989642884948
**Zfp202**	700.164363326587	-0.311462237129549	0.199874955177796	-1.55828546329124	0.119165593363214	0.387232884022306
**Plxna1**	179.131913219832	0.62035506527852	0.39816065029438	1.55805217019779	0.119220881546105	0.387332666087004
**Peli3**	50.4265942959285	1.19051142078409	0.764548612794875	1.55714286947965	0.119436568817741	0.387718331552173
**Zfp719**	2091.67169905119	-0.28136155038903	0.180687574077381	-1.55717155330523	0.119429760311472	0.387718331552173
**Ssr2**	5965.52423528025	0.160575052129623	0.103121995399451	1.5571367825809	0.119438013667211	0.387718331552173
**Tubg2**	122.916209990459	0.556324061940916	0.35721808172145	1.55737934446086	0.119380447261374	0.387718331552173
**Creb1**	5438.94642851154	-0.15126860756788	0.09716585604721	-1.55680826291885	0.119516014821537	0.387880408295369
**Rps25-ps1**	142.608444496038	0.892162881775519	0.573104576493297	1.55671917197812	0.119537174759301	0.387880408295369
**Cd48**	18096.9213423352	0.182456367326262	0.117232881498491	1.55635829294711	0.11962291695334	0.38799882717863
**5031425E22Rik**	1628.85743024078	-0.170182173882064	0.10934220109303	-1.55641803604512	0.119608719107412	0.38799882717863
**Cdc20**	1280.33751104813	0.178353230900939	0.114615010880731	1.5561070886826	0.119682629667673	0.388112614874155
**Xkr5**	25.15508941815	-1.16715034716555	0.750101791034228	-1.5559892818764	0.119710641071809	0.388123574355659
**Ehmt2**	5032.83750602623	-0.178420533702708	0.114686120089658	-1.5557290940109	0.119772525196188	0.388165022262326
**Hnrnpf**	111935.482335077	0.083016182880371	0.053361617423251	1.55572838472843	0.119772693929011	0.388165022262326
**Fbxo11**	1555.34196353881	-0.169178465270575	0.108759630070467	-1.55552630292105	0.119820775214955	0.388189892457288
**Nxf1**	21462.3376893435	0.126127337635256	0.081085326490213	1.55548905202324	0.119829639963968	0.388189892457288
**Snd1**	14140.7301010236	0.142591509832721	0.0917033881068144	1.55492084618109	0.119964921925031	0.388388591488126
**Hectd3**	7354.21478844084	0.131471595094504	0.0845475784525049	1.55500130814934	0.119945757775903	0.388388591488126
**Zfp445**	7301.43676412245	-0.125425614130199	0.0806566134024587	-1.55505678752408	0.119932545289749	0.388388591488126
**Gm48027**	70.4157015418016	-0.770718433726528	0.495954531535049	-1.55401026650779	0.120181967751622	0.389011352888722
**Pdik1l**	1574.66845046197	-0.208155540888321	0.134013622081499	-1.55324166047638	0.120365411925251	0.389459458276695

**Pigb**	2764.34921352944	-0.139814865782988	0.0900159570912112	-1.55322312066644	0.120369839551152	0.389459458276695
**Spc24**	255.12646460709	0.386407074407948	0.248801008894198	1.55307679870489	0.12040478822976	0.389492557613699
**Rnd3**	99.1316507376311	0.717324843953605	0.462006361645727	1.55262979799326	0.120511602567139	0.389758070946306
**Gm44553**	28.6338427943727	-0.998252890215478	0.643066832025162	-1.55233148485011	0.120582928097457	0.389908721122845
**Nifk**	4686.23828573458	0.175205011435961	0.112885105772028	1.55206490916337	0.120646693275089	0.389954860704733
**Dapl1**	6319.05658338304	-0.192677173659453	0.124139805252144	-1.5520982433322	0.120638718264315	0.389954860704733
**Capn15**	4189.07614755471	-0.182479604549667	0.117596669898261	-1.55174125855383	0.120724146300663	0.389965226170469
**Grk3**	91.3497245667088	0.827449614507354	0.533220550167812	1.55179618311212	0.120710999530123	0.389965226170469
**Tlnrd1**	403.897922387405	0.690325222922234	0.444839777047246	1.55185138232122	0.120697788147913	0.389965226170469
**Pak1ip1**	8211.77289739745	0.104487367614832	0.0673409643304757	1.55161674106804	0.120753955019919	0.389981567790298
**Galnt10**	14891.8994211754	-0.137746883702908	0.0887915925377576	-1.55135052504361	0.120817704818263	0.390027571157832
**Ccdc25**	2965.99310803305	0.157369982362	0.101435551432016	1.55142827283265	0.120799084111505	0.390027571157832
**Mid1ip1**	2142.86972976636	0.202343434633275	0.130501824740881	1.55050272312314	0.121020900409124	0.390378586083998
**Vta1**	5641.13045381791	0.126992740578809	0.0819052570855846	1.55048338894916	0.121025537413234	0.390378586083998
**Gm13456**	1624.63692733449	0.153401527484655	0.098923601753792	1.55070705842728	0.120971902227954	0.390378586083998
**Gm26586**	64.1037219141446	-0.637675765429861	0.411224741782512	-1.55067460840455	0.120979682480205	0.390378586083998
**Ehbp1**	88.4932410057293	-0.485409362376107	0.313177096273144	-1.54995166681266	0.121153117273698	0.390630175748243
**Rps10**	6180.47363973704	-0.137972245936186	0.0890163410761047	-1.54996536892284	0.121149828309136	0.390630175748243
**Rbm18**	5520.81511789268	0.141741622499887	0.0914585025148742	1.54979163885649	0.121191534505537	0.390674101719262
**Zfp160**	1315.81727677303	-0.153015660562687	0.0987610704601996	-1.54935198504508	0.121297129385345	0.390934519886455
**Jade1**	2101.49274034052	0.328736716859634	0.212233973693749	1.54893540905942	0.12139724788129	0.391177185044067
**Ppp1r12a**	14161.9096328728	-0.124705441675991	0.0805263258259493	-1.54862947485684	0.121470816251526	0.391254221519889
**Dnajb7**	39.2079111123485	-0.810950821897711	0.523651022728986	-1.54864745163959	0.121466492388678	0.391254221519889
**Gm38387**	67.8204801759955	-0.749995123174968	0.484360876699373	-1.54842217704645	0.121520685222737	0.391334853270932
**Bfsp2**	18.131254247359	1.40154172188859	0.905286315353255	1.5481750890509	0.12158014730597	0.391446338598317
**Cyb5r3**	9425.01018985739	0.0939447425322933	0.0607044792517649	1.54757513267956	0.121724622370234	0.391511507386769
**Gpr137**	314.475208807051	-0.241540390414163	0.156047172061661	-1.54786778397189	0.121654132461028	0.391511507386769
**Pnpla2**	9396.38968606207	-0.118355563032893	0.0764716627660436	-1.54770484584583	0.121693374866903	0.391511507386769
**Zfp239**	516.499817134469	0.347148729304354	0.224311771193362	1.54761708428179	0.121714515671794	0.391511507386769
**E030037K01Rik**	25.0805759382477	1.02028595966003	0.659112773135102	1.54796872590861	0.121629826329287	0.391511507386769
**Gkap1**	34.366633503533	-0.53842276271507	0.347976989707681	-1.54729415633882	0.121792330220614	0.391527020450784
**Aimp2**	4317.25997255097	0.202591008591108	0.130929733042778	1.54732621752857	0.12178460283574	0.391527020450784
**Gm48210**	23.906609314794	-0.974620684033123	0.629906826932228	-1.54724578677726	0.121803988973139	0.391527020450784
**Phf1**	7587.69504008786	-0.161831859842166	0.104655127810697	-1.5463347398982	0.122023746105111	0.392153409622321
**Rgs19**	13346.9922201615	0.117351496620326	0.0758964212004002	1.54620593124498	0.122054841529038	0.392173356030395
**Lpin2**	1829.68741462696	-0.244741903483663	0.158343499813454	-1.54563909331293	0.12219175428261	0.392293291000627
**Zc3hav1**	19622.4211266066	-0.121443262280183	0.0785711225573894	-1.54564753980038	0.122189713256484	0.392293291000627
**Ier2**	12976.6033492304	0.253854148900508	0.16422423312814	1.54577764843287	0.122158276912585	0.392293291000627
**Ppcdc**	13199.7753093493	-0.178800886409682	0.115674805735209	-1.54572022207649	0.122172151267008	0.392293291000627
**Dnaja3**	7422.56492978266	0.112271262652531	0.0726448240461676	1.54548192698739	0.122229737128978	0.392335295975008
**Sypl**	753.302330892818	0.191135852749543	0.123736768200996	1.54469730807148	0.122419496321837	0.392704398115468
**Abhd18**	940.119657379424	-0.154190642963775	0.0998160768786944	-1.54474757759876	0.122407331795865	0.392704398115468
**Hnrnph2**	9352.46768937296	0.141555785124504	0.0916362376332994	1.54475771572997	0.122404878623672	0.392704398115468
**Pfkfb3**	1916.62566716009	0.186648102029654	0.120851769851969	1.54443830039294	0.122482187548263	0.392825530062687
**Brd1**	2756.13084559615	-0.228008353980772	0.147773857591891	-1.54295460439604	0.122841790659516	0.393340079334755
**Tars2**	6850.28567104297	0.13844346808249	0.0897206960390504	1.543049421086	0.122818785316582	0.393340079334755
**Pomgnt1**	2550.32005036961	0.24550864894735	0.159069833897303	1.54340168045849	0.122733346214813	0.393340079334755
**Pink1**	12892.8963687623	0.160131362865739	0.103783298403	1.54293961870372	0.122845426941231	0.393340079334755
**Hemk1**	512.567004534888	0.250753357787944	0.162526126480194	1.54284952960164	0.122867288854837	0.393340079334755
**Gm336**	199.200037862041	0.405039960159643	0.262403620644822	1.54357611058991	0.12269105607607	0.393340079334755
**Gm6637**	132.445088342446	0.976560165854174	0.632682712177927	1.5435227596033	0.122703989677527	0.393340079334755
**Gm49463**	191.115881111587	-0.342360278177636	0.221864647618129	-1.54310423879203	0.122805486449622	0.393340079334755
**9030025P20Rik**	1263.63590542104	-0.191631200764702	0.124196559674885	-1.54296706177968	0.122838767936482	0.393340079334755
**Ccnf**	477.817057402817	0.308652581829084	0.200082150199015	1.54262927263665	0.122920751388739	0.393431297914354
**Tysnd1**	2847.10503625278	0.358789500776603	0.232600368672251	1.54251475534917	0.12294855512009	0.393440370232995
**Slc25a33**	519.148320009175	0.455885959231291	0.295597563423808	1.54225208743576	0.123012346998825	0.393507161289524

**Osgin1**	87.1158894477752	-0.477644783707642	0.309732492466541	-1.54212036297496	0.123044347507361	0.393507161289524
**Gm43980**	103.872462026196	-0.612248697129954	0.396995715916143	-1.54220479613256	0.123023834973158	0.393507161289524
**Med21**	3008.94234234724	0.129122317606821	0.0837392375810835	1.54195716771118	0.123084002398294	0.393553532626954
**Ap5z1**	3832.87775246989	0.145982990385796	0.094680094722964	1.54185513663611	0.123108800045583	0.393553532626954
**Pik3c3**	5129.6425904131	0.120594687674954	0.0782443488818392	1.54125747607755	0.123254133924688	0.393869774789449
**Mtmr11**	69.334102610968	-0.571503916518597	0.370807206326088	-1.54124274493203	0.123257717807119	0.393869774789449
**Sbno1**	17377.0155314493	-0.129351020302079	0.0839543455018792	-1.54073049499484	0.123382391696768	0.394142126016847
**Otud3**	192.070954887905	0.883230169053825	0.573270334636244	1.54068702964415	0.123392975035927	0.394142126016847
**Taf4b**	3199.4582511037	0.210700731908661	0.136805293246486	1.54015043503496	0.123523688367933	0.394479683342828
**Hint3**	548.756429608223	-0.282905699465693	0.183784822855115	-1.53933113230313	0.123723477305419	0.394723483328892
**Zswim6**	827.539555852343	-0.22324081281158	0.145048183718664	-1.53908037376447	0.12378467576106	0.394723483328892
**Slc43a2**	6609.27914583539	-0.161948876066032	0.105228857519528	-1.53901581641688	0.123800435020079	0.394723483328892
**Uqcrfs1**	6283.69295111918	0.124312322188829	0.0807595568851314	1.53928930498771	0.123733683759546	0.394723483328892
**Fam136a**	3482.68748470513	0.191085050921094	0.124127155998458	1.53942986435191	0.123699387901043	0.394723483328892
**Trbv17**	2885.27832740045	-0.0918740023069423	0.0596919337951687	-1.53913596805568	0.12377110575139	0.394723483328892
**Gm45223**	330.586482684193	-0.395992448714518	0.257215699056861	-1.53953452361778	0.123673856326038	0.394723483328892
**Snhg4.1**	658.941804088333	-0.195624086292659	0.127055430763001	-1.53967512539909	0.123639563067549	0.394723483328892
**Rpl35**	14372.1275377165	0.133053011466339	0.0864697259872866	1.53872363936832	0.123871778687178	0.394871053363113
**Cep162**	1179.73701281079	-0.212573309822169	0.138159275304068	-1.53861048673226	0.123899416867215	0.39487927014092
**Unc13a**	241.142242052944	0.403801466468453	0.262517223128419	1.53819037721163	0.12400207313009	0.395126525037581
**2810004N23Rik**	2640.40610275035	0.139917571027112	0.0909711886009363	1.53804268339165	0.124038178758512	0.395161662494517
**Skp2**	389.788550738813	-0.338794878235151	0.220297795578456	-1.53789499956432	0.124074290145937	0.395196804089252
**Celf3**	35.2882545466664	-0.658461520470814	0.428379066559959	-1.53710013367017	0.124268790252981	0.395736323366253
**A630072M18Rik**	367.512377645911	-0.348121096779534	0.226520317623038	-1.53682062797942	0.124337240536829	0.395874297663936
**Prss16**	102.951560653824	0.659000922887791	0.428900659694812	1.5364884804717	0.124418620917048	0.39600188997278
**Hist1h2br**	28.5121104659745	0.779955607930286	0.507634239952671	1.5364519304352	0.124427578679649	0.39600188997278
**Rab19**	3515.39534005751	0.182470477694563	0.118797250369402	1.5359823323113	0.124542713569463	0.396288274982639
**Map4k2**	13128.5112782658	-0.173801973791377	0.11316276075987	-1.53585837447164	0.124573119099084	0.396304994476937
**Cluap1**	1090.62597389019	-0.16452312677612	0.10713535748724	-1.53565667427502	0.124622606369966	0.3963823997924
**Nckap5l**	1132.89126739651	-0.307612793416779	0.200352103739267	-1.53536093545141	0.124695193796569	0.396453222084856
**Vps36**	5266.43856475491	0.127789186369707	0.0832302960554337	1.53536863889798	0.124693302610906	0.396453222084856
**Slc35a3**	1563.44556789886	-0.168550656823861	0.109793172066186	-1.53516519881814	0.124743254379271	0.396519023612007
**Gm48467**	20.7198976104352	-1.08839711764956	0.70902038313844	-1.53507168980365	0.124766219398891	0.396519023612007
**Dnajc24**	774.440301339543	0.213113881533681	0.138843501216043	1.5349215459647	0.124803100346809	0.396556251696848
**Smg8**	1634.19586281859	0.215177316876624	0.140220198986668	1.53456719097284	0.124890176906549	0.396614680948586
**Mcrip2**	422.795837248186	0.286673820703621	0.18682670827362	1.53443703714872	0.124922171831429	0.396614680948586
**Rab5if**	3516.190243898	0.188247670711378	0.122678050115806	1.53448535034325	0.124910294546198	0.396614680948586
**Hdac8**	1565.83685712237	-0.181107959910021	0.118021188827521	-1.53453766827156	0.124897433732746	0.396614680948586
**Nat9**	515.63466768362	0.20722983514388	0.135123308446975	1.53363499995414	0.125119472818241	0.396993060071438
**Rdh5**	501.268359238393	-0.266781695846896	0.173975888148061	-1.5334406318412	0.125167323883665	0.396993060071438
**Klhl20**	2065.24349548693	-0.182063215298321	0.118722138807972	-1.53352371450114	0.125146868197925	0.396993060071438
**Gm9791**	31.4844114993153	0.699331796060646	0.455977378698424	1.53369844367471	0.125103856833328	0.396993060071438
**Rpl3-ps1**	3466.11529610613	0.138476902611273	0.0902981464417319	1.53355199489759	0.125139905908704	0.396993060071438
**Grcc10**	11316.5254659405	0.119537483786088	0.0779746325044952	1.53303042215937	0.125268359340626	0.397233555671211
**Kpna2**	5279.43078254725	0.210408512042506	0.137271713749005	1.53278855706011	0.12532796097694	0.39734259177337
**Aig1**	1678.82520519788	0.244254225514464	0.15938214731386	1.53250680600677	0.125397419382027	0.397402883588616
**Slc25a37**	904.660071814493	-0.279799694975424	0.182569003186692	-1.53256954954892	0.125381948995139	0.397402883588616
**Brd2**	20876.2816987878	-0.118916238959781	0.0776135149749559	-1.53215891585573	0.125483224051505	0.397515440782812
**Gimap7**	12679.6885151371	-0.218913043548763	0.14289794523499	-1.53195375335012	0.125533847390701	0.397515440782812
**Tmem248**	6368.88953858377	0.242333774454913	0.158183463134456	1.53197919461992	0.125527568956242	0.397515440782812
**C920009B18Rik**	529.302767950691	-0.277036302867579	0.180822392930911	-1.53209067957324	0.125500059418877	0.397515440782812
**Plcd1**	118.065033094409	0.720399057708919	0.470364473258092	1.53157625345065	0.125627036134984	0.397730602447046
**Tmem115**	3868.90987087832	-0.152395231906453	0.0995239486643461	-1.53124181618255	0.125709639561288	0.397752367583777
**Gba**	9139.5828863008	0.11010310528529	0.0719023867398032	1.53128582064634	0.125698768380917	0.397752367583777
**Plppr3**	382.677178312737	-0.406049130357631	0.265159368475222	-1.53133993602634	0.125685400331128	0.397752367583777
**Ppp2cb**	314.646979260376	-0.35611923651141	0.232635147979924	-1.53080581160566	0.125817392870387	0.397853634248183

**2610002M06Rik**	1611.08492389845	-0.125505737434907	0.0819838834775383	-1.53085865308263	0.125804329901604	0.397853634248183
**Epcam**	238.907178578544	0.459677217723093	0.300250068810098	1.53098122356744	0.125774033256911	0.397853634248183
**Prr22**	94.4337393933711	-0.501459235377217	0.327668097044923	-1.53038773044929	0.125920784187735	0.398100681469933
**Rasgrp4**	78.2818962655215	-0.896935684470088	0.586295703490257	-1.52983499474851	0.126057577104201	0.398133742720163
**Dcaf10**	1384.38361785805	0.168736819098084	0.110292856821817	1.5298979821575	0.126041982924314	0.398133742720163
**Rps27-ps1**	22.7729461230553	-0.91201272149659	0.596108679954754	-1.52994370349684	0.126030664352638	0.398133742720163
**Gm5937**	30.2955519713035	0.590211275189438	0.385779276670895	1.52991959620719	0.126036632147508	0.398133742720163
**Gm5905**	88.3122284175517	0.365004151831918	0.23858822087095	1.5298498412851	0.126053901320552	0.398133742720163
**Papolg**	4100.51003654336	0.0905524487253117	0.0591962122725963	1.52970004750171	0.126090991882015	0.39815283458648
**Nsmce3**	1921.05207967511	-0.14180527522541	0.092707029469323	-1.52960650381246	0.126114158622905	0.39815283458648
**Fchsd2**	4223.97856342687	-0.20321447347926	0.132864932323282	-1.52948163165286	0.126145089242276	0.398155261399268
**Zfp988**	27.7895505859151	0.84495685270435	0.552476259444501	1.52939938732196	0.126165464248686	0.398155261399268
**Naa50**	4977.56281522798	0.120398734204567	0.078734597633333	1.52917189931247	0.126221834913303	0.39821496520601
**Zfyve19**	3289.84333855103	0.125283982250108	0.0819321296211803	1.52911907489002	0.126234927410295	0.39821496520601
**Gm48678**	155.982431543662	-0.567347158321297	0.371152101245993	-1.52861092909527	0.126360925024076	0.398532645237064
**Gfpt2**	17.1807674416184	2.24692136394843	1.4700831191006	1.52843151162983	0.126405435981282	0.398587683072959
**Atg13**	7572.02363058438	-0.107390068396898	0.0702792262312454	-1.5280485309207	0.12650048896831	0.398587683072959
**Nthl1**	472.540785360497	-0.230645370142769	0.150927679473131	-1.52818469712065	0.1264666871481	0.398587683072959
**Gm28043**	3091.88478561813	0.372048003612867	0.243445259470653	1.52826144333986	0.126447638809299	0.398587683072959
**Gm42820**	36.6150829107592	-0.739936254731853	0.484241668993008	-1.52803094428154	0.126504855178515	0.398587683072959
**Tmem41b**	1807.28920433513	0.110545098284803	0.0723537139644682	1.52784276338724	0.126551581930047	0.398655193217064
**Mef2c**	218.719965311982	0.615651514118524	0.403036958016351	1.52753116525246	0.126628983669221	0.39871444863769
**Trappc6b**	4621.52822997464	-0.0962441845084048	0.0630125396744291	-1.52738145463865	0.126666185257227	0.39871444863769
**Pithd1**	4116.27079591227	0.2148765377634	0.140682689095081	1.52738435087898	0.126665465489815	0.39871444863769
**Gm14168**	89.6041263764624	-0.506694048047983	0.331745082660127	-1.52735963404495	0.126671608166547	0.39871444863769
**Mrpl32**	1958.06783515849	-0.222899923217072	0.145957902315458	-1.52715214237129	0.126723183554839	0.398797124680167
**Tent2**	5000.65424172322	0.115593140141036	0.0757109272647223	1.52676957365567	0.126818320008023	0.398866721214857
**Kdelr2**	2513.50876797074	0.118657083794993	0.0777183394494738	1.52675783650955	0.126821239657704	0.398866721214857
**Lamtor5**	4793.69329333972	0.195342849378918	0.12794586337305	1.52676174304564	0.126820267889531	0.398866721214857
**Atp6v1f**	7358.45767720743	0.118430581392909	0.0775923163073951	1.52631841693869	0.12693058422136	0.399099777920958
**Gtf2e1**	2740.95425031677	0.113547668151985	0.0743961897957419	1.5262564986693	0.126945997774947	0.399099777920958
**Cln3**	4471.21740935523	-0.148778992622128	0.097539915209072	-1.5253139425355	0.127180811819884	0.39967851053967
**Lmo2**	79.9846153761486	0.622681669050663	0.408218789949577	1.52536258590051	0.127168685289398	0.39967851053967
**Rpl19**	36835.0767401883	-0.102103371410601	0.0669483913847785	-1.52510567167735	0.127232742803929	0.399736483258787
**Timm8b**	561.045415532601	0.232168082003444	0.152237722673249	1.52503648850392	0.127249996828462	0.399736483258787
**Stxbp1**	119.321887170014	-0.597416232625346	0.391805534465946	-1.5247774216351	0.127314623309462	0.399780095553448
**Gm37166**	38.9452120092491	-0.938763772529205	0.615631689261687	-1.52487889902978	0.127289305850132	0.399780095553448
**Selenbp1**	232.449110518234	-0.303010218298444	0.198761868383275	-1.52448867966035	0.127386682592139	0.399847003506841
**Gm21994**	222.719688888224	0.540345390747095	0.354426731488794	1.52456161666288	0.127368477228186	0.399847003506841
**Nfkbia**	19887.7196344334	-0.218538105896267	0.143374552764357	-1.52424612096573	0.127447240761843	0.399957413400591
**S100a13**	1628.14597349323	0.113703806266031	0.0746040836928968	1.5240962778135	0.127484662389665	0.399995186235356
**Nceh1**	3071.97621744609	-0.136783862642244	0.0897767281554692	-1.52360044136796	0.127608552860412	0.400043504078475
**Mreg**	22.7471137940366	1.31878236705241	0.865611628511737	1.5235266297425	0.127627003554134	0.400043504078475
**BC004004**	3294.70174603106	0.118709209504846	0.0778988738817126	1.52388864677431	0.127536530014245	0.400043504078475
**Trav4n-3**	84.9673373174703	0.398239230426755	0.261374445776962	1.5236349109912	0.127599937198614	0.400043504078475
**Gm44152**	432.70660490524	-0.321271178559604	0.21083971883742	-1.52376971630918	0.127566247034569	0.400043504078475
**Pigyl**	797.416323530459	0.218701847740343	0.143609531036407	1.52289229107572	0.127785654761128	0.400461130085739
**Il6st**	22593.324876699	-0.170133004014116	0.111728947019552	-1.52272986144176	0.127826303863261	0.400462208819172
**Zkscan5**	730.025094276632	0.198722278607952	0.130507565032473	1.52268781168744	0.127836828733924	0.400462208819172
**Ubb**	148108.729266465	0.122913511544298	0.080749264045131	1.52216262275284	0.127968338019881	0.400728569364642
**Ddx20**	3594.75404241662	0.161033556963639	0.105794484440046	1.52213565589893	0.127975093460008	0.400728569364642
**Gm37655**	63.3291421388653	-1.01534300652864	0.667091925819926	-1.52204361532434	0.127998152542504	0.400728569364642
**Anp32b**	9111.35110449635	0.15166004276705	0.0996958558158956	1.52122715157904	0.128202844064003	0.401172183507784
**Wapl**	8206.95644122768	-0.121463937708212	0.0798488231962019	-1.52117379876414	0.128216228732957	0.401172183507784
**Gm44775**	44.6399390376271	-0.743435960930928	0.488711145418709	-1.52121752880013	0.128205258058963	0.401172183507784
**Nras**	9730.40830625723	0.1317358583286	0.0866257691685616	1.52074676615293	0.128323397938976	0.401427790614343

**Ilk**	14285.8842600605	-0.135558946582598	0.0891534867395365	-1.52051200171941	0.12838234468282	0.401532474229297
**Hypk**	2586.62484383628	0.174585646710061	0.114836662152069	1.52029537813343	0.128436755130059	0.401543244163561
**Zfp131**	2349.56722751146	-0.131517711658533	0.0865022967124243	-1.52039560401225	0.128411578659038	0.401543244163561
**Rp9**	4718.78342628761	-0.142057147973602	0.093449125067972	-1.52015492783131	0.128472042323117	0.401573888293067
**C230013L11Rik**	17.6805590620352	-1.34474221123568	0.884809396922695	-1.51981004712721	0.128558723247829	0.401765133323293
**Mmab**	1707.86651052287	0.250035600901519	0.164580316715943	1.51923149675953	0.128704235834075	0.402140123743312
**Shank1**	22.2026584409899	-1.07015957336701	0.704458797452709	-1.51912301647259	0.128731534213775	0.402145674981454
**Gm16589**	117.325266576971	-0.502943448116037	0.331119506089894	-1.51891821190231	0.128783084256806	0.402226969005845
**Tnfsf10**	1071.44254227141	0.199203620761368	0.131160973504536	1.51877205115803	0.128819883246323	0.402262168115797
**Gm29253**	84.5402570473064	-1.99440789086849	1.3133467101743	-1.51856922122553	0.128870963411045	0.402341939858895
**Atg3**	10410.3288125293	0.139165233056546	0.0916693518249109	1.51812170901299	0.128983719390571	0.402523850489796
**Cenpc1**	1362.72353505708	-0.137975220231183	0.0908907405062639	-1.51803384440107	0.129005866914607	0.402523850489796
**Gatad2b**	2753.5958704116	-0.30074890457785	0.19810560282787	-1.51812417359627	0.128983098199926	0.402523850489796
**Wsb1**	5854.15305542245	0.162642151297832	0.107158851183078	1.51776684335635	0.12907318645186	0.402654167278155
**Tst**	83.4622927650208	0.54262082472597	0.357656507118154	1.51715630479697	0.129227225372987	0.402900280783076
**Gm20633**	195.396196661245	-0.735541620157248	0.484817958413022	-1.51715011251838	0.129228788416429	0.402900280783076
**Ighv5-6**	20.4973122209201	2.11331700795126	1.39290142475776	1.51720500129346	0.129214934005725	0.402900280783076
**Tiprl**	6056.38085110778	0.124436377506488	0.082025702939204	1.51704128154462	0.129256261725394	0.402906214838185
**Mfsd6**	3223.69723011115	0.290551533829448	0.191590892041921	1.5165205962185	0.129387766391209	0.403193854835951
**2010008C14Rik**	292.174417704852	0.310058069373795	0.204459959002838	1.51647330306611	0.129399715929771	0.403193854835951
**Prep**	3959.29249008146	0.107781293949126	0.071087529062713	1.51617724473222	0.129474540326615	0.403347238419628
**Trip12**	7685.01790599011	-0.190100258789291	0.125390817717527	-1.51606203906843	0.12950366594593	0.403358225797593
**Fam49a**	7866.02088613541	-0.169412898558243	0.111774284600534	-1.51566971923552	0.129602888106412	0.403587491678406
**Nxf2**	25.2662349519063	0.683739845180878	0.451320082529399	1.51497766584836	0.12977806019049	0.403734036410967
**Hcls1**	12523.4789679227	0.0786039045921522	0.0518789252439541	1.51514134540234	0.129736613146113	0.403734036410967
**Rsl24d1**	2566.66864080587	0.139343019737679	0.0919746983479848	1.51501469687323	0.129768682270358	0.403734036410967
**Slc22a21**	38.8057936118999	0.751085305981682	0.495757194932282	1.51502653649692	0.129765684056418	0.403734036410967
**Gm42690**	94.9695559263101	-0.445756038428105	0.294225416602124	-1.5150154040937	0.129768503175463	0.403734036410967
**Hsph1**	11008.4118513005	0.166148137385817	0.109690295645171	1.51470224789326	0.129847824906773	0.403818891722533
**Rpl18**	23569.5180829819	-0.0928994893724108	0.0613349716959998	-1.5146251282687	0.129867364901675	0.403818891722533
**Hspa2**	45.0634927335751	0.745210948556415	0.492029202096814	1.51456650414376	0.129882220171974	0.403818891722533
**Gm16196**	90.2464463318221	-0.418502694922943	0.276351430868994	-1.51438584416571	0.12992800745083	0.403881557191306
**Tbc1d2b**	2513.60423603398	0.155501322550815	0.102704142153631	1.51407060406781	0.130007933327167	0.404036259918806
**Gm37589**	112.704938372844	-0.51458407424833	0.33988664989955	-1.51398730841712	0.130029058453276	0.404036259918806
**Exosc9**	3534.20185558289	0.142302944566938	0.0940268248921218	1.51342922331158	0.130170666609021	0.404237129238933
**Afap1**	1043.81978312299	-0.312639757397618	0.206557313768195	-1.51357389237	0.130133946888897	0.404237129238933
**Gm15602**	17.6341249992196	-1.03276038152676	0.682374006982583	-1.51348142068536	0.130157417009535	0.404237129238933
**Emb**	37171.9340948237	-0.160266601629326	0.105906167799645	-1.51328864936858	0.130206354614394	0.404268281706209
**Thap1**	747.131157221116	0.145709098863271	0.0962937482423427	1.51317298913907	0.130235723380403	0.404279805615644
**Ascl3**	57.9112157546156	-0.51834026016908	0.342578185919851	-1.51305681877348	0.130265226856031	0.404291743070806
**Smarca4**	13043.1774752029	-0.148760067371727	0.0983308623073865	-1.51285226103984	0.130317190440854	0.404371303937135
**Arhgap9**	30071.1260342535	-0.135552429410171	0.0896064000838731	-1.51275388011672	0.130342187770306	0.404371303937135
**Pole4**	10685.4465905035	0.12137984746461	0.0802545859993782	1.5124350335014	0.130423228171855	0.404543072107071
**Dnajc19-ps**	132.662905659527	0.303690842522059	0.200843496214223	1.51207705624751	0.130514260885213	0.404745760434619
**Ube2e3**	1358.84805786501	0.361015434680384	0.238837488496709	1.51155263335202	0.130647709301842	0.404761296789054
**Gne**	5902.6077449305	0.107423603284405	0.0710602302464345	1.51172607957875	0.130603561219287	0.404761296789054
**Zfp362**	1482.62073156611	0.21985178391255	0.145436305743905	1.51167057488164	0.130617687831098	0.404761296789054
**Cop1**	677.214518463109	-0.261125224530696	0.172736626606396	-1.51169575127634	0.130611279989489	0.404761296789054
**4930405G09Rik**	149.705663437414	-0.446890863458151	0.295648733762696	-1.51156021461891	0.130645779364461	0.404761296789054
**Osbpl5**	5211.49535279538	0.142749993176374	0.0944584392739667	1.51124657863913	0.130725639081011	0.404909030457717
**mt-Tq**	41.5644631670233	-0.736908417407708	0.487675616741676	-1.51106266565312	0.130772485625393	0.404909030457717
**Gm29361**	54.7683160940224	-0.568372714561416	0.376136266797356	-1.51108192624145	0.130767578934029	0.404909030457717
**Eif4e**	13026.3442948776	0.118027795241211	0.0781161172437339	1.5109275704647	0.130806905534646	0.404936033498904
**Pmaip1**	879.32285360905	0.392149754917396	0.25956851143194	1.51077552802556	0.130845651713218	0.40497641603716
**Cbx3**	6337.79718530016	0.127043989402354	0.0841178424220611	1.51030965303308	0.130964429753259	0.40502581346852
**Wdr3**	6417.30093692358	0.0943502901154214	0.0624690709735624	1.51035206134811	0.130953614002767	0.40502581346852

**Zfp157**	1434.67445995055	-0.148471669751503	0.0982961411992621	-1.51045267840705	0.130927955556574	0.40502581346852
**Klf10**	1358.89095516215	0.783553226540241	0.51871509101722	1.51056570381182	0.130899137497141	0.40502581346852
**Nbn**	1920.69367753546	0.141329015204138	0.0935994058686565	1.50993496051095	0.131060020852619	0.405241905136325
**Sez6l2**	36.1000404715947	-0.666737875070342	0.441610068268837	-1.50978866420331	0.131097358471656	0.405277825669587
**Stat5b**	18201.6627669024	-0.147356352525102	0.0976469123004237	-1.50907334449798	0.131280040534294	0.405684808732785
**Gm44200**	18.9084952331508	-0.778915468597087	0.516155427243997	-1.50907154605754	0.131280500077964	0.405684808732785
**G3bp1**	27165.9668913448	0.0627038631714101	0.041554234498025	1.50896446364306	0.131307864391676	0.405689807690124
**Gpat4**	8242.31812221647	0.10828624349081	0.0717778762225801	1.50862980613997	0.131393412756475	0.405874535346752
**Tmod4**	67.6361276300638	-0.646027191100225	0.428366530136952	-1.50811780484737	0.131524379057022	0.406040290125685
**Sf3b5**	3258.60235058001	0.122294581169742	0.081089114250017	1.50815041329319	0.131516035031879	0.406040290125685
**Gm43857**	84.0532698087739	-0.570961726323557	0.37855570648986	-1.50826342473548	0.131487120229254	0.406040290125685
**Fignl1**	329.09442692426	0.444881988539068	0.295319201279704	1.5064445068633	0.131953102627371	0.40728404272272
**Pdpk1**	5154.51413556454	-0.189639724064952	0.125930300227473	-1.50591020367932	0.13209022681411	0.407458589377879
**Grpel2**	2360.13466143352	0.137113872226531	0.0910422317843328	1.50604691404463	0.132055130802153	0.407458589377879
**Raver2**	1117.9770333671	-0.229479718776749	0.152395067889028	-1.50582116570763	0.13211308835004	0.407458589377879
**Gm10709**	729.214487022087	0.324389225504982	0.215413858624266	1.50588837494804	0.132095831311404	0.407458589377879
**Gm10130**	1958.81349015082	0.379528663810156	0.252062978873649	1.50568983000237	0.132146815917194	0.407482852917266
**Smim40**	143.255964256217	-0.293900888896237	0.195285554483288	-1.50498017978789	0.132329172520419	0.407965324086135
**Pald1**	269.084086854514	0.430617977587086	0.286192088335615	1.50464668709536	0.132414936394699	0.407982423507742
**Marchf3**	224.721483327208	-0.538965069967404	0.358214996418335	-1.50458544549035	0.132430690495081	0.407982423507742
**Rnf122**	1860.89281334212	-0.146310497466674	0.0972259693775937	-1.50484997376012	0.132362652310616	0.407982423507742
**Gm8670**	50.0548061819915	-0.588550176701078	0.391178666549898	-1.5045559153109	0.132438287506638	0.407982423507742
**Mrm2**	1121.84140699036	0.208783728679111	0.138804083227875	1.50416128851448	0.132539842631493	0.408215461365213
**Gyg**	3682.2865808033	0.163073077237205	0.108437686155262	1.50384135828679	0.132622219246311	0.40831260387074
**Aff1**	3375.71028988195	-0.313927746846052	0.208751107604762	-1.50383751467526	0.132623209152151	0.40831260387074
**Pdcd6ip**	28145.6839524894	0.103701487094337	0.0689881606886789	1.5031780244484	0.132793142793641	0.408755919320062
**Gm17018**	1817.29376888476	0.160439261963452	0.10675091113726	1.50293107809788	0.132856817872999	0.408872046723604
**Lrsam1**	579.518809733612	-0.275560912067375	0.183427839034386	-1.50228511396091	0.133023491423318	0.408951634191519
**Pfn2**	193.617197277461	-0.501718307814948	0.333965069263853	-1.50230773811425	0.13301765113924	0.408951634191519
**Rab6a**	3662.73425020083	0.0864733086344776	0.0575600580522159	1.50231447918334	0.133015911012683	0.408951634191519
**Kmt5b**	5213.12889901611	-0.149828982628886	0.0997066081376893	-1.50269862176016	0.132916778301894	0.408951634191519
**Gm13684**	34.613819884426	-0.742822394891045	0.494480671469176	-1.50222736246497	0.133038400511882	0.408951634191519
**Gm4767**	507.883871339268	0.276817346070662	0.184241301851064	1.50247172208127	0.132975325634251	0.408951634191519
**Slc46a1**	134.618785979985	0.500559639439997	0.333382179816497	1.50145889536003	0.133236910624524	0.40927656610964
**Exosc4**	5787.75038264677	0.0900075271342237	0.0599384945230803	1.50166479572764	0.133183700054515	0.40927656610964
**Shld2**	1537.3589424698	-0.150520096488303	0.10024512262915	-1.50152039860475	0.133221014696129	0.40927656610964
**Rpl9-ps6**	1946.47011899807	-0.137314471845952	0.0914566473271026	-1.50141598078524	0.133248003055306	0.40927656610964
**Cnpy4**	2923.69420708922	0.153011489773922	0.101921808689491	1.5012634856204	0.133287425331449	0.40931786414883
**Mlx**	6876.60171786396	0.124530736193255	0.082958843973757	1.50111465189473	0.133325909774622	0.409356266624849
**Psme2b**	6575.6306393613	-0.114239468641872	0.0761121481703296	-1.50093607115409	0.133372097353594	0.409418300798865
**Gm45838**	53.708850586081	-0.564953225716389	0.376449948207839	-1.50073928394985	0.133423008136479	0.409494807013341
**Gm45155**	289.269773045123	-0.285755051324595	0.19048355858202	-1.50015598958664	0.13357400047237	0.409799965492764
**Gm7664**	122.571128612035	0.323207792261466	0.215449706048772	1.50015425033023	0.133574450896163	0.409799965492764
**Zfp783**	1546.74423435692	-0.197065716578114	0.131391101509655	-1.49984066130714	0.133655681799175	0.409969355287055
**Arhgap26**	3818.29813387467	-0.13008300649794	0.0867481922866801	-1.49954717290304	0.133731740533265	0.410050993709628
**Nat8f4**	738.890456205808	0.19674110824181	0.131201224937095	1.49953712959721	0.13373434388994	0.410050993709628
**Gm15834**	95.4961379094238	-0.45018510126043	0.300278859961459	-1.49922342624523	0.133815679655778	0.410220557263831
**Gm50253**	819.503911130517	-0.436695609290035	0.291392163132942	-1.4986525532974	0.133963791514789	0.410594721435232
**Gm6206**	112.570366557867	0.32165397174891	0.214657521590262	1.49845190313379	0.134015879909372	0.410674488473741
**Tnip1**	4689.87695572721	-0.204111071693534	0.136233277455633	-1.49824679774005	0.134069141062143	0.410757817560992
**Angel1**	2462.8770329436	0.188849191309572	0.126070862507111	1.49796065128784	0.134143474063544	0.410825796077601
**Triap1**	1734.19181402833	0.147556007742673	0.0984996255597005	1.49803623013002	0.134123837656171	0.410825796077601
**Dynll1**	9343.44518172636	0.164615416953276	0.109937079080505	1.49736029308848	0.134299534242758	0.411214650134277
**Elmod2**	732.304349020197	-0.250651339533499	0.167405407897083	-1.49727146023618	0.134322637827069	0.411214650134277
**Ranbp1**	6381.30894331581	0.154996823502992	0.103579321185619	1.49640702148676	0.134547620925485	0.411468416591395
**Xpnpep3**	1468.55008303378	-0.182325475406381	0.121854893203713	-1.49625075048546	0.134588323863175	0.411468416591395

**Ncbp1**	9158.78635989001	-0.109366215539392	0.0730916483373098	-1.49628881038061	0.134578409761784	0.411468416591395
**Pianp**	77.1275117961929	0.704870490289413	0.471021690376908	1.49647140395038	0.134530854393077	0.411468416591395
**Atp6v1h**	5683.23991950126	0.0760618723294082	0.0508270077369192	1.49648534737879	0.134527223447155	0.411468416591395
**Zfp382**	561.212403130938	-0.184362273270788	0.12320914184031	-1.49633599030937	0.134566120795478	0.411468416591395
**Gm5112**	106.316732567303	0.396784915300838	0.265154022119175	1.49643181773988	0.134541163274249	0.411468416591395
**Mks1**	1801.43417518713	0.172221619017664	0.115134888543647	1.49582477732087	0.134699322787619	0.411488412013281
**Eogt**	789.463950141572	-0.245163587903701	0.163894721296999	-1.49586018368115	0.134690093999359	0.411488412013281
**Nat14**	24.8098093218689	-0.853377982035313	0.570459352446107	-1.4959487970108	0.134666998775902	0.411488412013281
**Gm42467**	220.602410903032	0.394818908507487	0.26394115555458	1.49585958914938	0.134690248962052	0.411488412013281
**Gm42449**	638.386230100373	-0.435376017025332	0.291098477004952	-1.49563138050333	0.134749740952974	0.411562641635203
**Impa1**	6807.0789407255	0.1210497261988	0.0809427735784236	1.49549763181167	0.13478461750201	0.411589383329298
**Col14a1**	18.8301565287282	-1.75446012322128	1.17351904120192	-1.49504188822055	0.134903510413158	0.411759964178611
**Shmt2**	17333.7043441863	0.108243587300876	0.0723982107329587	1.495114122366	0.134884660800041	0.411759964178611
**Ccdc69**	3570.16784923324	0.175083395924263	0.117113969627598	1.49498301936991	0.134918873837289	0.411759964178611
**Creb3l1**	227.379993461288	0.376488735416498	0.251886636920788	1.49467530321942	0.134999202725534	0.411925336434207
**Eif4ebp2**	5441.36909712707	-0.216233627206113	0.144713348826112	-1.49422032563105	0.135118041731298	0.412208128472421
**Ggt1**	9714.19079590477	0.467961028599489	0.313341876427375	1.49345192521036	0.135318929516831	0.412581341408031
**U2af1**	2465.26375388746	-0.207437379600416	0.138885453797015	-1.49358607348176	0.135283841669564	0.412581341408031
**Ptgr2**	2751.91220994589	0.168560446149933	0.11286503336022	1.49346915631483	0.135314422152378	0.412581341408031
**Cfp**	960.632146633917	0.322939818646752	0.216310904550242	1.49294285148599	0.135452146904548	0.412748654760558
**Sdhb**	11530.9885905101	0.126234944786515	0.0845884199076275	1.49234309997002	0.135609223344987	0.412748654760558
**Dctn2**	7516.99351139211	0.0997152257203739	0.0668386740675703	1.49187917192324	0.135730823750698	0.412748654760558
**Mfn1**	1742.69884099209	0.127046516873608	0.0851276230717537	1.49242410734904	0.135587999093055	0.412748654760558
**Lrrc39**	70.1850049113949	-0.459712208431797	0.308030650793195	-1.49242358592566	0.135588135699825	0.412748654760558
**Cd38**	809.269678926127	0.468646463534863	0.314109245105964	1.49198557774626	0.135702926229318	0.412748654760558
**Ddx54**	16294.5690846299	0.10187471610377	0.0682439293338334	1.4928026140673	0.135488862844221	0.412748654760558
**Iqcd**	40.7441581774958	0.668520249015529	0.448115809946171	1.49184704975224	0.135739246424688	0.412748654760558
**Rab20**	2819.49508545438	0.167304963185826	0.112087731978241	1.49262510921627	0.135535346894588	0.412748654760558
**Upf2**	1756.28683628631	-0.168895267658008	0.113212558763912	-1.49184215516421	0.135740529857791	0.412748654760558
**Commd3**	3836.92045905979	0.130682912104238	0.0875838022385857	1.49208996143198	0.135675563175814	0.412748654760558
**Gm14698**	790.326564667454	0.16776766256883	0.112441331540373	1.49204620997033	0.135687031629208	0.412748654760558
**Fam122a**	1539.22994687851	-0.173205232253673	0.116082309460056	-1.49208982022599	0.135675600188558	0.412748654760558
**Gm28914**	28.8404804890041	-0.705467656762715	0.472753256142003	-1.49225340618449	0.135632726463047	0.412748654760558
**Tgfb3**	21.5183620841869	1.51430174704153	1.01514747145813	1.49170617040147	0.135776190813501	0.412777433657793
**Tnfrsf9**	1765.04783867621	0.209453570458268	0.14043922602054	1.49141786375008	0.135851820939938	0.412848050636569
**Ccr6**	45.0160580608307	0.724040102738007	0.485447296773149	1.49149064697821	0.135832724989463	0.412848050636569
**Rpl35a-ps6**	93.8531355063685	0.433260292154971	0.290588192018014	1.49097693593865	0.135967550371925	0.413040426298521
**Gm43290**	43.2007237269485	-0.538227055941968	0.36098248682672	-1.49100600606236	0.135959918052136	0.413040426298521
**Dmap1**	2691.92089626542	0.122420332545932	0.0821138665841023	1.4908606504426	0.135998084272613	0.41304775914475
**3110083C13Rik**	46.2381632597361	0.822105083316011	0.551464098980868	1.49076809321822	0.136022391457899	0.41304775914475
**Smpd1**	5616.5663476402	0.115630758041156	0.077575855136383	1.49055086583151	0.136079452435455	0.413141412721669
**1810030O07Rik**	6568.92347117406	0.105779243884565	0.0709723582346528	1.49042876009322	0.136111535107848	0.413159209919931
**Zfyve27**	6702.29571415683	0.124460285538408	0.0835292878006636	1.49001971422794	0.136219052412396	0.413326325604107
**Galnt7**	3646.57599852691	0.0904324289532953	0.0606897115871191	1.49007841013515	0.136203620223064	0.413326325604107
**D430042O09Rik**	991.894144446784	0.17603176421471	0.118171616221636	1.48962813442913	0.136322040189823	0.413559181222765
**Eml2**	257.409767551576	0.461868377531067	0.310095629874814	1.48943852487545	0.136371930203513	0.413630895903129
**Eef1a1-ps1**	480.94196555437	0.165752356786208	0.111385666994692	1.48809412609709	0.136726072337211	0.414625235145771
**Sfxn2**	1567.01774468025	-0.160936105547566	0.108174268535274	-1.48774849811059	0.136817232387041	0.414821845434407
**Gm48570**	25.5935002913879	-0.569451387131979	0.382799619176657	-1.48759653511877	0.136857327758654	0.414863584742806
**4933439K11Rik**	22.2158331219606	-1.50160350758736	1.00954551974883	-1.48740544949468	0.136907758486529	0.414936632135458
**Mrps17**	4539.7104268911	0.129473447701676	0.0870602000695185	1.48717149280946	0.136969523133435	0.41504399538722
**Ccr7**	96220.374085036	-0.0912706483526332	0.0613915000949598	-1.48669845518446	0.137094470899814	0.415342737544388
**Mrps33**	2122.71814354723	0.183201083811348	0.12327576922794	1.48610781306588	0.137250606023221	0.415416403977697
**Tulp4**	2337.87067106511	-0.171250626358793	0.115213343585715	-1.4863784092108	0.137179057431201	0.415416403977697
**Bbs7**	131.96279998412	0.490090758549366	0.329737560241627	1.48630552791812	0.137198325213954	0.415416403977697
**4930580E04Rik**	17.8136195665557	1.24291467596357	0.836185036095657	1.48641104816588	0.137170429277704	0.415416403977697

**Gm45140**	37.9649809859822	-1.1499106381045	0.773730415051854	-1.48619030056797	0.137228792373012	0.415416403977697
**Sumo3**	11570.6079066679	0.0978870006121926	0.0659181090949801	1.48497889208486	0.137549415675649	0.416009032429904
**Pla2g7**	86.1740333192479	0.456532089560017	0.307518936053934	1.48456578127582	0.13765888562427	0.416009032429904
**Tapbp**	64761.4656137834	0.0757720952765898	0.0510455461081766	1.48440169718259	0.137702384788407	0.416009032429904
**Nop56**	25388.7757175742	0.156553419746069	0.105474898630869	1.48427181991386	0.137736823138085	0.416009032429904
**Bcl11b**	19284.7279198609	0.331050842978749	0.222947133195152	1.48488495112862	0.137574303126679	0.416009032429904
**Gm9923**	582.154849440709	0.14616683960356	0.0984482033826487	1.48470804525948	0.137621179609006	0.416009032429904
**1110038F14Rik**	3271.06973484937	0.148290884078299	0.0998592093182373	1.4849995818184	0.13754393488295	0.416009032429904
**Rpl13-ps1**	17.2637282143852	-0.819103694561464	0.551590125243506	-1.48498614655195	0.137547493919294	0.416009032429904
**Trmt61b**	421.638334992939	0.347422167129154	0.234005696823839	1.48467397095335	0.137630210026197	0.416009032429904
**4933433G15Rik**	38.3333817047964	-0.782959099403624	0.527480875638697	-1.48433646709103	0.137719680416378	0.416009032429904
**Gm44045**	27.4721674368946	-1.01933253780334	0.686674817270991	-1.48444724076879	0.137690309990641	0.416009032429904
**Tmem38a**	217.799506280991	-0.273383232723213	0.184265017649752	-1.48364153006435	0.137904045553106	0.416434274775833
**Spcs2**	19725.4283592995	0.129020612008898	0.0870077408139369	1.48286360273167	0.13811065343508	0.416898384325012
**Tdp2**	3894.7049452235	0.138981445785923	0.093724230961992	1.48287635288557	0.138107265230008	0.416898384325012
**Sra1**	6527.35493845233	0.160559204388428	0.108289621228155	1.48268322086145	0.138158594682951	0.416963220919224
**Acss1**	18640.9056284308	0.115967074407459	0.0782250408952604	1.48248020173915	0.138212567717261	0.417046233152218
**Sptb**	30.8141086919334	-1.36611185947184	0.92174758667324	-1.48208889203865	0.1383166440122	0.417280367547431
**Nubp2**	10697.1067429847	0.134228743385261	0.0905829822014822	1.48183179801586	0.138385055946258	0.417406838925189
**Rad23b**	2740.48330053432	-0.107784284780359	0.072759633582457	-1.48137476061104	0.138506736567722	0.417534082283833
**Zfp410**	3644.06739691063	0.124907902336418	0.0843171418040544	1.48140579322166	0.138498471907019	0.417534082283833
**D330050I16Rik**	235.329495387631	-0.450100554735063	0.30381663763298	-1.48148751247389	0.138476710106248	0.417534082283833
**Gm10571**	21.8506648636729	-1.01594386485483	0.685865104709302	-1.48125900833726	0.138537567273326	0.417547139924598
**Trabd**	6945.54994651072	0.190750608961506	0.128824730112043	1.48069868879682	0.138686883549763	0.417917235435764
**Nsmce4a**	7059.73148709408	0.761896966137405	0.514663433591284	1.4803790524245	0.138772117087509	0.418094120257721
**Ergic3**	5172.83417185042	0.111007566705508	0.0750138781516045	1.47982705921642	0.138919405385443	0.41845786095554
**Fam219b**	3401.8741475388	0.182579412030591	0.123411112348525	1.47944061564707	0.139022591713214	0.418608633217104
**Exosc2**	5279.13055200366	0.101923643220501	0.0688923509084976	1.47946240586093	0.139016771824008	0.418608633217104
**Rpl18-ps1**	143.43173355209	0.291755268420666	0.197219743912333	1.47934107728258	0.139049179531509	0.418608697340082
**Polm**	2367.73447674092	0.174627015028741	0.118060099679771	1.47913660502069	0.139103808658532	0.418693163903054
**Erbb3**	454.254692538235	-0.40136655512419	0.271402088131314	-1.47886318004302	0.139176885798094	0.418753139110287
**Elovl7**	868.397037424117	0.235432445905716	0.15919459340722	1.4788972468649	0.139167779290757	0.418753139110287
**Traf2**	9776.49221434175	0.112711919844624	0.0762213458020807	1.47874481431089	0.139208530073899	0.41876838647851
**Fn3krp**	56.9744748260207	-0.632505832783671	0.427806880466011	-1.47848447901231	0.139278148440756	0.418897840233058
**Plod1**	985.31963201693	0.214220821478077	0.144931293978606	1.47808534373328	0.139384936479155	0.419068786319114
**Inpp5d**	18730.7656133255	0.0816542907775294	0.0552437381785389	1.47807323453811	0.139388177260803	0.419068786319114
**Slc25a19**	3821.01936840079	0.134914330279017	0.0912833378802434	1.47797323599203	0.13941494207047	0.419069294583058
**Gm27177**	96.0174700033684	-0.421031504375433	0.284951935958986	-1.47755270712052	0.139527540761765	0.419327762689897
**Trex1**	1607.53200709491	-0.286002304166692	0.193648747737969	-1.47691274799096	0.139699027290882	0.419683045380826
**Slc5a3**	480.225774771179	-0.322458988749982	0.218320677372448	-1.47699701480808	0.13967643748029	0.419683045380826
**Thoc1**	5322.05834072736	0.119881740293977	0.0812040794661424	1.47630194298257	0.139862852786853	0.419935017409003
**Cplane2**	326.726554547668	-0.266423390321656	0.180454330039381	-1.47640342164975	0.139835624711382	0.419935017409003
**Gm36888**	21.0301599474364	1.56932738968742	1.06298343539595	1.47634228100917	0.139852029069578	0.419935017409003
**Chml**	80.8746177628807	-0.61001999387474	0.413272404964482	-1.47607240780368	0.13992445517659	0.42003993907745
**Lsm14a**	8466.24851345604	-0.082176836846269	0.0556838233517212	-1.47577576214922	0.14000409952219	0.420198970699265
**Nsun3**	1463.47183078256	0.177476252027244	0.120304247799434	1.47522847508364	0.140151128379502	0.420516466264052
**Trav7-4**	651.858243578387	0.225046617643889	0.152555015857072	1.47518333880765	0.140163259555677	0.420516466264052
**Pdcd2l**	2264.58148234413	0.113037112255202	0.0766519319948162	1.47468053724785	0.140298451033498	0.420676221238844
**C130026I21Rik**	247.506966081076	-0.25920843098378	0.175759746734622	-1.47478837333076	0.140269448009091	0.420676221238844
**Gstp3**	2302.51814990194	-0.130622159849139	0.0885821295667624	-1.47458816454273	0.140323298777138	0.420676221238844
**Gm5822**	32.7791639032255	0.496832327491889	0.336923385556971	1.47461514632049	0.140316040479134	0.420676221238844
**Ap3b1**	7907.1945152932	-0.133943715338114	0.0908564949813405	-1.47423379435474	0.1404186537878	0.420712166636337
**Ccdc136**	22.7316040379659	1.67037531236008	1.13310642526194	1.47415571487377	0.140439670351093	0.420712166636337
**Tubb4a**	83.5654010531571	-0.46944409515098	0.318394694978559	-1.47440928682117	0.1403714255127	0.420712166636337
**Gt(ROSA)26Sor**	1379.57817763884	-0.236029465486338	0.16011259986213	-1.47414672979878	0.140442089008512	0.420712166636337
**1700029J07Rik**	173.231884671807	-0.400724390086809	0.271886920690294	-1.47386416775551	0.140518167133176	0.42086005693166

**Shoc2**	3872.25793952002	-0.107261264047052	0.0727834692548406	-1.47370364651749	0.140561400622212	0.420909538123183
**C1qtnf4**	92.01868478653	-0.354668892279323	0.240686811788499	-1.47357011231253	0.140597373436121	0.42093726263214
**Rufy1**	1971.40420691539	0.206440520150994	0.140111378767428	1.4734029596102	0.140642412722642	0.420992115742908
**Gm37472**	91.2542569874273	-0.532729396888635	0.361693531797191	-1.47287510020319	0.140784717251948	0.421338041735792
**Wdr46-ps**	50.6082461482515	-0.585488959503851	0.397565875930323	-1.4726841385312	0.140836225475896	0.421412154353152
**Cops8**	6292.20970145291	0.10749145614568	0.0730148182467521	1.4721868618835	0.140970424239942	0.421653563923457
**Gm11723**	20.9855330601566	-0.683219249671288	0.464053888277737	-1.47228429053132	0.14094412368223	0.421653563923457
**Vcp-rs**	5372.77576943505	0.225010349318798	0.152910100465135	1.4715205119501	0.141150404465917	0.422111771336012
**BC147527**	7556.65030926905	-0.143870455289387	0.097820146152161	-1.47076508212934	0.141354658604665	0.422642382473188
**Apbb2**	379.79216196416	-0.298596337486719	0.203060442425691	-1.47048008917832	0.141431774367267	0.422792727889398
**Ccdc141**	21.4828800984879	-0.936017281303543	0.63669102969302	-1.47012795477085	0.141527102468356	0.422927437400219
**Gm44722**	25.5390472959893	0.858499626727902	0.583967531828902	1.47011534021285	0.141530518335429	0.422927437400219
**BC048403**	317.112884513559	0.206796810843439	0.140701309999397	1.46975753704302	0.141627433414058	0.423077453150178
**Gm50037**	39.0532875935702	-0.517047301234357	0.351797055403482	-1.46973174815618	0.141634420598286	0.423077453150178
**Dhdh**	77.8108993254049	0.604589730394126	0.411506977816915	1.46920893930289	0.14177612639171	0.423148715089982
**Cd96**	27395.1738456876	-0.102538948723504	0.0697859242974993	-1.4693356827428	0.141741762959864	0.423148715089982
**6530409C15Rik**	37.727581830975	-0.757971053498852	0.515925463047157	-1.46914837081722	0.141792550337952	0.423148715089982
**Gm5915**	100.500086414192	-0.439659645440176	0.29920099588162	-1.46944579560868	0.141711913702253	0.423148715089982
**BC051537**	132.502705689475	-0.590717224562972	0.402034097516707	-1.46932120487224	0.141745687962117	0.423148715089982
**Mcub**	380.250984653981	0.306201424004714	0.208461896389991	1.46886039754657	0.141870658095207	0.423301639766366
**Noa1**	3029.78997887418	0.151279865202212	0.103006076655928	1.46864990992263	0.141927770118295	0.42339187310753
**Tlr3**	92.2646764539775	0.461962989783178	0.31468815870695	1.46800245576885	0.142103555840184	0.423836026760133
**Rbm44**	43.9946167131707	0.709058492863593	0.483114809618096	1.46768113654828	0.142190857019247	0.424016149517841
**Clk1**	29812.9312576489	-0.144782027365448	0.0986616932092755	-1.46745938221783	0.142251130822321	0.424115623153702
**Fam111a**	3672.31329288827	-0.140726708432221	0.0959073602297999	-1.46731917232453	0.142289250611202	0.424149020408762
**Crk**	5436.7186334785	-0.158521475009179	0.108061579078573	-1.4669550117708	0.142388293985892	0.424363977366313
**1300002E11Rik**	1431.09476044646	0.163249367580209	0.111311220416028	1.46660298009545	0.142484088890923	0.424569173346118
**Mylip**	3137.89349052404	0.131718865126412	0.0898546444108063	1.46591048231416	0.142672675865408	0.42505073806225
**Il15**	45.047570144675	0.682944704970169	0.465983649133378	1.46559800164724	0.142757836007153	0.42522404952074
**Trim26**	10776.3388382141	0.143494593799399	0.0979332257839668	1.46522891133941	0.142858474186838	0.425239094121836
**Il10ra**	4700.57891966466	-0.17897558813626	0.122148533188865	-1.46522912280518	0.142858416511842	0.425239094121836
**Chst11**	359.592514954424	0.473663957778828	0.323266048164616	1.46524499083066	0.142854088730841	0.425239094121836
**Ccdc85b**	1348.47915278356	-0.153859222811223	0.105010200829065	-1.46518358784661	0.14287083609069	0.425239094121836
**Vdac3**	5674.41395800859	0.1423763439475	0.0972046241137853	1.46470752030107	0.143000732290245	0.425384658995164
**Fbln2**	19.0271689739745	1.32153748433786	0.902243827530631	1.46472322005771	0.142996447128486	0.425384658995164
**Fam177a**	2127.55496406722	-0.170799099347919	0.116594957114127	-1.46489268125666	0.142950199903194	0.425384658995164
**Gm43331**	59.4231053843269	-0.527612283850691	0.36025361076854	-1.46455793385421	0.1430415660262	0.425425812735906
**Grasp**	325.864020284453	1.00740107332189	0.687910491648925	1.46443626831035	0.143074784581183	0.425444306594773
**Pik3cb**	1107.79344594162	0.17257120895159	0.117919035357765	1.46347202067938	0.143338264185832	0.426066973924948
**Tcf7l1**	160.486976595754	-0.309918573910401	0.211758323985873	-1.46354848336955	0.143317357261112	0.426066973924948
**Gm37706**	23.8888217096043	-0.735621937428655	0.502721476167863	-1.46327931529033	0.143390965322148	0.426143236624121
**Msmo1**	2610.25643170961	0.160636816053474	0.109800303315656	1.46299064030518	0.143469940066223	0.42627622406223
**Gm38376**	69.5854640289656	-0.878097444839301	0.600273035286393	-1.46283006768804	0.143513883428341	0.42627622406223
**Gm32031**	51.9748894482034	-0.398927862917582	0.272711677552725	-1.46281914473741	0.143516873050081	0.42627622406223
**Gprc5c**	103.830294958016	0.846459863370057	0.578841839169829	1.46233358767577	0.14364981873458	0.426590688616805
**Mxra8**	218.844229851841	-0.284986135054327	0.194993759405852	-1.46151413215829	0.143874400108692	0.427177109951509
**Mrps18a**	3905.32584284133	0.127598553431783	0.0873393934182522	1.46095076274159	0.144028954193745	0.427480016774823
**Gm43984**	117.244971531091	-0.665162023009319	0.455295894493164	-1.46094447820527	0.144030679003461	0.427480016774823
**Lrpprc**	6619.52364120388	0.0907457121812021	0.062123045383747	1.46074152708785	0.144086388056136	0.427564824218557
**Atp8a2**	546.517937222107	0.319160278035372	0.218543691851954	1.46039574663897	0.144181341082913	0.427766031151093
**Hes6**	7422.35148116631	0.126282888923815	0.0865149421642211	1.45966564578067	0.144381987961925	0.428280682288413
**Cpt1a**	6540.89089346523	0.190634753494568	0.130612382995248	1.45954578825427	0.144414947740297	0.428297822613188
**Acp5**	49877.9096191224	0.123382656603024	0.0845707450066584	1.45892833973864	0.14458483209691	0.428364599674414
**Adi1**	4495.10055356891	0.120933044867754	0.0828707403118396	1.45929726719814	0.144483307245994	0.428364599674414
**Klhdc2**	5319.392917487	0.31522682134052	0.216072205427953	1.45889574605943	0.144593804152865	0.428364599674414
**Paox**	807.921749950208	0.188426293709222	0.129164254595976	1.45881145134633	0.14461700992123	0.428364599674414

**Dars2**	1694.66845381733	0.136519635374579	0.09358482627961	1.45877959923428	0.144625779336649	0.428364599674414
**Slc38a7**	1655.46963475666	0.155600168117831	0.106679584128716	1.45857494091924	0.144682134890349	0.428364599674414
**Sgms1**	5790.28476227971	-0.174378201678933	0.119549189454729	-1.45863140080064	0.144666586185206	0.428364599674414
**Cstad**	26.5903504842037	-0.836572024979558	0.573481037938663	-1.4587614404594	0.144630778931308	0.428364599674414
**Zfp708**	392.085735238401	-0.39161825008652	0.268456732616675	-1.45877604286314	0.144626758489975	0.428364599674414
**Ssh2**	33884.7494960323	-0.152611704827933	0.104651244875368	-1.45828848008145	0.144761044179891	0.428517710528376
**Ccnj**	313.001560941988	0.307475772401183	0.210917299392022	1.45780252870435	0.144894981091978	0.428717492217312
**Smad7**	2369.23519343928	0.304272003613968	0.208703148715764	1.4579176475596	0.1448632436935	0.428717492217312
**Pym1**	913.997034521391	-0.148416164394403	0.101811984568138	-1.45774748448279	0.144910158250264	0.428717492217312
**Cep63**	3028.87167077885	-0.0958416970180584	0.0657623808208008	-1.45739396630457	0.145007661660189	0.428895384472969
**Zfp664**	2735.56819562474	-0.155947090293266	0.107008614389999	-1.45733221463619	0.145024726056482	0.428895384472969
**Unkl**	3022.60555289531	-0.228977556692175	0.157351231685414	-1.45520028181261	0.145613822910849	0.430290701351334
**Gdap2**	3051.08556242688	0.109219558982238	0.0750677657701964	1.45494617911754	0.145684162029277	0.430290701351334
**Bag1**	6688.42149845674	0.28115859279348	0.193229421401885	1.45505063749437	0.145655243366777	0.430290701351334
**Zfp248**	232.811119378123	-0.3312658247622	0.227681317659132	-1.45495391614936	0.145682019928637	0.430290701351334
**Phpt1**	1296.31867513368	0.113759448776442	0.0781602019814926	1.455465133053	0.145540536125031	0.430290701351334
**Gm4189**	19.1608004386915	0.836799943631118	0.575146544384663	1.45493344574711	0.145687687485522	0.430290701351334
**Gm29243**	44.7895125881484	0.495748755121614	0.340641685611732	1.45533789921024	0.145575739379557	0.430290701351334
**Gm8979**	260.237983834312	-0.267718811268473	0.184039738358909	-1.45467937335564	0.145758045523775	0.430417826708794
**Rsc1a1**	39.3744730714288	1.06383393796383	0.731441306802142	1.45443513795373	0.145825704008474	0.430456279142286
**Shtn1**	58.8264465988517	0.500293177498739	0.343968629431152	1.45447327079249	0.145815138804558	0.430456279142286
**Vmp1**	7866.44229360356	0.247369326210221	0.170105444344032	1.45421169301275	0.145887624142008	0.430477770338132
**Aldh4a1**	5676.68896750741	0.149743877169087	0.102972301307737	1.45421511675816	0.145886675216109	0.430477770338132
**Txndc16**	1309.53339377501	-0.213648033420233	0.14693903250132	-1.45399101779382	0.145948796492419	0.430497039747482
**Lin52**	2139.33635484145	0.157137626600711	0.10807274802687	1.45399862101815	0.145946688511473	0.430497039747482
**Acsf3**	290.849461049567	-0.317608829890619	0.218468976634111	-1.45379373668485	0.146003500489939	0.430537276496592
**Ndufv3**	3555.60211363689	-0.0967511384193459	0.0665530438110882	-1.45374475574664	0.146017084825651	0.430537276496592
**BC049352**	26.3880069784908	-1.01689035348904	0.699610497917182	-1.45350928340332	0.146082404047972	0.430649287293527
**Mgmt**	397.733095699937	-0.207428423333685	0.142745268728684	-1.45313694233849	0.146185735819428	0.430873295792503
**Mbd6**	415.940959800728	0.160969194701536	0.110799065790861	1.45280281519137	0.146278510106158	0.430985505561469
**Nr4a3**	278.662019615341	-0.377526241109507	0.259850579399385	-1.45285895448883	0.146262919229489	0.430985505561469
**Clock**	1988.8377989063	-0.240337901346165	0.165452603540374	-1.45260876047513	0.146332412331195	0.431063716788677
**Map3k14**	1804.66144051908	-0.168360110063547	0.115938876259087	-1.45214543642217	0.1464611704694	0.431362366932025
**Klrg1**	990.734075857101	1.55371386796751	1.07022436029976	1.45176462581391	0.146567062935475	0.431432320320246
**Gm37474**	451.557283778066	-0.28596669125775	0.196964393680744	-1.45186998479161	0.146537759778456	0.431432320320246
**Gm37625**	21.3065991529554	-1.00117006348968	0.689577472922127	-1.45186016481536	0.146540490787771	0.431432320320246
**Slc5a6**	2033.60637386979	0.151547455593106	0.10440930159558	1.45147466056341	0.146647733192651	0.431589154261598
**Ptpn21**	517.084616619461	-0.293405570996638	0.202193633412003	-1.45111181813908	0.146748726198698	0.431743782034608
**Gm7665**	195.64827834178	0.227570736265921	0.156827550350621	1.4510890194812	0.14675507371818	0.431743782034608
**Atp5g1**	6046.74176743999	0.107928415471028	0.0744068968329022	1.45051628363707	0.14691460166164	0.431916136485112
**Tctex1d2**	70.2753094954735	0.509258767126319	0.351027835372057	1.45076462835647	0.146845412269195	0.431916136485112
**Cib1**	5240.1819809271	0.146963273362189	0.101326958929829	1.45038669781815	0.146950714456856	0.431916136485112
**Amz1**	251.782260240824	0.400637994423938	0.276206727650029	1.45050049226741	0.146919002015332	0.431916136485112
**Mpst**	123.036614287219	-0.428546353551183	0.295467850701405	-1.45039926521233	0.146947211895758	0.431916136485112
**Ubac1**	4200.68301184589	0.118368777317739	0.0816421132249472	1.44984950342478	0.147100491444024	0.43227572616253
**6330537M06Rik**	332.038243449609	-0.365789696439215	0.252315573193069	-1.44973095322704	0.147133560542827	0.432292282952328
**Def8**	2663.11094793904	-0.145934056483997	0.100691568113558	-1.44931754682195	0.147248923063098	0.432550574329836
**D830050J10Rik**	72.2789121897278	-0.397838643120514	0.274526792741625	-1.44917965619096	0.14728741730756	0.432583007365372
**Gm50010**	204.054360840116	-0.319664959141916	0.220598362922624	-1.44908128467861	0.147314883897123	0.432583046136222
**Gbp8**	12125.2285527656	0.124804461297255	0.0861459563687429	1.44875588545372	0.147405767439417	0.432769271016004
**Glb1l**	551.079057320469	-0.24874190112524	0.171719644449391	-1.44853491819655	0.147467507711896	0.432775588745739
**Snrnp25**	1301.9002698704	0.157312165786362	0.108614366905356	1.44835504057618	0.147517781757528	0.432775588745739
**Tmem240**	67.8892258342338	0.794236837648058	0.54828509546215	1.44858367338728	0.147453883366445	0.432775588745739
**Trav11**	68.7741820887216	0.430383889481601	0.297145897811245	1.44839249894337	0.147507311428324	0.432775588745739
**Coasy**	3712.77217433992	0.103893189417321	0.0717925698122442	1.44713010955072	0.147860486493953	0.433538832468406
**Slu7**	10100.4388138076	0.121494700988582	0.0839509740533146	1.44721014090229	0.147838077185813	0.433538832468406

**Gm47798**	35.6948924908596	-0.816936179820324	0.564503691132542	-1.44717597537996	0.147847643440702	0.433538832468406
**1700056N10Rik**	184.913051852293	-0.311347395412661	0.215320190103872	-1.44597399464706	0.148184495959625	0.434408019132244
**Copa**	30528.7541808108	0.10230605247061	0.0707665665889384	1.44568342653777	0.148266014976198	0.434566145457581
**Fam207a**	4310.72703236181	0.148529623246357	0.102771772590875	1.44523753460632	0.148391176772712	0.434852105710921
**Cbx4**	10112.0990168343	0.324335807507129	0.224449608244594	1.44502728271053	0.148450222437602	0.434906626487177
**Tmem250-ps**	9639.90437026049	0.104390098017736	0.0722435456907225	1.44497473123252	0.14846498342797	0.434906626487177
**Gm12503**	23.7114477727225	-1.24062632831459	0.858851287397106	-1.44451821464286	0.148593259853729	0.435201486155244
**Zwint**	6456.27245594393	0.110454888687579	0.076480636509105	1.4442203115612	0.148677013153615	0.435365860669301
**Aldh1b1**	1148.84985277489	0.118952552948334	0.0823792789000201	1.44396205619499	0.148749648950009	0.435497625140338
**Gm13212**	892.918346318514	-0.152170715604378	0.105397259714936	-1.44378246660253	0.148800175518213	0.435564623005848
**Scyl3**	4463.47320360394	-0.088407305948581	0.0612595272326728	-1.44316010818688	0.14897537411054	0.435672785794316
**Sap30**	396.453489706952	0.320815221205662	0.222276001764921	1.44331920071586	0.148930573387675	0.435672785794316
**Dhrs11**	969.431569122334	0.155415511127625	0.107679839408504	1.44331113401857	0.148932844735594	0.435672785794316
**2510046G10Rik**	246.830783276656	-0.290771385832264	0.201454673820968	-1.44335884751262	0.148919410384359	0.435672785794316
**Slc35g2**	49.2311728598701	-0.570242860109334	0.395115807822235	-1.44322967803376	0.148955781859423	0.435672785794316
**Pard6g**	387.872767335404	0.325166729904384	0.225339916343849	1.44300546117275	0.149018932829037	0.435719303133632
**Uba1**	23842.1532767688	0.129140839130487	0.0895095704090651	1.4427601265463	0.14908805505806	0.43584053498142
**Polr1e**	1950.65594995397	0.11345667059953	0.0786481642101491	1.44258510976013	0.149137380420379	0.435903858891471
**Gm42876**	90.6864966430878	-0.59438632352394	0.41211026011058	-1.44229926079601	0.149217968641262	0.436058518523808
**Fbln1**	68.1005852632376	-1.0875712925307	0.7541555456193	-1.44210474728738	0.149272826033667	0.436096222275393
**Osbp**	849.954591057145	-0.267503772673979	0.185501492742348	-1.44205725096524	0.149286223453289	0.436096222275393
**Arl6ip5**	13459.6357832739	0.144821872497536	0.100442572216943	1.4418375525543	0.149348206334742	0.436173491301939
**Gm2573**	68.1349990434186	-0.44246625592313	0.306891592599462	-1.44176727741321	0.149368037003571	0.436173491301939
**Mapk12**	54.1644001944868	0.900845901307288	0.624889623676809	1.44160803312235	0.149412980966452	0.436223891252247
**Cdk14**	68.01778873944	1.00059393570348	0.694289062995264	1.44117772990226	0.14953447826877	0.436497735102077
**Nedd8**	6038.33094483511	0.117638221338161	0.0816544319029495	1.4406838501795	0.149674019468861	0.436824138686951
**Gm47603**	42.9652483241996	-0.601216045210647	0.417406075327378	-1.44036246894371	0.149764876141073	0.437008361732387
**Itk**	79746.6150670933	0.128770807173679	0.0894385479583509	1.43976853508002	0.149932896021903	0.437417634256087
**Morn1**	114.609696253391	0.496194484724335	0.344699143574337	1.43950019596532	0.150008854483381	0.437429582185626
**Dync1li2**	3417.59433835423	0.1043032148028	0.0724599674870987	1.43945986204549	0.150020274299113	0.437429582185626
**Zswim9**	505.087706664887	-0.247653688172059	0.172032418928865	-1.43957568994286	0.14998748151875	0.437429582185626
**Gpx3**	31.8306825627368	-0.718639985844952	0.499414996237203	-1.43896356989574	0.1501608446725	0.43771671576289
**Gm38077**	66.7283036649454	-0.705564238494658	0.49034426658472	-1.43891605669004	0.150174307635602	0.43771671576289
**Max**	6067.36674038967	0.25066418901681	0.17422143184963	1.43876781608107	0.150216317828605	0.43775818753936
**Qdpr**	6567.13651833095	0.118929168114232	0.0826812860675784	1.43840491325966	0.150319199383879	0.437910086159529
**Carmil2**	8115.77681434087	-0.158524516781318	0.110209851643816	-1.43838789742362	0.150324024626318	0.437910086159529
**Gm6035**	38.4603128975256	0.535311582374082	0.372190685386369	1.43827237862866	0.150356785833208	0.437924560882414
**Snhg10**	58.2600353912431	-0.451685479280096	0.314118979036229	-1.43794393024562	0.150449963756263	0.438114965608471
**Klrb1c**	475.242258712417	1.21124214799878	0.842642226008112	1.43743348079867	0.150594861149357	0.438455880844497
**Nfkbiz**	3055.45008112182	0.258598994301639	0.179917103833132	1.43732301594563	0.150626231962153	0.438466199340042
**Gpalpp1**	1957.05316116758	-0.181562137701306	0.126341285692331	-1.43707685659817	0.150696156449092	0.438588721309263
**Gm7666**	694.287613088184	-0.167265442084207	0.11643734564563	-1.43652744020179	0.15085231394253	0.438811016335454
**Gm37766**	113.332780568578	-0.430550395064863	0.299718859737528	-1.43651419013774	0.150856081455173	0.438811016335454
**Gm2238**	24.3109004996635	-0.889594444363499	0.619240017509767	-1.43659069053861	0.150834330380389	0.438811016335454
**Dnase2a**	839.361676163613	0.16846720785438	0.117358846182242	1.4354879358031	0.151148103720963	0.439579304232412
**Crot**	7842.87187985509	0.129975163140615	0.0905766711044973	1.43497394589236	0.151294522134419	0.439874483280531
**Stag2**	7463.18070093989	-0.140721589956318	0.0980682226793134	-1.43493566123333	0.151305432466871	0.439874483280531
**Dhrs7b**	2544.90604749376	0.108762690043109	0.075824636225007	1.43439778227698	0.151458780168931	0.440239070120243
**Smn1**	1748.97666232117	0.169793208076757	0.118388173742585	1.434207511689	0.151513054088756	0.440315601858451
**Ppp4r3a**	8992.08900624556	-0.122253508081021	0.0852569947113005	-1.43394109181304	0.15158907417852	0.440455290767276
**Spry1**	100.735069235815	0.666557962168039	0.464929161909785	1.43367638938806	0.151664632979082	0.4405123726915
**Eif5a**	79827.1905517799	0.120740767148503	0.084213981569722	1.43373778199218	0.151647106023454	0.4405123726915
**Slc29a3**	3201.87436235213	-0.190954792332336	0.133208744669084	-1.4335004267678	0.151714877041264	0.440577095012753
**Wwp2**	10281.7144428474	0.108070293254755	0.0753972714969937	1.43334488250101	0.151759301436465	0.440624896394762
**Nelfb**	17836.9105525578	0.0826497202478191	0.0576805210455731	1.43288789264781	0.151889877844021	0.440626175045984
**Rpp25l**	1077.82517562186	0.229904805972948	0.160439073192425	1.43297266306948	0.151865649801652	0.440626175045984

Gm20604	991.730224565543	0.216571892629657	0.151129463211472	1.43302231098785	0.151851461408989	0.440626175045984
Frat2	744.165751157123	0.850360712825952	0.593377677552776	1.43308510750359	0.151833516853191	0.440626175045984
Gm43010	26.2568634122548	-1.28850021214254	0.899254354024772	-1.43285401552479	0.151899561010966	0.440626175045984
1110012L19Rik	1327.01863404185	0.184366114975103	0.128715600306532	1.43235252398343	0.152042958344688	0.44096095980807
Ankrd37	344.296084830248	-0.275434847371883	0.192373473599741	-1.43177145069887	0.1522092402842	0.441361979970214
Sh3bp5	16007.4735550831	-0.0944768634587093	0.0659941486037311	-1.43159454978358	0.152259890339238	0.441427615837237
Gm45292	94.6597657465807	-0.479788443615677	0.335194174161847	-1.43137464967996	0.152322869754973	0.44152896591779
Polr2m	19171.2716295649	0.126445650147531	0.0883514943874517	1.43116594715448	0.152382660520757	0.441621037672534
Ftsj1	2237.57689572285	0.107364006632009	0.075034108207828	1.43086936323191	0.152467658978229	0.441786116682596
Dnajc8	9307.59587916052	0.15139251894554	0.105841273914093	1.43037317434803	0.152609943214275	0.442064991103722
Gm43178	46.9981606693831	-0.566361399741027	0.395963312252781	-1.43033806975396	0.152620013429222	0.442064991103722
Wnk1	9702.54567587749	-0.213742281652078	0.149448838686095	-1.43020369734037	0.152658564597264	0.442095387310988
Pkn2	4114.64004221554	-0.154993288608991	0.108405067438462	-1.42976054783579	0.152785755790849	0.442180324274958
Psmc6	4963.04157760557	0.103214750532429	0.0721840032806321	1.42988398871641	0.152750318129638	0.442180324274958
Rgs18	136.510951785888	0.574689679310278	0.401978583880653	1.42965247989655	0.152816785291514	0.442180324274958
Camkk2	1707.96270812995	-0.119366178519276	0.08348459231617	-1.42979890309839	0.152774744013591	0.442180324274958
Cox6b1	5218.613206703	0.119167421137342	0.0833564366707976	1.42961270775015	0.152828206257626	0.442180324274958
Pcmt1	8562.05698135545	0.116181838107716	0.0812847578606262	1.42931886820559	0.152912605319234	0.442259677638596
Uap1	3040.15820790207	0.100237926281401	0.0701311570450267	1.42929235028939	0.152920223761886	0.442259677638596
Foxj3	2598.06080122224	-0.237478641549463	0.166183318888229	-1.4290161198982	0.15299960031351	0.442259677638596
Cep131	466.341977764433	0.262027671448656	0.183373202188436	1.4289310996456	0.153024037728351	0.442259677638596
Ahnak	9589.11693524588	-0.226099873095288	0.158204711481196	-1.42916017467761	0.152958201350944	0.442259677638596
Gm13868	34.5184566918418	-0.690157124647478	0.482977542474433	-1.42896317934702	0.15301481669453	0.442259677638596
Slc39a6	3557.7972805912	0.106865892465996	0.0748044979068852	1.42860249659079	0.153118516188382	0.442451578870408
1700026D11Rik	74.5312311067442	-0.583219069213207	0.408281005180719	-1.42847465792599	0.153155283767415	0.44247667882713
Gm20540	55.344378445071	-0.617170260558301	0.432344512802974	-1.42749645776019	0.153436845473939	0.443208867852038
Phf11	283.714068686521	-0.314960451561561	0.220661580607899	-1.42734612293576	0.153480152195169	0.443227189025653
Gm6900	36.508166434221	0.53445731864584	0.374458854854228	1.42727915688867	0.153499445993082	0.443227189025653
Osbpl3	748.330985979983	0.735959084064878	0.515714914534599	1.42706573597747	0.153560947666105	0.443323534696741
Rtca	2056.86212336893	0.14295237216574	0.100183620933908	1.4269036278899	0.153607675001194	0.443348138493138
Pagr1b	20.0382378741958	1.20594651223101	0.845186359424763	1.42684095499575	0.153625743236183	0.443348138493138
Gm28557	174.482472592237	0.680326079388804	0.476894748762803	1.42657490180749	0.153702462830181	0.443488318405999
Ndufc2	1322.60830984126	0.17921650499965	0.125668277436345	1.42610775492191	0.153837240644486	0.443633492100141
Gm31793	20.7906628440549	1.31800274622371	0.924151633097264	1.42617585580244	0.153817587083038	0.443633492100141
Gm36264	16.8748865989648	-1.06070079160399	0.743768478544195	-1.42611689282684	0.153834603381568	0.443633492100141
8430406P12Rik	33.8916135309037	-0.713865821625391	0.500651481707874	-1.42587378187752	0.153904778598918	0.44374704416892
Tmtc4	1336.74790111166	0.126049425265337	0.0884242426491499	1.42550754735301	0.154010539999928	0.443970742550103
Aaas	5566.16273577433	0.0932664465202617	0.0654355729784303	1.42531718261266	0.154065535444825	0.444048041339693
Sik2	3039.57203133896	-0.17460236937908	0.122515397978664	-1.42514632658245	0.154114907626655	0.444109107438407
Ttc3	11888.2899994177	-0.161448416739492	0.113303954273883	-1.4249142298179	0.154181995773287	0.444181171270825
Kcnj16	64.8269805623457	-0.661576865009728	0.464308527634654	-1.42486477338684	0.154196294145549	0.444181171270825
Kctd4	57.7139601824615	-0.476100225429404	0.334272502519586	-1.42428773482948	0.154363196510071	0.444424860837468
Mppe1	16280.6562495776	-0.109869898906481	0.0771406729550903	-1.42427975667836	0.154365505068444	0.444424860837468
Gm37675	54.447980317056	-0.661750785586465	0.464559591116122	-1.42446910631332	0.154310721921187	0.444424860837468
Cep85l	307.320582241077	-0.418356001541363	0.293777773102816	-1.42405600370232	0.154430260930608	0.444448880636272
2310002F09Rik	28.9193731472238	-0.764031957383115	0.536499785853393	-1.42410486924574	0.154416117098759	0.444448880636272
Tcp1	16917.5340143718	0.107973951369577	0.0758337482359588	1.42382453566205	0.154497271309273	0.444479369001317
Gm37008	19.5405784014118	-0.975074417333574	0.684784164927993	-1.42391496076155	0.154471090465334	0.444479369001317
Odf2	3896.25732375437	0.13159558212038	0.0924649905669358	1.42319359266162	0.154680042521238	0.444910078362461
Ndst2	4164.92924432867	0.0801378142316473	0.0563116383470158	1.42311281617851	0.154703453661733	0.444910078362461
Washc4	7154.47155718832	-0.123804901323199	0.0870102889637216	-1.42287656779095	0.154771940079459	0.445025813837961
Gfm2	3438.5041990235	0.119211910456293	0.0838015293205648	1.4225505360442	0.154866491752127	0.445070239176656
Eif2ak3	1491.59836985201	0.708380568701211	0.497971935920787	1.42253110587721	0.154872128032342	0.445070239176656
Fahd1	161.133733376096	-0.268337944739286	0.188623882073207	-1.42260853604498	0.154849668106017	0.445070239176656
Atg4b	7902.74154959704	0.102062452824572	0.0717688245963215	1.42210010263709	0.154997193010056	0.445267232499446
Pilrb2	45.5149003895322	-0.861946519480714	0.606075726535668	-1.42217627557468	0.154975084195384	0.445267232499446

**Zfp169**	609.611964719521	-0.333496316302341	0.234551188869127	-1.42184875681199	0.155070161871222	0.44539565085761
**Cript**	3691.50684248877	0.172068409854384	0.121073382766065	1.42119106547845	0.155261221383124	0.445683164030949
**Plk1**	307.221747514565	0.480056641018196	0.33776437632785	1.42127670844787	0.155236331960889	0.445683164030949
**Trip4**	2541.8303453179	0.0827426231498869	0.0582237451919308	1.42111475098572	0.155283402331022	0.445683164030949
**1500002C15Rik**	344.308827158316	-0.232918026627397	0.163883200819419	-1.42124406566874	0.155245818195648	0.445683164030949
**Bst1**	51.3218145241523	0.627898710556369	0.441940368363661	1.42077699957857	0.155381599285856	0.445802596494399
**Ube2e2**	37.8340125753924	0.880354998230807	0.619629038847146	1.42077750240492	0.155381453060419	0.445802596494399
**Cdc5l**	6963.02007275629	0.132896019475644	0.0935626627348901	1.4203958672297	0.155492465304531	0.445901345337144
**Blzf1**	1570.56748320546	0.210012926516808	0.147845527329148	1.42048887315513	0.155465405657205	0.445901345337144
**Ctso**	6689.70079028966	-0.145320251671857	0.102311774884642	-1.42036683300341	0.155500913411665	0.445901345337144
**Snx13**	1773.99754417763	-0.121546822419519	0.0856091463436891	-1.41978781018974	0.155669464810447	0.446096349073805
**Gnb5**	219.277595663793	0.323620328238084	0.22794269952997	1.41974421161724	0.155682161804712	0.446096349073805
**Zfp125**	1057.5582055971	-0.188611513999463	0.132846351400421	-1.41977187940192	0.155674104163003	0.446096349073805
**E330011M16Rik**	30.9051682955869	1.3012888033	0.916404159508812	1.41999443127526	0.155609302272647	0.446096349073805
**Ttyh3**	2715.07933454012	0.35993472986424	0.253599634869248	1.41930302876744	0.155810689500993	0.4461823037646
**Rap1gap2**	434.204289243364	0.545733671191046	0.384521911439594	1.41925246638845	0.155825424739265	0.4461823037646
**Ythdf2**	11455.4475213254	0.121007780009443	0.0852575832358994	1.41931984718153	0.155805788396769	0.4461823037646
**Spata2**	3377.50685615528	-0.165229201871838	0.116419082383284	-1.41926219043591	0.155822590807957	0.4461823037646
**Tfcp2**	545.182357408397	0.204879299425019	0.144420630955418	1.4186290287588	0.156007198159673	0.446479768136873
**Gbp7**	13469.3432108111	-0.125515929587735	0.0884784439957959	-1.41860462186359	0.156014317659556	0.446479768136873
**Tram2**	461.66149024495	0.233645479518608	0.164694868240876	1.418656707487	0.155999124563246	0.446479768136873
**Ppa1**	3682.13061111383	0.182242326454628	0.128476599601355	1.41848653389099	0.156048767444225	0.446497263232005
**Depdc1a**	38.3594628870421	1.37339667438231	0.968712618396465	1.41775450045827	0.156262452227835	0.447027498139795
**Mvb12a**	3121.14624456077	0.112448411704295	0.0793245704657103	1.41757353420405	0.156315311523728	0.447097542871553
**Dmbt1**	214.892129853803	-0.424783704697314	0.299704122670344	-1.41734354840541	0.1563825087421	0.447160849820143
**Atl2**	2258.58712178514	-0.131520763406301	0.0927964689136429	-1.41730353477885	0.156394202155157	0.447160849820143
**Gm37289**	75.6495291337608	0.423123227971285	0.298634831079685	1.41685826278711	0.156524371314677	0.447451835777461
**Dnajc16**	832.782032342069	0.230265200924205	0.162542966313885	1.41664204945997	0.156587607942842	0.447551412725442
**Zfp407**	2448.85406547041	-0.169018559762527	0.119324122697681	-1.41646597470279	0.156639119421821	0.447617447357568
**Ddx52**	4744.25244588531	0.104462493616247	0.073770610175446	1.41604486350063	0.156762369554038	0.447644917916452
**Akap5**	186.400664699764	-0.418025354149029	0.295204541751182	-1.41605326147512	0.156759910930346	0.447644917916452
**Wdr46**	8494.08636664072	0.112051126068708	0.0791221659454976	1.41617869947966	0.156723190691814	0.447644917916452
**Agbl3**	96.7422885521647	0.632541500460701	0.446634840805933	1.41623859732776	0.156705658727288	0.447644917916452
**Gm43769**	456.63991894382	-0.505667179601552	0.357365689893511	-1.41498524873004	0.157072821136261	0.448450161740182
**Ulk3**	5326.25518149746	0.165678817839399	0.117123884114495	1.414560480913	0.157197402650209	0.448724542311474
**Gm8783**	105.181901476705	0.283721160964883	0.200611214655104	1.41428365035655	0.157278635471622	0.448875105800825
**Srd5a1**	58.8537309821354	0.584496474049617	0.413361250188849	1.41400886943946	0.157359298307185	0.449023988306106
**Rrm2**	705.244724226658	0.458288005020022	0.32417986490844	1.41368436053072	0.157454599327402	0.449183705752397
**Gm3226**	472.02502089898	0.362931714683681	0.256738478210235	1.4136241564324	0.157472284735434	0.449183705752397
**Gm11574**	26.1738629980925	-0.67421530392241	0.476980876836465	-1.41350594261574	0.157507015315322	0.449201455262178
**Ecm1**	551.480847406979	-0.139751488025099	0.0988824642157798	-1.41330911535674	0.157564854976139	0.449285092265475
**Pstk**	2056.97920807605	-0.128568721246527	0.0909824921311893	-1.41311496569187	0.157621923564168	0.449347689163874
**mt-Ty**	92.2321091106881	-0.574337795658987	0.406455322328825	-1.41304041085811	0.157643842463533	0.449347689163874
**Slc12a7**	34578.1397339291	-0.212721470530234	0.150585303767129	-1.41263101516995	0.157764244737493	0.449609550430219
**6720482G16Rik**	61.5867240523205	0.558306059513032	0.395340104313063	1.41221710983037	0.157886044095893	0.449875297797285
**Smox**	933.408582266607	0.159864474557539	0.113265091402303	1.41141875734441	0.158121175364167	0.449975683621668
**Cenpt**	1534.53175700483	0.122070845084214	0.0864866603386327	1.41144130905569	0.158114529782457	0.449975683621668
**Arhgef10l**	27.020292741882	1.17089307770642	0.829577132970178	1.41143364633764	0.158116787823241	0.449975683621668
**Henmt1**	61.736664602802	-0.435217771673806	0.308339834444025	-1.41148733655694	0.158100966966174	0.449975683621668
**Mis18bp1**	137.214995209114	0.420715856347617	0.298006529675435	1.41176724149577	0.1580185070022	0.449975683621668
**Mapk14**	13187.85578232	0.263416820123055	0.186605049057559	1.41162750661588	0.158059668809637	0.449975683621668
**Gm5855**	39.748943954649	0.487545166551586	0.345402081740672	1.41152932285346	0.158088595730717	0.449975683621668
**Ppil1**	3405.03901841451	0.18373501524115	0.130187253692565	1.41131339689396	0.158152225987356	0.449982778057561
**Ccl4**	739.138096211587	0.53526979300076	0.379328973156506	1.41109651748092	0.158216156735197	0.450083405249414
**Mak16**	3971.38265138881	0.152190141332383	0.107898293524183	1.41049627720269	0.158393194923713	0.450505698991506
**Etf1**	6531.23312907551	-0.128555596422166	0.0911506280835979	-1.41036435101975	0.158432126060366	0.450535103832014

**Gm10357**	59.252554691775	-0.529643450785911	0.375587955626465	-1.41017155329832	0.158489033288441	0.450615607637309
**Rnf166**	14385.3359425166	0.136272424859658	0.0966528407334615	1.40991639589212	0.15856437073269	0.450711677489354
**Btk**	146.570643631919	0.317556500026344	0.225239201728516	1.40986337009443	0.158580030462714	0.450711677489354
**Ap1m2**	345.40078060898	0.276621321977112	0.196236814430593	1.40963010829423	0.158648931907288	0.45082618937117
**Igf2bp3**	28.1341958848614	-0.646647218798785	0.458814080104663	-1.40938834887385	0.158720367312521	0.450913882102734
**Gm50205**	198.837390048856	-0.291075950502612	0.20653469406008	-1.40933198573378	0.158737025069738	0.450913882102734
**Tle1**	2074.02233151089	0.572230451451601	0.406076900399252	1.40916769924364	0.158785586416682	0.450970527247236
**Mbd2**	1806.77274962966	-0.11558577323162	0.0820311265161703	-1.40904773761501	0.158821052906331	0.450989967677969
**Gm13710**	48.913998645071	-0.495584542310843	0.351784297233133	-1.4087739168824	0.158902030089245	0.451057338878802
**Gm37447**	46.5875955273172	-0.736561111628846	0.522828088060439	-1.40880172364362	0.158893805352522	0.451057338878802
**Pex5**	4351.16233178129	0.111867302248131	0.0794241746012122	1.40847925470823	0.15898920562599	0.451223507393504
**Adgre4**	136.459606800919	-0.516517898908622	0.366749469935687	-1.40836713138044	0.159022386715113	0.451236403290121
**Spag7**	7662.56325568049	0.117159534676582	0.0832035722561713	1.40810702593232	0.159099380902752	0.451373594685752
**Gnb2**	1429.38868964658	-0.208822144596933	0.148328127893085	-1.40783914395153	0.159178706515317	0.45151734987612
**Shcbp1**	154.134447655888	0.558653562807391	0.396879069258763	1.40761659175116	0.159244631760867	0.451541778415689
**Gm17334**	3248.19461130561	0.36537522589369	0.259555450631347	1.40769621676195	0.159221042569344	0.451541778415689
**Guk1**	4835.31493552942	0.0945713499995581	0.067196881107407	1.40737707526032	0.159315605309366	0.451661747545822
**Nol11**	6591.33379009817	0.117346680973554	0.0833999848953414	1.40703479887691	0.159417070209463	0.451786830088221
**Pag1**	2848.61468320157	-0.214781590199538	0.152640553672493	-1.40710699111048	0.15939566537368	0.451786830088221
**Ankrd17**	5930.07069555433	-0.156317578350739	0.111125798350795	-1.40667226396238	0.15952459387941	0.45201025458692
**Traf4**	4178.83469998915	0.296507582572965	0.210809906847589	1.40651635877499	0.159570850422182	0.452060030582941
**Spata5**	3279.05438945655	-0.115682599506267	0.0822681211463327	-1.40616557050694	0.159674965209475	0.452095520748107
**Arhgef7**	4361.21458400544	-0.14517627083844	0.103248390471573	-1.40608749613788	0.159698144855237	0.452095520748107
**4930515G01Rik**	125.405665753328	-0.463543616307699	0.329647055194773	-1.40618157815429	0.159670212983408	0.452095520748107
**Gm43176**	35.6284639804973	-0.88590386128241	0.629953900422351	-1.40629950967596	0.159635205685709	0.452095520748107
**Tgfbr3l**	63.6208330642261	-0.9727138067484	0.691868375192012	-1.40592320971232	0.159746928471492	0.452152389424341
**Ankrd28**	1209.30707543406	0.23017420747829	0.163743977857209	1.40569571162495	0.159814500921842	0.452262408589345
**Vps37a**	2988.82278998287	-0.120542079938353	0.0857662869340409	-1.40547159318039	0.15988109066972	0.452288392402654
**Gm37612**	19.9531938092985	-0.975370238281109	0.693971166938484	-1.40549101280972	0.159875319907477	0.452288392402654
**Dgat1**	2886.89402594243	0.147563382054335	0.105059914689324	1.40456407651481	0.160150945204275	0.452839348964154
**Lonrf1**	37.3891744401322	1.16084033032012	0.82649929801405	1.40452669846114	0.160162067130623	0.452839348964154
**Tubb4b-ps1**	253.083206204093	0.292211188325432	0.208023729481568	1.40470122833426	0.160110140364863	0.452839348964154
**Stau1**	4495.43214324237	-0.152183699946781	0.108362076362646	-1.4043999991056	0.16019977116117	0.452864692175557
**Gfer**	979.373832982694	0.164479633894276	0.117138235489358	1.4041498338023	0.160274236706675	0.452993927854184
**Rab11fip5**	52.5396393773569	-0.685810701896736	0.488551717167446	-1.40376274977186	0.160389509775037	0.453238433559049
**Lgals9**	27372.8902973359	0.0883793124640054	0.0629812029311281	1.40326491636959	0.160537855956303	0.453576294836554
**Pacsin1**	3659.76128989116	-0.160309855151169	0.11424999552149	-1.40314977186162	0.160572181890006	0.453591945148946
**Rps11-ps1**	168.113353247767	-0.251168507092757	0.179041486030569	-1.40285088479367	0.160661309529434	0.453762368570584
**Mfsd5**	8653.93482020719	0.156978689889654	0.111949837879614	1.40222346778619	0.160848525860914	0.454209717202584
**Vars2**	1399.66489625616	-0.150039851988721	0.107012478176542	-1.40207809916517	0.160891926231016	0.454250865726952
**A530021J07Rik**	356.638187849964	-0.527694982082661	0.376461005842536	-1.40172547459904	0.160997240432953	0.45446677131889
**Ythdc2**	1387.43684971093	-0.143454822076691	0.102372559943581	-1.40130150262679	0.161123932056806	0.454742933444222
**Slc30a4**	417.496779160785	0.435786677431803	0.311058871565648	1.4009781339409	0.161220611942259	0.454777838898315
**Dyrk3**	33.2687555073389	0.900882681924059	0.643077159981341	1.40089360653113	0.161245890938151	0.454777838898315
**Pcsk1**	574.256353404241	-0.402698656989808	0.287408404193785	-1.40113737494708	0.161172997027402	0.454777838898315
**Ccdc30**	300.057797864232	-0.255680590317613	0.18260445308058	-1.40018814439747	0.161456985119749	0.454777838898315
**Chic2**	3503.2503343295	0.287295975719258	0.205180950736872	1.40020783940948	0.161451088985966	0.454777838898315
**Magohb**	243.594941081979	0.310624482862291	0.221834837868078	1.40025113209231	0.161438128943416	0.454777838898315
**Elovl5**	14641.1920277807	-0.0841360460373387	0.0600813855143254	-1.40036793953891	0.161403165533861	0.454777838898315
**Manf**	3142.58496007199	0.131840111462818	0.0941439446355758	1.40040989330925	0.161390609110018	0.454777838898315
**Gm10259**	247.904600361201	0.310307663883929	0.221614624559425	1.40021293495783	0.16144956354829	0.454777838898315
**Smim10l1**	911.97779780059	-0.230569097927535	0.164641528318814	-1.40043098653128	0.161384296358102	0.454777838898315
**Rps19-ps7**	34.8802442036278	-0.489743922894347	0.3496450185426	-1.40068897573805	0.161307100772854	0.454777838898315
**Gm11824**	30.8823021495644	-0.505225752233131	0.360856060048048	-1.40007556521528	0.161490691288115	0.454777838898315
**Gm15832**	71.4726388541238	-0.445855933616668	0.318467146537704	-1.40000605545628	0.161511505138984	0.454777838898315
**Relb**	4213.6533122684	0.0959714918367785	0.068569090172568	1.39963198571319	0.161623550552465	0.454958561447619

**Ikzf3**	7691.79328271619	0.160065527313941	0.114365277229481	1.39959899710433	0.161633434472537	0.454958561447619
**Ccr5**	985.550001201812	0.37676292981382	0.269229922468352	1.3994095691875	0.161690198970564	0.455037053970204
**Ttf1**	4449.65168096984	0.0725003498628046	0.0518306485533451	1.39879302857239	0.161875057465625	0.455394622475604
**Zcchc18**	2908.13170209495	0.265079415537144	0.189502396790888	1.39881827367943	0.16186748504805	0.455394622475604
**Gm7332**	62.280941173286	-0.720895369211693	0.515555966558724	-1.39828731693978	0.162026804966474	0.455740158132226
**Gzmb**	3027.74978881371	1.29714279346399	0.927935004460433	1.39788108782279	0.16214877876597	0.455779265074351
**Dhx8**	8578.09824008718	0.104546142095539	0.0747903690742071	1.39785567834018	0.162156410484661	0.455779265074351
**Gm9208**	24.5203048640219	-0.861145047366442	0.615976147829004	-1.39801687192196	0.16210800071007	0.455779265074351
**Gm21188**	41.8848541903982	0.807177251627574	0.577344695061934	1.3980855085903	0.162087391006787	0.455779265074351
**Ccdc85c**	28.5731377288533	0.797466937507336	0.570579806985416	1.3976431127499	0.162220265016971	0.455877423911613
**Golga3**	3108.22148238668	-0.184221509052794	0.131850071794405	-1.39720446523573	0.162352094355155	0.456054808507398
**U2surp**	7185.82684860475	0.0948392983268377	0.0678783221380156	1.39719567808413	0.162354736035573	0.456054808507398
**Hmgb1-ps7**	76.6428207685473	0.333034550844827	0.238368060143542	1.397144192239	0.162370214871264	0.456054808507398
**Ssbp4**	12791.8750483762	-0.285667835999914	0.204486250775932	-1.39700265869189	0.162412771615382	0.456093039091709
**Sec22b**	8462.44511547571	0.109359728056966	0.0782971678501293	1.39672648525799	0.162495836528362	0.456244992191268
**Nucks1**	2579.82288043971	-0.136559315615191	0.0978005613247107	-1.39630400649538	0.16262296782929	0.456520595775186
**H1f3**	181.224845362026	0.353046903681072	0.252864888434376	1.39618792417949	0.162657912179747	0.456537357003255
**Dusp5**	985.141933979239	0.780966207283627	0.559528469145199	1.39575776810199	0.162787451786088	0.456738226102812
**Osbpl2**	4908.37517532719	-0.110698347466877	0.0793094905060393	-1.39577680754925	0.162781716495121	0.456738226102812
**Atf6**	738.556364182469	0.198051936216604	0.142028142900879	1.39445557881316	0.163180074680258	0.45773818248718
**Gm15133**	135.579579099968	0.298569753820665	0.214123187501343	1.39438309930255	0.163201948925111	0.45773818248718
**Zfp119a**	854.971172158781	-0.273590988226566	0.196249202312169	-1.39409987405387	0.163287447225883	0.457896477298139
**Gm10254**	227.691865430114	-0.302002670744296	0.216651861587458	-1.39395373079859	0.163331577261327	0.457938730054579
**Il6ra**	24316.6149719563	-0.160904969038118	0.115452499054029	-1.39368978893059	0.163411301047123	0.45799926549262
**Bloc1s4**	1361.00829597265	0.256105832441157	0.183755655189124	1.3937303435781	0.163399049586567	0.45799926549262
**BC048644**	31.0354827936892	1.42809510037917	1.0249079498963	1.39338864580343	0.163502297318608	0.458172807904908
**Pus1**	2809.17610856081	0.0944615323971431	0.0678116381514677	1.39299882692922	0.163620145413762	0.458421520498692
**Rnaset2b**	32591.3615401328	0.115716860725308	0.0830784851691053	1.39286194843067	0.163661541021257	0.458455982555012
**Ctns**	1720.942126643	0.163723230910713	0.117555609759427	1.39273005555214	0.163701436314905	0.458486230361528
**Smarcad1**	1915.95826874067	-0.163139299261497	0.117145078326386	-1.39262614863736	0.163732871504336	0.458492777020405
**Gm43018**	19.8304039496993	-1.5093707768192	1.08441057271653	-1.39188128075708	0.163958351248088	0.459042597835131
**Znrd2**	147.62252447425	-0.322927949601564	0.232060033509872	-1.39157072726971	0.16405242830159	0.459224393808519
**Tacc1**	5494.55993208459	0.115891676917381	0.0832901247999185	1.39142157843776	0.164097624932923	0.459269320793617
**Mocs2**	2214.40619908671	-0.144531862618638	0.103898223432364	-1.39109079870578	0.164197894722811	0.459454621216601
**Stxbp3**	5049.56742589737	-0.158186594189657	0.113735591108222	-1.39082755581003	0.164277724940263	0.459454621216601
**Adh5**	10197.5578040877	0.0696844134069159	0.0501025989841704	1.3908343044027	0.164275678018064	0.459454621216601
**Pef1**	4288.40167448849	-0.100065880323438	0.0719524481657581	-1.39072238505234	0.164309626867776	0.459454621216601
**Utp4**	8653.18379929226	-0.103095207553855	0.074129070255797	-1.39075273975655	0.164300418757313	0.459454621216601
**Myd88**	11127.4535584606	0.166305252764665	0.119621182838755	1.39026591125451	0.164448145239942	0.459682629641692
**Lyrm4**	1758.38646546137	0.119211237916949	0.0857473548358031	1.39026140392582	0.164449513441024	0.459682629641692
**Nsmaf**	9433.91554447027	0.324201576788524	0.233266473521156	1.38983357485841	0.164579420125851	0.459964158021112
**Fkbp7**	104.83327295883	-0.470297537304289	0.338437332066894	-1.38961483484137	0.164645868535663	0.460068265741523
**Aco1**	2463.1960701409	0.126903922769484	0.0913386609023577	1.38937796455266	0.16471784730559	0.460106208118097
**Gm5873**	42.5588898081002	0.521638510744409	0.375445548662296	1.38938525866932	0.164715630458177	0.460106208118097
**Rdm1**	2270.52117392713	0.106440379539463	0.0766244016662263	1.38911857352067	0.164796696726771	0.460207983116592
**Nxn**	578.768638777356	0.463516189078104	0.333735266208703	1.38887386503601	0.164871109011434	0.460207983116592
**Psmb2**	11566.5147296358	0.132299297294335	0.0952520231708291	1.3889394985035	0.164851148348105	0.460207983116592
**Actg1**	90395.4860354187	0.0968421720236188	0.0697193515164313	1.38902858269981	0.164824058683815	0.460207983116592
**Gm5751**	17.489113912914	-0.71579122140643	0.515713045201925	-1.38796415577614	0.165147960178544	0.460899115928678
**Igsf9b**	22.461500776696	1.69404477502798	1.22073051545594	1.38773034144662	0.165219173221806	0.460934580817281
**Acp1**	4160.86536917651	0.112491690901332	0.0810591558373501	1.38777279061544	0.165206242722923	0.460934580817281
**Gm9247**	66.3333049880008	0.38100917439685	0.274580800760992	1.38760311478769	0.165257932469812	0.460961097880478
**Zbed3**	411.092884419547	-0.212622824695104	0.153243987705535	-1.38747906445548	0.165295730647735	0.460976198148028
**Rbm20**	40.7015477272992	-0.77868996491651	0.561261140643735	-1.38739333356198	0.165321856681761	0.460976198148028
**Ap1m1**	21339.0375511913	0.0816824027443685	0.058898286732491	1.3868383492267	0.165491060390995	0.461121598334379
**Dennd2c**	363.435722217865	0.268949830401795	0.193920918064272	1.38690468819179	0.165470828102347	0.461121598334379

**Dubr**	672.315800342669	-0.228966037857132	0.165071716878506	-1.387070069827	0.165420397539476	0.461121598334379
**Gm44986**	35.1650435713484	-0.602505589175788	0.434411624119874	-1.38694628716825	0.165458142051189	0.461121598334379
**Ilf3**	10823.9044108091	0.0902188412893771	0.0650590298990537	1.38672281817546	0.165526299897246	0.461138243896908
**Atp5d**	11424.8349900874	0.0679870964093652	0.0490417833267682	1.38630962818712	0.165652378121625	0.461370998830696
**Prim1**	1855.66634216101	0.184623383791086	0.133181198840465	1.38625710985109	0.165668408414297	0.461370998830696
**Slc8b1**	1293.9670610673	0.19290750242597	0.139169217423038	1.38613628788025	0.16570529161385	0.461392168220434
**Pih1d2**	42.5970905820951	0.835878809990576	0.603165101308228	1.38582091068864	0.165801595595906	0.461496988721887
**Tmtc2**	54.0872588847363	-0.658467441806582	0.475178915808383	-1.38572529188587	0.165830802192253	0.461496988721887
**Gm43481**	1335.84287328579	-0.27378717419198	0.197554821577284	-1.38587948401388	0.165783706387389	0.461496988721887
**R3hcc1**	1966.051846163	0.128274250195589	0.0925792107467821	1.38556214900599	0.165880642834916	0.461554174315693
**Gm42659**	369.568816599698	-0.282742031104087	0.204113901767958	-1.38521692376208	0.165986147339874	0.46176619414449
**Golph3l**	3734.86231829222	0.0923165127276008	0.0666671333523649	1.38473799735273	0.166132595912203	0.462092023616697
**Entr1**	9608.11687153641	0.172291796991864	0.124448423232991	1.38444339041003	0.166222730594902	0.46220816113546
**Cnbp**	74520.6021896155	0.104262707889191	0.0753193852293466	1.38427454727243	0.166274404549505	0.46220816113546
**Trav20**	18.5085537332409	-1.21752931066715	0.879536569852659	-1.38428503418693	0.166271194707809	0.46220816113546
**E430014B02Rik**	1591.52469681219	-0.241302747726609	0.174324225678724	-1.38421809583325	0.166291684043722	0.46220816113546
**Rtn4**	954.692526221597	0.18892030488356	0.136525896890045	1.38376900783675	0.166429195664892	0.462327539999061
**Rdh12**	814.131670897285	-0.130100709139432	0.0940371699573732	-1.38350302543565	0.16651068030048	0.462327539999061
**Hps1**	4956.0054933628	0.129590649051639	0.0936525940806	1.38373795540687	0.166438707138905	0.462327539999061
**Plekhm1**	3540.810300749	-0.160223031939982	0.115798663753389	-1.38363454936926	0.166470383736957	0.462327539999061
**Umad1**	878.667161350456	0.143559866084402	0.103748259169152	1.38373277040092	0.166440295365207	0.462327539999061
**Gm43339**	18.2925883629968	-1.35084840103535	0.97638302330638	-1.38352303224292	0.166504550102318	0.462327539999061
**Rnf187**	14888.7173649589	1.02956557651274	0.744253204186717	1.38335390525836	0.166556376906503	0.462372944135971
**Tpk1**	652.828103687237	-0.17406390979771	0.125842760512292	-1.38318572390748	0.166607925958752	0.462434576243095
**Gm4900**	30.3384344744809	-0.642281400382006	0.464409818217319	-1.3830056454178	0.166663134883572	0.462506343142029
**Rbm34**	2396.81055018144	0.168350390082105	0.121758625355139	1.38265678994871	0.166770127012616	0.462640297770737
**Ptrh1**	220.124544003194	0.324282194810658	0.234533986502048	1.38266611013251	0.166767267890417	0.462640297770737
**Gpr82**	17.6943740206964	0.9712240759959	0.702972825705201	1.38159547635657	0.167095943735607	0.463462556845972
**Nampt**	2542.24917776512	-0.125815287800492	0.0911841607436199	-1.37979323135126	0.167650317271772	0.464246064495467
**Slc25a10**	1231.97174903107	0.14782477990647	0.107134257525546	1.37980869350983	0.1676455552184	0.464246064495467
**Rnf128**	74.7889122748406	0.684474416713684	0.496000753760769	1.37998664623768	0.167590756445575	0.464246064495467
**Gng11**	95.4627499084292	0.446490204126955	0.323499318006944	1.38018901207504	0.167528456249301	0.464246064495467
**Gramd1c**	351.810436884109	0.314108777815624	0.22764587017716	1.37981320535785	0.167644165673411	0.464246064495467
**Plekha7**	576.598801804026	-0.303056554121716	0.219563094574434	-1.38027091806623	0.167503245677816	0.464246064495467
**Ubiad1**	1723.05862883219	0.12079002022981	0.0875309671981968	1.37996898807601	0.167596193499485	0.464246064495467
**Zfp112**	230.135690830616	0.299130095815909	0.21680502231538	1.3797194023521	0.167673056612534	0.464246064495467
**Rps15a-ps6**	51.1847104024349	-0.682839248185548	0.494735218163463	-1.38021152146871	0.167521527599381	0.464246064495467
**Btbd8**	313.68977613328	-0.375385394649515	0.272058859825027	-1.37979478003746	0.167649840301039	0.464246064495467
**Ctnnb1**	19457.1904288177	-0.0915153908700946	0.0663387612633908	-1.37951612492043	0.167735678055979	0.464337856487713
**Rasa2**	7187.89284408661	-0.101213722162179	0.0734183498226655	-1.37858890054939	0.168021540674175	0.46488418198472
**Sugct**	95.5385888487373	0.400389161706497	0.290415979471305	1.37867469426233	0.167995075185788	0.46488418198472
**Gm8116**	655.79163611199	0.4416959525381	0.320364815237525	1.37872803606918	0.167978621980431	0.46488418198472
**C1d**	2330.65335188835	0.179582194254042	0.130281579341574	1.37841585250675	0.168074931744337	0.464950263254128
**Ppp5c**	7926.06242756444	0.0912455754542129	0.0662040726121841	1.37824716598206	0.168126989400814	0.465012633313784
**Fli1**	12430.9973151084	-0.147201051812567	0.106852102487947	-1.37761493115375	0.168322208509866	0.465450271637018
**Gm38140**	46.7880698939097	-0.61109977103912	0.443615612078761	-1.37754342813937	0.168344297649258	0.465450271637018
**Cenpw**	224.392854667998	0.297068887636104	0.215675982950117	1.37738511063057	0.16839321378457	0.465503836772538
**Cc2d1b**	5930.31806395578	-0.126803764798377	0.0920711824900938	-1.37723619235606	0.168439235529241	0.465549383307182
**Akr1b3**	18405.2907838663	-0.0966154574709136	0.0701814720378663	-1.37665190919314	0.168619893744732	0.465844166316909
**Slc25a13**	271.89998582049	-0.291843523036917	0.211979954164034	-1.3767505714766	0.168589377528092	0.465844166316909
**Tmem120b**	671.773652071812	0.243775542933236	0.177084672292882	1.37660442192339	0.168634583021619	0.465844166316909
**St8sia6**	165.491685940276	-0.545978736741438	0.396730160085273	-1.37619669909665	0.16876074378574	0.46610770762442
**Tpm4**	19192.2567475303	0.0732075064060869	0.0531990724572831	1.37610493989101	0.168789146392393	0.46610770762442
**Atxn7l1**	4688.09325623441	-0.144090168252327	0.10474936110321	-1.37557085537118	0.168954534983224	0.466119561591816
**Sema4g**	21.2875713390955	-0.793343336118713	0.576759718091662	-1.37551793447654	0.168970929479752	0.466119561591816
**Gdpd5**	920.015147469462	0.275458071571573	0.200214050710148	1.37581788388247	0.168878023188327	0.466119561591816

**Zscan21**	2526.47735115269	0.16775910581296	0.12194059695078	1.37574450189607	0.168900748973509	0.466119561591816
**Igsf8**	774.787904487495	-0.163381435362482	0.118761778018321	-1.37570721901181	0.168912296048762	0.466119561591816
**Acvr2b**	47.0487820092288	-0.655419399104009	0.476409526667497	-1.37574788583405	0.16889970094586	0.466119561591816
**Rex1bd**	1892.87992564901	0.102985399548446	0.0748946962122193	1.37506932742781	0.169109952523793	0.466421411153055
**Klre1**	552.868232746162	1.00781915132356	0.733056389811525	1.37481804310127	0.169187862861892	0.46655462987659
**Gnl2**	6484.29050963223	0.123580030997116	0.089925507401969	1.3742489152128	0.169364419670365	0.466782394805638
**Safb2**	7337.02232327409	-0.164214011808957	0.11949373917683	-1.37424783039008	0.169364756339688	0.466782394805638
**Zfp830**	1942.69128776825	-0.183139495488298	0.133262668323855	-1.37427456460073	0.169356459656898	0.466782394805638
**Gm11451**	17.4847160019585	-0.871408463407143	0.634149042018751	-1.37413826351152	0.169398762459692	0.466782394805638
**Gm47782**	25.0462766438261	-0.659734258291325	0.480129928994913	-1.37407442954533	0.169418576879701	0.466782394805638
**Mta1**	346.752860356305	-0.234547282740166	0.170736252488813	-1.37374037043207	0.16952229904561	0.466925269098692
**Mtfr1**	5502.55338498807	-0.0908384644480575	0.0661260613506855	-1.37371654371361	0.169529698833129	0.466925269098692
**Timm21**	3562.19258603105	-0.117580601995603	0.0856342664243262	-1.37305551743713	0.169735088227199	0.467409259908419
**Slc16a3**	159.352651433271	0.400090153971584	0.291426690327656	1.37286723299694	0.169793624755744	0.467488755071861
**Herc2**	8378.35807018329	-0.239210668580058	0.174271416996309	-1.37263283160843	0.169866519920164	0.467526070634415
**Susd1**	660.12980719876	-0.214330524265812	0.156138964376777	-1.37269082782318	0.169848481820959	0.467526070634415
**Hscb**	577.704230750049	0.191003799006706	0.139164288822993	1.37250583912119	0.169906022393533	0.467534905768008
**Gm36936**	27.2001001944352	-1.00789956906804	0.734389576822303	-1.37243174587146	0.169929073131521	0.467534905768008
**Sh3bp5l**	2409.0305860706	-0.180166985917378	0.131295311025318	-1.37222711542712	0.169992746745756	0.467542966468708
**Nrm**	3002.48380682416	0.123965979484955	0.0903340333398863	1.3723064818608	0.169968048651292	0.467542966468708
**Gm37383**	53.4597581884891	-0.615100051857217	0.448290227275627	-1.37210229987688	0.170031593623434	0.467542966468708
**Gm48551**	415.262245791722	0.291720895618535	0.212618213947543	1.37204094702116	0.170050691174219	0.467542966468708
**Pfkm**	675.461461608026	-0.279482298912139	0.203760327437705	-1.37162274141704	0.170180910550341	0.46781936628432
**Gm14308**	67.5318533723257	1.35888118563286	0.990857332126312	1.37141961973153	0.170244184816579	0.467911672798682
**Tmem94**	1237.9866692117	-0.246464932351629	0.179796544085294	-1.37079905292678	0.170437606272402	0.467951016495568
**Pccb**	1033.13527548515	-0.167779928196749	0.122390217045526	-1.37086061489979	0.170418410966029	0.467951016495568
**Tlr6**	1075.33468644247	-0.227597558116858	0.1660406186364	-1.37073422145733	0.170457822774085	0.467951016495568
**Dock8**	27202.1953006749	-0.175804347473438	0.128226449996351	-1.37104589168959	0.170360650584196	0.467951016495568
**Mrpl42**	4429.31655482328	0.158644561276315	0.11571904849858	1.37094595345088	0.170391804692369	0.467951016495568
**Fam8a1**	367.882409009269	-0.310806665797188	0.226738463289174	-1.37077168685225	0.170446139660434	0.467951016495568
**Trav16d-dv11**	275.20534116444	0.305625246481716	0.222969094843798	1.3707067640734	0.170466385395986	0.467951016495568
**Acbd4**	1758.3048678108	0.110600165687586	0.080705752262311	1.37041242522728	0.170558195604084	0.468121492446184
**Sh2d1a**	3360.07013765503	-0.111729088107002	0.0815987588572656	-1.36924984732232	0.170921189194189	0.468750588674512
**Sub1**	11175.0650141409	0.17348650227744	0.126706289550544	1.36920197799838	0.170936147904026	0.468750588674512
**Eefsec**	10107.5528983121	0.0895793304662177	0.065415811782714	1.36938345676678	0.170879442696844	0.468750588674512
**Clstn1**	401.371224109185	-0.307410695394099	0.224497870316709	-1.36932566424983	0.170897499129372	0.468750588674512
**Trav7d-5**	136.661625968799	0.247893752340491	0.181016848845423	1.36945126335823	0.170858259333961	0.468750588674512
**Ccdc117**	5546.92737675748	-0.263231765498647	0.192291719258546	-1.36891888279765	0.171024632515802	0.468911629467809
**P2rx1**	65.6146229846695	0.547835403594519	0.400277010200306	1.36864069040681	0.171111618110415	0.469068504969695
**Ell2**	253.766495911424	0.307020680615756	0.224401299496128	1.36817692814231	0.171256701601956	0.469139750893953
**Pard6a**	741.052520702606	0.15292142611298	0.111765330159466	1.36823669643164	0.171237998508261	0.469139750893953
**Denn2b**	50.8245805073508	-0.580860199792203	0.424471498788701	-1.36843157067031	0.171177027777904	0.469139750893953
**Pcnx3**	2470.83607323101	-0.256459993403948	0.187426021924685	-1.36832650434742	0.171209898088929	0.469139750893953
**Hmgb1-ps9**	40.0254091454176	0.441983334633411	0.323152006283827	1.36772579479273	0.171397922619329	0.469444996821271
**Bin3**	4705.1187235832	0.133153713169715	0.0974474026842945	1.36641623585495	0.171808355660281	0.470405605584544
**Gm6034**	30.7006748341043	-0.718391469819059	0.525744971667979	-1.36642575494329	0.171805369600971	0.470405605584544
**Phlda1**	68.8015837137843	0.525076317683044	0.384394378496696	1.36598334173494	0.171944192015968	0.470695732035373
**Homer3**	203.657612153754	-0.259361344517804	0.189895266887622	-1.36581258063315	0.171997796651191	0.470760688176623
**Tigd2**	2573.79313735686	-0.130091105079682	0.0952655828387143	-1.36556247495937	0.172076331382302	0.470893843972028
**Txndc9**	5776.77827141069	0.112606498187018	0.0824750927882237	1.36533945437521	0.172146383853765	0.471003745508555
**Nit1**	2809.20528814628	-0.134701560611464	0.0986787454656678	-1.36505140976208	0.172236892441769	0.47107398137213
**Vwa5a**	3363.87423262411	0.144335311551179	0.105733924575956	1.36508043307797	0.17222777120263	0.47107398137213
**Gm17173**	71.5596357371234	0.810060912191977	0.593463242133617	1.36497234315583	0.172261742759803	0.47107398137213
**Avpr2**	164.457757994435	-0.492425446986098	0.360854523676054	-1.36460932225463	0.172375873205558	0.471304291879225
**Npff**	116.153687118515	-0.446904158027379	0.327573264982078	-1.36428764432845	0.172477053056434	0.471363634951471
**Fam222a**	109.359171258764	-0.524143927792791	0.384177487644952	-1.36432754299542	0.172464501006967	0.471363634951471

**Tmem198b**	358.535952177254	-0.218697414862373	0.160316550659717	-1.36415993209942	0.172517235689861	0.471363634951471
**Tyw3**	684.966599374136	-0.212690022925696	0.155905228872601	-1.36422636023001	0.17249633425526	0.471363634951471
**Gosr2**	16615.0775577232	0.0930647724559975	0.0682305157838676	1.36397580154306	0.172575181781702	0.471440211396374
**Ttpal**	4341.31752481218	-0.116487756749959	0.0854241595162019	-1.36363948336964	0.172681059059335	0.471647676824049
**Lcp2**	30564.1659412478	0.101306508405138	0.0743267488740524	1.36298855983602	0.172886116118378	0.471654940739999
**Zfat**	962.535902830575	0.235288941732201	0.172628625151965	1.36297755673532	0.172889583932133	0.471654940739999
**Clec2i**	7189.01585521784	-0.139637054735891	0.102445203080316	-1.36304141665293	0.172869458121261	0.471654940739999
**Zfp182**	1553.9688106765	-0.163375596672771	0.119822452087725	-1.36348066515246	0.172731073954791	0.471654940739999
**Gm17669**	95.6298492595339	0.521938560238158	0.382900993798591	1.36311623289414	0.172845881591956	0.471654940739999
**Gm28192**	51.2006738655001	-0.548235112554662	0.402236844213157	-1.36296592527992	0.17289324983907	0.471654940739999
**Gm42559**	55.8165216103775	-0.97871128337158	0.717862215911106	-1.36336926735921	0.172766161714612	0.471654940739999
**Wdr6**	9135.09313795798	0.17084461030362	0.125369388193402	1.36272987182537	0.172967659793856	0.471776253309986
**Rif1**	5542.68557247461	0.224826855668244	0.164995123681799	1.36262727437832	0.173000008539666	0.471782820103758
**Dok1**	2099.2579520828	0.184746211496737	0.135637250348798	1.362061019533	0.173178628800591	0.472188207996352
**R3hdm2**	1270.24546799534	-0.186637497305194	0.137070453225682	-1.36161727719614	0.173318699850462	0.472227119133772
**Zfp526**	241.983656788446	-0.226169245053531	0.166110445115785	-1.3615594425497	0.173336962084837	0.472227119133772
**Gm2245**	357.602680996687	-0.367748861444613	0.270097505510628	-1.36154112474816	0.17334274652978	0.472227119133772
**5830405F06Rik**	628.756221989792	-0.292844381100042	0.215072869625788	-1.36160540197176	0.173322449529221	0.472227119133772
**Gm38405**	75.4622577702988	-0.653414649273848	0.47983273789041	-1.36175504019712	0.173275204724926	0.472227119133772
**Tyw5**	625.928553411551	0.300497000905023	0.22072965131055	1.36138030899277	0.173393535557409	0.472283827100793
**Gm12428**	319.568559721805	0.217784713022064	0.159993413441775	1.36121049196391	0.173447179449823	0.47234829011249
**Spns3**	162.058927800666	0.432644195085611	0.317885184748642	1.36100773437337	0.17351124525558	0.472441107912939
**Trip10**	469.044236551894	0.293440684215259	0.215622740439117	1.36089859361617	0.173545738044664	0.472453385343775
**Gm47218**	48.4373794934002	-0.762243430961157	0.560207733148913	-1.3606442500831	0.173626140528084	0.47259061950268
**Wdfy2**	494.390690201524	0.354883538596112	0.260854859620734	1.3604635892622	0.173683267508684	0.472664463926483
**Brd9**	3528.13560399678	-0.0745008430604446	0.0547670054576059	-1.36032347282735	0.173727583567945	0.472703425018151
**Klra6**	157.486234288217	0.480666721094509	0.353468620149774	1.35985684073126	0.173875230995014	0.473023483216235
**Rpl39**	1076.85101237674	-0.18198163140828	0.133843606115176	-1.35965875913176	0.173937934483009	0.473112382815256
**Pgpep1**	396.336136765341	0.254855718372722	0.187481682107892	1.35936330156275	0.174031494092298	0.47312185169295
**Gm7027**	162.124704702467	-0.417828290196572	0.307338335300863	-1.35950593272898	0.173986323807709	0.47312185169295
**E530011L22Rik**	20.4480687683154	-0.870683275930712	0.640463812493991	-1.35945741031057	0.174001689532505	0.47312185169295
**Taok2**	3942.79065982364	-0.231123853485863	0.170064553089762	-1.35903602065666	0.174135174755989	0.473322054274646
**Nob1**	6714.22007889359	0.115647325934333	0.0851011108770345	1.35894026226562	0.174165519172251	0.47332288471838
**Xrcc3**	519.398658646276	0.22826660701751	0.168023520536663	1.35853960379137	0.174292524740436	0.473586361844293
**Gnb1l**	1257.84577420488	0.124303616746953	0.0916598827189137	1.35613981885778	0.175054685653077	0.473704322436477
**Agpat3**	11863.6626213434	0.180097453491453	0.132858100242273	1.35556246222877	0.175238422150402	0.473704322436477
**Gramd1a**	65654.949876374	-0.153116972309085	0.112877956299582	-1.35648249958307	0.174945699825149	0.473704322436477
**Klc4**	2322.73333747811	0.11591863664049	0.0854669738574128	1.35629742587916	0.175004554185778	0.473704322436477
**Mtfp1**	352.193581037075	0.330512945141676	0.243687946895041	1.35629582567755	0.175005063122294	0.473704322436477
**Gata6**	18.9327537513717	1.26057760174887	0.929619831740191	1.35601410244136	0.175094681074907	0.473704322436477
**Acot13**	2272.82579941091	0.128746561871356	0.0948308298161281	1.3576445774121	0.174576490823204	0.473704322436477
**Rnf145**	21458.424949884	-0.0916934419298713	0.0676359033012735	-1.35569183605691	0.175197238049959	0.473704322436477
**Krt10**	147.77476562643	-0.371564800363643	0.273971180527655	-1.35621856155829	0.175029637922414	0.473704322436477
**Get1**	1546.96094452684	0.148451835945386	0.109533306452227	1.3553122858583	0.175318082308552	0.473704322436477
**Cdk2**	2770.94579159498	0.133875862404557	0.0987232107661001	1.35607281576106	0.175076001170258	0.473704322436477
**Usp16**	3362.72062434332	-0.137512983696554	0.101288602958486	-1.35763530821839	0.174579433477327	0.473704322436477
**Slc43a3**	155.771025517798	0.365806481928537	0.26977860892406	1.35595065667904	0.175114868299227	0.473704322436477
**Actl6a**	8812.41099743619	0.11119211693347	0.0819495795201751	1.35683572245903	0.174833414192725	0.473704322436477
**Lmo4**	1000.53549911652	-0.140243428404828	0.103305824983704	-1.35755586315633	0.174604656104617	0.473704322436477
**Pigg**	574.594912582083	-0.256142064104351	0.188675453672723	-1.35758022105332	0.174596922544913	0.473704322436477
**Dhcr24**	237.644475682861	-0.398084104003626	0.293714301163441	-1.35534464078447	0.17530777846164	0.473704322436477
**Pank2**	5326.55186165495	0.162720888708196	0.120061866005182	1.35530867645497	0.175319231797989	0.473704322436477
**Cul9**	1410.29840498365	-0.159404980180999	0.117462774313022	-1.35706806784767	0.174759583507428	0.473704322436477
**Ptpre**	2482.09037795348	-0.161105270044554	0.118623417763669	-1.35812365788955	0.174424449459778	0.473704322436477
**Zfp316**	49.2517457197152	0.786606568258155	0.57971721543943	1.35687978088058	0.174819412283632	0.473704322436477
**Irf2bp2**	1773.19563253955	-0.208363820190611	0.153712793595994	-1.35553986962372	0.175245614887701	0.473704322436477

**Ecsit**	5853.34403288071	0.104388699439812	0.0770036788100725	1.35563262759541	0.175216085225547	0.473704322436477
**Gm5560**	46.9942368326406	0.48032546180307	0.35377433258175	1.35771710258849	0.174553467821781	0.473704322436477
**Rpl35a-ps3**	38.0675881334494	0.597587662097726	0.440746404826549	1.35585374163835	0.175145708158527	0.473704322436477
**Gm10244**	23.025887683599	-0.843177487071377	0.622142150373813	-1.35528108257053	0.175328019829253	0.473704322436477
**Gm16418**	70.4454353006873	0.379533755145821	0.279870891735254	1.35610299732365	0.175066399350197	0.473704322436477
**Gm17477**	59.8060223260114	-0.739686154729197	0.544787204907012	-1.357752436303	0.174542252011796	0.473704322436477
**Zfp703**	153.048905973036	-0.391384887472434	0.288328238395228	-1.35742822017988	0.17464518655069	0.473704322436477
**Trav12-2**	1269.65840916575	0.13450293311361	0.0991459604461671	1.35661536292889	0.174903457728636	0.473704322436477
**Gm4430**	356.646212129191	-0.344214930988758	0.253744012405515	-1.35654405290423	0.174926128836024	0.473704322436477
**Gm38293**	80.8466799052634	-0.468174149105472	0.345196062986297	-1.35625576101677	0.175017805857252	0.473704322436477
**Gm49344**	42.1605878055085	-0.837603659942765	0.616873982220608	-1.35781972345078	0.174520894866084	0.473704322436477
**Tbc1d14**	6535.66005407032	0.132383288821527	0.0977121842942788	1.35482887602665	0.175472084267449	0.474012280439258
**Tbcel**	1689.10391194407	-0.144711948049822	0.106831655878647	-1.35457928513441	0.175551637009441	0.474145893787754
**Gm31804**	40.9205829934746	-0.630720609140751	0.465657882047615	-1.35447209948925	0.175585808820109	0.474156913381056
**Srsf5**	26248.7014592558	0.183055167842869	0.135199937048897	1.35395897245621	0.175749467369228	0.474517538954237
**Lrrc58**	4694.61837652352	-0.19149614653748	0.141467293286954	-1.35364254230161	0.175850447416156	0.474627526539289
**Hexim1**	283.745167135099	-0.326511177811389	0.241203783927663	-1.35367353071588	0.175840556398787	0.474627526539289
**Mettl3**	3153.72652555993	0.0982398576701563	0.0725939349391051	1.35327913761066	0.175966471477348	0.474859340823528
**Kif1c**	207.485236192718	0.485718095307066	0.359056882285284	1.35276085564945	0.176132041883941	0.475224757528298
**Rcc2**	1767.64154436238	-0.161733889436514	0.119601695015913	-1.35227088056733	0.17628867615527	0.475321812145549
**Bzw1**	27055.0973471869	0.101364275868382	0.0749475659110425	1.35246921812904	0.1762252594885	0.475321812145549
**Gm16026**	181.909065160248	-0.260650904625097	0.192740978428009	-1.35233776828861	0.176267287501871	0.475321812145549
**Gm20632**	492.49654466329	0.152423444515406	0.112702329192067	1.35244271886915	0.176233731406064	0.475321812145549
**Clk4**	8859.50380915832	0.103752054854405	0.0767330928443872	1.35211616016588	0.176338158434807	0.475373885792517
**Emd**	4080.67046255813	0.127062150396448	0.0939980820380197	1.35175258517567	0.176454476799389	0.475562586071721
**Chmp5**	2795.58158401113	0.0903621172083875	0.0668502885661619	1.35170870831703	0.17646851816725	0.475562586071721
**Ctsa**	31641.9912158474	0.174585453806811	0.129213344510899	1.351141048687	0.176650254343345	0.475580454995044
**Btbd11**	8540.12755287117	-0.222303412631335	0.164498712620694	-1.35139910270258	0.176567621091303	0.475580454995044
**Fads2**	55.9088335428871	1.26232219621562	0.934193693790529	1.35124247209773	0.176617773418131	0.475580454995044
**Exoc4**	4596.94428587233	-0.154723795518953	0.114518285037315	-1.35108376333558	0.176668601988276	0.475580454995044
**Cisd1**	1370.08976951056	0.216421871700262	0.160134953706653	1.35149676376539	0.176536355885897	0.475580454995044
**Gm12167**	51.8272324989412	-0.395037643656059	0.292347972937341	-1.35125836408904	0.176612684399073	0.475580454995044
**2310001H17Rik**	977.649785299826	-0.249485424320752	0.18466338340857	-1.35102812325691	0.176686424036364	0.475580454995044
**Exosc7**	6500.42035051123	0.127873006436512	0.0946749096964868	1.35065358759204	0.176806426248318	0.475740925272328
**Iah1**	1437.76486450866	0.11078564224145	0.082020407477331	1.35070826455073	0.176788903822154	0.475740925272328
**Haus5**	3349.05413071773	-0.236129365026737	0.174847478022349	-1.35048768044887	0.176859602738399	0.475802759151264
**Isca2**	4289.86613455575	0.123500800669868	0.0914583865580478	1.3503496542822	0.176903851909547	0.475840558985784
**Lpxn**	12137.3054228467	-0.100832787200692	0.0746920448680312	-1.34998027405525	0.177022310384362	0.47599667998744
**Serpinb6b**	1454.0540566773	0.29422581433561	0.217945478220769	1.34999733299157	0.177016838363717	0.47599667998744
**Tom1l1**	227.78172072519	-0.458008221342785	0.339318153792829	-1.34978991316339	0.177083381283304	0.476068873306739
**Gm37204**	57.2582902771231	-0.618448671384443	0.458209151365311	-1.34970824904232	0.177109585284261	0.476068873306739
**Rogdi**	1409.06313029225	0.196036635545687	0.145288158342043	1.34929534370014	0.177242120643895	0.476148521757709
**Atg7**	4213.71006207646	0.145518590860247	0.107851492414799	1.34924967287962	0.177256784711047	0.476148521757709
**Znrd1**	1382.79389743945	0.131645128167086	0.0975645962690918	1.34931248835384	0.177236616041697	0.476148521757709
**Gm7429**	21.1733580660464	-0.75726667393026	0.561293647666464	-1.34914527730456	0.177290307618993	0.476148521757709
**Gm48062**	98.7116733778329	-0.522278018985545	0.387101692451505	-1.3492010734388	0.177272390097169	0.476148521757709
**Rps6kb2**	9599.20325853365	-0.219119976195234	0.162438734066602	-1.34893920132	0.177356495416943	0.476163962904202
**Zfp882**	1338.11441464302	-0.210510311703984	0.156049875219111	-1.34899378425266	0.177338962596238	0.476163962904202
**Tmem231**	87.4264802573493	-0.514043186519753	0.381228771780072	-1.34838507628774	0.177534561409591	0.476560832049562
**Capn7**	7131.40601930994	-0.072981495404684	0.0541351379018247	-1.34813539289468	0.177614839722362	0.476613935542449
**Wdr59**	1080.19941318608	-0.204084821032539	0.151378238785343	-1.34817806489303	0.17760111788827	0.476613935542449
**Prkcd**	6542.89483134574	0.0856812310205472	0.06357696635678	1.34767724744404	0.177762213142979	0.476928178527827
**Slx4ip**	985.131874679846	-0.148122975946896	0.109921353186055	-1.34753595778775	0.177807680696105	0.476968952115853
**Smarcd1**	1515.23361845724	-0.150754666075877	0.111912516535871	-1.34707600849595	0.177955754110891	0.477203678952571
**Fhl1**	161.882901945327	-0.51762134218961	0.384255302352427	-1.34707664154719	0.17795555024697	0.477203678952571
**Mphosph8**	1708.1319816072	-0.156694123499441	0.116345114436719	-1.34680449847912	0.178043205550177	0.477356949098884

**C9orf72**	1005.59615356278	-0.130081976599019	0.0966008152027083	-1.34659294878675	0.178111366365592	0.477404929430351
**Exoc3**	8467.15470563726	0.110063189206818	0.0817422180398323	1.34646687900229	0.178151994989558	0.477404929430351
**Tspyl4**	2716.81512876113	-0.0985782995759314	0.0732120031301914	-1.34647729007813	0.178148639541423	0.477404929430351
**Rnf111**	5405.36144558512	-0.126472410491404	0.0939384694142205	-1.34633245868341	0.178195322345507	0.477439839176698
**D3Ertd751e**	749.720258921681	-0.159552833408898	0.118579603036105	-1.3455335430691	0.178452996591202	0.477886449233103
**Slfn10-ps**	3952.53905565445	-0.236061665516381	0.175431162447387	-1.34560851232561	0.178428804976614	0.477886449233103
**Cdc20b**	32.392436530229	1.86006593985678	1.38224127897019	1.34568831661762	0.178403055839731	0.477886449233103
**Ap4e1**	1886.28058510932	0.11492337068842	0.0854345997201205	1.34516192578772	0.178572948739606	0.478045184045188
**Gss**	1616.41887465932	0.177068275508401	0.131629191602905	1.34520521893483	0.178558971313839	0.478045184045188
**Smg6**	2995.05234819075	-0.148272127977892	0.11024635163926	-1.34491641467699	0.178652228722037	0.478176179373728
**Cpox**	2577.41985146599	0.320574528172038	0.238403210552883	1.34467370396813	0.178730630139665	0.478223559027119
**Svil**	3426.77431715336	-0.161620490876002	0.120190663247237	-1.34470088199398	0.178721849708946	0.478223559027119
**Samm50**	9944.03738341483	0.0786456959461524	0.0584960357345761	1.3444619786374	0.178799043431703	0.478325386647426
**Fnbp1l**	21.164277282237	1.270002445112	0.944880836136084	1.34408741985438	0.178920119640633	0.478486816900452
**Zfp740**	9052.35732654991	-0.116192097747288	0.0864445485424215	-1.34412290545156	0.178908646299244	0.478486816900452
**Cep19**	1268.45714692819	-0.174990843680175	0.130240426521638	-1.34359851509778	0.179078249943286	0.478828437868038
**Igkv16-104**	19.003754664489	-1.6165016438196	1.20333018373373	-1.34335668270522	0.179156506100553	0.478956407639364
**Gm3756**	90.3573372291554	0.430445136084199	0.320459526893439	1.34321216865346	0.179203282503756	0.479000190400625
**Cwc27**	1740.67182003542	0.0974719412182331	0.0725871927555166	1.34282560763208	0.179328449463366	0.479253456274466
**2610028D06Rik**	46.0754834325117	-0.642691521596823	0.478652179084182	-1.34271094895358	0.179365587995222	0.479271421068461
**Notch1**	11417.5561752219	-0.145918362951004	0.108707942814547	-1.34229716038263	0.179499663784257	0.479365074125357
**Pml**	5925.64777346752	-0.180555750237643	0.134519509702742	-1.34222724002363	0.179522326738194	0.479365074125357
**Gng10**	1987.98101210159	0.164115274446094	0.12224813486014	1.34247671454336	0.179441475490461	0.479365074125357
**Rapgef4os2**	124.581174500424	-0.473339271406595	0.352648198114255	-1.34224213802232	0.17951749774231	0.479365074125357
**Washc2**	10229.8495306553	0.0917598129051897	0.0683977413933415	1.34156203166853	0.179738043953768	0.479415211155955
**Plin3**	5408.33835212124	0.134855330997527	0.10049270825979	1.34194145359188	0.179614979451965	0.479415211155955
**Dcun1d1**	5344.99209694259	-0.118245878687254	0.0881436959683832	-1.34151259926369	0.179754081837239	0.479415211155955
**Iffo2**	401.017184796676	-0.265630881494261	0.197961145514854	-1.34183342293465	0.17965001253166	0.479415211155955
**Rps7**	15278.0078567254	-0.0962323190152954	0.0717217088344181	-1.34174604285383	0.17967835258602	0.479415211155955
**Gm13940**	35.9693942276667	-0.625979756746822	0.466547326119829	-1.34172831286583	0.179684103373299	0.479415211155955
**4931406G06Rik**	57.6500216515989	0.502373786538274	0.374430901500435	1.34169958869618	0.179693420454474	0.479415211155955
**6330415G19Rik**	157.7308307615	-0.381837114998227	0.284692713682673	-1.34122545694596	0.179847263522171	0.479582557339457
**Bag6**	1055.0551404169	-0.163320348826923	0.121804321400221	-1.3408419910677	0.179971759520078	0.479742469653135
**Itpripl1**	2139.69469193406	0.22687716871311	0.16921541847231	1.34075943410698	0.179998570831334	0.479742469653135
**Rps6-ps4**	4849.60217738181	-0.0758400606817385	0.0565645823200115	-1.34076939263295	0.179995336529439	0.479742469653135
**Arv1**	1154.76326246703	0.138215324844097	0.103108085144393	1.3404896875986	0.180086194767395	0.479811357903812
**Socs5**	790.057423770801	-0.226268699029236	0.168784911207724	-1.3405742101601	0.180058735212438	0.479811357903812
**Zfp280d**	2831.1898573729	-0.1670300193249	0.124612194659484	-1.34039866468388	0.180115769626264	0.479811357903812
**1700025G04Rik**	962.36021173462	0.269751973237882	0.201332417205184	1.33983377829796	0.180299391303409	0.480219321968866
**Trav21-dv12**	127.211083028168	0.402856264884132	0.300749320563884	1.33950847878494	0.180405196075262	0.480419921338163
**Gm42685**	72.4736121190022	-0.521949467817278	0.389716958026382	-1.33930396680851	0.180471737898586	0.480515913157826
**Sf3a1**	8399.80056910356	0.119132726279797	0.0889685530761872	1.33904309062753	0.180556645334338	0.480579571036008
**Gm9968**	93.5623537940438	-0.678410834204594	0.506626267504339	-1.33907552315926	0.180546087895461	0.480579571036008
**H2az1**	19058.1483215419	0.169348130097422	0.126492439105075	1.33880041602129	0.180635655331874	0.480627494269561
**Sirt5**	1002.61385322687	-0.218410049393139	0.163128232842827	-1.33888564589292	0.180607903265673	0.480627494269561
**4930579G18Rik**	93.5004138054187	-0.448485742121874	0.335162021846351	-1.33811623301245	0.180858549874858	0.481007766477627
**Ar**	762.749028492461	-0.208254776886575	0.155636941508207	-1.33808063091238	0.18087015398743	0.481007766477627
**Tmem154**	1557.32810985701	0.195428820337916	0.146039318989266	1.33819317763513	0.180833472510595	0.481007766477627
**Med8**	3383.12764534864	0.145341920162968	0.108636866769958	1.33786921957841	0.180939072572705	0.481072092043035
**Commd7**	8015.47203257821	0.0946161233710879	0.0707241517440395	1.33781913303841	0.180955403283561	0.481072092043035
**Gm49553**	279.214395841125	-0.425680384344257	0.318227607089924	-1.33766013651974	0.181007251331711	0.481128754931473
**Gm29609**	54.3073713427116	-0.622832761981413	0.465646343130433	-1.33756609746842	0.181037922232799	0.481129118000037
**Tmem214**	7002.66705783044	0.0947475433471394	0.0708459545661216	1.33737408053003	0.181100560670971	0.481177311201318
**Gm48139**	83.4364989297681	-0.427771891226624	0.319871712037881	-1.33732329283299	0.181117130972585	0.481177311201318
**Itm2b**	55480.557709303	0.0857844701597461	0.0641569704764238	1.3371028825507	0.181189056407562	0.481206128739921
**Necap1**	2912.41602477692	0.144150184410894	0.107800938377663	1.33718858648417	0.181161086533275	0.481206128739921

**Ppp2r1a**	20405.4468452504	0.0906466054328238	0.0678158895162282	1.33665732440378	0.181334518018188	0.481511290935724
**Gm33378**	19.8079239444925	0.854745567335648	0.639518378220982	1.33654574511742	0.181370958935416	0.481526908162654
**Gm8817**	62.5668471674734	-0.417320718475713	0.312260197297369	-1.33645185037238	0.181401628431593	0.48152719999943
**Armc3**	129.671603786622	-0.357833553644784	0.26780280201122	-1.33618300838313	0.181489463454929	0.481679210991969
**Vat1**	394.108002748213	-0.249399968307213	0.186672358774911	-1.33603051862616	0.181539298349494	0.481730334134889
**Gm8717**	30.0767348462628	0.61316070292186	0.458987298518893	1.33589906496426	0.18158226662627	0.481763221961633
**Gpatch2**	1948.69686442943	-0.124488607139772	0.0932103507229213	-1.33556634187366	0.181691057593346	0.481970706144503
**Corin**	21.1123150895428	-1.05243954993101	0.788175383775805	-1.33528599293375	0.181782761241942	0.482050258218475
**Rdh13**	1500.88483080706	0.13392962329477	0.100307230674373	1.3351941070883	0.181812825067741	0.482050258218475
**Gbp6**	9363.02968067512	-0.161794135163354	0.12117300188555	-1.33523254062957	0.18180024967577	0.482050258218475
**Ppp3cc**	9578.96440918684	0.299339173053356	0.224345199134536	1.33427937931423	0.182112312991111	0.482366985308068
**Ccr1**	52.0818047873433	1.00023976750569	0.749570322022765	1.33441751643325	0.182067062543334	0.482366985308068
**Stox1**	20.5936400767431	-0.913570001125145	0.68459437414036	-1.33446904566271	0.182050184924393	0.482366985308068
**Slc39a8**	337.378296620184	0.258258267592108	0.193504926695669	1.33463406850761	0.181996141999443	0.482366985308068
**Smpd5**	2456.4951646532	-0.133162186387321	0.0998036555760543	-1.33424157280337	0.182124698962234	0.482366985308068
**Gm17638**	336.374504623155	-0.325131702760366	0.243694979777097	-1.33417480761302	0.18214657375027	0.482366985308068
**Gm37844**	416.769656073599	0.397285116586655	0.297751913363851	1.33428232953511	0.182111346481476	0.482366985308068
**Mob3c**	5027.46679884193	0.0757767291606107	0.0568146642672075	1.33375300440432	0.18228481739598	0.48253805279232
**2610001J05Rik**	3636.24694021813	0.119929728362427	0.0899227417318931	1.33369741683367	0.18230304171306	0.48253805279232
**Igkv1-117**	71.7888922015435	0.906887182607839	0.679943011635195	1.33376939991907	0.182279442404539	0.48253805279232
**Thrb**	91.2547296453803	0.601309714914752	0.450912224636421	1.33354050314249	0.182354492965221	0.482593171926937
**Gpx1**	18586.0317965527	0.0999176246609436	0.0749573225489917	1.33299351235015	0.182533932835866	0.482905839438339
**Gm6252**	727.668407444624	-0.171461047414546	0.128622960898298	-1.33305162792917	0.182514861855433	0.482905839438339
**Ndufs3**	7153.81770989135	0.10351788306875	0.0776841078849402	1.33254903592474	0.182679839340379	0.483210714870967
**Trp53i11**	9354.01852535029	0.138933807220419	0.104278256023921	1.33233727258105	0.182749384451086	0.483313536555181
**Gusb**	6674.29507309441	0.131320271332114	0.0985864500030901	1.33203164662079	0.182849789544315	0.483497924794391
**Haus1**	1340.32391225913	-0.159063405608106	0.119427652805419	-1.33188086570926	0.182899339570047	0.483547800940475
**Rreb1**	1866.62195703555	-0.262283726670773	0.197023659998568	-1.33122959279449	0.183113476950766	0.4838347180259
**Foxp3**	152.849711635697	0.599473148312257	0.450333071019157	1.33117727053796	0.183130688475374	0.4838347180259
**Gm42868**	75.8863986512911	-0.63358652875533	0.47591588083945	-1.33129940450353	0.183090514098031	0.4838347180259
**Gm43848**	86.5384243742997	-0.421364486649867	0.316490278221542	-1.33136628719734	0.183068516674802	0.4838347180259
**Psmd3**	11134.9734968148	0.0821204604061969	0.0617398126224134	1.3301054363159	0.183483534638907	0.484290204746069
**Nop9**	4712.56127775832	0.11974340297346	0.0900031223996801	1.33043609800237	0.183374627644974	0.484290204746069
**Rbm43**	3049.51821885257	0.114138318269992	0.0858079058390128	1.33016086517868	0.183465275202768	0.484290204746069
**Card11**	8603.80010858885	-0.163557877942001	0.12295177413501	-1.33026041383025	0.183432485157873	0.484290204746069
**Syne2**	4149.3826915462	0.134759625787477	0.101330124907092	1.32990683581053	0.183548968886433	0.484290204746069
**Fndc10**	103.868520072229	0.476319177561691	0.358007904095738	1.33047112120272	0.183363095181692	0.484290204746069
**Gm6478**	26.2261621984616	-0.641803541388134	0.482562080748777	-1.32999165701596	0.183521020157445	0.484290204746069
**C730034F03Rik**	418.096695631133	-0.309651305076077	0.232836270772426	-1.32991008681259	0.183547897617715	0.484290204746069
**Med23**	2393.37821239403	-0.162800932357716	0.122443858012919	-1.32959655959663	0.183651232280024	0.484316730884743
**Ifih1**	1549.52537664376	-0.138843840694221	0.104418158310305	-1.32969057241569	0.183620242304338	0.484316730884743
**Gm4540**	132.813882040945	-0.308049836076526	0.231682336814262	-1.32962158579869	0.183642982373559	0.484316730884743
**Ldlrad3**	40.3496806629311	0.796716707335392	0.599323506494728	1.32936001792281	0.183729221982005	0.48444132375677
**Scamp2**	8092.83587048785	0.0638936802965732	0.0480837018147016	1.32880119219602	0.183913568768989	0.484846261183363
**Ncstn**	13515.2929847242	0.0943970894544515	0.0710592215966033	1.32842841975296	0.184036615986226	0.48494525423498
**4930550C14Rik**	33.3577034741727	-0.747492199056568	0.562677879590275	-1.32845492273638	0.18402786569206	0.48494525423498
**Psme1**	24491.0301330434	-0.0689701360794404	0.0519194030206111	-1.32840772556765	0.184043448646645	0.48494525423498
**Zfp287**	2102.05529510137	-0.147416129298712	0.110995751287778	-1.32812407311436	0.184137121950049	0.485010991641885
**Tmem141**	472.660472796142	-0.209360840126544	0.157627770583674	-1.32819768592367	0.18411280868625	0.485010991641885
**Spp1**	24.0521900938554	0.854676884122624	0.643556512720112	1.32805257538328	0.184160738909272	0.485010991641885
**Hdac3**	6252.74589593353	0.0849287446101442	0.0639648515426716	1.32774082268427	0.18426374252285	0.485201168270815
**Plekhg3**	301.911268369019	0.481961221744889	0.363237887098721	1.32684733300935	0.184559189287432	0.485897935773112
**Mcemp1**	37.4985718718501	0.832161565362187	0.627235437636397	1.32671324901222	0.184603556561424	0.485933551743795
**Bin2**	91397.5554127885	0.106357408918009	0.0801850062499797	1.32640020737089	0.184707170151028	0.486125084361074
**Fads1**	1569.90437643348	-0.114999194799207	0.0867069395581884	-1.32629747267267	0.184741183665998	0.486133405314819
**Baiap2**	1311.40438619956	0.162358523737019	0.122425955739421	1.32617730248799	0.184780975622245	0.486156926511891

**Thbs3**	45.4290277603706	0.648732824018153	0.489273386788035	1.32591071073154	0.184869274798804	0.486265517272064
**Eif2ak1**	9208.9140541826	0.0806686110820351	0.0608421889024397	1.32586635256314	0.184883969916668	0.486265517272064
**Plxdc1**	251.366463929065	0.424822849808507	0.320453685258869	1.32569188419639	0.184941776748054	0.486279327274118
**Cd226**	11737.001535365	-0.0991763746428284	0.0748178273039089	-1.3255714341981	0.184981693405425	0.486279327274118
**Flvcr2**	24.666864716153	-0.808347599000169	0.609810819603751	-1.32557110010843	0.184981804130295	0.486279327274118
**Lmbr1**	325.22393863301	-0.305275784720913	0.230384510785108	-1.32507078570772	0.185147674662341	0.486393814273203
**Septin2**	3796.47682391891	0.538808792619638	0.406655043141476	1.32497752507199	0.185178605765413	0.486393814273203
**Ctnnbl1**	9180.20406420119	0.0798260930535507	0.0602358673846337	1.32522526062129	0.185096449456224	0.486393814273203
**Gm5582**	17.7416908507513	0.603110340054854	0.455163087755796	1.32504228984938	0.185157125278946	0.486393814273203
**Mysm1**	3350.87653659586	-0.1519617158095	0.114690317683769	-1.3249742339061	0.185179697393219	0.486393814273203
**Pde4dip**	3131.84457505783	-0.143551831415232	0.108369717566159	-1.3246489391983	0.185287616004678	0.486515075051776
**Gm18852**	598.922876414766	-0.29040580999685	0.219221058847819	-1.32471675633337	0.185265113396612	0.486515075051776
**4930417O13Rik**	281.09168058599	-0.255835106836931	0.19318128127378	-1.32432658666528	0.185394604413866	0.486714891994217
**Farsa**	12643.2561028072	0.0914386466118627	0.0690594739124738	1.32405651870086	0.185484274808236	0.486869185099679
**Ltbp1**	39.2825609268506	1.08835893140258	0.82229167586736	1.32356797878876	0.185646565706199	0.486949241749873
**Bex3**	1867.93460668841	0.125946583014593	0.0951616777521848	1.3235010772149	0.185668798298115	0.486949241749873
**H2-Q10**	3546.98898669522	0.140914889061467	0.106442429750615	1.32386013163753	0.185549501146596	0.486949241749873
**Gm28529**	75.8138093850168	-0.411329144524466	0.310732532500635	-1.32374019937428	0.185589342775546	0.486949241749873
**Gm44187**	39.4450710489403	0.611390862687741	0.461950171697153	1.32349958966691	0.185669292659341	0.486949241749873
**Ckap5**	3384.94206110635	-0.115305608912269	0.0871366389520311	-1.3232735425536	0.185744426877588	0.487065224548203
**Denr**	5374.61460944116	0.103913517629832	0.0785528156431657	1.32284905103682	0.185885581383492	0.487354260542376
**Ccnb2**	434.583863701824	0.471410630470687	0.356472339891532	1.32243256409215	0.186024151217557	0.487636425009988
**Mark4**	428.748966200558	-0.226574268680028	0.171359566918389	-1.32221546047637	0.186096414271188	0.487732595030758
**Elovl6**	1761.38430914829	0.380378381656008	0.287740272076071	1.32195044826901	0.18618465184426	0.487732595030758
**Gm49838**	47.9773750081183	0.908642780657701	0.68730648505875	1.32203434771903	0.186156713621137	0.487732595030758
**AC115752**	54.9167508748929	-0.603827332540312	0.456728774925329	-1.32206982719456	0.186144900012259	0.487732595030758
**Tbca**	1447.37451341801	0.130164504463579	0.0984925149494688	1.32156747678095	0.186312219367329	0.487935162444934
**Zc3h4**	1656.66386987397	-0.286226975933265	0.216587182459078	-1.32153238563572	0.186323911431819	0.487935162444934
**Tsg101-ps**	34.1692864064539	0.594223418406368	0.449724630047563	1.32130503580274	0.186399675556398	0.488052457251937
**Xylt2**	8581.42517278727	0.155058124154724	0.117420994397396	1.32053151951643	0.186657619643025	0.488079009743635
**Bsn**	272.122594469702	0.243990057922442	0.184750062719144	1.32064939156938	0.186618295887155	0.488079009743635
**Smug1**	1650.02205018115	0.143104131305384	0.108363874693631	1.32058891129514	0.186638472182997	0.488079009743635
**Slc25a43**	18.2078737123869	-0.844649340103377	0.639603667323107	-1.3205823907146	0.186640647553132	0.488079009743635
**Gngt2**	770.39288282631	0.328471819017748	0.248644274946082	1.32105120493515	0.186484291356032	0.488079009743635
**Nudt18**	2631.48243417403	0.147185056812056	0.11141068700232	1.3211035742827	0.186466831446612	0.488079009743635
**Inca1**	42.7567353192869	-0.610318768191627	0.462074923425567	-1.32082209453623	0.186560690830037	0.488079009743635
**Cyp4v3**	1453.29184527533	-0.147769058605808	0.111870080355983	-1.32089883314278	0.186535098891738	0.488079009743635
**Trim37**	1534.59981605871	0.16641415273546	0.12607108809185	1.32000251012522	0.186834179922575	0.48812143320626
**Tbc1d31**	388.78677945575	-0.217762062900735	0.164980503560446	-1.31992604096369	0.186859712197764	0.48812143320626
**Pdrg1**	11947.3434128867	0.0872065930343487	0.0660527609722098	1.3202565911066	0.186749363373776	0.48812143320626
**Zfp319**	1381.69761157835	0.218230514242972	0.165295910390513	1.32024146107064	0.186754413240273	0.48812143320626
**Gm12846**	34.5462799561752	-0.681701414922482	0.516439220077308	-1.32000318415095	0.186833954883696	0.48812143320626
**Gm44101**	28.7735967735932	-0.787816847375221	0.596797577368584	-1.32007380266	0.186810378391708	0.48812143320626
**Zfp712**	824.227021267531	-0.204841421288618	0.155203423476038	-1.31982540527042	0.186893317359693	0.488128294652194
**Dazap2**	74968.2147791142	0.110709414369504	0.0839015071340206	1.31951639667999	0.186996532142197	0.48817191752511
**Ywhae**	10598.7590020502	0.0929138344522654	0.0704230983618834	1.31936589859777	0.187046816624089	0.48817191752511
**Fam126a**	1803.61979881706	0.164974192037558	0.125039201746468	1.31937976037357	0.18704218470429	0.48817191752511
**Igkv5-48**	26.9081885593259	-1.77119172770179	1.34251194748862	-1.31931170595173	0.187064925938733	0.48817191752511
**Gm3365**	195.54045912382	-0.301494594312706	0.228479304245059	-1.31957069507413	0.186978392391246	0.48817191752511
**Phykpl**	2104.81557087317	-0.142559318236938	0.10809038433722	-1.31888991894212	0.187205916880597	0.488344343075593
**Slc15a2**	402.824179463546	1.04598534592791	0.793072826014861	1.31890201204336	0.18720187342162	0.488344343075593
**Snx5**	8599.04466659462	0.119663895081469	0.0907391348760124	1.31876830482217	0.187246583460212	0.488344343075593
**Zfp280b**	2283.81930289404	-0.15643192679483	0.118626685233731	-1.31869087032662	0.187272480201588	0.488344343075593
**Rsf1os2**	78.1050236905312	-0.934901597203486	0.709033397697903	-1.3185579131236	0.187316951802303	0.488344343075593
**Gm15283**	27.3929124618664	-0.840753626808036	0.637622076195178	-1.31857672153541	0.187310660280584	0.488344343075593
**Slfn5**	1545.51802622378	-0.254090405903147	0.192772196844955	-1.31808637377054	0.187474735418933	0.488674839701592

**Bax**	8812.36667698172	0.108130393670814	0.0821052547448549	1.31697287837219	0.187847715822784	0.488700771700569
**Cmc2**	781.24854119393	0.157399932715053	0.119538950499246	1.31672506791872	0.187930797749814	0.488700771700569
**Id2**	10397.050661487	0.218325747482415	0.16583183964292	1.31654902914017	0.187989833688751	0.488700771700569
**Dnmt3a**	5067.11811470694	0.210497285367646	0.159829794248341	1.31700904926761	0.187835591290646	0.488700771700569
**Alcam**	169.953204574367	0.516765428862454	0.392471390984481	1.3166957916759	0.187940614811215	0.488700771700569
**Epb41l5**	79.1570749744185	0.560606081164062	0.425859485615385	1.31641092919173	0.188036156131451	0.488700771700569
**Vamp4**	2668.67920479068	-0.122758524052697	0.0931855520974537	-1.31735576266497	0.18771940179979	0.488700771700569
**Cap1**	2301.06339219901	-0.186942108882831	0.141894606973863	-1.31747155772641	0.187680608746619	0.488700771700569
**Tmed2**	26392.6654673905	0.160215853218027	0.121579116139719	1.31779090278882	0.187573654104459	0.488700771700569
**Fam216a**	1298.33797650663	0.152290287098678	0.115667151565052	1.31662520463322	0.187964285980222	0.488700771700569
**Pla2g2d**	65.5756696866753	-0.647108775202336	0.491066918272335	-1.31776088171239	0.187583706808721	0.488700771700569
**Tmem183a**	4588.12021330411	0.103241777052269	0.078372789189058	1.31731661103983	0.187732519510305	0.488700771700569
**Fhit**	168.235180847179	-0.431665002818612	0.327915573516245	-1.31639067394653	0.188042950994513	0.488700771700569
**Evi2a**	2099.82706029187	0.140727396431019	0.106849672708408	1.31705968641629	0.187818618621468	0.488700771700569
**4931413K12Rik**	19.5899487008278	-1.02226544124052	0.775911964421674	-1.31750184056314	0.187670464525923	0.488700771700569
**Gm42638**	37.3103620032178	-1.00947725995371	0.766448864450679	-1.31708363959442	0.187810590337695	0.488700771700569
**Gm47856**	31.3871019431187	-0.724430295715014	0.550225088944007	-1.3166071672692	0.187970335113139	0.488700771700569
**Gm36298**	154.721624561174	-0.367852433827883	0.279418809550154	-1.31649130715324	0.188009194173647	0.488700771700569
**Gm37242**	68.2807037187502	0.64483055172694	0.489916586967159	1.3162047762432	0.188105321053619	0.48878224667577
**Gm15232**	149.993185707195	-0.412819671917073	0.313735660089274	-1.31582004990955	0.188234448074259	0.489037130800675
**Thumpd1**	13400.6038603614	0.0803902758539292	0.0611144888593122	1.31540453588658	0.188373981915773	0.489077085689049
**Fam89a**	162.090549627843	0.437325442161185	0.332423167039523	1.31556848475964	0.188318917103395	0.489077085689049
**Gm26049**	214.50577904398	-0.290494668363818	0.220800722416734	-1.31564183841548	0.188294283967571	0.489077085689049
**Gm26852**	31.9712396472789	-0.727558875996776	0.55308863244044	-1.31544716944643	0.188359661620318	0.489077085689049
**Tpi1**	5439.26748644469	0.100061532214737	0.0760783440153275	1.31524329965144	0.188428147229334	0.489137119586921
**Rab8a**	31922.2632280469	0.0892445702318791	0.0678637298424305	1.31505548603196	0.188491255369852	0.489220344401705
**Tpx2**	296.363187605717	0.415313521998736	0.315836836573974	1.31496226502212	0.188522584790096	0.489221075187342
**Spin1**	5241.41160568253	-0.0657080801703375	0.0499809247810005	-1.31466315315789	0.188623135286327	0.489240286865292
**Qtrt2**	988.026170952912	-0.173181911889445	0.131716944773174	-1.31480359028732	0.188575920522349	0.489240286865292
**Gm5510**	35.137513142339	-0.647292122268206	0.492331140558007	-1.31474950281342	0.188594103622265	0.489240286865292
**D130062J10Rik**	20.8596753763145	-0.895627447303398	0.681348468023483	-1.31449249442288	0.18868052224528	0.489308589371112
**Zfp692**	2091.22687448785	-0.117376934577306	0.0893124753798804	-1.3142277613296	0.188769568816041	0.489458959327687
**Egfl7**	41.1013831986448	0.571071433143433	0.434593013308002	1.31403730767919	0.188833649652074	0.489539025953957
**Gm37847**	56.9840232197885	-0.472280770583129	0.359435513866025	-1.31395132746723	0.188862584170471	0.489539025953957
**Syncrip**	7013.82601232934	-0.104320193515796	0.0794077377671477	-1.31372831476071	0.188937648829372	0.489628891185434
**Gp1ba**	75.6154323613515	0.539372637909578	0.410586543047066	1.31366370146171	0.188959401364386	0.489628891185434
**Arhgap18**	1572.94437009013	0.14096065593615	0.107316097304289	1.31350896535553	0.189011501900856	0.489683366565568
**Sarnp**	4849.44974831662	0.241824530898923	0.184161380292424	1.3131120678773	0.189145187985758	0.489949157831923
**Chd8**	4468.48071394944	-0.208535255653668	0.158847123569382	-1.31280473305255	0.189248754759041	0.490136855479652
**Airn**	363.769925638443	-0.298998651859248	0.227829824917103	-1.31237713046587	0.189392919316065	0.490429618679251
**Zfp518a**	1253.34092885741	-0.147402858222571	0.112341591662854	-1.31209515586123	0.18948803023844	0.490595283021212
**Gm45479**	17.0148386584995	-1.28625878719862	0.980929925173799	-1.31126470320571	0.189768349022352	0.491240327837227
**Entpd6**	7173.9604667241	0.0923577560639313	0.0704472713519931	1.31101963626755	0.189851129573536	0.491373891046355
**Paxx**	2956.55269828189	0.158774394077142	0.12113668047301	1.31070451540496	0.189957612559417	0.491568747101122
**Aldh16a1**	3046.98834002239	0.122716949255007	0.0936604323114112	1.31023257341997	0.190117169494123	0.491639866562084
**Hmbs**	3292.13195212734	0.148812253168477	0.113565638622869	1.31036337199366	0.190072938454625	0.491639866562084
**Gm5134**	95.2874452378764	-0.536436985633803	0.409401267643263	-1.31029634744862	0.190095602627111	0.491639866562084
**Gfod1**	144.602355654996	0.392478049461146	0.299623442313101	1.30990434670667	0.190228196507365	0.491639866562084
**Ptcd3**	7147.39877062778	0.102434085564135	0.0781987203019006	1.30992022847265	0.190222823191462	0.491639866562084
**4930469K13Rik**	19.3418839705987	-0.669159552599659	0.510853623188273	-1.30988510646824	0.190234706253032	0.491639866562084
**Gm15408**	17.6602634362719	-1.22020249310649	0.931455317389695	-1.30999573498166	0.190197278422584	0.491639866562084
**1110038B12Rik**	292.53655530379	-0.239501679405182	0.182802757193279	-1.31016448046215	0.190140198910135	0.491639866562084
**Gm14167**	21.8680790019383	-0.660505981307946	0.504327538471473	-1.30967661077922	0.190305259243457	0.491741549671884
**Arrdc2**	1481.57510631368	0.146578095500101	0.111952274276801	1.30929091389146	0.190435826254033	0.491845538317154
**Gpr180**	3368.90956962386	0.113309303863615	0.0865675184255877	1.30891246421733	0.190564004025273	0.491845538317154
**Gm1976**	223.813112344809	-0.38853298550272	0.296812419191914	-1.30901862718723	0.190528041097882	0.491845538317154

**Srp54c**	5196.91411420548	0.120041720504019	0.0916881457156331	1.30923926497896	0.190453315571263	0.491845538317154
**Rbm4**	3109.76129189264	-0.133361570345556	0.101882700286537	-1.30897168970284	0.190543940652255	0.491845538317154
**Gm37978**	32.8010688914771	-0.643071144263112	0.491248327708333	-1.30905513157272	0.19051567631582	0.491845538317154
**Gm37033**	780.084304686019	-0.328279564104672	0.250758476664654	-1.30914642835261	0.190484754815693	0.491845538317154
**Bet1**	1429.7752000297	0.21428147845278	0.163722012848616	1.30881287570605	0.190597744384113	0.491852056708232
**Gtf2e2**	1730.70396362714	0.126569293458439	0.0967346203653722	1.30841774103604	0.190731658448391	0.492117036543524
**Gpatch8**	6985.26075214002	-0.133054512191429	0.101712003130009	-1.30814956049344	0.19082258626923	0.492271036647718
**Gm35363**	55.5369710463921	-0.878752471045373	0.672027714051869	-1.30761344014683	0.191004455935211	0.492659553473746
**Ankrd39**	3943.0455058267	-0.112764435625804	0.0862473889327824	-1.30745332723855	0.19105879623476	0.492719059291508
**Ik**	9886.07983544474	0.0792263848322076	0.060623274323316	1.30686416589242	0.191258848116516	0.493127508280848
**Svbp**	1496.94233657096	0.141079945744833	0.107958117165242	1.30680257723368	0.191279769665072	0.493127508280848
**Zfp110**	8237.5631770349	0.118277510358543	0.0905197922927001	1.3066480530146	0.191332268656855	0.493182162150509
**Ppm1g**	13480.9441012504	0.0882781111901607	0.0676079261216062	1.30573612081186	0.19164230993257	0.493777310021533
**Gm9755**	171.780017723668	0.296730582194141	0.227259171585491	1.30569243971091	0.191657170025493	0.493777310021533
**Rps8-ps2**	31.3245323155383	0.558005201670295	0.42731555741034	1.30583872268067	0.191607408621347	0.493777310021533
**Fam234b**	3577.29628311703	0.122257150118144	0.0936532618998804	1.30542329907145	0.191748749030331	0.493923829766588
**Gm20518**	366.906366207101	-0.168280870981835	0.12891716522849	-1.30534107450763	0.191776733547756	0.493923829766588
**Prr14**	15099.4675578803	0.101831221527402	0.0780218829689505	1.30516231667886	0.191837582806141	0.493971937318682
**Gprasp1**	2446.15933293692	0.181018128596234	0.138700366596245	1.30510201983226	0.191858111086522	0.493971937318682
**Mrps18b**	4493.56439842894	0.096890452114104	0.0742625860427739	1.30470075548267	0.191994764142047	0.494162283336528
**Atp9a**	329.489619900138	-0.403965508346099	0.309616896868206	-1.30472694621074	0.191985842544849	0.494162283336528
**Nup54**	2857.93593524228	0.147127486805406	0.112797669115151	1.30434864443173	0.19211473668598	0.494309586211788
**Ranbp3l**	19.1992611939836	0.738168298036297	0.565909399589524	1.30439306816907	0.192099597432584	0.494309586211788
**Cbfb**	7915.30768037495	0.0811445482157114	0.0622152947278853	1.30425401937929	0.192146987069375	0.494311849020759
**Gabbr1**	721.80933575696	-0.265555697129092	0.203641442143418	-1.30403563407329	0.19222143297373	0.494422644362889
**Hpgd**	86.1405905195917	-0.700708726620214	0.537428452338641	-1.30381769623669	0.192295747477903	0.49453306561275
**Arl5b**	702.346804027788	-0.278223584090831	0.21345474972795	-1.30343121643079	0.192427584952135	0.494791360328132
**Pja2**	4197.63471204914	0.095731979371992	0.0735022036925953	1.30243685988474	0.192767089031518	0.495512245671056
**Osbpl7**	3385.34751764636	0.175829020104961	0.135001170631281	1.30242589218127	0.192770836197472	0.495512245671056
**Col4a3**	17.8778856343655	-0.919137544201731	0.706667312657479	-1.30066514714716	0.193373097061511	0.496979267721127
**Mef2b**	53.2462742967316	-0.479475457961233	0.368728001836709	-1.30035000209604	0.19348103772748	0.497175588954976
**Cd164**	23275.1231603824	0.084147165408713	0.0647440550158697	1.29968945238427	0.193707426872526	0.497595031827583
**Tcf4**	683.696598024611	0.374260498088343	0.287945685691324	1.29976074199475	0.193682984539609	0.497595031827583
**Ikzf1**	43778.6920418235	0.0968604174771715	0.0745764396873449	1.29880720886181	0.194010100073197	0.497648094189603
**Ero1l**	2829.11319468003	0.0991614001596447	0.0763184113807289	1.29931163877297	0.193837001734608	0.497648094189603
**Slc25a30**	2600.01128024228	-0.146259897946984	0.112591061670605	-1.29903649345522	0.193931405542367	0.497648094189603
**Babam1**	7137.71309642525	0.085861666309629	0.0661130290612113	1.29871021686411	0.194043396497991	0.497648094189603
**Zfp30**	17.5864192654543	-0.983847564952978	0.75707619674083	-1.29953572597895	0.193760141165919	0.497648094189603
**Arrb2**	6350.15530872504	0.134640748910831	0.103664518885116	1.29881226825587	0.194008363346725	0.497648094189603
**Col4a4**	28.4309223833064	-0.652879721181425	0.502485177338984	-1.29930145330632	0.193840495820393	0.497648094189603
**Trbj1-5**	474.997313487449	-0.23997183177272	0.184739134800578	-1.29897670047965	0.193951925290923	0.497648094189603
**Gm37940**	375.054054561676	-0.395600998107868	0.304611027643394	-1.29870872098234	0.194043910052733	0.497648094189603
**Gm45206**	26.7514884121352	-0.600244515701824	0.462171368544863	-1.29874881170524	0.194030146755868	0.497648094189603
**Cd9**	3005.35631690834	-0.131705728782511	0.101433682101269	-1.29844176070646	0.194135576800083	0.497736499390809
**Cd300e**	67.4005359610078	1.01966229575774	0.785312654119202	1.29841572068055	0.194144519923132	0.497736499390809
**Gm9917**	501.81731191318	-0.23200507465319	0.178695868040364	-1.29832366689522	0.194176137074749	0.497736499390809
**AC159819**	730.588227169923	0.211780050213083	0.163128529769592	1.29824041516348	0.194204734292993	0.497736499390809
**6720464F23Rik**	84.5951665401286	-0.38215803689691	0.29440134285529	-1.29808523694389	0.194258046724832	0.497792168196973
**Cetn2**	3372.89007091307	0.141506961605638	0.109022129185111	1.29796549254116	0.19429919299232	0.497816647801625
**Gm9001**	144.678233135938	0.341523126850406	0.263177029410755	1.29769352445031	0.19439266973643	0.497975174286607
**Arl3**	587.010940996751	0.194551703499602	0.149975830571048	1.29722037716895	0.194555371377593	0.498068072590859
**Ypel5**	5351.40890743916	0.0893666640157657	0.0688869898071836	1.29729378894193	0.194530120649382	0.498068072590859
**Btbd7**	2408.7858714381	-0.142766723795921	0.110049150940975	-1.29729963907212	0.194528108540876	0.498068072590859
**Gm10263**	118.37329615541	0.566316613472407	0.436491520957205	1.2974286699327	0.194483733224465	0.498068072590859
**Tarsl2**	463.997547351945	0.253798174626323	0.195698360092296	1.29688452425777	0.194670922080563	0.498202000848372
**Wscd2**	764.643183460309	-0.26976030361028	0.208003787780726	-1.29690091939411	0.194665280140398	0.498202000848372

**Gm37137**	40.3281196843978	-1.03354306935992	0.797039079372883	-1.29672822338036	0.194724714799029	0.498258741651235
**Mitf**	72.0407188175476	-0.625665070140716	0.482533912154564	-1.2966240390174	0.194760577084653	0.498269591349713
**Mef2d**	12661.9471072007	0.280900256994457	0.216785540741718	1.29575181090664	0.195061005072788	0.498348172446326
**Ska3**	90.4061172563285	0.499811433594863	0.385683768665333	1.29590995059106	0.195006510640678	0.498348172446326
**Angel2**	5899.77088397895	0.0910762599694417	0.0702883305302796	1.29575221494565	0.195060865828002	0.498348172446326
**Mbnl3**	1832.102449068	0.168998488494447	0.130377306259476	1.29622626316659	0.194897543838449	0.498348172446326
**H2bc21**	402.44329157599	-0.270244205740701	0.20856867762055	-1.29570848712172	0.195075936260008	0.498348172446326
**Zbtb3**	177.614798632579	-0.214792710468298	0.165732549577706	-1.29602006977869	0.194968570514241	0.498348172446326
**Gm17096**	72.5461899117837	-0.50953096180395	0.393107426763642	-1.29616213562484	0.194919631582487	0.498348172446326
**Trav4d-3**	78.4917399875159	0.344494040332031	0.265867413346673	1.29573623181429	0.195066374182708	0.498348172446326
**1700007L15Rik**	261.994589688787	-0.302626079100434	0.233534770140315	-1.29585020217164	0.195027098443377	0.498348172446326
**Nkap**	1882.16481982443	-0.111717354793516	0.086245068656443	-1.29534774026956	0.195200297253766	0.498510729083241
**Gm20470**	66.1000614571586	0.372985339422599	0.287943888556748	1.29534035708173	0.195202843082033	0.498510729083241
**Chd1**	5127.95430065282	-0.144556522051661	0.111624118944167	-1.29502945616947	0.195310068242851	0.498577279368756
**Ptpn2**	7693.93001405645	0.0783705551108982	0.060515873182245	1.29504130056726	0.195305982492779	0.498577279368756
**Wdr55**	3608.50130735801	0.14884039132136	0.114935590733335	1.29498957086921	0.195323827222398	0.498577279368756
**Gm15545**	346.11820744661	-0.282984840837698	0.218558562674863	-1.29477810145868	0.195396788359503	0.498682733103448
**Dbndd2**	410.372455758874	0.39683480959743	0.306525885834646	1.29462087195957	0.19545104860238	0.498740432846592
**Zfp971**	373.338806925749	-0.220521931376416	0.170372618321176	-1.29435077977555	0.195544283789603	0.498897551760488
**Mvd**	2459.63870529337	0.138168772822753	0.106796992665271	1.29375153152308	0.19575125928734	0.499263935349728
**Dna2**	101.732569883944	0.375457167947382	0.290188691598678	1.29383803992827	0.195721370071151	0.499263935349728
**Ccdc174**	1890.0861288074	-0.142116424133434	0.109876796175771	-1.29341616319145	0.195867162850199	0.499478699632721
**2310009B15Rik**	223.50587311093	0.325138251688414	0.251445305348465	1.2930774397948	0.195984276969847	0.499696480940757
**Gm28042**	473.816396109104	-0.412108907035482	0.318786301123114	-1.29274346351642	0.196099799999394	0.499910135672295
**Shld3**	365.81182749691	0.170377949852484	0.13184435570066	1.29226578526662	0.196265116519499	0.500250637495591
**Ss18**	18681.1610976807	0.076009926675808	0.0588240223343119	1.29215792561454	0.196302459082384	0.500264895319606
**Zfp994**	881.705225469295	-0.217293131105626	0.168240339627828	-1.29156379252628	0.196508249809817	0.500708359031902
**Ints2**	1806.281682054	-0.103206792431079	0.0799237198002218	-1.29131617858948	0.196594062839639	0.500846022338591
**Praf2**	2273.8254285596	0.146026321028832	0.11310261551126	1.29109588110536	0.196670432154415	0.500959586074542
**Srek1**	3888.20918326241	-0.126049426513949	0.0976813289213366	-1.29041473847533	0.196906697554931	0.501480335117675
**Slc30a9**	3932.06359985993	-0.106510463950956	0.0825483032953485	-1.29028047457104	0.196953293684125	0.50151794579521
**Slc11a2**	9305.57509065087	0.106951632982057	0.0829040472907684	1.29006528000915	0.197027993541281	0.50162709552916
**Ube2n-ps1**	216.547810743525	0.256967200013739	0.199204306288912	1.28996809758244	0.197061734990168	0.501631948019398
**Lrp6**	1262.05697224833	0.253558462307984	0.196584704757001	1.28981785547054	0.197113906928723	0.501647082909317
**Parl**	4955.32342945281	0.0970519467866849	0.0752529703923291	1.28967595937685	0.197163189976353	0.501647082909317
**Gm9844**	2391.84474678875	-0.0984227778496064	0.0763135037785708	-1.28971640635434	0.197149141099634	0.501647082909317
**Klk8**	4619.2413238574	0.102045052974692	0.0791368574890339	1.28947567811664	0.197232766585266	0.501696678354324
**Trav14-1**	523.251551760694	0.150391360183873	0.11663339328236	1.28943654944333	0.197246361769692	0.501696678354324
**Zfp839**	751.505683175241	0.283897482595888	0.220327524747828	1.28852481287037	0.197563337171794	0.501935747148654
**Mtrr**	2602.60305491163	0.0982741371513791	0.0762417048534476	1.28898136971468	0.197404563521923	0.501935747148654
**Notch3**	21.8136614385777	1.49797005678118	1.16230031565792	1.28879777162691	0.197468400942543	0.501935747148654
**Kcnk6**	110.084181221092	0.480172243935723	0.372639117548378	1.28857176105078	0.197547006014846	0.501935747148654
**Gm13436**	172.300307039517	0.269307915505431	0.208968167427349	1.28875090795377	0.197484697955119	0.501935747148654
**Gm36940**	23.5629677348272	-0.925891338593852	0.718491385353325	-1.28866032003785	0.197516203022231	0.501935747148654
**Gm37486**	43.875582526486	0.508259122265964	0.39442666009455	1.28860235295486	0.1975363650012	0.501935747148654
**Yod1**	1434.39498853724	-0.221476706555548	0.171923167768381	-1.28823072207422	0.197665660590027	0.502114753170573
**Rpl8**	21971.3027103115	-0.0870524240675623	0.0676412055348824	-1.28697327877561	0.198103601961066	0.502174783799955
**Tmem175**	5614.28216927131	0.116968466770902	0.0908838010213877	1.28701116652654	0.198090396083213	0.502174783799955
**Ndufa2**	4155.70011502072	0.118470193096631	0.0920101763934901	1.28757706745374	0.197893226417196	0.502174783799955
**Hspa8**	144677.533284983	0.132649748090986	0.103034155837069	1.28743470564022	0.197942814212622	0.502174783799955
**Slirp**	734.77972753315	0.276947530983624	0.215141432273336	1.28728124590973	0.197996277839947	0.502174783799955
**Scamp1**	270.448793809928	-0.217210799022608	0.168762426267747	-1.28708032840198	0.198066291185431	0.502174783799955
**Rftn1**	12776.0555706717	-0.0691033030789877	0.0536713631321903	-1.28752651407025	0.197910834247683	0.502174783799955
**Chmp3**	5329.70284726503	0.112891736486794	0.087683630639251	1.28748930289229	0.197923795697317	0.502174783799955
**Zfp780b**	1450.65265291854	-0.130226482592134	0.101150926946082	-1.28744724862038	0.1979384448477	0.502174783799955
**Zfp568**	828.510902639082	-0.22658681141648	0.176032602405107	-1.28718662520839	0.198029247881833	0.502174783799955

**Gm37366**	24.6124763478711	0.680398848724854	0.528490028850707	1.28743933013173	0.197941203256235	0.502174783799955
**Gm47567**	45.8574249476144	-0.794358869035961	0.617218104585057	-1.28699865272097	0.198094757732703	0.502174783799955
**Gm50218**	214.308209591521	-0.356896533148632	0.277209251572426	-1.28746256167207	0.197933110620085	0.502174783799955
**Ubxn7**	2037.90197589758	-0.139130422494859	0.108132356193984	-1.28666781518444	0.198210095645627	0.502363917820193
**Hes5**	47.9497084700836	-1.07413822764907	0.834925897803818	-1.28650725827822	0.198266087338537	0.502416020939423
**1810037I17Rik**	1784.95824398224	0.200345447130395	0.155738025065711	1.2864260160347	0.198294423698818	0.502416020939423
**Trim39**	3358.87377339014	-0.0977520910546538	0.0760009926806232	-1.28619492465624	0.198375041908115	0.50253947513604
**Tfpt**	1198.84829645607	0.138175091060165	0.107464324580166	1.28577638765216	0.198521113141844	0.502722876814389
**Bend5**	676.375974219719	-0.187753636129834	0.146017192783343	-1.28583239104192	0.198501563159098	0.502722876814389
**Gm16575**	363.040572518623	-0.380940415545719	0.296287227063477	-1.28571325642771	0.198543153037757	0.502722876814389
**Eif2a**	2224.10572030202	-0.10637170234618	0.0827526587604889	-1.28541733811903	0.198646485648401	0.5028229195762
**Man1c1**	116.31038291511	0.786644677034638	0.611956611532693	1.28545825342817	0.198632195965753	0.5028229195762
**Chrna2**	20.2713047136807	-1.35275279572311	1.05278068966737	-1.2849331384968	0.198815649574455	0.502962271136488
**Ctdsp1**	4606.12376522066	-0.113706572732012	0.0884918007172895	-1.28493907695785	0.19881357422767	0.502962271136488
**Wdr11**	2889.498181329	-0.155229351340557	0.120793785682321	-1.28507729486018	0.198765274927509	0.502962271136488
**2610301B20Rik**	1386.35872923985	0.220065413641567	0.171271217502316	1.28489431470638	0.198829217929123	0.502962271136488
**Prdx6**	22092.4666951649	0.0698754853050167	0.0543899189285775	1.28471390804561	0.19889227633505	0.503041027000222
**Paip1**	1632.44476104599	0.191066951494712	0.148753368471143	1.28445462081605	0.198982931845597	0.503189545352444
**Slc7a5**	4000.88381330534	0.12520273612826	0.0975428790382898	1.28356613381395	0.199293805745254	0.503894816612319
**Acvr1b**	647.62689534828	-0.179610185561155	0.13994954593967	-1.28339241371017	0.1993546303585	0.503928464445641
**A330035P11Rik**	94.2581726112402	-0.421966747791	0.328802153679896	-1.28334544974363	0.199371076179103	0.503928464445641
**Rpl18a**	112432.069526452	0.0572335489191543	0.0446027132074903	1.28318536706289	0.199427141307957	0.503989328883637
**Lysmd3**	1482.36582709179	-0.229673858788193	0.179005479124438	-1.28305490933343	0.199472839524207	0.504023978893991
**Cbx1**	1821.94222478748	0.102818272231752	0.0801634531733321	1.28260782390991	0.199629507721439	0.504095238744984
**Ncoa1**	1080.41839810458	-0.182610214920046	0.142379574944541	-1.28255906783803	0.199646598315217	0.504095238744984
**Carnmt1**	1394.65481324389	0.541869159475315	0.422534205412253	1.28242673027295	0.199692992336497	0.504095238744984
**Spice1**	5137.74708996463	-0.0952920775950669	0.0743024949603913	-1.28248826161039	0.199671420111018	0.504095238744984
**Rnf31**	7005.14066509377	-0.0773392851746192	0.0602865791209445	-1.28286073455029	0.199540871440981	0.504095238744984
**Gm37132**	24.0796267119512	-0.914135249979088	0.712644190823847	-1.28273725057986	0.199584144635268	0.504095238744984
**Pfdn1**	1784.52982858464	0.154442568790344	0.120451480222652	1.28219735037593	0.199773425464598	0.504217502009557
**Ammecr1**	154.594739409183	0.448996037139728	0.350229715724889	1.28200440162657	0.199841102162656	0.504307534717645
**Chil5**	890.576745406055	0.228899975415726	0.178574685049644	1.28181648676626	0.199907029319239	0.504393124256726
**1110004F10Rik**	8603.54299070192	0.104980076549729	0.0819278508330616	1.28137227429094	0.200062937861928	0.504705685541211
**Gm6939**	24.1051443358043	-0.60359739310197	0.471226804708934	-1.2809063216911	0.200226572089063	0.505037633489253
**Tfpi**	474.820170735118	-0.231733360949231	0.180945392059908	-1.28068119508956	0.200305667530317	0.505075436593888
**Kremen2**	549.038486875997	0.225475645532389	0.1760513979643	1.28073760356116	0.20028584697161	0.505075436593888
**Timm22**	2792.5662537781	0.146913879048808	0.114753062491432	1.2802610741633	0.200453332808463	0.505286060640371
**Med4**	4831.97530630224	-0.0713991969455596	0.0557679497858872	-1.28029086992952	0.200442857492011	0.505286060640371
**Rbm42**	10894.9495884728	-0.207473305415802	0.16208226608512	-1.28004938743172	0.200527767151974	0.505330960328568
**Flt3l**	10139.7828767957	0.147884448625099	0.115532197439769	1.28002800866137	0.200535285583203	0.505330960328568
**Serpini1**	2706.66344900935	0.0760530483657437	0.0594231191998524	1.27985621404291	0.200595709344621	0.505384803306213
**Otud4**	2654.59316465205	0.248616719131027	0.194264459105204	1.27978488847715	0.200620799941636	0.505384803306213
**Nr1h3**	181.697160367856	-0.292887041232338	0.228908101421001	-1.27949617953307	0.200722384116943	0.505518270309096
**Kremen1**	2349.75818886193	0.292545709427633	0.228665523834614	1.279360808406	0.200770028279134	0.505518270309096
**Gm49599**	761.528903589506	-0.231012567171515	0.180566078846949	-1.27937965229519	0.200763395637015	0.505518270309096
**Osbpl11**	4098.31683386202	-0.152484263398488	0.119254601274005	-1.27864469604936	0.20102220288252	0.505661883667107
**Galk2**	3728.67295024499	0.106654631378015	0.0834355406745072	1.27828777180325	0.201147977840035	0.505661883667107
**Tnfsf8**	10279.5293360913	-0.134394178542781	0.105101240454869	-1.27871163043495	0.200998622560658	0.505661883667107
**Ugcg**	6168.28742550348	0.258425902539628	0.202156003489853	1.27834888936454	0.201126436820257	0.505661883667107
**Nfia**	212.388062467119	-0.317576893949281	0.248369868888265	-1.27864501185589	0.201022091622266	0.505661883667107
**Ret**	105.193593441549	0.874170612987607	0.683762645871957	1.27847085281009	0.201083455563911	0.505661883667107
**Polg**	8314.32853653656	-0.108287227292129	0.0846876145295194	-1.27866663730838	0.201014472971681	0.505661883667107
**E030022I16Rik**	70.5270562406446	-0.554464992075538	0.433708242563428	-1.27842853250466	0.201098368944615	0.505661883667107
**Gm10752**	21.7754772123826	-0.621546678526358	0.486012768955513	-1.27886903025638	0.200943180113473	0.505661883667107
**Mrto4-ps1**	119.652166280396	0.326337095255175	0.255195946779465	1.27877068336508	0.200977820473269	0.505661883667107
**Gmeb1**	1686.71042119284	0.117904215185901	0.0922476725505747	1.27812672044664	0.201204748807863	0.505723915611023

**Trav3-3**	521.038946290349	0.177484067648048	0.138890508819681	1.27787038262256	0.201295132576436	0.505870399363142
**Serac1**	274.164606367692	-0.319828130079466	0.250303680796742	-1.2777603951385	0.201333922834784	0.505887198550103
**Gm15464**	21.9298788679212	-0.59310670151657	0.464314389993032	-1.27738169287726	0.201467524813382	0.506142185663976
**Fam118b**	3378.99674745621	0.116476639255761	0.0911958720325856	1.27721394247036	0.201526725964842	0.506210205807113
**Cacfd1**	3843.05985487963	0.130981788489299	0.102581662043439	1.27685383410763	0.201653855416101	0.506448778359538
**Ms4a6b**	85748.2103241451	-0.104605707466718	0.0819362534837784	-1.27667183962015	0.201718127370976	0.506448778359538
**Stamos**	28.3853925490638	-0.731363247164279	0.572843431197339	-1.27672450679168	0.201699526249651	0.506448778359538
**Haus7**	1881.66749178287	0.13932927577214	0.109146268718777	1.27653723217174	0.201765674044557	0.506487450361651
**Mrfap1**	6760.16211330267	0.0954167262555594	0.0747582163166886	1.27633765165501	0.201836185848273	0.506583749667288
**Gm11868**	21.977176279697	0.658861670269837	0.516319813920009	1.27607279927458	0.201929785941193	0.506737957807832
**Gm1965**	31.3498917931364	1.4195791318819	1.11266074797681	1.27584183630381	0.202011435180945	0.506862131233464
**Cyth4**	52145.9027184089	0.0742190687547022	0.058184461268385	1.27558229700461	0.202103215340165	0.506930971683378
**1500011B03Rik**	3716.85080054111	0.10528814377247	0.0825394372682951	1.27561014779189	0.20209336508834	0.506930971683378
**Nr3c1**	8831.83465804718	-0.112688887891686	0.088376632332392	-1.27509823488016	0.202274474079985	0.507195885285855
**Cdan1**	2221.71704996192	0.549068198964371	0.430627072911857	1.27504338092732	0.20229388780159	0.507195885285855
**Mettl9**	4652.91951423993	-0.134664104105036	0.105622615988495	-1.27495520580275	0.202325097282921	0.507195885285855
**Scarb1**	2249.92788431088	0.114170963836987	0.089551478103835	1.27491992599615	0.202337585511638	0.507195885285855
**Dqx1**	135.869521230103	-0.493391451108898	0.387123782955765	-1.27450565641242	0.202484269309075	0.507482842612231
**Gm44260**	64.8289675663818	-0.531360336888872	0.417028022236597	-1.27415978916498	0.202606792672029	0.507709165415579
**C8g**	468.230492700357	-0.183062745638077	0.143824086361631	-1.27282397732591	0.203080510767938	0.507789402521493
**Anxa9**	33.202331451593	0.961880910710328	0.755184903997037	1.27370251393969	0.202768865180214	0.507789402521493
**Gstcd**	885.229007432374	-0.156824210819562	0.123148165462632	-1.27345957798412	0.202855007616306	0.507789402521493
**Slc35a1**	1949.37585202235	0.180520357740875	0.141780041144325	1.2732423850626	0.202932044420587	0.507789402521493
**Dennd1a**	3451.76482803914	-0.186467504770795	0.14641506005871	-1.27355413231415	0.202821476515755	0.507789402521493
**Mblac1**	60.8601043269294	0.568671955371063	0.446753124804912	1.2728997824456	0.203053606476758	0.507789402521493
**Mettl4**	1600.33346516582	-0.150675193354991	0.11838113854029	-1.27279729873277	0.20308997998924	0.507789402521493
**Klra1**	93.2598599035849	1.03294005886178	0.81135866869146	1.27309918377736	0.202982848566027	0.507789402521493
**A630014C17Rik**	198.745993856192	-0.270122651877214	0.212227345754076	-1.27279852140367	0.203089546010999	0.507789402521493
**Cox16**	1361.78035363725	0.130963486109624	0.102799757866259	1.2739668733462	0.202675156502055	0.507789402521493
**E230013L22Rik**	75.0284840348553	-0.421129251104027	0.330843710679134	-1.27289483677825	0.203055361683719	0.507789402521493
**Gm37370**	182.427513330391	-0.320857877602962	0.251937226796598	-1.27356279055182	0.202818406310956	0.507789402521493
**4930455C13Rik**	30.3326188217677	-0.589509049526007	0.462828531317079	-1.2737093969735	0.202766424919869	0.507789402521493
**Gm48586**	29.0566920777181	-0.719844225488497	0.565364834065369	-1.27323841547114	0.202933452605066	0.507789402521493
**H6pd**	1674.81647774767	0.275519089437582	0.216499705106585	1.27260722734907	0.203157452672828	0.507811561350591
**Gmfb**	7568.37413029379	-0.0997113466141552	0.0783530333925685	-1.27259076383905	0.203163297756814	0.507811561350591
**A430018G15Rik**	25.1394926310509	0.84724667390824	0.665975534715694	1.272188886443	0.203306015345762	0.508087689738807
**Zfp142**	2370.97117671859	0.228266592739469	0.179452964863679	1.27201349341245	0.203368325055863	0.508104720179611
**Baz2a**	5723.36152825581	-0.24601567987413	0.193410352107841	-1.27198817019348	0.203377322470826	0.508104720179611
**Fndc11**	24.8130710032403	0.9326979856876	0.733574351819397	1.27144301511406	0.203571087974555	0.508508185354322
**Dcakd**	2158.9788841165	-0.110215592093289	0.0866922543848646	-1.27134301530554	0.20360664567776	0.508516391812404
**Gm15700**	24.2303198464377	-0.692550900214224	0.544811601693726	-1.27117502281743	0.20366639023928	0.508584993819386
**Slc25a47**	127.189090353157	-0.406663994203205	0.320002268163499	-1.2708159743275	0.203794124410976	0.508823327260933
**Cdon**	629.982627210152	-0.179537572755205	0.141311433748567	-1.27050988014638	0.203903065726595	0.508962752389037
**Scaf1**	1023.82042576941	-0.199868371827498	0.157328748856983	-1.27038683825793	0.203946869238077	0.508962752389037
**A230060F14Rik**	95.7638581753607	-0.326571896969101	0.257064182185088	-1.27039050790035	0.203945562728442	0.508962752389037
**Txndc12**	1749.13909479733	0.167073005237291	0.131538347902251	1.27014675113181	0.20403236114084	0.509095473396075
**Elmo2**	10084.7028440285	0.108274595150019	0.0852884562792032	1.26951055129392	0.204259029727602	0.509419045808456
**Abcc3**	72.726086286261	-1.13206876769022	0.891716632204324	-1.26953869290489	0.204248999417061	0.509419045808456
**Ppip5k2**	4236.87999820659	-0.130187375216805	0.102539125504148	-1.26963609818907	0.204214284723889	0.509419045808456
**Prkdc**	5834.052334719	-0.149015950291603	0.117418395710605	-1.26910225088473	0.204404597603985	0.509459020048379
**Pusl1**	2415.11561570403	-0.113594832445128	0.08949241950079	-1.26932351453661	0.204325702989881	0.509459020048379
**Tmem171**	37.9452540352255	-0.832490230463961	0.655997584895708	-1.26904465752921	0.204425136945326	0.509459020048379
**Trav7n-6**	82.2388436236683	0.289658230049949	0.228223917503633	1.26918437479428	0.204375312603033	0.509459020048379
**Gm42672**	75.7399666285872	-0.431935664479332	0.340371566571748	-1.26901218227429	0.204436719157575	0.509459020048379
**Asap2**	118.676115084509	0.396510850666532	0.312493079995267	1.2688628198504	0.204489995010339	0.509511203570195
**Hax1**	2916.05235873362	0.145373058085914	0.114602591633464	1.26849712570954	0.204620476518578	0.509675126225615

**Gm43058**	25.6498498633787	0.890729014560705	0.702157389404142	1.26856033704436	0.204597918068778	0.509675126225615
**Heatr6**	5547.2012218239	-0.0745454601056932	0.058784590564796	-1.26811226189497	0.204757863248644	0.509726525678277
**Arrb1**	1259.0028197063	-0.308752689010292	0.243509017694482	-1.26793123282879	0.20482250927745	0.509726525678277
**Zscan26**	5431.16854876431	0.118656325273299	0.0935919284624331	1.26780511121669	0.20486755645875	0.509726525678277
**Khk**	1195.57784725076	-0.128955087894069	0.101687846158512	-1.2681465166747	0.204745632432474	0.509726525678277
**9130208D14Rik**	752.77251780115	-0.229831367096416	0.181282376773663	-1.26780865954426	0.204866288994964	0.509726525678277
**BC035044**	479.017692135618	0.309350781486574	0.243946799420397	1.26810756370476	0.204759540799061	0.509726525678277
**Gm45694**	26.4719158145294	0.795653076175152	0.627511284318942	1.26795022823964	0.204815725261735	0.509726525678277
**Rasgrp2**	23612.8807840292	0.0800816973938709	0.0631795527272204	1.26752555117992	0.204967433318382	0.50989451322983
**Ldlrap1**	11834.8111875216	-0.079991694804808	0.0631256287348098	-1.26718254388962	0.205090025985574	0.510061329086007
**Naca**	9132.37896900929	0.0627981475549701	0.04955949508868	1.2671264596744	0.205110075857901	0.510061329086007
**Gm37503**	39.0045547365564	-0.509347709945249	0.401989800486944	-1.26706625224884	0.205131601346578	0.510061329086007
**Gcat**	448.153817420756	0.238850795998422	0.188550447359173	1.26677395542547	0.205236127277047	0.51010196746757
**Phax**	1915.55180430898	0.172815565552149	0.136434522036998	1.2666557039375	0.205278425244258	0.51010196746757
**Ngdn**	5792.76406417495	0.123707801812927	0.09765596859115	1.26677154092697	0.205236990867751	0.51010196746757
**Gemin5**	4828.85741892669	0.0864669884666173	0.0682622872384685	1.26668753662696	0.205267038226849	0.51010196746757
**Trerf1**	534.487801533114	-0.238878344158972	0.188602861564721	-1.26656797345038	0.205309810095788	0.51010196746757
**Senp7**	4016.39850946433	-0.107137264197625	0.0846025898386273	-1.26635915522185	0.205384527109158	0.510126732922289
**Junos**	23.5219431241655	0.636798336073936	0.502831455250696	1.2664250205995	0.20536095775976	0.510126732922289
**Vipas39**	3435.90077194021	0.106926530051553	0.0844604985095815	1.26599454109808	0.205515036651372	0.510221140674309
**Tex264**	5953.54294077137	0.0761180207303694	0.0601256939588618	1.26598157490622	0.205519678863987	0.510221140674309
**Snord17**	52.9853709457525	-0.626494590058506	0.494808498359554	-1.26613546884407	0.205464585999456	0.510221140674309
**Pam**	533.456075219409	-0.29166925325927	0.230431575362116	-1.2657521123176	0.205601844669113	0.510302326224935
**Wdr92**	2329.76133990343	0.11270996989685	0.0890488547676099	1.26570937033372	0.205617152326085	0.510302326224935
**Pus3**	3670.78264727561	0.105420081953197	0.0833080499644185	1.26542491389754	0.205719048936053	0.510317544568355
**Wdr89**	1906.82360421849	0.128039441527065	0.101180717021155	1.26545299634805	0.205708987744353	0.510317544568355
**Gm47586**	324.10841835073	-0.340452621572835	0.26904296395608	-1.26542101888385	0.205720444443625	0.510317544568355
**Gm47112**	57.7560466732219	-0.555954553504566	0.439453377814619	-1.26510474505692	0.205833782153971	0.510518322902584
**Rab27a**	3435.55504580786	-0.131135678485112	0.103669766270942	-1.26493656928276	0.205894066937375	0.510587474462106
**4921507P07Rik**	71.2520784947118	-0.481392298709433	0.380760403244733	-1.26429191325344	0.206125271056936	0.51067860000694
**Etv6**	1421.01798925944	0.153276798195666	0.121224623434341	1.26440317035661	0.206085355543263	0.51067860000694
**Ddx6**	16685.3471779801	-0.213155143026231	0.168569378787016	-1.26449503794842	0.206052400602399	0.51067860000694
**Cpne2**	107.221870761602	0.334724141616027	0.264739255130508	1.26435402052868	0.206102988247565	0.51067860000694
**Eea1**	447.903566102007	0.157359064002504	0.124421940192861	1.26472118790777	0.205971291910931	0.51067860000694
**Abhd17c**	132.996717806295	0.680632290193757	0.538314800965793	1.26437595431638	0.206095119275009	0.51067860000694
**Ctsf**	193.712374490758	-0.468070537238069	0.370379626085403	-1.2637588686645	0.206316588420245	0.511072234513096
**Rpl36-ps12**	83.2162266889756	-0.476387742977706	0.37706431516907	-1.2634124307523	0.206440999086754	0.511300034990566
**Mpc1**	3132.73632257351	0.11945894408172	0.094569487059573	1.26318697283901	0.206521993411275	0.511383530231573
**Sec31b**	575.421257536827	-0.193463883286837	0.153172282174911	-1.26304759934252	0.206572073994032	0.511383530231573
**Gm38056**	804.53185534064	-0.100146070717153	0.0792880263246763	-1.26306676252812	0.206565187631935	0.511383530231573
**Tbc1d23**	1430.25925144122	-0.118434463579054	0.0937776832813624	-1.26292801693249	0.206615050143717	0.511400131559691
**Cul5**	3222.49124068676	-0.107079013134661	0.0847916652546056	-1.26284833318384	0.206643690908203	0.511400131559691
**Uhrf2**	7368.46887667012	-0.0986642406661904	0.0781393902782844	-1.26266970237174	0.206707906731773	0.511471748248038
**Mier2**	2725.5639455147	0.144359222474644	0.114351436596668	1.26241721810472	0.206798696772174	0.511471748248038
**Mir17hg**	355.89047716415	-0.200625978064701	0.158923406780371	-1.26240672868259	0.206802469257362	0.511471748248038
**Gm45014**	30.0484316795165	-0.582739490343199	0.461585636373225	-1.26247318898808	0.206778567877735	0.511471748248038
**Bdkrb1**	23.1103444235304	0.971837841568935	0.769947845115275	1.26221256119424	0.206872309958944	0.511501450911542
**Mei4**	32.4506218667545	0.629302365959616	0.498578619195473	1.26219284528303	0.206879402593086	0.511501450911542
**Csk**	6761.35073119855	0.0935171732817933	0.0741038529814631	1.26197450630788	0.206957960013937	0.511571001114095
**Nbl1**	19.4923939717593	-0.981450137711283	0.77773479720474	-1.26193419818519	0.206972465069642	0.511571001114095
**S1pr1**	71521.6891578529	-0.0866299068132542	0.0686577189035563	-1.26176500176103	0.207033359197946	0.511641254843481
**Ncoa7**	1454.05096721747	-0.178770032954438	0.141701851219337	-1.26159278383543	0.207095354122394	0.511714206508792
**Inafm2**	786.081494419186	0.210937940099043	0.16721220063252	1.26149849892005	0.2071293004663	0.511717840615787
**1110003F10Rik**	17.7938098990701	0.74612596281662	0.591589518071388	1.26122241862741	0.207228723646187	0.511883210298317
**Vopp1**	4461.40365887668	0.114371934331348	0.090691684821417	1.26110717378954	0.207270236338372	0.511905503759206
**Chd7**	10205.8291950058	0.1796875587119	0.142525392739592	1.26074066703473	0.207402296955237	0.512151385852324

**Cetn3**	3144.96528743983	0.106677255515003	0.0846602244691356	1.2600634617251	0.207646469827533	0.512423029525223
**Pcx**	552.202143821488	0.281625091435268	0.223530694338598	1.25989449578084	0.207707424609862	0.512423029525223
**Mfsd2a**	111.58978885691	0.579304743871135	0.459709249644316	1.26015463974099	0.207613582577296	0.512423029525223
**Idua**	6517.32885215782	0.116670264562933	0.0925805330479982	1.26020299000056	0.207596144521805	0.512423029525223
**Rpl7**	26372.6539018969	-0.0800947859171027	0.0635688190303444	-1.25996970116544	0.207680292523308	0.512423029525223
**Ighv1-52**	58.458648374699	2.74962391633203	2.18236329772528	1.25992950816118	0.20769479276186	0.512423029525223
**Eps15l1**	5587.46492735459	0.103835303640095	0.0824225352371016	1.2597926445892	0.207744173887871	0.512433460856478
**Pno1**	1817.19789842506	0.124083888022188	0.0985524298174621	1.259064725771	0.208006954072364	0.512680187241102
**Hcfc1**	8794.35490838705	-0.202067342833563	0.160501493646914	-1.25897484342476	0.208039418494612	0.512680187241102
**Nck2**	2654.96587286153	0.203225144131073	0.161411880763326	1.25904699932874	0.20801335635925	0.512680187241102
**Morrbid**	76.7445746187536	0.509794053798219	0.404899599752128	1.25906287413054	0.208007622825562	0.512680187241102
**5930420M18Rik**	34.3001083935704	-0.634681666265442	0.504000582994326	-1.25928756370622	0.207926483534656	0.512680187241102
**AV356131**	31.182657528942	-0.905653288002682	0.719203068869651	-1.25924558334556	0.207941641624938	0.512680187241102
**BC034090**	16.9703410576948	-0.703281409529229	0.558963124332761	-1.25818927745679	0.208323311861014	0.512990119233311
**Mapkap1**	5706.44398409642	-0.0931255284678376	0.0740050877378486	-1.25836657065687	0.208259215868624	0.512990119233311
**Ypel3**	16268.9863459647	-0.0714570387439289	0.0567849867933516	-1.25837906776259	0.208254698387133	0.512990119233311
**Scfd2**	3893.63372890803	0.109062388111918	0.0866718877347664	1.25833636444693	0.208270135169764	0.512990119233311
**F420014N23Rik**	19.7181982211407	-1.11892085862503	0.889319538837354	-1.25817640314959	0.20832796680675	0.512990119233311
**Zfp69**	618.776596264026	-0.142523987532285	0.113287006777975	-1.25807885286977	0.208363240378888	0.512996809164085
**Bcr**	360.890471376663	-0.238544624423575	0.189680646055446	-1.25761183011705	0.208532172870735	0.513252334883501
**Gm13204**	82.0704440574059	-0.445993400361947	0.354623736119907	-1.25765242124443	0.208517486221426	0.513252334883501
**H2-Ke6**	9400.84019878098	0.128890091139156	0.102501999802117	1.25743977081406	0.208594435555188	0.513325397632844
**Sgsh**	5281.41939912144	-0.119343218790703	0.0949384957485404	-1.25705824439017	0.208732545660111	0.513399763026546
**Pole2**	1384.1667048332	0.14134957219879	0.11248858644426	1.25656812541449	0.20891006279368	0.513399763026546
**Larp4**	4560.69726247964	-0.138393944048315	0.110116434668355	-1.25679644882369	0.208827352294539	0.513399763026546
**Crat**	440.998614972831	-0.275127525327324	0.218957278012555	-1.25653519181741	0.20892199499736	0.513399763026546
**Larp7**	2618.34011842272	0.115005432801672	0.0915072202934241	1.25679080222194	0.208829397498342	0.513399763026546
**Gpr19**	367.750723729719	-0.279924052176007	0.222788479925855	-1.25645658280521	0.208950477901202	0.513399763026546
**Rps16**	15998.8016879888	-0.078112055169362	0.0621513640515599	-1.25680355308954	0.20882477914422	0.513399763026546
**Tmco3**	1967.94571661855	0.149755753743116	0.119182704118297	1.25652253698215	0.208926580114392	0.513399763026546
**Rnaset2a**	28706.7894985981	0.0961526014805048	0.076491637100423	1.25703416903301	0.208741263006315	0.513399763026546
**Gm38352**	28.4006098362855	-0.765546626542717	0.608922622424074	-1.25721495367529	0.208675809892418	0.513399763026546
**Cers6**	573.749671710071	-0.287048897426098	0.228500255807417	-1.25623009222373	0.209032559406356	0.513418994256316
**B3gnt2**	10701.270026548	0.092507403764646	0.0736479711006887	1.25607538649196	0.209088639090105	0.513418994256316
**Hist1h2bq**	19.9831125824456	0.709639244000641	0.564858458550661	1.25631338835124	0.209002369689515	0.513418994256316
**Gm38365**	208.457243358344	-0.364240837516511	0.289974868784398	-1.25611174183281	0.209075459568278	0.513418994256316
**Pgam1-ps2**	379.672692966853	0.195693968093083	0.155825211236286	1.25585562528994	0.209168319637564	0.513534623329558
**Btnl2**	17.2483104995421	-1.67804319505092	1.33690800567358	-1.25516728744957	0.209418038045468	0.513971612368461
**Wdr20**	5153.03300082603	0.125751554639249	0.100192833566304	1.25509530136237	0.209444165959122	0.513971612368461
**3110053B16Rik**	118.875664152733	-0.467325661490482	0.372299761870004	-1.25524029116532	0.209391543186536	0.513971612368461
**Gm15459**	10390.4101947759	0.13968166551788	0.111308968345389	1.25490036961308	0.209514929814875	0.514065205402208
**Nfyb**	3442.26080912815	0.111907024456237	0.0892167298653346	1.25432779956351	0.209722883521336	0.514202417893877
**Rpl36-ps2**	49.3553902819262	-0.424046472681385	0.33798876951428	-1.25461704923148	0.209617811257995	0.514202417893877
**Zfp90**	761.627741220573	-0.188089744816345	0.149930347807301	-1.25451416319055	0.209655181069311	0.514202417893877
**Ifi27**	9114.96104428174	0.0981787409233797	0.0782739089045902	1.25429715083032	0.209734019153643	0.514202417893877
**B130034C11Rik**	115.895398125341	-0.401285979958714	0.319886809639541	-1.25446241566164	0.209673978400351	0.514202417893877
**Tango2**	4089.71174053831	0.0929003134134125	0.0740810948582827	1.25403537287254	0.209829148622881	0.514326213460363
**Lgals3bp**	24673.020107722	0.100269536182917	0.0799611243150645	1.25397856823314	0.20984979541389	0.514326213460363
**Xlr**	75.4024673872267	-0.597894727097265	0.476842217594867	-1.25386281884387	0.209891871414084	0.514349334039147
**Gtse1**	290.450724678988	0.283663587671388	0.226348071796592	1.25321848522881	0.210126204419051	0.514843508479394
**Arid1a**	8808.0552678447	-0.165763486851972	0.132297208996886	-1.2529628410821	0.210219230227923	0.514911302767198
**Xrcc5**	3124.88201358718	-0.0959016172923398	0.0765349490219189	-1.25304345946418	0.210189890957998	0.514911302767198
**Rasgrf2**	670.005197364215	-0.201707088563832	0.161000245211836	-1.25283715126295	0.210264978158923	0.514943310669903
**Cct6b**	41.6818992892158	-0.700467287786914	0.559314273693836	-1.2523679811725	0.210435807905594	0.51512148907386
**Clip1**	11311.4375215711	-0.15435575060669	0.123239257995224	-1.25248847743526	0.21039192437243	0.51512148907386
**Socs6**	1564.78607261047	0.178314616392147	0.142380164952717	1.25238383065059	0.210430035306006	0.51512148907386

**Apba3**	3637.30806389081	-0.134152793356442	0.107170657870401	-1.25176793743927	0.210654436442198	0.515274587266086
**Dock9**	778.46743941429	0.212072091590681	0.169404489703768	1.25186818815443	0.210617898230226	0.515274587266086
**Dcun1d2**	2722.55927816137	0.116188771253521	0.09280177726136	1.25201019508814	0.210566149046251	0.515274587266086
**Zfp758**	1224.24736874242	-0.202245249126703	0.161570314022422	-1.25174757721048	0.210661857661238	0.515274587266086
**Tapt1**	7938.12400714718	0.269284308539159	0.21506503534357	1.25210640636668	0.210531093626008	0.515274587266086
**Rad51c**	104.899068970461	0.477402453354054	0.381480124837838	1.25144777478772	0.210771156300451	0.515332951649318
**Prkcg**	20.0112754211314	1.3489612588359	1.07787367313302	1.25150218662908	0.210751316389054	0.515332951649318
**Gm42427**	235.294206201251	-0.193950989027606	0.154985594589636	-1.2514130073904	0.21078383406612	0.515332951649318
**Tnfaip8**	10961.2002902093	0.121514395630864	0.0971224054012096	1.25114689168675	0.210880890158498	0.515410297227773
**Kcnj13**	72.4961316201658	-0.550960262314087	0.440334628592108	-1.25123082887141	0.210850273612465	0.515410297227773
**Prpsap2**	2411.98332881544	0.114512124779099	0.091553155481353	1.25077201519747	0.211017667653768	0.515584647111244
**2010016I18Rik**	3246.67609427564	-0.160866884478847	0.128611887437661	-1.25079327956228	0.211009907418204	0.515584647111244
**E2f2**	904.592776736059	0.3812529509461	0.305032090923968	1.24987816787163	0.21134405577625	0.51590213584297
**Zfand2b**	4079.43115355634	0.0939546499407057	0.0751709746489031	1.24987936340502	0.211343618983905	0.51590213584297
**Ankrd29**	75.9927083925612	0.368650930218476	0.294884278665138	1.25015457550758	0.211243086638594	0.51590213584297
**Rpl13a**	34942.4049554217	-0.086539488509018	0.069232560287994	-1.24998249593877	0.211305941605213	0.51590213584297
**Gm17056**	19.9014659492059	-1.15400923385229	0.923052640117585	-1.25020955869352	0.211223005952241	0.51590213584297
**Gm38021**	64.09595785646	-0.503374511248878	0.402660526657795	-1.25012132534331	0.211255230768376	0.51590213584297
**Galnt11**	2638.33219235362	0.146462413335946	0.11722939589763	1.24936593091244	0.211531263411389	0.516155378325175
**Alg11**	1585.20219329869	0.187009087993728	0.149688050482115	1.24932542972809	0.21154607052256	0.516155378325175
**Gm27219**	94.9893971912888	0.290385097130927	0.232430980003666	1.24933903873893	0.211541095025554	0.516155378325175
**Clhc1**	48.6429085344912	-0.549994145710888	0.440340365405714	-1.24902050531784	0.211657574025708	0.516347483189825
**Rhobtb1**	17.7947282456534	1.22572026421711	0.981652453154321	1.24862955344179	0.211800597839611	0.51661641178928
**Il20rb**	182.424925859847	-0.326268175715412	0.261325933414829	-1.2485105150184	0.211844160109628	0.516642691664975
**Kifc5b**	125.055906998893	0.373776587295333	0.299507203411578	1.24797194537486	0.212041331309593	0.516803400389625
**Thoc3**	3041.34146473514	0.164667483502876	0.13195458859278	1.24791024896489	0.212063926925486	0.516803400389625
**Lamc1**	119.945845656479	0.904052282961731	0.724525805046676	1.24778479477827	0.212109878475179	0.516803400389625
**Atg2b**	3112.02798211583	-0.158274386201063	0.126852558709423	-1.24770353717197	0.212139645473129	0.516803400389625
**Ap5b1**	1648.22141048143	-0.161515671403681	0.1294303274727	-1.24789664491692	0.212068909489069	0.516803400389625
**Zfp143**	4603.31268872029	0.0816214475227036	0.0654015615940492	1.24800456645564	0.212029384908933	0.516803400389625
**Gm16549**	18.5049998008085	0.724923848806765	0.58094550144325	1.24783451632869	0.212091665529756	0.516803400389625
**Xab2**	19256.9359949333	0.0792838345970782	0.063572031765647	1.24714960958541	0.212342645496567	0.516898357035286
**Wrnip1**	8660.18925057853	0.109397531054131	0.0877179524445269	1.24715098797267	0.212342140179467	0.516898357035286
**Actr1a**	15340.0162510456	0.0657746546409544	0.0527349294971637	1.24726922493548	0.212298797713483	0.516898357035286
**A130006I12Rik**	27.9721885896339	-0.555334411867369	0.445270843823455	-1.24718341560121	0.212330252453638	0.516898357035286
**Rps2-ps11**	21.4656320210702	-1.16939650923802	0.937502225837074	-1.24735331502162	0.212267976456193	0.516898357035286
**Lnx2**	3612.1997531822	-0.0996648798876493	0.0799507799211368	-1.24657795691247	0.212552288395028	0.51699686027846
**Cand1**	8021.88567508565	0.0645042080552257	0.0517501204035249	1.24645522662073	0.212597316913759	0.51699686027846
**Marchf8**	47.6995434651999	-0.844930328025018	0.677766538288016	-1.24663918959358	0.212529825311446	0.51699686027846
**Focad**	5312.72595759796	-0.136811462033954	0.109764142601699	-1.24641307070927	0.212612785086274	0.51699686027846
**4930430F08Rik**	365.586713093321	-0.260781112544926	0.209187252893562	-1.24663959652272	0.21252967603606	0.51699686027846
**D530018E20Rik**	28.0292204873849	-0.565317504725525	0.45345842817167	-1.24667989302761	0.212514894288812	0.51699686027846
**Gm47272**	22.3425049201049	-0.589730884807593	0.472974167416577	-1.2468564362167	0.212450142665951	0.51699686027846
**Tmem186**	4416.73885533112	0.0972788910174566	0.0780764280263531	1.24594443517091	0.212784795199501	0.517335290535186
**Lzts2**	230.874217254662	-0.313064681689504	0.251336717937354	-1.24559867041606	0.21291177066894	0.517564142306462
**Lcp1**	148001.24181104	0.0881745975514553	0.0708018156565813	1.24537198281947	0.21299504702285	0.517606871674744
**Zkscan8**	481.784832485413	-0.213935688852863	0.171775696929094	-1.24543630255898	0.212971416021108	0.517606871674744
**Pdpr**	1591.27726145772	-0.177601221414775	0.142626717252147	-1.24521705916289	0.213051973575294	0.517665373573771
**Dusp3**	63.7468451013483	0.487559678282769	0.391635668265854	1.2449317510881	0.213156838498117	0.517754920061095
**Tnfrsf1b**	9647.27552542873	0.101632227105951	0.0816382429763091	1.24490953505018	0.213165005561461	0.517754920061095
**Gm45807**	43.3774737395008	0.605128591523314	0.486106170963123	1.24484861059133	0.213187403779678	0.517754920061095
**Zmym1**	1322.00138238771	-0.201676150215162	0.162073262513405	-1.24435176467483	0.213370127226517	0.518118831053818
**Tlcd2**	37.6985628248144	0.578401597389056	0.464951364119926	1.24400451751308	0.213497900300982	0.518349216493464
**Caap1**	1207.55435128699	0.128756643540401	0.103525286998038	1.24372167683864	0.213602015159461	0.518522100272319
**Ddx3x**	8310.69255138808	-0.136371161928579	0.109690120253247	-1.24324015338603	0.213779350265477	0.518872646662526
**Ppt2**	742.645365455337	0.138180538375815	0.111174869414488	1.24291163195022	0.213900398839316	0.518947056720584

**Plcl1**	76.9042006524424	0.569517432373373	0.458220742846916	1.24288880689899	0.213908810903103	0.518947056720584
**H2ac12**	58.7944876045597	0.447679228713255	0.360142615472073	1.24306096940636	0.213845367120354	0.518947056720584
**Fam107b**	28135.5979933372	-0.0788809667617256	0.0634734144764927	-1.24274024664199	0.213963567908935	0.518999990692901
**Pdxk-ps**	20.466009690045	-1.25161101252696	1.00734719561709	-1.24248225236805	0.214058684620541	0.519150791683218
**Plcl2**	5210.14655437606	0.119318984004255	0.0960825610408881	1.24183808915625	0.214296306303201	0.519647106558869
**Clk2**	5226.5309740513	-0.0945384259963731	0.0761441273346499	-1.2415721251999	0.214394471688316	0.519731766616744
**2810455O05Rik**	111.36426683285	0.269132883166964	0.216769104605844	1.24156476844952	0.214397187472795	0.519731766616744
**Pik3r6**	17.4227250210791	1.77345010366701	1.4287062621847	1.24129791448884	0.21449571483576	0.519796992934097
**Amy2a1**	18.5205173488472	3.84853294556534	3.10059533919622	1.24122386978851	0.214523059278444	0.519796992934097
**Trav12-1**	505.332325329404	0.163736171658552	0.131902471564638	1.24134271114331	0.214479172811834	0.519796992934097
**Tmem41a**	1309.40508370672	0.146526747556564	0.118137258421672	1.24030936145107	0.214860991164171	0.520092344501449
**Arhgap1**	31407.9160854587	0.0737643783035585	0.0594569682679366	1.24063470527369	0.214740725188278	0.520092344501449
**Dffb**	3017.50344368125	0.116532286725057	0.0939458667839972	1.24041951726296	0.214820265742371	0.520092344501449
**Vps33a**	4640.04784153984	0.0884805781731874	0.071339842444146	1.24026876345376	0.214876001948622	0.520092344501449
**Yif1b**	2584.65775090871	0.104275359247729	0.0840673888184481	1.24037823362067	0.214835527959621	0.520092344501449
**Gm28036**	6185.68279232883	-0.444332740631652	0.358155551071487	-1.2406138598225	0.214748429422664	0.520092344501449
**4932422M17Rik**	39.5605458439015	-0.557713762532669	0.449532047982341	-1.24065406467878	0.214733570357548	0.520092344501449
**Gm50105**	88.6138210229999	-0.351559340131754	0.283500772026026	-1.24006484222018	0.214951411625568	0.520194961293822
**Ptch1**	1963.09577587517	-0.201929796264018	0.162891556340703	-1.23965784845018	0.215101974151324	0.520479392921132
**Fyn**	21721.7886617847	0.515902036292338	0.416206758449749	1.23953305855466	0.215148153915202	0.520511202401632
**Ankrd26**	2153.84249502893	-0.136600448349828	0.110226614661646	-1.2392691979985	0.215245821706743	0.520569837465703
**Coq4**	573.289671617483	0.17717851217438	0.142978671756086	1.23919539885388	0.215273144122186	0.520569837465703
**Nfic**	1889.52917685273	-0.183254585774709	0.147880988704545	-1.23920314152644	0.21527027746057	0.520569837465703
**Gm28935**	659.004673648789	-0.209633731793211	0.169180806540427	-1.2391106064571	0.215304539618201	0.520569837465703
**Gm48611**	75.1461597271119	-0.384337133480926	0.310239540870134	-1.23883993769127	0.21540478034825	0.520732298856608
**Sfr1**	3923.7545752607	0.117965663007294	0.0952348641144313	1.23868148607373	0.215463477657799	0.520794296280708
**Trmo**	2376.65940180897	0.139102848687815	0.11233677246061	1.23826638099818	0.215617305103457	0.521086177379626
**Rcl1**	5207.20809191811	0.0731577746469455	0.0590853978430097	1.23817012862173	0.215652985095599	0.521092483691359
**Wdr54**	20.5128713916351	0.63531053066051	0.513147083028857	1.23806711890591	0.215691174690605	0.521104851211264
**3110040N11Rik**	1278.54510515252	0.126957658100717	0.102562689656984	1.2378542189691	0.215770120180623	0.521215665136605
**Zfp646**	5220.82710799753	-0.14415081096787	0.116465332747899	-1.2377143272316	0.215822004806184	0.521261086715366
**4833408A19Rik**	66.3653317751618	0.394877544192247	0.319097678685338	1.23748171976405	0.215908296777279	0.521389583497254
**Ly6c2**	49131.7136456918	-0.140261884008857	0.113364255337714	-1.23726728139319	0.215987870442785	0.521402429871615
**Acoxl**	475.33504475722	0.190097933489974	0.153645063921601	1.23725376291277	0.215992887580107	0.521402429871615
**Pgk1**	9388.06371869353	0.0657526329766208	0.0531463300215367	1.23719987720649	0.216012887110611	0.521402429871615
**Gdf9**	20.6929834449418	-0.848244236636763	0.685778267563945	-1.23690743314153	0.216121450169692	0.521506100575526
**Gm7324**	610.58921275598	0.140378243705266	0.113491452450212	1.23690586977772	0.216122030637697	0.521506100575526
**Mia2**	6474.97861894375	0.129048387906949	0.1043567658767	1.23660777356231	0.216232732563509	0.521693334405637
**Gm9531**	674.220858196984	0.260905363673926	0.211000507303185	1.23651533832112	0.216267067888619	0.521696293435543
**Mllt10**	5811.83690484986	-0.0917633417311539	0.0742204299360861	-1.23636230361606	0.216323921679208	0.521753564043973
**Gm46436**	30.7782929537096	-0.722048409027912	0.584071754202492	-1.23623237013715	0.216372201597986	0.521790140890643
**Gm36266**	198.636534421998	-0.25951996260214	0.20994680453778	-1.23612247003949	0.216413043684857	0.521808772661406
**Fbxl6**	5581.0591418004	-0.0961801228956909	0.0778531758548634	-1.23540397472023	0.216680194267326	0.521893888882644
**Smim8**	926.922464915466	0.141887143491584	0.114839291415766	1.23552785586157	0.216634115917617	0.521893888882644
**Capn5**	43.9061145753976	0.802187396037237	0.649157715614793	1.23573574917386	0.216556804573517	0.521893888882644
**Nme1**	13720.9695600694	0.135024825931279	0.109268431318508	1.23571670519999	0.216563885818322	0.521893888882644
**Mrps34**	3945.2565167162	0.124653881081912	0.100887005437408	1.23557915651734	0.21661503638921	0.521893888882644
**D11Wsu47e**	1732.86943449016	0.143317991540802	0.115988515191007	1.23562226229717	0.216599005600269	0.521893888882644
**Gm47726**	18.2127136198231	-0.659834803136294	0.534079262296112	-1.23546231752106	0.216658492426443	0.521893888882644
**Ttc39b**	2915.79007331541	-0.111900070763967	0.090591318053769	-1.23521848636257	0.216749200984354	0.521980308713199
**Fn3k**	44.4205618139967	0.591860948240705	0.479300754890073	1.23484251214344	0.216889122135562	0.522109992598451
**Car1**	59.4346856916991	0.623833946145426	0.505244197916247	1.2347176844747	0.216935591898335	0.522109992598451
**Rfxap**	3169.7574730047	0.151533462919083	0.122712856610075	1.23486215792846	0.216881809225299	0.522109992598451
**Septin11**	7602.95771299057	0.111896919683354	0.090623968490082	1.23473868500473	0.216927773501859	0.522109992598451
**Syngr3**	33.9820213209256	1.58354033991322	1.28272732084319	1.23451049508503	0.217012738379993	0.522198559355205
**Ints13**	6167.19819488188	-0.093825200455625	0.0760062338997596	-1.2344408562509	0.217038672667211	0.522198559355205

**2900005J15Rik**	1022.74112207343	-0.259102500537082	0.209964263314254	-1.23403143205033	0.217191191945796	0.522485742212199
**Ptpmt1**	2415.2178787276	-0.15929403232334	0.129115208925034	-1.23373561991313	0.217301436261263	0.522671154200691
**Ifit2**	2529.78020114881	-0.125795273636832	0.101974996036824	-1.23358939471208	0.217355946849542	0.522722474741795
**C1galt1c1**	1188.91403245672	0.181014899932436	0.146758617711665	1.23341922099644	0.217419397470536	0.522795276353308
**Adipor1**	13782.2325863101	0.0538983505267364	0.0437049176146587	1.23323308836667	0.217488813751018	0.522882398272017
**Srr**	800.088494885881	0.203250976703534	0.164920461511571	1.232418190203	0.217792909399833	0.523337504809778
**Pde4d**	2023.64996137173	-0.164523551869498	0.133509634193073	-1.23229722606815	0.217838075643338	0.523337504809778
**Mul1**	1257.2561586583	0.156285298031618	0.126796818803691	1.23256481910309	0.217738169334397	0.523337504809778
**Tgif2**	1340.64014500527	-0.172133460835191	0.139696952974335	-1.23219194957542	0.217877389830053	0.523337504809778
**B9d2**	2030.45741293933	0.132002076977204	0.107116943601356	1.23231743307072	0.217830530174899	0.523337504809778
**Gm37584**	53.0081478319522	-0.411054886515095	0.333577973363373	-1.23226027897029	0.217851872493091	0.523337504809778
**Cryzl1**	2280.73307546288	0.101637603384185	0.0825028898897961	1.23192779695291	0.217976056769948	0.523494699973187
**Rapgef1**	2248.75430687352	0.253533687189623	0.205825058104765	1.2317921322319	0.218026743072972	0.523536633739839
**Notch2**	3361.92005135695	-0.250047534591423	0.203022255589552	-1.23162622671743	0.218088739287855	0.523605708511157
**Gm20696**	2774.46221808951	-0.32712906048873	0.265648708157878	-1.23143478753269	0.218160292767572	0.523617933456
**Gm36043**	31.6789401817964	-0.528404413787945	0.429075452856781	-1.23149532388729	0.218137664505873	0.523617933456
**Tsr2**	3018.64417450785	0.124897433150401	0.101440872249636	1.23123382499158	0.218235423905276	0.523718485299334
**Gm25395**	42.2571244929738	-0.489463824133848	0.397582386417707	-1.23110037279068	0.218285326140861	0.523758471752938
**Dgke**	1392.86237372541	-0.154553731189955	0.125550933770284	-1.23100423508388	0.218321280321562	0.523764983865233
**Gys1**	1202.02496987889	0.122839504006506	0.0998196038093389	1.23061502268769	0.218466883895185	0.523875009821402
**Fscn1**	128.009792921037	0.382915405824436	0.311122922143109	1.23075279438364	0.218415335805904	0.523875009821402
**Cryba4**	17.9555226992107	-0.927078281268206	0.753308993762739	-1.23067464870889	0.218444573396343	0.523875009821402
**Btg1**	63169.9918804331	0.103087658025173	0.0837799226113646	1.23045778525449	0.218525725884464	0.523936375952753
**Ankhd1**	13657.8759141592	-0.115268126637443	0.0937329864888072	-1.22974985600408	0.218790790893551	0.524306879313586
**Col9a3**	88.8309313811166	-0.493332077067605	0.401210838746037	-1.22960804999558	0.218843914078996	0.524306879313586
**Rps20**	9557.16603328572	-0.110664095593568	0.0900065198834857	-1.22951199242925	0.218879904304422	0.524306879313586
**Sppl3**	989.844985207281	-0.206403534984216	0.167874151024709	-1.22951349998986	0.218879339428567	0.524306879313586
**Gm44065**	24.9955827302371	-1.51498387375861	1.23199084816522	-1.22970383750403	0.218808029269863	0.524306879313586
**Gm48879**	17.6008998149146	-0.972102567291188	0.790457673111923	-1.22979711673132	0.218773088198458	0.524306879313586
**Rxylt1**	1325.46875539572	0.12193787056238	0.0992598936961763	1.22847069467572	0.219270323828337	0.52516225757153
**Gm42778**	25.3528838120737	-0.833235277276026	0.67843956242368	-1.22816433979668	0.219385282289195	0.525357734503169
**Arhgef33**	23.0315273322189	0.810593628071102	0.660194270555492	1.22781075847427	0.219518016066491	0.525595711770202
**Dcaf6**	490.220812736797	-0.24457273482483	0.199336633847482	-1.22693320391855	0.21984769748795	0.525745965900385
**Rbks**	688.284587830187	0.144661777448243	0.11789843265722	1.2270033976519	0.21982131390179	0.525745965900385
**BC031181**	5510.97204390566	0.123057743188336	0.100285558721113	1.22707341672744	0.219794998227724	0.525745965900385
**Suv39h1**	5313.73807130496	0.0982174054669927	0.0800453669601518	1.22702174025746	0.219814419875705	0.525745965900385
**Mfsd9**	242.30980924023	-0.315690941280065	0.257201331158038	-1.22740788260574	0.21966932517563	0.525745965900385
**Cpped1**	568.786138571669	0.227207048103739	0.185158641516409	1.22709394626663	0.219787282920924	0.525745965900385
**Adh1**	184.004960393912	-0.369089782703742	0.300773251755534	-1.2271363246215	0.21977135711826	0.525745965900385
**Trav12-3**	570.647177498683	0.140182719433039	0.114243196180465	1.22705530062025	0.219801806692239	0.525745965900385
**Gm18994**	21.278994955534	-0.782265846230063	0.637695582403672	-1.22670733154754	0.219932611130792	0.525869219057342
**Pgam1**	7277.37875266953	0.122632107783634	0.0999853091475673	1.22650126132672	0.220010100951861	0.525894896950618
**Prpf40a**	10471.9639723935	-0.108673607311218	0.0886019760973941	-1.22653706043487	0.219996637792724	0.525894896950618
**Eif2s1**	13183.3122920406	0.118001030628697	0.0962471982112169	1.2260204226386	0.220190989786993	0.52624744821381
**Tesk2**	811.282393321217	-0.194261019259572	0.158506554821438	-1.22557088871443	0.220360198421769	0.526332521832925
**Prr33**	101.74416087171	-0.333593804568593	0.272184434357573	-1.22561676003245	0.220342927770918	0.526332521832925
**Uty**	2489.7057915785	-0.150897511453714	0.123107399037404	-1.22573876658598	0.220296996764106	0.526332521832925
**Chchd2-ps**	32.5920930280069	-0.626378846869716	0.511038915769267	-1.22569696268009	0.220312733632788	0.526332521832925
**Polg2**	1059.85103335127	-0.166115891275172	0.135552505041253	-1.22547267735567	0.220397178476483	0.526341064148825
**Gm24265**	23.0401081476763	0.713913256768264	0.582649154821335	1.22528841046235	0.220466573497606	0.526427003879644
**Ascc1**	2870.70277817399	0.118238076986342	0.0965129782722818	1.225100282915	0.220537438609006	0.52651642729729
**Tnfaip2**	50.0193290949834	0.749083210948265	0.611544354148054	1.22490413960541	0.220611340543355	0.526613072707414
**1700034I23Rik**	22.1668204883324	-0.733303600174501	0.598758260692652	-1.22470727890452	0.220685530628063	0.526632679015924
**Gm37809**	19.9355997992342	-0.907339444390134	0.740863686955158	-1.2247049765918	0.220686398397039	0.526632679015924
**Fancl**	1412.15076213365	0.162070734996716	0.132386410748577	1.22422485873203	0.220867413976119	0.526801186214821
**Trappc4**	4246.03968885689	0.133915410593105	0.109372357048027	1.2243990548206	0.220801725705856	0.526801186214821

**Lrch1**	5423.61711901859	0.130619958196801	0.106688797997605	1.2243080871502	0.220836027305406	0.526801186214821
**Trav12d-2**	521.519218964967	0.125992874802771	0.102921648960805	1.22416300238983	0.22089074299177	0.526801186214821
**Chst15**	2326.54195823977	-0.218748765325806	0.178736765568609	-1.22385992960043	0.221005071919014	0.526846021760292
**Trmt13**	1525.7912916803	-0.122328694204926	0.0999784184468193	-1.22355100335975	0.221121652610042	0.526846021760292
**Hoxa5**	22.0261705777064	-0.806097677738326	0.658992610721492	-1.22322718741228	0.221243899599407	0.526846021760292
**Rbms2**	1143.86033691141	0.153667350591029	0.125616643038178	1.22330406922533	0.221214870794895	0.526846021760292
**Gpr55**	450.219571970066	0.256712064006477	0.20981974231011	1.22348860588658	0.221145205135945	0.526846021760292
**Fam131a**	50.1941792787536	0.627793560837663	0.513064237378765	1.22361590440419	0.221097156994849	0.526846021760292
**Ubr4**	11553.4125714709	-0.211670319272714	0.173037708358806	-1.22326122600861	0.221231047071453	0.526846021760292
**Gm6934**	190.213495103735	0.429194523207507	0.35066048858999	1.22396031823632	0.220967197358342	0.526846021760292
**Gm43650**	38.360803937569	-0.55250618140216	0.451580956589666	-1.22349309318683	0.221143511301333	0.526846021760292
**Trav7n-5**	133.541290380856	0.230454677757995	0.188304308781089	1.22384176575538	0.221011925260086	0.526846021760292
**Slmap**	3313.4358151039	-0.115813760298584	0.0946959067871582	-1.22300703618469	0.221327038742094	0.526884748369719
**Lsm1**	3225.56208538717	0.138755883419519	0.113451028973322	1.22304649570122	0.221312135382544	0.526884748369719
**Brms1**	1266.93817471997	0.392637806474854	0.321122901574917	1.22270259937613	0.221442044849745	0.52702451494631
**Gm6728**	18.4124664859799	0.934732233328244	0.764497989548695	1.22267454735891	0.221452644123851	0.52702451494631
**Orc2**	2694.3525127513	0.0961606307694153	0.0786673338657065	1.22237053226657	0.221567537589703	0.527218316188607
**Twistnb**	4138.77713792949	-0.134168359271172	0.109770958173442	-1.22225733931539	0.221610326410146	0.527240512342545
**Atp11a**	616.580831251362	-0.246073515558368	0.20138037295376	-1.22193395487886	0.221732603730852	0.527451786984757
**Il1rn**	25.6855609459711	1.63721786506093	1.34021620546249	1.22160727380247	0.221856176638342	0.52766607928911
**Gm38082**	88.6148345174922	-0.377624364635011	0.309157214894526	-1.22146385865147	0.221910441540892	0.527715488584339
**Tpp1**	9545.26721518833	0.103261830626338	0.0845649814566866	1.22109446306954	0.222050255875818	0.527888636366213
**Trgj2**	19.6090070007124	0.848223756094033	0.694615261665054	1.22114183621702	0.222032321856331	0.527888636366213
**Bnip3**	676.119677955507	0.184168682090804	0.150834272503974	1.22100023445236	0.222085931021992	0.52789380224974
**Sar1b**	3685.49049904835	0.170375380126121	0.139600195359207	1.22045230443786	0.22229345977713	0.528148076855887
**Slc6a4**	77.5517199749614	0.437090935049874	0.358114373347267	1.22053446490969	0.222262332612311	0.528148076855887
**Gm15801**	105.950169015038	0.235297235264107	0.192771640989483	1.22060088328523	0.222237171750556	0.528148076855887
**Scrib**	391.52573540775	-0.282451404952044	0.231538017208764	-1.21989221984136	0.222505769687505	0.528413475955693
**Dnajc28**	328.995811270629	-0.258905906677995	0.212224052605229	-1.21996495448894	0.222478160453368	0.528413475955693
**S100a9**	225.951527259696	0.592769734750257	0.485914854807573	1.21990453447858	0.222501066676004	0.528413475955693
**Gpt**	482.886636140214	-0.216342475949752	0.177382373313427	-1.21963908763068	0.222601721958816	0.528561683680691
**Ost4**	5549.41916184718	0.120708554352917	0.0990116502161301	1.21913486028589	0.222793010620272	0.528936186280491
**Plin2**	1040.79927304906	0.219084534658241	0.179754542892922	1.21879831870924	0.222920749806056	0.529159725021696
**Tle2**	228.78898768131	-0.301795501352155	0.247656888845995	-1.21860329732167	0.222994796955933	0.529231571654221
**Dnah14**	63.4837346014987	-0.299654233214039	0.245912171038021	-1.21854169295146	0.223018191012504	0.529231571654221
**Gm38948**	27.9991075649709	-0.601294826554515	0.493518050012728	-1.21838467010276	0.223077827864995	0.529293379561622
**Ctdnep1**	1473.10468628848	-0.121953394599279	0.10011366980399	-1.21814927809608	0.223167250485533	0.529375406533237
**Rrbp1**	2153.95637293012	-0.11233145239843	0.0922209536196209	-1.21806864914624	0.223197886359923	0.529375406533237
**Pbdc1**	2681.26153816114	0.148677122977324	0.122079501992693	1.21787130968329	0.223272880398202	0.529375406533237
**Chic1**	98.27000723756	0.436769348857932	0.358635929682309	1.21786277589319	0.223276123863091	0.529375406533237
**Dtl**	225.970974225038	0.386863665137283	0.317660766223251	1.21785157712991	0.223280380264003	0.529375406533237
**Nudc-ps1**	370.184480077414	0.168982112828526	0.138776170048451	1.21765943511432	0.223353418214427	0.529468904890135
**Trav12n-2**	517.655381047936	0.129480573512005	0.106364368933996	1.21733034106895	0.22347855473556	0.52968585844889
**Pi4k2b**	286.516849429827	0.166658350016588	0.136939243285414	1.21702403210475	0.223595072387107	0.529802639940399
**Gm4890**	117.621931542711	-0.391058324679109	0.321314671565481	-1.2170571694527	0.223582465090797	0.529802639940399
**Polr2a**	4597.10845774374	-0.200474354346081	0.164804491413252	-1.21643744431324	0.223818327283896	0.530092497070848
**Cep57**	3292.21280558893	0.128395954860062	0.105540150309613	1.21656028045629	0.223771562744831	0.530092497070848
**Blnk**	297.559874458059	0.343654710594189	0.282489780244223	1.21652086067357	0.223786569357631	0.530092497070848
**mt-Ti**	39.260843046996	-0.515474695349801	0.423827346362474	-1.21623746030995	0.223894477478056	0.530193159245825
**Ercc5**	2565.90669168216	-0.0920741349588719	0.0757244113556983	-1.21591087088646	0.224018876492485	0.530408029585589
**Sav1**	51.2153210549125	0.680532608797638	0.559792537720422	1.21568717505398	0.224104111501606	0.530530120933114
**Metap2**	8009.71820562422	0.109671668228926	0.090222877429787	1.21556384980377	0.224151112137458	0.530561675521997
**Rnf170**	1628.85247046881	-0.12910047146093	0.106220605594777	-1.21539950500223	0.224213756736115	0.530601626362089
**Tti1**	4149.21564704382	0.0819594689047004	0.067449981180843	1.21511477794123	0.224322318007813	0.530601626362089
**Higd1a**	3137.58422755505	0.156193551935658	0.128538505124854	1.21514990223312	0.224308923720273	0.530601626362089
**Sh2b3**	4733.53040453402	0.207975861269932	0.17116256648899	1.21507795504638	0.224336360653947	0.530601626362089

**Gm15738**	18.896562557559	-0.631224001626887	0.519383200633864	-1.21533388229833	0.224238774153545	0.530601626362089
**Sod1**	10551.4985656147	0.105296562128976	0.0866809499991511	1.21476013045551	0.224457591208084	0.530808684476329
**Ccdc39**	34.6277379061	-0.572030755070813	0.470973751868319	-1.21457035089877	0.224530002771005	0.530900248148669
**Gm7803**	1867.5509575186	0.118699819948675	0.0977408862585339	1.21443363665338	0.224582177280342	0.530943941096228
**Rab24**	4316.81074920848	0.152061943506193	0.125233775738992	1.21422469784122	0.224661931721555	0.531052814001881
**Malt1**	7727.26639531331	-0.151776761266704	0.125009000351882	-1.21412666959558	0.224699357248491	0.531061612744034
**Dnah11**	74.5663236076008	-0.593524382704046	0.489145623167427	-1.21338994890872	0.224980767303229	0.531365212942115
**Prmt2**	2056.61586640801	0.151565249755229	0.124890701816414	1.21358313750231	0.22490694944133	0.531365212942115
**Pkd1l2**	32.6631909937639	-1.04382065265177	0.860124844506018	-1.21356877355548	0.224912437347111	0.531365212942115
**Smyd1**	247.651606073969	-0.319001203351581	0.262909691108439	-1.21334897168172	0.224996427032417	0.531365212942115
**Rraga**	2561.10059151193	0.0885243229719149	0.0729570961359742	1.21337508837971	0.224986446266633	0.531365212942115
**Snx6**	6029.52037661769	0.0847573728255303	0.0698679721358826	1.21310766914331	0.225088658250304	0.53143270361258
**1500015A07Rik**	394.976587538042	-0.271887007239962	0.224142519264112	-1.21300950900615	0.225126184972645	0.53143270361258
**Pcdhga7**	30.0462390125289	-1.16247593132007	0.958337299759428	-1.21301334260065	0.225124719301639	0.53143270361258
**Fam114a1**	149.816916767713	-0.222893372528701	0.183846115100161	-1.21239098474976	0.225362750234767	0.531831789044364
**Gm49066**	80.9682854036079	-0.51280983520244	0.422947240524895	-1.21246762259525	0.225333429138663	0.531831789044364
**Hist1h4m**	182.418893313514	-0.408476596702246	0.336949126448679	-1.21227973198043	0.225405319603488	0.531852593739466
**Trbv19**	2706.51114406473	-0.125369725787312	0.103437751143964	-1.21203065999399	0.225500644450027	0.53199785216336
**Nsl1**	265.198560410874	0.307003217395113	0.253373562925959	1.21166239227897	0.22564164063156	0.532250798118486
**Ppm1j**	2315.45280971487	0.141008350831131	0.116439591335997	1.211000049152	0.225895385861801	0.532689852592697
**Gm13031**	75.6167718432733	-0.39727970548604	0.328052695642301	-1.21102405425506	0.225886185893967	0.532689852592697
**Icosl**	352.715086500495	0.201593176627233	0.166525559528926	1.21058399201605	0.226054882459192	0.532913469998189
**Mpc1-ps**	1330.00176638927	0.143888748590588	0.118859716624069	1.21057623791651	0.226057855776347	0.532913469998189
**Tpgs1**	3947.2626201279	0.125684864158703	0.10384859823294	1.21027020390572	0.226175227110423	0.532950957616111
**Det1**	2104.12523723514	0.115412889328215	0.0953499644696303	1.21041355358842	0.226120243678894	0.532950957616111
**Gm20900**	24.7877012504688	-0.559637375946415	0.46238902422067	-1.21031717154111	0.226157211078967	0.532950957616111
**Mrm1**	5838.75155330128	0.0681603881313943	0.0563572607156576	1.20943401552619	0.22649614699757	0.533149057101989
**Gdpd3**	33.6107184771694	-0.702541207360707	0.58071451008924	-1.20978758952096	0.22636040957721	0.533149057101989
**Dbr1**	1159.31125432385	-0.193386190569702	0.159856529086454	-1.20974846429396	0.226375426935608	0.533149057101989
**Nxt1**	2140.4963508173	0.130212730817257	0.107662369063908	1.20945444494133	0.226488302543807	0.533149057101989
**Rgs14**	10904.3606600002	0.128786122653198	0.106472470333736	1.20957203537703	0.22644315412711	0.533149057101989
**Lclat1**	1070.90911149177	-0.155107252955119	0.128204536148793	-1.20984216014871	0.226339465028317	0.533149057101989
**Gm36423**	36.7916376773237	-0.6076928600879	0.502447934166836	-1.20946434200308	0.226484502355745	0.533149057101989
**Gse1**	2028.3206254105	-0.208961873909122	0.172801774749181	-1.20925768391226	0.226563862581751	0.533228795026237
**Cchcr1**	390.852295090432	0.24461426545632	0.202300493242826	1.20916297105961	0.226600240558244	0.533234765602784
**Thap8**	22.5512325356048	-0.724311361132615	0.599099521290579	-1.20900006658711	0.226662819796073	0.533247029283926
**Cep152**	2056.32060452282	-0.103459852623842	0.0855766292667703	-1.20897321512073	0.226673135885241	0.533247029283926
**St14**	244.056751345	-0.28913348568245	0.23919883159288	-1.20875793479861	0.226755856733211	0.533361999484282
**Zfp622**	5464.05550604737	0.126781741277016	0.104907988229369	1.20850417033851	0.22685339264657	0.533511777303285
**Mbp**	15767.4376087516	-0.175952573560126	0.145663449140937	-1.20793908559643	0.227070693889733	0.533805079902045
**Wdfy3**	21.3203456440124	0.890039315333362	0.736870005902537	1.20786476339639	0.227099285256595	0.533805079902045
**Adamts14**	220.812353907478	-0.367183190070447	0.304003002360627	-1.20782751229171	0.227113616530006	0.533805079902045
**Gm16156**	17.3689457196171	0.695031686520772	0.575391703839042	1.20792788961587	0.227075000761481	0.533805079902045
**Wnt2b**	19.194968613649	-0.694824775024176	0.575488260028521	-1.20736568108225	0.227291346141368	0.534076516227136
**Gm6157**	38.8563953573834	0.510670719340387	0.422967805277029	1.20735127583981	0.227296891401864	0.534076516227136
**Tmem198**	111.436149694365	0.48339427415	0.400425891372872	1.20720034484451	0.227354997693881	0.534133397743026
**Tgm1**	61.3391329643407	-0.626920402950703	0.519366277193249	-1.2070872339627	0.227398550719521	0.534156076876489
**Ttc7**	4337.96456060924	-0.123995618266655	0.102748109769588	-1.2067922080972	0.227512177581117	0.534271194915374
**Zzef1**	4639.68040053733	-0.213720603785207	0.177099314202366	-1.20678391527251	0.227515372081117	0.534271194915374
**Sccpdh**	21.1763889381537	1.18816548763975	0.98466030328173	1.20667552421863	0.227557128613201	0.534289625325317
**Cd200**	398.014511200687	0.356771456568485	0.295691785383498	1.20656532986119	0.227599585448193	0.534309694265074
**Cd47**	35053.258655602	-0.0914756296286933	0.0758427713328516	-1.20612192857818	0.227770480795645	0.534610606573905
**Ywhaq**	26637.6699135864	0.142867123140871	0.11845804676589	1.20605671831835	0.227795621789503	0.534610606573905
**Plec**	19189.2284532684	-0.173307199383588	0.143715174931054	-1.20590744482432	0.227853179747829	0.534666054100752
**Dctpp1**	653.958166156751	0.17855407199021	0.148109151112918	1.20555732477382	0.227988222235838	0.534903278405316
**Trmt5**	1093.60310206612	0.151815029156073	0.125945649088354	1.20540114132542	0.228048481108451	0.534965001760586

**4933427D14Rik**	1425.99625444028	0.116253939212082	0.0964635389870703	1.20515938387523	0.22814177861145	0.535104198508576
**Gorab**	663.868767039608	0.260385197971463	0.216093122979954	1.20496753612847	0.228215834625133	0.53511858723039
**Gm31748**	39.1629576993984	-0.516687682970575	0.428771768748699	-1.20504128450072	0.228187364657719	0.53511858723039
**G6pc3**	2170.64094635012	0.118520287835011	0.0983689448991653	1.20485472276339	0.228259390214967	0.535141081924897
**Rpl10a**	17653.4999598683	-0.0673032248857328	0.0558940930224483	-1.2041205294932	0.228542996155574	0.535726270518206
**Gm5914**	98.0800950653165	-0.390005227189473	0.324002146663569	-1.20371186180578	0.228700965955179	0.536016825904469
**Septin1**	14210.2117703864	-0.0699916658221509	0.058163155637628	-1.20336775154047	0.228834041471786	0.536248957684553
**Hmgcs1**	4473.2643757748	-0.101290755154881	0.0841877222796102	-1.20315352894888	0.228916914212445	0.536363392898959
**Ggnbp2**	15112.0741450356	-0.0609344961549562	0.05066130257359	-1.20278186820095	0.229060743123894	0.536583050499204
**Prorsd1**	721.621858163233	0.248451363129945	0.206627530708048	1.20241171289508	0.22920405336418	0.536583050499204
**Colgalt1**	9000.82806786081	0.156602894865435	0.130226380500242	1.20254355733356	0.229153000831891	0.536583050499204
**Rpl35a-ps2**	63.2518139928033	0.377170482095887	0.313685680731509	1.20238348532943	0.229214984636044	0.536583050499204
**Gm37397**	25.1615148720126	-0.984437973142704	0.818553999772393	-1.20265489316091	0.229109895904952	0.536583050499204
**AI839979**	27.6449666626655	-1.09853323258687	0.913599742247793	-1.20242287928417	0.229199729224239	0.536583050499204
**Adk**	3451.41644749416	0.10648141632214	0.0885764480240653	1.20214141227711	0.22930874392107	0.536644016607902
**B430306N03Rik**	2038.26289936049	0.596557930855058	0.496246477997638	1.20214038246111	0.229309142845783	0.536644016607902
**Mdm2**	5777.77471625659	-0.0969641544278579	0.0807051161949268	-1.20146229879232	0.229571922564637	0.536991484379672
**Zfp523**	1710.57710640496	-0.181833079298719	0.151324638230729	-1.20160921198749	0.229514970698043	0.536991484379672
**Fam98c**	4431.63008243158	-0.0897264756448344	0.074684587284439	-1.20140552297769	0.229593934776027	0.536991484379672
**Kdr**	34.4218766595497	1.02814524603942	0.85576766524976	1.2014303505372	0.22958430884674	0.536991484379672
**Gm6311**	626.076894597211	-0.121339112611984	0.101026799220893	-1.20105866510409	0.22972844557527	0.537226345641069
**Pear1**	2044.74583449682	-0.112845939149736	0.0939629012465697	-1.20096269541119	0.229765672331754	0.537233669525367
**Rasl11b**	161.692079501769	0.539482248027323	0.449357697462564	1.20056305049112	0.229920741205475	0.537516486524432
**Spata48**	31.0782414413271	-0.484617033779493	0.403740559874395	-1.20031793171897	0.230015888184105	0.537637367872006
**Il1rap**	1584.77958942493	0.113428973216559	0.0945318173654637	1.19990259764115	0.230177171013807	0.537637367872006
**1700020L24Rik**	63.2862128595871	0.574351636059452	0.47861472701203	1.20002917512611	0.230128009836478	0.537637367872006
**Tmem71**	16923.8333184008	-0.102977759239168	0.0858167075793475	-1.19997331689698	0.230149703582345	0.537637367872006
**Ano6**	8601.45591179159	-0.109991303130241	0.0916566458319073	-1.20003631086346	0.230125238623994	0.537637367872006
**Gm26102**	20.3827285636647	-0.729391628365574	0.60773313951495	-1.20018406260966	0.230067863553766	0.537637367872006
**Gm43858**	81.7644532958902	-0.570279320465375	0.475550531813562	-1.19919815522139	0.230450904430106	0.538196961776373
**Pltp**	683.359365148722	0.186314297562465	0.155400667725574	1.19892855216995	0.2305557284437	0.538310985184812
**Gm48933**	16.8621948267672	1.01714661289885	0.848402102799226	1.19889685509131	0.230568054770089	0.538310985184812
**Pimreg**	48.9173073457189	0.603654082126061	0.503676430547904	1.1984957911757	0.230724060543857	0.538464251855779
**Fkbp4**	23411.5561261409	0.0773984494097939	0.0645720063186142	1.19863782809986	0.230668802467826	0.538464251855779
**AU020206**	1890.24122800723	-0.223533061995559	0.186516208822842	-1.19846453778114	0.230736220634018	0.538464251855779
**Cxcl16**	334.819703987326	0.250001568792464	0.208630483082155	1.19829837471075	0.230800879112986	0.538535384596966
**Ndufb1-ps**	285.728730884462	-0.186676227870658	0.155871816174521	-1.19762656554696	0.231062429234224	0.539065842085234
**Lmtk2**	1468.71936657216	-0.231037550622528	0.1929408433996	-1.19745278683179	0.231130119389186	0.539143936523375
**App**	1664.66401168511	-0.21243481234004	0.177443299945423	-1.19719827350697	0.231229282653611	0.539295412488595
**Gpatch1**	2199.99391657012	0.0904346572079339	0.0755507988371092	1.19700464587959	0.231304743925061	0.539391571707442
**Tbc1d13**	188.173524642087	-0.312188977136108	0.26096026813373	-1.19630846246727	0.2315762076039	0.539944703050407
**Gm49322**	4417.34177384351	0.566924603623178	0.474143599945942	1.19568123177833	0.231820978147541	0.539956120128723
**Rimbp3**	27.7757132076263	-0.711551185017598	0.594912578868426	-1.19606007721509	0.231673115521233	0.539956120128723
**2500004C02Rik**	143.765615815505	0.322549408810825	0.269707016374166	1.19592516778781	0.231725762724369	0.539956120128723
**Gm15470**	126.388820699601	0.344107523000185	0.287729470624355	1.19594118132388	0.231719513140149	0.539956120128723
**1700097N02Rik**	333.462418727167	0.232869698250101	0.194756359757807	1.19569752967087	0.231814615733275	0.539956120128723
**Gm42941**	18.3838540292708	-1.00468538687936	0.840179162523253	-1.19579898156728	0.231775013462426	0.539956120128723
**Rpl21-ps10**	119.385246872554	0.250400957409591	0.209371943833579	1.19596232821253	0.231711260351728	0.539956120128723
**Srrd**	1371.75813094914	0.199714014345184	0.167069305124819	1.19539621114708	0.231932265297728	0.540135486889786
**Dgcr6**	575.871671948631	0.120229542274585	0.100608081961415	1.19502866897606	0.232075829210967	0.540143560698081
**Nufip1**	2192.91705188964	0.123998421122304	0.103769443792406	1.19494156073888	0.232109863393425	0.540143560698081
**Eloa**	7923.34545687141	0.125752283496436	0.1052530315762	1.19476162931607	0.232180175871095	0.540143560698081
**Tfip11**	4801.77682894402	0.0962148762583121	0.0805295240547178	1.19477765934561	0.232173911142881	0.540143560698081
**Gtf2h3**	2903.9139149031	0.107244776977956	0.0897399630605899	1.19506152354383	0.232062993476441	0.540143560698081
**Gcc1**	2338.72730003896	0.130524197552049	0.109271277960621	1.19449685212877	0.23228367133729	0.540143560698081
**Eed**	6650.81740140821	0.182810765038281	0.153013337255895	1.19473745437336	0.232189623956183	0.540143560698081

**Fam120a**	3501.81474399161	-0.124561921467304	0.104250447539345	-1.19483344587364	0.232152110032571	0.540143560698081
**Pbrm1**	19221.8084837807	-0.0608649659921776	0.0509613999815259	-1.19433465356607	0.232347087288925	0.540143560698081
**Zfp956**	3317.49214247054	0.139871877461482	0.117085753135339	1.19461056290772	0.232239220332553	0.540143560698081
**Hsh2d**	3401.92953505064	0.0968136750074496	0.0810450114403675	1.1945667387398	0.232256351047864	0.540143560698081
**I830077J02Rik**	278.947512637521	0.493307212957737	0.413037854390037	1.19433898785437	0.232345392520943	0.540143560698081
**Gm15484**	23.2796011941538	-0.591801624749909	0.49560052329274	-1.19411016925086	0.232434875823253	0.540267936029945
**Pan2**	5691.25176457522	-0.0884797777149863	0.074143821811718	-1.1933533442567	0.232731018971766	0.540690436664205
**Aopep**	7512.41316658057	0.102015232867463	0.0854764859661187	1.19348884917777	0.232677976674964	0.540690436664205
**Phc2**	1615.33791214132	0.311584493378745	0.261105505021858	1.19332793597232	0.23274096579257	0.540690436664205
**Abca7**	9159.27826311669	-0.189470297496942	0.158779106995635	-1.19329489302488	0.232753901878105	0.540690436664205
**Slc1a5**	35441.7027798062	0.115398511172297	0.0967387562061844	1.19288810088009	0.232913199986835	0.540901001060067
**Rpl9-ps4**	1268.05980443204	-0.0826712388671696	0.0693002234982806	-1.1929433224587	0.232891570911416	0.540901001060067
**Rcn1**	4083.7055015101	0.182684165465399	0.153230233402028	1.1922201083261	0.233174950820773	0.541272247576773
**Ndufs2**	15949.1870982351	0.0768738587353836	0.0644844856070415	1.19212951784777	0.233210464479197	0.541272247576773
**Trmt2b**	2339.27025785636	-0.111766909399609	0.0937495507584559	-1.19218607977733	0.233188290385096	0.541272247576773
**Psrc1**	49.6203154460251	0.73187736127241	0.613824306796346	1.19232385092764	0.233134285923028	0.541272247576773
**Cdk5rap2**	2480.86012436289	-0.179957638579749	0.1510126765636	-1.19167239913106	0.23338972458116	0.541450561076153
**A430110L20Rik**	34.0811427730815	-0.669582140443051	0.561885195254752	-1.19167072935508	0.233390379566493	0.541450561076153
**Gm28068**	23.4602773221733	0.774983812060629	0.650291710838634	1.19174794810346	0.233360091149691	0.541450561076153
**Maco1**	2166.65663851132	-0.12031906189309	0.100991980581243	-1.1913724357183	0.233507408964441	0.541642314596305
**Odc1**	2166.10474437939	0.288403100045662	0.242204545586192	1.19074189688578	0.233754924861661	0.542136642779687
**Mif4gd**	4150.34737094899	0.145157236848733	0.121926613016773	1.19052955919283	0.233838319150541	0.542250242068445
**Tsc22d1**	1528.49284866421	-0.170576628455731	0.14332860347782	-1.19010877324377	0.23400364246886	0.542553766095031
**Mrpl14**	1326.7368867856	0.118928668325353	0.0999499595940926	1.18988210508873	0.234092732830273	0.542600645955666
**Rps15a-ps7**	33.4483029714763	0.569065691880295	0.478227542799165	1.1899475478753	0.234067008525047	0.542600645955666
**Rock2**	2993.96584426296	-0.167944474759371	0.141172028678017	-1.18964412661672	0.234186294494398	0.542695989259824
**Fibp**	3336.10629406677	0.0980461676457423	0.0824253520612644	1.1895146965568	0.234237191277277	0.542695989259824
**Zfp966**	101.147434690393	-0.301959747045489	0.253850026427654	-1.18952025057813	0.234235007065616	0.542695989259824
**Myadm**	769.833875423497	0.378648355555574	0.318378046296722	1.18930422483553	0.234319973432278	0.542807971386711
**Laptm4a**	6238.87430382556	-0.0853464391918402	0.0717832349301064	-1.18894668476476	0.234460647609688	0.543054009170346
**Lpcat3**	6379.51049142368	0.13736890070761	0.115580274059818	1.18851509762398	0.234630535291485	0.543367628540259
**S100a4**	1437.39074988029	0.559625646206492	0.471507250588122	1.18688661841034	0.235272346305191	0.543403396572786
**Rps6kl1**	160.074976200249	-0.320421206188736	0.269883124880012	-1.18725913793681	0.235125420593385	0.543403396572786
**Syne4**	78.8366692176944	-0.608487044624349	0.512299789406001	-1.18775579691312	0.234929633994439	0.543403396572786
**Eci2**	2765.29376103442	0.118348880445741	0.0996189109979577	1.18801620355162	0.234827025935457	0.543403396572786
**Orc5**	5278.68785342438	0.0843528653496366	0.0710009057849753	1.18805336941894	0.234812384052534	0.543403396572786
**Piwil2**	27.2101289374225	-0.732675070868878	0.617057587981463	-1.18736903190126	0.235082089640762	0.543403396572786
**Ramp1**	5251.49082228894	0.147009260780668	0.123729689341075	1.18814862918972	0.234774858419601	0.543403396572786
**Hcar2**	27.8403828688334	0.816423810012631	0.687492560822707	1.18753839174003	0.235015322492721	0.543403396572786
**Nup107**	6025.50109415823	0.0994649016019632	0.083783569448172	1.18716476580162	0.235162635834681	0.543403396572786
**Nrd1**	6633.77228831781	-0.0923085850409621	0.0776761557713606	-1.18837736142185	0.234684771389641	0.543403396572786
**Miga1**	1857.57551613572	-0.12072940060591	0.101674937079446	-1.18740570757939	0.235067629758442	0.543403396572786
**Phactr2**	238.756987115351	0.323424790625659	0.272447344746219	1.18710935108186	0.235184490331576	0.543403396572786
**Wfdc17**	41.3397496796744	0.872968345391922	0.735532214594869	1.18685263278747	0.235285753834952	0.543403396572786
**Csf2rb**	237.122525304082	0.392814462195296	0.330951266030995	1.18692539510789	0.235257049328598	0.543403396572786
**Ifi207**	93.4049532770915	0.514176095103689	0.432829701089179	1.18794087792452	0.23485670323864	0.543403396572786
**Psmb9**	18058.9584857088	-0.0829597992346564	0.0698904387357731	-1.18699783168186	0.235228475790484	0.543403396572786
**A730071L15Rik**	17.9033949345145	-0.956321974386834	0.805074558222116	-1.18786758893328	0.234885580683145	0.543403396572786
**Gm43338**	91.0913665978525	-0.468818188511095	0.395022654512945	-1.1868134223571	0.23530122325418	0.543403396572786
**Gm20219**	29.3829526381254	-0.629031196692948	0.529896526773986	-1.18708307171306	0.235194854910441	0.543403396572786
**Ccng1**	9035.79123606422	0.0825095984983834	0.0695702584305188	1.18598953575524	0.235626432017718	0.543762522006004
**Med19**	1337.61112388651	-0.133201625692754	0.112313330313609	-1.1859823346064	0.235629275893698	0.543762522006004
**Cnot1**	10889.9514873394	-0.160392046112604	0.135234529598147	-1.1860287944892	0.235610928395033	0.543762522006004
**Arhgap21**	70.7453558658675	0.716821603641224	0.604288020902867	1.1862250761983	0.235533425831531	0.543762522006004
**Pygo2**	4479.74803449013	-0.109553733881604	0.0923596197753657	-1.18616484290491	0.235557207252688	0.543762522006004
**Gm43660**	69.5913807071436	-0.3420317965199	0.288460215679354	-1.18571566520666	0.235734606012974	0.543925931607326

**Keap1**	9414.18932686338	0.097174197914926	0.0820040254854512	1.18499302125316	0.23602020618717	0.544106860115762
**Pitpna**	31566.1718952588	0.0999834914264492	0.0843738640879729	1.18500548134433	0.236015279691762	0.544106860115762
**Dkc1**	14087.9241717623	0.0990072550415314	0.0835255026763758	1.1853535970342	0.235877670428147	0.544106860115762
**Rnf227**	83.1116915525567	0.50154377162534	0.423157150315639	1.18524234141201	0.235921643325106	0.544106860115762
**Rps19bp1**	673.541886291937	0.17351407438298	0.146412524231324	1.18510404279921	0.235976312832823	0.544106860115762
**Gm45534**	41.8391198779013	-0.731848966428159	0.617468718525886	-1.18524055465569	0.235922349573596	0.544106860115762
**Ppp1r12b**	1008.94270504191	-0.197168259948966	0.166468239835643	-1.18441968356026	0.236246972259366	0.544549962242661
**Gm44661**	24.3184470427261	-0.751987105444895	0.634980413367557	-1.18426819097743	0.236306916213771	0.544608465522947
**Lrfn1**	20.0479033457822	-1.47107866001426	1.24240634716182	-1.18405597602976	0.236390905428392	0.544642710459887
**Pdp2**	1335.93537504038	0.175965577959686	0.148601942509503	1.18414049633593	0.236357451937379	0.544642710459887
**Fbxo42**	4213.82274199604	-0.120093333012388	0.101450754182631	-1.18375988409309	0.2365081262662	0.544753478376921
**AW112010**	26400.3346300872	0.134472544627476	0.113590840387686	1.18383264150983	0.236479318278461	0.544753478376921
**Wwc2**	111.482032859713	-0.395532493206048	0.334164856925621	-1.18364479390508	0.236553700800329	0.544778816648754
**Pdcd1lg2**	46.857785583983	0.54231599658609	0.45823380531573	1.18349189932075	0.23661425509074	0.54483864057647
**Cyb5rl**	1174.49116639914	-0.118298598717148	0.0999749586805653	-1.1832822966712	0.236697286563814	0.544950196432791
**Minpp1**	3236.86212367911	0.119846495963004	0.101319319807531	1.18285926307704	0.236864928774479	0.545176845267232
**Chil3**	245.636375132089	0.70218594984005	0.593596213005637	1.18293535985443	0.236834766512501	0.545176845267232
**Dnajc1**	3901.40969655012	-0.0632301034867678	0.0534677637857917	-1.18258365433211	0.23697419374924	0.545347309603788
**Xlr3c**	42.9434293541244	0.793484176136315	0.671023768552	1.18249786866503	0.237008210710589	0.545347309603788
**Gde1**	2759.08227720312	0.104945753181953	0.0887558619722224	1.18240926120234	0.237043350233781	0.545348528198816
**Slc7a4**	171.879719756102	-0.330089271850063	0.279229121980341	-1.18214486192921	0.237148226315829	0.545365546673985
**Slc15a3**	23.0465874052964	1.39510404378984	1.18017480066029	1.18211644835095	0.237159498739307	0.545365546673985
**Synrg**	2568.75690611241	0.175220173261098	0.148205576598213	1.1822778689099	0.237095463937523	0.545365546673985
**Gm43330**	36.8335357725049	-1.01691945433174	0.860307663546908	-1.18204160839292	0.237189191556567	0.545365546673985
**Pus10**	1237.01097560133	-0.0952415608648288	0.0806032949605785	-1.18160877804573	0.237360968896024	0.545601281822706
**Cdca7l**	4066.24314553013	0.0776420552477713	0.0657054895113262	1.18166770882039	0.237337575877539	0.545601281822706
**4930562F07Rik**	167.411353203716	-0.383826853112546	0.324892998052921	-1.18139466043533	0.237445978219395	0.545717076838245
**Ell**	4178.25564552787	-0.130980079109657	0.11087745575097	-1.18130487593291	0.237481631008932	0.545719419543202
**Traf6**	3776.17237492991	-0.139959737111925	0.118494653469617	-1.18114811946189	0.237543886957458	0.5457386409726
**Mmp17**	296.88537489276	0.294339998004729	0.24927935560553	1.18076363479736	0.237696634141358	0.5457386409726
**Knop1**	2055.58371835837	-0.110595528628918	0.0936488285345154	-1.18096008630964	0.237618579670669	0.5457386409726
**Il18**	54.9356701416978	0.425739484812469	0.360458079785169	1.18110678796882	0.237560303710983	0.5457386409726
**Jchain**	805.224961947337	0.56900744094679	0.481854595719725	1.18086959427437	0.237654531880658	0.5457386409726
**Galnt6os**	114.935150779296	-0.533148345739817	0.45152955172648	-1.18076069152342	0.237697803705969	0.5457386409726
**Dld**	4270.10358742079	0.0955048310052082	0.0808973803701868	1.18056765952343	0.237774517434205	0.545835237647257
**Rsrc2**	6526.38131453208	-0.0917503246943544	0.0777304264089621	-1.18036564229855	0.237854820745491	0.545860531675896
**Gm17195**	22.5416140958711	-0.711981130968342	0.603168343293733	-1.18040202023935	0.237840358836572	0.545860531675896
**Pradc1**	1151.24609346741	-0.155465664919098	0.131729535956308	-1.18018835935672	0.237925307777246	0.545942780638716
**Gm4631**	157.435151796835	-0.33675211712468	0.285385406287402	-1.17999067123127	0.238003925226922	0.546043658969221
**Anapc4**	6364.98664490592	0.080082438991555	0.0678725376983656	1.17989457455461	0.238042147984414	0.54605184536183
**Tmem203**	708.472666834365	0.203305845483684	0.17232418601864	1.17978706402647	0.238084915774324	0.546070453836393
**5033430I15Rik**	27.3785598903365	0.597542005987909	0.50664959389118	1.17939896368742	0.238239347597135	0.546285162384051
**Gm5432**	23.9901266015715	-0.635255091371077	0.538635898413805	-1.17937755957559	0.238247866719756	0.546285162384051
**Cybc1**	13490.8043061412	0.0978470092553645	0.0829711488103853	1.1792895561682	0.238282895512083	0.546285986408248
**Etaa1**	1971.38958593129	-0.157007924328576	0.133169187390279	-1.17901090639257	0.238393832983656	0.546381327465233
**Sting1**	21498.5796441361	0.0896464985179256	0.076030942805984	1.17907913816991	0.238366664830028	0.546381327465233
**Dffa**	3269.27260950113	0.111585727320445	0.0946756560992025	1.17861055225774	0.238553287828017	0.546508310018328
**Zfp46**	1194.17856710608	-0.159882706871698	0.135638989954396	-1.17873707940064	0.238502885895443	0.546508310018328
**Gm31763**	28.7572859313805	-0.642041580777163	0.544724041112907	-1.17865475418605	0.238535679189671	0.546508310018328
**Agtrap**	2311.50294710567	0.117878355016263	0.100028123352813	1.17845212991239	0.238616405773418	0.546521686039511
**Psmb10**	14784.6439214737	0.0920306746650057	0.0781138557560995	1.178160696002	0.238732548391823	0.546521686039511
**Slc22a15**	31.8233641276279	0.638590859678207	0.541996590047664	1.178219330904	0.238709177929991	0.546521686039511
**Qrfp**	132.971942418128	0.423537565224365	0.359486515187323	1.17817372093544	0.2387273568271	0.546521686039511
**Gm6712**	300.720737319725	-0.263667943484918	0.223789766490715	-1.17819481926962	0.238718947481586	0.546521686039511
**Tax1bp1**	23358.9030852002	-0.0732498069164964	0.0622061291734234	-1.17753359499808	0.238982596747796	0.546765260146438
**Snap47**	2460.84965090097	0.126202424416681	0.10721937602494	1.17704867436763	0.239176079171008	0.546765260146438

**B4galt5**	404.785334294605	0.321580559226016	0.273160408580898	1.17725903580488	0.239092131786319	0.546765260146438
**Ankra2**	1338.54595819144	-0.123318262109495	0.104768939726088	-1.17704982442223	0.239175620170701	0.546765260146438
**Aldh2**	427.35431067856	0.263950770507375	0.224193604048879	1.17733407974399	0.239062189586464	0.546765260146438
**Cdyl2**	2901.95743212978	0.14933542703529	0.126902146808817	1.17677620742122	0.239284841443156	0.546765260146438
**Iars**	9671.70904538001	0.118340296423954	0.10058063841899	1.17657133901837	0.239366642969217	0.546765260146438
**Rsad1**	662.562486433072	-0.207326609603505	0.176192220779117	-1.17670694362505	0.23931249544992	0.546765260146438
**Zfp386**	4927.44681858534	-0.0783907837539342	0.0665670849072907	-1.17762079957551	0.238947814002374	0.546765260146438
**Osbpl6**	289.132311349816	-0.296250657323629	0.251637637538739	-1.17729072733813	0.239079486682011	0.546765260146438
**Ect2l**	68.020750256338	-0.429831428747297	0.365180514797776	-1.17703823541988	0.239180245506153	0.546765260146438
**Gm10548**	220.860268364576	-0.333583538248716	0.283420434979017	-1.17699183643343	0.239198764632695	0.546765260146438
**Trappc2**	1549.3263451637	0.212111743781082	0.180118604639612	1.17762262374552	0.238947086445206	0.546765260146438
**Gm13961**	27.9436601391918	-0.501926250670414	0.426469678974911	-1.17693302810384	0.23922223812667	0.546765260146438
**Gm11993**	420.542256859366	0.184785007694904	0.157057793439478	1.17654147335331	0.239378569622339	0.546765260146438
**1700001K23Rik**	30.834772271795	-0.710679133874722	0.604060891722182	-1.17650247452466	0.239394144174035	0.546765260146438
**Gm37068**	83.5654385287227	-0.435370493961252	0.370138743937319	-1.17623594150138	0.2395006057938	0.546849883421664
**A830035O19Rik**	224.837706547525	0.414758561318551	0.352596618873454	1.17629761352705	0.239475969090949	0.546849883421664
**Sp100**	65836.2407662848	0.0585943105477291	0.0498292976527367	1.1759007914596	0.239634522574208	0.547076379629354
**Tatdn1**	536.224339096072	-0.227651041439266	0.193676701579466	-1.17541779461718	0.239827607742995	0.547358576941827
**Gm12352**	183.093010648457	-0.252812959947939	0.215072595713523	-1.17547732712859	0.239803802812169	0.547358576941827
**Upk1a**	46.5290056288202	-0.540016662048744	0.459466866782569	-1.17531143394567	0.239870141733771	0.547376368327158
**Rpl3-ps2**	123.303078169865	0.338263296876969	0.287841870079925	1.1751705781478	0.239926478625634	0.547425647799611
**Msl2**	1735.01919477819	0.222863393138909	0.18967165657904	1.17499576456769	0.239996410291802	0.547505926881124
**Id3**	2490.27200619352	0.170920086199564	0.145481563172902	1.1748573666096	0.240051784599141	0.54755297769668
**Ap2m1**	29636.8020376842	0.0614966484203505	0.0523581555498141	1.17453809773421	0.240179561385534	0.547685868126174
**Pecr**	674.640515370185	0.14382954904878	0.122451045075569	1.1745881708075	0.240159518128822	0.547685868126174
**Adam4**	65.4652858309042	0.594399465305733	0.506591702692272	1.1733304397739	0.240663319989237	0.548709583717321
**Cnbd2**	451.987280541702	0.20201877117377	0.172239812756864	1.17289242214251	0.240838948775653	0.549030572317413
**Vrk1**	5316.60500137469	0.0841723587735738	0.0717711207276842	1.17278869160958	0.240880554080052	0.549045984469749
**Crip2**	713.027286254014	-0.26752419746735	0.228137769682658	-1.17264317013127	0.240938929856291	0.549099612054611
**Gm11696**	459.729121513291	-0.148423211494132	0.126590048246251	-1.17247140316599	0.241007846799488	0.549159317555322
**Gm16103**	17.3427435669662	-0.863952209088501	0.736906470594873	-1.17240415651537	0.241034831516951	0.549159317555322
**Nufip2**	5330.76154009507	-0.167923714137798	0.14324664478122	-1.17226978959449	0.241088756610758	0.549202766794233
**Ccpg1**	9173.92715221495	0.0762997289133127	0.0650977669374305	1.17207905129294	0.241165319658241	0.549297765518199
**Cox20**	594.39683489406	0.208751735277019	0.17814817790269	1.17178709170433	0.241282546454048	0.549485342459378
**Srl**	52.4328712120757	0.754041231250547	0.643863848806967	1.17111906911334	0.241550919905554	0.549937558871812
**Gm12669**	67.3981485578921	0.451195894819534	0.385266770741613	1.17112590310089	0.24154817333506	0.549937558871812
**Poli**	2794.21661528347	0.16931290870398	0.144625817782498	1.17069629268136	0.241720875936356	0.550244993084248
**Ccnl2**	23649.5968447447	-0.104550938882343	0.0893761037631466	-1.16978626814401	0.242086991084441	0.550865705945671
**Gm21762**	37.3117536972676	0.787932454140483	0.673585955476779	1.16975784268359	0.242098433310514	0.550865705945671
**4930509H03Rik**	179.277632531947	-0.372045986363262	0.318006840983273	-1.16993076379395	0.242028832495175	0.550865705945671
**Tas1r3**	154.215536855419	-0.302740077735036	0.258840974449192	-1.16959874061385	0.242162484393657	0.550931889487561
**H2-DMb2**	936.75564894332	0.264939559847534	0.226552708220698	1.16943894393635	0.24222682710926	0.550998717303395
**Ptcd1**	3622.99422331	0.0919061602737244	0.0785960248147277	1.16934871057884	0.242263165212319	0.551001832310986
**Srsf6**	10268.1028250665	0.102727827744783	0.0878604084358806	1.16921636916532	0.242316467692794	0.551043524525236
**Aasdhppt**	1636.6388338939	0.135910446780041	0.116273941638878	1.16888139220522	0.242451421528198	0.551191321457195
**Tra2a**	2918.82613419452	0.191137008682745	0.163512571919349	1.16894380926882	0.242426271248785	0.551191321457195
**Mgat5**	1234.3023160897	-0.179455707620603	0.153555804599474	-1.16866769112822	0.242537543957938	0.551307570707622
**Bmf**	364.249925477978	0.565874580035274	0.484398692924092	1.16820005565942	0.242726078054101	0.551589559041807
**Rnf214**	1599.17461934808	0.11949966831288	0.102295040426734	1.16818633449262	0.24273161149939	0.551589559041807
**Fzd5**	83.7392050242102	0.538346304988446	0.460927216564704	1.16796380348452	0.242821365752874	0.551621231064058
**Gm6612**	57.2462867372702	-0.436177034784038	0.373468669405619	-1.16790796796481	0.242843889760629	0.551621231064058
**Fnip2**	17.4913087847988	0.752161843750222	0.644034062576163	1.16789140118078	0.242850573071738	0.551621231064058
**Zfp781**	329.406535282172	-0.214870639199926	0.184003278456446	-1.16775440634763	0.242905843979573	0.551667250444817
**Zfp366**	98.9259311744568	0.68181507981352	0.58398138980455	1.1675287803978	0.242996892607964	0.55179450019076
**Smco4**	123.889840207841	0.359188980768346	0.307694439920972	1.16735609801919	0.243066592702119	0.551873242248889
**Cops2**	3197.90092539764	0.107594935950415	0.092179061444329	1.16723835396606	0.243114125999744	0.551901640019878

**Mustn1**	33.9821471510175	0.735858841609536	0.630474417265057	1.16715099210786	0.243149398221233	0.551902199333328
**Scg5**	311.063724204196	-0.215828508435853	0.184963559433815	-1.1668704316489	0.243262698416266	0.552059642243797
**Gm6055**	508.542449076533	0.184333977270066	0.15798172144984	1.16680572650041	0.243288833898644	0.552059642243797
**Gm16151**	94.8431031612532	-0.332127281836775	0.284690236835678	-1.16662687673577	0.243361084549356	0.552144076205068
**Bloc1s2-ps**	90.585075178865	0.325638653525005	0.279172198047292	1.1664437067972	0.243435096065333	0.552152988153369
**Npm3-ps1**	19.1112948922087	0.618269520773931	0.530017836249913	1.16650700879886	0.243409516514985	0.552152988153369
**Cpsf4**	2169.05115032902	-0.0959919706899023	0.0823160020510238	-1.1661398549264	0.243557905114739	0.552352030928749
**Zfp521**	19.4360842632761	1.6123817404458	1.38300946589627	1.16585011180736	0.243675052311029	0.552458676153222
**Mrto4**	2510.12074148792	0.122883464730153	0.105400662729438	1.16586994377438	0.243667032707566	0.552458676153222
**Klhl10**	40.6380203077621	-0.791818872516913	0.67931516500333	-1.16561341967543	0.243770779622937	0.552596198319467
**Tmem33**	4420.32338240246	0.121728827164381	0.104468925906894	1.16521564769288	0.243931713256324	0.552704523075443
**Lrrc40**	1286.943678733	0.132994492685326	0.114136441254684	1.16522375521208	0.243928432309329	0.552704523075443
**Gmppb**	2379.16192189093	0.167660939201623	0.143896622509502	1.16514853703777	0.243958872754313	0.552704523075443
**Rps10-ps2**	1212.97148786947	-0.107319840996981	0.0921034616379467	-1.16520963586416	0.243934146140307	0.552704523075443
**Zfp607a**	1099.01936715521	-0.189880974131589	0.163007755324577	-1.16485852929821	0.244076262509949	0.552890981651705
**Nosip**	9834.67859739524	0.0915674740653194	0.0786518826519496	1.16421210755404	0.244338064785435	0.552933753628152
**Erg28**	2696.28926275609	-0.0854588904027289	0.073391440046277	-1.16442585605137	0.244251474348619	0.552933753628152
**Nme7**	1040.53677590484	0.1172303294576	0.100688099533743	1.16429180807324	0.244305775242264	0.552933753628152
**Dsg2**	20.9957057139677	0.745144447628902	0.639934177316103	1.16440795638397	0.244258724754227	0.552933753628152
**Gm26461**	18.9111195344724	-0.641733310787363	0.551099759483771	-1.1644594281596	0.244237876101122	0.552933753628152
**Gm18301**	26.7292738447442	-0.663115732562551	0.569586547355925	-1.16420539712674	0.244340783557328	0.552933753628152
**AC167036**	75.6900175416335	0.291560659160587	0.250385584542461	1.16444666610247	0.244243045258435	0.552933753628152
**Ddah2**	46.1886969085081	0.879430706995028	0.755629800997688	1.16383804057738	0.244489652787231	0.552986479581458
**Rad54l2**	859.836042937162	-0.205234081128014	0.176346861436741	-1.16380909450795	0.244501385729018	0.552986479581458
**Trav6-6**	465.493276954999	0.19310852184842	0.165925166332984	1.16382900868024	0.2444933137159	0.552986479581458
**Gm28942**	148.552685964656	0.446271689013723	0.383460312366367	1.16380150597526	0.244504461715048	0.552986479581458
**Snapc1**	2196.75392697663	-0.0969190141131176	0.0832955352711798	-1.16355593127032	0.244604019254824	0.553132251922827
**Zranb2**	3781.00114314072	-0.0872056397403102	0.0749666356172541	-1.16325934893948	0.244724293538964	0.55316842590307
**Tiparp**	2139.84034431402	-0.17449760026749	0.149990173039454	-1.16339355260021	0.244669864221406	0.55316842590307
**Gm37738**	24.7018395902894	-0.573052835946228	0.492627977472335	-1.16325678230163	0.244725334579571	0.55316842590307
**Rnf19b**	680.780534696582	0.259703680818654	0.223323107690703	1.16290554750088	0.244867826414444	0.553411121745897
**5930435M05Rik**	17.8134474720217	-1.0522890294316	0.905058566747009	-1.16267506666864	0.244961361414712	0.553543119433762
**Sgk1**	7388.65832903742	0.423851427547482	0.364794113134881	1.16189218050995	0.24527926379	0.553722516009815
**Il1r2**	17.1064821412133	0.763590684306461	0.657166333364451	1.16194431384997	0.245258085294397	0.553722516009815
**Pin1**	5321.76401856104	0.106289386567541	0.0914760507881719	1.16193676543462	0.245261151661153	0.553722516009815
**Tle6**	55.0027204908927	0.559202734542517	0.481293876531909	1.16187377776755	0.245286739972616	0.553722516009815
**Glipr1**	4344.61180670034	-0.0871331479023549	0.074986386225937	-1.16198622560393	0.245241060116265	0.553722516009815
**Gm12568**	38.2640503118074	-0.424919673691693	0.365559182143603	-1.16238271242431	0.245080042290119	0.553722516009815
**Gm49700**	45.9749920110936	-0.477493148180918	0.410826433390691	-1.16227464781174	0.245123921208251	0.553722516009815
**Gria3**	427.90508507107	-0.167788238776835	0.14451139220916	-1.16107274459017	0.245612317611339	0.554044743349881
**Mertk**	23.6874905424692	1.36899574180445	1.17872032492141	1.16142541437532	0.2454689386645	0.554044743349881
**Psmb6**	14622.4199985363	0.124370545297161	0.107123310591651	1.16100356318575	0.245640450405678	0.554044743349881
**Rbm15**	2615.99749535254	-0.100726272976618	0.0867393632248533	-1.16125216086157	0.245539368056582	0.554044743349881
**Xndc1**	2367.04375566488	-0.0900508724155348	0.0775512649717932	-1.16117864032634	0.245569259217111	0.554044743349881
**Gm37678**	36.5996711196619	-0.602284627175311	0.518692432758316	-1.16115946394758	0.245577056155268	0.554044743349881
**Tle4**	2777.21364936948	-0.153525539124987	0.132246706558576	-1.16090255190579	0.245681530958409	0.554058091213919
**Mapk9**	5506.25818891447	-0.0813386467767479	0.070072930771964	-1.16077129757061	0.245734918338792	0.554099185498618
**Med10**	5516.36788220516	0.100200388940924	0.0863290076650633	1.16068042076516	0.245771887023329	0.554103251370239
**Plekhb2**	8939.02488501567	0.0801187983715719	0.0690447536652014	1.16038937237822	0.245890311751921	0.554290935947785
**Drg2**	6664.47581801127	0.0639233958847325	0.055093856672529	1.16026358918173	0.245941504077956	0.554327031863304
**A730017L22Rik**	1552.13022558151	-0.151687498662449	0.130746300386236	-1.16016666027529	0.245980958134371	0.5543366643769
**Akr1c13**	1738.44151884805	0.123122737872422	0.106140687849871	1.15999566581451	0.246050570739436	0.554414248983454
**St6galnac6**	132.707401862173	0.405302052085167	0.349434993373668	1.1598782599651	0.246098375178345	0.55444195959075
**Rpl17-ps8**	646.280513510523	-0.163038955454273	0.14057595326288	-1.15979263643609	0.246133242834124	0.55444195959075
**Prrt1**	437.215901941667	-0.175271378260982	0.151148300641326	-1.15959873526399	0.24621221616512	0.554461324869772
**Csf2rb2**	98.5807105990932	0.455358075253594	0.392656988563652	1.15968412257045	0.246177436879139	0.554461324869772

**Zfp93**	235.275279494276	-0.298541400846267	0.257480230485419	-1.1594730992878	0.246263395484057	0.554497331043483
**Dus1l**	7456.51798550263	0.0929521753643681	0.0801781994607378	1.15931981498145	0.246325847778966	0.554558706022741
**Gm7591**	159.236197803401	0.227034915746008	0.195862832231474	1.15915262308519	0.246393979068632	0.55463284688349
**Greb1l**	29.7841848834324	-0.685296248439132	0.591423052420328	-1.15872427636129	0.246568592131233	0.554946622798435
**Ints4**	6403.49052055199	0.0836973922112891	0.0722629134082079	1.15823440079822	0.246768393330347	0.555316991389072
**Slc25a36**	3846.73455667126	-0.123289484084728	0.106475134899904	-1.15791808294613	0.246897467335969	0.555363058605607
**Ppargc1b**	1257.53162995536	-0.167478676773461	0.144657727432385	-1.15775824593776	0.246962707065926	0.555363058605607
**Mical3**	250.663811862389	-0.283499794105485	0.244870831754355	-1.15775240388729	0.246965091809957	0.555363058605607
**Gm20427**	11645.2948778385	-0.157031688322411	0.135628413567828	-1.15780819218887	0.246942319501958	0.555363058605607
**4833421G17Rik**	374.740301389095	-0.28396996553872	0.245234525174736	-1.15795263875013	0.246883364484316	0.555363058605607
**Spg7**	6320.26505933716	0.124068507858125	0.107181993550219	1.15754991812122	0.247047757127368	0.555390427886422
**Trbv12-1**	3397.24439272975	-0.12293042121044	0.10619867302678	-1.15755138653606	0.247047157573605	0.555390427886422
**Ufsp2**	2240.55305521957	0.126724388674894	0.109548348186969	1.15678958900056	0.24735833638575	0.555801312461539
**C130050O18Rik**	32.612440739351	0.587781252098339	0.508075554878612	1.15687764635472	0.247322352724112	0.555801312461539
**Zfp985**	23.5349232544013	0.570509562747048	0.493197378750017	1.15675708616492	0.247371619235437	0.555801312461539
**Gm2000**	8637.5909403426	0.09586770347027	0.0828586244080096	1.15700331927068	0.247271004228774	0.555801312461539
**Svip**	23.1172737286666	0.922600688956874	0.797719350811224	1.15654796140855	0.247457093652189	0.555914089631814
**Sec22a**	1514.56886061653	-0.124849843850782	0.107960786215235	-1.1564369640832	0.247502469380546	0.555936765506666
**Thg1l**	300.02423437127	-0.209892662854502	0.181538625475398	-1.15618735299362	0.247604531721167	0.55601688008122
**Qrsl1**	1890.44176207191	0.101442098335207	0.0877392358378093	1.15617713519557	0.247608710257658	0.55601688008122
**Atp6v0c-ps2**	992.343474122995	0.103560562488462	0.089580573986515	1.15606049258014	0.247656414387935	0.556044759405912
**Usp45**	1247.66464194686	-0.125084949586459	0.108208590367343	-1.15596136278852	0.247696961235391	0.556056563354617
**Dhx38**	5995.53179484112	0.11734147038777	0.101540519009157	1.15561227707717	0.247839784013224	0.556297931153329
**Cutc**	835.838532495321	0.204226249653662	0.176838453552671	1.15487466413991	0.248141755962089	0.556817096082973
**Usp8**	6144.97471609952	0.0944870919989781	0.0818140872478651	1.15490003222451	0.248131366228892	0.556817096082973
**Ube2d-ps**	1043.482465593	0.172411131671507	0.14930170439926	1.15478341232092	0.248179131503594	0.556821668105103
**Ccdc38**	53.8663154218564	-0.549594738996612	0.476257510081043	-1.15398650386235	0.248505702102615	0.557316303448747
**Pum3**	8472.76163235513	0.0774686582968353	0.0671293124730448	1.15402132753769	0.248491425191718	0.557316303448747
**Lemd2**	807.099797868306	0.587326497384418	0.508937367506105	1.15402510187537	0.248489877834001	0.557316303448747
**Baz1b**	7161.76543096426	-0.141452781164231	0.12259834988398	-1.15379025327905	0.248586171083672	0.557353527885239
**Tdp1**	3169.19720822523	0.0659710798387863	0.0571884034620758	1.15357442846843	0.248674687160343	0.557353527885239
**Frmd6**	1649.91115906016	-0.135869787022502	0.117781556354891	-1.15357438997588	0.248674702949233	0.557353527885239
**Gm15427**	28110.9844526752	0.053306121333457	0.0462119105832872	1.15351476839253	0.248699159393356	0.557353527885239
**Gm11914**	152.408787057755	0.267552204639045	0.231928037229415	1.15360009007618	0.248664161428846	0.557353527885239
**Mrps30**	4716.55337528811	0.1422160929611	0.123301622546372	1.15340001229598	0.24874623644464	0.557379756492916
**Helb**	2389.3566253243	-0.0955302107660028	0.0828437968015676	-1.15313655885211	0.248854338036229	0.557392217902922
**Rilpl2**	2095.5767064245	0.158701166138672	0.137631850535597	1.1530845914015	0.248875665470136	0.557392217902922
**Ddi2**	3102.55131931329	-0.126405322162151	0.109618821840822	-1.15313520104882	0.248854895262234	0.557392217902922
**Gm45607**	18.7375246659991	-0.721765977853039	0.625966968078589	-1.15304163743417	0.248893294736622	0.557392217902922
**Dtx3l**	10167.4257343187	-0.134310746872629	0.116512771718457	-1.15275557255799	0.249010724415483	0.55757595347659
**Prim2**	963.703010052913	0.110100163731373	0.0955293397142302	1.15252721374115	0.249104493546429	0.557610657194313
**Nup88**	9646.47740552862	0.0768407272412891	0.0666804222338322	1.15237313542837	0.249167775420775	0.557610657194313
**5830454E08Rik**	377.692265135732	-0.216516017925443	0.187882020757569	-1.15240413666202	0.249155041926555	0.557610657194313
**Gm6493**	205.078321815335	0.216334884785717	0.187689036810179	1.15262398093347	0.249064755804745	0.557610657194313
**Gm45867**	108.1618462614	-0.348409593752267	0.302509600354515	-1.15173069993138	0.249431752759267	0.558122142605478
**Tex261**	14605.3277353591	0.0527609193384189	0.0458221299328443	1.15142878377203	0.249555877875458	0.558167823276081
**Foxred1**	4946.63179048716	0.111051948096991	0.0964425836913039	1.15148250748289	0.249533787587733	0.558167823276081
**Snrpg**	2192.80378891362	0.117584168769545	0.102124920996636	1.15137586028995	0.249577640458693	0.558167823276081
**Gm28809**	116.767208941214	-0.327422777256475	0.284384974942454	-1.1513364140378	0.249593861953625	0.558167823276081
**Rpa1**	21704.5325751394	0.0729540979787147	0.0633850021976237	1.15096782281803	0.24974547344543	0.558427618146679
**Rhot1**	6495.01929209211	0.0920338626090831	0.0799763695208204	1.15076319618539	0.249829669659085	0.558536621001448
**Pank4**	8055.85715485556	-0.132954225390374	0.115573732195067	-1.15038445903929	0.249985558158049	0.558805850460544
**Gcnt2**	98.3195714679538	0.697547611452717	0.606513760075736	1.15009362914638	0.250105310076308	0.558994237003175
**Anln**	52.5482223924075	0.828408642113121	0.720408517560433	1.14991511332822	0.250178835469728	0.559079266841086
**Bcas2**	6531.31315883093	0.104393694008789	0.0908444338496958	1.14914793988931	0.250494983385532	0.559169298694648
**6330403K07Rik**	128.090263694395	0.723442860663644	0.629406298265904	1.14940518176705	0.250388944365235	0.559169298694648

**Mfsd3**	1539.15863526155	0.0888377574303475	0.077283552463069	1.14950406133051	0.250348193043968	0.559169298694648
**Llgl2**	3230.55873039336	-0.0797346879763937	0.0693758147766635	-1.14931533752905	0.250425975966127	0.559169298694648
**Gins1**	100.307620037342	0.508791369628325	0.442764258922449	1.14912475290252	0.250504542954924	0.559169298694648
**Rhof**	14711.2810040469	0.100029977654822	0.0870550813075154	1.14904237814072	0.250538506618278	0.559169298694648
**Pram1**	66.5320984407645	-1.12886009759287	0.982236485570277	-1.14927526535269	0.250442493971811	0.559169298694648
**Foxn3**	6753.00871211179	-0.127417431397864	0.11087523598349	-1.14919648438753	0.250474970204121	0.559169298694648
**Gm16124**	387.067051674331	-0.20473540182842	0.178159098873026	-1.1491717410085	0.250485170885543	0.559169298694648
**Maml1**	725.134563665969	-0.186551262270339	0.162371421814305	-1.14891684870314	0.250590269424516	0.559205618938124
**Gm49896**	28.4901207475964	-0.598201822442941	0.520794063163012	-1.14863410463975	0.250706887959024	0.559386637435619
**Cd2bp2**	6403.4669797309	0.0654445804486216	0.0569835675381167	1.14848162858258	0.250769792832643	0.559447773702953
**Tmem177**	1268.92741068915	0.202262439458293	0.176175025758622	1.14807668446385	0.250936908337303	0.559509996430506
**Commd5**	668.723862374611	-0.659975301848164	0.57485638748922	-1.14806987660121	0.250939718523475	0.559509996430506
**Gm20275**	42.4340525914834	-0.509840623923882	0.444051077362729	-1.14815761049806	0.250903504935844	0.559509996430506
**Srp54b**	6486.34374087427	0.0828040262302135	0.0721151802889191	1.14821908367241	0.250878133060328	0.559509996430506
**Srsf4**	5242.31271253145	0.095889154008138	0.0835571267032975	1.14758797712887	0.251138695387429	0.559795221844635
**Ndufc1**	921.296129756307	0.144771016836862	0.126144868697422	1.1476568038936	0.25111026999733	0.559795221844635
**Cic**	4844.32522287911	-0.218329168634317	0.190273590564413	-1.14744861852179	0.25119625723868	0.559844331726999
**Ptpn22**	39530.8689663857	-0.123094825394985	0.107301710431101	-1.1471841865375	0.251305505755134	0.559929419426421
**Prickle1**	182.720142623217	0.351777181410362	0.306628213733719	1.14724335744215	0.251281056765932	0.559929419426421
**Pmm1**	617.965926239973	0.123738783027055	0.107896788514646	1.14682544986275	0.251453768684212	0.560101361769456
**Gm7645**	40.2134291589345	0.70857181407477	0.617816449300513	1.14689697057598	0.251424204890329	0.560101361769456
**Mtfmt**	2300.3144894395	-0.137831879440035	0.120240058610949	-1.14630582380208	0.251668634067954	0.560500730319258
**Syt5**	18.9094088351728	1.09781513302094	0.958156933850865	1.14575712415793	0.25189566034692	0.560927066151275
**Slc16a10**	516.41732534756	0.213769091093495	0.186633335910515	1.14539607862976	0.252045121997401	0.56118058320094
**Casp9**	2006.20428962498	-0.0663651774949538	0.0579461716689678	-1.14529011293588	0.252089000242575	0.561198979488874
**Slc1a4**	102.235620250786	1.79590584708898	1.56844094626036	1.14502611741358	0.252198338596191	0.561287497448469
**Ccnyl1**	116.700758649373	0.372435589146797	0.325264980547972	1.14502209404577	0.252200005199921	0.561287497448469
**Kbtbd3**	351.615597836821	0.201534071593941	0.17608866959562	1.14450334627865	0.252414950929414	0.561607227025526
**Gm6681**	31.3181886394229	-0.480306096601572	0.419660119861909	-1.14451212747978	0.25241131133275	0.561607227025526
**Psma7**	15765.4671583423	0.104999768169042	0.0917689130326229	1.14417578566846	0.252550743055917	0.561830023765648
**Gab3**	5670.39133282886	-0.0940734366696207	0.0822486345629225	-1.14376897768013	0.252719458502103	0.562125989217623
**Atp6v0e**	11428.1729954055	0.098537447868469	0.0861853167299301	1.14332059806945	0.25290550591102	0.562417751158417
**Pde4b**	14877.0568834824	-0.152739546129045	0.133604820586545	-1.1432188259263	0.252947747795585	0.562417751158417
**Phc3**	3318.6936155197	-0.129355835396598	0.113152921851948	-1.14319483120242	0.252957707841575	0.562417751158417
**Gm6542**	177.517817981134	0.296090305528645	0.259061117926651	1.14293610673169	0.253065119957926	0.562577186114143
**Gpr183**	11545.7637166932	-0.116136090173981	0.101632692269976	-1.14270406086929	0.253161483198875	0.562712017317629
**Mpnd**	17236.0145804954	0.0790580421681426	0.0692290715450369	1.14197750170189	0.253463371578504	0.563303574888926
**Edrf1**	3026.2240155368	-0.125026719549116	0.109515076500328	-1.14163933902508	0.253603965041745	0.563457089278452
**Arhgef39**	215.526658718715	0.254338622285675	0.22277464133138	1.14168569979804	0.25358468701986	0.563457089278452
**Trav13d-4**	32.1584667901473	-0.504402418710733	0.441883350752925	-1.14148319426673	0.253668901621667	0.563521906506782
**B4galnt4**	80.9558913772169	0.633079616592057	0.554715912318036	1.14126817445448	0.253758341763516	0.563579871225556
**Gm47523**	38.5086411943953	-0.471414453922997	0.413069069107319	-1.14124849614561	0.253766528295064	0.563579871225556
**Rabep1**	3256.52747505818	-0.114439662609296	0.100294169824868	-1.14104003063317	0.253853265000238	0.563693051945991
**Fip1l1**	7840.49335338897	0.102882435111067	0.0902141863917576	1.14042413090439	0.254109644299171	0.564182847008883
**Ralb**	886.478012328623	0.131329051564509	0.115210824901924	1.13990201594604	0.254327125089027	0.564451371019448
**Snapc5**	2496.28479078005	0.122200182688757	0.107200871759114	1.13991780741623	0.254320545440838	0.564451371019448
**Lgalsl**	216.718413544317	0.470221574238281	0.412520013873274	1.13987578402131	0.254338055101737	0.564451371019448
**Gm6552**	129.559898585043	0.227785242068511	0.19990368905198	1.13947492989627	0.254505118984852	0.56474259397892
**Ngrn**	2364.61539545202	0.105011640322216	0.0921777420572709	1.13922990494789	0.254607275563197	0.564889726985256
**Fto**	7272.21999162496	-0.0879724549292079	0.0772691153392568	-1.13852028126577	0.254903295030203	0.56530770158915
**Trbv21**	34.5179576066227	-0.753970405696506	0.662192376503123	-1.13859723012524	0.25487118425449	0.56530770158915
**Gm48627**	42.2127019082274	-0.415083993314414	0.364555174889906	-1.13860403556133	0.254868344479941	0.56530770158915
**Gm5075**	34.613526765717	0.392937900696082	0.345206182959711	1.13827017038667	0.255007685787405	0.565459626365345
**Ilvbl**	8347.1558916033	0.0858757648352932	0.0754837213849349	1.13767264331555	0.25525720072423	0.565933264642141
**Rbbp8**	1619.56787175483	0.155338245961229	0.136565298776462	1.1374649881995	0.25534395293043	0.565966333707242
**Vps37c**	3038.52587766831	0.0875108804816111	0.0769292016796444	1.13755087237265	0.255308070561225	0.565966333707242

**Gm9828**	34.5307106749991	-0.498335755280999	0.438178519260881	-1.13728933157561	0.255417353106511	0.565969797904555
**H2-T23**	63650.1124181798	-0.0783701275519939	0.0689079261278978	-1.13731659417138	0.255405960150426	0.565969797904555
**Emc4**	5104.38132396733	0.123169360650317	0.108319225295462	1.1370960262534	0.255498144946692	0.566069216806105
**Sh3kbp1**	41434.6186730399	-0.111303840005822	0.0979008888808127	-1.13690326286339	0.255578727972569	0.566168145179779
**5830416I19Rik**	322.49324059381	-0.272892985377624	0.240050017533849	-1.13681718577315	0.255614717441125	0.566168274208575
**Ints12**	1183.27820951353	0.161161921629535	0.141788968810006	1.13663229926926	0.255692031763538	0.566232744862171
**Hpse**	188.431153708475	0.438156631668562	0.385505887951194	1.13657571871907	0.255715695401359	0.566232744862171
**Ramac**	5811.31866588723	-0.0843367001492141	0.0742153214743284	-1.13637856003072	0.255798164811035	0.566256207210942
**Med26**	2591.72912043751	0.118325198792887	0.104123423743694	1.13639366185414	0.255791847223328	0.566256207210942
**Kif3a**	445.465963710974	0.16609265808063	0.146193058514598	1.13611863496272	0.255906916978446	0.56641737474777
**Rttn**	1432.91972836362	-0.131708475676211	0.115942859820226	-1.13597746235024	0.2559659967982	0.566468569037814
**Mrps11**	1929.26253256109	0.124062228729092	0.10923731041267	1.13571295613575	0.256076716436471	0.566634015010459
**Oaz2-ps**	78.8723043649491	-0.297134011775952	0.261754718223013	-1.13516200889566	0.256307444214201	0.567064925369722
**Slc41a1**	2837.84089529399	0.128379536211109	0.113114736119508	1.13494970341861	0.256396392835709	0.567147615713001
**Lactb**	1876.50143944112	0.150917691591267	0.132988794422426	1.13481509661552	0.256452799514827	0.567147615713001
**mt-Tg**	24.2751438783608	-0.626548670134876	0.552080299196338	-1.13488684716144	0.256422731536983	0.567147615713001
**Rgs12**	353.581606952583	-0.236235439358212	0.208219011455372	-1.1345526890509	0.256562785589632	0.567152050019057
**Leng8**	7503.44167095604	-0.23496903721143	0.20708711863115	-1.13463859444557	0.2565267754109	0.567152050019057
**Chd4**	7920.46578883617	-0.17599941443422	0.155118622785712	-1.13461176532849	0.256538021374284	0.567152050019057
**Plekhg2**	5839.4007531469	-0.200264604947697	0.176550192788142	-1.13432107767796	0.256659890891628	0.567222230484221
**Abat**	31.1951120373853	-1.18650863163327	1.04602234340026	-1.13430524607757	0.256666529374405	0.567222230484221
**Scrn3**	599.424429243771	0.167253039522655	0.147517825435944	1.13378189400765	0.256886047685894	0.567428254651651
**Cptp**	1437.67128266724	0.182708696082153	0.161134339296422	1.13389049708419	0.256840483768205	0.567428254651651
**Clasp2**	2730.22963663619	-0.201448025479217	0.177647705411698	-1.13397482400553	0.256805108668527	0.567428254651651
**Gm45855**	84.94442898482	0.308582490075556	0.272181102250311	1.13373958560785	0.256903799497676	0.567428254651651
**Wbp1**	2965.71380604379	-0.120510681791783	0.106322871590482	-1.13344081089108	0.257029184009882	0.567600009805901
**Srsf12**	288.659771837583	-0.40456043246915	0.35694956176667	-1.1333826282538	0.257053606014781	0.567600009805901
**Gstz1**	1060.56569870552	-0.119300846808186	0.105282302763195	-1.13315195124979	0.257150447914985	0.567654750321717
**Slx4**	2688.08756714606	-0.166347500775948	0.14679289292471	-1.13321222479938	0.257125141670431	0.567654750321717
**Cpne3**	3276.83177481802	0.129099723285155	0.113939571511834	1.13305431617976	0.257191444306241	0.567665721800453
**Gm13237**	72.9839598508097	0.365206786283306	0.322480187625662	1.13249371681476	0.257426924467943	0.568105889193469
**Mapk3**	949.555265236908	-0.143760771582053	0.12695663029059	-1.13236127371213	0.257482579125647	0.56814913867565
**Zc3h12c**	23.008125156743	0.718557545859471	0.634898243873349	1.13176804754686	0.257731964445967	0.568619793303712
**Mfn2**	5328.45093013792	0.0931976363745945	0.082359323671081	1.131597883766	0.257803530214777	0.568698057622042
**Slc7a8**	36.1913459895048	1.33853818128391	1.18331515018192	1.13117640814294	0.257980849531127	0.569009552920209
**Gm4208**	60.9108256697138	1.10476495284204	0.976791600721029	1.13101397680585	0.25804920871038	0.569080669230155
**Ncl**	21454.361736555	-0.0786369901201768	0.0695536973006681	-1.1305939608105	0.258226030544049	0.569227844660169
**Ift57**	712.699891716713	0.137957741463175	0.122014049040792	1.13067095590814	0.258193610219652	0.569227844660169
**Klra14-ps**	22.7114170815004	2.64662165831608	2.34096528275953	1.13056852137347	0.258236742947646	0.569227844660169
**Gm10689**	24.7458791322251	-0.561230864262506	0.496439444015684	-1.13051223271609	0.2582604468792	0.569227844660169
**Rab29**	2011.19334067862	-0.13852411752533	0.122554563318758	-1.1303056677297	0.258347447174895	0.569339961557318
**Psmc2**	9074.93262141059	0.0805952360305111	0.0713103995474596	1.13020311962875	0.258390645560157	0.56935553098747
**Pola2**	7141.59348797213	0.0708459381131934	0.0626930593975586	1.13004435888085	0.258457533397921	0.569423287716869
**Pdia2**	25.0082918966568	-1.02262417701583	0.905360820561033	-1.12952112990944	0.258678061095116	0.569829471365266
**Car11**	216.304874922324	-0.267572338879762	0.236927039956987	-1.12934487734426	0.2587523764201	0.569913502271669
**Mthfsd**	2209.13899711487	-0.11822720859466	0.104706094191519	-1.12913397742074	0.258841319937344	0.56995006683241
**Emc1**	6889.05470061087	0.119665448059612	0.105976118799127	1.12917371777346	0.258824558488222	0.56995006683241
**Utp6**	3297.67916954774	0.0951151228654783	0.084268046438175	1.12872110943335	0.259015501435578	0.570174246454372
**Dmrta1**	574.742837257813	0.222740635631788	0.197325891566074	1.12879578986827	0.258983989103271	0.570174246454372
**Gm7392**	61.573808324125	0.3633755409178	0.321991818239645	1.12852414357732	0.259098626354645	0.570277560479138
**Snrnp70**	8798.54266694204	-0.113170404803211	0.100306007410678	-1.12825151478578	0.259213713569808	0.570451185016685
**Kctd1**	93.1097386494408	0.258568682576152	0.229215894211205	1.1280573865349	0.259295684235119	0.57055189170406
**Tcea1-ps1**	477.390382564814	-0.187259952048065	0.166044180750942	-1.12777184482572	0.259416286862297	0.570681210714328
**Phldb3**	28.5977994891614	-0.648537280121895	0.575073511479717	-1.12774674398261	0.259426890419923	0.570681210714328
**Mrpl37**	6983.98820322989	0.0861056863024685	0.0763659439994267	1.12754039029643	0.259514073498612	0.570793307665778
**Map3k4**	4151.05531726304	0.102623015475451	0.0910535462494795	1.12706225844595	0.259716159032778	0.571158062509349

**Ikbkb**	35705.3251351742	-0.0912207797493233	0.0809477878973034	-1.12690886457642	0.259781015027341	0.571195162082229
**1700030C10Rik**	72.3834124841464	0.39666264778292	0.352009880630077	1.12685089143781	0.259805529392863	0.571195162082229
**4931428F04Rik**	342.588601612693	-0.188807283869707	0.167575760139334	-1.12669806010559	0.259870162915181	0.571257555392649
**Sorcs2**	1409.03187125092	-0.332773770726317	0.295426644814359	-1.12641759491744	0.259988802712531	0.571438633608782
**Gm9008**	59.7133029905607	0.342245898149691	0.303959665120335	1.1259582682268	0.260183183823653	0.571712418684189
**Gm10033**	1116.755213256	-0.161345377837924	0.143296881624587	-1.12595177235344	0.260185933514268	0.571712418684189
**Myh9**	15920.247669374	-0.2396952992106	0.212916720286633	-1.12577020202038	0.260262800022278	0.571801581142084
**Atp6v0b**	6714.35726761321	0.0985948134750277	0.0876004906061149	1.12550526592765	0.260374987023356	0.571968307615645
**Sugp1**	7277.92336768507	0.0770819264519127	0.068504842815952	1.12520404811386	0.260502578137896	0.572168821259943
**Spef2**	511.356746889218	-0.252915773192787	0.224852878550567	-1.1248055832023	0.260671428041265	0.57245988733745
**Elof1**	2448.77238156919	0.14661259362716	0.130425519813242	1.12410971286215	0.2609664853197	0.572713817282319
**Adcy3**	6003.74441726933	0.100260545240517	0.0891695136208814	1.12438143003439	0.260851246540907	0.572713817282319
**E4f1**	10141.3116269493	0.126371906159907	0.112426377856355	1.1240414266603	0.260995451919278	0.572713817282319
**Pofut1**	2178.01888416313	-0.132927926417544	0.118250706724631	-1.12411950929893	0.26096232991201	0.572713817282319
**BC064078**	141.88678962092	0.481986002922554	0.428712714101642	1.12426337514283	0.260901310827629	0.572713817282319
**Gm19605**	435.646890470723	-0.221021150028975	0.196634780023345	-1.12401859936851	0.261005135616192	0.572713817282319
**Vps26b**	5703.17519123298	0.0712167523533098	0.063370850521268	1.12380931875624	0.26109392733136	0.572749131694312
**Cspp1**	2353.69432828536	-0.136654453251082	0.121591542757677	-1.1238812350907	0.261063412953854	0.572749131694312
**Dnajc4**	797.478618987644	-0.127016948182348	0.113058376947273	-1.12346339662726	0.261240737885089	0.572891406173235
**Maff**	45.3470133881242	0.418801438697642	0.372798310363892	1.12339950867493	0.261267858396958	0.572891406173235
**Gm43513**	68.7522230910889	-0.392315285502175	0.349217624573955	-1.12341204422543	0.261262536887343	0.572891406173235
**Gm4459**	208.51999127806	0.229463734943179	0.204289487062507	1.12322830823384	0.261340542785207	0.572971049487478
**Trav13n-3**	41.1645619648753	-0.363045358221485	0.323269754616382	-1.12304152503318	0.261419858891731	0.573065208202144
**Sertad1**	1343.49923331858	0.216035828132893	0.192389867438324	1.12290647636186	0.261477216677102	0.573111212015731
**Gm10736**	307.940855032002	-0.397323668918848	0.353919928258249	-1.12263717636416	0.261591619568417	0.573282218294791
**Gm41442**	25.9870461722478	-0.466149252875576	0.415290505336147	-1.12246547148547	0.261664580571337	0.573362369081151
**Usp1**	4832.35510296491	0.104410211062302	0.093053531794907	1.12204458066595	0.26184348539293	0.573674610586262
**5031439G07Rik**	426.217843003437	-0.165142938870414	0.147209782257247	-1.12182041395747	0.261938804700981	0.573759729832693
**Klf8**	137.987514515664	-0.369628848035101	0.329501511409214	-1.12178195011692	0.261955162557365	0.573759729832693
**Cul3**	1953.43187053218	-0.113730234107323	0.10140864733137	-1.12150430067063	0.262073261920572	0.573938622388111
**Chordc1**	8891.7331911914	0.16218811193037	0.144679043168359	1.12102007573852	0.262279317047011	0.574083470616268
**Tubg1**	4931.04021415224	0.0881961341805206	0.0786758523192113	1.12100640260855	0.262285137079807	0.574083470616268
**Csgalnact2**	1472.90046314326	0.163325437313233	0.14566818549753	1.12121556780154	0.262196114676187	0.574083470616268
**Gm49625**	32.6040034381373	-1.04617945954861	0.933227851167862	-1.12103325917609	0.262273705538583	0.574083470616268
**4933430I17Rik**	22.2749956707176	0.624502659665421	0.557263666989713	1.12065920794536	0.262432952032468	0.574327225718834
**Plk-ps1**	20.8011979833221	-0.792009864115322	0.70684325624627	-1.120488675695	0.262505575644146	0.574402272113496
**Foxd2os**	229.701437367074	-0.243945573651263	0.217740551753665	-1.12034975426739	0.262564747590074	0.574402272113496
**Gm15978**	31.3148642925465	-0.557788931559519	0.497882726749312	-1.12032191837893	0.262576605066543	0.574402272113496
**Dscc1**	140.817374592129	-0.261931040728377	0.233908046733636	-1.11980346288237	0.262797523295334	0.574805743276593
**Trav13d-2**	174.867994860907	-0.189991977182718	0.169687333631383	-1.11965915850528	0.26285903542232	0.574860488709159
**Iba57**	1002.67734442073	-0.138177158836191	0.123428256036341	-1.11949373079943	0.262929563945817	0.57493493465088
**Nmi**	6532.02361267637	0.084761474747867	0.0757588653938063	1.11883242056575	0.263211637742378	0.575286395276197
**Ppat**	4582.30466374675	-0.0890591487134949	0.0796070152699664	-1.11873493072783	0.263253238498251	0.575286395276197
**Trim24**	430.348531745489	-0.227564048818798	0.203429917534822	-1.11863609628532	0.263295417654057	0.575286395276197
**Uqcrq**	3026.61529594939	0.151731908896178	0.135644056600004	1.11860344418639	0.263309353476673	0.575286395276197
**Atp10d**	5985.34685024678	0.114264537271895	0.102121591593753	1.11890674135248	0.263179926706412	0.575286395276197
**Cebpzos**	443.900890495902	0.172292185922226	0.153993480375145	1.11882779389428	0.263213611927953	0.575286395276197
**Bop1**	13245.5450620188	0.0708349636573728	0.063343509246767	1.11826712002048	0.263452925294515	0.575474671337734
**Plbd2**	4398.61476896524	-0.0991523289970218	0.0886742585227457	-1.11816360969726	0.26349712319092	0.575474671337734
**Cul1**	8636.33247981403	0.091579162577507	0.0819069858959324	1.11808732278177	0.263529700232827	0.575474671337734
**Sytl2**	1654.23184452029	0.168002501679124	0.150297070206979	1.11780290492531	0.263651180579241	0.575474671337734
**Prmt1**	1959.70976552809	0.120034126544835	0.107366149688024	1.11798855499261	0.263571881480821	0.575474671337734
**Gm45669**	41.1344323394266	-0.513453574183635	0.459331794183616	-1.11782720178604	0.263640801412959	0.575474671337734
**Gm49616**	175.971021988629	0.400869359756379	0.358602684862283	1.11786491478815	0.263624691679356	0.575474671337734
**Rab43**	3488.66093268784	0.118460875734891	0.105984747083283	1.1177162657359	0.263688193521746	0.575475743119412
**A230072E10Rik**	275.27558082512	-0.205450471328061	0.183840953567728	-1.11754463486489	0.26376152616209	0.575556068098055

**Tatdn3**	286.956843539762	-0.217274282441357	0.194456634632649	-1.11734054665614	0.263848745142477	0.575627006081445
**Trbv20**	941.3165085769	-0.109352303257178	0.0978721358758264	-1.1172976075225	0.263867098110445	0.575627006081445
**Ercc8**	1112.71019932373	0.109916860971027	0.0983866132902219	1.1171932572452	0.263911702990008	0.575644615727236
**Zfp595**	822.761729578156	-0.158351196715748	0.141779102472267	-1.1168867199362	0.264042763477626	0.575850771504077
**Prkar1b**	49.8622027426198	0.48945736835717	0.438269444753297	1.11679555628772	0.264081749290594	0.575856092384706
**Qser1**	374.588943920188	-0.272068477187027	0.243659120274285	-1.11659467899565	0.264167667766265	0.575963738894843
**Zfp771**	527.782507714709	-0.17147904135263	0.153695886809694	-1.11570351628833	0.264549064936692	0.576695393168249
**Gm37349**	35.5485659147423	-0.778412472307049	0.697727550228792	-1.1156395817419	0.264576442039301	0.576695393168249
**Slc2a6**	109.868349979825	0.360852258614959	0.3236271883162	1.11502454565835	0.264839903417139	0.576870660512008
**AV099323**	20.3728157298237	0.562945745826414	0.504777360156517	1.11523572620583	0.264749420191576	0.576870660512008
**1810019N24Rik**	23.5752693296524	0.735085622803055	0.659221153340162	1.11508197071423	0.264815296748606	0.576870660512008
**Gm37262**	63.8220754111364	-0.559623776525315	0.501880084109832	-1.11505475958047	0.264826956538213	0.576870660512008
**Gm46223**	46.0331463113861	-0.352642329326244	0.316175886837596	-1.11533593802294	0.264706490507606	0.576870660512008
**A430028G04Rik**	128.327067299391	-0.408498178363018	0.366416302089162	-1.11484717255734	0.264915917839643	0.576956477871354
**Gm8750**	32.576888938186	0.402408739801974	0.361034991067154	1.11459761452076	0.265022893051241	0.577109691239415
**Src**	38.6449500136028	0.916551571110105	0.822424989044729	1.11445005115264	0.265086161366723	0.577167699966209
**Cep290**	193.430445411335	0.229708526895458	0.206139396976201	1.11433588273269	0.265135118619803	0.577194537730344
**Dennd2d**	26478.1491253006	0.114841052979214	0.103075388736079	1.11414620296277	0.265216470124142	0.577277182089901
**Gm16310**	21.6818791639717	-0.746020193944233	0.669631008254053	-1.11407653580642	0.265246353895468	0.577277182089901
**Spc25**	225.275723704848	0.380335998602551	0.341552190157625	1.11355163152966	0.265471586498533	0.577543328068618
**Tfap4**	260.227174165235	-0.22839711428952	0.205109902480229	-1.11353528780277	0.26547860158666	0.577543328068618
**Poc5**	2419.13330358612	0.127453675598555	0.114448397285775	1.11363442932543	0.265436049819734	0.577543328068618
**Dnaaf2**	1980.82899000581	-0.132206200314564	0.118745621263564	-1.11335642449605	0.265555382007823	0.577572074146152
**Tceal9**	805.311834834218	0.152003714786601	0.136540704555146	1.11324835536651	0.265601780114426	0.577572074146152
**Gm10232**	383.922128597191	-0.159645058759921	0.143401672163457	-1.1132719469125	0.265591650907837	0.577572074146152
**Trav7-3**	399.585583897097	0.145696074796915	0.130888269818371	1.11313317075008	0.265651239310675	0.577599914146309
**Gm15361**	73.9562409995177	0.333385460502105	0.299600133402826	1.11276806427136	0.26580805452663	0.577861136199795
**Gm7446**	5928.84932694722	0.0851342422440862	0.0765403522711883	1.11227920590761	0.26601812163288	0.578238038704551
**Tut7**	12763.7679985426	-0.0987277082509961	0.0888001344513135	-1.11179683297806	0.266225513876922	0.578609023746022
**Rbbp5**	4481.26379201698	0.109435566534547	0.0984398134172627	1.11170026369997	0.266267046403305	0.578619480096108
**Mir22hg**	39.4418664432383	0.771974824693575	0.694500474635662	1.11155406351386	0.266329932681827	0.578676330566402
**Dpm2**	4181.19917570351	0.0984862020355065	0.0886362056481777	1.11112836244848	0.266513101160682	0.578994476077328
**Ash2l**	9751.34786407288	0.0531484663750907	0.047856974533712	1.11056887513128	0.266753966298532	0.57927814869983
**H2ac6**	189.967714524587	0.600981864364239	0.541135993267054	1.1105930336215	0.266743562723508	0.57927814869983
**Gm4814**	132.267140819089	0.255416458483475	0.229978793181696	1.11060874331	0.266736797677212	0.57927814869983
**Ifitm3**	169.596127704657	0.301787996061922	0.271849329509698	1.11012963175676	0.266943170064337	0.579492932060311
**Lilra6**	29.5747071707028	-0.717882456310935	0.646692515818741	-1.11008313649968	0.26696320326368	0.579492932060311
**Kidins220**	4225.30847250914	-0.163955214942125	0.147689362822827	-1.11013556974182	0.266940611666256	0.579492932060311
**Prcc**	194.73977905766	0.262764920352819	0.236809374788115	1.10960522820487	0.267169177368725	0.579780295799339
**Prss50**	19.9008292893569	0.915133338263324	0.82473307077279	1.10961154668604	0.267166453448453	0.579780295799339
**Golga5**	4659.40837035933	-0.0815393061012425	0.0734965403964869	-1.10943053457167	0.267244495994269	0.579837372564721
**Mettl27**	2086.87589904877	-0.205307556951016	0.185077108821378	-1.10930821352501	0.267297243029243	0.579837372564721
**Plppr2**	54.3136662868422	0.423268657126575	0.381597152094367	1.10920287220043	0.2673426738391	0.579837372564721
**Tpm3-rs7**	2803.10647902699	0.0824007151697403	0.0742835525596148	1.10927267652703	0.267312568563637	0.579837372564721
**Gm18562**	45.1477719870123	0.642920238999439	0.579680805912001	1.10909354327843	0.26738983000372	0.579859833659319
**Gm6457**	150.585403816504	0.269987633450574	0.243493764930568	1.10880717429278	0.267513374864825	0.580047921803363
**Rnf144a**	2784.43584174833	-0.242806816081877	0.219021731421292	-1.10859691641664	0.267604108968718	0.580147634792407
**Gm8835**	22.598438040289	-0.506696136394089	0.457088346766021	-1.10852998108364	0.267632998496288	0.580147634792407
**Socs2**	102.373643404963	0.543562912509052	0.490451175152447	1.10829158955547	0.267735906540184	0.580285273440763
**Gm5835**	94.3664344139338	0.361991620194449	0.326644656707828	1.10821228133004	0.267770148073097	0.580285273440763
**Dock10**	32183.8773303245	-0.117105442842344	0.105701321344074	-1.10789005618149	0.267909300575644	0.580506992460179
**Tmem86b**	161.464356378457	-0.231473764841317	0.20896436088852	-1.10771886582519	0.26798324911357	0.580587385711883
**Coq2**	3055.59232856946	0.575521982290667	0.519688011715677	1.10743748040418	0.268104828723974	0.580691104632805
**Disc1**	335.945911204694	0.178658655395863	0.16131535885604	1.10751175004545	0.268072734986805	0.580691104632805
**Catsper2**	144.892965925009	0.306447775386687	0.276750235707497	1.10730809172852	0.268160747055902	0.580732392985135
**Pign**	1510.85957790976	-0.161916586952227	0.146245318112229	-1.10715740539449	0.268225879766248	0.580752808162125

**Lancl2**	304.728443923861	-0.201354333205868	0.181872883260636	-1.10711574807616	0.26824388765653	0.580752808162125
**Exosc10**	11866.8609757692	0.0669277072372293	0.0605192343622985	1.10589150610476	0.268773481646107	0.58181944639342
**Hnrnpk**	79040.2680835395	0.0677665838619451	0.0613027015009684	1.10544204745813	0.268968092703577	0.582000856458427
**Dock6**	112.799319335965	-0.333691028139618	0.301842721673025	-1.10551291841681	0.268937399871186	0.582000856458427
**Fgf2**	201.267996193377	-0.280692914168513	0.253893333386983	-1.1055544878789	0.268919398065361	0.582000856458427
**Cdc27**	1906.48211249568	-0.143808578255725	0.130128043644613	-1.10513133240114	0.269102685728017	0.582052301855369
**Slf2**	4220.98458688691	-0.0953733986923311	0.0862881466257329	-1.10528968835088	0.269034084608297	0.582052301855369
**Uba2**	9353.15148655873	0.0589103399705984	0.0533035861984931	1.10518530125209	0.269079304622478	0.582052301855369
**Oas1c**	1157.26460136821	-0.14701420251425	0.133092968126502	-1.10459782048377	0.269333895913902	0.582232706532495
**Fmc1**	823.535379300014	0.143196182068536	0.129618992722469	1.10474691293997	0.269269269400318	0.582232706532495
**Hgfac**	44.1010576120606	0.596090897384436	0.53954894701617	1.10479484888434	0.269248493058533	0.582232706532495
**Ccdc114**	171.777426821782	0.302490056232687	0.273832553643557	1.10465338108205	0.269309811038317	0.582232706532495
**Ppm1d**	2284.7833668858	0.433524971180203	0.392536808234768	1.10441864835494	0.269411575025333	0.582252435979471
**Rps6ka2**	653.224260776752	0.315435661910106	0.285632477535182	1.10434102113354	0.269445234662002	0.582252435979471
**Gm14410**	496.994122316217	-0.272856167963729	0.247080465142501	-1.10432108749011	0.269453878477014	0.582252435979471
**Fam214a**	3529.89972777042	0.149176095543742	0.135095717314713	1.10422520053859	0.269495460543008	0.582262439568926
**Cuta**	4846.90815575313	0.0962433552496273	0.0871666010435378	1.10413110179157	0.269536271421834	0.582270774443905
**Gab2**	25.6337620613252	0.836566103246117	0.758041760045924	1.10358841338165	0.269771719589013	0.582391796779972
**Stk16**	5294.12766455726	0.13663869517995	0.123817307957393	1.10355084789091	0.269788022790719	0.582391796779972
**Paip2**	17008.3982280223	-0.0801881025185873	0.072667313224837	-1.10349617950619	0.26981174975515	0.582391796779972
**Gm7030**	268.117868017319	-0.374858738362053	0.339702621672203	-1.10349086067335	0.269814058291159	0.582391796779972
**Cdiptos**	52.7287076643499	-0.457381428781939	0.414458479734049	-1.10356393015636	0.269782345087869	0.582391796779972
**Gm29650**	25.4594565513507	-0.770788045708869	0.698270313836439	-1.10385337946562	0.269656745065156	0.582391796779972
**Mpl**	127.472374372395	0.295408781458005	0.267866282275416	1.10282182195021	0.270104549593427	0.582540020249606
**Cln6**	1084.04141222443	-0.159964789119067	0.144996756430025	-1.10323012084939	0.269927243956756	0.582540020249606
**Amd2**	440.087722048358	-0.171885666908353	0.155859806337174	-1.10282227950746	0.27010435085244	0.582540020249606
**Taf1a**	1896.70884172828	0.141830471424753	0.128592335082667	1.10294654291468	0.270050380482044	0.582540020249606
**Csnk2a3**	1427.22389069394	0.141356896535652	0.128174481249633	1.1028474245224	0.270093429214473	0.582540020249606
**5430414B19Rik**	54.2927983687342	0.407218458713855	0.369140912062594	1.10315179219367	0.269961252338418	0.582540020249606
**Gm3636**	96.5240967463125	-0.33198893955431	0.301075906866375	-1.10267521240634	0.27016823493314	0.582597629374776
**Zfp593**	2418.90765552433	0.127222777152241	0.115391547451267	1.10253116421695	0.270230817680836	0.582602783100055
**Foxk2**	632.75177303212	-0.245211997818439	0.222414610227025	-1.10249950562216	0.270244573312071	0.582602783100055
**Ppp2r5c**	28756.222149642	-0.0796996240200645	0.0723272091897016	-1.10193141575567	0.270491489463526	0.582821191112282
**Zbtb4**	1414.72211369445	-0.559310765733486	0.50761479282729	-1.1018409503361	0.270530823874801	0.582821191112282
**Zdhhc17**	2845.82033111578	-0.127171047019454	0.115415772957656	-1.10185153866371	0.270526219861872	0.582821191112282
**Zfp580**	245.343375587008	-0.242104339628005	0.219723513124019	-1.10185904178291	0.270522957390707	0.582821191112282
**Traj22**	73.7133167097408	-0.843617019960152	0.765431395047215	-1.10214582968355	0.270398277835886	0.582821191112282
**Zfp638**	8269.5335233493	-0.0938888448857227	0.0852408143248206	-1.10145410540012	0.270699068486321	0.583103926471492
**Cit**	207.591052128345	0.332485683665084	0.301939698023276	1.10116584815374	0.270824482467379	0.583294337102035
**Atp6v1b2**	11400.3270314178	0.0766232942168969	0.0695930243186761	1.1010197497098	0.270888061684341	0.583351535869915
**Igf1**	50.5779022305184	-0.416837741594331	0.378715134634278	-1.10066301415936	0.271043349073944	0.583457992741731
**Fermt3**	45718.380018799	0.0683194557136144	0.0620718611202601	1.10065099516269	0.271048582018752	0.583457992741731
**Socs7**	595.169661397873	-0.201879014602499	0.183416038859146	-1.10066172979306	0.271043908270224	0.583457992741731
**Mfsd11**	1560.08811072152	0.132073954362637	0.120015026944476	1.10047847944691	0.271123701175949	0.583460278534476
**Tmem14a**	122.966337704421	-0.381133160481071	0.346327784655665	-1.10049836417258	0.271115041968958	0.583460278534476
**Slc9a7**	1090.80263003797	0.167883870728434	0.152577805084003	1.10031646238458	0.271194261852736	0.583514106813517
**Tmem254a**	2070.84094110273	0.155252712283352	0.141106632306156	1.10025099278469	0.271222778339925	0.583514106813517
**Rps26**	5481.38619889874	-0.143359107158364	0.130334848035506	-1.09992921554879	0.271362964187052	0.583731661747795
**Dennd11**	2530.62220764077	-0.126186839001885	0.114731075080856	-1.09984883269816	0.271397991620508	0.583731661747795
**Jmjd4**	994.538641243902	-0.117106344841028	0.106490137100467	-1.09969193419804	0.271466370247138	0.583799044081364
**Ccz1**	2848.24016503594	0.118360315238667	0.107639997343643	1.09959418580064	0.271508976372109	0.58381099081541
**Nup85**	6337.99476530964	0.0983636891952723	0.0895141162823247	1.09886231669915	0.271828125651151	0.583985826063485
**Tcea1**	5776.83367605053	0.0881991237005995	0.080251071485272	1.0990398267366	0.271750694507305	0.583985826063485
**Gm9493**	757.752746906063	-0.111698565265472	0.101617636461152	-1.09920451956364	0.271678867830062	0.583985826063485
**Zfp846**	1072.17713516935	-0.125889425970303	0.11456244209428	-1.09887170410266	0.271824030420532	0.583985826063485
**Gm12070**	2358.58918343633	0.0842682585470287	0.0766753829706106	1.09902624913303	0.271756616621288	0.583985826063485

**Gm36933**	216.032093931385	-0.379020290539412	0.344813692053272	-1.09920313280615	0.271679472575543	0.583985826063485
**Gm43063**	30.7252756477219	0.640759872929251	0.583138330810317	1.09881281863064	0.271849719754754	0.583985826063485
**Axl**	593.841895143862	-0.27707324822383	0.252206027689894	-1.09859883509411	0.27194308605609	0.584106762130018
**Xpa**	1360.25392235223	0.107012889302231	0.0974232633000176	1.09843260918783	0.272015629673189	0.584140289443587
**Stx18**	1877.09637627561	0.12485925403112	0.113683252461288	1.09830824970141	0.27206991078419	0.584140289443587
**Eif4g1**	11419.5217839844	-0.0968307835257541	0.0881609671358776	-1.09834075863204	0.272055720391998	0.584140289443587
**Gga3**	3325.01144905373	-0.115667567282429	0.105424365330708	-1.09716161837531	0.272570748088699	0.584869310072287
**Hgh1**	2230.76224597082	0.107869912005483	0.0983027325254658	1.09732363724008	0.272499941593746	0.584869310072287
**Dars**	12757.7175477623	0.0580481151617347	0.0529257793840217	1.09678337924031	0.272736097510871	0.584869310072287
**Hcn3**	625.886582809057	-0.21246758782838	0.193717606156818	-1.09679028170719	0.272733079443033	0.584869310072287
**Slc35f6**	1950.16951627937	-0.106203911626928	0.0968561532228754	-1.09651176608824	0.272854877092174	0.584869310072287
**Cald1**	43.6954913162785	0.54460824802517	0.496326313859724	1.09727861049715	0.272519618198809	0.584869310072287
**Ttll3**	732.702738750729	-0.19413030058198	0.176949415071934	-1.09709489857913	0.272599910049357	0.584869310072287
**Pced1b**	2095.44477964719	-0.137926848882136	0.125776466151365	-1.09660299022989	0.272814979771174	0.584869310072287
**Slc38a10**	12031.8958611824	0.0854093746915054	0.0778913899286732	1.09651881638929	0.272851793466494	0.584869310072287
**6030442K20Rik**	68.9782629509	-0.539836322880923	0.492187537840472	-1.09681022248047	0.272724360571704	0.584869310072287
**Gm26756**	21.302979921912	-0.748976098359294	0.682705688835884	-1.09707024653099	0.27261068553329	0.584869310072287
**Gm42993**	46.0187704168216	-0.918009578397953	0.837170768636012	-1.09656191160813	0.272832945211778	0.584869310072287
**Cfap36**	858.323698003332	0.130764482977185	0.119277255885062	1.0963069363634	0.272944474907616	0.584902229174392
**Blmh**	5512.51287380078	-0.110369008594192	0.100668481860576	-1.09636111078988	0.272920775656176	0.584902229174392
**Anxa1**	593.488464460874	0.691804534688044	0.631305505159198	1.09583162040317	0.273152467919011	0.585268348789754
**Pask**	135.914578679898	-0.303146948091439	0.276670899086477	-1.0956950987342	0.27321222832452	0.585316802407812
**Aamdc**	1236.60360210739	0.116725441163627	0.106555586333135	1.09544178001796	0.273323138649212	0.585440875243363
**Xylb**	62.2605005142104	0.840045960128141	0.767005312389958	1.09522834660765	0.273416609916352	0.585440875243363
**Rps2**	53395.1709149077	-0.102572382023579	0.0936542978266652	-1.09522343772648	0.273418759974355	0.585440875243363
**Slfn2**	33411.9517164881	-0.122231658130487	0.111601034694874	-1.09525559923954	0.273404673651206	0.585440875243363
**A630001G21Rik**	2003.74648162969	0.0943992625555754	0.0862240872356238	1.09481312684252	0.273598514334927	0.585666592905237
**Gm32027**	81.8060627173779	-0.32171360227537	0.293851662185455	-1.09481634333016	0.273597104899857	0.585666592905237
**Zfp52**	1549.93996787196	-0.179862737048272	0.164330952612863	-1.09451527048589	0.273729053740516	0.585866435722536
**Zfp943**	1328.64134242538	-0.0998770725406775	0.0912624698174835	-1.09439370576341	0.27378234328511	0.585900907665825
**Aatf**	5120.88271545581	0.0687069147942674	0.0627912763854408	1.09421115080563	0.273862382044649	0.585992606447249
**Snx32**	1360.97365685456	-0.16393029821227	0.149837296403698	-1.09405536636621	0.273930696275773	0.586059196363525
**Gpn3**	3738.24637784234	0.0877909736772312	0.0802685117589698	1.09371622512262	0.274079455975359	0.586218269010958
**Pglyrp1**	2709.99567293075	0.134570456113572	0.123034409364816	1.09376276773557	0.274059037423247	0.586218269010958
**Gm37660**	188.778657507897	-0.338887113495792	0.309918588536724	-1.09347140194411	0.274186878637096	0.586368437389349
**Cryl1**	1103.0729170316	-0.139233654132736	0.127397699370663	-1.09290556125065	0.274435266304349	0.586422089931871
**Mrpl44**	1850.97440106911	0.147705931211364	0.135141072514838	1.0929758693098	0.27440439474423	0.586422089931871
**Sbk2**	85.0769909307121	-0.53564728781219	0.490051425995776	-1.09304301425868	0.274374914288499	0.586422089931871
**Siglece**	48.5252301109121	0.550269995108886	0.503481229869217	1.09293050557579	0.274424313231078	0.586422089931871
**Fcsk**	1102.47446627327	-0.169681042963387	0.155205849362552	-1.09326448494233	0.274277691385176	0.586422089931871
**Smim14**	10624.4105853988	-0.0694553785939168	0.0635477615747234	-1.09296341637851	0.274409862527967	0.586422089931871
**Polr2b**	9994.88837757622	0.0793823317273734	0.0726569764747929	1.09256310376353	0.27458566985396	0.586663918628997
**Klc2**	118.252618565743	-0.37251275077539	0.341117074453437	-1.09203783297056	0.27481647236602	0.58707743425588
**Zc3h7b**	910.497969694683	-0.192654996611938	0.176488002199461	-1.09160392894133	0.275007228458578	0.58716651745553
**Psmc4**	14140.1965763839	0.0888758548330181	0.0814066131783974	1.091752271259	0.274942002947237	0.58716651745553
**Gm8730**	468.145518736805	0.179596527726635	0.164497104478346	1.091791422689	0.274924789985101	0.58716651745553
**6430562O15Rik**	39.5001015459452	0.426221765186791	0.390439520384563	1.0916460627935	0.274988701301818	0.58716651745553
**C4a**	21.1706395874029	-0.860813634944829	0.788643959023824	-1.09151109964798	0.275048050493	0.587174113483025
**Zfp991**	367.742900011577	-0.211296727937108	0.193607153396894	-1.09136839331525	0.275110814212327	0.587228542338613
**Vps26c**	8006.62808594424	0.0820496484821622	0.0751917974362907	1.09120477604864	0.275182786805981	0.587302610280624
**Gla**	513.217404639576	0.13661727997459	0.125224270328024	1.09098084274496	0.27528131228101	0.587433320369973
**Cyp2u1**	65.1411961969889	0.437085349568257	0.400718088680817	1.09075522646647	0.275380602571502	0.587442652552615
**Polr2k**	493.140629676429	0.192640698886825	0.176618444304496	1.09071676882572	0.275397529631666	0.587442652552615
**Gm20033**	75.1603490435784	-0.349504194099259	0.320402974695606	-1.09082693265036	0.275349043122145	0.587442652552615
**Gm39822**	39.2084778108812	0.8959640169989	0.821692438117451	1.09038853886937	0.275542028337589	0.587671323838032
**Gch1**	1742.65984137507	-0.132306326099118	0.121413639784931	-1.08971550752849	0.275838482864885	0.58813362293836

**Sytl3**	5534.76949113523	0.174218007347263	0.159896710296999	1.08956592680151	0.275904399209234	0.58813362293836
**Gm6736**	81.6604216504125	0.251501251710236	0.230828789259559	1.08955755699707	0.275908087882234	0.58813362293836
**Gm37906**	38.2701877803308	-0.670753315142549	0.615532541110261	-1.08971219284798	0.275839943442165	0.58813362293836
**Slc3a1**	42.0542157462936	-0.590263096988	0.5421787885603	-1.08868718113334	0.276291856583811	0.588725134689365
**Mrpl11**	1455.16660747742	0.126916911099157	0.116582586248711	1.08864381193602	0.276310988574046	0.588725134689365
**Cd300lf**	137.07745474408	0.412001410430206	0.378472768057262	1.08858931263417	0.276335031805708	0.588725134689365
**Gm39214**	40.2349906985741	-0.497043771050048	0.456539432492796	-1.08872035069587	0.276277224694202	0.588725134689365
**L3mbtl3**	2839.50540424431	0.116729165670735	0.107258737442242	1.08829516787471	0.276464823059278	0.588842419159913
**Isg20l2**	1215.87370881332	-0.120297413081882	0.110536556275358	-1.08830433238945	0.276460778593765	0.588842419159913
**Maip1**	1192.99853982591	0.0950214488233968	0.0873282804141638	1.08809481158621	0.276553253981204	0.588933444745991
**Mrgbp**	3303.34300392226	-0.109013341124426	0.100193416397812	-1.08802898477475	0.276582312059423	0.588933444745991
**Fau**	7747.59994845046	-0.0801585417642139	0.07371920191986	-1.08734955990644	0.276882353719726	0.589421900657929
**Gm8797**	2087.85460020889	0.0937897064793106	0.0862560887111845	1.08734012729642	0.276886520826934	0.589421900657929
**Clta**	5115.06805309109	-0.0698994073098681	0.0642977521897313	-1.08712054355504	0.276983539883246	0.589548782647618
**Hmmr**	88.0149566449313	0.466316024985478	0.428986151945725	1.08701883002619	0.277028487993401	0.589564814331716
**Top2a**	795.215588198181	0.327805886015378	0.301600424564152	1.08688801247245	0.277086304736714	0.589606037367136
**Uqcc2**	875.187989185643	0.189743665575719	0.174603662615484	1.08671068369039	0.277164690927299	0.589606037367136
**Nfu1**	4360.22242075436	0.0984018836146256	0.0905564030886737	1.08663639740935	0.277197532830852	0.589606037367136
**Haspin**	895.657829711911	0.162859997568588	0.149865418803235	1.0867083204993	0.277165735651303	0.589606037367136
**Polr1b**	6061.82908823419	0.0786639408263114	0.0724094654416787	1.0863764888538	0.277312459053711	0.589691284387223
**Snord34**	21.0030750610806	0.46714724449057	0.429974223045983	1.08645406969108	0.277278151003648	0.589691284387223
**2210016L21Rik**	2826.60884248731	0.0911652906405946	0.0839310812976737	1.08619225715994	0.277393942179178	0.58970538949235
**Gm13453**	24.1692270027894	0.452296767534304	0.416389829448409	1.08623394604393	0.277375502335908	0.58970538949235
**Dnajb1**	19493.8604080768	-0.139436495992572	0.128417329223437	-1.08580747501735	0.277564178586891	0.589739288472595
**Septin10**	31.5550202454999	0.724760827915227	0.66738773804681	1.0859666526633	0.277493746133786	0.589739288472595
**Nbas**	1411.40889257889	-0.154742805544658	0.142503301375702	-1.08588926748221	0.277527985778803	0.589739288472595
**Gm42967**	34.844120365271	-0.735579551936856	0.677438547903568	-1.08582476478968	0.2775565276703	0.589739288472595
**Gm49378**	276.87488044897	-0.866045941930933	0.797659957911651	-1.0857332543034	0.277597023710176	0.589739288472595
**Cops6**	10640.0442001674	0.0922055516783591	0.0849377343489738	1.08556641385706	0.277670865794033	0.589816639568156
**Stmn1**	2681.41119215029	0.229112888028692	0.211083286675916	1.08541463247376	0.27773805449059	0.589879838874271
**Gm42729**	165.380183320669	-0.364918599108261	0.336275295242388	-1.08517813907569	0.27784276451084	0.590022700862171
**Ipo9**	7086.70409917731	0.076426313162995	0.0704452482272983	1.08490373852326	0.277964292018487	0.590201232897897
**Rps19-ps1**	311.514269937154	-0.183145393138677	0.168910597716022	-1.08427414037447	0.278243267479134	0.590713980823054
**9130401M01Rik**	1796.80247962619	0.0796089452452066	0.0734454136448924	1.08391989770954	0.278400316445966	0.590967773978053
**Casd1**	2800.03734095524	-0.13764214933586	0.127044597662911	-1.08341599617693	0.278623818681582	0.591362541886543
**Mast4**	957.551932197638	-0.310850888991594	0.286972796076364	-1.08320681695862	0.278716634606915	0.591479866868843
**Zc3h18**	3367.0780500689	-0.130094446968893	0.120132962052054	-1.08292049697836	0.278843713131975	0.591669861004651
**Pgk1-rs7**	179.582453520615	0.243350994620842	0.224763592547871	1.08269756619508	0.278942684666888	0.591800172653313
**Fkbp8**	25504.2237257464	-0.0556374899627127	0.0514164320070039	-1.08209550509327	0.279210092901136	0.59182256001092
**Elp2**	11037.8719961859	0.070525486713177	0.0651681413775191	1.08220804249461	0.27916009564936	0.59182256001092
**Tgfbr3**	2754.57823540814	-0.220881006806664	0.204138586125649	-1.08201497325307	0.27924587470577	0.59182256001092
**Adgrg1**	97.2583407115288	-0.496594230328339	0.458960677368785	-1.0819973361886	0.2792537116	0.59182256001092
**Trp53i13**	3703.25097298294	-0.0627242389695361	0.0579692149360913	-1.08202671087899	0.279240659264667	0.59182256001092
**A930002I21Rik**	2277.50637434931	-0.162729818520627	0.150373901620461	-1.08216796110905	0.279177902001185	0.59182256001092
**Homez**	1019.04338567328	0.144338202116221	0.13339639684609	1.08202474376242	0.279241533319304	0.59182256001092
**Gm17971**	27.1939677856468	-0.571215903769134	0.527746346957818	-1.08236827608925	0.279088918805795	0.59182256001092
**Cdk4**	14912.4634954978	0.0739154988841251	0.0683577648278369	1.08130362469115	0.279562075609508	0.592216060290908
**Ostm1**	5747.18692487276	0.0855173283438247	0.0790917959890497	1.08124145209276	0.2795897234565	0.592216060290908
**Zc2hc1c**	22.158065823298	-0.642697767478293	0.594326941576592	-1.08138757057418	0.279524748239488	0.592216060290908
**Dynlt1f**	2739.40693899417	-0.0954420993532852	0.0882524869414031	-1.08146640011013	0.279489698988852	0.592216060290908
**Stard10**	160.406494247394	0.48653464632134	0.450296288634849	1.08047669634665	0.279929958600651	0.592777362944558
**Adgrv1**	61.9787451457361	-0.895367105641717	0.828629780218495	-1.08053937598722	0.279902062232061	0.592777362944558
**Gata1**	186.637829646564	-0.255351976723678	0.23637061012486	-1.08030341246228	0.280007090621677	0.592781368658573
**Rnf7**	2844.28320232527	0.0935562139694901	0.0865978663613599	1.08035241398551	0.279985277628025	0.592781368658573
**Maneal**	74.6103484738293	0.379909196570468	0.35170391854838	1.08019608692022	0.280054870545681	0.592802874152109
**Zbtb38**	6000.6787561855	-0.132089833299968	0.122293435339579	-1.08010567315561	0.280095125867121	0.592808448393314

**Luzp1**	497.858357572788	0.16820463202403	0.155790918235694	1.07968188344301	0.280283864041474	0.592889360410996
**Trpv4**	36.1565140467284	-0.913676735545542	0.846240349127464	-1.07968940087483	0.280280515339933	0.592889360410996
**Zfand1**	1245.55392963346	-0.145180579541543	0.134462837262655	-1.07970783970557	0.280272301726937	0.592889360410996
**Gm16378**	28.5714067936156	0.450084508725146	0.416821012484488	1.07980282961837	0.280229990876657	0.592889360410996
**Mical1**	20262.2126807759	0.0897297480653882	0.0831286889951008	1.07940771290976	0.280406014122272	0.592988533690061
**Gm48671**	102.860564168803	-0.386924694970858	0.35843614277613	-1.07948013270671	0.280373745711509	0.592988533690061
**Ofd1**	1478.35147705012	-0.110474194400736	0.102390547961892	-1.07894914716008	0.28061039785915	0.593195633474592
**Taf1b**	4324.71733902686	-0.0860717975783896	0.0797663835278838	-1.07904851356714	0.280566101454753	0.593195633474592
**Gm6682**	94.7081096912839	0.308318276850368	0.28576178555237	1.07893459671102	0.280616884681069	0.593195633474592
**Asah2**	50.5996638323385	-0.679431772356652	0.629969289478668	-1.07851570497812	0.280803676955129	0.593258999384895
**Cmya5**	153.87319997302	-0.259745326884255	0.240800382511136	-1.07867489318562	0.280732681778844	0.593258999384895
**Rps10-ps1**	337.483804023387	-0.188543290342743	0.174828805136659	-1.07844522643373	0.280835113055273	0.593258999384895
**2310039H08Rik**	1141.12016710015	0.117614109736267	0.109049185512917	1.07854184497633	0.280792018133351	0.593258999384895
**Rps19-ps6**	266.658660219886	0.258481791786681	0.239665923492203	1.07850873424269	0.280806786060668	0.593258999384895
**Macrod1**	2082.09924112579	0.136406716609377	0.126516765794553	1.07817106889125	0.280957420428741	0.593437811487355
**Notch4**	68.0398576154429	-0.411806068030145	0.38204077106941	-1.07791131003483	0.28107333760868	0.593572631799111
**Sephs2**	2470.51518747519	0.163873764548522	0.15203633541492	1.07785921109777	0.281096590625434	0.593572631799111
**Naa40**	5172.4550382936	0.0790653083057717	0.0733916837597227	1.07730609594166	0.281343539833613	0.593614326197012
**Ralgapb**	3801.74434302658	0.243240243848747	0.225876886711177	1.07687088922813	0.281537949955873	0.593614326197012
**Rngtt**	5367.64279604072	0.0791161598257004	0.0734391217029911	1.07730264184897	0.281345082444111	0.593614326197012
**Mpp1**	5097.48312544202	0.108907582388369	0.101101171293284	1.07721385415446	0.281384737325243	0.593614326197012
**Atg12**	7453.78564952526	0.0621639890685744	0.0577301563534899	1.07680271447622	0.281568412387921	0.593614326197012
**Smyd5**	4696.65686088454	0.12284335404527	0.114033432823402	1.07725735342469	0.281365308952003	0.593614326197012
**Wdr74**	6173.45832298939	0.0874604946097568	0.0812134356154298	1.07692149638772	0.281515338673483	0.593614326197012
**Gm11808**	3552.37007339955	-0.0849707436212267	0.0788874689081194	-1.07711332100403	0.281429642609549	0.593614326197012
**Igkv4-55**	43.7075202838136	0.843400081231473	0.782987472186684	1.07715654616551	0.28141033456969	0.593614326197012
**Glud-ps**	36.6406611927504	0.456911597115988	0.42407484327748	1.07743150615756	0.28128753505765	0.593614326197012
**Gm47205**	20.7927717664549	1.01685424192336	0.944252107382551	1.07688850675914	0.281530078301984	0.593614326197012
**Gm9029**	159.554562038769	0.202937073935696	0.1883398650538	1.07750461580572	0.281254889777707	0.593614326197012
**Cyp4f13**	4206.49418750743	-0.0943611929718388	0.0876438565180206	-1.0766435517638	0.281639539552278	0.593617238938738
**Rbpms**	75.2654960665571	0.382905346538878	0.355651415900177	1.07663101964523	0.281645140464936	0.593617238938738
**Armcx6**	32.5497275706456	-0.628609225787868	0.583932241793249	-1.07651056200873	0.281698979805675	0.593651307315504
**Slc9a9**	5811.12752916089	-0.0849585849813057	0.0789347050805263	-1.0763147198008	0.281786527684848	0.593756394320694
**Pola1**	966.690500053033	0.144865683254387	0.134674813025993	1.07567020142383	0.282074778820207	0.593966629676601
**Isyna1**	11981.5270793471	0.0741761480326301	0.0689548892087366	1.07571992187658	0.28205253498086	0.593966629676601
**Zbed5**	789.322207130204	0.152591240857259	0.14182696491358	1.07589724528222	0.281973214068235	0.593966629676601
**Lrg1**	46.2051385014816	1.11221000889425	1.03371863414383	1.07593108236404	0.281958079668213	0.593966629676601
**Ccdc142**	345.782016125371	0.265916931030563	0.247205618305958	1.07569129234533	0.282065343059517	0.593966629676601
**Rps13-ps1**	602.768253209077	-0.726602713659968	0.675615704223348	-1.07546747228919	0.282165487700628	0.593998876379264
**Gm10997**	24.5186987412811	-0.649786104660653	0.604186763913259	-1.07547226035217	0.28216334510743	0.593998876379264
**Mrpl48**	2267.32752585657	0.0809901586678922	0.0753356027523492	1.07505821562391	0.282348665279623	0.594225713745294
**Dse**	4762.9114477358	0.120103835239596	0.111715718738536	1.07508447867297	0.282336907884448	0.594225713745294
**Golm1**	11363.0610833057	0.0686961002014686	0.0639107486708476	1.07487553549508	0.282430456380737	0.594318469710372
**Dlg3**	561.846706136922	-0.166880758486002	0.155291405457938	-1.07462971304746	0.282540543446948	0.594391367569234
**Ints3**	3211.45005429169	-0.133905712992828	0.124599600616379	-1.07468813969236	0.282514375512704	0.594391367569234
**Gna12**	212.511281281203	0.244639500170326	0.227813296551735	1.07385962045797	0.28288560354629	0.594404148444466
**Ccar1**	12006.6299850113	-0.0741181464004777	0.0690092753049314	-1.07403165839623	0.282808492691963	0.594404148444466
**Ntan1**	4176.08713687837	0.0897860323203516	0.0836092887509924	1.0738762841023	0.282878133943545	0.594404148444466
**Anapc11**	4407.75905194447	0.0748845812845188	0.0697395916348102	1.07377430135597	0.282923850563781	0.594404148444466
**Tmem165**	3980.6077739154	0.625983719035557	0.582929691954111	1.07385801010945	0.282886325404097	0.594404148444466
**Polr2j**	1873.93465144766	0.145295137732373	0.135290936337559	1.07394583603038	0.282846958216733	0.594404148444466
**Gm16638**	240.477065435416	-0.279675553559473	0.260439079132319	-1.07386170497624	0.282884669138129	0.594404148444466
**Galnt2**	6429.56703771104	-0.100106405401578	0.0931783567368574	-1.07435255253842	0.282664699571678	0.594404148444466
**1110020A21Rik**	81.3438296697861	0.347105484858357	0.323180177903286	1.07403086139222	0.282808849892299	0.594404148444466
**Gm5605**	30.0443029463941	-0.536290544444476	0.49934464671929	-1.07398877302064	0.282827713444337	0.594404148444466
**Tlcd1**	327.894738885929	0.261634176821589	0.243815980991682	1.07308050833023	0.28323499559316	0.594603453302315

**Rnf19a**	5859.45639789315	-0.0832979635767741	0.0776388873590055	-1.07288971300684	0.283320602106605	0.594603453302315
**Loxl4**	45.7126910879117	-0.437874111979066	0.407993797685608	-1.07323717777809	0.283164713864874	0.594603453302315
**Nhej1**	472.582716726149	0.209895135491358	0.195543454857988	1.07339381747035	0.283094457297966	0.594603453302315
**Bmp2k**	975.50194998728	0.188087887334302	0.175281560915141	1.07306145810375	0.283243542307919	0.594603453302315
**Pkp3**	12067.6418425612	0.0935815793899773	0.0872150588521176	1.07299795037294	0.283272035748844	0.594603453302315
**Intu**	74.6504926826612	-0.471927311938087	0.439747971525885	-1.07317677964618	0.28319180699551	0.594603453302315
**Rpl31-ps3**	28.8776190389265	-0.715182626806923	0.666549672420325	-1.07296223582258	0.283288060327122	0.594603453302315
**Ptdss1**	6861.14593056794	-0.0665931451553086	0.0620938157487127	-1.07246018548456	0.283513387707326	0.594928812106052
**Nup214**	6503.72171252585	-0.145774374525588	0.135967200106375	-1.07212897236642	0.283662107335864	0.595157333610646
**Hyou1**	1492.21547429704	0.155791128082348	0.145320844310599	1.07204942843141	0.283697831614493	0.595157333610646
**Rasgef1a**	121.41413250872	0.233107953251865	0.217481413289895	1.07185230096486	0.283786377409619	0.595184606527801
**Leprot**	3322.64805906405	0.0912172360932394	0.0850978352500006	1.07191018226564	0.283760376323149	0.595184606527801
**2610318N02Rik**	168.050937594473	0.262866481704959	0.245301328114381	1.07160643493291	0.283896841835424	0.595337042427573
**Gm47447**	81.7053242933419	-0.364035645496843	0.33974352551524	-1.07150134780277	0.283944065002182	0.595356837290669
**Ifngr1**	34609.1901761591	-0.160919510333834	0.150201427042601	-1.07135806564736	0.284008460494798	0.595411922618781
**Tbl3**	8155.38447640459	0.0746016461490799	0.069638201008989	1.07127474673635	0.284045911171249	0.595411922618781
**Bclaf1**	16109.7970566871	-0.0708074320482676	0.0661108717946805	-1.0710406643581	0.284151145790852	0.595553286010435
**Fbxl5**	6816.48296454609	0.0926624874798585	0.0865234742217242	1.07095199670788	0.284191014316834	0.595557629018534
**Hsf1**	15907.3117356281	0.0749295327522034	0.0699931093470016	1.07052727691706	0.284382037843995	0.595776351924833
**Rad9b**	509.13930790066	-0.149139081826108	0.139309648567105	-1.07055816564111	0.284368142289224	0.595776351924833
**Gm32036**	960.273753574774	-0.250125645443305	0.233660140320478	-1.07046775329435	0.284408816346931	0.595776351924833
**Smdt1**	3677.08706643326	0.085499573898281	0.0799003633099455	1.07007741087003	0.284584465984141	0.595827455555689
**Izumo1r**	2332.0172975162	0.110988757329438	0.103700253152851	1.07028434314276	0.284491339683079	0.595827455555689
**Rpl13-ps5**	24.4457837893364	-0.689958630036502	0.644771061788458	-1.07008312085642	0.284581896026541	0.595827455555689
**9630013K17Rik**	109.697056643956	-0.32472581912374	0.303438100032843	-1.07015506321913	0.284549517465993	0.595827455555689
**Pds5a**	4111.65499997398	-0.121581255475068	0.113647191725009	-1.06981310870626	0.284703440608142	0.59591818725591
**Ap1g1**	3906.64300642374	-0.096151332971422	0.0898710697987384	-1.06988081021788	0.284672961822252	0.59591818725591
**Blk**	112.804164257554	-0.441177946577315	0.412472672424122	-1.06959315385548	0.284802478106989	0.595962218065885
**Xpo4**	3440.84738818591	-0.121458812662895	0.113560526463155	-1.06955133483202	0.284821310296186	0.595962218065885
**Gm33104**	650.781915085347	0.350532872411262	0.327749557864646	1.06951440207899	0.284837942771732	0.595962218065885
**Ppp1ca**	70528.6460929355	0.0687688643620172	0.0643112145451839	1.06931372464909	0.284928328304671	0.596072181239605
**Gfod2**	1104.9906164708	0.133654582477846	0.125065160663029	1.0686795728665	0.285214079093353	0.596354618813316
**Kdm6b**	1524.37632565615	-0.116904550800663	0.109407237781349	-1.06852666396987	0.285283009309483	0.596354618813316
**Pkn3**	63.1525570757915	0.375594605234003	0.351412282321518	1.06881467759957	0.285153184236624	0.596354618813316
**Hdac1**	20308.8332654458	0.0698319631383665	0.0653296640710656	1.068916611333	0.28510724614323	0.596354618813316
**Trim7**	55.2447447958293	0.385551619002044	0.360831003720597	1.06851023062472	0.285290418011981	0.596354618813316
**Traj13**	60.9965175029918	0.421551777384718	0.394504662247968	1.06855968439671	0.285268122988223	0.596354618813316
**Tmem263**	1131.23994538318	0.156572432243286	0.146633212694285	1.067782866967	0.285618468228241	0.596961162471467
**Gm28035**	480.081478987158	-0.234592844140694	0.21975755796038	-1.06750751290651	0.285742722902523	0.597141655805709
**Lztfl1**	1055.74244249185	-0.132770221505517	0.124417367119022	-1.06713575909787	0.285910536318204	0.597333906227253
**Sae1**	14476.5603890487	0.0645143788957858	0.0604555700914427	1.06713705285061	0.285909952189721	0.597333906227253
**Btrc**	2202.61017622265	0.0763564610324254	0.0715904125635964	1.06657383716842	0.286164319933005	0.597550744410131
**Oga**	16756.8558437359	-0.149990515915535	0.140609049942588	-1.06672021450097	0.286098196151302	0.597550744410131
**Sat1**	16089.1726617396	0.083390770021677	0.0781880725167625	1.06654080779136	0.286179241890206	0.597550744410131
**Rnf34**	5171.7912556639	0.0843157826744787	0.0790416740624465	1.06672566939644	0.286095732182986	0.597550744410131
**Gm44198**	38.1517265101567	-0.403286341575548	0.378144942568634	-1.06648614374196	0.286203939078712	0.597550744410131
**Pcdhgc3**	83.7401980150559	0.473287258222232	0.443819124354946	1.06639671940707	0.286244344050094	0.59755592596679
**Ro60**	296.107637841589	-0.208793481968437	0.195838810251164	-1.06614966512847	0.286355991646634	0.597642370151421
**Gm15550**	16.9098223510958	-0.720320159499847	0.675635496750427	-1.06613723370714	0.286361610372738	0.597642370151421
**0610009L18Rik**	128.948034630131	0.30018709018606	0.281732359384063	1.06550447681035	0.286647700788802	0.598160220014456
**Hdgfl2**	7247.97583657334	-0.0995211770306181	0.0934150727294653	-1.0653653005103	0.286710652904658	0.598212361999298
**Catsperg1**	29.2243442047059	-0.930845709710935	0.874302887571471	-1.06467189225066	0.287024433904454	0.598787767116707
**Lat**	74905.6080084922	0.0969149310592698	0.0910576173683913	1.06432535640793	0.287181335094262	0.599035781980446
**Amd-ps3**	86.8019824555344	-0.315754614442033	0.296695900667611	-1.06423652545093	0.287221564446762	0.599040395895119
**Tnfrsf22**	3280.93684992369	-0.0752713272193023	0.0707364608978811	-1.06410931878495	0.287279179835547	0.599071196116488
**Gm24366**	52.9399381441835	-0.310090816792614	0.291428864805662	-1.06403604529495	0.287312370949565	0.599071196116488

**Kcnh4**	21.0710438722249	-0.67217571459691	0.631869520535149	-1.0637887930211	0.287424389333141	0.599191103645762
**Spop**	20798.2713139192	0.0802481995089707	0.0754395859828947	1.06374125021267	0.287445932122759	0.599191103645762
**Eif5**	27447.9514558083	-0.159067328015831	0.149558531978684	-1.0635790944946	0.287519416972764	0.599265007042307
**Ndufa1**	3332.72594550879	0.137191809019937	0.129004485851278	1.06346541451355	0.287570941408301	0.599293125746673
**Nsg2**	8233.80947063282	-0.109398572489342	0.103056676404134	-1.06153794500744	0.288445498032244	0.59940534466558
**Tbc1d7**	431.709228362904	0.229130485578194	0.215533117219095	1.06308714194152	0.28774243492322	0.59940534466558
**Brix1**	4869.39389258886	0.151756375692901	0.143008439564916	1.06117076834486	0.288612301322337	0.59940534466558
**Ppp2r5b**	1248.40980338924	0.109710631438615	0.103444255903129	1.06057731752021	0.288882035281229	0.59940534466558
**Ddx59**	1266.34116283034	0.13414753928663	0.126246447143889	1.06258466928369	0.287970342411569	0.59940534466558
**Slc23a2**	4242.47448311727	-0.156113323299532	0.147063306682458	-1.06153823697583	0.288445365420901	0.59940534466558
**Gar1**	1381.91155154141	-0.129912167840947	0.122410333831656	-1.06128431950531	0.288560709654385	0.59940534466558
**Zbtb7b**	56.3438758901338	0.544626223028473	0.512595296718068	1.06248774913755	0.288014316671155	0.59940534466558
**Rwdd3**	165.05450316595	-0.45089934434577	0.424711356550869	-1.06166067233892	0.288389759194041	0.59940534466558
**Bcat1**	149.717789938407	0.291595219202724	0.274819615171277	1.06104223681775	0.288670706781482	0.59940534466558
**Nfkbib**	5497.65568935943	0.0739313667209159	0.0695971088430243	1.06227640702242	0.288110221758299	0.59940534466558
**Psma1**	10417.8648998591	0.0772974361492385	0.0727657872032676	1.0622771926223	0.288109865220504	0.59940534466558
**Efnb1**	240.883300536785	-0.263897617057107	0.24834333600172	-1.06263216604	0.287948794007791	0.59940534466558
**Ccnd3**	47452.5986089881	0.0825726180832295	0.0776835999879967	1.06293500939694	0.287811425126885	0.59940534466558
**Thyn1**	2291.5867961874	0.0940344471070894	0.0885763717079407	1.06161999293836	0.288408233675237	0.59940534466558
**Fbxo45**	243.494338334004	-0.26004125029912	0.244979004713587	-1.06148382226935	0.288470081160497	0.59940534466558
**Cdc42ep3**	3222.78195021244	0.125422328477893	0.118115461011019	1.06186207465416	0.288298304425572	0.59940534466558
**Hhat**	410.245263788702	0.220266212097412	0.207531107214303	1.06136479997651	0.28852414733087	0.59940534466558
**Flicr**	103.589244605646	-0.405499512041547	0.382398734748628	-1.06041018234044	0.288958031842694	0.59940534466558
**N6amt1**	1727.92078898697	0.116768103271412	0.11002762187678	1.0612617202813	0.288570977056218	0.59940534466558
**Sarm1**	461.056449294774	0.213896972927664	0.201278354342715	1.0626923775592	0.28792147871343	0.59940534466558
**Nsd3**	9666.05911454236	-0.130319907565496	0.12290026522332	-1.06037124760223	0.288975737448091	0.59940534466558
**Phf5a**	3475.53855621634	0.0977339983145774	0.0921369292098567	1.06074729375854	0.288804760702462	0.59940534466558
**Gm6969**	202.993973819142	-0.174561835778047	0.164412523575645	-1.06173077318975	0.288357924882899	0.59940534466558
**Eif1ax**	3695.94086810754	0.0820074314578948	0.0773241956140531	1.06056624070449	0.288887071505499	0.59940534466558
**Itgad**	232.502299469528	-0.299023167983279	0.281892513428545	-1.06077016500503	0.288794364042301	0.59940534466558
**Bnip3l-ps**	1613.87696012001	0.105816642027972	0.0997330277974004	1.06099899265998	0.28869035896433	0.59940534466558
**Rpl27-ps3**	711.903563452747	-0.105380553078546	0.0991203319771436	-1.06315778989568	0.287710400754361	0.59940534466558
**Gm10767**	137.585296592757	-0.298777851858914	0.281625830718682	-1.06090357939278	0.288733722438153	0.59940534466558
**Gm16278**	33.3930436111268	-0.642097696846597	0.604293886352002	-1.06255865125959	0.287982146771081	0.59940534466558
**Nbdy**	1631.65512557301	0.0902827249285849	0.0849931364298674	1.06223547830927	0.288128797316721	0.59940534466558
**Gm26779**	58.3091688725908	-0.300347442014146	0.283258315118597	-1.06033053924081	0.288994250391852	0.59940534466558
**Gm26766**	32.2284191138152	0.668159379093264	0.630026344050697	1.06052609609528	0.288905324291481	0.59940534466558
**Gm5106**	48.6095471599596	0.439648101599306	0.414409514693279	1.06090252759932	0.288734200482282	0.59940534466558
**2010103J01Rik**	20.1666482393966	-0.881883736929144	0.829760963750826	-1.0628166128022	0.287865124220029	0.59940534466558
**Gm35035**	56.3986932536309	0.48265951499764	0.454251426442799	1.06253824842621	0.287991403750745	0.59940534466558
**Rnf17**	26.3172113892157	0.545947848491622	0.514974892551851	1.06014459420787	0.289078822786319	0.599462123470031
**Casp3**	789.854703165635	-0.127225778411691	0.120016231311383	-1.06007143385134	0.289112102494374	0.599462123470031
**A430057M04Rik**	205.326644652302	-0.25819760443397	0.243578179860096	-1.06001943434453	0.289135757972471	0.599462123470031
**Slc36a3**	110.061820268781	-0.320274318621545	0.302225460112566	-1.05971984789851	0.289272070443323	0.599665835281599
**Gjd3**	19.8278146024993	0.655108542846742	0.618295006315529	1.05954040733822	0.289353737006648	0.599756226521146
**Dph2**	4359.20967812881	0.083607990427981	0.0789287017395229	1.05928500767567	0.289470000676492	0.599905250365971
**Tbc1d22a**	2327.49841684794	0.0787136275004537	0.074313158056897	1.05921521246867	0.289501778497357	0.599905250365971
**Eef2kmt**	3850.11164113585	0.0643090535717307	0.0607448809622973	1.05867445211796	0.2897480667912	0.60032820393539
**Gm3081**	77.8436779297425	-0.498168604072704	0.470592013755209	-1.05859978391355	0.289782085367302	0.60032820393539
**Uros**	3000.87342715874	0.113167141419877	0.106938757480905	1.0582425313861	0.289944885597251	0.600370150963885
**Ankmy2**	2664.11944095388	-0.0843668718061388	0.0797277427244029	-1.05818713691384	0.289970134410128	0.600370150963885
**Oard1**	3580.26862533708	0.117417642427676	0.110973542982443	1.05806879074098	0.290024081581049	0.600370150963885
**Sipa1l1**	6213.65756607795	-0.114626693417091	0.108329240790712	-1.05813252802671	0.289995026601859	0.600370150963885
**Rtn4rl1**	4110.33421060809	-0.0962278695045282	0.0909211439242273	-1.058366243002	0.28988850315862	0.600370150963885
**Gm38336**	107.206394995213	0.270944028913505	0.256077758142855	1.05805373679645	0.290030944287434	0.600370150963885
**Rpl37a**	4446.33421209394	-0.143686354752152	0.135846642433409	-1.05771001902079	0.29018766612758	0.600583849914787

**Gstp-ps**	544.015136405076	0.140968837111089	0.133283680399439	1.05766014780368	0.290210410182851	0.600583849914787
**Kdm4a**	2592.39120033877	-0.122989739491611	0.116318535670092	-1.05735288690738	0.290350564746889	0.600794989982498
**Gm8430**	439.717338564012	0.246552895499403	0.233211589744884	1.05720687281929	0.290417183851079	0.600853934603656
**Ctu1**	1905.90102488539	-0.136370107144578	0.129036061340364	-1.05683717968475	0.290585902775539	0.601124073786815
**Gm42432**	68.9768656693297	-0.419932969904684	0.397420790846708	-1.05664570041747	0.29067331515813	0.601225968357398
**Slamf9**	61.9886536755072	0.525803911236808	0.497664145469136	1.05654368719118	0.290719892534998	0.601243384522111
**Stxbp2**	14006.5854762319	0.0891097242521032	0.0843639822838885	1.05625317629323	0.290852562013947	0.601315073170145
**Itpr1**	3308.57404360268	-0.133826448842416	0.126692656954093	-1.0563078560339	0.2908275879548	0.601315073170145
**Ogg1**	476.388093347272	0.155264459858909	0.147015913978968	1.05610648301055	0.290919568848725	0.601315073170145
**Klhl6**	46489.9454720828	-0.132763246393382	0.125725822445429	-1.05597437193945	0.290979923678377	0.601315073170145
**Eef1g**	28372.0398608422	-0.0673207434666525	0.0637468198974284	-1.05606434289545	0.290938819609627	0.601315073170145
**Rab7-ps1**	307.208840235734	0.156721795397724	0.14841550139551	1.05596648546892	0.290983526872016	0.601315073170145
**Dgat2**	90.4810796381251	0.402112204742375	0.380871170390093	1.0557696040121	0.291073488370948	0.601422102840419
**Bora**	1045.42148583574	0.0823409260544325	0.078032135843962	1.05521815036675	0.291325564910462	0.601785124055342
**Arih2**	8592.64095654782	-0.097241609505233	0.0921491223863134	-1.05526354442716	0.291304809160958	0.601785124055342
**Casp8**	12053.6581827818	0.117297257564884	0.11119811705389	1.05484931465196	0.291494246337212	0.602054638817072
**Edf1**	5942.6976811377	0.072653303953932	0.0689003547429881	1.05446922914901	0.291668141358863	0.602075655160675
**Ppp6c**	5067.63978384361	0.0681089760200428	0.064593376546634	1.05442662485481	0.291687637830024	0.602075655160675
**Pot1a**	5083.17318966448	-0.0855894763413433	0.081162164573933	-1.05454896121428	0.291631656922782	0.602075655160675
**Ccnb1**	366.075626746746	0.251196652157308	0.238253299745676	1.05432601531835	0.291733681992242	0.602075655160675
**Gm16372**	18.0494030764569	0.601989755204048	0.57093441175443	1.05439388975379	0.291702618580564	0.602075655160675
**Mccc1os**	117.125258263656	-0.300229049524994	0.284665329243662	-1.05467374731824	0.291574562456658	0.602075655160675
**Hdac11**	20.4386592458103	1.01005686464787	0.958224888767924	1.05409166103647	0.291840953653359	0.602218164839703
**Rbis**	3039.31097924523	-0.116716919384837	0.110741125540191	-1.05396183048977	0.291900392771865	0.602261946956434
**Fpgt**	2087.3279515132	-0.115514087436584	0.109633121746409	-1.0536422350882	0.292046744835156	0.602455562347846
**Tbpl1**	3339.03346654674	-0.108118782505847	0.102627545904254	-1.05350645923772	0.292108935478679	0.602455562347846
**H3f3a-ps1**	124.192796347402	-0.236951849189915	0.224904333702696	-1.05356728920637	0.292081071876723	0.602455562347846
**Gm28379**	30.5078282519816	-0.746051288893983	0.708233825436766	-1.05339686145871	0.292159142031723	0.60248025140608
**Gm42997**	29.7776087575598	-0.443842181544729	0.421397435635626	-1.0532626542334	0.292220630024782	0.602528195145315
**Cops5**	4079.56398934485	-0.089214633533353	0.084740945532459	-1.05279251928078	0.292436094249646	0.602734931893246
**Ddost**	18793.722208626	0.0835865737413351	0.0794013261834532	1.05271004602885	0.292473902979994	0.602734931893246
**Abcc4**	2069.39506360447	-0.136899189353077	0.130023167105213	-1.0528830546198	0.292394593345842	0.602734931893246
**Gm2436**	28.5228235274455	-0.465738094458565	0.442386819170916	-1.05278474465268	0.292439658281046	0.602734931893246
**Actn4**	31389.4684399109	0.0661469452060635	0.0628437422166703	1.05256216248238	0.292541706382583	0.602795824175541
**Hsbp1**	4356.77645124459	0.0745456352919062	0.0708294166299336	1.05246716461592	0.292585267699151	0.602806755117093
**Cd40lg**	129.863921743445	0.370245546553207	0.351825506767259	1.05235561217605	0.292636425683416	0.602833332395553
**Mrpl38**	4779.2221123409	0.0868880127271711	0.0825813612007937	1.05215040614196	0.292730548921651	0.602948399916137
**Card19**	2032.87245372894	0.118617884414394	0.1127547371267	1.05199912160769	0.29279995263619	0.603012528256234
**Rarg**	2742.64638968739	-0.182186714469875	0.173273362896528	-1.05144097987335	0.293056102851383	0.603161981107951
**Rfx2**	798.675569993808	-0.175030003682215	0.166446547596131	-1.05156884423287	0.292997408272581	0.603161981107951
**Sfpq**	2179.77301303373	0.211251663100596	0.201010141744513	1.05095027179822	0.293281429681338	0.603161981107951
**Mrpl53**	454.836517735985	-0.24799765446986	0.235979822872196	-1.0509273693462	0.293291949031409	0.603161981107951
**Pxk**	6027.34286650201	-0.0762473293020131	0.0725601879761129	-1.05081493624457	0.293343594473733	0.603161981107951
**Gpsm3**	2474.03875984378	-0.0927170908755034	0.088204115924925	-1.05116512878401	0.293182755815528	0.603161981107951
**Zfp189**	393.972727589579	-0.192487969674561	0.183067593083664	-1.05145846095541	0.293048077907618	0.603161981107951
**Rhno1**	2829.69662748507	-0.0753785510424479	0.0717038295185491	-1.05124860901534	0.293144423221238	0.603161981107951
**Gm14023**	27.5726039510595	-0.67436168256241	0.641773334581698	-1.05077859459828	0.2933602890867	0.603161981107951
**Trav14-2**	486.391709262484	0.112477973449635	0.107044691717553	1.05075713372521	0.293370148074591	0.603161981107951
**Gm38309**	31.4642282681253	-0.921018781594681	0.876110633776218	-1.05125853526615	0.293139865492321	0.603161981107951
**Gm37115**	34.971100947106	-0.579481851795409	0.551449213844115	-1.05083448710695	0.293334613469045	0.603161981107951
**4930549C15Rik**	19.3160252904834	0.622143712201078	0.591668357181697	1.05150749511862	0.293025568852354	0.603161981107951
**Hsd17b10**	3669.36437689233	0.0812295854084313	0.0773206641503346	1.05055467773138	0.293463166005486	0.603274508382055
**Evi2**	1702.78408436751	0.293575852926735	0.279511312353012	1.05031832327402	0.293571783538802	0.603419070339277
**Nuf2**	138.035134438764	0.634944820118559	0.604774585772231	1.04988674302146	0.29377018736132	0.603542701063195
**Cacna1a**	318.24489460591	-0.24438363150369	0.232755095416649	-1.0499603932031	0.293736322920952	0.603542701063195
**Prrg1**	548.059151732906	-0.164089213057674	0.156295551823497	-1.04986489470268	0.293780233747688	0.603542701063195

**4430402I18Rik**	30.2082727026263	0.507327049397814	0.483235711882758	1.04985421590882	0.293785144199702	0.603542701063195
**Cd44**	4876.61095418318	0.10988444108885	0.104697021076701	1.04954696856513	0.293926449964066	0.603596907608991
**Inka2**	184.001811610663	-0.290978470336047	0.277211456188363	-1.04966250073853	0.293873310351649	0.603596907608991
**Zfp930**	1319.6156026548	-0.146550064822546	0.139621893345388	-1.04962095349918	0.293892419474309	0.603596907608991
**Nudt21**	4851.47311982062	0.0781529111556276	0.0744724390357585	1.04942059327615	0.293984584302266	0.603637619865885
**Aldh1l1**	184.013791463617	0.464024619237057	0.442238607863597	1.04926302449871	0.29405707886604	0.60370780347781
**Gm35065**	220.336729661178	-0.282194624091242	0.268987113371402	-1.04910090507423	0.294131679611628	0.603782292292915
**Gm10521**	20.6321142245015	-0.744381120364017	0.709633464063064	-1.04896563939079	0.294193933071506	0.603831418966748
**Acd**	7794.14415326403	0.0805509939448599	0.0768175155643288	1.04860191524164	0.294361374011236	0.603939084673835
**Pcid2**	5870.90631060586	0.084877228647639	0.0809380277508659	1.04866934624227	0.294330327229153	0.603939084673835
**Gm6877**	114.056222334195	-0.288941309245375	0.275506774827752	-1.04876299113161	0.29428721461174	0.603939084673835
**Efna2**	29.8402024735577	0.68983655112979	0.658216280218881	1.04803933275609	0.294620485479271	0.604189755708712
**Git1**	631.429825093546	-0.173318626302357	0.165518148301599	-1.04712762969378	0.29504071768242	0.604189755708712
**Serinc1**	10573.54244002	-0.051751769110554	0.0494060517300229	-1.04747834118276	0.294879016384552	0.604189755708712
**Mink1**	2403.92095230453	-0.149340418349938	0.142560874090712	-1.04755543414325	0.294843479370409	0.604189755708712
**Ccna2**	463.329802589325	0.378849743768686	0.361812413290167	1.04708885005793	0.295058601317868	0.604189755708712
**Wdr78**	241.726985944212	0.20255449271933	0.193429415525746	1.04717523014161	0.295018767229837	0.604189755708712
**Atp11b**	22663.3953624127	0.0662718593975062	0.0632764005726767	1.04733927337395	0.294943128777951	0.604189755708712
**Stim2**	3907.09408331408	0.266060910975853	0.253830662814286	1.04818270584793	0.29455443689744	0.604189755708712
**Eif3j2**	601.298875118137	-0.161369467081988	0.154018937360831	-1.04772484375695	0.294765397862901	0.604189755708712
**A430005L14Rik**	2568.00684828533	0.144870761233317	0.138290845514944	1.0475802696402	0.294832031732602	0.604189755708712
**Gm4879**	67.3103434544191	0.423564137754831	0.404516042103112	1.04708860383555	0.29505871486822	0.604189755708712
**Rpl28-ps3**	19.3654645067222	-0.5422703943675	0.517677889474735	-1.04750541870297	0.294866534318491	0.604189755708712
**Gm48583**	240.246707910344	-0.163787689040246	0.156417236384069	-1.04712046336172	0.295044022456847	0.604189755708712
**Gm49201**	21.2032607518599	-0.632205899598347	0.60338163511777	-1.04777119952441	0.294744034735738	0.604189755708712
**Gm29719**	593.996713718995	0.275534137271043	0.263089277371357	1.0473028016346	0.294959944355065	0.604189755708712
**Pbld2**	65.7818653025708	0.361453906236141	0.345344102271646	1.04664855678301	0.295261698104292	0.604505480400887
**Lrrc45**	5062.02157570397	-0.104745208361074	0.100085373087029	-1.04655860422275	0.295303202591345	0.604505480400887
**Pif1**	39.1573659178021	0.886763308489484	0.847357069855384	1.04650487974429	0.295327993153902	0.604505480400887
**Zfp748**	2122.97570414796	-0.121245975996159	0.115870685602411	-1.04639042537638	0.295380811495687	0.604535062571442
**Thap12**	3519.4126579919	0.17276767271606	0.165167219530418	1.04601671692028	0.295553314328085	0.604809555047744
**Aldoa**	66544.0864186378	0.0466508344628976	0.0446060588709795	1.04584075893888	0.295634559419786	0.604897253964105
**Ablim1**	85565.4792304362	-0.0530970926392237	0.0507904167313458	-1.04541557357324	0.295830942009578	0.604906326660385
**Echdc2**	67.242029579981	-0.48812869568047	0.466911021463657	-1.04544265018697	0.295818433389328	0.604906326660385
**Kyat1**	1717.63381834708	-0.0875051532236409	0.083695430339722	-1.04551888757194	0.295783215802377	0.604906326660385
**Patl1**	873.45091326175	-0.155418956608989	0.148644990595499	-1.04557143827284	0.295758941828595	0.604906326660385
**Dtnb**	3497.66466244536	0.160011665409909	0.153036552049239	1.04557808750439	0.295755870541736	0.604906326660385
**Zfp39**	1115.25185442608	0.155621495718622	0.148909256328243	1.0450760386284	0.295987827491112	0.605148591900539
**Glud1**	13369.0332529544	0.188919800694107	0.18080979920942	1.04485377186495	0.296090558203176	0.605280088947515
**Gm18853**	606.817534427014	-0.228595627078727	0.218806194001712	-1.04474020089641	0.296143059411816	0.605308884051367
**Rhbdd3**	668.344071590029	-0.158302295587463	0.151544809585155	-1.04459067929022	0.296212189261253	0.605371655796311
**Gm4956**	90.4417826136221	0.506360477402035	0.484810950400852	1.04444934047666	0.296277545804604	0.605414555894517
**Gm43359**	126.905619986807	-0.401869439247183	0.384792682256582	-1.04437911056535	0.296310024437299	0.605414555894517
**Itsn2**	12341.3774264334	-0.0812754290529185	0.0778429678228017	-1.04409468608559	0.296441584180199	0.605480998462083
**Mnat1**	696.978592886276	-0.120597240963846	0.115513313604183	-1.04401161390871	0.296480016354769	0.605480998462083
**Spryd7**	952.279931826319	0.116629803503135	0.111691052097306	1.04421796834295	0.29638455552118	0.605480998462083
**Nol6**	7451.48391727852	-0.109874850116158	0.105246475302612	-1.04397653033262	0.296496248279078	0.605480998462083
**Selenoo**	5026.12433444796	0.242578546033063	0.232385981206005	1.0438604978414	0.296549936625425	0.605512161644009
**Sirt4**	446.924348499951	-0.15475456026803	0.148280833745035	-1.04365855221806	0.296643392570004	0.605546046991262
**Cd3eap**	1784.48217514887	-0.123826589557933	0.118637721306721	-1.04373708626616	0.296607046417849	0.605546046991262
**Gm37759**	18.4977161385349	0.630677127373444	0.604356610851068	1.04355130075488	0.29669303417172	0.605568929979765
**Orc1**	73.5650512465468	0.683450065847871	0.655010456462208	1.04341855783382	0.296754482251187	0.605615901674907
**Fuca2**	3267.45749320443	0.144447367828798	0.138518057165276	1.042805326503	0.297038463902052	0.605965951000136
**Ncf2**	144.342224335775	0.326251372239943	0.312849005391516	1.04283972976566	0.297022527265582	0.605965951000136
**Snx21**	256.975301136032	0.249119530479262	0.238895052956048	1.04279903412271	0.297041378785622	0.605965951000136
**Adprh**	5297.89072986382	0.0911799462424751	0.0874518223406518	1.04263060279409	0.297119410057873	0.606046672398952

**Ppwd1**	574.611274039679	-0.134225826879445	0.128772613410556	-1.04234761821213	0.297250542692663	0.606235671914095
**Dhx58**	1986.27220951171	-0.0977334325463587	0.0938683334397714	-1.04117575080916	0.297793987839084	0.606366303657864
**Pafah1b1**	5750.05927541703	0.0566533131500381	0.0544195099734557	1.04104783702889	0.297853347103628	0.606366303657864
**Atp6v1d**	8527.70791334246	-0.0751374086956397	0.0721692822080755	-1.04112728292082	0.297816478763969	0.606366303657864
**Atl3**	1220.99076359559	-0.0972546583557272	0.0933240632487853	-1.04211770223146	0.297357112275238	0.606366303657864
**Rnf115**	9936.9643599248	0.0772885006435098	0.0742265926772775	1.04125082205437	0.297759154195405	0.606366303657864
**Htt**	2959.31677145019	-0.174662549887874	0.167630509480206	-1.04194964526132	0.29743502541308	0.606366303657864
**Fbxl14**	3686.80276805322	0.196098207987905	0.188186987726032	1.04203914605079	0.297393530122812	0.606366303657864
**Trim30a**	9562.62441392403	-0.0932424715204535	0.0895046028840627	-1.04176174761909	0.297522153069617	0.606366303657864
**Sclt1**	484.138783716828	0.348964569884469	0.335031476861116	1.04158741487185	0.297603005972852	0.606366303657864
**Zcchc3**	112.027383233971	-0.403216280162493	0.387227229241298	-1.04129113273496	0.297740450848546	0.606366303657864
**E130307A14Rik**	1018.32827808059	-0.173696238621525	0.16678142840455	-1.04146031295644	0.297661963182233	0.606366303657864
**Gm10075**	516.984109679604	-0.150230061262696	0.144231957507221	-1.04158651008515	0.297603425637604	0.606366303657864
**Gm42684**	38.5964272622757	-0.546134106486566	0.524400710239567	-1.04144425402679	0.297669412796668	0.606366303657864
**Gm44829**	213.925382513783	-0.375600252977609	0.360514725872332	-1.04184441306461	0.29748381921249	0.606366303657864
**Trim27**	11049.4621341284	0.0833115646489999	0.080062076495795	1.040587108097	0.298067217030596	0.606566594181983
**Per3**	253.798429857461	-0.242256667569769	0.232804304980532	-1.04060218126133	0.298060218459451	0.606566594181983
**Gm6787**	24.1389492149775	1.2563780003904	1.20733680374805	1.04061931723618	0.298052262244902	0.606566594181983
**Morf4l1-ps1**	1334.44994303279	0.101617259661716	0.097668369349615	1.04043161914545	0.298139417998921	0.606635176834839
**Tfe3**	2711.39287609157	-0.0980360599537473	0.0942768916670409	-1.03987369778782	0.298398583767026	0.60708411677431
**Txnl4b**	1831.68135410172	0.0781662737866977	0.0751758674551351	1.03977880711982	0.298442677369154	0.607095438588401
**Naa25**	5881.70023845205	0.0799543212952142	0.0769187733819555	1.03946433074518	0.298588838711677	0.607314358019866
**Dbf4**	1476.52798036305	0.124472528609061	0.119792270002537	1.03906978811257	0.298772280582923	0.607530626551182
**Socs4**	914.094079847112	-0.12565982351676	0.120928114864824	-1.03912827597805	0.298745082008713	0.607530626551182
**Dusp16**	206.105239729153	-0.244713432913057	0.235583142178191	-1.03875612936667	0.298918169308591	0.607563133473226
**Isg15**	318.743182500041	0.37315845068687	0.35925394752169	1.03870382847871	0.29894250008364	0.607563133473226
**Rskr**	23.8897408523251	-0.61928671520264	0.596200002970268	-1.03872309982783	0.298933534750123	0.607563133473226
**Bicra**	2216.78261029442	-0.177052023560435	0.170427064056099	-1.03887269631164	0.298863946225881	0.607563133473226
**Cul7**	269.02677093533	-0.361090218245001	0.347734152916724	-1.03840883967321	0.299079755895739	0.607763697917095
**Cers2**	25203.8727410413	0.0824375784332568	0.0794093239304492	1.03813474731834	0.299207326452792	0.60778778431309
**Ap4b1**	3059.4572234702	0.0944549610383328	0.0909810658584934	1.03818261686715	0.299185043955382	0.60778778431309
**Gm43633**	90.4092047111849	-0.46371679732788	0.446664847503137	-1.03817616255244	0.299188048269448	0.60778778431309
**Mmp9**	69.7842873156416	0.767996582221843	0.739919989047326	1.03794544489961	0.299295454502067	0.607888434724036
**Hmox1**	553.722440196334	-0.160548132182118	0.154725416480131	-1.0376325741074	0.29944114666671	0.607949252419451
**Cep44**	987.132220201533	-0.115574131602502	0.111369857595126	-1.03775055565448	0.29938620154022	0.607949252419451
**Zfp109**	380.077078641981	-0.187398728216647	0.180591919209954	-1.03769165883209	0.299413629508669	0.607949252419451
**Med28**	9872.12758111189	0.0976658554846888	0.0941804592521726	1.03700763683031	0.299732298075225	0.608461971240829
**Cux1**	4799.07429796903	-0.0986964886053564	0.0951964118626693	-1.03676689776644	0.299844506117591	0.608575985622645
**Sos2**	572.852631328384	-0.181627908442723	0.175208518545435	-1.03663857186044	0.299904330034895	0.608575985622645
**Zc3h3**	1246.48353290581	0.0880595860935437	0.0849450496718428	1.03666530814606	0.299891865259723	0.608575985622645
**Gm47773**	42.0859621513237	-0.481136820083188	0.464246706884224	-1.03638176199961	0.300024075450729	0.608740581622089
**Olfr482**	21.4455092850218	0.872892697517047	0.842422269206951	1.03617001760742	0.300122831695268	0.608784173406583
**Rgs3**	6363.9837099306	0.0992068448559012	0.0957416339079737	1.03619335503777	0.300111946202889	0.608784173406583
**Cdkl2**	63.3184663648797	-0.445772513852326	0.430261786136476	-1.03604951268187	0.300179044105364	0.608819822109437
**Parp8**	2792.76081664186	-0.0746546454584178	0.0720774936558342	-1.03575529158782	0.300316320072655	0.60901985268788
**Mir7069**	20.1121381728638	0.507993517581453	0.490513879380447	1.03563535903017	0.300372289511628	0.60905496922336
**Klra13-ps**	1214.61654836857	0.224159389454792	0.216489141792784	1.03543017261046	0.30046806085623	0.609170771347351
**Naglu**	2434.15493603287	0.109155057556506	0.105490322267504	1.03474001415703	0.300790343613176	0.609702683102977
**Calu**	12980.8257374614	0.0783249968898145	0.0756980749790417	1.03470262502052	0.300807809763441	0.609702683102977
**Fcer2a**	90.416696557866	0.40080207913297	0.38742104971102	1.03453872584345	0.300884382427061	0.60977944873353
**Actr3**	101384.805307595	0.0484574554824963	0.046856165833014	1.03417457704903	0.301054556924569	0.610045865928553
**Ryr3**	38.7836663000841	0.453836186592387	0.438981701787694	1.03383850566937	0.301211667123123	0.610285744999235
**Tm2d1**	2028.51701017442	0.1193879995705	0.115504670294876	1.0336205390285	0.301313593658494	0.610390915930756
**Orai1**	5005.9185319225	0.200281659627945	0.193824702102799	1.03331338810325	0.301457263931786	0.610390915930756
**H2-Q7**	95123.7978261979	-0.0730584391669796	0.0707026546337498	-1.03331960511855	0.301454355462008	0.610390915930756
**Gm9387**	101.961696191263	0.263045794459871	0.254521614114508	1.03349098808374	0.301374185732278	0.610390915930756

**Gm20098**	36.8927569738607	-0.820807807831156	0.794228774171512	-1.03346521118851	0.301386242772172	0.610390915930756
**Gm13339**	21.6090381258744	-0.62396945951589	0.603924624825592	-1.03319095441105	0.301514545174504	0.61042845796154
**Slc27a1**	1372.0754066817	0.122660380034214	0.118743233068295	1.03298838059821	0.301609336305018	0.610493226836569
**Cd3e**	32609.1180367966	-0.100206171114893	0.0970634831149773	-1.03237765531444	0.301895235356384	0.610493226836569
**Psme4**	1848.6573210364	-0.108158477988528	0.104743901408076	-1.03259928773466	0.301791461645635	0.610493226836569
**Bicdl1**	3040.95930361627	-0.127690269807162	0.12367642719309	-1.03245438686392	0.301859305083825	0.610493226836569
**Trav3-4**	112.020702950805	0.314222847208524	0.304276387626652	1.03268889728662	0.301749511006957	0.610493226836569
**Gm13991**	191.132085587797	-0.244335993116371	0.236616239185631	-1.03262563024968	0.301779129015862	0.610493226836569
**Gm15975**	41.4339407217253	0.427012400489432	0.413599329924313	1.03243010709803	0.301870674005625	0.610493226836569
**Gm16740**	60.586334991036	-0.322833974209724	0.312562264473331	-1.03286292334009	0.301668051941675	0.610493226836569
**Gm47003**	86.504167745365	-0.267445246286667	0.258987998545335	-1.03265497933817	0.301765389210278	0.610493226836569
**Iws1**	4625.8550092966	-0.0767539278121715	0.0743671087827875	-1.03209508973053	0.302027573899816	0.61061190981404
**Xkrx**	1678.41183248449	-0.204961027729483	0.198602194156455	-1.03201794219866	0.302063712368351	0.61061190981404
**Ints6l**	2682.77824552908	0.100102523757493	0.0969981809337363	1.0320041344474	0.30207018068163	0.61061190981404
**Gm5620**	184.723345347445	0.164446455682244	0.159401427430817	1.0316498310758	0.302236187475519	0.610869113141579
**Lrp12**	300.178835226241	-0.216026409674256	0.209466276246544	-1.03131832744279	0.302391566540774	0.61087104015687
**Slc7a3**	27.4641124865164	-0.886856072367292	0.859894223530417	-1.03135484353666	0.30237444847879	0.61087104015687
**Nfe2**	101.254247442562	0.421545729677215	0.408726603077192	1.03136357287123	0.302370356425451	0.61087104015687
**Gm6745**	36.437563162174	-0.366929187419488	0.355815554768944	-1.03123425185209	0.302430982056467	0.61087104015687
**Gm5854**	233.443193730037	0.178499996565348	0.173082477019034	1.03130021963875	0.302400055379813	0.61087104015687
**Gm20324**	1782.6689277958	-0.0833775416901339	0.0808624028910767	-1.03110393346145	0.302492083434121	0.61091614440803
**Borcs5**	2280.62997867567	0.101425340930248	0.0983739314893693	1.03101847608081	0.302532155633959	0.610918771796013
**Efr3a**	25448.8519152298	-0.0614191742744135	0.0596048219633561	-1.03043969013401	0.30280364966158	0.611024709356464
**Ice1**	2497.88400487976	0.215537470535916	0.209131925758082	1.03062920572559	0.302714734798092	0.611024709356464
**Serp2**	156.638461254263	-0.264735411998332	0.256878884080904	-1.03058456106869	0.302735679128972	0.611024709356464
**Bhlhb9**	2009.06600409838	0.0930314645010003	0.0902858158469963	1.03041063126302	0.302817284722005	0.611024709356464
**Naip2**	360.839879213667	-0.195815804587347	0.189976159545738	-1.03073883089106	0.302663309983268	0.611024709356464
**Gm12940**	509.047403923364	-0.197045868302987	0.191219107946681	-1.03047164281266	0.302788657234339	0.611024709356464
**Rpl28-ps1**	602.982528211646	-0.107673552217191	0.104518895389872	-1.03018264607133	0.302924274547787	0.6111623295838
**Megf9**	363.005024545304	-0.309320591511132	0.300385582489338	-1.02974513273157	0.303129662992272	0.611463668589027
**Oasl1**	759.671318306796	0.194219665306657	0.188617871242116	1.02969916915959	0.303151245733165	0.611463668589027
**Pacs2**	1732.0370186753	0.134556232077174	0.130739242963032	1.02919543533858	0.303387846873112	0.611477619967063
**Rasgrp1**	23064.2390347824	-0.151307586189498	0.14701258303409	-1.02921520775139	0.303378557560816	0.611477619967063
**Cyb561a3**	5883.95160102505	0.0848686621540421	0.0824451696992772	1.02939520245522	0.303294002623226	0.611477619967063
**1700056E22Rik**	237.891204461528	-0.274081608382168	0.266308407440013	-1.02918871776102	0.303391002913149	0.611477619967063
**Traj40**	56.2611915529859	0.658268706338848	0.639597008441108	1.0291929099907	0.30338903332489	0.611477619967063
**Gm37639**	46.7832581618517	-0.434712140562304	0.422265319193013	-1.02947630507066	0.303255908696269	0.611477619967063
**Anp32a**	1487.92946390385	-0.146737070574281	0.142591826749618	-1.02907069724229	0.303446454657252	0.611511163324507
**Nt5e**	8712.79579023213	-0.164607889874377	0.159993610753286	-1.02884039618436	0.303554680620036	0.611572829884914
**Gm8355**	1547.67330496726	0.157717834730095	0.15329352049769	1.02886171716875	0.303544660118288	0.611572829884914
**Clcn5**	184.561288943386	-0.227092521224375	0.220828125490852	-1.02836774400724	0.303776875591097	0.61194224350408
**Gm2531**	36.6478626087563	-0.659035361169605	0.641159646783364	-1.02788028609711	0.304006143929569	0.612325809778629
**Ddx28**	1235.09670479029	0.187087570202846	0.182055263310006	1.02764164463771	0.304118427168969	0.612473678029326
**Ppp4r1l-ps**	896.01437535532	-0.123834944761956	0.120515633386662	-1.0275425791826	0.304165046564495	0.612489282903099
**Rundc3b**	2255.76306684341	0.161412988247102	0.157100752721609	1.02744885336822	0.304209157537635	0.612499833289103
**BC024978**	828.330606605746	-0.176053638347062	0.171418900305202	-1.02703749722818	0.304402807795433	0.612811427422083
**Csf1r**	869.270676729668	-0.211266294654079	0.205787783048028	-1.02662214211605	0.304598423639103	0.612813759101296
**Fgf13**	4628.78480696925	0.116659955218267	0.113615825584702	1.02679318323745	0.30451785992598	0.612813759101296
**Ppib**	7362.07078176565	0.0911793018066739	0.0888027904163421	1.02676167470853	0.304532699996231	0.612813759101296
**Exoc6b**	2186.64548990633	0.138121613865393	0.134538209540131	1.02663484475905	0.304592439959496	0.612813759101296
**Ccdc155**	18.2462275708439	0.976759500824325	0.951380599822989	1.0266758655853	0.304573117310457	0.612813759101296
**Kifc1**	382.49993345177	0.256494368911392	0.249896523472382	1.02640230983341	0.304701989720481	0.612943859571763
**Smim24**	290.637564104746	0.244126368353843	0.237933104679608	1.0260294324431	0.304877710946092	0.613219056851873
**Eif1a**	5825.57442606399	0.139745246016536	0.136282031609392	1.02541211314687	0.305168775036332	0.613578126161335
**4930599N23Rik**	27.8210783184859	-0.591997751768176	0.577292876195136	-1.02547212373372	0.305140472147667	0.613578126161335
**Gm14026**	42.1383966691861	-0.483823535401186	0.471837427624267	-1.02540304578479	0.305173051642215	0.613578126161335

**Capzb**	40052.0543508912	0.055864109151442	0.0544992481204571	1.02504366717075	0.305342583972922	0.613840660329336
**Dclre1c**	1706.96732600508	-0.0740264229722484	0.0722344278711079	-1.0248080472699	0.305453768592388	0.613985844075808
**Cttnbp2nl**	32.5131199187652	0.939194326577835	0.916733544072598	1.02450088430871	0.305598753359916	0.614198923047475
**Gm12359**	103.551307962113	-0.267607407677346	0.261253049643144	-1.02432261764171	0.305682918387707	0.614211393143981
**Gm21985**	73.2349554336414	2.66839200611376	2.6049169122937	1.02436741591277	0.305661766337511	0.614211393143981
**Klrc1**	2431.85467465015	0.579364222997202	0.565804705777489	1.02396501315959	0.305851800506134	0.614315719639918
**Serpinb9**	3731.41757845594	0.21103056575445	0.206064682936383	1.02409866041722	0.305788677079075	0.614315719639918
**Gm9347**	44.787146123273	0.430263807832267	0.420180809600551	1.0239968080439	0.305836782564415	0.614315719639918
**Ak2**	7373.16992752428	0.0628136528852086	0.0613549648185884	1.02377457261907	0.305941763103002	0.614418093450676
**Sfmbt2**	508.939334513535	-0.242035647554882	0.236461712580546	-1.02357225156456	0.306037357177372	0.614531749336542
**Gm5451**	105.950351746744	0.249954135015874	0.244222860129115	1.02346739729331	0.306086907249933	0.614552930369163
**Spn**	7558.78267663011	-0.138603693106596	0.135453501582067	-1.02325662672234	0.306186525356822	0.614674618428773
**Fntb**	7742.15377614888	0.0613859177332092	0.0600178063049652	1.02279509219801	0.306404738990364	0.61487672494185
**Cdc25c**	34.5063540530361	0.653272776638844	0.63886723140409	1.02254857429938	0.306521334925318	0.61487672494185
**Tmem140**	765.746733163563	-0.200785023040806	0.1963511571699	-1.02258130756555	0.306505851331607	0.61487672494185
**Gm26621**	27.0893482833918	-0.71087300019597	0.695188765905705	-1.02256111585726	0.306515402416462	0.61487672494185
**Gm43088**	44.1774565586117	-0.510509925191153	0.499108498067661	-1.02284358444633	0.306381806999773	0.61487672494185
**Gm47545**	26.6495910572337	-0.497105642891364	0.486087055588297	-1.02266792990349	0.306464879458149	0.61487672494185
**Gm5709**	18.9421076034542	1.17432115827564	1.14865293474054	1.02234637004684	0.306616993713337	0.614990322039339
**Large1**	30.6260592729851	0.91004082642443	0.890377555084915	1.02208419476347	0.306741053041765	0.615160846732733
**Gm6169**	267.624564488437	0.153726659547655	0.150443081347098	1.02182604989977	0.306863237691876	0.615170996986118
**Eloc**	2115.57443868333	0.118749260112288	0.116205349900861	1.02189150683335	0.306832252686503	0.615170996986118
**F730311O21Rik**	56.8451227989565	-0.314483905854992	0.307748284469108	-1.02188678776067	0.306834486459871	0.615170996986118
**Fpgs**	2973.6092399781	0.0886118086915402	0.0867600414371597	1.02134354967686	0.307091700236627	0.615227889558789
**Dedd**	1675.92226414189	0.108529682747541	0.106277769248175	1.02118894210235	0.307164930326775	0.615227889558789
**Knstrn**	690.30526688597	0.165518258554281	0.162069687636361	1.02127832149376	0.30712259424513	0.615227889558789
**Tab3**	724.081788588554	-0.217916178390785	0.21337651062131	-1.02127538666865	0.307123984314016	0.615227889558789
**Hic2**	222.025527840809	-0.282547434217425	0.276672490130408	-1.02123429071047	0.307143449697852	0.615227889558789
**Phtf1**	3930.46673198057	-0.118178219269245	0.115704051549409	-1.02138358758145	0.307072738112969	0.615227889558789
**Gm6421**	450.236074415703	0.11798994974902	0.115511740677749	1.02145417475947	0.307039309610766	0.615227889558789
**Bub3**	13382.0000767571	0.0789822025156253	0.0773674641261844	1.02087102644087	0.30731554785009	0.615423360996274
**Ndufa12-ps**	780.001921318443	0.174875698567869	0.171309366776351	1.02081807818584	0.30734063766451	0.615423360996274
**Mocs1**	2441.04999478059	0.118560729602547	0.116172857147941	1.02055447815633	0.307465566133179	0.615595289143646
**Gm14399**	153.209856024037	-0.311735926060136	0.305507735351593	-1.02038636010763	0.307545260197897	0.615676618592079
**Znhit3**	1347.22530851099	0.177123678044546	0.173641572106336	1.02005341172607	0.307703130181766	0.615720518067125
**Slc35b1**	1991.22281951297	0.107583953090178	0.10547338068268	1.02001047462248	0.307723493036657	0.615720518067125
**Dnmt3b**	803.705062595231	-0.169825620556176	0.166461106606437	-1.02021201239334	0.30762792179215	0.615720518067125
**Morf4l1**	9171.97695136396	0.0602442434657526	0.0590566110083907	1.02011006790067	0.307676262443483	0.615720518067125
**Gm37399**	31.7313030039433	-0.427828077960169	0.419550025479415	-1.0197307876964	0.307856155953577	0.615907751315453
**Zkscan17**	3399.03540741328	-0.104948489262956	0.102938159478227	-1.01952949027765	0.307951660069663	0.615942410220573
**Tyrobp**	555.176872318854	0.19347866153678	0.189767849533385	1.01955448202907	0.307939801846748	0.615942410220573
**1700010I14Rik**	446.923121029366	0.163936497774184	0.16082415182324	1.01935247856532	0.30803565820404	0.615954043949373
**Ggta1**	2167.68540009551	0.0954406105625656	0.0936278110518872	1.0193617632444	0.308031251928388	0.615954043949373
**Gm10184**	364.751209701102	0.213674241275754	0.209726133796243	1.01882506203708	0.308286025318718	0.616376462498037
**Nmd3**	6998.43693451637	0.0757853863459905	0.0744106514681701	1.01847497435778	0.308452287899932	0.616630639487342
**Magi3**	255.893526405228	-0.203278965402313	0.199645643830546	-1.01819885223667	0.308583464807107	0.616736384445153
**Perm1**	237.909807771936	-0.258644403691985	0.254002227325465	-1.01827612464426	0.308546751407676	0.616736384445153
**Sppl2a**	6053.6069715579	-0.0903970943635164	0.08880188098887	-1.01796373406602	0.308695191106977	0.616742762087452
**Zcchc17**	3635.9672472634	0.130391803881763	0.128085856614908	1.01800313733146	0.308676465120663	0.616742762087452
**Gm13622**	39.764811815855	-0.511176140826794	0.502164776936126	-1.0179450337908	0.308704078478374	0.616742762087452
**Ap2a1**	3057.21939249382	-0.100189629231564	0.0984336909765684	-1.01783879317716	0.308754572913529	0.616765441860862
**Trmt6**	2521.18363615679	0.106685132114867	0.104824180525726	1.01775307548132	0.308795317119937	0.616768641381525
**Fbxw2**	12077.5743962904	-0.0410705815457139	0.0403637981760832	-1.01751032860058	0.308910721321761	0.61692094244195
**Gm37169**	210.59734193536	-0.200793997681336	0.197379224840237	-1.01730056870911	0.309010466151931	0.617016303664129
**Gm35638**	107.352132168568	0.425333190737185	0.418122574021886	1.01724522224653	0.309036788000083	0.617016303664129
**Lacc1**	68.8503206545911	0.435554625229939	0.428344012806431	1.01683369489926	0.309232549972129	0.617250733457163

**Gm26885**	100.86350238681	0.282684212794536	0.277987672822535	1.01689477783067	0.309203487879044	0.617250733457163
**Erp27**	131.161860509047	-0.307493296075553	0.302480686533926	-1.01657166809249	0.309357237806735	0.617421405461776
**Myh10**	128.170180349362	-0.326934038949618	0.321656393279891	-1.01640771263991	0.309435274392295	0.617498938525758
**Ubr2**	8823.84123181379	-0.139680524302698	0.137438716973316	-1.01631132317553	0.309481158197064	0.61751229703826
**Med7**	955.497475058317	0.172855190933191	0.170228503958209	1.01543035927536	0.309900727415485	0.618192850875059
**Pdcd5**	2112.86850319752	0.109770136478389	0.108128377049913	1.01518342800724	0.3100183986555	0.618192850875059
**Stk26**	6710.35982230139	-0.0812967878254991	0.0800769503983606	-1.0152333152183	0.309994623306037	0.618192850875059
**Gm12504**	55.3800841148473	-0.641134861721529	0.631387397283688	-1.01543816756523	0.309897006978059	0.618192850875059
**8030456M14Rik**	54.6009050746843	-0.449650059407234	0.442867237740641	-1.01531569980475	0.309955362926884	0.618192850875059
**Tsg101**	5984.57119128741	0.0978783665747135	0.0964783827746395	1.01451085476157	0.310339052542818	0.618612292868053
**Sfrp2**	128.101078639349	0.26806918600643	0.26422444098561	1.01455105745131	0.310319879485686	0.618612292868053
**Armcx5**	845.612271803295	0.175482605186419	0.172975295536335	1.01449518928301	0.310346523774954	0.618612292868053
**Gpsm2**	2209.76159352609	-0.137430653204065	0.135504501144785	-1.01421467215485	0.310480329036483	0.618800726616224
**Rpl12**	8416.50171164475	-0.0765161714953286	0.0754661902152106	-1.01391326734692	0.310624140048081	0.618942126893721
**Prmt7**	7789.3051760299	0.089518111481149	0.0882907526256133	1.01390132963007	0.310629836864603	0.618942126893721
**Snrpc**	3255.12678052214	0.0936954462432163	0.0924383287736404	1.01359952615169	0.310773883881734	0.61899432288263
**Gm9392**	123.416824473335	-0.214839421463027	0.211954196663274	-1.01361249196842	0.31076769455392	0.61899432288263
**Gm7993**	17.8332622756912	0.620969185931591	0.612637372633183	1.01359991027416	0.310773700516886	0.61899432288263
**Acot9**	5975.38031814709	0.0836646184969088	0.0825930912380876	1.0129735700984	0.31107278535544	0.619511359813659
**Bank1**	517.555408109956	-0.172058553100184	0.169956184875981	-1.01237006011718	0.311361148073228	0.619831448758935
**Pcsk9**	33.0351360866724	0.863760635459316	0.853148840926167	1.01243838592294	0.311328492517	0.619831448758935
**Itpa**	1902.93048649283	0.132359183933125	0.130746802198607	1.01233209307918	0.31137929497162	0.619831448758935
**D030055H07Rik**	49.7393472207376	0.470153379849998	0.464437133240157	1.01230790176048	0.311390857928269	0.619831448758935
**Birc5**	312.867016606433	0.345373782472182	0.341303362976642	1.01192610427287	0.311573386837004	0.619972702235922
**Ube2j2**	5240.77840907499	0.203117505907403	0.200697486130585	1.01205804728039	0.311510299836457	0.619972702235922
**4930589O11Rik**	75.4314675022703	-0.437414508814975	0.432300270957656	-1.01183029065883	0.311619204271657	0.619972702235922
**Gm18078**	28.3227642615822	-0.481771199459501	0.476117731425985	-1.01187409680497	0.311598255911968	0.619972702235922
**Senp3**	1747.49611375408	-0.116964523508212	0.11561668420605	-1.01165782699459	0.311701686445822	0.620058511971572
**Pmm2**	10046.9663259081	-0.0600090044798931	0.0593298338099069	-1.01144737185953	0.311802357852319	0.620180478752714
**Gm11707**	1001.32400013036	0.243403802554372	0.240673572326318	1.01134412142415	0.311851755629054	0.620200443449073
**H2aj**	6585.03893783603	-0.0916138746785657	0.0906127421388694	-1.01104847415568	0.311993229742184	0.62040349836542
**Sri**	25723.0921839235	0.0759050787916718	0.0750841201599735	1.01093385165797	0.312048090658145	0.620434292050523
**Hacl1**	981.473891274887	0.130876025860787	0.129497718606093	1.01064348676973	0.312187094302044	0.620567327900665
**A130051J06Rik**	138.125671247907	-0.223705592079546	0.221352712917457	-1.01062954743621	0.312193768373965	0.620567327900665
**Cntf**	40.8502944803855	-1.14963098057015	1.13763930804771	-1.01054083876815	0.312236243772018	0.620573472895521
**Gm20712**	18.1949563471223	-0.989423282753334	0.979217172461346	-1.01042272396667	0.312292805296666	0.620607609163774
**Rasa4**	653.636301844952	0.13159833494797	0.130253467504782	1.01032500300338	0.312339605946085	0.62062234185277
**Wdr5**	4461.2107584409	0.0698662693933974	0.069158477165893	1.01023435241072	0.312383024575965	0.620630351562664
**Abhd13**	2020.17686544995	0.166112896915133	0.164495683467685	1.00983134276448	0.31257610097657	0.620779130714371
**Gm13835**	5594.99885735778	0.0755913122113609	0.0748473153846094	1.00994019388576	0.312523944148532	0.620779130714371
**E230016M11Rik**	49.5816682532784	-0.571654106544863	0.566045862056815	-1.00990775635682	0.31253948623317	0.620779130714371
**Mfng**	11160.6504568552	0.0573744984141708	0.0568249911062079	1.00967016971346	0.312653338777868	0.620854273361419
**Epha2**	28.3786666952108	1.18533500980927	1.17414540209521	1.0095300017307	0.312720520690968	0.620882296065636
**Gm27252**	142.41111665499	0.288950891262348	0.286238407590986	1.0094763092563	0.312746257782126	0.620882296065636
**Pbx3**	40.4419498552243	0.544876991379225	0.539853734522101	1.00930484784285	0.312828455885142	0.620889026247913
**Gm12918**	2375.38356680626	0.117344900642531	0.116261657988791	1.00931728200405	0.312822494505996	0.620889026247913
**B430212C06Rik**	30.5374450250871	-0.386744824323317	0.383230935363016	-1.00916911615439	0.312893535350198	0.620903301585108
**Gm38200**	87.0586385275319	-0.363382797953524	0.360096741342961	-1.00912548277529	0.3129144582019	0.620903301585108
**Tyk2**	11452.8372013905	0.0835355032172045	0.0827950076774846	1.0089437220974	0.313001625051894	0.620919879887032
**Gcnt1**	92.0672690166182	0.537900516337574	0.533109495077023	1.00898693665146	0.312980899227719	0.620919879887032
**Nipal3**	1366.24915198202	-0.120108894199658	0.119071944600659	-1.00870859716348	0.313114407479559	0.621065422791468
**Gm6345**	19.7026991833605	-0.709470588863282	0.703485478521986	-1.00850779514862	0.313210747404551	0.621178319301889
**Tsr3**	3641.63549260304	0.0852628268621521	0.0845526989416533	1.008398642851	0.313263124209142	0.621204008074937
**Mcu**	882.034942209376	0.0981138584484144	0.0973236893624244	1.0081189799849	0.313397346935896	0.621391971268266
**Tbc1d32**	178.124315720244	-0.358712408734665	0.355960104673975	-1.0077320576788	0.313583110672904	0.621603858959989
**Cenph**	76.4606068756726	-0.361476823509341	0.358682279209225	-1.007791141247	0.313554739608162	0.621603858959989

**Zfp286**	289.813141799837	-0.269045004107508	0.267060285997259	-1.00743172315134	0.313727353035647	0.621811559972665
**Cndp2**	8229.39221690662	0.0861926502424983	0.0855778379644219	1.00718424644394	0.313846242052364	0.621819018498388
**Atp8a1**	3771.68143118279	-0.151059468042383	0.149982972050389	-1.00717745472887	0.313849505243237	0.621819018498388
**Ubald1**	7234.88261638402	-0.0991728210082106	0.0984527534206278	-1.00731383899957	0.313783981460492	0.621819018498388
**Dhx37**	1444.46819877247	0.151094552123031	0.150034063223984	1.00706832086166	0.313901943460367	0.621844722542746
**Sun1**	6252.34026829298	0.0861691701038376	0.0855964619814487	1.00669079199223	0.314083388440492	0.622047756618505
**H3f3a**	22064.994347641	-0.0531599027043189	0.0528042460142866	-1.00673538052103	0.314061955057651	0.622047756618505
**Susd2**	118.900531446404	0.650657665358922	0.646698794968264	1.00612166037955	0.314357050398304	0.62232729305044
**Enoph1**	2098.41866300745	0.0857568677845838	0.0852395724065759	1.00606872328665	0.31438251270429	0.62232729305044
**Ptpn11**	3752.72479333884	-0.113390910440078	0.112698432397152	-1.00614452240547	0.314346054371299	0.62232729305044
**Gm5763**	358.511825041946	0.227707112899354	0.226298790491299	1.00622328738478	0.31430817244593	0.62232729305044
**Slc25a1**	2621.10270637408	0.109134770740914	0.108561083967533	1.00528446062267	0.31475989545967	0.622849764138144
**Rplp1**	17675.5258546268	-0.078272488957618	0.0779000131679114	-1.00478145990686	0.315002093227541	0.622849764138144
**S100a11**	4040.91979233346	0.10827835233363	0.10776311271209	1.00478122437792	0.315002206664776	0.622849764138144
**Pygm**	304.840832111149	-0.193332047417295	0.19235148553216	-1.00509776091628	0.314849778164502	0.622849764138144
**Rbmxl1**	4228.57969269015	0.0751236738361634	0.0747265650812187	1.00531415774983	0.314745599948134	0.622849764138144
**Scd1**	75.1258389960514	0.552415493890864	0.549628151183901	1.00507132449631	0.31486250679444	0.622849764138144
**4933415A04Rik**	30.025106261543	-0.592275717864054	0.589362643434754	-1.00494275377266	0.31492441595786	0.622849764138144
**Adam17**	6891.96183565598	0.101454567450356	0.10095869352974	1.00491165152082	0.314939393464657	0.622849764138144
**C030037D09Rik**	36.0836904046333	0.582372183349632	0.579358414015289	1.00520190828585	0.314799636491262	0.622849764138144
**Kif18b**	192.486997228396	0.488066664281051	0.485888736268137	1.00448235954108	0.315146169841427	0.622899924509704
**Rpl7a**	3995.33901482597	-0.336965519027508	0.335407075611753	-1.00464642379089	0.315067134749369	0.622899924509704
**Gm37101**	106.523920213543	-0.335033644821467	0.333514916683994	-1.00455370378205	0.31511179939654	0.622899924509704
**Pcyt1a**	866.501666970128	-0.169463215255611	0.168745085507264	-1.00425570763254	0.315255376874598	0.622901184032313
**Gipc1**	2936.72700378517	0.08516814837059	0.0848089904969677	1.00423490329879	0.315265402212204	0.622901184032313
**Gm15751**	108.747311434608	-0.27858748917778	0.277376794575447	-1.00436480133166	0.315202809470082	0.622901184032313
**Sp2**	7664.48250884576	0.0910917900160024	0.0907697999166299	1.00354732630972	0.315596854466401	0.623477888142813
**Hk3**	358.891664027041	0.204082661318231	0.203413474212447	1.00328978750486	0.315721062103067	0.623566905936077
**AI847159**	156.546214794985	0.290117380710587	0.289164700277441	1.00329459450698	0.315718743454117	0.623566905936077
**Rab5b**	7607.14826242501	-0.0774165209298892	0.0771820429411823	-1.00303798629541	0.315842533604626	0.623728637925566
**Fbxo3**	5301.42992416076	-0.0697910462492767	0.0696023068784704	-1.00271168269086	0.315999991474689	0.623906759089861
**Plekhj1**	5002.40009518646	0.0866698541117867	0.0864375993475572	1.00268696453838	0.316011921323613	0.623906759089861
**Txnrd1**	6537.89942534462	0.0716809381565453	0.0715062375489015	1.00244315200509	0.316129609661167	0.624030073716924
**Pogk**	1763.79912011131	-0.173640781841938	0.17324034425348	-1.0023114568964	0.3161931908711	0.624030073716924
**Gm6560**	556.493856262108	0.108652566716165	0.108395987673624	1.00236705295138	0.316166348573782	0.624030073716924
**Rnf6**	9284.99528300488	-0.104804942952243	0.104586786446437	-1.00208588974972	0.316302112153284	0.624166860513374
**Ms4a4c**	15501.346864538	-0.146631600476861	0.14639457213174	-1.00161910610256	0.316527589636575	0.624299064952249
**Sh3glb1**	5288.06256187693	0.163593977084386	0.163320112857672	1.00167685548291	0.316499688374375	0.624299064952249
**Impdh2-ps**	1364.94070313242	0.101543461053157	0.101363316843525	1.00177721305144	0.316451205064349	0.624299064952249
**Gm49959**	20.8308888548927	-0.512510443585967	0.511633625999661	-1.00171376067121	0.31648185870216	0.624299064952249
**4930533K18Rik**	37.0494727468488	-0.831505220443928	0.830231669809971	-1.00153397019202	0.316568725477919	0.624302053486304
**A830082N09Rik**	17.1016428844851	-1.18666279930835	1.18499647835223	-1.00140618220101	0.316630476471207	0.624345691122114
**Ercc4**	4540.53474897982	-0.0940910510475103	0.09398519956437	-1.00112625693866	0.316765772376868	0.624352057299651
**Tmx4**	2019.71830334871	-0.0875617746231747	0.0874800497765478	-1.00093421124971	0.316858615497098	0.624352057299651
**Camta2**	5092.09046847016	-0.108389207183304	0.108290914695479	-1.00090767067672	0.316871447751813	0.624352057299651
**Gigyf2**	5766.90960319061	-0.133166758545259	0.133036465776036	-1.00097937635718	0.316836779142438	0.624352057299651
**Rap2c**	1720.12480772014	0.0995650685057189	0.0994408747206938	1.00124892088262	0.316706480707897	0.624352057299651
**Rps13-ps2**	1100.70369362238	-0.565526443074893	0.564999993453768	-1.00093176925172	0.316859796178551	0.624352057299651
**Tmem151a**	36.2659726524249	1.99540443145054	1.99398089609289	1.00071391624686	0.316965137526677	0.624458573644391
**Gm42417**	107.052021340283	-0.66347662733957	0.663066641136343	-1.00061831824705	0.317011370544094	0.624471579655369
**Fam161b**	77.5067448052796	-0.376934112917306	0.376783760498554	-1.00039904166399	0.317117433591844	0.624602425138336
**Med29**	1272.1645578075	0.0987551740200383	0.0987589710583194	0.999961552472242	0.317329114572893	0.624804672764996
**Gm10132**	1562.06520651748	-0.125215889385107	0.12522328097438	-0.999940972723162	0.317339074451879	0.624804672764996
**Trbv24**	208.089927236051	-0.191620707101418	0.191630110341904	-0.999950930255849	0.317334255328629	0.624804672764996
**Psma6**	9000.01884207462	0.0764118662992941	0.0764397028041734	0.999635837086513	0.31748677347981	0.624939438800371
**Vdr**	17.0695553727162	1.54388198094739	1.54445365427776	0.999629854007737	0.317489670002703	0.624939438800371

**Gm49703**	43.625450650779	-0.505682658156685	0.505908409932563	-0.999553769473988	0.317526505491894	0.624939438800371
**Dr1**	3319.97834340492	-0.0910870921940617	0.0911372408063424	-0.999449746208718	0.317576871743713	0.624960505565841
**Ncr1**	26.1889075287045	0.839766791844895	0.840454626831169	0.999181591766747	0.317706731599266	0.625045913424456
**Fbxo24**	34.8575601005554	-0.38618910350503	0.386484104878955	-0.999236705028226	0.317680038914378	0.625045913424456
**Gm4366**	1243.22424491342	0.105555202365339	0.10564876479637	0.999114401089202	0.317739275657162	0.625045913424456
**Samd8**	1032.31856300576	0.0927026904479896	0.092864457999124	0.998258025140944	0.318154255954478	0.625784123214918
**Gm23887**	93.5497517443072	-0.29495873345275	0.295589255867117	-0.997866896709365	0.318343905957665	0.626078997275952
**AA413626**	31.2207545692753	-0.58226192004822	0.583651428113723	-0.997619284390354	0.318464006266819	0.626237033164391
**Cox4i1**	13514.1516790282	0.050505684830437	0.0506349602620479	0.997446913536775	0.318547629449526	0.626323308864011
**Kif16b**	2835.56072051244	-0.109467090494545	0.109767747868605	-0.99726096799927	0.31863785431211	0.626422541534113
**Gm49373**	73.6413231098271	0.55279271432925	0.554440173678696	0.997028607543868	0.318750624232069	0.626566066120848
**Cfap298**	1387.98129464513	0.132608359298701	0.133017635506151	0.996923143266738	0.318801817113583	0.626588528409471
**Fam186b**	83.9032371563039	0.343175559482699	0.344271375380711	0.996816999679976	0.31885334517016	0.626611643782267
**Srpk3**	78.7819436180473	0.361043018673504	0.362277286254631	0.996593030730999	0.318962090162916	0.62674718227922
**Grifin**	92.8173146613371	-0.396503900548484	0.397940415666051	-0.996390125101611	0.319060629113361	0.626818269358081
**Rab11fip2**	618.323473466975	-0.128657827897544	0.129139153380571	-0.996272815250622	0.31911760847817	0.626818269358081
**Tmem67**	123.155256991923	-0.314594771529149	0.315764937844473	-0.996294185404779	0.319107228139298	0.626818269358081
**Exoc3l**	218.649601521861	-0.256193085078029	0.257267882623982	-0.995822263024087	0.319336511169154	0.627065000106698
**Lrrtm2**	30.2129396450766	-0.669896076248142	0.672642053025024	-0.995917625482181	0.319290170722647	0.627065000106698
**Gm11960**	18.9928243220405	-0.489247944713328	0.491326964449557	-0.995768561697895	0.319362608736197	0.627065000106698
**Yipf2**	2805.49075889288	-0.119648309527717	0.120171783104983	-0.995643955979183	0.319423169527305	0.627079661656138
**Fem1a**	4541.29781916783	-0.0890962706367347	0.0894909763075956	-0.995589436084547	0.319449669614382	0.627079661656138
**Pabpn1**	2791.52042982234	0.215194840990205	0.216250330709024	0.995119130151808	0.319678327508974	0.627195877135718
**Paqr5**	51.9254551511268	0.537312920551277	0.539868616837009	0.995266077326915	0.319606871810403	0.627195877135718
**Wdr36**	3983.88708051669	0.0574707913879059	0.057750038417614	0.995164556814859	0.319656236863713	0.627195877135718
**Nkrf**	205.002603937347	-0.264314509721241	0.265551517800507	-0.995341739751623	0.319570083675271	0.627195877135718
**Gm30124**	23.5051205329526	-0.90770538941459	0.912213242915405	-0.99505833363435	0.319707893961673	0.627195877135718
**Sms**	2811.64581801743	-0.0817484676024623	0.0821786306531969	-0.994765512064202	0.319850323474161	0.627397180005273
**Ovca2**	2009.55600060621	0.0729447850546293	0.0733487332519836	0.994492772002393	0.319983022588782	0.627579348634733
**St8sia1**	2079.32032482845	-0.184223500641613	0.185286397428047	-0.994263492619064	0.320094604169255	0.627720059476659
**Ggt7**	260.189595228179	-0.286289324357396	0.288195796655373	-0.993384801860046	0.320522465202824	0.628480896490903
**Inpp5e**	4437.09754751243	-0.100714069844297	0.101415454883911	-0.993084041870971	0.320669000144111	0.628689988213358
**Tshz3**	24.2398693794495	0.69845883992122	0.703696760612513	0.992556565577005	0.320926100498695	0.628912480452718
**Ippk**	1848.11489556601	-0.134883555395696	0.135873563704328	-0.992713753274431	0.320849470625995	0.628912480452718
**Zbtb8a**	1936.7026903726	0.0840492925270156	0.0846716625210939	0.992649607016713	0.320880740837686	0.628912480452718
**Cacul1**	2497.03786919895	-0.0958869196784824	0.096609201629542	-0.992523673326384	0.320942137165723	0.628912480452718
**Gm13123**	62.5573821798079	-0.471020939504408	0.474628351715817	-0.992399501213174	0.321002682188065	0.628952905152615
**Thumpd3**	5662.69320646577	0.0814341277418141	0.0820730111517551	0.992215669914198	0.321092330104511	0.62905033513077
**Rpl21-ps15**	2797.93893468359	-0.0588438759270849	0.0593176356580793	-0.992013172377179	0.321191099802639	0.629165609098731
**Arpc5l**	7678.59248628287	0.0830705361316274	0.0837526257450462	0.991855901742172	0.321267823428899	0.629237674800392
**Nqo1**	19.2975178206147	0.63707856672193	0.642785787459523	0.991121115542785	0.321626443484569	0.629473128987119
**Srp19**	3213.30579387223	0.103827381085861	0.104757789705237	0.99111847794809	0.321627731260728	0.629473128987119
**Ttc4**	5636.25658897257	0.0788407366887095	0.0795293552655326	0.991341328311742	0.321518938932633	0.629473128987119
**Kif18a**	396.401412093547	0.142865348107722	0.144097909029948	0.991446364971405	0.32146766988569	0.629473128987119
**2810414N06Rik**	171.424136313792	0.208095212119753	0.209899736036262	0.991402924317175	0.321488872891824	0.629473128987119
**Bcl2a1a**	124.197615058996	0.219752363416547	0.221712281959846	0.99116008131812	0.321607419272074	0.629473128987119
**Atp7a**	1454.61955778754	-0.133739148103151	0.134952742639562	-0.991007262893118	0.32168203383314	0.629501217971781
**Gm11878**	68.4512612885755	-0.248931435064993	0.251349055614927	-0.990381421788043	0.321987722611498	0.630012334906515
**2310043M15Rik**	30.6798203109702	-0.737678957884284	0.744897881149376	-0.990308841724784	0.322023186224349	0.630012334906515
**Tep1**	1271.01615857449	-0.171823049908008	0.173525105163434	-0.99019130255635	0.322080622878355	0.630046477305307
**Ppp6r3**	7415.40264616582	-0.0871755732685471	0.0880630072517569	-0.989922738151869	0.322211884566287	0.630225008082298
**Hmgn1**	8100.61138961122	0.0939221496751229	0.0949122178257933	0.989568591132412	0.322385028380126	0.630407159615989
**Igkj2**	36.5757716922338	0.768983478168594	0.777054209102258	0.98961368352539	0.322362979167339	0.630407159615989
**Itm2c**	13170.6602083414	0.0602424394706621	0.0608853135705849	0.989441228726244	0.322447311178526	0.630450711284283
**Aprt**	4421.80429878593	0.0625207224608096	0.063227102674456	0.988827888931059	0.322747356686617	0.630724308484562
**Gm5457**	17.6261900887457	-0.640429701042077	0.647653565397284	-0.988846098066679	0.322738446165088	0.630724308484562

**Esco2**	66.6558886895867	0.482161887953295	0.487498342116546	0.989053390130348	0.322637020457699	0.630724308484562
**Anapc5**	35623.6750147155	0.0473425415268578	0.0478706347786791	0.988968325691463	0.322678639028562	0.630724308484562
**Cdk1**	170.857321545254	0.466725693555128	0.47206328188043	0.988693066099019	0.322813336350244	0.630775017469096
**Gm6136**	1862.86777950735	-0.0926337401773615	0.0937033566313689	-0.988585078566445	0.322866189685643	0.63080006830461
**Nadsyn1**	1947.98403362437	0.175897720434897	0.177953032255907	0.988450256818026	0.322932184659243	0.630850785231304
**Dusp7**	1563.47687206865	-0.110933170971382	0.11225600200576	-0.988215943818217	0.323046901325464	0.630996656028926
**Spef1**	41.7418881489236	-0.434287273200882	0.439663052796589	-0.987772955763483	0.323263855235742	0.631180511643545
**Kpna4**	17215.6364174903	-0.0713063167317417	0.0722016384718443	-0.987599703288565	0.32334873166521	0.631180511643545
**Gfpt1**	1169.17660916834	0.103433865298338	0.104734905920592	0.987577774469572	0.32335947563445	0.631180511643545
**Brd7**	4740.34910293034	0.0984392682709766	0.0996819951581207	0.987533085737571	0.323381371485584	0.631180511643545
**Zbtb34**	98.6078430106715	-0.290538103007274	0.294094676286211	-0.9879067063578	0.323198340721428	0.631180511643545
**Gm37677**	32.1670509657985	0.46635119896303	0.472098746510802	0.987825539486704	0.32323809733003	0.631180511643545
**Dynlt1-ps1**	624.18621580546	0.128781959131369	0.130493623305108	0.986883158499346	0.323699921382242	0.631645778479256
**5430431A17Rik**	921.751974359707	0.267178900705297	0.270718037846078	0.986926851387742	0.32367849970735	0.631645778479256
**Pcmtd2**	5972.61576659584	-0.0736655478417813	0.0746905088772474	-0.986277225167248	0.323997092574147	0.631943143474662
**Prpf38b**	9743.820515526	0.0668735913509735	0.0677985869495821	0.986356712724758	0.32395809893376	0.631943143474662
**Atxn1**	1311.37827584522	-0.159335093143576	0.161536886356314	-0.986369718629577	0.323951719011439	0.631943143474662
**Rslcan18**	37.9176764263108	-0.719097102392713	0.729125981324584	-0.986245341424192	0.324012734412121	0.631943143474662
**Mpdu1**	3527.92901777699	0.0784050906428594	0.079507278567382	0.986137270142023	0.324065756736048	0.631968332783404
**Prune1**	1091.26414236351	0.0845513462767824	0.0857931052021452	0.985526122146565	0.324365706716408	0.63204370453864
**Txndc17**	2692.69959480381	0.0911308884619892	0.0924590027908457	0.985635640783831	0.324311941939497	0.63204370453864
**Tfg**	4808.28478917958	0.0867233866693165	0.088007884558692	0.985404740770483	0.324425301917149	0.63204370453864
**Dele1**	1292.77988758134	-0.104547588278485	0.106041743360062	-0.98590974616003	0.32417740384858	0.63204370453864
**Eif2b3**	2732.47019197843	0.0869339989298368	0.0882184189925264	0.985440454755844	0.324407766509026	0.63204370453864
**Eif4g3**	5414.54693906101	-0.128431072178894	0.130321996839697	-0.985490364584198	0.324383262038903	0.63204370453864
**Snrpert**	193.641605097864	-0.343441813063338	0.348384655078306	-0.985812113297993	0.32422532044375	0.63204370453864
**Gm43677**	35.2805643088714	-0.628963628090457	0.638104618544594	-0.985674777789595	0.324292730248305	0.63204370453864
**Strip1**	9907.4222295719	0.0719333564734785	0.0730360610737402	0.984901915792688	0.324672252256169	0.632446616244338
**Kif3c**	1338.7723075923	-0.150603901722077	0.152955246596768	-0.984627236221001	0.324807206155896	0.632631291396595
**Gtpbp10**	605.495769939654	0.14900461291145	0.151377806435771	0.984322711629941	0.324956865998475	0.632844560318623
**Orc3**	5534.93920697692	0.0589844573151723	0.0599339072757297	0.98415838373111	0.325037644247521	0.632923648097898
**Gpd1l**	6853.18644405973	0.0790225373914738	0.0803242691922685	0.983794041154874	0.325216789800831	0.63318087797053
**Gm9523**	58.1013391658583	0.297141211605251	0.302056798134663	0.983726284063896	0.325250112730435	0.63318087797053
**Nt5m**	1078.57845464983	0.126211801890517	0.128318976714458	0.983578618861416	0.325322742155275	0.633244033124232
**P2ry12**	78.8704784498956	0.427859955107914	0.435260101898599	0.982998334195104	0.32560825851229	0.633448611584881
**Eif4h**	25685.7810253334	0.0510872646985554	0.0519563929282358	0.983271967496264	0.325473602943227	0.633448611584881
**She**	242.919505649742	0.239238905868418	0.243387073119155	0.982956501355738	0.325628847739896	0.633448611584881
**Eif4b**	13240.6565953044	-0.0942393385047719	0.0958498134281065	-0.983197933665854	0.325510031584732	0.633448611584881
**Pde11a**	255.337430835962	-0.218176291426368	0.221945778747698	-0.983016179255137	0.32559947581354	0.633448611584881
**Arhgap27os2**	26.6346697316413	0.513911070454233	0.522990340633556	0.982639698147535	0.325784798977912	0.633673753548321
**Trav10n**	200.299591140901	-0.177994015937203	0.181154596955904	-0.982553128257243	0.325827422781192	0.633678437517063
**Hnrnpab**	26629.7134920188	0.096891521285364	0.0986209538429381	0.98246384272121	0.32587138746582	0.633685727792044
**Gm8615**	93.9965668873364	0.213848341283508	0.217711122808071	0.982257307413877	0.325973101356153	0.633728322442858
**Gm14325**	357.591510870807	-0.199661938552088	0.203268732360082	-0.982256032366038	0.325973729351994	0.633728322442858
**Stag1**	3947.25955059415	-0.0945256942665402	0.0962421893061276	-0.982164837978409	0.326018647115214	0.633737456525342
**Aar2**	4290.8510914806	0.141103418597554	0.143679359725889	0.982071599335846	0.326064575935142	0.633748553473545
**Rap1b**	59246.0716413593	0.134141576230054	0.136616023645615	0.981887575486896	0.326155237368471	0.633846580564257
**Hadhb**	14000.6968175256	0.0625917747137877	0.0637561125266367	0.981737628492286	0.326229122566973	0.633906524159721
**Rps6-ps1**	125.252307684487	0.266604960640424	0.27158537621507	0.981661694587333	0.326266542548413	0.633906524159721
**Heatr9**	74.6645449610573	0.467368273379906	0.476281951091426	0.981284871931229	0.326452280796238	0.634111019293185
**Abitram**	2319.35014389849	0.0690178758222252	0.0703298415285695	0.981345533022261	0.326422375925377	0.634111019293185
**Traj18**	76.9250856129119	-0.430782947435577	0.439073378676526	-0.981118346855967	0.326534383935142	0.634192319446079
**Klhl4**	89.5985331046247	0.76855874860751	0.783523415165451	0.980900804917513	0.326641660520124	0.634208445983873
**Pheta2**	305.570477592745	0.281326327842778	0.286802157356137	0.980907293153448	0.326638460641268	0.634208445983873
**Gm6159**	49.4979573548929	0.318542519802783	0.324759503116264	0.980856654681925	0.326663435146609	0.634208445983873
**Tmem161a**	6249.2608411549	-0.0693608287725851	0.07074243201146	-0.980469949935407	0.326854195865851	0.634500623907628

**Fis1**	6887.7656847249	0.0745069021414594	0.0760199749329575	0.980096378710563	0.327038546541203	0.634623938158834
**Ate1**	4624.25317685559	-0.0664608183482497	0.0678054658547394	-0.98016903962624	0.327002684398708	0.634623938158834
**Hist1h2ao**	351.572779346539	0.238925430688676	0.243765300692682	0.980145369376802	0.327014366683058	0.634623938158834
**Nicn1**	386.813953435325	0.199611205929899	0.203756056764039	0.979657778522187	0.327255073999576	0.634965915652175
**Smyd4**	2076.5805901735	-0.100476569115005	0.102585192691656	-0.979445146796291	0.327360079191535	0.635091451344622
**Zdhhc15**	2092.15378869253	-0.0713362722289273	0.0728598818470676	-0.979088497270167	0.327536254659033	0.635355012269159
**Nek1**	1591.07314066254	-0.133721162903566	0.136607551501359	-0.978870944057847	0.327643750426617	0.635485299787322
**Rnf32**	497.686605070565	-0.167760098818135	0.171403772807508	-0.978742159932118	0.327707395064193	0.635493065710375
**Gm7931**	23.4498389812264	-0.438652369858743	0.448199179212136	-0.97869962776332	0.327728416066669	0.635493065710375
**Eif4enif1**	2995.2573670825	0.092884047619486	0.0949138758062459	0.97861399959155	0.327770739393946	0.635496928833568
**Pafah1b3**	737.88887352587	0.140520188101199	0.143631039634006	0.978341370077568	0.32790551525892	0.635523641320394
**Ankfy1**	8799.93502710347	0.0869695207783688	0.0888884261251274	0.978412202460898	0.327870495432607	0.635523641320394
**Apbb1**	607.076957244265	-0.225110933340311	0.230087089203244	-0.978372728864689	0.327890011044144	0.635523641320394
**Tvp23b**	3124.80508202607	0.101905642311918	0.104182280551382	0.978147548437071	0.328001353894089	0.635631205670787
**Nusap1**	199.231627150857	0.311602953783948	0.318671424516054	0.977818937663331	0.328163882988322	0.635789782890322
**Zfp217**	1307.39800777752	-0.163620896483454	0.16732574833559	-0.977858447435685	0.328144338908157	0.635789782890322
**Pet100**	373.694336901146	0.136076171979412	0.139196577971209	0.977582739193185	0.328280737726491	0.635937986766206
**Ormdl2**	1906.55014524896	0.126980173649349	0.1299143030266	0.977414885744715	0.328363796456769	0.636011481581368
**Otud5**	12416.7517421936	0.178465740219213	0.182602988139383	0.977342934185657	0.328399404337502	0.636011481581368
**Hist1h2ap**	505.752347085011	0.238598177881967	0.244268961563927	0.976784673559616	0.328675765268787	0.636468481423163
**Shq1**	1207.41589020579	0.0999155253008148	0.102304088119915	0.976652322864161	0.328741306165053	0.636517173905473
**Ssr3**	5181.99250316571	0.0768411611656819	0.0787176825025154	0.976161374710523	0.328984500780283	0.63652162397755
**Josd2**	3845.95939331437	0.0772693623750963	0.0791498670478611	0.976241215015211	0.328944943387012	0.63652162397755
**Oxld1**	357.290992672568	-0.203143911493087	0.208084301677575	-0.976257746765814	0.32893675300997	0.63652162397755
**Cox6a1**	10856.7110765155	0.0574574673389169	0.058855809702382	0.976241217807789	0.328944942003465	0.63652162397755
**Vamp8**	5335.55757541683	0.0695088718603506	0.0711928168843231	0.976346700444391	0.328892684679248	0.63652162397755
**Gm15903**	26.3807854793085	-0.586369019727328	0.600690449035007	-0.976158386851853	0.328985981193956	0.63652162397755
**Dgcr2**	12494.219806637	-0.0732330121144187	0.0750518141852045	-0.975766047889295	0.329180413427509	0.636762414918408
**Vav2**	166.99585394728	0.355635491742313	0.36447616286469	0.975744171984003	0.329191256706565	0.636762414918408
**Arf4**	13065.659221194	0.0662933896307354	0.0679538082280551	0.975565481308313	0.329279837381605	0.636855578448748
**Usp42**	2559.39011843321	-0.148149549340873	0.151876300737182	-0.975461929358168	0.329331177299348	0.636876701117553
**Gstm2-ps1**	26.575023174033	-0.454764445393711	0.466294185290023	-0.975273678591681	0.329424523242361	0.636979041935192
**Tbc1d9**	61.9422633733959	0.569135314241028	0.583630783999716	0.975163287893507	0.329479269498039	0.63700672999762
**Gm16158**	35.3313308673288	-0.419809160650337	0.430544623712708	-0.97506538818254	0.329527826007267	0.637022445638144
**Ipo13**	4077.1259912791	0.078816761823335	0.080857180695835	0.974765149428403	0.329676767976486	0.63723219246878
**Nsmf**	906.249693363458	-0.11665174394357	0.119695934864201	-0.974567299014081	0.329774941129286	0.637270668471066
**Apol7c**	799.173465864562	0.313205732103566	0.321407893130199	0.974480523963641	0.329818004784004	0.637270668471066
**Trav19**	252.570396711851	0.1815049870954	0.186254408432077	0.974500354774641	0.329808163072015	0.637270668471066
**Abcg3**	2628.05371323563	-0.111009832564931	0.113963967387047	-0.974078343446194	0.330017641536116	0.637578221883837
**Cbx7**	11632.776651746	-0.113358161361477	0.116399093412199	-0.973874950727036	0.330118632604392	0.637695144531985
**Parp4**	2376.02072656903	-0.2300765074699	0.236317350376902	-0.973591262355269	0.330259526475387	0.637889109913296
**Gm5500**	73.5169013061927	0.305200335634851	0.313615450181841	0.9731674107825	0.330470104934332	0.638217605521542
**Art4**	2668.24290845624	-0.108357171905401	0.111388034855912	-0.972790049178693	0.330657659295867	0.638423322818892
**Nsa2-ps1**	117.952802168177	-0.221643199917229	0.227834973053554	-0.972823429812643	0.330641065845171	0.638423322818892
**Wdr12**	5206.87462700901	-0.0866642746950566	0.0891038817470326	-0.97262064228692	0.330741879451217	0.638507693520317
**Gm45552**	16025.1737826962	-0.0936143843449175	0.0962708740594216	-0.972406091245578	0.330848562810484	0.638635404165724
**Rapgef3**	17.0862655402379	2.93147152904221	3.01554163612257	0.972121059091573	0.330990326627493	0.638830790870825
**Fhl2**	255.010267071179	0.235320001535581	0.242162999431328	0.971742182282939	0.331178825913372	0.639064014894012
**Scimp**	78.8958362765457	0.622855770739768	0.64098596471761	0.971715146702425	0.33119227934407	0.639064014894012
**Chdh**	57.1561781125759	0.792413916283139	0.81600948893235	0.9710841932977	0.331506354404486	0.639122186021227
**Gm16286**	20065.6615563849	-0.098296509431924	0.101211163079917	-0.971202251221128	0.331447573065004	0.639122186021227
**Cog4**	4085.55187426911	0.0857643626880985	0.0882768042785433	0.971539051385264	0.331279916447263	0.639122186021227
**Ubap2l**	29577.1003168965	0.100848162342672	0.10381864156713	0.971387804929639	0.331355199023287	0.639122186021227
**Hacd1**	25.5007873020893	-0.563098663433478	0.579734971305931	-0.971303597857866	0.331397117693482	0.639122186021227
**Gm2606**	3458.71132256204	0.0698668932479268	0.0719361246374146	0.971235156190058	0.331431190798488	0.639122186021227
**Gm28071**	23.2066811904002	0.527450675460636	0.543147217338456	0.971100759836832	0.33149810547614	0.639122186021227

**4933421O10Rik**	199.715713886479	-0.239575986787581	0.246746787238926	-0.970938626875002	0.331578841573268	0.639183729712501
**Ugt1a7c**	42.7184895102589	0.696174831478576	0.717138910568297	0.970767059518345	0.331664289484539	0.639270239682922
**B230303O12Rik**	16.973955322266	-0.749903155763703	0.77256941996045	-0.970661194177336	0.331717022071243	0.639293678666411
**Vps39**	7760.35466714533	0.0916037240601365	0.0943916367870408	0.97046440954092	0.331815056868105	0.639404408838294
**Pla2g15**	947.58348629982	0.143484850155566	0.147909159373503	0.970087658961235	0.332002799910443	0.63968795771446
**Rpp14**	3522.72175392832	0.102964974602897	0.106180230110607	0.969718887363863	0.332186633315918	0.639767845205324
**Kcnip2**	133.72309873819	-0.369630958191946	0.381185920001329	-0.969686808449422	0.332202627829848	0.639767845205324
**Helz2**	3798.12850642838	-0.110337585068476	0.113790610609318	-0.969654565325277	0.332218704719936	0.639767845205324
**Topbp1**	3563.79788075199	0.167936251721913	0.17318332387693	0.969702208979742	0.332194949079088	0.639767845205324
**Slc35e4**	96.4492278836237	0.285919577803988	0.294884882405004	0.969597272915799	0.332247272787661	0.639767845205324
**Plvap**	17.903427197636	1.18319160484085	1.22045882638881	0.969464581072157	0.332313443834802	0.639817074108624
**Rnf25**	2270.23400898695	0.0891765998550602	0.0920647793069771	0.968628834244129	0.332730411473899	0.640309136368028
**Trim17**	22.6719526765876	-0.802014846150673	0.827985859896286	-0.968633505711238	0.332728079864743	0.640309136368028
**Champ1**	3079.20686506965	0.0834876813441	0.0861925222182695	0.968618613256033	0.332735512980718	0.640309136368028
**Chadl**	82.385837200058	-0.297373834973388	0.307031473837032	-0.968545117727019	0.332772197608541	0.640309136368028
**Atxn1l**	290.244530484006	-0.278638922998288	0.287631453731886	-0.968735927114634	0.332676962241478	0.640309136368028
**1110035H17Rik**	25.6172418029446	0.52518686896975	0.54237386858062	0.968311527146675	0.332888809571349	0.640455307987271
**Chrm3**	36.6014498211767	0.592025792441868	0.611510879511326	0.968136156326395	0.332976374682889	0.640545566582625
**Gpc2**	19.3635495451793	-1.02713864510781	1.06110139399416	-0.967992927840283	0.333047901706335	0.640604954490566
**Setdb1**	3651.90113639011	-0.119158490367458	0.123125659618691	-0.96777950864573	0.333154499739685	0.640633661421873
**Mtor**	1847.69057632017	-0.170321431395448	0.176003009610518	-0.967718857605655	0.33318479757265	0.640633661421873
**Dhx33**	2698.12273867588	0.0994876014007676	0.102806293642482	0.967718977855034	0.333184737501095	0.640633661421873
**Rpp38**	519.013948694403	0.161301517016046	0.166716399274482	0.967520398221165	0.3332839490983	0.640746118343328
**Styx**	3161.92217765821	-0.116702999824877	0.120698511611905	-0.966896760087021	0.333595646457796	0.641267122268574
**Nup35**	2404.45963985001	0.164318528403225	0.170011556699932	0.966513874661151	0.333787107596485	0.641556898560358
**C230062I16Rik**	532.822034016007	-0.120915297430989	0.125121819778921	-0.966380585293882	0.333853775323416	0.641606773724975
**Gm11007**	201.824872294613	0.297967674163064	0.308373138069849	0.966256905604963	0.333915644231327	0.641647415384515
**N4bp3**	1112.00316149598	0.14581025464301	0.150944734057212	0.965984375365778	0.334051999490354	0.64169155612287
**Psmb3**	15117.7434139129	0.10624972860759	0.109993145952647	0.965966812635139	0.334060787895913	0.64169155612287
**Ndufs5-ps**	47.5354502660917	0.372511087694897	0.385619064334503	0.966007965238369	0.334040195332389	0.64169155612287
**Pnkp**	6498.88636489806	0.0566999914981428	0.0587169814263992	0.965648950622833	0.334219872089264	0.641918884021274
**Tedc1**	960.294167650185	0.0808179123589253	0.0837529354799278	0.964956176112706	0.334566762423593	0.642502497740235
**Fam57a**	337.509215724721	-0.239180204687126	0.247886147480697	-0.964879268639854	0.334605286314423	0.642502497740235
**Trappc6a**	3890.72725849216	-0.0915148181543236	0.0948751667804318	-0.964581367915958	0.334754535402663	0.642710760855339
**Cep95**	1600.1164025788	-0.111928929308615	0.116088878817365	-0.964165822332605	0.334962796537219	0.642827298014841
**Agfg2**	2042.6751921653	0.122218612923435	0.126765086100959	0.964134657914386	0.334978418734522	0.642827298014841
**Kbtbd8**	476.916729883382	-0.267101119913438	0.277025476510194	-0.964175292750047	0.334958049269659	0.642827298014841
**Arl4a**	2540.99459441523	0.136887238787601	0.14196284155538	0.964246962711023	0.334922124435938	0.642827298014841
**Mrps25**	2565.60144987651	-0.138137843900445	0.143298251111708	-0.963988344789781	0.335051769311622	0.642889762426112
**Gcn1**	8994.74906380538	-0.146795914148063	0.152327091638989	-0.963688813123045	0.335201964602714	0.643099641573721
**P2ry1**	17.8355333217882	0.843237127573673	0.875301342662579	0.963367798578522	0.335362980271372	0.64326770398377
**Zc3h12a**	146.428229475626	-0.246346564106862	0.255718283843931	-0.963351389676978	0.335371212047133	0.64326770398377
**Idh3a**	6416.87493748377	0.0927338485368696	0.0962991537901036	0.962976774842643	0.335559178689327	0.643518198198206
**Arl5c**	4656.45132635952	-0.0972288778105434	0.10097208099066	-0.962928334809079	0.335583488899832	0.643518198198206
**Morc2a**	2634.14063548449	-0.119196030728064	0.123802980310357	-0.962788055903467	0.335653895944108	0.64357488907171
**Gm29488**	31.4072803835429	-0.689372538538401	0.716106588110667	-0.962667499481049	0.335714411723022	0.64361260317796
**Wipi1**	22.2232589727559	0.65975175539122	0.685406450911535	0.962570099119731	0.335763308969771	0.643628036427821
**Rps24**	9409.13352713513	-0.0713914595633699	0.074192469354378	-0.962246710274204	0.335925690571849	0.643735533083447
**C330011M18Rik**	89.6400964373874	-0.772120398141135	0.802425015803727	-0.962233707741229	0.335932220524682	0.643735533083447
**Ccdc134**	1951.80942197468	0.11660856291086	0.121187721333005	0.962214336800981	0.33594194888273	0.643735533083447
**Rnft1**	3186.83823497406	-0.122900807037892	0.127877259212015	-0.961084150498781	0.336509858213337	0.644455944997364
**Mkks**	1373.69098741752	0.162387522707478	0.168928988842085	0.96127682892412	0.336412995244274	0.644455944997364
**Gm7805**	264.090112816106	-0.206689934194353	0.215080668765202	-0.960987965031817	0.336558219119836	0.644455944997364
**Gm6225**	142.503574688544	0.303988638429003	0.316274824737188	0.961153448370752	0.336475018839745	0.644455944997364
**A930001M01Rik**	294.518801666364	0.216080757731571	0.224855085852827	0.960977853411778	0.336563303381564	0.644455944997364
**Maskbp3**	703.48725488091	-0.125243121789236	0.130298542061517	-0.961201252199009	0.33645098690829	0.644455944997364

**Magt1**	5622.41564128655	0.0744654050857956	0.0775094705049656	0.960726535746687	0.336689685235927	0.644541291491009
**Rps3a3**	155.617885067436	-0.251546738930405	0.261816935674057	-0.960773367401881	0.336666132363606	0.644541291491009
**Eif3k**	12400.0362178111	0.0450870630037632	0.0469348825786704	0.960630143863468	0.336738166587004	0.644555793356587
**Fdx1**	699.495467960868	0.143030385654193	0.148913390348193	0.96049378312962	0.336806758375984	0.644608780727606
**Eif3d**	9872.78886338006	0.0661524536772387	0.0688799726228713	0.96040185787288	0.336853003427292	0.644618991377682
**Zxdc**	3995.60507493935	0.407036862792828	0.423914220035008	0.960186857518518	0.336961180094358	0.644669416554977
**Tpbgl**	508.57956417317	0.194715578561892	0.202785447845398	0.960204889605005	0.336952106455841	0.644669416554977
**Zcchc7**	4827.75325604438	-0.069464192937244	0.072354419296443	-0.960054598083948	0.33702773705609	0.644716836906222
**Mcc**	44.6022577397889	-0.51504326239141	0.536517371565046	-0.959974997433923	0.337067798593225	0.644716836906222
**Wdr82**	4627.84803790072	0.138394390959014	0.14420522981436	0.959704381992068	0.337204017242703	0.644867771728838
**Dctn4**	2730.46957613303	-0.0934634551207587	0.0974174553833611	-0.959411788708272	0.337351338602577	0.644867771728838
**Gm5533**	41.614718569216	-0.546308774333945	0.56938181791525	-0.959477027795187	0.337318486996238	0.644867771728838
**Atxn7l3b**	5686.30799033604	-0.0769969323473832	0.0802352581943802	-0.959639615801425	0.337236623597196	0.644867771728838
**Gm45351**	20.3230649696345	0.531940082975091	0.554368805934354	0.959541874075219	0.33728583522836	0.644867771728838
**Gm44891**	77.6148983882152	-0.295018188135186	0.307577988554788	-0.959165477092114	0.337475389106602	0.645026650430946
**Mphosph6**	913.556574891945	0.123513114150933	0.128807652595762	0.958895777245125	0.337611252298565	0.645129820818396
**Snord35a**	34.9494880109155	0.374411452784949	0.390446390190466	0.958931782164267	0.337593112535179	0.645129820818396
**Letmd1**	4960.41485307673	0.084972792669026	0.0886285153848794	0.958752296594634	0.337683546004225	0.645189721642848
**Gm42572**	49.6983998508467	-0.378564984029175	0.394895097921558	-0.958646957183482	0.337736628317216	0.645212906449366
**Slc3a2**	18179.1667084466	0.0647815697427136	0.0675897077493484	0.958453171346019	0.33783429428388	0.645243027276496
**Stk35**	635.656800842452	-0.130144753827599	0.135775062508005	-0.95853208552142	0.337794520203136	0.645243027276496
**Pitrm1**	5763.76050662134	0.0694477549435581	0.072505846258604	0.95782283122193	0.338152104367945	0.645771749912218
**Traj16**	44.033489868737	-0.389991958677865	0.407234177008439	-0.957660188402566	0.338234138118247	0.645850135037471
**Calr**	18944.2496694811	0.0673765375261282	0.07043047196295	0.95663901786107	0.338749487870283	0.646087139682092
**Tns4**	24.343775960327	-0.856598948267222	0.89529386013365	-0.956779652369501	0.338678484548791	0.646087139682092
**Eng**	2348.35429956877	-0.173586041093602	0.181456474018855	-0.956626331643394	0.338755893336946	0.646087139682092
**Gucy1a1**	205.608338734936	0.234458969032188	0.245032425917877	0.956848744217908	0.338643605068533	0.646087139682092
**Naa30**	1430.22403131448	0.259532440469587	0.271325899728021	0.95653397161769	0.338802529679339	0.646087139682092
**Cage1**	78.6943066636901	0.317120084085641	0.33133657581457	0.957093503202963	0.33852006250098	0.646087139682092
**Tmem26**	38.4123219431234	-0.544126177337657	0.56895646660639	-0.956358191309721	0.33889129970632	0.646087139682092
**Zfp677**	1603.10540553703	0.152668448368538	0.15958481117431	0.956660268888515	0.338738758073451	0.646087139682092
**Gm13127**	194.140859228675	-0.181922665677313	0.190218028478243	-0.956390238783917	0.338875114442302	0.646087139682092
**Safb**	7706.81643205351	-0.0811631392136656	0.0847948958415429	-0.957170103320087	0.338481404390057	0.646087139682092
**Gm28874**	61.9796711209478	-0.344184761392431	0.359854359629007	-0.956455722107324	0.338842044271418	0.646087139682092
**Gm38115**	248.212892159308	-0.2546048153034	0.26615862689302	-0.956590504976327	0.33877398319261	0.646087139682092
**Gm42895**	74.4766096830063	-0.463336060936496	0.484404062832882	-0.956507379865526	0.338815957703045	0.646087139682092
**Ift122**	282.789514382109	0.260409965157571	0.272371212360375	0.956084759842469	0.339029413771857	0.646115925593027
**Dpep2**	23.6138969985571	0.885674726916878	0.926348143687432	0.956092731390761	0.339025386720071	0.646115925593027
**Gm15708**	415.182332645456	-0.202670363738415	0.211975184429334	-0.956104198159002	0.339019594013467	0.646115925593027
**Zdhhc12**	1599.14312921805	0.111090192535381	0.116244209279522	0.955662163508304	0.339242944135283	0.646244001362376
**Rpain**	3185.44018618388	0.113261590494801	0.118494623245979	0.955837382255613	0.339154398771038	0.646244001362376
**Serpine2**	72.4502891127589	0.572498537107048	0.599014541885697	0.955733954813223	0.339206663192018	0.646244001362376
**Tdo2**	31.2530091534074	-0.471512913116586	0.493406809154415	-0.955627089793612	0.339260670131978	0.646244001362376
**Manea**	867.929074549859	-0.130339946226877	0.136417258339706	-0.95545056258428	0.339349894701799	0.646335826280219
**Nap1l4**	26180.6703704603	0.0566870798123601	0.0593635960031821	0.954913172869808	0.339621607604112	0.646775159658931
**Rpl7-ps7**	47.9783494798693	-0.344701147974777	0.361026780612832	-0.954779995516279	0.339688965785604	0.646825260892298
**Gzma**	5942.56256223101	1.05924894071497	1.11004099294501	0.954243084216847	0.339960610607005	0.647264299339606
**Adam10**	6905.16886652444	0.101353213316013	0.106224977861892	0.954137297611745	0.340014148682521	0.647288019909446
**Slc36a4**	693.105433477996	-0.163507907779321	0.171396633567137	-0.953973858041234	0.340096875262034	0.64736729445565
**Ndufs1**	10490.0158782714	0.0692047213603019	0.0725630036398373	0.953719083953525	0.340225857450888	0.64748148769881
**Ptger2**	503.115524403101	-0.182421451441888	0.191279002924312	-0.953693027739546	0.340239050463041	0.64748148769881
**Mast2**	303.439824656073	0.321217495638164	0.337284105127805	0.952364759425727	0.340912025312244	0.647572437041899
**Trmt10a**	500.535072941077	0.146842357358556	0.15423371218842	0.952076918042184	0.341057974124756	0.647572437041899
**Kdsr**	2244.47129740999	-0.0753881605698525	0.0791210535417294	-0.952820484500904	0.340681033557249	0.647572437041899
**Acot2**	1293.39946457664	-0.113907685623335	0.119496202742907	-0.953232680275237	0.340472191714916	0.647572437041899
**Sac3d1**	1903.96737134698	0.151118986620734	0.158740766131022	0.951985997698928	0.341104083231479	0.647572437041899

**Cenpx**	2289.39686939409	0.0806601756303621	0.0847266945576939	0.9520042774174	0.341094812581982	0.647572437041899
**Bcar3**	70.0584866543403	0.38457530579464	0.404009927235274	0.951895683421372	0.341149888930939	0.647572437041899
**Ss18l2**	565.876511817232	-0.161519733775035	0.169673918533016	-0.951942026043363	0.341126384334543	0.647572437041899
**1110059G10Rik**	670.585144156941	0.122778782869448	0.128800575758538	0.953247158612252	0.340464857656691	0.647572437041899
**Pmepa1**	263.304391294134	0.170293583984565	0.178826098580064	0.952285965732914	0.340951973359944	0.647572437041899
**Utp15**	4276.62590751587	0.0714807043912989	0.075036462540512	0.952612929383587	0.340786223849321	0.647572437041899
**Gpr155**	598.284903230343	0.203272819199478	0.21354101725726	0.951914633592798	0.341140277434674	0.647572437041899
**Epop**	967.569524660717	-0.0953887855519199	0.100066162646977	-0.953257155352715	0.340459793827794	0.647572437041899
**Prss53**	36.1994020580325	-0.822511503525903	0.86323491465994	-0.95282464779581	0.340678923784939	0.647572437041899
**Ddx10**	6050.09350583852	0.0648506280980727	0.0680924953964509	0.952390240958225	0.340899106931385	0.647572437041899
**Atpif1**	1495.43893069904	0.129357282100029	0.135803615589052	0.952531945036497	0.340827272887841	0.647572437041899
**Churc1**	4971.29529046962	0.0869359851968761	0.0912523105871413	0.952699001674667	0.340742599324413	0.647572437041899
**Gm4707**	281.823265573009	-0.230058402044897	0.241645999533874	-0.952047219853299	0.341073034748209	0.647572437041899
**Zfp433**	1003.45116666493	0.108804509001453	0.114269169731584	0.952177295564786	0.341007073564154	0.647572437041899
**Gm37407**	148.032470468117	-0.264770780448224	0.278137346117485	-0.951942571338065	0.341126107771731	0.647572437041899
**Gm48254**	22.9354306444266	-0.599533896931743	0.628970942476233	-0.953198083478074	0.340489717267708	0.647572437041899
**Gm16437**	72.8732515664933	0.330895535564911	0.34772719250664	0.951595223771844	0.341302304745806	0.64765030974017
**Ctps**	2455.68608776675	-0.102770642972386	0.108005856148172	-0.95152843223053	0.341336192378705	0.64765030974017
**Xpot**	3319.94414782203	0.0772396487477455	0.0811775106541556	0.951490728470515	0.341355322865527	0.64765030974017
**Gm22146**	264.270360622506	0.456428486844861	0.479579391430954	0.951726648392842	0.341235630894507	0.64765030974017
**Gm10420**	125.816547364459	-0.185970213454249	0.195483846120616	-0.951332896015886	0.341435412824147	0.647724271595243
**Glrx5**	4353.19660158845	0.10831818912991	0.11391127733703	0.950899609434	0.341655340103764	0.647987633141054
**Cd2**	43251.3506752904	0.0691088817575429	0.0726899248192775	0.950735358845977	0.341738733985033	0.647987633141054
**Gm2260**	322.842854598772	-0.255874018076188	0.269098279646049	-0.950857130758121	0.341676906276628	0.647987633141054
**Gm36981**	77.4532060615306	0.440884824410368	0.463727251173988	0.950741676910747	0.341735525914306	0.647987633141054
**Angptl2**	165.715931360921	0.392112519905191	0.412475945867288	0.950631240036844	0.341791604294416	0.648007699733015
**Parp9**	5950.6356791601	-0.0930558233660237	0.0978965588117606	-0.950552547459355	0.341831566934113	0.648007699733015
**Nrbp2**	34.4128934461798	0.526715891890782	0.554170747287163	0.950457768601498	0.341879702676475	0.648020988220042
**Raf1**	14346.2427753729	0.0736040583377547	0.0774621822373453	0.950193451976743	0.342013965196087	0.648119548959079
**Phf11d**	158.679235344134	-0.173455371070253	0.182547255547119	-0.95019435132226	0.342013508306518	0.648119548959079
**Rps3a1**	31417.3593499635	-0.0553904784179558	0.0583141679122441	-0.94986313619894	0.342181800114277	0.648359622943802
**Plp1**	171.271180684007	0.289691950959754	0.305144269982995	0.949360612197956	0.34243723580863	0.648747648212674
**Etfbkmt**	3115.74740043898	0.0606563566111129	0.0638959936932895	0.949298275292073	0.342468930496479	0.648747648212674
**Gm10369**	69.6312051759619	0.461982512461069	0.486835719280328	0.948949500139394	0.342646296962671	0.64891366525842
**Gm16892**	58.7939714419947	-0.42152838203766	0.444236410847839	-0.948883008561049	0.342680117320259	0.64891366525842
**Gm29170**	180.945445888551	-0.3534340455083	0.372453466320191	-0.948934772980363	0.342653787618845	0.64891366525842
**Mrps10**	2720.21905673441	0.104991723322944	0.110702957894603	0.948409376946399	0.342921087618517	0.649213934591491
**Igkv1-135**	51.5461298641333	-0.515741900708244	0.543750938233793	-0.948489215271006	0.34288046056201	0.649213934591491
**Haao**	5677.20081581982	0.113644252050642	0.119883470345018	0.94795597527816	0.34315186692492	0.649572797589616
**Nod2**	25.1977615317654	-0.703034318837554	0.741720599586527	-0.947842515401975	0.34320963298563	0.649604106541091
**Myh7b**	19.0280699812111	-0.647587031430743	0.683826625627373	-0.947004704352682	0.343636381761413	0.650333709754334
**Hspa4**	10147.5866867486	0.0668461060865552	0.0705940784774131	0.946908119325376	0.343685600241118	0.650348745406424
**Ddx42**	11563.0104823722	0.0943324983561037	0.0996487454763783	0.946650134983036	0.34381708779288	0.650519434720511
**E2f8**	74.714117255135	0.809309744084282	0.855026512539498	0.946531753361152	0.343877434412276	0.650555496942518
**Helz**	6928.77134126412	-0.150308203008903	0.15895202732227	-0.94561991778915	0.344342481460922	0.650621982332673
**Txndc11**	6343.02222731158	0.0555135698620816	0.0586815799631306	0.946013551389729	0.344141674404685	0.650621982332673
**Flnb**	2222.16414562481	-0.165652589735641	0.175152312123794	-0.945763077444054	0.344269441776309	0.650621982332673
**Mtmr14**	5128.65440873838	0.0837957189207525	0.0885994645813486	0.9457813240374	0.344260133122206	0.650621982332673
**Gm14117**	819.254485878404	0.190886454272566	0.2018604364899	0.945635794670028	0.344334380629959	0.650621982332673
**Hikeshi**	2632.40898250119	0.103023735054936	0.108953842225404	0.945572298788693	0.344366778743066	0.650621982332673
**Epc2**	1281.09916583487	-0.0889445257177648	0.0939903267185709	-0.946315741449495	0.343987566808606	0.650621982332673
**Gm13586**	52.0821777601175	-0.395984210332771	0.418550693968497	-0.946084228360102	0.344105627385351	0.650621982332673
**Gm15682**	269.301454830697	-0.118085260261842	0.12478758181082	-0.946290156025784	0.344000612876989	0.650621982332673
**Gm47644**	21.0657724896657	-0.898752684737429	0.950364137631749	-0.945692970882789	0.344305208726493	0.650621982332673
**Gm49539**	40.8337254279951	-0.475264526723194	0.502592098257311	-0.945626738603983	0.344339001274542	0.650621982332673
**Dhrs3**	102.761255451428	0.400791547048386	0.423918852979225	0.945444025977367	0.344432234545249	0.650667632145964

**Zfp113**	1369.46789021087	-0.117315095854863	0.12413980258661	-0.945024024611396	0.344646610855287	0.650887467662209
**Iglv2**	62.103001801611	0.553945160241368	0.586201942841465	0.944973258799279	0.344672528411086	0.650887467662209
**1810010H24Rik**	58.1606322198029	-0.357727741523991	0.378501087896759	-0.945116811980119	0.344599243176349	0.650887467662209
**Foxp4**	742.069358175102	-0.139208368268693	0.147339820938977	-0.944811574912583	0.344755081444805	0.650939838013821
**Fam204a**	1700.18400064517	0.126865410152828	0.134283622905993	0.944757129777777	0.344782883040131	0.650939838013821
**Traj21**	98.6307129918437	0.321679900383029	0.340539017611164	0.944619804918597	0.344853012281913	0.65099423919086
**Zfp507**	1922.75706631267	-0.14705552771001	0.155690412529275	-0.944538108166159	0.3448947376043	0.650995014426325
**Rbsn**	3079.5171311736	0.0847996888357806	0.0897980209088788	0.944338059764478	0.344996922752434	0.651013266983669
**Myb**	1474.91910513099	-0.161063517084447	0.170568166884831	-0.944276531934582	0.345028355180594	0.651013266983669
**Cenpn**	212.3760391945	0.250451215767639	0.265207523558828	0.944359392248102	0.344986025154925	0.651013266983669
**Egln3**	132.004465013545	-0.332256375996816	0.35190667225705	-0.944160489671306	0.345087642106774	0.65104717153693
**Vamp2**	4158.79078784607	-0.112108255335984	0.118751433236426	-0.944058124442033	0.345139946718868	0.651067896617887
**Gm6192**	79.6442042504416	-0.22339171843199	0.236702685839607	-0.943765034349307	0.345289732201927	0.651272481482614
**Cdc42ep1**	24.3248708603729	2.27429143037549	2.41008441026096	0.943656338629747	0.345345292347183	0.6512993143644
**Tcp11l1**	59.2766264640327	0.425279356829908	0.450825991041919	0.943333714737766	0.345510236085826	0.651383037598186
**Nudt17**	86.270127861918	-0.354507471917048	0.375805523378027	-0.943326933384227	0.345513703639376	0.651383037598186
**Kmt5a**	1151.56003984086	0.105606906012536	0.111935594602232	0.943461339423044	0.345444981063698	0.651383037598186
**Lyl1**	47.6460382089815	0.467149940396919	0.49564392961157	0.942511170797589	0.345930994647562	0.652013718021727
**Supt16**	7520.71852217974	-0.106258522969438	0.112730630689899	-0.94258785140425	0.345891756175855	0.652013718021727
**Cnep1r1**	2229.62667721933	-0.105949673319356	0.112439685801131	-0.942280054986507	0.346049276792955	0.652080657070867
**Gm14303**	60.0573839159623	-0.262436391064739	0.278488690676672	-0.94235924061071	0.346008747692016	0.652080657070867
**Acaa1a**	11277.3642139926	0.0535909197852461	0.0568791345505149	0.942189437457974	0.346095660727212	0.652090078450159
**Gm49874**	153.819831860414	-0.30466847045592	0.32347967374742	-0.941847340596156	0.34627080380773	0.652342067862075
**Mtch2**	7722.36697246772	0.0594780449648347	0.0631719413632791	0.941526311860481	0.346435211989014	0.652495772807899
**Uprt**	310.963132169043	-0.109285038560145	0.11606581248037	-0.941578197960986	0.346408636235866	0.652495772807899
**Phyhd1**	64.6551082407849	-0.337913278997613	0.358980306256185	-0.941314253480141	0.346543840530786	0.652622361090426
**E430024P14Rik**	153.472061987739	-0.202979587252003	0.215717324988993	-0.940951716615064	0.34672960306585	0.652894162943189
**Akap17b**	341.97786726789	-0.146965074526565	0.156207916584655	-0.940829874309981	0.346792048837727	0.652933721297175
**Gm49920**	30.4789014245669	-0.509744245295301	0.542028533616691	-0.940438028038907	0.346992923698069	0.653233870813677
**Gm13623**	17.9892577775531	-0.520817044218821	0.553914490518785	-0.940248094486631	0.347090317288019	0.653339162526259
**Gm21926**	310.178486176213	-0.200889617893389	0.213725546810531	-0.939941999874628	0.347247312222716	0.653556605195095
**Atraid**	5034.18940063895	0.0914485192015789	0.0973260267551169	0.939610115099772	0.347417585893821	0.653588089335661
**Dyrk1a**	2506.04073401532	-0.121423684584847	0.12922411285992	-0.939636433925234	0.347404081066802	0.653588089335661
**Chuk**	7939.62841413406	0.0680043057822447	0.0723861955478273	0.939465118557207	0.347491993121125	0.653588089335661
**Hp1bp3**	16952.4782516482	-0.0812566560868763	0.0865036627271936	-0.939343532113029	0.347554394906613	0.653588089335661
**Prc1**	338.038268004993	0.301529244991663	0.320978561277192	0.939406182742737	0.347522239846842	0.653588089335661
**Dynlrb1**	5892.01589801643	0.0793696500360262	0.0844713627978484	0.939604232809274	0.34742060428526	0.653588089335661
**Nup160**	5232.52290923949	0.10062837416157	0.107120011896768	0.939398459538496	0.347526203632813	0.653588089335661
**Gbp2**	14117.2344959394	0.127947455278299	0.136287083524331	0.938808373982535	0.347829138725936	0.653926894994328
**AW209491**	1930.24977852932	-0.186804734155522	0.198976493483098	-0.938828154449259	0.347818981212188	0.653926894994328
**9030407P20Rik**	100.87602826315	-0.341076088596757	0.363330013753924	-0.938750105098008	0.347859061613407	0.653926894994328
**Tomt**	50.3810473913435	-0.411189576412823	0.438096087956219	-0.938583081924062	0.347944842286798	0.65401012524312
**Bcl9l**	5338.76414035619	-0.124683523477289	0.132854733188907	-0.938495155456758	0.347990005445206	0.65401699854486
**Rsph3b**	1017.83871506225	-0.145693476637449	0.155275356475539	-0.938291045948435	0.348094860031382	0.654136041683302
**Sinhcaf**	3080.58697424087	0.0692096383908454	0.0737685810855577	0.938199398339725	0.348141947527063	0.654146514093003
**Stap1**	2370.6452525795	-0.113293834458143	0.120767377169725	-0.938116212451326	0.348184690992834	0.654148822698711
**Ccl9**	52.1679048904382	0.672885594256596	0.717646853480604	0.937627735693518	0.348435752665791	0.654524589814825
**Atg2a**	5942.02413044074	-0.143142247880527	0.152674407141826	-0.937565441125672	0.348467778381454	0.654524589814825
**Acad11**	1328.5965388917	-0.132293732910379	0.141116003613206	-0.937482138971218	0.348510607046886	0.654527012543241
**Arg1**	19.267219273454	-0.499528664731065	0.53291681706517	-0.937348285389121	0.34857943304402	0.654555589115252
**Gm13370**	2416.90555465586	-0.0654904788054989	0.0698720894338764	-0.937290974638391	0.348608904223849	0.654555589115252
**Mdh1**	22560.0344841963	0.0669053714011924	0.0713911194677875	0.937166581781659	0.348672876801211	0.65459770281862
**Anapc7**	3784.81712504959	0.0798387851838617	0.0852152306922015	0.936907458154288	0.348806162472899	0.654769919238201
**Gm16214**	37.8717069929055	0.414754651234916	0.442758505090538	0.936751403906074	0.348886447861766	0.654842615718623
**Tbc1d19**	395.712774414965	-0.160672671878733	0.171579863878376	-0.93643081563828	0.349051418059592	0.654961176720598
**Zdhhc1**	586.611106476853	-0.191907252125194	0.20494452559098	-0.936386329772936	0.349074313771631	0.654961176720598

**5830468F06Rik**	2458.19329274553	0.117512815043164	0.125495110651708	0.936393572888289	0.34907058586449	0.654961176720598
**Ube2i**	21781.2828883787	-0.0489369320487149	0.0522668656982344	-0.936289777375496	0.349124010048175	0.65497642889976
**Skil**	2204.23583290898	-0.135302527047364	0.144538741138113	-0.93609869562982	0.349222374565944	0.655004994171596
**Gm42547**	83.8463316217899	-0.403348482108655	0.430866354719578	-0.93613362401241	0.349204392918192	0.655004994171596
**Tbccd1**	2759.48399142785	0.0725339398936726	0.0775072555428985	0.93583419236831	0.349358563839691	0.655155367254662
**Apobec3**	31581.9083239392	-0.0996133927595739	0.106457282283458	-0.93571234041405	0.349421315162752	0.655155367254662
**4931440P22Rik**	71.5231701275636	0.358273591093486	0.382893345402206	0.935700751647019	0.349427283518956	0.655155367254662
**Scnm1**	2829.95400764413	0.0945736917311144	0.101090736033037	0.9355327247811	0.349513826673812	0.65523966292674
**Gm16001**	192.142049516251	-0.16880182275334	0.180450291590307	-0.935447769386742	0.349557588592051	0.655243745353908
**Plscr3**	1863.30357944467	-0.0838161561224282	0.0896257324620012	-0.935179594297473	0.349695752800024	0.655346809808513
**Jpt1**	30095.9284518362	-0.0610506196797148	0.065277125928326	-0.935252874747398	0.349657995161327	0.655346809808513
**Ubxn2a**	2359.08457966278	0.141551025788775	0.151381580907365	0.935061088279915	0.34975681831851	0.655370878663131
**Las1l**	8609.22986092052	-0.0508836228038005	0.0544213801424865	-0.934993244761096	0.349791780767718	0.655370878663131
**B230307C23Rik**	1288.58111039291	-0.114717650617343	0.122728337868628	-0.934728299996539	0.349928338528871	0.655548784948213
**AW046200**	20.9297940502711	-0.563999639150615	0.603585514980394	-0.934415464176496	0.350089623831341	0.655772967511941
**Epor**	32.4001013353374	-0.35179805869955	0.376532010667156	-0.934311157439759	0.350143410583742	0.655795759012008
**Rac2**	157587.938553185	0.0513302316816082	0.0549469619042915	0.934177794415949	0.350212188120367	0.655846618518258
**Slc25a28**	1010.29824936743	0.245098423934984	0.262482962952084	0.93376888609615	0.350423122380221	0.656163652922773
**Zbtb11**	5555.46829782119	-0.0981076271284781	0.105086138339216	-0.933592466894052	0.35051415262736	0.656178151710742
**Gm37065**	431.692287670133	-0.165671143673796	0.177447986815666	-0.93363214002476	0.350493680448596	0.656178151710742
**Mrps18c**	1368.69951610572	0.136075306077902	0.145774069301524	0.933467157292829	0.350578820025197	0.656221248174986
**Nip7**	3615.77311440369	0.106727886440255	0.114397948582048	0.93295279996833	0.350844339030611	0.65632841625375
**Haus6**	2186.73350761629	-0.0689090206103468	0.0738572080682472	-0.93300332374698	0.350818252249761	0.65632841625375
**Agps**	3166.41783210061	0.133632852491488	0.143216270506981	0.933084292856054	0.350776448293364	0.65632841625375
**Riok2**	2570.86230196569	0.104843380613742	0.112367827222152	0.933037357805866	0.350800680246277	0.65632841625375
**Gm19522**	55.6992544127716	-0.412672365528027	0.4421875802819	-0.933251823275867	0.350689963181624	0.65632841625375
**Blm**	383.40445484437	-0.195903581348112	0.210132253983048	-0.93228706033828	0.351188193091626	0.656737759143693
**Hnrnpul1**	3856.98011526093	-0.116945924963293	0.125433088801765	-0.932337121571767	0.351162329075712	0.656737759143693
**2310074N15Rik**	19.2823066491888	0.519170125250118	0.556824085445716	0.932377278246761	0.351141583098652	0.656737759143693
**Kif15**	176.274735145173	0.324974780454193	0.34882506961256	0.931626791661341	0.351529432267676	0.65729788349849
**Psd3**	20.4596255978739	-1.10509661382295	1.18697490712361	-0.931019356172345	0.35184355170585	0.657676822085965
**Ogfod1**	1422.17773992049	0.0841575600899088	0.0903955042083859	0.930992761497331	0.351857308509531	0.657676822085965
**Gm42957**	19.9259157258383	-2.75629859458479	2.96039240428226	-0.931058528118688	0.351823289594067	0.657676822085965
**Pnp**	2706.63984442514	0.139141678785194	0.149470968400119	0.930894342055278	0.35190822154882	0.657693968324607
**Tmem134**	6871.56290301647	-0.0699498301299098	0.075155618924919	-0.930733205720655	0.351991588529173	0.657771757643996
**Adal**	1995.15245998469	-0.103418137174417	0.111130660636546	-0.930599499562479	0.352060773472709	0.657823029480549
**Pfkfb1**	213.906510928788	-0.219989386626167	0.236436986826636	-0.930435586998371	0.352145600171543	0.657874379936084
**Aen**	5598.56490839438	0.0605180001158071	0.0650461874508566	0.930385046187824	0.352171758250991	0.657874379936084
**Map2k6**	580.787022758992	0.163845983156699	0.176164967944825	0.9300713136565	0.352334162278164	0.658076866268496
**Dlg5**	136.252347232271	0.415155097219848	0.446551368431057	0.929691691861747	0.352530737334034	0.658076866268496
**Lrrc2**	18.483169087942	0.622502630288886	0.669415046403547	0.929920284333763	0.35241235969095	0.658076866268496
**Lgals3**	2568.86760611821	0.591970816109974	0.636664260247329	0.929800607748906	0.352474331612294	0.658076866268496
**Dhcr7**	3094.84156759676	0.0754895692885338	0.0811854078039242	0.929841597530103	0.352453105167454	0.658076866268496
**Glo1-ps**	32.7761820803949	0.385753586754435	0.414907697898907	0.929733501470065	0.352509084159611	0.658076866268496
**Pcsk7**	6436.40252866114	0.0782123175194474	0.0841512749280941	0.929425223637771	0.352668760865108	0.658100623513917
**Tg**	66.2669872910103	0.39839778188117	0.428625119791911	0.929478379789277	0.352641224647058	0.658100623513917
**Smagp**	49.8508020923535	0.346884764044914	0.373166139670517	0.929571917621444	0.352592773010172	0.658100623513917
**Gm9937**	35.8025542894378	0.634140973575545	0.682378174622431	0.929310164303575	0.352728369141903	0.658133915046651
**Cd37**	20940.8648659012	-0.0561585199979649	0.0604384678638515	-0.929185036994519	0.352793200524604	0.658176943010441
**Cd320**	1391.13638915774	-0.121452936945096	0.130847605057495	-0.928201451541497	0.353303081640033	0.658303880654081
**Mtrf1l**	961.19846883948	0.101415589650255	0.109233708527761	0.928427598194024	0.353185808187101	0.658303880654081
**Pstpip1**	10387.117106698	-0.0922812727256044	0.0993817491306471	-0.928553517450086	0.353120520584459	0.658303880654081
**Lsm3**	1811.13308149154	0.1040307310084	0.112079350616079	0.928188202702484	0.353309952888079	0.658303880654081
**Sf3b6**	4307.32117664701	0.0852449576122006	0.0918179968954627	0.928412299271289	0.353193741012489	0.658303880654081
**Sfn**	471.780387487308	-0.144471369646916	0.155652297694545	-0.92816727916493	0.353320804638672	0.658303880654081
**Nup98**	5276.62180662754	0.0923241557782592	0.0993953878844184	0.92885754302421	0.352962918486517	0.658303880654081

**Gm5113**	268.379723996501	-0.237618915583578	0.255864709056656	-0.928689683151896	0.353049928914469	0.658303880654081
**Arid1b**	2311.69047175122	0.188398978446989	0.202966366908531	0.928227574433026	0.353289533767095	0.658303880654081
**Gm16409**	115.024636249216	0.185995233156182	0.200238910796394	0.928866584503669	0.35295823220648	0.658303880654081
**Gm43302**	2213.37592891339	-0.375311580146297	0.404250849486227	-0.928412594861062	0.353193587741672	0.658303880654081
**Tgfb1**	349.742035884522	-0.22221179117391	0.239440614547088	-0.92804552642096	0.353383954471355	0.658343694798432
**Coa7**	1537.91877042342	-0.136857315034125	0.147485579530499	-0.927936924205015	0.35344028950462	0.658370805263541
**Ipo4**	6651.55582776543	0.0887088031625438	0.0956575434088114	0.927358157039735	0.353740608110223	0.658696615692836
**Pigc**	1719.75744880297	-0.0966591541221288	0.104219567300443	-0.927456874230549	0.353689372981691	0.658696615692836
**Suco**	3195.22430698419	-0.070280975221122	0.0757806893594363	-0.927425915694319	0.353705440241431	0.658696615692836
**Rpl36**	8025.78022464491	-0.105691619736377	0.113994036242309	-0.927167974925601	0.353839327596758	0.658724750111308
**Gm43200**	78.5018729758703	-0.458976326615098	0.495019894358819	-0.927187638003101	0.353829120104931	0.658724750111308
**Dclre1a**	319.594560374438	0.222264116540434	0.239892270547462	0.92651637350883	0.354177692019318	0.658809809130964
**Apool**	1500.32643780578	0.092171020510598	0.0994649352046112	0.926668481922712	0.354098686682923	0.658809809130964
**Srgap2**	2109.42851787648	-0.104036969849764	0.112278157521105	-0.926600259094995	0.354134120343961	0.658809809130964
**Srp72**	8423.89460897203	0.0540601955874412	0.0583284951067726	0.926823081728439	0.354018398724311	0.658809809130964
**Dnttip2**	7057.04574775249	0.0851819171575524	0.0919001749733782	0.926896136837912	0.353980463185953	0.658809809130964
**Numa1**	12753.5857791077	-0.183139651435825	0.197661607417362	-0.926531225910381	0.354169977169307	0.658809809130964
**Gm31749**	63.3154428943827	0.315828858589582	0.340743769338494	0.926880803140493	0.353988425346445	0.658809809130964
**9430064I24Rik**	35.2902724596855	0.581891520737519	0.628132457518119	0.92638346223453	0.354246735448648	0.658860459214393
**Tnnt1**	104.26207112281	-0.292335499944495	0.315623762822003	-0.926215115524615	0.354334198729964	0.658945352223302
**Kazald1**	50.8462357484496	-0.333338373666838	0.35995202806828	-0.926063329760171	0.354413069587455	0.659014247992627
**Upf3a**	2459.01700384636	0.0852501783005336	0.0920806455773651	0.92582081463479	0.354539108207415	0.659170823365692
**Tmem63b**	687.658529546601	0.142756333127718	0.154210372176609	0.925724587216656	0.354589126832074	0.659186039581524
**Zfp513**	1062.5519098874	-0.0736207190721515	0.0795495466428372	-0.925470001767515	0.354721480812582	0.65927652431751
**Zfp472**	2477.67152353127	-0.105700939275693	0.114208501257458	-0.925508505162974	0.354701461650892	0.65927652431751
**Dnajb9**	1611.062725424	0.112180791060441	0.121288636517433	0.924907677103923	0.35501393289354	0.659607776956833
**Slc39a9**	3197.83096543368	-0.0888922476162455	0.0961199515025713	-0.924805373147401	0.355067155178515	0.659607776956833
**Ing2**	798.320507574088	0.164304289504183	0.177649452023589	0.924879236229597	0.355028728378168	0.659607776956833
**Prob1**	18.6165272842417	-0.869548129899682	0.940034124291522	-0.925017621626275	0.354956741318831	0.659607776956833
**Cryz**	261.92600118611	-0.190872671248739	0.20641694994528	-0.924694756410936	0.355124707743741	0.659613318586741
**Slc26a11**	3765.03084270692	0.133935768451842	0.144851999541986	0.924638726944326	0.355153861451413	0.659613318586741
**Tpra1**	8378.17461249211	0.0625181698124492	0.067627163792807	0.924453522906705	0.355250239069966	0.659686909478742
**Gm45828**	100.396950658736	-0.353363045475824	0.38226135987692	-0.924401685772268	0.355277217353342	0.659686909478742
**Mrps16**	2581.35211196653	0.0917679198929175	0.0992879624719277	0.924260278972525	0.355350818132036	0.659745827890478
**4732490B19Rik**	53.7683585687239	0.594536802362328	0.643517283268312	0.923886300835277	0.35554551625333	0.660029535768581
**Asph**	96.5776479200109	0.202655115351542	0.219399778602084	0.923679671159054	0.355653119339782	0.660073757530854
**Pim3**	2901.38115603532	0.183628183713531	0.198793501109048	0.923713213405311	0.355635650707785	0.660073757530854
**Vwf**	178.798973308282	0.270908163641098	0.293456249096416	0.923163723639399	0.355921890385043	0.660276750537093
**Clpp**	4069.73847658555	0.0955342264059013	0.103478047654471	0.923231821351182	0.355886409106389	0.660276750537093
**Arf3**	21223.9877606609	0.0988535793742451	0.107081528643413	0.923161824701183	0.355922879829933	0.660276750537093
**Lrtm2**	65.7667723658043	0.481204749067938	0.521265034724674	0.923147951640579	0.355930108463671	0.660276750537093
**Clic5**	21.093432934024	0.57085426846534	0.618569461575682	0.92286202912637	0.356079110561538	0.660475402603667
**Adora3**	17.747927957294	0.86856013672895	0.941721700260545	0.922310844582477	0.356366458973145	0.660666404285408
**Traf3**	2485.69229127039	-0.117308563273052	0.127160970271153	-0.922520196432185	0.356257300601482	0.660666404285408
**Nek4**	195.895301702041	0.236627637093235	0.256573009596623	0.922262390207195	0.356391726624736	0.660666404285408
**C2cd5**	3234.85608597963	-0.101736746185424	0.110300151584148	-0.922362705075783	0.35633941637379	0.660666404285408
**Aoc2**	367.060771352677	-0.211324143397853	0.229117218870167	-0.922340732136783	0.356350873981655	0.660666404285408
**Cct4**	14682.3989890069	-0.0632878213293188	0.0686426582879917	-0.921989662227145	0.356533967984468	0.660835149243166
**E2f7**	42.5283123989596	0.880248683378044	0.954843961376648	0.921876996644503	0.356592739193964	0.660835149243166
**Gm37409**	84.884776647794	-0.424741631254317	0.460750855894749	-0.921846646230307	0.356608572317995	0.660835149243166
**Polr3k**	4115.62183488317	0.145235274318185	0.157600309890348	0.921541806733971	0.35676762475173	0.660946642585287
**Zfp954**	2281.57937240032	-0.1089493017996	0.118226105764665	-0.921533371119144	0.356772026736339	0.660946642585287
**4930423D22Rik**	17.111516322758	-1.11506569298899	1.21006793333851	-0.921490159575245	0.35679457649492	0.660946642585287
**Mon1a**	2827.37280190073	-0.0694850063685594	0.0754126535930903	-0.921397180153411	0.356843100451544	0.660958826121427
**Gm42798**	61.3267254223096	-0.423921242621312	0.460158464207104	-0.921250559525766	0.356919627044227	0.661022868281134
**Erp44**	6034.60052548274	0.0623551979279253	0.0676942683862848	0.92112965269241	0.356982740457474	0.661062056809074

**Gramd3**	37236.6440282711	-0.0812008876155947	0.0881636395247749	-0.921024676990296	0.357037543515206	0.66108573946476
**Rps4x**	25264.8458827372	-0.0635310676238652	0.0689907110677078	-0.920864079245617	0.357121394575366	0.66108573946476
**Gm12791**	24.2426702149433	0.770254514509823	0.836392309757671	0.920924912297424	0.357089631051992	0.66108573946476
**Bloc1s6os**	37.1869381157844	0.380219545203197	0.412936912628688	0.920769089841792	0.357170996141217	0.661099892657953
**Rad54l**	340.160325021177	0.198753404915202	0.21594785991681	0.920376821477962	0.357375876782145	0.661168450212076
**Neil1**	1827.54959744125	-0.0723900530186354	0.0786489421564086	-0.920419919630626	0.357353363122921	0.661168450212076
**Rad54b**	60.2210915120369	0.425894157007627	0.4627035961786	0.920447043258412	0.357339194711022	0.661168450212076
**Gm5866**	88.242218314149	0.186403219018477	0.202511532399516	0.920457303393169	0.357333835276234	0.661168450212076
**Smarcd2**	11903.593185828	0.297946517851571	0.323766456404812	0.920251347715409	0.357441426985565	0.661212087932796
**Ctsw**	45284.1906715738	0.0601785578867018	0.0654114072868882	0.920000965928839	0.357572254461021	0.661376454224944
**Zhx3**	1722.81482702328	-0.142985422364715	0.155461297339008	-0.919749319040565	0.357703773335821	0.661542060616494
**Nudt13**	1117.16416777318	-0.103661773907012	0.112748160770712	-0.919409888360142	0.357881219110525	0.661792555982226
**Gm49980**	106.76718191566	0.597973561617019	0.65055934702282	0.919168350056838	0.358007522955358	0.661948432199904
**Guca1b**	237.453844780487	0.219879820934623	0.239275806139925	0.918938794865205	0.358127586651062	0.662092735288137
**Rab11b**	18676.8552848488	0.0687776841686178	0.0748748250918645	0.918568879249252	0.358321116015383	0.662372809133551
**Mob3a**	57267.2350482457	0.0533927269054843	0.0581784098669252	0.917741255349064	0.358754343562715	0.662928253508389
**Tepsin**	2924.4115689758	0.0617147591371768	0.0672621558593512	0.917525737150408	0.358867212622609	0.662928253508389
**Erlin2**	2122.65897947867	0.11303780699893	0.123200259884683	0.917512731748577	0.358874024397193	0.662928253508389
**Snx25**	212.421043572364	-0.219376571129657	0.238999998427037	-0.917893609094015	0.358674567833412	0.662928253508389
**Cmc1**	462.839562076507	0.114234201498758	0.124468344983321	0.917777138549287	0.358735553335585	0.662928253508389
**Myo15**	17.9557763255962	0.926026765421975	1.00921195780716	0.917574111422603	0.358841876584288	0.662928253508389
**Ndufa11**	3483.04249855173	0.102568604718185	0.111918084585965	0.91646140208379	0.359424942954312	0.663208624236044
**Rsl1d1**	9534.01573603265	0.0771983018429231	0.0842141126602119	0.916690794503811	0.359304691267486	0.663208624236044
**Rbms1**	10311.6263963351	0.115577917238908	0.126033193153354	0.917043473605214	0.359119859775091	0.663208624236044
**Strap**	8375.31882643599	0.0625654897766923	0.0682408352487113	0.916833587230657	0.359229849485923	0.663208624236044
**Fam160b1**	3891.13982407242	0.342813944871426	0.373907888378674	0.916840632482837	0.359226157119925	0.663208624236044
**Ptpn13**	306.463749147751	0.260010192084495	0.2837123875892	0.916456959436597	0.359427272120503	0.663208624236044
**Itga2b**	900.688029684655	0.199543450903884	0.217742380027279	0.916419903552471	0.359446699941348	0.663208624236044
**Nemp1**	896.938359832211	0.114206956188146	0.124598818567954	0.916597424443956	0.359353634503169	0.663208624236044
**Gm7658**	207.112603705161	0.161936491670219	0.176621809309956	0.916854449079019	0.359218916010484	0.663208624236044
**Gm5425**	54.0241902752955	0.4268598386564	0.465729001838207	0.916541243881329	0.359383085560484	0.663208624236044
**Sidt2**	19072.6375181626	-0.104478047245501	0.114033736516665	-0.916202962710357	0.359560451871401	0.66334083130051
**Gon7**	846.229934409187	0.144049962545774	0.157239725288175	0.916116854578399	0.359605608542044	0.663346473167524
**Tbc1d16**	317.973425466971	-0.266909754587067	0.291414693855905	-0.915910419805547	0.359713881199079	0.663468526987463
**Pigbos1**	416.551110712858	0.179547930091077	0.196092985804866	0.915626478704069	0.359862838478268	0.663665583555953
**H1f0**	680.175182810364	0.179222804443873	0.195785930441135	0.915401857733381	0.35998070348489	0.663805259778983
**Mff**	9668.42363668007	0.0544490210791457	0.0594886473198325	0.915284235434167	0.360042432917041	0.663841401295789
**Tcf7l2**	141.379820626614	-0.30953414268837	0.338377066003127	-0.914760998269042	0.360317113878135	0.664209864384972
**Cr2**	274.357126058431	0.293629157663628	0.321043462778836	0.914608742137528	0.360397067650303	0.664209864384972
**Akr1b10**	1988.53486656677	-0.0766562063946317	0.0838112052350858	-0.914629567485819	0.360386131044855	0.664209864384972
**Tmem181c-ps**	33.9306183309818	-0.403701168166341	0.441404902890641	-0.914582428791823	0.36041088662128	0.664209864384972
**Fam32a**	15072.8432404493	0.0883171580853127	0.0967032739784441	0.91327991754446	0.361095341654625	0.664364765196417
**Lmf2**	6933.42090956753	0.056608770098999	0.0619257802978447	0.914138987457689	0.360643818584468	0.664364765196417
**Adcy6**	172.272380130848	-0.234168916119282	0.256334234378746	-0.913529621538129	0.360964061883753	0.664364765196417
**Cnpy3**	4267.98192686459	0.0735038118837619	0.080473646401355	0.913389850848417	0.361037541456921	0.664364765196417
**Sbds**	5894.6680803786	-0.0922482827978557	0.100977021650127	-0.913557176577106	0.360949576888577	0.664364765196417
**Hipk3**	2162.40959246316	0.125774181009143	0.137717974886858	0.91327352956266	0.361098700475028	0.664364765196417
**Pgm2**	2026.12351478185	0.0713611457337482	0.078070277721541	0.914062916341572	0.360683786899392	0.664364765196417
**Anapc15**	1634.98084445283	-0.095787194417162	0.104795735592971	-0.91403713972868	0.360697330748776	0.664364765196417
**Lhpp**	376.979762197681	0.174442423647735	0.191062019323335	0.913014654956228	0.361234834023734	0.664364765196417
**Nek3**	462.029117594707	-0.187952274702646	0.205867153244055	-0.912978451107396	0.361253874989078	0.664364765196417
**Chpf**	4515.83891206685	0.0811260522335007	0.0888505097915517	0.913062315836195	0.361209768334596	0.664364765196417
**Fancd2**	131.517385250649	-0.350759718439368	0.384153089198607	-0.913072752248584	0.36120427978863	0.664364765196417
**Asb6**	7712.23552200912	0.0637670603800444	0.0697862075121769	0.913748757143994	0.360848877854244	0.664364765196417
**Rtl8b**	152.567800102491	-0.210205367958353	0.23018767124101	-0.913191253141721	0.361141963420778	0.664364765196417
**Gm14335**	29.7537008231645	-0.638341170686415	0.698601593047647	-0.913741361369724	0.36085276491166	0.664364765196417

**1700058P15Rik**	30.9107736670288	0.341360405645184	0.373880218026435	0.913020772928532	0.361231616414232	0.664364765196417
**Gm28085**	26.6075971319015	0.55015662999976	0.601869938448599	0.914078931102395	0.360675372397198	0.664364765196417
**Gm45779**	54.7281934769218	-0.455687670252202	0.498857487424189	-0.913462625578918	0.360999281505763	0.664364765196417
**Dvl3**	1321.0086830459	0.164844859298506	0.180658246771843	0.912467945660364	0.361522436004768	0.664648499496168
**Sipa1l3**	812.22050169463	-0.264901686866505	0.290318552135639	-0.912451804811774	0.361530929243245	0.664648499496168
**Zfp862-ps**	616.92883818498	0.0999525120180267	0.109543645770786	0.912444636242726	0.361534701350616	0.664648499496168
**Dhx35**	1778.56610153435	0.136030149314062	0.149099930108916	0.912342140031141	0.361588637646142	0.664664791207629
**Cbl**	9128.50984274529	-0.171662426763818	0.188171155978563	-0.912267482607028	0.361627927589199	0.664664791207629
**Mastl**	106.385310846415	-0.244193464382641	0.267736912630944	-0.912064989407133	0.361734507104726	0.664783138377556
**Tnfrsf12a**	165.922661324135	-0.306806551249247	0.336447631469145	-0.911899869556323	0.361821430242134	0.664796721659014
**Snora19**	20.3510359258805	-0.501693175822299	0.550168127082805	-0.911890658738197	0.361826279428045	0.664796721659014
**Zmat5**	3926.52556958222	0.0874708987244428	0.0959611740753089	0.9115238487578	0.362019425651692	0.665074045702893
**Atg16l1**	5190.11543359919	-0.0718386023479803	0.0788430470563831	-0.911159639690311	0.362211266252487	0.665116294410959
**Gm9974**	28.2087447438445	0.426125456006684	0.467591089517757	0.911320736342863	0.362126403623608	0.665116294410959
**Slc44a2**	71461.1447506745	0.0734670820042211	0.0806193117491663	0.911283914613434	0.362145799506619	0.665116294410959
**Gm48225**	17.8384366397945	-0.758024623952706	0.831932110952669	-0.911161636836773	0.362210214117747	0.665116294410959
**5330426L24Rik**	25.4198040958538	-0.458838698726501	0.503644525148309	-0.911036804363924	0.362275981918846	0.665157614436641
**Fam98a**	1124.59208208195	0.112890774650216	0.124272380500912	0.90841403532451	0.363659513121103	0.665395449090152
**Tomm40**	8785.9554970129	0.0584866278075502	0.0642890608358112	0.909744629135587	0.362957201918408	0.665395449090152
**Actr6**	1574.42704564695	0.156633099146019	0.172097312425351	0.910142621860873	0.362747299306072	0.665395449090152
**Rps6kb1**	5360.77328259742	0.0641767556720845	0.0705922924206931	0.909118452898861	0.363287603107446	0.665395449090152
**Pygl**	163.400645596858	-0.229028057212924	0.25185937258076	-0.909348954800102	0.363165957155359	0.665395449090152
**Rab2b**	2555.95704790363	0.0880894963356699	0.0969687071543575	0.908432203756688	0.363649917777325	0.665395449090152
**Fbxo4**	1334.15073224266	0.112948337024415	0.124302781137284	0.908654947146119	0.363532292574578	0.665395449090152
**Mylk**	62.1210674776487	0.514066699240925	0.564808982504341	0.910160275712283	0.362737990370182	0.665395449090152
**Rhoq**	314.068017135132	0.258485812197828	0.284071626251779	0.909931821098975	0.362858466820842	0.665395449090152
**Als2**	924.174067569819	-0.102321530549991	0.112517188603138	-0.909385773145217	0.363146528862946	0.665395449090152
**Pbx4**	204.274164772707	0.212662779299766	0.233800816016469	0.909589551153604	0.363039011112925	0.665395449090152
**Mansc1**	73.8883390958504	-0.26665458826269	0.293230812791046	-0.909367558356515	0.363156140355441	0.665395449090152
**Ppp1r21**	1046.49034005441	-0.139188944005519	0.153236313200702	-0.908328718553908	0.363704573818436	0.665395449090152
**Ncaph**	391.39210797412	0.282990668093268	0.311547335768332	0.908339233251221	0.363699020212894	0.665395449090152
**Ppp6r2**	1041.99345466107	-0.107794949872644	0.118610051160339	-0.908818003348846	0.363446201770566	0.665395449090152
**Tmem135**	2264.01107300959	-0.0661245836279434	0.072676429987017	-0.90984908917177	0.362902102009981	0.665395449090152
**Slc35f2**	2347.3416096348	0.102015205097283	0.11209340100192	0.910091086410484	0.36277447497997	0.665395449090152
**Calhm6**	2391.52342425504	-0.13854642277104	0.152149336813587	-0.9105949830119	0.362508814921388	0.665395449090152
**Camk2n1**	64.9714162162675	-0.403720190236924	0.443967336709011	-0.909346604706494	0.363167197274078	0.665395449090152
**Tmem251**	4360.70999492653	0.141570851502622	0.155889053743084	0.908151330085947	0.363798274057268	0.665395449090152
**Rimkla**	72.455925563413	0.442377985999497	0.485823065847801	0.910574275075869	0.362519729981245	0.665395449090152
**C5ar1**	19.920373343127	0.68262020243786	0.751652318884001	0.90815951110397	0.363793952343156	0.665395449090152
**Lamtor4**	2531.95078865601	0.0683441230265585	0.0750798134215601	0.910286266200717	0.362671559499973	0.665395449090152
**Ptp4a3**	13198.8315542827	0.0748029955969218	0.0823049381125726	0.908851853999452	0.363428330822112	0.665395449090152
**Mtap**	5192.53443822814	0.0598428260032737	0.0658710436530818	0.908484558381123	0.363622268472034	0.665395449090152
**4833417C18Rik**	92.2218863179671	0.290604719183223	0.319569091244817	0.909364288177029	0.363157865964953	0.665395449090152
**Gm8494**	616.921037014727	0.198082086899355	0.217964167797726	0.90878280086467	0.36346478698265	0.665395449090152
**Trav14d-3-dv8**	284.166713713557	0.146161641716823	0.160944411390036	0.908149841640737	0.363799060348522	0.665395449090152
**2810029C07Rik**	18.9503217596485	1.06990410854425	1.17642332200498	0.909455030796911	0.363109984770406	0.665395449090152
**Pla2g4b**	81.7023950203216	0.426511900297642	0.468874660358573	0.909650139701442	0.363007047108391	0.665395449090152
**Gm33280**	39.8916955292467	-0.585434121565237	0.643693311151837	-0.909492317882952	0.363090311035097	0.665395449090152
**Gm37105**	98.0722135325526	-0.477420648835042	0.525673375375974	-0.908207779200495	0.363768454835825	0.665395449090152
**Gm42890**	682.005849460668	-0.174523602773134	0.192058094510402	-0.908702146702183	0.363507370728193	0.665395449090152
**Mrpl3**	3356.4772835034	0.0715326583870718	0.0787766062658166	0.908044428135156	0.363854749157371	0.665420065282346
**Bik**	81.764216840967	-0.22199359997657	0.244517861214444	-0.907882961489999	0.363940060566746	0.665421621530542
**Nop53**	29425.698363542	0.0464620659803922	0.051175735708006	0.907892487281303	0.36393502723781	0.665421621530542
**Fam162a**	1757.78507461964	0.10943878603509	0.12056027162058	0.907751654537649	0.364009446228617	0.665471266298215
**Cyhr1**	2425.35606840139	-0.0995248539827346	0.109677936679867	-0.907428212049907	0.364180396075412	0.665600403104173
**Pilrb1**	37.6227577160749	-0.498720629031064	0.549628151300923	-0.90737824809489	0.364206808121101	0.665600403104173

**Zfp953**	324.509728723823	-0.201017280868542	0.221505606181342	-0.907504258397748	0.364140198601418	0.665600403104173
**Gm11655**	32.7659615278009	-0.430981516350372	0.475072025856759	-0.90719194752233	0.364305301258401	0.665703192848037
**Stx16**	1348.11864459773	-0.122119116313529	0.134659610849046	-0.906872636446461	0.364474152929603	0.665884710600069
**Crlf2**	850.333980652121	0.119318394037748	0.131575396220515	0.906844269256661	0.364489155862512	0.665884710600069
**Eif2s3y**	6931.57189066418	-0.0789816842013449	0.0871045482670322	-0.906745810324561	0.364541232141816	0.665902642575771
**Zbtb32**	456.978851965863	0.171039687632643	0.188837941184483	0.90574852998184	0.3650689694249	0.666065527756711
**Alox8**	43.3925264736592	0.761416575973336	0.840484493699183	0.905925786473645	0.364975134609038	0.666065527756711
**Slc35a5**	2773.04696888108	-0.0739123133577658	0.0815662004577628	-0.906163495945108	0.364849321263894	0.666065527756711
**Pigp**	2288.4312865707	-0.0738963264751852	0.0815866311538305	-0.905740627234071	0.3650731532777	0.666065527756711
**Cers5**	4842.97380811078	0.0833711798076487	0.0920320550400707	0.905892841047056	0.36499257389121	0.666065527756711
**Tsc1**	3064.54064143069	-0.122106101897463	0.134873778797137	-0.905336107481067	0.365287353104022	0.666065527756711
**Ablim2**	276.231452372201	0.318419193647915	0.351679766589954	0.905423694787595	0.365240967537272	0.666065527756711
**Ift172**	769.779618747251	-0.193570214544098	0.213818977869293	-0.905299503687771	0.365306739282446	0.666065527756711
**Limd2**	110398.007622538	-0.0796368197720612	0.0878542773041733	-0.906464912304028	0.364689828510243	0.666065527756711
**1700029I15Rik**	150.221272322089	0.197645797073385	0.218310474966427	0.905342710210218	0.365283856221037	0.666065527756711
**Palm3**	255.120521355339	-0.222751797574252	0.24584986451629	-0.906048079434645	0.364910404790334	0.666065527756711
**Zfp35**	3122.18032861255	-0.107489073213609	0.118653711923284	-0.905905693730902	0.364985770409106	0.666065527756711
**Trbv30**	155.657484940766	-0.196347612772855	0.216627185206295	-0.906384914644353	0.364732154569971	0.666065527756711
**Trav3-1**	259.215403293157	0.126475830472586	0.139676136501303	0.905493476843165	0.36520401412782	0.666065527756711
**Prps1l3**	6029.24517142773	0.0691291933708069	0.0762784672511284	0.906274022827645	0.364790831532941	0.666065527756711
**Gm50433**	257.973362740757	-0.183821096387589	0.203046150034342	-0.905316827511864	0.365297564122016	0.666065527756711
**Lrrc1**	606.217959700602	-0.140983050771629	0.155775569663101	-0.905039545523963	0.365444437356947	0.666162424737785
**Fam177a2**	2645.40702199384	-0.112157353869049	0.123922656616913	-0.905059308208389	0.365433968059203	0.666162424737785
**Eef1akmt2**	1570.80793264244	0.100964823284357	0.111584553291951	0.904827956071944	0.365556538771695	0.666289691315859
**Wars2**	1437.62652470295	-0.100190016966924	0.110818581518185	-0.904090411502722	0.365947462297584	0.666693740712571
**E030024N20Rik**	51.7352403233109	0.308796021072457	0.34152851207276	0.904158833470014	0.365911185370809	0.666693740712571
**Wdr61**	4885.31948331643	0.113441703316517	0.12546552082364	0.904166360381794	0.365907194783234	0.666693740712571
**Gm5641**	1797.15541166198	-0.160770412752641	0.177825458794842	-0.904091089331156	0.365947102905908	0.666693740712571
**Zdhhc6**	4451.53953907477	-0.0970344670843143	0.107345322584306	-0.903946858123289	0.366023580752887	0.666755325077831
**Rapsn**	19.6117590637378	1.11944981188369	1.23854790561838	0.903840543272945	0.366079960032823	0.666780942115037
**Hrh4**	98.2755471427826	0.490313282698311	0.542582947545919	0.903665116856287	0.366173001356481	0.666798182400738
**Dnajb14**	464.825219353686	-0.159074537962804	0.17603300882518	-0.903663119913959	0.366174060564421	0.666798182400738
**Kat2a**	6035.02521981555	0.0580295662022666	0.0642282419181557	0.903489874068359	0.366265960016089	0.666888459899875
**Dynlt1c**	2696.87917227449	-0.104414156350126	0.115647087736504	-0.902868878012975	0.366595489846308	0.667115988141489
**Mprip**	1458.92806083359	-0.0912933295073471	0.101116057228976	-0.902856895424776	0.366601850190944	0.667115988141489
**Trim21**	4129.95211539232	-0.101031382911759	0.111902056474659	-0.902855462130301	0.366602610986682	0.667115988141489
**Eif3f**	11429.6486749822	-0.0725497365204078	0.0803351296133268	-0.903088560006163	0.366478895080983	0.667115988141489
**Gstp2**	1308.31165137003	0.117885638915029	0.130544096099709	0.903033093315754	0.366508331477539	0.667115988141489
**4933434E20Rik.1**	3995.90421364206	0.146939154513868	0.162827888815216	0.902420068104059	0.366833764628825	0.667459541484573
**Mmp19**	30.7766176733169	-0.851672042969536	0.944146685453927	-0.902054793064353	0.367027761699437	0.66768009511463
**Ifi206**	26753.3958898296	-0.0860963527006980	0.0954493776766752	-0.902010623812974	0.367051224260878	0.66768009511463
**Gm8437**	172.299257801797	-0.209401501756833	0.23216466695885	-0.901952499920879	0.36708210090614	0.66768009511463
**Gm23094**	49.245368070835	-0.482404115338564	0.535204810885013	-0.901344878684595	0.367404979113856	0.668190241908706
**Gm15787**	78.3126277396787	-0.347267836181203	0.38545320489307	-0.90093384040623	0.367623497229066	0.668510495769
**Frmd4b**	545.245868071783	0.265162744146041	0.294416422625025	0.900638428324896	0.367780595585644	0.668718998920262
**Derl1**	12552.6069195606	0.0664647045214893	0.0738486994301411	0.900011849015203	0.368113945030799	0.66873940609274
**Frs3**	93.8700483690868	0.328683880397608	0.365143213079969	0.900150594680844	0.368040114078039	0.66873940609274
**Rsph9**	35.5770740309442	-0.414467853408506	0.460477824002632	-0.900082114282527	0.368076553509488	0.66873940609274
**Prss30**	365.551751523088	0.16584382605291	0.184169643593732	0.900494906852033	0.367856934524462	0.66873940609274
**Dnph1**	23.500016844292	-0.67997616711411	0.75529451273016	-0.90027950111302	0.367971527155008	0.66873940609274
**Ak7**	19.4456398663995	-0.694384661450698	0.771556385151748	-0.899979152287267	0.368131345335307	0.66873940609274
**Gm6218**	35.381512538315	-0.30595126615831	0.339948624079326	-0.899992659146393	0.368124157292157	0.66873940609274
**Gm19195**	27.8008043538562	0.678621128932987	0.753993613988335	0.900035645319783	0.368101281610152	0.66873940609274
**Coro1c**	14298.0751433879	0.0730296937720455	0.0811738180741575	0.899670552706147	0.368295598815673	0.668953404249834
**B3galt5**	50.8738578667752	0.791638363161632	0.879990940882118	0.899598309919054	0.368334056944314	0.668953404249834
**4933434E20Rik**	6835.15867280084	0.0687611065668046	0.0764492390486288	0.899434806971279	0.36842110624625	0.668957290979738

**Gm13571**	47.3765527952326	-0.446490243659387	0.496384355078484	-0.899484923510113	0.368394422738353	0.668957290979738
**Mkrn2**	6235.01963553539	0.062481364560668	0.0694842520194047	0.899216192803212	0.368537516876464	0.669014476200743
**Hdac4**	6555.26233244666	-0.0741606212051195	0.0824679912617354	-0.899265521937473	0.368511247432096	0.669014476200743
**Gm42856**	799.930874081949	-0.161755286169458	0.1799673708248	-0.898803407685097	0.36875738468555	0.669336494699943
**Tmx3**	4629.95395643307	-0.0685140789910107	0.076248627469422	-0.898561472709616	0.368886287994001	0.669339157061432
**Tln1**	37615.3591776067	-0.160620382045227	0.17874767316404	-0.898587261037086	0.368872546600968	0.669339157061432
**Ripor2**	55098.4267898358	0.0590561984463197	0.0657126616846034	0.898703490809241	0.368810617145775	0.669339157061432
**Qrich1**	19816.4670074371	0.0775690022503699	0.0863456244705437	0.89835475423346	0.368996450103672	0.669345199373833
**Amz2**	1684.55874947473	-0.0990536762948282	0.110265948874344	-0.898316092193671	0.369017055718759	0.669345199373833
**Ifitm1**	276.351140815462	0.806655655453017	0.897901742046175	0.898378539298495	0.36898377378989	0.669345199373833
**Gm15892**	27.1046433296241	-0.648658196752036	0.722214374482937	-0.898151877988356	0.369104584547174	0.669426903626821
**Krt80**	23.7880730150196	-0.576099211905783	0.641532713439534	-0.898004419473898	0.369183193322995	0.669492413072901
**E030042O20Rik**	132.067508963465	-0.251562105754922	0.280221803149703	-0.897724955472255	0.369332201567546	0.66968555812426
**Timm44**	4292.49370037254	0.0884998849828559	0.098714870185621	0.896520299489255	0.369974943596799	0.669697836286464
**Ercc3**	2693.56574928617	0.101811735533457	0.113481995991663	0.897162009213656	0.369632474072123	0.669697836286464
**Atp5b**	88842.1069867659	0.0412789272655203	0.0460386532033619	0.896614570438954	0.369924620450101	0.669697836286464
**Gli1**	35.4677581560612	-0.377355238760649	0.420930667558006	-0.896478370059951	0.369997327477072	0.669697836286464
**Nacc2**	1003.64786287342	-0.167457647053651	0.186558381228814	-0.897615244893578	0.369390708698198	0.669697836286464
**Glmn**	824.719605649586	-0.145378513546264	0.16201164065965	-0.897333753021312	0.369540850768739	0.669697836286464
**Tmem209**	6454.41702561724	0.0531826262968062	0.059298907319107	0.896856766864404	0.36979535212424	0.669697836286464
**Tspan15**	38.6879574207866	-0.478366982433187	0.533294413643101	-0.897003550375323	0.369717022523134	0.669697836286464
**Trappc11**	4132.16226587698	0.0928853701030488	0.103580658509768	0.896744348215252	0.369855350222657	0.669697836286464
**Kcnrg**	21.494627882528	-0.818563890750814	0.912465628824598	-0.89709010936144	0.369670835991073	0.669697836286464
**Chtf8**	3757.12504721114	-0.0628627026145727	0.0700400781053954	-0.897524736051518	0.369438980146415	0.669697836286464
**Ppp2r5d**	9981.53729758771	0.0543724025659125	0.0606119069596836	0.897058107775401	0.369687911123046	0.669697836286464
**Rapgef4os3**	62.1241866230349	-0.465119710931707	0.518791039910261	-0.896545381763266	0.369961553914106	0.669697836286464
**Gm3200**	68.7881339554692	0.459726829372426	0.512837411740555	0.896437777057112	0.370018998712315	0.669697836286464
**Gm18194**	610.397505918802	0.280251089369617	0.31248556058667	0.896844925709414	0.369801671491599	0.669697836286464
**Gm47071**	382.149087526944	-0.213690686295675	0.238220750440948	-0.897028012463786	0.369703969568711	0.669697836286464
**Zfp442**	484.221898098387	-0.219984922091423	0.245474180400483	-0.896163179901549	0.370165617583622	0.669815211344284
**Efcab7**	215.2505599129	0.186184755733701	0.207759060718019	0.896157092211737	0.370168868463922	0.669815211344284
**Gm11223**	114.810937737184	0.286949950150687	0.320315127675415	0.895836397840884	0.370340147174623	0.669971262804884
**Gm3468**	22.1463847817397	-0.42622509010356	0.475754384849444	-0.895893140824002	0.370309837898508	0.669971262804884
**Rps15a-ps3**	123.836178831038	-0.372674789296469	0.416081570992285	-0.895677230807655	0.37042517450054	0.67004815458673
**Slc35a2**	2029.16877493512	0.0996269335028134	0.111251456089693	0.8955112769265	0.370513840282196	0.670054697730582
**Zxda**	60.5321136085401	0.324256282569613	0.362080284248529	0.89553697529426	0.370500109304915	0.670054697730582
**Mpzl3**	913.212431695896	-0.144492664055279	0.161410934687043	-0.895185102145856	0.370688147233129	0.670139184941763
**Gm43609**	43.7767583917091	-0.756632251495449	0.845174780842933	-0.895237610782498	0.370660083317088	0.670139184941763
**Gm4708**	16.8882270604586	-0.762905380622528	0.852141324965349	-0.895280346430271	0.370637243674358	0.670139184941763
**Ndufa13**	7606.55223428684	0.0780255179722563	0.0871909677888721	0.894880742248333	0.370850842277536	0.670316310284489
**Cyp4f17**	675.537919484804	-0.107331160494743	0.119954837345099	-0.894763086426949	0.370913746866184	0.670316310284489
**Gm32200**	79.1349781096738	0.351892957139254	0.393275365692096	0.894774979154834	0.370907388128072	0.670316310284489
**Gnpda2**	3654.621042641	-0.11730402397631	0.131135811820482	-0.894523184383026	0.371042030816229	0.670470288673895
**Grk4**	281.680568047592	-0.11343032973858	0.126816493581652	-0.89444461469475	0.371084050740943	0.670470288673895
**Tymp**	340.437323136072	0.228693944477865	0.255735585114034	0.894259374876939	0.37118313070084	0.670495539430601
**Zfp653**	1829.15112959684	-0.0962287876679971	0.107599281677416	-0.894325558385157	0.371147728979275	0.670495539430601
**Chmp7**	7080.49964651974	0.0658313153088576	0.0736220741839829	0.894179035819393	0.371226107076082	0.670496305501814
**Pxmp2**	117.905100590561	0.331174420737649	0.370493312761305	0.893874219400586	0.371389192890481	0.670637120373059
**Gm48585**	76.7683050766312	-0.332093126290398	0.371491310847012	-0.893945878661912	0.371350849066811	0.670637120373059
**Elac1**	1142.35123251616	0.0892545401700321	0.0999097200406089	0.89335191945042	0.371668742152107	0.671065013187893
**Gatm**	107.960971288309	0.553176474575278	0.619398665546402	0.893086319595626	0.371810948523171	0.671091078565819
**Atad3a**	8819.2650522308	0.0800397977231875	0.0896104496446516	0.893197144312786	0.371751607111799	0.671091078565819
**Grpel1**	6221.34828462629	0.0940800613560405	0.105334253253529	0.893157339138292	0.371772920230211	0.671091078565819
**Trmt44**	1172.1753952966	-0.117178628798969	0.131242690505738	-0.892839276209794	0.371943249749445	0.671252982052686
**Gnai3**	18717.9544007521	0.0528517164033068	0.0592207283291878	0.892452995672768	0.372150176313941	0.671549510785474
**Mcrip1**	7326.44397106732	0.0858861599678103	0.0962489908172328	0.89233309605188	0.372214419834619	0.671588527806491

**Use1**	7468.80910336446	0.0768759296400144	0.0862084513818226	0.891744700290766	0.372529788314035	0.671809604720613
**Gca**	68.5005068805555	0.272115395989526	0.305213546829185	0.89155739912756	0.372630212760635	0.671809604720613
**Sirt6**	1651.45349523542	0.0658800507380044	0.0738940254238197	0.891547731499927	0.372635396665492	0.671809604720613
**Uggt2**	32.9901116105272	-0.384214392747078	0.430900659158534	-0.891654223730767	0.372578296641991	0.671809604720613
**Plxnb1**	79.8712670334512	-0.464474326706944	0.520829516879981	-0.891797242002273	0.372501620266374	0.671809604720613
**Gm6767**	82.5508315033778	0.19707794316496	0.220967355403329	0.891887142357456	0.372453427000856	0.671809604720613
**Rtl10**	39.3752080485445	-0.382546442664812	0.429047020371873	-0.891618924035979	0.372597223369463	0.671809604720613
**Gm6361**	218.451929371516	0.147828407619955	0.165897834806565	0.891080994470608	0.372885720142956	0.672183994084494
**Myo7a**	459.608530556826	-0.176949365311298	0.198675016763276	-0.890647290203255	0.373118420609656	0.672372707404661
**Arfip2**	2082.91830042448	0.0985283059456764	0.110622350547139	0.890672684664124	0.373104792941969	0.672372707404661
**Zadh2**	1947.21446762752	-0.146714272179686	0.164713734517955	-0.890722759756825	0.373077921578515	0.672372707404661
**Pcnt**	6458.20321649962	-0.0992429559653508	0.111488238420547	-0.890165253046648	0.373377159182466	0.672377588255785
**Psmd4**	18837.6394171775	0.0622556590068967	0.0699161467224132	0.890433210715534	0.373233316344333	0.672377588255785
**Stk3**	417.715715513486	0.124762331024403	0.140135717314113	0.89029644558603	0.37330672919188	0.672377588255785
**Cyb5r1**	1868.49145105567	0.0921895241944218	0.103553206797341	0.890262378593849	0.373325017077923	0.672377588255785
**Pelo**	47.7708600378545	-0.358728549864358	0.402880391429945	-0.890409554535829	0.373246013879676	0.672377588255785
**Nfkbil1**	937.223178685304	-0.0836203252601516	0.0939307224145789	-0.890234026850973	0.373340237319453	0.672377588255785
**Ecpas**	3060.4890979362	-0.0943499883362788	0.106001821192429	-0.890078937087334	0.373423501915009	0.672384198340167
**Med16**	5215.78835038166	-0.0968380191014645	0.108850123446159	-0.889645468793278	0.37365628334154	0.672725816486651
**Tmem50a**	18887.1697389094	-0.0575714396149308	0.0647185221458459	-0.889566660456047	0.373698614692369	0.672725816486651
**Mast3**	5645.43955112129	-0.114090791196308	0.12829043149912	-0.88931645067458	0.373833032806704	0.67289091820142
**Cep89**	649.162674341898	-0.134412145150035	0.151156735507716	-0.889223657143442	0.373882891105739	0.672903793849586
**Slco2b1**	50.5931381509021	-0.59237475919935	0.666352230395527	-0.888981430808348	0.374013059576193	0.672954319026912
**Gna11**	2411.10220588763	0.14088175850183	0.158512040243845	0.888776387491484	0.37412326840922	0.672954319026912
**Impg2**	35.2488133169072	-0.454430953042744	0.511278509516079	-0.888812935777134	0.37410362258215	0.672954319026912
**Mrps14**	4925.06544883994	0.0866228931302348	0.097433448338643	0.889046776104704	0.373977941321552	0.672954319026912
**Rom1**	988.390353920797	0.179253570706626	0.201686315506221	0.888774085920059	0.374124505595973	0.672954319026912
**Zfp772**	541.744193929479	0.201935394962036	0.227250159447812	0.888603974812219	0.374215954130499	0.673041980280137
**Meis3**	3736.1395363202	-0.0583625699745279	0.0656942784394391	-0.888396544736085	0.374327483347401	0.673108149645464
**Gm15539**	16.9186647440847	0.665044322517099	0.74860626055589	0.888376650795385	0.37433818083031	0.673108149645464
**Cd52**	34682.037332978	0.0763178753263236	0.085936785010048	0.888069937889814	0.374503132164364	0.673210138882689
**Itgb4**	25.750149601365	-0.376673014808803	0.424262326905441	-0.887830455172033	0.374631958095971	0.673210138882689
**Gigyf1**	1189.76670969504	-0.125504637908167	0.141350002581392	-0.887899792119912	0.374594656566479	0.673210138882689
**Setd6**	4509.51136766625	0.0966582027474577	0.108884249884137	0.887715191593924	0.374693972075978	0.673210138882689
**Gmds**	993.792905402481	0.0805962490922989	0.090789044707937	0.887730996086282	0.374685468585465	0.673210138882689
**Gm10053**	1051.9414003315	0.139610828414839	0.157213788183734	0.888031705283237	0.374523696951346	0.673210138882689
**Zfp777**	618.733042316292	-0.17713497864408	0.199460144067896	-0.888072048036742	0.374501997166031	0.673210138882689
**Gm11605**	41.4523568351436	0.278828908538531	0.314248830875858	0.887287019529694	0.374924392293254	0.673547332044785
**Atg4a**	2225.22281581042	-0.117278301140548	0.132197151025556	-0.88714696368817	0.374999782159284	0.673605969845399
**Trav13d-1**	98.7077267923171	-0.259054594601227	0.292039282442423	-0.887053934781191	0.375049863340663	0.673619137884284
**Cant1**	10566.8579147045	0.0723107198692232	0.0815443960100139	0.886765043429144	0.375205411454676	0.673714069148789
**Map10**	341.815598966327	-0.184909283970987	0.208532338287646	-0.886717549370812	0.37523098755155	0.673714069148789
**Gm12816**	35.0994120297759	0.325261092040908	0.3667672389967	0.886832457911636	0.375169109829759	0.673714069148789
**Gm42913**	28.7443525146974	-0.514114394712118	0.579863402428708	-0.886612937734635	0.375287325920401	0.673738452150822
**Tspan14**	10609.5860725718	-0.0570613523144859	0.0643703727819983	-0.886453656369745	0.375373116588107	0.673815698117887
**Pik3r5**	20127.1011272839	-0.083306597235662	0.0940246026991688	-0.886008500373046	0.375612946664743	0.674114183318032
**Ncald**	437.15776669401	0.520972305124071	0.588066709727952	0.885906813812127	0.375667744095681	0.674114183318032
**Gm7730**	52.1295289520213	0.334892018136032	0.378006315538156	0.88594291780352	0.37564828760671	0.674114183318032
**Ntng2**	96.9297740644699	-0.343039629354902	0.387403661619008	-0.885483704313214	0.375895804746971	0.674446617558417
**Igkv9-120**	33.7057046068353	0.40977944760509	0.462830776113292	0.885376402680674	0.375953655086438	0.674473613024821
**Telo2**	3789.00900888591	-0.0830097747108881	0.0937749346303082	-0.885202160237594	0.376047607439513	0.674488577168401
**Gm33373**	38.3144613222377	-0.452975466598896	0.511674039740403	-0.88528131469932	0.376004925181287	0.674488577168401
**Ggct**	1380.23308532077	-0.0945004949947847	0.106801340035175	-0.884824993428552	0.376251027211108	0.674513102163062
**Tesc**	903.041219107246	-0.128765741035412	0.145549131119966	-0.884689177081235	0.376324294532027	0.674513102163062
**Gpbp1**	11594.9357733334	-0.0563353844462971	0.0636630748668483	-0.884898892554638	0.376211165377279	0.674513102163062
**Tmem59l**	21.4451873402503	-1.08803054549568	1.23010028667026	-0.884505562095961	0.376423361244634	0.674513102163062

**Ppp1r16b**	9900.93642280277	0.0914485310044268	0.103394458393421	0.884462595243356	0.376446545689025	0.674513102163062
**Hexdc**	151.504623673623	-0.314856043851883	0.355867286893126	-0.884756917672066	0.376287750175086	0.674513102163062
**Med14**	2765.8904142548	0.209367862273706	0.236680961924164	0.884599507166066	0.376372672580176	0.674513102163062
**Rpl17-ps9**	603.387443556079	-0.0956610704512809	0.108151545031885	-0.884509513230518	0.376421229299817	0.674513102163062
**Nutf2-ps2**	58.2237944933867	0.267765004815313	0.302569937917226	0.884968965054833	0.376173370066507	0.674513102163062
**Gm48088**	17.6311018999656	0.525528620582951	0.594301934287506	0.884278832464166	0.37654571201984	0.674614074394158
**Gm15567**	35.041800525249	-0.6676488597444	0.755173509197108	-0.884099947380618	0.376642261615093	0.67471033609243
**Rnft2**	23.4479774130185	-0.839858231057976	0.950179456267295	-0.883894327033014	0.376753259891497	0.674773421979033
**Dazap1**	780.837264401793	0.124604479728883	0.140981285726898	0.883837021959498	0.376784197994925	0.674773421979033
**Gm15446**	2448.638172885	0.0558661506704332	0.063211537725252	0.883796735229803	0.376805949101575	0.674773421979033
**Cramp1l**	1023.80572318569	-0.162987624002357	0.184570197832715	-0.883065770726869	0.377200736801579	0.675096775304689
**Fbxo8**	2249.28398627306	-0.0951815096270714	0.107782522221768	-0.883088534811151	0.377188438267661	0.675096775304689
**Dip2c**	94.7122570624684	-0.345910276412004	0.391614295340903	-0.883293282516377	0.377077832297058	0.675096775304689
**Ndufaf2**	211.014069936801	0.213307344611133	0.24149709207733	0.883270861674926	0.377089943198421	0.675096775304689
**Gm37390**	99.2310563354759	-0.381302487586467	0.431777595510823	-0.883099288964633	0.377182628310084	0.675096775304689
**Coa3**	3546.25521668853	0.11500227613604	0.130286170631278	0.882689817183336	0.377403885380634	0.675150247573862
**Ahcy**	3536.20216040847	0.0619381032879519	0.0701604218315484	0.882806882727447	0.377340621136461	0.675150247573862
**Rexo2**	7750.50882319507	0.0782280845764278	0.0886089782030442	0.882846029407664	0.377319467052247	0.675150247573862
**Gm7899**	19.1639319446543	-0.530062068070607	0.60047501677985	-0.88273792124301	0.377377888323769	0.675150247573862
**Gm47428**	407.138050457067	0.176653962500984	0.200148602705394	0.882614018350194	0.377444851867624	0.675150247573862
**Gm9625**	75.250287986487	0.241262779016248	0.273425236805351	0.882372021818895	0.37757566045298	0.675307568871464
**Gm4535**	22.1316242540384	-0.613512028168967	0.695642804850188	-0.881935418423671	0.377811732318415	0.675653100231672
**Sap130**	6887.77603702425	0.103691780274504	0.1176715345847	0.881196804651734	0.378211308478692	0.676290920074757
**Tspo**	19343.4184905056	0.0455115445561834	0.0516547742056714	0.88107140639067	0.378279172414204	0.676335517954229
**Gm7308**	57.4434869579028	0.354677725946364	0.402612888103146	0.880939821915237	0.378350392312172	0.676386105248796
**Slc7a7**	105.915447461517	0.234609779088056	0.266420711657241	0.880598875472897	0.378534967467747	0.676444006789065
**Gm4895**	31.7637887240384	0.452122777603921	0.513438554790813	0.880578159519257	0.37854618406624	0.676444006789065
**Ndnf**	144.304699944774	0.283878838752345	0.322339545822262	0.880682629331729	0.378489621247577	0.676444006789065
**Gm38130**	20.9003848049564	-0.989695621965938	1.12393530611699	-0.880562801595027	0.378554499705907	0.676444006789065
**Zbtb14**	1845.35650024908	0.116842545591475	0.132728073464155	0.880315238079836	0.378688559964395	0.676606830633741
**LTO1**	3293.20580496358	0.0876171679293079	0.0995483621154448	0.880146755480513	0.378779813142425	0.676693142367936
**Gm45477**	28.3636536896315	-0.489383946131883	0.556077783630676	-0.88006383376199	0.378824729992073	0.676696663698571
**Tgs1**	3028.52771120632	-0.0822427748253952	0.0934924695787045	-0.87967271798464	0.379036632897477	0.676921707419873
**Tprkb**	1790.31695157065	-0.0672944308712366	0.0764979040180776	-0.879689865167207	0.379027341184378	0.676921707419873
**Eif4e3**	1005.81659847941	-0.112217172441378	0.127579451556175	-0.879586571917245	0.379083315838284	0.676928355356283
**Bloc1s3**	1086.05031117592	0.126503376849642	0.143923365192074	0.878963444752812	0.379421096578623	0.677454755670183
**Mettl16**	4854.03105759221	0.072314860448573	0.082291492382287	0.878764722270835	0.379528857695531	0.677570384172727
**Zfp993**	256.462034058161	-0.228682307941106	0.260276273461253	-0.878613731862694	0.379610747752652	0.677639804275353
**Ndufb11**	4274.08119234164	0.0751419985988433	0.0855701238435206	0.878133573071054	0.379871235382427	0.678027985491719
**H2-T-ps**	2331.39882698273	-0.0654577243251657	0.0746058857074322	-0.877380165177033	0.380280182910398	0.678681033199586
**Cenpp**	47.8134585625286	0.451372965569928	0.514561358091469	0.877199499091985	0.380378288114659	0.678779239617518
**Klri2**	55.6288748599954	0.482771983226816	0.550412719668223	0.877109060120959	0.380427404078057	0.678790013142107
**Srp54a**	3737.97206134024	0.136259520998264	0.155367933702738	0.877011863071865	0.380480194589789	0.678807339917494
**Gm9725**	108.664002675738	-0.330821868963355	0.377274811871401	-0.87687239792759	0.380555949980416	0.67886562932655
**Dcun1d5**	5358.39787718228	0.103224197380143	0.117730287843994	0.876785398817051	0.380603211294826	0.678873081319059
**Rpl13**	127793.681208331	-0.0486701147969575	0.0555333747035945	-0.876411978503572	0.380806108738244	0.679158104741202
**Ercc6**	2697.25276105873	-0.106301188560084	0.121338000604677	-0.87607499736556	0.380989264011292	0.679341034453167
**Gm4811**	25.2246217058834	0.444509677585237	0.50739370988658	0.87606461988778	0.380994905214358	0.679341034453167
**Fmo5**	1301.29302822788	-0.12711543205256	0.145113474173334	-0.875972632980478	0.38104491159376	0.679353323374392
**Igkc**	3191.03137479585	0.335894663124974	0.383584852077743	0.875672387232059	0.381208160719468	0.67956748370366
**Surf2**	6128.27338098114	0.0704141235344311	0.080431607475063	0.87545339133328	0.381327259884173	0.679677786571004
**Comtd1**	1027.91452362739	-0.104938398319318	0.119874799692534	-0.875399988892359	0.38135630583704	0.679677786571004
**Zfp574**	5783.85858653617	-0.130322759311412	0.148901755119418	-0.875226482098174	0.381450686742039	0.67969707161151
**Gm20075**	618.07672417858	-0.1143184707456	0.130616616665988	-0.875221496801854	0.381453398759953	0.67969707161151
**Ybx1**	5640.85718199173	0.165130159091804	0.188706092004301	0.875065332220647	0.381538358806723	0.679767893603234
**Gm16201**	85.0096932758689	-0.384693128938546	0.439654388149138	-0.87498985409433	0.381579426218227	0.679767893603234

**Irf2**	5282.81732462891	-0.0681162251150625	0.0778662529010882	-0.874784936698945	0.381690934807104	0.679784463759445
**Wdr25**	703.315255475415	-0.141590195967396	0.161866403266576	-0.874734924048523	0.381718152906237	0.679784463759445
**Klra10**	41.0418568451874	0.374544576343907	0.428117878424188	0.874863198244669	0.381648345362621	0.679784463759445
**Hnrnpdl**	7425.32354626973	-0.0821988945862558	0.0939877626558838	-0.874570180877797	0.381807818562596	0.679867306711586
**Peli1**	51247.7893092937	-0.092763643800915	0.106102715306783	-0.874281525526467	0.381964957722358	0.679993428859021
**Dis3l**	1958.15132212051	0.0794208892109677	0.090841058706969	0.874284055485968	0.381963580282303	0.679993428859021
**Zfp869**	7470.77213271559	0.0949142937431969	0.108649931100995	0.873578959336566	0.382347588778664	0.680520835466554
**Gm10509**	414.617835725004	0.227285012943475	0.260171703481524	0.873596205513622	0.382338193366389	0.680520835466554
**Map1lc3b**	25067.0076454812	0.0620321639062621	0.0710458268654658	0.87312888938189	0.382592828977156	0.680880416330817
**Ifnar2**	6396.86623705377	0.0578376890734036	0.0662539396266695	0.872969809785046	0.382679533474051	0.680957810158172
**Wdr31**	21.4597753919114	0.62616571786452	0.717414448814763	0.872808902718652	0.382767246262042	0.680960087992661
**Zg16**	40.9985018220009	-0.440430499216841	0.504606700231394	-0.872819364100548	0.382761543235798	0.680960087992661
**Gm15879**	114.104975762445	0.282326396531255	0.323553967800403	0.87257899648265	0.382892592879347	0.681106184917574
**Alpk2**	18.873739290034	1.06309209309831	1.21866571489618	0.872341020268119	0.383022365797994	0.681167070379255
**Ska1**	20.5107840162797	1.23958403712545	1.42096273830634	0.872355061613314	0.383014708039618	0.681167070379255
**Ccr2**	1946.55588462823	0.401619253307241	0.460448243106412	0.872235390882846	0.383079976128037	0.681167070379255
**2310061I04Rik**	2670.08549793686	-0.0662497170321054	0.0759570980643876	-0.872199158740196	0.383099738383002	0.681167070379255
**Ndufb3**	1912.82397093308	0.0574581357705139	0.065893683326427	0.871982455220712	0.383217948962823	0.681273411136912
**E030030I06Rik**	194.734327856253	0.184662580351502	0.211785771504551	0.871931003861294	0.383246018678529	0.681273411136912
**Rev1**	1596.41268752006	0.0670034049991095	0.0768680722512159	0.871667560234016	0.383389762264513	0.681398667764512
**Il18bp**	355.226471542629	-0.174365265765865	0.200041982919437	-0.871643358164909	0.383402969372353	0.681398667764512
**Hmbox1**	1014.61591538801	-0.176503573656882	0.202640054508185	-0.871020164721445	0.383743142979978	0.68157684523582
**Itgam**	84.7089590458442	-0.522267566011922	0.59945624495526	-0.871235507857461	0.383625575845147	0.68157684523582
**Iqce**	2378.63609495815	0.0975579464504941	0.111993957230077	0.871100092035091	0.383699503885283	0.68157684523582
**Akna**	95631.9982460885	-0.0741931582367425	0.0851831235416908	-0.870984241384744	0.383762757563762	0.68157684523582
**S100a8**	272.303584334311	0.49628279881159	0.56974531208544	0.871060785028744	0.383720964531806	0.68157684523582
**Gm37663**	31.0128343492325	-0.52162589811728	0.598833749803354	-0.871069638757956	0.383716130552044	0.68157684523582
**Yjefn3**	38.5137718585502	0.445905894096714	0.512120773065498	0.870704563354404	0.38391548619734	0.681694423711008
**Pdp1**	1516.71586194139	0.114347543750774	0.13132030023489	0.870752987514068	0.383889039713775	0.681694423711008
**Sgsm2**	505.206680810918	-0.123140309827191	0.141459824074072	-0.870496698502267	0.384029022659383	0.681742373821981
**Pcnp**	5119.30420331472	0.0592692579925686	0.0680860023204752	0.870505771709038	0.384024066415328	0.681742373821981
**Zbtb18**	86.1297047936633	-0.394612253265814	0.453370710104396	-0.870396442626273	0.38408379009129	0.681762789283368
**Mrpl13**	2504.80958892298	-0.0905315589250163	0.104033676704581	-0.870213971021079	0.384183482311652	0.681786139293242
**Tdrkh**	387.454789365001	-0.168797912611289	0.193962196958893	-0.87026191318643	0.384157287876083	0.681786139293242
**Ccdc28b**	557.970057123011	-0.1548976443641	0.178018548650461	-0.870120813467823	0.384234384462692	0.681799684231828
**Arf1**	36104.5088991228	0.0381192295078109	0.04381759603444	0.869952552345632	0.384326334357198	0.681886054550881
**Gm9104**	105.723694856063	0.194371689556852	0.223594603545476	0.869304028249139	0.384680860157162	0.682438224892636
**Setd1b**	744.104267064904	-0.13145557669255	0.151233448282066	-0.86922290132122	0.384725223525932	0.682440093110223
**Fbrsl1**	520.92282769092	0.172048390027739	0.19800647778872	0.868902835650259	0.384900278438029	0.682596925981769
**Rps26-ps1**	584.59311671034	0.572436138257145	0.658766003054374	0.868952155398184	0.384873300599915	0.682596925981769
**Zfp775**	1829.35796077844	0.118484564029216	0.136395273299877	0.868685264251915	0.385019303510168	0.682679811332216
**Qsox1**	4818.22102614331	0.0792155500247415	0.0911929179450829	0.868659012232131	0.385033666517065	0.682679811332216
**Tmem63a**	8140.07477660375	0.114323618797517	0.131636039936592	0.868482665177299	0.385130158019998	0.682701141193523
**Dixdc1**	20.1296985780333	-0.722700970920872	0.832145918099085	-0.868478658853217	0.385132350324886	0.682701141193523
**Gm4779**	65.822019085556	2.28566639906067	2.63226880598182	0.868325603322314	0.38521610971784	0.682772805492014
**Nr2c1**	1041.59500805639	0.113199078971745	0.130414179270452	0.867996713279106	0.385396131927015	0.6828614472927
**Dlgap5**	769.104417281025	-0.118631822399136	0.136670460456994	-0.868013629298227	0.385386871471783	0.6828614472927
**Hspa14**	7463.63901745802	0.0921010733785915	0.106092538062005	0.868120181315335	0.385328544077916	0.6828614472927
**Gm48505**	52.1438650219635	0.398660373797957	0.459421842156136	0.867743623000125	0.385534699152623	0.683030161293893
**Kdm2b**	2651.74150319513	-0.0853383602613412	0.098403246221329	-0.867231148750904	0.385815372611807	0.683220142306613
**Bex2**	41.603893682679	0.483585057214605	0.557566296150137	0.867314004726693	0.385769985342446	0.683220142306613
**Tor1aip2**	3500.55808702972	0.102571782602326	0.118246511627666	0.867440241495693	0.38570084099074	0.683220142306613
**9930014A18Rik**	122.910141708811	-0.26960442992555	0.310865913394274	-0.867269193273074	0.385794531984287	0.683220142306613
**D16Ertd472e**	5529.9925280427	0.0744402179317082	0.0858557159121517	0.867038579095608	0.385920871999055	0.683300951680728
**Ist1**	10354.5333774052	0.0628814079170091	0.0725284496688836	0.866989549674419	0.385947735606935	0.683300951680728
**Abcd4**	1645.54462981474	-0.0810338570761225	0.0934885421093677	-0.866778486943619	0.386063391586112	0.683308092154121

**Tifa**	90.3896670336471	0.302303748816646	0.348733396239994	0.866862055874351	0.386017595814459	0.683308092154121
**Gm12922**	110.882578961196	-0.162295302027589	0.187261542167168	-0.866677162589578	0.386118921778704	0.683308092154121
**Uckl1**	12582.5834115788	0.0860721994184793	0.0993141924889387	0.86666565232422	0.3861252302177	0.683308092154121
**Gm46430**	2020.21075938985	-0.128116633462714	0.147847873580212	-0.866543632723988	0.38619210945291	0.683349698893823
**Akap8l**	19433.8464435368	0.104237036424168	0.120413229182654	0.865661000304638	0.386676092972505	0.683381608288474
**Ikbip**	79.0675078327848	0.28979992111565	0.334615737914912	0.866067815343886	0.386452973653228	0.683381608288474
**Gtpbp4**	6932.70110379833	0.052893684941772	0.0611035077375747	0.865640728334912	0.386687213270295	0.683381608288474
**Ube2w**	1662.9973609504	-0.131201091622834	0.151551399759776	-0.865720091208665	0.386643679453429	0.683381608288474
**Ccnt2**	8588.00309558556	-0.07953154973685	0.091793573491638	-0.866417404962396	0.386261302633352	0.683381608288474
**2200002D01Rik**	103.741361820489	-0.19819429386206	0.22894962081361	-0.865667709593681	0.386672412598974	0.683381608288474
**Zbtb7a**	10555.8615427326	-0.077300206140231	0.089270569395454	-0.865909186686194	0.386539964820653	0.683381608288474
**Commd6**	2171.69651508113	0.130019968117589	0.150151471887898	0.865925365118368	0.386531092101629	0.683381608288474
**Aknaos**	35.7435079434803	-0.347305642565439	0.40106041716365	-0.865968387061551	0.386507498231374	0.683381608288474
**9930120I10Rik**	18.0755379682061	-0.612947725303786	0.707800883474734	-0.865988923741809	0.38649623592197	0.683381608288474
**Gm49871**	23.6962225563826	-0.38797207163674	0.448167125106366	-0.865686146757554	0.386662299021973	0.683381608288474
**Prepl**	1369.78092601381	-0.113812068493162	0.131495919126559	-0.865517874996736	0.38675460930304	0.683423718994202
**Padi4**	20.689263802577	0.560232515835876	0.647338983119029	0.865439175525234	0.386797786760823	0.683423718994202
**Abl1**	2655.89016932591	-0.148054766656865	0.171099448266431	-0.865314109174207	0.386866408863914	0.683468326546552
**Acadsb**	1930.31052786969	-0.0752047216517521	0.086938059715406	-0.865037958035142	0.387017955314599	0.683653651526603
**St6galnac3**	478.1873212404	0.196837522162557	0.227567077456821	0.864964846243658	0.387058083725889	0.683653651526603
**Mapre3**	738.540419696098	0.102220403170662	0.118212533176876	0.864717136360783	0.387194061563112	0.683663920668977
**Oasl2**	1160.03715904131	-0.182603902011941	0.211170316315376	-0.864723343688268	0.387190653757572	0.683663920668977
**Tmsb15b1**	140.09373990784	0.204364797294602	0.236325476715227	0.864759907121066	0.387170580905779	0.683663920668977
**Gm4724**	152.520488115131	-0.45639089383189	0.528035455763539	-0.864318652943389	0.387412866128389	0.683896989870605
**Rpsa-ps1**	78.1831487665369	0.21265460679519	0.246018718567775	0.864383848648517	0.387377062447744	0.683896989870605
**Sptssa**	6549.17955411184	-0.0390305629904314	0.0451673052444381	-0.864133088728767	0.387514783913678	0.684000274462622
**Gm19967**	18.9637891719459	-0.667740322496746	0.772824459339681	-0.864025865676247	0.38757368168452	0.684027610024975
**Gsk3b**	11552.0695871006	-0.0891473490233763	0.103186242657322	-0.86394607195294	0.387617516028366	0.684028357045465
**Gm13572**	54.3866110850804	-0.26233118840802	0.303693496026966	-0.863802458201892	0.387696417247993	0.684036505820945
**6720475M21Rik**	61.9747609249171	-0.477533318673595	0.552841603354582	-0.863779635570071	0.387708956875475	0.684036505820945
**Tulp3**	236.709626636794	0.1516199014797	0.175615523294737	0.863362751966042	0.387938052180923	0.684361492228384
**Pigl**	797.658486634588	-0.120666434090938	0.139825999565195	-0.862975658791384	0.38815085021789	0.684361492228384
**Tns3**	45.1464250138078	-0.435838555593381	0.504962915586023	-0.863110026778064	0.388076975594462	0.684361492228384
**Cd1d2**	194.073142096036	0.210302690284765	0.243672083840067	0.863056148946453	0.388106596238294	0.684361492228384
**Atp5md**	750.293299007218	-0.125526295229786	0.145458404751402	-0.862970382799939	0.388153751097612	0.684361492228384
**Slain1os**	26.2054067714998	-0.394195787376647	0.456744268784817	-0.863055793618206	0.388106791593182	0.684361492228384
**Sin3b**	7294.5354014113	0.0659734693792589	0.076459194968399	0.862858540513356	0.388215248054159	0.684393338956074
**Padi1**	27.5738737114794	-0.688754132938937	0.798505180581581	-0.862554369950727	0.388382527549418	0.684535065614785
**Traj23**	120.217304749755	0.363812787316601	0.421771366664827	0.86258294438872	0.38836681108511	0.684535065614785
**Hspb1**	27.3789407316979	-0.584405459511557	0.677783514361567	-0.862230265458777	0.388560818070593	0.684543024411711
**Fam129b**	350.559520092981	0.440542145979824	0.510916566159674	0.862258488291303	0.388545290653921	0.684543024411711
**Sfxn5**	117.336194223083	-0.179776689168924	0.208487790092306	-0.862288813600689	0.38852860693415	0.684543024411711
**Rap2a**	180.948991796288	0.318427278335608	0.369236087509947	0.862394790506576	0.388470306289146	0.684543024411711
**Trmt1**	3515.17099372044	-0.0989769231106176	0.114810201923259	-0.86209170833769	0.388637053821961	0.684600788940485
**Xpo6**	19317.3019090734	0.0861688374981788	0.0999868275395683	0.861801895495472	0.388796541688067	0.684655238447114
**Ubb-ps**	27325.2217374831	0.0802210661090517	0.0930941506880039	0.861719726923605	0.388841767396283	0.684655238447114
**Gm26513**	50.4700283465678	-0.452781369902303	0.525314963403236	-0.861923610492594	0.38872955545194	0.684655238447114
**A430027C01Rik**	60.4414470930206	-0.402023077176873	0.466531298301442	-0.861727988326973	0.388837220162372	0.684655238447114
**Fbl**	5458.85455722315	-0.0821535044791293	0.0953535034108416	-0.861567761439886	0.388925417869994	0.684726012220949
**Vps54**	1358.48294375513	0.11736522140047	0.136263336106278	0.861311815446313	0.38906632990799	0.684897571261335
**Lrp5**	39.8618791900721	0.516143029598999	0.59936708825877	0.861146765830025	0.389157215077395	0.684904527864908
**Uqcr10**	2704.97691906852	0.103611758166163	0.120313300710344	0.861182907911486	0.389137312204516	0.684904527864908
**Eepd1**	118.709928610374	0.300847199116336	0.349499539744596	0.860794264095988	0.389351364692496	0.685051528488435
**Kdm6a**	4145.55275232277	-0.0860087707145785	0.0999181226786049	-0.860792500988364	0.389352335918554	0.685051528488435
**Gm15743**	94.3031892257484	0.332590128957362	0.386392002257861	0.860758315425498	0.38937116768055	0.685051528488435
**Cmip**	1313.31112025424	-0.118896918825883	0.138167031179346	-0.86053031472863	0.389496780336595	0.685119532012025

**Spock2**	25.3053006458089	0.518199210836232	0.602162223712861	0.860564131109183	0.389478148286844	0.685119532012025
**Fiz1**	6692.85007230216	-0.0488099988086406	0.056728438976044	-0.860414982144189	0.389560330022233	0.685154829798004
**Hmgn2**	7350.15397513105	-0.118718472429509	0.13802811857139	-0.860103532948658	0.38973197390474	0.68519739931429
**Rabif**	5420.75103494194	0.098716066492275	0.114778722736745	0.860055453994627	0.389758474966934	0.68519739931429
**Lhfp**	22.7811736550625	0.556164034297525	0.646583194819773	0.860158505128716	0.389701674647384	0.68519739931429
**Zfp972**	28.3776753799795	0.451131198045587	0.524461794473647	0.860179335843413	0.389690193665386	0.68519739931429
**Utp14b**	89.7071655263793	-0.283186001776538	0.329316465219801	-0.859920567857204	0.389832829895078	0.685251662277638
**Gm44695**	21.0533505352496	-0.860867360407606	1.00142798657031	-0.859639806308891	0.389987625162428	0.685447296116495
**Gm41077**	123.620492859609	-0.229644605959395	0.267167717087664	-0.859552226079933	0.390035919352613	0.685455719522543
**Gm11712**	21.2815278485559	-0.492772122744983	0.573402793569371	-0.859382145101752	0.390129717151655	0.685544100943306
**Rad51**	316.798726486649	0.227640243106635	0.264975907121063	0.859097891502376	0.390286510508069	0.685635944606773
**Ndufs5**	1533.93285846906	0.0819952095842661	0.0954486222720589	0.859050739889715	0.390312522886511	0.685635944606773
**Cenps**	102.778199626019	0.303377233587387	0.353122395180724	0.859127706788808	0.390270062695793	0.685635944606773
**Zswim1**	3612.27858892995	0.116102206674084	0.13522104002872	0.858610513936478	0.390555435496154	0.685744609099222
**Ppp1r8**	4881.00064003527	0.0608788136940806	0.0709002121719481	0.858654887328638	0.390530946506757	0.685744609099222
**Psmf1**	8236.73367988258	0.0668147131479675	0.0778105428588418	0.858684577862127	0.390514561281174	0.685744609099222
**Gpr137c**	72.2859992191788	0.411334256220306	0.478955917118132	0.858814436817686	0.390442901318341	0.685744609099222
**Trbc1**	61074.2435765226	-0.048315348920058	0.0562853807419619	-0.858399610754306	0.390671842458996	0.685744609099222
**Gapdh-ps16**	733.151947110812	0.0881886357262831	0.102735551488747	0.85840426656923	0.390669272477593	0.685744609099222
**Gm47111**	49.7249395994967	-0.76243508227131	0.888218757251511	-0.858386603577902	0.390679022395362	0.685744609099222
**Tomm7**	824.666583967329	0.10908667805324	0.127171989318839	0.857788563641506	0.391009225861278	0.686236961334677
**Gm15880**	30.7063554794671	-0.442151778797313	0.515496131329364	-0.857720847791989	0.391046625361685	0.686236961334677
**Man2b1**	20746.3342580361	0.0425607167235304	0.0496655210695162	0.856946948446562	0.391474204655923	0.686693933401721
**Mgrn1**	5821.36070817068	0.104608299844995	0.122083991477215	0.856855174697647	0.391524928455991	0.686693933401721
**Fam219a**	382.801012357919	-0.126698064788549	0.147845192879305	-0.856964384983287	0.391464567847702	0.686693933401721
**Gm9843**	1213.95912756635	-0.0806694004707119	0.0941451807630944	-0.856861708871825	0.391521316854058	0.686693933401721
**Gm26660**	61.8837971126515	-0.377638280911735	0.440651852828537	-0.856999189922113	0.391445332318422	0.686693933401721
**Zfp810**	1602.74327375915	0.111026670863872	0.129594259021734	0.856725226117093	0.391596758495163	0.686739605232073
**5830462O15Rik**	99.2941691045755	-0.322215997479552	0.376134775336927	-0.856650378021875	0.391638134963217	0.686739605232073
**Arl6ip6**	1544.60039631811	0.0854414420834361	0.0997689537950602	0.856393084555593	0.39178038854243	0.686912605125522
**Ly6e**	142812.627641817	0.0684876090485689	0.0799906017404966	0.856195697473993	0.391889542057551	0.687006939559201
**Pex26**	498.446192764224	-0.0952498664043211	0.11125694468564	-0.856125131545291	0.391928568941916	0.687006939559201
**Gm42632**	17.777065192486	-0.671118038778513	0.783962112299933	-0.856059276652584	0.391964992488241	0.687006939559201
**Hectd4**	3016.0985364565	-0.183084311581387	0.213903762690354	-0.855919079116054	0.39204254089263	0.687066434973325
**Mir8119**	26.4827628981428	0.368141370006668	0.430175128016743	0.855794177836194	0.392111636214463	0.687111104518605
**1700094D03Rik**	156.274903002519	0.426591020927635	0.498598938113018	0.855579481460787	0.392230423395127	0.687242831250641
**Pigk**	4805.60376469511	0.0726728628386405	0.0849630087774183	0.855347096158342	0.392359022095783	0.687287458963951
**Tusc3**	2332.73758265686	-0.0746337249905042	0.0872640606634601	-0.855263030657425	0.392405549037628	0.687287458963951
**Gm10778**	533.340024219558	-0.0992291263201704	0.11600958584972	-0.855352819280927	0.392355854694601	0.687287458963951
**Gm28374**	25.9191311093925	-0.635408767381307	0.742978542151092	-0.855218194514277	0.392430365443845	0.687287458963951
**Ibtk**	4394.31603171234	-0.100308464501428	0.117306992312616	-0.855093652338405	0.392499303422553	0.687331798625157
**Gm7072**	1200.86338619312	-0.120590415539341	0.141108879519904	-0.854591262786772	0.392777466249014	0.687742475351229
**Nedd9**	23672.8901764563	-0.0848993856875205	0.099397514745327	-0.854139923971408	0.39302746518768	0.687796882980252
**Men1**	11225.4131425608	0.04834212604947	0.056615863063876	0.853861858379315	0.393181535167569	0.687796882980252
**Mmadhc**	3663.64153801427	0.0646580323590474	0.0756990843653466	0.854145501245276	0.39302437531816	0.687796882980252
**Haus2**	1901.87011373104	0.0927587849185187	0.10857931984343	0.854295136977056	0.392941481013693	0.687796882980252
**Snai1**	18.7481961849027	0.711492397333509	0.83288394811318	0.854251542421159	0.392965630170283	0.687796882980252
**Cycs**	6689.73718227345	0.120924369664349	0.141561101264913	0.854220323124326	0.392982924618277	0.687796882980252
**Mrpl23-ps1**	231.629487170726	0.147276453623316	0.172464578021903	0.853951897325909	0.39313164257801	0.687796882980252
**Smim6**	21.8280484492456	-0.419349420047844	0.491141528634399	-0.853826026916986	0.393201391247452	0.687796882980252
**AC241534**	37.5895566413778	0.577902768288292	0.676730752272045	0.853962623019643	0.393125699487459	0.687796882980252
**Donson**	5057.47640395284	0.302555542215182	0.354403674417261	0.85370317537669	0.393269474297692	0.687839616662086
**Adam9**	380.096568585068	-0.13304361273249	0.156033190465494	-0.852662259456346	0.39384662565951	0.687885296634802
**Tomm6**	2588.81513143631	0.128859611829844	0.151140590593187	0.852581105605745	0.393891644159841	0.687885296634802
**Igsf3**	597.305688426574	-0.190327050914327	0.223023773586825	-0.853393554657219	0.393441094567653	0.687885296634802
**1810024B03Rik**	48.9267990371895	-0.531385570569331	0.622761718340948	-0.853272696313054	0.393508097680992	0.687885296634802

**Sh3pxd2a**	324.946321265835	-0.247203246605209	0.289798441057218	-0.853017862012587	0.393649398875703	0.687885296634802
**Pla2g4a**	34.5164213850863	-0.348029477743451	0.408088286280533	-0.852828883954303	0.393754203764691	0.687885296634802
**Zfp558**	48.7179580822984	-0.344675065274552	0.40428539733641	-0.852553833369708	0.393906773594962	0.687885296634802
**5430405H02Rik**	614.9648226083	-0.10762838428243	0.126222115684019	-0.852690383924981	0.393831024891259	0.687885296634802
**Etaa1os**	45.7746224982905	0.346273302126853	0.405990240217341	0.852910409721869	0.393708988512546	0.687885296634802
**Cmc4**	494.776898409525	0.130208347752912	0.152606499570848	0.853229371744175	0.393532118234704	0.687885296634802
**Zfp87**	2494.28384610103	-0.142251655991123	0.166698302361215	-0.853347958414604	0.393466372028867	0.687885296634802
**Gm18752**	89.06705808233	-0.196589970109288	0.230379567743319	-0.853330753395294	0.393475910333715	0.687885296634802
**Gm37648**	28.7525217876982	0.555404454182686	0.651250588205069	0.852827566288198	0.393754934585285	0.687885296634802
**B230362B09Rik**	50.5657661685233	-0.432933511473387	0.50773922178668	-0.852669033426136	0.393842868074037	0.687885296634802
**Gm8251**	387.154776857218	-0.176700172862658	0.207288966739916	-0.85243404722241	0.393973229838233	0.687925108883094
**Gm10655**	32.0466994965549	-0.52484511231523	0.615826042199075	-0.852261964175861	0.394068711793034	0.688015589110564
**Lman1**	3368.57136245284	0.0936257730613156	0.10989534022115	0.851953985245473	0.394239631862773	0.68822457402668
**Zfp968**	36.744765152498	-0.349648038413927	0.41043854837254	-0.851888887630907	0.394275765040226	0.68822457402668
**Asb7**	937.382548438255	-0.0896713370404961	0.105282971409018	-0.851717384496378	0.39437096941548	0.688314506544054
**Psmd11**	13492.4825890625	0.0616051427621196	0.0723429577181465	0.851570694719695	0.39445241049891	0.688380400069922
**Ube2o**	527.840968828344	-0.14364962896223	0.168764255747959	-0.851185153666447	0.394666508560457	0.688410687799549
**Polr2h**	1513.21815971731	0.0887660343642066	0.104266334538756	0.851339358546376	0.394580867312125	0.688410687799549
**Acsl5**	16848.2715552925	0.0698214587563629	0.0820824716847304	0.850625685646253	0.394977316447714	0.688410687799549
**Slc40a1**	674.342834529008	-0.20869403912167	0.245205055255049	-0.851100067674372	0.394713767847793	0.688410687799549
**Zyx**	27030.4597633217	0.0722087672260954	0.0848998539887074	0.850516977752399	0.39503772539719	0.688410687799549
**Mif**	12648.5279476459	-0.0413350893284021	0.0485804542363989	-0.850858436342734	0.39484799572672	0.688410687799549
**Atg14**	1952.01704691675	-0.0573969229602028	0.0674522755134099	-0.850926414614315	0.394810230535696	0.688410687799549
**Alpk3**	23.262092160621	-0.801560915400076	0.941680136524562	-0.851202955558115	0.394656621293996	0.688410687799549
**Pus7**	3258.07875398235	-0.0638968027056634	0.0750667960121797	-0.851199279842663	0.394658662793871	0.688410687799549
**Trbv12-2**	4087.21315046216	0.0674371904338151	0.0792853819003446	0.850562724394495	0.395012303318902	0.688410687799549
**Thap6**	379.78889760673	0.131157288809032	0.154156580305216	0.850805645463545	0.39487732509955	0.688410687799549
**Gm49327**	43.6485454867624	0.447766450924252	0.526456904561535	0.850528214265094	0.395031481011053	0.688410687799549
**Gm30531**	156.055242963014	0.202433233342289	0.237927155306165	0.850820214623243	0.394869230684924	0.688410687799549
**Fggy**	270.37939941382	-0.210684427765604	0.247745639748147	-0.850406198792363	0.395099290983804	0.68844183656218
**Prps1**	13916.9340254415	0.112156260103471	0.131919910371288	0.850184477747197	0.395222530235783	0.688519462861629
**Mical2**	58.6991259547055	-0.422125259891485	0.496565247559333	-0.850090218689834	0.395274929304201	0.688519462861629
**Arglu1**	2727.98084306748	-0.0974742493783544	0.114654138148074	-0.850159016959925	0.395236683597343	0.688519462861629
**C1qtnf6**	20.9822339977253	-0.489387259024777	0.576034946655133	-0.849579112980047	0.395559128561394	0.688633873466124
**Cenpl**	771.113673458369	0.135156521271183	0.159073638222165	0.84964751408037	0.39552108711853	0.688633873466124
**Camsap1**	787.172416925023	0.185937338058779	0.218801523676566	0.849799100730364	0.395436789703035	0.688633873466124
**2610020C07Rik**	128.73413793632	0.271645697605159	0.319692419851211	0.849709535595454	0.395486595609045	0.688633873466124
**Gm4432**	85.720192639226	-0.279709046666367	0.329215230249325	-0.849623653360553	0.395534357066393	0.688633873466124
**Rcbtb1**	13719.6036821185	0.0744984197006698	0.0877481282098933	0.849002949925835	0.395879650817672	0.689115737730231
**Mpc2**	2054.85241493636	0.117519505291591	0.138447125866636	0.848840339269996	0.395970140279202	0.689197117019704
**Cxcr6**	21139.0638456947	0.108774289641477	0.128200245785681	0.848471771445124	0.396175286879498	0.68947802025185
**Snap23**	3875.10318870628	0.0612636713542248	0.0722116219310455	0.848390739827519	0.396220398066692	0.689480376845595
**Tmem168**	4764.74195671571	0.0748787126047092	0.0883152717166315	0.847856901181997	0.396517669389899	0.689882249973817
**Mettl21c**	42.5570379889905	-0.456944551679522	0.538964889148003	-0.847818774246798	0.396538905756988	0.689882249973817
**Rtn4ip1**	1761.47041235478	0.0805950837310039	0.0950891015699962	0.847574352899705	0.396675062600599	0.689983650348043
**Klf9**	257.843532328211	0.384506177733253	0.453664129295701	0.847556932328298	0.396684767944591	0.689983650348043
**B3galnt2**	6149.38141845416	0.0812253279431447	0.095867730457456	0.847264533702409	0.396847690374854	0.6901908451696
**Mrpl15**	3394.22969848179	0.0773377034368402	0.0912881264983345	0.847182502296738	0.396893404940603	0.690194170803342
**Ndufa5**	1236.61936070227	0.0997590637839398	0.117869109976893	0.846354603029555	0.397354955516129	0.690920551099938
**Gm15601**	51.1729497547948	-0.366696058783416	0.433386839528025	-0.846117199088839	0.397487366967182	0.691074527342149
**Pfdn2**	1349.43274159076	0.0977643845899081	0.115572311026139	0.845915286472003	0.397600004154239	0.691194093717823
**Gm20468**	96.4650011802355	-0.254560802023669	0.300963982927942	-0.845818159193544	0.397654193572714	0.691212038403227
**Htatip2**	1634.57598126338	0.0687031278953879	0.0812547836526563	0.845527177686872	0.39781656512301	0.691265506908168
**Mrps35**	2370.91492549105	0.0974413571811799	0.115230111447463	0.845624081736715	0.397762486937104	0.691265506908168
**Arfgef1**	3717.2990282774	-0.118283740901134	0.139887745091473	-0.845561852639687	0.397797213941274	0.691265506908168
**Ndufab1-ps**	193.713458152391	0.18127872949473	0.21442586007947	0.84541449164548	0.397879456165078	0.691298554503598

**Xpo5**	4443.42131992236	-0.0738636025358447	0.0874125113192854	-0.845000348589098	0.398110644415321	0.691547726166047
**Gm12655**	101.028264750082	-0.22888573099545	0.270861756609572	-0.845027861668094	0.398095283201953	0.691547726166047
**Slc16a4**	45.2973320098593	-0.33749338261866	0.399595785746123	-0.844586941747882	0.398341502389174	0.691791764482095
**Anapc13**	483.806123786207	-0.133115479381527	0.157631004680883	-0.844475232845295	0.398403897609323	0.691791764482095
**Smim13**	489.391365468175	0.23010715663963	0.272454318531308	0.844571515254539	0.39835011853607	0.691791764482095
**Snhg8**	359.460640339619	0.205458978616449	0.243309600612981	0.844434326055472	0.398426747647079	0.691791764482095
**Ltf**	464.200246257764	0.589207408545235	0.698056005482274	0.8440689628308	0.398630870130181	0.691939982684962
**Slc22a23**	71.9292921199583	-0.440545582351083	0.521945157535791	-0.844045731607107	0.398643851165456	0.691939982684962
**Romo1**	1150.11032051729	-0.0900578967447513	0.106692549143393	-0.844087965540268	0.398620252073704	0.691939982684962
**Abr**	7086.980607097	-0.0717677188705915	0.0850650057435418	-0.843680879619998	0.398847754853116	0.691988997271586
**Avpi1**	557.364894940209	0.164464689687282	0.194923885686453	0.843738001159252	0.398815827372419	0.691988997271586
**Susd4**	78.9011319726892	0.308866874434807	0.36608414760941	0.843704586641512	0.398834503877196	0.691988997271586
**Gm13012**	339.422605695391	0.105974793444953	0.125580015239135	0.843882629279436	0.398734995893304	0.691988997271586
**Nprl2**	4631.70179442063	0.069079985245876	0.0819061259954678	0.843404377954568	0.399002324287264	0.692028577192561
**Iscu**	4390.5699286532	0.0955549406536082	0.113279943864763	0.843529202024363	0.398932540909635	0.692028577192561
**Lysmd2**	1332.34326183962	-0.135188479335902	0.160285586173991	-0.843422559463049	0.398992159387686	0.692028577192561
**Xlr4c**	2461.42543934102	-0.0809008213651211	0.0959411339125088	-0.843233950506533	0.399097614272847	0.69211766543003
**Snapc2**	2559.45527299104	0.0793720928341635	0.0941619539147742	0.842931667560796	0.399266661448184	0.69223403533096
**Zc4h2**	483.825392414446	0.127417300950633	0.151153470173629	0.842966428784399	0.399247219566289	0.69223403533096
**Gm37569**	31.8048234770294	-0.459490115239971	0.545144081599449	-0.842878297223425	0.39929651247846	0.69223403533096
**Rrad**	4560.03285082669	0.0821972826700897	0.0975361402471185	0.842736676495849	0.399375730115893	0.6922952012581
**Appl1**	4598.67166254552	-0.0546619028314168	0.064876377697838	-0.842554790682132	0.399477484491185	0.692392537312438
**Hmga1b**	1338.72689265309	0.0872173010852428	0.10352457235261	0.842479221147385	0.399519765770432	0.692392537312438
**Gm12424**	19.7293911239781	-0.514722960608223	0.61122812808063	-0.842112685855196	0.399724881062415	0.692671830078135
**Cnksr3**	800.547170377006	-0.124586512729355	0.148169409305119	-0.840838289857792	0.400438533275459	0.693193150520677
**Abca1**	1934.87216739753	-0.141093846934039	0.167920803886174	-0.840240420893173	0.400773599114258	0.693193150520677
**Cacnb4**	60.9476124366564	-0.303600758285416	0.360915085907372	-0.841197196072138	0.400237471070663	0.693193150520677
**Ggps1**	5317.03410906209	-0.0703369860331707	0.0836327140287673	-0.841022401939231	0.400335384596336	0.693193150520677
**Cbx8**	786.323602772564	0.202924002249869	0.241501778759974	0.840258830770571	0.400763279088395	0.693193150520677
**Ndufb5**	3275.37482793834	0.0894547589161259	0.10641272209797	0.840639701273392	0.40054981030872	0.693193150520677
**Tead2**	80.4235393728468	-0.272821142434198	0.3242852309852	-0.841299930944589	0.400179929325329	0.693193150520677
**Nfyc**	6198.66948328916	-0.0592221918592001	0.0704294956281428	-0.84087201436007	0.400419637949312	0.693193150520677
**Ttc28**	1376.36908994756	-0.246491878188219	0.2929607440445	-0.841381936655571	0.400134001541101	0.693193150520677
**Ptpdc1**	249.562207833136	-0.191069154086295	0.227295835123675	-0.840618808445529	0.400561518466101	0.693193150520677
**Phtf2**	1285.2438074999	-0.0984078507865175	0.117010888910918	-0.84101447055442	0.400339827820572	0.693193150520677
**Fpr1**	26.8386422771753	0.757222921387937	0.899955586578576	0.841400323172308	0.40012370449837	0.693193150520677
**Atp11c**	1816.69328806795	-0.110293645737195	0.131139886253327	-0.841038137886876	0.40032656928227	0.693193150520677
**Naip6**	25.2436327736136	1.00433691199467	1.19507389261293	0.840397332920367	0.400685644049918	0.693193150520677
**St6galnac4**	4830.72731151397	0.0745870269531032	0.0887617467918436	0.840305983702847	0.400736847294946	0.693193150520677
**1810041H14Rik**	297.747710679643	-0.174617128377227	0.207791605731551	-0.840347365152073	0.400713651595761	0.693193150520677
**Gm42727**	171.455652182034	-0.345536795194298	0.411059043374711	-0.840601370444283	0.400571290726403	0.693193150520677
**Hcfc2**	2555.83090262599	-0.0649952810786097	0.077377155177853	-0.839980236146142	0.400919467961098	0.693211755058978
**Arpin**	42.0994637582404	0.66963480087058	0.797252269679491	0.839928371906403	0.400948548714528	0.693211755058978
**Phf11b**	6678.81646928572	0.0927899708164914	0.110486755562335	0.839828903873825	0.401004324894497	0.693211755058978
**Dynlt1a**	715.063389437815	0.190129297883874	0.226351511130054	0.83997361861937	0.400923178398355	0.693211755058978
**Gm47817**	117.886682944624	-0.375393798346444	0.446978450555554	-0.839847643392794	0.400993816450835	0.693211755058978
**Pdss2**	912.364877478176	-0.102613946693416	0.12225765114807	-0.839325356980213	0.401286757683817	0.693623896536189
**Txn1**	3273.53560222068	0.084393140130323	0.1005894459974	0.838986031720511	0.401477148068751	0.693800770138113
**Gpi1**	18073.4482974754	0.0586321283985934	0.0698796490801426	0.839044402345956	0.401444393313356	0.693800770138113
**Abhd16a**	9717.10173110076	0.0596085354171163	0.0710631890185553	0.838810307282324	0.40157576575838	0.69381380276247
**Myl12a**	29476.8929866583	0.0664810449974153	0.0792553189696188	0.838821240791418	0.40156962937834	0.69381380276247
**C030015A19Rik**	59.7904625430924	-0.502614762373712	0.599251766924036	-0.838737222175643	0.401616785872723	0.69381380276247
**Txnrd3**	417.8173692883	0.18547214031606	0.221164523028251	0.838616147728033	0.401684746259099	0.693855135584808
**Prrg2**	158.047082308993	-0.193285309007119	0.230529032552469	-0.838442372602794	0.40178230016113	0.693947572469464
**Gm9776**	83.1039986056304	-0.281357413303873	0.3356186923367	-0.838324621745469	0.401848411231819	0.693958474108096
**Gm43051**	39.6000589670919	-0.542060298789554	0.646638375213226	-0.838274249669783	0.401876694563456	0.693958474108096

**Serp1**	20759.5973981013	0.0504518594512426	0.0601913834156768	0.838190727447253	0.401923593946908	0.693963408921919
**Gimap5**	12652.2166982586	0.0611552972451987	0.0729739026047884	0.838043397190955	0.402006330810238	0.694030213057622
**Gm11695**	160.36347181938	-0.226662822150879	0.270539228913255	-0.837818689220689	0.402132540670541	0.694172046817014
**Ampd2**	6880.72708650577	0.0676770646323915	0.0808093996505114	0.837490006423568	0.402317192008278	0.694384088149425
**Gm12537**	1793.35213296433	0.0628181700444793	0.0750118608864593	0.837443163016089	0.402343512394761	0.694384088149425
**Tasp1**	3589.58298556452	0.0728324054768327	0.0869947232609248	0.8372048642351	0.402477423764786	0.694539126739868
**Sorbs3**	95.9095521022815	-0.331647455899934	0.396186134315875	-0.837100108191861	0.402536299600902	0.694564659747199
**Rbpms2**	49.5811374415625	-0.372593284907513	0.445157761996809	-0.836991549324448	0.402597318178595	0.694593883996509
**Pter**	17.8662868789011	0.739626994736499	0.88378947708396	0.836881422459202	0.402659223759036	0.694624632009101
**Dguok**	6328.32137810682	0.0534285969402055	0.0638618281955403	0.836628052310232	0.40280167230595	0.694739459796004
**Stk25**	6959.24208423583	0.0793844474707169	0.094888669287129	0.836606183510731	0.402813968693044	0.694739459796004
**Creg1**	3133.73186491462	0.0775718157753415	0.0927363760432839	0.836476678139067	0.40288679157546	0.694789008958577
**Pidd1**	1441.98838842396	-0.0936054929358509	0.111942244667575	-0.836194532402161	0.403045473822919	0.69498659783626
**Rbm38**	30666.5239957646	-0.0836490181086357	0.100104370220848	-0.835618044687671	0.403369814037713	0.69539510356361
**Arhgap4**	19879.0069772431	-0.114920211797387	0.137527445103063	-0.835616568832973	0.403370644575198	0.69539510356361
**Rdh11**	2282.44938560545	0.0747994350913532	0.0895423960809166	0.835352172436388	0.40351945022026	0.695575536278376
**Map3k8**	1946.80403800813	0.342994926351556	0.410882866934948	0.834775440772661	0.40384415615831	0.69583076747802
**Ndufa3**	1680.80261929977	0.11225745545432	0.134466980275115	0.834832872907867	0.403811814256776	0.69583076747802
**Dennd5a**	677.111384155355	0.135067897610647	0.161795192282623	0.83480785618593	0.403825901795181	0.69583076747802
**Zfp992**	511.688788019366	0.156432978531952	0.187394241247249	0.83478007376733	0.40384154711097	0.69583076747802
**Sf3b1**	74907.5986661655	-0.0592839789089327	0.0710695625786057	-0.834168338145634	0.404186130197317	0.696201892513204
**Mia3**	7387.45598184097	-0.0576497534573227	0.0691113328429082	-0.834157743540587	0.404192099556438	0.696201892513204
**Gm42937**	21.1939828544437	-0.379448876817471	0.454838596337562	-0.834249511525314	0.404140396117954	0.696201892513204
**Ptpro**	45.7473787482825	0.418418367023357	0.501653624514427	0.834078229631775	0.404236902067998	0.696202958343584
**Gm38160**	40.0720508738807	0.665438859996643	0.797922515661848	0.83396425960569	0.404301124233857	0.696237466071353
**Kcnab2**	15219.1445138077	0.0663609238987032	0.0796045845645092	0.833631935418571	0.404488423927154	0.696331704995101
**Slc37a3**	2925.84835015218	0.0897918766193561	0.107694647966743	0.833763592848963	0.404414214886012	0.696331704995101
**Hmgn3**	42.0038055148119	0.335540977428303	0.402477460891262	0.833688864676466	0.40445633460847	0.696331704995101
**Hmgb1-ps4**	69.1441297270183	0.218838703723508	0.262575567562927	0.833431327044787	0.40460151300131	0.696450299362206
**Mbtps1**	13231.6163058724	-0.0607289940295922	0.0728857642839573	-0.833207892188614	0.404727492320635	0.696591053686099
**Mtx2**	1957.27234866998	0.0818890347722117	0.0983039088356442	0.833019111265689	0.404833950986758	0.696622099562995
**Extl2**	772.805615120132	-0.120111203370337	0.144180866049975	-0.833059244689964	0.404811317262417	0.696622099562995
**Tnfsf9**	139.935118279136	0.292307152807339	0.35094101614145	0.832923879976235	0.404887660838819	0.696622519080005
**Alkbh1**	6018.58484087787	0.0692817210154523	0.0831851240063865	0.832861907017573	0.404922615488594	0.696622519080005
**Triobp**	5672.30112353813	0.0462850806716	0.0556077061945986	0.832350115461081	0.405211350596152	0.696851726249371
**Ncapd2**	2502.78059263871	0.0956963874525296	0.114962469535331	0.832414159502023	0.405175212427061	0.696851726249371
**Klrc2**	332.794721734693	0.380758052447117	0.457376464526731	0.832482827556738	0.405136467203149	0.696851726249371
**Ccnb2-ps**	50.3313491322813	-0.45626227218064	0.548186451448104	-0.832312201396742	0.405232745295614	0.696851726249371
**Gm11532**	357.708157979974	0.135900780855204	0.163391997268962	0.831746861087059	0.405551843830117	0.697324356528934
**Igkv6-25**	41.6958411915357	1.41385632943777	1.70041765950793	0.831475915068366	0.405704828773003	0.697435193866049
**Amy2a4**	40.5255681994381	2.55200092338465	3.06910608196167	0.831512777738035	0.405684012883777	0.697435193866049
**Mios**	3600.35386082635	-0.0627371096765382	0.0754643649750143	-0.8313474803281	0.405777359250957	0.697483784195192
**Brf1**	9585.39595275801	0.082147692247594	0.0988267657881782	0.831229187684503	0.405844169071563	0.697522531856524
**Snrpd3**	4956.62137621525	0.0859284585988474	0.10341500992274	0.830908962471151	0.406025060187751	0.697757320686922
**Ybx1-ps2**	21.1831823245715	-0.418169589853927	0.503396056566177	-0.830696991761106	0.406144826202038	0.69788702578686
**Ttc21b**	936.72204422931	0.116139582348156	0.139840312988691	0.830515749471675	0.406247247013331	0.697986901230951
**Ripk3**	5677.18810877954	0.0606006124263346	0.072982948897626	0.830339323659555	0.406346960821074	0.698082104410996
**Leprotl1**	9439.46720760765	0.0673522589116241	0.0811760535082624	0.829706003196727	0.406705026484038	0.698460018840446
**Sec31a**	13846.9809702039	-0.0608306265948223	0.0733253673184325	-0.829598661683495	0.406765733702728	0.698460018840446
**Zkscan7**	434.556514242158	-0.15063994132346	0.181590547548751	-0.829558274683975	0.406788576055009	0.698460018840446
**Chrac1**	5234.87900548308	0.0737948554746159	0.0889245611120452	0.829859091254149	0.406618456564676	0.698460018840446
**Gm33682**	58.3076964055491	-0.259281584001223	0.312553619626632	-0.829558730789788	0.406788318083326	0.698460018840446
**Cdkl5**	89.8637844327743	0.277025359942482	0.333976297170972	0.829476110397933	0.406835049449724	0.698463703876586
**Mfsd12**	761.073987908301	-0.104322852617104	0.125827445256024	-0.82909457793438	0.407050891630493	0.698534670758221
**Pkd1l3**	402.174168937127	-0.223986422477029	0.270159491195405	-0.829089592543763	0.407053712439042	0.698534670758221
**Gm14434**	303.53022647693	0.689009039114185	0.83101455203412	0.829117898630848	0.407037696586594	0.698534670758221

**Gm46432**	27.3609994571915	-0.379814582431946	0.458105330284175	-0.829098806155207	0.407048499249069	0.698534670758221
**Strn4**	11890.0401270187	-0.0670294518267487	0.0808659064578123	-0.828896314440226	0.407163081067742	0.698646266839204
**Gm19327**	57.1611451126857	-0.380836256166671	0.459518036286463	-0.828773249564589	0.407232727868062	0.698689687828512
**Il16**	52744.0105329135	-0.0873475472767674	0.105438823737481	-0.828419212018559	0.40743312995011	0.698804458258358
**Cd207**	20.9294673936572	0.518004067118248	0.62540985134083	0.828263363628966	0.407521366182399	0.698804458258358
**Upf3b**	1441.04824362556	-0.0752392911633149	0.0908205206321307	-0.828439328905329	0.407421741260546	0.698804458258358
**Ubxn1**	5077.49449018013	0.0399772337333987	0.0482583405763693	0.828400505610722	0.407443720301396	0.698804458258358
**Rpl10-ps1**	524.944373340236	0.140328678180114	0.16941284355117	0.828323728228607	0.407487188379933	0.698804458258358
**Ptbp1**	26853.6935394191	-0.0726890626898684	0.0877784703703244	-0.828096711906735	0.407615731508614	0.698890215601875
**Pnldc1**	19.6323376163482	-0.999549029991086	1.20726088287481	-0.827947831466961	0.407700044989857	0.698958721456335
**Maoa**	65.8285082816779	0.260330089989897	0.314494660729883	0.82777268582468	0.407799246199921	0.699019278111902
**D17H6S53E**	773.86795718011	-0.159762183635005	0.193012710581512	-0.82772882238517	0.40782409238324	0.699019278111902
**Zfp683**	301.392613418191	0.165297707864685	0.199806798412038	0.82728770581575	0.408074010380099	0.699371566408443
**Isy1**	4868.28245258371	0.0625403100375309	0.0756083333899248	0.827161600229965	0.408145473239966	0.699417968661462
**Zbp1**	5372.5060918156	0.072496928256675	0.0876582003325174	0.827041029608975	0.408213806457932	0.699458998299003
**Plag1**	84.5863338064552	-0.433451774596073	0.524306276878585	-0.826714830073355	0.408398713709613	0.699547617341274
**Zfyve21**	550.685788347978	-0.121840556107244	0.147373387584022	-0.826747339561422	0.408380283364608	0.699547617341274
**Gm11714**	22.5641314454542	0.617721490535143	0.747122916909611	0.826800351795228	0.408350230623808	0.699547617341274
**Acox1**	1597.33424782829	0.0873928255183627	0.105750927988682	0.826402445638262	0.40857583657531	0.699774940969257
**Gm42735**	196.832840472964	-0.272066956212839	0.329250266162833	-0.826322661431919	0.408621081779588	0.699776370568521
**Cd247**	35376.1158569192	-0.0445421502633377	0.05392295493304	-0.826033186027154	0.408785266805915	0.699919391970232
**Gm20163**	201.889241548712	-0.17867820233286	0.216312516714275	-0.826018785444924	0.408793435571622	0.699919391970232
**Adat1**	1274.6339897232	-0.079574618426447	0.0963702326439315	-0.825717820153648	0.40896418110958	0.700135658598833
**Kcnmb4**	238.290211967252	0.235989537235994	0.285963900992308	0.825242404433214	0.409233983433882	0.700448718748592
**Gm10359**	3198.57265606619	0.0609200106961244	0.0738210572594382	0.825238935308471	0.409235952579776	0.700448718748592
**Mrtfb**	2105.39174564297	-0.094317209272823	0.114329825087377	-0.824957172817688	0.409395905533326	0.700523226423895
**Crybg2**	395.086271185252	-0.14305284967393	0.173412671602326	-0.824927315588463	0.409412857278521	0.700523226423895
**Rps7-ps3**	1892.08822858742	-0.0902617084388625	0.109400803312665	-0.825055261988314	0.409340217349373	0.700523226423895
**Crbn**	14055.992330416	0.0527147909143066	0.0639574704852011	0.824216319288363	0.409816655994305	0.700766177225829
**Ndel1**	7281.29789484306	0.0504043956815498	0.0611527541403541	0.824237540730621	0.409804600196027	0.700766177225829
**Cpeb3**	22.1453516419229	0.525161435393239	0.637171414651301	0.824207463356841	0.409821687068352	0.700766177225829
**Rasl12**	46.8216536890448	0.511425448157508	0.620312037347991	0.824464813457426	0.409675500886294	0.700766177225829
**Slc25a44**	3657.51306775429	0.100371389858139	0.121749981556478	0.824405791072569	0.409709025457534	0.700766177225829
**Gm37470**	71.4590406597317	-0.402146367694737	0.487827774465745	-0.824361360185274	0.409734263169812	0.700766177225829
**Rsl1**	941.707653367247	0.107068976722065	0.129918337919081	0.824125203854992	0.409868420625799	0.700770041645043
**1110032A03Rik**	648.4242796771	-0.118773407823372	0.144146529323009	-0.823976882282194	0.409952693605836	0.700832364217302
**Lrrc56**	501.311555720976	0.158946832456811	0.192919001815487	0.82390449339372	0.409993827083524	0.700832364217302
**Snord55**	27.368148930475	0.416870734563991	0.506194790271619	0.823538176559072	0.410202016610889	0.70111217873281
**Rita1**	656.402834683749	-0.109846949640946	0.13341293038932	-0.823360594212235	0.410302964923443	0.701132612330408
**2700081O15Rik**	20.0413516781212	-0.560424765397395	0.680642870322788	-0.823375649452729	0.410294406061323	0.701132612330408
**Usp2**	739.721663630175	0.170182658408334	0.206761370465322	0.823087301198157	0.410458349680325	0.701246038806557
**Plekhf2**	2803.90473966222	-0.0919239440866587	0.111681341997462	-0.823091327902806	0.410456059984516	0.701246038806557
**Itga5**	547.694213758245	0.139020861205591	0.169017373763158	0.82252408796979	0.410778683080831	0.7016650996234
**Il2rb**	47733.5421587368	0.0983039417604747	0.119518549771756	0.822499452580415	0.410792698104072	0.7016650996234
**Csnk2b**	5605.67622736594	-0.0574367207115391	0.0698911827088271	-0.821802099856081	0.411189538444593	0.701983212688925
**Ccl22**	92.1202070056837	0.462030050630812	0.562127690759937	0.821930778763445	0.411116294422397	0.701983212688925
**Fig4**	4634.78996780434	0.0613106416195447	0.0745849203069247	0.822024631349675	0.411062878424182	0.701983212688925
**Tceanc**	580.925485740737	-0.110429005176168	0.13437966659344	-0.821768709326141	0.411208545596924	0.701983212688925
**Slc31a1**	2080.28048748917	0.124480605469461	0.15149108964327	0.821702489318596	0.411246242072188	0.701983212688925
**Gm37598**	17.4136405401883	-0.610425181001368	0.74275558246368	-0.821838563604761	0.411168782495711	0.701983212688925
**Arhgef12**	990.721042466225	-0.0999220741058356	0.121615540288785	-0.821622581033338	0.411291733482744	0.701984818510354
**Mapk1ip1**	1986.68471564507	-0.107661349268215	0.131097343030273	-0.821232122479814	0.411514062651288	0.702288214577748
**Klhl11**	292.728720441888	-0.187751875347203	0.228691630821102	-0.820982712279821	0.411656115460115	0.702454560461883
**Zfp335os**	366.439835676076	0.132176951219919	0.161058754374822	0.820675359951637	0.411831209505391	0.702525102108526
**Gm15912**	25.2719859242492	-0.556252788151516	0.677728302894708	-0.820760747006806	0.411782561340072	0.702525102108526
**Gm7809**	24.5401271042523	-0.449067980709232	0.547179160618097	-0.820696424553087	0.411819207905613	0.702525102108526

**Tpm1**	1800.26692393242	0.0725768431927206	0.0884760606174369	0.820299216378279	0.412045553004663	0.702742636344025
**Wdr83os**	3627.80933299148	0.0579389241480405	0.0706318102558158	0.820295047489171	0.412047928995523	0.702742636344025
**Sar1a**	12722.0332339881	0.0734469259770353	0.089571834625129	0.819977912525976	0.41222869875198	0.702858707831557
**Rdh10**	298.596376457353	-0.190530716789345	0.232371283258013	-0.819940889932555	0.412249805025021	0.702858707831557
**C230066G23Rik**	321.66722025395	0.174775854271352	0.213123051345277	0.820070157442523	0.412176113478196	0.702858707831557
**2810006K23Rik**	1164.20616647636	0.0857521965893757	0.104600490353254	0.819806831686696	0.412326235877995	0.702912961022348
**Cox5a**	4470.93113196249	0.0828208417033975	0.101045391313613	0.819639971964165	0.412421379626852	0.702923058818854
**Coq8b**	2090.60073012995	0.0575712520774786	0.0702347087605719	0.819698025284583	0.41238827603077	0.702923058818854
**Mfsd8**	1508.29267183817	0.0984610520580311	0.120170288692903	0.819346055743025	0.412589002895179	0.703098423984358
**H2ac15**	19.7799383962028	-0.522846777211524	0.638160391489999	-0.819303084590949	0.412613513142308	0.703098423984358
**Gm11423**	322.751394915354	0.197038956306565	0.240652239696514	0.818770506998194	0.412917360848102	0.703540101090349
**Tmem129**	4770.78141562541	0.0634859008863142	0.0775461279275851	0.818685633737886	0.412965795228929	0.70354654940234
**Rala**	3377.75563444946	-0.0694640489170958	0.0848832210386165	-0.818348409345753	0.413158271359559	0.703722287516222
**5830432E09Rik**	696.333072642996	-0.105144899750698	0.128484239877331	-0.818348614982539	0.413158153972938	0.703722287516222
**Cd180**	39.6058935911306	0.266633758169415	0.325936774826242	0.818053618869976	0.413326571182442	0.703932855828116
**Abcd2**	75.484010472487	-0.419351635207526	0.512773889174584	-0.817810040761942	0.413465663806414	0.704093641478186
**Abca3**	4179.5980693479	-0.114701471034881	0.140284907814571	-0.817632294319885	0.413567181463513	0.704190412612986
**Itgax**	989.717475182178	0.226806109505559	0.277591089729679	0.817051115460605	0.413899217487223	0.704326741074844
**Afg3l1**	3066.73427763233	0.0728956361067088	0.0892210632517928	0.817022723669952	0.4139154421751	0.704326741074844
**Hacd2**	2513.15547288982	0.0892970424787629	0.109286462889252	0.817091523670724	0.41387612658119	0.704326741074844
**Bhlha15**	30.8728334784047	-0.599721030583955	0.733812633160567	-0.8172672470913	0.413775719889237	0.704326741074844
**Atp5g2**	2839.84774896505	-0.0781193127431669	0.0955775665668045	-0.817339419167624	0.41373448562798	0.704326741074844
**Kctd12**	325.956395236605	0.200973039832261	0.245953375173253	0.817118446496995	0.413860742198927	0.704326741074844
**Stag3**	69.5759560406203	0.678228004280904	0.830245927595406	0.816900127707	0.413985504819088	0.704369895198614
**Slfn9**	630.874086244511	0.122417045936175	0.149934936090194	0.816467790152219	0.41423263838924	0.704638203940328
**Ifi27l2a**	2149.07240734978	-0.161311543799512	0.197567884422038	-0.816486668728628	0.414221845161568	0.704638203940328
**Tm2d3**	3011.56207053358	0.100786573547562	0.123467980066252	0.81629725774635	0.414330142425693	0.704727985125393
**Gpr132**	4035.33341956743	0.0601343622310171	0.0737269137611462	0.815636504544798	0.414708064516165	0.704838202198383
**Polq**	163.662772337869	-0.207841273799376	0.254764483306085	-0.815817303504063	0.414604635127207	0.704838202198383
**Prkab2**	439.227152472389	-0.129433467890941	0.158642211313653	-0.815882902911867	0.414567111544017	0.704838202198383
**Mettl18**	713.647796021383	-0.142781550866227	0.175031414859038	-0.815748138591098	0.414644200393489	0.704838202198383
**Dpm3**	454.318290349049	-0.116759778143037	0.143125521877033	-0.815785868318797	0.414622617110869	0.704838202198383
**Crem**	785.642728381013	0.107958794406208	0.132321107630384	0.815884905587185	0.414565966023286	0.704838202198383
**Gm8228**	47.3691278746387	-0.358081935609339	0.438987695323062	-0.815699254043595	0.414672165776311	0.704838202198383
**Fundc1**	3907.66564775699	0.0700609815410364	0.0859358216769103	0.815270979830065	0.414917216281535	0.70511762046464
**Pxylp1**	3351.71753074651	0.0917690580005252	0.112575315193591	0.815179223284555	0.414969728794806	0.705130811670415
**Mtpap**	3774.64868710853	0.0721995301124477	0.0885854592602115	0.815026875916151	0.41505692627104	0.705202931556262
**Timm23**	6168.26390131978	0.177075601361491	0.217415588254285	0.814456786577729	0.415383318429878	0.705215674505361
**Bbs2**	650.290575230964	-0.075330655999133	0.0924945155876912	-0.814433758807184	0.415396505660691	0.705215674505361
**Ppp3r1**	10197.1505746289	0.0550097686867268	0.0675135351821332	0.814796152183785	0.41518900382628	0.705215674505361
**Pced1a**	2119.68244797223	0.117280353974711	0.14401779832199	0.814346249846839	0.415446621359062	0.705215674505361
**Mtg1**	2659.17131975289	0.0819106626035365	0.100518129837605	0.814884466472562	0.415138445472089	0.705215674505361
**Cfl1**	97286.680755166	-0.0392089060265526	0.0481289904149595	-0.814662964847184	0.415265258080225	0.705215674505361
**Unc13d**	15532.6132787671	-0.0704495040981523	0.0865109804166982	-0.814341760535108	0.415449192449153	0.705215674505361
**Gm12276**	21.6435505061474	-0.42580533700335	0.52273466511441	-0.814572603311386	0.415316997826249	0.705215674505361
**3010003L21Rik**	104.463353694437	-0.250390911193576	0.307488337510202	-0.814310270174941	0.415467227672353	0.705215674505361
**Bad**	1286.6801642409	-0.0980746441314339	0.120492003967113	-0.813951473146737	0.415672751289989	0.705265206859002
**Scd2**	2786.41287805035	0.114743040644	0.140933070724938	0.814166895348118	0.415549347441405	0.705265206859002
**Gna15**	1281.92210649739	0.131560042911748	0.161663276129523	0.813790528445946	0.415764962017724	0.705265206859002
**Fgfr1op2**	17588.5771643135	0.0542785103614108	0.0666927319680456	0.813859453042307	0.415725471278665	0.705265206859002
**Rtraf-ps**	103.722442527865	0.20399863831245	0.25066295187671	0.813836415733221	0.415738670389325	0.705265206859002
**Trav11d**	76.8327189087625	0.206740080358217	0.253964485241603	0.814051146409467	0.415615651122791	0.705265206859002
**Mbnl2**	7181.11551122397	-0.0795365527427356	0.0977573282140927	-0.813612178194428	0.415867159089224	0.705362629253918
**Rin2**	29.5612716801077	-0.690851690866635	0.849916415628993	-0.812846626047762	0.416305999115874	0.705423553937932
**Tra2b**	13461.9982187626	-0.0885218309713777	0.108881149782464	-0.813013374199645	0.416210390240348	0.705423553937932
**Anks1**	1148.51357095319	-0.144186353414255	0.17731937498271	-0.813144944980286	0.416134960264787	0.705423553937932

**Lcat**	19.4757749167257	-0.691100795351346	0.849852182497566	-0.813201177315709	0.416102724537244	0.705423553937932
**Arhgap10**	524.091931797967	0.123917935870575	0.152403279608185	0.813092317889468	0.416165130586446	0.705423553937932
**Cdc123**	6441.13150054186	0.0893484445483969	0.109857029446888	0.813315679463132	0.416037089635773	0.705423553937932
**Capg**	2780.9007838124	0.143048202108089	0.175864268477848	0.813401172086911	0.415988087553945	0.705423553937932
**H3c6**	131.924338591135	0.434543454347019	0.534550729650287	0.81291340605092	0.416267707714306	0.705423553937932
**Gm16712**	23.608098374085	0.709091411833689	0.872238161136029	0.812956189522993	0.416243176925462	0.705423553937932
**Gtf3c2**	9858.5602451255	-0.0631067741878686	0.0776996962748781	-0.812188170782751	0.416683666157068	0.705987583616873
**Rundc3a**	327.575735715108	0.158421115080265	0.195215662764556	0.811518465459054	0.417067993073845	0.706212110031142
**Ndufa8**	5352.2614579465	0.0658493948052613	0.0811307986389252	0.811644848934938	0.416995448803687	0.706212110031142
**Rer1**	10687.1518106459	0.0772468671541487	0.0951576599701683	0.811777708472081	0.416919195289239	0.706212110031142
**Srd5a3**	3545.03630503533	0.0575076378029723	0.0708343083947678	0.811861357952076	0.416871189655144	0.706212110031142
**Gm38250**	257.74686545199	0.166513788955694	0.20519462091713	0.811491978744133	0.4170831974238	0.706212110031142
**Lrrc70**	180.49994476889	0.165247229028355	0.203634672571156	0.811488667140481	0.417085098429257	0.706212110031142
**Gm4294**	1460.61353776987	0.136019936067604	0.167635178350837	0.811404488042081	0.417133422631946	0.70621804452687
**Dusp19**	560.860085436991	-0.120357607095256	0.148370340324446	-0.811197216586994	0.417252423813448	0.706262112696489
**Tmem184c**	2605.62995546597	-0.100774063172648	0.124230640197396	-0.811185251983917	0.41725929368621	0.706262112696489
**Espl1**	117.676848378196	0.250978527511281	0.309420302064617	0.811124951519401	0.417293918208055	0.706262112696489
**Knl1**	149.173563883836	-0.272815961505598	0.336382677940475	-0.811028567748885	0.417349265278172	0.706279924067034
**Pf4**	136.232639329364	0.289644400222422	0.357233582309996	0.810798353137689	0.417481480398095	0.706427801399568
**Gm38979**	26.00589389787	1.03883092511962	1.28141372970758	0.810691270926744	0.417542987456443	0.706456013459805
**Zbtb21**	2645.04622756749	0.0950750059795706	0.117296714807451	0.8105513111398	0.417623387149105	0.706516181158305
**Gm5454**	17.6870043356616	-0.638375717124325	0.787777844448222	-0.810349924948014	0.417739089153882	0.706636052366905
**9430038I01Rik**	374.742771662984	-0.12564561381105	0.155172462924815	-0.809715921515845	0.418103465168329	0.706797157027933
**Gimap9**	11639.9252179127	-0.0940460166596135	0.11613685814052	-0.809786127895958	0.418063106771296	0.706797157027933
**Bak1**	106.221365049985	0.217956526573113	0.26910741037984	0.809923912037471	0.417983907718508	0.706797157027933
**Hopx**	1560.77021177363	0.151847020388949	0.187517643995437	0.809774574560271	0.41806974809129	0.706797157027933
**Selenoh**	1894.04952836711	0.0749313969403108	0.0925154585677319	0.80993380025731	0.417978224257357	0.706797157027933
**Gm12289**	39.4628831077938	-0.442363155119438	0.546095861547584	-0.810046708403581	0.417913331166197	0.706797157027933
**1700037H04Rik**	2088.11608300031	0.071946552713655	0.0888734936482687	0.809538927302612	0.4182052211288	0.706817512530998
**Ndufab1**	4253.4970042942	0.0866843299440557	0.107072760119459	0.809583407090126	0.418179647827778	0.706817512530998
**Mtln**	274.009621529638	-0.139725640380669	0.172652935931588	-0.809286211246558	0.418350535699138	0.706911462843037
**Gm38155**	29.148272728771	-0.473878819699654	0.585515300162588	-0.809336356484733	0.418321699260195	0.706911462843037
**Mcoln1**	7307.25280869453	-0.0973785458382672	0.120416037930491	-0.808684187852768	0.418696825645798	0.707371871573631
**Rbm39**	35907.9704091905	-0.0534949986542664	0.0661593205062166	-0.808578417144412	0.418757683444744	0.707371871573631
**Znhit2**	2795.58684956847	-0.0765207775350466	0.0946277937043888	-0.808650128461123	0.418716421992866	0.707371871573631
**Fam45a**	3305.09372817168	0.0924679292902654	0.114397951770884	0.808300567089347	0.418917576156364	0.707566110783131
**Cdh1**	222.494014469866	0.462738398770892	0.572804615981315	0.807846839673489	0.419178757168874	0.707855138153941
**Klhl12**	2570.25583216694	-0.0673177119761745	0.0833317579228625	-0.807827815638886	0.419189710145225	0.707855138153941
**Gm14252**	76.6899373230432	0.350190597932319	0.433528046287983	0.807769188016257	0.419223465713815	0.707855138153941
**Fbxl19**	48.9959954831525	-0.445630218214846	0.551819040521071	-0.807565860348072	0.419340546483835	0.707976962492852
**Ndc80**	685.664221250712	0.123031036795073	0.152368548362815	0.807456907065332	0.419403292213429	0.708007036148168
**B230216N24Rik**	92.8319702287963	0.205953084570456	0.255106855855803	0.807320853371615	0.419481652704483	0.708063460274723
**Zbtb39**	222.395493195654	-0.300037542212433	0.371709173362753	-0.807183582525213	0.41956072294126	0.70812107019981
**Grm6**	234.699014359572	-0.28092830554233	0.348278506225969	-0.806619703829955	0.419885618138692	0.708544081034074
**Trav11n**	21.7517183857517	-0.463350647065653	0.574454437729333	-0.806592510447227	0.419901290137475	0.708544081034074
**Pex14**	2960.81924400883	0.0819484815588311	0.101609147024825	0.806506933266646	0.419950611933133	0.708545512892899
**Tnfrsf26**	11359.4435725008	-0.0726642122219832	0.0901054793215823	-0.806435000058631	0.419992072756215	0.708545512892899
**Ssu72**	8862.47136995782	0.0656067352341589	0.0813633716196022	0.806342386361393	0.420045456930162	0.708559711470781
**Ctsl**	1425.36791609874	0.090141055854531	0.111881115280832	0.805686068004139	0.420423884671151	0.708894505164616
**Sfxn3**	22244.4369598756	-0.0615201894585485	0.0763496945240346	-0.805768639181421	0.420376263804538	0.708894505164616
**Trbv13-1**	2919.182768909	-0.059298752319411	0.0735957060738034	-0.805736577347935	0.420394754290902	0.708894505164616
**Vps28**	24403.6565409012	0.057554081794053	0.0714218352463531	0.805833140460947	0.42033906650478	0.708894505164616
**2610206C17Rik**	29.3574049249927	0.394842164592714	0.490259291910272	0.805374158344312	0.420603799372713	0.709046118189348
**5430402O13Rik**	56.7900045459737	-0.292985929556266	0.36378679234871	-0.805378138289921	0.420601503388896	0.709046118189348
**Fbxw4**	1099.86337903306	0.119358677285312	0.148223127194174	0.805263520914321	0.420667627750794	0.709077857345877
**Cavin2**	69.7443014952956	0.373563648340528	0.46403250733258	0.805037669640651	0.420797942361302	0.709145794415725

**Gm12596**	36.2936714970179	-0.41640838128591	0.517245451335578	-0.805049865998247	0.420790904542712	0.709145794415725
**Recql5**	1930.90404855564	0.104978493085529	0.130495912870751	0.804458092028551	0.421132463024084	0.709263155252853
**Abraxas1**	1937.98066147066	0.112674606787954	0.14007675005093	0.804377648303428	0.421178905871346	0.709263155252853
**Rapgefl1**	54.2091892499992	0.411929995618505	0.512163988037252	0.804293166329654	0.421227683369689	0.709263155252853
**Atg9b**	31.6119855701886	0.48885290018054	0.607765938445646	0.80434402334355	0.42119831957353	0.709263155252853
**Tm6sf1**	8205.5919045695	0.0877686756470999	0.109095337536603	0.804513534940505	0.42110045573012	0.709263155252853
**Kctd12b**	27.1491096723667	-0.495115009657913	0.615403884330752	-0.804536699010842	0.421087083492997	0.709263155252853
**Zfp629**	92.4660600969227	0.282926546718514	0.351737764967659	0.804367841322151	0.421184567974461	0.709263155252853
**Ighj4**	46.5833535817667	0.350876565678168	0.436208752866743	0.80437763656099	0.421178912650866	0.709263155252853
**Gm14438**	30.4385955662614	0.366539428492598	0.455792959525498	0.804179662788523	0.421293222316763	0.709297713863152
**Ttll12**	2647.06063212688	-0.10147279385284	0.126224829591465	-0.803905176035992	0.42145174053679	0.709319136803941
**Ttc17**	9346.50300532399	-0.0609793056300435	0.0758741645222295	-0.803689978189847	0.421576043419998	0.709319136803941
**Gm10522**	32.4921250507893	-0.748839880054745	0.931432541758511	-0.803965769373875	0.421416744407059	0.709319136803941
**Gm45266**	42.5628432774823	-0.374577100976808	0.466014121518639	-0.803789163633374	0.421518749113075	0.709319136803941
**Gm45453**	17.036703250239	0.499638997297807	0.621577784192501	0.803823769131154	0.421498760379482	0.709319136803941
**Gm36279**	29.3851139965679	0.357791515167475	0.445184819413821	0.80369208374756	0.421574827100627	0.709319136803941
**4732491K20Rik**	52.5789378515787	-0.260507017641142	0.324173442926735	-0.803603821735692	0.421625815259644	0.709327137630388
**Arap1**	10621.9215857063	-0.0774942184402336	0.0964505188202881	-0.803460877018454	0.421708400790305	0.709390335353136
**Tmbim1**	533.296525482647	0.191567699129644	0.238554127342207	0.803036615899076	0.421953571191438	0.70957549853399
**Cox18**	1648.08208623655	0.0888429533909893	0.110627802118732	0.803079801726857	0.421928611310216	0.70957549853399
**Gm49741**	21.1767718337549	-0.538159407822192	0.670084087214875	-0.803122202258208	0.421904106144726	0.70957549853399
**Top1**	4289.10672791218	-0.0558561316404678	0.0695723081612601	-0.802850057971343	0.422061405022501	0.709681096888225
**Gbp11**	666.36468555892	-0.155025329566668	0.193139594758294	-0.802659494862642	0.422171570596281	0.7097905930309
**Polr2i**	590.642951136694	-0.0966841273954351	0.120632259208067	-0.801478211799666	0.422854852742107	0.709873361308942
**Rwdd1**	2515.29569379002	0.0726556656410523	0.0906778472821392	0.801250446704894	0.422986671912779	0.709873361308942
**Pon3**	50.4729575022089	0.419076516117422	0.522732445339504	0.801703662846564	0.422724396523659	0.709873361308942
**Aplf**	1027.99003812529	0.131159612701096	0.163606060682692	0.801679425284101	0.4227384203474	0.709873361308942
**Cdh13**	20.915856080809	-0.774683134850814	0.966585601744631	-0.801463557343039	0.42286333329251	0.709873361308942
**Slc25a38**	2989.61643417139	0.0565392739712843	0.0704970727809203	0.802008817401373	0.422547857788246	0.709873361308942
**Arap2**	6532.74100520054	0.116315553649214	0.145138774628193	0.801409230215587	0.422894773330869	0.709873361308942
**Slc35e2**	1669.89833669956	-0.109612048559551	0.136800775411316	-0.801253123236932	0.422985122728723	0.709873361308942
**Il3ra**	171.60792494511	-0.254363527073183	0.317227136452823	-0.801834073583459	0.422648945709767	0.709873361308942
**Gm12989**	436.783070602739	-0.132966279548505	0.165842062572701	-0.801764507060538	0.422689193337162	0.709873361308942
**Gm17324**	22.7179477098225	0.506658129572762	0.632128494692376	0.80151129687537	0.422835706738166	0.709873361308942
**Gm17529**	41.2139526235928	0.454399749776494	0.566958378461005	0.801469326566708	0.422859994624447	0.709873361308942
**A430110C17Rik**	38.6728363335199	-0.417607548363854	0.521066283918998	-0.801448033104312	0.422872317295433	0.709873361308942
**Gm44280**	254.54306462721	-0.186890183321601	0.232907379853164	-0.80242276324359	0.422308449908521	0.709873361308942
**Samd4b**	774.039218996137	0.186551912775232	0.232654332071834	0.801841560885408	0.422644614074125	0.709873361308942
**Gm47541**	17.5483753972747	0.655982684958998	0.818566680389154	0.80137965626348	0.422911888858465	0.709873361308942
**Gm49309**	50.0163748684393	-0.362156394010285	0.451953485726913	-0.801313421507966	0.422950222805246	0.709873361308942
**Gatd1**	4690.86772848799	0.0813386331541133	0.101539741945055	0.801052194894556	0.423101429838891	0.709990333330288
**Gm5931**	90.5026185785858	0.225557731590927	0.281638829757834	0.800875830171824	0.423203533758187	0.710077034946988
**4930509E16Rik**	17.6451768484928	-0.745394145648034	0.930803412723825	-0.800807276229011	0.423243226008892	0.710077034946988
**Gm13653**	45.3678705855346	-0.240112735384336	0.299887549702707	-0.800675905426454	0.423319294868538	0.710129046017626
**Zfp707**	1980.09491725833	-0.0867544341065229	0.108400068077132	-0.800317155195811	0.42352706611708	0.710401956653911
**Igbp1**	2826.47254545517	0.0642790102470828	0.0803665268827536	0.79982316942549	0.423813257060716	0.710705242159245
**Igf2bp2**	195.770371915305	-0.192632382860389	0.240849314702116	-0.799804571163668	0.423824034183856	0.710705242159245
**Ints5**	6019.46138667926	0.0910451678131056	0.113838973228713	0.799771512610077	0.423843190998948	0.710705242159245
**Mrps28**	1862.43071662848	0.0741713806186099	0.0927577395061946	0.799624710708442	0.42392826608077	0.710772258845998
**Gm10603**	56.1975004474683	-0.286407091768347	0.358218799527297	-0.799531158460382	0.423982486972665	0.71078753561318
**Alms1**	51.255990553792	-0.368103753019591	0.460502909506687	-0.799351633660516	0.424086547084773	0.710886353448379
**Rragb**	565.254487279621	0.117014095770646	0.146422600029351	0.799153243742357	0.424201559582023	0.711003507534724
**Nol9**	7480.76070382228	0.064203022548201	0.0803527535956375	0.799014590978332	0.42428195151224	0.711062615398677
**Prss36**	197.903722798646	-0.244316432913534	0.30588996702449	-0.798706918340913	0.424460374264208	0.711210348498632
**Gm15583**	214.246335964319	-0.159220888791077	0.199342492874667	-0.798730298267034	0.4244468144479	0.711210348498632
**C330007P06Rik**	1654.49573040611	0.0755921139975618	0.0947109423311135	0.79813495818982	0.424792176782699	0.71127206482352

**Zfp282**	974.042937674228	-0.12620190724041	0.158123983893402	-0.798119957093216	0.424800881179117	0.71127206482352
**1700028J19Rik**	66.7410060213031	0.305367540495768	0.38261874391203	0.798098748047681	0.424813187919918	0.71127206482352
**Endov**	2853.53227147469	0.10171172248522	0.127384355265635	0.798463220017248	0.424601728729227	0.71127206482352
**Gm12671**	3222.35464478065	0.0606496364944743	0.0759659589710664	0.798379133442841	0.424650508582035	0.71127206482352
**Gm35339**	29.0705445551327	-0.441200448390947	0.552689360479923	-0.798279250405389	0.424708456449748	0.71127206482352
**Gm30082**	23.4196576151837	-0.434041063966062	0.543758877018529	-0.798223408040606	0.424740855815979	0.71127206482352
**Zcchc8**	3679.54141228556	0.0620541371017641	0.0778360071788569	0.797242039396649	0.425310475009684	0.711955136511701
**Melk**	88.3981295311012	-0.403800949001312	0.50649845964271	-0.797240231068339	0.425311525035517	0.711955136511701
**Ints1**	3362.43376596753	-0.153651126202343	0.192776452008359	-0.797043023676358	0.425426044772768	0.71199555894684
**Gemin2**	1240.9369604374	0.107358646701979	0.134690560360055	0.797076249552958	0.425406749008513	0.71199555894684
**Pnpt1**	3367.5811755297	0.0799678541845769	0.100385986529592	0.796603758643185	0.425681193810773	0.71204672246189
**Hs1bp3**	298.518695414065	-0.190654963983256	0.239297985750528	-0.796726154569543	0.425610090620308	0.71204672246189
**Phyh**	2953.71862561429	-0.0879203174747998	0.11036927255373	-0.796601404000363	0.425682561756109	0.71204672246189
**Gp6**	47.2361255007898	-0.428327956759985	0.537663392896194	-0.796647051704117	0.42565604287771	0.71204672246189
**Gm15503**	113.652425902532	-0.295514145217675	0.370828168389498	-0.796903176209857	0.425507266102142	0.71204672246189
**Abcc10**	763.276602582884	-0.129564002827657	0.162723263656705	-0.796222985675827	0.425902439727176	0.712338896378422
**Cep78**	822.483724911247	0.117764152560524	0.148003557108308	0.795684609622897	0.426215374482092	0.712710989675722
**Fam240a**	432.201828398508	0.192162233216701	0.24149557934601	0.795717394650006	0.426196314127052	0.712710989675722
**Isoc2b**	813.709799831686	0.143802623717002	0.180844249935294	0.795173879006132	0.426512364071132	0.713131931310359
**Akt1s1**	1811.16764104191	-0.0828845411086141	0.104276292889515	-0.79485507982561	0.426697806784393	0.71336629618055
**Gm8935**	32.9721069521058	-0.432734295355283	0.54449127076756	-0.79474973904589	0.426759092931251	0.713393064642236
**Plcb3**	951.141265484636	0.118116868065205	0.148658472959253	0.794551872583678	0.426874223407932	0.713509826905568
**Yipf4**	3318.24660716901	0.0809496785214255	0.101940521072795	0.794087352796834	0.427144579777497	0.713724066818852
**Jdp2**	31.8429043023066	0.503937924955226	0.634525448724241	0.79419655424133	0.427081014191882	0.713724066818852
**Bahd1**	705.361070734322	-0.0926371788365406	0.116668518622596	-0.794020357249991	0.427183580260747	0.713724066818852
**Txlna**	9387.54710817504	0.0550071026398709	0.0692716165626875	0.794078518293161	0.427149722536569	0.713724066818852
**Ppp2r3d**	1232.67713517677	-0.100806567689804	0.126985558708938	-0.793842770112633	0.427286970068495	0.71382111825374
**Gm10175**	543.573743436897	-0.138274451063144	0.174200880172123	-0.79376436517725	0.427332621431849	0.713821702099188
**Plce1**	71.0053380171579	0.49317402368171	0.62163871486109	0.793345092401321	0.42757679170324	0.714078167129618
**Actr3-ps**	3488.92734573379	0.0458037426926922	0.0577324468826199	0.793379549386139	0.427556722057921	0.714078167129618
**Ccny**	2762.19393878896	0.202618533510244	0.25543452710647	0.793230796969702	0.427643367584401	0.714113665009263
**Glb1**	4989.33376775889	0.0591463116635111	0.0746200759787773	0.792632691506947	0.42799185613066	0.71461986617048
**Gm45758**	80.0753035084775	-0.25532792409235	0.322207505293584	-0.792433198785062	0.428108128118124	0.714738268145505
**Gm29036**	31.9110697890289	0.37055377001103	0.467689398566279	0.792307397060908	0.428181459625084	0.714784962317242
**H2-DMa**	9798.12789438285	-0.0571909169401853	0.0721916626696666	-0.792209443933693	0.428238562874089	0.714804558814303
**BC052040**	774.400255088277	0.110192224228042	0.139216757356242	0.791515520980508	0.428643222579431	0.715404221818038
**Ctdsp2-ps**	59.5153314385154	-0.224674703346126	0.283991508517924	-0.791131764884958	0.428867104612282	0.715523772578049
**Elmo1**	26073.6085328747	-0.0636333213272914	0.0804204070091065	-0.791258384455649	0.428793227659528	0.715523772578049
**Rab10os**	3687.99860550078	-0.0746215152894674	0.0943280440667741	-0.791085154237306	0.428894301739625	0.715523772578049
**Gm16283**	76.4939857769997	-0.272147976653194	0.344071805087718	-0.790962736931649	0.428965736525802	0.715523772578049
**4833407H14Rik**	150.237372998576	-0.21258991395788	0.268742838399948	-0.791053317824602	0.428912878736117	0.715523772578049
**2810013P06Rik**	2471.14940563441	-0.110448903830618	0.13965883473986	-0.790847954849034	0.429032722175376	0.715523772578049
**Gm43362**	817.564636434183	0.198369831882336	0.250818718414493	0.790889265108666	0.429008613229867	0.715523772578049
**Osgep**	5860.12224880379	0.0486048032301934	0.0614731555342481	0.790667126289207	0.429138264209575	0.715523928459576
**Chchd4**	1948.59020681124	-0.131815038556317	0.166720257780503	-0.790636004953032	0.429156429948309	0.715523928459576
**Cnih4**	1282.6501701484	0.116576401331001	0.147450390975937	0.790614392809753	0.4291690453685	0.715523928459576
**Gm10171**	1443.39011450056	0.168270154416943	0.212862834911415	0.79050978761497	0.429230108462143	0.715550023173719
**Tmem86a**	319.962754234371	-0.221110672137873	0.279776539494128	-0.790311698535088	0.429345756439974	0.715667098722592
**C6**	33.8332136570784	0.456110885183658	0.577338503858961	0.790023326237536	0.429514145753747	0.715757363616256
**Gm48114**	41.0037737822079	0.453118074581952	0.573577643804575	0.789985592144759	0.429536182671531	0.715757363616256
**Gm47210**	43.5735888437776	-0.394271747677712	0.499010106092918	-0.790107741032999	0.429464849427426	0.715757363616256
**Stx1a**	2283.35854983141	0.136956455758144	0.1734105849469	0.78978140694284	0.429655439347339	0.715804689976318
**Gm42763**	23.9911906033638	-0.717121940864956	0.90797648520754	-0.789802326985418	0.42964321987558	0.715804689976318
**Rpl10**	23858.7269954022	-0.0555250816462046	0.0703226776653513	-0.789575759763231	0.429775569347882	0.715805606800572
**Copg2**	1185.43071802381	0.0953205627043768	0.120717532873797	0.789616557223952	0.429751735721831	0.715805606800572
**Selenow**	15325.4687336593	0.0547328256495462	0.0693217933991858	0.789547167863506	0.42979227301772	0.715805606800572

**Gm20522**	59.7211118021763	-0.239495078461201	0.303476944985548	-0.789170585833486	0.430012311142463	0.716088749247717
**Snhg5**	1161.37896240562	0.0939385024987656	0.119055641087577	0.789030252079063	0.4300943253469	0.716088749247717
**Gm43484**	62.2107093229455	-0.312543735089365	0.396114906325735	-0.789022907490263	0.430098617939988	0.716088749247717
**Rpl34-ps1**	1027.55347935472	-0.0674793927451634	0.0855497341116991	-0.788773845364126	0.430244198657179	0.716255450421677
**Phospho2**	3254.42966824037	0.088773655574728	0.112620172276017	0.788257145950333	0.430546308781349	0.716398512066418
**Ubfd1**	4635.39452982315	-0.0760514455645419	0.0964828366077415	-0.788238076723791	0.430557460764675	0.716398512066418
**Zfp300**	491.259289026374	-0.198501364987923	0.251819167676206	-0.788269482500871	0.430539094262045	0.716398512066418
**Gm2026**	213.925864866804	-0.143421004187488	0.181947581894887	-0.78825452195536	0.430547843324895	0.716398512066418
**Tmppe**	2796.74174639006	-0.0801493647095577	0.101674832651868	-0.788291090519784	0.430526457878522	0.716398512066418
**Fam114a2**	6835.34896639424	0.0617301099508125	0.0783408681275588	0.787968163057629	0.430715328495433	0.716585516957294
**Trim30c**	1002.70710702382	-0.114949199407453	0.145910692019375	-0.787805182859313	0.430810668991502	0.716592812340241
**D830035M03Rik**	123.264761420558	0.278380952680222	0.353341522470149	0.787852360894664	0.430783069427048	0.716592812340241
**Eny2**	2363.54881614158	-0.0861322516162592	0.109391313051322	-0.787377436230695	0.431060951331374	0.716782124327614
**Gm12185**	2186.40012995798	-0.162663187972442	0.206586694918938	-0.787384628212714	0.431056742474858	0.716782124327614
**Trav6d-3**	86.0455352425534	-0.260853555190961	0.331241814785166	-0.787501890001851	0.43098812246691	0.716782124327614
**Lmbr1l**	2830.12591720937	-0.114726821639671	0.145905383609756	-0.786309722104042	0.431686058142065	0.716913703430072
**Rplp2**	5307.4561343849	-0.0766659292956197	0.0974503390224438	-0.786717933099881	0.431447003816052	0.716913703430072
**Mapre1**	17552.1283270414	0.0374395116099872	0.0476083045345484	0.786407161019945	0.43162898951507	0.716913703430072
**Gbp3**	2768.72963691893	0.122498864806209	0.155733091053459	0.786594961787272	0.431519009536125	0.716913703430072
**C2cd2l**	1663.56285726806	-0.0840996368375328	0.106923953811871	-0.78653691562419	0.431553000824533	0.716913703430072
**Fcrla**	167.846528740834	0.211395353994431	0.268692253204181	0.786756415466099	0.431424471938814	0.716913703430072
**Leng9**	1207.03489719551	-0.112259274645855	0.142699969540186	-0.786680438738579	0.431468957860924	0.716913703430072
**4930473A02Rik**	31.8022465893072	-0.29742297829456	0.378027302085113	-0.786776448828013	0.431412742440204	0.716913703430072
**Lrrc51**	225.018040967398	-0.245116320386564	0.311716721402207	-0.786343187763391	0.431666457274683	0.716913703430072
**Gm15720**	26.9855227405089	-0.379587119982772	0.482730998341474	-0.786332597837977	0.431672659747928	0.716913703430072
**Gm26935**	45.3427245330768	-0.372566381641818	0.473741843563075	-0.786433342766805	0.431613655972949	0.716913703430072
**Gm40466**	173.534329910012	-0.232738456387401	0.295726870665802	-0.787004765117932	0.431279076709423	0.716913703430072
**Gm10524**	113.721607872602	-0.171112544671431	0.217688386714544	-0.786043515016775	0.431841994246735	0.717097091721551
**4930578M01Rik**	29.1503314050013	-0.495335871884514	0.630312744533086	-0.785857300492061	0.431951092512061	0.717202672783197
**P2rx5**	20.4272872102983	-1.01682811042694	1.29587654111505	-0.784664339669272	0.432650396074623	0.717456566085859
**Saal1**	1600.05907813015	0.0799949873606124	0.101916528348382	0.784906910164411	0.432508150241832	0.717456566085859
**Cd5l**	100.111153555714	0.202286862969201	0.257593798196421	0.785293995373884	0.432281215582249	0.717456566085859
**Cwc22**	4417.87676004513	0.0856719695861323	0.109098417796133	0.78527233773659	0.432293910883891	0.717456566085859
**Fam210b**	1583.18035816841	0.0793521908951357	0.10106916956572	0.78512756398515	0.432378780092954	0.717456566085859
**Gpr83**	1898.01681439083	0.230391117469546	0.293373206414125	0.785317515139151	0.432267428980599	0.717456566085859
**Ccdc40**	103.440640079747	0.268083221385361	0.34157934897386	0.78483439408943	0.43255067157633	0.717456566085859
**Itga1**	381.552931003923	0.578286926492849	0.736420730219241	0.785267039292452	0.432297016765553	0.717456566085859
**Clec4a1**	60.0280929381532	-0.363348905887132	0.462891135895132	-0.784955419775955	0.432479706955969	0.717456566085859
**Gm5537**	110.770537721914	0.19551922489663	0.249143748836056	0.784764722414478	0.432591527316265	0.717456566085859
**Gm14296**	224.175995508614	-0.202440935221425	0.257975889498143	-0.784728121745198	0.432612990985449	0.717456566085859
**Fancf**	32.356981158458	0.462377371089954	0.588932617544875	0.785110821366118	0.432388595566565	0.717456566085859
**Eif4a2**	19063.951840085	-0.0676237535221069	0.0862086868018899	-0.78441925089879	0.432794146150216	0.717619421328943
**Pdgfrb**	17.6237417839649	-0.943742457823422	1.20388656807583	-0.783913105145618	0.433091099490613	0.717923513543676
**A230072C01Rik**	212.570416739638	-0.151213022843313	0.19290483277214	-0.783873688752663	0.433114229849983	0.717923513543676
**Trav7n-4**	142.840508950964	0.169086473597168	0.215685747106355	0.783948294524031	0.43307045023515	0.717923513543676
**C1qc**	433.323596003749	-0.120895346980278	0.154252240300486	-0.783750996061852	0.43318623304789	0.71796733713428
**Mrpl30**	3749.25562997667	0.0539817193629262	0.0688892609382116	0.783601371646934	0.433274050716725	0.718037359817357
**Klf16**	1398.2268211126	0.340118252434018	0.434390163562614	0.782978715826728	0.433639610892559	0.71849204509296
**Gm4824**	44.6669606782266	0.230650559437233	0.29455933181761	0.783035994867243	0.433605975019104	0.71849204509296
**Gm38111**	24.873348089074	-0.53999162118881	0.689850956219902	-0.782765634112824	0.433764751748567	0.718623824340465
**Kiss1r**	33.6522128622607	0.521300585325223	0.666337488919739	0.782337169968257	0.434016447835102	0.718965219568723
**Tppp3**	107.972642017051	0.528575959320033	0.675734071626907	0.782224815225649	0.434082463237336	0.718998988040651
**Pck2**	1097.03382777433	0.118131678714636	0.151047668690911	0.782082105195342	0.434166322625086	0.719062302459899
**Gnl3l**	3656.41588364282	-0.101082804155909	0.129268141096606	-0.781962232135508	0.434236769766339	0.719103392665077
**Zcchc9**	1439.68080671598	0.07839053656699	0.100292538228722	0.781618831784041	0.434438616244525	0.719286463818954
**Elob**	4337.40498235187	0.0860319305252835	0.110065662542346	0.781641872115965	0.434425071726577	0.719286463818954

**Crtc1**	2110.9445304248	0.0892439040547251	0.114208694641442	0.781410770299987	0.434560938571727	0.7193685431717
**Zbtb45**	1332.88518602917	-0.127609031013989	0.163328780472077	-0.781301560234242	0.434625152693697	0.7193685431717
**Gm10273**	1461.07279438383	0.158370433706076	0.202685634729378	0.781359931686969	0.434590830344325	0.7193685431717
**Cnot3**	3685.48114987457	-0.0913537087695851	0.11695726609096	-0.781086219119877	0.434751786568734	0.719502562857215
**Gm8337**	25.6930390921257	0.364343564065165	0.466508567464346	0.781000799289738	0.434802024605409	0.719510134604854
**Spata1**	203.444121216341	-0.230088097659188	0.294658080904051	-0.780864712595855	0.434882068310575	0.71956702198569
**Uhrf1bp1**	222.467461532595	-0.190854318011824	0.244465492421371	-0.780700442101087	0.434978700543316	0.719651342341562
**Erh**	7907.04103156767	0.0897349303626844	0.115086176325905	0.779719452217875	0.435556026485197	0.719806467230774
**Ifngr2**	2961.49667453801	-0.0783023887829823	0.100439991758435	-0.779593739626195	0.435630041999678	0.719806467230774
**Kat5**	3481.95054912518	0.0666504293313739	0.08546674374977	0.779840513481049	0.435484756379756	0.719806467230774
**Tspan2**	75.7348155806054	0.355852880461193	0.456073462610853	0.780253423262266	0.43524172243357	0.719806467230774
**Crtc2**	11896.1760666467	0.0628794891121216	0.0805884372257748	0.780254479137743	0.435241101057758	0.719806467230774
**Gabrd**	59.5229871904137	0.637923781393704	0.817686896209372	0.780156542010135	0.435298738595111	0.719806467230774
**Acbd6**	2775.37862595421	-0.0470407588405565	0.0603125158800396	-0.77995020028877	0.435420188351762	0.719806467230774
**Tpp2**	9984.77603980164	-0.0487480744638596	0.06250883352119	-0.779858969010106	0.435473891996569	0.719806467230774
**Adap1**	36.2995845097312	0.421923356755164	0.541455609664518	0.779239053440751	0.435838908877241	0.719806467230774
**Lyrm9**	1553.31594657766	-0.112149500983301	0.14379158113673	-0.779944834716428	0.435423346710902	0.719806467230774
**Rpl30-ps10**	173.693663897172	-0.352472823235493	0.452129012611488	-0.779584617230416	0.435635413254041	0.719806467230774
**Gm16213**	60.5668963180101	0.40222634637146	0.515565709064583	0.780165048411074	0.435293732269946	0.719806467230774
**Gm28913**	41.9743986653167	-0.338437304465199	0.434221261472956	-0.779412098148209	0.435736999449443	0.719806467230774
**4833445I07Rik**	69.8693269753221	0.212936596043936	0.273239989311779	0.779302460742545	0.435801565476241	0.719806467230774
**Gm43305**	1665.43582593966	0.429262507070202	0.550321092262524	0.780021905584944	0.435377981433962	0.719806467230774
**Gm39302**	20.5671498283469	0.404427841672384	0.519015028679915	0.779221832363937	0.435849051459594	0.719806467230774
**Gm50228**	38.6813726203208	-0.349911891003478	0.449008102497666	-0.779299725454948	0.435803176371579	0.719806467230774
**Herpud2**	6393.55926803756	0.179429349236454	0.230345107935738	0.778958801619141	0.436003983831421	0.719911429658567
**Ddx55**	2293.31601784932	-0.0563014768400128	0.0722763260942959	-0.778975355866298	0.435994231986525	0.719911429658567
**Slc25a15**	1028.28055468225	0.0687957488205096	0.0883443652933424	0.778722543221373	0.436143173616448	0.720065799106703
**Gm45235**	24.8420553249676	0.589895119609437	0.757617491620126	0.778618664608676	0.436204380993622	0.720091401918964
**Faap24**	1206.34315459436	0.129489399721236	0.166444516462617	0.777973359971393	0.436584718415222	0.720568284075484
**Kif14**	24.9211169560236	0.705797577680984	0.907220459629135	0.777978020876545	0.43658197062996	0.720568284075484
**Cep170b**	259.672936493331	0.205253151895638	0.263895890451659	0.777780781445084	0.436698259673236	0.720604720669303
**Kif5a**	19.8929526613034	-0.636023035531364	0.817740429757088	-0.77778108111922	0.436698082976885	0.720604720669303
**Lmntd2**	436.954707447142	-0.146759054819339	0.188723395819556	-0.777641024219698	0.436780668976627	0.720665235713582
**Slc25a39**	6247.07067033218	0.0643562443253744	0.0827953912297167	0.777292592854804	0.436986164159492	0.72085332795866
**Txk**	25048.9329950303	0.0825575031274048	0.106202136318075	0.77736198149673	0.436945236218458	0.72085332795866
**Tmem138**	1416.56448795386	0.100628396971928	0.129538351296035	0.776823203052519	0.437263085283154	0.720935625848459
**Plscr4**	29.2622102846234	0.804187667491626	1.03534651770208	0.776732865511051	0.437316392392485	0.720935625848459
**Atp1b3**	97347.3701788585	0.0443582162257926	0.0571096370959069	0.776720330953947	0.437323789181147	0.720935625848459
**C77080**	37.1590434868727	-0.430985560295128	0.554876009428347	-0.776724084249281	0.437321574310145	0.720935625848459
**Gm7206**	57.2731393922103	0.27224403714763	0.350529467991491	0.776665193678494	0.437356327186991	0.720935625848459
**Gm12764**	57.6988711381829	-0.264991918245457	0.341084642637443	-0.776909555928412	0.437212132974512	0.720935625848459
**Gm13556**	32.5828759059708	-0.516568570153956	0.664861689257999	-0.776956438459342	0.437184471480521	0.720935625848459
**Hmox2**	5906.82894768325	0.0501315397641745	0.0645956324448194	0.776082497636341	0.437700277135923	0.720981839414953
**Slc52a2**	3228.45933592539	0.0821032055876485	0.105801913784061	0.776008700137684	0.437743848933608	0.720981839414953
**Mrpl51**	3421.66876487745	0.108614564056333	0.139941935880227	0.776140213961976	0.437666201784102	0.720981839414953
**Tug1**	3801.2652084964	-0.0848074131666097	0.109274368649437	-0.776096116726884	0.437692236373834	0.720981839414953
**Gm13192**	45.9468364119289	0.31061711491387	0.40005363716045	0.776438672370551	0.437490018192674	0.720981839414953
**Lactbl1**	29.1723121722206	-0.682619869446324	0.87971473656489	-0.775955933296988	0.43777500526083	0.720981839414953
**Fdx2**	908.199539242707	0.202442897736297	0.260814301596821	0.776195540263138	0.437633538920438	0.720981839414953
**Rps2-ps13**	629.329042555172	-0.0838908123505826	0.108150249889481	-0.775687642296815	0.437933438157042	0.720981839414953
**Gm9575**	52.4096820383821	0.230168396926936	0.296679307628712	0.775815471482046	0.437857947546081	0.720981839414953
**H4f16**	115.995882866237	0.398730116553242	0.513483491062793	0.776519836553971	0.437442113058214	0.720981839414953
**Gm37885**	17.2270626614123	-0.431734303676477	0.556538743385219	-0.775748874283932	0.43789727615293	0.720981839414953
**Gm3807**	36.5064350588611	-0.75215230976558	0.969596777762581	-0.77573722089014	0.437904158209033	0.720981839414953
**Pcbp2**	7797.81045220548	0.042590078068964	0.0549223015859747	0.775460547702905	0.438067569260012	0.721022153722513
**Gm15559**	94.2399890667562	-0.292121474576076	0.376723189833375	-0.775427376013888	0.438087163756787	0.721022153722513

**Gm5525**	37.3104581262499	-0.255663263491983	0.329744991040825	-0.775336306656226	0.438140960962941	0.721022153722513
**Gm50196**	155.063287263413	-0.238750183436543	0.307924655021268	-0.775352605071695	0.438131332755866	0.721022153722513
**Nrros**	17991.5394182763	0.0556052768027134	0.0717384390626431	0.775111328449145	0.43827387813968	0.7211655699506
**Axin2**	286.770769399826	-0.164890762252767	0.212850005394391	-0.774680564124205	0.43852843848246	0.721370947088064
**Timmdc1**	482.125663303517	-0.11895869021109	0.153560924780785	-0.774667711730112	0.438536034915058	0.721370947088064
**Gm32687**	991.476694567907	0.102012002016346	0.131671181094811	0.774748135227036	0.438488501694456	0.721370947088064
**B430319F04Rik**	47.6117983458405	-0.467617483372055	0.604013046997873	-0.774184408261138	0.438821747266002	0.721765581593987
**Gm7407**	42.3600851278945	0.270819868918211	0.349959997749412	0.77385950011387	0.439013881866641	0.722006234899557
**Proscos**	25.7655578842547	0.460628830088861	0.595634404608616	0.77334154394848	0.439320275413016	0.72243472966843
**Tmem101**	595.493881313145	0.0888319490655354	0.114899758828543	0.773125635521071	0.439448030867541	0.722535756586447
**Septin6**	15876.9359271724	-0.0848004208245067	0.109712122746306	-0.772935740388467	0.439560411589578	0.722535756586447
**Tanc2**	359.172538254201	-0.147446446419775	0.190763552170312	-0.77292776708276	0.439565130585624	0.722535756586447
**Gm45640**	32.2474445845561	-0.414962063187255	0.53686374523911	-0.772937392154202	0.439559433996686	0.722535756586447
**Cactin**	5940.61338959474	-0.0622898816924756	0.0806669964799024	-0.772185458869721	0.440004592614122	0.723182681320614
**Slc16a1**	8449.62841964237	-0.0769743211254818	0.0997109446408194	-0.771974645338685	0.440129444590518	0.723312438299206
**Vamp3**	3254.88047874884	0.0867419630426278	0.112426478696321	0.77154389293762	0.440384616100955	0.723656314099775
**Ccl3**	97.2177694963956	0.430522840075053	0.558228289700981	0.771230781416803	0.440570152086863	0.723659323093161
**AA465934**	43.5203334933354	0.346860958381565	0.44962574649732	0.771443719768464	0.440443969398497	0.723659323093161
**Gm37954**	238.160903244013	0.156460738013425	0.202845791433112	0.771328490022029	0.440512249495339	0.723659323093161
**Gm5628**	16.9046786577352	-0.523778780405266	0.679145763488947	-0.77123175695668	0.440569573955652	0.723659323093161
**Ypel2**	136.716026786836	-0.24439044933909	0.316915985166123	-0.77115216896044	0.440616741375928	0.723660412118042
**Jag2**	106.552219590126	-0.314464705246434	0.407934443681136	-0.770870687968278	0.440783582875354	0.723775888156979
**Srfbp1**	1762.27776599403	-0.0871808889959694	0.113104265521713	-0.770801070974932	0.440824852360381	0.723775888156979
**Gm6988**	19.1121188062952	0.423784250950334	0.549692845476058	0.770947365311474	0.440738130524047	0.723775888156979
**Smoc1**	102.530489828992	0.316577013629922	0.410899859382762	0.77044809434949	0.441034133676996	0.723837550087413
**Tns2**	24.2412514594272	0.531840764646027	0.690317289491046	0.770429442725012	0.441045193886572	0.723837550087413
**Sema6c**	50.1909270726553	-0.328310581886236	0.426183464830297	-0.770350351384389	0.441092095953503	0.723837550087413
**Slc39a1**	2298.72744048961	0.114615710603521	0.148782013328738	0.770359992039321	0.441086378782247	0.723837550087413
**Traj34**	37.6129118481433	-0.253627587586481	0.329230410448909	-0.770365007414283	0.441083404544925	0.723837550087413
**Rab11fip1**	2351.92315359915	-0.0848078701554038	0.110108483455964	-0.770221035596419	0.441168787846406	0.723888013130878
**Rbak**	883.198606148741	-0.103600754921377	0.134532813017807	-0.770077965348604	0.441253645846601	0.723951863557361
**Ddx18**	4176.67169529725	0.061775339251716	0.0802331781390283	0.769947553924282	0.441331003775424	0.723960493845309
**Kif4**	135.647764267965	0.341192459839752	0.443156477485592	0.769914188720947	0.441350796717525	0.723960493845309
**U2af2**	2325.97092099768	0.0652991585394342	0.0848530450592293	0.76955586560098	0.441563393597073	0.723998895827974
**Slc10a3**	2732.89207207958	0.0594080584550139	0.0772140658469494	0.769394252243757	0.441659299689853	0.723998895827974
**Ninj1**	1933.40269804398	-0.0645379166703964	0.0838680556296611	-0.769517263585776	0.441586300077708	0.723998895827974
**Tigar**	625.827883819696	0.147522928780916	0.191754403279197	0.76933267897958	0.441695842203104	0.723998895827974
**Trir**	3736.49401925707	0.0623260796790616	0.0809810853840813	0.769637494773727	0.441514956992079	0.723998895827974
**Cep68**	3903.04407708545	-0.140775982314179	0.182888951837947	-0.769734753791562	0.441457250020615	0.723998895827974
**Shisa3**	17.4120893656168	0.779152652248998	1.01263266205558	0.769432669362418	0.441636500767509	0.723998895827974
**Polr3a**	3890.24159911773	-0.0692000832566788	0.0900026115404382	-0.768867503645571	0.441971970490007	0.724225492825608
**Gm10343**	26.3352774147695	-0.453167695257077	0.589312613166993	-0.768976745333404	0.441907115699568	0.724225492825608
**2410018L13Rik**	56.7174749780204	0.346366130062042	0.450477687558874	0.768886316964977	0.441960800978341	0.724225492825608
**Gm13394**	3552.3109028297	0.0537519506693619	0.0699404749445716	0.768538542409968	0.442167301654118	0.724470226906929
**Fndc5**	31.0086240674688	0.437430006853534	0.569257853664182	0.768421558065291	0.442236776723409	0.72449882664968
**1700008O03Rik**	18.9750347890429	0.455871390599473	0.593662314131044	0.767896798816919	0.442548499408842	0.72449882664968
**Kif24**	157.271405193105	-0.200616462991504	0.261211122475124	-0.768024198550768	0.442472808607581	0.72449882664968
**Proz**	223.823417100389	-0.174297595011177	0.226982530872923	-0.767889909152339	0.442552592911288	0.72449882664968
**Rora**	1576.35459629446	0.16369476587003	0.21310883485231	0.768127543766427	0.442411414530397	0.72449882664968
**Cdc40**	6681.92639213775	-0.0500119552508905	0.0651124738988065	-0.768085625630134	0.442436316162932	0.72449882664968
**Zfp938**	1169.15108548816	-0.0882128582383034	0.114814430423083	-0.768308111735127	0.442304156589491	0.72449882664968
**Gm38257**	74.2807050816603	0.489624687510421	0.637467046263219	0.768078429121258	0.442440591357768	0.72449882664968
**Psmb7**	3655.74763802608	0.0669486622592316	0.0872029912546468	0.767733552438937	0.442645498262056	0.724538561681614
**Crtc3**	3244.63055523107	-0.118765274084161	0.154703865153294	-0.76769429106686	0.442668828667189	0.724538561681614
**Gm4833**	26.2124550863945	-0.380676266213723	0.495926482808478	-0.767606246913692	0.442721149970054	0.724548936443512
**Coq6**	1690.48224275542	0.0770597940912283	0.100426497918442	0.767325314418609	0.442888121145134	0.724663899308445

**Pold4**	3072.22918638611	-0.090613714691842	0.11811465793164	-0.76716739715985	0.442981994480625	0.724663899308445
**Doc2g**	139.629717559197	-0.203481346318311	0.26526837139763	-0.767077300796252	0.443035557029147	0.724663899308445
**Dpp8**	5631.59378317355	-0.0505499350468512	0.0659081321585106	-0.766975688603607	0.443095970213172	0.724663899308445
**Pi4ka**	4622.2144135976	-0.0948706224024511	0.123697692566369	-0.766955473737306	0.443107989454807	0.724663899308445
**Rpl17**	3143.55563580085	-0.129779135901857	0.169134435642254	-0.767313500701657	0.44289514337225	0.724663899308445
**Zfp563**	612.911137134063	0.165597388718408	0.215917784191523	0.766946499281968	0.443113325495771	0.724663899308445
**Rsu1**	4001.99682651075	0.0799137600631062	0.104223651092092	0.766752644200639	0.443228597015929	0.724777190035284
**Ccdc12**	6473.9398729673	0.0683271159309397	0.0891559657765811	0.766377385246018	0.44345178491588	0.724916461761543
**Setdb2**	2130.69417477448	0.067250516863887	0.0877457173502097	0.766425062040094	0.443423425247406	0.724916461761543
**Gm36445**	280.783920197463	0.261564375597793	0.341273856563632	0.76643543174255	0.443417257156868	0.724916461761543
**Ddhd1**	1417.33040332739	0.166326464416124	0.217117927406413	0.766065089156757	0.443637574152604	0.725035762209581
**Urm1**	666.235576712132	0.108552746967243	0.141706196201577	0.766040934532083	0.443651945923075	0.725035762209581
**B930036N10Rik**	71.0846540335281	0.350697341041654	0.457815869444049	0.766022683895875	0.443662805053296	0.725035762209581
**Trim36**	178.91345620868	0.301657933616284	0.394040082531876	0.765551391822891	0.443943276454751	0.725287272262291
**Gm6365**	25.7922723101025	-0.387364384161089	0.505983386605063	-0.765567396906334	0.443933749982833	0.725287272262291
**Gbp4**	22219.0881620886	-0.0645578734671679	0.0843307273934469	-0.765532036335601	0.443954797294523	0.725287272262291
**BC005624**	5398.6555483533	0.0554836013875651	0.0724984510852097	0.76530740391065	0.444088516266272	0.725375402634224
**Gng2**	2788.90343665203	0.0760943897440837	0.09943251971187	0.765286748888449	0.444100812922026	0.725375402634224
**Ctla2b**	617.579121372665	0.142632652883971	0.186409229886537	0.7651587476156	0.444177020882687	0.725424680560582
**Psen1**	11193.4919899109	-0.0627089227274789	0.0820049950862054	-0.764696378087188	0.444452363436269	0.725673609360843
**Ivd**	6869.71904151245	-0.0689581610172434	0.0901730279866657	-0.764731567264666	0.444431404743395	0.725673609360843
**Fbxo6**	1449.85977330072	0.0910014756903518	0.119007390444717	0.764670793555675	0.444467601950974	0.725673609360843
**Snx8**	117.815231716848	0.19740075193106	0.2582256840739	0.764450494686531	0.444598827458901	0.725812652742427
**Ifitm6**	41.8135323662405	0.468327731545133	0.6127610546676	0.764290954814651	0.444693874431319	0.725843307242818
**Gm10145**	193.939497474437	0.301093447069333	0.39396506332969	0.764264334823421	0.444709734601902	0.725843307242818
**4933417D19Rik**	24.098228196815	-0.400452380224477	0.524141390847073	-0.76401594534883	0.444857740414274	0.726009675371074
**Rpl31**	4491.58672752827	-0.0563779295507432	0.0738107924754927	-0.763816884495073	0.444976373459813	0.726128077527576
**Gm28037**	73.2194842009047	0.554456755922525	0.726164758833275	0.763541261370722	0.445140664620526	0.726320953963511
**Bcl7c**	722.517884460883	0.139725198042115	0.183044233655755	0.763341162141668	0.44525995978473	0.726430163281749
**Batf**	4656.78413241034	0.110297416612494	0.144520238951584	0.763197026331	0.445345901967487	0.726430163281749
**Gm12657**	119.745056482437	0.153134633935283	0.200639672501867	0.763232076816005	0.445325001952577	0.726430163281749
**Srpk1**	19459.3148758404	-0.0788826061859523	0.103500095845611	-0.762150078620411	0.445970436998144	0.726506821327291
**Cox7c**	1576.31663163001	0.0908035912691607	0.119183319972583	0.76188170702116	0.446130608819119	0.726506821327291
**Tpm3**	58410.7854148096	0.034860925535693	0.0457018750579455	0.762789830646833	0.445588746881313	0.726506821327291
**Hp**	67.5475209046534	0.538499390013366	0.706473839009555	0.762235429366101	0.44591950410548	0.726506821327291
**Bace1**	2861.97734714266	-0.0733514960507745	0.0961723872692038	-0.76270848768109	0.445637267550124	0.726506821327291
**Gtpbp6**	3188.06837312677	0.0982581404982637	0.128828413806996	0.762705505677257	0.445639046357574	0.726506821327291
**Igflr1**	2847.92183516436	0.0958223205032508	0.125605911156552	0.762880660798042	0.445534570711879	0.726506821327291
**Ust**	2332.91622832536	0.0671154753064592	0.0880040368967887	0.762640870499752	0.445677603149038	0.726506821327291
**Zfp65**	935.880676156435	-0.114013304693554	0.149603437912452	-0.762103507008137	0.445998229915031	0.726506821327291
**H2ac14**	78.2948231782187	0.231698210844318	0.303989023508843	0.762192687650044	0.445945009727825	0.726506821327291
**Rps8-ps5**	131.648508310544	-0.166156123666912	0.218032833805955	-0.762069275377064	0.44601865923077	0.726506821327291
**Gm16085**	35.5407140216673	-0.392626835757512	0.515240255005692	-0.762026708788843	0.446044063562694	0.726506821327291
**Isoc2a**	115.328308539238	-0.241637051674117	0.317151544456746	-0.761897761172885	0.446121026322605	0.726506821327291
**Lamtor3**	3374.13578758442	-0.109430330247671	0.143496710416933	-0.762598180332625	0.44570307013621	0.726506821327291
**Igkv3-4**	51.7284888116532	-0.618285768963225	0.811444694277649	-0.76195675851159	0.446085812650868	0.726506821327291
**Gm45053**	52.5155890139697	-0.463218316605956	0.60796760682972	-0.761912824634577	0.446112035261568	0.726506821327291
**Fopnl**	4435.67178962127	0.0674276762723497	0.0885320398107133	0.761618916908658	0.446287481188287	0.726687180023131
**Ddx23**	1206.79815880397	-0.125555280509929	0.164882090415722	-0.761485254058597	0.446367283178381	0.726742021186376
**Slc33a1**	1349.47829798753	-0.0854208177882186	0.112188207491253	-0.761406387519639	0.446414373421251	0.726743597685573
**Utp23**	1830.01707899075	0.140782177217824	0.185022819858636	0.760890885380444	0.44672224306922	0.726925799498367
**Cat**	9462.49488094685	0.0577489538301915	0.0758848604673742	0.761007577460329	0.446652541317453	0.726925799498367
**Cd209a**	284.613529916793	0.308835825430735	0.405855979614981	0.760949304538312	0.446687347745355	0.726925799498367
**Mtmr2**	2524.55709633247	-0.0810913121748689	0.106603948463739	-0.760678317674623	0.446849228529617	0.726925799498367
**Slc35e3**	859.417356447339	-0.163686778853447	0.215183382176635	-0.760685036166426	0.446845214666952	0.726925799498367
**G730003C15Rik**	62.3797021649691	-0.317983447325856	0.418000439369812	-0.760725150923898	0.446821249130648	0.726925799498367

**Gm44745**	154.759592750641	0.320730512815013	0.421536555633456	0.760860496032287	0.446740396052158	0.726925799498367
**Gga1**	3122.31592004833	-0.0653048662707687	0.0858790334215943	-0.760428519848103	0.446998481072764	0.727093534248328
**Tubb5**	53256.1055646049	0.078157890505709	0.102850313444061	0.759918836302024	0.447303101550843	0.727513931785367
**Ttc30a1**	216.98374508231	-0.227466810208991	0.299391841456476	-0.75976288833528	0.447396329912404	0.727590460360177
**Gm5846**	53.271237753635	0.318853479966844	0.419737409702328	0.759649896808033	0.447463885081329	0.727625225719969
**Gls**	3377.69665349834	0.123643877587149	0.162838837423463	0.759302139118155	0.447671838203077	0.727888262776379
**Gm10110**	51.5344012779854	-0.329151526327945	0.433602088787344	-0.759109641857317	0.447786971870504	0.727959307699441
**Klk1**	134.199008388837	0.40572615767943	0.534501031209278	0.75907460227251	0.447807931048466	0.727959307699441
**Ear2**	25.463856117477	-0.386266551378204	0.50916076837468	-0.758633766327336	0.448071667840841	0.728237779285007
**Amy2a3**	96.5592029186397	2.32436452524214	3.06364173949534	0.758693320853183	0.448036033287921	0.728237779285007
**Memo1**	2602.7634974592	0.0587001729694111	0.0773894447009258	0.758503607258844	0.448149554347715	0.728244104474658
**Ces2c**	18.0418161755335	-0.546861819929088	0.721071350694923	-0.758401813360157	0.448210472643823	0.728244104474658
**Gm3379**	192.933280936809	0.131890249439283	0.173906936232753	0.758395566596405	0.448214211156643	0.728244104474658
**Trbv31**	1209.53393449861	0.098255077653967	0.129573664748705	0.758295119957614	0.448274328083486	0.728266686010052
**Dnase1l1**	1551.62592690512	-0.0957795582919856	0.126369793677979	-0.757930795836032	0.448492413091516	0.728329807770743
**Tef**	2275.09725531193	-0.0745447355601344	0.0984259556607529	-0.757368674347136	0.448829018072692	0.728329807770743
**Riok3**	8123.59945951778	-0.0490115198264063	0.0646769671526804	-0.757789395268129	0.448577071924862	0.728329807770743
**Gm9803**	18.8069031955952	0.526974412368987	0.695332618476625	0.757873855426938	0.448526503154925	0.728329807770743
**Yipf6**	2235.73070170346	-0.0867243740394396	0.114449218466245	-0.757754183048597	0.448598155467645	0.728329807770743
**Rps3a2**	265.125539721202	-0.131341173332213	0.173402201395972	-0.757436596968508	0.448788337591197	0.728329807770743
**Zfp932**	1687.78012243687	0.0882766922154133	0.116456143024943	0.758025209511754	0.448435891130481	0.728329807770743
**Gm15448**	29.5213719557438	0.418969266923149	0.55320998723242	0.75734219662077	0.448844876782825	0.728329807770743
**Gm15518**	35.1303636303929	0.345724115746834	0.456027747343662	0.758120789273589	0.448378675197264	0.728329807770743
**Gm28731**	24.2032709404657	0.448807318871613	0.592432767015816	0.757566670615354	0.4487104392165	0.728329807770743
**Gm37238**	75.1799144311362	-0.324333729923182	0.428159225930739	-0.757507278321845	0.448746007061255	0.728329807770743
**9530085L11Rik**	37.8154950606602	0.324209002574142	0.428109547497661	0.75730383606059	0.448867853224705	0.728329807770743
**Zbtb49**	1462.78675013948	-0.0705763922563585	0.0932497851247435	-0.756853135499948	0.449137854828932	0.728692872584378
**Derl2**	4164.69820249236	0.0813659268768693	0.107550902678504	0.756534114084492	0.449329026965024	0.728702910445439
**Tmem70**	2527.80293889537	0.1002418054367	0.132496927437446	0.756559471796248	0.449313829783001	0.728702910445439
**P2rx7**	682.162264853996	0.220122175001912	0.290945468460413	0.756575368458998	0.449304302870256	0.728702910445439
**Evi2b**	11154.7370994173	-0.0656419683546121	0.0867614563996922	-0.756579834854466	0.449301626168057	0.728702910445439
**Mosmo**	491.635149304002	-0.110038361635086	0.145466100226209	-0.756453644278421	0.44937725541941	0.728706124693181
**Tmcc2**	50.7083284315699	-0.494754230237543	0.654368338869647	-0.756079108430244	0.449601766878814	0.728995167802991
**Gm6296**	21.774263832751	0.397826392607485	0.526339204362628	0.755836520080684	0.449747217769699	0.729155973906487
**C1qa**	228.148340808637	-0.2032158470731	0.268920301006882	-0.755673135543232	0.449845194745955	0.729193766977439
**Gm22204**	35.8983547055153	-0.390411679933566	0.516661335508919	-0.755643306555938	0.44986308363189	0.729193766977439
**Kit**	257.379785166341	0.23595866683695	0.312467246637827	0.755146881395998	0.450160856350357	0.729336021718439
**Zfp511**	2636.0180308168	0.0645780749795888	0.0855033064667158	0.755269914675491	0.450087046391334	0.729336021718439
**Ube2t**	802.800052545459	0.12985797839082	0.172024720976387	0.754879750153146	0.45032113697396	0.729336021718439
**Lamtor2**	5192.92192729015	0.10526243617042	0.139418589913848	0.755010047336339	0.45024295370967	0.729336021718439
**Rad52**	1915.12971078941	-0.0821029183637471	0.108728462293582	-0.755118913960705	0.450177635493052	0.729336021718439
**C130046K22Rik**	70.3159345654938	0.280145304612562	0.371009214193115	0.755089884281804	0.450195052306636	0.729336021718439
**1700052K11Rik**	350.093235770667	-0.197756164688428	0.261901160810493	-0.755079374510757	0.450201357903619	0.729336021718439
**Pcdhgb4**	62.6139388273971	0.405628151972399	0.537328857416263	0.754897389883089	0.450310552015165	0.729336021718439
**Glyctk**	1358.2281382965	-0.117395349834593	0.15561182618353	-0.754411491168643	0.450602173645797	0.729416319101791
**Plcb2**	7268.06771006934	-0.111558732812216	0.147815850081995	-0.754714279627884	0.450420436665419	0.729416319101791
**Npl**	17.234085090295	0.729548467257559	0.966895364413797	0.754526802080455	0.450532957866459	0.729416319101791
**Mapk6**	2228.85350774775	-0.0764862567974819	0.101376852823876	-0.754474563639914	0.450564313427587	0.729416319101791
**Rpl10-ps5**	239.626869590401	0.13741121511346	0.182124099754506	0.754492213269319	0.450553719289453	0.729416319101791
**Ptpn1**	16692.4870608854	-0.0390737351539698	0.0518107040654266	-0.754163369496533	0.450751129967744	0.729577844138697
**Gm4739**	418.211765783568	0.221543592146431	0.293788922068764	0.754091034428373	0.4507945605467	0.729577844138697
**Gm49867**	56.4682013351079	-0.307946663502936	0.408441140984583	-0.753956035772018	0.450875621203739	0.729634092976
**Yeats2**	3787.28560833779	0.0952693561852162	0.126392695633234	0.753756818840771	0.450995257135347	0.729677817710408
**Gm44686**	32.9200249815095	-0.395272457084473	0.52436003322228	-0.753818811581535	0.450958026650685	0.729677817710408
**Slc16a7**	19.688172422361	0.69273368443787	0.919481277774745	0.753396182371835	0.451211876192439	0.729884259212738
**Dusp10**	7297.68658932678	-0.0909427923409901	0.120711415594707	-0.75339015695362	0.451215495906028	0.729884259212738

**Gm45435**	57.5727112973609	0.365729623715835	0.485595973641751	0.753156211269684	0.451356049289465	0.730036673029573
**1110006O24Rik**	91.0584173752372	-0.210909773543518	0.280074698660732	-0.7530482923022	0.451420894807188	0.730066615978329
**Dnajc9**	17142.0394280497	0.0660227211259296	0.0877337082642522	0.75253539867562	0.45172915041348	0.730273313177281
**Zmym6**	1528.14719650091	-0.090459016710812	0.120202783234212	-0.752553429104505	0.45171831187756	0.730273313177281
**Retsat**	712.507863033101	-0.0968261223491331	0.128679830253024	-0.752457647470808	0.45177589026655	0.730273313177281
**Eno3**	1241.30353255097	-0.07047081776685	0.0936488173686173	-0.752500883054029	0.451749899015927	0.730273313177281
**Sco2**	216.051726769079	0.30690898031979	0.407879516400188	0.752450093666062	0.451780431354454	0.730273313177281
**Sla2**	19194.4118065637	0.0456682094563417	0.0607485478455585	0.751758043211903	0.452196577802716	0.730796048865374
**Ebf1**	67.6681685258936	-0.334582157757754	0.445030174513105	-0.751819038167042	0.452159891377539	0.730796048865374
**5430427O19Rik**	870.223091070065	0.095600804552068	0.127238756050472	0.751349726447699	0.452442209998856	0.730999742343061
**Spag4**	18.9213352630209	0.658716400633391	0.876691309513369	0.751366408546959	0.452432173027425	0.730999742343061
**Gm37309**	33.5728033012398	-0.41259576613852	0.54916322775639	-0.751317177270194	0.452461793937417	0.730999742343061
**Gm11201**	26.0340633424515	0.373648414335048	0.497509739821261	0.751037385658596	0.452630156552021	0.731196778428357
**Slc25a14**	654.512560433035	-0.130886666283649	0.174295623375873	-0.750946373457629	0.452684930124288	0.731210296665136
**Rps6-ps2**	25.4833153616278	-0.365069419011862	0.486489316872308	-0.750416106480885	0.453004133387827	0.731650894812627
**Kcnj10**	17.4125311720607	-0.716347249848979	0.954849479426458	-0.750220076864116	0.45312216892966	0.731766528218166
**Gm29675**	23.9778907511797	-0.486132754845453	0.648060586316827	-0.750134732939599	0.453173562590832	0.731774526106142
**Atg4d**	6543.06995407112	0.0854243246099531	0.113955855234233	0.749626462232815	0.453479708672876	0.731893861472755
**Dhx40**	1755.0638230638	-0.0667408823648113	0.0890061267202345	-0.749845935601627	0.453347499229013	0.731893861472755
**Slc6a9**	23.84651064524	1.85726344381534	2.47698269094797	0.749808809969741	0.45336986195871	0.731893861472755
**Ammecr1l**	4388.35737067424	0.0546443319974816	0.0728919610239571	0.749661982334675	0.453458310100613	0.731893861472755
**Gm19412**	27.7437443234451	-0.407301026737486	0.543242753936773	-0.749758784237536	0.453399996084998	0.731893861472755
**Ube4a**	5447.4774718524	-0.0945908906576967	0.126211409462796	-0.749463864323452	0.453577670708347	0.731902033522932
**Snhg3**	294.089865503652	0.134027706879156	0.17881569036116	0.749529902037435	0.453537882847532	0.731902033522932
**Thop1**	1079.58098930872	0.0916340834495317	0.12231726869116	0.749150830704858	0.453766300908882	0.731971824279573
**0610009B22Rik**	278.853854944573	0.198354583019849	0.264905292824397	0.748775461996284	0.453992551798202	0.731971824279573
**Gskip**	3915.62715847711	0.0544374796837544	0.0726929964967657	0.748868285903945	0.453936596911021	0.731971824279573
**Asna1**	653.622788329244	-0.0852525155675127	0.113852418806458	-0.748798457347109	0.453978689680848	0.731971824279573
**Srsf11**	16308.2907641865	-0.0361197067350907	0.0482318142839207	-0.748877214580172	0.453931214848902	0.731971824279573
**Zfp619**	132.445942495426	-0.177606795026087	0.237152766464956	-0.748913022072343	0.453909631031313	0.731971824279573
**Sft2d1**	4530.95937608358	0.0699856551211792	0.0934542873473428	0.74887580984982	0.453932061595331	0.731971824279573
**Rps19-ps2**	346.213814810377	-0.118202698892799	0.157764191060489	-0.749236554241122	0.45371464056335	0.731971824279573
**Pias2**	2310.5372342816	-0.0667716249097795	0.0892045153484663	-0.748522926770517	0.454144801444746	0.732142381457424
**Rpl4**	105984.453460041	-0.0442511111439513	0.0591483458609738	-0.748137762769597	0.454377066386912	0.732441885939495
**A530072M11Rik**	201.226313099552	0.222835064909995	0.29790342376746	0.748011090614174	0.454453467958656	0.732490107878942
**Rrm2b**	2257.62976386192	0.106004928388136	0.141739726995338	0.747884383829966	0.454529897659076	0.732538365287314
**Phb**	11030.4756296659	0.0524831103463709	0.070203994507134	0.747580115844229	0.454713462090731	0.732759257738152
**Islr**	38.8080958931111	-0.304872419249242	0.407940900187724	-0.747344576405424	0.454855591366292	0.732838400118472
**Gm50087**	27.6504136954152	-0.426899667832761	0.571165947511074	-0.747417925898819	0.454811328111149	0.732838400118472
**Cnih1**	2187.44341137463	-0.0865349160918507	0.115850549261637	-0.746953006639791	0.455091927878031	0.733144208933048
**Limk1**	4076.51052157037	0.0919380786662353	0.123201970543144	0.746238702684061	0.455523232462451	0.733539046899422
**Pik3cd**	66573.3245858632	-0.0870208752515417	0.11659244815815	-0.746368024912756	0.455445129227368	0.733539046899422
**Ppp2r3a**	111.314252655823	0.261759569213364	0.350715364903274	0.746359000511872	0.455450579205079	0.733539046899422
**Gm38220**	29.0218091126531	-0.383298720033346	0.513589169246623	-0.74631386910982	0.455477835319835	0.733539046899422
**Rps27l**	1206.26234984479	0.111719380619752	0.149726858788889	0.74615457455949	0.455574045088337	0.733545905217343
**Zbtb2**	3644.66170641637	0.0721639570149985	0.096751657310546	0.745867916074783	0.455747208169338	0.733749745491393
**Bub1b**	383.31900489467	0.278854166374302	0.373917545672789	0.745763790978463	0.455810116652937	0.733776053136528
**Depdc1b**	675.392350599469	0.0932171875418393	0.125030899373973	0.745553203316744	0.455937360762108	0.733905914141234
**Wdr18**	6902.95113055667	0.0592856851166595	0.0795677315158011	0.745097088822824	0.456213028893015	0.734053547709624
**Tecpr1**	28188.1794444639	-0.0987032813497524	0.132456360531598	-0.745175852285376	0.456165418852057	0.734053547709624
**Rps23-ps2**	90.6181634842735	0.201480410275544	0.27040970028054	0.745093131150676	0.456215421255337	0.734053547709624
**A430090L17Rik**	25.611518633971	-0.471279627517554	0.632316195524546	-0.745322721216398	0.456076648656273	0.734053547709624
**Cdadc1**	2940.63816688226	-0.0853849500282058	0.114649362206267	-0.744748582853771	0.456423723344623	0.734088864830175
**Man2b2**	5304.20573812686	-0.0685589865755702	0.0920517818001072	-0.744787175596968	0.456400388836293	0.734088864830175
**Cd300c2**	77.5328951475545	0.298572945233153	0.400811339291389	0.744921403074605	0.45631923548052	0.734088864830175
**Zfand2a**	1678.80582744911	0.068514353476507	0.0919907276079267	0.74479629912834	0.456394872531457	0.734088864830175

**Traj15**	71.4529842748463	-0.41905663438916	0.562746585736713	-0.744663130813238	0.456475392992619	0.734097036883518
**Ints7**	5370.67132118257	0.0673683141604195	0.0904816124210014	0.744552537889818	0.456542269272079	0.734129659855102
**Arhgap19**	531.968442320573	0.154944851008874	0.208218482331921	0.744145520962334	0.456788442552746	0.734450560133022
**Ighv1-26**	26.2230140607881	0.44138491733731	0.593313136280377	0.743932487496331	0.45691731986672	0.734582819012336
**Cfap410**	1160.44611707525	0.0917996295236625	0.123461390048377	0.743549294947122	0.457149188503443	0.734800713320229
**Josd1**	1488.84653228689	-0.0961523505549669	0.129371839003042	-0.743224733419038	0.457345631372441	0.734800713320229
**Sec62**	3896.48038638869	-0.0627183287244691	0.0843800554651867	-0.743283805381537	0.457309874174251	0.734800713320229
**Rtp4**	3409.45473630451	-0.132089309683367	0.177629298522501	-0.743623438149393	0.457104319523147	0.734800713320229
**Ppfibp2**	61.2480277647281	0.289083947581383	0.388988095061745	0.743169138725172	0.457379285158647	0.734800713320229
**Aurkaip1**	4869.12290723654	0.0752008519662055	0.10116811153588	0.743325647029941	0.457284547709006	0.734800713320229
**Rnf170-ps**	143.905524654344	-0.1412140617116	0.189954922551974	-0.74340827715566	0.457234534569589	0.734800713320229
**Arvcf**	141.857442610681	-0.303935485763102	0.409476658512601	-0.742253506871745	0.457933755298265	0.735055961086017
**Nphs1**	37.8923414411992	-0.284922648361013	0.383716122759358	-0.742534992567143	0.457763258645353	0.735055961086017
**Pomp**	6704.41591202729	0.0586161892266071	0.0789540722122432	0.742408688801204	0.457839756773837	0.735055961086017
**Mospd3**	5303.61744387581	-0.0638038245300653	0.0859427748342712	-0.742398935257817	0.45784566447972	0.735055961086017
**Tnfrsf13c**	346.655534886229	0.155643711774676	0.209682520995756	0.742282718824362	0.457916059882081	0.735055961086017
**Klhl21**	3132.40904009721	0.259040969622434	0.348975022531365	0.742290860083408	0.457911128305893	0.735055961086017
**Snord72**	28.0544125928364	0.273188931770053	0.367897146246648	0.742568771074129	0.457742801305993	0.735055961086017
**Gm37537**	34.9373239535322	-0.392456242990099	0.528764648446674	-0.742213467074621	0.457958010406894	0.735055961086017
**Gm36955**	38.1836033266647	-0.405048614821622	0.545509439653341	-0.742514400995556	0.457775729808494	0.735055961086017
**Esr1**	229.130273489877	-0.257906667269382	0.347669011952186	-0.741816665860491	0.458198421633241	0.735090536199431
**Ubap1**	7818.96349458059	0.0516083824812212	0.0695477972420342	0.742056319938045	0.458053213181742	0.735090536199431
**Milr1**	554.637824175113	0.117478509679197	0.158362226824081	0.741834161057229	0.458187820270573	0.735090536199431
**Gm44702**	18.1698286709529	-0.471159075252151	0.635064439232942	-0.741907507561337	0.458143376832645	0.735090536199431
**Gm8942**	17.5169038648859	0.36440478309817	0.49124866596221	0.741792921481851	0.458212809960704	0.735090536199431
**Cd63**	47.5692722441763	0.457073276547281	0.616475019123851	0.741430329483398	0.458432560496396	0.73521850949025
**Eomes**	2523.61048287514	0.0738903105383941	0.0996408914449041	0.74156613280855	0.458350249350047	0.73521850949025
**Rps12-ps4**	168.687989705265	-0.121130501929679	0.163365724414885	-0.741468275328399	0.458409560475487	0.73521850949025
**Grap**	23389.580458448	-0.0617709395204218	0.083343930751108	-0.741157022037872	0.458598238774595	0.735409367837502
**Ly6g6c**	25.3360315639293	0.626457352177598	0.845411782912032	0.741008541446817	0.458688261401787	0.73545442755965
**Gm7517**	109.018948712016	0.166241314112869	0.224360359204944	0.740956712237273	0.458719687380608	0.73545442755965
**Eef1akmt1**	2625.41516767787	0.0518520711268699	0.0699963903635519	0.740782072583417	0.458825586787153	0.735549371350612
**Csrnp1**	3295.16830878066	0.145139833104286	0.195970478786854	0.740620903733907	0.458923329814978	0.735631221555912
**Pex10**	682.722892422621	-0.106653200768184	0.144060035968624	-0.740338568230072	0.459094584120719	0.735756037631222
**Gm14327**	32.9993648635267	-0.314327261295522	0.42453647315292	-0.740401075462601	0.459056666452581	0.735756037631222
**Acaa1b**	117.485343813918	-0.355094029532031	0.479696955262991	-0.740246577836529	0.45915038983407	0.735770638931703
**Bin1**	11718.1218051366	-0.0504421696653364	0.0681520093781262	-0.740142075422447	0.459213790540192	0.735797406705492
**Pik3r4**	4522.30139090471	-0.0756311433674985	0.102196391821711	-0.740056884781632	0.459265478589759	0.73580540377619
**Zfp583**	148.724133890155	-0.241831839600265	0.326829232128629	-0.739933322442495	0.459340453840536	0.735850704673172
**Fgd3**	3697.23619505116	-0.0867168279015898	0.117212017147118	-0.73982881629575	0.459403871511151	0.735877483318208
**Alg2**	1555.82882973904	0.0741969435846317	0.100314344567775	0.739644403841987	0.459515790843633	0.735981939044839
**Ddx58**	7133.17116773846	0.0749329053531116	0.10132413509997	0.739536589966255	0.459581229814325	0.736011935987836
**Trav12d-3**	818.093627037752	-0.109948486431362	0.148693501357184	-0.73943034112331	0.459645723973679	0.736040413887526
**Zfp40**	501.248998601053	-0.126918236787134	0.171701146383005	-0.739181068156783	0.459797055161345	0.736065727215053
**Atp6v1g2**	156.9740650442	-0.162309405504048	0.219549156210889	-0.739285034409971	0.459733934872478	0.736065727215053
**Tbc1d10a**	8213.19664112505	0.0454340115966203	0.0614659668605594	0.739173463254732	0.459801672461621	0.736065727215053
**Nars2**	2184.76980399709	0.0900104189478901	0.121809186934099	0.738946061569126	0.459939750907766	0.736137191981073
**BC049715**	654.516236711026	0.136091526967518	0.184158609808451	0.738990846580949	0.45991255558544	0.736137191981073
**Nmrk1**	2013.37321868903	-0.0779189101577871	0.105491137985121	-0.738629913811119	0.460131754579031	0.736369699055635
**Zbtb48**	2050.31237519155	-0.0732544243971148	0.0991977430866314	-0.738468659847837	0.460229705020962	0.736420249528647
**Herc6**	3413.51488072411	0.0798958573231195	0.108197801817011	0.738424034327822	0.460256813946698	0.736420249528647
**Cmpk2**	1836.41944389455	0.127319257831406	0.172665653632245	0.73737454527337	0.460894610694461	0.736509658352669
**Mbip**	1097.97720121637	-0.0972179613784628	0.131864707535728	-0.737255352059398	0.460967078166784	0.736509658352669
**Spaca1**	229.404302601912	-0.145190109904025	0.19686936046721	-0.73749469983272	0.460821565186444	0.736509658352669
**Utp11**	2102.85443930281	0.0767916735296301	0.104080392574089	0.737811144159229	0.460629220298635	0.736509658352669
**Tgfb1i1**	76.5755607590641	-0.277033321283944	0.37549268824023	-0.737786193873118	0.460644384242547	0.736509658352669

**Taf1**	2569.07953372122	-0.100695683225035	0.136545530330468	-0.737451332030648	0.460847929008686	0.736509658352669
**Loxl2**	20.2389389319308	-0.500620528984844	0.678164083730347	-0.738199708588374	0.460393099947694	0.736509658352669
**Art3**	147.526818467633	0.273128722147371	0.370224474535651	0.737738158693962	0.460673579193287	0.736509658352669
**H2az2**	4962.69913525702	0.0678079979209253	0.0919303747029457	0.737601670177382	0.460756540198372	0.736509658352669
**Casc1**	31.6619799885844	-0.395939901322235	0.536893149365258	-0.737464990548556	0.460839625736079	0.736509658352669
**Ndufaf6**	895.380127602632	0.0790857033214555	0.107264407099894	0.737296792661184	0.460941882254672	0.736509658352669
**Tln2**	17.1707570376474	-0.814225919075411	1.10350283684817	-0.737855755224885	0.460602107890586	0.736509658352669
**Col23a1**	575.127835255159	0.539811734410575	0.731547425966376	0.737903948875881	0.460572819166467	0.736509658352669
**Rpl17-ps5**	1239.10037263533	-0.114228342965435	0.154797420485148	-0.737921488661981	0.46056215997201	0.736509658352669
**Il12b**	81.5242792965364	-0.297886540272937	0.404347669376632	-0.736708933508081	0.461299372978458	0.736593276223163
**Ndrg2**	2217.26600791974	-0.0590672564638008	0.080195602596256	-0.736539841980792	0.461402230120231	0.736593276223163
**Chac2**	277.795721190879	-0.179081488028344	0.243082084813035	-0.736711996550809	0.461297509870068	0.736593276223163
**Cfdp1**	3770.86896251648	0.0391647375443959	0.0531467945126443	0.736916269429535	0.461173269537695	0.736593276223163
**Ttc37**	2913.31732481128	0.101440117565839	0.137687287787373	0.73674279736333	0.461278775382341	0.736593276223163
**Abhd2**	2331.60269653973	-0.121686917950838	0.165193924747061	-0.736630709253747	0.461346954628879	0.736593276223163
**Hs2st1**	934.259481791069	-0.0780116941731109	0.105883352958058	-0.736770153132686	0.461262136686665	0.736593276223163
**4930503L19Rik**	1882.37357559401	-0.0823697737276326	0.111819027499509	-0.736634681677897	0.461344538247051	0.736593276223163
**Gm2223**	132.136583321911	0.17393671315407	0.236173821655305	0.736477531400283	0.461440136421834	0.736593276223163
**Clpb**	4186.98183402204	0.0560741035356156	0.0761756433167079	0.736115917032454	0.461660156888777	0.736689206168911
**Rmnd1**	3565.8299441815	-0.0431518626787653	0.0586209379223517	-0.736116892839954	0.46165956309012	0.736689206168911
**Ndufb7**	5215.1147221636	0.0616006265541862	0.0836940443518655	0.736021625328626	0.461717537315001	0.736689206168911
**Psma5-ps**	349.567567248266	0.116874863362951	0.158798538587247	0.735994577801092	0.461733997596254	0.736689206168911
**Traj12**	82.4800465507978	0.29427017050456	0.399668453197099	0.736285709193664	0.461556841334754	0.736689206168911
**Xkr8**	336.896955388623	0.191730412181103	0.260703963585762	0.73543343777369	0.462075563815865	0.737159528100292
**Gm20257**	487.981283073308	0.11402529736299	0.155076703903271	0.735283214647854	0.462167028634942	0.737230802814415
**Ercc1**	2829.79737182251	0.075382724006117	0.102564761563201	0.734976836656185	0.462353601185558	0.737313933942742
**Dlec1**	20.1340410150636	0.833055592430633	1.13345968089167	0.734967115703034	0.462359521564272	0.737313933942742
**Ints8**	4915.57718083646	0.0716294963992791	0.0974531523391682	0.735014667868162	0.46233056114157	0.737313933942742
**2310033P09Rik**	1793.32416828518	0.064879545145693	0.0883116368873308	0.734665865478943	0.462543013778418	0.737457276950979
**5830448L01Rik**	41.7356096926743	-0.321411634486058	0.437491800775414	-0.734668933032311	0.462541145119815	0.737457276950979
**Stk10**	50633.3578989915	0.0821904056013161	0.111921139654304	0.734359977526866	0.46272937240083	0.737530520020218
**Tubgcp2**	5092.21032242372	0.101446664477438	0.138129229344806	0.734433001317924	0.46268487971384	0.737530520020218
**Gm7019**	28.6655193465621	-0.34496268991094	0.469655935680403	-0.734500862660627	0.462643534595571	0.737530520020218
**Pik3c2b**	93.6600778870755	-0.31774274680752	0.4329833965891	-0.733845106557416	0.463043145997354	0.737732138673438
**Pclaf**	285.596728608569	0.295899869012757	0.403190625353446	0.733895706908771	0.463012303785068	0.737732138673438
**Zfp180**	5490.69140142388	0.0587115591629071	0.0799910529510232	0.733976576091015	0.46296301432175	0.737732138673438
**Gng3**	44.8248020373158	0.341819567292437	0.465751850817793	0.733909197982255	0.46300408082306	0.737732138673438
**Gm37283**	79.5093648381529	-0.316083105125917	0.430835305899762	-0.733651817289685	0.463160971307765	0.737845255777621
**Kbtbd7**	329.995422709592	0.123300524874521	0.168111198576276	0.733446230344831	0.463286311376745	0.737895725094851
**Aasdh**	1201.51438407845	-0.0828287169375654	0.112926028809356	-0.733477638511474	0.463267161555407	0.737895725094851
**Prxl2c**	5322.85518691864	-0.0654020283896458	0.0891849051167555	-0.733330694292104	0.463356758468382	0.737933337698231
**Gm7476**	20.6826437619521	0.312360354162137	0.426058037847585	0.733140385615443	0.463472810522871	0.738043564973105
**Rexo1**	8591.90890997132	-0.0679556374179657	0.0927152822519831	-0.732949690357138	0.46358911456098	0.738154171194155
**Mrpl20**	1773.54056049463	0.0872849033000817	0.119111035251212	0.732802826505475	0.463678697135503	0.738168163301192
**Rbm45**	406.53219191054	-0.172171606072786	0.234956209424969	-0.732781680867927	0.463691596138554	0.738168163301192
**Cmss1**	648.533102696903	0.131573675105023	0.179676759582486	0.732279875320326	0.463997760063619	0.738580938006106
**Klhl26**	2121.48990169957	0.11107143637621	0.151713615901981	0.732112511562381	0.464099897839814	0.738668898006257
**Lypla1**	8255.98648431233	0.0607683221615999	0.0830312879252523	0.731872570931403	0.464246349239086	0.738827363393625
**Arhgap33**	29.6048654794054	-0.395711266261609	0.54098215794003	-0.73146823874638	0.464493197700991	0.739145558086701
**Trp53bp2**	2098.05106024018	-0.104720815902265	0.143196775897993	-0.731307078986638	0.464591607547243	0.739227502789247
**Plekha8**	188.009564772754	0.213449643936009	0.292075239203126	0.730803626210724	0.464899108318159	0.739265302944564
**Hps5**	2857.35880014816	-0.0756449133420026	0.103508703068314	-0.730807276100042	0.464896878617848	0.739265302944564
**Acox3**	4743.35305354704	-0.0747792786644875	0.102344148127087	-0.73066491863931	0.464983848532347	0.739265302944564
**Marveld1**	631.374526038039	0.157978895257727	0.216234521950831	0.730590535833354	0.465029294542062	0.739265302944564
**mt-Tc**	57.0017371513144	-0.335981257153461	0.459786739436362	-0.730732812271466	0.464942369399461	0.739265302944564
**H2bc11**	50.9388534195351	0.415292118232392	0.568444070770537	0.730576919677349	0.465037613935807	0.739265302944564

**Kbtbd6**	32.7698536205447	-0.404259845573364	0.553025617946824	-0.730996598447335	0.464781230637017	0.739265302944564
**Naa80**	2045.80700743665	-0.0879591242957278	0.12032747787198	-0.730997822370301	0.464780483052989	0.739265302944564
**4930577N17Rik**	44.0368924846997	0.397807305320232	0.544095385550324	0.731135230852712	0.464696556870981	0.739265302944564
**Tnfrsf21**	176.575099563739	0.314071249063145	0.430036334132232	0.730336541671316	0.465184497154144	0.739424202729532
**Myo10**	45.2341237730267	-0.37492934719483	0.513528332060664	-0.730104502102795	0.465326309624303	0.739449216843996
**Pak2**	11360.8838876926	-0.0752323357741105	0.103026626766695	-0.730222255499781	0.46525434087777	0.739449216843996
**Rpl31-ps8**	197.654915979838	0.148118361255928	0.202883749375217	0.730065181228464	0.465350343209627	0.739449216843996
**Gm43571**	46.9025474557691	-0.255147218597675	0.349514985246808	-0.730003660408162	0.465387947162652	0.739449216843996
**Rpl5-ps2**	28.5712744054054	0.294176550951515	0.403110038811028	0.72976736530598	0.465532395753589	0.739604150019086
**Myo19**	432.253324048336	0.148323132548206	0.203269595950397	0.729686758389586	0.465581676935967	0.739607872112112
**Gm49759**	498.444892491478	-0.182501991530309	0.250178290491518	-0.72948772322232	0.465703374794633	0.739726620628934
**Olfm2**	34.7948930741847	0.535252461397274	0.734333353603289	0.728895751188274	0.466065433996562	0.740227098425947
**Hemgn**	29.9450667554883	0.455605149096883	0.625277009715731	0.728645291634845	0.466218665956881	0.74027315558636
**Rpl36a-ps3**	99.9632548352855	0.185835183598806	0.255026341240073	0.728690152927644	0.466191217617506	0.74027315558636
**Ighj2**	59.9097195843837	-0.237791946237809	0.326360248355301	-0.728617984071794	0.466235374502699	0.74027315558636
**Lrch4**	16114.9934817788	0.110455218355427	0.151711149383112	0.728062629573103	0.466575248681331	0.740738155513525
**Sgpl1**	10047.4007654012	0.0496977916338736	0.0682825188872197	0.727825985973923	0.466720115147771	0.74081886313926
**Gm13408**	123.274731972215	-0.170388559230032	0.23408169926305	-0.727902094723592	0.466673520818195	0.74081886313926
**Cep104**	2647.4575483036	-0.0878890577279748	0.120793663048017	-0.727596593316637	0.466860566612402	0.740967158351291
**Mzt2**	547.737202843853	0.117549889411704	0.161646249263651	0.727204559011917	0.467100653813015	0.741250896423876
**Mr1**	2336.59812197772	0.0779443317526464	0.107234548787079	0.726858392507526	0.46731270785294	0.741250896423876
**Fam92a**	543.305446243883	-0.114844320033049	0.157964098645863	-0.727027983051493	0.467208813721416	0.741250896423876
**Urb2**	1418.08912067649	-0.0730612729816739	0.100476810576878	-0.727145622578976	0.467136753214876	0.741250896423876
**D730045B01Rik**	16.9441059253879	-0.503036539372104	0.692083335864505	-0.726843883249615	0.467321597072716	0.741250896423876
**Gm47503**	18.5946198244938	-0.708867083267042	0.975079272413911	-0.726984054857578	0.467235723671662	0.741250896423876
**Glcci1**	1400.37456464554	-0.103725864380544	0.142745718744928	-0.726647813276215	0.467441730195601	0.741299677638823
**Gm10443**	474.07057859602	0.201241949200602	0.276948578726868	0.72664012260222	0.467446442661633	0.741299677638823
**Bcat2**	3844.94042547823	-0.051823002312553	0.0713329611468846	-0.726494477158211	0.467535691984063	0.741350558862964
**Rpl10-ps6**	1388.6749178777	-0.0815588867396677	0.11227291547952	-0.726434210702807	0.467572625117734	0.741350558862964
**Polr2c**	5222.56842906567	0.0719810784533723	0.0991210272849592	0.726193830159132	0.467719953779338	0.741360357278043
**Gnl3**	4292.35595107608	0.0580590572563266	0.0799429941716859	0.72625572581931	0.467682015618028	0.741360357278043
**Gm14760**	2385.61550787616	0.0568503877464582	0.0782808866759755	0.726235868811456	0.467694186533814	0.741360357278043
**5730480H06Rik**	660.991148201003	-0.128919539636124	0.177585305059824	-0.725958375850378	0.46786428809845	0.741514543106747
**Mettl1**	2110.45704386062	-0.07361868819937	0.101426860824497	-0.725830293878024	0.467942813159454	0.741564407819926
**Pias4**	5639.98463530767	0.0545436816489246	0.0752139892098217	0.725180012680434	0.46834160303733	0.741674235081327
**P3h1**	640.971895260046	-0.0978673424111113	0.13488471793699	-0.725562865148509	0.468106793079618	0.741674235081327
**Caprin2**	409.172519573446	-0.117961844740161	0.162612207527178	-0.725418137629338	0.468195549272916	0.741674235081327
**Nae1**	3930.96439269645	0.0494133935168788	0.0681386055952013	0.725189385448164	0.468335853780162	0.741674235081327
**Btbd10**	2170.64257121424	0.0576881443902009	0.0795401025227683	0.725271184729587	0.468285679747108	0.741674235081327
**Deaf1**	3593.36424521356	0.0518894195835608	0.0715409160337883	0.725311087141431	0.468261205490836	0.741674235081327
**Gm12346**	768.373109227782	0.0931651974838969	0.128465977684164	0.725213003188635	0.468321366827822	0.741674235081327
**Gm14548**	74.2886437423396	0.395085365008685	0.545129773704246	0.724754698911445	0.468602531684328	0.741938313077768
**Rbfaos**	119.706037803589	0.139814653608376	0.192893398527761	0.724828608316807	0.468557182723219	0.741938313077768
**Casp8ap2**	5387.0806823685	-0.0694186448266263	0.0957935992226873	-0.724668927672836	0.468655161814726	0.741947089793493
**Cnppd1**	13114.3428417439	-0.0438909317545067	0.0605908418963446	-0.72438227264762	0.468831080076096	0.742103839306969
**Gm7336**	4933.31558128893	0.0573598678478773	0.0791876062974524	0.724354107035594	0.468848367094046	0.742103839306969
**Gm10163**	1976.78436384799	-0.0571685130945861	0.0789443442978942	-0.724162238638178	0.468966138252107	0.742215693093456
**Lsm11**	1547.16949501781	-0.087880197825666	0.121405099353641	-0.723859197789378	0.469152181748218	0.742435565713235
**Pygb**	11234.9405188282	0.0873685447504705	0.120738265679436	0.723619345191166	0.469299461513054	0.742444941271204
**Fam220a**	1096.9986301884	-0.515287936449462	0.712068928453154	-0.723648955683035	0.469281278019971	0.742444941271204
**Gm6793**	193.000878295428	-0.170951697844607	0.236244706078347	-0.723621285244432	0.469298270134725	0.742444941271204
**Uxt**	827.530279967022	0.125969191427385	0.174165544382561	0.723272745329515	0.469512333829548	0.742634105618215
**Glrx**	1792.84972073422	0.0854379029526596	0.118152014605792	0.72311846088887	0.469607108352003	0.742634105618215
**Arl4d**	376.54695722503	0.20929171721687	0.289370238775716	0.723266214598824	0.469516345341083	0.742634105618215
**Zfp965**	148.497589771503	-0.189563472676677	0.262147453149644	-0.723117735454279	0.46960755400012	0.742634105618215
**Psmg3**	1449.42891588228	0.106828257714106	0.147786474752174	0.722855443255198	0.469768700354871	0.742681710229253

**Cbr1**	3955.97391491455	0.0628207743709149	0.0869084405181905	0.722838587326464	0.469779057298659	0.742681710229253
**Gm29721**	68.1381880302168	0.376897785157122	0.521412440723066	0.722840031654138	0.469778169842139	0.742681710229253
**Gm13167**	26.5918079412683	0.348568318279086	0.482548133625545	0.722349324325796	0.470079734662414	0.743082501913689
**Faim**	2741.6481091168	0.0642478984773437	0.0889880442631277	0.721983486763344	0.470304630174247	0.743363432406019
**Slc9a5**	20.3842687182364	0.616795421104161	0.854545591634794	0.721781759969291	0.470428665407308	0.743484902790667
**Gm36930**	56.8774467354135	-0.304505999062229	0.421957925822727	-0.721650146678744	0.470509599867536	0.743538237399735
**Zfp72**	280.760109149628	0.201198520902313	0.278920231102559	0.721347892574823	0.470695497525294	0.74375741621601
**Lhfpl5**	20.7384862498983	-0.409075407646517	0.567196152887689	-0.721223875662496	0.470771784325886	0.743803369660381
**Dimt1**	1673.82943014264	-0.095463135629152	0.132381531916334	-0.721121248917753	0.470834918504677	0.743828535279546
**Itm2a**	2974.66619899954	0.085763006102039	0.1190026678338	0.720681373478249	0.471105575112622	0.744181508476148
**Rnf5**	5657.30394383183	-0.054665967622402	0.0758725796410889	-0.720497021203133	0.471219033111773	0.744286117155393
**Bcl9**	1427.22753757531	-0.126988640235392	0.176349730121581	-0.720095461148943	0.471466221908375	0.744389382442126
**Mcph1**	1191.94591611581	-0.0855175858846107	0.11873627368881	-0.720231343192894	0.471382568834838	0.744389382442126
**Prkce**	275.61529129445	-0.151679836992166	0.210641913435282	-0.72008383573086	0.471473379245568	0.744389382442126
**Khdc3**	26.8836189449799	-0.548999836170737	0.762198201557679	-0.720284874785541	0.471349615423976	0.744389382442126
**Ddx31**	3990.88958222311	0.0470533194036857	0.0653580692954745	0.719931294037533	0.471567299039095	0.744399805971158
**Gm15027**	23.4863478408722	-0.288675237587277	0.400982576233803	-0.719919654112248	0.471574466154985	0.744399805971158
**9530059O14Rik**	19.8131502786941	0.445358028309182	0.618733693992337	0.719789519519359	0.471654598740255	0.744451719157588
**Mib2**	5297.02529994574	-0.0805843497669873	0.111972450307418	-0.71968014940947	0.471721951047866	0.744483451788984
**Poc1b**	2165.74695120588	0.0617310917546265	0.0858230264733784	0.719283557004075	0.471966225071363	0.74449612658419
**Dph3**	1593.7465655233	0.0833446248789228	0.115851092568945	0.719411643263726	0.471887325002346	0.74449612658419
**Csf3r**	61.5973340127745	0.537114488735649	0.746486977390423	0.719522918689485	0.47181878616837	0.74449612658419
**Gm44625**	37.071341625858	-0.454642818595836	0.632018598842007	-0.719350378974351	0.471925062387278	0.74449612658419
**Gm50210**	38.7293587349029	-0.255668113423822	0.355368503847211	-0.719445056767735	0.471866743757807	0.74449612658419
**Stk32c**	20.9261316249049	1.09668231890563	1.52578419936892	0.7187663362612	0.47228490267895	0.744688297313921
**Abhd11**	5546.29356554363	0.0745452012600103	0.103718924794902	0.718723235970863	0.47231146360916	0.744688297313921
**Akap10**	3338.18955825298	0.0471163752063428	0.0655448534112437	0.718841720656901	0.472238448365108	0.744688297313921
**Gm37780**	57.5488974194972	0.326079927080603	0.453706518277863	0.718702319548565	0.472324353833555	0.744688297313921
**Gm49085**	110.329486820398	-0.227925769502549	0.317063472081369	-0.718864800181258	0.472224226515663	0.744688297313921
**Cdca2**	319.730561522695	0.147480358057969	0.205244540333304	0.718559226074761	0.472412543662017	0.74475282145897
**Exd2**	3898.44512006206	0.0538261285326691	0.0749329863304333	0.718323547059917	0.472557814581003	0.74490731135983
**Fbxw8**	1085.57628407407	0.335110091804432	0.466586257436016	0.718216806568476	0.47262361677615	0.744936513908351
**Mrps36**	1645.02788107251	0.0992347249231345	0.138216431777073	0.7179661900344	0.472778133906209	0.745105526701354
**Gtf2ird1**	458.636899954145	-0.15497542253827	0.215940170423232	-0.717677596690445	0.47295610000808	0.745162410541678
**Acss2**	12281.1451553904	-0.0468480283187318	0.0652709679169751	-0.717746799439571	0.472913421569701	0.745162410541678
**Gm43064**	52.6977250330498	1.63791827742116	2.28213162822579	0.717714200689878	0.472933525474134	0.745162410541678
**Trmt10c**	4670.57753244445	-0.0897668342633761	0.125114047743354	-0.717480058254646	0.473077936770617	0.745279848999661
**Apaf1**	1317.22293397476	-0.0795374266557071	0.110917969701873	-0.717083326258936	0.473322683302211	0.745292847086333
**Xrcc2**	444.295908357549	0.121440571602246	0.169344525126197	0.71712133304426	0.473299233654385	0.745292847086333
**Acad12**	262.375186153565	0.120844602599553	0.168498201982254	0.717186303342747	0.473259149381223	0.745292847086333
**H2-K1**	482240.254804043	-0.0305377231589986	0.0425766585679268	-0.717240952816406	0.473225434121806	0.745292847086333
**Gm29336**	86.4054068325564	-0.22040160578854	0.307267014332984	-0.717296668719836	0.473191062303291	0.745292847086333
**Rpl6**	17548.6700403329	-0.0477796088887839	0.0666549114758354	-0.716820528763369	0.473484843118547	0.745390368768396
**Arhgap27**	8206.58366996276	0.0766368150652064	0.106930591793564	0.716696819682421	0.473561188675458	0.745390368768396
**Gm12397**	63.0643499500333	-0.213958828466763	0.298513998122527	-0.716746383125866	0.473530600385487	0.745390368768396
**1810021B22Rik**	72.2680470221674	-0.213320628441561	0.297652680580619	-0.716676322301031	0.473573839039183	0.745390368768396
**Cfap300**	17.9761124520834	-0.867774951623971	1.21105275072079	-0.716545956489092	0.473654301224078	0.745442551377126
**Gm15928**	17.303589763364	0.444616386047781	0.620674631195863	0.71634373905557	0.473779125325623	0.745564533881538
**Rpl24**	5698.24683869922	-0.051954050266137	0.0725345348873928	-0.716266401194876	0.473826868962323	0.745565206135343
**Nfkb2**	8957.26578898753	0.0624401499518821	0.0871981053109711	0.716072324383704	0.473946691690227	0.745604834361312
**Rexo5**	1715.60014725014	-0.0728643612872213	0.101753178596302	-0.716089288731756	0.473936217263305	0.745604834361312
**Gm12928**	30.4348334305004	-0.493635352207309	0.689444024797013	-0.715990471238975	0.473997232690304	0.745609902715496
**Lrig2**	2808.26832539493	-0.0937369038458254	0.130991827177308	-0.715593528739355	0.474242370608482	0.745846594837594
**Exoc6**	2328.49284287207	-0.0741753083890105	0.103644727583907	-0.715668902009132	0.474195817334728	0.745846594837594
**Lias**	2833.95511296009	0.0502758411889525	0.0702811786923072	0.715352845874447	0.47439104191297	0.746005952836593
**Gm49339**	25.4084633234231	1.04136570312714	1.45596861684626	0.715239113726795	0.474461303875548	0.746041988341185

**Higd2a**	3529.36160697113	0.0434698854761102	0.0607889148155928	0.715095599386484	0.474549972973095	0.746106957108068
**Gm48529**	178.85400259129	0.134753931821107	0.188605476263518	0.714475181159802	0.474933397719149	0.746635293610759
**Rab35**	406.464866352878	-0.115805052182535	0.162192874718107	-0.713995928512921	0.475229697090718	0.746795284491064
**Stkld1**	32.5025075028211	-0.575589967148111	0.80605132205465	-0.714086003458085	0.475174000248954	0.746795284491064
**Gm15696**	24.6683628717583	-0.551480288899841	0.772351635690642	-0.714027475848747	0.475210189738439	0.746795284491064
**Gm13690**	123.454659317117	0.332584084268273	0.465663647944961	0.714215261887013	0.475094080994204	0.746795284491064
**Gm45290**	50.9730599701061	-0.352564705378556	0.493838391359428	-0.713927291898111	0.475272140183816	0.746795284491064
**Snrnp27**	1045.57666538582	0.0947631456476651	0.132755056256436	0.713819483188768	0.475338810477875	0.746825569518384
**Tomm34**	9623.00028871462	0.0510841357418477	0.0715956596423076	0.713508835550428	0.475530947668881	0.747052955375729
**Espn**	379.349764959682	-0.136046255170123	0.190764050288626	-0.713165058952593	0.475743624941842	0.747089126789775
**Zfp358**	1284.16534236938	-0.0855074091127363	0.119887498280448	-0.71323040633238	0.475703193793416	0.747089126789775
**Gm3325**	228.189743038916	-0.210909890855455	0.295712671541995	-0.713225746315382	0.475706076935206	0.747089126789775
**F530104D19Rik**	32.1213980632075	-0.415299490936164	0.582266104661172	-0.713246894523995	0.475692992664154	0.747089126789775
**Mrvi1**	113.558139524715	-0.311835621355805	0.437384374686079	-0.71295555900829	0.475873257583628	0.747218228175907
**Soat2**	192.661337263486	0.337394104142027	0.473443895408168	0.712637986072564	0.47606979965982	0.747452354846531
**Mterf1a**	1155.43925598234	-0.109345446104147	0.153536343838276	-0.712179561989071	0.476353591273608	0.747791620051439
**Zfp493**	202.859952641786	-0.207476136728278	0.291343575498368	-0.712135616422542	0.476380801045652	0.747791620051439
**Timm17b**	2899.03334583103	0.0536838268978673	0.0753939893269295	0.712043856242694	0.4764376189324	0.74780631153679
**Gm6906**	26.8574144619876	-0.339087892977237	0.476278220931588	-0.711953387904216	0.476493640545623	0.747819750406114
**Nptn**	4880.33486278302	-0.0653757016546655	0.0918468541154827	-0.711790319703994	0.476594628010425	0.747878082480896
**Gm44135**	19.1648034605649	0.724098406204825	1.01736351820105	0.711740094126048	0.47662573486534	0.747878082480896
**Hic1**	66.9757017027328	0.498740848661591	0.700972560166905	0.711498390953903	0.476775447559462	0.747964030983119
**Mafb**	78.3703661341494	0.365036177132748	0.513033843759557	0.711524554516192	0.476759240416805	0.747964030983119
**Adamdec1**	36.3058390772821	0.823212183435001	1.15720495342214	0.711379761208727	0.476848937102551	0.747980147424672
**Fam71f2**	36.6852615792648	-0.2728988461843	0.383646690353322	-0.711328555794322	0.476880660089373	0.747980147424672
**Tfb1m**	624.185453429734	0.0875132543664921	0.123142998058061	0.710663665385428	0.477292680600348	0.748551882972
**Gm9790**	17.4190850288325	-0.439140238986623	0.618385311104589	-0.710140152265599	0.47761722920152	0.74898633365131
**Cops7b**	2339.20566249561	0.0551898702481795	0.0777366390590897	0.709959562391528	0.477729212732749	0.749012856221884
**Gm43628**	337.375041846709	0.185997308518564	0.261975323755426	0.709980260172164	0.477716377339037	0.749012856221884
**Paqr7**	5986.97959492684	-0.0981994461764132	0.138364517606035	-0.709715524438252	0.477880563141553	0.749101077737908
**Gm5148**	1517.141429727	-0.339781296306353	0.478747855522013	-0.709729124396526	0.477872127865158	0.749101077737908
**Ap1b1**	23884.9379523305	0.046772552119994	0.0659347149186617	0.709376724805643	0.478090727455448	0.749281439478367
**Rangrf**	367.955477087989	0.156900146813992	0.221162084265934	0.709435106540818	0.478054508473024	0.749281439478367
**Lrrc71**	25.9239821988439	-0.690376280302288	0.973412528257909	-0.709232992447546	0.478179902827337	0.74934666622082
**Gm4258**	17.9566097422866	-0.563518870023587	0.794848304827709	-0.708964045844868	0.478346788861246	0.749533646786659
**Rims3**	188.501434024555	0.226972857518775	0.320254988938243	0.708725438661453	0.478494875400503	0.749538404591704
**Nsd2**	471.672014214076	0.133635553625804	0.188543235404534	0.70877935948791	0.47846140838106	0.749538404591704
**2700099C18Rik**	94.1244279410758	0.250421958994624	0.353276680785446	0.708855049356375	0.478414432136169	0.749538404591704
**9930024M15Rik**	88.3383094244059	-0.284406031675297	0.40133351713834	-0.708652578292538	0.478540099650476	0.749538404591704
**Golt1b**	1613.75479577816	0.0797700373010748	0.112638195166156	0.708197047932133	0.47882289914667	0.749611832867663
**Tdg-ps**	42.3263457177967	0.257150548952324	0.363173702112968	0.70806489417104	0.478904959091307	0.749611832867663
**Mlec**	8939.80288117024	0.0522109591980176	0.0737480344936498	0.707964077368236	0.478967565736602	0.749611832867663
**Pomgnt2**	404.770096335395	0.105306409549538	0.14873739806985	0.708002230213038	0.478943872517387	0.749611832867663
**B230208H11Rik**	16.899101205397	-0.428646204112618	0.605334695124353	-0.708114382944069	0.478874228487782	0.749611832867663
**Gm43313**	106.170003554931	-0.198921647326163	0.280785997185049	-0.708445753422188	0.478668488055607	0.749611832867663
**Gm5864**	59.4712964172173	0.24047173817454	0.339526879120617	0.70825537818204	0.478786681832808	0.749611832867663
**Gm44705**	20.8590075253098	0.35740864498716	0.504785062641971	0.708041246538745	0.478919643731573	0.749611832867663
**Lrrc20**	1454.68237201746	0.121266342242884	0.171309261872234	0.707879661131966	0.479019991163338	0.74961942603642
**Nrbf2**	2150.9164495357	0.0923480407192833	0.130497528958516	0.707661221298988	0.479155664459319	0.749757279531826
**Tmem242**	1002.1011811116	0.0967192256510113	0.136718903747717	0.707431254930805	0.479298519566438	0.749831887689472
**Gm44684**	20.6922600894744	-0.904145825761605	1.27798731393301	-0.707476369995486	0.479270492255706	0.749831887689472
**1700017B05Rik**	3127.11004319363	0.0846414572876338	0.119752870515327	0.706801072269916	0.479690108135524	0.750295516566553
**Mrpl23**	4671.97480906811	-0.0513871114088771	0.0727019815863226	-0.706818580286738	0.479679226490117	0.750295516566553
**Ikbke**	29173.8676607768	-0.0756590628606349	0.107105641786168	-0.706396615518021	0.479941525029677	0.750570473932517
**Gm19337**	40.4846013846836	-0.304752998254757	0.431438403825462	-0.706365023494861	0.479961166170213	0.750570473932517
**Gmpr2**	3385.82087940962	0.0465352040324032	0.0658918399247088	0.706236221140228	0.48004124867854	0.750621212208767

**Rabggtb**	1656.17585517697	-0.0879376800053034	0.124560869311343	-0.70598158548092	0.480199589133139	0.750794297079865
**Dusp28**	627.653910456016	-0.108730885528242	0.154077872944238	-0.705687867118941	0.480382267796395	0.750930892228949
**Plpp2**	65.8804099921317	0.357192414244691	0.506156335933746	0.70569582732922	0.480377316430029	0.750930892228949
**Slc2a8**	1430.83531475686	0.0783199847260005	0.111013856957682	0.705497375483999	0.480500764655588	0.751025059114206
**Meaf6**	1349.61996365173	0.0572884965413646	0.0812200901552496	0.705348842039691	0.480593172134205	0.751025059114206
**Pilra**	72.3802224559268	-0.319522022567208	0.453083597963773	-0.705216485441515	0.480675523629192	0.751025059114206
**Hist1h4n**	122.764191362258	-0.328589758731749	0.465945463936003	-0.70521076856514	0.480679080809155	0.751025059114206
**Gm29770**	18.1650994610816	-0.403578344123487	0.572282733143051	-0.705207969331842	0.480680822565639	0.751025059114206
**Dnajc7**	24245.0960968878	0.0549170410412263	0.0778912270823165	0.705047835274045	0.480780467906762	0.751106269364154
**Ddit4**	1234.67485464561	-0.352960538179447	0.500791042522449	-0.70480601330569	0.480930965854726	0.751266900859811
**5830462I19Rik**	125.445618079239	-0.235065672045409	0.333711961927366	-0.70439690171062	0.481185634941703	0.751590211119787
**Zfp672**	3441.70095230233	-0.0807840426214025	0.114743034574059	-0.704043107464286	0.481405928553668	0.751636267956812
**Plekhn1**	3828.85283482056	0.0732323878945949	0.104005089722318	0.704123116379372	0.481356105380522	0.751636267956812
**Gm15893**	17.8245889340188	-0.421288247547416	0.598297061541321	-0.704145606970058	0.481342100539212	0.751636267956812
**Tomm20**	4093.34355086586	-0.0819350191294741	0.11637730167923	-0.704046390036703	0.481403884374076	0.751636267956812
**Gpm6b**	154.208651841299	-0.186000770370064	0.264257868721778	-0.703860858599045	0.48151942909359	0.751738991799039
**Fam168a**	2212.91247803107	-0.130750578132505	0.185843902510595	-0.703550540890362	0.481712721563266	0.751966252592865
**Farp2**	137.681098303613	0.20991669428442	0.298425794730288	0.703413371066462	0.481798176139653	0.752025147244429
**Nek2**	131.436544711062	0.38174204558359	0.542765944916968	0.703327187637	0.48185187123687	0.752034462666339
**Pycrl**	2572.86499573147	0.0645937121503131	0.0918970426610814	0.702892174545119	0.482122948303309	0.752308506279981
**Zfp868**	2780.28819826872	0.0926932940850509	0.131867510851067	0.702927457163727	0.482100958958819	0.752308506279981
**Ppfibp1**	288.658804613068	-0.142195711913589	0.202323338754991	-0.702814182429962	0.48217155761377	0.752309855759994
**Fasl**	1342.01992150617	0.37573936320349	0.535041005786669	0.702262740873556	0.482515324701293	0.752473670953907
**Clint1**	18897.5002648532	0.0700657377618646	0.0997667683537623	0.702295352631038	0.482494990920125	0.752473670953907
**Abhd14a**	1269.23936663576	0.102719203163187	0.146250026410901	0.702353399065989	0.482458799496451	0.752473670953907
**H2ac8**	117.076757497442	-0.207211030481182	0.294972931487553	-0.702474730261565	0.482383155366087	0.752473670953907
**Gm16861**	103.197674181339	0.265038741219464	0.377351228984068	0.702366179998989	0.482450830899299	0.752473670953907
**Pphln1**	3282.03645833083	0.0636051487760545	0.0905997655557264	0.702045401397116	0.482650850092973	0.752610533885698
**Dlat**	4693.7474868813	0.0632495621990904	0.090144599792012	0.701645604340407	0.48290020373779	0.752639045947768
**Araf**	20339.5387911125	0.0503197782436439	0.0717475860027734	0.701344547560093	0.483088019193192	0.752639045947768
**Mrnip**	176.452839380446	-0.164648600212772	0.234587147637057	-0.70186539148133	0.482763113717338	0.752639045947768
**Eldr**	65.0395662371491	0.354236220461021	0.50493375473768	0.701549890727849	0.48295991075041	0.752639045947768
**Gm21182**	18.1244143020483	0.405952676960578	0.578497660084378	0.701736074267555	0.482843771469428	0.752639045947768
**Rnf113a2**	1169.97280277489	0.0924381023931423	0.131778617004507	0.701465112431564	0.483012799562765	0.752639045947768
**Gm37949**	104.598491372518	-0.299215470047356	0.426491076073535	-0.701574984410144	0.482944256697841	0.752639045947768
**Gm44878**	17.1917943223543	-0.370700181008511	0.528569737490931	-0.701326910557136	0.483099023336658	0.752639045947768
**Gm49336**	3581.32747376623	0.0596705913921461	0.0850651160270534	0.701469582115998	0.483010011078322	0.752639045947768
**Gm20036**	29.4448784371996	0.446950231098373	0.637421633933392	0.701184596356323	0.483187821514151	0.752702966247996
**Rnf121**	1858.62155488887	0.0773777979746355	0.110434624868784	0.700666100569219	0.483511416397069	0.753132600649329
**Gbf1**	8571.74301865758	-0.0745008525509112	0.106402599638542	-0.700178875365793	0.483815602367048	0.75318089747185
**Tmem156**	963.028373938699	0.0899941166825628	0.128526506782177	0.700198884538911	0.483803108133298	0.75318089747185
**Ptges3**	6704.33329404522	0.0771945180403161	0.110253144485608	0.700157064911586	0.483829221565657	0.75318089747185
**Ptprv**	297.456936387587	-0.275990607243328	0.394027475524021	-0.700434930016708	0.483655728580667	0.75318089747185
**Gm6755**	50.2306072918561	0.250563074523825	0.357783553728425	0.70032026881262	0.483727316474921	0.75318089747185
**Gm7623**	65.12196254516	0.197597274035964	0.282079987486183	0.700500860755471	0.483614567803625	0.75318089747185
**Sergef**	1748.50576342494	0.0739563348481894	0.105727929426792	0.699496672725423	0.484241691656016	0.753748526664412
**Traf1**	23533.8209540994	-0.0677191010259998	0.0968506389609498	-0.699211711481885	0.484419732624895	0.753802264168524
**Dctn6**	5552.02071802288	0.0488332034177974	0.0698376465813947	0.699238960764278	0.484402706008354	0.753802264168524
**Gm20743**	41.4513546123133	0.262062543880948	0.374747787993349	0.699303777840042	0.484362206594275	0.753802264168524
**Spsb3**	4616.44103470707	0.0770369940931318	0.11027207999131	0.698608334033444	0.484796833973882	0.754240098037762
**Cd27**	39793.5958524114	0.0424513969621546	0.0607595983537489	0.698678037912595	0.484753262029412	0.754240098037762
**Csf2ra**	466.78488702807	0.109102427093409	0.156304710410111	0.698011127157631	0.485170235373286	0.754741410362527
**AW146154**	1310.35306357725	0.0824519633619973	0.118136214416293	0.697939778834034	0.48521485606222	0.754741410362527
**Zfr2**	170.720226000865	-0.191707134135356	0.274835867712422	-0.697533170364616	0.485469188260731	0.754781319861764
**Tardbp**	7620.70771948749	-0.0443157499376797	0.0635245034356077	-0.69761662887457	0.485416979364904	0.754781319861764
**Cyren**	790.635886835353	0.115640826661987	0.165807732094538	0.69743928827187	0.485527921439287	0.754781319861764

**Usp14**	5598.56881067743	0.0385015794856567	0.0552026123659808	0.697459374393371	0.485515355122463	0.754781319861764
**Gm42872**	22.1343306706806	-0.365223072809088	0.523574044281817	-0.697557636398996	0.48545388280029	0.754781319861764
**Gm9030**	388.266232942575	0.0827832208642007	0.118638969795755	0.697774272709191	0.485318370866066	0.754781319861764
**Armcx2**	1092.28368269727	-0.0807791992757605	0.115849129554637	-0.697279294081041	0.485628023589182	0.754862461053048
**Exoc2**	9550.76699782213	-0.0450689909776691	0.0646505894520633	-0.697116474260248	0.485729905091651	0.754909382629546
**Gpcpd1**	8217.47528315974	-0.0604528007254769	0.0867303354624557	-0.69702025713536	0.485790116620864	0.754909382629546
**Gm26931**	21.9370732152798	-0.606506160125654	0.870166049027377	-0.697000487209966	0.485802488904515	0.754909382629546
**Gm44443**	48.5261991273365	0.31784659855439	0.45607014137249	0.69692481423552	0.485849847638905	0.754909382629546
**Cep41**	907.781093283799	0.103105592287677	0.147976472362288	0.696770173269474	0.485946635009239	0.754912128165198
**Gm4737**	729.022950789108	-0.0639096084722239	0.0917228205146772	-0.69676889691805	0.485947433901098	0.754912128165198
**Ap5m1**	4900.20431431073	-0.0439827836526984	0.0631685266646688	-0.69627686404943	0.486255459256107	0.755316174240781
**Ikbkg**	5987.51172825805	0.0518235343275081	0.0744504596573625	0.696080784000682	0.486378239872569	0.755343037135468
**Psmg1**	2193.95886115971	-0.0597989929142424	0.0859061088095404	-0.696097096503589	0.486368024735932	0.755343037135468
**Prps2**	5979.74235310206	0.0676183125069932	0.0971606991813335	0.695943041546001	0.486464500910753	0.755343037135468
**Oas3**	5985.61238661758	0.0731840259803754	0.105147542287885	0.69601271116736	0.486420869364819	0.755343037135468
**5730409E04Rik**	49.7522001854767	-0.503743764588031	0.723945463728147	-0.695831094781464	0.486534613522098	0.75537746627921
**Srpk2**	5830.91077097491	-0.0837419125242728	0.120372089857643	-0.695692104567675	0.486621671131052	0.75540380460171
**Gm37691**	24.0625853661766	0.440437148756126	0.63312952882253	0.695650935086275	0.486647459574282	0.75540380460171
**Alkbh8**	1970.58488589877	0.0778741319145423	0.111990925312963	0.695361090167978	0.486829038489572	0.755611225323108
**Casp1**	1345.69870460128	0.152448692873855	0.219375461245929	0.694921355415197	0.487104588653138	0.755815559173559
**Taf9**	345.528417088282	0.499881928813323	0.719289828662019	0.694965935696845	0.487076649558267	0.755815559173559
**Fxn**	1320.1537577393	0.0830356401915558	0.119472532983783	0.695018663434774	0.487043605454315	0.755815559173559
**Nedd4l**	2516.20252357223	-0.0923953995142283	0.133069408444703	-0.69433989820901	0.487469075389917	0.756264870122673
**Traj11**	31.9967611195574	-0.322489147956126	0.464476779633549	-0.69430628633482	0.487490149510439	0.756264870122673
**Ccdc71**	5355.46602584429	0.0464767668524736	0.0669522305429033	0.694178020293007	0.48757057483606	0.756315175004115
**Ighm**	83985.3491180859	0.0737942608215396	0.106344979223403	0.693913914511346	0.487736196897515	0.756423154971864
**Gm20535**	19.9569365380804	0.557362038160585	0.803159203200511	0.693962088636414	0.487705984398153	0.756423154971864
**Chrna9**	30.4488877865223	-0.384420929286578	0.554382636066112	-0.693421662724542	0.488044971475325	0.7567534490774
**Gm33460**	22.1834844577699	0.422925908869014	0.609911940049809	0.693421264772215	0.488045221141368	0.7567534490774
**Rasal3**	91335.2907717795	0.0640891549696576	0.0924555056727225	0.693189167084574	0.488190845575051	0.756904767659753
**Rassf5**	61043.9275527229	0.0319043944489062	0.046074361797499	0.69245439772186	0.488652014442297	0.757545237265573
**Efcab9**	50.004676823919	-0.333080291021329	0.481163616619166	-0.692239145930599	0.488787159011059	0.757640811773463
**Gm10451**	235.093823583742	-0.199689682654445	0.28851183223242	-0.692136891264752	0.488851366056465	0.757640811773463
**Gypc**	2579.02305830355	0.0652520052751865	0.0942775757644537	0.692126465345421	0.488857912883214	0.757640811773463
**Zcwpw1**	1203.51680531476	0.0937118015798353	0.13543126287806	0.691951028059243	0.488968083636911	0.757675326795975
**Mest**	21.7195501110338	-0.490489049007842	0.708928501368326	-0.691873789897194	0.489016591742926	0.757675326795975
**Fam83e**	24.9079739740503	-0.600320116151003	0.867688536976466	-0.69186129650032	0.489024438250623	0.757675326795975
**Ccdc58**	615.296328896169	-0.110026795010211	0.159048244636857	-0.691782517068497	0.489073917422813	0.757677486661219
**Unc13b**	17.5958929527687	-0.552019247999819	0.798424745659581	-0.691385444903508	0.48932334846239	0.757878068188911
**Pabpc1l**	18.823411954346	-0.59999133046191	0.867858806512196	-0.69134670981008	0.489347684569016	0.757878068188911
**Zfp449**	52.4505885136579	-0.394633548255952	0.570753638389387	-0.691425374649508	0.489298262473295	0.757878068188911
**Zw10**	4531.0777712598	0.0548736988184397	0.0793941937229213	0.691155061161577	0.489468101300992	0.75792182720721
**Gm43127**	36.9333675469506	-0.277818857193991	0.401966876218426	-0.691148633458608	0.489472140233583	0.75792182720721
**Lsm12**	2093.63056345767	-0.0976202399814405	0.141365479274849	-0.690552180646897	0.489847007357493	0.758334465311466
**Dnaja4**	6325.29060860193	0.0782853658746595	0.113375730983826	0.690494916287042	0.48988300580094	0.758334465311466
**Gm12279**	29.8856741354455	-0.294970092174507	0.427135990024141	-0.690576535491275	0.489831697454729	0.758334465311466
**Ppp1r14b**	1317.83213574191	0.0925942445830155	0.134115581015688	0.690406318801873	0.489938704182641	0.758346184853229
**Eif2d**	5174.33141219399	0.0386805929332184	0.0560341701905807	0.690303662955298	0.490003244863673	0.758371587203309
**Fbxl18**	594.396653948563	-0.114941711760168	0.166579104634855	-0.690012784089114	0.49018614793943	0.758580154496326
**Trav13-3**	45.9870421699679	-0.289190966908452	0.41930677725326	-0.689688272636198	0.490390242332688	0.758821471907705
**Trbd1**	129.703025284275	-0.122421399930645	0.177529107951062	-0.689584943807593	0.490455238333051	0.758847524588952
**Ajm1**	20.9963482587241	-0.549127235610537	0.796757637698341	-0.689202349157073	0.490695938712023	0.758924885208055
**Arl15**	860.543560712617	-0.0818818912036962	0.118787398300672	-0.689314627435805	0.490625294905825	0.758924885208055
**Gm4609**	252.467869755345	0.0958370351358324	0.139055630874064	0.689199240141719	0.490697894935563	0.758924885208055
**Gm17249**	91.4913161802492	-0.17105444549703	0.248144476560773	-0.689334084190816	0.490613053563665	0.758924885208055
**Rabl2**	160.161954111894	-0.210591731702678	0.305619971163238	-0.689064038914514	0.490782968953701	0.758981965041071

**Gm37510**	186.060433361311	0.222771750650953	0.323333755316879	0.688983896632223	0.490833401423856	0.75898546675522
**Fbxo39**	35.7380685987318	-0.339287009681474	0.492616297159756	-0.688744996131224	0.490983754848171	0.758994508500209
**Hopxos**	235.216607937033	0.160482270115773	0.232974747366131	0.688839764524211	0.490924108846175	0.758994508500209
**Gm37522**	42.3988039855284	-0.477838742842609	0.693707967879981	-0.688818299583493	0.490937618261442	0.758994508500209
**Phlpp2**	236.279622365324	-0.158870980645044	0.230776034470184	-0.688420619627077	0.491187942696312	0.759086741179462
**Slc4a11**	26.9069637059892	-0.720748231040061	1.04688666723796	-0.688468249329831	0.49115795799331	0.759086741179462
**0610040B10Rik**	58.9677989792083	-0.32675956329301	0.474625280751621	-0.688457982633269	0.491164421184768	0.759086741179462
**Cyba**	22545.7213656923	0.072496057563247	0.105385925835268	0.687910240277888	0.491509307546718	0.759301869451095
**Dst**	76.9013093641925	0.287503067561403	0.417947110759911	0.687893420386768	0.491519900260276	0.759301869451095
**Zscan2**	74.8910572301647	-0.291960331476782	0.424334695922622	-0.688042562350408	0.49142597895096	0.759301869451095
**Gm9242**	159.827655046678	-0.156402364106179	0.227355950538797	-0.687918498440577	0.491504106823556	0.759301869451095
**Itpk1**	4694.89774253352	-0.0740883872254389	0.107727454918602	-0.687739140235135	0.49161706745438	0.759305869740577
**Gm3055**	379.6338782955	-0.0982224730505743	0.142819966610004	-0.687736283532322	0.491618866731825	0.759305869740577
**Rps21**	601.122934265572	-0.105703917289406	0.153783924981309	-0.687353488358768	0.491859999987856	0.75946599320801
**Pin4**	545.54466500716	0.122488405715997	0.178205866798867	0.687342161715946	0.491867135919765	0.75946599320801
**Trav14n-1**	681.357713804091	0.116768143485926	0.169870181289263	0.687396355261948	0.491832993785775	0.75946599320801
**Rbm41**	479.740375401281	-0.100617715425248	0.146475537617595	-0.686925046064219	0.492129962881206	0.759797356958569
**Coch**	29.2553792412907	0.592390005401312	0.862813354226673	0.686579551069028	0.492347718287266	0.759926441438798
**Nrp1**	588.166680250461	0.24238753695593	0.353044943668867	0.686562833720327	0.492358256054369	0.759926441438798
**Kat7**	11890.4727398971	0.0578090595676135	0.084192893203589	0.686626357260629	0.492318214686012	0.759926441438798
**Samhd1**	30589.4377344162	-0.0674227449419643	0.0982147779991493	-0.686482689423259	0.492408776626357	0.759929979755289
**Abhd8**	15643.3881668566	0.0643793240512915	0.0938341073308139	0.686097261247673	0.492651777864763	0.76014067225015
**Ncbp2**	2049.96141078267	-0.105351330122723	0.153596943885382	-0.685894702444981	0.492779511054207	0.76014067225015
**Mthfd2l**	282.332747647069	-0.141500006368535	0.206325272083904	-0.685810346640392	0.492832710896219	0.76014067225015
**Taf1c**	4109.77389635118	-0.0555380804782963	0.0809681895082865	-0.68592469234615	0.492760598361128	0.76014067225015
**Med30**	2295.13526182185	0.0594357468302951	0.0866454258592569	0.68596519944215	0.492735053770388	0.76014067225015
**8430432A02Rik**	21.251479746686	-0.484338593457084	0.706261910614965	-0.685777593521014	0.492853367812008	0.76014067225015
**Zfp277**	3586.96222503966	0.0491464976545873	0.0716702675515783	0.685730629081558	0.492882988410216	0.76014067225015
**4833419F23Rik**	34.9431139103149	0.388012003950676	0.565932075432365	0.685615855320162	0.492955380544284	0.760177914585662
**Tnfaip1**	2757.84028268874	0.0566441298477188	0.0826321647296964	0.685497348798875	0.493030133048338	0.760218789161626
**Sgk3**	2544.04463041918	0.0633780028557792	0.0924667624889443	0.685413884403673	0.493082785040841	0.76022558159379
**Tigit**	565.932576312296	0.278627751107178	0.406663572975323	0.685155419917793	0.493245851756856	0.760402591344563
**Ripor1**	2349.37103169045	-0.0995826545051747	0.145539017597017	-0.684233383936321	0.493827804743075	0.761082908345329
**Gm16433**	187.059894175791	0.184866498563695	0.270173314531651	0.684251510494896	0.493816360429548	0.761082908345329
**Gm44423**	22.8582119127292	-0.457246829484749	0.668268074210897	-0.684226655634678	0.493832052733556	0.761082908345329
**Tcn2**	1774.45035835943	0.0782830300220432	0.114464323204656	0.683907682589252	0.494033462465641	0.761169968521669
**Lin54**	1779.28234442371	-0.0648171797472915	0.0947663448842752	-0.68396834157151	0.493995157069817	0.761169968521669
**Rprd1a**	1146.33649500691	-0.088939594648221	0.130021330922426	-0.684038488279161	0.493950862274458	0.761169968521669
**Gm13032**	24.6222812279581	-0.443367800914272	0.648640095584413	-0.683534372809325	0.494269237948067	0.761458778094221
**Gm27010**	21.3268230579458	-0.539394908038911	0.789362190216691	-0.683330053965265	0.49439830739219	0.761583158625353
**H2ac7**	17.465565974343	0.388362240496423	0.568472811043877	0.683167660707079	0.49450090504542	0.761666741036235
**Scmh1**	1488.93143382137	0.121021817528454	0.177303390979873	0.682569108574988	0.49487915969684	0.761998370646469
**Golim4**	1492.69199331148	-0.0638450387974924	0.093523686832819	-0.682661697368929	0.494820638158012	0.761998370646469
**4930579K19Rik**	58.3472781716524	-0.231406424505822	0.339046662527211	-0.682520874209314	0.494909648105347	0.761998370646469
**Vdac3-ps1**	595.429986741589	0.118521668512535	0.173642637784782	0.682560861920531	0.494884372244682	0.761998370646469
**Retnlg**	29.8123076524128	0.774084335987565	1.13437388967693	0.682389063281442	0.494992969452606	0.762052195375155
**Ppm1l**	927.802820178302	0.0740853090739779	0.108598564476856	0.682194183973461	0.495116171805636	0.762167401244765
**Lockd**	169.57388356604	0.180992690222486	0.265363540322513	0.682055605689141	0.495203790716891	0.762227813844491
**Epc1**	7060.1132155065	-0.068947277088205	0.101169088093857	-0.681505372710694	0.495551768316439	0.762614436308441
**Cbfa2t2-ps1**	48.5756107144678	0.242699074122419	0.356097773411899	0.681551787861613	0.495522409468061	0.762614436308441
**Sec16a**	7044.77782121018	-0.129808109111553	0.190521690919106	-0.681329818590935	0.495662819482088	0.762636368184675
**Gm5898**	325.292528476183	-0.128026371034191	0.187902574090426	-0.681344423587192	0.495653580218441	0.762636368184675
**Cdc42bpg**	50.6642267267461	-0.59481155286387	0.873194638318209	-0.681190111301518	0.495751204327325	0.762697883868938
**Ocrl**	516.239880959648	-0.141068193295052	0.207143501972614	-0.681016744197469	0.495860895515998	0.76271565733232
**Bud13**	1644.50892258916	0.0603084313099226	0.088566134291173	0.680942346559358	0.495907971655113	0.76271565733232
**Gm6473**	94.9658708780663	0.211909958188012	0.311152605096226	0.681048317504773	0.495840917788032	0.76271565733232

**Impdh2**	4798.66480542284	0.0543156914562704	0.0797744547416712	0.680865718633334	0.495956461533166	0.762715788051737
**Cilk1**	470.043864845841	-0.138815873266719	0.203926372589633	-0.68071564998639	0.496051431766069	0.762787392440515
**Top3a**	3014.66930377297	-0.0602888811031885	0.0885978052994431	-0.680478268049914	0.496201677612115	0.762787697307353
**Psma4**	7975.37876054136	0.0684312017203947	0.100543112639033	0.680615508354853	0.496114811320944	0.762787697307353
**Fndc9**	78.2040411057165	0.243144347838301	0.357390938248217	0.680331597186247	0.49629452194627	0.762787697307353
**Dlgap4**	8054.6388473325	-0.0361180804940657	0.053094799522196	-0.680256462386052	0.496342086722872	0.762787697307353
**Rbm47**	72.9327041723536	0.31331005585563	0.460545332446808	0.680302315064319	0.496313058973869	0.762787697307353
**Gm12774**	21.2636460835464	-0.350501248458924	0.515123194448692	-0.680422182957701	0.496237179022819	0.762787697307353
**Prelid1**	20710.7003870251	-0.0336455613017172	0.049483663453773	-0.679932708158289	0.496547070121632	0.762923591888601
**Trav4-3**	209.365415979781	0.148436522411292	0.218297871635373	0.679972375815135	0.496521952313441	0.762923591888601
**Gm6467**	244.805323459993	0.124621227226666	0.183296865198963	0.679887389734643	0.496575766859776	0.762923591888601
**Ext1**	265.958816736839	-0.17642559304071	0.259558529406063	-0.679714103190587	0.496685504306807	0.763017791884797
**Rd3**	36.3861456946324	0.434452563791865	0.639369778744176	0.679501249879528	0.496820315953875	0.763150489226478
**Kdm4c**	4977.13870583817	-0.0474326247717817	0.0698206090902545	-0.679349913869518	0.496916177189419	0.763223335995095
**Bola1**	825.08390381682	-0.11595873013409	0.170721000972785	-0.679229441447427	0.496992495453252	0.763266155054278
**Aqp1**	44.9752571432036	0.34505677326241	0.508308875981826	0.678832870262032	0.497243764068681	0.763407831361206
**Snw1**	11145.4416244325	0.0450461000519036	0.0663601774323707	0.678812230389397	0.49725684340219	0.763407831361206
**Mab21l3**	26.8305492716692	-0.686987247975075	1.01209417227751	-0.678777990025525	0.497278541669824	0.763407831361206
**Gm21399**	177.975529685366	-0.128882072054656	0.18984059175445	-0.678896282736831	0.497203581202269	0.763407831361206
**2810025M15Rik**	28.1806825740406	-0.540932586917169	0.797146000788242	-0.678586590639956	0.497399841581735	0.763519659406079
**Tex14**	31.1198337477755	0.584739980005309	0.862308413436092	0.678110025246374	0.497701934721547	0.763865545364404
**Gm50069**	197.97213437621	-0.267678491598696	0.394760517051441	-0.678078176607045	0.497722126943983	0.763865545364404
**Zmat2**	3574.07930465894	0.051981876566165	0.0767074642758842	0.677663863052599	0.497984843876091	0.763896727507357
**Btbd6**	5076.56292331507	-0.0457561498799749	0.0675095035437937	-0.677773461188212	0.497915340333895	0.763896727507357
**Ndufb8**	6221.63606593766	0.0835580085836926	0.123255778884755	0.677923658750477	0.497820098352942	0.763896727507357
**Twnk**	2214.76986829594	-0.0709823227050473	0.104743878174782	-0.67767514380747	0.497977689753087	0.763896727507357
**Hpgds**	181.558836296344	-0.211838831590774	0.312504227041711	-0.677875091790355	0.497850894153422	0.763896727507357
**Fchsd1**	2111.42905162048	-0.066763315603302	0.0985328123212833	-0.677574444801277	0.498041553821712	0.76390935107259
**Hps3**	4985.83885050074	0.0702014297957427	0.103737548027966	0.676721506631502	0.498582668374814	0.763970372414153
**Rnf216**	940.276926584619	-0.135827473312116	0.200685801553816	-0.676816557327263	0.498522351562582	0.763970372414153
**A930024E05Rik**	225.1634351876	-0.184645287299985	0.272753092499436	-0.676968629788742	0.498425858223324	0.763970372414153
**Marf1**	5683.17116918255	0.08973145706836	0.132490573117557	0.677266728922233	0.49823673655354	0.763970372414153
**Haghl**	1894.91359508412	-0.109727491880893	0.162135853267781	-0.676762663342996	0.498556550870977	0.763970372414153
**Gnasas1**	28.0141038798401	-0.592016148472738	0.873987352590041	-0.677373816358225	0.498168806881804	0.763970372414153
**Trav6d-7**	328.106509361098	0.109734738239734	0.162039454693726	0.677209994609927	0.498272727302459	0.763970372414153
**Gm2451**	897.712661560229	0.0658908430604488	0.0973539244276959	0.67681753404184	0.498521731783895	0.763970372414153
**Gm44041**	18.6969084792026	-0.313201459865593	0.462820061557669	-0.676724035711591	0.498581063433119	0.763970372414153
**Gm49492**	39.3206532381822	0.466554732775906	0.689267703504453	0.676884656576532	0.498479139855132	0.763970372414153
**Gm49802**	30.1105384636221	0.437156865176332	0.646040411276589	0.67667108364398	0.498614667126176	0.763970372414153
**Pip4p1**	9933.50265329589	-0.0534962731226048	0.0790694804637237	-0.676572968594986	0.498676934694864	0.763991488574329
**Mettl23**	7429.11511553659	-0.0397036679696817	0.0586939848340194	-0.676452077362947	0.498753662586086	0.764034752223309
**Rbm12**	1815.24434147557	0.148432442076526	0.219511445302531	0.676194545901498	0.498917134946723	0.764194554148372
**Gm8515**	55.2332993964649	-0.228254332833294	0.337586952686986	-0.676134936544581	0.498954977031062	0.764194554148372
**Trim47**	44.8581870912637	0.433721394935795	0.642448801534446	0.675106551525787	0.499608071531596	0.76422914116332
**Spry2**	24.3576606550925	0.381809395886095	0.565466683750234	0.6752111253556	0.499541639307203	0.76422914116332
**Rad1**	2513.26182977439	0.0670108833237702	0.0992566854800645	0.675127151381951	0.499594984767315	0.76422914116332
**Xpnpep1**	4412.68091475017	0.0569960033079932	0.084367211994279	0.675570544062285	0.499313348502064	0.76422914116332
**Il18r1**	3220.33123438618	-0.0439919088370266	0.0651203915922779	-0.675547363296925	0.499328070481147	0.76422914116332
**Dvl1**	697.620259504704	-0.0937246517294097	0.138743151520334	-0.675526328344024	0.499341429865829	0.76422914116332
**Frrs1**	3365.18097734509	-0.0621498975915592	0.0919612556688491	-0.675826978867708	0.499150503522907	0.76422914116332
**Lrrn3**	48.4237153806581	0.506478434347638	0.750022534010708	0.675284289978955	0.49949516308039	0.76422914116332
**Amy2a5**	26.4264950495972	2.07490725120221	3.07092131449257	0.675662786086416	0.499254768373973	0.76422914116332
**A730011C13Rik**	210.144125229886	-0.104739095016757	0.15508692846452	-0.675357337035135	0.499448763826213	0.76422914116332
**4921531C22Rik**	276.51282664435	-0.118693104733188	0.175812786594558	-0.675110764309233	0.499605395201798	0.76422914116332
**Gm9320**	37.9194925419354	-0.291578130010582	0.431709904925293	-0.675402919145529	0.499419811376702	0.76422914116332
**Gm9449**	32.1770179773518	0.247857425782686	0.36673578378076	0.675847399529628	0.499137536908629	0.76422914116332

**Gm12174**	131.857122107772	-0.150059268190003	0.222336969065698	-0.6749182055534	0.499727733201253	0.764337982144451
**Tcf25**	23702.1538297253	-0.0638188884979625	0.0946709957533561	-0.674112361342731	0.500239881196054	0.764344438109851
**Akt1**	1157.29106611118	-0.1176613416054	0.1746347523244	-0.673756741079997	0.500465981390358	0.764344438109851
**Yeats4**	2090.20021061061	0.0500865491059359	0.0743449881374481	0.67370444680597	0.500499234193632	0.764344438109851
**Brd3**	6796.56724109062	-0.0375825686531292	0.0557688352866963	-0.673899113365464	0.500375455855444	0.764344438109851
**Cdr2**	590.725203821284	-0.206284298245182	0.305868922576337	-0.674420586791386	0.500043958044677	0.764344438109851
**Poglut1**	3637.35734640468	0.0576477397289003	0.0855700614312385	0.673690526390755	0.500508086083483	0.764344438109851
**Timm8a1**	1699.82672334884	0.0901773619691939	0.133724979456782	0.674349417255567	0.500089193262594	0.764344438109851
**Hmgb2**	6876.30923672925	0.121552582380465	0.180126293545182	0.674818650781681	0.4997909893864	0.764344438109851
**Zfp759**	216.005361295222	-0.144048994949519	0.213560040392482	-0.674512866193434	0.499985308669706	0.764344438109851
**Irak4**	2748.83100357685	-0.0805060447633923	0.119493263677912	-0.673728729850348	0.500483792982677	0.764344438109851
**1700028N14Rik**	53.4154684906399	-0.309244608672888	0.458317693957617	-0.674738533445069	0.499841898290465	0.764344438109851
**Gm17089**	55.7939653121949	-0.223801865060265	0.33193332806779	-0.674237402923754	0.500160393750525	0.764344438109851
**D130017N08Rik**	77.757604306057	-0.37604400727129	0.557845776541422	-0.674100303497355	0.500247546579145	0.764344438109851
**Gm28417**	285.139048335343	0.120770665957007	0.179219047790186	0.673871820245328	0.500392809152093	0.764344438109851
**Gm37900**	34.0698739700486	0.397589924787799	0.589831777460438	0.674073422255495	0.500264635678792	0.764344438109851
**Gm49911**	17.0653997128963	-0.390239001936875	0.578466935603242	-0.674609001688095	0.499924212387328	0.764344438109851
**Rab4a**	308.097790551633	-0.134460130925262	0.199623807633543	-0.673567609591418	0.500586251576526	0.764348451907951
**5330439K02Rik**	32.7932986825953	0.336148466113648	0.499081782064392	0.673533833920386	0.500607731401285	0.764348451907951
**Tmem265**	95.6561749826963	-0.23766059011424	0.352935323552211	-0.673382838878926	0.50070376350792	0.764421005870971
**Cct6a**	16639.6166074914	0.0374077712955845	0.0556150676689993	0.672619361325279	0.501189480978519	0.764970463257336
**Rsph3a**	921.710068251531	-0.0799907748865166	0.118929791388216	-0.672588204795612	0.501209307778685	0.764970463257336
**Gm16793**	22.9807253357792	-0.549188712432211	0.816387464760865	-0.672705959042473	0.501134375751557	0.764970463257336
**Def6**	13872.7712912777	0.040856614370982	0.0608661769434875	0.671253172495395	0.502059260449182	0.765039714642632
**Tsta3**	4763.23309717919	0.0587415510098707	0.0874272220784901	0.67189085519764	0.501653182562268	0.765039714642632
**Gatb**	1521.91744182988	0.0809482314054245	0.120494612793424	0.671799589448882	0.501711290147638	0.765039714642632
**Neil2**	32.5858995019212	-0.534941680489834	0.796618913886873	-0.671515163856379	0.501892402670483	0.765039714642632
**Rnpep**	5520.35088918379	0.0487537442139972	0.0726317138699089	0.671245956020263	0.50206385691305	0.765039714642632
**Sdhaf3**	485.173799597857	-0.0897392299592615	0.133657986758905	-0.671409409458896	0.501959752289544	0.765039714642632
**Zfp688**	1898.27079478939	-0.0782811414395864	0.11662507558967	-0.671220499054663	0.502080071658276	0.765039714642632
**Opa3**	4965.25294702042	0.0560393228286582	0.0833646987848036	0.672218860567316	0.501444375692196	0.765039714642632
**Nat8f1**	471.683132312512	0.139532084898911	0.207673148089474	0.671883130691481	0.501658100505864	0.765039714642632
**Gm10300**	27.5389026073223	0.388918922195887	0.579092787064153	0.671600356425787	0.501838151316458	0.765039714642632
**Traj58**	22.4794329161688	0.418864175978715	0.62355138992622	0.671739623623124	0.501749471463195	0.765039714642632
**Zfp984**	2005.64198368534	-0.0655452860853439	0.0976219820312601	-0.671419333243565	0.501953432130418	0.765039714642632
**B230369F24Rik**	44.6223463917311	-0.333047393498522	0.495680777252381	-0.671898949450176	0.501648029240482	0.765039714642632
**Trav9d-4**	119.798142084004	-0.22503716390786	0.334936884522315	-0.671879313109415	0.501660531046495	0.765039714642632
**Gm26982**	62.8589177825738	-0.24721314883218	0.367951236070988	-0.671863890095714	0.50167035048275	0.765039714642632
**Trav13n-1**	121.93617074694	0.168209610674504	0.250579262823006	0.67128304545024	0.502040233397085	0.765039714642632
**Gm49312**	22.8055203088353	-0.420675910387085	0.626216579696847	-0.671773830374685	0.501727691223214	0.765039714642632
**Tmem106a**	431.302376654237	0.094519291941159	0.140843379970297	0.671095027406277	0.502159994532639	0.765073486603841
**Mir1904**	56.4642543880823	0.275032603571775	0.409864341862356	0.671033255349981	0.502199344541698	0.765073486603841
**Plat**	354.270629941245	-0.224459064179769	0.334583020836535	-0.670862088633695	0.502308389612242	0.765165631778819
**Lsp1**	77416.4800923591	0.038999168042627	0.0581572199208649	0.670581711018744	0.502487036655082	0.765215834210314
**Ndufb4**	1307.90742618959	0.0740509353389382	0.110424304977845	0.670603589977729	0.502473094920997	0.765215834210314
**Gm49886**	34.5134921953091	0.288522937107136	0.430248768904113	0.670595613421586	0.502478177727101	0.765215834210314
**Iffo1**	2129.30973288875	-0.0816838741056741	0.121848716628553	-0.670371230537304	0.502621169705576	0.76534613172118
**Atp6ap2**	8428.13961747719	0.0351151597659279	0.0523947358624793	0.670203965873495	0.502727776077673	0.765360537937768
**Gm6627**	325.459424329323	-0.189425436894512	0.282625989300047	-0.670233609314006	0.502708881917174	0.765360537937768
**Gata3**	6224.63587706548	0.0815313522962088	0.121691133445782	0.669985971759676	0.502866732875003	0.765498126742481
**Wwox**	817.142429350317	-0.088356109821576	0.131918771894413	-0.669776625060579	0.503000196623526	0.76553614142592
**Med22**	5443.97031145737	0.0544539848704695	0.0813271495961807	0.669567114313653	0.503133783693641	0.76553614142592
**Pcgf5**	3374.79270902421	-0.053905814447468	0.0805086144035973	-0.66956579549653	0.503134624649796	0.76553614142592
**Alas1**	2519.49182980895	0.0480697172674654	0.0717632566158928	0.669837456300999	0.502961413259553	0.76553614142592
**Nol4l**	50.3931380416318	-0.40125547194166	0.599143037078014	-0.669715655711464	0.503039069625203	0.76553614142592
**Gm20628**	180.709274260745	-0.247129730877984	0.369161365281575	-0.669435520939434	0.503217699107158	0.765588614930142

**Npc2**	28646.2851447934	0.0347206723300393	0.0518948769422069	0.669057802540051	0.503458606365617	0.765733326303382
**Elf2**	7918.28351066563	0.0493637728050623	0.0737745620914265	0.669116446179475	0.503421199697636	0.765733326303382
**H3f3c**	117.998964507619	0.210362709107225	0.314382404005859	0.669130035354348	0.503412531861299	0.765733326303382
**Gm16523**	142.949076516243	-0.216129421565025	0.323082870146876	-0.668959705188861	0.503521182418045	0.765754586561253
**Snrk**	11353.8034900378	-0.0597949790568697	0.0894472204077322	-0.668494546664535	0.50381796176345	0.766131984511356
**Trbv3**	2352.21701568894	-0.0710048825058341	0.106363825736299	-0.667566082869863	0.504410613990571	0.766959186012682
**6330409D20Rik**	17.8752695054449	-0.340853947663432	0.510920528496323	-0.667136919838766	0.504684679318898	0.767169963608006
**Rab5a**	6620.71502584259	0.0629540837628422	0.0943947884632948	0.666923299344241	0.504821127526807	0.767169963608006
**Nus1**	1194.68927702623	-0.0734396675633921	0.110112792504552	-0.666949460575675	0.504804416226118	0.767169963608006
**Snx14**	3427.30842556805	0.0511857530606523	0.0767350310460745	0.667045446686774	0.504743104608203	0.767169963608006
**Ifit1**	2181.28214275577	-0.158014238532929	0.236963066878012	-0.666830661059407	0.504880305458275	0.767169963608006
**Gm10052**	488.804576692397	0.1290111221535	0.193418760781045	0.667004181148407	0.504769462700231	0.767169963608006
**Gm15478**	24.3348449808872	-0.320904043903483	0.481248694334159	-0.666815406839651	0.504890050302407	0.767169963608006
**Tlr4**	40.6456765123928	0.583485775766602	0.875435870859904	0.666508873109653	0.505085894070341	0.767393542847012
**Oip5os1**	4154.45646791044	-0.0454867765973419	0.0682572527204397	-0.666402100647699	0.505154120173473	0.76742320396967
**Cd300ld**	48.889485978133	0.293399119282068	0.440511802458987	0.666041449160455	0.505384607297632	0.767577375906024
**Rps29**	2699.99546835649	-0.110200822402169	0.165463063415188	-0.666014638721197	0.505401743671066	0.767577375906024
**Gm50340**	87.1350399386029	0.22260681374111	0.334223004492473	0.666042764109384	0.505383766832296	0.767577375906024
**Ddc**	1195.27916939836	-0.075601975983937	0.113538749473009	-0.665869373538501	0.505494597840089	0.767613603280079
**Supt4b**	24.6204913589207	0.29784772615281	0.44733641987039	0.66582489804677	0.505523028573801	0.767613603280079
**Gm37423**	108.533144316299	-0.223653382867338	0.335989236336974	-0.665656392167959	0.505630752744127	0.767683976211041
**Gm3219**	30.6397449240535	0.318485364829071	0.478493645663756	0.665599988035944	0.505666814056889	0.767683976211041
**Gm45716**	204.324384079738	0.106597717389261	0.160189517128291	0.665447523035416	0.505764297522213	0.767757999620184
**Msl3**	1828.2454141403	-0.0648697873652926	0.0975823165718372	-0.664769905493455	0.506197674023063	0.768267843342458
**Gm6722**	19.3164735366629	0.389829745895596	0.586346239365538	0.664845648737196	0.506149222042992	0.768267843342458
**Rnaseh2b**	1072.49046636729	-0.0882027442786884	0.132721888603292	-0.664568182436944	0.506326725571747	0.768389696121931
**Nsun6**	1088.63891692505	0.0885087726317317	0.133274512131432	0.664108772309341	0.506620695998051	0.768641742512125
**Hbb-bs**	411.683858504716	0.212431009878736	0.319932660217563	0.663986633106719	0.506698866344222	0.768641742512125
**Rpl19-ps1**	62.9770358926133	0.18792884961373	0.283051731976665	0.663938172366397	0.506729883473319	0.768641742512125
**Rpl35a-ps4**	28.6031099463481	0.298985656439526	0.450328773820641	0.663927498798039	0.506736715187913	0.768641742512125
**Gm16550**	46.7916866120378	0.211389858221868	0.318317944326393	0.664084014079694	0.506636541007387	0.768641742512125
**Zfp26**	2238.87347499845	-0.117108620305742	0.17650169814159	-0.663498547259286	0.507011309589022	0.768836224154962
**Ifi204**	34.8273637782054	-0.408815937303226	0.616099587840556	-0.663554959898829	0.506975192432235	0.768836224154962
**Gm8990**	27.9342267728583	0.363433319124335	0.547683060963156	0.663583274759678	0.506957064871989	0.768836224154962
**Smt3h2-ps4**	430.07109285516	0.504734808329874	0.76090010729771	0.663339120981871	0.507113386644795	0.768917016297348
**Aga**	1708.55877422579	-0.0676337488935233	0.10201921246323	-0.662951097744457	0.507361874296725	0.768968267432904
**6430548M08Rik**	59.178109512354	0.229761110445997	0.346593682519559	0.662911997632938	0.507386917309262	0.768968267432904
**Gm15590**	233.241990620193	0.102761892984052	0.155031198487379	0.66284653661126	0.50742884552733	0.768968267432904
**Ighv4-1**	17.7463857715273	0.811386652644562	1.2240705179296	0.662859402918178	0.507420604428853	0.768968267432904
**Gm6397**	107.958126506342	-0.194725779075551	0.293779756476239	-0.66282912550273	0.507439997763999	0.768968267432904
**Gm45844**	43.2359342699944	-0.297689986348547	0.448885801504369	-0.663175322879198	0.507218274125546	0.768968267432904
**Swi5**	6355.59741084225	0.0570453578224884	0.0861043894463465	0.66251393441486	0.507641907541136	0.7691263016467
**Gm4668**	48.9793615793734	-0.375294615779282	0.566416728543102	-0.662576857051148	0.507601596257412	0.7691263016467
**Calhm2**	750.536968135893	-0.0878364554483286	0.132659934071843	-0.662117436307183	0.507895962058337	0.76942661567632
**1700001G17Rik**	61.7521862533742	-0.325189135027998	0.491183545233994	-0.662052176184121	0.50793778360507	0.76942661567632
**Tnfsf13b**	29.2490874927925	0.467231821758473	0.70629065240562	0.66152910302166	0.508273057086037	0.769776742050213
**1110032F04Rik**	211.515960224816	0.285323545364664	0.431291210627558	0.661556596410808	0.50825543179683	0.769776742050213
**Ppp1r37**	1247.50543842509	-0.0994503953048213	0.150349158649693	-0.661462932004405	0.508315478900328	0.769776742050213
**Elk1**	845.200534431383	-0.120428912482205	0.182132041429141	-0.661217606398255	0.508472771773689	0.76986696116441
**Epb41**	12963.8064351398	-0.0887955366909909	0.134289296728286	-0.661225718313613	0.508467570333136	0.76986696116441
**Gm42737**	21.2597038685257	-0.774710382250308	1.17197790003934	-0.661028149271676	0.508594261496182	0.769976919522996
**Epas1**	168.790604943106	-0.191567969771623	0.289880871136374	-0.660850676419763	0.508708080083681	0.770075244296144
**Plaat3**	4445.87879533936	-0.0682122419293376	0.103249930720753	-0.660651696840581	0.508835707445647	0.77012047279042
**Rps4x-ps**	33.6318788972787	-0.30058939691118	0.454976478542558	-0.660670190850455	0.508823844507543	0.77012047279042
**Fam217a**	18.5083044790297	-0.756473549151021	1.14624171108009	-0.659959886155432	0.509279571844936	0.770257467800417
**Glp1r**	25.7820643327873	-0.618080349306789	0.936533881117975	-0.659965818395127	0.509275764869616	0.770257467800417

**Abcb8**	3652.86893731199	0.0623653900384386	0.0944560277672653	0.660258445253527	0.509087992034449	0.770257467800417
**Mmaa**	548.071047604201	0.1195749403828	0.181165062754787	0.66003311325346	0.509232579885957	0.770257467800417
**S100pbp**	1168.05933123178	-0.0601171972939369	0.0910903708150454	-0.659973131693601	0.509271071629533	0.770257467800417
**Rspry1**	6114.16288327647	-0.0527482990370413	0.0799336418026107	-0.659901111065335	0.509317291172974	0.770257467800417
**Gm17066**	230.415464995513	-0.119480244718008	0.180990310520518	-0.660147188953873	0.509159378715552	0.770257467800417
**Gm45133**	49.3241520517272	-0.274643075485426	0.415955774557495	-0.660269894744456	0.509080645861217	0.770257467800417
**Ndufaf8**	1809.23430625204	0.0861422473482296	0.130585209075676	0.659663126918982	0.509470034129099	0.77041452281883
**Dnajc19**	3436.40587362116	-0.0573510205874783	0.0869505494269633	-0.659582037899047	0.509522084151963	0.770419295651327
**Kdm2a**	11028.3147574025	-0.085430412476987	0.129588258476664	-0.659245007852092	0.509738449333216	0.770672495312175
**Hmgb3**	640.725822817837	0.170769409318112	0.259271944157793	0.658649781305229	0.510120687882665	0.771102425073594
**Hmgcr**	3143.37201204623	0.0621053947168121	0.0942885551142147	0.658673734490702	0.510105302893387	0.771102425073594
**Zfp825**	1805.07668948638	0.0921560624879307	0.139934254078042	0.658566861238525	0.510173948825289	0.771108960349169
**Smarcal1**	1906.60425259241	0.0620393360930041	0.094258810276011	0.658180767520182	0.510421981561359	0.771409856460995
**4833412K13Rik**	86.6204518124287	-0.241903515610032	0.367709828395693	-0.65786524299731	0.510624726334583	0.77156825976736
**Gm35551**	173.299037037582	-0.178530773477163	0.271377351210543	-0.65786909880572	0.510622248475958	0.77156825976736
**Dpp4**	28831.8649352222	-0.0332535078127029	0.0505643345405311	-0.657647492345573	0.510764669661849	0.771705714820382
**Tpd52l2**	5777.93778216128	0.0553142220856562	0.0841334393907389	0.657458229286951	0.51088632094974	0.771815509032124
**Gm12808**	53.01635776181	0.270198451687468	0.411031739821728	0.657366391716266	0.510945356195705	0.771830694811209
**Dgkq**	2763.67572731895	-0.0734437293974114	0.11175187634836	-0.657203545902601	0.511050045892521	0.771840817028843
**Ncdn**	7033.46611327284	-0.0512168868760529	0.0779277984511527	-0.657235131673289	0.511029739276563	0.771840817028843
**1110019D14Rik**	59.0887306057345	-0.264234667487677	0.402105697905583	-0.657127389300813	0.511099009004186	0.771840817028843
**Igfbp4**	25986.8812893388	0.109032717210925	0.165984684047444	0.656884204929172	0.511255375130958	0.771918461053175
**Ckap2l**	178.226378615009	0.22889110726378	0.348455488146727	0.656873302472994	0.511262385931539	0.771918461053175
**Gm11560**	138.180397416992	-0.202480064008808	0.308273823540047	-0.656818868639699	0.511297390240019	0.771918461053175
**Mapk8ip3**	6661.14693022213	-0.0877321925974097	0.133597389048435	-0.656690922047905	0.511379672709373	0.771943429892228
**Sv2a**	151.578266779282	-0.237791266183076	0.362132945767384	-0.656640797150286	0.511411909922255	0.771943429892228
**Ankmy1**	89.6068895696767	0.29190413143882	0.444759533846942	0.656318997625523	0.511618896608763	0.772181892132593
**Egf**	232.166271741634	-0.242811185779716	0.370133140986406	-0.656010388944432	0.511817439799658	0.772407566221934
**Creb3l2**	520.160338999991	-0.104635464983271	0.159607116137075	-0.655581452229276	0.512093462715086	0.772676117207854
**Sh3d21**	39.2841918070997	-0.415921530158931	0.634380405170184	-0.655634264187829	0.512059473765207	0.772676117207854
**Fut8**	2105.03895115874	0.0628856065613902	0.0960043081197697	0.655029006437269	0.512449078710059	0.772820284533452
**Itsn1**	127.714382208203	-0.165352208316653	0.25241818965364	-0.655072475337628	0.512421092590454	0.772820284533452
**Gnb4**	563.358448310858	-0.101506081065437	0.154940965494588	-0.655127459296636	0.512385693994679	0.772820284533452
**Tmem91**	666.982526740782	-0.113005703490768	0.172534146438147	-0.654975874768536	0.512483286989277	0.772820284533452
**Gm11687**	57.887118650415	-0.175629142239486	0.268134350946988	-0.655004260435878	0.512465011020784	0.772820284533452
**Gm47694**	98.7505134568331	-0.206338867420351	0.314868248987532	-0.655318115065078	0.512262959954584	0.772820284533452
**Tmem108**	377.118797626372	0.0901970751426446	0.137733076901614	0.654868657345645	0.512552321450794	0.772850423837335
**Mfsd1**	5624.30767867021	0.0589167581718062	0.0900113201393442	0.654548317707136	0.512758608542242	0.772939576657429
**Entpd1**	414.756897150112	0.209068382680403	0.319383012489157	0.654600822539053	0.512724794373262	0.772939576657429
**Gm6710**	238.001882365574	-0.216459911081371	0.330678596745731	-0.654593049600407	0.512729800228976	0.772939576657429
**Lzts3**	64.4562045710859	-0.422377615138385	0.645463917934419	-0.654378352379567	0.512868077605168	0.77300062500441
**Arhgef10**	458.96725434964	-0.135319119704748	0.206804636384752	-0.654333104278144	0.512897222467545	0.77300062500441
**Scin**	19.296635327208	0.776259978536542	1.18698254220799	0.653977586808115	0.513126245694568	0.773226713112929
**Mxd4**	3702.70210373416	-0.103689256139244	0.158577366159505	-0.653871726151312	0.513194451084281	0.773226713112929
**Erich1**	1020.08223261626	0.0704327952711925	0.107705357789378	0.653939569180266	0.513150739682071	0.773226713112929
**Myo9b**	10101.7462417684	-0.116128714414969	0.177719140659429	-0.653439545026336	0.513472951783238	0.773329935380145
**Pknox1**	9434.97171639012	-0.042731955505061	0.0653912105372761	-0.653481640024109	0.513445821998553	0.773329935380145
**Tnfsf4**	24.3911686133213	0.527255511473668	0.806880349715809	0.653449438518824	0.51346657546398	0.773329935380145
**Zfp345**	23.7598064885949	0.493078707842097	0.754342725563423	0.65365342719228	0.513335114707312	0.773329935380145
**A930006K02Rik**	544.340478069298	-0.102488205880685	0.156857390026362	-0.653384618113691	0.51350835272466	0.773329935380145
**Ccdc127**	2430.77108699378	0.0542908344918449	0.0831076037922803	0.653259533598632	0.513588975679534	0.773377435950154
**Snhg16**	826.474362173903	0.0977748131564448	0.149721624514829	0.653044030702195	0.513727893059958	0.773497837279663
**St6galnac2**	43.2930586781826	-0.311767607444248	0.477451194135309	-0.652983197599657	0.513767110805925	0.773497837279663
**Gpr18**	17404.6106283072	-0.119087681498415	0.182510426880149	-0.652497961536286	0.514079987451961	0.773847876969708
**Trim34b**	347.559604664657	-0.126448309244081	0.193799337135218	-0.652470287634961	0.514097834366878	0.773847876969708
**Wdr24**	7341.50176547737	-0.0449857098758383	0.0689613165466325	-0.652332526822025	0.514186681189303	0.77390768320755

**Tmem161b**	1433.09004954424	0.0539360559202996	0.0827609921173176	0.651708667820738	0.51458912987176	0.774439438337088
**Kansl1**	6703.20941419424	-0.0669158573567657	0.102732074633447	-0.651362854254863	0.514812283181621	0.774553341553787
**Vamp9**	18.8019539164972	-0.332274704222033	0.51003603846886	-0.651472992417418	0.514741205627496	0.774553341553787
**Gm48745**	90.8299063156691	0.201800437680654	0.309808583085795	0.65137135863266	0.514806794708156	0.774553341553787
**Psma2**	16262.0627004072	0.0503509804382306	0.0773226730267626	0.651180028667702	0.5149302807508	0.774607070654535
**Gm45615**	21.8356304558886	-0.345201457249493	0.53013699829825	-0.65115518886174	0.51494631370859	0.774607070654535
**Poldip3**	9286.77501431064	0.0661832527727832	0.101672827861604	0.650943365742429	0.515083046370854	0.774738789773343
**Gabpa**	2762.98432687529	-0.0561916403576675	0.0863468622579524	-0.65076644232654	0.515197265587383	0.774836624402061
**Rps15a**	6001.63069304381	-0.0507653065634484	0.0780680690465389	-0.650269786142469	0.515517969958683	0.775166459832344
**Cyc1**	17933.8730851193	0.0329577471186292	0.0506947037801272	0.65012209680864	0.515613356952643	0.775166459832344
**Cdh24**	238.393656963447	0.154324127633007	0.237373969798707	0.65013079472814	0.515607739039271	0.775166459832344
**0610012G03Rik**	2331.25535099088	0.059167858742749	0.0909847060370997	0.650305543863854	0.515494876766756	0.775166459832344
**Qprt**	1169.10267241716	0.110217365026889	0.169580592624489	0.64994091199427	0.515730389929949	0.775268436760752
**Polr3gl**	2476.43994477461	0.0773760047770677	0.11911471192427	0.649592342768383	0.515955580531011	0.77538503408938
**Recql4**	177.596732773199	-0.231673586773549	0.356596032805248	-0.649680774491608	0.515898445034238	0.77538503408938
**Hpf1**	2210.79515986117	-0.0709455811191726	0.109204263172607	-0.649659446051447	0.515912224981276	0.77538503408938
**Cep55**	116.583940693458	0.252842039579948	0.389306314751218	0.649468118033287	0.516035847339521	0.775431703845969
**Arfgap1**	7296.42903952025	0.0527048022287246	0.0811996700538002	0.649076556515613	0.516288894024178	0.775737971120336
**Agpat2**	1737.97102483287	0.0733853167665219	0.113090946648637	0.6489053186063	0.516399576763022	0.775830294723011
**Gm5421**	129.156868659907	-0.169238365836335	0.26086477311604	-0.648758986561411	0.516494170889413	0.775898431757506
**Ociad1**	11473.1187834561	0.0303344292797726	0.0467716728124757	0.648564129861128	0.516620146983056	0.776013694567399
**Hus1**	854.359030292048	-0.0931608517195438	0.143708061506039	-0.648264618861543	0.516813813789151	0.776114133172322
**Slc4a1ap**	2834.08074499129	-0.0689113657554399	0.106306606975496	-0.648232200387356	0.516834778154292	0.776114133172322
**Gm16973**	805.103089769497	-0.10118397956377	0.156085116813113	-0.64826154876074	0.516815799141751	0.776114133172322
**Eif3e**	12805.7947805493	0.041369286719387	0.0638288496164544	0.648128345849467	0.516901941729152	0.77614102304424
**Ccdc124**	4569.92953652444	0.0565013572834727	0.0872223374862461	0.647785405801379	0.517123755959877	0.776202294382576
**Xrcc4**	1539.83831719605	-0.0573466346415477	0.0885410063541957	-0.647684468506497	0.517189051768907	0.776202294382576
**Atg4c**	94.1862352794638	-0.144285889481583	0.222704352411844	-0.647880869498037	0.517062004921232	0.776202294382576
**Gm13841**	464.385825319451	0.0937058238645116	0.144639640635904	0.647857139664734	0.51707735429336	0.776202294382576
**Gm16536**	369.701606771593	-0.142601732988231	0.220153895193246	-0.647736588367235	0.517155335170068	0.776202294382576
**Czib**	4368.67582384111	-0.0619432318316263	0.0956877248524805	-0.647347733757101	0.517406914593697	0.776455309833608
**Gm17235**	17.3348576715132	-0.426076605820432	0.658322018847759	-0.647216094284953	0.51749209646415	0.776509186171375
**Mrs2**	1685.92576781213	0.121766203685726	0.18817008146143	0.64710714232584	0.517562603084421	0.776541033784157
**Raver1**	8566.34551221463	-0.112200525314443	0.173470452112894	-0.646799059712044	0.517762001011406	0.776544436506447
**Ugdh**	2167.66829082975	-0.0598108655190106	0.0924693921460469	-0.646817980857329	0.517749753679482	0.776544436506447
**Gm6104**	16.9543912602248	-0.397730805879795	0.614907094483666	-0.646814469125238	0.51775202675194	0.776544436506447
**Gm49839**	142.881673507989	-0.332553753380283	0.51410707198937	-0.646856990497028	0.51772450388369	0.776544436506447
**Zfp655**	4372.98750157611	0.0647108400294083	0.100126156375296	0.646293060395299	0.518089581259118	0.776758729620686
**Fkbp3**	6231.49989358404	0.0756389796179497	0.117000997120893	0.646481495707208	0.517967577096378	0.776758729620686
**Acad9**	4046.5283458047	0.0623198489227355	0.096429486840559	0.646273779573026	0.518102065641519	0.776758729620686
**Gm36527**	62.5333646798637	-0.295857573448814	0.457711573082002	-0.646384297116754	0.518030507355314	0.776758729620686
**Slc35b2**	4876.72770495921	0.0613853288154089	0.0950183171723854	0.646036792085484	0.518255528360409	0.77691488539387
**Trbj2-7**	1416.91318710663	-0.0535318569105203	0.0829105540093895	-0.645657932818274	0.518500909836672	0.777134865052453
**Gm43682**	35.1331162861355	-0.447211076201925	0.692605046926685	-0.645694220950809	0.518477403956508	0.777134865052453
**Gm49396**	17896.6511289547	0.0515634340879249	0.0799068346102996	0.645294414919529	0.518736411372359	0.777413889480147
**Mylpf**	716.612134008203	-0.0747282563054708	0.115998502169104	-0.64421742443304	0.519434453436607	0.778341482521848
**Ubqln2**	998.159725162297	-0.0790397609591388	0.122696911272669	-0.644187047084576	0.51945414927967	0.778341482521848
**Cgrrf1**	630.453760060197	-0.142589340958719	0.221452482401373	-0.643882332735751	0.519651739063106	0.778563511687492
**Gm5436**	47.3612122231007	0.244590614907323	0.379965455933061	0.643718030384354	0.519758295806635	0.778578372918611
**Gm16573**	50.8003271372853	-0.269604033865454	0.418825374596173	-0.643714660615783	0.519760481356278	0.778578372918611
**Pkd2l2**	345.213523110104	-0.116999999166373	0.181891388883652	-0.64324100159141	0.520067732197788	0.778823011527994
**Apod**	18.8080339260274	0.580359417003266	0.902251968962368	0.643234303684264	0.520072077635182	0.778823011527994
**Pagr1a**	1045.68820167121	0.157286870041476	0.244496611756709	0.64330899684609	0.520023619608921	0.778823011527994
**Hnrnpa0**	764.096388997542	-0.0777527994440872	0.120898353906243	-0.64312537707821	0.520142749165579	0.778854822636323
**F11r**	36.1989489596832	-0.369472988787465	0.574579037705124	-0.643032489077821	0.520203018767381	0.778871053469937
**Gm5616**	31.9943723590208	-0.272160596565159	0.423327487510762	-0.642907924938941	0.520283846817353	0.778918059316013

**Ccdc66**	1345.26081467915	-0.0748878766389864	0.116564748187547	-0.642457327823465	0.520576287481865	0.779281831830871
**Nab2**	2812.57633384832	-0.0740002766703052	0.115213721344168	-0.642287010669943	0.52068684655235	0.779373291642955
**Nrp2**	226.35386194089	-0.22671718777733	0.353058495206652	-0.642151912092155	0.520774552564132	0.779430530466663
**Bmp8a**	18.9790785783977	0.550864059853592	0.858153775682852	0.641917655626764	0.520926649923095	0.779584122218465
**BC037704**	102.620878345354	-0.209880700130036	0.327019550558975	-0.64179863182885	0.521003938113227	0.779625741011408
**Pstpip2**	35.7101763623698	0.361607694039158	0.563516137736824	0.641698914766551	0.521068694005826	0.779648600460526
**Zfp385a**	17.9171910335921	0.791595566775718	1.2340429128848	0.641465186105416	0.521220492775048	0.779736965834781
**Spata7**	22.5045580924217	-0.635221861844121	0.990517175248116	-0.641303227967757	0.521325692395089	0.779736965834781
**Igkj5**	26.2129129600302	0.481893621778425	0.751393218086184	0.641333472513659	0.521306046274337	0.779736965834781
**Med20**	4216.45560535898	-0.0428306798274659	0.0667756325840331	-0.641411817006241	0.521255157371223	0.779736965834781
**Kbtbd4**	4581.7940179194	0.0719464393961909	0.112234361948546	0.641037540973192	0.521498292574552	0.779847071851306
**Tmem131l**	13631.5841626852	-0.0387497290941961	0.0604439908334518	-0.641084888007605	0.521467532022443	0.779847071851306
**Fam241b**	26.6081800478298	-0.576155909518897	0.89894202039914	-0.640926663171311	0.521570331624358	0.779880785291801
**1700022I11Rik**	60.7267694585769	0.165724553804996	0.258608311526333	0.640832279623466	0.521631658132065	0.779898475869338
**Mbd3**	5452.29640065271	0.044195853892767	0.0689781134739047	0.640722856380913	0.521702761451562	0.779930779145375
**Slc22a5**	1744.79141583852	0.0740473197943248	0.115677564982244	0.640118244239414	0.522095728814222	0.78029615848674
**Coprs**	20.0659218942955	-0.574783637874252	0.897890659496823	-0.640148810765401	0.52207585846426	0.78029615848674
**Gm46516**	19.6005299286544	-0.82403038857308	1.28705579335699	-0.640244496645936	0.522013658556413	0.78029615848674
**Sh3gl1**	28163.1504655706	0.0533677798756834	0.0833894256990397	0.639982580864542	0.522183924056068	0.780335018203733
**Gm45706**	43.2798397758838	-0.524250153071209	0.819235718700286	-0.63992589813214	0.522220775969941	0.780335018203733
**Snap29**	1551.06803747983	-0.0703784752897895	0.109995961994298	-0.639827808346618	0.52228455157118	0.780356313209472
**Gm47512**	44.6729391993604	0.361929450644829	0.565774190467264	0.639706541484187	0.522363401879191	0.780400125477426
**Gm22068**	18.2306056298885	0.405222048283598	0.633540901348193	0.639614660113142	0.522423149185367	0.780415392653947
**Utf1**	57.4351600511664	0.164714137468442	0.257575028917363	0.639480225085355	0.52251057400316	0.780471998726684
**Ifi47**	33983.8397755072	-0.0816864961441908	0.12777678775305	-0.63929057523393	0.522633918507206	0.78058224207754
**Sesn2**	891.763418137907	0.132776571417426	0.207780501564236	0.639023250102117	0.522807806870982	0.780767947385657
**Slc37a1**	9229.698413993	0.0541142126392912	0.0847332603144184	0.638641926894946	0.523055899562883	0.781064424697911
**Zfp106**	7701.34344532488	-0.061153644668201	0.0957821738503868	-0.638465825214239	0.523170493474977	0.781161514800078
**Hnrnpa1**	5973.45707000735	0.0828840950619743	0.129851095447031	0.638301084612603	0.523277706104507	0.78124756633397
**Nlgn2**	21.8287407941066	0.601627163573285	0.942896880256551	0.638062524302328	0.52343298033611	0.781331325422138
**Nnt.1**	680.903439693983	0.409881037548013	0.642312996522639	0.638132872551281	0.523387189581624	0.781331325422138
**Osbpl9**	11415.1822514299	-0.0430811056829411	0.0675453024562344	-0.637810537762492	0.523597019107321	0.781489884083431
**Thoc6**	2762.89069833816	0.0563251361732084	0.0883189376849838	0.637746984390925	0.523638395389532	0.781489884083431
**Chka**	992.216182356257	-0.0850008345443873	0.133349164681322	-0.637430573693676	0.523844418832654	0.781501278881474
**Mtus2**	167.02684653929	-0.226462145593089	0.35524281334074	-0.637485508752212	0.523808646169459	0.781501278881474
**Traj45**	41.522957127754	0.52750162426659	0.827467264750229	0.637489417089891	0.523806101182178	0.781501278881474
**Trav9d-2**	53.1053832324274	-0.193513193825738	0.30350255537179	-0.637599883100441	0.523734171792894	0.781501278881474
**Primpol**	1298.7482034029	-0.0815618502170821	0.128022099031046	-0.63709196173469	0.524064944142206	0.781756255310872
**Gm12263**	213.678973107292	0.189979434835168	0.298323725412117	0.636823083959287	0.524240088075602	0.781943493734122
**4930451G09Rik**	98.7847387570496	0.23616918452284	0.370968530313599	0.636628622711456	0.524366776616891	0.782034235339628
**Wbp2**	15774.4169920515	0.0320324084062858	0.0503197430230303	0.636577344833124	0.524400185987086	0.782034235339628
**Nup210l**	53.4684910324444	-0.212117310209111	0.333508371060888	-0.636017949217787	0.524764723052397	0.782062578915867
**Taf4**	553.40744138196	-0.110500411624955	0.173759481346867	-0.635938889598599	0.524816253694001	0.782062578915867
**Vcpkmt**	692.540009253433	0.0752570640725398	0.11826054863915	0.636366606941529	0.524537500704621	0.782062578915867
**9130023H24Rik**	324.585482340567	0.138654328712238	0.217988391935599	0.636062899868544	0.524735425611388	0.782062578915867
**Gm32391**	26.2991995846708	-0.364421574514111	0.572812544668632	-0.636196916261543	0.524648082851647	0.782062578915867
**Gm46378**	24.8956381170466	-0.395373769584846	0.621364090461098	-0.636299676235634	0.524581115941435	0.782062578915867
**Gm47167**	23.48846632214	0.280378633696408	0.440668606450358	0.63625733622119	0.524608707671305	0.782062578915867
**AL645861**	67.0279863043247	0.229643179759205	0.361101310564127	0.635952219061311	0.524807565439118	0.782062578915867
**Fam3a**	3002.67735322179	-0.0557942626863224	0.0877861285796189	-0.635570375286786	0.525056483554375	0.782198639629979
**Gins2**	525.083936874195	-0.131443917910431	0.206800777472747	-0.635606497793525	0.525032933257102	0.782198639629979
**Gm43788**	112.016240894125	-0.208360229411811	0.327822246027248	-0.63558904844576	0.525044309402491	0.782198639629979
**Gm6565**	679.958357233188	0.0761964039752088	0.119909207531116	0.635450817698347	0.525134433738281	0.782240808509557
**Zfp939**	71.8834200079368	-0.313672374801376	0.493812933971212	-0.635204858404261	0.525294815015973	0.782405747254625
**Ssbp2**	7593.09730164483	-0.0497284979709769	0.0783307007039758	-0.634853225160193	0.525524146025075	0.782533853066389
**Map2k4**	6518.14860104047	0.0515861347944498	0.0812579010291967	0.63484453992867	0.525529811077523	0.782533853066389

**Zfp960**	303.050811077137	-0.150239662421116	0.236602249205302	-0.634988310236862	0.52543603909126	0.782533853066389
**Polr3h**	2330.22467384654	0.0651512345128538	0.102653923047037	0.63466872554886	0.525644494952911	0.782630663041956
**Gprc5b**	59.6845486130359	-0.310133574715602	0.488848554778825	-0.63441647046685	0.525809063518784	0.782801720886762
**Celf1**	17292.3485999986	-0.0584842983605867	0.0922086953508346	-0.634260121977286	0.525911076853064	0.78280745019869
**Zc3h6**	809.490260913459	-0.152615504695025	0.240620433295072	-0.634258290557865	0.525912271866684	0.78280745019869
**Tjp2**	123.322008869057	0.174632869673216	0.275373173845226	0.634168053607751	0.525971153809085	0.782821145798597
**Fancg**	2512.91165179304	0.0436855269438715	0.0688957103152835	0.634081958716384	0.526027336092271	0.782830821194363
**Commd4**	7661.86165465063	-0.0624051201077004	0.0984585874691745	-0.63382099735321	0.526197648385358	0.783010326339417
**Rps11**	39151.8502675786	-0.0380336145507784	0.0600660075744029	-0.633196979234332	0.526605018124539	0.783098836408868
**Coro1a**	234929.368398754	-0.0439503621302183	0.0693608614948709	-0.633647869749546	0.526310652914241	0.783098836408868
**Acsl3**	618.55047739586	-0.110915951377288	0.175111386588889	-0.633402279188652	0.526470977015384	0.783098836408868
**Rnf113a1**	665.727270387227	0.0930309967403463	0.14689944069822	0.633297147342193	0.526539615814556	0.783098836408868
**Teddm2**	75.9591019724489	-0.190690112288266	0.301147093094535	-0.633212528564587	0.5265948652986	0.783098836408868
**Rpl30-ps8**	32.9442988269346	-0.323221256754852	0.510315967394273	-0.633374766627927	0.526488939058055	0.783098836408868
**Gm6485**	156.403951857392	0.118559005478152	0.187215870138504	0.633274334010471	0.526554510855312	0.783098836408868
**Rpl15**	29760.1057066416	-0.0402112913539781	0.063519585637802	-0.633053426753581	0.526698754429463	0.783131717847231
**Parn**	6570.36144215555	0.047737081635131	0.0754154692799092	0.632987927953506	0.526741526395176	0.783131717847231
**Lamtor1**	5023.36247712195	0.0595390529801323	0.0940682269857652	0.6329347845489	0.526776231355131	0.783131717847231
**Cd4**	316.986850874046	-0.192339819429847	0.304037965875668	-0.632617768231293	0.526983281080692	0.783217841915531
**Nat10**	5726.86450510382	0.0732943688621587	0.115852955755938	0.632649968953443	0.526962248247106	0.783217841915531
**Ano10**	2973.92409915283	0.0702232978934803	0.110983915812727	0.632734008160015	0.526907357625568	0.783217841915531
**Slc4a7**	1725.65208056995	0.0789843895570772	0.124881370501372	0.632475358333845	0.527076305364064	0.783282216695422
**Prss12**	3307.84516073335	-0.118819967824496	0.187972346050159	-0.632114086573084	0.527312331090896	0.783485186327133
**H2-Q5**	13992.5376189951	-0.0516488565560774	0.0817067069798791	-0.632125053929739	0.527305165114279	0.783485186327133
**Gm10233**	1224.4584696592	0.137054569429576	0.2169575851546	0.631711351930438	0.527575508849216	0.783802309347266
**Ddx17**	4263.3787902834	-0.0865594322843064	0.137042075031833	-0.631626690300769	0.527630841781708	0.783810613175674
**Ccdc73**	77.3821678865343	-0.244743187798911	0.387565628508749	-0.631488372022615	0.527721249856136	0.783871015646978
**Lynx1**	285.695867550301	-0.141268628602993	0.223836934956676	-0.631122958462311	0.527960130726339	0.784151925709768
**Gm26590**	27.5510778246043	0.301148628358681	0.477274650031054	0.630975536494735	0.528056520110511	0.784221167519446
**Gm568**	69.0967485485973	-0.285384285710736	0.452393570620009	-0.630831877914654	0.528150457492916	0.784286755132964
**Car2**	737.620683553161	0.142399410857616	0.225822076601206	0.630582328357065	0.528313656513942	0.78445517204016
**Ccdc97**	5429.21518341518	0.0517633707846257	0.0821093802739896	0.630419698844362	0.528420025874227	0.784465267354456
**Pdzd11**	1593.22170746488	0.071458870851399	0.113337627668174	0.630495558461957	0.5283704078216	0.784465267354456
**Ddit3**	1929.4166209918	0.121172237159259	0.192253057092913	0.630274696233818	0.528514875390658	0.784532161236985
**Banf1**	6645.48562576786	0.056486814693346	0.0896457914553165	0.630111171716316	0.528621850909552	0.784617040766938
**Secisbp2l**	3734.10570841858	-0.0790771860287995	0.125522390737877	-0.629984702840253	0.528704592690397	0.784665938308617
**Ffar4**	17.666699797366	0.626316441741843	0.994303890959393	0.629904446152289	0.528757103742379	0.784669964557229
**Rnd1**	37.628675860807	-0.394445478071807	0.626551925463633	-0.62954954256972	0.528989345004595	0.784940682666673
**Sod2**	5106.58640778335	0.035726114639816	0.0568314527489175	0.628632788918043	0.529589488970143	0.785757210706455
**Gabrr2**	1479.91047774441	-0.0910348048686279	0.144901406737291	-0.628253423610137	0.529837938043608	0.785977818654974
**Gm23301**	20.002438406693	-0.288714171832575	0.459495290062501	-0.628328903639695	0.529788500908424	0.785977818654974
**Epb41l2**	3032.78769529262	-0.0650038880453971	0.103512460921274	-0.627981283285648	0.530016201183518	0.786168246450973
**2410004B18Rik**	1173.51347527571	0.0865861596853318	0.137903930685614	0.62787303635838	0.53008711585676	0.786199424374527
**Enkd1**	145.042533839358	0.162202481437411	0.258437163551332	0.627628314784511	0.530247455497762	0.78636321470854
**Klhl3**	1086.33049223057	-0.0956047774342925	0.15259492506971	-0.626526585930807	0.530969604478747	0.786524302031705
**Pcdhgc4**	20.8469734620249	-0.61622251191333	0.982926617454278	-0.626926263843897	0.530707570380058	0.786524302031705
**Eef1aknmt**	3272.70658870833	0.0610846317391777	0.0974100033854036	0.627087872048374	0.530601636552575	0.786524302031705
**Rps6**	41632.8253009455	-0.0341532330887056	0.05445075706203	-0.627231556207721	0.530507460912884	0.786524302031705
**Wdr95**	407.60991389054	0.182054678801464	0.290377248161912	0.626959171057203	0.530685998902795	0.786524302031705
**3830403N18Rik**	46.4674490565475	-0.370539943732918	0.591634345631091	-0.62629890652759	0.531118903432213	0.786524302031705
**Cxcl10**	88.7079873821608	0.302085840988722	0.481756498803702	0.627050889274689	0.530625877733339	0.786524302031705
**Gadd45a**	201.308170994904	0.136677977618735	0.218149641161692	0.626533130611156	0.530965313171634	0.786524302031705
**Suox**	2880.0629451091	0.0565720029057866	0.0902221979473169	0.627029757563882	0.530639729235111	0.786524302031705
**Gm16089**	19.6204839082663	0.279938304774403	0.446910162392723	0.62638608009188	0.531061737546843	0.786524302031705
**Hmgb1-ps3**	478.902952169349	-0.0708154094312665	0.112981556395896	-0.626787342025312	0.530798641909889	0.786524302031705
**Lsm5**	385.403572655882	0.117406534927725	0.187429652145686	0.626403205595592	0.531050507506956	0.786524302031705

**1700030C10Rik.1**	219.416653825615	0.238791801064612	0.381084724902676	0.626610791407596	0.530914392802866	0.786524302031705
**Gm29562**	82.0177295318726	-0.17005282786644	0.271161779115363	-0.627126833365747	0.530576099099489	0.786524302031705
**9530056E24Rik**	140.935102274484	0.218449606776151	0.3486146972011	0.626621908169688	0.530907104004567	0.786524302031705
**AC167036.1**	24.4997317308297	0.252502370349501	0.403201084018224	0.62624427452702	0.531154731098519	0.786524302031705
**Gm10651**	38.7602012977433	-0.264488585032449	0.422406742153986	-0.626146693785562	0.531218727599376	0.78654515042129
**Zbtb40**	1216.22574426375	0.116153648956995	0.185582519878493	0.625886797059575	0.531389195072871	0.786687900565774
**Gm50315**	16.9079481477355	0.323087838287093	0.516240542273659	0.62584747192487	0.531414991027613	0.786687900565774
**Mefv**	27.5876564304031	0.545941075762384	0.873090499498462	0.62529723559699	0.531775993976793	0.786852693876639
**Prpf18**	6014.05592028387	-0.0361505211364625	0.0578111652271071	-0.625320748932283	0.531760564639767	0.786852693876639
**Cklf**	2953.43946669158	-0.0724460259653638	0.115857277339284	-0.625304060557268	0.531771515447462	0.786852693876639
**Gm45051**	229.852754391451	-0.129923490985987	0.207758864533309	-0.625357147950515	0.531736680227261	0.786852693876639
**Gm33782**	131.513784238897	0.260866834262377	0.417163413760132	0.62533488234511	0.531751290471711	0.786852693876639
**Il15ra**	1299.09941470046	0.0867880530747949	0.138837792343828	0.625103954835777	0.53190283247473	0.786906822606171
**Zfp691**	112.341719136842	-0.132396072627814	0.211803456676915	-0.625089291294103	0.53191245589759	0.786906822606171
**Best1**	29.9900250146119	-0.382182679780062	0.611677548341352	-0.624810704294122	0.532095304370658	0.787103427616265
**Mpv17l**	18.3285880628633	-0.34625129813905	0.554830580233	-0.624066715993993	0.5325837714564	0.787172020511927
**Gpd1**	40.6777614128852	0.335119053302037	0.536832581664599	0.624252448059145	0.532461807424618	0.787172020511927
**Tmem216**	751.879471811256	0.116064745478406	0.185984720851214	0.624055271568555	0.532591287090033	0.787172020511927
**Plxna4**	18.3087944444382	0.764162564695672	1.22424647676362	0.624190127723126	0.532502729527609	0.787172020511927
**Sh2b1**	5902.3882282706	0.0603168046799655	0.0966486869244268	0.624083022743283	0.532573062793668	0.787172020511927
**Mmrn1**	85.9850816905596	0.297791672167038	0.476891534818606	0.624443191847195	0.532336567079764	0.787172020511927
**Gm16341**	20.7794112704778	-0.320791822951692	0.513574173125202	-0.624626080785195	0.532216498153555	0.787172020511927
**Gm37285**	21.2018478598884	-0.436697887498348	0.699223509693189	-0.624546917322568	0.532268468295573	0.787172020511927
**Gm46367**	31.8479560794919	0.320098065722851	0.512837093701287	0.624171047013419	0.532515259026886	0.787172020511927
**Mtif3**	3155.03162594384	0.0802850461100628	0.128695638268465	0.623836574341273	0.532734917390182	0.787248074329019
**Uqcc3**	1835.39656880086	0.0796071637644678	0.12761141761171	0.623824774102056	0.532742667802902	0.787248074329019
**Abcc5**	4525.21759796364	0.0909010729368346	0.145827846013546	0.623345097810679	0.533057768114848	0.787425673042062
**Taf8**	8585.56014624539	0.0405196714642511	0.064982678419043	0.623545727108364	0.532925962862746	0.787425673042062
**Zdhhc9**	1932.57147429696	0.0750512181321434	0.120402214188589	0.623337524462705	0.533062743817965	0.787425673042062
**Ccdc138**	273.170843880869	-0.146570159616361	0.235085790091447	-0.623475198391811	0.532972295469025	0.787425673042062
**Gm49730**	21.6523150475572	-0.416960063732113	0.669012248973954	-0.623247278912447	0.533122037112909	0.787439439331469
**Cfh**	24.124632401504	-0.803015384460036	1.28880944541311	-0.62306758172667	0.533240112015174	0.787540017342122
**Naprt**	63.8096612625025	0.334901006444627	0.537640643073545	0.622908648665565	0.533344554304657	0.787620444440345
**Riox1**	1827.30379325509	0.158394847152672	0.254362453856289	0.622713158924639	0.533473033866182	0.787736350354178
**4930562C15Rik**	22.2931416466662	0.427819331223983	0.687249006804882	0.62250993015323	0.533606616247476	0.787840085829995
**Zfp536**	92.4200925619843	-0.264138115638842	0.424445609771846	-0.622313223550186	0.533735927699959	0.787840085829995
**Gm10481**	137.469496928996	0.114244534302111	0.183576899602501	0.622325219292211	0.533728041457376	0.787840085829995
**Gm49797**	214.556572352409	-0.140307064994651	0.225464576878125	-0.622302034924512	0.533743283381049	0.787840085829995
**Cyp17a1**	22.2685721469637	-0.740943905910052	1.19215795239044	-0.621514879319771	0.534260907590568	0.788004707718315
**Cwc25**	2020.02049224224	0.0748075084169593	0.120352524080193	0.621569917113778	0.534224707150191	0.788004707718315
**Pdcd1**	921.209009506939	-0.203765367927722	0.327863721870693	-0.621494097502149	0.534274576900712	0.788004707718315
**Iars2**	5636.33840943907	-0.0636051025615538	0.102374887048812	-0.621295948597501	0.53440491887275	0.788004707718315
**Setmar**	496.689607134767	0.11313224982716	0.182027305583587	0.621512522335338	0.534262457896097	0.788004707718315
**Sugp2**	5098.5462134352	-0.0692178384327728	0.111274747470557	-0.622044444100739	0.53391264416357	0.788004707718315
**Eipr1**	6082.53847310927	0.0504619141830106	0.0811855757755243	0.621562558385202	0.534229547192796	0.788004707718315
**Zfp335**	4595.3983528327	0.0725643435806269	0.116794888277216	0.621297255821617	0.534404058930628	0.788004707718315
**Smim12**	2780.6439912053	0.0558812577621104	0.0899320068297478	0.621372298161882	0.534354694374185	0.788004707718315
**2610507I01Rik**	474.598151067629	0.0825815947049822	0.132802069675638	0.621839666404922	0.534047300740989	0.788004707718315
**Gm10138**	126.747464857256	0.195197759189843	0.314002281357105	0.62164439807955	0.534175720171697	0.788004707718315
**Gm14536**	17.3148221539538	-0.283316272001031	0.456105791262248	-0.621163505109129	0.534492048895011	0.788059438049844
**Dop1a**	3957.21761991767	-0.091691858197308	0.147647665045763	-0.621018003697439	0.534587777539882	0.788126834833076
**Fcf1**	4239.30832261011	0.0716258509616942	0.1153684707775	0.620844243483402	0.534702109618585	0.788173635971923
**Tubb2a**	864.2757522804	0.0923151732747774	0.148699321145726	0.620817718356011	0.534719563910634	0.788173635971923
**Arhgap27os3**	1358.27648925451	-0.0877608875129743	0.14138136476341	-0.620738720833788	0.534771548241098	0.78817653031849
**Zfp639**	3023.47790706993	-0.0827839726613814	0.133395274365764	-0.620591494376264	0.534868437434323	0.788191085212735
**Fbxo34**	3014.92414590018	0.0716292231211142	0.115435612280398	0.620512350617794	0.534920525311044	0.788191085212735

**Nanp**	827.885035784392	0.086808467713341	0.139918942989794	0.620419693419734	0.534981510208174	0.788191085212735
**H2aw**	1330.69150207231	0.0858191537309613	0.13831923827567	0.620442642692437	0.534966405183087	0.788191085212735
**Mad2l1bp**	2704.52954419712	0.111415228947834	0.179620387500061	0.620281642292949	0.535072378877674	0.788251259720972
**Nuak2**	602.574053770365	0.106396793364096	0.171569433919322	0.620138394896884	0.535166676086575	0.788306040734714
**C330008A17Rik**	25.7189128671291	0.306265139262894	0.493917749738783	0.620073158789835	0.53520962262994	0.788306040734714
**Nploc4**	6541.93958396932	0.0638538713690257	0.103032023277161	0.619747815659759	0.53542382999657	0.788547835242168
**Mus81**	4985.3698458599	0.0529065964952512	0.0853791681098964	0.619666338598569	0.535477481620592	0.788553147466885
**Slc4a8**	318.459081723433	0.103298749740905	0.166745649501797	0.619498919759175	0.535587733330365	0.78864180114817
**Adam19**	5427.50718291467	0.0739231750129639	0.119492362699021	0.618643512800584	0.536151229972093	0.788808116775935
**Cnot2**	7592.80698974965	-0.0405430859352038	0.0655331968827298	-0.61866485786974	0.536137165345303	0.788808116775935
**Prdx5**	6098.02177856111	0.0605582784691347	0.0978078263059809	0.619155754261268	0.535813756677791	0.788808116775935
**Parp16**	1154.00014028566	-0.0792298632683138	0.128042510295944	-0.618777803443486	0.536062746689612	0.788808116775935
**Gm12335**	23.4885733118178	-0.317937364811396	0.513609799973473	-0.619025113671539	0.535899814732823	0.788808116775935
**C230085N15Rik**	7396.15117273686	-0.104032667618992	0.168132784256992	-0.618753017614801	0.536079077367151	0.788808116775935
**Gm6063**	62.1665440890811	-0.164300255883368	0.265374592206014	-0.619125796925652	0.535833490128883	0.788808116775935
**Gm18990**	53.7693200096246	-0.26300340137103	0.425127170780439	-0.618646417937047	0.536149315717663	0.788808116775935
**Eef1akmt4**	1554.72549606895	-0.0618380995655795	0.0999137263960207	-0.618914955893811	0.535972385368746	0.788808116775935
**Taf3**	4105.51724248378	-0.045640350295199	0.0737873935816482	-0.618538588772572	0.536220368887575	0.788836182668427
**Olfr77**	42.0471475577059	-0.219377319514554	0.35471530732791	-0.618460255259732	0.536271989104506	0.788838473890935
**Brdt**	48.1556870045347	0.33161864823533	0.536387230122957	0.618244860451492	0.53641394286366	0.788899990451996
**Gm12799**	23.4055420937563	0.267071436698437	0.431938150981223	0.618309441043207	0.536371379696548	0.788899990451996
**Ilkap**	8053.33468783312	0.0513445770625837	0.0830899981632565	0.617939321188828	0.536615337836378	0.78897526157393
**Ltbr**	19.8342006228999	0.731586611316587	1.18363332746048	0.618085512078498	0.536518971955664	0.78897526157393
**Mtarc2**	10535.6934805969	0.0406263496251066	0.0657446657000222	0.617941382658713	0.536613978899383	0.78897526157393
**Them6**	195.072649029367	-0.185693661980408	0.300547607720511	-0.617851073208643	0.536673513215025	0.78898717556719
**Abca5**	23.5096049874877	0.51099157643162	0.827254883142605	0.61769544894126	0.536776112535054	0.789064390821424
**Gp5**	140.280620791923	0.16954899023	0.274638778698597	0.617352695178097	0.537002116592462	0.789175746236469
**Gm10243**	307.293691558676	-0.101191757450634	0.163894126075693	-0.617421501755952	0.536956743258206	0.789175746236469
**Peg13**	1139.24733794134	-0.0855506275920962	0.138548604403178	-0.617477368037161	0.536919904601615	0.789175746236469
**Zc3h10**	1995.55714752315	-0.0698265299706682	0.113129180591852	-0.617228283678539	0.537084162545155	0.78922271278784
**Pfkfb2**	476.969605062752	-0.0873940125427085	0.141630338069343	-0.617057148447388	0.537197031804284	0.789241364115627
**Gm44834**	67.3906775843832	-0.255083094980041	0.413382463804358	-0.617063173489536	0.537193057890292	0.789241364115627
**Sec14l1**	2927.43498254464	0.0556826426072776	0.0903733557481809	0.616140035370972	0.537802100797417	0.789277994178009
**Bcl6**	1457.31915245377	0.0791561578579049	0.128305794977427	0.616933614509237	0.537278513867904	0.789277994178009
**Ripply3**	49.4047334770439	-0.31136422543767	0.50497558404992	-0.616592633926018	0.537503454341096	0.789277994178009
**Mob2**	8243.54537087918	-0.0288051824792102	0.0467421223431911	-0.616257479018947	0.537724597783903	0.789277994178009
**Psmd13**	9823.34523519339	0.0422800399976992	0.0686160227037476	0.616183193541324	0.537773619352292	0.789277994178009
**Birc3**	6751.58770400431	0.0549693555624477	0.0892312748273284	0.616032390760067	0.537873142189723	0.789277994178009
**Mcoln3**	541.337833755474	0.0941109302430307	0.152648045169486	0.616522341563817	0.537549831182479	0.789277994178009
**Rtl8a**	120.168694014799	-0.146372535234551	0.237570103647151	-0.616123548323023	0.537812981323079	0.789277994178009
**Slc25a16**	1431.8159749941	0.0574295564296777	0.0931720926840822	0.616381523428948	0.537642744908789	0.789277994178009
**Gm12207**	32.730412552941	0.286188839875008	0.464309037784866	0.616375768260646	0.537646542418893	0.789277994178009
**Mfsd14a**	11359.1578406876	0.0589052679245174	0.0955544919429902	0.616457339961176	0.537592719124342	0.789277994178009
**Rnasek**	1798.25256107787	-0.0469211185291417	0.0761066323727946	-0.616518128135102	0.537552611143163	0.789277994178009
**Ifngas1**	832.221162455124	0.105439195452113	0.171155468556367	0.616043392252981	0.537865881402262	0.789277994178009
**2900026A02Rik**	170.365701398727	0.242267305149805	0.393351057141896	0.61590607359779	0.537956512770993	0.789326824819121
**Ube2g1**	7834.76501687491	0.060322696439485	0.0980240989688293	0.615386390429021	0.538299577050674	0.789756651358238
**Rps17**	4868.89692460438	-0.0474824743396247	0.0771774122759001	-0.615237968460001	0.538397576657501	0.789826889059887
**Poldip2**	3720.47040230107	0.046544448906909	0.0756947537075263	0.614896629253199	0.538622989026864	0.790010466172978
**Vmac**	2385.91444240527	0.0597107029176776	0.0970955569687631	0.614968437092207	0.538575564891428	0.790010466172978
**Hexa**	8178.6790973927	0.0301701752261431	0.0490954992438633	0.614520183943627	0.538871639504903	0.790194420788301
**Gm10705**	252.01957913161	0.326107775589208	0.530706146607804	0.614478987427678	0.538898854210261	0.790194420788301
**Gm2574**	1117.072411087	0.0554569961124507	0.0902306994198718	0.614613390664211	0.538810069038907	0.790194420788301
**Ccnq**	3218.16259946297	0.0583690312881354	0.0950130666783008	0.614326358770881	0.53899968776816	0.790268733615232
**Prdx2**	5944.45455689457	0.0385020936360556	0.0627153151136433	0.613918523191471	0.539269169218566	0.790285982093902
**Arnt**	4775.06415866782	0.0710233903150159	0.115839805773276	0.613117311798887	0.539798774160605	0.790285982093902

**Rpp40**	694.410476692072	0.0941061886297659	0.153441021437891	0.613305280086772	0.539674502762681	0.790285982093902
**Zhx1**	3143.52643967577	-0.0516609340867216	0.0841457553505833	-0.613945811900819	0.539251135823259	0.790285982093902
**Tbck**	4004.03009265342	-0.0928708735470919	0.151392419406627	-0.613444675176561	0.539582353794299	0.790285982093902
**Dipk1a**	6248.91826155142	-0.0414100612920969	0.067515524626074	-0.613341324405626	0.539650674433576	0.790285982093902
**Ice2**	2374.30106105833	-0.0578270044247736	0.0943222064525773	-0.613079428478463	0.539823821679148	0.790285982093902
**Ppm1h**	21358.5279076957	0.0611258084997214	0.0996212287147871	0.613582158022995	0.539491476657716	0.790285982093902
**Trmt12**	1108.1479422887	-0.114288328935843	0.186376370422934	-0.613212547687752	0.53973580910584	0.790285982093902
**Fcmr**	224.648018106466	0.182446055667844	0.297553592129728	0.613153598187114	0.539774783039805	0.790285982093902
**Zfp322a**	2561.43466371992	0.0519647625161589	0.0847303434257236	0.613295785372478	0.539680779655784	0.790285982093902
**Gm10277**	17.8460090376838	-0.58961253697212	0.960016527460859	-0.614169152411972	0.539103555452622	0.790285982093902
**5430403G16Rik**	1103.96152896401	-0.0748980586024385	0.121982083782654	-0.614008682913558	0.539209589466763	0.790285982093902
**A230056P14Rik**	45.2550121761361	0.278322955421785	0.45335564000218	0.613917487428736	0.539269853695102	0.790285982093902
**Trav15d-2-dv6d-**	58.9421359444215	-0.17470609636835	0.28501906104445	-0.612962851425238	0.539900903205338	0.790285982093902
**Magef1**	845.037206903137	-0.122865501314993	0.200409655068987	-0.613071766790372	0.539828887469361	0.790285982093902
**Gm19696**	41.884103689833	-0.230367401598662	0.375838402894421	-0.612942689795796	0.539914234768094	0.790285982093902
**Gm49971**	18.4497140708447	-0.521039128002166	0.850052260901846	-0.612949523185057	0.539909716277653	0.790285982093902
**Pemt**	363.08877243495	0.0997407175506116	0.162824607586432	0.612565379576709	0.540163755648706	0.790291193027747
**Mcoln2**	2005.31637995757	0.0572038006027674	0.0933504640009358	0.612785391213416	0.540018251656913	0.790291193027747
**Rdh14**	2874.3684286281	-0.0550002632738428	0.0897744547281646	-0.612649371587748	0.540108205486477	0.790291193027747
**Coa5**	9552.06656349783	0.0469862422303969	0.0767144527437065	0.61248227094016	0.540218724382932	0.790291193027747
**Stap2**	42.9657483131768	0.295027034080935	0.481654602354254	0.612528215528072	0.540188335909577	0.790291193027747
**Traj4**	28.8674008378888	-0.672807394015106	1.09784172672008	-0.612845529223225	0.53997848299005	0.790291193027747
**Wdr45b**	4671.22355288034	0.0480068780536228	0.078404066422087	0.612300869640849	0.540338714409711	0.790319977996271
**Cel**	22.3528950676264	1.8809774764224	3.07178139041185	0.612340930996464	0.540312214207222	0.790319977996271
**Tert**	124.348307321761	0.348049059538024	0.568572542165647	0.612145388189751	0.540441570055282	0.790324621393849
**Katnal1**	332.095521942383	-0.125745852935109	0.205418601446885	-0.612144431173257	0.540442203180727	0.790324621393849
**Bfar**	8228.63071804012	-0.0531993243974117	0.0869468871009778	-0.611860023644405	0.540630372737183	0.790471372135309
**Lyz1**	27.3911507477847	0.320454309495574	0.523754131896802	0.611841110131261	0.54064288744699	0.790471372135309
**Tns1**	891.002679539831	-0.135730847044489	0.221881004124318	-0.611728108858026	0.540717661244956	0.790507347210685
**H2-Oa**	1933.83606183158	-0.0831770900598053	0.136009507957725	-0.611553495845735	0.540833214161614	0.790602927502047
**Sirpb1b**	22.4672764511096	-0.565215723092816	0.924514411507994	-0.6113649674437	0.540957989661284	0.790711970602305
**Msc**	17.6683475302904	1.03012006916437	1.68555299487547	0.611146651749432	0.541102497531007	0.790849833373163
**Med11**	2818.84982625239	0.0665972208938923	0.109019637418792	0.610873623052545	0.541283248253435	0.790951780788549
**Atat1**	402.045112766941	-0.135944793720758	0.222666823445432	-0.610530080850027	0.541510723228519	0.790951780788549
**Acad10**	962.819917522367	-0.0725788938165973	0.1188651163301	-0.61059876991193	0.541465237247927	0.790951780788549
**Zfand6**	13925.8130309345	0.0376822767474407	0.0616874441989029	0.610858129021187	0.541293506531176	0.790951780788549
**Bag3**	326.883932024362	0.117933074379333	0.193186339256298	0.610462804116146	0.541555275812918	0.790951780788549
**Npm1**	27369.5909833489	0.0336204607394432	0.0550762522790468	0.610434794457388	0.541573825152482	0.790951780788549
**2610008E11Rik**	1657.51736272977	0.107469632131006	0.176031155017225	0.61051483824264	0.541520817160352	0.790951780788549
**AU022252**	1021.20649163049	0.0637863429401683	0.104480265071248	0.61051092181543	0.541523410704847	0.790951780788549
**Cnst**	2822.8687288486	-0.0932038071538762	0.152723373096433	-0.61027860545632	0.541677266815902	0.790956231416752
**Trbj2-4**	544.238735876986	-0.0671446066673396	0.110017070262974	-0.610310804558274	0.541655941020401	0.790956231416752
**Klhl9**	2477.01409456191	-0.0600380379064159	0.0984108008586917	-0.61007569679902	0.541811664941579	0.791079170170911
**Gm10557**	672.142410533241	0.0631619431739438	0.103639988365036	0.609436031114534	0.542235461362975	0.791624586741073
**Smc2**	2354.32828699781	-0.0705375914244685	0.115792870927768	-0.609170416618051	0.54241148696182	0.791667294626299
**Klhl25**	5746.11797967697	0.0580901823956427	0.095348250478505	0.609242247278972	0.542363881195885	0.791667294626299
**Gpr182**	17.1971855829776	-0.598022905529672	0.981710120694141	-0.609164449793821	0.542415441567908	0.791667294626299
**Snrnp200**	14655.6897317422	0.0823062655520993	0.135179418156633	0.608866842855699	0.542612703457084	0.791808517167372
**Rps8**	14436.2572460195	-0.0572887910763068	0.0940899635048053	-0.608872497579202	0.542608955020944	0.791808517167372
**Cenpe**	271.333339083869	0.168163008530993	0.276357397216198	0.608498307716498	0.542857028008338	0.792091692779644
**Rprd1b**	4992.11947576332	0.0398864470463215	0.0655907708384316	0.608110661552872	0.543114081562342	0.792218622939791
**Vbp1**	5027.76543564383	0.0490130463156704	0.0806108249397143	0.608020651721718	0.54317377700172	0.792218622939791
**Adck2**	3646.62701000843	0.0581061382764779	0.0955564450536833	0.608081833138874	0.543133200505314	0.792218622939791
**M6pr-ps**	52.7280586739575	0.224579356090036	0.369221085949631	0.608251707814631	0.543020544812615	0.792218622939791
**Gm42992**	82.3207780899601	-0.325611848213927	0.535556336776931	-0.607988041320759	0.543195405358984	0.792218622939791
**Rps19**	47659.5668773597	-0.0419196951042037	0.0690194765073994	-0.607360374570641	0.543611779418781	0.792744792365914

**Zfp455**	231.106320148022	0.138571708770248	0.228179508759163	0.607292519489583	0.543656801821798	0.792744792365914
**Tmem269**	17.427416920817	-0.54951675482522	0.905494384144829	-0.606869312993252	0.543937644670088	0.793035296379464
**1700113A16Rik**	129.687923981444	-0.135863284994926	0.22388627162334	-0.606840624973641	0.543956684855572	0.793035296379464
**Edem1**	19323.9271628269	0.0436916172977289	0.0720277689261479	0.606594067109411	0.54412033851893	0.793053757658199
**Ttk**	51.142505424699	0.35550929097472	0.586023826592817	0.606646478935294	0.544085547933376	0.793053757658199
**Gm34086**	17.9435553395649	0.355297751747564	0.585662904454534	0.606659136245749	0.544077146269366	0.793053757658199
**Axin1**	2351.41333662721	-0.0647506351843475	0.10680057734929	-0.606276078195546	0.54433144093842	0.793141671140402
**Txnl1**	3074.01387822583	0.048489481591224	0.0799616674060293	0.60640908530589	0.544243136923851	0.793141671140402
**Trub1**	1412.67258216694	0.0626882516684705	0.103403775016736	0.606247225097193	0.544350597579241	0.793141671140402
**Bmyc**	2578.85336664731	-0.0407400529494434	0.0672056379473118	-0.606199928961065	0.544381999960872	0.793141671140402
**Manba**	2608.18820649018	-0.0646614723558471	0.106693036929754	-0.606051474553298	0.544480572464643	0.793211943447243
**Gm28151**	25.1693642311943	0.383171787409922	0.6324055556904	0.60589566926181	0.544584035433306	0.793289326645336
**Tsc2**	4906.5939519333	-0.0624668162590505	0.103154435566878	-0.605565974121894	0.544803002675445	0.793471175288015
**Etv1**	25.2393424911381	-0.46093695440496	0.761418884435787	-0.605365803011985	0.544935967780463	0.793471175288015
**Mns1**	925.172995624206	0.0762659741407033	0.125991022483234	0.605328638799265	0.544960656150721	0.793471175288015
**Gm2619**	50.2693905106419	0.235230345420351	0.388594151740191	0.605336813142836	0.544955225846223	0.793471175288015
**A830073O21Rik**	24.218722320763	-0.549189317476686	0.907225925323965	-0.605350114174233	0.544946389885206	0.793471175288015
**Gm42986**	31.1071758263387	-0.536553661811063	0.886664140929038	-0.605137432589601	0.545087684160305	0.793582799530068
**Fcgr1**	17.8765018818126	0.687973051698993	1.13739869672339	0.604865342013226	0.545268473108405	0.793772665444303
**Pvt1**	1005.61930047253	0.0633788384801529	0.104801979094031	0.604748488797981	0.545346124659212	0.793812368245284
**Slc30a6**	2349.0968741846	-0.050443595806898	0.0834324188262615	-0.604604259549768	0.545441975757058	0.793878552743762
**Tma7-ps**	167.022422019419	0.170199564309936	0.281609339362024	0.604381817362724	0.545589821575522	0.793947064884143
**Gm48880**	23.1620832464864	-0.418942705934778	0.693162746701517	-0.604392991297294	0.545582394364838	0.793947064884143
**Cdv3**	6199.20878888943	0.0789693736139717	0.130706362929363	0.60417390434656	0.54572802857816	0.794052319415726
**Irgq**	613.660856198525	-0.1371788211913	0.227071616871738	-0.604121391661144	0.545762938330413	0.794052319415726
**Rack1**	52982.9505535836	-0.0432510340551894	0.0716243897905198	-0.603859023185899	0.54593737407884	0.7940757484987
**Zbtb43**	1851.6288571936	-0.0795115376362497	0.131699316050207	-0.603735387706476	0.546019582740416	0.7940757484987
**Emc8**	3513.75143746707	0.0588781275646473	0.09749899567398	0.603884451912978	0.545920466573405	0.7940757484987
**Ehmt1**	6619.9112542757	0.0433899074356119	0.071880151095516	0.603642407177948	0.546081412117276	0.7940757484987
**AB124611**	4654.08564195787	-0.0446939653543804	0.0740361097761428	-0.603677928101814	0.546057791315007	0.7940757484987
**Gm49527**	421.821635873653	0.893367433716278	1.47957150543193	0.603801459028153	0.545975649321441	0.7940757484987
**Pnpla6**	4809.52891319494	-0.0612958306151738	0.101590935115201	-0.603359251941783	0.546269723579232	0.794276278899876
**Mapk7**	3816.9107768538	-0.0545129850340317	0.0904149607570145	-0.602919965651841	0.54656193316083	0.7944745421916
**Lcmt1**	980.99974570808	-0.0625362077852179	0.103734089340206	-0.602851079938864	0.546607762384917	0.7944745421916
**Pitpnb**	7497.14752553418	-0.0568096407976801	0.0942115967104141	-0.603000509292933	0.54650835040557	0.7944745421916
**Gmfg**	8539.75681677658	-0.0535467907807755	0.0888012555319822	-0.602995875001907	0.546511433360297	0.7944745421916
**Rxrb**	8146.65042679334	0.0579504318475865	0.0961555520708485	0.602673798855504	0.546725715106768	0.794572688889259
**Mapkapk3**	6602.95315071079	0.0570935790067902	0.0947584187377714	0.602517219760573	0.546829904387713	0.794587068129976
**Foxo4**	103.427963919763	-0.233493348446282	0.387536092014875	-0.602507361913841	0.546836464225517	0.794587068129976
**Amy2a2**	38.0504373334928	1.84705158589851	3.06698419647764	0.602237073154733	0.547016341240428	0.794775148817467
**Sult2b1**	129.068471419767	0.170152523791826	0.28264963838815	0.601990948094415	0.547180162800459	0.794856585550211
**Wdr70**	1946.74990436863	0.0592357576719586	0.09841044627769	0.601925506005835	0.547223725331516	0.794856585550211
**Gm10874**	167.210126878336	-0.244208766848373	0.405677544084753	-0.601977532178496	0.547189093171036	0.794856585550211
**Pspc1**	1960.04789484694	0.0479608517300128	0.0797169719347579	0.601639156204591	0.547414358653534	0.79489203005722
**Hprt**	7825.74399584288	0.0445541328751713	0.0741147345585382	0.601150812190807	0.547739542525186	0.79489203005722
**Itgav**	797.45770734334	-0.0846823156901021	0.140857961353625	-0.60118941717115	0.547713832340967	0.79489203005722
**Rmdn1**	790.450080285762	0.0709653901207921	0.118007970591778	0.601360990829009	0.547599574760501	0.79489203005722
**D6Wsu163e**	1388.24584799165	0.0617542983792897	0.10270283828569	0.601291058845978	0.54764614376833	0.79489203005722
**Sh3bgrl**	2535.30786376574	0.0514470190623705	0.0855247292012841	0.601545535926679	0.54747669215192	0.79489203005722
**Crtap**	611.103528483592	0.0897659756816543	0.14936536653377	0.600982528713303	0.547851623096448	0.79489203005722
**Zfp612**	18.0326619867967	0.742015970438101	1.23483282836747	0.600903987480714	0.547903937199433	0.79489203005722
**Mospd2**	453.534931732344	0.0906934705363043	0.150921265753467	0.600932347628555	0.547885047018873	0.79489203005722
**Frat1**	2107.56383559229	-0.0765670768717284	0.12740860367094	-0.60095687940729	0.54786870710528	0.79489203005722
**Plxnc1**	1460.7354136557	-0.0701081005966641	0.116529746623732	-0.601632652845622	0.547418688554086	0.79489203005722
**Gm5559**	4484.21295703304	0.0390968008672419	0.0650527510446182	0.601001498621116	0.547838988145338	0.79489203005722
**Gm38319**	55.6234146694984	-0.238807398549617	0.39723176780271	-0.601179004062495	0.547720767214932	0.79489203005722

**Pex7**	1801.35242182131	0.0761593353260371	0.126759544713643	0.60081736249594	0.547961638525395	0.79490255369588
**Rhot2**	3484.21770328546	0.0509190193512346	0.084785227143105	0.6005647571751	0.548129917311467	0.795000285997495
**Stx6**	6937.52912972009	0.0457995505580308	0.0762585919892341	0.600582168688567	0.548118317416007	0.795000285997495
**Gm13597**	100.80459656209	0.171099198884316	0.284957112481909	0.600438421747337	0.548214088111011	0.795049184202964
**Gm6483**	319.775676364617	0.0878060139042329	0.146258616130542	0.600347632346409	0.54827458036373	0.79506373668243
**Lmln**	80.4228362267985	0.264717116426244	0.441773467244208	0.599214611229496	0.549029780514888	0.795576395438025
**Arap3**	19.3242885484022	0.441561278667694	0.736694330434622	0.599381942314106	0.548918215911389	0.795576395438025
**Abhd6**	2606.10248729558	-0.0686487171693353	0.114542977223104	-0.599327159408675	0.54895474006955	0.795576395438025
**Tmub1**	2292.58020738231	0.0732466500600389	0.122238445800647	0.599211234896533	0.54903203173149	0.795576395438025
**Senp1**	4861.33341801076	-0.044132009201448	0.073615179440902	-0.59949604873105	0.548842144190799	0.795576395438025
**Pgbd1**	62.5056795684401	-0.339430802852259	0.566240798723576	-0.599446037123086	0.548875484962365	0.795576395438025
**H2-Q1**	6746.89507128563	0.0417195344674613	0.0696211434015122	0.599236559889006	0.549015146054273	0.795576395438025
**Gm1859**	60.0394097598717	-0.186022410842323	0.310342712353252	-0.599409631474059	0.548899755805009	0.795576395438025
**Ceacam16**	20.1474563955304	-0.38890251641213	0.649165379805592	-0.599080802073266	0.549119003102979	0.795605211505382
**Gm11646**	27.932112353668	-0.268817552154131	0.448754766191569	-0.599029965599018	0.549152902223299	0.795605211505382
**Stradb**	1726.08160447284	-0.0640639596277946	0.106978009896868	-0.598851667642309	0.549271804227597	0.795654117545102
**4933412E12Rik**	722.881368135426	-0.0846379729579668	0.141339392706476	-0.598827908746834	0.549287649340332	0.795654117545102
**Fxyd5**	62221.051214153	-0.030533161126462	0.0509977453357366	-0.598715902545322	0.549362350752011	0.795689177387575
**Sptan1**	8827.32845818957	-0.0946935713157181	0.158206590792604	-0.598543782792548	0.549477154057153	0.795720790555325
**Gm5869**	305.529427426978	-0.0726603173187543	0.121397597661595	-0.598531756133267	0.549485176241194	0.795720790555325
**Gm2446**	81.921101068047	0.257136643530908	0.429681355572259	0.598435655157639	0.549549280880814	0.795740490611927
**9330020H09Rik**	94.9017907550392	0.273649225394166	0.457467933269746	0.598182310699379	0.549718293246831	0.795912078166895
**Mrpl28**	5096.06055448089	0.0558156939747461	0.0933556359818101	0.597882424427182	0.549918387915665	0.795951764601306
**Kcnh2**	166.014252731094	-0.262165048642432	0.438385035181826	-0.598024630411245	0.54982349860752	0.795951764601306
**Platr25**	1118.93814691152	-0.0793104380403406	0.132661998656363	-0.597838407709958	0.549947760437546	0.795951764601306
**Trbj1-7**	429.441206336653	-0.136936583773141	0.229047938609758	-0.597851194838508	0.549939227458644	0.795951764601306
**Cep128**	991.858470509413	-0.067547489013449	0.113034906163719	-0.597580794339878	0.55011968231235	0.7961274645661
**Edem3**	1337.36160707605	-0.0913658124820677	0.15295743667114	-0.597328344868287	0.550288183665522	0.79622506060768
**Lamb2**	22.5032998611478	-0.351204580335282	0.587886470817156	-0.597402045750622	0.550238988230749	0.79622506060768
**Chil1**	30.4947959221215	0.77956832843751	1.30576155764611	0.597021963062561	0.550492717133134	0.796447869237609
**Gm43533**	31.035613827135	-0.480952764415236	0.805721965472028	-0.596921500251607	0.550559791948365	0.796471781282629
**Gpr34**	1445.93032976522	-0.0836546408234401	0.140173881630911	-0.596791926214255	0.550646309051078	0.796523812697864
**Itih5**	154.821957437847	0.180070356695151	0.301837073869341	0.596581309203586	0.550786953146743	0.796654123437968
**Cmtr2**	856.174237836422	0.12926566444075	0.216746642786609	0.596390618921903	0.550914305979333	0.796740938185454
**Vrk2**	1986.81626479342	0.0507107306197399	0.0850366052980316	0.596340016655318	0.550948103221978	0.796740938185454
**Tfb2m**	1629.34778081747	-0.0854871509321424	0.143418465910544	-0.596067949753862	0.551129834126771	0.796824397965158
**2610044O15Rik8**	1512.12060478483	-0.075668545343604	0.126945558108638	-0.596070839113946	0.551127903983297	0.796824397965158
**Gm17655**	529.859316741267	-0.139653164656281	0.234306972841797	-0.596026498752873	0.551157524492115	0.796824397965158
**Gm4130**	290.964452848477	0.109125938291143	0.18313038581199	0.595892035105395	0.551247354475159	0.796881152703219
**Gm23833**	32.8518992308606	-0.372488514807008	0.62531630593505	-0.595680156221126	0.55138891720242	0.797012674833368
**Prodh**	152.094851567905	0.217724825528567	0.365649206245839	0.595447280643576	0.551544529070765	0.797066019530324
**Acly**	30664.1842141285	0.048190174129792	0.0809491094150172	0.595314444816512	0.551633302150136	0.797066019530324
**Gm12430**	80.5700735697521	-0.162614014570715	0.273127159363416	-0.595378412566962	0.551590552157663	0.797066019530324
**Trav15d-1-dv6d-**	56.4856884839452	-0.186954043272142	0.314076131372936	-0.595250719801283	0.551675891540088	0.797066019530324
**Gm45242**	42.617354914088	-0.338135338952423	0.568059397997075	-0.595246448073312	0.551678746525208	0.797066019530324
**Icam4**	30.8955590510931	0.341898138302244	0.575810066141877	0.593768949878	0.552666659305019	0.797165124228384
**Tmem147**	4064.43999954571	0.0591694916525883	0.0995146311471831	0.594580826663328	0.55212369948707	0.797165124228384
**Fbxw5**	11097.5909148288	-0.0552743611292128	0.092964325617105	-0.594576045835829	0.552126896000099	0.797165124228384
**Snrpb**	26103.5951873983	-0.0371721673734379	0.062481307757342	-0.594932607969779	0.551888519632115	0.797165124228384
**Gale**	1144.57607541841	0.0722714334624705	0.121671831769729	0.593986565429938	0.552521098597516	0.797165124228384
**Atp13a1**	28530.2302527584	0.0392194358333859	0.0659832488186884	0.594384734543078	0.552254816258116	0.797165124228384
**Tmem30a**	11199.5261590551	0.0417489124840063	0.0702908636860024	0.593945077563759	0.552548847934627	0.797165124228384
**Casp4**	225.991239112164	0.197411710527185	0.332197177090272	0.594260650425515	0.552337792856313	0.797165124228384
**Stard9**	77.3306106849081	0.268306453063294	0.45168517807903	0.594012082053197	0.552504032034308	0.797165124228384
**Bicral**	1066.18188587354	-0.112220281453718	0.188968966228809	-0.593855613931009	0.552608688395867	0.797165124228384
**Gm8894**	77.1105523175147	0.262934339884413	0.442371200418685	0.594374904233271	0.552261389685792	0.797165124228384

**Mapk1**	21849.9436222546	0.0499951209292449	0.0841554028805721	0.594080940949148	0.552457977674081	0.797165124228384
**Nr6a1**	64.2499489933499	0.299851115180553	0.504437735793794	0.594426415598548	0.552226944989103	0.797165124228384
**Nfxl1**	867.049467089674	0.0852469337526451	0.143488603716562	0.594102469078563	0.552443579569159	0.797165124228384
**Trav13-1**	544.067371450067	0.0853274373605854	0.143711507962106	0.593741159428128	0.552685249393229	0.797165124228384
**Gm13771**	22.0525439254133	-0.336084661736357	0.565700286992029	-0.594103749749543	0.55244272305674	0.797165124228384
**Gm17435**	487.316198835396	-0.127247489965763	0.214148815049789	-0.594201233082605	0.55237752814168	0.797165124228384
**Gm42521**	31.485795316614	-0.702549777350115	1.18332883582997	-0.593706293700988	0.552708572837158	0.797165124228384
**Gm7232**	50.7246736569287	-0.19307912542235	0.325140717487312	-0.593832500938259	0.552624148738124	0.797165124228384
**Fkbp15**	5752.44421728868	-0.0709007799462233	0.119459920857259	-0.59351102392694	0.552839207564226	0.79726495074562
**Gm7600**	44.2964040965936	-0.231150289056234	0.389501517711509	-0.59345157475725	0.552878981836478	0.79726495074562
**Gm6978**	23.6394161202147	-0.327257017752684	0.55160998675098	-0.593276092915304	0.552996395572182	0.797361292554069
**Pld2**	434.228957045063	0.0857539790234491	0.144671035377339	0.592751540070069	0.553347443276144	0.797654755477783
**Zswim3**	731.85845794557	0.102050724478329	0.172131529015389	0.59286479973814	0.553271637005617	0.797654755477783
**Cry2**	3052.27455231546	0.0504210530866382	0.0850636428628915	0.592745048174207	0.553351788546528	0.797654755477783
**Cnih2**	34.3106773911873	0.286574163487172	0.483747488072774	0.592404447677588	0.553579788734771	0.797910421797822
**Cstf2**	5783.62590448523	0.0448075891372108	0.0756523559034361	0.592282799420072	0.553661232021377	0.797954818710403
**Palm**	158.620344696763	0.189008999951757	0.319374038748332	0.591810783032045	0.553977301685446	0.798118345310192
**Gm17455**	38.0497377010508	0.246742483309551	0.416869575431724	0.591893718926395	0.553921760098427	0.798118345310192
**Trav6n-7**	350.230506666178	0.0894720495478878	0.1511372086445	0.591992205958633	0.553855807564603	0.798118345310192
**Gm8885**	19.7375363073721	0.298383349936506	0.504125013246633	0.591883644128026	0.553928506974787	0.798118345310192
**Zfp276**	4614.40446908613	-0.0437579095792313	0.0739732865607556	-0.591536642667514	0.554160910963066	0.798208864345
**Spic**	141.823513608493	0.165918728061394	0.280587714244268	0.591325705433246	0.554302209346736	0.798208864345
**Sdhaf4**	961.408235428307	0.0810834817200327	0.137201569399927	0.590980715998106	0.554533341902089	0.798208864345
**Dnaja1**	21782.4642560941	-0.0676423425267812	0.11443743926437	-0.591085775438543	0.55446295024227	0.798208864345
**Tada2b**	228.993020852959	0.117979400211007	0.199513844436299	0.591334403606644	0.55429638244118	0.798208864345
**Stx4a**	1043.27302756288	-0.09282710745882	0.156959247098046	-0.591408975100619	0.554246428234189	0.798208864345
**Gpr157**	41.8762021283143	0.278597171680117	0.471423175862276	0.590970461243315	0.55454021300102	0.798208864345
**Prkca**	3608.70424153428	0.0448269983655232	0.0758544399504635	0.590960771641018	0.554546705463268	0.798208864345
**Mafg**	1159.99205215127	-0.0697126398715353	0.117940869230996	-0.591081279340055	0.554465962617249	0.798208864345
**Gm37255**	86.8056981156415	-0.216468922917925	0.365978970514931	-0.591479129561335	0.554199434935687	0.798208864345
**Jagn1**	3083.29697479181	0.0629681433113939	0.106569169840389	0.590866414796162	0.554609930667733	0.798226952642625
**Gm9432**	27.8825554163627	0.249263639987745	0.421943658886545	0.590751003689735	0.554687268385283	0.798249600314913
**Gm45244**	22.8365019564845	-1.4257463784822	2.41368939569529	-0.590691735657851	0.554726986320258	0.798249600314913
**Elac2**	4850.60828688922	0.0420889681173333	0.0712922047439428	0.590372653903782	0.554940839961231	0.79847806631904
**Mcee**	445.401843118001	0.0729293499706277	0.123545486904187	0.590303634702466	0.554987103022538	0.79847806631904
**Awat2**	40.4166705391003	0.574535726387879	0.973858407582853	0.589958172475909	0.555218692129296	0.798592508615364
**Orai2**	27152.2418399649	-0.0953362895398522	0.161566174890881	-0.590075797760519	0.555139833823608	0.798592508615364
**Gm49041**	23.0133240160958	0.465410368079935	0.788842994488885	0.58999112793223	0.555196597590944	0.798592508615364
**Gm28512**	80.3722595644817	-0.198169541284671	0.33602431088116	-0.589747630952679	0.555359856973733	0.798649745946437
**Gm47827**	21.5335783410476	-0.2735333022656	0.463798055133773	-0.589768109714702	0.555346125509969	0.798649745946437
**Gm9108**	100.796374928973	0.191482634269738	0.324730979936613	0.589665434160654	0.555414973412989	0.798656117545945
**Bmpr1a**	91.3313995567867	0.272777342577937	0.462831203825936	0.589366793602198	0.555615247071785	0.798725430901388
**Ube2k**	3000.0314867058	0.0330418821417507	0.0560619180349589	0.589381942322175	0.555605087222448	0.798725430901388
**Vasp**	5004.89091549593	0.0425277944615515	0.0721557718121182	0.589388671114029	0.555600574427254	0.798725430901388
**Mrpl21**	1909.75058328624	0.0718317704769108	0.121895527157276	0.58928963311533	0.555666997995085	0.79872695561107
**Lrrc75b**	1382.11491625994	0.0520502260157125	0.0883931648722458	0.588848991785059	0.555962577705812	0.799078932492062
**Anks3**	6903.18866061464	0.0417349791278121	0.0709019872993192	0.588629186818475	0.55611005031027	0.799163436794922
**Gm16224**	32.3650550326943	0.282903839477201	0.480630224152106	0.588610173187257	0.556122807923863	0.799163436794922
**Gm16146**	18.6388474607365	-0.575749904794836	0.978381173350354	-0.588471978485897	0.556215536984817	0.799223802322612
**Rpl37**	2985.899839955	0.075874878815915	0.128958013203651	0.588368856893701	0.55628473679723	0.799232252844564
**D730003I15Rik**	529.099770764284	-0.118010076476878	0.200590957886099	-0.588312044174428	0.556322862803781	0.799232252844564
**Ripk1**	11060.2323244988	0.0410731675028406	0.06986013431619	0.587934276177336	0.556576408599536	0.799305000939017
**Pibf1**	1404.88114429937	-0.0706930126437157	0.120214461964671	-0.588057472357123	0.556493717074757	0.799305000939017
**Pus7l**	1121.48145025828	-0.0671665691249991	0.114237809318733	-0.587953931588434	0.556563215127203	0.799305000939017
**Rpsa-ps12**	36.243929508173	-0.240973588689708	0.409831867705143	-0.587981578980281	0.556544657386273	0.799305000939017
**Smim15**	4164.29719088753	-0.0392862297348209	0.0668366512544168	-0.587794705412095	0.556670098281688	0.799366694488704

**Trim14**	5412.58830178935	-0.086055672472006	0.146456882201769	-0.587583670895367	0.556811774041016	0.799440342424955
**Gm21982**	8231.382079092	0.380109551968361	0.646921025804123	0.587567163234314	0.556822857023157	0.799440342424955
**Cyp51**	2473.50229631816	0.0611853155786655	0.104150011204559	0.587472962038313	0.556886104270919	0.799458304026683
**Smpd4**	3339.81620688009	0.058479765483451	0.0995748099867505	0.587294773560023	0.557005750655742	0.79955722083288
**Gm17017**	122.676763144602	0.184992362888922	0.315199867804301	0.586904950746296	0.557267544628592	0.799860147632786
**Fhl3**	37.4903229317635	-0.307286088289522	0.52365051228849	-0.586815215641825	0.557327816668628	0.799873796087765
**Zfp605**	512.391444299938	-0.0923306550448307	0.15736356396764	-0.586734646298539	0.557381935080498	0.799878611207961
**Sqor**	734.427300800795	-0.0850429543581418	0.144999706433098	-0.586504320940678	0.557536658687626	0.799881895598036
**Impa2**	896.294532341089	0.0731557788954031	0.124730825475834	0.586509217880362	0.557533368896722	0.799881895598036
**Rcor3**	1400.78394643633	-0.0629922094492882	0.107421146264408	-0.58640418241524	0.557603934369637	0.799881895598036
**Tsga10ip**	34.4919551363336	-0.310171595639726	0.528984033991571	-0.586353416565817	0.55763804170492	0.799881895598036
**Rhog**	14647.7679916029	-0.0541683771821815	0.0923589464282367	-0.586498431143004	0.557540615498483	0.799881895598036
**Ssr4**	4966.58197476314	0.0351039357538535	0.0599380110361166	0.585670681209309	0.558096840099555	0.800094733788576
**Dock2**	54242.6237314491	-0.0700720435535593	0.119657788362559	-0.585603699620818	0.558141861636354	0.800094733788576
**Nid2**	57.705478673696	0.9138983335032	1.55986603340413	0.585882578331922	0.557954425516571	0.800094733788576
**Siglecg**	163.547915065249	0.240343258594422	0.41027381469685	0.585811840738631	0.558001965795637	0.800094733788576
**Kcnn4**	34544.180913226	0.0268661031530145	0.0458508895570136	0.585945080075441	0.557912421905507	0.800094733788576
**2010001A14Rik**	66.9449387043934	0.207067147822552	0.353594504052915	0.585606239489414	0.558140154436693	0.800094733788576
**Rps18-ps5**	1598.73367365241	0.0562664837497521	0.0960102818881502	0.586046438393976	0.557844308429511	0.800094733788576
**Gm20560**	22.0607672223893	-0.680455480076408	1.16234534599109	-0.585415928599267	0.55826808095591	0.800202870155761
**Traj14**	19.6503460757052	-0.27077784449293	0.462800523130705	-0.585085433052669	0.558490273128367	0.800448538628678
**Gm10638**	48.6423055874971	0.318998500836683	0.545341604649996	0.584951703880027	0.558580191469636	0.800504599998822
**Gm2237**	68.1079382332123	0.311606033487135	0.532853236877021	0.58478772750526	0.558690457368972	0.800517007980621
**Gm31834**	33.911728802184	0.353129504758595	0.603787325133579	0.58485743250816	0.558643582953467	0.800517007980621
**Tmem205**	18.0416660031283	0.478836661102713	0.818946548874199	0.58469830755251	0.558750592264127	0.800530376641739
**Olfml3**	898.916825195059	0.14581588509728	0.249509766164088	0.584409529691055	0.558944816772624	0.800690353548314
**Gm16576**	251.760112290784	-0.139536377228566	0.238776299888684	-0.584381185626952	0.558963882022685	0.800690353548314
**Gm43187**	45.6273531751989	0.273286234393089	0.467773437481629	0.584227774591886	0.559067077353385	0.800765379316179
**Tmem147os.1**	74.2758142850709	0.317713699697272	0.543939451240158	0.584097547940123	0.559154684453566	0.800818066072972
**Entpd5**	9000.28118375282	0.0683190508780986	0.11700973563998	0.583874927196702	0.559304463068679	0.800959776840241
**Dap3**	5734.16695019251	-0.0391611573365104	0.0671036292675135	-0.583592240300322	0.559494682081754	0.801108562936503
**Txnrd2**	1877.03806231808	-0.0730026941994384	0.125096847549626	-0.583569415452123	0.559510042210841	0.801108562936503
**Mib1**	462.524431337144	-0.104865011287725	0.179745029108633	-0.583409798912145	0.559617462910677	0.801137110325099
**Ppp3ca**	4632.52638669443	0.129721621215557	0.222380909168587	0.583330744084756	0.559670669903922	0.801137110325099
**Traj24**	41.7048808626586	0.235469479393423	0.403675930092371	0.583313152556661	0.559682510024668	0.801137110325099
**Acap2**	3527.52969809595	-0.0741049873647481	0.12707886384508	-0.583141720995306	0.559797899789	0.801162207037831
**Gm3815**	102.627046516749	-0.144261762482388	0.247389555407019	-0.583136027085059	0.559801732530718	0.801162207037831
**Serpinc1**	48.0840068870107	0.355717943057022	0.610187889097909	0.582964607152249	0.559917126385812	0.801181816242394
**Zfp85**	945.3554950919	0.0663255791545685	0.113770214034664	0.582978415900317	0.559907830396364	0.801181816242394
**Sh2b2**	37.5415477545987	0.531599289471701	0.912417228518839	0.582627413047281	0.560144147219858	0.801361115647658
**Armc9**	1019.4542633838	-0.0577213910344622	0.0990692575848565	-0.582636757775455	0.560137855144723	0.801361115647658
**Laptm4b**	301.96324687207	0.133955882020726	0.230165153221555	0.581998969634561	0.560567374905686	0.801465918120212
**Tgm4**	28.823372649346	-0.335390569440254	0.576373230683533	-0.581898241600339	0.560635224995617	0.801465918120212
**Miip**	7650.97859474956	-0.0598832154815724	0.102883571239358	-0.582048375267365	0.560534096879273	0.801465918120212
**Cxcl9**	72.1196676655952	-0.283591835525421	0.487038162662537	-0.582278468642134	0.560379126077307	0.801465918120212
**Trpc4ap**	30079.5542964363	-0.0343926768297822	0.0590865272354756	-0.582073078905419	0.560517457671495	0.801465918120212
**Gm12253**	18.2724937793723	0.540634698150986	0.929182755001002	0.581838928070079	0.560675180265221	0.801465918120212
**2310034G01Rik**	20.7260133274773	-0.314742589377255	0.540640155985009	-0.582166503714131	0.560454533281345	0.801465918120212
**Gm37084**	41.8765391246935	0.315059106410051	0.541107794088227	0.582248324367475	0.56039942744638	0.801465918120212
**Gm45413**	60.5786808021347	-0.266574788841596	0.458098944996899	-0.581915308369463	0.560623728593275	0.801465918120212
**Gm15197**	19.0361050605388	-0.335702249253576	0.577207645553784	-0.581597024640062	0.560838147528318	0.801626151179581
**Abtb1**	6782.30068110067	-0.0420049968770452	0.0722415119058989	-0.581452350163442	0.560935623840409	0.801692755088737
**Trim23**	1447.6716249612	0.0737475950589574	0.126920228401203	0.581054698592542	0.561203589003889	0.802002988566504
**Mrpl34**	2006.74879345408	-0.0628559675188586	0.108272760742561	-0.580533525586466	0.561554885221217	0.80233986777655
**Fut4**	40.764299559727	0.542276812428763	0.934189550241079	0.58047835397947	0.561592079818974	0.80233986777655
**Gm19466**	37.898564649856	0.354059909836005	0.60986536276825	0.580554219752513	0.561540934309246	0.80233986777655

Trav7d-4	276.228776997409	0.0899830196209326	0.155040253650172	0.580384883940965	0.561655096480249	0.802357149160407
Washc1	1172.63795149994	0.0614603307955211	0.105985350388322	0.579894585151016	0.561985707515926	0.802756666968403
Yipf3	8693.98265091869	0.0279862589702669	0.04826850241793	0.579803755416929	0.562046964803118	0.802771394525266
Nop14	4176.69147250936	0.0421228284468816	0.0726679818963504	0.579661459526471	0.562142938323491	0.80283570009637
Repin1	299.206734179515	-0.109966630283992	0.189753675118834	-0.579523059119276	0.562236292073309	0.802896252872859
Slc37a4	2505.35415446568	0.0713243353212077	0.123096631284139	0.57941744284271	0.56230753733075	0.802925225710976
Nek8	1822.15791819156	0.0734353769698157	0.126774799374526	0.579258475123816	0.562414779931749	0.80300558965065
Gm47469	679.289393122208	-0.0984989466074719	0.170206677401342	-0.578702011644434	0.562790258397244	0.803468886614603
Zc3hav1l	99.1577443249674	-0.189359643246347	0.327281274113152	-0.578583800003417	0.562870038283968	0.803509983080313
Ccdc107	2998.30503322639	-0.0595904982203735	0.103008314504236	-0.578501827810442	0.562925363723646	0.80351616597459
Erlec1	795.975369271658	0.0825893643934097	0.142841604099257	0.578188441065255	0.563136901825485	0.803566117422848
9430091E24Rik	362.006779346063	0.123196584020602	0.213097860447195	0.578122106726311	0.563181682854165	0.803566117422848
Gm19557	173.197395143335	0.184465631901299	0.319056411960697	0.578159927166807	0.563156150796143	0.803566117422848
Gm3052	38.9258317304697	-0.333076615673949	0.576185103779255	-0.57807224360586	0.563215345612612	0.803566117422848
Gm32401	270.859949864011	0.159635477749298	0.276034243393614	0.578317660108802	0.563049673481134	0.803566117422848
Gnaq	114.028068421077	0.147920856623032	0.256101853104177	0.577586045669338	0.563543630305611	0.803888917501857
Ppp1r3f	392.019439593976	0.251402693162744	0.435227811996627	0.577634715045949	0.56351076420379	0.803888917501857
H2-T22	24552.2499607325	0.0350036625718909	0.0606226978307886	0.577401927403393	0.56366797248732	0.803993504343505
Plac8	2566.80428760202	0.0997567969500807	0.172871200575366	0.577058507247367	0.563899932714964	0.804046189813141
Dcun1d4	1077.24278788611	-0.0754035946518223	0.130669759995537	-0.577054665550756	0.563902527816361	0.804046189813141
Trav6n-6	275.884687036813	0.085430523724017	0.148048273233603	0.5770450533335	0.56390902098274	0.804046189813141
Zfp456	536.136150453567	-0.0988236911406637	0.171193178582697	-0.577264187503394	0.563761002210048	0.804046189813141
Myo1e	226.03468274024	-0.191588146652928	0.332117847368169	-0.576867964703335	0.564028652889214	0.804059674206644
Parp2	4450.16404325641	0.0530274083730023	0.0919330780732127	0.576804448239761	0.564071564300327	0.804059674206644
Eml3	5967.22757309533	-0.0554280964637876	0.0960876168119098	-0.576849528616026	0.564041108057388	0.804059674206644
Rpp30	1443.48700689026	0.0748730330356637	0.129853004527661	0.576598387599991	0.564210788680397	0.80414825799415
Trav6-5	179.642802270435	0.119608292514995	0.207451090768361	0.57656140573659	0.56423577713926	0.80414825799415
Gm26549	54.3700716169629	0.210883316449129	0.366192794228021	0.575880573766333	0.564695907046167	0.804731247836343
Azin1	3344.52132060981	-0.0537865937424794	0.0934307732365593	-0.575683919540042	0.564828846400075	0.80477512729234
Ercc6l	291.051323878276	-0.15219091647178	0.264356856910234	-0.575702549389362	0.564816251872581	0.80477512729234
Gatad1	1103.75053240326	0.0870431699530664	0.151284054741705	0.575362486824402	0.565046169112953	0.804939199594323
Gm6598	237.23453280587	0.100148414770807	0.174053002391879	0.575390331649224	0.565027341458567	0.804939199594323
Fcgr4	48.3866165252036	-0.230146918923949	0.400066075566127	-0.575272268707792	0.565107173382896	0.804953329505902
Mms22l	903.081062034764	0.0848225002410436	0.147567339507953	0.574805377151034	0.56542292972142	0.805278049082557
Sec61g	804.475545065095	-0.0752874075505381	0.1309838132815	-0.574784056627946	0.565437350704411	0.805278049082557
Chtf18	1203.14600868796	-0.0615903822558943	0.107191854022323	-0.57458080949946	0.565574833847945	0.805328271908735
4833438C02Rik	1519.96101482645	-0.0735627833608151	0.128017887553341	-0.574628942616817	0.565542273549202	0.805328271908735
Ica1l	20.3786069714329	0.47194326811666	0.821757165381494	0.574309891046175	0.565758117099754	0.805516458944684
Sardh	31.3785735698885	0.436192220725418	0.760913800798637	0.573247876786571	0.566476871806677	0.805593508037708
Tusc2	4989.37870609158	0.0820406934979899	0.143090863753125	0.573346832538066	0.566409881595005	0.805593508037708
Lrig3	55.2406349034226	0.316177127326871	0.551504180280821	0.573299602490551	0.566441854511387	0.805593508037708
Lta	1966.38044130157	-0.0812674330588554	0.141670889335701	-0.573635370257934	0.566214571515774	0.805593508037708
Katnbl1	1653.30801449056	-0.0624130315236967	0.108870341434687	-0.573278550441027	0.566456106214049	0.805593508037708
Gm9797	325.663062362766	0.111955133164558	0.195093395998196	0.573854038429845	0.566066577396679	0.805593508037708
Htatsf1	6419.14385008665	-0.039101940492129	0.0681639197919803	-0.573645714792498	0.566207569940029	0.805593508037708
Wdr90	1968.40387902932	-0.0712843823994903	0.124210532490064	-0.573899660281968	0.566035702972548	0.805593508037708
Gm13369	39.9288232215789	-0.274212343724651	0.478006668917434	-0.57365798754581	0.566199263326141	0.805593508037708
4933404O12Rik	238.178338665479	-0.176813715026171	0.308203665852702	-0.573691148471526	0.566176819184272	0.805593508037708
2900097C17Rik	5195.55833664062	-0.0642189042573659	0.11201246707544	-0.573319255740654	0.566428549915633	0.805593508037708
Gm42983	17.2237784529103	0.52002126199673	0.906991172560204	0.573347655114265	0.566409324750387	0.805593508037708
Rpl19-ps7	32.4292029918748	0.22033224970519	0.384165298486843	0.573535013633554	0.566282498865433	0.805593508037708
Ubqln4	2865.41074307587	0.057782413541166	0.10085654886309	0.572916822878839	0.566701013448099	0.805828975522047
Cela2a	31.0718157212618	1.75760161126871	3.06815863059479	0.57285226185583	0.566744729754098	0.805828975522047
Rps15	10543.4757999675	-0.0362367858359649	0.0632776511527742	-0.572663257497923	0.566872719863831	0.805938233952394
Plk4	1016.22062529949	-0.0549891140577261	0.0960695314611784	-0.572388698283047	0.567058670753403	0.806129869637439
Spata24	103.464729123895	-0.137109369463694	0.239665944782221	-0.572085323128752	0.567264171818979	0.806349260744398

**Map3k15**	125.430812336626	-0.165449333380636	0.289418109681352	-0.571661992965108	0.567550987936594	0.806684188789185
**Tpd52-ps**	18.5911332214491	0.305711812731914	0.534943163493948	0.57148466153895	0.567671154770301	0.806709450371179
**A530083M17Rik**	22.224378781128	-0.4585579699959	0.802351029725494	-0.571517893050857	0.567648634847574	0.806709450371179
**Gm10240**	1993.24926597819	-0.0417693378835713	0.0731167916571606	-0.571268746027926	0.567817484157271	0.806771875371156
**Znf41-ps**	427.819754569148	-0.108129102850488	0.189260158694161	-0.571325225533715	0.567779205351564	0.806771875371156
**Comp**	72.0343482449762	-0.223903377601222	0.392071030883495	-0.571078605569701	0.567946360326547	0.806853181918553
**Snhg15**	3433.21115818064	0.0730555198910033	0.127935672834641	0.571033225310259	0.567977120888531	0.806853181918553
**Rab11a**	4777.72544807967	0.0489535912763417	0.0857949462924917	0.570588285112382	0.568278761436211	0.807081682574872
**Nr2c2**	4074.27775271307	-0.109047471075904	0.191104317323887	-0.57061751719135	0.568258941634725	0.807081682574872
**Cdh23**	1313.11823175433	0.404015856141587	0.709129013245465	0.569735335313007	0.568857220091867	0.807081682574872
**0610010K14Rik**	1008.68320985134	-0.0781692196008116	0.137392630239106	-0.568947690023639	0.569391639852611	0.807081682574872
**Mrps36-ps1**	41.1392242263294	-0.199977181763002	0.351255158341549	-0.569321693970827	0.569137847138939	0.807081682574872
**Rcc1**	2145.95309210109	-0.0704187394918138	0.123694629706309	-0.569295042630473	0.569155930497201	0.807081682574872
**Cdipt**	27589.1961577832	0.0282338583711282	0.049517380648241	0.570180772922833	0.568555095423345	0.807081682574872
**Pold3**	3274.48881154128	-0.0515238821819348	0.0904245319674448	-0.569799821584753	0.56881347657305	0.807081682574872
**Cfap20**	2441.49454627307	0.0394151220757269	0.0691064704951679	0.570353568823673	0.56843791469174	0.807081682574872
**Nol12**	2218.06223869493	0.0593248912202488	0.10400821315259	0.570386601423614	0.568415515103397	0.807081682574872
**Lss**	3252.42504023596	-0.0546508429921144	0.0960988641733627	-0.568693953484446	0.5695638519021	0.807081682574872
**Tpst1**	627.219805461711	0.0982217489740101	0.172629910638874	0.568972946869452	0.569374499286083	0.807081682574872
**Rnps1**	16266.2790581408	-0.0355108386593581	0.0623642722603714	-0.569409974209271	0.569077949552987	0.807081682574872
**Rnf13**	4492.56781715533	0.0439959508187707	0.0773007087311453	0.569153265745469	0.569252132980137	0.807081682574872
**Zfp800**	2640.66958426121	0.0855438661460983	0.150249752540528	0.569344472783898	0.569122391569496	0.807081682574872
**Myzap**	32.8547256601011	0.323484296000608	0.568313293201684	0.569200650187517	0.56921997947753	0.807081682574872
**Phf3**	3605.50640702344	-0.0961783592759112	0.168882632798171	-0.569498223010607	0.569018076306142	0.807081682574872
**Rft1**	955.623267940665	-0.0797715102026994	0.140253378943954	-0.568767118506117	0.569514191945971	0.807081682574872
**Dennd1b**	3587.41267818447	0.0876921128997269	0.154097314979598	0.569069700606639	0.569308839610442	0.807081682574872
**Rpl27a-ps4**	45.1005628194836	-0.205308952120247	0.360405127710885	-0.569661573419522	0.56890725759228	0.807081682574872
**Tspear**	36.7874872451621	-0.366244595109847	0.643702925606221	-0.568965248627585	0.569379723674304	0.807081682574872
**Gm16150**	42.8211126231474	-0.22889755772082	0.402370854571515	-0.568872111685557	0.569442932587311	0.807081682574872
**9430065F17Rik**	26.8137224995718	-0.308448816307159	0.541948381177798	-0.569147961355319	0.569255732416418	0.807081682574872
**Gm28041**	19.2293084067741	-0.404918246017423	0.711627391542674	-0.569003176141993	0.569353984504387	0.807081682574872
**5930430L01Rik**	23.182429176636	-0.394907453433899	0.693744722232354	-0.569240299462248	0.569193075467992	0.807081682574872
**Gm45605**	39.1043517265175	-0.343956785534077	0.603644629266197	-0.569800125534453	0.568813270396081	0.807081682574872
**9930017N22Rik**	278.011887998144	-0.110319285313839	0.193991258796557	-0.568681733384356	0.569572146362415	0.807081682574872
**Gm48942**	52.8866251859088	-1.45356393557394	2.54971187632531	-0.57008948700072	0.568617005205322	0.807081682574872
**Gm44699**	25.006034603594	-0.566155829946449	0.996097304223187	-0.568374020837221	0.569781026948415	0.807305066237404
**Ubac2**	18326.401467618	0.0362100119789419	0.063731234391756	0.5681674350815	0.569921281422427	0.807358593128939
**Gm12345**	48.3047340825578	0.256302018591244	0.451059463800456	0.568222239329025	0.569884072311031	0.807358593128939
**Zcchc2**	1090.30620856411	-0.117868419134823	0.207524903973399	-0.567972406578882	0.570053704594515	0.807473590731371
**Polrmt**	7585.59786205559	0.0404245302526006	0.0713365566156524	0.566673416413974	0.57093608512382	0.807708642812996
**Mtr**	3158.11380647745	0.0421148907985387	0.0742340591083454	0.567325716852847	0.570492907949263	0.807708642812996
**Mtm1**	986.211306688826	-0.0773091208846635	0.13638882670452	-0.566828843334432	0.570830472185967	0.807708642812996
**Irs2**	807.967166680942	-0.168036526711327	0.29615094590912	-0.567401620803509	0.570441348957937	0.807708642812996
**Isg20**	1113.47950343185	0.0590566048025393	0.104195459261052	0.566786741201249	0.570859079760296	0.807708642812996
**Tmem120a**	1950.98644745685	0.0656895219341381	0.115770973640118	0.567409255262366	0.570436163250657	0.807708642812996
**Emc6**	3551.45858917588	0.0628175280729236	0.110790413577333	0.566994255591222	0.570718084409877	0.807708642812996
**Selenon**	283.533546386319	-0.092677073651864	0.163501040665123	-0.566828646930032	0.570830605637322	0.807708642812996
**Sec23ip**	5558.36238076736	-0.0490234144271996	0.0865187699391842	-0.566621722218879	0.570971213505831	0.807708642812996
**Slc27a4**	1771.63571082839	-0.0701725159681094	0.123849250171486	-0.56659621169241	0.570988549361805	0.807708642812996
**Tomm6os**	42.0519566405887	0.26610685897069	0.468918675372365	0.567490426265015	0.570381029224541	0.807708642812996
**Appbp2os**	41.2623941681965	0.288462705873567	0.508331654110727	0.567469492684264	0.57039524776102	0.807708642812996
**Gm43336**	67.8836281156625	-0.268004925637227	0.472612728628614	-0.567070900555091	0.570666012295379	0.807708642812996
**Gm18537**	76.0619019160695	-0.15086205696151	0.26600655337321	-0.567136617682682	0.570621366287422	0.807708642812996
**D9Wsu149**	17.2115366950422	0.564264992092354	0.99571738403189	0.566691915940559	0.570923514166648	0.807708642812996
**Cox19**	761.071311679508	0.0619548521597311	0.109387280195271	0.566380771595505	0.571134963173428	0.807843232919542
**Gm6563**	2780.8631861057	0.1077929152541	0.190354034432054	0.566275968753245	0.571206193996135	0.807871465649143

**Golga7**	8657.42558027806	0.0415946097720675	0.0735424477594804	0.565586420350083	0.571674961290175	0.808037013537934
**Mettl26**	3462.42196303671	0.0673807584027517	0.119214603096859	0.565205575931049	0.571933944509312	0.808037013537934
**Xpc**	5439.81801435454	0.0431889404826593	0.0763769904243225	0.565470572258968	0.571753734832284	0.808037013537934
**Kars**	13580.0651955911	0.0468288617032356	0.0828294172353278	0.565365099336501	0.571825458030685	0.808037013537934
**Scap**	8543.33201869976	0.0471991352243106	0.0835200654607039	0.565123302573532	0.571989899663776	0.808037013537934
**Ttc13**	7169.84325179563	-0.0401238636752512	0.0709525942871192	-0.565502418599165	0.571732079675233	0.808037013537934
**Rtel1**	5102.30082143658	-0.080330472354518	0.142001552045179	-0.565701368735463	0.571596804624694	0.808037013537934
**Rps27rt**	1948.55429198326	-0.0625462362214395	0.110528540448494	-0.565883128173447	0.571473231602314	0.808037013537934
**Ighv3-6**	20.4113960208024	0.375933818012354	0.665107174816873	0.565222917819015	0.57192215040326	0.808037013537934
**Gm11457**	20.9317812947685	-0.334762794093998	0.591897151877856	-0.56557595020001	0.571682080494501	0.808037013537934
**Gm44190**	22.9274976466528	0.338262676376397	0.598514769734459	0.565170140289893	0.571958044419547	0.808037013537934
**Gm45110**	66.871072502925	-0.250947141233446	0.444027186311189	-0.565161658947554	0.571963812683337	0.808037013537934
**Gm37494**	1483.25178615218	0.0600726235885219	0.106223012014709	0.565533046457046	0.571711253440494	0.808037013537934
**Mmp11**	147.709697466324	0.165652909541627	0.293262253795794	0.564862703595588	0.572167153505888	0.8080675937234
**Rpl22l1**	2124.17196396657	0.0679476754255876	0.120299773169043	0.564819647083696	0.572196442134934	0.8080675937234
**Fbxo48**	25.0630508138167	-0.494957437935827	0.876470723456838	-0.564716452802546	0.572266641594199	0.8080675937234
**2610005L07Rik**	4423.4045621776	-0.0358412147222034	0.0634362798003147	-0.564995533077046	0.572076802477087	0.8080675937234
**Gm42463**	25.2975800733212	-0.563054487511236	0.997060418684716	-0.564714511738412	0.572267962071424	0.8080675937234
**Krcc1**	5406.5994198901	-0.0467131279427392	0.0827428776737682	-0.564557690716485	0.572374649875488	0.808073412605328
**Gm14403**	37.9551977858299	-0.486463540130951	0.861563765503221	-0.564628596987036	0.572326410056023	0.808073412605328
**Gm20682**	47.5286147686019	-0.192448138632462	0.340958129695619	-0.564433347884284	0.572459248971212	0.808120443114083
**Lst1**	81.1562839832666	-0.188744073432328	0.334528849713609	-0.564208658218601	0.5726121360704	0.808263856316517
**Zscan29**	2643.8533694286	-0.0598928937413533	0.106293171158061	-0.563468876587481	0.57311564759635	0.808655657348571
**Tmem245**	3262.22411201364	-0.0649130373410962	0.115218765537996	-0.563389453427963	0.573169717214569	0.808655657348571
**E130218I03Rik**	131.653818751682	-0.126209275556338	0.223900069094892	-0.56368573742087	0.572968025621446	0.808655657348571
**Gm4705**	40.449009124752	-0.216873705279754	0.385024093456287	-0.563273075544236	0.573248949202484	0.808655657348571
**Trav12n-1**	107.667598686647	0.134458790892547	0.238674806194655	0.563355609401386	0.573192758251928	0.808655657348571
**2610037D02Rik**	75.9491218577841	-0.226476569951068	0.401961575725596	-0.563428406166054	0.573143198706586	0.808655657348571
**Prkcz2**	1104.29839939299	0.0983982166637415	0.174678114379815	0.563311649047156	0.573222687148251	0.808655657348571
**Aplp1**	276.446692706783	0.173232019146238	0.307740623714724	0.562915669225472	0.573492309801511	0.808781735034674
**A530064D06Rik**	25.4992933241192	0.369041462664715	0.655563115309851	0.562938112359004	0.573477026665233	0.808781735034674
**5033417F24Rik**	48.9462829764017	0.265438209429541	0.471428366046604	0.563050992572857	0.57340016137471	0.808781735034674
**Mmd**	1423.33217982827	-0.0552803622150985	0.098267449608251	-0.562550085867466	0.573741288866525	0.80898805267739
**Atf3**	81.4202042808698	0.232998904189373	0.414129146448725	0.562623776151486	0.57369109827553	0.80898805267739
**Rps5**	28334.9259190761	-0.038927131618032	0.0692592857789413	-0.562049278738997	0.574082444653992	0.809254491186659
**Arhgef3**	24548.2804227933	0.0449383182767496	0.0799478200075957	0.562095605264536	0.574050882440323	0.809254491186659
**Map11**	1165.21518756133	-0.0592937912634114	0.105496234230033	-0.562046519443741	0.574084324584913	0.809254491186659
**Ccdc43**	2156.02994426112	0.0495109792055546	0.0881497267088821	0.561669117467222	0.574341479171767	0.809348147755541
**Zfyve28**	112.153684425432	-0.20862124626964	0.371445172359499	-0.561647483380745	0.574356221882494	0.809348147755541
**Cym**	33.6485472891949	-0.663753205920072	1.18148909887806	-0.561793762253391	0.574256542535487	0.809348147755541
**Ermp1**	2462.82639883375	-0.0539727512863389	0.096094063162859	-0.561665825232788	0.574343722678094	0.809348147755541
**Tirap**	501.537340494691	0.105302876343666	0.187619576700438	0.561257402855124	0.574622075916501	0.809577973016479
**Palld**	34.8857649118969	0.289348626608522	0.515523638310479	0.561271307668453	0.574612598280938	0.809577973016479
**Cand2**	1121.33453620414	-0.0895871423978261	0.159698608243545	-0.560976350283547	0.574813659524726	0.809703096113991
**Gm5561**	27.9367042030461	0.219160423980747	0.390635520038943	0.561035575973476	0.574773284951905	0.809703096113991
**Arhgap17**	3028.85921767801	-0.0452117680816887	0.0806187255506714	-0.560809759430787	0.57492723288338	0.809718306090759
**Prrc2b**	6091.35637567462	0.115982122085859	0.206800516883876	0.56084058122053	0.574906219319872	0.809718306090759
**Gm12033**	2975.38116908774	0.0335481002465864	0.0598367432714822	0.560660530844352	0.575028978522306	0.809789222859593
**Gm20186**	1146.1628635545	-0.123411044438152	0.220159041517114	-0.560554059409632	0.575101577083832	0.809819084013399
**Rassf7**	1445.62560649245	0.0720330279129646	0.128620385885379	0.560043630853024	0.575449677824274	0.810091546561723
**Naa38**	934.305743782672	0.066605208166414	0.118899977972317	0.560178473556331	0.57535770847239	0.810091546561723
**H2ac11**	121.51987429317	0.176653716853299	0.315470653311191	0.559968779977268	0.575500732798751	0.810091546561723
**Gm20511**	20.147944927864	-0.259062455915965	0.462699577619725	-0.559893435063558	0.575552126912387	0.810091546561723
**Gm3650**	23.9305934353722	-0.440450224585149	0.786426597973954	-0.560065269562178	0.575434918693083	0.810091546561723
**Mrpl19**	3119.85496070183	0.0588496433938153	0.105220252724394	0.559299582257816	0.575957280408432	0.810589395087144
**Rpl10-ps3**	1544.48798934254	0.0541878847277286	0.0969005401479768	0.559211379471964	0.576017467857831	0.81060170054799

**Ap4m1**	905.980810619692	0.0845414782427224	0.151244138345046	0.558973585143848	0.576179747750401	0.810757660770055
**Aldh5a1**	90.907250871794	-0.212914394507635	0.380976356776302	-0.558865112547264	0.576253780740262	0.810789429692321
**Gm14176**	129.218542924033	0.135831760452915	0.243093791453682	0.558762770701185	0.576323633582696	0.810815311996656
**Hcfc1r1**	1234.31221995391	-0.0471983865822414	0.0844917448465818	-0.558615361393508	0.576424253984058	0.810879763859252
**H2bc3**	20.1073752803843	0.352365534488781	0.630948580968928	0.55846949358007	0.576523830330342	0.810879763859252
**Gm48604**	45.9808911761478	-0.293139813153356	0.524884737368034	-0.558484162872153	0.576513816003034	0.810879763859252
**Sumo1**	10309.4814011526	0.0550989056800206	0.0987011478249955	0.558239766144514	0.576680669713249	0.810883215480248
**6330549D23Rik**	87.7444758008293	-0.207727092641189	0.372073682932208	-0.558295580069385	0.576642562612438	0.810883215480248
**Gm29257**	27.1240328088561	-0.239366845385319	0.428737862654257	-0.558305823291258	0.576635569155305	0.810883215480248
**Mfsd10**	2849.91653418541	-0.0642519577203366	0.115203777616903	-0.557724399750146	0.577032593532774	0.810937601723167
**Diaph3**	87.5139233701215	0.187282149274256	0.335757818523477	0.557789391466279	0.576988207622291	0.810937601723167
**Mctp2**	5590.90206045102	-0.0528956532095833	0.0948210796103549	-0.557846983254627	0.576948876816416	0.810937601723167
**Plcxd1**	239.134816217592	-0.26444408868706	0.473955573131534	-0.557951216692773	0.576877696528461	0.810937601723167
**Traj33**	94.3716898266218	-0.195189231233693	0.34981775310451	-0.557974057924324	0.576862098963666	0.810937601723167
**Gm13283**	37.8324205200009	-0.287734850355745	0.515790789070588	-0.557851858646446	0.57694554735307	0.810937601723167
**Gm6278**	105.116604448743	-0.110445900104939	0.198053972136039	-0.557655566882932	0.577079604500975	0.810937601723167
**Traj50**	40.6837345022139	0.257410779374798	0.461908723570368	0.557276289101267	0.577338673228195	0.811156974949323
**Zfp850**	751.532946140773	-0.100724478251584	0.180733839817476	-0.557308351071973	0.577316770922878	0.811156974949323
**Aifm1**	2772.14707325472	0.0555035547572608	0.0996244560389514	0.557127807408654	0.577440109755286	0.811227158471294
**Mfap3**	4950.98340005022	-0.070414735874746	0.1264380390972	-0.556911008566136	0.577588232793278	0.8112670903542
**Tpt1**	20047.1742474104	-0.0394066590269452	0.0707599661771487	-0.556906131473975	0.57759156516488	0.8112670903542
**6330418K02Rik**	182.450069111862	0.111127655307469	0.19956116065664	0.556860137222153	0.577622992110412	0.8112670903542
**Rwdd2b**	931.918676512803	0.0559221715601889	0.100468409009594	0.556614483213808	0.577790856173757	0.811430527694286
**Slc24a5**	45.9841427877764	0.343942598626108	0.618030168309762	0.55651425490563	0.577859352311757	0.811454399284944
**Sart1**	11081.112990033	0.0415087267887949	0.0746030193908982	0.55639472943181	0.577941041149264	0.811496790713683
**Dnaja2**	12958.8843949671	0.0387866205719912	0.0697302869525811	0.556237788012652	0.578048309899094	0.811545723700446
**Rpl11**	10369.519572781	-0.0456014619330503	0.0819903869197143	-0.556180591972369	0.578087405462236	0.811545723700446
**Tor4a**	16187.7778227182	-0.0333973023388317	0.0600543786033947	-0.556117690591571	0.57813040226804	0.811545723700446
**Smc5**	2769.60334226773	-0.0433101555767046	0.0778980147589128	-0.555985357402824	0.578220864722575	0.811557161307999
**Pon2**	4372.41307142404	0.0372782556470678	0.0670526389791567	0.555955085655253	0.578241559307286	0.811557161307999
**Gm37566**	83.681323628012	-0.22669116560098	0.407806158240578	-0.555879701716638	0.578293095318464	0.811557205462508
**Fam120b**	8512.36081677713	-0.039285477047382	0.0707293988945045	-0.555433492457326	0.578598189203169	0.811696195448213
**Fcrl5**	29.7504675951198	-0.473863611879909	0.852962438810996	-0.555550385712717	0.578518256571878	0.811696195448213
**Klhl18**	2314.81065791763	-0.0972988490359412	0.175148942833646	-0.55552061840507	0.578538611224845	0.811696195448213
**Bloc1s1**	2237.29977959464	0.0570390109627642	0.102679985607857	0.555502716767029	0.578550852387494	0.811696195448213
**Eef1a1**	269525.37298038	-0.0307535415426124	0.0553779195193619	-0.555339417037145	0.5786625225682	0.811714178590504
**Rpl30-ps9**	33.9714625118436	0.290228807937028	0.522762154405842	0.55518328075393	0.578769303599382	0.81171944074904
**Trav9n-4**	309.462533318249	-0.101159823583959	0.182197288254753	-0.555221345789265	0.57874327020805	0.81171944074904
**Setd4**	1455.04286933661	0.096285016852314	0.173453501432988	0.555105639591326	0.57882240555167	0.811721666454047
**Riok1**	4686.18626221653	0.0503786610110215	0.0907873063259287	0.554908643617653	0.578957149388771	0.811838373491044
**Dusp14**	25.3227115956882	-0.343624292876106	0.619548492828534	-0.554636637573434	0.579143223788316	0.81190397123564
**Ephb6**	1674.96290028761	-0.0779046952558145	0.140462890369117	-0.554628308239221	0.579148922177775	0.81190397123564
**Gm16157**	33.112740844452	-0.289669698971946	0.522479216849401	-0.554413820933742	0.579295669526978	0.81190397123564
**Frg2f1**	156.358919587933	-0.153759288926205	0.277309569812983	-0.554468022974829	0.579258584071932	0.81190397123564
**Trav15n-1**	62.6205587119295	0.186530754419876	0.336361987033549	0.554553610724364	0.579200026542264	0.81190397123564
**Gm27042**	88.9897538599556	-0.254157491151042	0.458446657109352	-0.554388361676762	0.579313089331871	0.81190397123564
**Cdk8**	378.159591804638	-0.111406080156825	0.201002896383572	-0.554251118572091	0.579406998432479	0.811963364843523
**Pparg**	26.2536787625878	0.505386485314368	0.912369530565193	0.553927403736611	0.57962852979711	0.811984952348246
**Ptpn14**	50.2140065384523	0.272822446088208	0.492368707963618	0.554101919304683	0.579509096685777	0.811984952348246
**Clcn7**	5811.96339148102	0.0568054802627322	0.102526292404686	0.554057685403396	0.579539367920912	0.811984952348246
**Actg2**	23.1396909230427	0.660516460188591	1.19227981453601	0.553994500398078	0.579582609530077	0.811984952348246
**2610507B11Rik**	11461.8494994681	-0.0659101006787422	0.119025720039213	-0.553746708333528	0.579752204364837	0.811988354523644
**Casp6**	2488.85667315585	0.0751909325252201	0.135797735387377	0.553697985542471	0.579785554150252	0.811988354523644
**Gm43714**	18.7672823692483	0.324279408162627	0.585640533035825	0.553717493701534	0.579772201093772	0.811988354523644
**Mpg**	1420.03552383211	-0.0525292767546223	0.0949479260745593	-0.553243013579601	0.580097016866927	0.812352354672251
**Rorc**	29.8043389161498	-0.32194911974363	0.58214079732531	-0.553043389542271	0.580233699368517	0.812471554336597

**Irf2bp1**	3238.77941347412	-0.0584307392571062	0.105674147184299	-0.552933151712133	0.580309185636394	0.8125050504774
**Cryzl2**	112.159401923008	-0.143177564197427	0.259085522404604	-0.552626649565669	0.580519089701573	0.812726725582203
**Arl6ip1**	18107.1438084625	0.0539916747720003	0.0977399870774086	0.552401083593756	0.580673588378702	0.812870800647051
**Commd1**	1917.4307381941	0.0648327691938913	0.117398040904069	0.552247454000266	0.580778826127606	0.812930873824367
**Trbv29**	1723.73742625109	0.0499855710352043	0.0905227716102966	0.552187810271583	0.580819685061543	0.812930873824367
**Mien1**	3740.84084667364	0.0692119040142463	0.125406975386316	0.55189835972871	0.581017992254333	0.812991786160334
**Tnks1bp1**	457.617964059136	-0.176393533903865	0.319562039829523	-0.551985254562668	0.580958455886478	0.812991786160334
**Ccdc166**	1984.77985366121	-0.0741294282856572	0.134302727867111	-0.55195772612308	0.580977316805816	0.812991786160334
**2900060B14Rik**	52.0222757021953	-0.212408258608321	0.385032843032285	-0.551662702162035	0.581179468416847	0.81314552333904
**Itpr3**	5500.33146460318	-0.121732562200749	0.220867918603594	-0.55115547323661	0.581527100636212	0.813559666583041
**Gm5784**	20.4347206636575	0.337882590291686	0.613278347051436	0.550944920713706	0.581671432552765	0.813689343282486
**Bcap29**	5159.32534454846	-0.052000313912123	0.0944396902939395	-0.550619276177996	0.581894692012011	0.813929397428608
**Cyp4f18**	113.299965584342	0.187380897353027	0.340602135775668	0.550145984628948	0.582219248533849	0.813957856327141
**B4galnt1**	107097.593450193	-0.0290180027789011	0.0527376867131965	-0.550232757396238	0.582159738358828	0.813957856327141
**Hint1**	3202.04310098232	-0.0484832914239786	0.0880917823030451	-0.550372465585847	0.582063930180313	0.813957856327141
**Slc50a1**	9693.30091241188	0.044325533878348	0.0805227391210597	0.550472256187262	0.581995500937206	0.813957856327141
**Ermard**	1934.63656885017	0.0533135135791622	0.0968991539997278	0.550195862177621	0.582185041352606	0.813957856327141
**Hcst**	6061.75861124326	-0.0486735092015361	0.0884751499604773	-0.550137628738454	0.582224979289408	0.813957856327141
**Gm16477**	356.770235307039	-0.0962532105022883	0.174992349360582	-0.550042392447416	0.582290297456989	0.813976953249625
**Mtrf1**	429.979790177021	-0.100071070798415	0.181984717218046	-0.54988722310409	0.582396728262436	0.813982641844759
**Polr1a**	4854.03972057621	-0.0690975835133661	0.12565805435104	-0.54988583000286	0.582397683833078	0.813982641844759
**Syne3**	252.064534830413	0.179686271010392	0.326824198443135	0.549794880141522	0.58246007070201	0.813997634949141
**Ccrl2**	83.6536576219505	0.179229575733911	0.326179605848319	0.549481244444378	0.582675232384462	0.814009543105059
**Gm12411**	902.305652183862	-0.0590304897239693	0.107390866339047	-0.549678866893601	0.582539654276642	0.814009543105059
**Gm17590**	20.5835619440861	-0.434193068997326	0.789988202042591	-0.549619687831639	0.582580252295932	0.814009543105059
**Gm6740.1**	270.960007764942	0.0872122848992043	0.158709815812722	0.549507819995927	0.582656999474418	0.814009543105059
**Bap1**	11680.4845726828	0.0376354165190952	0.0685625547362239	0.548920860138415	0.583059761899953	0.814044511148149
**Fcna**	332.195261526799	-0.175403652130662	0.31943117080878	-0.549112510487159	0.582928240234094	0.814044511148149
**Gucy1b1**	35.5292644002327	0.288018437517182	0.524489957253099	0.549140042691408	0.582909347163935	0.814044511148149
**Gm8909**	39.4109946681154	-0.429339402992094	0.78201273245364	-0.54901843048642	0.582992801697667	0.814044511148149
**Aunip**	31.1531455696435	0.384417350760671	0.700414731103138	0.548842469596866	0.58311356205681	0.814044511148149
**Gm16538**	21.7762371836814	0.350648346971158	0.63883729265816	0.548885218507099	0.58308422279563	0.814044511148149
**Gm43413**	212.796803568331	-0.12787033999584	0.232767132764979	-0.549348778226985	0.582766118556729	0.814044511148149
**Snhg4**	36.3224547855582	0.515869291906835	0.939760398195511	0.54893704065141	0.583048657351802	0.814044511148149
**Cpd**	23.124514040417	-0.328550061298084	0.598802132951097	-0.548678842673588	0.583225868192412	0.814129164166179
**Trim13**	387.176423977152	0.130835300516923	0.238670720622672	0.548183288572577	0.58356605566507	0.814363071230909
**Dok3**	153.853310371376	-0.163228030819119	0.297750472633678	-0.54820410317178	0.58355176502034	0.814363071230909
**Slc12a9**	1366.32888380235	-0.0685786055112734	0.12509397458182	-0.548216696611702	0.583543118842429	0.814363071230909
**Selenom**	19.7841074016906	0.740077290330043	1.35036225501661	0.548058335887757	0.583651847649341	0.814363071230909
**Traj31**	32.5336816855939	0.808110109681894	1.47432956707021	0.548120398404388	0.583609235053788	0.814363071230909
**Slc25a18**	19.7212694041311	-0.489551122152698	0.893612220943162	-0.547833960502467	0.583805917585717	0.814490247381508
**Fam221a**	81.1740177806647	0.221485859110079	0.404429927785137	0.547649528122332	0.583932574381134	0.814490247381508
**E130311K13Rik**	306.10733003356	0.125597370111955	0.229349432208419	0.547624508604929	0.583949757229733	0.814490247381508
**Gm11847**	1083.65617800386	-0.096611678449968	0.176399801158955	-0.547685869344663	0.58390761645652	0.814490247381508
**Cacybp**	8093.92053514829	0.0621275211074264	0.113466929536332	0.547538576758023	0.584008775105577	0.814500466395696
**Gm13033**	19.2061052535233	-0.343578505359137	0.627633462618404	-0.547419036464011	0.584090879835623	0.814513550157241
**Gm37607**	39.5713112585864	0.255514683570384	0.466800574867629	0.547374397820397	0.584121540697901	0.814513550157241
**H2bu2**	268.742578593983	-0.100134137166478	0.183129120586551	-0.546795271258634	0.584519392177963	0.814996200561735
**Gripap1**	5655.113627221	-0.0486969298656448	0.0890899082857283	-0.546604332664307	0.584650591825526	0.815034891214262
**Zfp710**	1646.7688154462	0.112873711091403	0.206472225363738	0.546677456943934	0.58460034431683	0.815034891214262
**Septin7**	18858.9125136744	0.0367087969059561	0.0671739062510455	0.546474054505127	0.584740117724048	0.815087582712122
**Abca17**	18.1086497901491	-0.339248401357774	0.620911428563176	-0.546371649403867	0.584810493999422	0.815113573989287
**Cep120**	6044.01223962298	-0.0454870691694647	0.0832989099529716	-0.546070401103033	0.585017544925952	0.815257933616186
**Eif2s2**	3234.45175085013	0.0469831378650213	0.086036850550862	0.546081563471998	0.585009872311954	0.815257933616186
**Jmjd6**	6408.02386071791	-0.0597207366778673	0.109440651916493	-0.545690615251781	0.585278624025043	0.815549635546746
**Cdkn2aip**	2754.70018127194	-0.0703189357077161	0.128921606158197	-0.545439494613721	0.585451283531497	0.815718089539818

**Trappc12**	9394.76390028101	0.045595392896519	0.0836879384872638	0.544826336037157	0.585872963762549	0.816039846175233
**Cln5**	2807.75754711287	-0.0677945087678616	0.124392454502223	-0.54500499277993	0.585750083742515	0.816039846175233
**Pmvk**	1037.86803679284	0.0557237084726627	0.102310682511777	0.54465190833077	0.585992946593435	0.816039846175233
**Lrriq3**	26.2036644434603	-0.440500369532766	0.808615794411024	-0.544758552303094	0.585919588511765	0.816039846175233
**Gm4017**	83.5235722044165	-0.151069517728475	0.277345542742582	-0.544697838784776	0.585961351505249	0.816039846175233
**Gm31024**	174.857649192915	-0.144902699502355	0.265972587348642	-0.544803135341214	0.585888922067415	0.816039846175233
**Ankrd40**	650.214396078575	0.101679298321342	0.186736396952132	0.544507123308194	0.586092547897903	0.816094417453311
**Asl**	4217.92743948758	0.0399292305695364	0.0733394129072293	0.54444437154201	0.586135718875365	0.816094417453311
**Ccp110**	516.214876920853	0.0973965441844014	0.178996262220693	0.544126134121822	0.586354677576935	0.816255027350453
**Pomt2**	784.139647792171	0.0596891788055643	0.109696156052784	0.544131909023713	0.586350703900506	0.816255027350453
**Cnn3**	4844.70216808873	0.0571676843979771	0.105079491314728	0.544042264410587	0.586412389245267	0.816263252414989
**Gm9046**	68.7093621197259	-0.131999841343439	0.242689848160435	-0.543903432071775	0.586507927087856	0.816324123939877
**Tnni3**	20.3710840108968	-0.335148749523059	0.616537937253864	-0.543597934971939	0.586718181130633	0.816532053815705
**Ostc**	7563.53508706537	-0.062685416820476	0.115328960232156	-0.543535784024158	0.586760959913386	0.816532053815705
**Trbv4**	2080.93073743256	0.0779259450993177	0.143424551261536	0.543323611012865	0.586907010458443	0.816663172359033
**Aurkb**	292.490222519878	0.159120203752982	0.292919217385267	0.543222138763592	0.586976865432498	0.816688253299768
**Aff4**	3982.43668922449	0.189720088348377	0.349376468128898	0.543024804631037	0.587112724148085	0.816733044442604
**Rpl23a-ps2**	19.4998022364451	-0.358676213958571	0.660465885686784	-0.543065465956053	0.587084728837047	0.816733044442604
**Dvl2**	2160.07563517244	-0.0755367395641726	0.139257965079464	-0.542423117565083	0.587527057759458	0.816780480405345
**Golph3**	8083.62298542628	-0.0380791788663512	0.0702075077709604	-0.542380438721422	0.58755645239331	0.816780480405345
**Diablo**	7075.72879046725	0.0325977254393979	0.0601187166162676	0.542222576830212	0.587665184131205	0.816780480405345
**Atp5l**	3566.80947049634	0.0538931325914886	0.0992700088160538	0.542894407225772	0.587202506895187	0.816780480405345
**Zmym4**	1215.99939158242	0.072607130494309	0.133790159702121	0.542694101389567	0.587340436193732	0.816780480405345
**Apol10b**	214.672399532077	-0.148113280266306	0.273124205121859	-0.542292764569231	0.587616839238025	0.816780480405345
**Ctif**	18.1684983089536	0.372591399947708	0.686905144182324	0.542420453687578	0.587528892458981	0.816780480405345
**Trav4-2**	29.2392834413104	0.266761562961267	0.491929274721164	0.542276251220205	0.587628213368149	0.816780480405345
**Gm12366**	154.2855461775	-0.156189324094164	0.28783349949186	-0.542637755403384	0.587379238376431	0.816780480405345
**Trav4d-4**	114.073875028842	-0.170616436578204	0.314344018006059	-0.542769789800534	0.587288315882591	0.816780480405345
**Adarb1**	63.1899350782873	-0.258898901082269	0.478166011006081	-0.54144145573529	0.588203339792405	0.816808618373528
**Atrn**	1439.14388878971	0.134824214290809	0.248814276631625	0.541866874023548	0.587910218374888	0.816808618373528
**Amigo3**	132.951013688119	-0.219357615708582	0.404794554824427	-0.541898632514275	0.587888338871019	0.816808618373528
**Kcnh3**	442.520223818568	-0.153980540018332	0.284469407324743	-0.541290332294156	0.588307483018416	0.816808618373528
**Blvrb**	398.411456522489	0.0956131597640482	0.176599985966979	0.541410913712791	0.588224386434201	0.816808618373528
**Zfp438**	800.656773749423	0.102597800461293	0.189461524436295	0.54152314443026	0.58814704946441	0.816808618373528
**Ier5**	8640.80021768336	0.0812730375598997	0.150138217517573	0.5413214496861	0.588286038490524	0.816808618373528
**Camk2b**	556.219815707689	-0.166646314648892	0.307820371526266	-0.541375198212545	0.588248998583671	0.816808618373528
**Ttc30b**	248.20214929499	-0.158327794316596	0.292451675108412	-0.54138104785313	0.58824496746577	0.816808618373528
**2510016D11Rik**	38.6348830367867	-0.253853385381746	0.468657798337392	-0.541660431731458	0.588052452665873	0.816808618373528
**Gm45380**	25.8388591554709	-0.307473944322146	0.567489213933949	-0.541814605057734	0.58794622906208	0.816808618373528
**Gm47585**	139.755265696984	-0.205988623744071	0.380397298424153	-0.541509165804823	0.588156681727311	0.816808618373528
**Dcp1a**	4671.25476938661	0.0416723719054193	0.0770211437287554	0.541051065823906	0.5884723853967	0.81696558384985
**Rcn2**	4736.95506381005	0.0563524836994345	0.104239084670508	0.540608005889158	0.588777798531972	0.816999325476548
**Nup37**	1022.52557350761	-0.0638712266116177	0.118115478598402	-0.540752383764899	0.588678266939123	0.816999325476548
**Tbcc**	1662.83616240595	0.0960790932078351	0.177716823743242	0.540630263270101	0.588762454174663	0.816999325476548
**Chchd10**	803.265818223238	0.0629514784137269	0.116404113630357	0.540801149121158	0.588644650707319	0.816999325476548
**Gm37198**	127.679798961261	0.202413195434438	0.374294930151906	0.540785298246732	0.588655577356229	0.816999325476548
**Tes3-ps**	235.445058351939	0.110079098283672	0.20363730412778	0.540564503911318	0.588807789560937	0.816999325476548
**Ubxn6**	3412.5410869745	-0.0552894713288238	0.102329177793368	-0.540309934283545	0.58898330847976	0.817009927734457
**Dsn1**	802.080583885051	0.0658511927260106	0.121856991357981	0.540397329625173	0.588923049017793	0.817009927734457
**Rilpl1**	29.3582692280382	-0.611951421067362	1.13224379462764	-0.540476727689742	0.588868306179734	0.817009927734457
**Fbxo10**	794.278155192234	-0.104452564663351	0.193340230214401	-0.54025261347584	0.589022832967153	0.817009927734457
**Nf2**	2597.87903550529	-0.0538381417248051	0.0997226508901251	-0.539878766200512	0.589280642360869	0.817295579762363
**Gosr1**	6537.51569050979	0.0352803415433855	0.0653757996817075	0.539654454938272	0.589435354975965	0.817352197110311
**Nme2**	6827.62694284329	0.0641302271685217	0.11883991846009	0.539635401971927	0.589448497106302	0.817352197110311
**Bnip3l**	10691.7657150043	0.0429103633027214	0.079524890483437	0.539584060309502	0.589483911625112	0.817352197110311
**Mrpl47**	1241.9421257904	0.0628466192156325	0.116486438388449	0.539518763601108	0.589528953491063	0.817352197110311

**Dctn1**	10919.1514363149	-0.0602914559263521	0.111791535275665	-0.53932040362162	0.589665792533283	0.81744369516842
**Scaf11**	9634.83010713523	0.0357388338908029	0.0662729858528472	0.539266994400366	0.5897026394968	0.81744369516842
**Fbxl4**	1880.00957040831	-0.0581188213405218	0.107802663630018	-0.539122312784288	0.589802460517216	0.81744369516842
**Idi1**	2190.85594424452	-0.0449529123763043	0.0833739964736647	-0.539171855465793	0.589768278372999	0.81744369516842
**Chd5**	18.1264355175929	-0.622747230454254	1.15537675127509	-0.538999274277401	0.589887355309145	0.817484550285853
**Pts**	3045.31358201157	-0.067859496252287	0.125915416605773	-0.538929212018155	0.589935699771558	0.817484550285853
**Pla2g6**	961.622319913709	-0.0706677695502366	0.131169018866513	-0.53875351177364	0.590056944739403	0.817558274087524
**Gm7180**	32.0598697535787	0.196473265496343	0.364716226135606	0.538701739645912	0.590092673173663	0.817558274087524
**Cad**	5030.25780767713	-0.083464751480368	0.155081218197168	-0.538200256940541	0.590438802687387	0.817776657548285
**Igf2r**	3829.27755614986	-0.0985633069866247	0.183195598713434	-0.53802224332258	0.590561692342574	0.817776657548285
**Arrdc1**	24167.6168051228	0.0566863152193142	0.105331316621872	0.53817152426578	0.590458637162472	0.817776657548285
**Clcn6**	1080.79575789145	-0.0852047792527749	0.158343990242759	-0.538099230176947	0.590508543907874	0.817776657548285
**B630019K06Rik**	62.7205227637811	0.193758471521682	0.360087921452546	0.538086561582202	0.590517289613036	0.817776657548285
**AC147806**	21.5042907314726	-0.280526512589245	0.521183090242606	-0.538249451759194	0.590404843685968	0.817776657548285
**Rint1**	2230.73254282192	0.0522931244521507	0.0972890923406363	0.537502439318252	0.590920600417591	0.817796332850112
**Rara**	1791.0322824679	-0.0811179548848735	0.15094511128131	-0.537400345041301	0.590991105043106	0.817796332850112
**Kank3**	325.758164632086	-0.107763813773347	0.200340152433723	-0.537904221716099	0.590643173691021	0.817796332850112
**Bbs12**	316.353901582879	0.147666572009393	0.274774714138602	0.537409610168519	0.590984706539297	0.817796332850112
**Btbd9**	6155.78358170941	-0.048070515581865	0.0894131678027927	-0.53762232972092	0.590837811020175	0.817796332850112
**Srsf3**	11547.6504158762	-0.042474284943338	0.0790052755908378	-0.537613274881908	0.590844063583483	0.817796332850112
**Gm6158**	21.9512233109085	0.243764799910218	0.45333854922545	0.537710283686886	0.590777078477228	0.817796332850112
**Gm17039**	52.6136219285325	-0.262003958651316	0.487340940845599	-0.537619429627039	0.590839813594773	0.817796332850112
**Gm14970**	44.0583614359935	0.17826609944841	0.33182417269539	0.53723060017106	0.591108336616033	0.817886728140044
**E330020D12Rik**	174.200307243737	-0.186693457471359	0.347620468967778	-0.537061174866155	0.5912253581472	0.817976816957189
**Rwdd4a**	2462.72834588574	0.0409746001420936	0.0763177050194602	0.536895077382706	0.591340091499251	0.818063724473547
**Dus3l**	7275.72449760496	0.0438323691633754	0.0817220893962443	0.536358890077397	0.591710537589676	0.818140061726017
**Slc20a1**	7529.51879547342	-0.0506313678248782	0.0944635133008173	-0.535988616722772	0.591966417702502	0.818140061726017
**Kctd15**	59.3100014303728	0.367177314545554	0.684888486545724	0.536112552274655	0.591880765475986	0.818140061726017
**Dnajc12**	190.20181268491	0.134376567381184	0.250469159967897	0.536499453259664	0.5916134136781	0.818140061726017
**Acot6**	240.150387454054	-0.0980670184128779	0.182953175715554	-0.536022498813288	0.591943001124344	0.818140061726017
**Kti12**	4252.05097457363	-0.0432179887173145	0.0805391161628125	-0.536608678818227	0.591537947951353	0.818140061726017
**Traj57**	20.8563866646103	0.509604476075705	0.949732046399102	0.536577109309794	0.591559759387194	0.818140061726017
**Gm14963**	25.2199880926152	-0.251781135408148	0.46954777274777	-0.53622048707576	0.59180617605182	0.818140061726017
**Gm5141**	364.134813119508	-0.118928525907728	0.22184405057544	-0.536090670898049	0.591895887346436	0.818140061726017
**1700022N22Rik**	65.6090088403973	0.129799530397471	0.242070985741166	0.536204411280661	0.591817285117276	0.818140061726017
**Gm9616**	1119.5107840445	0.0898512535426644	0.167615319848544	0.536056331985962	0.591919618778626	0.818140061726017
**Gm47976**	23.571313333476	-0.281462886401247	0.525319589115308	-0.535793623982802	0.592101189279988	0.81825455529598
**Rrp9**	7801.38340884421	0.0331054532332	0.0618099736303092	0.535600507309816	0.592234678069773	0.818367256190952
**Ndufs6b**	30.3535401995328	-0.246243829058278	0.459833522828522	-0.53550647535131	0.592299681145305	0.818385310049682
**B3galt6**	1195.84505340231	0.088998929716168	0.166228612974091	0.535400784039747	0.592372748087895	0.818414501676542
**Tmem260**	2903.07096145821	-0.0403834698775647	0.0754384357453816	-0.535316904155678	0.592430739209619	0.81842286145239
**Itgae**	10490.0726100186	-0.0733431162578515	0.137100544354247	-0.534958607227294	0.59267848036834	0.818478073195787
**Ccnc**	2041.50890474127	0.0552946595801762	0.10333393199184	0.535106508717222	0.592576209468728	0.818478073195787
**Usp37**	3099.75360702611	0.0696479222463119	0.130179769498482	0.535013408877823	0.592640585187646	0.818478073195787
**Gm8989**	84.8140734461877	-0.173078898796574	0.323469026218897	-0.535071010723137	0.592600754878134	0.818478073195787
**Rpl13-ps3**	126.422073453315	0.106703471754456	0.199594443392584	0.534601414452106	0.592925505341177	0.81866816228282
**Rnls**	201.412827145024	-0.1090266654672	0.203944509366442	-0.534589853906311	0.592933501093792	0.81866816228282
**Gm19187**	17.9391741089829	-0.452797791941781	0.8470885372846	-0.534534198034666	0.592971995692951	0.81866816228282
**Gimap1**	28518.9219646007	-0.0359371102668632	0.0672670379692094	-0.534245469278919	0.593171714434327	0.818872148619388
**Rnase4**	53.9589127572605	0.326741198589573	0.611734138499065	0.534122877940499	0.593256522330452	0.81891747896285
**Mzb1**	80.1898356790564	0.249331461507663	0.466958217639851	0.533948118030474	0.593377429708177	0.818959916959943
**Rgl2**	11186.633157012	0.0497934250465544	0.0932586546359846	0.533928194020302	0.59339121482082	0.818959916959943
**Zdhhc4**	3477.75551555596	0.0518506684281822	0.0972228280384484	0.533317837737419	0.593813581874604	0.8193993002538
**Snx19**	3663.69508911769	0.0497753590991729	0.0933309421832629	0.533321082320533	0.593811336256752	0.8193993002538
**Stard4**	1689.30799694059	0.0555745472618845	0.104246487170625	0.533107146055895	0.593959412624958	0.819426699441917
**Mboat1**	2162.62968281184	0.0480675413167569	0.0901722188388906	0.533063752181129	0.593989449833444	0.819426699441917

**A330023F24Rik**	48.3517157933883	-0.197526054899479	0.370495420884474	-0.533140340649638	0.593936435820435	0.819426699441917
**Angpt2**	29.2083994432119	-0.290506538706804	0.545287278595429	-0.532758694563902	0.59420063010289	0.819646268802525
**Il9r**	24.2419040744088	0.54769020714275	1.03098813923909	0.531228426688757	0.595260496442004	0.820046663726154
**Slc9a3r1**	28967.0449836807	-0.0387679498969784	0.0728873986974511	-0.531888235686673	0.594803405714039	0.820046663726154
**Mrps23**	3126.94050651823	0.0438865636959337	0.0825910345977097	0.531372005565732	0.595161016778495	0.820046663726154
**Tmem80**	2562.22453953607	-0.0861154808646829	0.161971099376711	-0.531671892060178	0.594953262733332	0.820046663726154
**Gpkow**	7311.00286420784	0.0415846500612186	0.0781416235733253	0.532170284665215	0.59460806177072	0.820046663726154
**Rnf43**	560.567190955777	0.149742006857905	0.281760506609405	0.53145136861032	0.595106032775787	0.820046663726154
**Kyat3**	244.143186684046	0.109170799731103	0.205426066838638	0.531435963367088	0.595116705596433	0.820046663726154
**Havcr1**	36.4444779762556	0.284135642521705	0.534872224570104	0.531221531927695	0.59526527371803	0.820046663726154
**Ramp3**	642.731130348002	-0.0779112920345239	0.146666908047203	-0.53121248052389	0.595271545325871	0.820046663726154
**Tmem69**	461.202740069834	0.0793262904988261	0.149324896346988	0.531232851583535	0.595257430508321	0.820046663726154
**Gspt1**	5111.74404297707	0.0417322099135831	0.078479416243184	0.531759943069245	0.594892269432626	0.820046663726154
**Gm11652**	21.0288925949234	-0.349358856263365	0.656629011280778	-0.532049072248466	0.594692008532433	0.820046663726154
**Gm8752**	33.3146227732453	0.213508892825352	0.401879985939329	0.531275257030552	0.595228048867026	0.820046663726154
**Gm42664**	22.6925132562252	-0.385134182725703	0.723960597501271	-0.531982243308521	0.594738293838011	0.820046663726154
**Gm48838**	79.4881286171206	-0.174354003776025	0.327920916699732	-0.531695280468112	0.594937061207231	0.820046663726154
**Mir1931**	20.0960008318195	-0.357557054895567	0.673212444793423	-0.531120685098571	0.595335150965575	0.820062590590486
**Stx7**	1966.31753227412	0.0600413327807794	0.113084984516819	0.53093992131068	0.595460412420792	0.820092203329778
**Pdlim5**	4845.77088600706	0.0588574008985367	0.110855204368266	0.530939446947476	0.595460741149711	0.820092203329778
**Polr1c**	2510.71426972379	-0.0659068034827294	0.124174170090502	-0.530760974159879	0.595584426867285	0.820190859478047
**Myo18a**	2074.81723716992	0.10661450475775	0.201088784521629	0.530186230979392	0.595982816624009	0.820366285489449
**Emc2**	2697.48187709861	0.0412286436826895	0.0777807230595228	0.530062489276922	0.596068605476761	0.820366285489449
**Ndfip1**	27425.2965535248	0.0389983565159527	0.0735407886044752	0.530295598619396	0.595906997846667	0.820366285489449
**Adgrb2**	541.617808523014	-0.0960064539191665	0.181174563866549	-0.529911328998057	0.596173410982695	0.820366285489449
**Sin3a**	6328.93502852761	-0.064219997461466	0.121136833933709	-0.530144262286152	0.59601191245777	0.820366285489449
**Lrrn4**	71.0268027642996	0.265102017478484	0.500043366330403	0.530158052938388	0.596002351675885	0.820366285489449
**Gm9845**	86.455944758226	-0.154946153320173	0.292339142609971	-0.530021918846827	0.596096733764659	0.820366285489449
**4732471J01Rik**	73.0537599281089	0.139257639512291	0.26283646170754	0.529826183960899	0.596232449160702	0.820366285489449
**mt-Tr**	21.6903677628107	-0.315929892186808	0.59624477500937	-0.529866097664072	0.596204773319227	0.820366285489449
**Gm34121**	73.8697459347945	-0.183897160648707	0.346879764624532	-0.530146694627057	0.596010226159655	0.820366285489449
**Med15**	13466.3413640371	0.0449069343390533	0.0848665732513832	0.529147491392571	0.59670313870341	0.820870556709415
**Eaf1**	2780.49265685993	-0.0489086733666421	0.0924215338653169	-0.52919131852881	0.596672738427265	0.820870556709415
**Ubxn8**	1730.20282680571	-0.0485165585589959	0.0917094808171449	-0.529024459921769	0.596788482080138	0.82090978902774
**Jrkl**	164.102979834432	-0.154509704979056	0.292112222599006	-0.528939541126827	0.596847391035048	0.82090978902774
**Gm28693**	43.6248170115767	-0.280072152809857	0.529556012557056	-0.528881074274804	0.596887951570148	0.82090978902774
**D8Ertd738e**	5191.10257056694	0.0585484972003652	0.110732987192443	0.528735823757864	0.596988722448992	0.820976723654108
**Trak1**	1689.58257633982	-0.0993352822871562	0.187939821236394	-0.528548349326196	0.597118798578434	0.821083943555318
**Gm37943**	21.9594502064355	-0.522516981241139	0.989214691905659	-0.528213931229168	0.597350861216514	0.821259708593369
**Gm43766**	18.0467854339476	-0.609661158229479	1.15413091879291	-0.528242635477711	0.597330940878266	0.821259708593369
**Ak3**	5668.25704263825	-0.0480577886757062	0.0910615880590975	-0.52775039069731	0.597672593534209	0.821630348658047
**Cbarp**	134.768930854381	-0.111828159117404	0.211929077731862	-0.527667842064088	0.59772989681077	0.821637440813687
**AI662270**	2646.5591753565	0.0498524299686113	0.0945297201143144	0.527373083389276	0.597934531563393	0.821847035401639
**Amd1**	3044.55440713421	-0.0587881279135715	0.111524320538339	-0.527132805022217	0.59810136716833	0.821968461899325
**Gm50230**	9467.41201095037	-0.0836486813308264	0.15869736383551	-0.527095594464494	0.598127205948954	0.821968461899325
**Lhfpl1**	146.81371877583	-0.168624565883197	0.319994084213692	-0.526961510234012	0.598220317410566	0.822024726732214
**Msrb1**	108.643616187777	-0.143915648370088	0.273157692600507	-0.526859218204645	0.598291355992481	0.822050653677496
**Gm37292**	35.1231177672778	-0.297482657398604	0.564764823687616	-0.526737227464344	0.598376079712152	0.822095377803155
**Fance**	1683.87081420994	-0.0548485076615937	0.104203638883591	-0.526358851276459	0.598638900170437	0.82238475588366
**Gm38375**	57.4737624902343	0.265779841879362	0.50511031610282	0.526181773379699	0.598761916629021	0.822482043442027
**Clk3**	18172.6558965445	0.0554448745324116	0.105403212635955	0.52602641936455	0.598869850892233	0.822516609497924
**Pkd1**	9829.95755493111	-0.0841886704075278	0.160078804223066	-0.525920160486788	0.598943680737398	0.822516609497924
**Psenen-ps**	81.9520834284671	0.138136163096976	0.262627178852063	0.525978170655322	0.598903374118359	0.822516609497924
**Tmem167**	5650.8155406299	-0.0475866728660095	0.0905711847596485	-0.525406319816747	0.599300761133398	0.822791852677438
**Mre11a**	2701.67560745882	0.0412638491780951	0.0785165261451643	0.525543490064816	0.599205428750748	0.822791852677438
**Gm38227**	81.4480060200186	-0.185676625438317	0.353372802047477	-0.525441189481726	0.59927652630377	0.822791852677438

**Gm16845**	121.648572996069	-0.333390515665603	0.634715695241463	-0.52525960546599	0.599402734206587	0.82286015707381
**Zfp120**	2570.86393256998	-0.0898382088938518	0.171136462586589	-0.524950717900909	0.599617450646325	0.8230832101955
**A630009H07Rik**	25.7559324505566	-0.48736546024876	0.92878649698452	-0.524733576372054	0.599768412677176	0.823218717644099
**Fbxo22**	3921.95528504061	-0.0510444052514963	0.0972909948237655	-0.524657038855024	0.599821627506466	0.82322004917859
**Supt4a**	9089.17960594992	0.0399783676424834	0.0762392626828695	0.52438030268971	0.600014053822684	0.82332982778191
**Pik3cg**	3195.63902852117	-0.0778335381122004	0.148431026175244	-0.524375126399157	0.600017653381705	0.82332982778191
**Serhl**	979.377341715253	-0.0606382708543431	0.115652019707704	-0.524316574908064	0.600058370390776	0.82332982778191
**Nr1d1**	1037.36655212493	0.109708972610843	0.209440462044693	0.523819378260504	0.600404173870084	0.823515381831991
**Dnajc18**	1481.05885784437	-0.062838841782279	0.120048393872548	-0.523445918393488	0.600663976860765	0.823515381831991
**Rhoh**	34892.4649146712	0.0627760506172946	0.119918808458767	0.523487945086442	0.600634737820961	0.823515381831991
**Avl9**	4885.23507306003	0.0652431211327239	0.124581366646407	0.523698871580853	0.60048800060765	0.823515381831991
**Nmnat3**	1468.02361221023	-0.0900805221751903	0.171974671032014	-0.523801101840291	0.600416886955104	0.823515381831991
**Pip5k1c**	12239.7205692213	-0.0545948459922876	0.104248502404782	-0.523699091429665	0.600487847671819	0.823515381831991
**Ltbp4**	32.9440062698616	0.486567464261016	0.929389467372827	0.523534515230124	0.6006023385382	0.823515381831991
**Gm7353**	593.732758358611	-0.0676806548970401	0.129186417062482	-0.523899156242609	0.600348681699839	0.823515381831991
**Gm10131**	1435.58729447029	0.0905381118935303	0.172910403889731	0.523612864563479	0.600547831959581	0.823515381831991
**Ap3s1**	2922.01461175309	0.0529417775532906	0.101173787612107	0.523275630999064	0.600782456717567	0.823606157168845
**Ly6g5b**	94.4804369600426	-0.193740924605461	0.370325661361807	-0.52316364978061	0.60086037498437	0.82361713637959
**Gm8991**	109.019559124734	-0.146422560752758	0.27990570636086	-0.523113882372898	0.600895005382989	0.82361713637959
**Sh3yl1**	107.671181035885	-0.180075206616079	0.344655041929837	-0.522479536663032	0.601336490595155	0.82386393203268
**Ehhadh**	1473.59509543834	-0.0651128845811908	0.124574273714051	-0.52268323659387	0.601194705715492	0.82386393203268
**Dxo**	2935.25815514662	0.0342095342434537	0.0654507874688905	0.522675670781103	0.601199971612595	0.82386393203268
**Gm13496**	35.3270575876252	-0.257827345355955	0.493426128989558	-0.522524710809166	0.601305045929739	0.82386393203268
**Gm43890**	20.2156112476411	0.265442765194541	0.507903658430319	0.522624243375002	0.601235766260467	0.82386393203268
**Ndufaf1**	1708.46440827391	0.0780830768866528	0.149567471089765	0.522059217273187	0.601629100653935	0.82405283679788
**Hpcal1**	16357.3153731281	-0.0394028281171873	0.0754569890525412	-0.522189244653678	0.601538573772593	0.82405283679788
**Rps13**	6983.61306787326	0.104413445766544	0.200004259868393	0.52205610938112	0.601631264487073	0.82405283679788
**Rpl18a-ps1**	29.3935562903195	-0.20437356697774	0.3915813586351	-0.521918529753579	0.601727056194964	0.824112405429295
**Slc25a42**	892.150646568227	0.114097604297143	0.218999469917122	0.520994887979968	0.602370332521329	0.824205395062398
**Pttg1**	4223.02554650873	0.068147872300029	0.130731711835715	0.521280348456445	0.602171488636265	0.824205395062398
**Ufm1**	1595.41982374058	0.0469038022635692	0.090023936113855	0.521014791046783	0.602356467635325	0.824205395062398
**Prxl2b**	381.231056119901	-0.110933353227882	0.212745643666344	-0.521436544204226	0.602062699514466	0.824205395062398
**Ryk**	22.3662382529743	0.438739934862536	0.842092346794339	0.521011664020844	0.602358645976437	0.824205395062398
**Cip2a**	157.134752446306	-0.132952923851305	0.254830945828129	-0.521729899872426	0.601858403524497	0.824205395062398
**Cars2**	2534.26253740196	-0.0690874968094772	0.132572513360534	-0.521129871179193	0.602276303265543	0.824205395062398
**Fnbp1**	15351.3905777046	-0.0705466074963108	0.135380078832672	-0.521100357634639	0.602296861826823	0.824205395062398
**Gm37790**	22.3882555913523	-0.505280435886261	0.968654560379852	-0.521631195013544	0.601927139142658	0.824205395062398
**Gm7912**	19.3030803802728	-0.303695017323659	0.582726716022263	-0.521161994076236	0.602253927439421	0.824205395062398
**Gm25432**	175.418472687116	0.102851884938596	0.197240444142642	0.521454336536652	0.602050307855582	0.824205395062398
**Slc30a1**	97.3241241703891	0.239253422697555	0.459309940503067	0.520897549997523	0.602438142234231	0.824226604774227
**4930500M09Rik**	29.6040092137143	-0.3641686015658	0.699318854292566	-0.520747580778719	0.602542623798715	0.824297979093276
**Etv5**	298.852643979429	-0.154912839398451	0.297831462774344	-0.520135911617311	0.602968850079503	0.824523142471816
**Ppp3cb**	1702.80004170085	0.0944299660845269	0.181504905398536	0.520261234136806	0.602881511180715	0.824523142471816
**Gpx7**	40.0793126695011	-0.23978839920872	0.46093146604346	-0.520225710054065	0.602906267799174	0.824523142471816
**Thap3**	3331.66610526786	-0.0482895607284242	0.0928360850227206	-0.520159383246352	0.602952491961686	0.824523142471816
**Leng1**	1853.40839365612	0.0661410746365259	0.127120264320428	0.520303155363226	0.602852297002055	0.824523142471816
**Rpl7-ps9**	153.596686524664	-0.109697968814969	0.211009238063597	-0.519872825576987	0.603152216693095	0.824630757347775
**Gm5611**	63.609221666075	0.158849571014573	0.30552538089557	0.519922667468564	0.60311747579402	0.824630757347775
**Ezr**	39110.3069806577	-0.0427444672972991	0.0822779141722845	-0.51951325853734	0.603402869528151	0.824901875338806
**Gm15775**	18.6713230840763	0.293053962283449	0.564237229800173	0.519380761860105	0.603495244272918	0.82495658575591
**Rps27a-ps2**	1047.26266455671	-0.144100494564527	0.277638394592342	-0.51902221512306	0.603745249686133	0.825226743520507
**Rps27**	15450.1873138262	-0.0707946314378275	0.136467731454582	-0.518764624305263	0.60392488985769	0.825400684316733
**Gm45890**	44.3485608344256	-0.231087720618655	0.445605069073988	-0.518593114523761	0.604044511653202	0.825492573297443
**Tox2**	42.077860925226	-0.2866414673131	0.552904072189789	-0.51842893140188	0.604159033339816	0.825577477090927
**Camta1**	632.778480018949	0.0637130711126658	0.123006122011108	0.517966667601406	0.604481525841689	0.825874915693011
**Gm1818**	204.354852273329	-0.138719260045576	0.267783278245663	-0.518028089559483	0.604438671138442	0.825874915693011

**Ywhab**	22734.7425412781	-0.0364702616334278	0.0704440851949298	-0.517719288035453	0.604654138796388	0.82600116759054
**Rbm22**	4451.78362441809	0.04207218875861	0.0812700222333127	0.517683982389321	0.60467877566522	0.82600116759054
**Irak3**	116.636441241667	-0.133567527138597	0.258218973733105	-0.517264572806536	0.604971481109873	0.826311350239456
**Kctd14**	17.1111815502008	0.470940911889071	0.910543919019355	0.517208343334244	0.605010728419916	0.826311350239456
**Suv39h2**	122.500487429569	0.169816853171839	0.328851695168472	0.516393424959665	0.605579657054923	0.826375119919639
**Harbi1**	1840.00269732238	-0.0510602284782646	0.0988644639874189	-0.516466953027352	0.605528314199914	0.826375119919639
**Malsu1**	1145.41416813614	0.0624226053064365	0.1209001998702	0.516315153932368	0.60563431394504	0.826375119919639
**Mtg2**	4529.01107379233	0.0398907480147965	0.0771580434962742	0.517000512289075	0.605155801210165	0.826375119919639
**Mindy2**	7402.18045134181	0.2077458258265	0.401899525122439	0.516909856420483	0.605219086821228	0.826375119919639
**1810058I24Rik**	2796.78262391839	0.0438122832205162	0.0848054953307384	0.516620804461431	0.605420889806794	0.826375119919639
**Zfp628**	178.39070424708	-0.195384292954235	0.377931261748277	-0.516983675947856	0.605167554203413	0.826375119919639
**Gm37519**	42.0592398860205	-0.35572436033886	0.688864972241055	-0.51639199940969	0.605580652500942	0.826375119919639
**Gm18982**	19.1960260410805	-0.376741987357892	0.729510616048961	-0.516431123920213	0.605553332547378	0.826375119919639
**Gm43713**	23.1831196197687	-0.301196619693801	0.583186491389338	-0.516467072096018	0.605528231058751	0.826375119919639
**Gm32717**	40.7723158856848	0.180335537115812	0.349269908612859	0.516321425547431	0.605629934376265	0.826375119919639
**Cox14**	3913.02220567233	0.0449808883844356	0.0872374812272678	0.515614249192421	0.60612385622492	0.826618752936423
**Plxna3**	127.928827786769	0.215556044931304	0.418043934247391	0.515630122272609	0.606112767818093	0.826618752936423
**Got2**	10129.6562030305	0.0354720711079295	0.0687634141252562	0.515856746776931	0.60595446536195	0.826618752936423
**Nbeal2**	8005.98967708924	-0.0830485641445241	0.161057283874944	-0.515646123828891	0.606101589753991	0.826618752936423
**Ampd1**	1512.54089898179	0.103120616940731	0.199997765030846	0.515608846552992	0.606127630350157	0.826618752936423
**Gm34655**	23.4439320260704	-0.360419516805834	0.69884811758774	-0.515733687671533	0.606040422696691	0.826618752936423
**Slc26a8**	20.0883473205175	-0.591040636411422	1.146584066807	-0.515479547921286	0.606217957713412	0.826670390280418
**Cox7b**	2667.8184207867	0.0580753303777205	0.112688042032797	0.515363736294382	0.606298868235419	0.826709178503418
**2810408A11Rik**	195.299672902069	-0.120633966880578	0.234154542498161	-0.515189522242669	0.606420590066648	0.826803602810433
**Cep250**	4479.46096478702	-0.085481972168817	0.165953720884322	-0.515095242898485	0.606486466774483	0.826821877224912
**Srsf10**	7609.35064033048	-0.0552847037474396	0.107394290065654	-0.51478252441207	0.606704998455845	0.826938905595467
**Mrpl1**	1404.13050403361	0.065637804356777	0.12749578586446	0.514823324643498	0.606676484735833	0.826938905595467
**Gm10224**	22.9618977148826	0.224167382495731	0.435490320596955	0.514747106637067	0.60672975106868	0.826938905595467
**AW549877**	3783.66273827659	-0.0655385061678211	0.127400148261612	-0.514430375961886	0.606951126416127	0.826965490049324
**Zfp512**	5114.27131128403	-0.0436178293656938	0.0848028737173708	-0.514343765177844	0.6070116683322	0.826965490049324
**Cops9**	1178.22620439838	0.074059637863638	0.14394330566478	0.51450560706248	0.606898541216343	0.826965490049324
**Faddos**	28.0499324985417	-0.295062620181645	0.573334871033452	-0.514642724678139	0.606802703680137	0.826965490049324
**Ighd**	1259.26380473034	-0.0776031165337152	0.150867350425829	-0.514379793339495	0.606986483912737	0.826965490049324
**1700066M21Rik**	338.443234127031	-0.0940861168748893	0.182971081550665	-0.51421304436481	0.607103048763756	0.826992353499364
**Ly6a2**	118.88509460942	0.425719127525156	0.8279808913362	0.514165401617093	0.607136354971165	0.826992353499364
**Ppard**	148.952230530123	-0.141290831457801	0.274992796559975	-0.513798300265607	0.607393016432046	0.82719894208468
**Tbc1d2**	132.445713471984	0.26213727433571	0.510127857390081	0.513865829003846	0.607345799610827	0.82719894208468
**4833439L19Rik**	7033.66890675088	0.0482118675449369	0.0938872280033496	0.513508264864491	0.607595830935615	0.827247977174399
**Poln**	37.8870430620052	0.335575350657039	0.653613177002244	0.513415828297915	0.607660475863877	0.827247977174399
**Rpl23a**	5965.94015814014	-0.0739909840502217	0.144089829764322	-0.513505943974283	0.607597453997953	0.827247977174399
**1500009L16Rik**	101.275093804209	0.264342295162398	0.514894978483119	0.513390703364695	0.607678047357336	0.827247977174399
**Terc.1**	20.8873784249371	0.339427399695954	0.6611731329563	0.513371434465696	0.607691523500265	0.827247977174399
**Ormdl1**	5380.48185103016	0.0534084830817513	0.104117113425567	0.512965460955973	0.60797548129724	0.827503402752772
**Rpa2**	5880.99566347085	0.0541686644346263	0.105601611326498	0.512953010415232	0.607984190751338	0.827503402752772
**Rtn3**	9371.41246248311	0.034802517538233	0.0678626794545669	0.512837362419985	0.608065091976224	0.827542032671391
**Pan3**	2388.03363444753	-0.101695609650151	0.198333193624325	-0.512751334215788	0.608125275873147	0.827552463246669
**Nectin1**	20.7670315209503	0.295195268244416	0.576362444033466	0.512169506011873	0.608532382859953	0.827966652171325
**Gm15512**	34.5251698537565	-0.357056255367303	0.697149270070955	-0.512166146757871	0.608534733692715	0.827966652171325
**Dicer1**	2211.86181853126	0.0983057678905304	0.192025690591063	0.511940707453992	0.608692507159549	0.828109811372216
**Znrf3**	3232.94703839094	-0.0694212055351435	0.13562570283269	-0.511858770758094	0.608749854984877	0.828116331261047
**Lig3**	2993.72212328356	0.0614523020590692	0.120207164061659	0.511219963791409	0.609197041020381	0.82821587920063
**Invs**	1317.07385832479	0.0390660447431811	0.0764471851153573	0.511020055012245	0.609337013800004	0.82821587920063
**Ctbp2**	100.866004105565	-0.160497366346347	0.314128896812184	-0.510928373591519	0.609401212378759	0.82821587920063
**Cenpi**	148.198730730114	0.14494753664586	0.283558088952075	0.511174049668383	0.609229188055249	0.82821587920063
**Nudt7**	1040.63851144657	0.0553726694044119	0.108269888027308	0.511431852505896	0.609048695706351	0.82821587920063
**Shfl**	4863.83504937089	-0.0459258963664768	0.0898847288744386	-0.510942147143052	0.609391567458055	0.82821587920063

**9930104L06Rik**	1836.08061248473	-0.0507809573505208	0.0993741312313326	-0.511007811804744	0.609345586753633	0.82821587920063
**Gas5**	12858.0384936247	0.0521705284201991	0.102038292981449	0.511283821944023	0.609152331625474	0.82821587920063
**Med12**	7190.35744243293	-0.0797818313029979	0.156014710011607	-0.511373775569384	0.609089354325685	0.82821587920063
**C230071H17Rik**	76.6987813749299	0.303786576780269	0.594557352425182	0.510945791084969	0.609389015801398	0.82821587920063
**Gm44432**	40.5802048886848	-0.212655362836603	0.416059372326043	-0.511117828322724	0.609268552778354	0.82821587920063
**Slc17a9**	8899.75685345797	-0.0601988335723735	0.117846465301264	-0.510824261198504	0.609474119211256	0.828243527073028
**Magee1**	855.917703442514	0.080753912889924	0.158142842315733	0.510639063440496	0.609603817311927	0.82834833989169
**Sqstm1**	34606.5229078136	0.022875162137331	0.0448210408859861	0.510366597587946	0.609794653493056	0.828375316728846
**Cuedc1**	154.243123543912	0.183331576056935	0.359350805465864	0.510174384663651	0.609929296165356	0.828375316728846
**Atf4**	4311.2801612514	0.0566296028866853	0.110968817622307	0.510319962851452	0.609827319310636	0.828375316728846
**1700041G16Rik**	73.4568538899354	0.172424384406323	0.337778809206149	0.510465368776557	0.609725470687433	0.828375316728846
**Gm12251**	523.130200625537	0.0542466245534159	0.106306689891694	0.510284203267762	0.609852368033626	0.828375316728846
**Gm46522**	23.6969310517268	0.223374108320405	0.437850750231376	0.510160387306328	0.609939101649303	0.828375316728846
**Marchf7**	7563.74443790514	-0.0597447314764577	0.117152697039204	-0.50997316311433	0.610070263409897	0.828410646376228
**Krr1**	4108.94696465013	-0.0425506613227315	0.0834332787470844	-0.50999627440889	0.610054071886136	0.828410646376228
**Rmc1**	9370.32539943274	-0.0376473060544013	0.0739212656584313	-0.509289251463288	0.610549490891148	0.828796897250513
**Gstt2**	3355.23628556629	-0.0315016381549967	0.0618568255883974	-0.509266970222693	0.610565106510373	0.828796897250513
**Inf2**	536.56468029915	0.15885332572561	0.311829577671033	0.509423534842464	0.610455383271607	0.828796897250513
**Trav15-1-dv6-1**	135.951618880348	0.137989439139689	0.270907276784477	0.509360401010815	0.61049962751826	0.828796897250513
**Zfp821**	273.963328789704	-0.0847100379292452	0.166423354404724	-0.509003308052782	0.610749905367445	0.828897052046028
**Fv1**	231.732223814135	-0.145152578188103	0.285249677619568	-0.508861497756676	0.610849309409055	0.828897052046028
**Gm14494**	243.718364190527	0.101956374351868	0.200324893457023	0.508955090864636	0.610783703119485	0.828897052046028
**Gm10241**	1730.85716416574	0.39426441692001	0.774773470844177	0.508877022454611	0.610838426790849	0.828897052046028
**Etfrf1**	1206.27160840509	0.0651538323311326	0.128082054585506	0.508688219766468	0.610970780937634	0.828990493002178
**Zfp54**	1287.49093583526	-0.0758929318715516	0.149224410860038	-0.508582553177132	0.611044860702938	0.828999522625352
**Gm9027**	49.137025118044	0.145548918629195	0.286215774363465	0.508528640508691	0.611082658836927	0.828999522625352
**Gm37127**	35.8097599717305	-0.25647700719148	0.504529512200243	-0.508348869569569	0.611208703604571	0.829099134185367
**Tmco6**	1119.40658565063	-0.0697359972887117	0.137291695078201	-0.507940391070196	0.611495147428341	0.829132685286858
**Fars2**	2200.03816375579	-0.0457885563293233	0.090130349536738	-0.508025948691782	0.611435145585107	0.829132685286858
**Eif2s3x**	6406.87533219943	-0.0384329900450347	0.0756646658849569	-0.507938409501068	0.611496537140228	0.829132685286858
**Psenen**	3983.0112464337	0.0553504509101669	0.108909135973885	0.50822596667592	0.611294882486481	0.829132685286858
**Pls1**	84.1922739699845	0.169326087289269	0.333230888310237	0.508134429397875	0.611359071456534	0.829132685286858
**Gm21378**	46.3806247249398	-0.167079671529839	0.329016164796721	-0.507815996314551	0.611582390533961	0.82917774287073
**Lrp1**	64.6499471962141	-0.242453781682842	0.47763825056418	-0.507609642645787	0.61172712691528	0.829262743869656
**Trav6-4**	54.311413610284	0.205448303300906	0.404763192063299	0.507576546804129	0.611750341736427	0.829262743869656
**Atrnl1**	149.548278862846	-0.131215109141134	0.258566890875183	-0.507470653713646	0.611824622242492	0.829292092273114
**Llph**	2645.96258084001	0.0506366444955622	0.0998149815267134	0.507305053019625	0.611940793662912	0.829336766618807
**Rpp21**	2435.03664800372	0.0613250353997466	0.120908592265472	0.507201632660639	0.612013349578074	0.829336766618807
**Gm3604**	303.540844927438	-0.0871518759757711	0.171829884215896	-0.507198595712657	0.612015480246461	0.829336766618807
**Slc20a2**	1175.66656641324	0.0670620423365235	0.132263014203542	0.507035490914495	0.612129916476671	0.829420508549566
**Ctsc**	20898.5499378947	-0.0682933520334387	0.134751624786427	-0.506809117453533	0.61228875842181	0.829493075956707
**Gm19589**	152.603486323009	0.153519010482211	0.302885732308953	0.506854546471724	0.612256880283277	0.829493075956707
**Elk3**	2315.24008610913	-0.048696110626645	0.0960998085017252	-0.506724325322363	0.612348260136941	0.829502367174843
**Snrpf**	1500.72850717781	-0.0510183499944356	0.1008801575598	-0.505732259232377	0.61304461876856	0.830130578575768
**Ears2**	1964.33427017379	0.0575737159523531	0.113852164011094	0.505688376259082	0.613075429516165	0.830130578575768
**Msn**	134554.099257368	0.0411960009156179	0.0814315972543877	0.505897001957655	0.612928957081091	0.830130578575768
**Gm43667**	62.7007361828685	-0.130988344634218	0.258972651702121	-0.505799912744782	0.612997119787764	0.830130578575768
**Gm48670**	78.0523793777945	-0.171727743171296	0.339422956435633	-0.505940272793134	0.612898579323694	0.830130578575768
**Chrne**	37.2808889911429	0.304647304698583	0.603032296980118	0.505192352423252	0.61342374117155	0.830316801790234
**Gins4**	4031.40809832277	0.0391419906189776	0.0774492346828403	0.505388991631314	0.613285649198714	0.830316801790234
**Gm6741**	651.917113127473	-0.105098952729854	0.208028537519544	-0.505214111405172	0.613408460020236	0.830316801790234
**Gm15265**	39.7370417362415	-0.226536377356403	0.448375786332778	-0.505237758731847	0.613391852890652	0.830316801790234
**Surf4**	23190.7493073876	0.0254984951033178	0.0504916231333837	0.505004464521936	0.6135557002629	0.830319900352257
**Cdc14a**	1056.53151590702	-0.0591865947434154	0.117175839443674	-0.5051092019005	0.613482138636809	0.830319900352257
**Gm5424**	2324.02075419919	0.078545930854861	0.15554758306585	0.504964007197777	0.613584116246854	0.830319900352257
**Rps27a**	8386.09756852725	-0.0561709571430406	0.111262770627476	-0.504849527171214	0.613664526652596	0.830357402015024

**C530008M17Rik**	39.9504301989351	0.286557864215746	0.567949739160453	0.504547928201072	0.613876391055935	0.830501441905072
**A730043L09Rik**	41.1409661375521	-0.1889725235165	0.374494969109661	-0.504606307437908	0.613835378842692	0.830501441905072
**Srms**	67.2814220294628	-0.231203031214934	0.458412198361511	-0.504356193053579	0.614011096120972	0.830612366206915
**Maea**	8863.58436149804	0.0340807465770234	0.0675840833798924	0.50427178815838	0.614070399586594	0.830621279619363
**Gm1943**	33.241263183517	0.29433961575753	0.583902378153887	0.504090455476714	0.614197813724424	0.83065101286205
**Gm49883**	38.3826850200577	-0.25630994645059	0.50842726197246	-0.504123137410506	0.614174848771726	0.83065101286205
**Trp53rkb**	714.909902118142	0.100233163537249	0.198943573134271	0.503827100107423	0.614382882174025	0.830829992655004
**Scp2**	16429.0496845253	-0.0301518584500142	0.0598592267204671	-0.503712795870527	0.614463215150695	0.83086732010036
**Vps52**	9134.7340212635	0.0465044095312216	0.0923821270134332	0.503391846828332	0.614688802747059	0.830897637714716
**Cd5**	18099.4056099906	-0.0381604236563394	0.0758082613730173	-0.503380805273578	0.61469656424703	0.830897637714716
**Ptpn9**	1298.31865984531	0.14273296546455	0.283538081529065	0.503399630465226	0.614683331378884	0.830897637714716
**Gm3531**	117.143878271973	0.146264654419663	0.290533029539876	0.503435546214164	0.614658085323992	0.830897637714716
**Tssk4**	65.9679064717607	0.191494860159778	0.380600639380612	0.503138566638816	0.614866853218154	0.831056528234556
**Slc16a2**	114.441670435707	0.146404535413221	0.291120328271361	0.502900420188979	0.615034285697246	0.831068968333033
**Gm13680**	162.237287415359	0.129367380392867	0.257230910540325	0.502923152280279	0.615018302689296	0.831068968333033
**Gm43403**	25.9367347002657	-0.273274158943704	0.543302929134468	-0.502986721200004	0.614973608143313	0.831068968333033
**Os9**	13295.9017319608	0.0402666814721568	0.0801796389518713	0.502205821808792	0.615522748157186	0.831158791902715
**Lix1**	28.1508422035172	0.317231653854602	0.631657451632891	0.50222102665698	0.615512053825819	0.831158791902715
**5730455P16Rik**	1672.29994606313	-0.0533738223081547	0.106247639006917	-0.502353019860327	0.615419219825874	0.831158791902715
**H2-Q6**	62963.9205012891	0.0376945387753536	0.0750494633318304	0.502262602581016	0.615482811812785	0.831158791902715
**Trav5-1**	157.896619164208	0.131880486204497	0.262553853137373	0.502298803192559	0.615457350969687	0.831158791902715
**Gm5138**	737.234827833766	0.0482395768647992	0.0959901786059272	0.502547006010264	0.615282795864092	0.831158791902715
**Gm15326**	41.6686207702369	0.294682375884483	0.586608999671897	0.502348883241317	0.61542212911626	0.831158791902715
**Gm16759**	56.9622016486615	-0.194866774471547	0.387954176117975	-0.502293277060354	0.615461237614003	0.831158791902715
**Gm10226**	89.883964305571	0.182504889718481	0.363480468182675	0.50210370485921	0.615594574238879	0.83118455066684
**Panx1**	5802.94517002633	0.0649261148863456	0.129395587274424	0.501764521139731	0.615833172595684	0.831435464021094
**Adssl1**	100.801460484586	0.194075997858318	0.386970387537168	0.501526742378129	0.61600046197848	0.831518828012929
**Vps53**	4016.76475667407	-0.0353858695726632	0.0705518875421929	-0.501558084487832	0.615978410077678	0.831518828012929
**Dusp4**	34.3950403993125	0.357539245474313	0.713804322932236	0.500892519122857	0.616446767667188	0.831976726203196
**Alg12**	1043.37074263554	0.0681362141348928	0.136084608353306	0.500690085082922	0.616589251250283	0.831976726203196
**Grtp1**	61.7935331486935	-0.231908348693241	0.463196399153723	-0.500669584472043	0.616603681449573	0.831976726203196
**Gm14038**	78.4392836572625	0.15505449948515	0.309684140763625	0.500685954091202	0.616592159007024	0.831976726203196
**Gm48978**	32.9541904234061	-0.245432145936443	0.490067067935713	-0.500813382483065	0.616502466421588	0.831976726203196
**Rab36**	19.1637555790652	0.912098145843224	1.82234041609503	0.500509201128129	0.616716578983233	0.832057807606713
**Nxpe1-ps**	17.2248097386633	0.301652416340876	0.602794585331008	0.500423234849119	0.616777096360072	0.832068211245347
**Kif2c**	136.877231130884	0.198067528236538	0.396001990971902	0.50016801115172	0.616956780619336	0.832239362402096
**Fasn**	8475.15139790058	0.067240956697276	0.134566721385895	0.499684884975758	0.617296977076775	0.832381764371329
**Ppp1r16a**	2796.56771135511	0.044076303621439	0.0881879208925619	0.499799781822009	0.617216064264462	0.832381764371329
**Ndst1**	1874.38255725823	0.0940286912728405	0.188177254559865	0.499681491755035	0.617299366725885	0.832381764371329
**Gm13562**	92.4951005022309	-0.154159225313605	0.30843688460745	-0.499808009375419	0.617210270423943	0.832381764371329
**Gm2792**	28.6477846512371	-0.2566391601701	0.513645066558641	-0.499642996455802	0.617326477022489	0.832381764371329
**Abhd12**	3785.87512589883	0.0425657763336392	0.085211794839945	0.499529160412492	0.61740664905391	0.832386922067106
**Rbm3os**	113.756110561647	-0.108082147753671	0.216386069416673	-0.499487550400243	0.617435955138689	0.832386922067106
**Cisd2**	5254.20282990096	0.036957006613853	0.0740172465850557	0.499302639843329	0.61756619569163	0.832491277740869
**Ifi203-ps**	53.0933658810633	0.174324331034466	0.349289676517374	0.499082402814144	0.617721333906887	0.832629175224193
**Atp13a2**	3498.01465682085	-0.0491318865476054	0.0984886025495393	-0.498858601662993	0.617879000211621	0.832699230784683
**A730081D07Rik**	27.8475220236872	-0.44069037115509	0.883367148959787	-0.498875661919313	0.6178669807636	0.832699230784683
**Pcbd2**	903.052785969176	0.0853815285793948	0.171201460314297	0.498719627873784	0.61797691510875	0.832759963342903
**Fzr1**	6900.3505110375	-0.0352239644111211	0.0706822401616637	-0.498342502028192	0.618242655795439	0.832988893986403
**5430434F05Rik**	119.437751546665	0.193452862085828	0.388203495894772	0.49832849042211	0.618252529997523	0.832988893986403
**Pura**	1858.19598702762	-0.0861006079345319	0.172827294889945	-0.498188714863354	0.618351035835344	0.833050382323658
**Dnlz**	2525.71113356148	0.0446288770185016	0.0896063547217453	0.498054821637234	0.618445402573508	0.833106284376402
**Trpv2**	11116.7048066545	0.0456935365748078	0.0917587191031775	0.497974873901934	0.618501752017043	0.833110968245217
**Bend4**	3057.63308135614	-0.0817900374615328	0.164270738619386	-0.497897788424995	0.618556086183659	0.833112937002813
**Ctbs**	621.67212385401	-0.0610308552060546	0.122653204634342	-0.497588753494063	0.61877393226795	0.833120179599876
**Lrrc42**	1582.30989557212	0.0582195989383724	0.117020758124565	0.497515140659056	0.61882582864993	0.833120179599876

**Cgas**	574.569651348553	0.0778701034432019	0.156459395742816	0.497701675719129	0.618694326816573	0.833120179599876
**C330013E15Rik**	59.6714322477249	-0.186846391182882	0.375334831166103	-0.497812554732481	0.618616166097231	0.833120179599876
**5031434O11Rik**	438.626120249452	-0.0947156029257915	0.19033408447014	-0.497628174110091	0.618746141872194	0.833120179599876
**Esam**	39.7988276558937	-0.215835657705428	0.434079708118931	-0.49722586351881	0.619029784821996	0.833172690140152
**Pdzd2**	60.325555579806	-0.320696982782559	0.6448044491147	-0.497355412517496	0.618938442079238	0.833172690140152
**Dap**	4617.09468051723	-0.0447990716316583	0.0901099965653912	-0.497159841740182	0.619076337891788	0.833172690140152
**4930570N18Rik**	105.054075481437	-0.103354248878118	0.207853309743176	-0.497246105947618	0.619015511850084	0.833172690140152
**Gm7536**	614.222482265497	0.0922208377724528	0.185544665441888	0.497027697092894	0.619169519901519	0.833226930146745
**Psph**	2679.10231214432	0.0562499259428221	0.113220886235598	0.496815806809471	0.619318947448809	0.833355962308717
**Gm20300**	555.321449412194	-0.0896556911842593	0.180487530666593	-0.496741746386217	0.619371179450237	0.833355962308717
**Klrk1**	4580.49339167549	0.263523522424941	0.530616321118849	0.496636669353254	0.619445289584738	0.833384514001598
**Prdm4**	4047.70382642231	0.0545839426552121	0.109965875723	0.496371645261182	0.619632226496817	0.833440912151162
**A430093F15Rik**	545.173547015611	0.0652901059727092	0.131500183750811	0.496502013232409	0.619540267307522	0.833440912151162
**Gm10584**	18.7082159762993	0.404113506677102	0.814166724562221	0.49635227587371	0.619645889817279	0.833440912151162
**Irak2**	1589.39641480463	0.0568796308152613	0.114665114049863	0.496050008641047	0.619859128552388	0.833585413382263
**Gm20707**	345.913183357578	-0.0765737997491749	0.154362884236351	-0.496063546156147	0.619849577634996	0.833585413382263
**Ttc41**	97.6421142249473	-0.169394144768087	0.341540387740649	-0.495971050125753	0.619914836232236	0.833589185399501
**Tmem8b**	46.8018938889177	0.292709823934781	0.590413549208802	0.495770844566548	0.620056097237821	0.833650654553882
**Gm45343**	248.242861261237	-0.160225655844193	0.323194393803201	-0.495756296879819	0.620066362338738	0.833650654553882
**Dnajb6**	7781.94204600973	0.0347526191236225	0.0701244741886532	0.495584737364716	0.620187423302191	0.833742277021808
**Ier3ip1**	2083.3590691188	0.0591454752994736	0.119370062722377	0.49547997169969	0.620261356250922	0.833770533223493
**Helq**	1332.02660677655	0.0560385572273266	0.113158614394653	0.495221309726241	0.620443909992504	0.833861580531869
**Trav8d-2**	379.136143659119	-0.0833319566165078	0.168293309683391	-0.495159057560157	0.62048784868542	0.833861580531869
**Prrt4**	17.5196854877738	-0.319453867019281	0.645091541074887	-0.495207031372617	0.620453987790316	0.833861580531869
**Emc9**	2108.18217050287	-0.0366347181214896	0.0740017680093181	-0.495051930608964	0.620563463975524	0.833874411736979
**Mitd1**	3149.8154605244	0.0601481130007058	0.121530830090205	0.494920613609415	0.620656159221381	0.833874411736979
**Gm37123**	55.8153215957968	0.191041598564852	0.385977848356678	0.49495482546012	0.620632008866615	0.833874411736979
**Mea1**	4088.32452577666	0.0493860966482799	0.0998169515889314	0.494766628935563	0.620764863006896	0.833949351726461
**Lmnb1**	850.086358775937	0.0713554986088945	0.144253349999933	0.494654013989468	0.620844367531951	0.83398505534535
**Cenpa**	1477.32503593812	0.0650183870777502	0.131492722247294	0.494463769298747	0.620978687595658	0.834050705687316
**Rab3a**	494.935285527859	0.107898592751703	0.218290363798929	0.494289307479876	0.621101875452323	0.834050705687316
**Pde12**	4091.56496176042	0.0491368295598827	0.0993955939870512	0.494356214283342	0.621054631161889	0.834050705687316
**Gm5801**	29.4695163035413	-0.218565671293952	0.442185587573357	-0.494284927949381	0.621104967984703	0.834050705687316
**Zfp318**	764.617531765628	-0.0995887214223577	0.20161679086081	-0.493950533569949	0.621341114762391	0.834165467125149
**Efemp2**	262.492782648976	-0.125902902331648	0.254949926574687	-0.49383384425006	0.621423529058409	0.834165467125149
**Hmgn5**	503.584123222816	-0.0746667072811641	0.15121173390793	-0.493789108500185	0.621455125885773	0.834165467125149
**Igsf23**	258.324990311122	-0.135419085501327	0.274190094386626	-0.493887592125693	0.621385567896355	0.834165467125149
**Capn3**	229.578444836118	-0.201415622318017	0.407795518427929	-0.493913280593381	0.621367424947544	0.834165467125149
**Tfam**	3509.47880206126	-0.0560276878309691	0.113569805926455	-0.493332601688613	0.621777596206474	0.834527221756849
**Prpf8**	15941.4654407191	-0.0825805945550275	0.167494537028824	-0.493034555155767	0.621988171493729	0.834667656125591
**Prdm16**	280.425750917989	-0.161882367538829	0.32831946382012	-0.493063571849401	0.621967669312852	0.834667656125591
**Jup**	217.254459381528	0.105813764933394	0.214737667800872	0.492758285106815	0.622183388936813	0.834789315241974
**Gm11224**	23.8396329169311	0.217989077131387	0.442387190849318	0.492756304071283	0.62218478886855	0.834789315241974
**Iqcb1**	479.34660841367	0.0869655454998811	0.176607773563558	0.49242195711495	0.622421080286069	0.835035245812481
**Atf7**	3964.38801804292	-0.0851173906686457	0.172902073652833	-0.492286696569939	0.622516683375589	0.835092404218386
**Efcab2**	258.423192724163	-0.115070544717956	0.233817377520517	-0.492138548204606	0.622621402970568	0.835161781442687
**Dleu7**	113.412921921036	-0.149097085556382	0.303077141062129	-0.491944344709975	0.622758688485027	0.83527482588173
**Tor1aip1**	4716.24762175667	0.0362255841825591	0.0736848938088435	0.491628369263	0.62298208454389	0.835361135746581
**Gm26920**	418.519478209518	-0.886464010623705	1.80311046424386	-0.491630450936042	0.622980612678121	0.835361135746581
**Gm28791**	105.342505991282	0.218001910504535	0.443419493367964	0.491638084849892	0.622975215063118	0.835361135746581
**Arpc2**	47133.9004975657	0.0274271936769084	0.055826202355584	0.491296067431048	0.623217060871648	0.835492724933606
**2810021J22Rik**	1037.19986011481	0.0736195347948972	0.149885725715355	0.491171086796526	0.623305446752206	0.835492724933606
**Pde6b**	44.9099069558209	0.195262784375385	0.397464469358882	0.491271042894345	0.623234757704581	0.835492724933606
**Brox**	4271.56121037171	0.0294162271168386	0.059888840545873	0.49118044110917	0.623298831226184	0.835492724933606
**Zscan18**	567.617283831288	-0.100584006289564	0.204807572909254	-0.491114683215988	0.623345336949894	0.835492724933606
**Atp5j**	6282.60946757472	0.0498965863896793	0.101653923243229	0.490847621004169	0.623534225854055	0.835674814730148

**Jmjd8**	2965.73537283494	0.0424128455504921	0.0864630834266237	0.490531263397349	0.623758012655806	0.835714650291744
**Fsd2**	265.62166116413	-0.100506067381422	0.204896040655106	-0.490522252455819	0.623764387374488	0.835714650291744
**1700123O20Rik**	3066.48326231158	0.0604510384154802	0.123194489774281	0.49069595991054	0.623641504386132	0.835714650291744
**Samd14**	37.3668808795724	0.340622561995878	0.694530670062816	0.490435594392212	0.623825694368504	0.835714650291744
**Traj32**	31.8828294353963	0.394218902296114	0.803821783393315	0.490430727856028	0.623829137318747	0.835714650291744
**Gm28551**	26.0060393661268	-0.509116830632468	1.03860948245576	-0.490190816887862	0.623998878397072	0.835870979078692
**Hspa4l**	1416.80989051861	-0.0532260387702703	0.108711176756325	-0.489609627624359	0.624410162400315	0.836007280051794
**Sdf4**	23364.910441848	0.0354559270206262	0.0724034892300176	0.489699148448313	0.624346804525512	0.836007280051794
**Ppargc1a**	50.7693733791339	-0.294795071783687	0.601860069610137	-0.489806662160932	0.624270715952844	0.836007280051794
**Clec4a3**	35.7184423324849	0.321731184572809	0.656964898648526	0.489723553320212	0.624329532592338	0.836007280051794
**Nxpe4**	19.8267039038487	0.361799794558567	0.738974415698374	0.48959718614432	0.624418968009746	0.836007280051794
**Gm15344**	22.1569543381626	-0.259965942051771	0.530673810194998	-0.489878974725068	0.624219541852792	0.836007280051794
**Uqcr11**	2623.31048116977	0.0715494121829449	0.14624075196737	0.489257687890646	0.62465927274276	0.836186913652847
**Hspbap1**	3070.02652023255	0.0385723166979851	0.0788335410512024	0.489288140347931	0.624637716157616	0.836186913652847
**Nudt14**	1333.55403249328	-0.0341430260646533	0.0698295570061274	-0.48894805478513	0.624878473037765	0.836249904058403
**Grk2**	10506.2902140893	-0.0411218045245315	0.0841420042715647	-0.488719099105515	0.625040580256239	0.836249904058403
**Fem1c**	2260.43192058658	0.0583191258441199	0.119314953054315	0.488783043124289	0.624995304222481	0.836249904058403
**Rabgap1**	5081.92330693886	-0.0465522204963974	0.0952637867730963	-0.488666491993202	0.62507783017071	0.836249904058403
**Tdrd7**	731.885554344672	-0.077873992819634	0.15932822579233	-0.48876457659885	0.6250083794366	0.836249904058403
**S100a10**	10825.9361934638	0.0357693858239694	0.0731619879344972	0.488906696411723	0.62490775461514	0.836249904058403
**Slc16a13**	583.208595769583	-0.105189752083941	0.215219485184009	-0.488755709056757	0.625014658137295	0.836249904058403
**Sec61b**	1996.95507156485	0.04887993771702	0.100131983598787	0.48815509251144	0.625439990545336	0.836663378132512
**Orm2**	28.4666156442145	-0.239364486831585	0.490730577016964	-0.487771698039737	0.625711560269276	0.836955607399234
**Inpp5k**	19674.2995634417	0.0313165171237258	0.0642428749997861	0.487470666962369	0.625924825145216	0.837098749771954
**C130089K02Rik**	298.110135191266	-0.190095096397818	0.389923767265625	-0.487518618654298	0.625890851767148	0.837098749771954
**Ppm1m**	5348.95961653504	-0.0476440534971832	0.0978193010230684	-0.487061888593412	0.626214473481399	0.837343979857977
**Smad2**	5897.7605560271	0.0372324309269018	0.0764315786198199	0.487134134859369	0.626163277704097	0.837343979857977
**Naxd**	3334.87182383608	-0.0414059867964271	0.0850349777584408	-0.48692888371241	0.626308729267981	0.837398951809553
**Mrpl57**	2341.51409805915	0.0447235378889263	0.0918636101079312	0.48684716218294	0.626366645411296	0.837405331049194
**Cdk17**	8983.55486822739	0.100449404353078	0.206584314859513	0.486239259845976	0.626797538810803	0.837428129929062
**Tecpr2**	595.472906852159	0.0968453955808165	0.199118545748635	0.486370544826462	0.626704470588094	0.837428129929062
**Ssh3**	494.881779320364	-0.0963707716613374	0.198147420867211	-0.486358950520585	0.626712689578816	0.837428129929062
**Lipt1**	306.64114192249	-0.0724117015438353	0.148774977898004	-0.48671962561795	0.626457035327817	0.837428129929062
**Osbpl1a**	35.204160200738	-0.415264529939146	0.8540594281924	-0.486224396372563	0.626808075935497	0.837428129929062
**Cenpj**	1838.33060769459	-0.0457174653536149	0.0940187229280153	-0.486259161258955	0.626783430271574	0.837428129929062
**Fcho1**	19374.0711520762	0.0391369473025513	0.0804917189488614	0.486223276302697	0.626808869986886	0.837428129929062
**Gm45894**	92.7948088953445	0.359184025500677	0.73828275772601	0.4865128187566	0.626603618983335	0.837428129929062
**Ulk4**	18.9522035769178	0.710817825037413	1.46326446718423	0.485775361172575	0.627126445219964	0.837497363957197
**Proser2**	19.7318282791785	0.482129714190472	0.992299985863596	0.485870927198367	0.627058682380004	0.837497363957197
**Ptgs1**	116.329810492563	0.136678861608524	0.281253580006504	0.485963099937655	0.626993328592699	0.837497363957197
**Cacna1c**	26.8228021980356	-0.29869617783017	0.614630475066167	-0.485976842912019	0.626983584580344	0.837497363957197
**Gm42585**	20.6459152690921	-0.439252066374455	0.904165814868261	-0.485809194675708	0.627102454595488	0.837497363957197
**Dhrs13**	189.759749683221	0.140562574299759	0.289401936053371	0.485700186448776	0.627179751444611	0.83749757148413
**Pxdc1**	55.994735576241	-0.201066232238355	0.414258029548211	-0.485364719321526	0.627417654136536	0.837531343292901
**Acot11**	450.439947782522	0.146272986204123	0.301361713142048	0.485373489150482	0.627411434357593	0.837531343292901
**1700021A07Rik**	80.5057271765597	-0.153808969389219	0.31687293648072	-0.485396358229468	0.627395215167402	0.837531343292901
**Gm50469**	24.2567605685087	-0.270806201563729	0.55771582510355	-0.485563058056402	0.627276993887176	0.837531343292901
**Map2k2**	12389.4919134059	0.0260306349322094	0.0537230646362681	0.484533693460151	0.628007158236016	0.837788373848065
**Hibch**	1439.59056543704	-0.058248074644896	0.120200638485078	-0.484590393021308	0.627966929766924	0.837788373848065
**Oas1a**	1040.52919105611	0.07339393273219	0.151412917948019	0.484727021490905	0.62786999609435	0.837788373848065
**Gm15964**	173.528392195432	0.12945507758224	0.267172755455608	0.484536970700777	0.628004832996157	0.837788373848065
**Gm2007**	116.75418862355	-0.142094718375645	0.293203589093935	-0.484628168484396	0.627940128602452	0.837788373848065
**A930038B10Rik**	246.125952097287	-0.814183775779075	1.68039997997378	-0.484517844252639	0.628018403482862	0.837788373848065
**Gm38190**	30.7008703192804	0.29189812964467	0.601986506872025	0.484891482304809	0.627753324748814	0.837788373848065
**Gm50388**	5512.63604274991	-0.0580570748857	0.119830409999493	-0.484493668059265	0.628035557015507	0.837788373848065
**Jakmip1**	7887.92559095247	0.0733080983265351	0.151421923775584	0.484131336458135	0.628292663197995	0.837989461148706

**Tbc1d22bos**	41.369137454246	-0.303291176433132	0.626391100446192	-0.484188195229932	0.628252313907398	0.837989461148706
**Skp1a**	10666.8032773948	0.0394708760981032	0.081589865346941	0.483771800949333	0.628547829890537	0.838225567014066
**Als2cl**	20458.6585134534	-0.0673915281697913	0.139315836647167	-0.483731999115564	0.628576080461903	0.838225567014066
**Emsy**	5276.65266216597	-0.0477196946929422	0.0987360006751221	-0.483305930629676	0.628878529696903	0.838486968390092
**Gm8623**	26.8679566274439	0.203646074561815	0.42131485761149	0.483358397841275	0.628841281926418	0.838486968390092
**Bclaf3**	1818.66808648675	-0.0359769560983875	0.0744710762101883	-0.483099720445097	0.629024932554847	0.838540259075019
**Cntd1**	17.9142905705626	-0.335718370177687	0.694898877118552	-0.483118308623216	0.629011734924675	0.838540259075019
**Smarcc2**	4499.26250732797	-0.0588285010060584	0.12184672203385	-0.482807415941114	0.629232484708475	0.838740744491317
**Slc16a6**	1204.30354859063	0.0565157945596339	0.11707343453975	0.482737990747552	0.629281784691017	0.838740744491317
**Gm37422**	174.697451223893	-0.136816540877832	0.283544868520711	-0.482521660827867	0.629435414753342	0.838874552166828
**Lipt2**	227.673756606631	0.125604648076227	0.260451566090247	0.482257219496637	0.629623233667436	0.839053898249137
**Ddt**	753.12727858657	-0.0494734146837149	0.102674300944277	-0.481848079107596	0.629913871988232	0.839059760214878
**Med25**	6538.66129440695	-0.047205780357255	0.0979614529260985	-0.481881178231061	0.629890357454474	0.839059760214878
**Ift140**	4056.14718283523	0.0764584488514065	0.158665928380001	0.481883222390949	0.629888905238987	0.839059760214878
**Taf13**	2269.47925629603	0.0656506970184317	0.136233430864409	0.481898581000818	0.629877994195426	0.839059760214878
**Apoo-ps**	68.9129242254509	0.163786467485385	0.339999006807774	0.481726311565332	0.6300003822493	0.839059760214878
**Epha3**	28.7673711056739	-0.38353598495958	0.795690288178104	-0.482016672388655	0.629794102563666	0.839059760214878
**6820431F20Rik**	3783.07228482228	-0.0327703068178451	0.0680172331638672	-0.481794176174365	0.629952166927139	0.839059760214878
**Gm44264**	63.290046189061	-0.161852615019594	0.336162478616981	-0.481471387542947	0.6301815105219	0.839230059270924
**Ikzf5**	1027.43414355627	0.0686767107711353	0.142664188573109	0.481387175422383	0.63024134968998	0.83923881915533
**Vcam1**	815.203731232304	-0.0958941994267405	0.199262220021121	-0.481246266435133	0.630341481754036	0.839301227649007
**Tmem8**	2534.74654196985	-0.0531261503965443	0.110467444104769	-0.480921332317227	0.630572410748641	0.839325010657741
**Dync2li1**	124.395734814553	-0.141576351154506	0.29437651188114	-0.480936302457686	0.630561770754165	0.839325010657741
**Disp1**	1168.65009663503	-0.077844455850324	0.161836527335564	-0.481006711722844	0.630511728550949	0.839325010657741
**Gm6363**	38.6700885897276	0.165776485736032	0.344612358974189	0.48105206159611	0.630479497787152	0.839325010657741
**Bcl2a1b**	1474.58197239899	-0.061177285218751	0.127238010475854	-0.480809822394707	0.630651668591658	0.839359603175838
**Wars**	8775.28084251951	0.0384871551655334	0.0800785634154894	0.480617452711307	0.630788409068628	0.839414239888927
**Naxe**	3159.41827654237	0.0408970546974995	0.0851106438408204	0.480516335582984	0.630860290351448	0.839414239888927
**Ipp**	1424.28293532265	0.057542707116692	0.119842543766486	0.480152584451266	0.631118899534437	0.839414239888927
**Rnf123**	21188.2037939393	-0.0452520288723026	0.0942268621245431	-0.480245525023335	0.631052819056633	0.839414239888927
**A530040E14Rik**	340.042687036578	-0.0817825604103663	0.170234407177751	-0.480411461855492	0.630934845783206	0.839414239888927
**Gm13803**	219.398956865285	-0.128734752021307	0.267957518442034	-0.480429706805019	0.630921875057078	0.839414239888927
**Gm4673**	95.1113068822145	-0.238224148740026	0.495939064469939	-0.480349635281586	0.630978800466675	0.839414239888927
**Gm10060**	271.164493536346	0.0766649228238673	0.159665248629375	0.480160357259875	0.631113372977001	0.839414239888927
**Gm9836**	45.4471220859848	0.149434708384943	0.311307836165786	0.480022315613547	0.631211525264412	0.839466576940524
**Ly96**	92.9976874989517	-0.139404333795464	0.290523481542506	-0.479838438722097	0.631342278065805	0.839472575497157
**Inpp1**	5189.64605229545	-0.0409110046718392	0.085305513323932	-0.479582187337496	0.631524514801386	0.839472575497157
**Cnnm4**	1033.48118074165	-0.0706269180430539	0.147272427019717	-0.479566470603476	0.631535692704404	0.839472575497157
**Gm10275**	1651.7322948116	-0.0515782622320139	0.107517478529196	-0.479719790099134	0.63142665389904	0.839472575497157
**H2-D1**	705126.714226148	0.032677588435619	0.0681169309167587	0.47972784439675	0.631420926010382	0.839472575497157
**Gm42669**	39.0001604778275	-0.487526837822183	1.01642808386055	-0.479647154150327	0.631478310629309	0.839472575497157
**Cyth1**	36369.7846034508	-0.0366679410399362	0.0765197028076637	-0.47919607231229	0.631799148166812	0.839676856896014
**Stxbp4**	610.236001024334	-0.0706028770005922	0.147357804748521	-0.479125466893879	0.631849373423187	0.839676856896014
**Zfp36l1**	7049.75471085665	0.0523018972847816	0.109178901705621	0.479047659096287	0.631904724069283	0.839676856896014
**Gm17022**	30.6125276941186	-0.24511026034706	0.511642004887587	-0.479065944558077	0.631891716034275	0.839676856896014
**Gm44735**	22.3167479263113	-0.377102858989709	0.787310822124751	-0.478975835708704	0.631955819377409	0.839676856896014
**Ahr**	199.481146219992	0.175350234222757	0.366629662772128	0.478276179011032	0.632453648547891	0.84013306090119
**Adprm**	1517.46676844559	0.055421495967641	0.116079676688297	0.477443576246872	0.633046290394793	0.84013306090119
**Uimc1**	6511.88920326178	0.0369032021750291	0.0772839132382173	0.477501728739847	0.633004890127805	0.84013306090119
**Ing5**	3621.32337087346	-0.0374625959513833	0.0784461546231175	-0.477558092316527	0.632964764533774	0.84013306090119
**Traf5**	5343.05426963993	-0.0293925335076451	0.0615886180882024	-0.477239698178507	0.633191445554575	0.84013306090119
**Plod2**	23.5118932915701	-0.290621958506599	0.607921374023106	-0.478058464342714	0.632608593823908	0.84013306090119
**Tmem258**	2261.58336517947	0.0466284219038596	0.0976793181779561	0.477362278664866	0.633104170177298	0.84013306090119
**Ralgps1**	485.017535252905	-0.111135321008623	0.23256931660122	-0.477858913775732	0.632750626062568	0.84013306090119
**Edc3**	7691.81784017704	-0.0284858203270987	0.0596857357957774	-0.477263452436385	0.633174532497903	0.84013306090119
**Rinl**	12875.375213639	-0.0310584116974325	0.0650819771561206	-0.477219854936623	0.633205574110715	0.84013306090119

**Kbtbd11**	6217.73753245301	-0.0658607524058566	0.137684533921819	-0.47834531976739	0.632404445162265	0.84013306090119
**Trbj2-6**	259.404310921945	-0.0766884826951811	0.160601114447468	-0.477509031982874	0.632999690841053	0.84013306090119
**Trav9-1**	364.878577690869	0.0665758308958772	0.139448374184195	0.47742278305761	0.633061093884154	0.84013306090119
**Gm38014**	46.0610898424254	0.273395458806396	0.572217901835826	0.477782079045886	0.632805317610638	0.84013306090119
**4932442E05Rik**	84.5786338630072	0.150488063084378	0.314984603287777	0.477763235134668	0.632818731157295	0.84013306090119
**Gm30881**	26.375607939338	0.200194465351155	0.418826324526761	0.477989213255299	0.63265788248713	0.84013306090119
**Gm20008**	708.46762804968	0.0634423367040956	0.132823042832519	0.477645560221748	0.632902497706063	0.84013306090119
**Synpo**	138.972653622885	0.131349363242293	0.275366714044323	0.476997968683867	0.633363568111801	0.840271932536637
**Ndufa7**	3985.66262787531	0.0454945607794302	0.0954254310008036	0.476755098743511	0.633536522707326	0.84043062706679
**G3bp2**	16493.5362589921	-0.0308778161837819	0.0647965941115302	-0.47653455566868	0.633693595057236	0.840568227739445
**Kptn**	2357.76540336188	0.0455016753116498	0.0956140213821595	0.475889149456273	0.634153352786356	0.840585412446787
**Hadh**	2356.58117979566	0.0450327108023474	0.0946065717689164	0.475999816506861	0.634074508628331	0.840585412446787
**Txlng**	2042.62455961591	-0.0715579053482158	0.150317522895006	-0.476045001075475	0.634042318314113	0.840585412446787
**Inip**	5302.09939191901	-0.0505860673784205	0.106298279052529	-0.475887924332459	0.634154225642613	0.840585412446787
**Dipk2a**	303.969951141964	0.136747013834521	0.287284945126909	0.47599784170421	0.634075915529699	0.840585412446787
**Zfp28**	814.019379053314	0.0642708351593234	0.134919356700754	0.476364820667459	0.633814492777158	0.840585412446787
**BC003965**	3379.87556975826	0.0556724135954223	0.116997592933018	0.475842384443713	0.634186671521698	0.840585412446787
**Zbtb10**	67.0667405856305	-0.195346822469051	0.410140702057548	-0.47629221262132	0.633866212539735	0.840585412446787
**Gm3851**	27.2616135004206	0.233806764659549	0.490983690499377	0.476200674653257	0.633931418908673	0.840585412446787
**Foxn2**	3526.81504335036	-0.0434568073671943	0.0913630237018984	-0.475649837389208	0.634323863607228	0.84064444784222
**Abhd14b**	1203.36680135317	-0.0712466785466335	0.149808185531753	-0.475586018839621	0.634369337861899	0.84064444784222
**Gm14052**	40.8264475443416	-0.267989428387057	0.563529533967273	-0.4755552499625	0.634391262885818	0.84064444784222
**9130604C24Rik**	49.4810883565501	-0.247074002146083	0.519709908445423	-0.475407526643355	0.634496530775383	0.840702813979619
**Gm49721**	416.743992001626	-0.369102821758285	0.776496718349145	-0.475343698223231	0.634542017304031	0.840702813979619
**Arhgdib**	165405.102913931	0.0215397157393707	0.0453328127527734	0.475146244660341	0.634682738864106	0.840722334559123
**Rasa3**	50308.4304838658	0.0236465785324978	0.0497766435885616	0.475053696427448	0.634748700849774	0.840722334559123
**Bag2**	569.205839570077	-0.0925390049764589	0.194969076656299	-0.474634267974666	0.63504767681783	0.840722334559123
**Nav2**	38.0730131059838	-0.227145730035399	0.478256626839304	-0.474945285205051	0.634825972566592	0.840722334559123
**Gm9174**	24.2635672040168	0.199522987820111	0.420351494819803	0.474657495640984	0.635031118171989	0.840722334559123
**Rai1**	2443.0728535363	-0.0963504893449782	0.202896977110585	-0.474873951879846	0.634876818629135	0.840722334559123
**Gm7935**	240.375044107744	0.0905144040510029	0.190494286561283	0.475155479384332	0.634676157150074	0.840722334559123
**Nrarp**	282.310544993827	0.185099224706423	0.390031988443235	0.474574471302274	0.63509030578519	0.840722334559123
**Gm6981**	20.3630198974398	0.290413597564561	0.611646804739484	0.474806040535526	0.634925227120064	0.840722334559123
**Gm37170**	20.3396872289527	-0.445164763407359	0.937762560966265	-0.474709464780363	0.634994070833519	0.840722334559123
**Tgfbr1**	1330.87398181925	0.0738066686884172	0.155566454070681	0.474438201534654	0.635187456838861	0.84078030556199
**Myom1**	46.0356797302343	0.434021027091948	0.915085243229079	0.474295733980377	0.635289033208028	0.840798227829364
**Snn**	26.2645551692694	0.301017834150075	0.634774017057275	0.474212595445466	0.635348312406063	0.840798227829364
**Rbm3-ps**	122.291587386285	-0.117567842149605	0.247931585721139	-0.474194692893383	0.635361077537872	0.840798227829364
**Gm29642**	47.6550717168478	-0.211178493182473	0.44550785228576	-0.474017443461396	0.635487468291229	0.840894863777704
**Anxa11**	1286.62897517424	-0.0410973496552642	0.0867400231352596	-0.473799154874311	0.635643137311612	0.840909143850388
**Mier3**	779.849629625109	0.0900071460907427	0.189977548086533	0.473777806889819	0.635658362150281	0.840909143850388
**Gm11626**	43.9513514959457	-0.193181772208298	0.407742117210147	-0.473784198527457	0.635653803781003	0.840909143850388
**Zfyve1**	2479.63276281878	-0.0731939250895092	0.15468454199822	-0.473181897453926	0.636083411701397	0.841399979927645
**Gm3724**	92.0344683894654	0.135579950291948	0.286572991707988	0.473107913917098	0.636136190955007	0.841399979927645
**Nectin4**	22.6209371759008	-0.31416655004504	0.664436592056711	-0.472831499349791	0.636333399203116	0.841590177206942
**Enkur**	24.5611480734771	0.395024328734935	0.835869008313899	0.472591189296241	0.63650486959632	0.841605037360848
**Lmbrd1**	3856.0835559275	0.0509420187761183	0.107770961910257	0.472687798950323	0.636435932580308	0.841605037360848
**Gm7511**	18.5432051969184	0.338373842546783	0.715954870196425	0.472618954954449	0.636485056744459	0.841605037360848
**Serpinf1**	24.0800008580207	0.303546373710298	0.642859649559526	0.47218140680984	0.636797309951679	0.841920784652489
**Deptor**	91.8314785843781	0.229403241448062	0.485990890660383	0.472031978081606	0.636903963511942	0.841920784652489
**Serpina3f**	225.884729731314	-0.155127246980328	0.328592036105806	-0.472096794611227	0.636857700308068	0.841920784652489
**Trbc2**	103444.1947582	-0.0287734526666399	0.0609696378688369	-0.47193084414475	0.636976151319888	0.841945576406969
**Ugp2**	1332.19108912939	0.0395038732758947	0.0838168972802767	0.471311567926417	0.637418256032447	0.842196086718575
**Cuedc2**	3932.62387173338	-0.038516695992567	0.081711317364972	-0.471375290897932	0.637372757892224	0.842196086718575
**Mdm4**	7100.25906274743	-0.0610588331112996	0.129526066680171	-0.471401893659502	0.637353763950462	0.842196086718575
**Gm12319**	97.9346115005066	-0.123967898453679	0.263038951835758	-0.471291029668811	0.637432920619771	0.842196086718575

**Gm5791**	35.2629556736092	-0.195286840290295	0.414357232857226	-0.471300667165099	0.637426039302395	0.842196086718575
**Rasa1**	3432.52053069172	0.111223182879836	0.236086887783259	0.471111224872198	0.637561309680949	0.842224476579705
**Gm1866**	26.1513699017748	0.266092098222192	0.564797178375428	0.471128589890577	0.637548909768545	0.842224476579705
**Chd1l**	5132.35185366074	0.0424611602099196	0.0902305657017243	0.470585104722536	0.637937046612841	0.842508929970541
**Osgin2**	1239.98947896753	0.069979462796797	0.148665131302961	0.470718736690095	0.637841602435878	0.842508929970541
**Rpl32**	24873.6560319707	-0.0322304358990225	0.068486921118853	-0.470607166630976	0.637921288886484	0.842508929970541
**Gm6789**	205.019836555252	0.0971510348316979	0.206532282195056	0.470391523296805	0.638075319233455	0.842620918970964
**Xpo7**	7322.39901438978	-0.0272457975011453	0.0579789747838386	-0.469925479067629	0.638408260026066	0.842989939933858
**Nup50**	7001.43660145856	0.0265469352883457	0.056530590329319	0.469603008454299	0.638638674888135	0.843082238623688
**Srebf1**	9956.49578110835	0.0379673756758602	0.0808272008029487	0.469735129989495	0.638544265893603	0.843082238623688
**Ganc**	2826.14878141819	0.0437188004934409	0.0930930442411787	0.469624780775	0.638623116817548	0.843082238623688
**Gamt**	19.3606274206072	0.495494948487549	1.05533266425353	0.469515409947089	0.638701272659636	0.843094240872739
**Cabin1**	9865.18850171061	-0.0565520727244075	0.120526673531429	-0.46920794432828	0.638921007538715	0.843313646824219
**Rit1**	2219.65261959686	-0.0522766763390984	0.111494029485346	-0.468874222058405	0.639159543002554	0.843487179153371
**Otos**	36.9827543724961	-0.246719142439769	0.526154651137248	-0.468909933432124	0.639134015714836	0.843487179153371
**Znhit6**	1167.66093476175	0.0612866222292154	0.130735389181569	0.468783721170547	0.639224236973087	0.843501909554052
**Gm18422**	50.5969236839234	-0.215622300692915	0.460399604445541	-0.468337284851904	0.639543409226088	0.843852411587294
**Ccdc90b**	621.830154820447	-0.0555701863042058	0.118715165365454	-0.468096777131532	0.639715383903528	0.843877080764618
**Tecr**	15257.2705136558	0.0267370051439261	0.0571054711545557	0.46820391467505	0.639638772975034	0.843877080764618
**Zfp831**	1809.80139654283	-0.123535148003981	0.263915243591647	-0.468086444431099	0.639722772719005	0.843877080764618
**Hnrnph3**	2483.97821572759	0.0419777717327893	0.0897024811954456	0.467966673534116	0.639808422352278	0.843877223617997
**Gm1123**	34.8810279083822	-0.194604960053734	0.415878982111419	-0.467936511399839	0.639829992420144	0.843877223617997
**Gm42717**	56.358720441211	-0.210480854709379	0.449923168866862	-0.467815105497852	0.639916817390246	0.84392109906412
**Ttc32**	485.123868349511	0.0827092006661375	0.176904415804107	0.467536100160013	0.640116370282199	0.844113620096795
**C330018D20Rik**	763.260828579665	-0.0683367333072366	0.14635606806046	-0.466921079616641	0.640556343135751	0.844503689947128
**Zfp667**	850.185570678054	0.0769384810845077	0.164747473897656	0.467008563253014	0.640493751457257	0.844503689947128
**Gm49510**	281.557179909952	0.545878054134109	1.16915947525937	0.466897857551049	0.640572958193062	0.844503689947128
**Il18rap**	4685.15073742287	0.152569726974392	0.327040191466707	0.466516749180424	0.640845661539461	0.844651199404206
**Sdccag8**	2115.62152483903	0.0317336365726053	0.0680216652616731	0.466522488835417	0.640841554150766	0.844651199404206
**Gm49284**	91.4796463755603	0.137516865488686	0.294740057373405	0.466569989550036	0.640807562303615	0.844651199404206
**Arsk**	880.284496720201	-0.0584535940393978	0.125545778761675	-0.465595853687449	0.64150481141686	0.844883927908347
**Nmt2**	4144.68680459694	0.0422723286405638	0.0907402288509679	0.465860943661406	0.641315038825616	0.844883927908347
**Polr1d**	3300.48622448256	-0.0429619858730778	0.0922276377016018	-0.465825504628876	0.64134040756084	0.844883927908347
**Spg11**	2367.275748668	-0.0784701369971645	0.168509055787171	-0.465673115492816	0.641449498822155	0.844883927908347
**Cyb5d1**	756.865856279666	-0.0557689855620456	0.119647336201062	-0.46611138478109	0.641135774582752	0.844883927908347
**Gm42743**	43.3266061297828	-0.275206553494292	0.59105128567461	-0.465622121403862	0.64148600584023	0.844883927908347
**Hoxaas3**	87.7190637756215	-0.166799329804102	0.357829327743391	-0.466142143395576	0.641113759194648	0.844883927908347
**Gm32633**	1057.11435659771	-0.0911175814101224	0.195530397369478	-0.466002128753132	0.641213976815975	0.844883927908347
**Gm47021**	61.9677542286273	0.141567434798699	0.304027059466608	0.465640903961207	0.641472559177745	0.844883927908347
**Rps12-ps9**	220.761130530935	0.0978203021881853	0.210133458808744	0.465515119499451	0.641562612055239	0.844889434028785
**Il4ra**	28974.3322169973	0.026115329493165	0.0561151388492656	0.465388307481784	0.641653405928852	0.844938385193124
**Hap1**	27.3075676830959	-0.316251420244893	0.679984483803583	-0.46508623031499	0.641869706352184	0.845035565258773
**Gtf2b**	2809.06168023258	-0.0428533832351087	0.0921458137979093	-0.465060554233024	0.641888092948039	0.845035565258773
**Arhgap29**	153.745209924189	0.217139256176756	0.466759766478846	0.465205597763528	0.641784230421427	0.845035565258773
**Hepacam2**	82.6592664837177	0.157348771019287	0.338583837732303	0.464726172616936	0.642127563062953	0.845138991913043
**Abi1**	12410.0805912855	0.0269717010324628	0.0580237670239146	0.464838848214498	0.642046865358622	0.845138991913043
**Gm10157**	71.3979358793522	0.156805707958457	0.33741485947272	0.46472674085397	0.642127156083804	0.845138991913043
**Mzf1**	92.1342420409767	0.123553745910201	0.266334750364796	0.463903961991331	0.642716554109342	0.845349310776369
**Nol10**	740.147613412565	0.0621799114869538	0.134032491303396	0.463916703198506	0.642707425223547	0.845349310776369
**Ldlrad1**	18.5517394143859	-0.431317918277498	0.928936217322233	-0.464313814268995	0.642422928091854	0.845349310776369
**Gm12254**	21.2538470400825	-0.20162594963227	0.434346041064887	-0.46420579577045	0.642500309191409	0.845349310776369
**Ccdc142os**	65.9178733695941	-0.152290792244947	0.328078739944763	-0.464189762099754	0.642511795546164	0.845349310776369
**Gm43721**	19.109449124397	-0.410139672805011	0.883754412960075	-0.464087835704578	0.642584816553507	0.845349310776369
**Gm42507**	39.7069765721353	-0.233361277636605	0.502933740207008	-0.464000044102338	0.64264771403403	0.845349310776369
**Gm10353**	105.986210462173	-0.136870332467449	0.294737805401126	-0.464379967412644	0.642375539949212	0.845349310776369
**Nubp1**	9586.25906425416	0.0375238882621818	0.0809664487747283	0.463449846572671	0.643041956290147	0.845614901768243

**Cyb5b**	7432.74492268725	0.033856499334666	0.0730614652297906	0.463397486324448	0.643079480097027	0.845614901768243
**Gm4596**	45.0741404930869	-0.156144002011782	0.336902610820428	-0.463469254902889	0.643028047602766	0.845614901768243
**Tex9**	592.46062186851	-0.107807257496415	0.23282412353849	-0.46304161208876	0.643334540365892	0.845879702315201
**Narf**	4360.94314593563	0.0356056056507428	0.076937923205402	0.4627835554605	0.643519519617495	0.845955617772039
**Oaz1**	57887.0266444345	0.0207840957415396	0.0449045867276373	0.462850173137162	0.643471764851185	0.845955617772039
**Gm45186**	23.026260246851	-0.337399683200716	0.729140176621024	-0.462736376377298	0.643553340752264	0.845955617772039
**Gtpbp3**	1318.65187547264	-0.0458261334659015	0.0991773798932988	-0.462062352475979	0.64403660694163	0.845977293204577
**Lrp11**	198.343828674415	0.107326178334962	0.232183159513646	0.462247901870998	0.643903555465426	0.845977293204577
**Nr1d2**	3677.64134683634	-0.0413864989656121	0.0896025555406641	-0.461889716380129	0.644160408952752	0.845977293204577
**Agpat4**	2305.34329177871	0.0422471292388713	0.0913296556754782	0.462578435519215	0.643666568737648	0.845977293204577
**Fam53b**	12124.6676276447	0.0486177602300805	0.105130629878971	0.462450955407104	0.643757965412174	0.845977293204577
**Rab33a**	24.5513248728579	0.252855980259156	0.547353187500816	0.461961282099555	0.644109086042601	0.845977293204577
**Abcd1**	5892.29317993915	-0.040108395967111	0.0867470446182185	-0.462360373700702	0.643822911104913	0.845977293204577
**Fbxl8**	1547.00677431213	0.0413714355968065	0.0894482644307164	0.462518036097295	0.643709871340534	0.845977293204577
**Zfp451**	4373.59958202396	-0.0397114692686383	0.0859729408169535	-0.46190660562826	0.644148296780648	0.845977293204577
**Csl**	19.229012452241	-0.240254807306162	0.520085306917445	-0.461952691434712	0.644115246694803	0.845977293204577
**Glis3**	17.0032653017946	0.674630629463772	1.45955810680828	0.462215670836862	0.6439266664798	0.845977293204577
**Ezh2**	1151.39371394049	-0.069133000851385	0.14975115656691	-0.461652533685079	0.644330515173339	0.846130171479816
**Wdr47**	1969.53946291916	0.0435605468140203	0.0944636623710536	0.461135485546964	0.644701404016348	0.846546668590466
**Gm14018**	21.7018573735865	-0.215877966300306	0.46832680912249	-0.460955815672392	0.644830305476435	0.846645373168251
**Ogfr**	4881.91591212135	-0.0413217258262265	0.0896829330604824	-0.460753505891239	0.6449754623769	0.846694856341982
**Bach2it1**	157.152315303475	-0.113834810644515	0.247057197071395	-0.460762981179694	0.644968663572575	0.846694856341982
**Adrm1**	12536.0544137873	0.0344465371316383	0.0747830450998353	0.460619610844306	0.645071539271422	0.846750436879357
**Gm5064**	34.5846189090571	0.204612293367362	0.444348896204537	0.460476654977844	0.645174124331803	0.846814550362034
**Nln**	994.326328913257	0.0449306773119218	0.0976154218627181	0.460282570669113	0.645313409945242	0.846882289698582
**Tdrd3**	1678.9210748588	0.0395284048851131	0.0858837131835029	0.460254958942622	0.64533322665551	0.846882289698582
**Crybg3**	603.012133262452	0.105473223771392	0.229395687508583	0.459787299913584	0.645668899960491	0.847252236565411
**Gm9234**	79.0326117759858	-0.130386762706482	0.283666381927451	-0.45964827351247	0.645768703363016	0.847312637096431
**Ctcf**	11826.9818877774	0.0498561151498686	0.108492948239875	0.459533231963041	0.645851293492814	0.84735044392725
**Sec24d**	2337.70066534487	-0.0382621328133193	0.0832819756839445	-0.459428735919094	0.645926316623371	0.847378317461865
**Ada**	1263.07878680578	-0.062481798780909	0.136045277993284	-0.459272087223735	0.646038789594396	0.84745531199125
**Prkra**	183.302305449213	-0.118849489942451	0.258924311501004	-0.459012478409118	0.646225205227267	0.847617925055584
**Cops7a**	5376.28191511215	0.0355086289096286	0.0774090715831451	0.45871405228635	0.646439521600646	0.847617925055584
**Dkkl1**	122.916212901678	0.203011250886631	0.442626032105097	0.458651855430021	0.646484192312822	0.847617925055584
**Fanci**	525.563533071526	-0.0873331015640063	0.190413383444152	-0.45865001705419	0.646485512681221	0.847617925055584
**Tbc1d25**	3235.26102812872	0.0469849514208459	0.102377008376692	0.458940461006309	0.646276922237364	0.847617925055584
**Cc2d2b**	18.2189743366051	-0.706694250284119	1.54065324259413	-0.458697798275617	0.646451195351178	0.847617925055584
**Glg1**	18739.4789762242	-0.0424689451766451	0.0926165109624445	-0.458546157000733	0.646560109428001	0.847627149085575
**Ccndbp1**	7809.68502977667	0.0320711330790527	0.0699523912347228	0.458470861581259	0.646614192050484	0.847627149085575
**Mboat4**	145.886150280864	-0.0931954024960029	0.203298959795367	-0.458415540294992	0.646653928993952	0.847627149085575
**Naip5**	74.891453844112	0.14908125973552	0.325312125582811	0.458271450744219	0.646757432372317	0.847692302602779
**Mcts2**	109.970363202162	-0.109250917484665	0.238514794082368	-0.458046713223738	0.646918880987384	0.847833386652102
**Gm4045**	20.202888747097	-0.269313781686075	0.588231924300796	-0.457836051666519	0.647070232698713	0.847961215621558
**Ift74**	318.776165031129	0.0823625558797602	0.180086776269761	0.457349271200154	0.647420020382038	0.848349044587092
**Lrp10**	18690.0714086171	0.0304100369179165	0.0665357428268064	0.457048131213839	0.647636450679867	0.848453494769003
**Gm37109**	147.733543776436	-0.163590146847766	0.357918884700251	-0.457059277508558	0.647628439270204	0.848453494769003
**Gm37082**	22.7492392882845	-0.304501225666049	0.666284823083677	-0.457013600064859	0.647661270234281	0.848453494769003
**Nrip2**	78.5004993919708	-0.450412976458935	0.985925560411067	-0.45684278260434	0.64778405248466	0.848454502495647
**Nexn**	18.1738992399558	-0.293911256704936	0.643430623409034	-0.456787796557973	0.647823578062139	0.848454502495647
**Gm44663**	24.2387341709707	0.296660378916363	0.649241272474158	0.456933949663792	0.647718521125154	0.848454502495647
**Ddrgk1**	4777.12702463354	-0.0302424226572843	0.0662193832102183	-0.456700458252193	0.647886361430164	0.848466206537158
**Lrrc75a**	123.966826548062	0.179136046118745	0.392508324230541	0.456387890549626	0.648111072051372	0.848689949498336
**Txndc15**	6203.39042053429	-0.0362298498576725	0.0794538647630665	-0.455985998487435	0.64840004668993	0.848967167197235
**Rnf10**	7547.80775801886	-0.0388617547199098	0.0852336868049312	-0.455943608409785	0.648430529746698	0.848967167197235
**Rpl3**	58748.4172119459	0.0258126388735725	0.0566234553478746	0.45586477750234	0.648487219273262	0.8489708469665
**Vps41**	9375.28787309347	0.035424099740882	0.0777319331528819	0.455721327182362	0.648590383666465	0.849035363913973

**Fbxo27**	179.111932705118	0.164154482121044	0.36045509409307	0.455408967194839	0.648815045244281	0.849258902468362
**Ikzf4**	301.177600744978	-0.122984911828064	0.270277143114115	-0.455032602502157	0.649085783974812	0.849497123219129
**Zbtb41**	545.145018645492	-0.0909512432386543	0.199890171603591	-0.455006079133408	0.649104865365127	0.849497123219129
**Ephx1**	10055.3773790162	-0.0410695254858345	0.0903032869080783	-0.45479546638917	0.649256392149093	0.849624862643739
**Abcc1**	3339.87782641122	-0.054567769086323	0.120140981484277	-0.454197796723212	0.649686468711419	0.849975895673017
**Oaz3**	17.3210065257016	-0.237995375558814	0.523957692651364	-0.454226321889645	0.649665939658872	0.849975895673017
**Hook2**	667.30690593264	0.0758708684446688	0.167035185142763	0.454220877953484	0.649669857541908	0.849975895673017
**Ebpl**	1277.93677180909	0.0414539942257977	0.0914150977807833	0.453469888805521	0.650210420987983	0.850491304923421
**Muc20**	58.1402943173931	0.814901072332646	1.79695585693962	0.453489755569452	0.650196118480733	0.850491304923421
**1810062O18Rik**	204.444647464229	0.124661591288243	0.274932886743012	0.453425535100747	0.650242352631367	0.850491304923421
**Ddx25**	37.5494945033388	0.392704997688793	0.866251263524546	0.45333844142516	0.650305056008236	0.850502719747823
**Cnot11**	2010.89327679036	0.0582532167209736	0.128582994307894	0.453039820969525	0.650520067577666	0.85063416466759
**Cdk6**	2768.3181043618	-0.0366509802366696	0.0809231484911895	-0.452910952181501	0.650612864180897	0.85063416466759
**Gm16062**	93.773129229282	-0.138190006866804	0.305034682782659	-0.453030473801124	0.650526798160065	0.85063416466759
**Gm28900**	29.5852703109464	-0.159263630159558	0.351653776194376	-0.452898961822965	0.650621498545015	0.85063416466759
**Ilf2**	4983.70002157047	-0.0328807742127605	0.0726786699780174	-0.452412987506591	0.650971492435777	0.850644548945818
**Ube2cbp**	520.377937288473	0.0570854846261454	0.126257755627667	0.452134479520531	0.651172105823959	0.850644548945818
**Efcab14**	5957.34607898028	-0.0430848674745966	0.0952016548725877	-0.452564270361254	0.650862531769074	0.850644548945818
**Szrd1**	7438.11409713401	-0.0291470332599582	0.0644566275889999	-0.452196063464735	0.651127743829538	0.850644548945818
**Clspn**	116.272652736463	0.156742052377768	0.34626746853979	0.452661790721346	0.650792297205604	0.850644548945818
**Gm10154**	63.5183041730313	-0.134388917207896	0.296957797613962	-0.452552242398422	0.650871194572008	0.850644548945818
**Gm14540**	30.4249115307704	-0.561739833887215	1.2406870832305	-0.452765118199309	0.6507178837148	0.850644548945818
**Trav7d-3**	301.904195270041	-0.0640256155003451	0.141629712424994	-0.452063443497088	0.65122327815786	0.850644548945818
**9330179D12Rik**	33.3576212083319	-0.285733832451591	0.631960634776473	-0.45213865663113	0.651169096803273	0.850644548945818
**Gm37718**	123.732311992123	0.126627865015846	0.279890749663939	0.45241889975959	0.65096723402714	0.850644548945818
**Gm31597**	131.664021487139	-0.132485190859162	0.292973818674626	-0.45220829444251	0.651118933391692	0.850644548945818
**Icam5**	38.0972137936196	-0.220688334371114	0.488683300039814	-0.451597863796726	0.651558709223234	0.851002444535907
**Gm2808**	33.009436119377	0.224564782531026	0.497338338091962	0.451533222619773	0.65160528611574	0.851002444535907
**Rpl27**	6678.29017797969	-0.0367639731220172	0.0814351339923335	-0.451451005477295	0.651664529250773	0.8510092812369
**Vps45**	1681.90770799517	0.047952624077817	0.106354763031957	0.450874250581598	0.652080182826756	0.851078319022936
**Smpdl3a**	10702.2449154444	0.041019433354258	0.090932866478359	0.451095791245293	0.651920510931184	0.851078319022936
**Lima1**	91.5352741540723	0.162268771631384	0.359795779616994	0.451002432002178	0.651987796169919	0.851078319022936
**Caprin1**	21794.3357782767	0.0346853940393975	0.0769711977979347	0.450628222396303	0.652257522404073	0.851078319022936
**Ipo5**	4903.07749675034	0.0371500425919081	0.082437214700094	0.450646503852171	0.652244344271451	0.851078319022936
**Myo5a**	534.211276658535	-0.0574593537708179	0.127445279535127	-0.450855096245294	0.652093988757989	0.851078319022936
**Elf1**	26694.9652396953	-0.0318862913416365	0.07072334903533	-0.450859465460377	0.652090839534593	0.851078319022936
**Ndufb4c**	74.2453685597904	0.141314760914293	0.313182584853295	0.451221644334053	0.651829811433673	0.851078319022936
**Gm13842**	912.349974830575	0.0643962728017207	0.142897097128048	0.450647872461791	0.652243357717679	0.851078319022936
**Tmem147os**	423.206328348132	0.103033238082076	0.228635322182513	0.450644445917363	0.652245827722161	0.851078319022936
**Brpf1**	6544.27413730336	0.046264076046491	0.102808852948012	0.450000897003351	0.652709793788184	0.851104619390964
**Grik5**	37.4469165294649	-0.298330262079628	0.662482358130878	-0.450321821280395	0.652478406884409	0.851104619390964
**Abhd1**	106.779743412823	-0.127400418567011	0.282954929118067	-0.450249864754438	0.6525302847443	0.851104619390964
**Nutf2**	5690.24092231724	0.0414887744567039	0.0921095088358391	0.450428788309431	0.652401290918742	0.851104619390964
**Opa1**	3782.82678214856	-0.0450936061641815	0.100198640036426	-0.450042097854703	0.652680086040004	0.851104619390964
**Retreg2**	7522.70321777644	0.0484013592330703	0.107550951885089	0.45003190008754	0.652687439057612	0.851104619390964
**Gm42502**	17.0503941172343	0.680004404805962	1.51076142467676	0.450107074286368	0.652633236101547	0.851104619390964
**Gm43693**	42.9711847119701	-0.20728141343167	0.460537864414335	-0.450085496651332	0.65264879406579	0.851104619390964
**Jade3**	505.683177145676	-0.0964253325411311	0.214372237580354	-0.449803265709664	0.652852302886612	0.851149572777126
**Msantd4**	1284.80473544609	0.0730120269878118	0.162317434934524	0.449810132948834	0.652847350806208	0.851149572777126
**3222401L13Rik**	18.0582165957961	-0.278625567824993	0.619689833928947	-0.44962100807505	0.652983737488533	0.851250496534029
**Spata21**	25.7327727994937	-0.291199715187897	0.648390489456074	-0.449111638624096	0.653351125000734	0.851384146663223
**Ticam1**	753.065626656797	-0.0728527325453585	0.162153510559734	-0.449282487279368	0.653227889427473	0.851384146663223
**H1f5**	23.0933475261239	-0.338463819061792	0.753438302338613	-0.44922566056335	0.653268878298986	0.851384146663223
**Lbhd1**	1604.63480505746	-0.0523941704705151	0.116593704840772	-0.449373922392021	0.653161939861458	0.851384146663223
**1810006J02Rik**	527.360029413867	0.0845002792453853	0.188152906691394	0.44910429889867	0.653356419461299	0.851384146663223
**Tubgcp5**	3884.01732881294	-0.0405122859119448	0.0902439581884371	-0.448919647643909	0.653489622118488	0.851416898935094

**Nhlrc1**	156.256044832463	0.130572138493898	0.290816227533209	0.448985050117218	0.653442441196422	0.851416898935094
**Morc3**	10680.2600080832	0.0411966250423374	0.0918810134487534	0.448369293023904	0.653886699247322	0.85164735422206
**Lemd3**	384.363850627309	-0.0854921718053136	0.190609974367246	-0.448518877824288	0.653778765125659	0.85164735422206
**Fam72a**	78.9055180113868	-0.131511490370788	0.29328744897344	-0.448404767510858	0.653861101689647	0.85164735422206
**Gm1720**	22.3092760649378	-0.316168036127785	0.70485438352069	-0.448557948307779	0.653750574695134	0.85164735422206
**Gm17241**	90.1629330359284	0.109070707594169	0.243298513433087	0.448299934328067	0.653936748031522	0.85164735422206
**Frmd8**	35983.1892315167	-0.0321349336096901	0.0717276018781983	-0.44801349505953	0.654143457228632	0.851846153351367
**Gm26616**	17.0957846475739	-0.445108757062864	0.993797428357959	-0.447886807071249	0.654234890209677	0.85189481572051
**Slf1**	1322.10260341549	-0.0433288419516505	0.0968171679307305	-0.447532631636683	0.654490532461936	0.852157273178213
**Mtif2**	2229.46270289874	0.0333304006439776	0.0745389126373122	0.447154371652227	0.654763603585498	0.852442378063017
**Taf6l**	1760.31821201382	0.0572189154616696	0.127993654170953	0.447044940097154	0.654842612357303	0.852474805263882
**Gm11713**	23.0118533811328	-0.267730452365449	0.599072654896645	-0.446908150751163	0.654941378686851	0.852532945391881
**Zfp958**	1465.65677026969	-0.0472314886826111	0.105712652711031	-0.446791254134168	0.655025786607806	0.852572387211234
**Eml4**	6441.59346153648	0.0487781160710906	0.109269057439253	0.446403741500273	0.655305630643242	0.852866178412949
**Med1**	6960.50574897741	-0.0647797385358138	0.145348777989842	-0.445684782711699	0.655824958258144	0.852879679730845
**Calr3**	64.5088829117582	0.173096718375262	0.388766292316336	0.445246210374676	0.656141835246602	0.852879679730845
**Mfsd13a**	139.3268880367	-0.144400964610351	0.323978446428801	-0.44571163977751	0.655805555521027	0.852879679730845
**Lpin3**	51.0369963907763	-0.280789451926528	0.630685654809018	-0.445212999194592	0.656165833487113	0.852879679730845
**B4galt1**	12146.1324123869	-0.0469464776957917	0.105519425211433	-0.444908391054285	0.656385958427888	0.852879679730845
**Dnajc2**	3514.9949403234	-0.0358066017978572	0.0804325150399355	-0.445175707611268	0.656192780630479	0.852879679730845
**Pdap1**	1091.45235076888	-0.042312549225536	0.0951076817016314	-0.444890974824489	0.656398545160624	0.852879679730845
**Gsdme**	489.340761543388	-0.0758510995867077	0.170400333283691	-0.445134690320276	0.656222420511344	0.852879679730845
**Arhgap25**	11031.3603812918	-0.0315294501409262	0.070738014612613	-0.445721445726077	0.655798471326163	0.852879679730845
**Map4**	8705.83779051781	-0.0673409730564688	0.151206914178021	-0.445356440362153	0.65606218612643	0.852879679730845
**Actr1b**	8705.34810895089	0.0345831822824351	0.0776044911827335	0.445633773965515	0.655861809881211	0.852879679730845
**Ccdc50**	1032.01252195509	-0.0687128821032406	0.154239698327337	-0.445494142224097	0.655962692087805	0.852879679730845
**H2-T24**	1731.96446300335	-0.0731076249050502	0.164301897605535	-0.44495910254531	0.656349309720446	0.852879679730845
**S1pr3**	49.6373367648779	0.40343474658366	0.906048762495437	0.445268249660781	0.656125909959339	0.852879679730845
**Gm4924**	556.481097080389	-0.0795542208036939	0.178608234435664	-0.44541183140327	0.656022163578201	0.852879679730845
**Ube2d3**	48429.3942707544	0.0360673073029165	0.0810609569234763	0.444940556733929	0.656362712504276	0.852879679730845
**Gm12734**	59.7217183129016	-0.184294543717292	0.413495004866243	-0.445699564803466	0.655814278989813	0.852879679730845
**Itga10**	55.685868850342	0.215215009716052	0.483604207905578	0.445023029572298	0.656303111450409	0.852879679730845
**Gm20689**	227.44923243039	-0.0688457467993233	0.154437092369328	-0.445785049065042	0.655752522576535	0.852879679730845
**Gm36949**	33.5517426951898	0.495059738754916	1.11152646692302	0.445387270107352	0.656039910110523	0.852879679730845
**Gm6254**	161.296004179694	-0.119945338158192	0.269741838924705	-0.444667162633503	0.656560303211022	0.853019516630613
**Acsl1**	966.2588836474	0.0550825171650162	0.124068480604955	0.443968660665748	0.657065242106824	0.853035518425805
**Cacnb1**	777.31481401015	0.0766982884716682	0.172887497544278	0.44363120272491	0.657309242621999	0.853035518425805
**Slc6a19os**	56.7098998430423	0.262033879663805	0.590546387475846	0.443714304618488	0.657249152024645	0.853035518425805
**Clec4n**	119.752755928799	0.119759306716436	0.269853209562321	0.443794264706637	0.657191335343995	0.853035518425805
**Gnl1**	3882.27198673984	0.0912547300595873	0.205494611853043	0.444073590235286	0.656989379748331	0.853035518425805
**Arhgap6**	33.062890977664	0.500866185467623	1.12720299581972	0.444344263921498	0.656793703432372	0.853035518425805
**Sh3rf1**	180.513689928367	-0.103209547582975	0.232296158401143	-0.444301568710177	0.656824567232715	0.853035518425805
**Ppp1r3b**	200.617927858366	0.112173917608111	0.252691643468101	0.443916213724224	0.657103161710449	0.853035518425805
**Gak**	22391.4852086625	0.0358441108362718	0.0807172183096129	0.444070194525063	0.656991834735808	0.853035518425805
**Gtf2f2**	1614.83065919892	0.0406918960997782	0.09156148251872	0.44442155129433	0.656737834907942	0.853035518425805
**Traj5**	18.0798187698342	0.239150694007513	0.538982208956878	0.443707955537818	0.65725374293753	0.853035518425805
**Gm11221**	75.4267272326761	0.121627584796951	0.273830488575947	0.444171083466543	0.656918896908427	0.853035518425805
**Gm43587**	43.0323189318201	-0.151018927661015	0.340438075170762	-0.443601755136421	0.657330536569533	0.853035518425805
**Zfp91.1**	28.8202634961071	1.36186819939334	3.06755322145327	0.443959110430087	0.657072146946115	0.853035518425805
**Morn2**	32.7688921523549	0.220366150185947	0.497014583713516	0.443379646004448	0.657491155632277	0.85316601682486
**Nqo2**	936.650646544659	0.0700707875573014	0.158061673327754	0.443312955519608	0.65753938619795	0.85316601682486
**Ptma**	24849.8734360339	0.0287454521922975	0.0648839442500254	0.443028741926185	0.657744945484031	0.853193862425725
**Renbp**	169.621900519722	-0.0870688785021887	0.196508512370328	-0.443079424152903	0.657708287344995	0.853193862425725
**Cxcr5**	3873.34318587142	0.0398133619813249	0.0898906209223254	0.442908966172652	0.657831581811902	0.853193862425725
**Gm4275**	19.4818628455921	-0.261561392792733	0.59020393202716	-0.4431712135403	0.657641898748101	0.853193862425725
**Gm2629**	34.2283155938066	0.383737450978593	0.866357239940436	0.442932122325169	0.657814832120141	0.853193862425725

**Gm14292**	104.257740109689	0.144642762849836	0.326667910216936	0.442782282330029	0.657923219907669	0.853242483628407
**Pex12**	1021.66909829871	0.069257144243376	0.156548713713275	0.442399957180249	0.658199810004897	0.853250053989894
**Dis3**	2470.43526474717	0.0441127934135966	0.0996848766330493	0.442522425703355	0.658111206028528	0.853250053989894
**Usp22**	6964.76298245971	0.0519906009667053	0.117512260601975	0.442427034424965	0.65818021964715	0.853250053989894
**Lyrm2**	1030.50044143719	-0.0573295661709886	0.129522053899046	-0.442623973641378	0.658037741395196	0.853250053989894
**Ccdc162**	128.72668471251	-0.0923202353868366	0.208542995539535	-0.442691614494119	0.657988808600646	0.853250053989894
**Castor2**	694.898893593086	0.099169598208748	0.224352484588044	0.442025852269224	0.658470498524301	0.853252569432959
**Hotairm1**	42.8366782422923	0.185688279279158	0.419898756224062	0.442221551092362	0.658328892486094	0.853252569432959
**Mrip-ps**	22.6450646005938	0.415432182569907	0.940001873577108	0.441948249516791	0.658526654621706	0.853252569432959
**Gm7774**	135.646498283198	-0.0948376424106342	0.214559322452478	-0.442011287725051	0.658481037796961	0.853252569432959
**Gm48747**	21.6350150820759	-0.535771213500145	1.21189915403076	-0.442092241518759	0.658422458443195	0.853252569432959
**Gm48766**	140.957318927552	-0.153634786385323	0.347353915447425	-0.442300430635499	0.658271819370616	0.853252569432959
**Nsun5**	4678.45138896523	-0.0296918439662055	0.0672831812351694	-0.441296672082522	0.658998235055605	0.853386306977824
**Ptprc**	187850.497286092	0.0246711324130037	0.0558801711881208	0.4415006591506	0.658850584428559	0.853386306977824
**Prrt3**	47.3834264524273	0.21875129486355	0.495451026709449	0.441519510649503	0.658836939942369	0.853386306977824
**Khnyn**	11724.6342915297	-0.0410048199850845	0.0928722915283639	-0.441518339972922	0.658837787260568	0.853386306977824
**2010315B03Rik**	1301.8032981797	-0.0639989637018561	0.14489162453141	-0.441702299279435	0.65870464563948	0.853386306977824
**Gm14326**	452.037452777433	0.0771328009956016	0.174792609606842	0.441281820604973	0.659008985422743	0.853386306977824
**Gm10257**	40.1625458830696	0.192408629976944	0.435971070588299	0.441333480492887	0.658971591281383	0.853386306977824
**Tmem40**	27.954065363599	0.260466730560283	0.590430995143554	0.441146777019988	0.659106741092135	0.853442757777038
**Lrch3**	7622.72900381221	0.0465460728766018	0.105594849258605	0.440798705650966	0.659358730664244	0.853698892282374
**Zfp184**	29.6214257447477	0.41364132614488	0.939628599182995	0.440217897267645	0.659779298484709	0.853869298036678
**Rsph1**	32.6571805254989	0.278819511135787	0.633193716435298	0.44033840497575	0.659692029079968	0.853869298036678
**Etfdh**	5916.47038586722	0.0293176680684699	0.0665514455210981	0.440526390357301	0.659555902865925	0.853869298036678
**9330198N18Rik**	43.02615061221	-0.205323236337365	0.466179903316183	-0.440437768502659	0.659620075374877	0.853869298036678
**4930481A15Rik**	1772.42468775433	-0.0898313525843632	0.204084273236673	-0.440167932392259	0.659815483460975	0.853869298036678
**AC147806.1**	284.709266846376	-0.135423928985737	0.307653487677932	-0.440183304950881	0.659804350442035	0.853869298036678
**Gm49909**	746.252756594429	1.3469601697901	3.06065665061331	0.440088622655595	0.659872921862776	0.853873501666401
**Cenpo**	2612.90115952509	0.0395189853538276	0.0899735678822182	0.439228834467927	0.660495733913606	0.853930010324301
**4930579G24Rik**	526.959131231188	0.0918755414845689	0.209292751717622	0.438981000204572	0.660675303412042	0.853930010324301
**Arl8b**	4655.08948918506	0.0469899071045722	0.107004739441604	0.439138559187053	0.660561141036532	0.853930010324301
**Neurl2**	179.792524312747	0.0686826467106367	0.15611614119893	0.439945838932299	0.659976335012348	0.853930010324301
**Tprn**	2162.65784428929	-0.0469342704961042	0.106899033378973	-0.439052337636348	0.660623613532496	0.853930010324301
**Usp47**	9069.59390738321	0.0443508712363207	0.101020830874859	0.439026989307393	0.660641980311488	0.853930010324301
**Map7d3**	23.8611969043924	0.273021899287254	0.620701033128543	0.43986055236791	0.660038108123078	0.853930010324301
**Gm10231**	401.582565168924	-0.0937775809147639	0.213529307005667	-0.43917896905962	0.660531862562749	0.853930010324301
**Tmsb15l**	461.412241989349	-0.0836244322693887	0.190200357200687	-0.439664959099702	0.660179785197817	0.853930010324301
**Trav13n-2**	165.482879805296	-0.090160541757899	0.205201205103677	-0.43937627808933	0.660388912206751	0.853930010324301
**A930029G22Rik**	472.749348904147	0.105590904269803	0.24028736392988	0.439435942626663	0.660345687700372	0.853930010324301
**Gm44174**	1248.55146620271	-0.0422314267255161	0.0960820760116012	-0.439534910969419	0.66027399170054	0.853930010324301
**Gm48719**	37.7087198657665	-0.178611148032707	0.406630325830669	-0.439246993366365	0.660482577565505	0.853930010324301
**Gm49417**	70.1406674495397	0.117526276730333	0.267601025524871	0.439184702300067	0.660527708656324	0.853930010324301
**Arhgef9**	22.4894833580685	-0.298094934430853	0.679511450164072	-0.438690082939553	0.660886113831229	0.854132422542543
**Parp3**	3220.65177608727	0.0359148249645694	0.0818915593025701	0.438565650360528	0.66097629092806	0.854178907254444
**2310022A10Rik**	3092.73757925613	-0.0311287785664784	0.071010568693609	-0.438368247701135	0.661119360011182	0.854293730990339
**Prkacb**	18540.6351461623	-0.048929283287274	0.111761143394057	-0.43780227905109	0.661529618727307	0.854364101706959
**Kcnk7**	137.914769613222	0.163909892086997	0.37443390025332	0.437753878524634	0.661564707966754	0.854364101706959
**Rhbdd2**	3056.20321582747	-0.0483426788599788	0.110343127479889	-0.438112277257953	0.661304894977659	0.854364101706959
**Zfp637**	1746.58350718677	0.0391460792091786	0.0894522279921736	0.437619946286901	0.661661809553215	0.854364101706959
**Cyp2d22**	618.085168398264	-0.0639595698960656	0.146110335624354	-0.437748429108423	0.661568658711469	0.854364101706959
**Hvcn1**	17671.97940823	0.0273858743204903	0.0625150648647173	0.438068398069384	0.661336702003968	0.854364101706959
**Gm42508**	43.988508604694	-0.174071320207121	0.397675946478969	-0.437721521123799	0.661588166733396	0.854364101706959
**Gm44283**	54.5203893912321	-0.199500216527591	0.455420386995995	-0.438057281193575	0.661344760471602	0.854364101706959
**Gm50431**	42.4800368157609	0.242721763894204	0.554547632987359	0.437693264664492	0.661608652633423	0.854364101706959
**Bcl11a**	116.525143856226	0.174394593324168	0.399283420715138	0.436768932233193	0.662278932195982	0.854880735202104
**Armc1**	3338.39477093237	0.0376232407473197	0.0861314520537786	0.43681187127588	0.662247788955654	0.854880735202104

**4930532G15Rik**	29.5627263308058	0.324770189198783	0.743251593232136	0.436958618260707	0.662141359305999	0.854880735202104
**Gm28707**	126.69336673368	-0.129548400193028	0.296553978474859	-0.436845935634653	0.662223082846622	0.854880735202104
**Nfkbie**	5832.83964350593	-0.0287538611983673	0.0658825155507393	-0.436441458830189	0.662516464453393	0.854998541305447
**Gm2991**	102.299600886849	-0.131045950087419	0.300275751872254	-0.436418689389111	0.6625329814901	0.854998541305447
**Rps2-ps6**	421.690867598812	0.0757550052205852	0.173527741487855	0.436558469389675	0.662431587150328	0.854998541305447
**Mcam**	37.5719865967781	-0.474191960588539	1.08687044330112	-0.436291154581671	0.662625498744826	0.855047906291232
**Gm10073**	200.683292892858	-0.0732031785398742	0.167828757762413	-0.436177801205586	0.662707732720678	0.855083994798536
**Gm49958**	23.5798840030886	-0.2356169539886	0.54042989471397	-0.435980607833147	0.662850799418952	0.855198562715502
**Fndc3b**	197.383569378405	0.117795568886484	0.270547530218357	0.435396947779977	0.663274325456031	0.855603333106666
**Suclg1**	9516.67840758558	0.029388135918455	0.067491896079475	0.435432068523443	0.663248837457686	0.855603333106666
**Gm26642**	31.1802982596331	-0.272355683754619	0.625639350593304	-0.435323774785489	0.663327430172687	0.855603333106666
**Golga2**	2920.3581817042	-0.0493045195486813	0.113337925395682	-0.435022252053325	0.663546275733374	0.855773989205486
**Trbj2-1**	1010.94108267851	0.0401817728568704	0.0923736263271492	0.434991831051031	0.663568356927881	0.855773989205486
**Abi2**	430.761493660754	0.0920372526042961	0.211721305269238	0.434709452066034	0.663773336677425	0.855968284313462
**Taok3**	12399.7233135962	-0.0475617312001463	0.109528105322799	-0.434242252798707	0.66411253327397	0.856256575697922
**Gm7889**	77.7048294817307	0.215654460968246	0.496692942772027	0.434180642399881	0.664157268870382	0.856256575697922
**Gm45464**	42.890487172793	-0.192534806305778	0.443447742532515	-0.434176990520276	0.664159920554674	0.856256575697922
**Phactr4**	304.530455155413	-0.0770579127467085	0.17751223676909	-0.434099159298782	0.664216435966032	0.856259378345477
**Fkbp10**	17.5824700925711	0.289085264675271	0.66674620862083	0.43357616576965	0.664596245602592	0.8562789609443
**Ap1s1**	2875.93082704632	0.0407273146324207	0.0938418310223307	0.433999573417628	0.664288750824536	0.8562789609443
**Ctu2**	5233.5659978304	0.0421187714677051	0.0970776981118351	0.433866606717267	0.66438531022879	0.8562789609443
**Atp5k**	219.540607705743	0.100334348141805	0.231315662037513	0.433755100100108	0.66446628977886	0.8562789609443
**Pgm3**	1715.53914906166	0.0388534134780852	0.0896159973943926	0.433554439026043	0.664612025915389	0.8562789609443
**Llph-ps2**	142.424008880741	0.0865697094713017	0.199579314544325	0.433760931933029	0.664462054424559	0.8562789609443
**Gm13807**	107.513587122943	-0.204945451076804	0.472589865923732	-0.433664506699091	0.664532084389134	0.8562789609443
**Asf1a**	3370.80753361503	0.0533406799599389	0.123075253112937	0.433398905229082	0.664724995733391	0.856354489229911
**Ndrg1**	1080.73206643577	0.067143191392542	0.155188620122943	0.43265537988127	0.665265150246175	0.856436338976961
**Zmynd10**	108.182043074392	-0.13343621129371	0.308703357941955	-0.432247359352663	0.665561641890356	0.856436338976961
**Rab11fip4**	1255.65956128339	-0.0766305246606816	0.177338457945302	-0.432114531436363	0.66565817372919	0.856436338976961
**Zfp263**	9065.96310872438	0.0377257024723072	0.0872243097073993	0.432513626062058	0.665368150953569	0.856436338976961
**Rab33b**	1370.83677270894	0.0526319995661862	0.121671912718835	0.432573125465782	0.665324916917702	0.856436338976961
**Nup210**	30643.6641767505	-0.0691216655825095	0.159906264356794	-0.432263650586449	0.665549802721901	0.856436338976961
**Tmem159**	138.208936510071	0.106430151350664	0.245844444902352	0.432916641223832	0.665075329915182	0.856436338976961
**Thap11**	3724.06404096768	-0.0400655494372055	0.0926216351895641	-0.432572253288396	0.665325550659688	0.856436338976961
**Fer1l5**	112.312982929671	0.154707108010399	0.357982440910536	0.432163956469202	0.66562225375526	0.856436338976961
**Pnrc1**	13895.4225282263	0.166954342181354	0.385632167284475	0.432936762918416	0.665060711318724	0.856436338976961
**Ssh1**	1534.57379235492	0.0513293193586879	0.11878602659118	0.432115803783433	0.665657249032807	0.856436338976961
**Gm9949**	31.1114167038475	0.184999827794567	0.428035307422664	0.43220693383569	0.665591020289697	0.856436338976961
**Gm5526**	166.209352875965	0.136632270666059	0.316129733817168	0.432203162341824	0.665593761170197	0.856436338976961
**Gm11579**	84.4218454614747	0.167009093393235	0.386185343046562	0.43245839439613	0.665408284920029	0.856436338976961
**Trav7-2**	289.729115632214	0.121551295533163	0.281206988818315	0.432248487293805	0.665560822190073	0.856436338976961
**Gm37933**	28.883297172059	-0.397328666789286	0.91849836689599	-0.432585055248421	0.66531624851684	0.856436338976961
**B3gnt5**	1437.65449667801	-0.0337007030188117	0.0780110463789316	-0.431999115293411	0.665742056172044	0.856466191358743
**Gm6161**	254.132369312245	-0.106113773786131	0.245671818753164	-0.431933032957057	0.665790085546312	0.856466191358743
**Gm43412**	53.9157464667763	-0.209414271771392	0.485158143913328	-0.431641258419858	0.666002166900243	0.856669072967113
**Fadd**	1840.41880862139	0.04239318701153	0.0982650095093482	0.431416912522631	0.666165254750577	0.85680890695493
**Tmem234**	10429.6593900158	0.0266097200279156	0.0617288065221881	0.431074591055813	0.666414135148439	0.856991677709892
**Gm10291**	32.5788970805144	-0.19877603286775	0.461157639309781	-0.431037059616446	0.666441424130953	0.856991677709892
**Gm49215**	24.9106649117397	-0.28673505954058	0.665283128307323	-0.430997040718766	0.666470522222315	0.856991677709892
**Tubb1**	293.422650869686	0.0911512984952377	0.21163852384418	0.4306933200987	0.666691376504055	0.857098935855656
**Gnpat**	9615.01028894114	-0.0324258518777197	0.0752892082896779	-0.430683926877808	0.666698207363842	0.857098935855656
**Apobec2**	182.485444525116	-0.104847191802666	0.243458176116674	-0.430657920284506	0.666717119805977	0.857098935855656
**Bag4**	2335.76363886286	-0.0432824141125807	0.100528511983184	-0.430548639970128	0.666796592645905	0.857131172321873
**Sec24b**	6567.79988473866	0.0581460384736705	0.135277195390824	0.429828828914461	0.667320160276378	0.857246755128772
**Cs**	18956.8092378848	0.0249468562144877	0.058013684301301	0.430016754063114	0.667183453873584	0.857246755128772
**Ankrd52**	732.108609724297	0.0926681016131776	0.215669170552973	0.429677090033674	0.667430550988118	0.857246755128772

**Cemip2**	127.932243787179	0.123875671977459	0.288109268663368	0.429960731746529	0.667224206227773	0.857246755128772
**Gstk1**	581.396658454859	-0.0637879218752886	0.148447560351984	-0.429700034975592	0.667413857978709	0.857246755128772
**Zfp330**	3906.81018971846	-0.0479853546316153	0.111603575763862	-0.42996251959835	0.667222905674269	0.857246755128772
**Ngp**	1120.805315367	0.270231669750843	0.628741267441816	0.429797889440827	0.667342668297005	0.857246755128772
**Gm9762**	131.069487108045	0.090713325752178	0.211073659032941	0.42977094426558	0.667362270767032	0.857246755128772
**Mgat2**	8656.01143147755	-0.0566973462700818	0.131945240062089	-0.429703612221268	0.667411255459354	0.857246755128772
**Lrrc61**	2247.51619326182	0.046459370737623	0.107981274389742	0.430253958384812	0.667010915042357	0.857246755128772
**Flii**	16561.729690977	0.0433406753867056	0.100897043200616	0.429553473638771	0.667520487786434	0.857292389440936
**Ttll5**	1273.79684317272	-0.0594857176363659	0.138509566461911	-0.429470101999952	0.667581147310032	0.857300418758287
**Septin2.1**	2765.58217157655	-0.575526034486729	1.34033342006366	-0.429390199387399	0.667639284878369	0.857305208363497
**Syce1**	65.4906819399946	-0.684382415478409	1.59473531812491	-0.429151099684119	0.667813267015472	0.857455713347282
**Gm10358**	323.136190013112	0.0553892430710964	0.129088515104936	0.429079558518977	0.667865327785187	0.857455713347282
**Mcm3ap**	4917.57970550722	-0.0562867865533614	0.131283227830044	-0.428743164558909	0.66811014433133	0.857560406014074
**Srrt**	7049.33413606708	-0.0359255987172341	0.0837920927540081	-0.428746884538405	0.668107436858524	0.857560406014074
**Gm35013**	48.6692966708978	-0.168796653585262	0.393597739083248	-0.42885574998072	0.668028204402943	0.857560406014074
**Gm47322**	19.3538723117866	0.411087955716925	0.959074124214887	0.42863001444591	0.668192499228421	0.857596253998715
**Scly**	2056.99544265884	0.0423787076609594	0.0990293877372072	0.42794072173221	0.668694278573882	0.858133933186064
**Tmem181a**	1136.73591117721	-0.0439623414585067	0.102756538381213	-0.427830113305415	0.668774811164871	0.858133933186064
**Gm12418**	156.442435727354	-0.110607082733137	0.258503678564168	-0.42787430858815	0.668742632683523	0.858133933186064
**Ap2b1**	4957.68888394288	-0.0503247350517329	0.117761619578437	-0.427344114592558	0.669128705805949	0.858168730049189
**Pafah2**	423.607496088442	0.0625877410234434	0.146440909931011	0.427392461935185	0.669093496934537	0.858168730049189
**Arel1**	3732.07404715322	-0.0354650040126967	0.0829503818383881	-0.427544795173976	0.668982565265892	0.858168730049189
**Kiz**	1624.98365992375	0.0368628829924273	0.0862115220607875	0.427586500171467	0.668952196233397	0.858168730049189
**Gm45481**	30.7999966942548	-0.246142664931664	0.575810353619216	-0.427471759381455	0.669035750277135	0.858168730049189
**Gm9045**	227.714341919898	0.080455808249069	0.188146797831863	0.427622522286923	0.668925965836993	0.858168730049189
**Cenpf**	100.715467393074	-0.175206840354415	0.410073450561906	-0.427257214809534	0.66919199225641	0.858180045738117
**Ms4a6d**	882.32243029876	0.0910202736163966	0.213242296758285	0.426839679557429	0.669496103154186	0.858421019719018
**Sem1**	2871.37488780179	-0.0334058519045697	0.0782660749158696	-0.42682416283784	0.669507405768211	0.858421019719018
**Gm44746**	17.1380634907028	-0.331807653675329	0.777477077082776	-0.426774838070245	0.669543335174636	0.858421019719018
**Ap2s1**	6305.35364272293	-0.0353045730844015	0.0827421794904775	-0.426681691270467	0.669611187718768	0.858438164908033
**Uhrf1bp1l**	6496.80515034604	0.0301780019678525	0.0707765013976405	0.426384483153592	0.66982770622669	0.858491286950458
**Ddx19b**	1839.56440116963	-0.0573166364438422	0.134443316129621	-0.42632566715761	0.669870557406605	0.858491286950458
**Atxn7l1os2**	84.7105618949582	-0.141266411124121	0.331327527764028	-0.426364848334399	0.669842011317136	0.858491286950458
**Gm4613**	17.6239042909209	-0.199237161440667	0.467330310144353	-0.426330492835197	0.669867041554242	0.858491286950458
**Dgkg**	19.7650772573107	0.418605006438807	0.982769130307363	0.42594439887208	0.670148361564667	0.858637805592328
**Gm5578**	20.182266433928	-0.246706498865088	0.579101668762744	-0.426015865905168	0.670096284982026	0.858637805592328
**Gm43581**	200.613367002585	0.166099851519419	0.389956445335886	0.425944624088338	0.670148197451641	0.858637805592328
**Zc3h15**	5041.38726860998	0.0496403475425051	0.11661358662616	0.425682366683756	0.670339312684645	0.858723115872269
**Hspa13**	740.529505112162	-0.0656400459001658	0.154202315495843	-0.425674839506125	0.670344798291166	0.858723115872269
**Csde1**	25333.6836119124	0.0251830553209411	0.0591667255243727	0.425628680609802	0.670378438049425	0.858723115872269
**Pfdn5**	4821.45508503207	0.0294291573877681	0.0691868444782508	0.425357705062257	0.67057593339209	0.85883646122565
**Ube2z**	2588.41105120403	0.0797608351381334	0.187510533299894	0.425367224627154	0.670568994853478	0.85883646122565
**Hspb11**	246.760655167328	0.0751707345742864	0.176840657592144	0.425076085996333	0.670781210183045	0.859029545583082
**Scp2-ps2**	2011.11718345087	-0.0427052646704277	0.100522546176426	-0.424832699675915	0.67095863829153	0.859186937306756
**Msgn1**	26.4104709566431	0.20896447459231	0.49224091628151	0.424516669948673	0.671189050665234	0.859342315050955
**Gm33994**	163.226100236161	-0.114543109893886	0.269818434459181	-0.424519214646968	0.671187195241681	0.859342315050955
**Cdkal1**	2536.94395663122	-0.0490119321738795	0.115539906577142	-0.424199167420615	0.671420567965759	0.859510316128051
**Gpr68**	10354.2120813847	0.0449045596310937	0.105860265417265	0.424187106031665	0.671429363534948	0.859510316128051
**Myo5b**	40.7154092566553	-0.219688061988923	0.518760621024821	-0.42348638868333	0.671940427332073	0.860066452355921
**Pop4**	1426.802177063	0.0519662251632135	0.122723425282844	0.423441776037832	0.671972970422907	0.860066452355921
**Gm49288**	22.3323428164946	0.273932208574645	0.647075752156	0.42333870132195	0.672048161541642	0.860092826625378
**Noc3l**	842.257630611948	0.0490565948778314	0.115909735596161	0.423231013559969	0.672126721297691	0.86012350747078
**4833413E03Rik**	17.892950031649	0.262972439438493	0.622102580856616	0.422715557740313	0.672502803233485	0.860171230081943
**Hook1**	1377.99611245485	-0.0458479138615018	0.108487043227438	-0.422611885231161	0.67257845367803	0.860171230081943
**Rbmx2**	696.610437559663	0.0574297373655223	0.135859389257123	0.422714526243252	0.672503555906678	0.860171230081943
**Gm5422**	320.868832282195	0.0615413241334827	0.14548130441484	0.423018781561084	0.672281558044742	0.860171230081943

**Eif1-ps1**	17.9543383101629	0.240818499821439	0.569613734949606	0.422775093094875	0.672459361430821	0.860171230081943
**Rnps1-ps**	165.227855254118	0.10100543028484	0.2390200816632	0.422581356269325	0.672600731474241	0.860171230081943
**Gm37164**	120.760664863965	-0.0982245385327717	0.232429732316961	-0.422598854086466	0.672587962816079	0.860171230081943
**Gm9794**	1867.44528912454	-0.0376047914256688	0.0888957211612213	-0.423021388818802	0.67227965579999	0.860171230081943
**Hltf**	6631.69970387741	0.0253256600346088	0.0599656437901424	0.422336165075443	0.672779664438162	0.860190617701405
**Ache**	28.4482396965079	0.527662330799042	1.24913999786946	0.422420490656792	0.672718124141147	0.860190617701405
**Alg10b**	1337.05746588153	-0.0501619963966427	0.118759287837574	-0.422383775703075	0.672744918221923	0.860190617701405
**Zfp516**	270.414512136312	-0.124691154682305	0.29536326482619	-0.422162027345143	0.672906756031797	0.860283306676919
**Gm19680**	167.155143834788	0.0875220055871175	0.207392772012178	0.422010876936338	0.673017078277097	0.860354543437629
**Gm43071**	28.6175824927427	-0.28251951030175	0.669758695729029	-0.421822832765507	0.673154338507546	0.860460202146784
**Elp3**	6981.34305288688	0.025929088866604	0.0615546536446525	0.421236857513478	0.673582132813747	0.860727732280124
**Cttn**	19.7355542342639	0.398187177595272	0.945185614973993	0.421279345862906	0.673551110418828	0.860727732280124
**Trav13-2**	136.869537384237	-0.130108627362809	0.308765184730462	-0.42138373688856	0.673474892832811	0.860727732280124
**Gm3511**	32.1421901546255	-0.183715127189561	0.436065322998106	-0.421301849746853	0.673534679683641	0.860727732280124
**Ldha-ps2**	98.1195503336712	0.110176563696198	0.26164527986127	0.42109134838823	0.673688378886729	0.860745228361574
**Rpl10a-ps1**	816.853631295192	-0.052524956160728	0.12474207557283	-0.42106848005797	0.673705077196376	0.860745228361574
**Ndufa4**	2525.7005614382	0.0475464461106941	0.11295121320682	0.420946750024137	0.673793966400457	0.86078900020853
**Trdmt1**	692.333576805028	0.092304797031413	0.219598505184091	0.420334359535067	0.674241212736163	0.860802040923976
**Dtd1**	3775.20293770617	0.0381369626449965	0.0907299540951416	0.420334861020707	0.674240846439694	0.860802040923976
**Cdk11b**	7283.49038209493	-0.0271306741060774	0.0645263939343633	-0.42045855117326	0.674150502709471	0.860802040923976
**Kcnh5**	23.2051799870681	-0.282352185494182	0.671379012517535	-0.4205555732751	0.674079640706976	0.860802040923976
**Immp1l**	2389.14386962008	0.0575051492608269	0.136724924235844	0.420590097834928	0.674054425712356	0.860802040923976
**Gm2423**	935.978767705753	-0.082690699556917	0.196695929798953	-0.420398630726304	0.674194268231783	0.860802040923976
**Gm16244**	26.8128888332204	-0.229442413705033	0.545748708022816	-0.4204176946864	0.674180343914139	0.860802040923976
**Gm34678**	39.3275181310771	0.248676051523983	0.591051687308009	0.420734864418706	0.673948699498619	0.860802040923976
**Eif3s6-ps2**	138.861581325814	0.0876232861542229	0.208526848050938	0.420201460738613	0.674338287748068	0.860856226204837
**Gm20517**	57.0987092594265	0.268827417456958	0.63994679094453	0.420077764684438	0.674428645551324	0.860894937114245
**Gm47882**	1526.33378113048	0.0482172423127583	0.114800125137594	0.420010363707944	0.674477882761652	0.860894937114245
**Gpr137b**	128.573270588534	0.129585708183205	0.308616323985755	0.419892591907052	0.674563919800375	0.860935014117489
**Gpnmb**	47.5285120633295	0.338286646666856	0.806058190946344	0.419680180000025	0.674719106000665	0.861063331436987
**Gm49326**	23.8811812741478	-0.237831542524488	0.567342846751842	-0.41920250495115	0.675068141560228	0.861438994700722
**Tmem230**	3679.87961196974	0.0390755386348608	0.0932415118225289	0.419078775870078	0.675158561385903	0.861484610232219
**Rmnd5b**	12799.1464189737	-0.0291337046627145	0.0696288234349582	-0.41841443278054	0.675644136014098	0.861642564450203
**Rrnad1**	3037.72282917346	0.0385402197965422	0.0921657176728962	0.418162205749047	0.675828526494801	0.861642564450203
**Kif11**	295.742964019788	0.155138256291106	0.370951366667112	0.418217238785172	0.67578829295436	0.861642564450203
**Atp5mpl**	1303.35858492696	0.0558133924007369	0.133356250234889	0.418528507680962	0.675560747988999	0.861642564450203
**Acp6**	4814.2391023525	-0.037695753608267	0.0901091069634702	-0.418334559941302	0.675702524919633	0.861642564450203
**Nfkbid**	3719.40368288955	-0.0505031221053804	0.1207543392965	-0.418230288034411	0.675778753047792	0.861642564450203
**Taf10**	9095.4547533041	0.166105290481787	0.396746705112091	0.418668355254161	0.6754585257364	0.861642564450203
**Wbp1l**	8084.19231466573	-0.0465888712026845	0.111269305681605	-0.418703710940714	0.675432683275928	0.861642564450203
**Fbh1**	3916.20854043637	0.0500450926685574	0.119678917970353	0.418161306245722	0.675829184111193	0.861642564450203
**Gm12350**	50.4721904691683	0.132109713722648	0.315621125757268	0.418570567498225	0.675530003535156	0.861642564450203
**Rps19-ps3**	44.8305387519539	0.138284598877432	0.331081937574613	0.417674850795108	0.676184862245678	0.862026284336987
**Tnpo3**	11525.9453182601	0.0354719618432495	0.085005071994584	0.417292298105571	0.676464621315842	0.862075832692398
**4930402H24Rik**	1089.41425378937	-0.0562884376455721	0.13491004768101	-0.417229395535195	0.67651062596074	0.862075832692398
**Kcnq5**	80.1793515334038	0.180500701100188	0.432422340617801	0.417417612703144	0.676372974390718	0.862075832692398
**Troap**	44.0881832269472	0.288704585515631	0.691806155119153	0.417320059064676	0.676444318347301	0.862075832692398
**Rmi2**	521.088639836323	-0.0564224356767132	0.135166914994522	-0.417427857098016	0.676365482520275	0.862075832692398
**Ttbk2**	150.19375132368	-0.114226186062058	0.273810220147754	-0.417172835989901	0.676551992579438	0.862075832692398
**Pctp**	803.997462472433	0.0632789711783479	0.151758490122028	0.416971538972652	0.676699225460226	0.862193716792818
**Dmxl1**	1627.11252330715	-0.0927145435549888	0.2226774940452	-0.416362434616627	0.677144812478491	0.8623448171947
**Acer2**	667.003730176492	-0.0581992726746439	0.13974692208098	-0.416461928520465	0.677072020521452	0.8623448171947
**Urb1**	2978.93407381034	-0.0521995377983904	0.125346176019288	-0.416443001742295	0.677085867541814	0.8623448171947
**B3gntl1**	1082.05638808423	0.0718714391170756	0.17258411601059	0.416442954186267	0.677085902334418	0.8623448171947
**Slc30a7**	5131.45164161542	-0.0336871740235734	0.0809086577138093	-0.416360559864087	0.677146184118171	0.8623448171947
**Scoc**	1168.54184020425	0.0557000286805273	0.133757746487812	0.416424694218383	0.677099261613406	0.8623448171947

**Shisa5**	427896.812559595	-0.0372964790597654	0.0896103359108423	-0.416207334574368	0.677258293139702	0.862412961123995
**Gm48961**	37.0073381114689	-0.23079938028379	0.554622458752008	-0.416137818874349	0.677309157448082	0.862412961123995
**Tmed10-ps**	389.20045195019	0.0545503525669716	0.131147727304332	0.415945847390753	0.677449629714984	0.862522124710649
**Asb3**	2845.199204812	-0.0276833702793592	0.0666069391723781	-0.41562291592044	0.67768595530011	0.862623226516778
**Uhmk1**	2907.51720958452	0.0911867929149877	0.21944266912981	0.415538114244531	0.677748019564493	0.862623226516778
**Cd53**	66088.744633571	-0.0263089226400188	0.0633017395950696	-0.415611368791955	0.677694406231279	0.862623226516778
**Gm8899**	77.502630543566	-0.1414680760089	0.340397076670707	-0.415597211916578	0.677704767198388	0.862623226516778
**Skiv2l**	11508.6427680839	-0.040300574795973	0.0970251603668861	-0.415362104464269	0.677876843781053	0.862658443753197
**H4c9**	120.309409826249	0.142446249126345	0.342954149195852	0.415350709301369	0.677885184397543	0.862658443753197
**Tjp3**	1110.48161754712	-0.046289859740048	0.11163970333345	-0.414636176538267	0.678408260885481	0.863114975112839
**Rps23**	17391.5889575356	-0.0274696285684963	0.0662463843295851	-0.414658533390004	0.678391892115133	0.863114975112839
**Utp3**	4462.8630538186	-0.0406013497000522	0.097898030657492	-0.414731015806649	0.678338824504518	0.863114975112839
**Zfp24**	3710.41201704608	-0.0544477762156579	0.131366085587278	-0.414473613735589	0.678527287268076	0.863196711245202
**Gtf3a**	4840.24982188958	-0.0209213700880952	0.0504977421426711	-0.414303079709706	0.678652158696771	0.863262619511213
**Rell2**	471.669654614717	-0.0678649356477497	0.163854354218875	-0.414178408448617	0.678743453266765	0.863262619511213
**Slc39a3**	3425.7857050907	0.0326997864648429	0.0789493621605715	0.414186835332961	0.678737282259125	0.863262619511213
**Nfam1**	1813.84875246174	0.0548407803813656	0.132475300029502	0.413969852260405	0.678896185831019	0.863378455740627
**Tmem42**	1823.02563867189	0.0472248768755459	0.11409609592612	0.41390440656379	0.678944116588317	0.863378455740627
**Pwp2**	6978.52985925085	0.0341576556160891	0.0826110483155133	0.413475634465163	0.679258170436931	0.863618902512233
**Map2k5**	2765.71280097049	-0.0316875178314986	0.0766326522186145	-0.413498905676679	0.679241124023503	0.863618902512233
**CAAA01066123**	933.250584607179	0.0416586519479238	0.100765500635226	0.413421773179388	0.679297625038816	0.863618902512233
**Nemf**	9328.35209395921	0.0283738429976955	0.0686631503006026	0.413232467101739	0.679436302963456	0.863655842674667
**Atp6v1c1**	5999.50553309246	0.0346242688905015	0.0837814347694422	0.413268989553401	0.679409547257681	0.863655842674667
**Papss2**	62.803606187654	-0.17736796135616	0.42940527729807	-0.413054917424876	0.679566378486784	0.863751506478684
**Ddx21**	6814.1587078786	0.0344167490600187	0.0833816387948119	0.412761724972958	0.679781196502122	0.863815509135801
**Gm12854**	49.0884335855561	0.143086770441394	0.346622951671871	0.412802354117756	0.679751426542984	0.863815509135801
**Gm44550**	22.1886817628965	-0.309696682423373	0.750201963488289	-0.412817744415578	0.679740149829133	0.863815509135801
**Fcgr2b**	309.633770853857	-0.0993231380113856	0.240798084011809	-0.41247478533307	0.679991458342279	0.863943348580817
**Prkx**	4274.5368330937	0.0283126578778105	0.0686387337833199	0.412488056192709	0.679981733255498	0.863943348580817
**Actr2**	19651.9038813498	-0.0263575247565594	0.0639471029495037	-0.412176995373392	0.680209697328427	0.86404871022327
**Dgcr8**	3991.33939915843	-0.0332783902645811	0.0807459107448337	-0.412137159115644	0.680238893845254	0.86404871022327
**Fbxo38**	9143.67171742357	0.0236826586497454	0.0574487443690058	0.412239795836555	0.680163671017246	0.86404871022327
**Scfd1**	6746.1484509441	0.0384775539733375	0.0934559384351769	0.411718662479927	0.68054564449153	0.864227986739467
**Rest**	4669.54984254482	-0.0301381385686978	0.073213852807445	-0.411645302262158	0.68059942168243	0.864227986739467
**Tmc3**	75.1095184382992	-0.143597946855915	0.348669414738874	-0.411845549927166	0.680452632646905	0.864227986739467
**Pou4f1**	34.200316528947	-0.215725475801081	0.52392486258847	-0.411748880813332	0.680523493215475	0.864227986739467
**Pdk3**	3208.05863761849	-0.0395694647705671	0.096174692733948	-0.411433233065061	0.680754889536529	0.864286100590371
**Gm16364**	33.074272056096	-0.201066383470835	0.488655777850452	-0.411468343534801	0.680729149126263	0.864286100590371
**Homer1**	206.138210302826	0.103314867315664	0.251162881561953	0.411346082164535	0.680818783764415	0.86429758103254
**Mtfr2**	27.5653367719429	-0.242026397790322	0.589130109440628	-0.410819942678068	0.681204568825266	0.864349719931603
**Sil1**	1999.24800921163	0.042872426857485	0.104361923099794	0.410805259083708	0.681215336579958	0.864349719931603
**Rab28**	2199.50374136899	0.0548447997509036	0.133488105365654	0.41085907692428	0.681175871266987	0.864349719931603
**Clec4a2**	23.385668302798	0.351299410657069	0.855373253883643	0.410697212079133	0.681294571468852	0.864349719931603
**Scn11a**	29.8952754134679	-0.245179733330469	0.596539546175626	-0.411003318895284	0.681070101082932	0.864349719931603
**Mroh8**	59.1814862210271	-0.142186011780024	0.346199846054783	-0.410705011571624	0.681288851693034	0.864349719931603
**Gm16094**	43.2688682068706	-0.185970010170534	0.452821568377753	-0.410691590590035	0.681298694012218	0.864349719931603
**Gm20570**	24.2506898172445	-0.17108823142462	0.416172423632016	-0.411099394648738	0.680999653846032	0.864349719931603
**Trbv23**	145.248575230648	0.092880757518907	0.226251836685351	0.410519352592379	0.681425010106136	0.864440373902455
**Klc3**	192.89517222099	-0.138395762361168	0.337370113202752	-0.410219390945264	0.681645017632635	0.864649858544311
**Rps6ka5**	663.374866623318	-0.0550641628959142	0.134358843697707	-0.409829091859429	0.681931323880535	0.864664993191083
**Zfyve9**	38.819390131096	0.401469318792349	0.97949665721332	0.409873087198209	0.681899048543446	0.864664993191083
**Tk2**	8346.55620268924	-0.0406376853902836	0.0991151498167169	-0.410004781967545	0.681802439672072	0.864664993191083
**Ambra1**	4089.9103369238	-0.0655734226818661	0.159961728763601	-0.40993194552663	0.681855870506845	0.864664993191083
**Traj41**	18.0847262548104	-0.208169529317555	0.507885107425946	-0.409875238068303	0.681897470662635	0.864664993191083
**Tsn**	7493.06484580764	0.0283077859836857	0.0690913161724812	0.409715540995304	0.682014628403906	0.864701037959474
**Hsdl1**	23792.7028119331	-0.0409477017250037	0.0999857411827117	-0.409535412156187	0.682146784596252	0.864799009162695

**Eif3h**	10506.0000809967	0.0306832103233873	0.0749601956494645	0.409326710763548	0.682299915974728	0.864801635669294
**Nub1**	12119.9303225417	-0.0205141713150075	0.0501191325032724	-0.40930818811894	0.682313507307271	0.864801635669294
**Gm28180**	22.5430873566402	-0.19896672490396	0.485946238508706	-0.409441845901634	0.682215435752079	0.864801635669294
**Cd14**	41.2104559948246	0.326076960932221	0.796982170492685	0.409139593086059	0.682437221763056	0.864888868601341
**4930594C11Rik**	36.6362182112066	-0.33609804392197	0.822437119317127	-0.408661083051593	0.682788397255685	0.865264337747936
**Bbs4**	1420.43555462734	0.055999844247215	0.137157639781809	0.408288188221232	0.683062110034011	0.865409723435763
**Slc39a10**	1858.92278401554	0.0360100320311489	0.0881992853009218	0.4082803155183	0.683067889215624	0.865409723435763
**Wdr45**	4865.77744629717	-0.031767542574895	0.0777833968033687	-0.408410327659015	0.682972452483972	0.865409723435763
**Gm10155**	109.3455084563	-0.0870936065593189	0.21341906104026 7	-0.408087291429355	0.683209589851144	0.865519658086869
**Ifit3**	3126.14532243505	-0.0750610584139585	0.183987728218428	-0.407967744048924	0.683297356203884	0.865561254257143
**Prxl2a**	740.33488368636	0.0412824741506392	0.101287639266309	0.407576624844595	0.683584528378881	0.865577098494537
**Gm11520**	24.2398305256375	-0.269145463247	0.660101883027527	-0.407733215382717	0.683469549121601	0.865577098494537
**1700018L02Rik**	64.6949239967211	-0.209096234044116	0.512964375531359	-0.407623304888418	0.68355025199749	0.865577098494537
**Pvrig**	113.520724444459	0.161663329158634	0.39643285754321	0.407794979862417	0.68342419952394	0.865577098494537
**Gm35315**	275.401701821989	0.0923470388133192	0.226524029213009	0.407669946248757	0.683516004672128	0.865577098494537
**mt-Tn**	72.8673105555019	-0.161708700349165	0.396874353490607	-0.407455656751053	0.683673356276712	0.865589725805854
**BC085271**	36.1601611248936	-0.182420215943311	0.447752097775001	-0.407413425531238	0.683704368051003	0.865589725805854
**Selenof**	19134.6761671814	0.0413918649235017	0.101712330239699	0.406950315915052	0.684044479678558	0.865950740443082
**Cep76**	622.085146744854	0.0685818732286588	0.16859201799001	0.406791935029347	0.684160810657052	0.865994855324682
**Lime1**	3028.99893104466	-0.0906502089201399	0.222862915239215	-0.406753222369179	0.68418924629923	0.865994855324682
**Gm21596**	4558.56622368829	0.0381505202816332	0.0938373478364249	0.40656008680186	0.684331117015255	0.866104852273846
**Ociad2**	18.6251546315608	-0.234292567613974	0.576482807095624	-0.406417268182485	0.684436033804581	0.866105874922437
**A530041M06Rik**	512.445227143323	-0.110624563456106	0.272199854841561	-0.406409340374177	0.684441857874861	0.866105874922437
**Gm8172**	1549.871266023	-0.0403852010094216	0.0994433526245748	-0.406112625364578	0.68465984952625	0.866242592659798
**Gm5853**	26.1940077432773	0.155510951138747	0.382878865209392	0.406162275511602	0.684623370546967	0.866242592659798
**Gm37303**	23.9484067834001	-0.26369100147865	0.649444966355755	-0.406025167857262	0.684724108135919	0.866254337366333
**Lrrc41**	4615.03907812612	0.0303631720907603	0.074865580252539	0.405569181302519	0.685059177072179	0.866403879018387
**Hmg20a**	9136.55348607046	-0.0423772990019071	0.104485187191711	-0.405581883335795	0.685049842499757	0.866403879018387
**Ufl1**	3969.33372623969	0.0295645105728613	0.0728629323747847	0.405755157104994	0.684922510480899	0.866403879018387
**Fam124b**	91.332034716442	-0.17516504713487	0.431903762104618	-0.405564995038039	0.685062253518442	0.866403879018387
**Gtf2a2**	3097.27085337464	0.031603523899833	0.0779690833853054	0.405334044311584	0.685231985119377	0.866548987923436
**Ppp2r3c**	1517.52026986241	0.0429348430039288	0.106006091630427	0.4050224128026	0.68546103627701	0.86665808190601
**Bckdhb**	555.73297470932	-0.0622205667090761	0.153687414160069	-0.404851412518868	0.685586734868534	0.86665808190601
**Ctnnd1**	22.1389478787795	0.409372191313095	1.01168402355125	0.404644317576651	0.685738977510211	0.86665808190601
**Tmed8**	803.556537934392	-0.0673396219227663	0.166575075249553	-0.404259891954915	0.686021615875241	0.86665808190601
**Begain**	33.6311207410046	0.219619744455726	0.542831315241662	0.404581936025473	0.685784838842742	0.86665808190601
**Fam172a**	6233.79888153986	0.0332335294966801	0.0820716241905241	0.404933249761581	0.685526577004329	0.86665808190601
**Trbj1-4**	482.560629588224	-0.0581867569612022	0.143905537140624	-0.40433994492055	0.685962755511583	0.86665808190601
**Traj26**	110.468374970363	0.145620261507171	0.360054644859085	0.404439336046234	0.685889679073731	0.86665808190601
**Gm42991**	27.927904707728	0.346423910865058	0.856423755733938	0.404500585773897	0.685844647221097	0.86665808190601
**Gm48771**	71.1937446590995	-0.189962049580867	0.46909954561908	-0.404950402009387	0.685513968784879	0.86665808190601
**Gm48398**	27.3588568094908	-0.223927344983082	0.553487022758855	-0.404575601189195	0.685789496118271	0.86665808190601
**H2bc9**	17.9615381395943	-0.349416161736752	0.863135297028022	-0.404822005240516	0.685608352396675	0.86665808190601
**Fam220a.1**	2981.11443103713	0.106166864871153	0.26263062476382	0.404244040338506	0.686033271283471	0.86665808190601
**Gm7551**	73.1479685086854	-0.112199686820248	0.277615013973927	-0.404155687454228	0.68609823693722	0.866670668544154
**Slc25a32**	2584.85784400008	0.0382812156005733	0.0948162104295297	0.403741252968816	0.686403000527815	0.866744891534176
**Pggt1b**	2658.96663051715	-0.0453524809928591	0.11235596866435	-0.403649948747664	0.686470149968821	0.866744891534176
**3110082I17Rik**	522.782559181229	0.0683364480901507	0.169305943944217	0.403626987323411	0.686487037275276	0.866744891534176
**Gm15713**	20.8746227474355	-0.288094035629389	0.713541310875919	-0.403752426437279	0.686394783200822	0.866744891534176
**Thoc2l**	2936.75245126725	-0.0724605708578705	0.179381738964581	-0.403946194724859	0.686252285685316	0.866744891534176
**Gm7902**	48.5663740302354	0.21615499885705	0.535477242152718	0.403667946723686	0.686456913210475	0.866744891534176
**Pbx2**	141.733652477581	-0.120235585510747	0.297961060283099	-0.403527848224558	0.68655995233395	0.866767500114258
**Pdcd10**	5968.48492454206	-0.0391073688045964	0.0970045109110364	-0.403150002379395	0.686837878028856	0.867001619728406
**Gzmk**	1100.06188994581	0.264968447242723	0.657299446726073	0.403116796404588	0.686862304812052	0.867001619728406
**Rell1**	1675.3425374414	-0.0458527392557401	0.113764020272222	-0.403051326298251	0.686910466503104	0.867001619728406
**Kcnmb1**	23.4662480433313	-0.355922905339534	0.883548762491428	-0.40283334712156	0.68707082740642	0.867015044668023

Bend6	200.940744008296	-0.10378263822268	0.257616145786019	-0.402857662147014	0.687052938855626	0.867015044668023
Zfp408	3040.46399240163	0.0386626177792671	0.0959874631450203	0.402788202880772	0.687104040464075	0.867015044668023
Gm12470	50.6206167693645	-0.124591279312293	0.309360858052698	-0.402737696347706	0.687141199328189	0.867015044668023
Ift27	2021.40192626789	0.0376249123447317	0.0935475476201691	0.402200948094332	0.687536144536565	0.867193496772581
Srsf7	31519.9475582801	0.0405353609549583	0.100748284961425	0.402342937852279	0.687431658627956	0.867193496772581
Gm6851	46.205247306252	-0.137915570931558	0.342845632496424	-0.402267253420522	0.687487351737591	0.867193496772581
Gm16209	60.5275721056739	-0.133167465643963	0.331121080588286	-0.402171512026269	0.687557806383186	0.867193496772581
Zfp967	55.9084841748295	0.125610235393765	0.312272450410503	0.402245651925562	0.687503247714362	0.867193496772581
Itprip	9826.98760430514	-0.0437286107707118	0.108831925939765	-0.401799475596107	0.687831608119375	0.867469397241636
Dhx34	3986.09272896064	0.0464577063287434	0.115655388933507	0.401690805393018	0.687911592126514	0.867478833081221
Fam173a	6950.16740724485	0.0303999835935312	0.075689685182233	0.401639715112293	0.687949197066887	0.867478833081221
Armc5	3179.86723164983	0.0542723874699035	0.135157916259308	0.401547974191752	0.688016724799187	0.867494561307577
Rtraf	3037.65514249514	0.0357916573331859	0.0892365866742867	0.401087252068766	0.688355886103381	0.867505694419817
Cntln	26.1070409670874	-0.23223300547505	0.578881214776169	-0.401175577212063	0.688290860554212	0.867505694419817
Wdr34	216.611399299176	0.111317422889212	0.27745040486794	0.401215571994556	0.688261416891152	0.867505694419817
Eid2	28.0702778368551	0.267858723230013	0.667537808489706	0.401263748395075	0.688225950649659	0.867505694419817
Olfr173	25.0749506511562	0.230813004191771	0.575276785196708	0.401220786465159	0.688257578097353	0.867505694419817
Gm10282	1142.61338414118	0.130480104040991	0.325285873718638	0.401124409582729	0.688328530217424	0.867505694419817
Zbtb17	3685.28476181992	0.0442189157346935	0.11036016914374	0.400678216405233	0.688657050868481	0.867785469410058
Ogfod3	755.747387380924	0.0617453232148499	0.15411820443127	0.400636144462645	0.68868803039746	0.867785469410058
Slc9a6	1197.6248637895	0.0551224035383974	0.137655351654858	0.400437780847092	0.688834101791934	0.867900123295659
Gm45746	95.0011327970009	0.150101942703879	0.374955369643086	0.400319490948372	0.68892121386634	0.867940478683291
Ass1	6430.3401024974	-0.0517521171158465	0.129395398551209	-0.399953303558668	0.689190910910557	0.868210839719991
Nck1	4943.50758928375	0.0335120535727218	0.0838068594074722	0.399872442538205	0.689250470384677	0.868216457098998
1810062G17Rik	68.2239787787833	0.159928820097688	0.40003842752462	0.399783643504712	0.689315878947568	0.868229440813495
Gm4285	127.216460770222	0.121586548429948	0.304269381847691	0.399601654598329	0.689449937581462	0.868328883899856
Fkbpl	277.206034560464	-0.10519766044524	0.263370945769589	-0.39942773542406	0.689578060912803	0.868351310862066
Gm12166	312.087153650795	0.0578361024407119	0.144824485774528	0.399353066102059	0.689633071278028	0.868351310862066
Gm49893	30.7571130719778	0.209603631431564	0.524685031347949	0.399484679204741	0.689536110406849	0.868351310862066
Trim11	8077.3025339104	-0.0425316306603404	0.106582645698698	-0.399048366472103	0.689857566519243	0.868466996107109
Nagk	821.79851087462	-0.0598608488729371	0.1500538362251	-0.398929146890576	0.689945412024421	0.868466996107109
Gm3695	22.8866967155763	0.254607363336441	0.638080430076043	0.399020799472096	0.689877878558023	0.868466996107109
Btbd18	39.8828400851787	-0.244995863584366	0.614131284882874	-0.398930765481344	0.689944219357431	0.868466996107109
Gm2225	40.5195520053319	0.136777945840594	0.343003923480387	0.39876496004109	0.690066397955383	0.86854990275445
Rpl6l	760.262182506111	-0.0519765787591208	0.130462994720585	-0.398400932543669	0.690334670619659	0.868818163334982
Tbc1d5	2109.08492948842	-0.0711074208974144	0.178591879266122	-0.398155958656195	0.690515227780512	0.86897599585796
Cwf19l1	1525.95109252562	-0.0348945538184925	0.0876601096227036	-0.39806650902767	0.690581160711413	0.868989566309674
Traf3ip1	820.676937600844	0.0517277662382408	0.129974650057072	0.397983500748239	0.690642347852217	0.868997163228251
Dhrs7	5248.42044455107	0.0335274589010654	0.084347234642545	0.397493279336915	0.6910037415264	0.869219423573339
Scaf8	4806.70659060753	-0.0480867923266169	0.120940530477378	-0.397606924137078	0.690919955738186	0.869219423573339
Gm43672	165.324568813982	0.110815791051538	0.278820738154506	0.397444579571161	0.69103964708404	0.869219423573339
Gm20337	26.9958704049499	-0.33913266241645	0.853122138832742	-0.397519472276804	0.690984430178591	0.869219423573339
Adgrd1	191.649230704354	-0.135801623261761	0.341762847982393	-0.397356307344317	0.691104730555165	0.869231899693306
Sdhaf2	6142.68735315931	0.0402539032133558	0.101389618988274	0.397021939869518	0.691351281749192	0.869472595300664
Lnpk	895.595067943335	-0.04723768525387	0.119133812989514	-0.396509471731824	0.69172922182907	0.869484769963957
Rad51d	2797.46500712591	0.0335630608613273	0.0846680531694965	0.396407613083268	0.691804350707117	0.869484769963957
Mxd3	142.60912204636	0.194415665558423	0.490533140967224	0.396335434492922	0.691857590011302	0.869484769963957
Srp9	5021.24962782635	-0.0356257464734502	0.0898377042413782	-0.39655673276924	0.691694364074701	0.869484769963957
Gas2	116.000618631485	0.129617045417256	0.326954278669585	0.39643783205616	0.691782061487676	0.869484769963957
Gimap1os	3210.11885050743	0.0516028512163768	0.130027893919214	0.396859855689409	0.691470808886836	0.869484769963957
Cisd3	1093.99477740558	0.0454805709447021	0.114705876264784	0.396497306203528	0.691738194717026	0.869484769963957
Gm44503	29.2308800549445	-1.21545723558275	3.06603930997902	-0.396425848692419	0.691790900267855	0.869484769963957
Gm42977	17.4580425561396	0.183288191938462	0.462085879122968	0.396653955940701	0.691622658404986	0.869484769963957
Pak1	516.071925962955	0.0818667063108454	0.206749831419253	0.395969881807708	0.69212724696054	0.869740900423709
Gm4342	22.4183024258627	-0.198789261552436	0.502107829388528	-0.39590950373055	0.692171789764124	0.869740900423709
Gm44292	103.638548710631	-0.13204233774455	0.333582827130399	-0.395830741289732	0.692229896890538	0.869744556678432

**Gm50055**	27.5845176062201	-0.234313378387792	0.592385835451478	-0.39554183163278	0.69244305599567	0.869943010151792
**Echs1**	2304.97197441112	0.0353201756480367	0.089367640179566	0.395223322189866	0.692678082214023	0.870005884673166
**Wdr13**	2299.11772922315	0.0548435716251182	0.138752485453809	0.395261904287658	0.69264961113882	0.870005884673166
**Gm11793**	43.1881499943578	-0.129817026148103	0.328327115994623	-0.395389292641394	0.692555609914544	0.870005884673166
**Gm47761**	31.2667593621676	-0.179970334760774	0.455419670072343	-0.39517470717105	0.692713957549631	0.870005884673166
**Lrrfip1**	25697.4002943613	0.027473321062574	0.0696199813518382	0.394618334120662	0.693124580759071	0.870309512024266
**Bloc1s5**	1527.28095517642	0.0582718728888585	0.147722381575958	0.394468815538933	0.693234946195385	0.870309512024266
**Dcaf1**	3952.75656090017	0.0619732490403387	0.157024888714081	0.394671504293711	0.693085335371498	0.870309512024266
**Gm11973**	42.8313225188578	-0.189538627275668	0.480576868226624	-0.394398148989327	0.693287110168724	0.870309512024266
**Eid1**	1308.39165423541	0.0342352967979089	0.0867893035760143	0.394464471856535	0.693238152532334	0.870309512024266
**Dnajb6-ps**	143.932138511675	0.0697188385416042	0.176665064675286	0.394638513674161	0.693109685950419	0.870309512024266
**Traj17**	176.19088060352	0.094567550986098	0.239849072471994	0.394279410845503	0.693374762456005	0.870350205689418
**Olfr756-ps1**	29.8822556693428	0.273067362946802	0.693023673155903	0.394023138781542	0.693563956024206	0.87051834117201
**Vwa7**	48.2414848807444	-0.217880617540015	0.553252844378488	-0.393817437639706	0.693715829290208	0.870590745102546
**Adam11**	61.5675167411307	-0.248396432174652	0.631223391308958	-0.393515886126394	0.693938493039353	0.870590745102546
**Glt8d1**	659.611241406208	0.0644153244681202	0.163700057809134	0.393496039831734	0.693953148346004	0.870590745102546
**Agk**	5619.23277097877	-0.0306565518092672	0.0779055712565666	-0.393509107433484	0.693943498687488	0.870590745102546
**Ric8b**	2248.65189998458	-0.060074509620783	0.152650662427515	-0.39354241026834	0.693918906718886	0.870590745102546
**Med18**	438.536559205082	-0.106154530669945	0.269630269501482	-0.393704055802837	0.69379954662764	0.870590745102546
**Acp2**	1519.81519565116	0.0358155361443757	0.0910512235864334	0.393355901586283	0.694056635351322	0.870615120903743
**Gm7984**	79.3535770856283	-0.113209676162994	0.287830903497202	-0.393320087549577	0.694083083632216	0.870615120903743
**Plaa**	6254.08908714629	-0.0280753867914012	0.0714304569642469	-0.393045039673396	0.694286215945896	0.87066199002463
**D230025D16Rik**	3444.39923751206	-0.0328225793666045	0.0834937596747094	-0.393114161998224	0.694235164665808	0.87066199002463
**Nlrc3**	10884.171634455	-0.0509784599106221	0.129693226941628	-0.393069562017811	0.694268104459486	0.87066199002463
**Ap3m1**	5553.62447529341	-0.031352180610732	0.0797869468716647	-0.392948744625624	0.694357338340524	0.870681886060133
**Dcaf7**	16503.9164812271	0.0450247849155543	0.114641752198393	0.392743342213026	0.694509055149189	0.870802831382651
**Sema4d**	20781.1171562173	-0.0279050698879785	0.0711484110633969	-0.392209319518234	0.694903558699524	0.870914013810947
**Paics**	10978.1333553755	0.0228374035979619	0.058243885275836	0.392099591052464	0.694984629666741	0.870914013810947
**Ctps2**	10928.8248550639	0.0215387282794897	0.0549101843851658	0.39225379627952	0.69487069881218	0.870914013810947
**A430033K04Rik**	872.262279490653	0.0634778760665483	0.161887560096474	0.392110894924352	0.694976277836557	0.870914013810947
**mt-Tl1**	186.330694478762	-0.158675876977698	0.404280010048149	-0.392490039165677	0.69469616975599	0.870914013810947
**Gm15387**	5982.55977969411	0.0383085241145483	0.0976507998158694	0.392301181216979	0.694835690965183	0.870914013810947
**Gm45854**	72.6844041712259	-0.137711779145411	0.35121367858072	-0.392102550509747	0.69498244307746	0.870914013810947
**Acbd5**	3090.17068496502	-0.0335163569462807	0.0855443390760104	-0.391800992424523	0.695205261724879	0.870982691341252
**Ppox**	7320.98563496286	-0.0338554321850962	0.0864069998400148	-0.391813536493346	0.695195992496559	0.870982691341252
**Gm5597**	73.6364931560642	0.113899357690197	0.290674518418979	0.391845003510153	0.695172740675114	0.870982691341252
**Bnip2**	7662.6881080625	0.0374781045186652	0.09575749943187	0.391385580670163	0.695512248969347	0.871036838601076
**Impact**	1374.46690752494	-0.0404716294497423	0.103410545854425	-0.391368492597611	0.695524878040156	0.871036838601076
**Glce**	989.993814215159	-0.0345959027303377	0.0883705751691179	-0.391486676013257	0.695437535410353	0.871036838601076
**Gm12977**	53.0188830800614	-0.174085465496987	0.444781468929431	-0.391395500167763	0.695504917927469	0.871036838601076
**Gm37802**	57.8924493603375	0.21881405211657	0.558760180800913	0.391606380760575	0.695349072570355	0.871036838601076
**Mta3**	9242.12405671844	-0.0335360391710422	0.0857532991082752	-0.391075789733739	0.695741215443277	0.871100066089767
**3110045C21Rik**	23.2020702764583	-0.221514387783412	0.566364145694412	-0.391116544836742	0.695711091762471	0.871100066089767
**Gm7105**	17.452615985117	0.202080523132027	0.516722264135883	0.39108150965774	0.695736987596146	0.871100066089767
**Gm10501**	61.9985946094342	0.151725189670683	0.388046864428921	0.390997076845277	0.695799396557842	0.871103693910846
**Ssrp1**	14653.1014954997	0.0209039595343217	0.0534914791898255	0.390790455805862	0.695952130285868	0.871157809712058
**Gm15789**	19.8311538935698	-0.269343799672095	0.68923073545951	-0.390789014208026	0.695953195954521	0.871157809712058
**Nckap1l**	23035.5653117054	0.0302571529099369	0.0774686445434329	0.390572896792755	0.696112962664792	0.871288581516334
**Stat5a**	6845.83753507091	-0.0345308828192627	0.088444526785725	-0.390424191006424	0.696222902565103	0.871356971860073
**Dram1**	21.5984673132061	-0.281748589643711	0.72319417203017	-0.389589131854837	0.696840389918094	0.871682086346722
**Btd**	1821.37334858826	-0.0401255183268083	0.102971729823398	-0.389675092334816	0.696776816863465	0.871682086346722
**Hira**	499.016777404402	-0.0823063232921075	0.211070190503565	-0.389947642989014	0.69657526299613	0.871682086346722
**Mark2**	14838.4675228613	-0.0371529794828116	0.095370562262416	-0.389564437929847	0.696858652985995	0.871682086346722
**Mettl21a**	1571.07282618806	0.0319481282649615	0.0820288244457913	0.389474413181118	0.696925234739861	0.871682086346722
**Tmem218**	356.120736779959	0.0583811300341728	0.149865205861211	0.389557600769849	0.696863709625808	0.871682086346722
**Maf**	40.3545600506354	-0.184794748123941	0.474280103870433	-0.389632090015786	0.696808619453429	0.871682086346722

**Dnaaf4**	93.0592867537895	-0.14561904355944	0.373839541522818	-0.389522849739939	0.696889411066876	0.871682086346722
**Gpaa1**	5649.00574884855	0.0277161358752951	0.0712767388493886	0.388852468879934	0.697385284695387	0.872170982871517
**Eri2**	976.629232286517	0.0572170915356992	0.147164691901847	0.388796326049871	0.697426818822181	0.872170982871517
**Cks2**	539.400848942599	-0.0935372947645311	0.240771376373325	-0.388490094518121	0.697653383017349	0.872385077073594
**Zfyve16**	1258.51855913606	0.0455512274692591	0.11728044730489	0.388395751517226	0.697723187747977	0.872403131990547
**Mdm1**	522.177730531478	0.0651906665339732	0.167943980633876	0.388169116201261	0.697890886497025	0.872405132767443
**Mtmr10**	821.203941883164	-0.0592078808079913	0.152528872299139	-0.38817490692433	0.697886601469146	0.872405132767443
**Dok4**	51.5193550830157	0.281512949180497	0.725112305671969	0.388233583926859	0.697843182116749	0.872405132767443
**Ehd3**	22158.8289921473	0.0397720856844764	0.102484258602717	0.388079947366881	0.697956870999876	0.872418405231242
**Trav9n-1**	51.9105142395969	0.127577523783158	0.328924367658143	0.387862792566805	0.698117574044303	0.87248085455394
**Ppp1r2-ps2**	20.0088079551522	-0.277593355027121	0.715648491231082	-0.387890645237854	0.69809696122161	0.87248085455394
**Ovgp1**	375.71521631305	0.0693384403343506	0.178836019472936	0.38772077648957	0.698222678816566	0.872543005005363
**Hadha**	7699.42464483824	0.0213350625388984	0.0550558324949915	0.387516845573796	0.698373616304343	0.872575130626142
**Nop10**	4783.53918530316	0.0341090888995706	0.0880597193467922	0.387340422528988	0.698504203732179	0.872575130626142
**Slc66a2**	1636.93997968398	0.054931477207787	0.141778625433028	0.38744540680947	0.698426493833837	0.872575130626142
**Poglut3**	43.8886466592281	-0.214470405164525	0.554040402010449	-0.387102464705236	0.698680353021275	0.872575130626142
**Enpp1**	1096.69911305793	0.0576743845407341	0.148965896016914	0.387165022886753	0.698634042489313	0.872575130626142
**Prox2**	459.786138335897	-0.0677650301288613	0.175063837604518	-0.387087539357772	0.698691402113984	0.872575130626142
**Cyp20a1**	799.806904350751	0.0632904122185361	0.163450657462981	0.387214179501667	0.698597653647741	0.872575130626142
**Tmsb4x**	182534.390981177	0.0362280905754427	0.0935364097823858	0.387315385096863	0.698522737033492	0.872575130626142
**Arhgef4**	1520.94317982148	0.0522855138313656	0.135173120501978	0.386804074931455	0.698901260248101	0.872768042299043
**Gm45205**	32.3905394612043	-0.248666012410092	0.643537409855038	-0.386404906073923	0.69919681723132	0.873067933205001
**Gbp5**	1639.47932391797	-0.0600030189376841	0.155335573241844	-0.386279959480143	0.699289340929234	0.873114274565922
**Ywhaz**	38221.8793122885	0.0314455707175727	0.0815246642233431	0.385718494116396	0.699705164483416	0.873194617750791
**Mrps31**	938.304977356685	-0.0595093307357624	0.154238553537929	-0.385826561328121	0.69962512245998	0.873194617750791
**Mpi**	1194.95957835891	-0.0413982738988072	0.10727187452056	-0.385919180435994	0.699556525022487	0.873194617750791
**Lrrc3b**	19.5520271263101	-0.368317282611364	0.955080934737161	-0.385639864869384	0.699763404818769	0.873194617750791
**mt-Tw**	84.3925422095796	-0.172409660326882	0.447126884945188	-0.385594483650916	0.699797019290283	0.873194617750791
**Chchd2**	15497.0953326969	0.0290898895342924	0.0753667248281702	0.385977891444996	0.699513042556887	0.873194617750791
**Gm6419**	37.5277811977857	0.123659700905942	0.320481031628772	0.385856536586486	0.699602921309363	0.873194617750791
**Gm8233**	31.7900741301421	-0.21218761257878	0.550231461825411	-0.385633369409377	0.699768216056231	0.873194617750791
**Bst2**	4517.00476690586	0.0342276041515947	0.0887853765255581	0.385509477923336	0.699859985744994	0.873204037958973
**Recql**	4347.47272952197	0.0316425573159569	0.0821909937410726	0.384988134048371	0.70024620690903	0.87354757994344
**1700001G11Rik**	25.4062651588384	-0.251077224786499	0.652064870666697	-0.385049457624956	0.700200773246782	0.87354757994344
**Eif1**	29825.4159908125	0.0244157282058798	0.0634366303656274	0.384883750368765	0.700323545583326	0.873574897700797
**Tkt**	15639.9896868685	0.0210584154767843	0.0548155530294458	0.384168622096582	0.700853473167462	0.874028347977816
**Tmem268**	3488.28428178383	-0.0372341775706818	0.0968868247008945	-0.384305891803451	0.700751741649549	0.874028347977816
**Gas2l3**	20.335420334208	-0.467577600553451	1.21691192043653	-0.384232903549603	0.700805833075287	0.874028347977816
**Chek1**	83.9033775397315	-0.12049252885853	0.313857994733376	-0.383907789128931	0.70104679304336	0.874061902040214
**Gm10356**	29.1302373237679	-0.171827814274517	0.447561805850868	-0.383919744777712	0.701037931529406	0.874061902040214
**4930403P22Rik**	63.5081096370697	0.176918304745331	0.460825960234809	0.383915664506365	0.701040955817545	0.874061902040214
**Gm39321**	26.416536461673	-0.369863832426582	0.964019287997565	-0.383668498163407	0.70122416380072	0.874213873645696
**Ncaph2**	17732.8870325301	0.0201801401959558	0.0526227377198828	0.38348708315742	0.70135864574448	0.874297501662582
**Rpl14**	24458.2202103337	0.0200066435250816	0.0522088886591745	0.3832037807908	0.701568674939499	0.874297501662582
**Epb41l1**	27.6479256498623	-0.386988699189173	1.00943174808297	-0.38337282329797	0.70144335075073	0.874297501662582
**Rnf144b**	36.9163477192732	0.298230937170767	0.778236709370296	0.383213659263231	0.701561351046088	0.874297501662582
**C920006O11Rik**	374.501316073207	-0.111109452123024	0.28985193370702	-0.383331760813205	0.701473792776568	0.874297501662582
**Alg14**	822.802959756857	-0.0434162082429136	0.113329736520584	-0.383096348547745	0.701648326928882	0.874327614663377
**Trav6d-4**	37.7164999841578	0.129787628673391	0.338895766999697	0.382972115061876	0.701740439716732	0.874373249139376
**Gm8129**	49.1794051716729	-0.306344646553911	0.801355430015235	-0.382283110689207	0.702251380808263	0.874940697983379
**Wdr7**	1980.97034046427	0.0416061982354112	0.108983077031349	0.381767512615221	0.70263381802636	0.875347965736983
**Npnt**	27.3144439933372	0.480386465162038	1.25864620321755	0.381669180691125	0.702706762822314	0.875369630942462
**Gm10847**	104.108508068179	-0.116595383071112	0.305648032182843	-0.381469438027867	0.702854944775395	0.875485009077146
**Unc50**	5414.9792183053	-0.0511915093957694	0.134315154444103	-0.381129810762145	0.703106928041368	0.875660438282314
**Dctd**	695.175827622203	0.0505709462370486	0.132662586677845	0.381199760259868	0.703055026999168	0.875660438282314
**A530032D15Rik**	54.9303442014956	-0.123662195875443	0.324529472454544	-0.381050740754413	0.703165597974421	0.875664295209274

**Socs1**	3061.07053376096	-0.165983286899938	0.435755461920302	-0.380909251644207	0.703270587283044	0.875725829288677
**Nup62**	5100.42768409642	-0.0335113500478658	0.0880320572337302	-0.380672122189434	0.703446557381666	0.875875733280356
**Iqcg**	99.3086930813816	0.22810693618274	0.599427024632651	0.380541628603635	0.703543401428933	0.875927099898522
**B9d1**	17.7312891600311	0.256304204021593	0.675830857932381	0.379243121282925	0.704507332845921	0.876339220706826
**Rffl**	2466.70554135818	-0.0564034797453255	0.148620542591613	-0.379513348301478	0.704306693873203	0.876339220706826
**Fbf1**	1119.11602647682	-0.0738673867166334	0.195228762505756	-0.378363237919185	0.705160773719725	0.876339220706826
**Habp4**	3209.07300377595	0.049549341169972	0.130832088302543	0.37872468301042	0.704892322017903	0.876339220706826
**Nkiras1**	907.361281906017	0.0574534238849726	0.151611097873037	0.378952627419698	0.704723042540341	0.876339220706826
**Pde1b**	396.885527923552	-0.123088114224782	0.324791696140138	-0.378975557834685	0.704706014422808	0.876339220706826
**Ypel1**	364.068261057696	-0.0854278349594736	0.225073291073126	-0.379555630755486	0.704275301730348	0.876339220706826
**Prrc1**	2533.35573440217	0.0395456956651469	0.104217039775993	0.379455180747289	0.704349880557111	0.876339220706826
**Ptdss2**	1554.33303624174	-0.0274109147778655	0.0724481801506213	-0.378352012719679	0.705169111463156	0.876339220706826
**Gabpb1**	3872.79986223266	0.0299562686664877	0.0791742274373747	0.378358837668262	0.705164042090665	0.876339220706826
**Ing3**	3850.3511666002	-0.0357893309657028	0.0944968450853341	-0.37873572322318	0.704884122833581	0.876339220706826
**Mlf2**	9972.93379837783	0.0222532737398218	0.058824712408616	0.378298045645224	0.705209197090979	0.876339220706826
**Ldah**	4330.4900064867	-0.0295275379163781	0.0779979742288556	-0.378568010365765	0.705008681069294	0.876339220706826
**Ywhag**	3646.20738206326	-0.0407303084363996	0.107564100679188	-0.378660800203951	0.704939766242722	0.876339220706826
**Blcap**	2044.60173063004	0.0370243177261134	0.0976780178915911	0.379044523274471	0.704654801605016	0.876339220706826
**Macrod2**	91.1067002828282	0.12959042708946	0.341254594788063	0.379747054160376	0.704133187833117	0.876339220706826
**Marchf2**	8617.98598304536	-0.0266847351398624	0.0702852769842279	-0.379663227987997	0.704195419620993	0.876339220706826
**Gm23119**	32.6918497721899	-0.16625116200679	0.439233402452057	-0.378503003366044	0.705056963079624	0.876339220706826
**Gm17484**	92.0183751183425	-0.108834476722273	0.287295687558765	-0.378823913602991	0.704818628118324	0.876339220706826
**Gm38346**	47.3088422226658	0.212856488565204	0.562435732446494	0.378454774982586	0.70509278404076	0.876339220706826
**Gm43692**	46.8021033026778	-0.193949298212823	0.511333811445406	-0.37930075006106	0.704464542731264	0.876339220706826
**Gm42067**	73.1788646535827	-0.145152074887574	0.382707059228339	-0.379277234081459	0.704482003537938	0.876339220706826
**Gm18029**	30.5361919939724	-0.223676963825962	0.591096087860876	-0.378410496058989	0.705125672171207	0.876339220706826
**Rpl19-ps6**	55.9177295958684	0.142722446415927	0.375777804222063	0.379805419086398	0.704089859412721	0.876339220706826
**Gm11353**	301.250992269055	0.124986597560065	0.330564749821966	0.378100198606716	0.705356160782133	0.876452726555009
**Rpl5**	33892.117217201	-0.0249967824822478	0.0661687736537707	-0.377773096008154	0.70559916151703	0.876685537614244
**Gm8930**	19.1339036750921	0.174950229131739	0.463311672183507	0.377608076021978	0.705721764352604	0.876768733020893
**Cdkl3**	174.868417312514	-0.0955730307302097	0.253170571756922	-0.377504502466317	0.705798719004675	0.876795207770156
**Tmem254b**	2031.43097380653	0.0662344743249169	0.175546678334468	0.377304059258362	0.705947655886582	0.87691109292904
**Irf3**	11852.6987552562	0.028644037312269	0.0760358529984934	0.376717511314518	0.706383547874875	0.876913206835774
**Fas**	3215.10029762916	0.0664633680226927	0.176250252843654	0.377096582560085	0.706101830781712	0.876913206835774
**Dyrk4**	76.4437787660474	-0.117369073760898	0.311264747283494	-0.377071527647171	0.706120449778415	0.876913206835774
**Tsen34**	2709.28504673267	-0.0295355007850567	0.0784024753996464	-0.376716431904775	0.706384350125007	0.876913206835774
**Akap12**	1030.08334893047	0.0646834322412706	0.171638157072321	0.376859279688117	0.706278184145522	0.876913206835774
**Gm15536**	29.5260245301724	-0.141118689231572	0.374368575788496	-0.37695121427953	0.706209860409937	0.876913206835774
**Gm42732**	61.1030588974231	0.127908149161995	0.339205082734783	0.377082053519828	0.706112627690754	0.876913206835774
**Gm50021**	44.5501710929293	0.228999323185761	0.607904693704776	0.376702673226888	0.706394576021228	0.876913206835774
**Btla**	4661.29440159917	0.0366003718866085	0.0972104604246384	0.376506517166253	0.706540371339079	0.876968477248983
**Atg4a-ps**	52.3815701140304	0.133515118357972	0.354628411523874	0.376493010766519	0.706550410527121	0.876968477248983
**Chmp2b**	2958.81443625676	0.0281841371460751	0.0749160366883886	0.376209666073318	0.706761029910265	0.877060288149287
**Smyd3**	4065.51356689588	0.0238586054169095	0.0634252539783184	0.376168858938514	0.706791365039008	0.877060288149287
**Gm44668**	18.2198528664663	-0.310395384403125	0.825032378885731	-0.376222063941705	0.706751813699138	0.877060288149287
**Nom1**	4999.62807818019	-0.0275057333044814	0.0731911555461583	-0.375806791124302	0.707060538698799	0.877099248151418
**Ebi3**	39.8856295626464	-0.192240325005696	0.512934007963922	-0.374785687868093	0.707819859174055	0.877099248151418
**Shpk**	1683.11860058324	0.0509904682179964	0.136190677117274	0.374404983493022	0.70810303584021	0.877099248151418
**Kctd6**	103.395464644441	0.160748399383844	0.428056412665513	0.375530875434997	0.707265688500852	0.877099248151418
**Fyttd1**	3645.52765495896	0.0285131874616639	0.0759913069066715	0.375216437541761	0.707499506383177	0.877099248151418
**Ivns1abp**	20157.0574070345	0.0451292577031195	0.120100240571235	0.375763258162269	0.707092905066636	0.877099248151418
**Arhgdia**	61358.4299218318	0.0241714534760426	0.0644323449939647	0.375144711531246	0.707552846134995	0.877099248151418
**Coq3**	675.263621647655	-0.0536351357289726	0.142998032567894	-0.375076039619687	0.707603916021044	0.877099248151418
**Cct2**	9729.91056873279	-0.017945696287271	0.0478277998140364	-0.375214757046055	0.707500756083883	0.877099248151418
**Slc26a2**	2494.20218367682	-0.0509888707294005	0.135596526684393	-0.376033752310482	0.706891803665031	0.877099248151418
**Gpat2**	41.3973614477027	-0.443340115545878	1.18313855432493	-0.374715297650693	0.707872213992574	0.877099248151418

**Zfp874a**	1822.83290835086	0.0396248461803679	0.105600309764612	0.375234185095609	0.70748630846207	0.877099248151418
**Zscan25**	66.7199357879105	-0.150249526472909	0.401113289280988	-0.374581272892348	0.707971902711916	0.877099248151418
**Rpl37rt**	238.526429775686	-0.0819676373729404	0.218310993006516	-0.375462711447123	0.707316373303473	0.877099248151418
**Sdhaf1**	2126.84525610434	-0.044977195382215	0.120061201776669	-0.374618900332841	0.70794391460633	0.877099248151418
**Gm4149**	324.519977682291	-0.0717564619882812	0.191244471677674	-0.375208032727975	0.707505756631209	0.877099248151418
**Gm17029**	244.823945713847	-0.0971529639942427	0.259480476347125	-0.374413386941198	0.708096784726465	0.877099248151418
**Gm10177**	173.270047460659	0.0893334989076378	0.238285106818422	0.374901730537913	0.70773355200116	0.877099248151418
**Gm6548**	257.847232913134	0.0815642082104094	0.217324736440474	0.375310282420382	0.707429719885776	0.877099248151418
**Igkv4-59**	29.4209235977768	-0.381442650349934	1.01639925615996	-0.375288202975529	0.707446138750554	0.877099248151418
**Mir7052**	27.7938709235315	-0.142332576398682	0.380031763658672	-0.374528105304689	0.708011450588202	0.877099248151418
**Gm43423**	17.9829223550487	0.241087212632577	0.643285795482206	0.374774655877266	0.707828064454205	0.877099248151418
**Gm40773**	66.1791244323768	0.135893417584058	0.362728885430879	0.374641841447595	0.707926850701655	0.877099248151418
**Krt222**	73.873769963007	-0.13531326614045	0.361568984994108	-0.374239140402639	0.708226406367733	0.877151227569911
**AI467606**	33198.5815907986	-0.0295553840172162	0.0789988813639483	-0.374124082606365	0.708312002259284	0.877151227569911
**Teddm3**	32.2098018157647	-0.160232374526408	0.428249389532236	-0.37415669103796	0.708287743226703	0.877151227569911
**Trmu**	2523.45059639809	0.0418317851738269	0.111859131337443	0.373968442930544	0.708427794424897	0.8771567465624
**Mrpl50**	1127.69474303083	0.0579613293730529	0.154967348297541	0.374022850683136	0.708387315602647	0.8771567465624
**Glrp1**	19.9297276174275	0.328338017167657	0.87825127317294	0.373854302518048	0.708512716423238	0.877192965559909
**Gm4204**	939.794573151067	0.0451131224537431	0.120709923386044	0.37373167995034	0.708603953296656	0.877236996550551
**Stard8**	22.6897810171887	0.317062013853454	0.848648540932575	0.373608152916914	0.708695867362151	0.87728185890686
**Chkb**	6126.97761977911	0.0394755865342407	0.105755712748983	0.373271433836752	0.708946435025283	0.877454165163254
**Gm2004**	52.5559815372616	-0.190976434581491	0.511547644175909	-0.373330689244301	0.708902338153361	0.877454165163254
**Tox**	8451.57900730384	-0.0453337051587957	0.12152909138215	-0.373027598933027	0.709127903099143	0.877507017765232
**Zfp219**	504.551991086449	-0.0654336131127761	0.175465341723692	-0.372914744701067	0.709211897648044	0.877507017765232
**Fam78b**	23.3784867681569	0.309032001575535	0.828630694894009	0.372942981088895	0.709190881687529	0.877507017765232
**Gm44664**	41.7532364889764	0.293508632464483	0.786996449584644	0.372947848264613	0.709187259137012	0.877507017765232
**Gm3839**	57.2842382237468	-0.100716579803611	0.270207535734454	-0.372737864359899	0.709343552321567	0.877601001407894
**H3c1**	55.419552462036	-0.183113288488787	0.491425224142134	-0.372616787850975	0.709433676375932	0.877643592577182
**Ddias**	189.639492501239	0.0914572735605565	0.245526684583651	0.372494230986111	0.709524906483393	0.877687545255049
**Rhoa**	21379.9333760625	0.0267374509239825	0.0720672853648274	0.371006772193916	0.710632487646825	0.877748381896294
**Rps25**	13862.257981686	0.0403565991653323	0.108467416816166	0.372061955100581	0.709846721584255	0.877748381896294
**Ntmt1**	2240.22193066643	0.0515318724649556	0.138846952862075	0.371141544000216	0.710532109586992	0.877748381896294
**Miga2**	984.105582933219	0.0613391423966338	0.165127087604206	0.371466264479017	0.710290278354626	0.877748381896294
**Dut**	1972.98812101525	0.0366536954789253	0.0985837381565541	0.371802653909493	0.710039787586576	0.877748381896294
**Tmem68**	2056.58521360472	-0.0387145207705366	0.104334005410707	-0.371063304031493	0.71059038211523	0.877748381896294
**Slc12a3**	24.5427410864414	-0.310464546316249	0.833834415508868	-0.372333571920008	0.70964450579758	0.877748381896294
**Gabarapl2**	5685.81830854463	0.039059859441058	0.105148677138106	0.371472666173015	0.710285511071783	0.877748381896294
**Ccar2**	6339.09307221299	0.0486163361262159	0.130833869654193	0.371588307024118	0.710199396342148	0.877748381896294
**Sft2d2**	6811.95409513003	0.0405332573847132	0.10890929006057	0.372174470719353	0.709762952441881	0.877748381896294
**Sv2c**	108.430994817895	-0.159181836792822	0.428856804685972	-0.371177127314983	0.710505607967003	0.877748381896294
**Cd200r4**	782.879697503234	0.0737896299541755	0.198407399878987	0.371909666671613	0.709960107615963	0.877748381896294
**Fbxo47**	22.3122443707094	-0.357625638071524	0.963742768619103	-0.371079970419853	0.710577968975272	0.877748381896294
**A530017D24Rik**	85.6847481392608	-0.162936366064617	0.438792708322583	-0.371328791418368	0.710392656018251	0.877748381896294
**Trav6n-5**	101.030218826375	0.0955613977187364	0.257202551551665	0.371541406343866	0.710234321611087	0.877748381896294
**4930506C21Rik**	33.0199459554926	-0.195101042458054	0.525480460577945	-0.371281250388405	0.710428061549748	0.877748381896294
**Cmtm4**	65.7434663375129	0.154311011552709	0.414985876053611	0.371846418052007	0.710007201131208	0.877748381896294
**9530082P21Rik**	56.2013838657968	0.193615102243122	0.520569361416292	0.371929499877521	0.709945340480154	0.877748381896294
**Wdr49**	21.4003801591716	0.445011666580604	1.1960243498324	0.372075758025298	0.709836444962982	0.877748381896294
**Slc9a8**	6168.8452131636	-0.0411112184621468	0.110860713523474	-0.37083667564021	0.710759182748083	0.877836059144188
**Hyal2**	831.231308841039	0.0711633710622691	0.192248868587118	0.37016275614658	0.711261224993023	0.878106786750393
**Parvb**	20.7785882981386	0.43735428140674	1.18164701759155	0.370122612671729	0.711291134181849	0.878106786750393
**Gpam**	848.800351832188	0.0716756498708539	0.193708128018889	0.370018804083764	0.711368479588041	0.878106786750393
**Micall1**	2098.11599657257	-0.0577637216859298	0.156034978456206	-0.370197261264354	0.711235517058457	0.878106786750393
**Trim45**	281.535593221612	-0.0854768763349602	0.230868523107131	-0.37024049525927	0.711203306163645	0.878106786750393
**Gatd3a**	5034.34108275557	0.030009394476083	0.0810890996280665	0.370079266063229	0.711323430387767	0.878106786750393
**Gm8225**	119.830984130035	0.0759067591075493	0.205086288651761	0.370121082235975	0.711292274452968	0.878106786750393

**Fam43a**	86.9472018775295	-0.235346018580003	0.636236099265222	-0.369903592159891	0.711454324837596	0.878143960243303
**Alpk1**	24.3321172567807	0.282005488849067	0.763258331393842	0.369475808189445	0.711773101815603	0.878399809313005
**Dgkd**	14687.0888296594	-0.0620119362760764	0.167820427261584	-0.369513636021302	0.711744911159006	0.878399809313005
**Lage3**	1034.17301409397	0.0435187104467873	0.117882892529172	0.369169007589613	0.712001755176323	0.878448815373029
**Dpysl2**	3077.54838344704	0.0611764586849766	0.165674535444677	0.369256859666311	0.71193627784439	0.878448815373029
**Armc8**	6387.35786786568	-0.0289809742253026	0.0784789890704867	-0.369283225593962	0.711916627378991	0.878448815373029
**Prcp**	1351.58330634243	-0.0394156163109497	0.106781703099523	-0.369123315763315	0.712035810747244	0.878448815373029
**Mlh1**	4232.07830227823	-0.0319431174395437	0.0865716660883345	-0.368978892088666	0.712143458080681	0.878512837154725
**Mrpl40**	3138.51184161591	0.0416630153212315	0.112964931166091	0.368813709627945	0.712266585172836	0.878595943523436
**4930413G21Rik**	27.5618153739854	0.160396919712956	0.435373104788539	0.368412559133301	0.712565634183555	0.878896023313524
**Zbtb8os**	2212.79018141791	0.0400178253027006	0.108880372278343	0.367539387176218	0.713216717730649	0.879561385402038
**Fam174b**	1734.71653756439	0.052475927206848	0.142757423837798	0.367588079107337	0.713180404925819	0.879561385402038
**Dlg1**	1558.17185183645	0.0464469766037968	0.126450970897751	0.367312139037305	0.713386200343621	0.879667426748653
**Ildr1**	1129.61951092521	0.0506388340646484	0.137926232247554	0.36714432954103	0.713511362449426	0.879667426748653
**Atp1b1**	4484.54266520229	0.0282441282812813	0.076933339524131	0.367124688151907	0.713526012646731	0.879667426748653
**Zgrf1**	414.849224016879	0.0562290983686306	0.153123576886375	0.36721385113904	0.713459508252655	0.879667426748653
**Trnt1**	5931.23857199449	0.0387373480557485	0.105613069178367	0.366785553692471	0.713778984258774	0.879910456498631
**Mis12**	3191.94346097148	-0.0525795816595855	0.143450865732319	-0.366533735374588	0.713966844232426	0.880073188185116
**Mycbp**	782.138589563916	-0.0511666476127021	0.139795521440919	-0.366010635285812	0.714357139598231	0.880163174955364
**Ccdc9**	1372.79262015138	-0.0518201826946713	0.141533471390878	-0.366133764581791	0.714265263656363	0.880163174955364
**Ipo11**	2598.41217419099	0.0387064702642629	0.105771345355865	0.365944766364045	0.714406291008623	0.880163174955364
**Zfp429**	1401.51022376827	-0.0485018125877789	0.132494279369537	-0.36606722055149	0.714314916601178	0.880163174955364
**Itpa-ps1**	163.05553955258	-0.0724878116283709	0.198068665321496	-0.365973141237218	0.714385117523959	0.880163174955364
**Rasgef1b**	350.673314216726	0.0827570568159831	0.225936397526747	0.366284749699021	0.714152608064254	0.880163174955364
**Gm27011**	27.8842936538378	-0.192425648912671	0.52587980137637	-0.365911846032193	0.714430856614781	0.880163174955364
**Col20a1**	122.739170426503	0.0922349078118921	0.25222200968579	0.365689369959407	0.714596879088153	0.880230067525955
**Dnajc27**	1318.84017584759	-0.0465494533991771	0.127282039908495	-0.365718945364502	0.714574807702807	0.880230067525955
**Eva1b**	1133.31611997407	0.0338684350915422	0.0926434846086419	0.365578164882444	0.714679870792725	0.880263481910495
**Pprc1**	9781.8123103371	-0.0317913169099225	0.0870118380771994	-0.365367720214304	0.714836933662444	0.880388116595211
**Hinfp**	2737.65367411523	-0.0324872108771058	0.0889733403068589	-0.365134216216465	0.715011220722247	0.880408327227299
**Sec24c**	31395.2174543585	0.0269150038100595	0.0737269429344335	0.365063336940411	0.715064127855899	0.880408327227299
**Rab2a**	15239.6907602066	0.0299642513715108	0.0820834256825393	0.365046306515016	0.715076840252267	0.880408327227299
**Gm20784**	28.5414409273179	-0.19681629468576	0.53896933440564	-0.365171600908988	0.714983315840297	0.880408327227299
**Sf1**	11139.3472312875	0.0223986495145961	0.0613815220967928	0.364908668756627	0.715179583142708	0.880466027938092
**Insig2**	2482.26525933497	-0.042956628417163	0.118208604858563	-0.36339679728528	0.716308493889207	0.881029813302024
**Pgam2**	109.714464034007	-0.0856502804878473	0.235441769217823	-0.363785409752874	0.716018258576696	0.881029813302024
**Raph1**	69.3570622229135	-0.149378385649819	0.410441174197714	-0.36394590757569	0.715898402709387	0.881029813302024
**Poglut2**	1037.28410481755	0.0506782258841938	0.139406013051376	0.363529698432141	0.716209232022558	0.881029813302024
**Ncor2**	1213.17340941141	-0.0845335333290926	0.232456001840263	-0.363653907233515	0.71611646667327	0.881029813302024
**Tmf1**	6139.29427137053	0.0607816270038209	0.16724773628846	0.363422718612992	0.716289133263229	0.881029813302024
**Sc5d**	3501.24482653296	0.035947536062598	0.0988620751622914	0.363613003303711	0.716147015312556	0.881029813302024
**Spdya**	23.8637391247925	0.257100007577242	0.706743443503639	0.363781241892651	0.716021371126495	0.881029813302024
**Rpl30**	6984.67223075905	0.032882678486625	0.0903387951842413	0.363992882787096	0.715863324089227	0.881029813302024
**Ifit3b**	865.729387046845	-0.089337706745647	0.245666046277831	-0.363655084205704	0.716115587671572	0.881029813302024
**Gm34727**	21.8605575621446	0.247763105768639	0.681480413493746	0.363565996707714	0.716182122220451	0.881029813302024
**Gm5512**	91.6784983878242	0.10388948204608	0.285460443682772	0.363936525515707	0.715905408810774	0.881029813302024
**Cmtm3**	697.418347982077	0.0548715945757958	0.151052206715653	0.363262449247686	0.71640884128939	0.881057114993148
**Proca1**	21.0668065758537	0.180321844543986	0.496661837712539	0.363067646538919	0.716554352264847	0.881057114993148
**Cd300lb**	44.9670582674424	-0.171152086944729	0.47129953856204	-0.363149277563315	0.716493375422396	0.881057114993148
**Oip5**	95.8872274361388	0.169950143456238	0.468013181868946	0.363131104080372	0.71650695051651	0.881057114993148
**Slc48a1**	3400.52033234841	0.0423889038139318	0.116840490200559	0.362792930269039	0.71675957343301	0.881240683410107
**Mccc2**	1723.79973567943	0.0363438326201649	0.100227466262947	0.362613502817846	0.716893622082391	0.88126961031064
**Cdca3**	309.148037620716	0.0786647005506888	0.216939215073552	0.362611713719063	0.716894958746068	0.88126961031064
**Gm5940**	740.023710673772	-0.0779469238526656	0.215080170018004	-0.362408695539626	0.717046642433762	0.881387311399391
**Npdc1**	99.4780095834374	0.155510390068189	0.429232421407805	0.362298797369833	0.717128756779491	0.881419486784278
**Mbd4**	1186.94834107668	0.043790020523163	0.12089339953164	0.362220110384955	0.717187552581416	0.881422998676029

**Wdr77**	7812.49747480821	-0.0297465568246598	0.0821424917966881	-0.362133606785217	0.717252190979849	0.881433690006978
**Gm7816**	183.667861848518	0.0872081585204002	0.240881457238185	0.36203765752782	0.717323889859893	0.881453055643947
**Gm42047**	1372.97181444444	-0.105059351837714	0.290285486785596	-0.36191734213468	0.71741380005704	0.881494794720741
**Dnajc10**	5548.70322439388	0.0292325809591266	0.080788607283855	0.361840387425127	0.717471309407537	0.881496719090414
**Zfp566**	1114.69466406588	0.0428192315232112	0.118493011494289	0.361365037340408	0.717826580781591	0.881795699179507
**Firre**	77.9125452454872	0.173998956565006	0.48150403792607	0.361365518998454	0.717826220764816	0.881795699179507
**Fam234a**	5693.96447707128	-0.0291473317831713	0.0806871286906422	-0.361238926903985	0.717920844567786	0.881842746168897
**Nucb2**	487.435612442595	0.0599443181674743	0.165980484489128	0.361152808729153	0.717985217800047	0.881853073261777
**Mterf3**	2673.93999747273	-0.0298907675588209	0.0828207757902417	-0.360909050580793	0.718167437575793	0.881870660371085
**Gm128**	148.029446776398	0.122104200884496	0.338276683308911	0.360959554439618	0.718129682436462	0.881870660371085
**Mir8099-2**	25.0467919728895	0.15117734984454	0.418868143645711	0.360918709474382	0.718160216829304	0.881870660371085
**Gm4787**	25.6997916580385	0.188172836260361	0.52153532441328	0.360805543655271	0.718244818336208	0.881896953364267
**Sec1**	21.7231761488648	0.365638854150621	1.01361240816291	0.360728470967823	0.718302438997119	0.881898981710893
**Mfge8**	3840.01925157143	0.0284354608635495	0.0788668914731355	0.360550039850823	0.718435842839632	0.88199404562712
**Phkb**	1565.02031415055	-0.0437228015024625	0.121311324557389	-0.360418136245626	0.718534465953037	0.882046399191493
**Elp6**	1114.94002871881	0.0458058072501661	0.127122567196838	0.360327896613667	0.718601939997729	0.882060510130421
**Ripk4**	29.5153963102213	-0.28991302651139	0.805280918596209	-0.360014772257084	0.718836086586214	0.882073064117979
**Fbxl15**	398.160997438794	0.0667757408104475	0.185428905751383	0.360115056171329	0.718761093900764	0.882073064117979
**Tspan17**	63.9581951543727	0.16371492501349	0.454517943548669	0.360194635519291	0.718701586094473	0.882073064117979
**Gm7535**	61.635150532676	0.223540195427861	0.620892924657223	0.360030186446835	0.718824559621458	0.882073064117979
**8430429K09Rik**	348.071687731227	-0.0582580523414574	0.162059483619286	-0.35948561010054	0.71923184084348	0.88248996388185
**Dbt**	1470.65211817911	-0.0351202375818876	0.0977594128879627	-0.359251723638492	0.719406785822466	0.88263588913841
**1700084E18Rik**	58.3495352788928	-0.110822270608423	0.308570264812458	-0.359147601846142	0.719484672719968	0.882662720962982
**Ghdc**	392.552996725595	0.0609280159398736	0.169824749838407	0.358769943708725	0.719767199213877	0.882940580616043
**Gm40309**	1854.11466419335	-0.0492325574299883	0.137371354654465	-0.358390273968141	0.7200512691796	0.883178186605237
**Gm49320**	190.143889072843	0.321823970048502	0.898043465102904	0.358361240356697	0.720072993800691	0.883178186605237
**Arid5b**	5403.63415953499	-0.0321835226158153	0.0899347617264258	-0.357854093322835	0.720452506878417	0.883574887210711
**Hykk**	77.4268666253209	-0.122084217171753	0.341268989256437	-0.357736041114524	0.720540858712843	0.883614468887716
**Ewsr1**	58002.8632293052	0.0279415654059023	0.0783621866421475	0.356569496120643	0.721414116857286	0.883978139514461
**Nedd1**	3481.1179814961	-0.0272318647118631	0.0763533260182105	-0.356655906585762	0.721349418802869	0.883978139514461
**Rab27b**	31.5837959549539	0.3340645146461	0.937223494087673	0.356440610754524	0.721510620822205	0.883978139514461
**Tmem144**	47.0784168954846	-0.162719064560717	0.456463192570118	-0.356477953117154	0.721482659968584	0.883978139514461
**Prdx1**	16390.072718499	0.0369539576181475	0.103599805903123	0.356699100891208	0.721317078704539	0.883978139514461
**Rchy1**	8501.67447807741	-0.0330208171500787	0.0925310457328037	-0.356862033586337	0.72119509351233	0.883978139514461
**Slc10a7**	2714.02744022517	0.0308362251656597	0.0864184202672696	0.356824680088936	0.721223058872358	0.883978139514461
**Ttyh2**	76.5242690124295	0.0957781471839722	0.26831525632988	0.356961242137561	0.721120821076299	0.883978139514461
**Traf7**	9719.75533385841	-0.0220319163536918	0.0617949451737714	-0.356532662853517	0.721441695612729	0.883978139514461
**Vapb**	1679.71739786721	0.051848956669529	0.145280367903448	0.356888941140259	0.721174948924612	0.883978139514461
**Bcl2l2**	18.5499950677063	-0.229201041427834	0.642485497570314	-0.356741190726644	0.721285566015573	0.883978139514461
**Gm50387**	5632.56622170551	0.0290647060957678	0.0815111778872238	0.356573255952463	0.721411301719234	0.883978139514461
**A930007A09Rik**	112.763481865485	-0.102734770782642	0.288325969580558	-0.356314663337801	0.721604929256708	0.884024947154249
**Krtcap3**	1211.17558408722	0.0358311883712578	0.100600694029181	0.356172377507299	0.721711476858343	0.884086740329388
**Ifi35**	11712.1908049253	0.0391441965791028	0.11002633799717	0.355771147996487	0.722011957792022	0.884386071123203
**Mrpl36**	3431.17202733912	0.0384227618505427	0.108071788367225	0.355529990120855	0.72219258165903	0.884401065454366
**Arl2**	2243.12930175643	-0.0326330747030042	0.09176421436229	-0.355618744515886	0.722126104060259	0.884401065454366
**Cdkl4**	202.941263346773	0.0882481530598805	0.248168590440913	0.355597591552955	0.722141947571795	0.884401065454366
**Trav7d-2**	346.154239890131	0.0597515293421886	0.168119445720159	0.355411172611449	0.722281580002901	0.884441316141259
**Crls1**	54.6644496947583	-0.173437403475371	0.488102918870778	-0.355329576550407	0.722342700396512	0.884447426384944
**Rtn1**	39.3905196381133	0.238576745052652	0.671672579086695	0.355197982590053	0.722441275979516	0.88449939282123
**Chfr**	6522.03458199305	0.0279764302249429	0.0789761992399634	0.354238751600828	0.723159964437248	0.88452282095156
**Mpp2**	31.8254864734606	-0.286735399815995	0.807833473594912	-0.354943697170661	0.722631771433155	0.88452282095156
**Rpf1**	4306.41733556508	-0.033616994614455	0.0947309798697799	-0.354868013195535	0.722688472669354	0.88452282095156
**Atp6v0d2**	30.3765732521399	0.178789533303159	0.504772215462881	0.354198444023325	0.723190169595669	0.88452282095156
**Dmpk**	19.4307776366844	-0.242650155693915	0.684481010025042	-0.354502392528081	0.722962411329744	0.88452282095156
**Mat2b**	20630.6053343242	-0.0232351703314222	0.0654991498210641	-0.354740029372868	0.722784359588395	0.88452282095156
**Pigw**	237.450559614972	-0.0850039612390359	0.239692727419663	-0.354637214712851	0.722861392641256	0.88452282095156

**Mettl15**	784.987749545606	-0.0461943695355883	0.130398186429086	-0.354256227027437	0.723146869067598	0.88452282095156
**Tomm5**	3063.73910488612	0.0447355455208299	0.126100813231576	0.354760166682478	0.722769272200964	0.88452282095156
**Gm14276**	128.278277695122	0.0842834270960277	0.237595743701337	0.354734583132827	0.722788440069254	0.88452282095156
**Gm12749**	19.0530826579767	-0.200715333123397	0.566274119562205	-0.354449066608542	0.72300236836004	0.88452282095156
**Gm13493**	517.166360652264	-0.0427929508820531	0.120670128280134	-0.35462754114846	0.722868640625756	0.88452282095156
**AC160138**	22.2738737216587	0.172974247945038	0.488058653198638	0.354412828891362	0.723029521657336	0.88452282095156
**2010204K13Rik**	70.7240178543782	-0.106768794381471	0.301854962905704	-0.353708924821702	0.723557033417486	0.884902838835713
**Ezh1**	11640.5115775869	-0.0312734507259095	0.088502568584001	-0.353362068765572	0.723817018652052	0.885152096461031
**Thap7**	4157.14326329387	0.037253414760661	0.105484328519948	0.353165397015502	0.723964447711792	0.885229274792921
**Ide**	8413.72861728621	-0.0578761155096121	0.163895576555465	-0.353127989943193	0.723992489957249	0.885229274792921
**Hnrnpll**	2163.11334163063	-0.0531318069820097	0.150512161392934	-0.353006737065593	0.724083389832386	0.885240088750547
**Dgka**	82897.0455161028	-0.0326392201517793	0.0924784642188487	-0.352938604976604	0.72413446825245	0.885240088750547
**Zfp652**	3450.65383043945	-0.0508651110445842	0.144138152769314	-0.35289137585932	0.724169876498987	0.885240088750547
**Gm10250**	1037.6787013596	0.0381862436081544	0.108289745716831	0.352630282353866	0.724365632138691	0.88541069471797
**Ralgps2**	2194.10246624332	-0.0383392512858322	0.10883250425742	-0.352277580557633	0.724630099986611	0.885587296106457
**Mamdc4**	99.1993943224379	-0.0794076101753106	0.225453540663081	-0.352212743884017	0.724678720314568	0.885587296106457
**Nol7**	3223.3535925156	-0.0306393054743021	0.0869785562978897	-0.352262750480321	0.724641220804997	0.885587296106457
**Gm11836**	30.8278312386854	0.177589418844456	0.504353749163304	0.352112815933394	0.724753657390045	0.885610188405967
**Bcl2l13**	5210.38362786166	0.0343677340453905	0.0976453615484302	0.351964839910442	0.724864631089561	0.885651960864702
**Aptx**	2236.89121591105	0.037747128381854	0.107277699137385	0.351863702198845	0.724940482013678	0.885651960864702
**Gm10221**	331.163592613151	0.0506007855816312	0.143816624966624	0.351842393696655	0.724956463237477	0.885651960864702
**Cpsf6**	9611.48349396574	-0.02546073208842	0.0723927364402178	-0.351702855015649	0.725061119208364	0.885711144690767
**Cul2**	5334.67122409076	0.0280385937185186	0.0798201106883572	0.351272298130356	0.725384075287223	0.88603696700006
**Nav1**	180.891088783621	0.154358299433227	0.439739747956044	0.351021940024071	0.725571888667836	0.886046635582968
**Hibadh**	7046.55719407803	-0.0193085603623984	0.0550206846478003	-0.350932753490743	0.725638798515404	0.886046635582968
**Ubr7**	3826.59212830508	0.025957883401764	0.0739779072645683	0.350886965603533	0.725673150485385	0.886046635582968
**Adnp2**	2157.3954221575	-0.0438392055774764	0.124882717348765	-0.35104301466347	0.725556078280654	0.886046635582968
**Gm17809**	19.4395025795802	-0.25667053537837	0.731476797166902	-0.350893611899224	0.725668164124757	0.886046635582968
**9230114K14Rik**	68.184079168808	-0.138519228661235	0.3949338403911	-0.350740337986892	0.725783160311492	0.886112293276629
**Sntb2**	3838.61882870386	0.0728271851413368	0.20789766556351	0.350303044259479	0.726111280846877	0.886375538604295
**AC123856**	66.9493719053146	-0.209091803060431	0.596843587004362	-0.350329311754677	0.726091569781234	0.886375538604295
**Nudt6**	1450.29593965485	0.0333946476810091	0.0954162417004092	0.349989132729233	0.726346853341177	0.886457080636428
**Cdk3**	40.6195735183375	-0.19176157417342	0.547742220123451	-0.350094564794002	0.726267729771095	0.886457080636428
**Ly6g6g**	17.128835357855	0.388638387448381	1.11031736067509	0.350024597662855	0.72632023765714	0.886457080636428
**Fam160a2**	5933.84495186622	-0.0369454652330816	0.105642552630417	-0.349721436231598	0.726547764567812	0.886506670235253
**Gm25265**	22.150238281895	-0.194791010718556	0.557007098555332	-0.349710104635599	0.726556269557533	0.886506670235253
**Gm9049**	252.81542494703	0.0655311005376336	0.187319074634618	0.349836772712323	0.726461200075547	0.886506670235253
**Alg13**	236.936396127843	0.0751698788653985	0.21507792534238	0.349500669330599	0.726713468408003	0.886561173869999
**Zfp58**	1322.65619805488	0.0464446965474431	0.132867592006455	0.349556245026152	0.72667175304195	0.886561173869999
**Pqbp1**	6376.31723992783	0.0256458491035953	0.0734428918981135	0.349194434488956	0.726943344182441	0.88658574977762
**Rhoj**	21.8079288746279	-0.238527043218037	0.682832057467451	-0.34932021806754	0.726848921520559	0.88658574977762
**Heatr1**	5339.53191702656	-0.0407929348481999	0.116840416733847	-0.34913376713747	0.726988887164885	0.88658574977762
**Gm13665**	77.4212246738284	0.117170064976019	0.335607712249739	0.349128046523641	0.726993181679618	0.88658574977762
**Gm15454**	25.8306017817548	0.203474331742086	0.582855574334017	0.34909905764319	0.727014944017049	0.88658574977762
**Lhfpl2**	31.0223235295548	0.424719253673128	1.21703594946196	0.348978396127816	0.727105528584591	0.886627597423572
**Igkv1-110**	70.6527490665681	-0.253800901476783	0.727658848469461	-0.348791060550725	0.727246175120819	0.886730479097636
**Zfp341**	126.317390842865	-0.136117766002329	0.390509022199914	-0.348564971010186	0.727415929333657	0.886868833063326
**Dcaf4**	2531.21255821452	-0.0342630452063783	0.0983334345041237	-0.348437389369751	0.727511727025125	0.88689328934734
**Erlin1**	990.735658967069	0.0521759687329253	0.149851063977617	0.348185507316242	0.727700871123311	0.88689328934734
**Fitm2**	494.780560365219	0.0799171719680809	0.229511598885694	0.348205373306134	0.727685952686259	0.88689328934734
**Trbj1-6**	652.142716680211	-0.0589801253583512	0.169403540818647	-0.348163474466521	0.727717416892287	0.88689328934734
**9230116N13Rik**	211.629057159379	-0.0894992345852117	0.256988855933721	-0.348261150313437	0.727644067292275	0.88689328934734
**Rnf219**	1349.03642208804	-0.0306961362740263	0.0882692468645347	-0.347755728800259	0.728023640111971	0.887115097490991
**Tmem106c**	2089.72511211388	-0.026907529915029	0.0773880365577	-0.347696247532613	0.728068315082407	0.887115097490991
**Nat8b-ps**	17.5861420172585	0.207184189499427	0.595680918435779	0.347810687042787	0.727982363099861	0.887115097490991
**Hyal3**	45.2258064346906	0.159055635171185	0.457845125893554	0.347400520778208	0.728290442152396	0.887317134229901

**Zik1**	105.790948721019	-0.146648227608832	0.422326283779095	-0.347239168485045	0.728411647141852	0.887396190197477
**Ube2a**	3270.19200244049	0.034466010554306	0.0992876436538662	0.347132928992257	0.728491456078179	0.887424806604087
**Gltp**	28043.0591371998	0.0235520756721476	0.0679276045962395	0.346723188785188	0.728799287536435	0.887675914751047
**Gm5173**	20.3409610643571	-0.235490154457576	0.679216399958808	-0.346708581347355	0.728810262685428	0.887675914751047
**Mpzl1**	42.7694860653536	0.260858707657551	0.752554000100819	0.34663121533153	0.728868391776768	0.887678099337343
**Anapc1**	11788.5364732197	0.0344424937517334	0.0995022192837612	0.346147995488523	0.729231495114619	0.887835503075487
**C2**	36.3315907403818	-0.183410331027975	0.529702090519727	-0.346251854222472	0.729153447966953	0.887835503075487
**Ankrd13b**	235.150331240038	0.122042780255549	0.352378988578437	0.346339549778187	0.729087549214359	0.887835503075487
**Mrps21**	1241.95550525335	0.0461947649049276	0.133478360640159	0.346084299233064	0.729279362588439	0.887835503075487
**Pcdhga12**	28.5229540179967	-0.192404870393499	0.555726314402217	-0.346222349755859	0.729175619523803	0.887835503075487
**Ppan**	7602.49723485326	-0.0263769709616028	0.076419951324145	-0.345158175379117	0.729975459974684	0.888496145589293
**Prmt3**	7263.69550128039	0.0256801164025171	0.0743944307857032	0.345188693982885	0.729952517895242	0.888496145589293
**Dnaaf3**	81.5842144511468	-0.0975153908018186	0.282540898371857	-0.345137257521837	0.729991184920923	0.888496145589293
**Strn**	2483.45990770005	0.0504884118066147	0.146435026242787	0.344783711261168	0.730256979597018	0.88868236233474
**Fmnl1**	7470.74623662043	-0.0420599813358038	0.12196917499931	-0.344841074279972	0.730213852104202	0.88868236233474
**Wnt10a**	32.7806438132588	0.296348778119583	0.859798423479929	0.344672390675186	0.730340676680578	0.888715579771092
**Mrpl24**	4615.28786883411	-0.0262514134221816	0.0762807138350707	-0.344142209771932	0.730739340585359	0.888933544578676
**Cd209d**	95.0291424884442	-0.152172079360126	0.442151771230979	-0.344162546124083	0.730724047538849	0.888933544578676
**Mfsd2b**	175.600833274975	0.12339166588891	0.358556956405413	0.344134073219301	0.730745459346804	0.888933544578676
**Zfp974**	322.156349713132	-0.0844898276061029	0.245510192397521	-0.344139796319739	0.730741155521531	0.888933544578676
**Supt5**	20201.6527583603	-0.0346288525836549	0.100707625635421	-0.343855317461433	0.730955096553148	0.889119920981006
**Fxyd4**	53.8545097451761	-0.235803130121751	0.687090768788021	-0.343190653743888	0.73145503542525	0.88914377849907
**Ern1**	244.452594289365	0.0704453453426409	0.205377598666284	0.343004036468003	0.731595423509579	0.88914377849907
**Pcnx**	669.553054448333	-0.0657332493973125	0.191251207207838	-0.343701095313236	0.731071087367648	0.88914377849907
**Dapk1**	174.212148048027	0.0996125401119314	0.290067722288228	0.343411322453005	0.731289042796144	0.88914377849907
**Slc25a46**	6835.4837343915	0.0261161000154325	0.0761260881661844	0.343063733400048	0.731550513838922	0.88914377849907
**Mrpl43**	2099.52820611992	-0.0259213438665634	0.0754592052466579	-0.343514668380522	0.731211307679034	0.88914377849907
**Ccnl1**	11536.5002424092	0.0359384147081102	0.10458088982017	0.343642273171585	0.731115329248749	0.88914377849907
**Polb**	1618.28158544971	0.0343219143457022	0.100029100971852	0.343119292408319	0.731508717931668	0.88914377849907
**Tmem201**	2637.29890155931	-0.0354414756590037	0.103275068956167	-0.343175521616414	0.731466418658012	0.88914377849907
**Tssc4**	3292.42410692822	-0.0299297479767838	0.0872269130954105	-0.343125153862158	0.731504308526113	0.88914377849907
**Gm10123**	16602.0851669186	-0.0738524510536498	0.215192788987478	-0.343192034459608	0.731453996776593	0.88914377849907
**Rbm6-ps1**	61.32992944614	-0.0993776279572506	0.289794622753005	-0.342924333837455	0.731655384789656	0.889148071736126
**Dhrs1**	2115.47662200932	-0.0358645762573869	0.104657200656321	-0.342686179569821	0.731834560970137	0.889189531099962
**Macroh2a1**	20255.7969096437	0.0239291734099831	0.0698151361511027	0.342750508402542	0.731786161510185	0.889189531099962
**Klhdc9**	34.8913618794596	-0.310764825827917	0.906934845771835	-0.342653970433173	0.73185879474744	0.889189531099962
**Pde5a**	28.2611495602997	0.311374650996242	0.90897169032681	0.342557039245404	0.731931726251736	0.889195325916265
**Ccdc78**	20.7922952821948	-0.328342733236653	0.958671550210927	-0.342497629312574	0.731976427775584	0.889195325916265
**Ntpcr**	1441.48270393569	0.0404141540032248	0.118028914526325	0.342408927214278	0.732043171152378	0.889198645114489
**Gm10608**	25.605832898845	0.153883570318769	0.449499827176463	0.342344003301158	0.732092024057261	0.889198645114489
**Thoc5**	3069.4050602922	0.0251570095319686	0.0735729333466615	0.341932941744128	0.732401358258866	0.889437241008474
**Zfp82**	1669.94441069294	0.0548496562588814	0.160383414982919	0.341990824080675	0.732357797707643	0.889437241008474
**Zfp296**	1100.77105256482	0.0867736399060294	0.253857767235034	0.341819912981784	0.732486422880438	0.889471991779212
**Gm13422**	34.4177806244267	0.117659396235183	0.344311881517968	0.341723311192335	0.732559127286949	0.889491729109999
**Cd79a**	605.126752543994	-0.0935299390900211	0.274122228257042	-0.341197938177852	0.732954575355262	0.889492085942149
**Hsp90ab1**	107085.024200934	0.0241040426179618	0.0706253217640502	0.341294623739771	0.732881794843905	0.889492085942149
**Gng5**	5056.35166310122	0.0295311672871707	0.0864461479873573	0.341613455020455	0.732641810118949	0.889492085942149
**5830418P13Rik**	57.4227397092602	0.15319868143885	0.448893434512124	0.341280735382892	0.732892249222153	0.889492085942149
**Gm37070**	24.743435045655	-0.177484917861109	0.519676168740304	-0.341529838267036	0.732704746031779	0.889492085942149
**Gm42940**	55.6820123918919	-0.183279528012309	0.537155275291625	-0.341203998997879	0.732950012973945	0.889492085942149
**Gm7240**	113.316656789219	0.138670787397029	0.406265542065775	0.341330418257767	0.732854850961278	0.889492085942149
**Daam1**	393.319974927433	-0.0988309850420084	0.289849457318534	-0.340973503819249	0.733123528628351	0.889512584155986
**Ankrd13c**	2335.72728397684	-0.0368438454552128	0.108025787918759	-0.341065278625149	0.733054439364406	0.889512584155986
**BC029722**	3721.75347731054	0.0238309214806165	0.0698955396500241	0.340950532751316	0.733140821884482	0.889512584155986
**Cd40**	69.9615110726013	0.183697666600835	0.53986216684956	0.340267716244736	0.733654926875771	0.88956065958498
**Pip4k2c**	4912.58277472922	-0.0223242324266456	0.0655659666512079	-0.340485065146742	0.733491267955509	0.88956065958498

Nradd	104.91447537971	-0.1169066134292	0.343880256883113	-0.339963144406214	0.733884283103199	0.88956065958498
Agpat1	3076.35925399097	-0.0430641071732252	0.126661352498174	-0.339994057570528	0.73386100302352	0.88956065958498
Heatr5b	2670.07771412348	-0.0271298805819521	0.0796500296948756	-0.340613565191145	0.733394515957921	0.88956065958498
Ppm1e	39.4203510577934	0.213308492245744	0.626488972123597	0.34048243742056	0.733493246502945	0.88956065958498
Rtl8c	438.501491225042	0.0518472709318906	0.152452127937987	0.340088863521673	0.733789608102499	0.88956065958498
Mmgt1	749.419899732383	-0.0547052232159358	0.160958625115832	-0.339871337597273	0.733953422398911	0.88956065958498
Pgghg	2248.97479560948	-0.0612142873936089	0.180119772147931	-0.339853235786542	0.733967055045955	0.88956065958498
Snora41	53.5314770588459	-0.118850582397293	0.349234082578321	-0.340317822131918	0.733617197176149	0.88956065958498
Gm17354	127.806331209945	0.119281365417166	0.35098420512648	0.339848242954928	0.733970815210022	0.88956065958498
Gm37053	48.5699938367879	-0.176185162361764	0.518067610655085	-0.340081407789577	0.733795222660362	0.88956065958498
Gm43712	16.8594290062849	-0.23367438098996	0.687338357183313	-0.339969941365631	0.73387916442936	0.88956065958498
Gm45709	36.7128843417994	-0.177608947457505	0.521691061705367	-0.340448515404722	0.733518788256703	0.88956065958498
Slc9a1	2208.72061948046	0.0469019191537519	0.138201968604181	0.339372294240481	0.734329287433574	0.889789801760291
Dtx4	1133.29162945939	-0.068288107343354	0.20119344629935	-0.33941516783678	0.734296993778922	0.889789801760291
Fbxo30	436.828229979413	-0.0656395132325922	0.19334033958032	-0.339502420317842	0.734231274093432	0.889789801760291
Zfp968-ps	45.6998411610752	-0.188363753822226	0.55536301089089	-0.339172307352737	0.73447992965065	0.889903902461
Fstl1	22.1155190935838	0.239138091904375	0.705757033198177	0.338839119775693	0.734730929372801	0.890053063945354
Map3k13	65.1688886733784	0.164119234973081	0.48448154064513	0.338752297465332	0.734796339755416	0.890053063945354
Rpsa-ps2	353.999855326005	-0.0519179163966252	0.153281788732151	-0.3387089674909	0.734828984506258	0.890053063945354
Gm37125	151.802611084601	-0.0920822800688815	0.271735404553693	-0.338867436947056	0.734709596136255	0.890053063945354
Yes1	177.253122614571	0.0739325345278515	0.218506707074315	0.3383536163158	0.73509672372732	0.890240494640795
Gm5903	270.791204187638	0.0506080524227248	0.149541568196213	0.338421303408576	0.735045722427493	0.890240494640795
Gm6180	222.068082488113	-0.0516639954145064	0.152760632108112	-0.338202288780414	0.735210751182708	0.890310160343216
Rars2	2831.92309753207	0.0337012440441234	0.0997743524536405	0.337774620584809	0.735533036810075	0.890631988090091
Pcdhgb7	101.95315076757	-0.104819779774532	0.31062980496062	-0.337442763381383	0.735783152506139	0.890866384973047
Agtpbp1	1797.13065526898	0.0405464362479549	0.120237307052973	0.33722009617274	0.735950989077708	0.891001132147325
Atf5	22.1165314879495	-0.285613864160966	0.847420529394523	-0.337039113703129	0.736087414844611	0.89109783310591
Fam189b	36993.9139658482	-0.0220986999494525	0.0655973625477644	-0.336883970500512	0.736204369428118	0.891170949456776
Adcy5	17.8984375871982	-0.313614172244187	0.931776576855325	-0.336576578585621	0.736436114637823	0.891314529406881
Tmub2	6615.69590986297	0.0209483859708261	0.062231213941327	0.336621843671195	0.736401987423832	0.891314529406881
Wnt5b	313.969841774785	0.110156912828418	0.327511376833977	0.336345301629809	0.736610491982479	0.89145710615729
Mtpn	13002.2257123142	-0.0235471896394484	0.0700967742727429	-0.335924011964195	0.73692816926288	0.891499206378135
Ccdc157	407.684835426636	-0.0631410524611154	0.187820438272263	-0.336177750632157	0.736736829938616	0.891499206378135
Timd4	39.5623777952628	-0.31657902451627	0.942057695282803	-0.336050568984773	0.736832733070721	0.891499206378135
Rpl15-ps3	1614.21937798859	0.0515519991600633	0.153461975360625	0.335926857704781	0.736926023255585	0.891499206378135
Gm12403	41.0906294959882	0.188346929329555	0.560602296031279	0.33597245438154	0.736891638531538	0.891499206378135
Prkar2b	327.271737793331	0.0537718093183178	0.160126015808795	0.335809325216248	0.737014657629777	0.891535387707078
Actr3b	51.1651245274272	0.208307469754277	0.620554211249592	0.335679729470878	0.73711239328196	0.891585167801017
Mir142b	23.2781219251571	-0.269102188393449	0.802605913710607	-0.335285578883086	0.737409671176974	0.891876280989836
Pigv	3737.95897099784	0.0275912459330612	0.0823236137329811	0.335155913132703	0.73750747680884	0.891917302846618
4933440M02Rik	85.2968353313537	0.154890526776457	0.462234869589126	0.335090528575088	0.737556797365706	0.891917302846618
Gm10644	162.765754029278	0.132587692666813	0.396077182300456	0.334752160921592	0.737812050446352	0.891919626641523
Mex3a	118.121778958193	0.15783970181567	0.471507559314691	0.334755400411991	0.73780960654755	0.891919626641523
Gm11249	220.125554951264	0.0598276983186435	0.178743381513609	0.334712803416939	0.737841742290554	0.891919626641523
Zfp383	156.916830733681	-0.0634757593717382	0.189549174500867	-0.334877530007117	0.737717472887423	0.891919626641523
Ndor1.2	59.0279857096937	-0.555474405992396	1.65912965787275	-0.334798671916091	0.737776962415075	0.891919626641523
Ppp1cb	4732.35234768426	0.0318537114096291	0.0952948135445239	0.334264900940766	0.738179673639565	0.892054266228356
Ms4a1	299.333901780544	-0.0918449036073873	0.274770582159099	-0.334260323232881	0.738183127668343	0.892054266228356
Gm9800	2051.11990267817	-0.027342939403101	0.0817703258980484	-0.334387066491484	0.738087497729889	0.892054266228356
Arfgef2	2604.82946821308	0.0590023180929195	0.176519671680337	0.334253500084501	0.738188275964469	0.892054266228356
Gm43514	19.7789625810318	0.195278612210534	0.584334097095895	0.334190000516926	0.738236189098037	0.892054266228356
Gm20708	479.489916329664	-0.392957523922696	1.17653604740172	-0.3339953117378	0.738383096427125	0.892094958626147
Gm6052	31.2295783427681	-0.144594862239023	0.432848637504048	-0.334054100465248	0.738338734902932	0.892094958626147
Wtap	8548.08648363961	0.0215773130459594	0.0646358168835375	0.333829045354807	0.738508564483318	0.892178137741596
Myo1h	35.5193905952995	0.329569514809763	0.988146755006271	0.333522842776195	0.738739649511597	0.892388888174964
Gm14455	76.2429301775384	0.138271579250906	0.414709032495344	0.333418296724603	0.738818553754012	0.892415787773244

**Elp4**	901.795657445881	-0.0370879641967997	0.11136222890862	-0.333038989613191	0.739104852025597	0.892678125348583
**Gm46209**	45.7310865083315	-0.122299634494333	0.367287739119194	-0.332980444127059	0.739149044959254	0.892678125348583
**Gm24060**	26.0594754103583	-0.159364773518362	0.479112205122257	-0.332625159231117	0.739417249467957	0.892933599008783
**Fbxl17**	295.28658023566	0.0993623015363782	0.298793681839976	0.332544854779069	0.73947787567844	0.892938377428552
**Sema4f**	4435.96668034876	-0.0357953916986673	0.107726324394256	-0.332280822723179	0.739677219287952	0.893042214720731
**Clec16a**	3081.69277100792	-0.0257491572109698	0.0774853618738145	-0.332309956206986	0.739655222714405	0.893042214720731
**Uba52**	12274.5594425559	-0.0236176786705285	0.071125084834825	-0.332058355014637	0.739845195485576	0.893108154850703
**Gm5182**	161.498603376682	-0.0709789584582697	0.213706865544743	-0.332132326574268	0.739789341232216	0.893108154850703
**Adcy1**	80.0698762168576	-0.114675199091913	0.345498208889431	-0.331912571878518	0.739955277053878	0.893172613799445
**Tmbim4**	2420.90930437625	-0.0381477198889628	0.115079342780605	-0.331490595681366	0.740273943387098	0.893420383421454
**Xntrpc**	231.427335401964	-0.194527282605883	0.58670816370658	-0.331557143123662	0.740223685387991	0.893420383421454
**Chchd7**	1160.07795494065	-0.0441294085579695	0.133181432609214	-0.331348054255098	0.740381597338617	0.893481874034203
**Eps8l1**	961.146774359498	0.105763317811323	0.31982637293337	0.330689795345165	0.740878811181568	0.893540978334215
**Asnsd1**	3400.72408895467	0.0289603247218494	0.0875774029005002	0.330682616322298	0.74088423443146	0.893540978334215
**Nudt15**	627.519005853823	-0.0442421189393341	0.133643400794371	-0.33104604250087	0.740609707537232	0.893540978334215
**Atp6v0a2**	14981.4991756608	-0.0226084990899469	0.0683410491350788	-0.33081873012017	0.740781412165176	0.893540978334215
**Tlcd5**	31.2105471688106	0.166523334920545	0.503402976642797	0.330795292533016	0.740799116916328	0.893540978334215
**Gm10108**	174.676556330982	0.0653434281351147	0.197551681794945	0.330766245781396	0.740821059020269	0.893540978334215
**Shkbp1**	3308.36126245528	-0.0205184030021321	0.0620457820084751	-0.330697790211257	0.740872771634307	0.893540978334215
**Gm43518**	42.7362812245312	1.0149969732919	3.06469517024607	0.331190189205801	0.740500830340841	0.893540978334215
**Bcl10**	3403.54626040829	-0.0376118692407443	0.113819795358387	-0.330451035536611	0.741059184430105	0.893615180918739
**Rnu3a.1**	29.1290551871759	0.199393156598186	0.603319640289637	0.330493395677395	0.741027182041341	0.893615180918739
**Efcab11**	67.3192218993933	-0.140352104556428	0.425071579670183	-0.330184635409707	0.741260455604175	0.893784646556123
**Spcs1**	5999.20213083846	0.0351427655298716	0.106456171375633	0.330114873339465	0.741313165313319	0.893784646556123
**2510002D24Rik**	6076.48140284551	0.0329451489052786	0.0998668239382375	0.329890824661185	0.7414824566383	0.893920357249403
**Pdhx**	1509.80704980989	-0.039979806308183	0.121267246700607	-0.329683466854721	0.741639147479509	0.894040857381579
**Ncapg**	213.468791311792	0.107658530857009	0.326665371293651	0.329568238073912	0.741726225246776	0.89407742741841
**Rcn3**	9198.37036325752	0.0174719515535426	0.0530617219573677	0.329275999892735	0.741947082888018	0.894275237899985
**Gm12333**	21.8386208298566	0.260762394154269	0.79214372633959	0.329185708961206	0.742015324136512	0.8942890823328
**Nbr1**	7434.14211550004	0.0240853320514327	0.0732829130362518	0.328662317770011	0.742410939444206	0.89435822027174
**Mief2**	940.732483085328	0.0449337384916201	0.136667848992342	0.328780608042919	0.742321521508031	0.89435822027174
**Gtf2a1**	1762.55933533432	-0.0472684109081872	0.143777999143017	-0.328759693346192	0.742337331084244	0.89435822027174
**Caml**	1413.66610754801	0.0400033034368621	0.121716645638035	0.328659266176504	0.742413246249563	0.89435822027174
**Rln3**	99.5963893563738	0.0845926244021841	0.257093607752176	0.329034335555035	0.742129735638522	0.89435822027174
**Rpl9-ps7**	415.629190435219	0.0471879075542291	0.14352159030084	0.328786125176826	0.742317351083228	0.89435822027174
**Spata5l1**	1092.09733641802	0.0407476130256445	0.124108995223038	0.328321190195897	0.74266882392912	0.894597711081045
**Ptges3-ps**	801.593197441894	-0.0517075091214581	0.157554333929819	-0.328188427647384	0.742769197127174	0.894650224669996
**Braf**	1121.37842885076	-0.0562644502188859	0.172053609317561	-0.327016971292001	0.743655049074134	0.894759667704729
**Sugt1**	8154.81304435181	-0.022994304842193	0.0703145809988762	-0.327020434674289	0.74365242957355	0.894759667704729
**Ccdc14**	345.214969278051	-0.0855808253536496	0.26128248564121	-0.327541377844852	0.743258452310497	0.894759667704729
**Tnf**	435.802230763367	0.0778474762917999	0.2379959387129	0.327095818158936	0.74359541463276	0.894759667704729
**Fastkd2**	2675.8802479456	-0.0238653788583314	0.0727834019109509	-0.327895897027872	0.742990376089329	0.894759667704729
**Ell3**	70.9584020240442	0.171644406810729	0.524436914652365	0.327292762990393	0.743446465591844	0.894759667704729
**Hivep3**	796.841867037056	-0.0542817097139385	0.165731850702214	-0.327527324916388	0.743269079333291	0.894759667704729
**Ccdc163**	521.72089409109	-0.0621591140101054	0.189842253060475	-0.32742507533507	0.743346403382029	0.894759667704729
**Zfp7**	2309.80310802703	-0.0306489967575221	0.0936955907560811	-0.327112476800653	0.743582815358807	0.894759667704729
**Dock4**	128.599063662986	-0.0952878153153468	0.290775153902946	-0.327702742260955	0.743136429768203	0.894759667704729
**Stam2**	3708.63658992683	0.029972192151428	0.0914157334911908	0.327866889065834	0.743012309824902	0.894759667704729
**Ighv6-3**	43.2519372374649	-0.244977211534865	0.748699558710924	-0.327203627522708	0.74351387740539	0.894759667704729
**Gm13166**	155.331274840518	-0.0889437548346547	0.271462127545427	-0.32764701153302	0.74317857219089	0.894759667704729
**4732487G21Rik**	87.5706819066716	-0.109823490824356	0.335146748532705	-0.327687770521931	0.743147751011695	0.894759667704729
**Mzt1**	2083.91891948454	-0.0283781221041082	0.0868511039323702	-0.326744518137684	0.743861126061186	0.894939281006803
**Eml6**	46.9876876800459	-0.206571805258659	0.632744975998703	-0.326469293466319	0.744069317983972	0.895121411167617
**Rfxank**	4525.55831241307	-0.0239278300047193	0.073341177270207	-0.326253693972804	0.744232419894463	0.895143169726038
**Mrpl41**	1894.62830722206	-0.0380167698440911	0.116513437039001	-0.326286570976062	0.744207547563685	0.895143169726038
**Gm10146**	448.991153964044	0.0922675456493612	0.282903438430015	0.326145013158567	0.744314641730695	0.895143169726038

**Gm13077**	20.3789556427114	0.153583725818143	0.470872689587441	0.326168259944544	0.744297054265271	0.895143169726038
**Gm32802**	162.144071571135	0.0794455971327016	0.243659933099615	0.326051132502864	0.744385668939477	0.895160267532576
**Gm6829**	18.1967110493346	-0.185927837524631	0.571892383872142	-0.32510983319232	0.74509794706085	0.895948439548063
**Rpa3**	583.988255103779	0.0375463268046846	0.1156522275307	0.32464853990571	0.745447085819961	0.896153261147501
**Acat2**	5153.17284585662	0.0365875237940596	0.112667094376491	0.324740102658525	0.745377780615333	0.896153261147501
**Brk1**	10566.4109284133	0.0181882175419402	0.0560460972673494	0.324522463271248	0.74554251846441	0.896153261147501
**Fam129c**	540.259183092063	-0.059720718828767€	0.183915549240244	-0.3247181604572	0.745394388804991	0.896153261147501
**Tspyl1**	4906.04388752651	0.0274640583342831	0.0846326346261846	0.324509079217368	0.745552649640324	0.896153261147501
**Decr2**	1062.1262698713	-0.0336263603119613	0.103697481100777	-0.324273646331699	0.745730869802733	0.896164201167703
**Ccr10**	100.735438696137	0.0839535118040996	0.258958697764831	0.324196532222064	0.745789247313073	0.896164201167703
**Rps6kc1**	920.263457040944	-0.05662446411671	0.174587561409272	-0.324332751197377	0.745686126775549	0.896164201167703
**Gm7968**	27.3897650318257	-0.216566788062663	0.668001290271407	-0.324201152328122	0.745785749724108	0.896164201167703
**Traj49**	40.7158390574131	-0.279494889925389	0.862344760765041	-0.324110382113797	0.745854467049879	0.896174228862661
**Slc43a1**	229.471668536578	-0.0670838290721265	0.207069803858817	-0.323967221787032	0.745962850265808	0.896236113735291
**C3**	28.9043505226552	-0.310526815237267	0.958766195657843	-0.323881689450058	0.746027607125443	0.896245578337573
**Coa4**	572.459233515732	-0.0483951821691644	0.149543169857461	-0.323620144037958	0.746225635285922	0.896377127615512
**Gm15473**	62.5989978288488	-0.150930125047265	0.466428559612329	-0.323586800029377	0.746250882786081	0.896377127615512
**Oser1**	7533.4063718412	-0.0309186602666693	0.0955829427767399	-0.323474663663456	0.746335792479658	0.896401961145146
**Ighj3**	33.3686712637689	0.149951641504563	0.463659118573518	0.323409236436242	0.746385335420722	0.896401961145146
**Tmtc3**	90.1246527181848	-0.113276993322351	0.350496019584956	-0.323190527117795	0.746550954553921	0.896532534936829
**Ybx3**	159.179916383692	-0.101004069523784	0.312819116812866	-0.322883302506752	0.746783622271711	0.896675267555845
**Clasrp**	4098.23431238038	-0.0327378729970111	0.101370738754092	-0.322951903077548	0.74673166760141	0.896675267555845
**Relch**	4177.53180315363	-0.0388629858618115	0.120552820835169	-0.322373094155537	0.747170064867783	0.897032612339051
**Vezt**	1054.13116467195	0.0249941990301406	0.0775398480638774	0.32234005681247	0.747195090242435	0.897032612339051
**4931406P16Rik**	2496.461131809	-0.0389321248554546	0.120871899050783	-0.322094094336168	0.747381412121774	0.897187940789486
**Tada3**	2940.95349030305	-0.034606078265251	0.107479741766947	-0.321977683387897	0.747469601072343	0.897225451671891
**Anp32e**	5775.73810765831	0.0341650250470842	0.106168530403292	0.321799924302474	0.747604271649346	0.897318746829582
**Rps8-ps1**	21.7855285141698	0.167434171834686	0.520492733632337	0.321683975617145	0.747692118716647	0.897355831717435
**Rufy4**	17.8996577418321	0.467592400831918	1.45570918678508	0.321212784171949	0.748049144708488	0.897638451433533
**C630043F03Rik**	45.7395623694918	-0.172908103789024	0.538214321043417	-0.321262547332098	0.748011436161379	0.897638451433533
**Scamp4**	14280.9209232225	0.048567911663552	0.15123234540959	0.321147645578154	0.748098505059666	0.897638451433533
**Usp54**	272.216658623925	-0.130225746887445	0.405720704946814	-0.320973875130471	0.748230188887054	0.897659744654584
**Snrpd2**	1854.27487820265	0.0302516934292017	0.0942401585660338	0.321006393553597	0.748205545758323	0.897659744654584
**Tm7sf2**	721.883660866537	-0.0305334556210568	0.0951719871586452	-0.320823979120659	0.748343786486173	0.89772767747717
**Cog7**	2566.35425001397	-0.0416821272322822	0.129991590147123	-0.320652491327375	0.748473753961112	0.897746893595589
**Cbx3-ps6**	38.9978898825356	0.187082680379582	0.583437607454001	0.320655847325254	0.748471210442874	0.897746893595589
**C920021L13Rik**	34.1691047409476	-0.292460994282299	0.912517214295093	-0.320499153003071	0.74858997233649	0.897817947488664
**Tcta**	1897.63360624419	-0.0404413946264904	0.126348545283919	-0.320078039170251	0.748909172836561	0.898064066696019
**Dock5**	644.3802730799	0.0647090420740101	0.202165373713969	0.320079748995803	0.74890787671713	0.898064066696019
**Wdr60**	32.8637251713456	0.237360752434485	0.742767822264558	0.319562513775593	0.749299995025664	0.898293091535694
**Rpl5-ps1**	213.357305059311	0.0530225482856488	0.165923318781672	0.319560557702067	0.749301478056744	0.898293091535694
**Gm30211**	20.3668313932766	0.297853561084213	0.932175152900026	0.319525316843709	0.749328196683706	0.898293091535694
**Gm4316**	30.8876132978571	0.136273917438287	0.426477762474105	0.319533465584999	0.749322018510662	0.898293091535694
**Ccdc32**	1579.33418503794	0.0404558011416104	0.126716465873237	0.319262385222146	0.749527553533334	0.898327045031166
**Prpf4**	4253.63268672955	0.0259858406822363	0.0813783831255633	0.319321172087449	0.749482979410817	0.898327045031166
**Gm36161**	29.1670096445936	0.17963614041237	0.562537529909383	0.319331832742443	0.749474896243417	0.898327045031166
**Lyz2**	2509.06180046005	0.0604074075829647	0.189297620452158	0.319113401630063	0.749640521508226	0.898394105370027
**Acadl**	3790.2477800704	0.0262969656032094	0.0824278843216099	0.319029971709649	0.749703785244572	0.898401592828254
**Ak6**	5870.3968928415	-0.0382868147424908	0.120055818731144	-0.318908447313422	0.749795938499275	0.898443696040538
**4930444A19Rik**	231.623041819057	-0.0923700107865255	0.289880074325741	-0.318649051685865	0.749992652935544	0.89861107385791
**P4htm**	531.299664788908	0.0457170238760544	0.143606897007704	0.318348385966461	0.750220685172216	0.898815947099955
**Wdpcp**	78.4061578634843	-0.12356799811087	0.388807929014742	-0.31781244385627	0.750627210898098	0.898824603854497
**Itgb3**	1613.94094720688	-0.0504791991268963	0.158744153545539	-0.317990918087043	0.750491825957575	0.898824603854497
**Fn1**	26.1309011358015	-0.835319271889775	2.62767426111894	-0.317893006850123	0.75056609739264	0.898824603854497
**Foxo3**	1376.83246815607	-0.0525663474127885	0.165174150906187	-0.318248025640794	0.750296805758124	0.898824603854497
**Rps16-ps2**	1092.69685024453	-0.0343416235383916	0.107933946983605	-0.318172590719841	0.75035402270093	0.898824603854497

**Ccdc173**	80.0101654828355	-0.114729098724503	0.360956231696295	-0.31784767417739	0.750600485675625	0.898824603854497
**Gm3854**	87.5117013142248	0.130772089348278	0.411171148381889	0.318047824763277	0.750448659957333	0.898824603854497
**Npas1**	28.5296947166388	-0.147049253697814	0.463580195092476	-0.317203485512319	0.751089205332507	0.898967632745532
**Ube2b**	8748.45370822707	0.0258705795825424	0.0815969122356021	0.31705341383316	0.751203072942846	0.898967632745532
**Ctc1**	2012.30425186986	-0.0411064705457834	0.129601316715495	-0.317176334219055	0.751109806105667	0.898967632745532
**Auh**	3199.36213390518	0.0263560509606526	0.0830191477398268	0.317469543812346	0.750887345638743	0.898967632745532
**Tnfsf11**	51.3918579651614	0.159491461654103	0.502462879234993	0.317419392049281	0.750925394715566	0.898967632745532
**Ehd1**	6113.2919350518	0.0332562370461075	0.104791907645768	0.317355011405316	0.750974239829793	0.898967632745532
**Rassf2**	14855.184635968	-0.0432684232688995	0.13646766167202	-0.317059900776265	0.751198150831426	0.898967632745532
**Mecr**	1857.69000914424	-0.0284677612924099	0.0897415249395866	-0.317219495786084	0.75107705778001	0.898967632745532
**Arid3a**	571.812203068575	-0.0679519753798772	0.214536835369144	-0.316738033647953	0.751442386833915	0.899185729749544
**Cln8**	818.7603419736	0.0435497078023711	0.137731086280598	0.316193743754026	0.75185545610482	0.899318878527639
**Gpat3**	36.5308450874521	0.147218336413183	0.465639886747253	0.31616350017088	0.751878410472274	0.899318878527639
**1810055G02Rik**	418.761045774448	-0.0757956888345511	0.239484455834079	-0.316495233774439	0.75162664228889	0.899318878527639
**Cdk7**	3590.62522662279	0.0281846702113367	0.089106691817742	0.316302509232252	0.751772906763059	0.899318878527639
**Gm28884**	21.823207419848	0.177817817511163	0.562109369044067	0.316340248541957	0.751744264559217	0.899318878527639
**Gm2427**	112.871488905964	-0.19116439247047	0.6046823508752	-0.316140188635875	0.751896103682371	0.899318878527639
**Cdkn3**	51.1238573997367	0.153116663878412	0.484615489800894	0.315954952123633	0.752036700882551	0.8993740143439
**Hadhb-ps**	4450.41289798317	0.0285099139732982	0.0902415064117014	0.315929056450252	0.752056356730039	0.8993740143439
**Cr1l**	10146.3975776384	0.020263760200063	0.0642552216457374	0.31536363397491	0.752485574976867	0.89979360213243
**Depdc5**	2560.04741521887	-0.0418196225815187	0.132627484363889	-0.315316412598	0.75252142469386	0.89979360213243
**Ptprn**	41.8933185464888	0.138679970915046	0.439995284357381	0.315185130035178	0.752621095128593	0.899844494722001
**Gemin7**	6240.20218787009	0.0346610273188401	0.110000247095805	0.315099540536958	0.752686077361499	0.899853909027704
**Smg9**	5701.0051568879	0.0219172255644259	0.0696083624391743	0.314864835149337	0.752864282048018	0.899950915841476
**Exog**	1925.74975368832	-0.046224766067816	0.146853910038118	-0.314767009307533	0.752938562133539	0.899950915841476
**Sbsn**	56.3217930384882	-0.183378572451676	0.582555089323847	-0.314783229624718	0.752926245735319	0.899950915841476
**Gm44103**	68.7956885757479	-0.126145562287632	0.400952502148241	-0.314614727709051	0.753054195540677	0.900020855516873
**mt-Ta**	92.3995456435943	-0.127831612696063	0.406674096784073	-0.314334288087045	0.753267158869732	0.900138830753099
**Gm4032**	55.0104022242601	0.147481549518096	0.469099894762912	0.314392629724709	0.753222853207249	0.900138830753099
**Cmbl**	22.3110245861215	-0.166978279183433	0.531611778464659	-0.31409815573628	0.753446490214783	0.900284852226764
**6030458C11Rik**	2591.14437389077	0.0240143014218321	0.0764753720835736	0.314013528376022	0.753510763927985	0.900293380892725
**Zfp61**	1192.36184115487	-0.0410149430519647	0.130682003662068	-0.313853031807085	0.753632664308646	0.900370755289358
**Gm7331**	126.087281428822	-0.0764187146929427	0.243667293770206	-0.313619089006711	0.753810359580995	0.900514771487319
**Gm9040**	106.565754902186	0.0592275225290155	0.188985723513895	0.313396808117418	0.753979208927706	0.900648199156536
**Gm27782**	19.1715868283792	0.226681121592545	0.723807220822052	0.313178861817786	0.754144777047862	0.900777687381986
**Igkj1**	29.3443841650841	-0.212024617753061	0.678991571322524	-0.312263990759244	0.754839903939212	0.901471302589825
**Pde2a**	31962.1195262721	-0.0332943709495556	0.106600753778573	-0.31232772536218	0.754791471397945	0.901471302589825
**Aars2**	2305.32590452154	0.0452495619392642	0.144955412123793	0.312161935013648	0.754917459094882	0.901495597041157
**Trbj1-3**	474.535420443662	-0.0418776620942804	0.134250177552678	-0.31193748014112	0.755088037630342	0.901562643702455
**2810403G07Rik**	48.858882442899	0.170359527727093	0.546008935099799	0.312008681132581	0.755033925856732	0.901562643702455
**Aifm2**	59.1882607561725	0.160576247717409	0.514946220795359	0.311831102419571	0.755168885473808	0.901590856827609
**Tagln**	25.1029489321221	0.183389429088649	0.588694624610635	0.311518776326425	0.755406271073545	0.901805940852022
**Gm15477**	16.9800915700859	-0.198550462051129	0.637726785772195	-0.311340948005991	0.755541441034896	0.901898976241428
**Atp6v0d1**	1601.45006989145	0.0396057499847591	0.127452252528016	0.310749705863794	0.75599090679875	0.901905230714345
**Tmem160**	3334.01570475433	-0.0410520494722769	0.131915257224136	-0.311200162408247	0.755648459577742	0.901905230714345
**Styxl1**	27.1274100189557	-0.230920998450403	0.742159308522845	-0.31114747979112	0.755688507613563	0.901905230714345
**Prr11**	21.1249674096615	-0.224398442359906	0.721323956669945	-0.311092457535808	0.755730334885908	0.901905230714345
**Tbce**	3048.53976763129	-0.0265203607935281	0.0852854616236668	-0.310959925509376	0.755831087115037	0.901905230714345
**Thbs1**	246.877497009628	0.118135194207542	0.38046043200717	0.310505861501298	0.756176302783983	0.901905230714345
**Aph1c**	35.7601964412319	0.133847331886567	0.430864500408058	0.310648316953019	0.75606799154622	0.901905230714345
**Zfp317**	2282.10539852961	0.038260181916358	0.123200076505185	0.310553231797285	0.756140285832112	0.901905230714345
**Tsga10**	198.673275274937	-0.0738451601791308	0.237498914008405	-0.310928411977991	0.755855044641471	0.901905230714345
**Rpl7a-ps11**	4187.94486967424	-0.0394842769799036	0.127049755699234	-0.3107780629927	0.755969347655432	0.901905230714345
**Gm12168**	40.3656022298287	0.13358962663192	0.430138490764736	0.310573523411991	0.756124857715888	0.901905230714345
**Retreg3**	5099.29586879395	-0.0340438073101268	0.109889381754794	-0.309800699271308	0.756712519816089	0.902013052221727
**Fam89b**	1686.09774683483	0.0351654569969555	0.113515785589952	0.309784730063731	0.756724664423368	0.902013052221727

**Ctnnal1**	92.7406464152359	0.132808737760855	0.428705090241168	0.309790438191773	0.756720323376186	0.902013052221727
**Gm4950**	140.23651786974	0.102664092765588	0.331000904922933	0.310162574297165	0.756437329407035	0.902013052221727
**Trbv13-3**	3477.27597364918	-0.0196803839242141	0.0634335109550557	-0.310252162112833	0.756369206490722	0.902013052221727
**Ly6c1**	450.522169248975	0.0904990539717825	0.292070975496842	0.309852952070415	0.756672781900794	0.902013052221727
**Mir155hg**	215.876252360788	0.103423663259714	0.333748347816538	0.309885169279	0.756648281242954	0.902013052221727
**Gm41664**	65.2112920923961	-0.0916505984313002	0.295521661095385	-0.310131575775484	0.756460901241841	0.902013052221727
**Clba1**	44.2951377620278	-0.115806178244774	0.373969397091434	-0.309667526662508	0.756813799631278	0.902051062086993
**Mettl6**	2874.39228724102	0.035614432440264	0.115104947065468	0.309408356011038	0.757010914866281	0.902132861710786
**Hsf4**	24.2522482408363	0.267630941621437	0.865098255980897	0.309364791538023	0.757044049882014	0.902132861710786
**B3gnt9**	292.365034465472	0.0505110043568292	0.163280314932205	0.309351463327357	0.757054187370802	0.902132861710786
**Gm42515**	71.5800279940213	-0.105464875587017	0.341039574358596	-0.309245270978797	0.757134959176538	0.902160885643127
**Lonp2**	12703.4160883112	-0.0198927193897863	0.0643735630255229	-0.3090200146588	0.757306301955228	0.902296816628244
**Eif3s6-ps1**	50.6999758021864	-0.0875570569430689	0.283462604257786	-0.308883978443389	0.757409784621045	0.90235188086147
**Numbl**	116.91747227197	0.148667431085198	0.482053378681808	0.308404499708589	0.757774558462895	0.902649963539449
**Noxred1**	27.9087715768053	0.164229265625306	0.53243742498936	0.308448012700436	0.757741452784308	0.902649963539449
**Topors**	6220.2534650423	0.0276943957249445	0.0898765011750569	0.30813833830717	0.75797706980752	0.902686470331577
**Mtmr7**	197.27118401908	0.0878450138667787	0.285031928692709	0.308193591748396	0.757935028353192	0.902686470331577
**Gm49774**	21.5244692482919	0.224414349579555	0.728104379232795	0.308217277605198	0.757917006385106	0.902686470331577
**Hdac9**	50.9766909394153	0.176208927418066	0.57283266476656	0.307609775517733	0.758379280887138	0.903028967651979
**Igf1r**	1787.0620671228	0.0431478502199053	0.140262195935467	0.307622805504608	0.758369364901755	0.903028967651979
**Parpbp**	33.5409936651918	0.158295410502762	0.515041360238389	0.307345045899797	0.758580752090759	0.903200612867923
**Mtfr1l**	10770.4634656528	0.0243281579844845	0.0792113859359688	0.307129558421694	0.758744759903817	0.90332762990589
**Car15**	63.1536469216359	0.0995342354465846	0.324209679231451	0.307005749126719	0.758838996237282	0.903371567219013
**Cdc42ep4**	111.392203378629	-0.144059735620779	0.469558590446558	-0.306798211238721	0.75899696987177	0.903491369212773
**Calcoco1**	3242.98976891728	0.0271141496415076	0.0884644108286861	0.306497826499008	0.759225634462121	0.903695295529509
**Gm24451**	40.1495441590565	0.138098251965773	0.451084473286796	0.306147207771373	0.759492565752928	0.903944735899153
**Ucp2**	67755.124146342	-0.0240798238478107	0.0786989320392728	-0.305973959542351	0.759624472804752	0.90396517015214
**Gm12932**	69.8885533857402	-0.0849320584522202	0.277567044889866	-0.305987544327965	0.759614129421678	0.90396517015214
**Rhbdf2**	1629.3937240774	0.0326177765669547	0.106758309585438	0.305529159215946	0.759963165132873	0.904299923953986
**Gm49392**	146.961481780223	-0.36488252494072	1.19563820955588	-0.305178039664905	0.760230556933022	0.904549791255277
**Tmcc1**	2445.42916120053	0.0373133721153453	0.122310281073647	0.305071428074616	0.760311751661761	0.904571020224156
**C1qb**	370.463306672136	0.0586717124887611	0.192363837219357	0.305003857985305	0.76036321399181	0.904571020224156
**Gabarap**	7837.15289856921	0.0186204889192728	0.06111730109176	0.304668049580862	0.760618986123161	0.904676455333868
**Nudt5**	3163.90032575701	-0.0295218130930777	0.0969004185806327	-0.304661357768151	0.760624083281277	0.904676455333868
**4933433G19Rik**	32.8598769547691	-0.21911504900135	0.719093646101221	-0.304710033511418	0.760587007172947	0.904676455333868
**Oxnad1**	1170.18550301058	0.0389610112866811	0.128134964972384	0.304062293184988	0.761080433247063	0.904688668903477
**Bcorl1**	161.97588961645	-0.103939571270623	0.341562888124951	-0.304305809806243	0.760894919335072	0.904688668903477
**Nphp4**	125.986204204367	-0.0715398631658768	0.234913597084178	-0.304536919334821	0.760718870010405	0.904688668903477
**Cpsf2**	4837.85238359757	0.0297388808284875	0.0976885793323858	0.304425358949083	0.760803850380117	0.904688668903477
**Ythdf3**	5215.77437787636	0.0184111258165368	0.0605831798362781	0.303898307521851	0.761205367252195	0.904688668903477
**Snip1**	2805.79509199215	0.041583454285232	0.136798109590472	0.303976819633831	0.761145551291208	0.904688668903477
**Apol9b**	25.2309338079087	0.258361352421548	0.850211728331766	0.303878838425917	0.761220200377848	0.904688668903477
**Ddx3y**	7537.94062651585	-0.0235293831677545	0.0774418792972868	-0.303832801854265	0.761255275095779	0.904688668903477
**Xlr3b**	1509.06306820559	-0.147808220860661	0.486061647386737	-0.30409356849144	0.761056606570707	0.904688668903477
**Gm13502**	103.203799729787	0.0810197005565132	0.266671093407925	0.303818833609303	0.761265917432963	0.904688668903477
**Gm14414**	142.448498921612	-0.0571118668082692	0.187630652809526	-0.304384523280674	0.760834957396112	0.904688668903477
**Mphosph9**	2864.54102694423	0.0373375014956195	0.122950671041504	0.303678712603494	0.761372677437233	0.904747306061725
**Ssna1**	5674.50157177849	0.0365421555108194	0.120404388848788	0.303495211928791	0.761512495841548	0.904845215064872
**Il1rl2**	933.121773442854	0.0448246796072108	0.147837261181755	0.303202854604443	0.761735273693151	0.905041676035514
**Cep72**	570.477162007698	-0.0544453083360803	0.179703490776629	-0.302973014607466	0.761910426872008	0.905113283792388
**Ccdc22**	3063.29796858641	-0.0468241940557866	0.154536671931634	-0.302997298120289	0.761891920663326	0.905113283792388
**Hk1os**	111.360775362083	0.0697237245009062	0.230439588199424	0.302568343598005	0.76221884240715	0.905411405941158
**Gstt1**	28.7144052754083	0.282458958159297	0.936221867448109	0.301700876662072	0.762880099926443	0.905991990845717
**Gpr89**	2806.0795189895	0.027083041905827	0.0897568171670376	0.3017379933986	0.762851802826446	0.905991990845717
**Syf2**	9019.34475359264	0.0273211536641254	0.0905424788527251	0.30174956562174	0.762842980446553	0.905991990845717
**1700001O22Rik**	127.984800622687	-0.101969430745846	0.33841475312399	-0.301314968701988	0.763174327645944	0.906218960517988

**Parp10**	6721.51552894952	-0.037829098404707	0.125616098129849	-0.301148491060463	0.763301265722837	0.906218960517988
**Gm13736**	99.7766094558385	-0.0760341850546956	0.252472787691647	-0.301157941613725	0.76329405956941	0.906218960517988
**Mfsd4b4**	454.662473518965	-0.0442204363704128	0.146809753339812	-0.301209118361899	0.763255037078124	0.906218960517988
**Cast**	18400.1821468295	0.0158891422591882	0.052815484084895	0.300842499779954	0.763534598603453	0.906291123931797
**Wfikkn1**	98.863033680261	-0.0982674147958829	0.326575359824476	-0.30090272226508	0.763488674400623	0.906291123931797
**Trav16n**	807.033316340268	0.0473749455231349	0.157402023146929	0.30098053745416	0.763429335661912	0.906291123931797
**Nup155**	1561.80218163735	-0.0389966591394368	0.129671814888403	-0.300733503059226	0.763617718968011	0.906321512336468
**Gm20498**	89.6655596666521	0.120030696149877	0.400176147354793	0.299944654231126	0.764219372376322	0.906489359419881
**Fbxl3**	3904.68029581599	-0.0424168020371324	0.14137019826986	-0.300040620698314	0.76414617133211	0.906489359419881
**Lyrm1**	445.966569431743	0.0498784538259189	0.166168352838494	0.300168190716783	0.764048867079159	0.906489359419881
**Igfbp7**	17.1476825859382	0.387400075662399	1.29080559563922	0.300122711716751	0.764083555838043	0.906489359419881
**Rnf24**	53.3410011548545	0.14491603226965	0.482777032117902	0.300171761763221	0.764046143310895	0.906489359419881
**1600012H06Rik**	1236.73385925147	-0.0541472097698638	0.180517164069687	-0.299956018303948	0.764210704009365	0.906489359419881
**Gm13340**	367.815126998254	-0.0753979255015993	0.251232023793359	-0.300112717969485	0.764091178555669	0.906489359419881
**Gm13274**	25.6922358671505	-0.272528758472079	0.907347256180945	-0.300357725904371	0.763904305666598	0.906489359419881
**Foxj2**	1997.39345616344	-0.0445155802309167	0.148580183337831	-0.299606443005258	0.764477369021626	0.906522419000133
**Lasp1**	14910.0470698279	-0.0427804981411922	0.142746047971382	-0.299696550266448	0.764408630216481	0.906522419000133
**Gm5527**	258.847727441607	0.0483562629700436	0.161377875774505	0.299646173541238	0.764447060141433	0.906522419000133
**4930568A12Rik**	37.1761008994259	0.199212959007873	0.664631959179252	0.299734245783003	0.764379874541152	0.906522419000133
**Pcdhga4**	20.4608712596799	0.37553259316041	1.25481399046127	0.29927351465245	0.76473136128383	0.906687137678654
**Gm50211**	20.0216601952568	0.176622441012694	0.590088310843297	0.299315268184659	0.764699505972386	0.906687137678654
**Nfs1**	5821.04400836898	-0.0170535392575187	0.0570600093857909	-0.298870249778918	0.765039047426554	0.906910608903986
**Tmem126b**	4726.55347479898	-0.0305815684490457	0.102449896834753	-0.298502676858449	0.765319533356352	0.906910608903986
**Arhgap45**	136826.498139234	-0.0291245951309335	0.0974568244727945	-0.298846133028516	0.76505744938644	0.906910608903986
**Slain2**	7003.1256057701	0.0302801336747649	0.101378201827661	0.298684856595109	0.765180512594036	0.906910608903986
**Gm10553**	50.2774504412382	-0.103883876338375	0.347984905926142	-0.298529834395817	0.765298809048634	0.906910608903986
**1700018A23Rik**	56.9049634136726	-0.112721368722009	0.377409162034433	-0.298671521683209	0.765190688165242	0.906910608903986
**E330037G11Rik**	20.1362770870295	-0.181611685285209	0.608417456676033	-0.29849847878693	0.765322736980155	0.906910608903986
**Tcof1**	16925.5247795931	-0.0230319872215604	0.077211944203682	-0.298295651781581	0.765477522693653	0.906957615240536
**Mir7091**	30.7922920497814	0.105466721932345	0.353512092970423	0.298339785341285	0.76544384174348	0.906957615240536
**Uvrag**	6469.70882728801	-0.0323368686198982	0.108458769427034	-0.298148953659786	0.765589479961079	0.907022062838099
**Zbtb22**	682.325603049611	0.0397427054634106	0.133398004465029	0.297925786992033	0.76575980598526	0.907025601595693
**Fancm**	952.83698781897	-0.0554270954185039	0.186015006424973	-0.297971096438715	0.765725223838576	0.907025601595693
**Gm10827**	33.4780552313782	-0.165818818927474	0.556590695715302	-0.297918776228144	0.765765156947865	0.907025601595693
**Icam2**	9336.86899522894	0.0182853098927847	0.0614374915124789	0.297624617194383	0.765989683776646	0.907119708983
**Zdhhc24**	873.372834229349	-0.0407842325069262	0.13704912605208	-0.297588417247023	0.76601731596927	0.907119708983
**Trav9-2**	577.170604084457	0.0395797819756284	0.132960503258235	0.297680747332588	0.765946839032573	0.907119708983
**Comt**	2539.15618076632	0.0287294945423514	0.0966348507950489	0.297299517782495	0.766237849762686	0.9071864492341
**Snai3**	2013.69043359968	0.0397467737590496	0.133697729189259	0.297288323444784	0.766246395434196	0.9071864492341
**Zfand4**	69.0065044668068	-0.153968325559875	0.517709993824825	-0.297402652829554	0.766159118597338	0.9071864492341
**Igkv12-46**	43.7840733884373	-0.187089510125249	0.629628224114339	-0.297142826448761	0.766357469311149	0.907249785419668
**Gm17108**	31.995518755309	-0.125604532110946	0.42304723168199	-0.296904276176342	0.766539591389815	0.907397215951418
**Papola**	17667.3882546517	0.0212919012299317	0.0719230145867149	0.296037385978321	0.767201530428313	0.907402550969158
**Chaf1b**	1851.2421007233	0.0285094961517697	0.0964162702865693	0.29569175479443	0.767465494495524	0.907402550969158
**Psd4**	18357.2677334574	-0.029980290981775	0.101379211862852	-0.295724245936465	0.767440679341002	0.907402550969158
**Zfp64**	2668.84861843108	0.0228591439757549	0.0772008147292885	0.296099776354855	0.767153884775891	0.907402550969158
**Tmem59**	21950.9097855183	-0.0279261972042697	0.0942795984314334	-0.29620615349332	0.767072649787378	0.907402550969158
**Akr7a5**	2273.14831629098	0.0295485815659901	0.0998712329433702	0.295866794622882	0.767331810403327	0.907402550969158
**Cotl1**	40758.8218796578	0.0364185064858486	0.122738052709289	0.296717323453939	0.766682330146635	0.907402550969158
**Rmdn2**	196.692486263949	0.0750632256136397	0.2529402224093	0.296762708985741	0.766647677479366	0.907402550969158
**Neto2**	277.71215496176	0.109150692883354	0.368025440963009	0.296584639903535	0.766783639092411	0.907402550969158
**Lonp1**	6574.63193878724	0.0239930395017777	0.0810120968507402	0.296166133632899	0.767103210686117	0.907402550969158
**Smg7**	5779.50773446895	0.0435195226091125	0.146905293246261	0.296242032178921	0.76704525157707	0.907402550969158
**Zfp518b**	72.2987241063112	-0.130417337166945	0.440809492201218	-0.295858731434516	0.767337968392661	0.907402550969158
**Nup188**	2741.71554233841	0.0409561922926425	0.138456980435182	0.295804459723979	0.76737941697119	0.907402550969158
**Sh2d3c**	18123.1696275523	0.0375585555578279	0.12676685815539	0.296280558691366	0.767015831718469	0.907402550969158

Gm9396	50.959574213827	-0.0896784811767143	0.302565410628129	-0.296393698772576	0.766929436916843	0.907402550969158
Gm9105	37.157889763238	-0.113741270078885	0.384286868483518	-0.295980111232354	0.767245270202412	0.907402550969158
Prmt9	4069.82211489254	0.0189203942754327	0.0640313177931546	0.295486567003864	0.767622212459346	0.907451661919267
Large2	24.1127513719158	-0.296293997117382	1.00271962219463	-0.29549037493541	0.767619303957644	0.907451661919267
Xaf1	2641.98132098795	-0.0286985414343964	0.0972133587085376	-0.29521191136333	0.767832003324586	0.907572746830285
Med9	1237.81476532409	0.0340979163197063	0.11552041094872	0.295167893185928	0.767865627414758	0.907572746830285
Gm13641	33.4793121499712	-0.165347089688079	0.56025882698896	-0.295126255442893	0.767897433568659	0.907572746830285
Gm6685	47.7605788374229	-0.127707771493431	0.43289276791509	-0.295010175634258	0.767986106438736	0.907609471173417
Gm47168	18.1638932093072	-0.232967969898224	0.790146600787053	-0.294841450518383	0.768115000248607	0.907693719732811
Gm36501	60.1672957100268	-0.0863725402294962	0.293221196938622	-0.294564448720861	0.768326623531306	0.907875711059827
Otub2	155.539686246587	0.0650992495371921	0.221180842088829	0.294325896051374	0.768508886365239	0.908022984587363
Rpl7l1	8385.13592792079	-0.0391162086908441	0.133050640074658	-0.293994892988828	0.768761805826237	0.908253713309141
Lrr1	22.5812584724216	0.25553943588596	0.869690436559232	0.29382803943085	0.768889307972856	0.908295430480444
Gm18588	657.546401418999	-0.0612916884679909	0.208618597785573	-0.29379781629531	0.768912403831862	0.908295430480444
Cdk5	2371.58292255488	-0.0236234981773934	0.0804626863240124	-0.293595693316337	0.76906686706161	0.908369386922036
Zpr1	11904.2798608169	0.0213205026047808	0.0726629804470622	0.293416296353459	0.769203970653021	0.908369386922036
Ccdc148	54.9413763874599	0.158664224454274	0.540778554560977	0.293399623775916	0.769216712987052	0.908369386922036
Ing1	2861.28856316904	0.0220506902497538	0.0751175494863386	0.293549116025998	0.76910246292234	0.908369386922036
Cox6b2	41.8134751441978	0.149925633440993	0.51110071462186	0.293338727870721	0.769263254368703	0.908369386922036
Rfc3	942.260577436439	0.0534191954011603	0.18231015950084	0.293012718256737	0.769512430378322	0.908527451331576
Egln2	6729.91588662373	-0.0261977378014549	0.0893940400753099	-0.293059109750322	0.769476970930157	0.908527451331576
Mterf4	891.053524307014	0.0438706536086227	0.149890179868746	0.292685308984477	0.769762700133365	0.90874913521201
Cmtm7	11159.5720678907	-0.0224258140135064	0.0766755906054741	-0.29247657352776	0.76992226883089	0.90874913521201
Cpsf1	15583.7510615283	-0.0290925102149545	0.0994523822059954	-0.292527032230311	0.769883694570476	0.90874913521201
Trav7d-6	74.2756065556934	0.0675071295868762	0.23082098602478	0.292465302871676	0.769930885008589	0.90874913521201
Cops3	6751.18777713035	0.0312813889370407	0.107029330629598	0.29226931302876	0.77008071962979	0.908857905715422
Coa6	771.490435999837	0.0380072663230789	0.130207956222332	0.291896650755971	0.770365644343376	0.908989924960205
Arhgap15os	33.5564084424243	0.126970724724072	0.434817765236401	0.292009055000412	0.770279700670819	0.908989924960205
Rpl7a-ps10	18.8127245623689	-0.166874889187959	0.571548851395335	-0.291969599414929	0.770309867875144	0.908989924960205
Spast	1143.14860661427	-0.0405087970163551	0.13887419348997	-0.291694201768889	0.770520442847769	0.909060839597598
2310057M21Rik	1461.37081606641	-0.0419310720046485	0.143763437935692	-0.291667148523569	0.77054112925465	0.909060839597598
Pop1	417.879606159531	0.0657604723453116	0.225991818471877	0.290986075469342	0.771061969106057	0.909160049451026
Idh1	773.735445111085	0.037537762239929	0.129044538689924	0.290889971950902	0.771135471068647	0.909160049451026
Nmnat1	372.743508505369	0.0335833514364877	0.115475928699818	0.290825558318638	0.771184737099749	0.909160049451026
Rad51ap1	145.160124691419	0.11349994729138	0.39016352612452	0.290903530677947	0.771125100948691	0.909160049451026
Lrrc14	3667.52026267459	-0.0220879340788723	0.0758999143835053	-0.29101395249627	0.771040648562688	0.909160049451026
Baiap3	7920.17896877464	0.0326906365229603	0.112325567482939	0.291034688321745	0.771024789771853	0.909160049451026
4931415C17Rik	65.4172495371058	-0.163671779696087	0.561903047304071	-0.291281174717526	0.77083628393391	0.909160049451026
Rras2	10050.0427465236	0.0622406508290362	0.214030502214459	0.290802713562158	0.771202209872591	0.909160049451026
4930594M22Rik	73.9476941362485	-0.137180844424052	0.470860170235275	-0.29134093961591	0.770790579464763	0.909160049451026
Srsf3-ps	123.129597194104	-0.0656293204111173	0.225554058286933	-0.29096936188853	0.771074751851409	0.909160049451026
Scrn2	1177.75208869787	-0.0284295391618125	0.0978312798133652	-0.290597641327479	0.771359064216752	0.909276933856764
Cox6c	3648.64095578788	0.0307546311426677	0.10590988257873	0.290384904541895	0.771521790852058	0.909325740092998
Aldh6a1	2177.2665947319	-0.0259577542628491	0.0894349452878527	-0.290241741405468	0.77163130486663	0.909325740092998
Aspm	79.7693843462551	-0.129182559476714	0.445009419012488	-0.290291742056563	0.77159305587169	0.909325740092998
Trav13-4-dv7	110.928651747678	-0.074644272819578	0.257148186043053	-0.290277267626071	0.771604128318787	0.909325740092998
Gm5921	122.88504312403	0.0583064669713109	0.200981457855393	0.290108687604717	0.771733089727892	0.909377676850314
Zbtb42	1068.99033280003	-0.053621761137185	0.184933044274943	-0.289952297856865	0.771852731384254	0.909450645959896
Hmgxb3	5868.41141320741	0.0356269369478957	0.12300238989012	0.289644266096957	0.77208839843709	0.90966030313842
Gm44037	27.1965870835911	0.161817127134214	0.559264946193437	0.289338940757151	0.772322015629422	0.909867514039089
Lsm2	1930.46472292981	0.0319924604440837	0.110647009183447	0.289139857282919	0.772474353842667	0.90991092124534
1700084J12Rik	17.2050832901445	0.16181004115147	0.559553614036572	0.289177010196014	0.77244592385392	0.90991092124534
Gm13464	211.85234077436	0.041516307886784	0.143802069537922	0.288704522961234	0.77280750120301	0.910235296490942
Zfp108	63.7315087811163	-0.120492682213628	0.417910499766282	-0.288321739417923	0.773100467740703	0.910282785350482
Klhl36	2027.13885139711	-0.0321106795206495	0.111340570052512	-0.288400530961043	0.77304016134003	0.910282785350482
Mettl25	648.177194769658	0.0312811784917438	0.108502857048662	0.28829820101156	0.773118484109609	0.910282785350482

**Palb2**	801.493556497176	-0.0418348506473861	0.145060997161416	-0.28839489225925	0.773044477110598	0.910282785350482
**Traj42**	23.7806661567405	0.16111909565712	0.558908709593897	0.288274440693882	0.773136670454116	0.910282785350482
**Plekhg5**	121.908769826091	0.0730247578415065	0.253453812429925	0.288118600945079	0.773255954579684	0.910286427827153
**Klf1**	50.417546052574	0.182718726545008	0.634343157733559	0.288043977959569	0.77331307500757	0.910286427827153
**3300005D01Rik**	50.0024314980068	-0.114073287727059	0.395949559368081	-0.288100554800755	0.773269767952037	0.910286427827153
**Hddc2**	1621.23773883128	0.0329635076731894	0.11484158963237	0.287034581972541	0.774085841240849	0.910477958246247
**Septin8**	369.752526277161	0.0784690088884852	0.272932880253681	0.287502952431203	0.77372724161142	0.910477958246247
**Rrn3**	5382.83914881088	0.0235350908355088	0.0818870696750427	0.287409122452476	0.773799077024143	0.910477958246247
**Ndufaf4**	1651.70057551402	0.0366906729541108	0.127681694726911	0.287360478983192	0.773836318802167	0.910477958246247
**Hdhd3**	39.7276165588984	-0.175418609693305	0.610168370052214	-0.287492138732615	0.773735520385887	0.910477958246247
**Rabggta**	8456.05499246812	0.0227914463900769	0.0794159727910777	0.286988191280299	0.774121362086306	0.910477958246247
**Gm10143**	324.035682210946	-0.223171702981291	0.776909054639251	-0.287255891340999	0.773916393590449	0.910477958246247
**Sars2**	2535.77613723109	-0.0309623115966059	0.107934680736099	-0.286861566508998	0.77421831969509	0.910477958246247
**Gm7785**	21.2501630939653	-0.182137550888883	0.63495683800462	-0.286850286487596	0.774226957069332	0.910477958246247
**A430078I02Rik**	30.2535316227891	-0.151479395413154	0.527460601018835	-0.287186180580234	0.773969767144358	0.910477958246247
**Prkag2os1**	29.8195237078293	0.161360061223113	0.560961087172275	0.287649294956458	0.773615206919742	0.910477958246247
**Gm10269**	539.017593105424	0.0345639658978912	0.120195158875076	0.287565374690465	0.773679452747962	0.910477958246247
**Gm6501**	95.2311923382907	0.0870785419554186	0.303444761717827	0.286966700174554	0.774137817751118	0.910477958246247
**Fbxw11**	7916.81881198677	-0.0241003745971037	0.0840630103938563	-0.28669416529562	0.774346505560026	0.910511658200353
**Dcaf5**	2973.33133381457	0.0353696423280491	0.123384507977871	0.286661939231405	0.774371183064221	0.910511658200353
**Vps16**	16996.306879654	-0.0277107717463983	0.0967169693696491	-0.286514061875622	0.77448442488721	0.910576860390073
**Cyp39a1**	282.950960009704	-0.0785920842050377	0.274556663461426	-0.286250871547613	0.774685983180682	0.91074588054749
**Ints9**	9164.65338031394	0.0180078337514811	0.0629399790713189	0.286111212891824	0.774792943736003	0.910801872420672
**Gm7290**	31.598920233677	0.106897175958371	0.373717034499309	0.286037740028596	0.774849216208612	0.910801872420672
**Senp8**	331.863291849748	0.045450190012564	0.15903314855798	0.285790669584799	0.775038454812316	0.910945781823255
**Trp53**	29591.7726014525	-0.0194573739218434	0.0681102021495288	-0.285674881409495	0.775127145019921	0.910945781823255
**Gm5953**	23.3687912816592	-0.258068431257068	0.903438148356113	-0.285651465710903	0.775145081086895	0.910945781823255
**Pex3**	2030.09274085185	0.0264581941114916	0.0927677503507066	0.285208965523762	0.775484052007316	0.911252035072723
**Gm5619**	121.384070312351	-0.0762498103884399	0.267392807581841	-0.285160289381015	0.775521342281222	0.911252035072723
**Plxdc2**	86.8697754703678	-0.1072034169515	0.376142908762781	-0.285007146098051	0.775638667092897	0.911293583879083
**Rad18**	3256.68028220169	-0.0247013631138217	0.0867027133075317	-0.28489723298747	0.775722875934388	0.911293583879083
**Gm4691**	27.7208243893551	-0.114383342432228	0.401503314193929	-0.284887666897266	0.775730205024443	0.911293583879083
**Naif1**	1799.51141947105	-0.0258935573868387	0.0909429316312056	-0.284723143650603	0.775856258151854	0.911373718480599
**Gen1**	89.0087123279106	-0.105111410300985	0.369325896386409	-0.284603412133905	0.775947996816317	0.911413536029423
**Ufd1**	6469.96229367583	-0.0216115287494659	0.076271366001909	-0.283350487638113	0.776908178794901	0.911454260536911
**AI597479**	1456.37530586622	-0.0464263524282699	0.163455700950251	-0.284030181623339	0.776387251125481	0.911454260536911
**Sirt3**	1484.33368829554	-0.0419416578646731	0.147469872270175	-0.284408314857919	0.776097487659015	0.911454260536911
**Anapc2**	7801.74594308757	-0.0215568169926552	0.0758413704912254	-0.284235594017243	0.776229839761296	0.911454260536911
**Myef2**	1061.06885064262	-0.0366222926924712	0.129112575327659	-0.283646210289989	0.776681520173457	0.911454260536911
**Sgip1**	31.2684485554575	0.131315158420851	0.462455738632615	0.283951841119158	0.776447287340505	0.911454260536911
**Tceanc2**	2170.99551316588	0.0214056207017737	0.0753264780510017	0.284171266938573	0.776279133818242	0.911454260536911
**Gabarapl1**	272.211894809489	0.0654888950664235	0.230755188983651	0.283802480693352	0.776561753345049	0.911454260536911
**Tamm41**	2363.02104035085	-0.031185533559737	0.110026482669463	-0.283436612741892	0.776842165667039	0.911454260536911
**AW554918**	985.988157429485	-0.0406376421071459	0.14333872188443	-0.283507774960564	0.776787622499899	0.911454260536911
**Camk4**	3869.44244456119	-0.0208574629846895	0.073479687461085	-0.283853452639352	0.776522689205742	0.911454260536911
**Arhgap11a**	678.792643711695	0.0639522573447676	0.224944849237808	0.284301941393458	0.776178998487733	0.911454260536911
**Atp1b2**	19.3118395349234	-0.191275398791082	0.674487779144881	-0.283586159312747	0.776727545112342	0.911454260536911
**Gm12258**	907.972872338461	0.0433444387458375	0.152956456898199	0.28337763324817	0.77688737206696	0.911454260536911
**Iqck**	24.3025960950504	-0.135659143339921	0.478556503558809	-0.283475707322093	0.776812201003854	0.911454260536911
**2010320M18Rik**	143.252178636554	0.101444953552248	0.356713039178977	0.284388128299816	0.776112955829811	0.911454260536911
**Pdcl**	2750.28864613933	-0.0214997319622304	0.0759693914824245	-0.283005188572616	0.777172859628032	0.911629029193694
**G2e3**	1342.22458742382	-0.0282035319578224	0.0996571431919096	-0.283005623626105	0.777172526131787	0.911629029193694
**Aspscr1**	9106.82443407328	0.0171498185956406	0.0606734034898387	0.28265792932669	0.777439069077985	0.911846806241885
**Nr4a2**	204.307237531296	0.0864587162308787	0.305940119172772	0.282600126013723	0.777483383720595	0.911846806241885
**Med12l**	103.184756460482	-0.0878309274009033	0.310865717546696	-0.282536550167227	0.777532124685281	0.911846806241885
**Clybl**	1324.47140847202	0.0291661379587665	0.103294937168151	0.282357865335527	0.777669119622528	0.911939593502432

**Cox5b**	3756.27780187622	0.0277654955797961	0.0983797869507846	0.282227644929603	0.777768961977512	0.911988802937912
**Gtf2ird2**	1843.20679678499	-0.0292675805450477	0.10373933499738	-0.282126163097025	0.777846772489491	0.912012173087053
**Apoe**	1581.63560092148	0.0310320621726262	0.110132591720735	0.281770016375486	0.778119863214205	0.912165645278886
**Pfas**	2181.05663626219	0.0247310475096727	0.0877483136058581	0.281840715717431	0.778065649289971	0.912165645278886
**Dcp2**	691.938627169716	-0.040863098233288	0.145075286950631	-0.281668222701457	0.778197922903142	0.912165645278886
**Coro7**	14954.2071646151	0.0230464030085554	0.0818253789008505	0.281653483529618	0.778209225708197	0.912165645278886
**Oxa1l**	2999.41436386862	0.0208713487496424	0.074259170142434	0.281060894023052	0.778663694673251	0.912237756953082
**Usp18**	5321.65593771993	0.0473661458267001	0.168419346954207	0.281239339086019	0.778526833546897	0.912237756953082
**Mybbp1a**	16103.0427836352	0.0237287055539422	0.0844057247837251	0.281126731803357	0.778613198597585	0.912237756953082
**Rpl21-ps8**	597.410933555678	-0.0265188789461734	0.0943366933115584	-0.281108845511381	0.77862691688307	0.912237756953082
**U2af1l4**	801.414024513487	-0.0379998980791148	0.135133090773193	-0.281203499910275	0.778554320390988	0.912237756953082
**Gm13217**	64.3703839106181	0.0984077069551799	0.349990702307392	0.281172346312074	0.778578213857979	0.912237756953082
**1700023H06Rik**	26.8749460760672	0.144393278291085	0.513773094781262	0.281044842086487	0.778676006284125	0.912237756953082
**Pfdn4**	313.601745303892	-0.0643789978208774	0.229262551448628	-0.280809043666703	0.778856866773689	0.912381804308454
**Evi5l**	1002.62324391954	-0.0341902653258776	0.121970496178224	-0.280315866518396	0.779235178840694	0.912492626107107
**Ddx19a**	350.056822034369	-0.0478844190714988	0.170646007752217	-0.280606734972835	0.779012049748694	0.912492626107107
**Zfp354c**	547.113621674433	-0.0749055720744962	0.267243908222211	-0.280289165701816	0.779255662307133	0.912492626107107
**Fcgr3**	210.424835681366	0.0755395167303777	0.269546993454119	0.280246185506928	0.779288634780991	0.912492626107107
**Itpripl2**	58.1506454953604	0.119650524381018	0.426875800921915	0.280293528287646	0.779252315549556	0.912492626107107
**Igkv3-2**	23.7914010294769	-0.44034888635713	1.57136832028403	-0.280232763173903	0.77929893187303	0.912492626107107
**Mars2**	1157.50062198474	0.0348982637746272	0.124576411913991	0.28013540636185	0.779373621393237	0.912512271681768
**Ubxn2b**	1992.35073727672	-0.0265885772880034	0.09496193122443	-0.279991960411639	0.779483672968711	0.912532660474147
**Tbc1d10b**	1650.93767008318	0.0366211209073137	0.130807592957859	0.27996173677098	0.779506861076475	0.912532660474147
**Gm18190**	104.801253248386	0.1015777734358	0.363279871087607	0.279612996810671	0.779774434685513	0.912778082411383
**Traj2**	40.06415638183	0.119678658233826	0.428514807569409	0.279287100748416	0.780024504711223	0.913002980295257
**Gucd1**	6713.06092693254	0.0272015530872947	0.0976011666985708	0.278701105810582	0.780474213811707	0.913461500930778
**2210406H18Rik**	31.6441875371279	-0.203210742956016	0.729364945146722	-0.278613257064524	0.780541637760324	0.913472562848294
**Slfn4**	26.6368487565577	0.228880059405217	0.822761345840745	0.278185236292712	0.780870167477381	0.913592950851954
**Cnp**	42230.3643555655	-0.020887086771124	0.0751011477166637	-0.278119408373428	0.780920697534188	0.913592950851954
**Fgd6**	218.090825814764	0.0558938113901733	0.201038061341812	0.278026016651348	0.78099238738185	0.913592950851954
**4930412M03Rik**	191.242745632419	-0.0854410780324626	0.307213346240696	-0.278116426509417	0.780922986459638	0.913592950851954
**5830417I10Rik**	2709.20483800014	-0.0520792445882947	0.187269893487564	-0.27809726175635	0.780937697669122	0.913592950851954
**Nim1k**	191.746709216835	-0.0698758225776481	0.251007673409608	-0.278381220894475	0.780719733550772	0.913592950851954
**Oxr1**	6114.37463496012	0.037244030950447	0.134202899336091	0.277520315393297	0.78138060877099	0.913808814621049
**Ankle2**	5913.41684830181	-0.0232564923558885	0.0838269055647846	-0.277434699506055	0.781446340557936	0.913808814621049
**F8**	31.7696204553683	0.233163890513783	0.839811919280604	0.277638224893872	0.78129008604753	0.913808814621049
**Gm5786**	76.8291386654018	-0.0867301063829259	0.312489101579621	-0.277546019827598	0.781360874439471	0.913808814621049
**Gm8034**	25.0077298783178	-0.125344215591862	0.451840772424988	-0.277407934921745	0.781466889447143	0.913808814621049
**Ppp2r2a**	11723.8510039364	-0.0107802734128468	0.0389316307825143	-0.276902693161487	0.781854824583531	0.913991132202886
**Slc25a20**	2819.33663535503	-0.0364600985065218	0.131640210069773	-0.276967793405959	0.781804836213088	0.913991132202886
**Gm26542**	37.9820758026846	-0.160358517255804	0.578810688784093	-0.27704829983819	0.781743019176286	0.913991132202886
**Gm50103**	31.2972023614342	-0.155259264346063	0.560506884034555	-0.276997961610211	0.78178167133119	0.913991132202886
**Ptprcap**	31627.3322795603	0.0230353038668924	0.0832747769107672	0.27661801954241	0.782073426823948	0.914178856562681
**Gm29055**	41.1418717793995	-0.11131582858846	0.403279735703719	-0.27602633788235	0.782527837069473	0.914642172591328
**Pdlim1**	12852.8634519182	-0.0217367465559342	0.0788159179857156	-0.275791326313978	0.782708346052404	0.914785299565919
**Gnal**	672.958127533305	-0.0475833408029994	0.172722049797064	-0.275490829682178	0.78293917020748	0.914805924365635
**Apex2**	1601.17866528722	-0.0247276138758012	0.0898126015472441	-0.275324547444422	0.783066906831707	0.914805924365635
**Zfp970**	218.939115901315	-0.0817701839908572	0.296679827369441	-0.275617606750973	0.782841785054717	0.914805924365635
**Gm42595**	126.483595059001	0.0957494178172604	0.347781488733627	0.275314877068103	0.783074335713885	0.914805924365635
**Gm43340**	66.8066172502857	-0.0936302635404339	0.339771886469123	-0.275568012743581	0.78287988081345	0.914805924365635
**Gm43372**	49.7423294655139	0.124318195206874	0.45150196658257	0.275343640577783	0.783052239349097	0.914805924365635
**Gm4925**	26.7515539315418	-0.182069906052584	0.662016074523205	-0.275023391514645	0.783298267182887	0.914999688338702
**Zwilch**	163.763069321809	-0.0854515022066053	0.310867394318281	-0.274880877726005	0.783407758808271	0.915059752078719
**Gm14388**	225.07128111785	-0.0560120438847364	0.204034648266284	-0.274522216499403	0.78368333288042	0.915313785204712
**Nphp3**	115.447231352781	0.100008727540062	0.364677991874672	0.274238450820558	0.783901380872041	0.915500597272514
**Hsd17b4**	4215.31450556374	-0.0219166205557482	0.0799662301538026	-0.274073449674882	0.784028176976696	0.915580818544564

**Gm44941**	43.5716433534455	-0.116791705880754	0.426297831443314	-0.273967393841374	0.784109679235708	0.915608138114499
**Pkig**	290.137418373732	0.056700572994651	0.207034032406221	0.273870785086188	0.784183923623068	0.915626579916515
**Gm48334**	33.379913161046	-0.166663025943843	0.608713268599444	-0.273795618628963	0.7842416908392	0.915626579916515
**Gpatch4**	3892.36711823233	-0.0219813813290907	0.0803155201714333	-0.273687841181524	0.784324522453913	0.915655441972758
**Cd8b1**	143123.219901288	0.0215545327406236	0.0788181214652259	0.273471789734716	0.784490574662229	0.915781447988794
**Ppil4**	9329.67426443249	-0.0204616928266224	0.0750453633309021	-0.272657655562268	0.785116387680645	0.916264410015196
**St3gal3**	962.469933582043	0.0512720878327532	0.188084638274876	0.272601145436565	0.785159831347773	0.916264410015196
**Gm10080**	132.100695084013	0.0633168610556718	0.232306848031613	0.272557014966065	0.78519375829	0.916264410015196
**Phf20-ps**	25.2133697519828	0.179599767325658	0.658947891882609	0.272555340927707	0.785195045277349	0.916264410015196
**Gm43312**	19.4942490545889	-0.172977570551378	0.634243895434415	-0.27273036728702	0.785060489586667	0.916264410015196
**Mtbp**	588.864082912355	-0.0495712625429108	0.182108298417058	-0.272207598301668	0.785462399762077	0.916508518442761
**Gm34432**	36.1100666019106	0.0882272839413862	0.324225755090498	0.272116827723202	0.785532190947561	0.916522083136669
**Rabl3**	664.028373092562	-0.0478171300133794	0.175820123628726	-0.271966194918356	0.785648012477791	0.916589347890756
**Top2b**	10606.7626789098	-0.0530762029926094	0.195521426876942	-0.271459777275535	0.786037431668297	0.916672761645895
**Vsir**	43549.8596863974	-0.020850619529982	0.0768209895063096	-0.271418262951031	0.786069357249688	0.916672761645895
**Nrn1**	79.4110286101956	-0.0978728905717146	0.360483991763175	-0.271504124477221	0.786003327929068	0.916672761645895
**Nxt2**	434.888548328865	0.0536717741513797	0.197561957632361	0.27167059283375	0.785875314645031	0.916672761645895
**Gm9905**	20.6769595928389	-0.177235743888737	0.653178157346419	-0.271343647816959	0.78612673910776	0.916672761645895
**Map6**	22.8519810493009	-0.223649949135817	0.824207653283688	-0.271351458876636	0.786120732053211	0.916672761645895
**Zfp964**	29.1556162837365	0.24764891850641	0.911889725829609	0.271577704509289	0.785946744560611	0.916672761645895
**Zfp987**	138.320786461723	-0.122774888624077	0.45296200054547	-0.271048980877486	0.786353360471422	0.916869165380213
**Rbm19**	2832.51557931823	0.0273826898434475	0.101110636034816	0.270819084097333	0.786530181210638	0.916939631942588
**Kmt5c**	3182.33861491239	-0.0330484672765314	0.122028528152852	-0.270825746870725	0.786525056510159	0.916939631942588
**Ppp1cc**	21091.7905284375	-0.0228557862543477	0.0844280101824955	-0.270713311908497	0.786611537577801	0.916954660374015
**Ctss**	18868.6678641589	-0.0222656388353551	0.0822899736037869	-0.270575355177055	0.786717652700153	0.916954660374015
**A730085K08Rik**	40.3958623900312	0.114814589419284	0.424285136234426	0.270607145087087	0.786693199825546	0.916954660374015
**Pten**	8981.65981638753	0.0400260480635342	0.148110741364782	0.270244059915641	0.786972498318697	0.916980378318991
**Dlg4**	319.415953606222	0.0603235559225154	0.223123875097167	0.27035903663938	0.78688405101918	0.916980378318991
**Gm16301**	18.5507446706169	-0.15260275863009	0.56451472087444	-0.270325561030023	0.78690980227158	0.916980378318991
**Gm45221**	34.301057448934	-0.182285196244308	0.674348966499148	-0.270312857733931	0.786919574396184	0.916980378318991
**Reep1**	31.9619848006538	-0.180912317803716	0.669843217881338	-0.27008158472654	0.787097489099213	0.917058202879052
**Hyi**	409.0691084596	-0.0488077917199979	0.180947106615269	-0.269735132177456	0.787364030410955	0.917165301041282
**Marco**	46.0315777114323	0.131874715666743	0.488720544561216	0.269836652324782	0.787285923877547	0.917165301041282
**Trav6-1**	79.2141185547455	0.0912211540762875	0.33815941996594	0.269757838138814	0.787346560944743	0.917165301041282
**Arpc5**	45027.5682196517	0.0185429324505661	0.0688298821614942	0.269402356480274	0.787620072968251	0.917237431268728
**Phf23**	3204.62811991823	-0.0242020824903592	0.0898750477651703	-0.269285892938778	0.78770968716861	0.917237431268728
**B3galt2**	68.1538785915947	-0.147495222931986	0.547900537583243	-0.269200726800851	0.787775221003979	0.917237431268728
**Trafd1**	4642.2545465591	-0.0185709333519929	0.0689293027026664	-0.269420008963395	0.787606490309126	0.917237431268728
**Gm17251**	398.332380765594	-0.0299667382250351	0.111199361452629	-0.269486603462206	0.787555249919611	0.917237431268728
**Gm27029**	214.500438423361	-0.125941611348758	0.467812808059948	-0.269213687994235	0.787765247498191	0.917237431268728
**Bcor**	3143.64751557009	0.0347736024519946	0.129370749364957	0.268790299373606	0.788091058702686	0.917537373428271
**Gm9887**	202.222705643319	-0.0616970641948906	0.230046438832638	-0.268193954698756	0.78855002805436	0.917881166545225
**Gstm1**	325.488897965233	0.0428622199305818	0.159806244798768	0.268213673280134	0.788534850716079	0.917881166545225
**Gm21986**	32.1308805620828	0.162306002498038	0.605213914894018	0.268179561810736	0.78856110627167	0.917881166545225
**Fbxo44**	88.8759858506212	-0.102944834510898	0.384168953440071	-0.267967605370165	0.788724254262331	0.918003255607294
**Rxra**	330.5238172795	0.0725526026150061	0.270871131486132	0.267849151059203	0.788815435442864	0.918041570003191
**Nle1**	1079.91475253108	0.0256225041337512	0.0958123146803846	0.267423913295739	0.789142789536314	0.918162647732058
**Zfp560**	662.687468141376	0.0462511165082861	0.172958763724031	0.267411234403151	0.789152550500429	0.918162647732058
**Cradd**	867.062447214549	0.0384985519332152	0.143960941625209	0.267423590722566	0.789143037871879	0.918162647732058
**2810047C21Rik1**	18.1253081756479	-0.178345871796269	0.666396952569971	-0.267627081889368	0.788986382808379	0.918162647732058
**Card10**	51.8206092531153	-0.212266001506502	0.794272087154425	-0.267245953797734	0.789279796348405	0.918242893610589
**Fam71e1**	53.6779066540961	-0.130841634457585	0.489878807236548	-0.267089803691804	0.789400017992208	0.918277996878212
**Mir6924**	23.2979471752231	-0.114392531592844	0.428347607320196	-0.267055376609897	0.789426524447056	0.918277996878212
**Atp5pb-ps**	131.50557380106	0.0553826582459977	0.207463706732549	0.266951068783293	0.789506835694987	0.918303625160251
**Afdn**	159.747417858933	-0.0823612611271883	0.30877336098444	-0.266736938914036	0.789671710838427	0.91835981605145
**Gm15443**	63.0025680332784	0.086784970935445	0.325349396555833	0.266743912403575	0.789666341261233	0.91835981605145

**Trav13-5**	44.39689726183	-0.133741795808389	0.501562176927975	-0.266650481157782	0.789738284019325	0.918369456921956
**Galnt3**	36.2804707052572	0.229594410196877	0.861593231158811	0.266476571418837	0.789872200591766	0.918457402754369
**Fundc2**	1335.55676116784	-0.0274992574817114	0.103347175914163	-0.266086201567342	0.790172821605235	0.918565082277458
**Tmem150a**	72.9487641974256	0.10656136286781	0.400291542936398	0.266209378509756	0.790077960554076	0.918565082277458
**Ms4a6c**	651.983585350232	-0.0437689334036371	0.164511759521667	-0.266053524264158	0.790197987578279	0.918565082277458
**Xlr4a**	2595.86722163281	-0.0245628386342676	0.0922795633441664	-0.266178531238362	0.790101716368905	0.918565082277458
**Chm**	2716.40478449314	-0.0200697250907217	0.0755321802376179	-0.265710919869968	0.7904618527809	0.918736255386048
**AC147806.2**	138.394603017684	0.179264088738258	0.67448034022939	0.265781043636188	0.790407843281094	0.918736255386048
**Rhoc**	145.301381922592	0.070202559564073	0.264433434355604	0.265482917223191	0.790637467942881	0.918824289047731
**Ly6a**	7528.17029716675	0.126087551874753	0.474975500826255	0.265461169376978	0.790654219407133	0.918824289047731
**Fgfr1**	437.496238944913	0.0535003252527199	0.201602573326708	0.265375210097243	0.790720431222839	0.918833468641466
**Sema7a**	18.4234869154461	0.384540235662664	1.44995582242781	0.265208242702725	0.790849045438474	0.918915154411476
**1700034P13Rik**	20.0028674660885	-0.120900850440577	0.456039774737794	-0.265110319620895	0.790924477787561	0.918935038821605
**Zkscan1**	1825.87945898846	-0.0331467773678228	0.125072308063463	-0.265020913750179	0.79099335084786	0.91894729995648
**Prr12**	440.462753784363	-0.0502073944628707	0.18952848127266	-0.264906857933617	0.791081215155342	0.918981620996957
**Tax1bp3**	1611.68737225981	0.0360220447864876	0.136058777154917	0.26475355386645	0.791199319019702	0.919045787111263
**Gm11944**	39.2725085383955	0.144144192896925	0.544590279795777	0.264683741602916	0.791253103252611	0.919045787111263
**Snapin**	6435.35103224992	0.027214491738767	0.102945333686424	0.264358672357735	0.791503553711115	0.919268924288792
**Snrpa**	8172.00300942624	-0.0170018706523103	0.0643753799671016	-0.264105169724807	0.791698880422601	0.919360251958061
**Gm42858**	32.7189456010009	0.172981607331025	0.654889985706514	0.264138421882276	0.791673258505113	0.919360251958061
**Gm10074**	65.8813440703123	-0.105490875521104	0.400062606177351	-0.263685917884409	0.792021947509798	0.91966763647103
**Slc8a1**	22.5389165627106	0.246589285340004	0.936600346767405	0.263281223620177	0.792333830698921	0.919908440511942
**Phf2os1**	23.455354793628	-0.127386168555497	0.48386992469783	-0.263265315849188	0.792346090920103	0.919908440511942
**Gm19585**	3139.25083648686	-0.0349381059479074	0.132755119802575	-0.263177088762115	0.792414089034268	0.91991960516562
**Pomc**	78.4323143842967	-0.13955271363398	0.531591065462921	-0.262518922345797	0.792921398856023	0.920440730939616
**Ube3b**	13044.0893503545	0.0277888079904622	0.105895363610567	0.262417607749631	0.792999499190636	0.920463580754833
**Slc25a26**	487.019193740238	0.032262770344705	0.123062192655386	0.262166386349471	0.793193167068411	0.920620560805609
**Gm13410**	36.5685872984063	0.108529380462023	0.414345250780165	0.261929828464726	0.79337554242711	0.92070447428582
**Gm12091**	61.5835922222344	-0.0743305986798342	0.283872256231053	-0.261845238653171	0.793440760063917	0.92070447428582
**Gm48990**	35.6743835225879	0.087982571728926	0.33591833296478	0.261916552610871	0.79338577784097	0.92070447428582
**Tap2**	44803.4932459512	0.0131244787139291	0.0502713361987555	0.261072804232603	0.794036363653095	0.920920873110689
**Gpd2**	2777.7864000812	-0.0335001382053461	0.128142776477801	-0.261428221911124	0.793762295616516	0.920920873110689
**Otud6b**	1742.83347237439	0.0298083056781013	0.114094696417964	0.261259345210089	0.79389251586404	0.920920873110689
**Smim26**	166.491293109577	0.0567146610108983	0.217012746104283	0.26134253415532	0.79382836843418	0.920920873110689
**Ywhaq-ps3**	893.408190555843	-0.0579550458292096	0.221811299065323	-0.261280854823098	0.793875929555527	0.920920873110689
**Gm10182**	193.812318755354	-0.0977503086846685	0.374392565858334	-0.261090410437413	0.794022786638896	0.920920873110689
**AC124742**	44.9704191963204	-0.0889239145960083	0.340565211405664	-0.261106864758675	0.794010097955418	0.920920873110689
**Pisd-ps1**	7720.26021888829	-0.0316664786019848	0.121436146510831	-0.260766497553184	0.794272581847863	0.921127038505687
**Gm11427**	310.67886743067	-0.0653446444536536	0.25075352889975	-0.260593120026551	0.794406295837958	0.921214307000199
**B230118H07Rik**	34.9080718167298	0.132519133251124	0.509253818421637	0.260222169098014	0.794692404694905	0.921258669174515
**Tfdp2**	1290.73957257096	-0.0305689610947493	0.117483688307715	-0.260197492393009	0.794711438445506	0.921258669174515
**Ormdl3**	4886.01760829847	0.019693742643222	0.0757193309530213	0.260088703840248	0.794795351193651	0.921258669174515
**H2-K2**	9957.95143993948	-0.0297631164049365	0.114404264560479	-0.26015740339122	0.794742360340913	0.921258669174515
**Gm15899**	184.005936225816	0.0456779352744597	0.175514117872879	0.260252199811888	0.794669241430221	0.921258669174515
**Gm45715**	30.8531646283375	-0.139274690888728	0.535151294642142	-0.260252927131312	0.794668680437067	0.921258669174515
**Gm17399**	45.5629662000969	0.11880455494803	0.456996677276849	0.259968093544929	0.794888385276741	0.921298733858449
**Iglc2**	218.371680433686	-0.0997846710552316	0.383972293046101	-0.259874665079678	0.794960454360183	0.921314495392278
**Gm7162**	29.8677697678994	-0.121159002101439	0.466421519676619	-0.259762890411749	0.795046677682316	0.921346657368556
**Eya2**	4952.74324671187	-0.0369331853322578	0.142265038310999	-0.259608304125431	0.795165930143648	0.921417087900681
**Cltc**	9312.72494934143	-0.0302328461781967	0.116522731223367	-0.259458784228479	0.795281278794701	0.921482984775946
**Osgepl1**	1029.56627458608	-0.0317252087256638	0.122330395824822	-0.259340358639029	0.79537264260068	0.92152108304845
**Wdr86**	53.8670820095725	-0.134994419594495	0.521402274338799	-0.258906464812958	0.7957074100242	0.921841163131484
**Zfp422**	3591.08542425633	0.0249373962629158	0.0964083002336085	0.258664411699922	0.795894180576603	0.921921973340111
**Gm30420**	30.2390355794669	-0.292394416190639	1.13034309803927	-0.258677579133128	0.79588402015533	0.921921973340111
**Col1a1**	30.5553458630864	0.204679244093187	0.792358664293042	0.258316408107692	0.79616272404622	0.922040738592386
**Atp2b4**	305.125668307894	0.0681426577313109	0.263807931733562	0.258304052056073	0.796172259268135	0.922040738592386

**Myl4**	47.3775289280731	-0.15107906226513	0.584574104084622	-0.258442960797422	0.796065064505369	0.922040738592386
**Chchd1**	1439.57214529437	0.0396563619666212	0.153655561726524	0.258086082410746	0.796340472449321	0.92216776838274
**Faf2**	6576.22908091469	0.0146838218018809	0.0570018587885087	0.257602508303486	0.79671369368673	0.92221628432947
**Pigo**	4599.27696996829	0.0269269108480057	0.104551597979001	0.257546621653874	0.796756829856215	0.92221628432947
**Sppl2b**	4151.44174661864	-0.0229073478770255	0.0888569002528443	-0.257800438816143	0.796560925806913	0.92221628432947
**Dcdc2b**	261.077591196949	-0.0758174053263007	0.294310196840299	-0.257610528416184	0.796707503405231	0.92221628432947
**Gm9200**	24.700546954428	0.138921240682774	0.538994950772698	0.257741265448994	0.796606596522811	0.92221628432947
**Ighv9-3**	22.0831364633779	0.232233736078119	0.901875161001338	0.257500978095764	0.796792060345333	0.92221628432947
**Gm19409**	68.6825208232093	-0.0906126901193487	0.351462391417429	-0.257816177013741	0.796548778994154	0.92221628432947
**Neurl1a**	17.5149351516368	-0.335240613196557	1.30465572317566	-0.256957147576487	0.797211853832583	0.92249887499743
**Treml1**	117.18395072444	-0.0859667889021434	0.334464047500125	-0.257028489443582	0.797156780294725	0.92249887499743
**Rps28**	1599.95464850426	0.0472521496264314	0.183848441952133	0.257016861958144	0.797165756256131	0.92249887499743
**Fdps**	4268.87969739356	0.0167675493524929	0.0653142528057657	0.2567211386825	0.797394052006646	0.92264195017394
**Tcf3**	11693.6447043977	0.0171677656497107	0.0670524562605336	0.256034851027754	0.797923926753815	0.922867943219773
**Cbx2**	851.285624704825	-0.0307887876641301	0.120121769591601	-0.256313137650303	0.797709053640006	0.922867943219773
**Fbxw7**	1906.77457508573	-0.0284420521376996	0.111008964183479	-0.256214012507043	0.797785589260683	0.922867943219773
**Syp**	98.2421454877886	-0.114987247760098	0.449146082979828	-0.25601302586727	0.797940779236288	0.922867943219773
**Cdc26**	2543.29655230241	0.0313496586298325	0.122327388838747	0.256276692631426	0.797737193016618	0.922867943219773
**A230020J21Rik**	17.8352006490485	-0.16400963310004	0.640358906998984	-0.256121420827368	0.797857082077659	0.922867943219773
**Gmpr**	35.4693713057458	0.14243773148794	0.557283932608993	0.255592747526562	0.798265319072296	0.923001487924704
**Mrpl54**	2359.33796897981	0.0202682350754665	0.0793090844500038	0.25556006876165	0.798290555146149	0.923001487924704
**Trbv26**	603.408548898549	0.0348543122733485	0.136337244617689	0.255647767938141	0.798222830207994	0.923001487924704
**D130020L05Rik**	57.8264191878274	-0.099051589158821	0.387485957333385	-0.255626267956851	0.798239433243987	0.923001487924704
**Rpl14-ps1**	2973.80160065044	0.0235205287772139	0.0921158928715837	0.255336273079427	0.798463386323411	0.923133581209039
**Epb42**	19.4820979013117	-0.333053193478463	1.30533122034856	-0.255148416192428	0.798608470594323	0.923233578221185
**Orc4**	4275.14620196834	-0.016598341874927	0.0651264028141182	-0.254863483283447	0.798828541187391	0.923420242351238
**Gm44822**	53.1189387169976	-0.151950358314714	0.596719005722443	-0.25464306793907	0.798998792001214	0.923549293248469
**Cabyr**	52.4612075984367	-0.10003396014637	0.393375978777278	-0.254296056554606	0.79926684610804	0.923791366106094
**Aldoc**	191.019567671769	-0.0841973358892622	0.331705092788317	-0.253831905870056	0.79962542318534	0.923799236977154
**Zscan30**	123.901641525281	-0.0608161768106974	0.239569370197655	-0.253856228617713	0.799606631729835	0.923799236977154
**Hjurp**	6115.44722118542	-0.0336852735960535	0.132589750644621	-0.254056391480363	0.799451992772783	0.923799236977154
**Xrcc1**	4988.83678366273	0.0182477366660736	0.0718484714026712	0.25397529425233	0.799514644760227	0.923799236977154
**Rn7sk**	38.5319351794943	-0.143613173543655	0.565708954181081	-0.253864062928877	0.799600579062149	0.923799236977154
**Ifi208**	29274.8406360116	0.0258009202962524	0.101549151722395	0.254073223248427	0.79943898948488	0.923799236977154
**Zdhhc7**	6748.31396879009	-0.0255657606663116	0.100774955617092	-0.253691609286858	0.799733816863208	0.923856726709206
**Kynu**	64.8406819164576	-0.106063989946465	0.418616338997777	-0.253368012821469	0.799983843440923	0.923880453053215
**Aktip**	1929.01891682074	-0.0304088528665014	0.119921200996086	-0.253573618458791	0.79982498001596	0.923880453053215
**Kri1**	1254.12423511032	0.0373516178171976	0.147528676858247	0.253182083732011	0.800127510693906	0.923880453053215
**Zranb3**	396.471033498915	-0.0438045930485379	0.173049136225668	-0.253133843970268	0.800164786622912	0.923880453053215
**Hist1h2an**	65.2785377976761	0.104539693237513	0.412970678431397	0.253140716029987	0.800159476402831	0.923880453053215
**Phf11c**	3456.97750362317	-0.0197280807211804	0.0778924574458327	-0.253273312565591	0.800057017404591	0.923880453053215
**Gm5469**	20.2590343243833	0.12228094763036	0.48260049443095	0.253379242336967	0.799975166620299	0.923880453053215
**Ddx27**	8160.44636653199	0.0177132192476698	0.0701668876261642	0.25244413493217	0.800697789763774	0.924292679363208
**Sacm1l**	5529.13983317213	0.0185346441286751	0.0734115243481363	0.252475946975016	0.800673203558238	0.924292679363208
**Gm27162**	241.561796779996	0.081666406071401	0.323389006045163	0.252533031565074	0.800629085746618	0.924292679363208
**Carmil1**	94.1371221597251	-0.124677405509258	0.494580878662049	-0.252086991002439	0.800973825006967	0.924543590992219
**Fam76a**	2499.11700829805	-0.0247876135447991	0.0983920338636687	-0.251927036889436	0.801097461018642	0.924573298691289
**C730045M19Rik**	63.4917477035527	-0.122490150743158	0.486261384162718	-0.251901867457707	0.801116916102822	0.924573298691289
**Eef1b2**	17667.5064097349	-0.0178908770920606	0.0710496907953126	-0.251807951474448	0.801189510938601	0.924589360177203
**Gm11942**	45.4918116288005	-0.0861644012550644	0.342409211041149	-0.251641598638857	0.801318101962832	0.924670035344441
**Racgap1**	20377.5764484384	0.0264174719849395	0.10549464074672	0.250415298805223	0.802266199701417	0.924883560776648
**Gm9752**	439.587768893575	-0.0670948084736348	0.267401497621464	-0.250914108823036	0.8018805160266	0.924883560776648
**Rgl1**	223.907045063591	0.0934209627808725	0.372702485203488	0.250658277016496	0.802078321088601	0.924883560776648
**Dock7**	17.3076092567444	0.168160323777252	0.670960930044203	0.250626103916622	0.802103197713146	0.924883560776648
**Plekha4**	38.1979681706358	-0.220282563532563	0.877483492326247	-0.251038983022443	0.80178396990297	0.924883560776648
**Ifit1bl2**	170.07877621438	-0.079372735335974	0.316172812994703	-0.251042253077288	0.801781441710248	0.924883560776648

**Gm14586**	235.046037517214	-0.0888658252722554	0.354542700364554	-0.250649146579186	0.802085380831239	0.924883560776648
**Gm12758**	341.668085904505	-0.0941884872462941	0.375934740380427	-0.250544781126054	0.802166078358992	0.924883560776648
**Gm13421**	54.5376124171702	-0.131426522143369	0.524801724514185	-0.250430812255871	0.80225420385779	0.924883560776648
**Trav4n-4**	157.554520348294	-0.0758648113926436	0.302892833733849	-0.250467501846893	0.802225833656368	0.924883560776648
**Gm4332**	644.397847107444	-0.035546514722222	0.141738772451534	-0.250788927457211	0.80197730267132	0.924883560776648
**AL731706**	32.5277661917201	-0.200640892250678	0.799632350218232	-0.250916427025395	0.801878723687634	0.924883560776648
**Gm49870**	39.7131452958358	-0.107568898905808	0.428412118589794	-0.25108743249349	0.801746512150357	0.924883560776648
**Ruvbl2**	6404.18555928538	0.0195605778397424	0.0781736742339894	0.250219502043536	0.802417604409342	0.924900532318825
**Atf6b**	5446.32375856932	0.0142210378262341	0.0568842567652787	0.249999536513492	0.802587707065084	0.924900532318825
**Atp5g3**	8838.73306620152	-0.0141661811825012	0.056752937831435	-0.249611416145133	0.802887869198296	0.924900532318825
**Akirin1**	9318.75215477345	0.0197010578332975	0.0788627973798869	0.249814341969082	0.802730928047754	0.924900532318825
**Bard1**	69.0242181025399	0.118617782789987	0.475065735199533	0.249687093808536	0.802829339790725	0.924900532318825
**Cd55b**	57.0670109034254	-0.103922412253001	0.416056802035282	-0.249779385277754	0.802757962696102	0.924900532318825
**Lzts1**	186.378730788622	0.100754614633575	0.40367852818065	0.249591215781698	0.802903492427136	0.924900532318825
**Sowahc**	28.6167699000562	-0.162256203231628	0.650165611569778	-0.249561343055152	0.802926596534188	0.924900532318825
**Gm12115**	62.7843906251609	0.0752335435219169	0.301073150365107	0.249884599243347	0.802676593507819	0.924900532318825
**Gm10416**	44.5441341243265	0.0866491894287358	0.346261639914021	0.250241954177343	0.802400242362717	0.924900532318825
**Gm9118**	497.601521687619	0.0318165817523865	0.12727686575776	0.249979299560547	0.802603357075455	0.924900532318825
**Arl2bp**	15114.5024461115	0.0223908748942892	0.0898077344821565	0.249320117286088	0.80311317121686	0.925047824478367
**Msh5**	146.931134651821	0.0607006149907159	0.243728946567833	0.249049675245784	0.803322356398206	0.925136683622507
**Dip2a**	199.252153872704	-0.0636399574560675	0.255626811857896	-0.248956504184878	0.803394426879412	0.925136683622507
**Pcolce**	25.4519410782537	0.130562082157895	0.52417633449735	0.249080459313554	0.803298544408585	0.925136683622507
**Gm10785**	110.525083223724	-0.0668111908937308	0.268407760557875	-0.248916762894136	0.803425168413428	0.925136683622507
**Itfg2**	2918.0492952245	0.0353136682459892	0.142022345952107	0.248648675736547	0.803632552873645	0.925307865226892
**Tctex1d4**	80.5948923621511	0.104998397498555	0.422570032778393	0.248475730302482	0.803766345788965	0.925394294212825
**Stx3**	139.864781558203	-0.0928255210029119	0.373960278706161	-0.248222943153408	0.803961915672277	0.925551830587278
**Gm41392**	18.8645806973294	-0.217495938714102	0.876662992553251	-0.248095266438306	0.804060697986398	0.92559792651751
**Taf9-ps**	61.0133616406721	0.066746950112647	0.269233906085303	0.247914354782266	0.80420067304823	0.925691431457445
**Pole**	991.380790971385	-0.0469572851480011	0.190021144842308	-0.247116104825962	0.804818370331649	0.925726188868953
**Exosc8**	9221.79830269542	-0.0218255787203503	0.0881198564601096	-0.247680597734863	0.804381544970248	0.925726188868953
**Ndc1**	1473.4827701071	-0.028413558287114	0.114870593777742	-0.247352758897461	0.804635231286489	0.925726188868953
**Hs6st1**	364.860404798361	0.0934277923230399	0.377715092184755	0.247349905407912	0.804637439447296	0.925726188868953
**Fcrl1**	163.5795643424	-0.0698016578530309	0.282244903049815	-0.247308833919709	0.804669222617214	0.925726188868953
**Igkv8-24**	39.3200552295437	0.275427110285855	1.11430633866524	0.247173600946911	0.804773874930367	0.925726188868953
**D330023K18Rik**	93.4647745513304	0.074461142184948	0.300883420752651	0.247475058608033	0.804540591638347	0.925726188868953
**Gm43062**	239.191856465222	-0.0501267839625596	0.202750663298501	-0.24723363735072	0.804727414316459	0.925726188868953
**4833413G10Rik**	27.9393534825577	-0.130514766499355	0.528123103968809	-0.247129439175346	0.804808051018716	0.925726188868953
**Gm49338**	636.943711959112	0.0476358310103512	0.192687641178258	0.247217884442742	0.80473960501615	0.925726188868953
**G430095P16Rik**	917.870704697953	-0.0440552009668809	0.178478810553787	-0.246837150192708	0.805034258186168	0.92589113753989
**Gm8546**	34.4735867764945	-0.0836599589732943	0.339007668554652	-0.246778957331484	0.805079296530687	0.92589113753989
**Rubcn**	2433.75668330314	-0.0242696493430025	0.0984131774277069	-0.24660975265056	0.805210256108028	0.925974164756546
**Lin37**	3616.2907910034	0.0291796650146832	0.118525358805953	0.246189214769267	0.805535764046309	0.926078143446497
**Gm7367**	165.64390936579	0.0628138317661957	0.255087360881294	0.246244390741988	0.805493054397079	0.926078143446497
**Cnot10**	6894.5398870411	0.0220232148867823	0.0893752172435482	0.246412994183487	0.80536254839855	0.926078143446497
**Gm17151**	17.6841261665734	0.131355654868262	0.533236886241995	0.246336399932862	0.80542183482706	0.926078143446497
**Gm18860**	308.095351371071	-0.0415057270698131	0.168846829678951	-0.245818811929918	0.805822493993738	0.926340193905255
**Ighj1**	246.007567967185	0.0469717199025568	0.191207425255569	0.24565845097163	0.805946637992536	0.926415317686634
**Gm4152**	45.8631356495408	0.0828913423865741	0.33754488553784	0.245571317884127	0.806014094428914	0.926425274339222
**Zfp592**	8051.36736962321	-0.0325158871531196	0.132601475135833	-0.245215123887659	0.806289866650889	0.926528845038982
**Cyb561**	41.5627291485095	0.214334279456717	0.875104956751171	0.244924083452154	0.806515213620116	0.926528845038982
**Ncoa4-ps**	1600.82194780944	0.0189096757656626	0.0772135273159073	0.244901073982757	0.806533030091816	0.926528845038982
**Akr1a1**	21799.9680359561	-0.0126906157896927	0.0517564282489111	-0.245198832667896	0.806302480201913	0.926528845038982
**Tti2**	3305.53956706347	0.0199341692196235	0.0813678920458433	0.244988148499563	0.806465607915933	0.926528845038982
**Vamp7-ps**	47.4556503132221	0.0733889828915294	0.299733594254641	0.244847372127334	0.806574612388127	0.926528845038982
**Gm13414**	136.404398396997	-0.0628856582386804	0.256432758279701	-0.245232546186976	0.806276377412134	0.926528845038982
**4921525O09Rik**	318.404501741416	-0.0534107107912731	0.217911856533725	-0.245102362215921	0.806377173920762	0.926528845038982

**Hsd17b11**	161.051220536581	-0.0619147957186878	0.253235460165578	-0.244494967956718	0.806847498730625	0.926774751239135
**Rsad2**	230.383386622809	-0.084147518423901	0.344440224020119	-0.244302240434572	0.806996748423735	0.926878618333172
**Rpl18-ps2**	64.9774141314128	-0.0774018604622694	0.316967463076789	-0.244194971026152	0.807079821732983	0.926906468735176
**Ap3m1-ps**	34.8687760734151	-0.117665586153664	0.482118688860722	-0.244059375569355	0.807184834872309	0.926959510464469
**Zfp235**	1258.85751512291	-0.0290494343680097	0.11934273987753	-0.243411827127651	0.807686382570264	0.927467885888329
**Lmna**	638.099814909202	-0.0684545155447744	0.281370749563384	-0.243289381184783	0.807781229899515	0.927479574510929
**Necap2**	9489.78688442029	-0.0181698132009323	0.074697056565866	-0.243246709258358	0.807814284482324	0.927479574510929
**Trav3d-3**	471.094648834737	0.0256938636054575	0.105784763348752	0.242888132393414	0.808092059306622	0.927730898250962
**Tcp11l2**	38313.7614703376	0.0204463181167199	0.0843257435096394	0.242468281520491	0.808417331343298	0.927982347448692
**Gm47879**	52.7049624048429	0.0996708910840993	0.411092997950904	0.242453390305623	0.808428868656991	0.927982347448692
**Loxl3**	52.8712935600036	0.119234847277464	0.492276445841838	0.242211156525194	0.808616550738271	0.928087333467068
**Med24**	5336.28831705018	-0.0189497144899803	0.07831634377125	-0.241963727843699	0.80880826918357	0.928087333467068
**Nudcd2**	1763.3966430712	-0.0316426320394193	0.130902224478262	-0.241727229354105	0.808991529172443	0.928087333467068
**Lbh**	1601.03914969297	0.031906948122728	0.131880231652991	0.241938823755504	0.808827566584344	0.928087333467068
**Gphn**	1279.94097388042	0.018848105694742	0.077939922396481	0.241828643334561	0.80891294334917	0.928087333467068
**Rpl41**	10600.214531238	0.0328516632405111	0.135696494581867	0.242096624100275	0.808705293987774	0.928087333467068
**Gm45094**	34.0567111608681	0.106359979183185	0.43953431708909	0.241983333377874	0.808793077548761	0.928087333467068
**Gm45897**	17.1377174658537	-0.147754975919816	0.61114251712972	-0.241768444803611	0.808959591035442	0.928087333467068
**Gfm1**	2762.92741917871	-0.0203276913692674	0.0841903792764871	-0.241449100763757	0.809207061257605	0.928241577490494
**Gm49179**	108.38202953785	-0.127648971899514	0.528782418768903	-0.241401694475212	0.809243799580308	0.928241577490494
**Kctd3**	1567.14688557329	0.0388879750133098	0.161154090537128	0.241309264218463	0.809315431214331	0.928256168994338
**Polr2f**	1485.09744957731	0.0336502065304043	0.139583268193541	0.241076218990274	0.809496043781289	0.928385206384117
**H2ac13**	35.9478879655625	-0.106717659703034	0.44278971506936	-0.241012056222484	0.809545772400696	0.928385206384117
**Tom1l2**	2888.18108300636	-0.0351077510106071	0.146283459942918	-0.23999809017579	0.810331737163939	0.928418581690722
**Alkbh7**	913.692777644178	0.0321961537487258	0.133877334877591	0.240489951328684	0.80995045239911	0.928418581690722
**Stx5a**	10070.3696221912	0.0136721355117959	0.0570057526536823	0.239837821190714	0.810455985452909	0.928418581690722
**Drap1**	3456.42210434407	0.0286255173368507	0.1192099776939	0.240126857588663	0.810231913883501	0.928418581690722
**Hhex**	125.021622752249	0.0982664631987795	0.409586003036737	0.239916555913083	0.81039494588113	0.928418581690722
**Aebp2**	2970.84562971612	-0.0392169559745131	0.163242751959944	-0.240237042708862	0.810146498448304	0.928418581690722
**Pnpla7**	12563.4819493945	-0.0137159615070881	0.0569635036909278	-0.240785074975515	0.809721697791184	0.928418581690722
**Zfp365**	408.015571606525	0.0392263780423338	0.163489019039521	0.239932799601981	0.810382353006106	0.928418581690722
**Ankle1**	77.7219924697906	-0.125232868341682	0.521616468140597	-0.240086109221395	0.810263502564413	0.928418581690722
**Fbxo28**	4667.16879675882	0.046003194108663	0.191238320586147	0.240554267406568	0.809900598692471	0.928418581690722
**Tcp11**	287.628848738856	0.053337162172078	0.221860382451985	0.240408682174796	0.810013448147845	0.928418581690722
**Nfyc-ps**	56.7933689346093	0.0916666639962564	0.381463562022335	0.240302542948753	0.810095723775389	0.928418581690722
**Trbj2-2**	353.356525263349	0.0303355180292099	0.126468914016145	0.239865410920958	0.810434596215743	0.928418581690722
**C030005K06Rik**	23.614665609344	-0.134749263254917	0.561843095447438	-0.239834331589687	0.81045869081398	0.928418581690722
**Gm4202**	735.358976620027	0.0349376602755246	0.145200315695057	0.240616971859063	0.809851994957228	0.928418581690722
**Fh1**	2778.12372975951	0.019407692639235	0.0809597548863978	0.239720249480347	0.81054713578116	0.928426988928596
**Flcn**	7796.0164651093	0.0174350988592032	0.0727454019758081	0.239672864341328	0.810583872989192	0.928426988928596
**Dolk**	1536.6127959555	-0.0456567427282223	0.190727452063321	-0.239382124777006	0.810809289495971	0.928617675140865
**Slc27a6**	72.5413415901192	0.108123518715707	0.452512725754893	0.23894028291764	0.81115188882684	0.928874971665389
**Cyb561d1**	2693.5412815545	-0.0242510319715677	0.101526389954712	-0.238864318748903	0.811210794255723	0.928874971665389
**Gm14719**	84.8525170024993	0.064461831786854	0.269853637693747	0.238877016214288	0.811200948096553	0.928874971665389
**Tex15**	30.0577104600598	0.211570972023882	0.891338855500666	0.237363120342198	0.812375097941822	0.929009048926797
**Zfpl1**	3865.74517178634	-0.0212356133745935	0.0894418515069004	-0.237423678253744	0.81232812221785	0.929009048926797
**Pfkfb4**	582.928789260239	0.0521123089492068	0.21884440194891	0.238124934817262	0.811784195714206	0.929009048926797
**Sgo2a**	90.4507353150155	0.111303839222503	0.467257566017942	0.238206606628236	0.811720853240717	0.929009048926797
**Ccdc34**	188.241290410903	-0.0685622122285816	0.288964037119212	-0.237269014207108	0.812448098890097	0.929009048926797
**Rab22a**	4671.80547319118	-0.0177506048585936	0.074490734090509	-0.238292790040517	0.811654013026149	0.929009048926797
**Rasal1**	829.401463615467	-0.0424216025586781	0.177859335203901	-0.23851209445962	0.811483935972792	0.929009048926797
**Gp9**	160.71799297575	0.103676675265215	0.436884193811867	0.237309284093396	0.812416860133322	0.929009048926797
**Gng12**	413.550034949045	0.0287680245500495	0.120673129532386	0.238396274808874	0.811573756424263	0.929009048926797
**Acaa2**	11774.0160704779	-0.0188809103628186	0.0793012323931131	-0.238091008084488	0.811810508743457	0.929009048926797
**Gemin8**	364.347430411861	0.0369976038350919	0.155754305819044	0.237538241017079	0.812239255928966	0.929009048926797
**Spin2c**	893.539563895528	0.0361197838977781	0.151490616257658	0.238429183206602	0.81154823505254	0.929009048926797

**Arfgap3**	812.166049664826	0.0261671034772722	0.110279810355007	0.237279184585432	0.812440209344368	0.929009048926797
**Armt1**	1775.07886555156	0.0165080184058586	0.0695590460890369	0.237323818166339	0.81240558561881	0.929009048926797
**Igkv6-23**	47.0713449022615	0.145751027079178	0.614191727641887	0.237305421938474	0.812419856128796	0.929009048926797
**Gm26770**	55.9254965856621	0.11506920970694	0.484204564801659	0.237645858944091	0.812155778949605	0.929009048926797
**Gm43604**	84.345296530585	0.059680682784146	0.250904119640454	0.237862506481235	0.811987736453566	0.929009048926797
**C030029H02Rik**	27.308317692081	-0.131295758125383	0.552682392076503	-0.237560957265324	0.812221635231712	0.929009048926797
**A430019L02Rik**	40.2678898906473	-0.16418755752119	0.69039385912878	-0.237817233380931	0.812022851783636	0.929009048926797
**Mindy3**	1627.2106280226	0.0302390385337144	0.127613254823272	0.236958445857303	0.812689027626074	0.929096276172643
**Afp**	1522.35966404098	0.0362172804374618	0.152860553473364	0.236930193006089	0.812710946141273	0.929096276172643
**Gm8226**	4073.6973934067	-0.0314755102572075	0.132810716320181	-0.236995260091258	0.812660467424495	0.929096276172643
**Gm45308**	28.9894187099239	-0.110441748648326	0.466261272003247	-0.236866656700489	0.812760238037933	0.929096276172643
**Gm9833**	104.678548905142	-0.0675629120104615	0.285470365601776	-0.236672243957924	0.812911069368687	0.929201285458281
**Dmtf1**	5752.67575673419	-0.022589191040587	0.0955362884711569	-0.236446186073126	0.81308646069155	0.929266944303819
**Paqr3**	104.395829767648	-0.0717706507749058	0.303479005565096	-0.236492968076208	0.813050163212315	0.929266944303819
**Mroh1**	3728.10025454838	-0.0297509456163741	0.125874772878821	-0.236353519739933	0.813158360343801	0.929281714819936
**Acap1**	51234.909652726	-0.0187258515120411	0.0794167191557186	-0.235792308107363	0.813593837134173	0.929620201533589
**Rbx1**	7665.5275388664	0.0241371409276303	0.102438286119138	0.235626169102031	0.813722765125683	0.929620201533589
**Bbs9**	4602.04935833222	0.0237055979673803	0.100544907663147	0.235771244097221	0.813610183049321	0.929620201533589
**Gm7432**	139.532406119556	0.062078318398913	0.263491651831238	0.235598805379508	0.81374400053769	0.929620201533589
**Gm49376**	78.233814974492	-0.0628070009300587	0.266592618885797	-0.235591672389714	0.813749536063514	0.929620201533589
**Ttc27**	3236.56211947531	-0.0160951150846814	0.068370237122723	-0.23541113446471	0.813889644834574	0.929712855854603
**Gadd45gip1**	4881.51693731218	-0.0250518465122423	0.106549377830785	-0.235119594522909	0.814115910714168	0.929903907584135
**H1f4**	378.422942530486	0.069215310153993	0.29470137993452	0.234865918067205	0.814312803137608	0.930061382939931
**Cog6**	3473.48248660497	-0.0244648905805133	0.104299286850258	-0.234564313135116	0.814546910784752	0.93019391746886
**Gm43966**	24.0912728389012	0.141321053816768	0.602423048504769	0.234587727291529	0.814528735975886	0.93019391746886
**Gm10284**	142.644431399909	0.0465591678814893	0.198597492899629	0.234439857229316	0.814643519070918	0.930236823682909
**Gm12960**	159.269299416511	0.0630867603098626	0.269330274024261	0.234235681593596	0.814802015511073	0.930350388290433
**Gm3362**	657.387289735029	-0.0358291298972285	0.153152642057126	-0.233943922977602	0.815028513582242	0.930541575865183
**Fam83f**	27.3555113879914	-0.123549115820407	0.528750771964531	-0.23366228925089	0.815247166163514	0.93058893054466
**Mettl22**	1793.09244114833	0.0310249228043694	0.132695534621235	0.233805326553954	0.815136114186077	0.93058893054466
**Mtmr12**	7654.89547791102	-0.0272767811148061	0.116730363166246	-0.233673402317433	0.815238538015371	0.93058893054466
**Cir1**	864.925249446485	0.0319916213571275	0.136993156118869	0.233527150286021	0.81535208940176	0.930641280166862
**Pdf**	526.435098149051	-0.0421246711057252	0.180571711997733	-0.23328499597021	0.815540108705896	0.930788461022657
**Dop1b**	5390.97857316753	-0.0343714030559835	0.147393351850035	-0.233195070364874	0.815609933622092	0.9307972034458
**Madd**	19598.575995771	-0.0346880967666604	0.148797416192736	-0.233122977899894	0.815665912621667	0.9307972034458
**Abhd5**	1279.51379313051	0.0258037644208779	0.110871017971488	0.232736786339544	0.815965802097589	0.930898555233411
**Ids**	1236.81958930271	0.0378346878063059	0.162549752591395	0.232757584697233	0.815949650854027	0.930898555233411
**Stbd1**	25.6930917547246	0.205088834262864	0.880882151431463	0.232822102172904	0.815899549440624	0.930898555233411
**Gm50464**	19.9780536364742	0.148507617764351	0.638181707246498	0.232704284811143	0.815991041751848	0.930898555233411
**Klc1**	4377.63524415105	0.0188620271776075	0.0811472035930297	0.232442109431208	0.816194645800036	0.931016112743534
**Tmem220**	19.5921990029559	-0.257642667339928	1.10925085095003	-0.232267270400799	0.816330431786804	0.931016112743534
**Cherp**	9168.41254574883	-0.0209132120826245	0.0900005005888131	-0.232367730688199	0.816252410208325	0.931016112743534
**1810014B01Rik**	458.179810884775	-0.0305391830914795	0.131458968396287	-0.232309620743548	0.816297540552076	0.931016112743534
**Fam3b**	32.690012418555	0.198105006621956	0.85395427423951	0.231985496879652	0.816549278369129	0.931064447361579
**Mrrf**	1067.19511271853	0.022145551625984	0.0954152515975698	0.232096559566668	0.816463016995337	0.931064447361579
**Wdfy1**	903.804696731009	0.022750197998798	0.0981321262856232	0.231832314858656	0.816668257091095	0.931064447361579
**Anp32b-ps1**	51.4714371910971	0.0654165876013687	0.282109119771287	0.231883987495348	0.81662812172406	0.931064447361579
**Gm7452**	34.2317650118759	0.0844999781150336	0.364378750192372	0.231901498291056	0.816614520781264	0.931064447361579
**AU040320**	3891.53852693674	-0.0187492586339931	0.0809514568647794	-0.231611132895504	0.816840059819754	0.931125574953329
**Gm38355**	25.4637924927827	0.135483350442808	0.58478933263274	0.231678901926712	0.816787419399851	0.931125574953329
**Pisd-ps2**	1185.04538973552	-0.0213595557359377	0.0923035124926448	-0.231405665495554	0.816999664172882	0.931205224958576
**Trmt10b**	1007.39503921191	-0.0267792819774745	0.115742729012863	-0.231369021673045	0.817028129406125	0.931205224958576
**Commd8**	7235.84255651031	0.0150808020885846	0.0652245145989355	0.231213711306496	0.817148778499555	0.931275372690401
**Pifo**	78.7632698166782	-0.0786239796192598	0.340436154561589	-0.230950733539189	0.817353076280806	0.931334414252513
**Nmb**	378.877516318528	0.0320705808510561	0.139102351516424	0.230553836807492	0.817661434284544	0.931334414252513
**2010300C02Rik**	163.531698024667	0.0785669784027572	0.340877522418817	0.230484479719453	0.817715322264911	0.931334414252513

**Cgref1**	21.3120139027138	0.205129112142448	0.888038827239954	0.230991152470207	0.817321675488546	0.931334414252513
**Lrrc28**	1562.83690125258	0.0230560386110912	0.0998699619177872	0.230860592798372	0.817423106172153	0.931334414252513
**Nono**	21990.9333316922	0.010832204155788	0.0469985605427087	0.230479487684405	0.817719200931092	0.931334414252513
**Gm8566**	114.008368262671	0.0527209048027772	0.22876149410284	0.230462320634592	0.817732539264043	0.931334414252513
**Birc2**	7716.54984629435	0.0169034010739321	0.0732519608722616	0.230756977323905	0.817503606317132	0.931334414252513
**Mrpl48-ps**	50.1208657333233	-0.0976298932923027	0.423366850875147	-0.230603537075448	0.817622819481311	0.931334414252513
**Trbv13-2**	3690.22035581889	0.0181961125601729	0.0790637978456872	0.230144681333006	0.817979345919669	0.931480852327544
**Traj19**	100.683260853798	-0.0562457396361396	0.244355552317474	-0.230179912437854	0.817951970359281	0.931480852327544
**Lrrc8b**	1423.47431001938	-0.0295206129879524	0.128365648869327	-0.229972841238887	0.818112873691967	0.931565583954641
**Spg20**	2477.78878776264	0.0225606031113096	0.0982916295743996	0.229527206019439	0.818459177631214	0.931800526692563
**Tmem11**	4445.04465633496	-0.0204601638433735	0.0891182339032972	-0.229584485096226	0.818414663971182	0.931800526692563
**Gpx4-ps2**	1917.29704708981	-0.0191687405097438	0.0835315529862851	-0.22947903905116	0.818496610392318	0.931800526692563
**Gm14508**	60.529063336183	-0.120369254740949	0.524894409379504	-0.229320893097797	0.818619515777874	0.931805801496204
**Gm10095**	345.974200497622	-0.0249882414479521	0.108936367462934	-0.229383832322613	0.818570601126564	0.931805801496204
**Hspb6**	279.498985485457	0.0514414241428311	0.224581507892412	0.229054585239826	0.818826490803122	0.931855652953602
**Rpl26**	7523.32867540385	-0.019912401030625	0.0869060275821045	-0.229125661184004	0.818771249194618	0.931855652953602
**AL935121**	51.0026832790538	0.602259273549036	2.62953673166897	0.229036265702507	0.818840729250211	0.931855652953602
**Snord37**	19.6757135475632	0.116132303637869	0.507412281711152	0.228871684473689	0.818968648976072	0.931881894083507
**Gm14227**	17.6735212441571	0.195828457476959	0.855690089727579	0.228854418004658	0.818982069509958	0.931881894083507
**Gm45737**	18.2878539654311	0.185089090304653	0.809953646514483	0.228518127057218	0.819243465546211	0.932104533862428
**D430020J02Rik**	34.1249151652119	0.150903788666309	0.660553593875368	0.228450484662386	0.819296045820564	0.932104533862428
**Septin9**	28739.6000290856	0.0211178327642335	0.0924796528370509	0.228351124992253	0.819373282269448	0.932125103510194
**Stard7**	13359.3606473114	0.01261762627171	0.0553026074347939	0.228156082632943	0.819524901991014	0.93216298857088
**Zfp398**	2591.67595974129	-0.0275229177431918	0.120621433359643	-0.228176012973829	0.81950940846883	0.93216298857088
**Atox1**	1341.69903780355	-0.0244468557284427	0.107334956147228	-0.227762292974803	0.819831042093897	0.932287935469268
**Map1lc3a**	677.835953267527	0.0375546030896088	0.164904216797591	0.227735856722843	0.819851595158837	0.932287935469268
**Gtf3c1**	5081.73393296104	0.0393294670451675	0.172662126841784	0.227782825131109	0.819815079298363	0.932287935469268
**Gm13552**	720.46960024681	-0.0298667211416807	0.131161011007871	-0.227710360816665	0.81987141726202	0.932287935469268
**Zfp14**	603.57035341555	0.0377599872604549	0.165881985596047	0.227631632963493	0.819932625917827	0.932290257366662
**Psmb5**	8926.39200757935	0.0191372683435295	0.0841773538521259	0.227344617854677	0.820155781248444	0.932296747948895
**Sumf2**	1740.19559558309	0.0235044971067677	0.103416428508744	0.22728010864135	0.820205939410188	0.932296747948895
**Mcm7**	8601.26425352355	-0.0148810286190339	0.0654814272575504	-0.227255715739122	0.82022490593348	0.932296747948895
**Exosc1**	4941.86846227188	0.0219095855728623	0.0964144476278178	0.22724380123442	0.820234170007967	0.932296747948895
**Rbm7**	12189.3767515621	-0.0167706091018061	0.0737418946792826	-0.227423083916472	0.820094772142135	0.932296747948895
**Cluh**	4944.75493490066	-0.0319890979381064	0.140935430762198	-0.226976976372124	0.820441645158682	0.932318111609
**Cnnm2**	519.551193158917	0.0322620911251901	0.14211564915817	0.22701293852082	0.820413681318537	0.932318111609
**Gm6123**	40.0008917234312	0.0934595743922744	0.411492848389094	0.227123204590672	0.820327940847554	0.932318111609
**Gm3375**	149.651207584696	-0.0474880577233915	0.209276620682031	-0.226915254884317	0.820489639758964	0.932318111609
**Smarce1**	6980.41807760036	0.0169878710387847	0.0748924271091209	0.226830291052429	0.820555708694731	0.932325951968768
**Nsfl1c**	7421.24091414037	0.0151206939075907	0.0668580898691614	0.226161021608325	0.821076185447627	0.932555229123498
**Psd**	242.994393707013	0.0449454596646507	0.198803067723746	0.226080312438167	0.821138956597474	0.932555229123498
**Rnu12**	19.4484472134215	0.109300462076301	0.482626857589693	0.226469912226111	0.820835957915494	0.932555229123498
**Zfp114**	34.3967810465598	0.2076148280194	0.917994225841244	0.226161365916156	0.821075917666403	0.932555229123498
**Gm12138**	175.0368162213	-0.0449570424369766	0.19862235103019	-0.226344327331738	0.820933624603013	0.932555229123498
**Gapdh-ps15**	946.805950615803	0.0233531594379917	0.103315144636465	0.226038104289205	0.821171784228503	0.932555229123498
**Gm49594**	1276.33613451723	-0.0255158071422044	0.112742725598963	-0.226318877840213	0.820953416872145	0.932555229123498
**Il17ra**	46927.7365393826	-0.0200814274212846	0.0890045785698087	-0.225622408913877	0.821495110424807	0.932587911734783
**Tarbp2**	5262.68212292567	-0.0147229347073674	0.0652253407406606	-0.225724151689856	0.82141597249664	0.932587911734783
**Ndufaf7**	1508.97239478477	-0.0279815188071703	0.124039476844192	-0.225585591934722	0.82152374798626	0.932587911734783
**Ccdc88a**	176.927079877687	0.0738808723300474	0.327499874075616	0.225590536602738	0.82151990183303	0.932587911734783
**Ppip5k1**	1153.3675290734	-0.0327009668085818	0.145035662609806	-0.225468455276122	0.821614862645317	0.932587911734783
**Kdf1**	43.3765031046351	0.143442396638381	0.636019515047245	0.225531439279384	0.821565870288052	0.932587911734783
**C030034L19Rik**	29.347821254822	-0.222235873770299	0.984594844449504	-0.225713017921146	0.821424632515703	0.932587911734783
**Odf2l**	260.067527140774	-0.0476197245211416	0.211385906508994	-0.2252738856037	0.821766213831912	0.932692518284912
**Sms-ps**	1076.39851332774	-0.0235910287207727	0.104799138808635	-0.225107085697053	0.821895968850356	0.932726759435836
**Gm28198**	17.1713093799801	0.115884940708629	0.514854450390955	0.225082915415477	0.8219147715153	0.932726759435836

**Ssbp1**	4208.89570899008	0.0279444890741489	0.124351966946043	0.224720925293238	0.822196384893257	0.932917773816266
**Fmnl2**	54.2820786613194	0.0791758298973174	0.352339903004494	0.224714343229831	0.822201505679158	0.932917773816266
**Gm16337**	16.9756562855468	-0.229376059167194	1.02139757139802	-0.224570789661501	0.822313190942783	0.932977314925506
**Gm48632**	18.7477786768663	0.203100187296474	0.905191708234896	0.224372567102404	0.822467414822927	0.933085107305412
**Gm7887**	215.367065540995	0.0415570633728085	0.185322067290792	0.22424239045208	0.822568700407911	0.933132830261156
**Adpgk**	7678.95460313857	-0.0179326420689593	0.0800588598217378	-0.223993223347033	0.82276257626168	0.933137101337393
**Rgs16**	67.7577622310427	0.115479957522883	0.516375918588267	0.2236354434161	0.823040982209825	0.933137101337393
**Pdss1**	718.031653454233	-0.0385589725025864	0.172358242387765	-0.223714119895919	0.822979758306212	0.933137101337393
**Slc6a6**	6930.4086767736	-0.0273424197035045	0.122134411451912	-0.22387154757175	0.822857255592574	0.933137101337393
**Cdca4**	6622.01263206487	0.0135199740230128	0.060438449718299	0.223698226642623	0.822992125918205	0.933137101337393
**Gm14440**	109.058579632401	-0.0546784130852413	0.244505347091894	-0.223628700703593	0.823046229256019	0.933137101337393
**Gm3739**	18.8189076832797	0.140329435820636	0.626502864311661	0.223988498400268	0.822766252827034	0.933137101337393
**Trav14n-2**	291.881746196239	-0.0443087864351058	0.198047546313977	-0.223728025212998	0.822968937677492	0.933137101337393
**2510009E07Rik**	193.496871694618	-0.0618335383702976	0.277042664030767	-0.223191393955953	0.823386549648149	0.93345577831541
**Gpr141**	41.5011524753086	-0.106076762659066	0.475543105625797	-0.223064452841711	0.823485343850578	0.933500616047019
**Gm42742**	3845.06023092549	-0.0264638067730655	0.118744144518078	-0.222864098945415	0.823641278560881	0.933610216983225
**Tma16**	777.737498997838	-0.032483451873568	0.145928420337068	-0.222598530146061	0.823847980523925	0.933643025684371
**Mphosph10**	2576.77148040252	0.0316251609374259	0.14203462347268	0.222658110847943	0.823801605608817	0.933643025684371
**Gm7609**	190.96796254945	0.0810722418555603	0.364201572501239	0.222602668348678	0.823844759514885	0.933643025684371
**Slc38a6**	206.255653114229	0.0636465606172235	0.286175369987701	0.222404047629811	0.82399936117039	0.933680277143811
**Ttc30a2**	25.1513505149101	-0.123976768205559	0.557373048245531	-0.222430504301933	0.823978767530074	0.933680277143811
**Mvb12b**	234.115718540476	0.0470042896791535	0.211887700697713	0.221835857033588	0.824441664944219	0.93411428162264
**Haus4**	2078.21101624161	-0.0211945422420308	0.0955973837586923	-0.221706299991748	0.824542525477377	0.934161387255322
**Gm21092**	1148.3782752649	-0.01990334861247	0.0898303593918347	-0.221565946604452	0.824651794266078	0.93421801152136
**Eya3**	1715.80357405036	-0.0171029939001452	0.077345022317907	-0.221125980542713	0.824994341382151	0.934365340603326
**Rps3**	51908.7464121488	-0.0123404776755838	0.0558346915951527	-0.221018104032211	0.825078336558652	0.934365340603326
**Ap1s2**	1214.34921967004	0.0324731292759437	0.146729492303746	0.221312898764215	0.824848807223158	0.934365340603326
**Pyurf**	1307.6626907681	-0.026387238407098	0.119329409135525	-0.221129381250261	0.824991693544214	0.934365340603326
**AK157302**	185.164404977403	0.039613325642308	0.179185081894042	0.221074908823786	0.825034106768424	0.934365340603326
**Fnta**	6579.81096969581	-0.0276270735605552	0.125149056570219	-0.220753350586019	0.825284488276782	0.934416624163882
**Prrg4**	520.526440914221	0.0599638292742172	0.271741608881067	0.220664879114857	0.825353379758005	0.934416624163882
**Leo1**	2043.41405494869	0.0342215325170514	0.15496590896157	0.220832651170639	0.825222739178459	0.934416624163882
**Gm5555**	264.720222623484	-0.0375665755010375	0.17025003804692	-0.220655313396667	0.825360828527823	0.934416624163882
**Yipf5**	2885.1235540712	-0.0250728870476931	0.113692392495698	-0.220532671512228	0.825456330451649	0.934457604635867
**Gm26457**	27.0580131410867	0.0991305542998251	0.45001234782072	0.22028407615899	0.825649920971602	0.934609612410168
**Gmppa**	6300.59565418583	0.0165763988709215	0.0753428733035673	0.22001283126186	0.825861161634478	0.934611256772972
**Rab3gap2**	3758.79951941309	-0.0160158124681445	0.072814429011128	-0.219953829009589	0.825907113200614	0.934611256772972
**Trp53bp1**	4930.96765226095	0.0205352129411586	0.093317199245547	0.220058179062189	0.825825844702563	0.934611256772972
**Arhgap30**	32888.0956075449	0.0206349045077226	0.0938442096597894	0.219884685294166	0.825960963802362	0.934611256772972
**Trim30b**	121.695139449382	-0.057044068613936	0.259497350356837	-0.219825244980322	0.826007257842255	0.934611256772972
**Gm37768**	51.765480996513	0.0832236237790678	0.378316438440741	0.219984159615374	0.825883491335695	0.934611256772972
**Prpf3**	3443.99873749487	-0.0169013871742427	0.0772701987447002	-0.218730991362979	0.826859605877207	0.934735045152677
**Pigt**	5353.942844768	-0.0137524190524975	0.0627953819837276	-0.219003669028739	0.826647189673226	0.934735045152677
**Irf1**	32386.9392456979	0.0267190008924855	0.122174867281443	0.218694740473386	0.826887846314855	0.934735045152677
**Adar**	11470.0661595139	-0.0229289936911043	0.104460156441782	-0.219499898067673	0.826260659258557	0.934735045152677
**R3hdm4**	9111.09569721198	-0.0210349088127783	0.0960709352924475	-0.218951847910572	0.826687557405943	0.934735045152677
**Pgrmc2**	808.425414869046	-0.0292635358569308	0.133609431362634	-0.219022980327681	0.826632146630738	0.934735045152677
**Mat2a**	24771.789645766	-0.0213446758326087	0.097207364006019	-0.219578794784385	0.826199207681577	0.934735045152677
**Trbj1-2**	728.388862974871	0.0201134192573198	0.091757587245748	0.219201701581924	0.826492930045124	0.934735045152677
**Hmgb1-ps2**	2142.70956664824	0.0266640377086717	0.121823823681115	0.218873754763003	0.82674839144916	0.934735045152677
**Trav10d**	140.178944980529	0.0513730638136939	0.234406741224683	0.219162057990695	0.826523810319197	0.934735045152677
**Gm27017**	28.0225042760505	-0.180241241480972	0.822207271563212	-0.219216306781488	0.826481553429889	0.934735045152677
**Gm3534**	30.3989984951153	-0.0837533978346482	0.382882580705301	-0.218744341098954	0.826849206123004	0.934735045152677
**Gm49380**	473.630709925343	-0.44741644064712	2.04203190351334	-0.219103550672903	0.826569384935027	0.934735045152677
**Vps18**	2416.88921091083	-0.0335495206667953	0.153557316608933	-0.218482071761103	0.827053525585258	0.934788207635529
**Rpl15-ps2**	160.95380895611	0.046142696549292	0.211148781095577	0.218531673779378	0.827014882511899	0.934788207635529

**Zfp78**	479.82599715606	0.0342976770926492	0.157076785005342	0.218349752265955	0.827156612793013	0.934837666603035
**G0s2**	529.68861665442	-0.0504752955021493	0.231295282390735	-0.218228815479599	0.827250834587927	0.934877099663054
**Acot8**	2543.4890778962	0.0250692759101355	0.115003036103286	0.217987948488772	0.827438501357488	0.93488802996486
**Gtf3c4**	1653.88210117853	-0.0263466712624492	0.120817756789115	-0.218069528541543	0.827374938762867	0.93488802996486
**2610017A05Rik**	221.462050989306	-0.0598159278220947	0.274368389968561	-0.218013189598658	0.82741883478172	0.93488802996486
**Pbk**	91.6108150157794	-0.086261956507715	0.396128563015738	-0.217762526011758	0.827614143765061	0.935003017519136
**Lcn2**	100.271084037604	0.136173822726153	0.626206212157129	0.21745843474958	0.827851096180914	0.935003017519136
**Nrf1**	10031.3443103286	0.0191084305158844	0.0878950960618273	0.217400416770045	0.827896306430601	0.935003017519136
**Ppih**	333.183045246329	0.068691778265725	0.315714356640535	0.217575719383379	0.82775970439952	0.935003017519136
**Rps24-ps3**	56.5514731570035	0.0837598165366236	0.385152136345343	0.217472028927086	0.827840503061692	0.935003017519136
**Gm8806**	132.495346456593	-0.0537096644338318	0.246850131423645	-0.217580052009999	0.827756328328723	0.935003017519136
**Kcnh6**	47.3066550775559	0.100408462406219	0.464024958771744	0.216385908792484	0.828686949247787	0.935004523680798
**Taz**	9982.88168343673	0.0185577527982085	0.0856214001147208	0.216741991760747	0.828409421080809	0.935004523680798
**Tbk1**	6990.83337617002	-0.0177326396524499	0.0825183372757466	-0.214893322355658	0.829850491264755	0.935004523680798
**Timm9**	1759.21207598109	-0.0203129572410431	0.0939580196139754	-0.216191841042398	0.828838213078051	0.935004523680798
**F12**	102.483857507019	0.0643002924470578	0.296563949244568	0.216817629421407	0.828350472460634	0.935004523680798
**Rhobtb2**	1631.44409557511	0.0313194947239376	0.145955793536661	0.214582059163488	0.83009318282482	0.935004523680798
**Pi16**	40.3468258371831	-0.147817804201239	0.688661191139547	-0.214645178359247	0.830043967548013	0.935004523680798
**Epb41l3**	60.2321107107896	-0.128459809060249	0.597987971146048	-0.214820055350035	0.829907616006505	0.935004523680798
**Pigf**	630.214340990809	-0.0271041292480919	0.1255290761507	-0.215919132676106	0.829050783137713	0.935004523680798
**Irf7**	9544.78742345627	-0.022211752252326	0.102888937365263	-0.215880859702854	0.829080617064568	0.935004523680798
**Farsb**	8330.62617374905	-0.0110193196907552	0.0509660951363689	-0.21620882787412	0.828824972637895	0.935004523680798
**Gzf1**	1025.55037411915	-0.0373835931440463	0.173489221379888	-0.215480782302825	0.829392493653475	0.935004523680798
**Rpl28**	2895.04159228812	0.0174086270657336	0.0811274870475265	0.214583585653715	0.830091992582538	0.935004523680798
**Mki67**	523.007218480515	0.0719261856142117	0.331079437439189	0.217247516700347	0.828015455853726	0.935004523680798
**Stoml1**	3874.92575106883	-0.0257972195957804	0.118963402260153	-0.21685004888618	0.828325206471271	0.935004523680798
**Arhgap39**	2639.1869852004	0.034977979633027	0.162784746939501	0.214872586594532	0.829866658409903	0.935004523680798
**Hk1**	14023.7282606705	-0.0174777040925305	0.0814105139847818	-0.214686079684961	0.830012076336929	0.935004523680798
**Nudt22**	793.423334317744	-0.0299094825675498	0.13871332677705	-0.215620829393145	0.829283318195186	0.935004523680798
**Nod1**	11239.2114580665	0.0184438967144209	0.0858622001914063	0.214808107331344	0.829916931710403	0.935004523680798
**Eme1**	180.070848421313	-0.0632104688293267	0.293853908472907	-0.215108484205016	0.829682739300711	0.935004523680798
**Dennd6a**	3667.95835624541	-0.0211192547939997	0.0978940347194125	-0.215735870469866	0.829193638953318	0.935004523680798
**Rpl21**	3277.05282907501	-0.0220178393803727	0.102449707336721	-0.214913638630579	0.829834651252193	0.935004523680798
**Insig1**	953.33997456955	0.0277360708193315	0.128970563185017	0.215057375375977	0.829722585840709	0.935004523680798
**Thtpa**	2193.49029459617	0.0215182174037222	0.0998601211952075	0.215483589907309	0.829390304917966	0.935004523680798
**Bdh1**	4169.62382814587	0.0194670802700844	0.090445669294375	0.215235073408817	0.82958404705022	0.935004523680798
**Urgcp**	6393.97727710958	-0.0194766955625299	0.0903923338095872	-0.215468444520503	0.82940211188371	0.935004523680798
**Psma3**	12072.1490578685	0.017585444125268	0.0813953084362513	0.216049849347778	0.828948890858837	0.935004523680798
**Ankrd23**	1972.10946586952	0.0392281409505148	0.181249577193231	0.216431627361501	0.82865131536997	0.935004523680798
**Sf3b4**	10757.3490915571	-0.015831224693575	0.073109213636771	-0.21654212794887	0.828565190673996	0.935004523680798
**Zfp788**	732.841167226139	-0.0335857212309237	0.155103938785713	-0.216536868720818	0.828569289695256	0.935004523680798
**Gm11131**	2006.28690327868	-0.0232952919323964	0.107750270417183	-0.21619706235727	0.828834143302348	0.935004523680798
**H3f3a-ps2**	56.6389298112159	0.0841496352108385	0.389428967695768	0.216084683450098	0.828921738525797	0.935004523680798
**Gm11460**	20.0595395311431	0.118952765906247	0.549779717366445	0.216364413143604	0.828703703466318	0.935004523680798
**4833418N02Rik**	81.1246767862837	0.0944606137796011	0.438492700506864	0.215421177297619	0.829438960479323	0.935004523680798
**Gm9403**	101.76687511125	-0.103230529625564	0.477799745475529	-0.216053965292141	0.828945682569961	0.935004523680798
**Gm45191**	119.494603240644	0.0857944435932204	0.39813504097943	0.215490812821103	0.829384674128121	0.935004523680798
**Gm9891**	24.9538377508127	-0.118838338437064	0.548184312604995	-0.216785368906927	0.828375614748971	0.935004523680798
**Ift22**	390.576021425153	0.036953534119713	0.172350542839746	0.214409154220495	0.830228003618793	0.93508953917236
**Mboat7**	7082.21125969348	0.010973387643891	0.0512047099725645	0.214304263216616	0.830309793701694	0.935114818051433
**Trav9d-1**	111.988650934607	0.0448055372095245	0.209346228461129	0.21402600629055	0.830526776954201	0.935292340227797
**Lyrm7**	824.856071615985	-0.0346005056029116	0.161901985541404	-0.213712669966379	0.830771130400688	0.935366966897009
**Dgkh**	627.629492078656	0.0354570888850418	0.16585267482089	0.213786656882881	0.830713430672578	0.935366966897009
**Zfp750**	38.4254241796776	0.111230994338299	0.520370961342992	0.21375326949688	0.830739468177946	0.935366966897009
**Wdr1**	62096.6426991803	0.014571719525651	0.0684269695488721	0.212952869631959	0.831363724021276	0.935701242207945
**Tomm40l**	3613.7135803708	-0.0183057205173485	0.0859620007887626	-0.212951308128946	0.831364941988401	0.935701242207945

**Cdk16**	5789.87077050182	-0.0339852228343602	0.159412974943844	-0.213189816238811	0.831178911155877	0.935701242207945
**Fbxl12**	6020.05277202268	-0.0294934526771763	0.138403882095858	-0.213097004437702	0.831251301099474	0.935701242207945
**2610306M01Rik**	309.276714499562	0.0336612728041571	0.157900980218549	0.213179631675287	0.831186854687602	0.935701242207945
**Tbc1d22b**	5552.5351905338	-0.0187988565738076	0.088331026649502	-0.212822801759133	0.831465177914797	0.935747218656057
**Dcaf8**	18414.5373578162	-0.0164432022748775	0.0773253004901353	-0.212649704180267	0.831600199649689	0.935765503526398
**Gm11478**	75.6705141745253	0.074408688366638	0.349891898806426	0.21266193535908	0.831590658769629	0.935765503526398
**Tubb3**	707.704645082814	0.0491458534768789	0.2312299197243	0.212541065340837	0.831684944012548	0.935794034761905
**Gm5617**	252.579547902194	-0.0514656398579276	0.242484147057913	-0.212243317686397	0.831917213841788	0.93598854255659
**8430430B14Rik**	18.021416860128	-0.118977986019391	0.561203911112526	-0.212004912409697	0.8321032018861	0.936130954742202
**Per2**	762.814289625771	-0.0337796615234989	0.159497723366804	-0.211787734711513	0.832272637758886	0.936254726434409
**Usp49**	140.297469052336	0.0782339568445966	0.369994390112085	0.211446332526546	0.832539005800781	0.936487515305748
**Gm10335**	695.325221068453	0.0432330629752752	0.204584981238626	0.211320805239601	0.832636949253683	0.936530830850902
**Med31**	683.664850554215	-0.0356402638157537	0.169111635312988	-0.21074992119727	0.833082417863081	0.936538487872553
**Dpy19l1**	1290.9167734196	-0.0260064364484807	0.123480044966775	-0.210612463378016	0.833189686087196	0.936538487872553
**Napepld**	334.150166573278	0.0331166815373463	0.157270474584405	0.210571511435053	0.833221644434593	0.936538487872553
**Tmem81**	323.363770059164	-0.0541191110407989	0.256900605048641	-0.210661672169094	0.833151284570808	0.936538487872553
**Fam174a**	1641.65495180522	-0.0255001563942626	0.120779532863645	-0.211129781591813	0.83278600163765	0.936538487872553
**B4galt3**	3218.9014566321	0.0181970509251085	0.0863158631680751	0.210819312432467	0.833028268070912	0.936538487872553
**Rps12-ps3**	948.161571107442	0.0270073090429692	0.128085379715591	0.210853956188739	0.833001233941519	0.936538487872553
**Faap20**	4847.62145722136	0.0105220911547232	0.0498372229474262	0.211129162750963	0.832786484518024	0.936538487872553
**Rpl38-ps2**	194.354587565602	-0.0873203679111252	0.414417054338615	-0.210706502053791	0.833116300608345	0.936538487872553
**Zfp963**	677.498311080624	0.0323386139412844	0.153590846056168	0.210550399139401	0.83323812029481	0.936538487872553
**Glis2**	18.581143501671	-0.21234134677713	1.01096370216868	-0.210038546707091	0.83363758807819	0.936867132119098
**Tnfrsf10b**	370.163950912879	0.0412535847072825	0.196535197682219	0.209904308204305	0.833742359677464	0.936867132119098
**Mkrn1**	15551.9053385016	0.0134298356017666	0.0639934884540441	0.20986253330151	0.833774965108468	0.936867132119098
**Fbxw17**	1154.98414001	-0.02653595867965	0.126472501871093	-0.209816033422797	0.833811258732855	0.936867132119098
**Pitpnm2os2**	17.8285849167471	0.21279118499105	1.01428226566828	0.209794839359483	0.833827801030541	0.936867132119098
**Smarcc1**	7793.25502349959	0.0235331954187253	0.1122455820518	0.209658099575491	0.833934530337062	0.936903205699949
**Gm49902**	55.6855025807302	-0.106785083523188	0.509467537617182	-0.2096013497202	0.833978826118391	0.936903205699949
**Pde8b**	38.3582667909221	-0.142413661746118	0.680191840651184	-0.209372787550315	0.834157234371502	0.936970027230664
**Dnah17**	205.598286752006	-0.0611998972522535	0.29227087512502	-0.209394443514343	0.834140330058623	0.936970027230664
**Ciao2b**	2098.42374654451	-0.0212450113246846	0.101541322377547	-0.209225277229425	0.834272380700949	0.937032568444283
**Rab39**	40.1013804885306	-0.105940548879457	0.506610631535715	-0.209116315933431	0.834357438003104	0.937061307955446
**Trdv2-1**	28.2393340927867	-0.122538522189644	0.586537003934224	-0.208918655375042	0.834511740616924	0.937167806777911
**Gm45769**	30.6452498199972	-0.195535908763807	0.937022064281673	-0.208678019672574	0.834699600137777	0.93731197258915
**Gm11767**	70.4172600618308	0.0742924975524623	0.356189472351352	0.208575781485138	0.834779418319295	0.937334803282059
**Mgll**	31.638808460731	0.125130474663153	0.601579033437678	0.208003383941266	0.835226325139026	0.937538266589472
**Gm20400**	19.9279623291817	0.0901689078242395	0.433257144763069	0.20811868636015	0.835136296992938	0.937538266589472
**Vkorc1**	1457.59941079552	-0.0195764544380311	0.0941344662866233	-0.207962664582642	0.835258119336359	0.937538266589472
**Dleu2**	883.78274875263	-0.0421164385734428	0.202369833435075	-0.208116189347731	0.83513824663773	0.937538266589472
**Gm49359**	1375.12377723757	-0.0309674959766124	0.148840682327243	-0.208058008686946	0.835183673860119	0.937538266589472
**Eno2**	54.9136051872186	0.0715268770791137	0.344476564668986	0.207639312554818	0.83551060629967	0.937621296450673
**Kif3b**	2699.93237146278	0.0358637667967456	0.172690576436406	0.207676455408396	0.835481602723607	0.937621296450673
**Spata2l**	1994.07498321982	-0.0243251699613275	0.117093323218631	-0.2077417336248	0.835430629753148	0.937621296450673
**Usp33**	5027.46366442639	-0.0153945091339624	0.0741826120332578	-0.207521799408475	0.83560236974454	0.93765749466349
**Esd**	4105.86421727724	0.0169030767473114	0.081642397873402	0.207037975213834	0.835980201032825	0.937761132020623
**Tsc22d4**	9714.80730940219	0.013754955910736	0.0663852310955908	0.207199036347854	0.835854419867241	0.937761132020623
**Borcs6**	420.225148670675	-0.0281151520008469	0.135692556841507	-0.207197451763594	0.835855657332532	0.937761132020623
**Wdcp**	923.75650077979	0.0353305930552429	0.170660654183847	0.207022486959311	0.835992296851792	0.937761132020623
**Gm29724**	22.361116235585	-0.145678834734674	0.703323433077871	-0.207129220900769	0.835908941930451	0.937761132020623
**Zcchc12**	104.026943286336	-0.075405384545228	0.36461296299485	-0.206809390225364	0.836158722350708	0.937814291983779
**Ccdc28a**	1305.68629609312	0.0323743588376246	0.15653494061229	0.206818737791011	0.836151421881877	0.937814291983779
**Gm43188**	20.5152976377605	0.262349701439694	1.27027331423824	0.206530121115723	0.836376838631327	0.937992159879987
**Tbp**	6020.01437148847	-0.0184912070522845	0.0895869824563214	-0.206405066286277	0.83647451371424	0.938034937911556
**Pfdn6**	3443.47785511148	-0.0236343292301185	0.114629334642964	-0.206180462477012	0.836649948660113	0.938081125786601
**Chpf2**	6028.59909161929	0.0247913051609171	0.120311059438699	0.206060068597008	0.836743990008865	0.938081125786601

**Usf2**	1074.92844218052	-0.0201890604624456	0.0979630873259028	-0.206088446307136	0.836721823571159	0.938081125786601
**Gm28530**	42.1112245210075	-0.0821736297307087	0.398809231593579	-0.206047461344753	0.836753837845445	0.938081125786601
**Npepps**	2687.81688222435	-0.0293868534819261	0.142841039578852	-0.205731164996904	0.837000913114319	0.938154782726554
**Zscan12**	1108.47082730645	0.0281041457680406	0.136776466404894	0.205475009749448	0.837201020875234	0.938154782726554
**Sestd1**	29.7998775553388	0.130372653527515	0.634431633195443	0.205495197127651	0.837185250169507	0.938154782726554
**Tmem62**	1735.74363179549	0.0278575482105899	0.135606145038827	0.20542983655065	0.837236311144304	0.938154782726554
**Rpl35a**	3539.85435954554	0.0188149581296294	0.0915122146092718	0.205600511472303	0.837102977964485	0.938154782726554
**Fastkd5**	1545.17773681734	0.0363206507554972	0.176589404429938	0.205678539280127	0.837042023316401	0.938154782726554
**Gm7962**	98.7197771537918	0.0605488489543946	0.294594606751258	0.205532781547217	0.83715588878853	0.938154782726554
**Plekha5**	2120.92636335525	0.0194574211332913	0.0948186437899306	0.205206701504811	0.837410633817316	0.938191545708933
**Pex11a**	40.4749408095259	-0.191830937023547	0.93529327749021	-0.20510244395032	0.837492087029418	0.938191545708933
**Jarid2**	6720.6059739786	-0.0288093250469178	0.140476422638486	-0.205082991905753	0.837507284504782	0.938191545708933
**Gm15672**	162.494433379362	0.0354642882764333	0.172781844492579	0.205254715161676	0.837373122813918	0.938191545708933
**Vps9d1**	1310.99176739939	-0.0296293947495028	0.144871363914262	-0.204522094283851	0.837945528135689	0.938615745136422
**Cd33**	46.8717957373664	0.105733567858675	0.518001479544802	0.204118273854331	0.838261074482199	0.938616467369752
**Ifi44**	159.400531701279	-0.0622713207990429	0.305151910598631	-0.204066625953225	0.838301434165196	0.938616467369752
**Dusp11**	5611.75921533449	-0.0222858105271515	0.109243207521857	-0.20400179592578	0.838352095477587	0.938616467369752
**1700030J22Rik**	19.3260721684376	-0.247393853846558	1.21074558774051	-0.204331823589994	0.838094202913017	0.938616467369752
**Cerk**	3601.74703517307	-0.0178415402926374	0.087461559865995	-0.203992934953064	0.838359019921385	0.938616467369752
**Cdpf1**	2988.06300536222	0.0240251981553403	0.117777709792937	0.203987649255352	0.838363150457694	0.938616467369752
**Gm34106**	68.5983315109534	-0.0898593291596811	0.439771713992011	-0.20433176191344	0.838094251107124	0.938616467369752
**Gm9044**	113.254039597712	0.0373428574935547	0.183223548694564	0.203810360401903	0.838501696344969	0.938704883076922
**Ykt6**	2371.89347771863	-0.0231502651072554	0.113652755478296	-0.203692950600536	0.838593451316316	0.938740907387837
**Podnl1**	1548.41183394395	-0.0324091516450236	0.159367939916021	-0.203360548314182	0.838853233367643	0.938965006618879
**Acyp1**	1113.32486476798	-0.0268863798354492	0.132376054782549	-0.203106066876028	0.839052129880762	0.939120927015994
**6530402F18Rik**	922.960151422052	-0.035632208928105	0.175805426233488	-0.202679801707495	0.839385311412789	0.939427114989085
**Traj35**	24.9936399949786	0.0984619620778386	0.486176701676985	0.202522995730175	0.83950788287328	0.939497564661525
**Ccdc116**	113.524451770781	-0.0586185814701015	0.289575668183074	-0.202429236675514	0.839581173930678	0.939512858293139
**Phf6**	3523.88392047842	-0.0176461822264218	0.0872246228328209	-0.202307349155792	0.839676454970063	0.939534718627273
**Gm36756**	71.9865457770799	0.0604623520333143	0.298946084592429	0.202251694032876	0.839719962075768	0.939534718627273
**Cebpz**	3685.65905420232	-0.0138873066463302	0.0687200414284998	-0.202085248461023	0.839850079975578	0.939563247992752
**Srbd1**	1336.52342598421	0.0260020701239084	0.128680740722656	0.202066525090575	0.839864717140187	0.939563247992752
**Zswim7**	300.472924034344	0.0311819955688993	0.15470002452008	0.201564257443617	0.84025739007957	0.939678773248916
**4930404N11Rik**	32.4430011579024	-0.177891267918679	0.882823291092437	-0.201502689964772	0.840305526283142	0.939678773248916
**Tor1b**	3007.67863167687	-0.0232723015001014	0.115420892788047	-0.201629886391864	0.840206079095672	0.939678773248916
**Smad1**	5051.99497659623	0.0170156863799976	0.0844868178366137	0.20140048845139	0.840385433307338	0.939678773248916
**Myo5c**	19.1034835938383	-0.239424992490105	1.18849542860164	-0.201452177878216	0.840345019333434	0.939678773248916
**Zfp658**	868.98643928008	-0.0402745357048468	0.199629508183278	-0.201746405485661	0.84011498210709	0.939678773248916
**Gm43024**	24.7054472535518	0.14193106850131	0.703486577362587	0.201753769110162	0.840109225148173	0.939678773248916
**Dpp7**	1451.85511616929	0.0376645286189563	0.18765786968562	0.200708495103643	0.840926516756271	0.939750292519757
**Orc6**	1016.71636930569	0.0247565470967333	0.123340333071941	0.200717368602317	0.840919577913837	0.939750292519757
**Spsb1**	1101.60333725419	0.0253697928030861	0.126354704917393	0.200782335882721	0.84086877558685	0.939750292519757
**Cstf2t**	3582.70199807389	-0.0151056328417953	0.0752012664703548	-0.20086939423753	0.840800699788108	0.939750292519757
**B2m**	240356.805616654	-0.0164120914679078	0.0816439185313526	-0.201020379265667	0.840682638969553	0.939750292519757
**Ighv1-55**	38.7906835037456	-0.157410043757739	0.783780197131279	-0.200834423137861	0.840828045504627	0.939750292519757
**Gm20379**	35.7554625400965	0.109200725551673	0.54384722099882	0.200793019317294	0.840860421547531	0.939750292519757
**Gm47467**	110.698527020719	-0.058678225999019	0.291605894629294	-0.201224416514673	0.840523100336472	0.939750292519757
**Ldhd**	143.442996194995	0.0670812519842865	0.334353399485399	0.200629788982349	0.84098806341264	0.939752422891495
**Septin4**	235.117037673572	-0.0690359211985547	0.344557571878208	-0.200361062513399	0.841198209607563	0.93989634179108
**Gm43863**	104.430836541704	0.0589877816180892	0.294478726348594	0.200312539888744	0.841236155884978	0.93989634179108
**Arf5**	8206.60178897473	-0.0186226625141577	0.0931048413924814	-0.200018197073709	0.841466349482241	0.939953584913631
**Syk**	232.950739741488	-0.0553822373605515	0.2768080881203	-0.200074491091036	0.841422323161987	0.939953584913631
**Tyms**	823.772666928434	-0.0305645793516387	0.152746198213141	-0.200100426126411	0.841402040104651	0.939953584913631
**Gart**	14976.4652047075	0.0114546540180597	0.0573676421128999	0.199670992151235	0.841737901814152	0.940190268581951
**Gm48986**	36.5176492246877	0.131354103402788	0.658197842893056	0.199566292750872	0.841819791924957	0.940215087989902
**Hddc3**	144.766229527106	0.0470892655327878	0.236679189307326	0.198958200214395	0.842295442253159	0.940327479068849

**Terf2ip**	3687.11623872369	0.014729234396095	0.074052128273604	0.198903593177959	0.842338158726712	0.940327479068849
**Ncoa5**	5172.06641356027	-0.0217608441747498	0.109304493874971	-0.199084625007652	0.842196547963638	0.940327479068849
**Zc2hc1a**	48.6353099836256	0.0942014555899634	0.472556114536197	0.199344485643615	0.841993282892401	0.940327479068849
**Arxes2**	100.561300962177	-0.0453407853479924	0.227796725296945	-0.199040549370885	0.842231025323268	0.940327479068849
**Rptoros**	28.8441508385736	-0.125100665430422	0.628830550275725	-0.198941774339	0.842308291381275	0.940327479068849
**Gm43696**	534.10631959095	-0.0487614057828478	0.244760047937335	-0.199221262594833	0.84208966763648	0.940327479068849
**Gm50107**	39.7980774842923	0.108956511100068	0.549086971304745	0.198432155185115	0.842706961407043	0.940672541151231
**Abhd17a**	11725.9265536864	-0.0150944306618494	0.0765173793600341	-0.197268003531932	0.843617816819979	0.940680927544279
**Lrrc8a**	6243.40058232485	0.0208319182778691	0.105666479637911	0.197147840538024	0.843711846668399	0.940680927544279
**Mlkl**	70.7653532885477	-0.0833261960465844	0.4204670335708	-0.198175336931745	0.842907883072149	0.940680927544279
**Tctn2**	18.2347438981193	0.29910426560222	1.51065737430146	0.197996098050048	0.843048116604497	0.940680927544279
**Baz1a**	7115.82591615772	0.0218769264682868	0.110582104353759	0.197834239058257	0.843174756675277	0.940680927544279
**Nepro**	719.612480863286	-0.027996224336079	0.141473517280472	-0.197890212064045	0.843130962384905	0.940680927544279
**Galnt6**	30986.5557795258	0.0147946278532859	0.0750166818017838	0.197217838725227	0.843657071472842	0.940680927544279
**Armh3**	1830.39559682377	-0.0247637519910908	0.124853307200159	-0.198342779590058	0.842776883209677	0.940680927544279
**Mrps7**	4679.18249623803	0.0234092102402189	0.118462916028551	0.197607918368116	0.843351839012988	0.940680927544279
**Fkrp**	4218.47367201109	-0.0133901230219374	0.0677559139041726	-0.197622941679675	0.843340083929117	0.940680927544279
**Rab26**	50.2879406571472	0.0749739010547306	0.379819966011374	0.197393259343521	0.843519804116611	0.940680927544279
**4930404I05Rik**	44.5561564624352	-0.112210207186185	0.569176152135179	-0.197144955503222	0.843714104290878	0.940680927544279
**Gm21811**	2582.79345311945	0.0125372126192923	0.0633614107464534	0.197868268266013	0.843148131550899	0.940680927544279
**Anxa11os**	47.4707224915385	0.113187192594544	0.574188608629283	0.197125458244021	0.843729361489071	0.940680927544279
**Mir6538**	29.445505879081	0.0833692974063412	0.422192256937457	0.197467613478026	0.843461623134205	0.940680927544279
**Gm7292**	335.122832060076	-0.0348744153724931	0.176861865012816	-0.197184482759842	0.843683173190607	0.940680927544279
**Gm37254**	289.002698254858	-0.0380665451338462	0.192646158638143	-0.197598256840141	0.843359398754276	0.940680927544279
**Btf3**	8257.79649896627	-0.011843363279211	0.0601501017905142	-0.196896811919921	0.843908288564864	0.940813846251348
**Parp6**	3169.36625959403	0.0192554756700925	0.0980541666278212	0.196375904587303	0.844315954425269	0.940848753821062
**Ube2l6**	451.177033355726	0.0429440144435977	0.218758211174314	0.196308125821062	0.844369001648293	0.940848753821062
**Ip6k2**	1161.06860065145	0.0328472333051734	0.167047224432933	0.196634415307877	0.844113636839194	0.940848753821062
**Sorl1**	11998.9467067763	-0.01871242396121	0.0953089800227982	-0.196334321873279	0.844348499157999	0.940848753821062
**Dbp**	961.449855706302	0.0307466587625582	0.156259258668568	0.196766956560143	0.844009910404114	0.940848753821062
**Gm5913**	99.5551699350383	0.0573956705974068	0.29246731907045	0.196246441413788	0.844417279719328	0.940848753821062
**Hmgb1-ps1**	144.654730608317	-0.0339610498906621	0.172822885343682	-0.19650782836501	0.844212705971369	0.940848753821062
**Gm11769**	156.35285549417	0.0684385293419035	0.34819621997815	0.196551614908967	0.844178437585439	0.940848753821062
**Urod**	4997.51630775952	0.0126369798613697	0.0644529837348889	0.196065086968645	0.844559222423007	0.940873845285586
**Pgap3**	1139.57099160154	0.0280514842552271	0.143045401393346	0.196101964704836	0.844530358505484	0.940873845285586
**Afmid**	1905.81197490375	-0.0509669144523637	0.260223689788512	-0.195858088453766	0.844721242424311	0.94098781314103
**Gm38380**	111.402543440189	-0.0742986532625677	0.379825891586649	-0.19561239743873	0.84491355599342	0.941135508397308
**Snx9**	1772.28737028464	0.0208708538913038	0.106892409326662	0.195251038149236	0.845196425190342	0.941384044088798
**Paip2b**	1733.92658207627	-0.0287576459239044	0.147527158100076	-0.194931199748296	0.845446808815434	0.941596364610177
**Sh2d4b**	21.3142511569495	0.1629922450081	0.837145465744712	0.194700027268384	0.845627790562595	0.941625308006892
**Gm24119**	30.852663263611	0.074363706598959	0.381826987314925	0.194757597208877	0.845582719087863	0.941625308006892
**Gm42466**	118.315545942169	-0.0631987638122937	0.324647276083631	-0.194669009931916	0.845652074227678	0.941625308006892
**Hsf2**	398.04903525521	-0.0399318247964341	0.205243494147191	-0.194558297510746	0.845738752864404	0.941655280446891
**Cavin1**	27.8668521713437	0.129366225943385	0.665469998155178	0.194398284373473	0.84586403316897	0.941667835654083
**Sap30bp**	6053.19265823645	-0.0151397525982272	0.0778828975763367	-0.194391234396332	0.845869552950935	0.941667835654083
**Wdtc1**	2469.96449978621	-0.0156392809493493	0.0806652637643393	-0.193878755483139	0.846270818732178	0.941954489681449
**Stat2**	4928.12100793568	0.0151731960865742	0.0782641936079531	0.193871493298467	0.846276505235723	0.941954489681449
**Trav8-1**	258.116945320313	0.0277608299418142	0.143220102302267	0.193833334116916	0.846306385125359	0.941954489681449
**Rbm28**	2781.98480466527	-0.0176709850519426	0.0912244932787127	-0.193708777290255	0.846403918740233	0.941996507104806
**Cnn2**	88552.0478947853	-0.0128829744417773	0.0665362824797601	-0.193623297870545	0.846470854344163	0.941997473425946
**Snx30**	2349.50862727796	-0.0264832750957457	0.136825594271176	-0.193554979511058	0.846524352586875	0.941997473425946
**Tsen15**	860.51970192862	0.027630568647536	0.143114481300774	0.193066197050086	0.846907125413567	0.942077573529134
**Ppdpf**	6131.08301255491	0.0155110664960724	0.080388379148103	0.192951601468362	0.846996872151349	0.942077573529134
**Sart3**	8238.620788494	0.0189568706580891	0.0982201844350565	0.193003818585002	0.846955977519857	0.942077573529134
**Drg1**	11698.2660371562	0.0173232079632997	0.0897423654111803	0.193032665050987	0.846933386146676	0.942077573529134
**Arl16**	2195.04156380829	-0.0232131716581543	0.12019879827573	-0.193123159225805	0.846862515623929	0.942077573529134

**Gm20775**	256.35063600154	0.0257393364352071	0.133273733735917	0.193131352395438	0.846856099203386	0.942077573529134
**Gm18541**	39.88167284946	0.090164687202457	0.467347325932583	0.19292864685282	0.847014849539077	0.942077573529134
**Gm48914**	19.9493068299759	0.160715162687146	0.834614979050829	0.192562039648414	0.847301976429643	0.94233040955688
**Ocel1**	1379.33809172954	-0.0213181753063277	0.110767206484033	-0.192459266447247	0.847382472076058	0.942333915399823
**9130221H12Rik**	106.825043076496	-0.0635106310176156	0.330087732877092	-0.192405305292771	0.847424737019452	0.942333915399823
**Cd302**	60.8317700871685	-0.069576388975414	0.361834553629835	-0.192287851664361	0.847516733805992	0.942369711000001
**Lipe**	1597.39493829239	0.0245536077614628	0.127874832398358	0.192012824579687	0.847732159781765	0.942399923198366
**Cd6**	58920.1777191766	0.0111142774713043	0.0578657054113952	0.192070197577088	0.8476872191448	0.942399923198366
**Kcmf1**	1820.43727469807	-0.0279776566008796	0.145640430926023	-0.192100891373294	0.847663176700634	0.942399923198366
**Gm45311**	40.3747782068252	0.0863494092534842	0.449858943005615	0.191947744056311	0.847783138372586	0.942399923198366
**Traj56**	19.0910277909235	-0.276351606365372	1.44150421607902	-0.191710577938554	0.847968919685692	0.942451953574634
**Traj28**	31.0686560992501	-0.078452434473498	0.409333575539147	-0.191658928467242	0.848009379817298	0.942451953574634
**Gm15725**	76.6760780944304	0.0804826269525132	0.419688046541341	0.191767737050823	0.84792414398857	0.942451953574634
**Il17rb**	28.5299351121687	0.161175696042781	0.843652071800039	0.191045220453133	0.848490164772725	0.942655678215717
**Mapkapk5**	1041.20313075859	-0.030160903926658	0.157874851935666	-0.191043117740808	0.848491812155456	0.942655678215717
**Usp11**	7462.59357651826	0.0129120098715961	0.0675748308251488	0.191077206023737	0.848465105564941	0.942655678215717
**Htra2**	3415.09530065819	0.0161304297609255	0.0844193629122007	0.191074999910882	0.848466833946899	0.942655678215717
**Trbv1**	1840.60548679265	-0.0192749951134761	0.100827137241687	-0.191168723428824	0.848393406778144	0.942655678215717
**Prkcsh**	4948.85325731716	-0.0163450009456948	0.0856020363296974	-0.190941730436667	0.84857124543923	0.942677461533132
**Car5b**	117.986059748032	0.113934120222252	0.597976114521831	0.190532894969156	0.848891568827743	0.942901353864308
**AC154542**	17.1201670856795	0.173156913174123	0.908808713740178	0.190531748382452	0.848892467215782	0.942901353864308
**2410002F23Rik**	12091.3450087829	0.016816437902544	0.0885460669940076	0.18991738959656	0.849373865537073	0.943294073728695
**Odf3b**	26.3698724661162	-0.105157498504834	0.554224260890756	-0.189738172659247	0.849514306662543	0.943294073728695
**Mfsd4a**	238.580069589406	0.205123522711175	1.08098492951667	0.189756135455921	0.84950023012477	0.943294073728695
**Gm8741**	232.757691224872	0.0330251467150093	0.174167285131942	0.189617393932453	0.849608956104646	0.943294073728695
**Gm4353**	42.1767994977966	-0.0850737093243922	0.449010336306856	-0.189469378420394	0.849724952878409	0.943294073728695
**Gm29125**	75.4489192377826	0.27188959372253	1.4349408612151	0.189477908861203	0.849718267655503	0.943294073728695
**Gm43482**	23.027997548038	-0.143021182820395	0.754089155792169	-0.189660840129907	0.849574908813223	0.943294073728695
**Gm20735**	292.850840137228	-0.0271412178412272	0.143023228719284	-0.189767900531025	0.849491010454804	0.943294073728695
**Adgre1**	235.549573381912	0.070467378491064	0.372467203688612	0.189190827523101	0.849943256422429	0.943310439441719
**Actr10**	6551.17410328503	0.0123534395235943	0.0653739280030319	0.188965844656319	0.850119586483965	0.943310439441719
**Padi2**	13001.7208842709	0.0147412676691049	0.0779754947011378	0.189050005076656	0.850053624973792	0.943310439441719
**Nfe2l3**	23.9352224850284	-0.0980731661875412	0.518697001464254	-0.189076023016686	0.850033233384282	0.943310439441719
**Tvp23a**	693.279061200958	-0.0347402091438856	0.183744853484934	-0.189067658141153	0.850039789353868	0.943310439441719
**BC025920**	548.249656792468	-0.0415464636509535	0.220009410082499	-0.188839484799193	0.850218624163946	0.943310439441719
**Gm11766**	34.5878999672598	0.113926168877685	0.602962240787525	0.188944118173779	0.850136614986586	0.943310439441719
**Gm10851**	61.404916047779	-0.0885011659282394	0.468565170492067	-0.188876962056952	0.850189250184715	0.943310439441719
**Dntt**	566.424847866702	0.0475242419816358	0.252128486858963	0.188492155621511	0.850490864214469	0.943479621756363
**Gm10552**	39.3730422098922	-0.074562004000836	0.395469216743561	-0.188540601503214	0.850452890782043	0.943479621756363
**Sema4b**	1293.97939010515	-0.0325978838579676	0.173196578421639	-0.188213209262192	0.85070951804147	0.943655746270992
**Nup205**	2645.20310161397	0.0266145806005099	0.141579966237416	0.187982673734219	0.850890233404289	0.943789765433717
**3300002I08Rik**	405.249020908683	-0.0398428989965015	0.212464600358015	-0.187527234792827	0.851247272160916	0.943920004745923
**Scyl2**	1483.01639979898	-0.0202126628788878	0.107779141180686	-0.18753779866368	0.851238990328965	0.943920004745923
**Gm49364**	164.034068459223	-0.084322781927313	0.449597589930382	-0.187551676912614	0.851228110125711	0.943920004745923
**Gm4013**	85.2213861227168	-0.0460636550008774	0.245612597873585	-0.187545978503049	0.851232577534174	0.943920004745923
**Atad2**	3800.39740181287	-0.0271900907865278	0.145199904761868	-0.187259701245123	0.851457017780051	0.94398294568154
**Mtmr3**	10717.1280495015	-0.0259043910609679	0.138328103800926	-0.187267737713285	0.851450717056348	0.94398294568154
**Gm21960**	29.0447005341059	-0.0877489338347388	0.468680231501612	-0.187225592070736	0.851483760041361	0.94398294568154
**Abcg1**	18481.6736894916	0.0212770930450436	0.113699572383387	0.187134327764213	0.851555313886297	0.943995854854818
**9130230N09Rik**	79.7875588506071	-0.0541928639997489	0.289759418063257	-0.18702710117922	0.851639384178618	0.94402263640538
**Il13ra1**	110.442645354386	0.0763432392817455	0.408436185638579	0.186915953987732	0.851726530176011	0.944052823296525
**Taco1**	562.142516349119	-0.0247396898616242	0.13245857541521	-0.186773032882726	0.851838591468832	0.944110620086824
**Gtpbp2**	6180.89324063373	-0.0164017522803002	0.0879045778103397	-0.186585871735692	0.851985344928194	0.944200803760703
**Acadm**	4975.16297362946	0.0124576955582534	0.0668187928639713	0.186439997256679	0.85209972897615	0.944200803760703
**Zfp59**	912.604582948821	-0.0234476514854933	0.125716315047414	-0.186512398781734	0.852042956631091	0.944200803760703
**Afg1l**	548.651166814185	0.0361314525715158	0.194044035116043	0.186202335721932	0.85228609235082	0.944340901284851

**Ltn1**	7129.4023017258	-0.0195318889173872	0.104956247173534	-0.186095534504901	0.852369843697954	0.944367292022828
**Prdm1**	374.465954438475	0.0674642165229633	0.362919944193722	0.185892832847323	0.852528802801453	0.944476998224179
**Aggf1**	3798.34726737328	-0.0399214831009909	0.214929822379633	-0.185741944319281	0.852647133819497	0.944541682198666
**Kpna6**	1019.14871634935	0.0207912267720293	0.112085701676405	0.185494014500211	0.852841574541661	0.944690663624109
**Cwc15**	3602.19496084658	0.0168226721928845	0.0908208081076952	0.185229272271352	0.853049210387959	0.944787826532865
**Gm14419**	39.7225400374775	-0.0957572377911927	0.516897804668989	-0.185253713454081	0.853030040878489	0.944787826532865
**Srek1ip1**	503.899383940396	0.0339335326814266	0.183486233830468	0.18493775785261	0.853277855291081	0.944974639149675
**Rnf40**	9633.80006955562	0.0163371773871877	0.0884224725196669	0.184762729673206	0.853415141906792	0.945060256590436
**Ampd3**	1154.28382542919	-0.0269357170375402	0.146072029833262	-0.18440023780245	0.853699483278261	0.945308696979309
**Tspyl2**	2597.93460496609	-0.0154885591226365	0.0841906462326678	-0.183970070497293	0.854036934431664	0.945350191488565
**Tmem256**	494.104404916048	0.0280348582775502	0.152380986761947	0.183978715936175	0.85403015212511	0.945350191488565
**Gm7420**	23.1530967990758	-0.121139247538289	0.657963423201436	-0.184112434318711	0.853925252050637	0.945350191488565
**Gm44510**	668.148156030618	-0.0286026736643003	0.155261186798349	-0.184222948787897	0.853838557048833	0.945350191488565
**Gm48161**	143.216993287102	-0.0455601264201184	0.247421967365794	-0.184139374951947	0.85390411783864	0.945350191488565
**A930007I19Rik**	56.2870165972453	-0.0689014110887957	0.37469198715304	-0.183888135992226	0.854101212204923	0.94535493121052
**Txnl4a**	5734.87382697867	0.0195744164150156	0.106601688790589	0.183622010468034	0.854309994858113	0.945519603075036
**Psmg2**	2149.38824919044	0.0162576837936944	0.088631471215535	0.183430146997772	0.854460523256769	0.945619782620938
**Twf1**	1462.83893423758	-0.0286128991262248	0.156218760807628	-0.18315917357365	0.854673127181901	0.945722223666097
**Idh2**	9337.98459375774	0.0177694588987801	0.0970145156467071	0.183162888360854	0.854670212513486	0.945722223666097
**Lax1**	10020.1407977544	0.0238304102601524	0.130408196493334	0.182737058719852	0.855004336672067	0.945842952856578
**Gm10036**	210.142385350103	-0.0458803119570344	0.250967318928238	-0.182813890481706	0.854944049259777	0.945842952856578
**Gm15911**	36.8164803954608	0.0718954778724613	0.393486174533925	0.182714114308132	0.855022340578099	0.945842952856578
**Gm29151**	17.4990901546933	0.133736419363239	0.731179543691524	0.182905034087854	0.854872532901707	0.945842952856578
**Eif5a2**	450.10713426229	-0.0376238740113205	0.206024165992447	-0.182618741981461	0.855097177666367	0.945859335099259
**Mybl2**	243.437900372479	0.0480736421354652	0.263849873607202	0.182200739679084	0.855425192530268	0.946021623206214
**Igkv8-27**	33.1225405307369	0.161095231065405	0.884012963346966	0.182231751959249	0.855400855707891	0.946021623206214
**Zfp973**	275.988154982878	0.0325769222101975	0.178870496205239	0.182125744051262	0.855484045761588	0.946021623206214
**Pira2**	111.226094086463	0.0462013223677721	0.25350056225565	0.182253332918366	0.85538392017625	0.946021623206214
**Itga2**	173.752795498477	-0.055882002311532	0.308699985067767	-0.181023663798573	0.85634900183504	0.946119192321127
**Katna1**	6167.91071036242	-0.0161216533663805	0.0892100008081923	-0.18071576303472	0.856590685391536	0.946119192321127
**Arl6**	122.790860902732	-0.0484433304183383	0.267467344434054	-0.181118672714389	0.856274428276524	0.946119192321127
**Napb**	123.139829411707	-0.0798800067941056	0.441905538668437	-0.180762628671282	0.856553897821043	0.946119192321127
**Iqgap3**	24.6630467325845	-0.244246904981847	1.34780644450896	-0.181218086600582	0.856196398581467	0.946119192321127
**Elf4**	9407.76962406462	0.0292217667273814	0.161702828469894	0.180712774191343	0.856593031519528	0.946119192321127
**Hacd3**	7526.84194133035	-0.0184139898702636	0.101793987674805	-0.180894670607557	0.856450252083179	0.946119192321127
**Ankrd46**	3196.83746283097	0.0124179393196006	0.0687150681724807	0.180716393796343	0.856590190267895	0.946119192321127
**Olfr524**	35.6916040648098	0.117313625076441	0.648841453092898	0.180804762885032	0.856520824486203	0.946119192321127
**Ptk2b**	24624.4277509713	-0.0226426481858096	0.125255797984446	-0.180771258098737	0.856547124114294	0.946119192321127
**Snhg12**	225.446863591747	0.029833869801394	0.164370548788453	0.181503742740377	0.85597219566196	0.946119192321127
**Gm15890**	21.6571857836424	0.109080402554393	0.602469028040781	0.181055618591914	0.856323920017888	0.946119192321127
**Gm44270**	20.8778441252218	-0.117716350481893	0.648377794539344	-0.181555185068495	0.855931821359215	0.946119192321127
**Gm5586**	22.2402240357316	0.0835252687216153	0.460178656818566	0.181506176968452	0.855970285159423	0.946119192321127
**Gm49368**	112.979809068654	0.122497838636806	0.67384629989769	0.181788990538948	0.855748324836382	0.946119192321127
**Gm47079**	28.7947498422628	0.149238219964176	0.82439943072342	0.181026592695749	0.856346702891791	0.946119192321127
**Gm36635**	48.2530289919431	0.0923334402911709	0.509485606430646	0.181228751363636	0.856188027920767	0.946119192321127
**Rtl6**	1333.51260144886	0.0269807880567302	0.149545277377538	0.180418857284374	0.85682375126133	0.946307692480885
**Dusp18**	20.7125763017601	0.177090028617345	0.982342269879488	0.180273244924165	0.856938059005455	0.946367605533289
**Kdm5c**	3543.76997244373	0.0310607133782538	0.172686419262873	0.179867725040795	0.857256413605977	0.946586496789725
**Mettl5**	1255.87727935436	0.0201632422278781	0.112064648650248	0.179925092085082	0.857211376024489	0.946586496789725
**Napg**	2774.49232051894	-0.0165608815024902	0.0921986604232526	-0.179621714962721	0.857449555875591	0.946600760311896
**Zfp524**	1711.20896713114	-0.019337693076978	0.107623414739145	-0.17967923730954	0.857404394372922	0.946600760311896
**Casc3**	6164.55466200725	-0.0184742202825724	0.102787987803305	-0.17973131566623	0.857363507416517	0.946600760311896
**Ankrd9**	276.652487476609	0.0457247199312814	0.254683395910436	0.179535535749497	0.857517217110063	0.946609134790757
**Rfc4**	819.548601711242	0.0334180908166047	0.186358256112811	0.179321761824038	0.857685060335036	0.946651175994125
**Spag1**	188.008633164094	0.0643356863258195	0.358965843679217	0.179225091909613	0.857760962210779	0.946651175994125
**Dohh**	4658.87803499428	-0.015164961985826	0.0846155603502917	-0.17922190579423	0.85776346386085	0.946651175994125

**Gm36602**	31.3861431475404	0.118312481217066	0.660296058771477	0.179180959276349	0.85779561406639	0.946651175994125
**Srgap3**	143.177890222151	0.0521010185874356	0.290958604979518	0.179066773402709	0.857885271270791	0.946659141600753
**Tnfaip8l1**	4064.91573305423	-0.0139789674367521	0.0780866163152194	-0.17901873709474	0.857922989278337	0.946659141600753
**Ap1g2**	18502.9507560584	0.0133426824811984	0.0746593221243649	0.178714219491206	0.858162103399373	0.946856681133248
**Pigh**	2642.70935606181	-0.0238492432248406	0.133641105891407	-0.178457392025922	0.858363780260236	0.946946585840547
**Itfg1**	6280.53626512209	-0.0183106021991566	0.102600022583979	-0.178465869090518	0.858357123395561	0.946946585840547
**Fam98b**	414.579008244316	0.0290495942544064	0.16317120032992	0.178031381736914	0.858698330348108	0.947195725027305
**Myo1d**	35.9732219052942	0.209698443306374	1.17797044283711	0.178016727483689	0.858709838929685	0.947195725027305
**Tmem18**	1460.66329303943	0.0186748152243645	0.104953288297642	0.177934541425741	0.858774383542754	0.947200613291557
**Nfatc2ip**	444.257022310533	0.0291199131704729	0.163940012433474	0.17762541760383	0.859017161603985	0.94725147545794
**Arid5a**	27802.5087217527	0.0295702561406594	0.1665010384323	0.177598028331114	0.859038673091925	0.94725147545794
**Slc26a1**	42.0892850765002	-0.0808282440710507	0.454859689187928	-0.177699290555633	0.858959142435835	0.94725147545794
**Mast1**	414.713169525499	0.0522153476922353	0.294182396128878	0.177493107607161	0.859121078617086	0.94725147545794
**Tpt1-ps3**	748.329146426978	0.0269898630877776	0.152045910555137	0.177511272675698	0.85910681152586	0.94725147545794
**3110080O07Rik**	18.0516589219656	0.117907120728784	0.664766835758322	0.177366129575769	0.859220810169679	0.947295151542376
**Cnot9**	5398.18790693402	0.0106354544750038	0.0600344719625809	0.177155792785731	0.859386018606403	0.9474110050501
**Gdi1**	28208.3942542387	0.0180884440075779	0.102236043735575	0.176928247090255	0.859564750661292	0.947541750116831
**Gtpbp1**	19213.0148259301	-0.0217337051898908	0.122983885127168	-0.176719943165056	0.859728375044939	0.947655824105149
**Kctd20**	8227.12704344458	-0.011394686458016	0.0646055536739638	-0.176373172429107	0.860000779562174	0.947734584157761
**Camk2g**	3863.35720840359	0.0157054405034089	0.0890019293514754	0.176461798276157	0.859931158257304	0.947734584157761
**Haus8**	2131.31314862265	-0.0176939396501683	0.100393337130393	-0.17624615493344	0.860100561874467	0.947734584157761
**Zmynd15**	38.5446862661501	0.0980888707199383	0.556241112719511	0.176342360312731	0.860024984716954	0.947734584157761
**Specc1**	89.0843992171527	0.0708514615109426	0.401927871785242	0.17627904528303	0.860074723683062	0.947734584157761
**Acy1**	1359.34917716354	-0.0248673580816957	0.141249412500189	-0.176052824868652	0.860252442253796	0.947769384183545
**Gm9830**	131.554312133684	0.0314647253958912	0.178656017417937	0.176119035063255	0.860200426846548	0.947769384183545
**Gm48146**	28.6724163806295	0.0850769076035072	0.484392621653023	0.175636258275728	0.860579715135902	0.94806366296556
**Dhfr**	423.890706177194	0.0202944139832547	0.115764435088131	0.175307847939695	0.860837745501286	0.948149053254842
**Rpl38**	819.209835283859	-0.0427137921029755	0.2434892971681	-0.175423694592566	0.860746723665767	0.948149053254842
**Gm15334**	48.1366335461315	-0.0830808379744856	0.473753638680012	-0.175367176505426	0.860791130242412	0.948149053254842
**Nf1**	169.336639229476	-0.0447381096124445	0.255353998163145	-0.175200349061546	0.860922210063055	0.948175806525726
**Il4i1**	23.4488095514018	-0.0894163709104252	0.510802224098951	-0.175050864487042	0.86103966648225	0.94822148187333
**Dynlt1b**	1690.18150522313	-0.0177709078123443	0.101595759786894	-0.174917810050541	0.861144215625268	0.94822148187333
**Gm43584**	48.5746588903037	0.0621615386816196	0.355234418315461	0.174987375875324	0.861089553128054	0.94822148187333
**Apip**	548.715991198437	-0.0270559401945665	0.155608575237407	-0.173871781508751	0.861966229900701	0.948327960304238
**Glul**	3442.86124617652	0.0194669759392456	0.111814584792327	0.174100507330073	0.861786474497543	0.948327960304238
**Them4**	562.734592777398	-0.0212503399204352	0.122321984181251	-0.173724617554825	0.862081889690751	0.948327960304238
**Abcb7**	1631.28463149551	0.0160149147021495	0.0921710357813862	0.173752139881927	0.862060258988518	0.948327960304238
**Slc7a6**	6092.13412817391	-0.0125104536728621	0.0717351397420492	-0.174397843481565	0.861552809003919	0.948327960304238
**Nfatc1**	23315.7720148851	-0.0188322962705986	0.108151617025196	-0.174128661120353	0.861764348955395	0.948327960304238
**Mcrs1**	7503.92572005645	-0.0149547440076279	0.0857142067111786	-0.174472174233838	0.861494397104844	0.948327960304238
**Tnfrsf23**	278.776120869048	0.0388468086766781	0.223426819562645	0.173868154023408	0.861969080795558	0.948327960304238
**Ptger4**	2942.86759197838	-0.0251389679422568	0.144707553353238	-0.173722569138401	0.862083499612737	0.948327960304238
**Myof**	20.155340524141	-0.20829222085785	1.19298851665148	-0.174597004036964	0.861396302857918	0.948327960304238
**Rpl7a-ps3**	62.6761804580327	0.0713137135444827	0.408745890075768	0.174469554987485	0.861496455394205	0.948327960304238
**Gm6210**	125.379915560363	0.0491149751332189	0.282666223396	0.173756080734172	0.862057161751568	0.948327960304238
**E430024I08Rik**	608.498207038036	-0.027387666481027	0.157519482997684	-0.173868438112063	0.86196885752598	0.948327960304238
**Gm47138**	77.0869006177329	0.0727056998695842	0.417986903984612	0.173942530678571	0.861910627432068	0.948327960304238
**Jkamp**	2666.64989233222	0.020923976728102	0.120641483802668	0.173439318454729	0.862306121728439	0.94844491670737
**Vangl2**	57.5040571404991	-0.0907709497888067	0.523443510679473	-0.173411166509598	0.862328248470852	0.94844491670737
**Serbp1**	16590.9926783705	0.0116150009620529	0.0670002629038114	0.173357543070061	0.862370395485593	0.94844491670737
**Trmt112-ps2**	915.043926817981	0.0213346927956513	0.123258477317084	0.173089050425047	0.862581431543594	0.948544603031084
**Acot1**	33.2497744146745	-0.0808326546335675	0.466806655710322	-0.173160887156948	0.862524966686215	0.948544603031084
**Bms1**	5335.64900061459	0.017907209181074	0.103661999682807	0.172746129110646	0.862850983029101	0.948774803879242
**Marchf9**	48.4072810700131	-0.0736116258533073	0.426772372124053	-0.172484515543827	0.863056633729202	0.948934713395509
**Gm15772**	306.780414483475	-0.0268378443785701	0.155873054246242	-0.172177574298202	0.863297927702408	0.949133787803994
**Fam83d**	33.1309012582907	-0.160648583098269	0.933964183802775	-0.172007220281364	0.863431852639671	0.949166490068574

**Pi4kb**	1774.56706480374	-0.0230955248667875	0.134406610448793	-0.171833251278862	0.863568623571942	0.949166490068574
**Gpx4**	11288.8817627038	-0.0124559771157166	0.0724732238747729	-0.171870056963927	0.863539687341152	0.949166490068574
**Gm3791**	397.956660996773	-0.0408559035257259	0.237686694072429	-0.171889737812905	0.863524214546042	0.949166490068574
**Fads6**	44.8902435428037	-0.140283978250388	0.819100599141527	-0.171265871856785	0.864014714257843	0.949458110925506
**2810039B14Rik**	150.690133126155	0.049410057704884	0.28835495643311	0.17135151174814 5	0.863947378830441	0.949458110925506
**Gm37334**	38.9084266891402	-0.0981773859426684	0.57324017780368	-0.171267454278635	0.864013470050067	0.949458110925506
**Pipox**	17.5108279815033	-0.198933104555511	1.16286881596446	-0.171070977073644	0.864167956380312	0.949494072148705
**Gm43703**	100.675600378206	-0.0752828478273102	0.439883023551135	-0.171142880713056	0.864111419298523	0.949494072148705
**4930432K21Rik**	797.776794972801	-0.020517674015034	0.120329886128906	-0.170511870949948	0.864607599400648	0.9498858878134
**Lactb2**	1312.69920335224	0.0196134158647831	0.115162470694364	0.170310829096713	0.864765695289917	0.9498858878134
**Lpgat1**	726.460914015301	-0.0252626662239576	0.148321850911337	-0.170323294030756	0.864755892920612	0.9498858878134
**Nat2**	320.075415951657	-0.0357354672320385	0.209805154943355	-0.170326926627168	0.864753036266688	0.9498858878134
**Rfng**	3615.89184962933	0.0219497863230816	0.12895974457824	0.170206496568894	0.864847742746776	0.949909792448135
**Gsdmd**	7018.87670968672	0.0212184157493162	0.124769079916736	0.170061490903645	0.864961778117631	0.949968825385063
**AI182371**	62.9750576834135	0.0699903546790688	0.41213107184968	0.169825474126341	0.865147392469501	0.94997403924334
**Slc25a3**	22651.8495937914	-0.0095115051731107	0.0559875453993017	-0.169886089937949	0.865099720721824	0.94997403924334
**Gm371**	83.1236202302523	-0.070655717940199	0.415808987290476	-0.169923498769497	0.865070300518038	0.94997403924334
**Top1mt**	1053.90733603101	0.0191681957323433	0.113396657881394	0.169036690238191	0.86576778199871	0.949993241013487
**Snca**	28.7511693038943	-0.152356740035011	0.900825473250297	-0.169130141807921	0.865694276708629	0.949993241013487
**Ncoa3**	13828.8268544961	0.0211302655545745	0.124669267925796	0.169490572184569	0.865410787388286	0.949993241013487
**Mid1**	595.191993488161	0.0257913560845742	0.152137238534636	0.169526910919331	0.86538220684201	0.949993241013487
**Rab3gap1**	6618.45185979203	-0.01311691341466	0.0775630738399612	-0.169112862155574	0.865707868108775	0.949993241013487
**Nt5dc3**	273.553032153314	0.039721942173646	0.234664146082194	0.169271458110746	0.865583125129043	0.949993241013487
**Slc14a1**	3851.2774676088	0.0309804928317808	0.182964571377181	0.169325091729997	0.865540940589575	0.949993241013487
**Rplp0**	94477.8523785726	-0.0103296291235105	0.0610941742419824	-0.169077154273283	0.865735954454564	0.949993241013487
**Gm47234**	187.083880739296	0.05326828447045	0.314547846422837	0.169348749566205	0.865522333074196	0.949993241013487
**A630089N07Rik**	810.554435524954	0.0231515322330906	0.136667504426552	0.169400416947927	0.865481695567904	0.949993241013487
**Gm36684**	16.8597528830844	-0.0888159117361156	0.525739217575825	-0.168935298655566	0.86584753390063	0.950014594504019
**Brms1l**	1693.54397834098	-0.0243486503928784	0.144944945132299	-0.167985509054172	0.866594679176163	0.950577029016681
**Trappc9**	5751.06978767675	-0.0178987098227743	0.106554587764298	-0.167976904592478	0.866601448361022	0.950577029016681
**Dand5**	976.027645613329	0.0244122775354162	0.145249345568989	0.16807151481327	0.866527018435042	0.950577029016681
**Trav9n-2**	80.7775905223512	0.0498037722519357	0.296346149033051	0.168059454845088	0.866536505954529	0.950577029016681
**Rpl30-ps1**	51.9796873389227	0.0959746725734359	0.5736058723673	0.167318148570104	0.86711972493436	0.951079319629035
**Tmem192**	4608.56955257217	0.0144093227371992	0.0864050831370736	0.166764757512474	0.867555149740194	0.951376641815463
**D930028M14Rik**	773.642572164202	-0.0233618511294224	0.140106579120327	-0.166743426869046	0.867571934140153	0.951376641815463
**Gm14123**	19.4924321439122	-0.149864762243455	0.898135615164643	-0.166862063716271	0.867478583363285	0.951376641815463
**Adat2**	115.245572741908	0.0440549676646778	0.264563694705173	0.166519324254872	0.867748276914699	0.951398772653282
**Tnfrsf4**	315.687182279624	0.0459404685900044	0.27581396049508	0.166563246137151	0.867713714966955	0.951398772653282
**Gm7582**	39.0468139555907	-0.0624520979678649	0.375115649447321	-0.16648758338896	0.867773253828328	0.951398772653282
**Zpbp**	449.357497913008	-0.0283222232659426	0.17087146749373	-0.165751624196602	0.86835241768447	0.951413960930707
**Irak1**	12590.8020332749	-0.0201651908255443	0.121751366657754	-0.16562599155235	0.868451291493708	0.951413960930707
**Uba7**	27422.0163688457	-0.0130003692627661	0.0782769447528448	-0.166081715424817	0.868092643290802	0.951413960930707
**Dennd4b**	8219.9513951049	-0.0252698671547778	0.152312095935148	-0.165908472335232	0.868228979943004	0.951413960930707
**Fes**	128.032603859703	0.0667351782469927	0.402348348706432	0.165864178296119	0.868263838534042	0.951413960930707
**Dmac2l**	658.198637804013	0.0177205166081532	0.106943469861064	0.165699847135827	0.868393166360395	0.951413960930707
**9330161L09Rik**	36.6201900898966	-0.0712659909831745	0.430208888900016	-0.165654389813729	0.868428941674427	0.951413960930707
**Gda**	17.034142170858	-0.190785856569181	1.1484889054985	-0.166119024446623	0.868063282822623	0.951413960930707
**Hnrnpa3**	1067.33343476998	0.126014999072186	0.758206270596549	0.166201473080721	0.867998400220521	0.951413960930707
**Gm48905**	20.5532198785048	-0.0926275243650791	0.559116846483545	-0.16566756116837	0.868418575674254	0.951413960930707
**Gm2999**	73.6425596769083	0.0522331584174914	0.314280751710297	0.166199037431474	0.8680003169316	0.951413960930707
**Gpatch11**	1920.27743566002	0.0205837177003375	0.124375670435226	0.165496335644336	0.868553333798191	0.951459599600813
**Syap1**	1821.79120050628	0.0134183009881841	0.0811958514494396	0.165258455310856	0.86874055700986	0.95153688772948
**Gm44502**	474.426474783349	-0.0382669333472991	0.231565407895118	-0.165253237498371	0.868744663760634	0.95153688772948
**Spata6**	4573.39941472466	-0.0189269873596076	0.114627720017848	-0.165117018437257	0.86885187809081	0.951588172869736
**Atp5e**	2195.62986202361	0.0145631279847087	0.0883418386916021	0.164849726928913	0.869062262997538	0.951688159883277
**Gm43420**	25.9239380440519	-0.0984473731021434	0.597202482640508	-0.164847561696097	0.869063967288219	0.951688159883277

**Zxdb**	163.729691676833	0.04169630390262	0.253441262840542	0.164520581357954	0.869321345916474	0.951903853204022
**Wdr76**	499.338781619782	-0.0262338493541978	0.159682852704877	-0.164287203728022	0.869505054771745	0.951972703449026
**Gm5850**	47.2741182744261	0.0500674398777582	0.304643313717218	0.164347739219488	0.869457402116125	0.951972703449026
**Tram1**	16404.956385514	-0.0121803140785216	0.0742751283010223	-0.163989135490379	0.869739696647792	0.952145766101759
**Spint2**	6477.7228248453	-0.0094916918099958	0.0578998644792399	-0.163932884737564	0.869783979010518	0.952145766101759
**Abcb1b**	1068.85430921405	0.0296428452986711	0.18114073743489	0.163645382692151	0.870010316042087	0.952261221858514
**Gm9892**	433.707916340835	0.0249438031064567	0.152383937191569	0.163690501546096	0.869974795355187	0.952261221858514
**Map3k1**	6320.62100127651	0.0326728956811682	0.199979029255052	0.163381609576159	0.87021798175499	0.952299821313741
**Ift88**	175.970879820233	-0.0388473113728155	0.23767278890836	-0.163448712623951	0.870165151404032	0.952299821313741
**Gm11405**	40.4472116660789	-0.0659055867321744	0.403412306565952	-0.163370293021538	0.870226891356004	0.952299821313741
**Gm43028**	109.53324207153	0.0312261584436232	0.191287909423418	0.163241673442642	0.870328155589242	0.952344496362478
**Pycard**	7662.40279269572	0.0150509298299485	0.0923623143339239	0.162955312872887	0.870553619439083	0.952392791883749
**Rnf14**	8303.1979193859	-0.0127016925239554	0.0779061812827813	-0.163038314993918	0.870488267257115	0.952392791883749
**Gm49207**	25.8752872992086	-0.0715687871226072	0.439180950113075	-0.162959680068501	0.870550180881274	0.952392791883749
**Prex1**	25133.7142439283	-0.0191288955476472	0.117560570164698	-0.162715232844213	0.870742652760354	0.952517753124265
**Lrrc29**	596.621917048584	-0.0420582563058557	0.258730427397295	-0.162556281953158	0.870867810986344	0.952517753124265
**Gm12286**	38.9150117027695	0.0528512681632135	0.325232260293734	0.162503154254996	0.870909644555517	0.952517753124265
**Gm12844**	23.1974689633345	0.0758684529348438	0.466869188695808	0.162504733171151	0.87090840128737	0.952517753124265
**Dusp12**	1227.39846267765	0.0190955788435325	0.117599964692425	0.162377419869775	0.871008651188861	0.952559919265886
**B4galt4**	74.0711077566726	-0.0779901459883859	0.48185837738266	-0.161852838197003	0.871421742685473	0.952747182868096
**Zfp3**	781.896128081092	0.0228469680431883	0.141050738287923	0.161976947589961	0.871324007287298	0.952747182868096
**Zfp647**	129.00691343535	-0.06836231350364	0.4222524576097	-0.161899148889808	0.871385273064601	0.952747182868096
**Gm43774**	22.690772329078	0.101181149196218	0.624438834970143	0.162035324406202	0.871278036576035	0.952747182868096
**Gm16253**	37.0921556727353	-0.0544786040206805	0.336846567514029	-0.161731213183318	0.871517523554688	0.952785791899758
**Zfp808**	518.249821905639	0.032191227574083	0.199277789619659	0.161539465263656	0.871668530713552	0.952818663784491
**C530043K16Rik**	63.4538807746441	-0.083867207606641	0.518957158100638	-0.161607189143689	0.871615195620114	0.952818663784491
**9230112E08Rik**	84.3329589346855	0.0818882867885359	0.507316702786601	0.161414529304354	0.871766923994319	0.952860115245455
**Lin7c**	3292.4018805379	0.0111026299279631	0.0688771777732408	0.161194611726332	0.871940124842625	0.952871144757643
**Zbtb46**	328.6284098309	0.0354933021672061	0.220220894166001	0.161171365240446	0.871958433474675	0.952871144757643
**Gm3571**	358.640012300831	-0.023835077730865	0.147756958353149	-0.161312725955671	0.871847100661133	0.952871144757643
**Parg**	3001.01932050283	-0.0277893295068546	0.172741120443008	-0.160872694559273	0.872193668744689	0.95302224335749
**Micu2**	6159.35135991437	0.0155380177983474	0.0966040956412384	0.160842226152102	0.872217666526519	0.95302224335749
**Arpp19**	3296.89236043276	0.0147707044151743	0.0920486955268994	0.160466200315222	0.872513844818166	0.953213662400322
**Bcl2**	27048.2601583837	-0.0132955330455726	0.0828195181832101	-0.160536227899331	0.872458685949072	0.953213662400322
**Ccl25**	364.204022056008	-0.0317226803739418	0.197881541924099	-0.160311467484469	0.872635725959013	0.95321921306776
**Dnmbp**	2188.70150772746	-0.0216896769133935	0.135301590611036	-0.160306148770615	0.872639915498029	0.95321921306776
**Cd3g**	46523.7496219316	0.0081104817010970	0.0506885049905654	0.160006330875347	0.87287608715666	0.953319997276838
**Borcs8**	1818.59520740355	-0.0141478791656443	0.0885965015394813	-0.159688914571188	0.873126133728195	0.953319997276838
**Cd163**	92.8583425002311	0.0825625083006313	0.517748105644633	0.159464626524968	0.873302825607166	0.953319997276838
**Gopc**	3618.06338296391	-0.0151449701535214	0.094999843230683	-0.159421001535189	0.873337193672092	0.953319997276838
**Pwwp3a**	7201.24766054271	0.0179359440349305	0.112472424229686	0.159469702531729	0.873298826709895	0.953319997276838
**Rpusd1**	1321.32578915556	0.0211621817021215	0.132680445525845	0.159497366912287	0.873277032663449	0.953319997276838
**Nsa2**	4614.15008952527	-0.0141614887355609	0.0886356601008843	-0.159771910306105	0.873060752122221	0.953319997276838
**Gm10293**	89.8293628224098	0.0391409158019257	0.245192578123407	0.159633362891702	0.873169896196281	0.953319997276838
**Gm2800**	64.8641545482319	0.0416529673348216	0.260222689746228	0.160066623611653	0.872828592631367	0.953319997276838
**AA388235**	22.2126391332975	0.0889977678976144	0.556932794540539	0.15979983360656	0.873038755159913	0.953319997276838
**Gm13292**	704.689382315791	0.0171627467728557	0.10782799859796	0.159167813517966	0.873536661638574	0.953455800741715
**Gm38431**	2927.01492140518	0.0834652808701739	0.524577689900039	0.159109475063796	0.873582623298099	0.953455800741715
**Slc25a35**	1042.97741646082	-0.0327571312271218	0.206396531314638	-0.158709698358185	0.873897596848322	0.953469355597494
**Marchf5**	4247.23411742995	0.0225387937230459	0.141819268184848	0.158926174218222	0.873727038728518	0.953469355597494
**Hells**	621.089982208142	-0.0261334992116239	0.164553175311438	-0.158814919020329	0.873814694316627	0.953469355597494
**Adrb2**	3000.19294841785	0.0294860874888674	0.18571802913732	0.158768040054234	0.873851629714052	0.953469355597494
**Rpsa-ps10**	1425.89016387391	0.0221823722968162	0.139562068065414	0.158942702729362	0.87371401640072	0.953469355597494
**Zfp446**	1081.55825692668	-0.026145730741719	0.165107361060567	-0.158355936245191	0.874176333380134	0.953575388374577
**Gm15500**	3740.23176740916	-0.0181758870111978	0.114725599061779	-0.158429218586256	0.874118591414902	0.953575388374577
**Gm43005**	20.9511139933278	-0.0792715094341468	0.500338786182583	-0.158435667238516	0.874113510306714	0.953575388374577

**Gm9993**	169.417875204073	-0.0476223686757212	0.301519117217585	-0.157941456963591	0.874502930095852	0.953865614669828
**Nsmce1**	5217.10608932812	0.0118899770991819	0.0753609009314237	0.157773818415487	0.874635030106423	0.953943667847089
**Zfp512b**	3380.3649738641	-0.028704794419766	0.18255396019864	-0.157240053234298	0.875055662982535	0.954072244783823
**Alkbh4**	5354.40786656026	0.0104764775937463	0.0665761571986367	0.15736080354546	0.87496050278095	0.954072244783823
**Aqr**	10883.855460444	-0.0164894682551848	0.104766314729666	-0.157392844233696	0.874935252645565	0.954072244783823
**Gm13577**	201.343877265812	0.0245505946924356	0.156072721903886	0.157302278021105	0.87500662500942	0.954072244783823
**C630004M23Rik**	28.6242405484766	0.0965646948698224	0.613015849889928	0.157523977377031	0.87483191257151	0.954072244783823
**Nudt8**	735.921331012017	-0.0229087964944103	0.145968577281582	-0.156943343019764	0.875289500311165	0.954261167674741
**Usp19**	12161.3004781418	-0.0157011159233555	0.101572140998268	-0.154580929072109	0.877151705655537	0.95453715411724
**Vps4b**	10225.3753502623	0.0133064383212308	0.085254612531552	0.156078808244026	0.875970902208242	0.95453715411724
**Irf4**	215.190112758588	0.0383976288038383	0.248575964568699	0.154470400509002	0.87723884805304	0.95453715411724
**Ndufa6**	3265.96898486128	-0.0124458932175566	0.0803523369637804	-0.154891490252073	0.876906862477728	0.95453715411724
**Smarca2**	15199.0030789948	0.0197650052898253	0.126496161547895	0.15624984227163	0.875836090705538	0.95453715411724
**Uck2**	4912.51413954023	-0.0155158935718244	0.100261315887438	-0.15475453752516	0.87701483312126	0.95453715411724
**Yme1l1**	5932.01905493878	-0.0129530001282019	0.0838468661704711	-0.154484010193856	0.877228117890499	0.95453715411724
**Hdc**	178.686450203759	0.0476863367175112	0.306967232037057	0.155346668115229	0.876548026310665	0.95453715411724
**Nfatc2**	2874.49093461908	-0.0336047448627896	0.21671679010342	-0.155062950345255	0.876771690182796	0.95453715411724
**Ccne2**	147.591585759188	-0.064854689496131	0.418630281622138	-0.154921161567261	0.876883470539965	0.95453715411724
**Reep4**	6021.83832725493	0.0111261014938975	0.0713926512463582	0.15584379203826	0.876156151261586	0.95453715411724
**Ints14**	5500.57875900827	-0.0153468892104533	0.0981416533523241	-0.156374879434308	0.87573753687871	0.95453715411724
**Gxylt1**	935.940902045484	0.0266475366042159	0.171771795642816	0.155133364616081	0.876716179422501	0.95453715411724
**Timeless**	2957.4875650094	-0.0159124457029728	0.102503983057088	-0.1552373403296	0.876634211765051	0.95453715411724
**Nup43**	1174.11725873908	0.0212146699575144	0.135836667287004	0.15617778602217	0.875892886048132	0.95453715411724
**Phldb1**	26.3973396174856	-0.0837030792980101	0.535475354012393	-0.15631546563406	0.875784366375025	0.95453715411724
**Smim20**	4329.97533064968	0.00973171379432186	0.062922229543782	0.154662571000451	0.877087338875505	0.95453715411724
**Snx29**	1481.58043657439	-0.0254312476901213	0.164445911960774	-0.15464809910378	0.877098748509881	0.95453715411724
**Kcnip3**	107.637884776265	-0.0582494230435954	0.376963259460912	-0.154522812453651	0.877197525494469	0.95453715411724
**Rbpj-ps3**	114.351700066632	-0.0314573668412384	0.202274007735655	-0.155518581914632	0.876412505910919	0.95453715411724
**Gm12743**	137.201334886106	0.0490955623972115	0.317334047678505	0.154712558442361	0.877047929003714	0.95453715411724
**Gm6576**	39.5704320750934	0.0522868350677504	0.338032378553123	0.1546799608119	0.877073628792705	0.95453715411724
**Trbd2**	156.180724521269	0.0244023491686018	0.157476152656557	0.15495901288509	0.876853629899799	0.95453715411724
**Gm28373**	192.881411541122	0.0499362293750256	0.321864812039627	0.155146594182149	0.876705750023771	0.95453715411724
**Trmt112-ps1**	27.4117957820058	0.0659861402685596	0.425929202545378	0.154922789689513	0.876882186982367	0.95453715411724
**Gm9048**	167.026935344672	-0.0343359528607882	0.221847069274268	-0.154773073960869	0.877000219252068	0.95453715411724
**Gm16374**	58.0523963389839	0.0451869662060664	0.292049728289211	0.154723534484232	0.877039275583042	0.95453715411724
**Gm4828**	25.6788225729346	0.0705823835690294	0.453023977516859	0.155802754538313	0.876188499335578	0.95453715411724
**1600010M07Rik**	51.3620946421852	-0.0579822297201438	0.375686206011914	-0.154336860902221	0.877344134691397	0.954585798255237
**Pde8a**	314.024782147174	0.0421794333249881	0.273479132016309	0.154232730716992	0.877426235564018	0.954609210369553
**Snord7**	23.4232423330106	0.086354456757826	0.560207144460554	0.154147367829413	0.877493540447445	0.954616522841093
**Dram2**	1728.89655930458	0.0214918060382814	0.139718242900407	0.153822475806549	0.87774971161768	0.95482928587917
**Tma7**	4785.77452866177	0.0189131047491988	0.123115594659944	0.153620707445214	0.877908808572508	0.954936428046184
**Cldn34c1**	27.7598575956207	-0.0687310992983985	0.448265834325393	-0.153326651364884	0.878140684412804	0.955056789363097
**Gm5851**	50.7017879052001	0.0478400871413762	0.311970284669711	0.153348217738191	0.878123678046317	0.955056789363097
**Pdcd2**	1113.04606720367	-0.0245327092577027	0.160429366735594	-0.15291906810388	0.878462098591838	0.955159029759443
**Prkcz**	1687.43128165373	-0.0215142466330558	0.141054101884688	-0.152524785494318	0.878773043136374	0.955159029759443
**Gnptg**	10561.1057295598	0.00947620714296164	0.0620955081771182	0.152606966609117	0.878708230797334	0.955159029759443
**Abcb1a**	2458.76747164411	-0.0286436043161242	0.187821040880256	-0.1525047682724	0.878788829890906	0.955159029759443
**Ap1s3**	50.9792451684866	0.0833936270835186	0.545512182853267	0.152872162537844	0.878499088919351	0.955159029759443
**2410004P03Rik**	36.0016744594996	0.129802403444756	0.848797533556686	0.152925047862533	0.878457382896977	0.955159029759443
**Tspyl3**	6803.89422117753	-0.0077691285244220	0.0509242307048276	-0.152562511340708	0.878743290453602	0.955159029759443
**Rps2-ps9**	51.8265910094485	-0.0747163666977441	0.48936306180096	-0.152680846859941	0.878649965648973	0.955159029759443
**Gm43292**	51.4702345830944	-0.0836774918653666	0.548925222683684	-0.152438781108052	0.878840871577864	0.955159029759443
**Gm6125**	34.713617766969	0.0755717967974061	0.494922499842573	0.152694203277168	0.878639432273129	0.955159029759443
**Haus3**	3000.12667126341	-0.0143881909898964	0.0944345218145938	-0.152361559241494	0.87890177433237	0.955159339137537
**Lig4**	220.513614326154	-0.0605730391353433	0.398289976179497	-0.152082760697058	0.879121661009405	0.95533241465691
**Gm47558**	17.8246430023471	-0.152181603859859	1.00126328365327	-0.151989597885383	0.879195140018346	0.955346377578724

**Arhgef5**	39.8443054857067	0.113166530211764	0.74527039150949	0.151846271502285	0.879308185874821	0.955372678933923
**Pcdhga2**	22.6935271740934	-0.36991436388549	2.43677060330739	-0.151805165157283	0.879340608147406	0.955372678933923
**Aarsd1**	2111.45109571122	0.0146932268784625	0.0968584327114343	0.151697962347143	0.879425164384491	0.955398670381027
**Shf**	38.9181307173734	-0.0531217501563561	0.350418783210161	-0.151595041994358	0.879506344126328	0.955420990306691
**Ccnd2**	51327.3349900867	0.0169747327571257	0.112231124920924	0.151247996213937	0.879780090246331	0.955586605225852
**Gm7368**	19.1859005668779	0.0750216795267735	0.495770287265834	0.151323468658271	0.879720557139529	0.955586605225852
**Ttf2**	907.673535443766	0.017436490014412	0.115414497677281	0.151077120858486	0.879914880239603	0.955601265968393
**Mvk**	583.983008065482	0.027315433725546	0.180789011646387	0.151090121444845	0.879904624987465	0.955601265968393
**Trav8n-2**	371.692018907912	-0.0300774170649126	0.199754435396321	-0.150571961044258	0.880313381020937	0.95596497170715
**Gm8738**	30.5272305434726	-0.0800430945429452	0.531852136620276	-0.150498774060756	0.880371117880463	0.95596497170715
**Snx12**	3076.47800483608	0.0086902773452127	0.0578366686152413	0.150255496266993	0.880563043131396	0.956086338054492
**A430072P03Rik**	90.1698228211	-0.0557799875689395	0.37136332079252	-0.150203276537652	0.880604240920533	0.956086338054492
**Gm4617**	471.643708410061	-0.0194315535483528	0.129474126142193	-0.15008059237266	0.880701031593425	0.956125544633981
**Traj20**	100.472443970055	-0.0341289354247228	0.227537590473401	-0.14999251487948	0.880770520713499	0.95613510815588
**Alkbh6**	3215.66206271418	0.0114886110496497	0.0767262948119623	0.149734990824274	0.880973700697407	0.956276276918432
**Gm6505**	73.9328040767609	-0.0491607482160581	0.328452486078885	-0.149673850251361	0.881021940218625	0.956276276918432
**Tada2a**	693.59944464746	0.0182895694079143	0.122390046928765	0.149436738255027	0.881209024242211	0.956347585576453
**Ddx49**	4080.87719299668	-0.0105661105678721	0.0706814867171358	-0.149489082058464	0.881167723823887	0.956347585576453
**Cnot7**	4986.18850484528	0.015398449937909	0.103390931478699	0.148934241308015	0.881605521865252	0.95658023743498
**Plxnb2**	177.610204884956	-0.0759940700453607	0.509754805612374	-0.149079653999668	0.881490780228907	0.95658023743498
**Gm11775**	35.6474295996869	0.0720186770354554	0.483389908962502	0.148986720037307	0.881564111883124	0.95658023743498
**Il27ra**	34786.3470352626	0.0105998682813948	0.0715822208468825	0.148079623068253	0.882279930580226	0.956830858700545
**Rps9**	26247.4421618012	-0.0089232699011338	0.0607830585419958	-0.146805213741731	0.883285769305254	0.956830858700545
**Csf1**	67.7943473798347	0.0958071902838529	0.648190403019844	0.147807171839476	0.882494949202805	0.956830858700545
**Slc39a14**	1171.5104092762	-0.0188428868602857	0.128327760543723	-0.146834065992025	0.883262995325766	0.956830858700545
**Hdac7**	7317.50747683886	-0.0205818248910035	0.140356568987196	-0.146639555522913	0.883416530361587	0.956830858700545
**Csnk1a1**	25373.8252918273	-0.0095114433410914	0.0644471736241149	-0.147585112057303	0.882670205280396	0.956830858700545
**Eif1ad**	6333.44277190691	0.0132073514603356	0.089635665056087	0.147344825880094	0.88285985263221	0.956830858700545
**Pcyt2**	11649.1415187735	0.0116503866399192	0.0790824314294672	0.147319530132429	0.882879817848364	0.956830858700545
**Sec61a2**	695.153824747967	-0.0209593428114937	0.142109578815706	-0.147487192532424	0.882747488059864	0.956830858700545
**Cd93**	21.1545380717158	0.105141654216089	0.717711191409348	0.146495770826181	0.883530028294631	0.956830858700545
**Pum1**	16992.8294735571	-0.0145833906248521	0.0988056374527393	-0.14759674650981	0.882661022887395	0.956830858700545
**Tspan9**	1520.78168729486	0.0278564756502005	0.188137005978097	0.148064839797884	0.88229159732089	0.956830858700545
**Terf2**	1354.19777712366	0.0243185936087021	0.165829122546485	0.146648509232057	0.88340946273807	0.956830858700545
**Nsf**	9192.65486049618	-0.0116199203287875	0.0790749714371188	-0.146948144496363	0.883172950555471	0.956830858700545
**Zdhhc23**	224.608361103862	-0.0561974879853314	0.37950545553105	-0.148080843545959	0.88227896739822	0.956830858700545
**Fhl4**	68.1955741652266	-0.0660297091562362	0.449065935203929	-0.147037893502768	0.883102110674701	0.956830858700545
**Plekhm3**	1277.63032664587	0.0188237211932099	0.128388711940868	0.146615079383922	0.883435850685348	0.956830858700545
**Atp6v1a**	4309.90568141978	-0.0093930612801899	0.0633159843300141	-0.148352132239336	0.882064874906961	0.956830858700545
**Rnf169**	795.884017458196	0.0227333999129057	0.153843964031451	0.14776920275051	0.882524915111347	0.956830858700545
**Nap1l1**	4449.4378573168	0.0116548881603572	0.0785183484248127	0.148435217935303	0.881999308015687	0.956830858700545
**Gm24616**	34.6915387997508	-0.0594549703092941	0.405565651591306	-0.146597647202154	0.883449610879734	0.956830858700545
**H2-Eb2**	28.1020660798943	0.101269870747294	0.688290137014492	0.147132532200097	0.883027412319575	0.956830858700545
**Gm5518**	1654.72627827008	0.0152312211459279	0.10255415167277	0.148518815645102	0.881933337885954	0.956830858700545
**Gprasp2**	44.7457358314202	-0.0875755195926399	0.593232923218922	-0.147624172841671	0.882639376949903	0.956830858700545
**Kantr**	1638.26876286102	0.0225940470128477	0.152498726597743	0.148158922483633	0.882217349077929	0.956830858700545
**Igkv10-96**	41.3265833064628	0.093681396335087	0.639519238782728	0.146487221421832	0.883536776930439	0.956830858700545
**Gm30214**	25.827225861271	0.0644697666355693	0.438542626201804	0.14700912245165	0.88312481988094	0.956830858700545
**Gm21769**	167.956179066928	0.0258248633845472	0.175214748267582	0.147389781053752	0.882824370972377	0.956830858700545
**Sfxn1**	5161.59430561999	-0.0147812454584709	0.101606961477421	-0.145474731687115	0.884336064700075	0.95709657001009
**Tgds**	1738.67125396688	0.0184860179878505	0.127350714004513	0.145158337998759	0.884585858926094	0.95709657001009
**Riox2**	7536.27364986024	-0.0084674944025200	0.0579826331130745	-0.14603500993146	0.883893750752486	0.95709657001009
**Zfp51**	6655.31508198671	-0.0137040196362226	0.094423779800793	-0.145133139820648	0.884605753493491	0.95709657001009
**Acsbg3**	104.579232742586	-0.053509821664352	0.368621771632803	-0.145161859071241	0.884583078960516	0.95709657001009
**Ola1**	1251.4500834832	-0.0129248557718179	0.0886462491603719	-0.1458026244115	0.884077204065668	0.95709657001009
**Evc**	20.8628159374148	0.22314554386856	1.53507785641174	0.145364316823751	0.884423236421658	0.95709657001009

**Aph1b**	35.7050434803955	0.145336951084022	0.996730429838733	0.145813699204043	0.884068461093288	0.95709657001009
**St7l**	3068.04163515542	0.0135444683703993	0.0931040628287297	0.145476662982099	0.884334539969363	0.95709657001009
**Bcas3**	6521.10464415603	0.0134472828149522	0.0926614443609949	0.145122741261874	0.884613963426838	0.95709657001009
**Adgrg3**	549.214507814885	-0.0283556784544443	0.195052487703347	-0.145374605514235	0.884415113517152	0.95709657001009
**Selenoi**	1475.69879154484	0.0114038457558191	0.0785934153425272	0.145099251713628	0.884632509083388	0.95709657001009
**Gm11518**	705.725271999415	-0.0136289141554806	0.0934930443072557	-0.14577463228912	0.88409930245251	0.95709657001009
**Gm43009**	24.8305550222163	-0.0977310109374029	0.672653287005919	-0.145291806083961	0.884480483877977	0.95709657001009
**Zfp937**	610.382953721701	0.0243095135963467	0.167812156786846	0.144861457368816	0.884820257939149	0.957198222421453
**9230116L04Rik**	19.5170103420451	-0.0975985820911058	0.673900622837535	-0.144826371698777	0.884847960131426	0.957198222421453
**Vps51**	6894.22986398299	-0.0100104491057164	0.0692805921072453	-0.144491390752267	0.88511245429917	0.95728718116494
**Erp29**	3242.9585948047	-0.0127997789652398	0.0885800017684701	-0.144499646756565	0.88510593537147	0.95728718116494
**Tap1**	35424.0906343322	-0.0090852373764420	0.0628365788045792	-0.144585169168693	0.885038407470177	0.95728718116494
**Cnrip1**	365.848068258113	-0.0326935169933477	0.226405300375449	-0.144402613097538	0.885182553483716	0.957297288623398
**Kdelr1**	2792.13687230009	-0.0152641156034715	0.106010354195746	-0.143987025788882	0.885510714794509	0.957586461671613
**Psma5**	6790.53682740709	0.0149820999192428	0.104188799589305	0.143797605676423	0.885660293619618	0.957682490156761
**Nprl3**	3504.69647447071	-0.0175818875098519	0.122395415590313	-0.143648252061194	0.885778236124279	0.957744298813577
**Dnm1l**	3943.23548696511	-0.0122184984772492	0.0854130910613669	-0.143051824087136	0.886249252331086	0.958020276286656
**Ackr4**	18.5361783977048	0.132160154407498	0.923442983042156	0.143116745521325	0.886197980068021	0.958020276286656
**Gm15163**	313.740566144102	-0.0264914164833688	0.185232508559378	-0.143017101530409	0.88627667496545	0.958020276286656
**Trav15n-2**	65.1274247499571	0.0397296253797461	0.277404028346712	0.143219352712824	0.886116946124343	0.958020276286656
**Cacna2d2**	20.7313048621613	-0.0756652470878762	0.529607755164987	-0.142870353294401	0.886392572981232	0.958044093516001
**Clp1**	4189.02374244338	-0.0188614893982454	0.132085636430577	-0.142797429818637	0.8864501669873	0.958044093516001
**Gm14597**	95.4538351840879	-0.0325184243210442	0.228142591699401	-0.142535526044564	0.886657020100717	0.958044093516001
**Moap1**	75.7332148339839	0.105679130074796	0.741465870717257	0.142527301995124	0.886663515627584	0.958044093516001
**Gm48909**	17.9449857523946	0.0803877761197819	0.563358640000297	0.142693784051558	0.886532026088576	0.958044093516001
**E430002N23Rik**	43.9118806930628	0.0875764207422363	0.614282742889489	0.142566955943269	0.886632196177176	0.958044093516001
**Iglv1**	479.67449784474	0.152092628030751	1.06877599343752	0.142305430665198	0.88683875713301	0.958167738353322
**Atxn7**	2251.70919746498	0.0246021942151239	0.173535850005456	0.141770096578606	0.887261604925401	0.958274721374151
**D830025C05Rik**	222.653260617422	-0.0545310697913524	0.384595020261535	-0.141788288767415	0.887247234816287	0.958274721374151
**Samd1**	88.6589996466918	-0.0358345888569268	0.252858221569364	-0.141718108410791	0.887302670866844	0.958274721374151
**Gm11737**	96.5685445584781	0.0615779310452515	0.434190247505472	0.141822464689227	0.887220239169567	0.958274721374151
**4930595D18Rik**	32.6795713568419	-0.0510529306525352	0.359319185490263	-0.142082395580624	0.887014923388016	0.958274721374151
**Gm36738**	194.598738138213	0.0394862540877467	0.278203811027103	0.141932829539492	0.887133062590048	0.958274721374151
**Gpr65**	3868.24409828477	0.0197944852163425	0.13976947457689	0.14162237696224	0.887378290827534	0.958290708557978
**Med17**	3512.36110696186	-0.0117133866681135	0.0829242726764683	-0.141254017551818	0.887669273982941	0.958460561410057
**Gm9761**	183.233180158832	0.0305756749959899	0.216553472608948	0.141192263636443	0.887718057575577	0.958460561410057
**Kcnb1**	80.6134714289149	0.0682976695219487	0.483532903570888	0.141247201622828	0.887674658325153	0.958460561410057
**Ltb**	94187.1177169083	-0.0113703774859585	0.0806222970811529	-0.141032665870501	0.887844136610238	0.958471646417021
**Jtb**	6625.93400856948	-0.0138913829933233	0.0985027946627767	-0.141025267769106	0.887849981030833	0.958471646417021
**Tk1**	309.318510936741	-0.0330653575386988	0.235123884802251	-0.140629513528616	0.888162631507232	0.958743480486364
**Sap18**	6197.27041336835	-0.0126902222615575	0.0905242847617403	-0.140185832950331	0.888513165037924	0.958769341485179
**Pacc1**	1911.01973236778	0.0101729094940533	0.0724687034921344	0.140376590222253	0.888362452993001	0.958769341485179
**Cdo1**	38.9014130296635	-0.0582072540599017	0.414335345943117	-0.140483438426933	0.888278036944487	0.958769341485179
**Lpcat2**	74.6395289081976	0.0347527857070374	0.247784506266733	0.140254070888625	0.888459251666805	0.958769341485179
**Rnf157**	408.161109313033	0.0648781307480216	0.462569174154874	0.140256061953449	0.888457678575782	0.958769341485179
**Ticam2**	35.394469276292	-0.0978115175600116	0.697970207525718	-0.140137095402324	0.888551671873331	0.958769341485179
**A730063M14Rik**	18.8697659222421	-0.124723386611694	0.891439139274121	-0.139912396838726	0.888729206375273	0.95889524136231
**Rab10**	16236.3830287947	0.0077318200797272€	0.0555716944282573	0.139132343529834	0.889345570338649	0.958974569871776
**Itgb5**	31.8570960511898	0.126441154793985	0.908253879647231	0.139213448604365	0.889281481538113	0.958974569871776
**Emilin2**	49.8977394041989	0.0896319468810323	0.64254174491739	0.13949591227346	0.889058285893271	0.958974569871776
**Rhbdd1**	2885.23261167242	0.0200899913126159	0.144045779739037	0.139469489137497	0.889079164414206	0.958974569871776
**Acad8**	2946.9611871611	-0.013305662472262	0.0956698007585385	-0.139079023545206	0.889387703902267	0.958974569871776
**Polr3b**	5368.08333383108	0.0158606723720089	0.113562746314463	0.139664395999104	0.888925158502134	0.958974569871776
**1110008P14Rik**	738.939812241027	0.0187431370182088	0.134153567334611	0.139714041084416	0.888885932042597	0.958974569871776
**Nudcd3**	7411.73470989508	0.0098615338372762€	0.0707397281073853	0.139405877024381	0.889129428515155	0.958974569871776
**Gm10744**	44.070024884365	-0.0676592362324892	0.486361818755974	-0.139112968212738	0.889360880718683	0.958974569871776

**Gm6551**	203.876492171728	-0.0334148379643668	0.240309593496773	-0.139049121918703	0.889411332366957	0.958974569871776
**Jak1**	135726.120492655	-0.0089468952635165	0.0644325115932833	-0.138856844817596	0.88956327336072	0.959072767591164
**Sall2**	66.4150664876472	0.0727475631109355	0.524387155105463	0.138728728197594	0.889664515783939	0.959116295512283
**Mis18a**	3582.65158282215	0.0132586940977186	0.0957588212675192	0.138459245030576	0.889877477076936	0.959149011991332
**Gm14391**	128.162803628651	-0.0444556370247126	0.320794627151982	-0.138579743119111	0.889782251486606	0.959149011991332
**Gm8319**	666.698226911119	-0.0218066711221357	0.157482479842704	-0.138470458072012	0.889868615720012	0.959149011991332
**Olfr755-ps1**	53.6241944663948	-0.0511126931297383	0.369604890517941	-0.138290088797559	0.890011158244138	0.959227484299103
**Gm47941**	20.7199593097958	0.0741519318351495	0.536760396458964	0.138147173905403	0.890124103782918	0.959283599159253
**Smcr8**	1073.25715042077	-0.0228373181198746	0.165644564172288	-0.137869408718546	0.890343627791522	0.959454557728835
**Nfrkb**	7940.08462411295	0.0144977346911642	0.105279970625457	0.137706484956585	0.890472393979112	0.959527696910953
**Atp9b**	8390.20477275994	0.0111227393738326	0.0809080958314884	0.137473750426638	0.890656339857004	0.959529054227185
**Rbbp9**	756.357255305929	-0.0216062319427667	0.157113281886337	-0.137520085401804	0.890619717715189	0.959529054227185
**Gm37563**	23.6857477212677	0.0869806998445019	0.632659715675379	0.137484176231528	0.890648099510562	0.959529054227185
**Vpreb1**	41.697560394471	0.0568780771123505	0.414315573753812	0.137282015727817	0.890807885210626	0.959626707271746
**Trappc1**	2033.65318872517	0.0145220855594367	0.1059849921359	0.137020206981906	0.891014822891765	0.959784014513641
**Glod4**	3218.11179885086	0.0111062666516705	0.0812924239149015	0.136621177187394	0.891330236486583	0.95981147028154
**Limd1**	16276.3062930419	-0.0151046476894178	0.110631232387845	-0.136531496245696	0.891401127266281	0.95981147028154
**Ankzf1**	3694.78315297442	-0.0139381394114193	0.101955777676845	-0.136707695522632	0.891261846499755	0.95981147028154
**Ift81**	75.1780632486119	0.0525648645403263	0.385018385972499	0.136525595803835	0.891405791464115	0.95981147028154
**Gm9821**	21.8821976249706	-0.067610314447191	0.495112010177952	-0.136555593597681	0.8913820787629	0.95981147028154
**Gm6087**	79.5470130601884	-0.0459683722590337	0.336612995098584	-0.136561490282248	0.891377417554234	0.95981147028154
**Lrrc27**	116.773446690993	0.0460660442153248	0.33849103396553	0.13609236166656	0.89174826585557	0.959871070844611
**Entpd7**	308.905409456416	0.0330750703851844	0.243555684840784	0.13580085558999	0.891978714665519	0.959871070844611
**Phkg2**	6622.44484225941	0.0081866008364812	0.0603696300697013	0.135607934437054	0.892131232627995	0.959871070844611
**Cdk10**	1878.33274590746	-0.0124933831038228	0.0921031099617703	-0.135645616190469	0.892101442194474	0.959871070844611
**Igsf6**	141.100498731412	0.0410748142966004	0.302809779044868	0.135645600436551	0.892101454649171	0.959871070844611
**Dis3l2**	3960.8604043161	0.0095567558661778	0.070378371213428	0.135791091800011	0.891986433543519	0.959871070844611
**Flvcr1**	2974.63169503714	0.0144857168536426	0.106587848691214	0.135904017498353	0.891897159441626	0.959871070844611
**Xlr4b**	4501.62511499816	0.0119899250702778	0.0879355432758248	0.136349019106749	0.891545374044894	0.959871070844611
**Gm5466**	18.2960433441617	-0.0698091643458824	0.512817487780147	-0.136128673474199	0.891719560360265	0.959871070844611
**Gm29093**	59.4215109445209	0.0623648302170293	0.459046971556851	0.135857186913836	0.891934181480591	0.959871070844611
**Gm48369**	37.9751718487782	-0.0662452287962519	0.486732567333724	-0.136101903267203	0.891740722940765	0.959871070844611
**1700123M08Rik**	37.0322288614838	0.0651055474669881	0.480504831164944	0.135494053845713	0.892221265249506	0.959902394956743
**Agrp**	22.8936350418889	-0.0721778170194403	0.534162093876713	-0.13512343508991	0.892514281577801	0.959991263138997
**Nyap1**	28.3604416413559	0.0962034012023272	0.71135919458343	0.135238852516222	0.892423029384662	0.959991263138997
**Ccdc6**	3023.19195034486	0.0182165114314005	0.134754943166337	0.135182509846148	0.892467575272113	0.959991263138997
**Ccdc125**	1358.93292895926	-0.0159436525051814	0.118030017724068	-0.135081336194109	0.892547566477522	0.959991263138997
**Cd3d**	38668.3589836227	-0.0090475354269530	0.0670364766561045	-0.134964363854722	0.892640050005535	0.960025204282112
**Popdc2**	31.3784241154899	-0.0675555296636599	0.501259088371999	-0.134771680415947	0.89279239727505	0.960093269577115
**Epha1**	61.1707713784358	-0.0682114010288637	0.506281475932949	-0.134730193126595	0.892825200172207	0.960093269577115
**4930513N10Rik**	185.998033630011	-0.0309546678942798	0.229913370239837	-0.134636223469688	0.892899500160562	0.960107644604202
**Ap3d1**	12321.3589692693	-0.0142676000188426	0.106666516681734	-0.133758938256262	0.893593197860585	0.960406045878211
**Esyt1**	49265.1681426501	0.0097979404330604	0.0733163847428847	0.133639164934566	0.893687912814792	0.960406045878211
**Stk17b**	59257.6719091526	0.0132325432867348	0.0991094344134093	0.133514466761445	0.893786523878789	0.960406045878211
**Pms1**	1681.24937478291	0.014418125231846	0.107882453987908	0.133646619064321	0.893682018156042	0.960406045878211
**Cbr4**	498.834639336193	0.0238739843981204	0.17815164773412	0.134009338121592	0.893395190290054	0.960406045878211
**Specc1l**	7544.91340896003	0.0230018661418182	0.171677801868738	0.133982762427289	0.893416205116853	0.960406045878211
**Spryd3**	4167.78866984603	-0.0100430373612488	0.0750281406406621	-0.133856940549129	0.893515700239544	0.960406045878211
**Spats2l**	71.7067626833851	-0.0426961508333284	0.318599903202973	-0.134011813575874	0.893393232819399	0.960406045878211
**A330040F15Rik**	34.5529779117879	0.0714976066192303	0.535470657028037	0.133522921715366	0.893779837666365	0.960406045878211
**1700042O10Rik**	51.8129966251877	-0.0494197333342172	0.370010130970999	-0.133563189755177	0.893747993638034	0.960406045878211
**Oxct1**	6482.20857789972	0.0089066853900968	0.0669268674592098	0.133080864654621	0.894129428321216	0.960513133333607
**Mx2**	129.977710771156	-0.0454059839213727	0.341491268457103	-0.132963821085447	0.894221992981141	0.960513133333607
**Herc3**	5906.39465915062	0.0218803958524384	0.164524076346653	0.132992060118522	0.894199659828199	0.960513133333607
**Ino80e**	4896.99774649328	-0.0085276596474341	0.0642026472247473	-0.132824112650406	0.89433248418199	0.960513133333607
**Gapdhs**	61.3693799616653	-0.0433324111160446	0.326367545754878	-0.132771814108594	0.89437384605386	0.960513133333607

**Gm5844**	574.790233370146	0.0190610450210296	0.143473930258265	0.132853717652525	0.894309070304629	0.960513133333607
**Gm9522**	94.8945273988316	0.0688312801977214	0.517344750219023	0.133047218839238	0.894156037183236	0.960513133333607
**Gm18584**	64.516832880424	0.0386468986368876	0.290659521863601	0.132962781983188	0.894222814768303	0.960513133333607
**Adamts17**	41.3284574965545	-0.0851359789684298	0.641651067848646	-0.132682673238396	0.8944443464491	0.960523380835444
**Snrnp35**	191.597843742616	-0.0317490439224929	0.239901387766773	-0.132342060285865	0.894713740633095	0.960747199887943
**Gm12338**	211.12098846727	0.0255376050324223	0.193095922785631	0.132253465863043	0.894783812837769	0.960756970281754
**Pacsin3**	21.8045518717758	-0.129215400944068	0.979467099188347	-0.131924187194389	0.895044257217088	0.960840193552913
**Arl6ip4**	3434.3302473787	-0.014107226520174	0.106901569289468	-0.131964634513217	0.895012264626201	0.960840193552913
**Gm14253**	679.516483650976	0.019246157819955	0.14583589988492	0.131971331031264	0.895006967901917	0.960840193552913
**Il11ra1**	483.836209036102	-0.0249149918381271	0.18905114272836	-0.131789691818613	0.895150640169031	0.960888932293986
**Gm5819**	21.1228288236226	0.0633834119054127	0.481527997836507	0.131629754012628	0.895277149990829	0.960959267875034
**Thnsl2**	286.488340242445	0.0419844512522594	0.319313055519367	0.131483666347344	0.895392706763924	0.961017838054571
**Sdf2**	4045.59120372293	-0.0100051889608397	0.0761423670173754	-0.131401076073147	0.895458037461492	0.9610224966817
**Brca1**	241.140991929235	-0.0375174981649121	0.286555422188805	-0.130925800943989	0.895834004159349	0.961229581446296
**Gm45837**	13173.55842657	-0.0403798528744764	0.308122924174038	-0.131051115338852	0.895734871851865	0.961229581446296
**Hdlbp**	7219.66857348544	0.0140650387239996	0.10741041893722	0.130946689000631	0.895817480156526	0.961229581446296
**Surf1**	4175.00562160943	-0.0122144279638149	0.0938793754342332	-0.130107682409665	0.896481232104663	0.961544430684871
**Hmg20b**	2262.09843611526	0.0100167852474411	0.0770180871483997	0.130057569829546	0.896520879290846	0.961544430684871
**Esrra**	1742.41242746973	-0.0157683708346041	0.121318956379839	-0.129974501142548	0.896586600676825	0.961544430684871
**Wdr41**	2179.54344823045	0.0103821539447358	0.0797982747841564	0.130104992530454	0.896483360229231	0.961544430684871
**Tssk6**	228.998081375059	0.0260320585648876	0.200323115458152	0.129950347993293	0.896605710037581	0.961544430684871
**Cracr2a**	4123.22522864927	0.0111689297601526	0.0856204028297357	0.130447059240811	0.896212736744751	0.961544430684871
**2210418O10Rik**	183.919890385557	-0.0322759486538686	0.248082744792541	-0.130101546082374	0.896486086921839	0.961544430684871
**Gm13092**	27.2617868836048	-0.0574557547510629	0.442178832469579	-0.129937822736044	0.896615619727918	0.961544430684871
**Stam**	1754.36483682968	-0.0230345277768682	0.177418431210425	-0.129831650633571	0.896699621257685	0.961569071196226
**Tex30**	2245.90560375033	-0.0165483669736166	0.127637853330857	-0.129650934591643	0.896842603324393	0.961656951383631
**Pdxk**	2569.80510078124	0.0141263188733874	0.109032129499976	0.12956106551501	0.896913708753777	0.961667753731169
**Cd209e**	37.528085375879	-0.109529920183827	0.846318036436427	-0.129419338201774	0.897025846688371	0.961718204038489
**4933440N22Rik**	124.012654087369	-0.0285431441235155	0.220670550629116	-0.129347319078785	0.897082830675745	0.961718204038489
**Drosha**	1493.84392583875	-0.0150782301232644	0.117365059033116	-0.128472905373053	0.897774738897078	0.961940854537384
**Spindoc**	2125.57495698388	0.0117106297204989	0.0911608724155343	0.128461141388806	0.89778404806247	0.961940854537384
**Gba2**	8426.3569166817	-0.0100968823663213	0.078466653862955	-0.12867736636197	0.897612945522284	0.961940854537384
**Uri1**	5031.01947414099	-0.0144919027281432	0.112685680063945	-0.128604652515915	0.897670484708411	0.961940854537384
**Fam214b**	633.995368808388	-0.0194022150508855	0.150940368879583	-0.128542252777745	0.897719862667888	0.961940854537384
**Ufsp1**	112.568873749035	-0.0579140014967405	0.451077020243168	-0.128390494079082	0.897839953524231	0.961940854537384
**Zscan20**	893.837439233421	0.024385163199673	0.189613995407667	0.128604236977577	0.897670813529558	0.961940854537384
**Gm10156**	164.070210492197	-0.0258779330796241	0.200838448279271	-0.128849497202051	0.897476738830951	0.961940854537384
**1700066B19Rik**	303.722130129025	-0.0483499229041956	0.375628953095205	-0.128717242123615	0.897581391665944	0.961940854537384
**Ikzf2**	365.924856673295	0.0425361289701297	0.331919582047719	0.128151911700149	0.898028755090444	0.962017661498077
**Traj9**	89.1871662677946	-0.0568741731391518	0.44382462311833	-0.128145601160097	0.898033748998871	0.962017661498077
**Gm7327**	264.291867248355	0.0243831049103006	0.190422373149117	0.128047479437757	0.898111399115981	0.96203543955612
**Atg10**	829.911904580389	-0.0121620802141251	0.0950382828324717	-0.127970327868441	0.898172454868783	0.962035440919545
**Trim32**	2326.43244739738	0.0121696995462378	0.0953567754187524	0.12762280910607	0.8984474796802	0.962264609660204
**Ston2**	40.9349298579371	-0.0866528776286824	0.680425302457897	-0.127351051343427	0.898662556072193	0.962364135926978
**Supt20**	13416.130060562	0.0117436352810909	0.0921591426233181	0.127427783579656	0.898601827364802	0.962364135926978
**Ubxn4**	8557.25227183541	0.0114887610589392	0.0904568735279041	0.127008159920485	0.898933940172677	0.962460214563556
**Dnajc17**	1012.80942873174	0.0111119258088679	0.0875444272005088	0.126928990961555	0.898996600745473	0.962460214563556
**Gm43773**	26.8564272373846	0.0878653301652997	0.691810481470191	0.127007804187317	0.89893422172662	0.962460214563556
**Gm49694**	31.6681584780463	0.0557983174229205	0.439594111798313	0.126931448637148	0.898994655537245	0.962460214563556
**Rbmx**	4093.77889075493	-0.0131238656607616	0.103750919253851	-0.126493969934386	0.899340922002473	0.96259977567779
**Dph6**	2397.16672009983	0.00861463862870997	0.0680846582711522	0.126528337623456	0.899313719119399	0.96259977567779
**Tmem267**	144.974917644094	0.03597196010155	0.284463242664481	0.126455565100825	0.899371320526015	0.96259977567779
**Trav9-4**	400.818944684129	0.023297376872645	0.183936694723166	0.12665975599762	0.899209699289778	0.96259977567779
**Arl14ep**	1483.23777899937	0.014594256181046	0.115486755673739	0.126371687349813	0.899437712676793	0.962605449884414
**Hace1**	2722.70223721982	-0.011672162424219	0.0925313756788727	-0.126142752537549	0.899618926105883	0.962734000180006
**Map4k3**	214.048589016245	0.0310456833389688	0.246879741652905	0.125752251404316	0.899928039482975	0.9628137162525

**Gdf11**	432.919942883275	0.0218617611619565	0.174027795721765	0.125622237937835	0.900030959076537	0.9628137162525
**Flna**	99343.1694105766	-0.0244868285410446	0.194875207862569	-0.125653893122789	0.900005900449966	0.9628137162525
**Kif23**	2333.57832113498	0.0148406007040176	0.118056491920994	0.125707620669851	0.899963369303244	0.9628137162525
**Baiap2l1**	32.975337142886	-0.074342232215599	0.591965081178411	-0.125585502556346	0.900060039372934	0.9628137162525
**Fam83h**	43.1696191598046	0.0745525768474795	0.592579915858071	0.125810164759846	0.89988219539652	0.9628137162525
**Uchl4**	150.067713348868	-0.0303120535228453	0.241938288166908	-0.125288368999014	0.900295259822344	0.962999959881928
**Usp15**	10552.3691756847	-0.0079034244156077	0.0632676885013348	-0.124920391479784	0.900586574758294	0.963173363028705
**Wdr48**	6045.53984341092	0.0098163226758341	0.0785715504809722	0.12493482202838	0.900575150344988	0.963173363028705
**Mypop**	305.611603174499	-0.018764731637462	0.150295853087462	-0.124851958666765	0.900640752101601	0.963173363028705
**Dbi**	1918.95482203726	0.0128910990249957	0.103431010787173	0.124634758249838	0.900812709833235	0.963226511186365
**Gm24888**	27.2944427577677	0.0509648993840634	0.408743220762557	0.124686837102723	0.9007714785421	0.963226511186365
**Ctrl**	107.845614267658	-0.0324225777211299	0.261081305733884	-0.124185749837553	0.901168204876658	0.963541250202993
**Amdhd1**	32.2473681620122	-0.0817639990729533	0.660702814051774	-0.123753066180442	0.90151079381897	0.963574131131972
**Pdzd9**	32.8987496690293	-0.0521197972089264	0.420435248898048	-0.123966288139568	0.901341967291944	0.963574131131972
**Cldnd2**	21.7020467814984	0.0653845947030063	0.528644406744835	0.123683507985295	0.901565870107169	0.963574131131972
**Gm10819**	204.071546648761	0.0278330233441079	0.224725768533377	0.123853279157766	0.901431445859693	0.963574131131972
**Trav5n-4**	111.237914706857	0.0286229253676202	0.230871788051305	0.123977579110963	0.901333027363886	0.963574131131972
**Gm16385**	42.3596417964373	0.0373939448730458	0.302188113808941	0.123743930235086	0.901518027647817	0.963574131131972
**Col7a1**	26.7931214028081	0.0619932862364612	0.502289870101076	0.123421334824025	0.901773463484658	0.963730633757987
**Ndufb2**	1614.88184142359	0.0125844192322471	0.10231472418096	0.122997147604968	0.902109356698522	0.963834988750106
**Prpf6**	10615.4791832471	-0.0095881232676230	0.0778584973619327	-0.123148064662123	0.901989850814509	0.963834988750106
**P3h4**	58.2491323945095	-0.0447287032276779	0.365491635946825	-0.122379553534258	0.902598430701887	0.963834988750106
[truncated: 138,084 more chars]
